# The fitness consequences of genetic divergence between polymorphic gene arrangements

**DOI:** 10.1093/genetics/iyad218

**Published:** 2023-12-26

**Authors:** Brian Charlesworth

**Affiliations:** Institute of Ecology and Evolution, School of Biological Sciences, University of Edinburgh, Edinburgh EH9 3FL, UK

**Keywords:** inversion polymorphism, mutational load, heterokaryotype advantage, efficacy of selection, population subdivision

## Abstract

Inversions restrict recombination when heterozygous with standard arrangements, but often have few noticeable phenotypic effects. Nevertheless, there are several examples of inversions that can be maintained polymorphic by strong selection under laboratory conditions. A long-standing model for the source of such selection is divergence between arrangements with respect to recessive or partially recessive deleterious mutations, resulting in a selective advantage to heterokaryotypic individuals over homokaryotypes. This paper uses a combination of analytical and numerical methods to investigate this model, for the simple case of an autosomal inversion with multiple independent nucleotide sites subject to mildly deleterious mutations. A complete lack of recombination in heterokaryotypes is assumed, as well as constancy of the frequency of the inversion over space and time. It is shown that a significantly higher mutational load will develop for the less frequent arrangement. A selective advantage to heterokaryotypes is only expected when the two alternative arrangements are nearly equal in frequency, so that their mutational loads are very similar in size. The effects of some *Drosophila pseudoobscura* polymorphic inversions on fitness traits seem to be too large to be explained by this process, although it may contribute to some of the observed effects. Several population genomic statistics can provide evidence for signatures of a reduced efficacy of selection associated with the rarer of two arrangements, but there is currently little published data that are relevant to the theoretical predictions.

## Introduction

Wright and Dobzhansky (1946) obtained evidence that some natural inversion polymorphisms in *Drosophila pseudoobscura* are associated with major differences in fitness among karyotypes, which can lead to their stable maintenance within a single population under constant environmental conditions. There have subsequently been many other experimental studies documenting strong effects of inversion karyotypes on fitness components in several *Drosophila* species (reviewed in Krimbas and Powell 1992; Kapun and Flatt 2019), and in some other species such as the seaweed fly *Coelopa frigida* (Butlin *et al*. 1984; Mérot *et al*. 2020). The startling observations of Wright and Dobzhansky (1946) raised the question of the causes of fitness differences between apparently functionally insignificant chromosomal variants. This question is still the subject of ongoing inquiry, stimulated by the new evidence from genome sequencing that inversion polymorphisms are abundant in natural populations of many species (Wellenreuther and Bernatchez 2018; Faria *et al*. 2019; Berdan *et al*. 2023).

Well before the work of Wright and Dobzhansky, Sturtevant and Mather (1938) had proposed a process that could cause fitness differences between inversion karyotypes, with a fitness advantage to heterokaryotypes over homokaryotypes. In their words:

… if a chromosome exists, in a population, in two sequences, differing by an inversion, it will in effect show two distinct lines of descent. There is free exchange of material within any line (*i.e*. sequence), but none between the sequences. Therefore, fluctuations of the genic contents must occur almost independently in the two sequences. Under such conditions, it is inevitable that in time the gene content of the two sequences will become different. It must be supposed that each sequence is susceptible to the same mutations, and with the same frequencies, but, as a result of the relative rarity in the population of a given mutant allelomorph at any one moment, certain genes will be present in one sequence but not the other. ….. It is thus clear, considering two sequences A and B, that the homozygotes AA and BB are more likely to be homozygous for deleterious recessive mutations than is the heterozygote AB. …. Thus in general the sequence heterozygote AB will be at a selective advantage with respect to either of the corresponding homozygotes.

Sturtevant and Mather (1938) did not attempt a quantitative model of this process, simply noting that “there can be no stability in the exact relations of the sequences with each other”, and that “…. a single gene difference can not in general cause such heterosis. The simplest effective condition is that in which each sequence contains a deleterious recessive not present in the other.” This proposal raises the question of what strength of selection on the two arrangements might be expected on its basis.

Ohta (1971) developed a mathematical model of a closely related, but not identical, process. This was based on the concept of associative overdominance, first outlined by Frydenberg (1963). Here, a polymorphic neutral locus can acquire an apparent heterozygote advantage, because of linkage disequilibrium (LD) generated by genetic drift with a locus subject to selection in favor of heterozygotes or to selection against recessive/partially recessive deleterious alleles maintained by mutation pressure. Unlike the model of Sturtevant and Mather (1938), this process does not require the generation of heterosis by multiple selected loci, often referred to as pseudo-overdominance (Waller 2021). Ohta (1971) summed the effects of individual selected loci that were completely linked to a dialellic neutral locus (equivalent to an inversion polymorphism) over a large segment of genome, and generated expressions for the apparent fitness advantage to heterozygotes at the neutral locus.

It was, however, shown by Zhao and Charlesworth (2016) that Ohta's formulae for the relative fitnesses at the neutral locus induced by LD with the selected locus do not predict any change in allele frequency at the neutral locus. Using a different approach, they found that an induced selection pressure in favor of increased variability at the neutral locus only exists when the product of population size and selection coefficient is of the order of 1. Otherwise, variability is reduced by background selection effects, even for recessive or partially recessive deleterious mutations (see also Charlesworth 2022). Ohta's results therefore do not solve the quantitative problem of whether the strength of selection on inversions revealed by the experiments cited above can be explained by this process. Nonetheless, they are still invoked as a potential contributor to the selective maintenance of inversion polymorphisms, e.g. Faria *et al*. (2019), Berdan *et al*. (2021), Jay *et al*. (2021), and Matschiner *et al*. (2023).

A different perspective was developed by Nei *et al*. (1967), who examined a purely deterministic model involving the balance between mutation and selection at numerous autosomal loci. This process results in an equilibrium frequency distribution of the number of mutant alleles per haploid genome. A new autosomal inversion then has a chance of arising on a haplotype with a lower number of mutations than average and acquiring a selective advantage. But, as time goes on, the mutant-free loci on the inverted haplotypes will accumulate mutations (Nei *et al*. 1967). Unless the inversion goes to fixation, the loci in the inversion subpopulation will eventually acquire the same frequencies of mutant alleles as the corresponding loci in the standard arrangement subpopulation. Nei *et al*. (1967) interpreted this as implying that the inversion would then be selectively neutral. However, if reverse mutations from mutant to wild-type alleles do not occur, the loci at which mutations were present in the original inversion haplotype will all be homozygous in inversion homokaryotypes, causing these to have a reduced fitness compared to homokaryotypes for the standard arrangement. A reanalysis and extension of this model by Connallon and Olito (2021) suggested that it could produce a net heterozygote advantage to an autosomal inversion in a randomly mating population, resulting in the maintenance of the inversion, but only if the inversion has a sufficiently large direct selective (but nonheterotic) advantage that is independent of the deleterious mutations that it carries.

Berdan *et al*. (2021) conducted simulations of a finite population with multiple loci experiencing mutations to deleterious and completely recessive alleles. They found that a selective advantage to heterokaryotypes could develop, provided that recombinational exchange between arrangements in heterokaryotypes was sufficiently infrequent, and there was a small additional selective advantage to heterokaryotypes that kept the inversion in the population long enough for mutation accumulation to occur. Studies of the effects of deleterious mutations have shown that complete recessivity is unlikely to be frequent, especially for mildly deleterious mutations (Muller 1950; Crow 1993; Manna *et al*. 2011). Multilocus computer simulations with less extreme assumptions about the degree of recessivity of deleterious mutations showed that autosomal inversion polymorphisms are unlikely to be established at higher than neutral rates (Jay *et al*. 2022).

These theoretical studies therefore suggest that deleterious mutations in themselves are unlikely to create an initial selective advantage to autosomal inversions in a randomly mating population. Indeed, if a new inversion arises on a unique haplotype, the process of accumulation of new mutational load within the inversion subpopulation will take a long time, and cannot contribute to any initial selective effect of the inversion. It is, however, an open question as to whether the fitness differences among karyotypes mentioned above could have a significant component resulting from the process proposed by Sturtevant and Mather (1938), whereby genetic drift causes the inverted and standard arrangements to differ in their genetic content. There is a strong, but not perfect, analogy with population subdivision, where genetic drift can cause local populations to diverge at weakly selected loci subject to mutation to deleterious variants. In the case of inversions, however, the existence of heterokaryotypes means that selection does not act independently on the two subpopulations, as described below in the section on the mathematical model.

Provided that mutations are at least partially recessive with respect to their fitness effects, interpopulation crosses may show heterosis, due to different loci having accumulated different deleterious mutations in different populations (Whitlock *et al*. 2000; Glémin *et al*. 2003; Roze and Rousset 2004; Spigler *et al*. 2017; Charlesworth 2018). Such heterosis has been observed in populations of animals and plants (reviewed in Charlesworth 2018). In addition, theory predicts that populations with a smaller effective size (*N_e_*) should allow the accumulation of deleterious mutations and hence acquire larger mutational loads than populations with a larger *N_e_*, unless mutations have strong selective effects relative to drift and are highly recessive (Wright 1931; Kimura *et al*. 1963; Nei 1968; Bataillon and Kirkpatrick 2000; Charlesworth 2018).

Such differences in *N_e_* can arise either from differences in the adult population size itself, differences in the mating system (especially the frequency of inbreeding), differences in recombination rates associated with different levels of genetic hitchhiking effects, or a combination of all 3 factors. Again, there is empirical support for this predicted effect of *N_e_*, both from measurements of fitness components (Leimu *et al*. 2006; Lohr and Haag 2015; Charlesworth 2018) and from population genomic indicators of the efficacy of selection against deleterious mutations, e.g. Robinson *et al*. (2022) (population size), Glémin *et al*. (2006) (mating system), and Campos *et al*. (2014) (recombination rate).

The purpose of the present paper is to investigate the properties of a population genetic model of mutation, selection, and drift acting on a polymorphism for an autosomal inversion and a standard arrangement, in which the inversion polymorphism is maintained by sufficiently strong selection that the frequency of the inversion is constant over time and space. Similar assumptions were used in an earlier paper that examined neutral differentiation between the two arrangements (Charlesworth 2023). In order to simplify the calculations, and to maximize the effects of drift and mutation within arrangements, no recombinational exchange between arrangements in heterokaryotypes is allowed. The results should therefore provide upper bounds on the likely size of effects.

Models are developed of both a single, randomly mating, population and of a population divided into a large number of local populations. It might be expected that population subdivision, with its greater opportunities for drift, would enhance divergence among arrangements at sites under selection. The model assumes a large number of freely recombining sites subject to selection and mutation, with a wide distribution of selection coefficients over sites. No attempt is made to investigate the consequences of enhanced Hill–Robertson interference among sites due to restricted recombination in heterokaryotypes, which was included in the simulations of the fates of autosomal inversions in Berdan *et al*. (2021) and Jay *et al*. (2022). Unless an autosomal inversion is very rare or very common, there should be sufficient recombination within homokaryotypes to prevent major Hill–Robertson effects; very low recombination rates are sufficient to prevent the operation of Muller's ratchet (Charlesworth *et al*. 1993).

The overall conclusion is that a low-frequency arrangement will have a higher mutational load and exhibit weaker population genomic signals of purifying selection than its counterpart. Heterokaryotypic superiority in fitness is, however, unlikely to be observed unless the inverted and standard sequences are approximately equal in frequency, and it is likely to be small in magnitude unless the inversion contains millions of sites under selection. Population subdivision has only a small effect on the load and population genomic statistics.

## A model of the mutational load associated with an inversion polymorphism

### General considerations and notation

The main symbols used in this paper are defined in Table 1. Consider first a single randomly mating, discrete generation population of size *N*, assuming a Wright–Fisher model of reproduction such that *N* is equal to the effective population size. The frequencies of the two karyotypes, the inverted (*In*) and standard (*St*) arrangements are denoted by *x* and *y* = 1 – *x*; designation as *In* vs *St* is purely arbitrary in the situation considered here, so the convention *x* ≤ ½ is used. Balancing selection on the inversion is assumed to be sufficiently strong that *x* can be treated as constant over time. Let *q_i_* and *p_i_* = 1 – *q_i_* be the respective frequencies of the mutant (A_2_) and wild-type allele (A_1_) at a given nucleotide site within haplotypes carrying a type *i* karyotype, where *i* = 1 corresponds to *In* and *i* = 2 to *St*. In a given generation, there will be random drift as well as selection within the populations of *In* and *St* karyotypes, so that in general *q*_1_ ≠ *q*_2_. Drift occurs independently within karyotypes under the assumption of constant sizes of the two subpopulations, so that the effective population sizes of carriers of *In* and *St* are *N*_1_ = *Nx* and *N*_2_ = *Ny*, respectively.

**Table 1.** Definitions of the most important symbols used in the text.

*x* and *y*	Frequencies of the inversion (*In*) and standard arrangement (*St*), respectively
*N*	Size of the population (single population case) or size of a local population (subdivided population case)
*d*	Number of demes in the subdivided population case
*N_T_* = *dN*	Sum of local population sizes for a subdivided population
*L_i_*	Mutational load for the homokaryotypic subpopulation of type *i*
*L*_12_	Mutational load for *In*/*St* heterokaryotypes
*H_i_*	Homozygous load for subpopulation *i*
*B_i_*	Inbreeding load for subpopulation *i*
*t_i_*	Reduction in fitness of homokaryotype *i* relative to the heterokaryotype's fitness
*u* and *v*	Mutation rates from wild-type to mutant alleles and vice versa
*α* and *β*	*u* and *v* scaled by 4*N* or 4*N_T_*, depending on context
*m* and *M*	Migration rate and migration rate scaled by 4*N*
$\left\langle q_{i}\right\rangle$	Expected frequency of mutant alleles for subpopulation *i*
*V_qi_*	Variance in frequency of mutant alleles for subpopulation *i*
*s*	Selection coefficient against homozygotes for a deleterious mutation
*h*	The dominance coefficient for heterozygotes for a deleterious mutation
*a*	Shape parameter of the gamma distribution of selection coefficients
$\bar{\gamma }$	Mean selection coefficient against a deleterious mutation, scaled by 2*N* or 2*N_T_*

Subscripts *i* = 1 and *i* = 2 are used to denote parameters applicable to the *In* and *St* subpopulations, respectively. Subscript *d* is used to denote within-deme parameters.

The expectation of *q_i_* is denoted by $\left\langle q_{i}\right\rangle ,\;$with $\left\langle p_{i}\right\rangle \;$ = 1 – $\left\langle q_{i}\right\rangle$, where the angle brackets denote an expectation taken over the probability distribution of *q_i_*. The variance in *q_i_* in a given generation over this probability is denoted by $V_{q_{i}}=\left\langle q_{i}^{2}\right\rangle -{\left\langle q_{i}\right\rangle }^{2}$, with the corresponding *F* statistic (Wright 1951) given by *F_i_* = *V_qi_*$\left\langle p_{i}\right\rangle \left\langle q_{i}\right\rangle$. In the absence of recombination but the presence of selection, there will be a negative covariance *C*_12_ between *q*_1_ and *q*_2_, with a correspondingly negative correlation coefficient *R*_12_. This is because a higher frequency of the mutant allele in one karyotype results in a higher frequency of mutant homozygotes in *In*/*St* individuals, enhancing the strength of selection against the mutation in the other karyotype. For neutral sites in the absence of recombination between the arrangements, *C*_12_ = *R*_12_ = 0.

Following Kimura *et al*. (1963), equations can be written for the genetic loads at a single diallelic autosomal locus, assuming a homozygous selection coefficient *s* and dominance coefficient *h*. The fitness of mutant homozygotes relative to wild-type is 1 – *s* and the fitness of heterozygotes is 1 – *hs*; *s* may vary across loci, but *h* is treated as a constant, although it is easy to relax this assumption. *L_i_* is the genetic load for individuals homozygous for karyotype *i* produced by random mating within the population, defined as the expected reduction below 1 of their mean fitness relative to wild-type homozygotes. *L*_12_ is the corresponding load for heterokaryotypes. The homozygous load *H_i_* is the reduction below 1 in the expected relative fitness of individuals made homozygous for gametes with karyotype *i*, with probability $\left\langle q_{i}\right\rangle$ of being homozygous for the mutant allele. *B_i_* is the inbreeding load for karyotype *i*, defined as *H_i_ – L_i_* (Charlesworth and Charlesworth 2010, p. 173).

Following Charlesworth (2018), some simple algebra yields the following expressions for these quantities:


(1a) $$L_{i}=\left\langle q_{i}\right\rangle \left[2h+(1-2h)\left(\left\langle q_{i}\right\rangle +F_{i}\left\langle p_{i}\right\rangle \right)\right]s$$

(1b) $$L_{12}=\left\{h\left[\left\langle q_{1}\right\rangle \left\langle p_{2}\right\rangle +\left\langle q_{2}\right\rangle \left\langle p_{1}\right\rangle \right]+\left\langle q_{1}\right\rangle \left\langle q_{2}\right\rangle +(1-2h)C_{12}\right\}s$$

(1c) $$H_{i}=\left\langle q_{i}\right\rangle s$$

(1d) $$B_{i}=\left\langle p_{i}\right\rangle \left\langle q_{i}\right\rangle (1-F_{i})(1-2h)s$$

where


(1e) $$F_{i}=V_{qi}/\left\langle p_{i}\right\rangle \left\langle q_{i}\right\rangle$$

The selective difference between heterokaryotypes and a homokaryotype of class *i* is measured by *t_i_* = *L_i_* – *L*_12_. Heterozygote advantage exists if both *t_i_*s are positive. To obtain conditions for heterozygote advantage, it is useful to rearrange Equations (1) by writing $\left\langle q_{1}\right\rangle =\left\langle q\right\rangle +\left\langle \delta q\right\rangle$, $\left\langle q_{2}\right\rangle =\left\langle q\right\rangle -\left\langle \delta q\right\rangle$, where 2⟨δq⟩ is the expected difference in the frequency of A_2_ between *In* and *St*. ⟨δq⟩ will be nonnegative if *x* < ½, due to the greater effectiveness of drift relative to selection against nonrecessive, mildly deleterious mutations in a smaller population (Kimura *et al* 1963). Substituting these expressions for the *q_i_* into Equations (1a) and (1b), the following expressions are obtained:


(2a) $$\begin{array}{rl} L_{1} & =\left(\left\langle q\right\rangle +\left\langle \delta q\right\rangle \right)\left\{\left[2h+(1-2h)\left(\left\langle q\right\rangle +\left\langle \delta q\right\rangle +F_{1}\left(\left\langle p\right\rangle -\left\langle \delta q\right\rangle \right)\right)\right]\right\}s\\ & =\{\left\langle q\right\rangle \left[2h+\left(\left\langle q\right\rangle +\left\langle p\right\rangle F_{1}\right)(1-2h)\right]\\ & \;+\left\langle \delta q\right\rangle \left(2h+\left[F_{1}+2\left\langle q\right\rangle (1-F_{1})(1-2h)\right]\right)\\ & \;+\left\langle {\left(\delta q\right)}^{2}\right\rangle (1-F_{1})(1-2h)\}s \end{array}$$

(2b) $$\;\begin{array}{rl} L_{2} & =\{\left\langle q\right\rangle \left[2h+\left(\left\langle q\right\rangle +\left\langle p\right\rangle F_{2}\right)(1-2h)\right]\\ & \;-\left\langle \delta q\right\rangle \left(2h+\left[F_{2}+2\left\langle q\right\rangle (1-F_{2})(1-2h)\right]\right)\\ & \;+\left\langle \;{\left(\delta q\right)}^{2}\right\rangle (1-F_{2})(1-2h)\}s \end{array}$$

(2c) $$\;L_{12}=\left[{\left\langle q\right\rangle }^{2}+2\left\langle p\right\rangle \left\langle q\right\rangle h-\left\langle {\left(\delta q\right)}^{2}\right\rangle (1-2h)+2(1-2h)C_{12}\right]s$$

Equation (2c) shows that when *h* < ½ the mean fitness of heterokaryotypes is increased by a difference in the expected frequencies of deleterious mutations between the two karyotypes, as expected intuitively.

These expressions yield the following results for the selective differences between heterokaryotypes and homokaryotypes:


(3a) $$\begin{array}{rl} \;t_{1} & =\{\left\langle p\right\rangle \left\langle q\right\rangle F_{1}(1-2h)+\left\langle \delta q\right\rangle \left[2h+\left(2\left\langle q\right\rangle (1-F_{1})+F_{1}\right)(1-2h)\right]\\ & \;+\;{\left\langle \delta q\right\rangle }^{2}(2-F_{1})(1-2h)-2(1-2h)C_{12}\}s \end{array}$$

(3b) $$\begin{array}{rl} t_{2} & =\{\left\langle p\right\rangle \left\langle q\right\rangle F_{2}(1-2h)-\left\langle \delta q\right\rangle \left[2h+\left(2\left\langle q\right\rangle (1-F_{2})+F_{2}\right)(1-2h)\right]\\ & \;+\;{\left\langle \delta q\right\rangle }^{2}(2-F_{2})(1-2h)-2(1-2h)C_{12}\}s \end{array}$$

It is easily seen that, for an inversion with *x* < ½ and ⟨δq⟩ > 0 (see above), we have *t*_1_ > 0 provided that *h* ≤ ½ and *C*_12_ ≤ 0, so that *In*/*In* then has a lower fitness than *In*/*St*. The expectation for a low-frequency inversion is thus that *t*_1_ > 0, although the magnitude of the effect is likely to be small for a single, large population. Given that *F*_1_ > 0 and *h* < ½, this is also the case even when ⟨δq⟩ = 0, reflecting the fact that genetic drift causes a reduction in mean fitness by increasing the frequencies of homozygotes for recessive or partially recessive mutations; this effect is not experienced by *In*/*St* individuals unless *C*_12_ > 0, which can be ruled out by the argument given above.

If ⟨δq⟩ > 0 and *h* ≤ ½, the condition for *t*_2_ > 0 is more stringent than for *t*_1_ > 0, due to the opposite sign of the term in ⟨δq⟩ in Equations (3a) and (3b), especially if *F*_2_ is close to 0. It may therefore be difficult to find conditions in which there is an advantage to *In*/*St* over both homokaryotypes, unless the inversion frequency is close to ½, so that ⟨δq⟩ ≈ 0.

These results can be generalized to the case of a subdivided population with a constant frequency of the inversion across all local populations (demes), by taking expectations of within-deme allele frequencies across populations, as described in Section 5 of Supplementary File 1.

### A single population: modeling drift and selection

In order to obtain numerical results for the load statistics described above, expressions for the means and variances of the *q_i_*, as well their covariance, are needed. Recombination is assumed to be absent in heterokaryotypes. We first consider the expected changes in allele frequencies due to selection within each karyotype. For *In* karyotypes, the marginal fitness of haplotypes carrying the wild-type allele (A_1_) at a locus is easily seen to be


(4a) $$w_{11}=1\;\text{–}hs(xq_{1}+yq_{2})$$

Similarly, the marginal fitness of *In* haplotypes carrying the mutant allele is


(4b) $$w_{12}=1\;\text{–}hs(xp_{1}+yp_{2})-s(xq_{1}+yq_{2})$$

The net expected change in the frequency of A_2_ within *In* karyotypes due to selection (neglecting second-order terms in *s*) is thus


(5a) $$\Delta_{s}q_{1}\approx p_{1}q_{1}[w_{11}-\;w_{12}]=-sp_{1}q_{1}\{h+(1-2h)[xq_{1}+yq_{2}]\}$$

Similarly, the net expected change in the frequency of A_2_ within *St* karyotypes due to selection is


(5b) $$\Delta_{s}q_{2}\approx -sp_{2}q_{2}\{h+(1-2h)[xq_{1}+yq_{2}]\}$$

Equations (4) and (5) bring out the interdependence between the evolutionary processes in the two karyotypes when *h ≠* ½. To proceed further, the effects of mutation and drift also need to be analyzed, such that an expression for the stationary joint probability density function (p.d.f.) for *q*_1_ and *q*_2_, *ϕ*(*q*_1_, *q*_2_), can be obtained. Kimura (1964, p. 41) derived a pair of coupled forward diffusion equations describing the joint stationary p.d.f. for two variables, using the principle that a zero flux of the probability density of each variable across all their values guarantees a stationary joint distribution. This method can be applied to the above selection equations, together with the terms arising from mutation.

Let the rates of mutation from A_1_ to A_2_ and vice versa be *u* and *v*, respectively, with scaled mutation rates *α*_1_ = 4*N*_1_*u*, *α*_2_ = 4*N*_2_*u*, *β*_1_ = 4*N*_1_*v*, *β*_2_ = 4*N*_2_*v*. The mutational bias toward deleterious mutations, *κ*, is equal to *u*/*v*. As shown by Kimura (1964), in order to analyze this type of situation it is most convenient to use the natural logarithm of *ϕ*  ,ψ=lnϕ. If drift affects *q*_1_ and *q*_2_ independently, as assumed here, the conditions for a stationary distribution are


(6) $$V_{\delta q_{i}}\partial_{qi}\psi =2\Delta q_{i}-\;\partial_{qi}V_{\delta q_{1i}}\;\;\;\;\;\;\;(i=1,\;2)$$

where $V_{\delta q_{1}}=\;p_{1}q_{1}/(2N_{1})$ and $V_{\delta q_{2}}=p_{2}q_{2}/(2N_{2})\;$ are the variances in the changes in allele frequencies per generation due to drift within *In* and *St*, respectively. Correspondingly, $\partial_{q1}V_{\delta q_{1}}=(1\text{–}2q_{1})/2Nx$; $\partial_{q2}V_{\delta q_{2}}=(1\text{–}2q_{2})/2Ny$. The $\Delta q_{i}$ are given by the selection equations (4) and (5) together with the relevant mutation terms.

For a meaningful solution of Equation (6) to exist, *ψ* must have an exact differential, d$\psi =\partial_{q1}\psi \;dq_{1}+\partial_{q2}\psi \;dq_{2}$, which requires $\partial_{q_{1}q_{2}}^{2}\psi =\partial_{q_{2}q_{1}}^{2}\psi$ (Kimura 1964, p. 41). Kimura showed that this is the case for the mutation terms, so we need only consider the selection terms contributed by Equations (4) and (5). The following expression satisfies both this condition and Equation (6) for the selection contribution to *ψ*:


(7) $$\;\psi_{s}(q_{1},q_{2})=-2\gamma [h(xq_{1}+yq_{2})+\frac{1}{2}(1-2h)(xq_{1}+yq_{2}{)}^{2}]$$

where *γ* is the scaled selection coefficient, 2*Ns*.

The full solution to Equation (6) is thus


(8) $$\phi (q_{1},q_{2})=C\;\exp(-\psi_{s})\;q_{1}^{\alpha_{1}-1}p_{1}\;\beta_{1}-1q_{2}^{\alpha_{2}-1}\;{p_{2}}^{\beta_{2}-1}$$

where *C* is a normalization constant, given by the inverse of the double integral of *ϕ* over the closed intervals (0, 1) for *q*_1_ and *q*_2_.

### A single population: obtaining the mutational load and population genomic statistics

To obtain the single-locus load statistics for a given *h*, numerical integration of Equation (8) and its product with powers and crossproducts of the *q_i_* can be performed, for the purpose of determining the expectations and variances of the *q_i_* and their covariance *C*_12_. The corresponding *F_i_* statistics can be obtained from Equation (1e). The means of the single-locus load statistics, with the terms in *s* omitted, can then be obtained using Equations (1). Details of the integration procedures are given in Section 1 of Supplementary File 1.

In order to calculate the load statistics themselves under reasonably realistic assumptions, a gamma distribution of the scaled selection coefficient *γ* = 2*Ns* is assumed, with a p.d.f. given by


(9) $$\psi (\gamma )=\frac{\gamma^{a-1}\exp\left(-\frac{\gamma }{b}\right)}{b^{a}\Gamma (a)}$$

where *a* is the shape parameter, $b=\bar{\gamma }/a\;$ is the location parameter, and Γ(*a*) is the gamma function. This distribution has been widely used in population genomic methods for estimating the distribution of fitness effects of deleterious mutations (e.g. Charlesworth 2015; Booker *et al*. 2017).

The values of $\bar{\gamma }\;$ and the shape parameter *a* are chosen to correspond to estimates from the population genomics studies just mentioned, which indicate $\bar{\gamma }$ values of hundreds or thousands for nonsynonymous mutations and shape parameters of approximately 0.3, implying a wide distribution of selection coefficients. It is assumed that fitness effects are multiplicative across sites, so that the products of the number of sites, *n_s_*, and the expectations of Equations (1a)–(1c) over the distribution of *γ* correspond to the natural logarithms of the corresponding multisite load statistics. The exponentials of the negatives of these expressions then yield the mean fitnesses of the karyotypes concerned, relative to that of mutant-free individuals. The exponential of the negative of the product of *n_s_* and the expectation of Equation (1c) yields the fitness of totally inbred individuals of karyotype *i* relative to outbred individuals that are homozygous for karyotype *i*, i.e. a measure of the inbreeding depression experienced by carriers of karyotype *i*.

The net selection coefficient against homozygosity for karyotype *i* relative to heterokaryotypes for a given *n_s_* is measured by


(10) $$t_{si}=1-\exp\;(n_{s}E\{L_{12}-L_{i}\})$$

where E{} indicates the expectation over the distribution of *γ* (as opposed to an expectation over the distribution of *q*, denoted by angle brackets).

It is also of interest to summarize the expected patterns of variation at the loci themselves. To do this, the p.d.f. for *q*_1_ and *q*_2_ are used to calculate the expected nucleotide site diversities within each karyotype subpopulation for a given selection coefficient, $\pi_{i}=2\left\langle p_{i}\right\rangle \left\langle q_{i}\right\rangle (1-F_{i}),$ which are then averaged over the distribution of *s*. In addition, the expected proportion of segregating sites for a sample of *n* alleles, $\;S_{ni}$, can be determined as described in Section 2 of Supplementary File 1. Division by the sum of the harmonic series, $a_{n}=\sum_{j=1}^{j=n-1}\left(1/j\right)$, yields the expected values of Watterson's theta ($\theta_{wni}$) for each subpopulation (Watterson 1975). The skew of the distribution of segregating variants toward rare variants for subpopulation *i* can conveniently be measured by $\Delta \theta_{wni}=1-\pi_{i}/\theta_{wi}$ (Campos and Charlesworth 2019).

For practical purposes of computation, it is convenient to divide the range of values of *γ* into several zones according to the strength of selection and to compute the integrals of the load statistics over each zone separately. The overall values of the load statistics are then given by summing the results over all zones. The details are given in Section 1 of the Appendix, and the computer code for generating the numerical results for this case is given in Supplementary File 2.

### A finite island model metapopulation

In this case, a metapopulation of total size *N_T_* is divided into a large number *d* of subpopulations (demes), each of size *N* = *N_T_*/*d*. A Wright–Fisher model is assumed to apply to each deme. *N* is assumed to be sufficiently large that the frequency of the inversion is held at the same frequency *x* in all demes. A fraction *m* of each deme is derived by migration from a pool with equal contributions from all demes. Let the current mean frequency across all demes of the mutant allele A_2_ at a locus be ${\bar{q}}_{i}$ for karyotype *i*, so that migrants contribute *m*  ${\bar{q}}_{i}$ to the new frequency of A_2_ among type *i* haplotypes within a deme and *m*  ${\bar{p}}_{i}$ to the new frequency of A_1_.

This model poses the problem that the evolutionary processes within demes change the values of the ${\bar{q}}_{i}$, so that they cannot realistically be treated as fixed quantities. Following previous treatments of this problem, it is assumed here that the process of change in the ${\bar{q}}_{i}$ can be described by a pair of coupled diffusion equations, using the expectations of *p_i_q_i_*/(2*N_T_*) over all *d* demes as the drift variance terms together with the corresponding expectations of the expressions for the deterministic changes in ${\bar{q}}_{i}$ (Whitlock 2002; Cherry and Wakeley 2003; Roze and Rousset 2003; Wakeley 2003). The mutational contributions to the latter are simply obtained by substituting ${\bar{q}}_{i}$ for *q_i_* in the standard formulae for the deterministic changes in the ${\bar{q}}_{i}$, since the mutational changes are linear in *q_i_*. The expectation of *p_i_q_i_* over demes conditioned on ${\bar{q}}_{i}\;$ can be written as ${\bar{p}}_{i}{\bar{q}}_{i}(1\;\text{–}F_{STi})$, where $F_{STi}\;$ is the variance among demes in *q_i_* divided by ${\bar{p}}_{i}{\bar{q}}_{i}$. The total effective population size for the metapopulation for karyotype *i*, *N_emi_*, is thus equal to $N_{T}/(1\;\text{–}F_{STi})$ (Wakeley and Aliacar 2001).

The nonlinearity with respect to *q_i_* of the selection terms for allele frequency changes within demes (after division by *p_i_q_i_*) when *h* ≠ 0.5 means that an exact closed expression for their contributions to the expected changes in the ${\bar{q}}_{i}\;$ cannot be obtained, except in the absence of dominance (Whitlock 2002; Roze and Rousset 2003; Wakeley 2003). However, a useful approximation can be obtained by neglecting the 3rd moments about their means of the within-deme allele frequencies; these moments are necessarily smaller than the variances of the ${\bar{q}}_{i\;}$ and will be considerably smaller when selection is sufficiently strong in relation to drift that the ${\bar{q}}_{i\;}$ are close to 0. When selection is sufficiently weak, a neutral approximation for the 3rd moment can be used (Whitlock 2002), but this will in general overcorrect when selection is strong and so is not employed here. The error introduced by ignoring this correction affects only the small portion of the distribution of selection coefficients where *s* is *O*(1/2*N_T_*) and is thus unlikely to be important for the load and population genomic statistics calculated here.

The following expressions are obtained after some algebra:


(11a) $$\;\Delta_{s}{\bar{q}}_{1}\approx -s{\bar{p}}_{1}{\bar{q}}_{1}\{\left(1-F_{ST1})[h+(x{\bar{q}}_{1}+y{\bar{q}}_{2})]+(1-2h)xF_{ST1}(1-2{\bar{q}}_{1}\right)\}$$

(11b) $$\Delta_{s}{\bar{q}}_{2}\approx -s{\bar{p}}_{2}{\bar{q}}_{2}\{\left(1-F_{ST2})[h+(x{\bar{q}}_{1}+y{\bar{q}}_{2})]+(1-2h)yF_{ST2}(1-2{\bar{q}}_{2}\right)\}$$

We also have the following expression for the variance in ${\bar{q}}_{i}$ due to drift, e.g. Whitlock (2002):


(12) $$V_{\delta {\bar{q}}_{i}}={\bar{p}}_{i}{\bar{q}}_{i}(1\;\text{–}F_{STi})/(2N_{Ti})$$

where *N_T_*_1_ = *N_T_x* and *N_T_*_2_ = *N_T_y*.

Combining Equations (11) and (12), and using the approach that led to Equation (8) and carrying out some rearrangements of terms, we obtain the equivalent of the *ψ* function for the panmictic case, which describes the selection contribution to the logarithm of the p.d.f. for the metapopulation. The details are given in Section 2 of the Appendix. Section 5 of Supplementary File 1 describes how the p.d.f. can be used to obtain the load statistics. The computer code for generating the numerical results is given in Supplementary File 3.

## Results

### General considerations

Intuitively, the mutational load associated with each homokaryotype in an inversion polymorphism would be expected to be strongly affected by the dominance coefficient (*h*), the scaled strength of selection (2*Ns* = *γ*), the mutation rate to deleterious mutations (*u*), the number of selected sites that it contains *(n_s_*), and its frequency (*x*). Dominance coefficients less than one-half are well known to be required for inbreeding depression and heterosis, whose magnitudes are inversely related to *h* (Charlesworth and Charlesworth 2010, Chap. 4). It would thus be expected that the reduction in fitness associated with an arrangement, and any fitness advantage to *In*/*St* heterokaryotypes, would decrease with *h* but increase with *n_s_* and *u*, if *γ* is sufficiently small that deleterious mutations are significantly affected by drift.

It is less clear how these fitness effects are related to *γ*, since stronger selection reduces the frequencies of deleterious mutations but also reduces their effects on fitness if they rise to high frequencies. However, if selection is so strong in relation to drift that allele frequencies are at mutation–selection equilibrium, no differentiation in allele frequencies between *In* and *St* will occur, removing any possibility of a selective advantage to heterokaryotypes (Sturtevant and Mather 1938; Nei *et al*. 1967). The mean fitnesses of both homokaryotypes with *n_s_* selected sites will then be equal to the deterministic value, exp(−2*n_s_u*) ≈ 1 −2*n_s_u* when fitnesses are multiplicative and *h* is > 0 (Haldane 1937).

The rarer of the two arrangements experiences more genetic drift than its counterpart, so that *x* < 0.5 means that inversion homokaryotypes should have a lower overall mean fitness than standard homokaryotypes. It is less clear when an advantage to heterokaryotypes can be generated; the increased load associated with the rarer arrangement may simply generate a net selective advantage to its counterpart, especially when mutations are only partially recessive. Finally, it is likely that population subdivision will increase the magnitude of the mutational loads for each homokaryotype and the selective differences among the three karyotypes, because drift within demes in a metapopulation occurs at a faster rate than in a single population with the same size as the metapopulation. However, this is counterbalanced by a slower rate of drift for the metapopulation as a whole (Whitlock 2002; Cherry and Wakeley 2003; Wakeley 2003), so the net effect is hard to predict intuitively.

### A single population: load statistics

Numerical results for a single randomly mating population are presented here. These are based on Equations (1) for individual selected sites together with the procedures for combining the effects of mutation, drift, and selection for all sites that were described above. The number of sites was set to 10^5^, corresponding to an inversion containing 100 genes with a mean of 1,000 nonsynonymous sites per gene. The expectations of the single-locus mutational loads (*L_i_* and *L*_12_) and the inbreeding loads (*B_i_*), defined by Equations (1), are then multiplied by 10^5^ to obtain their net values. If multiplicative fitnesses are assumed, these quantities are equivalent to the negatives of the natural logarithms of the corresponding mean fitnesses (for the *L*s) or differences in log mean fitnesses (for the *B*s). The values of the corresponding mean fitnesses for a different number of sites, *n_s_*_1_, can be found by taking the exponentials of their negatives multiplied by *n_s_*_1_/10^5^. The selection coefficients *t_i_* against the 2 homokaryotypes are obtained by exponentiation of the product of *n_s_*_1_ and the expectation of *L_i_* – *L*_1*2*_ (Equation 10). If these products are small, as is mostly the case in practice, *t_i_* ≈ *n_s_* E{*L_i_* – *L*_12_}. Heterokaryotype advantage requires both of the *t_i_* to be positive; if one is positive and the other negative, there is directional selection in favor of the karyotype with the negative *t_i_*.

**Fig. 1.**
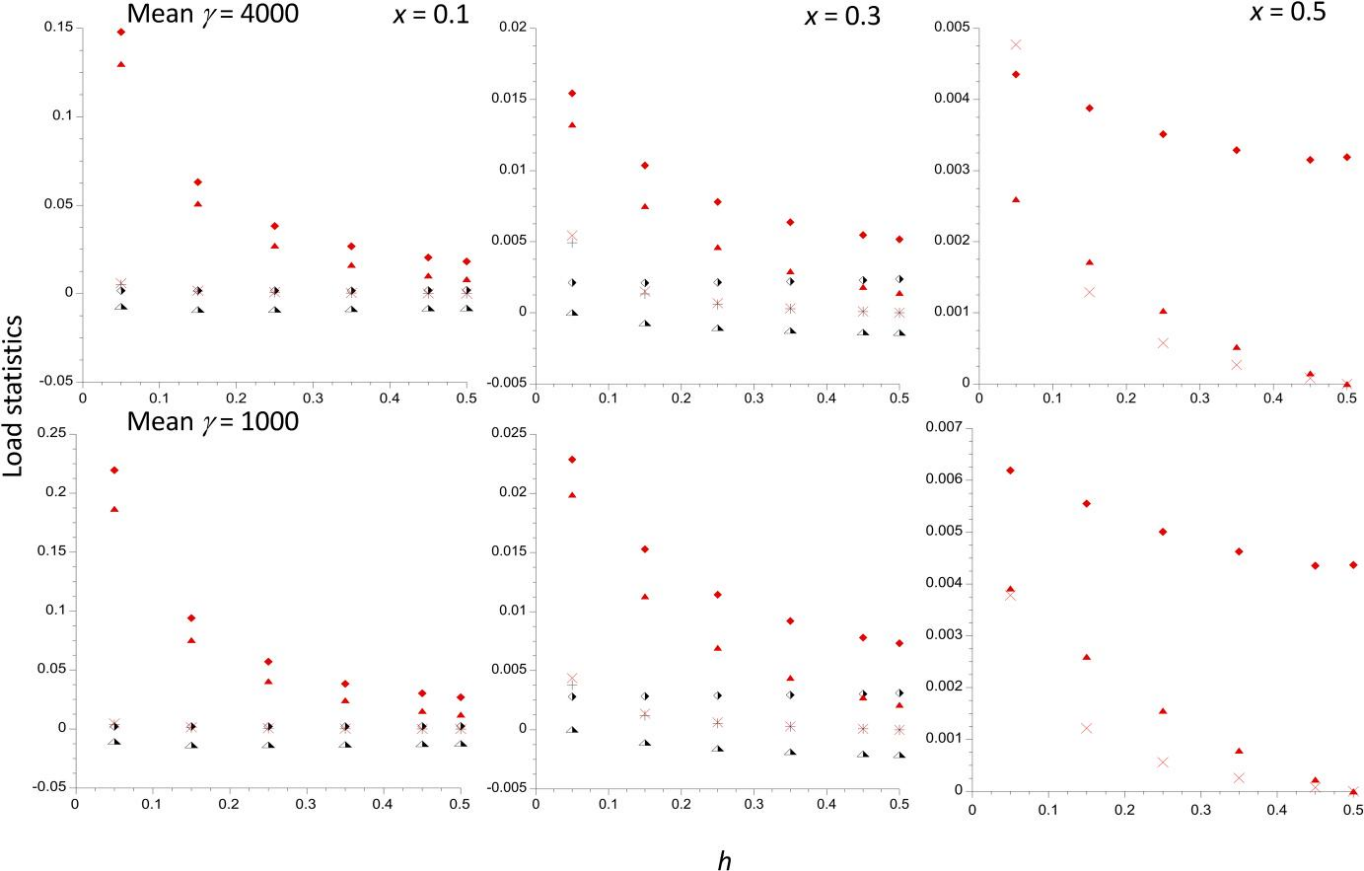
The mutational load statistics for a single population of size *N* = 10^6^ with 10^5^ selected sites, plotted against the dominance coefficient *h*. The results for 3 different frequencies of the inversion are shown. The selection coefficients follow a gamma distribution with a mean of 5 × 10^−4^ and a shape parameter of 0.3. The mutation rate to deleterious alleles is 5 × 10^–9^ per basepair, with a mutational bias toward deleterious variants of 1.5. The upper and lower panels have products of 2*N* and mean *s* of 4,000 and 1,000, respectively. The filled and half-filled symbols denote values for the *In* and *St* subpopulations, respectively. The lozenges are the net mutational loads within the respective karyotypes, and the crosses are the corresponding inbreeding loads. The triangles are the selection coefficients against homokaryotypes relative to the heterokaryotype. Only the values for *In* are shown when *x* = 0.5.

Figure 1 displays the values of the relevant load statistics and the selection coefficients against homokaryotypes as a function of the dominance coefficient *h*, for three different inversion frequencies and two different mean scaled selection coefficients ($\bar{\gamma }\;$= 1,000 and 4,000), using a gamma distribution of selection coefficients with shape parameter *a* = 0.3, and a mutation rate to deleterious alleles of *u* = 5 × 10^−9^. These values are broadly consistent with population genomic estimates from *Drosophila melanogaster* (Charlesworth 2015). A strong mutational bias to deleterious mutations of *κ* = 1.5 was assumed, in order to maximize the magnitude of the mutational loads and selection coefficients.

If there were no effects of drift, the net load (*L*) for both subpopulations would be approximately 2*n_s_u* = 0.001, and the net inbreeding load (*B*) would equal *n_s_u*(*h*^−1^–2)—see Charlesworth and Charlesworth (2010, Chap. 4). This gives *B* = 0.002 with *h* = 0.25 and 0.036 with *h* = 0.05, with zero selection coefficients for both homokaryotypes. The numerical results show that the loads for both *In* and *St* subpopulations are always substantially larger than the deterministic values, even with *h* = ½ (Supplementary Table 1). With *x* = 0.1 and $\bar{\gamma }\;$ = 1,000, *L*_1_, the load for *In* is more than 20-fold greater than the deterministic value for *h* between 0.05 and 0.5. Both *L* values are higher for the lower $\bar{\gamma }\;$ values, as might be expected from the larger effects of drift in this case. Similarly, with *x* < ½, *L*_1_ is always larger than *L*_2_, and decreases with *x* for a given *h* and $\bar{\gamma }\;$. Furthermore, *L*_1_ strongly decreases with *h* for *x* = 0.1 and 0.3, whereas *L*_2_ is only slightly affected by *h*. The fact that *L*_1_ < *L*_2_ with *h* = ½ and *x* < ½ shows that the increase in load due to smaller population size is partly caused by increased mean frequencies of deleterious mutations, not simply by increased frequencies of homozygotes.

In contrast, the *B_i_* values are always lower than the deterministic values, reflecting the reduction in variability caused by drift, and are decreasing functions of *h* (vanishing when *h* = 0.5), although this is hard to see from Fig. 1 due to their overall very small values. These effects are especially noticeable for *B*_1_ when *x* = 0.1 and $\bar{\gamma }\;$ = 1,000. The small *B_i_* values reflect the fact that, except for the lowest dominance coefficient (*h* = 0.05), the *L_i_* are always quite close to the corresponding homozygous loads (the *H_i_* of Equation 1c). For example, with $\bar{\gamma }\;$ = 1,000 and *x* = 0.1, for *h* = 0.05, we have *L*_1_ = 0.212, *H*_1_ = 0.224, *L*_2_ = 0.002, *H*_2_ = 0.006; for *h* = 0.25, we have *L*_1_ = 0.057, *H*_1_ = 0.058, *L*_2_ = 0.002, *H*_2_ = 0.003.

There can be substantial selection against the inversion homokaryotypes at the lower *h* values, especially for the smaller *x* values: *t*_1_ reaches 0.19 with *h* = 0.05 and $\bar{\gamma }\;$ = 1,000, but decreases sharply with *x* and *h*; it is only 0.04 for *h* = 0.25, *x* = 0.1, and $\bar{\gamma }\;$ = 1,000. The values for $\bar{\gamma }$ = 4,000 are somewhat smaller, consistent with smaller effects of drift in causing divergence between the 2 subpopulations. In contrast, *t*_2_ is mostly negative and quite small (approximately −0.01 for a wide range of *h* values when *x* = 0.1 and $\bar{\gamma }\;$ = 1,000), indicating weak directional selection against the inversion. Only when *x* is close to 0.5 does the heterokaryotype experience a slight advantage over both homokaryotypes (a maximal value of *t*_1_ = *t*_2_ ≈ 0.004 for *h* = 0.05 and $\bar{\gamma }$ = 1,000). With *x* = 0.5, the selective advantage to heterokaryotypes declines sharply with *h*, and is only 0.0016 for *h* = 0.25 and $\bar{\gamma }$ = 1,000; it vanishes when *h* = 0.5. With 10^6^ instead of 10^5^ selected sites, the heterokaryotype advantage would be approximately 10 times as large. With *x* = 0.5, symmetry implies that *t*_1_ = *t*_2_, and the equality of effective population sizes means that the mean fitness of the heterokaryotypes is superior to that of the homokaryotypes purely because they have a lower frequency of mutant homozygotes than either of the homokaryotypic subpopulations.

**Fig. 2.**
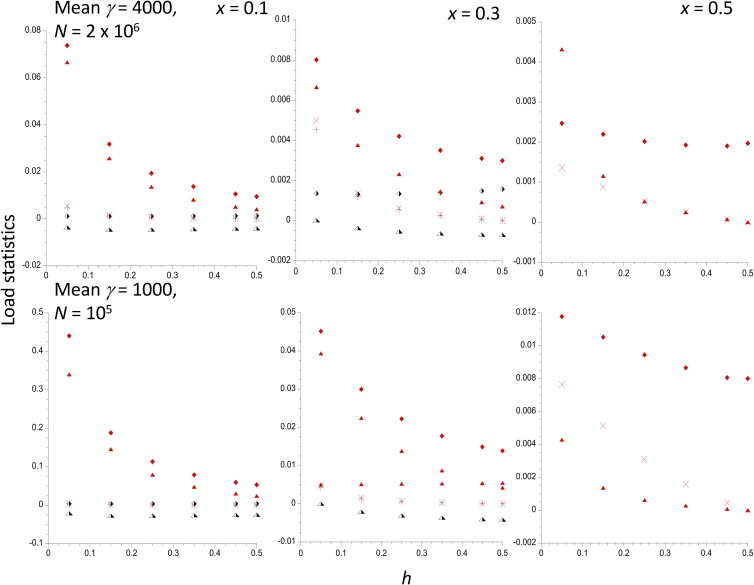
The mutational load statistics for a single populations of size *N* = 2 × 10^6^ (upper panel) and *N* = 10^5^ (lower panel), with the same mean selection coefficient (0.001), plotted against the dominance coefficient *h*. The results for 3 different frequencies of the inversion are shown. The other parameters are the same as in Fig. 1. The filled and half-filled symbols denote values for the *In* and *St* subpopulations, respectively. The lozenges are the net mutational loads within the respective karyotypes, and the crosses are the corresponding inbreeding loads. The triangles are the selection coefficients against homokaryotypes relative to the heterokaryotype. Only the values for *In* are shown when *x* = 0.5.

It is also of interest to examine the effect of differences in population size and selection coefficients on the load statistics for a constant scaled selection strength. Figure 2 is similar to Fig. 1, but with population sizes of 2 × 10^6^ and 10^5^, and a mean selection coefficient (0.001) that is one-half of that in Fig. 1. A comparison of the top panels of Figs. 1 and 2 shows that reducing the mean selection coefficient by one-half, but keeping $\;\bar{\gamma }\;$ constant, results in a reduction in the *L* values, by a factor of close to 2 for the case of the *In* subpopulation with *x* = 0.1, but by considerably less for the *St* population, where *L*_2_ is only slightly greater than the deterministic value of 0.001, even for *h* = 0.05. The relative effect on a subpopulation is reduced as it increases in size, as might be expected intuitively. These effects reflect the fact that the load caused by drift is greater when the selection coefficients involved are larger (for fixed $\bar{\gamma }\;$), in contrast to the deterministic formula *L* = 2*u*. The two selection coefficients on homokaryotypes relative to heterokaryotypes, *t_i_*, show a similar pattern. In contrast, the inbreeding loads are only slightly reduced by a reduction in the strength of selection when $\bar{\gamma }\;$ is fixed. Further results for different deme sizes are shown in Supplementary Table 2.

The effect of a reduced population size while holding mean *s* constant can be seen by comparing the upper and lower panels of Fig. 2. As might be expected from the greater effects of drift in a smaller population, the *L_i_* and *t_i_* are considerable larger when the population size is reduced by a factor of 20, especially for the inversion subpopulation with *x* = 0.1, for which *N*_1_ = 10,000 in the lower panel. However, with *x* = 0.5 and *h* = 0.25, the selective advantage to the heterokaryotypes is only 0.0003 for the smaller population size. The inbreeding loads are barely affected by the population size difference.

### A single population: population genomics statistics

**Fig. 3.**
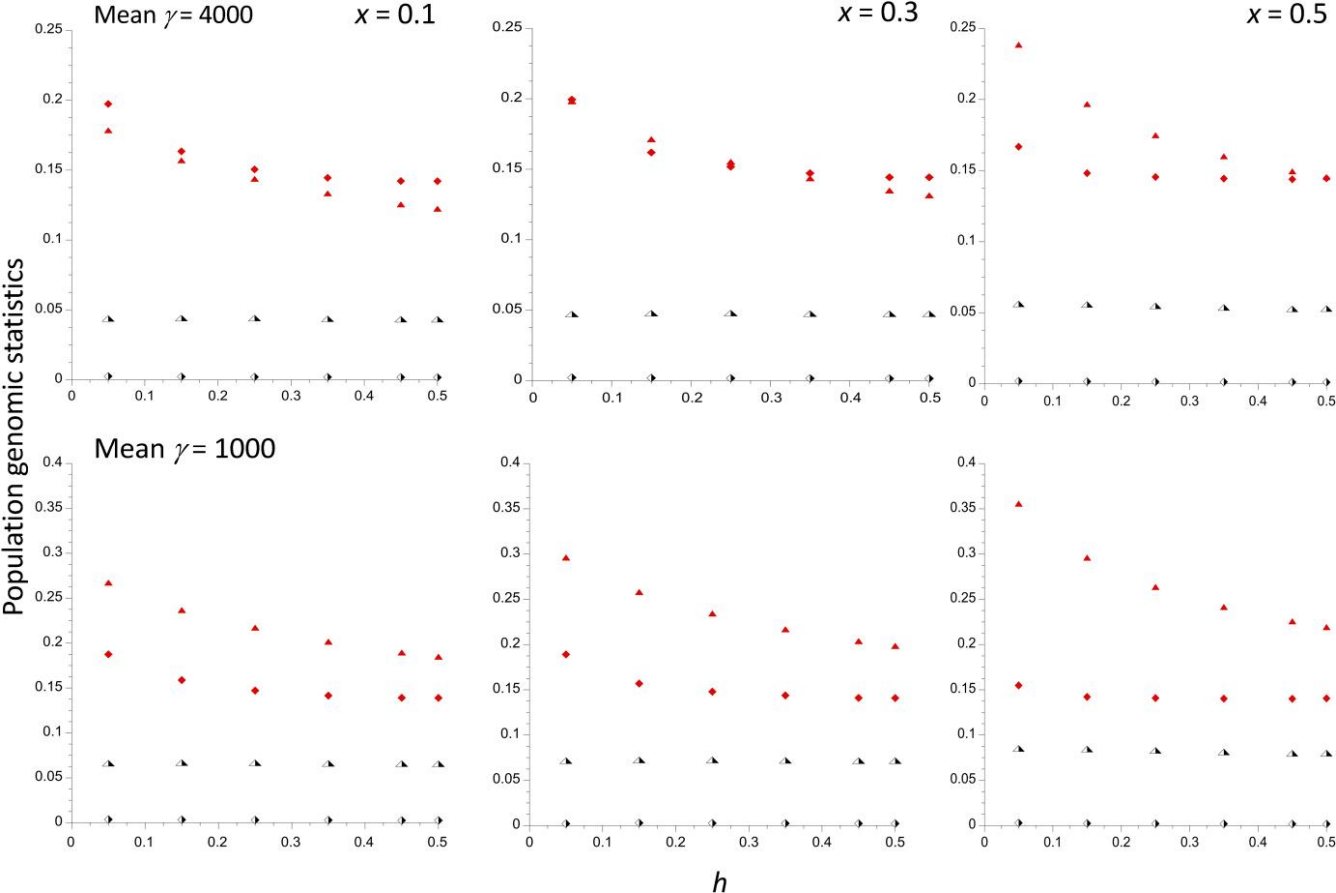
The population genomics statistics for the *St* subpopulation, plotted against the dominance coefficient *h*. The evolutionary parameters are the same as in Fig. 1. The results for 3 different frequencies of the inversion are shown. The half-filled triangles are the mean frequencies of A_2_ and the half-filled lozenges are the mean diversities at selected sites. The filled triangles are the ratios of mean diversities at selected sites to mean diversities at neutral sites. The filled lozenges are the values of Δ*θ_w_* for the selected sites. Note that the distinction between filled and half-filled symbols does not refer to the *In* vs *St* subpopulations, as was the case for the previous figures.

It is also of interest to examine the effects of an inversion polymorphism on population genomics statistics that can be used to assess the effects of the differences in effective population size between the *In* and *St* subpopulations, and between these and the part of the genome that is independent of the inversion polymorphism. Figure 3 shows the results for the *St* population using the same parameter values as in Fig. 1 for 3 different values of *x*; the case with *x* = 0.1 is nearly equivalent to the situation for the rest of the genome. It can be seen, somewhat surprisingly, that the mean frequency of deleterious alleles in the *St* subpopulation (${\bar{q}}_{2}$, half-filled triangles), which includes both fixed and segregating sites, is nearly independent of *h*. It increases slightly as the size of the *St* population, given by *N*_2_ = *N*(1 – *x*), decreases as *x* changes from 0.1 to 0.5; the effect of *N*_2_ is somewhat greater for $\bar{\gamma }\;$ = 1,000, as would be expected from the greater effects of drift in this case. This difference partly reflects the higher proportion of sites that can become fixed for deleterious mutations with weaker selection, as well as their higher mean frequency at segregating sites. Its behavior implies that the effect of population size on the load for a subpopulation when the mean strength of selection is strong is largely caused by changes in the variance of allele frequency rather than its mean.

The mean nucleotide site diversity at the selected sites for *St* ($\pi_{\mathrm{sel}2}$, half-filled lozenges) decreases somewhat with increasing *h* and decreasing *N*_2_, but is always between 0.0011 and 0.0025 for $\bar{\gamma }\;$ = 4,000 and between 0.0017 and 0.0037 for $\bar{\gamma }\;$ = 1,000. The ratio of $\pi_{\mathrm{sel}2}$ to the mean nucleotide site diversity at neutral sites ($\pi_{\mathrm{sel}2}/\pi_{\mathrm{neut}2}$, filled triangles) decreases with *N*_2_, especially when *h* is small. The measure of skew toward low-frequency variants at selected sites, Δ*θ_w_*_2_, for a sample of 20 haploid genomes (filled lozenges) increases with *N*_2_, but the effect is weak unless *h* is small; the effect of *h* on Δ*θ_w_*_2_ is smaller than on $\pi_{\mathrm{sel}2}/\pi_{\mathrm{neut}2}$, and plateaus around *h* = 0.25 (for the definition of Δ*θ_w_*, see *A single population: modeling drift and selection*).

**Fig. 4.**
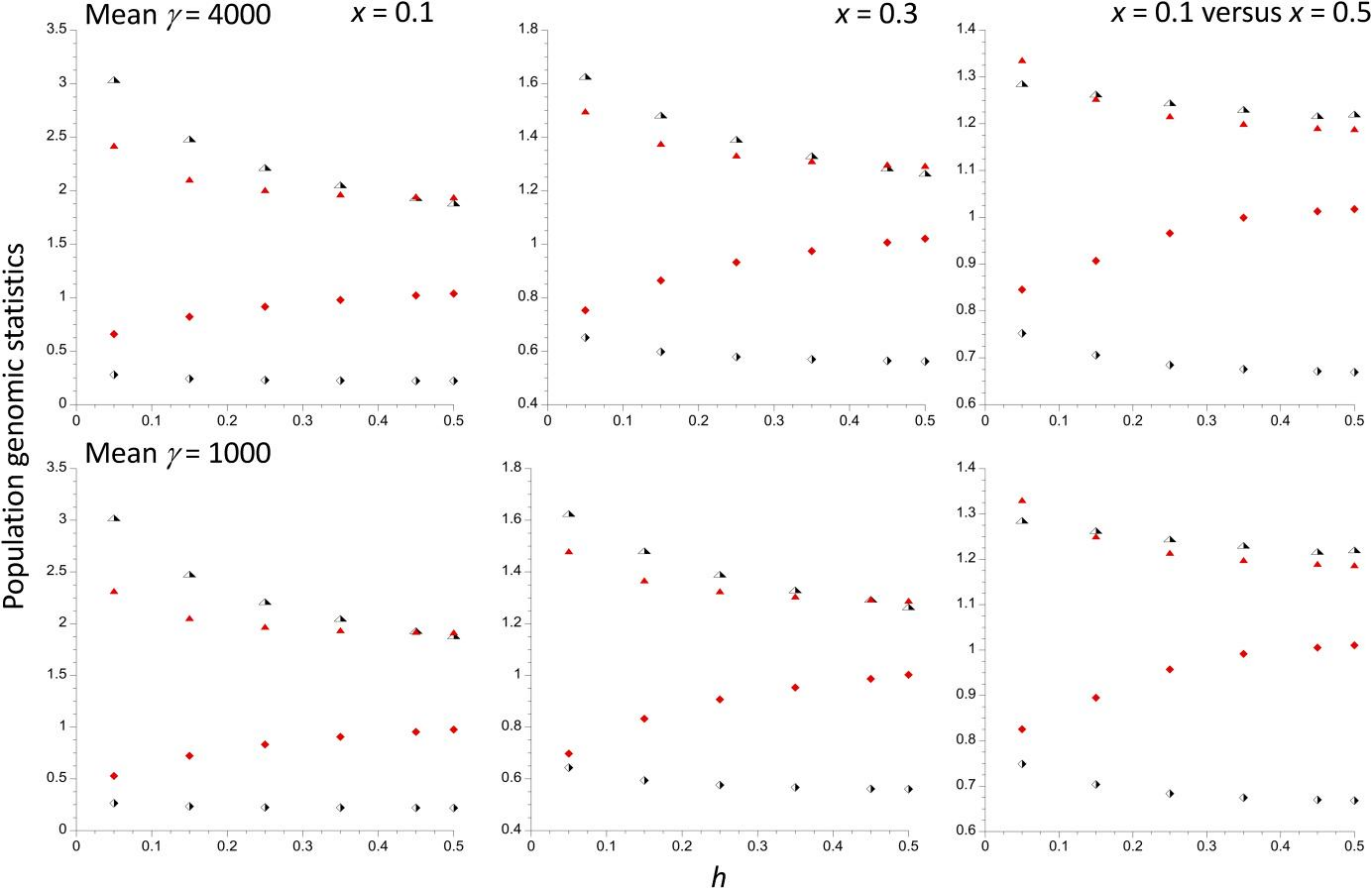
The first 2 panels in each row show the ratios of the population genomics statistics for the *In* subpopulation to those for the *St* subpopulation, plotted against the dominance coefficient *h*. The third panel shows the ratios of these statistics for the *St* subpopulation with an inversion frequency of 0.5 to their values for a frequency of 0.1, for which the corresponding ratio of population sizes is 0.556. The evolutionary parameters are the same as in Fig. 1. The half-filled triangles are the ratios of the mean frequencies of A_2_; the half-filled lozenges are the ratios of the mean diversities at selected sites. The filled triangles are the ratios of mean diversities at selected sites to mean diversities at neutral sites. The filled lozenges are the ratios of Δ*θ_w_* values for the selected sites.

These effects of *h* and *N*_2_ can also be seen in the right-hand panels of Fig. 4 (labeled as *x* = 0.5 vs *x* = 0.1) which plot the ratios of ${\bar{q}}_{2},$  $\pi_{\mathrm{sel}2}$, $\pi_{\mathrm{sel}2}/\pi_{\mathrm{neut}2\;}$, and $\Delta \theta_{w2}$ with *x* = 0.5 to their values with *x* = 0.1, a 1.8-fold difference in *N*_2_. The plots for $\pi_{\mathrm{sel}2}/\pi_{\mathrm{neut}2\;}$ bring out clearly that a lower subpopulation size is associated with larger $\pi_{\mathrm{sel}2}/\pi_{\mathrm{neut}2\;}$, especially when *h* is small. For $\Delta \theta_{w2}$, the ratio is approximately 0.85 for *h* = 0.05, but increases rapidly toward 1 as *h* increases. The 4-fold difference in $\bar{\gamma }$ between the upper and lower panels has a remarkably small effect on the ratios for all 4 statistics.

The other 2 panels of Fig. 4 display plots against *h* of the ratios of these statistics for the *In* subpopulation to their values for the *St* subpopulation for *x* = 0.1 and 0.3. The ratios of population sizes for *St* vs *In* are 9-fold for *x* = 0.1 and 2.3-fold for *x* = 0.3, flanking the ratio *N*_2_/*N*_1_ for the right-hand panels. *N*_2_ for *x* = 0.3 is 1.4-fold greater than *N*_2_ for *x* = 0.5, which enhances the contrast between *In* and *St*. Accordingly, the patterns are more marked than for the right-hand-most panels, and are strongest for the case with *x* = 0.1, with a maximal ratio of $\pi_{\mathrm{sel}1}/\pi_{\mathrm{neut}1\;}$ to $\pi_{\mathrm{sel}2}/\pi_{\mathrm{neut}2\;}$ of 2.4 at *h* = 0.05 and $\bar{\gamma }\;$ = 4,000. This is still much less than the ratio of 9 for neutral diversity. The ratio Δ*θ_w_*_1_/Δ*θ_w_*_2_ with *x* = 0.1 is close to 0.5 for both $\bar{\gamma }\;$ values when *h* = 0.05, but rapidly becomes close to 1 as *h* increases—for *x* = 0.1, *h* = 0.25, and $\bar{\gamma }\;$ = 4,000, we have Δ*θ_w_*_1_/Δ*θ_w_*_2_ = 0.915. The skew in the site frequency spectrum at selected sites is thus unlikely to be a powerful statistic for detecting a reduced efficacy of selection on a low-frequency inversion, given that very small *h* values are unlikely for mildly deleterious mutations (Crow 1993; Manna *et al*. 2011).

### A subdivided population

As described in *General considerations, A finite island model metapopulation*, this case assumes that a metapopulation of total size *N_T_* is divided into a large number *d* of demes, each of size *N* = *N_T_*/*d*. A Wright–Fisher model applies to each deme, and the deme size is assumed to be sufficiently large that the frequency of the inversion is held at the same frequency *x* in all demes. A fraction *m* of each deme is derived by migration from a pool with equal contributions from all demes. For sites that are independent of the inversion, the level of neutral genetic differentiation between demes is described by $F_{STn}\approx 1/(1+M)$, where *M* is the scaled mutation rate 4*Nm*. Different levels of subdivision are characterized by different values of *F_STn_*; for simplicity, the subscript *n* is dropped in what follows. The scaled selection parameter *γ* is now defined as 2*N_T_s*, and the scaled mutation parameters are *α* = 4*N_T_u* and *β* = 4*N_T_v*.

**Fig. 5.**
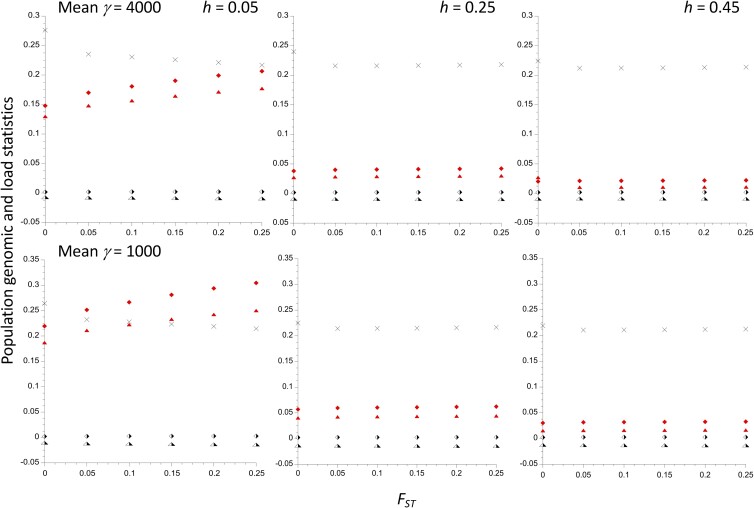
The mutational load and polymorphism statistics for an inversion polymorphism with inversion frequency *x* = 0.1 in a subdivided island population of total size *N_T_* = 10^6^ with 200 demes and 10^5^ selected sites, plotted against *F_ST_* for neutral sites independent of the inversion. The results for 3 different values of the dominance coefficient, *h*, are shown. The selection coefficients follow a gamma distribution with a mean of 5 × 10^−4^ and a shape parameter of 0.3. The mutation rate to deleterious alleles is 5 × 10^–9^ per basepair, with a mutational bias toward deleterious variants of 1.5. The upper and lower panels have products of 2*N_T_* and mean *s* of 4,000 and 1,000, respectively. The filled and half-filled symbols denote values for the *In* and *St* subpopulations, respectively. The lozenges are the net mutational loads within the respective karyotypes, and the triangles are the selection coefficients against homokaryotypes relative to the heterokaryotype. The crosses are the ratios of the nucleotide site diversities for *In* vs*St* subpopulations.

Figure 5 shows the effects of population subdivision on the load statistics of most interest, together with the ratio of the diversities at selected sites for the *In* vs *St* subpopulations. There is an inversion frequency of 0.1 in a metapopulation of 200 demes with a total size *N_T_* = 10^6^ (*N* = 5,000); the selection and mutation parameters are the same as in Fig. 1. The results for *F_ST_* = 0 were obtained from the single population calculations described above. The results can be summarized very simply: there is a remarkably small effect of population subdivision as *F_ST_* changes from 0 to 0.25, with the most marked effect occurring over the change from *F_ST_* = 0 to *F_ST_* = 0.05, especially for the smallest dominance coefficient (*h* = 0.05). For example, with $\bar{\gamma }$ = 1,000 and *h* = 0.05, the selection coefficients against the *In* and *St* homokaryotypes relative to the heterokaryotype change from *t*_1_ = 0.1868 and *t*_2_ −0.0107 with *F_ST_* = 0 to 0.211 and −0.0120 with *F_ST_* = 0.05, reaching 0.250 and −0.0142 at *F_ST_* = 0.25. With the more plausible value of *h* = 0.25, the changes are much smaller: *t*_1_ = 0.0405 and *t*_2_ −0.0136 at *F_ST_* = 0, and 0.0442 and −0.0146 at *F_ST_* = 0.25. Both the absolute values of the loads and selection coefficients and their dependence on *F_ST_* are much smaller with $\bar{\gamma }$ = 4,000 than 1,000. As in the case of a single population, a selective advantage to the heterokaryotype is not found unless there are nearly equal frequencies of *In* and *St* (Supplementary Table 3). The magnitude of such an advantage is not greatly increased by subdivision; for example, with *x* = 0.5, $\bar{\gamma }$ = 1,000 and *h* = 0.05, *t*_1_ = *t*_2_ = 0.0039 at *F_ST_* = 0.0, and 0.00860 at *F_ST_* = 0.25; with *h* = 0.25, the corresponding values are 0.00160 and 0.00196.

The relation with *F_ST_* of the ratio of diversities at selected sites for *In* vs *St* with *x* = 0.1 is also shown in Fig. 5, and is similarly rather weak. This finding also applies to the population genomic statistics that are not displayed in Fig. 5—see Supplementary Table 3 for details (the Δ*θ_w_* statistic was not calculated, as this statistic is hard to evaluate with population subdivision and was not very informative in the single population case). For example, with *x* = 0.1, $\bar{\gamma }$ = 1,000 and *h* = 0.05, the ratio of $\pi_{\mathrm{sel}}/\pi_{\mathrm{neut}\;}$ for *In* vs *St* decreases from 2.32 with *F_ST_* = 0 to 1.80 with *F_ST_* = 0.25, indicating a greater efficacy of selection on the smaller subpopulation when there is greater subdivision. With *h* = 0.25, the change is much smaller, from 1.97 to 1.90. Similarly, the ratio of the mean frequency of A_2_ for *In* vs *St* with *h* = 0.05 changes from 3.02 with *F_ST_* = 0 to 3.78 with *F_ST_* = 0.25, but only from 2.21 to 2.32 with *h* = 0.25.

These results are all for a relatively large deme size of *N* = 5,000. Intuitively, it might seem that reducing the deme size would enhance the effects of drift within demes, and lead to larger loads and magnitudes of the selection coefficients, as well as reducing the values of such signatures of purifying selection as the mean frequencies of mutant alleles and the ratios of diversities at selected sites to neutral sites. The example in the upper part of Table 2, where deme sizes of 500 and 5,000 are compared for the same neutral *F_ST_* value, shows that this expectation is met for the case of equal frequencies of *In* and *St*. For the mean fitnesses of the homokaryotypes and the selection coefficients, the effects are small and are only visible in the table in a few cases. However, the mean frequencies of mutations and the ratios of diversity at selected sites vs neutral sites show a clear pattern of reduced efficacy of selection with the smaller deme size, which is reduced in magnitude by larger *h* and *F_ST_*. Further results for cases with smaller deme sizes are shown in Supplementary Table 4.

**Table 2.** Some load and population genomics statistics for a subdivided population of total size 10^6^ for 2 different local deme sizes.

*x* = 0.5	${\bar{w}}_{1}$	*t*_1_ = *t*_2_	${\bar{q}}_{1}$ = ${\bar{q}}_{2}$	*π*_sel1_/*π*_neut1_
*F_ST_* = 0.05, *h* = 0.05	0.992 0.992	0.005 0.005	0.090 0.088	0.341 0.325
*F_ST_* = 0.05, *h* = 0.25	0.994 0.998	0.002 0.002	0.087 0.085	0.262 0.254
*F_ST_* = 0.05, *h* = 0.45	0.995 0.995	0.0003 0.0003	0.086 0.083	0.229 0.226
*F_ST_* = 0.25, *h* = 0.05	0.987 0.987	0.090 0.083	0.101 0.099	0.263 0.258
*F_ST_* = 0.25, *h* = 0.25	0.994 0.998	0.002 0.002	0.089 0.087	0.238 0.232
*F_ST_* = 0.25, *h* = 0.45	0.995 0.995	0.0003 0.0003	0.086 0.084	0.228 0.221

*x* = 0.1	${\bar{w}}_{1}$ /${\bar{w}}_{2}$	*t*_1_	*t*_2_	${\bar{q}}_{1}$ /${\bar{q}}_{2}$	(*π*_sel1_/*π*_neut1_) /(*π*_sel2_/*π*_neut2_)
*F_ST_* = 0.05, *h* = 0.05	0.780 0.780	0.210 0.211	−0.012 −0.012	3.23 3.29	2.17 2.04
*F_ST_* = 0.05, *h* = 0.25	0.944 0.944	0.042 0.042	−0.014 −0.014	2.26 2.29	1.93 1.88
*F_ST_* = 0.05, *h* = 0.45	0.971 0.970	0.016 0.016	−0.013 −0.013	1.93 1.96	1.88 1.85
*F_ST_* = 0.25, *h* = 0.05	0.739 0.739	0.250 0.250	−0.014 −0.014	3.72 3.78	1.83 1.88
*F_ST_* = 0.25, *h* = 0.25	0.941 0.942	0.045 0.042	−0.015 −0.015	2.29 2.32	1.91 1.90
*F_ST_* = 0.25, *h* = 0.45	0.969 0.970	0.017 0.016	−0.014 −0.014	1.93 1.96	1.90 1.86

Results for *N* = 500 and 5,000 are shown in the upper and lower parts of each cell, respectively.

${\bar{w}}_{1}$ and ${\bar{w}}_{2\;}$ are the mean fitnesses of the products of random mating among *In* and *St* karyotypes, respectively.

The selection and mutation parameters are the same as in Fig. 1.

The patterns are somewhat more complex, however, when there is a large difference in frequency between arrangements, as shown in Table 2 for the case of *x* = 0.1. Here, the differences in the load statistics between the large and small deme size cases are negligible. There is, however, a signature of a slightly enhanced efficacy of selection with the smaller deme size in the *In* subpopulation relative to *St*, indicated by a consistently smaller value of ${\bar{q}}_{1}$/${\bar{q}}_{2}$ when *N* = 500. Confusingly, $\pi_{\mathrm{sel}1}$/$\pi_{\mathrm{neut}1}$ is greater than $\pi_{\mathrm{sel}2}$/$\pi_{\mathrm{neut}2}$ when *N* = 500, indicating the opposite pattern. The first of these results is explained by the fact that the much smaller size of the *In* subpopulation means that mutations are behaving nearly neutrally within demes, so that a lower deme size will not have much of an effect, whereas it will reduce the overall efficacy of selection on the *St* subpopulation, resulting in an increase in ${\bar{q}}_{2}$. For example, with *h* = 0.05 and *F_ST_* = 0.05, the ratio of ${\bar{q}}_{2}$ for *N* = 500 vs ${\bar{q}}_{2}$ for *N* = 5,000 is 1.024, whereas the value for ${\bar{q}}_{1\;}$ is 1.006. The corresponding ratio for $\pi_{\mathrm{sel}1}$/$\pi_{\mathrm{neut}1}\;$is 1.072 whereas that for $\pi_{\mathrm{sel}2}$/$\pi_{\mathrm{neut}2}$ is 1.005. This is entirely due to a higher ratio of $\pi_{\mathrm{sel}1}$ for *N* = 500 vs *N* = 5,000, since there is no effect of local deme size on neutral diversity for a fixed neutral *F_ST_*. It is not entirely clear how to interpret this pattern, but one factor is likely to be the fact that the relaxation of the efficacy of selection in *St* subpopulation leads to an increase in *F_ST_* at selected sites, which would work against any increase in $\pi_{\mathrm{sel}2}$ due to a reduced efficacy of selection.

## Discussion

It should be borne in mind that the results described above relate to a situation in which an inversion polymorphism is maintained by balancing selection that is invariant over space and sufficiently strong that the frequency of the arrangements is constant over time and space. This obviously does not apply to many natural situations, e.g. when there are clinal patterns of variation in inversion frequencies or temporal fluctuations in frequencies, as is often the case (Krimbas and Powell 1992; Kapun and Flatt 2019). Nevertheless, the theoretical results described above address several questions that can in principle be answered by comparisons with empirical studies. First, does a low-frequency arrangement accumulate a larger mutational load than its counterpart? Second, can mutational load contribute a significant selective advantage to heterokaryotypes, which might help to stabilize the polymorphism? Third, can differences in population genomic statistics between common and rare arrangements shed light on differences in mutational load between arrangements? These questions are each discussed in turn below. The focus will be on the results for a single population, since the analysis of the properties of a subdivided population showed that there were no major differences from those of a single population over the range of *F_ST_* values examined, although subdivision tends to slightly magnify the differences between rare and common arrangements.

### Are the relative values of the fitness components of carriers of different gene arrangements consistent with mutational load?

The relevant variables with respect to the relative fitnesses of the *In* and *St* subpopulations are the *L_i_*, the mutational loads associated with individuals produced by random mating among outbred individuals homozygous for karyotype *i*, where *i* = 1 for the rarer arrangement (*In*) and *i* = 2 for the more common one (*St*). These quantities are, course not directly observable, since the fitness of mutation-free individuals is unknown, but they can be used to predict the relative values of the mean fitnesses (or fitness components) for *In* and *St*. On the assumption of multiplicative fitnesses, the ratio of the mean fitnesses of type 1 and type 2 homokaryotypes is ${\bar{w}}_{1}$/${\bar{w}}_{2}=\exp(L_{2}-L_{1})$, corresponding to a difference *L*_2_ – *L*_1_ in the natural logarithm of mean fitness. The mutational and selection parameters used here (a mutation rate of 5 × 10^–9^ per site and a gamma distribution of selection coefficients with shape parameter 0.3) are consistent with the results of population genomic studies of *D. melanogaster*, e.g. Kousathanas and Keightley (2013) and Wang *et al*. (2023).

In order to relate the theoretical predictions to data on inversion polymorphisms, it is necessary to have information about *h* and the parameters of the distribution of *s*, which is made difficult by the fact that there is much uncertainty about the relation between *h* and *s*. The evidence from *Drosophila* studies of the effects of mutations on fitness components suggest that only very strongly selected deleterious mutations such as homozygous lethals have *h* values low as 0.05, whereas the much more abundant deleterious mutations with *s* ≤ 0.02 have *h* values of the order of 0.25 or more (Crow 1993; Manna *et al*. 2011). It therefore seems safe to use *h* = 0.25 as a working value for comparing theory with data, since mutations like lethals that are strongly selected against when heterozygous will be held close to their deterministic equilibrium values in both the *In* and *St* subpopulations (Nei 1968), and hence will not contribute to the genetic differences between them. It is worth noting that a higher load for the rarer arrangement does not require mutations to have *h* < ½, contrary to what is often stated (e.g. Jay *et al*. 2021). If drift is sufficiently strong in relation to selection that there is a higher expected frequency of deleterious mutations in the smaller subpopulation, a higher load can arise even for completely dominant mutations.

Estimates of the distribution of *γ* = 2*N_e_s* from population genomics studies reflect the more weakly selected part of the distribution of selection coefficients and are thus the most useful source of information for the present purpose. With a shape parameter of 0.3 and *h* = 0.25, $\pi_{\mathrm{sel}2}$/$\pi_{\mathrm{neut}2}=0.15$ with $\bar{\gamma }$ = 4,000 when *x* = 0.1. This is only slightly higher than the observed ratios of nonsynonymous to silent site diversities in normally recombining regions of the genome in putatively ancestral range populations of *D. melanogaster* (e.g. Campos *et al*. 2014), suggesting that this value of $\bar{\gamma }$ can be used as a working estimate for nonsynonymous mutations.

One of the best-studied *D. melanogaster* inversions is *In(3R)P*. This has a frequency of 0.1 in populations in its ancestral range in Africa (Kapun and Flatt 2019), which are the most relevant populations for comparisons with the theoretical predictions. It is in the middle of the size range for polymorphic inversions in this species, covering approximately 8 Mb of sequence (Kapun *et al*. 2023). This corresponds to approximately 1,000 protein-coding sequences, i.e. 10^6^ nonsynonymous sites rather than the 10^5^ selected sites illustrated in the figures. Functional noncoding sequences may also contribute to the mutational load, and these appear to be under weaker selective constraints than nonsynonymous mutations (Andolfatto 2005; Casillas *et al*. 2007; Campos *et al*. 2017). As a rough estimate of their contribution to the load and population genomic statistics, it is plausible to assume that functional noncoding sites are three times as abundant as nonsynonymous sites (Halligan and Keightley 2006) but have $\bar{\gamma }$ = 1,000 rather than 4,000. With *x* = 0.1 and *h* = 0.25, the results in Supplementary Table 1 imply that, by combining the effects of 10^6^ nonsynonymous sites with $\bar{\gamma }$ = 4,000 and 3 × 10^6^ noncoding sites with $\bar{\gamma }$ = 1,000 and *h* = 0.25, we would have *L*_1_ = 2.09 and *L*_2_ = 0.08 for an inversion with frequency 0.1, giving a predicted ${\bar{w}}_{1}$/${\bar{w}}_{2}\;$= 0.13.

Predictions of this kind are highly sensitive to the frequency of the inversion, and to assumptions concerning the abundance of functional sites and the strength and mode of selection. With the model just described and an inversion frequency of 0.3, *L*_1_ = 0.42 and *L*_2_ = 0.11, giving ${\bar{w}}_{1}$/${\bar{w}}_{2}\;$= 0.73. If only nonsynonymous sites are considered, ${\bar{w}}_{1}$/${\bar{w}}_{2}$ rises to 0.69 for *x* = 0.1 and 0.95 for *x* = 0.3. Synergistic epistasis among deleterious mutations is expected to reduce the *L_i_* by a factor of approximately 2 (Kondrashov 1995; Charlesworth 2013), so that the model of a mixture of 10^6^ nonsynonymous and 3 × 10^6^ functional noncoding sites would generate ${\bar{w}}_{1}$/${\bar{w}}_{2}$values of 0.37 and 0.86 for *x* = 0.1 and *x* = 0.3, respectively. These complexities means that it is difficult to make rigorous comparisons between theory and data, and the estimate of ${\bar{w}}_{1}$/${\bar{w}}_{2}=\;$0.13 for *In(3R)P* is probably at the lower end of what is to be expected.

A large number of studies of the effects of inversions on fitness and components of fitness under laboratory conditions have been published, with *D. melanogaster* and *D. pseudoobscura* being been the most intensively studied species—see reviews by Sperlich and Pfriem (1986), Krimbas and Powell (1992), and Kapun and Flatt (2019). Many of these studies do not, however, provide useful information for the present purpose, either because they involve traits like male mating success that are hard to quantify in terms of relative fitness measures, because only small numbers of independently sampled arrangement haplotypes were used, or because samples from laboratory rather than natural populations were used. The most informative available estimates are shown in Supplementary File 4, with their standard errors where available.

For the two large-scale studies of egg-to-adult viability in *D. melanogaster* (Mukai and Yamaguchi 1974; Watanabe *et al*. 1976), where balancer crosses were used to extract 2nd or 3rd chromosomes from wild flies, the crosses involving pairs of independently extracted chromosomes were divided into cases where either each member of a pair was inversion-free or at least one member of each pair carried an inverted chromosome. There is no evidence for a difference between these categories in either study. One possible explanation for this discrepancy is the fact that a single component of fitness such as viability reflects only a portion of the net effect of deleterious mutations on fitness. Table S8 in Charlesworth (2015) presents estimates of the ratios of the fitness effects of deleterious mutation on various fitness components to their effects on net fitness (the *α* parameters). The estimates of *α* are subject to considerable uncertainty, but even the lowest estimate for viability (0.09) cannot explain the absence of effects on viability in these experiments.

Studies such as these that involve populations of *D. melanogaster* outside the ancestral range of the species in south-eastern Africa are difficult to interpret, because of the effects on variability of the severe population bottlenecks associated with the spread of the species out of Africa (e.g. Haddrill *et al*. 2005). Indeed, a curious feature of the data of Mukai and Yamaguchi (1974) on the 2nd chromosome is the much higher frequency of homozygous lethal *In* chromosomes than *St* chromosomes (54 vs 36%); this is associated with a higher frequency of pairs of crosses in which combinations of different lethal-bearing chromosomes were lethal (19 vs 11%), suggesting that there may have been a recent population size bottleneck that affected the *In* chromosomes more severely than the more abundant *St* chromosomes. Another possibility, proposed by a reviewer, is that high frequency lethals associated with the inversions were also associated with the *Sd* segregation distorter haplotype, as has been found in samples from US natural populations (reviewed by Larracuente and Presgraves 2012). Unfortunately, no information on the fitness effects of inversion genotypes appears to be available for samples from the ancestral range of *D. melanogaster*.

Species that are less subject to the problem of recent range expansion, such as *D*. *pseudoobscura*, are thus more favorable material. Estimates of the net relative fitnesses and of two measures of viability for *D. pseudoobscura* 3rd chromosome arrangements in population cages are also given in Supplementary File 4 (the horizontal lines separate data from different experiments). AR and CH cover approximately 30 and 20% of chromosome 3 (the homolog of chromosome arm 2R of *D. melanogaster*), respectively (Powell 1992), and are thus suitable for comparison with the theoretical prediction for *In(3R)P* given above. For the viability experiments of Dobzhansky (1947), involving CH and ST from a California population, CH had a frequency of between 0.20 and 0.35 in the original population, but which varied substantially over the year (Wright and Dobzhansky 1946, Fig. 2). The larval viability of CH/CH individuals was 86% of that of ST/ST individuals, which is reasonably consistent with expectations for an inversion with a frequency of around 0.3. The relative viabilities of CH/CH and AR/AR individuals were similar, as expected from the similar frequencies of CH and AR in this population.

The results for egg-to-adult viability measurements that used a balancer chromosome to extract 3rd chromosomes from a natural population of *D. pseudoobscura* (Crumpacker and Salceda 1968) show smaller effects than those predicted from mutational load; only the results for the 2 most common arrangements are shown in the table, with AR and CH having frequencies of approximately 0.5 and 0.28 in the population; the ratio of viabilities of CH/CH vs AR/AR in crosses between carriers of independently extracted chromosomes is 0.98, which is unlikely to differ significantly from 1.

The net fitness estimates obtained from population cage experiments on the natural population used for Dobzhansky's viability estimates, but which segregated for ST, AR, and CH (Wright and Dobzhansky 1946), show patterns that are inconsistent with the mutational load predictions; in particular, AR/AR has a much lower fitness than CH/CH despite their similar frequencies. In contrast, the ratios of net female fitnesses for CH/CH to AR/AR estimated by Anderson and Watanabe (1997) for a laboratory population derived from the same population but segregating only for CH and AR is 0.85, which is consistent with the fact that the population equilibrated at about 25% CH. However, the ratio for ST/ST vs AR/AR in another experimental population was 0.70, despite the fact that ST is usually at least as frequent as AR (Powell 1992).

The overall conclusion from these analyses of the relative performance of rare vs common arrangements is that some measurements fit the expectation of a larger equilibrium mutational load for the less frequent *Drosophila* inversions, but that the overall patterns imply that other factors obscure the contribution of load to homokaryotype fitnesses.

### Can mutational load create a selective advantage to heterokaryotypes?

The theoretical results described earlier make it clear that the mutational load model used here can only create a heterokaryotypic advantage when *In* and *St* are present at nearly equal frequencies (see Figs. 1 and 2, and Supplementary Tables 1 and 2). Furthermore, the size of such an advantage is likely to be small, even in subdivided populations, except for large inversions. For example, with *x* = 0.5, *h* = 0.25 and 10^5^ selected sites, the selection coefficient against both homokaryotypes vs the heterokaryotyope, as given by Equations (3), is 0.0016 with $\bar{\gamma }$ = 1,000, and 0.0010 with $\bar{\gamma }$ = 4,000. However, if the above model of 3 × 10^6^ sites with $\bar{\gamma }$ = 1,000 and 10^6^ sites with $\bar{\gamma }$ = 4,000 is used, the selection coefficient becomes 0.057. With *x* = 0.4, the selection coefficients under this model are *t*_1_ = 0.13 for *In*/*In* and *t*_2_ = 0.0013 for *St*/*St*. For lower values of *x*, the selective advantage to *In*/*St* heterokaryotypes over *St*/*St* is replaced by a selective disadvantage, as shown in Figs. 1 and 2.

The reason for this behavior is that a rarer arrangement accumulates a larger mutational load than its counterpart, due to the lower efficacy of selection with smaller *N_e_* for mildly deleterious, partially recessive mutations (see *Introduction*). When a haplotype from the *In* population is made heterozygous with a haplotype from the *St* subpopulation, there is a smaller expected number of heterozygous mutations in the *In*/*St* individuals than in the *In* subpopulation. This means that *t*_1_ > 0 if *h* < ½, as is evident from Equation (3a), where *F*_1_, 1 – *F*_1_, and −*C*_12_ are all positive, as is the difference in the expected frequency of mutations, 〈*δq*〉, between the *In* and *St* subpopulations. In contrast, *In*/*St* individuals have a larger expected number of mutations than the *St* subpopulation. As can be seen from Equation (3b), if *h* < ½ and the magnitude of 〈*δq*〉 is sufficiently large, *t*_2_ is negative. But if the 2 arrangements are equally frequent, 〈*δq*〉 = 0, and the remaining terms guarantee that *t*_1_ = *t*_2_ > 0 when *h* < ½. In all cases, *t*_1_ = *t*_2_ = 0 if *h* = ½. The only surprising aspect of the theoretical results is that *t*_2_ is so sensitive to the effect of the relative subpopulation sizes on 〈*δq*〉.

These theoretical predictions can be compared with the data in Supplementary File 4. The 2 studies of viability in *D. melanogaster* showed no difference in viability between chromosomal heterozygotes that were free of inversions and chromosomal heterozygotes where at least 1 of the pairs of chromosomes involved carried an inversion. Since the inversions were rare, most of the latter cases will have involved heterokaryotypes, so the lack of any difference is consistent with an absence of heterokaryotypic superiority, as expected for rare inversions. For *D. pseudoobscura*, the results on net fitness and on viability from population cage experiments indicate strong heterokaryotypic advantages, much larger than the theoretical predictions for inversions of the size involved here. In contrast, the balancer cross data on viability showed a small heterokaryotypic advantage (Crumpacker and Salceda 1968), consistent with the theoretical predictions for equally frequent arrangements. Mérot *et al*. (2020), Huang *et al*. (2022), and Pei *et al*. (2022) found no evidence for heterokaryotypic superiority for several fitness components in seaweed flies, sunflowers, and zebra finches, respectively. Overall, it seems likely that the mutational load model may contribute modestly to heterokaryotypic superiority for inversions that are at intermediate frequencies, but cannot explain the large net fitness effects seen in the *D. pseudoobscura* or *C. frigida* inversions. This parallels the finding that mutational load is unlikely to provide a selective advantage to new autosomal inversions in randomly mating populations (Nei *et al*. 1967; Connallon and Olito 2021; Jay *et al*. 2022).

### Population genomic indicators of a reduced efficacy of selection on low-frequency arrangements

As described in *A single population: population genomics statistics*, the two most informative population genomic statistics concerning a reduced efficacy of selection on a low-frequency arrangement are the mean frequencies of variants at the selected sites (the ${\bar{q}}_{i}$) and the ratios of diversities at selected and neutral sites (the $\pi_{\mathrm{sel}i}/\pi_{\mathrm{neut}i}$). The measure of skew toward low-frequency variants relative to neutral expectation (Δ*θ_wi_*) is much less sensitive to subpopulation size, unless the dominance coefficient is implausibly small. In addition, measures of skew are sensitive to population size changes (Tajima 1989), and must be treated with caution when making inferences about selection. For this reason, only the other two statistics will be considered here.

A problem with using the ${\bar{q}}_{i}\;$ is that these are not directly observable unless bioinformatic methods for classifying variants as deleterious are used; simply using the mean frequency of derived nonsynonymous variants in a sample as a proxy (cf., Campos *et al*. 2014) is not necessarily adequate when selection is weak, since it does not take into account fixed sites. Stenløk *et al*. (2022) used PROVEAN scores to estimate the mean numbers of deleterious missense mutations in inverted and standard arrangements of Atlantic salmon, but found no significant differences; an enrichment of small indels in the large (3.09 Mb) Chr18 inversion was, however, detected. The frequencies of this inversion are, however, highly variable between populations, so it is not clear how to interpret this difference.

There are, however, theoretical reasons for expecting the ratio ${\bar{q}}_{1}$/${\bar{q}}_{2}$ to behave similarly to the ratio *R_π_* = $\left(\pi_{\mathrm{sel}1}/\pi_{\mathrm{neut}1}\right)$/ $\left(\pi_{\mathrm{sel}2}/\pi_{\mathrm{neut}2}\right)$, at least when *h* is not too small. Welch *et al*. (2008) analyzed the properties of the ratio of selected site to neutral site diversity under a similar model to that used here, assuming *h* = 0.5 and a gamma distribution of selection coefficients (see also James *et al*. 2017). With $\bar{\gamma }$ >> 1/*a*, where *a* is the shape parameter of the gamma distribution in Equation (9), the following approximate relation holds:


(13a) $$\ln\;\left(\frac{\pi_{\mathrm{sel}}}{\pi_{\mathrm{neut}}}\right)=k-a\;\ln(\pi_{\mathrm{neut}})$$

where *k* is a constant that depends on the effective population size and the mean strength of selection.

In the present case, this relation implies that


(13b) $$R_{\ln(\pi )}=\ln\;\left(\frac{\pi_{\mathrm{sel}1}\;\pi_{\mathrm{neut}2}}{\;\pi_{\mathrm{sel}2}\pi_{\mathrm{neut}1\;}}\right)\approx a\;\ln\left(\frac{\pi_{\mathrm{neut}2}}{\pi_{\mathrm{neut}1}}\right)$$

While we would not expect this relation to be exact for *h* < 0.5, it is plausible to assume that it would be a reasonably good approximation when *h* is not too close to 0. The argument used by Welch *et al*. (2008) also implies that a similar relation should apply to the mean frequencies of deleterious mutations:


(13c) $$R_{\ln(q)}=\ln\;\left(\frac{{\bar{q}}_{1}}{{\bar{q}}_{2}}\right)\approx a\;\;\ln\left(\frac{\pi_{\mathrm{neut}2}}{\pi_{\mathrm{neut}1}}\right)$$

The accuracy of these approximations can be tested using the numerical results in Supplementary Tables 1 and 2. Table 3 gives some examples for the case of a single randomly mating population, showing that the two ratios on the left-hand sides of Equations (13b) and (13c) behave very similarly as functions of *h*, with $R_{\ln(q)}$ > $R_{\ln(\pi )}$ when *h* < 0.25, approaching the prediction on the right-hand sides of the equations for *h* ≥ 0.35. Similar results apply to the case of a subdivided population.

**Table 3.** Values of the ratios with respect to *In* vs *St* of the natural logarithms of *π_sel_/π_neut_* (*R*_ln*(π)*_) and $\bar{q}$ (*R*_ln*(q)*_) for the case of a single population.

	$\bar{\gamma }$ = 1,000				$\bar{\gamma }$ = 4,000			
	*x* = 0.1		*x* = 0.3		*x* = 0.1		*x* = 0.3	
*h*	*R*_ln(*π*)_	*R*_ln(*q*)_	*R*_ln(*π*)_	*R*_ln(*q*)_	*R*_ln(*π*)_	*R*_ln(*q*)_	*R*_ln(*π*)_	*R*_ln(*q*)_
0.05	0.840	1.106	0.395	0.485	0.884	1.110	0.403	0.487
0.15	0.720	0.907	0.313	0.393	0.744	0.909	0.319	0.394
0.25	0.679	0.794	0.282	0.329	0.694	0.795	0.286	0.330
0.35	0.661	0.718	0.267	0.285	0.675	0.719	0.270	0.285
0.45	0.653	0.658	0.257	0.250	0.665	0.659	0.261	0.250
0.50	0.650	0.632	0.254	0.235	0.662	0.633	0.257	0.235

The predicted values of the ratios with the shape parameter *a* = 0.3 are 0.659 for *x* = 0.1 and 0.254 for *x* = 0.3.

The mutational parameters and population size are the same as in Fig. 1.

These results suggest that *R_π_* can used as a conservative proxy for ${\bar{q}}_{1}$/${\bar{q}}_{2}$, at least for the case of a gamma distribution of selection coefficients with $\bar{\gamma }$ >> 1/*a* and an intermediate dominance coefficient. An objection to using the correlation between $R_{\ln(\pi )}$ and a measure of neutral diversity such as synonymous site diversity to investigate whether the efficacy of purifying selection declines with *N_e_* is that $\pi_{\mathrm{neut}}\;$is the denominator of $\pi_{\mathrm{sel}}/\pi_{\mathrm{neut}}$. In addition to the statistical problem of a negative correlation introduced by this relationship, discussed by James *et al*. (2017), sites under sufficiently strong purifying selection could maintain a constant diversity across different *N_e_* values (Campos *et al*. 2014). If this were the case, the expectation of $\pi_{\mathrm{sel}}/\pi_{\mathrm{neut}}$ would simply be proportional to $1/\pi_{\mathrm{neut}}$, and we would then have *R_π_* = $\pi_{\mathrm{neut}2}/\pi_{\mathrm{neut}1}$. As described in *A single population: population genomics statistics*, this is not the case with the model used here; *R_π_* with *x* < 0.5 is always less than *y*/*x*. For example, with $\bar{\gamma }$ = 4,000 and *h* = 0.25, *R_π_* = 2.00 for *x* = 0.1 (*y*/*x* = 9) and *R_π_* = 1.33 for *x* = 0.3 (*y*/*x* = 2.33). This indicates that *R_π_* provides a signal that purifying selection is weakened by smaller subpopulation size. Comparisons of this kind could easily be done using real data.

Overall, therefore, these considerations suggest that, despite the above reservations, *R_π_* is quite a useful index of the efficacy of purifying selection, and that one might expect $R_{\ln(\pi )}$ to be approximately equal to *a* ln($\pi_{\mathrm{neut}2}$/$\pi_{\mathrm{neut}1}$). A related principle was used by James *et al*. (2017) and Castellano *et al*. (2018) to test for relations between the efficacy of purifying selection and *N_e_* for animal mitochondrial genomes and different regions of the *D. melanogaster* nuclear genome, respectively. Unfortunately, there appears to be relatively little relevant information for autosomal inversions, other than cases such as the mimicry supergene in *Heliconius numata* (Jay *et al*. 2021) and the behavioral supergene of the white-throated sparrow *Zonotrichia albicolis* (Jeong *et al*. 2021), which are largely maintained as heterozygotes due to negative assortative mating. These systems are analogous to sex chromosomes, where one arrangement is permanently heterozygous and effectively lacks recombination. There is thus likely to be intense Hill–Robertson interference (Charlesworth and Charlesworth 2000), which would greatly reduce the efficacy of selection below the simple effect of a lower subpopulation size. This is consistent with the strongly elevated $\pi_{\mathrm{sel}}/\pi_{\mathrm{neut}}$ values found for the *H. numata* inversions; *Z. albicolis* showed, however, only a modest effect. Jay *et al*. (2021) also found a large increase in the density of transposable elements (TEs) in the *H. numata* inversions and interpreted this as evidence for an increased mutational load. The accumulation of TEs in low recombination regions of genomes, including low-frequency *Drosophila* inversions (Sniegowski and Charlesworth 1994), has long been documented (Charlesworth *et al*. 1994). Most insertions are found in intergenic regions, where direct selective effects are likely to be weak, and where ectopic exchange inducing deleterious chromosome rearrangements is probably a major factor in causing their elimination. It is thus likely that a reduced frequency of ectopic recombination is the major factor in causing higher densities of TE insertions in such cases (Charlesworth *et al*. 1994), so that this phenomenon cannot be taken as evidence for an increased mutational load.

### What strength of selection has the main effect on the load and population genomic statistics?

Another question raised by the theoretical results is: what part of the distribution of selection coefficients contribute to the differences between the *In* and *St* subpopulations? Stronger selection reduces the frequencies of deleterious mutations and their chances of fixation within a subpopulation, but also increases the sizes of any resulting loads. The major contribution to the relevant load statistics is thus likely to come from selection coefficients that are neither too large nor too small. This expectation can be tested by examining the contributions from the different zones described in *A single population: obtaining the mutational load and population genomic statistics*. These are shown in Supplementary Table 1 for the case of a single population. These results show that the major contributions to the *L_i_* and *t_i_* come from zone 2a, defined by an intermediate intensity of selection (for more details, see Supplementary Table 5). For example, for a randomly mating single population with $\bar{\gamma }$ = 4,000, *x* = 0.1, and *h* = 0.25, zones 1, 2a, 2b, and 3 contribute 4, 36, 24, and 36%, respectively, to the distribution of selection coefficients against deleterious mutations. Zone 2a alone gives *L*_1_ = 0.0375, *L*_2_ = 0.0012, *t*_1_ = 0.0270, and *t*_2_ = −0.0090, compared with net values of *L*_1_ = 0.0381, *L*_2_ = 0.0016, *t*_1_ = 0.0271, and *t*_2_ = −0.0090. It covers the interval (0.278, 463) of *γ* for the whole population.

The finer dissection of the distribution of selection coefficients used in the case of a subdivided population reveals that the so-called quasineutral zone 2 in this case (see Section 5 of Supplementary File 1) contributes most to the load statistics. For example, with $\bar{\gamma }$ = 4,000, *x* = 0.1, *h* = 0.25, and *F_ST_* = 0.05 (*M* = 19), zones 1, 2, 3, and 4 for the subdivided case contribute 4, 17, 21, and 58%, respectively. Zone 2 covers the *γ* interval (0.278, 55.6), and contributes *L*_1_ = 0.0344, *L*_2_ = 0.0011, *t*_1_ = 0.0249, and *t*_2_ = −0.0082, compared with values of *L*_1_ = 0.0401, *L*_2_ = 0.0019, *t*_1_ = 0.0285, and *t*_2_ = −0.0094 for the whole distribution. In this case, with 200 demes of size 5,000 each, both the *In* and *St* subpopulations behave as effectively neutral within demes for this part of the distribution of selection coefficients. A similar pattern holds even with *F_ST_* = 0.25. Nevertheless, the results are very similar to those for the single population, implying that the expectations of the population genetic parameters for an island model with a relatively low level of isolation between demes are mainly controlled by the size of the metapopulation, as was shown to be the case analytically by Wakeley (2003). Intuitively, this reflects the fact that, under these conditions, a mutation spends relatively little of its total sojourn time in the deme in which it arose.

### Confounding factors

The present study assumes that selected sites are at statistical equilibrium under mutation, selection and drift, an absence of recombinational exchange between *In* and *St* in heterokaryotypes, and complete independence among sites under selection. These assumptions are likely to violated in many real-life situations. First, consider the question of departure from equilibrium. If the inversion arises as a unique mutational event, as the available evidence seems to suggest (reviewed in Charlesworth 2023), the *In* subpopulation will initially completely lack genetic variability, and much time will be needed for equilibrium to be approached. The *St* subpopulation can be assumed to be close to equilibrium initially and will thus approach its new equilibrium much faster than the *In* subpopulation, so that only the latter need be considered here. For this subpopulation, the magnitudes of the *L_i_, B_i_*, and *t_i_* will be below their equilibrium values for a long time after the inversion has approached its equilibrium frequency under balancing selection.

It is difficult to make exact predictions about the rate of approach to statistical equilibrium when both drift and selection play a role, which has been shown above to be the situation that contributes the most to the selective differences among karyotypes. For the limiting case of complete neutrality, it is known that the divergence of the expected nucleotide site diversity from its equilibrium value at time *t* is equal to the product of its initial value and $\exp(-t/2N_{e})$ in a randomly mating population (Malécot 1969, p. 40), so that the timescale for approach to equilibrium is of the order of 2*N_e_* generations. For the other limiting case of fully deterministic evolution, with *γ* >> 1, the divergence at time *t* of the mutant allele frequency *q* from its equilibrium value of *u*/*hs* is approximately equal to the product of its initial value and exp(−t/hs). For the intermediate situation when *γ* is not much greater than 1, the two measures of the rate of approach to equilibrium are not very different, so it is plausible to assume that the true rate lies between them. The time needed to approach equilibrium with respect to mutations that have the largest effect on the load statistics is thus likely to be < 2*Nx* generations under a Wright–Fisher model. For a population with an *N_e_* of 10^6^ and *x* = 0.1, this would correspond to 10^5^ generations, i.e. about 10,000 years for a species like *D. melanogaster* with approximately 10 generations per year. For *x* = 0.5, about 50,000 years would be required.

A problem with assessing whether these timescales are consistent with data on inversion polymorphisms is that the occurrence of recombinational exchange between *In* and *St* due to gene conversion and/or double crossing over (reviewed by Krimbas and Powell 1992; Korunes and Noor 2019), means that estimates of inversion age based on sequence divergence between different arrangements tend to produce underestimates of the age of the derived arrangement (Charlesworth 2023). A variety of lines of evidence suggests, however, that inversions such as *In(3R)P* are close to selection-drift-recombination equilibrium with respect to neutral variability (Charlesworth 2023); since selection against deleterious mutations will cause a faster approach to equilibrium than with neutrality, it is likely that the load statistics will also be close to their equilibrium values, unless demographic disturbances have caused serious perturbations.

However, the theory developed here for predicting mutational loads has ignored recombination. If the estimate of a typical rate of gene conversion of 10^–5^ in female meiosis in inversion heterokaryotypes in *Drosophila* (Chovnick 1973; Korunes and Noor 2019) is accepted, the effective rate is 0.5 × 10^–5^ due to the absence of exchange in males. An *hs* value that somewhat exceeds 10^–5^ would thus be sufficient to overcome the effects of gene flow between *In* and *St*; with *h* = 0.25 and *N_T_* = 10^6^, this would correspond to *γ* > 80, which lies outside the range of *γ* values that contribute to a noticeable difference in load statistics between *In* and *St* with *x* = 0.1, as discussed in the previous section. It is therefore likely that recombination will significantly reduce such differences, providing another reason for regarding the above estimates as providing upper bounds to the predictions.

In addition, the fitness effects of any deleterious mutations that were present on the initial inversion haplotype have been ignored; however, the final equilibrium state considered here, where reverse mutations have been included, means that such effects will have been removed. During the approach to equilibrium they must, of course, play a role in reducing any selective advantage to a new inversion (Nei *et al*. 1967; Connallon and Olito 2021; Jay *et al*. 2022).

The low frequency of recombination in inversion heterokaryotypes for a rare inversion may create Hill–Robertson interference effects, as noted above in *Population genomic indicators of a reduced efficacy of selection on low-frequency arrangements*. Berdan *et al* (2021) used simulations to study this possibility, and found strong interference effects under the assumption that mutations were completely recessive. As pointed out previously, this assumption is implausible, even for mutations with large homozygous fitness effects. In addition, recombination between *In* and *St* will reduce interference effects. These will tend to increase the load in the less frequent subpopulation and in heterokaryotypes, and so will not contribute to the maintenance of the inversion polymorphism. There is little evidence for any effects of Hill–Robertson interference on the available population genomic statistics for the *D. melanogaster* inversions (Charlesworth 2023).

## Conclusions

The theoretical results described here show that a long-maintained autosomal inversion polymorphism with no recombination in heterokaryotypes may develop a substantially higher mutational load for the less frequent arrangement. The magnitude of the difference between arrangements can be large for rare polymorphic inversions of the size usually encountered in *Drosophila* populations, but declines quickly as the frequency of the rare arrangement increases. It is also strongly influenced by the abundance of relative weakly selected noncoding sequences, since drift acts more strongly on these than on strongly selected nonsynonymous mutations. A selective advantage to heterokaryotypes is only expected when the two alternative arrangements are nearly equal in frequency, and is likely to be small even in this case. Experiments on the effects of several *Drosophila* inversion polymorphisms on fitness components give inconsistent results, although mutational load may contribute to some of the effects that have been detected. It should also be possible to detect molecular signatures of an increased load, such as an enhanced ratio of nonsynonymous to synonymous nucleotide site diversities, but the data are currently too scanty to draw firm conclusions. The effects of recombinational exchange in heterokaryotypes and Hill–Robertson interference, which oppose each other, were ignored here, and deserve further study.

## Supplementary Material

iyad218_Supplementary_Data

## Data Availability

No new data or reagents were generated for this work. The codes for the computer programs used to generate the results described above are available in Supplementary Files 2 and 3. The numerical results used to produce the figures are presented in Supplementary Tables 1–4. Supplemental material is available at GENETICS online.

## Appendix

### Load calculations for a single population

Zone 1 corresponds to a quasineutral region where *γ* is sufficiently small (e.g. 0.25) that the selection term in Equations (5) can be ignored. The two *q_i_*s can be then treated as independent variables, each following a beta distribution with parameters *α_i_* and *β_i_*. In this case, we have

(A.1a)
$$\left\langle q_{i}\right\rangle =\frac{\alpha_{i}}{\alpha_{i}+\beta_{i}}=\frac{u}{u+v}$$

(A.1b)
$$F_{i}=\frac{1}{1+\alpha_{i}+\beta_{i}}$$

(A.1c)
$$\;\pi_{i}=2\left\langle p_{i}q_{i}\right\rangle =\frac{2\alpha_{i}\beta_{i}}{\alpha_{i}+\beta_{i}}(1-F_{i})$$

where Equation (A.1c) give the expected nucleotide site diversity for subpopulation *i*. In most applications, we have *α_i_*, *β_i_* << 1, so that this equation can be approximated by

(A.1d)
$$\pi_{i}\approx \frac{2\alpha_{i}\beta_{i}}{\alpha_{i}+\beta_{i}}=4N_{i}\tilde{u}$$

where $\tilde{u}=2\kappa v/(1+\kappa )$ is the net mutation rate at base composition equilibrium (Charlesworth and Charlesworth 2010, p. 274). This is identical in form to the infinite sites model formula for nucleotide site diversity, which does not explicitly take reverse mutations into account (Kimura 1971).

These expressions can be inserted into Equations (1). For this zone, the exponential term in *γ* in the gamma distribution p.d.f. can be neglected. As it is assumed that *x* ≤ 0.5, the focus is on ensuring that the *St* population behaves as neutral. As shown in Section 3 of Supplementary File 1, if the upper bound to *γy* (the scaled selection coefficient for *St* in zone 1) is denoted by *γ_c_*_1_, the probability *P_z_*_1_ that *γy* ≤ *γ_c_*_1_ and the integral of *γy* over this zone, *I*_1_ are given by

(A.2a)
$$P_{z1}\approx \Gamma {\left(a\right)}^{-1}(\bar{\gamma }y{)}^{-a}a^{a}\gamma_{c1}^{a}$$

(A.2b)
$$I_{1}\approx a(a+1{)}^{-1}\gamma_{c1}P_{z1}$$

Zone 2 involves a moderate intensity of selection, with *γ_c_*_1_ ≤ *γy* ≤ *γ_c_*_2_, where *γ_c_*_2_ is such that there is a nontrivial probability that either *q*_1_ or *q*_2_ reaches an intermediate or high frequency. In order to increase the accuracy of the integrations of the joint p.d.f. over the distribution of *γ*, zone 2 was divided into 2 subzones, with zone 2a having an upper limit of *γy* = *γ_c_*_2*a*_, and zone 2b with *γ_c_*_2*a*_ ≤ *γy* ≤ *γ_c_*_2_. Numerical values for *γ_c_*_1_, *γ_c_*_2*a*_, and *γ_c_*_2_ can be found in Supplementary Table 1. The probability of *s* falling into this zone, *P_z_*_2*a*_, is given by the excess over *P_z_*_1_ of the integral of the gamma distribution from 0 to *γ_c_*_2*a*_; the probability for zone 2b, *P_z_*_2*b*_ is found in a similar way by using *γ_c_*_2*a*_ and *γ*_c2_ as integration limits. The bivariate distribution of Equation (8) is used to determine the expected value of the load statistics in Equations (1) for a given *γ*, using the means and variances of the *q_i_* generated by the distribution. The integrals over *γ_c_*_1*a*_ ≤ *γy* ≤ *γ_c_*_2*a*_ and *γ_c_*_2*a*_ ≤ *γy* ≤ *γ_c_*_2_ of the products of Equations (1a)–(1c) with *n_s_* and the p.d.f. for the gamma distribution of *γy* yield the net contributions from zone 2a and 2b to the load statistics.

It was found that use of Equation (8) for values of *γy* that were close to *γ_c_*_1_ (usually set to 0.25) gave inaccurate results, with expected frequencies of A_2_ somewhat greater than those obtained for zone 1. To avoid this problem, an approximation to Equation (8), described in Section 4 of Supplementary File 1, was used for values of *γy* less than a threshold value. Trial and error showed that accurate results, where both methods agreed closely in the results for integration over the whole zone were obtained by setting this threshold to 0.25*γy*/(*hx*), equivalent to 40*γy* with *h* = 0.05 and *x* = 0.5 (this adjusts for the fact that selection against rare mutations is effectively weaker when they are more recessive).

Zone 3 involves a high intensity of selection, such that *q*_1_ and *q*_2_ are both confined to their boundaries close to 0; here *γ_c_*_2_ ≤ *γy* ≤ *γ_c_*_3_, where *γ_c_*_3_ is assigned as the upper limit to the distribution of *γy*, usually chosen to correspond to the upper 99th percentile of the gamma distribution. The probability of *s* falling into this zone, *P_z_*_3_, can be found as for *P*_*z*2*a*_ and *P_z_*_2*b*_. The two frequencies *q*_1_ and *q*_2_ can be treated as independent variables, since their expected product is negligible and A_2_ alleles within *In* and *St* haplotypes have little chance of encountering each other. Provided that *h* is sufficiently greater than 0, their p.d.f.s for a given *h* and *s* are well approximated by gamma distributions (Nei 1968), with shape parameters *α_i_* and means *u*/*hs*. The corresponding variances are (*u*/*hs*)^2^/*α_i_*, allowing the *F* and other load statistics of Equations (1) to be determined easily. The net contributions of zone 3 to the load statistics can then be found by integrations of the same type as described for zone 2.

The integrals over the distribution of *γ* for each of zones 2a, 2b, and 3 were evaluated numerically using Simpson's rule (Atkinson 1989), usually using 650 points over a single interval for zones 2a, 2b, and 3 (trial and error showed that this number was sufficient to produce a high degree of stability for the values of the statistics of interest). This procedure was also used for the integrals involved in obtaining the load statistics from the probability distribution of allele frequencies. No integration over *γ* values is needed for zone 1, since neutrality is assumed and the integral of *γ* over this interval is obtained using Equation (9).

### Probability distribution for a subdivided population

Writing *γ_m_* = 2*N_T_s* for the scaled selection coefficient with regard to the whole metapopulation and $G_{i}=F_{STi}/(1-F_{STi})\;$, we have

(A.3a)
$$\;\psi_{sm}({\bar{q}}_{1},{\bar{q}}_{2})=-2\gamma_{m}(a_{1}{\bar{q}}_{1}+a_{1}{\bar{q}}_{2}+b_{11}{\bar{q}}_{1}^{2}+b_{22}{\bar{q}}_{2}^{2}+b_{12}{\bar{q}}_{1}{\bar{q}}_{2})$$

where

(A.3b)
$$a_{1}=[G_{1}+(1-2G_{1})h]x^{2}+hxy$$

(A.3c)
$$a_{2}=[G_{2}+(1-2G_{2})h]y^{2}+hxy$$

(A.3d)
$$b_{11}=\frac{1}{2}(1\text{–}2G_{1})(1-2h)x^{2}$$

(A.3e)
$$b_{22}=\frac{1}{2}(1\text{–}2G_{2})(1-2h)y^{2}$$

(A.3f)
$$b_{12}=(1-2h)xy$$

When selection is sufficiently weak in relation to migration that 2*N_T_s* << *M*, where *M* is the scaled migration parameter 4*Nm*, we have $F_{ST1}\approx 1/(1+Mx)$ and $F_{ST2}\approx 1/(1+My)$. For sites independent of the inversion, $F_{STn}\approx 1/(1+M)$. Purifying selection is expected to reduce the *F_STi_* below their neutral values (Charlesworth and Charlesworth 2010, p. 353), so that use of the neutral approximations overestimates the effect of drift relative to selection. An approximate correction for the effect of selection on *F_STi_* is used here, based on Equation (B7.8.4) of Charlesworth and Charlesworth (2010, p. 354); the term 4*Nhs* is added to *M* in the formula for *F_ST_*. This procedure is only rigorous for *Nhs* >> 1; numerical studies show that it very slightly reduces the effect of population subdivision compared with using the purely neutral expression (Supplementary Table 6).

Combining Equations (A.3) with the mutational terms, the bivariate p.d.f. has a similar form to Equation (8), except that ${\bar{p}}_{i}$ and ${\bar{q}}_{i}$ are used instead of *p_i_* and *q_i_*, and the scaled mutational parameters involve the products of 4*N_T_*_1_*/*(1 – *F_ST_*_1_) and 4*N_T_*_2_*/*(1 – *F_ST_*_2_) with the relevant mutation rates. Thus, *α_i_* and *β_i_* are replaced by *α_im_* = 4*N_Ti_ u/*(1 – *F_STi_*) and *β_im_* = 4*N_Ti_ v/*(1 – *F_STi_*), respectively.

In order to use Equations (1) to calculate the load statistics, it is necessary to consider the p.d.f. of allele frequencies within demes. Following Cherry and Wakeley (2003) and Wakeley (2003), by conditioning on the current values of the ${\bar{q}}_{i}$ for a deme with frequencies *q_i_* we obtain the general expression:

(A.4)
$$\phi (q_{1},q_{2}|{\bar{q}}_{1},{\bar{q}}_{1})=\tilde{C}\;\exp(-\psi_{s})\;q_{1}^{\alpha_{1d}-1}{p_{1}}^{\beta_{1d}-1}q_{2}^{\alpha_{2d}-1}\;{p_{2}}^{\beta_{2d}-1}$$

where $\psi_{s}$ has the same form as in Equation (7), $\alpha_{1d}=Mx{\bar{q}}_{1}+4Nxu$, $\beta_{1d}=Mx{\bar{p}}_{1}+4Nxv$, $\alpha_{2d}=My{\bar{q}}_{1}+4Nyu$, $\beta_{2d}=Mx{\bar{p}}_{2}+4Nxv$ (the subscript *d* is used to indicate within-deme values).

An exact calculation of the load statistics for a given *s* would require multiplication of this function by the bivariate p.d.f. for the ${\bar{q}}_{i}$, followed by integrations over the *q_i_* and ${\bar{q}}_{i}$ to obtain the requisite quantities to insert into Equations (1); this would then be followed by integration over the distribution of *s*. In order to avoid this cumbersome procedure, partitioning of the distribution of *s* into different zones was used together with some approximations, similarly to what was done for the case of a single panmictic population. The details of the procedure are described in Section 5 of Supplementary File 1.
