# Supplementary material for: The fitness consequences of genetic divergence between polymorphic gene arrangements
Source: Genetics. 2023 Dec 26;226(3):iyad218. doi: 10.1093/genetics/iyad218 (PMC11090464; doi:10.1093/genetics/iyad218)
Supplement: iyad218_Supplementary_Data [file iyad218_supplementary_data.zip › Supplementary_File_1_GENETICS-2023-306559.docx]

**Supplementary File S1 Integrations used for determining the load statistics**

**1. Integration of the bivariate distribution of *q*_1_ and *q*_2_.**

For convenience, *q*_1_ and *q*_2_ are replaced by *X* and *Y*. Omitting the constant *C*, and writing *A_i_* and *B_i_* instead of *α_i_* and *β_i_*_,_ in order to accommodate the expressions needed for the case of a subdivided population, the bivariate distribution function of Equation (8) can be written as:

$f\left( X, Y \right)=exp\{-2\gamma\left( a_{1}X+a_{2}Y+b_{11}X^{2}+b_{12}XY+b_{22}Y^{2}) \right\} X^{A_{1}-1}{(1-X)}^{B_{1}-1}Y^{A_{2}-1}{(1-Y)}^{B_{2}-1}$

(S1)

The integral of this function over (0 ≤ *X* ≤ 1, 0 ≤*Y*≤ 1) is equivalent to 1/*C*. In order to obtain the expectations and variances of *q*_1_ and *q*_2_ and their covariance, the integrals of *f X^i^, fY^i^* (*i* = 1, 2) and *f XY* are also needed. To avoid singularities at the boundaries when carrying out this integration, the following approximations are used, where the *ε_i_* are chosen to ensure that their products with *γ* are negligible.

1. 0 ≤ *X*≤ *ε*_1_ ≈ 0, 0 ≤ *Y* ≤ *ε*_2_

$f\approx X^{A_{1}-1}Y^{A_{2}-1}, \int_{0}^{\varepsilon_{1}} \int_{0}^{\varepsilon_{2}} X^{i}Y^{j}f dY dX\approx A_{1}^{-\left( i+1 \right)}A_{2}^{-\left( j+1 \right)}\varepsilon_{1}^{A_{1}+i}\varepsilon_{2}^{A_{2}+j}$ (S2a)

(*i*, *j* = 0, 1, 2)

2. 0 ≤ *X*≤ *ε*_1_, 1 – *ε*_2_ ≤ *Y* ≤ 1

$$f\approx exp\{{-2\gamma\left( a_{2}+b_{22}) \right\}X}^{A_{1}-1}(1-{Y)}^{B_{2}-1},$$

$\int_{0}^{\varepsilon_{1}} \int_{1-\varepsilon_{2}}^{1} X^{i}Y^{j}f dYdX\approx A_{1}^{-\left( i+1 \right)}B_{2}^{-\left( j+1 \right)}\varepsilon_{1}^{A_{1}+i}\varepsilon_{2}^{B_{2}+j}exp\{-2\gamma\left( a_{2}+b_{22} \right)\}$(S2b)

3. 1– *ε*_1_ ≤ *X* ≤ 1 0 ≤ *Y* ≤ *ε*_2_

$f\approx exp\{{-2\gamma\left( b_{1}+b_{11}) \right\}(1-X)}^{B_{1}-1}Y^{A_{2}-1},$

$\int_{1-\varepsilon_{1}}^{1} \int_{0}^{\varepsilon_{2}} X^{i}Y^{j}\phi dY dX\approx B_{1}^{-\left( i+1 \right)}A_{2}^{-\left( j+1 \right)}\varepsilon_{1}^{B_{1}+i}\varepsilon_{2}^{A_{2}+j} exp\{-2\gamma\left( a_{1}+b_{11} \right)\}$(S2c)

4. 1– *ε*_1_ ≤ *X* ≤ 1 , 1 – *ε*_2_ ≤ *Y* ≤ 1

$f\approx{\exp\{-2\gamma({a_{1}+a}_{2}+b_{11}+b_{12}+b_{22})\}\left( 1-X \right)}^{B_{1}-1}(1-{Y)}^{B_{2}-1}$

$\int_{1-\varepsilon_{1}}^{1} \int_{1-\varepsilon_{2}}^{1} X^{i}Y^{j}f dY dX\approx B_{1}^{-\left( i+1 \right)}B_{2}^{-\left( j+1 \right)}\varepsilon_{1}^{B_{1}+i}\varepsilon_{2}^{B_{2}+j}\{-2\gamma({a_{1}+a}_{2}+b_{11}+b_{12}+b_{22})\}$(S2d)

5. 0 ≤ *X* ≤ *ε*_1_, *ε*_2_ ≤ *Y ≤* 1– *ε*_2_

$f\approx X^{A_{1}-1}exp\{-2\gamma(a_{2}Y+b_{22}Y^{2})\}Y^{A_{2}-1}(1-{Y)}^{B_{2}-1}$ ,

$\int_{0}^{\varepsilon_{1}} \int_{\varepsilon_{2}}^{1-\varepsilon_{2}} X^{i}Y^{j}f dY dX\approx{A_{1}}^{-(i+1)}{\varepsilon_{1}}^{A_{1}+i} \int_{\varepsilon_{2}}^{1-\varepsilon_{2}} exp\{-2\gamma(a_{2}Y+b_{22}Y^{2})\}Y^{A_{2}+j-1}(1-{Y)}^{B_{2}+j-1}dY$

(S2e)

The integral on the right of this expression must be evaluated numerically, as is also the case for the integrals in Equations (S2f) - (S2h) below.

6. 1– *ε*_1_ ≤ *X* ≤ 1, *ε*_2_ ≤ *Y ≤* 1– *ε*_2_

$f\approx{(1-X)}^{B_{1}-1}exp\{-2\gamma\left[ a_{1}+b_{11}+{(a}_{2}+b_{12} \right)Y+$ $b_{22}$ $Y^{2}]\}Y^{A_{2}-1}(1{-Y)}^{B_{2}-1}$

$\int_{1-\varepsilon_{1}}^{1} \int_{\varepsilon_{2}}^{1-\varepsilon_{2}} X^{i}Y^{j}f dY dX\approx$ ${B_{1}}^{-(i+1)}{\varepsilon_{1}}^{B_{1}+i} \int_{\varepsilon_{2}}^{1-\varepsilon_{2}} exp\{-2\gamma(\left[ a_{1}+b_{11}+{(a}_{2}+b_{12} \right)Y+b_{22}Y^{2})\}Y^{A_{2}+j-1}(1-{Y)}^{B_{2}+j-1}dY$ (S2f)

7. *ε*_1_ ≤ *X* ≤ 1− *ε*_1_, 0 ≤ *Y ≤* *ε*_2_

$f\approx Y^{A_{2}-1}exp\{-2\gamma(a_{1}X+b_{11}X^{2})\}X^{A_{1}-1}(1-{X)}^{B_{1}-1}$ ,

$$\int_{\varepsilon_{1}}^{1-\varepsilon_{1}} \int_{0}^{\varepsilon_{2}} f dY dX\approx{A_{2}}^{-(j+1)}{\varepsilon_{2}}^{A_{2}+j} \int_{\varepsilon_{1}}^{1-\varepsilon_{1}} exp\{-2\gamma(a_{1}X+b_{11}X^{2})\}X^{A_{1}-1}(1-{X)}^{B_{1}-1}dX$$

(S2g)

8. *ε*_1_ ≤ *X* ≤ 1− *ε*_1_, 1– *ε*_2_ ≤ *Y* ≤ 1

$f\approx\left( 1-Y \right)^{B_{2}-1}exp\{-2\gamma\left[ \left( a_{1}+b_{12} \right)X+{b_{11}X^{2}+(a}_{2}+b_{22} \right)]\}X^{A_{1}-1}(1{-X)}^{B_{1}-1}$

$\int_{\varepsilon_{1}}^{1-\varepsilon_{1}} \int_{{1-\varepsilon}_{2}}^{1} f dX dY\approx$

$${B_{2}}^{-(j+1)} {\varepsilon_{2}}^{B_{2}+j}exp[-2\gamma{(a}_{2}+b_{22})]\int_{\varepsilon_{1}}^{1-\varepsilon_{1}} exp\{-2\gamma[{(a}_{1}+b_{12})X+b_{11}X^{2}]\} X^{A_{1}-1}(1{-X)}^{B_{1}-1}dX$$

(S2h)

9. *ε*_1_ ≤ *X*≤ 1− *ε*_1_, *ε*_2_ ≤ *Y* ≤ 1– *ε*_2_

In this case, the double integration of *f* (*x*, *y*)*X^i^Y^j^* over the indicated ranges of *X* and *Y* must be carried out numerically.

**2. Frequencies of fixed and segregating sites**

If a sample of *n* haploid genomes is taken, the expected fractions of sites fixed for A_1_ and A_2_ in subpopulation *i* are given by the following general expressions:

$P_{f1in}=\int_{0}^{1} \left( 1-q_{i} \right)^{n}\phi(q_{i})dq_{i}$ (S3a)

$P_{f2in}=\int_{0}^{1} {q_{i}}^{n}\phi(q_{i})dq_{i}$ (S3b)

where *φ*(*q_i_*) is the marginal p.d.f. for the frequency of A_2_ in subpopulation *i*.

The fraction of sites segregating for A_1_ and A_2_ in subpopulation *i* is given by:

$S_{in}=1-P_{f1in}-P_{f2in}$ (S3c)

For the neutral case, the properties of the beta distribution of the *q_i_* under mutation and pure drift can be exploited to obtain explicit expressions, such that the integrands of Equations (S3a) and (S3b) can be written as $q_{i}^{\alpha_{i}-1}{(1-q_{i})}^{n+\beta_{i}-1}$ and $q_{i}^{{n+\alpha}_{i}-1}{(1-q_{i})}^{\beta_{i}-1}$, respectively. The integrals of these quantities are beta functions, and can be represented in terms of Γ functions, Γ(*α_i_*) Γ (*β_i_* + *n*)/ Γ (*α_i_* + *β_i_* + *n*) and Γ (*α_i_* + *n*)Γ (*β_i_*)/Γ (*α_i_* + *β_i_* + *n*), respectively.

With selection, the full bivariate distribution must be used to obtain the values of the *P_f_* functions. For regions with *q_i_* close to 0, it can be assumed that (1 – *q_i_*)*^n^* ≈ 1 – *nq_i_*; the integration over these regions for *P_f_*_1_*_ni_* can then be performed similarly to those in section S1 for the constant of integration and the expectation of *q_i_* in section S1. For regions *q_i_* with close to 1, (1 – *q_i_*)*^n^* ≈ 0 when *nq_i_* << 1 so that these can be ignored if the boundary value of *q_i_* is chosen to meet this condition. For *P_f_*_1_*_ni_*, the contribution from the lower boundary terms can be neglected, and the upper boundary terms evaluated from the integrals involving 1 – *np_i_*.

**3. Integrating over the quasi-neutral zone 1**

Assuming a gamma distribution of the form given in Equation (9), the exponential term in the p.d.f. can be set equal to 1 when *γ* /*b* is sufficiently small. Writing $\bar{\gamma}=ab$, the probability that *γ* is less than or equal to some upper bound *γ_c_* is given by:

$P\left( \gamma\leq\gamma_{c} \right)\approx{\Gamma\left( a \right)}^{-1}\bar{\gamma}^{-a}a^{a+1}a^{-1}\gamma^{-a}={\Gamma\left( a \right)}^{-1}\bar{\gamma}^{-a}a^{a}\gamma_{c}^{a}$ (S4a)

The integral of the product of *γ* with the approximate p.d.f. over this interval is similarly given by:

$I_{1}={\Gamma\left( a \right)}^{-1}\bar{\gamma}^{-a}a^{a-1}{{(a+1)}^{-1}\gamma}_{c}^{a+1}={a{(a+1)}^{-1}\gamma}_{c}P\left( \gamma\leq\gamma_{c} \right)$ (S4b)

**4. An approximation to the bivariate distribution of *q*_1_ and *q*_2_**

If selection is weak relative to drift, the frequency of A_2_ in subpopulation 2 experienced by subpopulation 1 can be treated as an average over the distribution of *q*_2_, so that *q*_2_ in Equation (5a) can be replaced with its expectation and the resulting expression substituted into Equation (6), similarly the treatment of temporal changes in fitness in the standard forward diffusion equation (Ewens 2004, p.192). Similarly, the frequency of A_2_ in subpopulation 1 experienced by subpopulation 2 can be replaced by its expectation. Ignoring the covariance term, as is legitimate with weak selection, the joint p.d.f. can be broken down into two components, one each for *q*_1_ and *q*_2_, such that:

$\psi_{s}(q_{1})\approx-2\gamma\{\left[ h+\left( 1-2h \right)y\left\langle q_{2} \right\rangle\right]{xq}_{1}+\frac{1}{2}\left( 1-2h \right)xq_{1}^{2}\}$ (S5a)

$\psi_{s}(q_{2})\approx-2\gamma\{\left[ h+\left( 1-2h \right)x\left\langle q_{2} \right\rangle\right]{yq}_{2}+\frac{1}{2}\left( 1-2h \right)yq_{2}^{2}\}$ (S5b)

Each of these can be evaluated numerically in a similar way to the joint p.d.f. described above, provided that the expected values of *q*_1_ and *q*_2_ are known. Since these are unknown *a* *priori*, the simple procedure of iterating the integral of the distributions of *q*_1_ given < *q*_2_> and *q*_2_ given < *q*_1_>, assuming a given *γ,* was adopted, until their means and variances converged (5 iterations proved to be sufficient for this purpose). The values of the load statistics for this *γ* value were then obtained using the final values of the means and variances in Equations (1). The numerical results from this procedure agreed closely with those using the full joint distribution, except for the case of very small *γ* values, where the latter gave inaccurate results.

**5. Load calculations for a subdivided population**

The load calculations for a subdivided population are divided into four different zones according to the scaled strength of selection. Zone 1 is such that the allele frequencies within both *In* and *St* over the metapopulation are effectively neutral. This case requires 0 ≤ 2*N_T_ys ≤ γ_c_*_1_, where *γ_c_*_1_ is the assigned threshold scaled selection coefficient for the metapopulation that defines effective neutrality, generally set to 0.25. The general formulae for the load statistics given by Equations (1) of the main text imply that, for a given *s* and *h,* they can be found from the expectations over the probability distributions of $\bar{q}_{1}$ and $\bar{q}_{2}$, the mean frequencies of *A*_2_ within *In* and *St* over the metapopulation and the *F_STi_*, the *F*-statistics for the variances of the allele frequencies among demes within karyotypes. Under neutrality, the expected value of $\bar{q}_{i}$ over the distribution of mean allele frequencies is $\left\langle\bar{q}_{i} \right\rangle\approx u/(u+v)$ for both *In* and *St*, and allele frequencies within demes follow a beta distribution with this expectation (Wakeley 2003).

The *F_STi_* can be calculated from the neutral formula for a diallelic locus:

$F_{STi}\approx\frac{1}{1+M_{i}} \left( i=1 or 2 \right)$ (S.6)

where *M*_1_ = 4*Nmx,* *M*_2_ = 4*Nmy.*

The net *F*-statistics for use in Equations (1) are then given by the standard result for hierarchical *F*-statistics (Wright 1951):

$F_{i}=F_{STi}+(1-F_{STi})F_{im}$ (S.7a)

where *F_im_* is the ratio of the variance in the $\bar{q}_{i}$ to the product of the expectations of $\bar{q}_{i}$and 1 – $\bar{q}_{i}$. In the case of neutrality, we have:

$F_{im}=\frac{1}{1+\alpha_{im}+\beta_{im}}$ (S.7b)

The expected load statistics contributed by zone 1 are then obtained as described for the case of a single population. By Maruyama’s invariance principle for neutral sites (Maruyama 1971; Charlesworth and Charlesworth 2010, p.318), the expected neutral diversity within a deme and karyotype is given by Equations (11) of the main text, with *N_T_*_1_ and *N_T_*_1_ replacing *N*_1_ and *N*_2_, provided that *α_im_* = 4*N_Ti_ u/*(1 – *F_STi_*) and *β_im_* = 4*N_Ti_ v/*(1 – *F_STi_*) are both << 1. In other cases, the more general expression $\pi_{i}=2\left\langle{p_{i}q}_{i} \right\rangle=2\bar{p}_{i}\bar{q}_{i}(1-F_{i} )$ is to be used. As explained in the main text, the neutral values for the *F_STi_* are also used in the calculations involving selection.

For Zone 2, the allele frequencies for both *In* and *St* within demes are effectively neutral, whereas the mean allele frequencies for the metapopulation are affected by moderate selection. The selection coefficients for zone 2 are now such that *γ_c_*_1_ *≤* 2*N_T_sy* ≤ *γ_c_*_2_, where *γ_c_*_2_ = *dγ_c_*_1_. The bivariate p.d.f. for $\bar{q}_{1}$ and $\bar{q}_{2}$, described by Equations (A.3) and (A.4), together with the relevant mutational terms, is used to determine their means and variances (see section 1 above). For values of 2*N_T_sy* close to the lower boundary, the approximation described for zone 2a of the single population case was used. Since the distributions of allele frequencies within demes are close to neutrality, the covariance between $\bar{q}_{1}$ and $\bar{q}_{2}$ can be ignored. The variance of $\bar{q}_{i}$normalised by $\bar{p}_{i}\bar{q}_{i}$ yields *F_im_*, the *F*-statistic for the metapopulation distribution. This can then be combined with the neutral value of *F_STi_* from the beta distribution, and the expected load statistics obtained as described for zone 1.

Zone 3 involves moderate selection, where coupling between *In* and *St* in the metapopulation is ignored, but is allowed for the within-deme allele frequencies. In this case, the upper limit to *γy* is *γ_c_*_3_ = *γ_c_*_2_ /*x*. $\bar{q}_{1}$ obeys Equations (A.3) and (A.4) with $a_{1}=[G_{1}+\left( 1-2G_{1} \right)h]x^{2}$, $b_{11}=\frac{1}{2}\left( 1-2G_{1} \right)\left( 1-2h \right)x^{2}$and the other terms are set to zero, so that the distributions of $\bar{q}_{1}$and $\bar{q}_{2}$ are treated as independent; this is justified by the fact the covariance *C*_12_ is always very small compared to the variances. Similarly, $\bar{q}_{2}$ obeys Equations (A.3) and (A.4) with $a_{2}=[G_{2}+\left( 1-2G_{2} \right)h]y^{2}$, $b_{22}=\frac{1}{2}\left( 1-2G_{2} \right)\left( 1-2h \right){xy}^{2}$.

In order to calculate the unconditional expected load statistics, the following approximation avoids integrations over the entire range of the $\bar{q}_{i}$. The distribution of $\bar{q}_{i}$ is divided into three regions, two of which are boundary regions with $\bar{q}_{i}$close to 0 or 1, respectively, and an intermediate region, denoted by labels 1, 3 and 2, respectively. We then use the expected values of $\bar{q}_{i}$ for each region to determine the corresponding values of the mutation-migration parameters $\alpha_{id}$ and $\beta_{id}$associated with Equations (A.3). These parameters are then used to obtain the values of the means, variances and covariance of the *q_i_* across demes, integrating over the bivariate p.d.f. of Equations (A.3) and (A.4) as described in section 1 above. The results are then substituted into Equation (1) of the main text to yield the load statistics for a given *s*. The means of the statistics over the three regions are obtained by weighting by the probabilities of the $\bar{q}_{i}$ falling into the respective regions, and integration of over zone 3 by the same method as before.

The probabilities of the three regions and the corresponding expectations of the $\bar{q}_{i}$can be found as follows, noting that the assumption of independence between the two distributions means that the probability that $\bar{q}_{1}$ falls in region *i* and $\bar{q}_{2}$ into region *j* is simply the product of the probabilities for each $\bar{q}_{i}$taken separately. In order to avoid singularities for values of $\bar{q}_{i}$close to 0 or 1, it is convenient to approximate the p.d.f. for $\bar{q}_{i}$as follows:

Boundary region 1 ($\bar{q}_{i}\approx0$): $\phi\left( \bar{q}_{i} \right)\approx C_{i}{\bar{q}_{i}}^{\alpha_{im}-1}$ (S.8a)

Boundary region 3 ($\bar{q}_{i}\approx1$): $\phi\left( \bar{p}_{i} \right)\approx C_{i}{\bar{p}_{i}}^{\beta_{im}-1}$ (S.8b)

where *C_i_* is the normalization constant for the p.d.f. of $\bar{q}_{i}$. The *C_i_* are obtained using a procedure like that described for the bivariate distribution in.

The probability that $\bar{q}_{i}$falls below an assigned critical value $\bar{q}_{ci}$, and hence into boundary region 1, is given by:

$P_{c1i}\approx C_{i}\int_{0}^{\bar{q}_{ci}} {\bar{q}_{i}}^{\alpha_{i}-1}d\bar{q}_{i}=C_{i}\alpha_{im}^{-1}{\bar{q}_{ci}}^{\alpha_{im}}$ (S.9a)

The expectation of $\bar{q}_{i}$in this region is given by:

$\bar{q}_{1i}\approx C_{i}\int_{0}^{\bar{q}_{ci}} {\bar{q}_{i}}^{\alpha_{im}}d\bar{q}_{i}/P_{c1i}=\alpha_{im}{(1+\alpha_{im})}^{-1} \bar{q}_{ci}$ (S.9b)

Similarly, the probability that $\bar{p}_{i}$falls below an assigned critical value $\bar{p}_{ci}$, and hence into boundary region 3, is given by:

$P_{c3i}\approx C_{i}\int_{0}^{\bar{p}_{ci}} {\bar{p}_{i}}^{\beta_{im}-1}d\bar{p}_{i}=C_{i}\beta_{i}^{-1}{\bar{p}_{ci}}^{\beta_{im}}$ (S.9c)

The expectation of $\bar{p}_{i}$in this region is given by:

$\bar{p}_{3i}\approx\beta_{im}{(1+\beta_{im})}^{-1}\bar{p}_{ci}$ (S.9d)

The probability that $\bar{q}_{i}$ falls into the intermediate region 2 is *P*_2_*_i_* = 1 – *P_c_*_1_*_i_* – *P_c_*_3_*_i_*. The overall expectation of $\bar{q}_{i},$ $\bar{q}_{1t}$, is obtained by integrating the modified version of Equations (A.3), similar to the procedure described for the bivariate distribution in section 1. The expectation for the intermediate region 2 is then given by:

$\bar{q}_{2i}\approx{(\bar{q}}_{it}-\bar{q}_{i1}P_{c1i}-\bar{q}_{i3}P_{c3i} )/P_{2i}$ (A.9e)

In practice, the critical values of all four mean allele frequencies are set to 0.001 or 0.01/*γ* , whichever is smaller, providing a conservative cut-off for neutrality.

For zone 4, both the *In* and *St* mean allele frequencies over the metapopulation are subject to strong selection. The lower bound to this region is *γ*_c3_ = *dγ*_c2_ /*x*. The upper bound *γ*_c4_ is chosen to correspond to the 99^th^ percentile of the distribution of *γ*. Both the *In* and *St* metapopulation distributions are assumed to be gamma distributions with means equal to *u*/*hs* and shape parameters *α*_1_*_m_* and *α_2m_*, respectively. Otherwise, the treatments of the distributions for the metapopulation, and the within-deme distributions are the same as for zone 3. The standard procedure for evaluating the total contribution to the load statistics is used

**Literature Cited**

Charlesworth B, Charlesworth D. 2010. Elements of Evolutionary Genetics. Greenwood Village, CO: Roberts and Company.

Ewens WJ. 2004. Mathematical Population Genetics. 1. Theoretical Introduction. New York: Springer.

Maruyama T. 1971. An invariant property of a subdivided population. Genet Res. 18:81-84.

Wakeley J. 2003. Polymorphism and divergence for island-model species. Genetics. 163:411-420.

Wright S. 1951. The genetical structure of populations. Ann Eugen. 15:323-354.
