# Supplementary material for: The fitness consequences of genetic divergence between polymorphic gene arrangements
Source: Genetics. 2023 Dec 26;226(3):iyad218. doi: 10.1093/genetics/iyad218 (PMC11090464; doi:10.1093/genetics/iyad218)
Supplement: iyad218_Supplementary_Data [file iyad218_supplementary_data.zip › Supplementary_File_2_GENETICS-2023-306559.docx]

**Supplementary File S2 Computer code for the single population case**

**This program is written in GFortran for the MacIntosh (https://gnuc.org.wiki/GFortranBinariesMacOS)**

Program invload2

PROGRAM FOR LOAD WITH INVERSION AND STANDARD ARRANGEMENTS

! Single random mating population

! Uses gamma distribution of selection coefficients

! Coupling between selection on In and St into account

! No intermediate results are printed out

integer :: nsimp,nint,i,j,k,CONT,nsamp

real:metpop,ak,h,u,v,x,y,AM,ANET,ANE1,ANE2,alpha1,beta1,alpha2,beta2,h1,h2,ga1,gamm

real:: aj,gamc1,beta,a,z,Pz1,Pz2,Pz3,anint,gammq,gambar,gambar1,gambar2,ashape,r0,r1,r2,F1,F2,y2

real::alw1,alw2,alb,alhom1,alhom2,B1,B2,qbarneut,pbarneut,alw1s,alw2s,albs,B1w,B2w,B1ws,B2ws,ainteg1

real :: albn,alhomn1,alhomn2,gamser,xsc,gln,alhon1,alhnon2,x1a,x2a,upgam,upfac,cp12,aintfix(2),fp(1000)

real :: alw1a(1000),alw2a(1000),albna(1000),alhomn1a(1000),alhomn2a(1000),B1wa(1000),B2wa(1000)

real :: ainteg(2,2),ainteg0,alpha1i,beta1i,alhom1s,alhom2s,qb1(1000),qb2(1000),pi1b(1000),pi2b(1000)

real :: a1,a2,b11,b12,b22,asum,q1bar,q2bar,q1var,q2var,sbar,qt1,qt2,pi1t,pi2t,Pseg1b(1000),Pseg2b(1000),asi

CHARACTER*20 FIN

CHARACTER*20 FOUT

write (*,*) 'Input file?'

read(*,*) FIN

OPEN (2,file=FIN)

WRITE (*,*) 'Output file?'

READ (*,*) FOUT

OPEN (1,FILE=FOUT)

write (1,*) 'Genetic loads with inversion and standard arrangements in a panmictic population'

write (1,*) 'Includes mean allele frequencies, fixation frequencies and diversities'

write (1,*) 'A single randomly mating population with no exchange between In and St'

write (1,*) 'Coupling between selection on In and St is allowed for'

write (1,*) ''

write (1,*) 'A range of h values is covered'

write (1,*) ''

READ (2,*) ninvs

READ (2,*) nsimp

READ (2,*) ANET

READ (2,*) u

READ (2,*) ak

READ (2,*) x

READ (2,*) sbar,ashape

READ (2,*) gac1

READ (2,*) gamc1

READ (2,*) gamc21

READ (2,*) gamc22

READ (2,*) upgfac

READ (2,*) nsamp

READ (2,*) ndat

write (1,*) 'Mutation rate towards deleterious variants= ',u

v=u/ak

! reverse mutation rate

aninvs=ninvs

awf=asi(nsamp)

! sum of harmonic series (Watterson's correction factor)

write (1,*) 'Number of selected sites in inversion= ',ninvs

write (1,*) 'Mutational bias towards deleterious variants= ',ak

write (1,*) ''

write (1,*) 'Inversion frequency= ',x

write (1,*) 'Population size= ',ANET

write (1,*) 'No. of values for Simpsons rule = ',nsimp

write (1,*) 'Sample size= ',nsamp

write (1,*) 'Wattersons correction factor= ',awf

write (1,*) 'Number of dominance coefficients modelled= ',ndat

write (1,*) ''

write (*,*) 'Number of selected sites in inversion= ',ninvs

write (*,*) 'Mutation rate towards deleterious variants= ',u

write (*,*) 'Inversion frequency= ',x

write (*,*) ''

write (*,*) 'Mutational bias towards deleterious variants= ',ak

write (*,*) 'Inversion frequency= ',x

write (*,*) 'Population size= ',ANET

write (*,*) 'No. of values for Simpsons rule = ',nsimp

write (*,*) 'Sample size= ',nsamp

write (*,*) 'Wattersons correction factor= ',awf

write (*,*) 'Number of dominance coefficients modelled= ',ndat

write (*,*) ''

alpha=4*ANET*u

beta=alpha/ak

! scaled mutation rates

y=1-x

gambar=2*ANET*sbar

upfac=(1-x)*gambar/ashape

anint=nsimp

nint=nsimp+1

write (1,*) ''

write (1,*) 'Mean selection coefficient= ',sbar

write (1,*) 'Mean scaled selection coefficient for whole popn= ',gambar

write (1,*) 'Shape parameter= ',ashape

write (1,*) 'Upper bound gamma value for neutrality in the St popn= ',gamc1

write (1,*) 'Upper bound whole popn gamma value for zone 2a with h=0.25 = ',gamc21

write (1,*) 'Upper bound whole popn gamma value for zone 2b with h=0.25 = ',gamc22

write (1,*) 'Upper limit to gamma distribution of z= x/scale parameter = ',upgfac

write (1,*) 'Threshold gamma value factor for use of approximate p.d.f.= ',gac1

write (1,*) ''

write (1,*) ''

write (*,*) 'Mean selection coefficient= ',sbar

write (*,*) 'Mean scaled selection coefficient for whole popn= ',gambar

write (*,*) 'Shape parameter= ',ashape

write (*,*) 'Upper bound whole popn gamma value for zone 2a with h=0.25 = ',gamc21

write (*,*) 'Upper bound whole popn gamma value for zone 2b with h=0.25 = ',gamc22

write (*,*) 'Upper limit to gamma distribution of z= x/scale parameter = ',upgfac

write (*,*) 'Threshold gamma value factor for use of approximate p.d.f.= ',gac1

write (*,*) ''

write(*,*) 'Continue: type 1 if not desired'

read (*,*) CONT

! allows program to be cancelled if desired

if(CONT.eq.1) go to 400

do 410 idat=1,ndat

READ (2,*) h

write (1,*) ''

write (1,*) ''

write (1,*) 'h = ',h

write (1,*) ''

write (*,*) ''

write (*,*) 'h = ',h

write (*,*) ''

h1=h

gamc21=gamc21*0.25/h1

gamc22=gamc22*0.25/h1

! gamc2's corrected for dominance coefficient

ANE1=x*ANET

ANE2=y*ANET

! popn sizes for In and St

alpha1=alpha*x

alpha2=alpha*y

beta1=beta*x

beta2=beta*y

gambar1=gambar*x

gambar2=gambar*y

! numbered parameters are values for In (1) and St (2), respectively

alw1s=0

alw2s=0

albs=0

alhom1s=0

alhom2s=0

B1ws=0

B2ws=0

qt1=0

qt2=0

pi1t=0

pi2t=0

Ptsegw1=0

Ptsegw2=0

! initial values of mean load statistics

!!!!!!!!!!!!!!!!!!!!!!!!!!!!!!!!!!!!!!!!!!!!!!!!!!!!!!!!!!!!!!!!!!!!!!!

! Zone 1: neutral approximation (both In and St popns behave as quasi-neutral)

! No need for selective coupling to be modelled

sc1=gamc1/(2*ANE2)

! corresponding upper bound selection coefficient for calculating the load statistics

z=exp(gammln(ashape))

! calculates gamma function with parameter ashape

xsc=ashape*gamc1/gambar2

call gser(gamser,ashape,xsc,gln)

Pz1=gamser

! probability of falling within the neutral zone for St metapopulation

Sselneut1=ashape*sc1*aninvs*Pz1/(ashape+1.0)

! integral of selection coefficients for St over zone 1

write(*,*) ''

write (1,*) 'Zone 1: quasi-neutral zone'

write (1,*) 'Upper bound scaled selection coefficient for neutrality in St metapopulation= ',gamc1

write (1,*) 'Probability of zone 1= ',Pz1

write (1,*) 'Integral of selection coefficient over zone 1= ',Sselneut1

write (*,*) 'Upper bound scaled selection coefficient for neutrality in St metapopulation= ',gamc1

write (*,*) 'Probability of zone 1= ',Pz1

write (*,*) 'Integral of selection coefficient for St over neutral zone= ',Sselneut1

write (*,*) ''

qbarneut=ak/(1+ak)

pbarneut=1-qbarneut

! mean allele frequencies under neutrality

F1=1.0/(1+alpha1+beta1)

F2=1.0/(1+alpha2+beta2)

! F for In and St assuming neutrality for both arrangements

pi1=2*pbarneut*qbarneut*(1-F1)

pi2=2*pbarneut*qbarneut*(1-F2)

! diversities for In and standard

qt1=qbarneut*Pz1

qt2=qbarneut*Pz1

pi1t=pi1*Pz1

pi2t=pi2*Pz1

! contributions to mean allele frequencies and diversities for In and St

Pfix11=betamom(beta1,alpha1,nsamp)

Pfix12=betamom(beta2,alpha2,nsamp)

! Frequencies of fixation of A1 in In and St in sample of size nsamp

write(*,*) 'Pfix11= ',Pfix11,' Pfix12= ',Pfix12

! Frequencies of fixation of A2 in In and St in sample of size nsamp

Pfix21=betamom(alpha1,beta1,nsamp)

Pfix22=betamom(alpha2,beta2,nsamp)

write(*,*) 'Pfix21= ',Pfix21,' Pfix22= ',Pfix22

! Frequencies of fixation of A2 in In and St in sample of size nsamp

Pseg1=1-Pfix11-Pfix21

Pseg2=1-Pfix12-Pfix22

! Frequencies of segregating sites in In and St in sample of size nsamp

Pfix1=1-Pseg1

Pfix2=1-Pseg2

q1f=Pfix21/(Pfix11+Pfix21)

q2f=Pfix22/(Pfix12+Pfix22)

! frequencies of A2 among fixed sites

write(*,*) 'q1f= ',q1f,' q2f= ',q2f

pi1t=pi1*Pz1

pi2t=pi2*Pz1

Ptsegw1=Pseg1*Pz1

Ptsegw2=Pseg2*Pz1

! compiles integrals of load statistics

theta1=Pseg1/awf

theta2=Pseg2/awf

! Watterson's theta values

delth1=1-(pi1/theta1)

delth2=1-(pi2/theta2)

! delta-theta for In and St

call loadstats1(qbarneut,qbarneut,h1,F1,F2,alw1,alw2,alb,alhom1,alhom2,B1,B2)

! this subroutine ignores covariance between q1 and q2

alw1=alw1*Sselneut1

alw2=alw2*Sselneut1

! total loads within In and St for neutral zone

alb=alb*Sselneut1

! total between arrangement load for neutral zone

alhom1=alhom1*Sselneut1

alhom2=alhom2*Sselneut1

! homozygous loads for neutral zone for In and St

Bneut1=B1*Sselneut1

Bneut2=B2*Sselneut1

! inbreeding loads for neutral zone for In and St

alw1s=alw1

alw2s=alw2

albs=alb

alhom1s=alhom1

alhom2s=alhom2

B1ws=Bneut1

B2ws=Bneut2

! compiles integrals of load statistics

tt1=1.0-exp(alb-alw1)

tt2=1.0-exp(alb-alw2)

write (1,*) ''

write (1,*) 'Mean load statistics for zone 1'

write (1,*) 'Mean q1 and q2= ',qbarneut

write (1,*) 'F1 and F2= ',F1,F2

write (1,*) 'Diversities= ',pi1,pi2

write (1,*) ''

write (1,*) 'Contributions to loads within In and St=',alw1,alw2

write (1,*) 'Contribution to load between In and St =',alb

write (1,*) 'Contributions to homozygous loads for In and St=',alhom1,alhom2

write (1,*) 'Contributions to inbreeding loads for In and St= ',B1w,B2w

write (1,*) ''

write (1,*) 'Contributions to selection coefficients for In and St homokaryotypes'

write (1,*) tt1,tt2

write (1,*) ''

write (1,*) 'Contributions to mean A2 freqs=',qt1,qt2

write (1,*) 'Contributions to mean diversities=',pi1t,pi2t

write (1,*) 'Contributions to mean freqs. of seg. site=',Ptsegw1,Ptsegw2

write (1,*) 'Delta-theta values=',delth1,delth2

write (1,*) ''

write (*,*) ''

write (*,*) 'Mean load statistics for zone 1'

write (*,*) 'Mean q1 and q2= ',qbarneut

write (*,*) 'F1 and F2= ',F1,F2

write (*,*) 'Diversities= ',pi1,pi2

write (*,*) ''

write (*,*) 'Contributions to loads within In and St=',alw1,alw2

write (*,*) 'Contributions to load between In and St=',alb

write (*,*) 'Contributions to loads homozygous loads for In and St=',alhom1,alhom2

write (*,*) 'Contributions to inbreeding loads= ',Bneut1,Bneut2

write (*,*) 'Contributions to selection coefficients against In and St homokaryotypes'

write (*,*) tt1,tt2

write (*,*) ''

write (*,*) 'Contributions to mean A2 freqs=',qt1,qt2

write (*,*) 'Contributions to mean diversities=',pi1t,pi2t

write (*,*) 'Contributions to mean freqs. of seg. site=',Ptsegw1,Ptsegw2

write (*,*) 'Delta-theta values=',delth1,delth2

write (*,*) ''

pi1neut=pi1

pi2neut=pi2

! saves these for final comparison of selected and neutral site diversities

! write (*,*) 'Continue: type 1 if not desired'

! read (*,*) CONT

! allows program to be cancelled if desired

! if(CONT.eq.1) go to 400

!!!!!!!!!!!!!!!!!!!!!!!!!!!!!!!!!!!!!!!!!!!!!!!!!!!!!!!!!!!!!!!!!!!!!!!

! 2. Zone 2a: moderate selection (gamma for St ≤ gamc21)

! Selective coupling between In and St is allowed for;gamma for St is limited to moderate value

! Integration over distribution of gamma values in this zone

write(1,*) ''

write (1,*) 'Zone 2a: moderate selection; cut-off at moderate gamma for St population'

write (1,*) 'Lower and upper bounds of St popn gamma'

write (1,*) gamc1,gamc21

write (1,*) ''

write(*,*) ''

write (*,*) 'Zone 2a: moderate selection; cut-off at moderate gamma for St population'

write (*,*) 'Lower and upper bounds of St popn gamma'

write (*,*) gamc1,gamc21

write(*,*) ''

xsc=ashape*gamc21/gambar2

call gser(gamser,ashape,xsc,gln)

Pz2=gamser-Pz1

Ptot=gamser

write (1,*) 'Probability of zone 2a= ',Pz2

write(1,*) ''

write (*,*) 'Probability of zone 2a= ',Pz2

write(*,*) ''

x1a=gamc1*ashape/gambar2

x2a=gamc21*ashape/gambar2

! lower and upper limits of scaled gamma distribution variable for integrations

del=(gamc21-gamc1)/anint

a1=h1*x

a2=h1*y

b11=0.5*(1-2*h1)*(x**2)

b12=(1-2*h1)*x*y

b22=0.5*(1-2*h1)*(y**2)

! coefficients for bivariate distribution of q1 and q2

write(1,*) 'Coefficients for bivariate distribution of q1 and q2'

write(1,*) 'a1=',a1,' a2=',a2

write(1,*) 'b11=',b11,' b12=',b12,' b22=',b22

write(1,*) ''

write(*,*) 'Coefficients for bivariate distribution of q1 and q2'

write(*,*) 'a1=',a1,' a2=',a2

write(*,*) 'b11=',b11,' b12=',b12,' b22=',b22

write(*,*) ''

bgam=gambar2/ashape

! shape parameter of gamma distribution

do 580 i1=1,nint

ga1=gamc1+(i1-1)*del

! write(*,*) 'Index of gamma value',i1,' gamma for St popn= ',ga1

! write(*,*) ''

xi=ga1/bgam

! scaled variable for gamma distribution of selection coefficients for St popn

Pxi=(xi**(ashape-1))*exp(-xi)

Pxi=Pxi/z

! probability density of xi

! write(*,*) 'Pxi=',Pxi

gamint=ga1/y

gamm=gamint*x

sc2=ga1/(2*ANE2)

sc=aninvs*sc2

! write(*,*) 'sc=',sc

! product of selection coefficient and number of loci for load calculations

gac=gac1/(x*h1)

! equivalent to gac=40*gac1 with h=0.05 and x=0.5

if(ga1.le.gac) then

! uses approximate univariate distributions for weak selection

b111=0.5*(1-2*h1)*x

b221=0.5*(1-2*h1)*y

! coefficients for 2nd order terms in q1 and q2 in distribution function,

do 110 it=1,5

a22=h1+(1-2*h1)*q1bar*x

! approximation for coefficient of 1st order term in q2 in distribution function

call integ3(ga1,a22,b221,alpha2,beta2,nsimp,ainteg10i,ainteg11i,ainteg12i,aint10,aint11,aint12,aint2)

const2=ainteg10i+aint10+aint2

! integration constant for q2

q2bar=(ainteg11i+aint11+aint2)/const2

! write(*,*) 'Mean of q2= ',q2bar

q2var=(ainteg12i+aint12+aint2)/const2

q2var=q2var-q2bar**2

! write(*,*) 'Variance of q2= ',q2var

a11=h1+(1.0-2*h1)*q2bar*y

! approximation for coefficient of 1st order term in q1 in distribution function

call integ3(gamm,a11,b111,alpha1,beta1,nsimp,ainteg10i,ainteg11i,ainteg12i,aint10,aint11,aint12,aint2)

const1=ainteg10i+aint10+aint2

! integration constant for q1

q1bar=(ainteg11i+aint11+aint2)/const1

! write(*,*) 'Mean of q1= ',q1bar

q1var=(ainteg12i+aint12+aint2)/const1

q1var=q1var-q1bar**2

! write(*,*) 'Variance of q1= ',q1var

110 continue

! Iterates means and variances of q1 and q2

F1=q1var/(q1bar*(1-q1bar))

F2=q2var/(q2bar*(1-q2bar))

! write(*,*) ''

! write(*,*) 'Mean q1= ',q1bar

! write(*,*) 'Variance= ',q1var

! write(*,*) 'Mean q2= ',q2bar

! write(*,*) 'Variance= ',q2var

! write(*,*) ''

! write(*,*) 'F1 and F2= ',F1,F2

pi1=2*q1bar*(1.0-q1bar)*(1-F1)

pi2=2*q2bar*(1.0-q2bar)*(1-F2)

! write(*,*) 'pi1 and pi2= ',pi1,pi2

! diversities

call integfix2(gamint,a1,a2,b11,b12,b22,alpha1,alpha2,beta1,beta2,nsimp,aintfix,ainteg0,nsamp)

! integrals for fixation frequencies of A2

Pfix21=aintfix(1)/ainteg0

Pfix22=aintfix(2)/ainteg0

if(Pfix21.le.10.0e-6) then

Pfix21=0

end if

if(Pfix22.le.10.0e-6) then

Pfix22=0

end if

call integfix1(gamint,a1,a2,b11,b12,b22,alpha1,alpha2,beta1,beta2,nsimp,aintfix,nsamp)

! integrals for fixation frequencies of A1

Pfix11=aintfix(1)/ainteg0

Pfix12=aintfix(2)/ainteg0

! write(*,*) ''

! write(*,*) 'Fixation integrals=',aintfix(1),aintfix(2)

! write(*,*) 'Freqs. of fixation of A1 for In and St=',Pfix11,Pfix12

! write(*,*) ''

! write(*,*) ''

! write(*,*) 'integration constant=',ainteg0

! write(*,*) 'Fixation integrals=',aintfix(1),aintfix(2)

! write(*,*) 'Freqs. of fixation of A2 for In and St=',Pfix21,Pfix22

! write(*,*) ''

Pseg1=1.0-Pfix11-Pfix21

Pseg2=1.0-Pfix12-Pfix22

! write(*,*) 'Freqs. of segregation for In and St=',Pseg1,Pseg2

Pfix1=1-Pseg1

Pfix2=1-Pseg2

q1f=Pfix21/(Pfix11+Pfix21)

q2f=Pfix22/(Pfix12+Pfix22)

! frequencies of A2 among fixed sites

! write(*,*) 'q1f= ',q1f,' q2f= ',q2f

! write(*,*)

115 alw1=0

alw2=0

alb=0

alhon1=0

alhon2=0

B1w=0

B2w=0

call loadstats1(q1bar,q2bar,h1,F1,F2,alw1,alw2,alb,alhomn1,alhomn2,B1,B2)

! this subroutine ignores covariance between q1 and q2

alw1=alw1*sc

alw2=alw2*sc

alb=alb*sc

alhomn1=alhomn1*sc

alhomn2=alhomn2*sc

B1w=B1*sc

B2w=B2*sc

! write(*,*)

! write(*,*) 'Load statistics'

! write(*,*) 'alw1,alw2',alw1,alw2

! write(*,*) 'alb,alhomn1,alhomn2',alb,alhomn1,alhomn2

! write(*,*) 'B1w,B2w',B1w,B2w

fp(i1)=Pxi

alw1a(i1)=alw1*Pxi

alw2a(i1)=alw2*Pxi

! write(*,*) 'alw1a',alw1a(i1)

! write(*,*) 'alw2a',alw2a(i1)

albna(i1)=alb*Pxi

alhomn1a(i1)=alhomn1*Pxi

alhomn2a(i1)=alhomn2*Pxi

! write(*,*) 'albna',albna(i1)

! write(*,*) 'alhomn1a',alhomn1a(i1)

! write(*,*) 'alhomn2a',alhomn2a(i1)

B1wa(i1)=B1w*Pxi

B2wa(i1)=B2w*Pxi

! write(*,*) 'B1wa',B1wa(i1)

! write(*,*) 'B2wa',B2wa(i1)

qb1(i1)=q1bar*Pxi

! write(*,*) 'i= ',i1,'Pxi=',Pxi,' qb1=',qb1(i1)

qb2(i1)=q2bar*Pxi

pi1b(i1)=pi1*Pxi

pi2b(i1)=pi2*Pxi

Pseg1b(i1)=Pseg1*Pxi

Pseg2b(i1)=Pseg2*Pxi

! Stores p.d.f.-weighted load statistics for current value of gamma

go to 580

end if

100 call integ1(gamint,a1,a2,b11,b12,b22,alpha1,alpha2,beta1,beta2,nsimp,ainteg,ainteg0,cp12)

! write(*,*) 'Integration constant for p.d.f= ',ainteg0

q1bar=ainteg(1,1)/ainteg0

q2bar=ainteg(2,1)/ainteg0

! write(*,*) 'Means of q1 and q2= ',q1bar,q2bar

q1var=ainteg(1,2)/ainteg0

q1var=q1var-q1bar**2

q2var=ainteg(2,2)/ainteg0

q2var=q2var-q2bar**2

! write(*,*) 'Variances of q1 and q2= ',q1var,q2var

cp12=cp12/ainteg0

cov12=cp12-q1bar*q2bar

r12=cov12/sqrt(q1var*q2var)

! write(*,*) 'Covariance and correlation of q1 and q2= ',cov12,r12

F1=q1var/(q1bar*(1-q1bar))

F2=q2var/(q2bar*(1-q2bar))

! write(*,*) 'F1 and F2= ',F1,F2

pi1=2*q1bar*(1.0-q1bar)*(1-F1)

pi2=2*q2bar*(1.0-q2bar)*(1-F2)

! diversities for given gamma value

call integfix2(gamint,a1,a2,b11,b12,b22,alpha1,alpha2,beta1,beta2,nsimp,aintfix,ainteg0,nsamp)

! integrals for fixation frequencies of A2

Pfix21=aintfix(1)/ainteg0

Pfix22=aintfix(2)/ainteg0

if(Pfix21.le.10.0e-6) then

Pfix21=0

end if

if(Pfix22.le.10.0e-6) then

Pfix22=0

end if

call integfix1(gamint,a1,a2,b11,b12,b22,alpha1,alpha2,beta1,beta2,nsimp,aintfix,nsamp)

! integrals for fixation frequencies of A1

Pfix11=aintfix(1)/ainteg0

Pfix12=aintfix(2)/ainteg0

! write(*,*) ''

! write(*,*) 'Fixation integrals=',aintfix(1),aintfix(2)

! write(*,*) 'Freqs. of fixation of A1 for In and St=',Pfix11,Pfix12

! write(*,*) ''

! write(*,*) ''

! write(*,*) 'integration constant=',ainteg0

! write(*,*) 'Fixation integrals=',aintfix(1),aintfix(2)

! write(*,*) 'Freqs. of fixation of A2 for In and St=',Pfix21,Pfix22

! write(*,*) ''

Pseg1=1.0-Pfix11-Pfix21

Pseg2=1.0-Pfix12-Pfix22

! write(*,*) 'Freqs. of segregation for In and St=',Pseg1,Pseg2

Pfix1=1-Pseg1

Pfix2=1-Pseg2

q1f=Pfix21/(Pfix11+Pfix21)

q2f=Pfix22/(Pfix12+Pfix22)

! frequencies of A2 among fixed sites

! write(*,*) 'q1f= ',q1f,' q2f= ',q2f

! write(*,*)

30 alw1=0

alw2=0

alb=0

alhon1=0

alhon2=0

B1w=0

B2w=0

call loadstats2(q1bar,q2bar,h1,F1,F2,alw1,alw2,albn,alhomn1,alhomn2,B1,B2,cp12)

alw1=alw1*sc

alw2=alw2*sc

alb=albn*sc

alhomn1=alhomn1*sc

alhomn2=alhomn2*sc

B1w=B1*sc

B2w=B2*sc

! write(*,*)

! write(*,*) 'Load statistics'

! write(*,*) 'alw1,alw2',alw1,alw2

! write(*,*) 'alb,alhomn1,alhomn2',alb,alhomn1,alhomn2

! write(*,*) 'B1w,B2w',B1w,B2w

fp(i1)=Pxi

alw1a(i1)=alw1*Pxi

alw2a(i1)=alw2*Pxi

! write(*,*) 'alw1a',alw1a(i1)

! write(*,*) 'alw2a',alw2a(i1)

albna(i1)=alb*Pxi

alhomn1a(i1)=alhomn1*Pxi

alhomn2a(i1)=alhomn2*Pxi

! write(*,*) 'albna',albna(i1)

! write(*,*) 'alhomn1a',alhomn1a(i1)

! write(*,*) 'alhomn2a',alhomn2a(i1)

B1wa(i1)=B1w*Pxi

B2wa(i1)=B2w*Pxi

! write(*,*) 'B1wa',B1wa(i1)

! write(*,*) 'B2wa',B2wa(i1)

qb1(i1)=q1bar*Pxi

qb2(i1)=q2bar*Pxi

pi1b(i1)=pi1*Pxi

pi2b(i1)=pi2*Pxi

Pseg1b(i1)=Pseg1*Pxi

Pseg2b(i1)=Pseg2*Pxi

! Stores p.d.f. weighted load statistics for current value of gamma

580 continue

fpint=asum(nsimp,fp,x1a,x2a)

alw1i=asum(nsimp,alw1a,x1a,x2a)

alw2i=asum(nsimp,alw2a,x1a,x2a)

albi=asum(nsimp,albna,x1a,x2a)

alhon1i=asum(nsimp,alhomn1a,x1a,x2a)

alhon2i=asum(nsimp,alhomn2a,x1a,x2a)

B1wi=asum(nsimp,B1wa,x1a,x2a)

B2wi=asum(nsimp,B2wa,x1a,x2a)

qbw1=asum(nsimp,qb1,x1a,x2a)

qbw2=asum(nsimp,qb2,x1a,x2a)

pibw1=asum(nsimp,pi1b,x1a,x2a)

pibw2=asum(nsimp,pi2b,x1a,x2a)

Psegw1=asum(nsimp,Pseg1b,x1a,x2a)

Psegw2=asum(nsimp,Pseg2b,x1a,x2a)

alw1s=alw1s+alw1i

alw2s=alw2s+alw2i

albs=albs+albi

alhom1s=alhom1s+alhon1i

alhom2s=alhom2s+alhon2i

B1ws=B1ws+B1wi

B2ws=B2ws+B2wi

qt1=qt1+qbw1

qt2=qt2+qbw2

pi1t=pi1t+pibw1

pi2t=pi2t+pibw2

Ptsegw1=Ptsegw1+Psegw1

Ptsegw2=Ptsegw2+Psegw2

! compiles integrals of load statistics

tt1=1.0-exp(albi-alw1i)

tt2=1.0-exp(albi-alw2i)

theta1=Psegw1/awf

theta2=Psegw2/awf

! Watterson's theta values

delth1=1-(pibw1/theta1)

delth2=1-(pibw2/theta2)

! delta-theta

write(1,*) ''

write (1,*) 'Net probability of zone 2a using Simpsons rule= ',fpint

write (1,*) ''

write (1,*) 'Contributions to mean load statistics over zone 2a'

write (1,*) 'Contributions to mean loads within In and St=',alw1i,alw2i

write (1,*) 'Contribution to load between In and St=',albi

write (1,*) 'Contributions to homozygous loads for In and St=',alhon1i,alhon2i

write (1,*) 'Contributions to inbreeding loads= ',B1wi,B2wi

write (1,*) 'Selection coefficients for In and St homokaryotypes'

write (1,*) tt1,tt2

write (1,*) ''

write (1,*) 'Selection coefficients for In and St homokaryotypes'

write (1,*) tt1,tt2

write (1,*) ''

write (1,*) 'Contributions to mean A2 freqs=',qbw1,qbw2

write (1,*) 'Contributions to mean diversities=',pibw1,pibw2

write (1,*) 'Contributions to mean proportions of seg. sites=',Psegw1,Psegw2

write (1,*) 'Contributions to delta-theta values=',delth1,delth2

write (1,*) ''

write (*,*) ''

write (*,*) 'Net probability of zone 2a using Simpsons rule= ',fpint

write (*,*) ''

write (*,*) 'Contributions to mean load statistics over zone 2a'

write (*,*) 'Contributions to mean loads within In and St=',alw1i,alw2i

write (*,*) 'Contribution to load between In and St=',albi

write (*,*) 'Contributions to homozygous loads for In and St=',alhon1i,alhon2i

write (*,*) 'Contributions to inbreeding loads= ',B1wi,B2wi

write (*,*) 'Selection coefficients for In and St homokaryotypes'

write (*,*) tt1,tt2

write (*,*) ''

write (*,*) 'Contributions to mean A2 freqs=',qbw1,qbw2

write (*,*) 'Contributions to mean diversities=',pibw1,pibw2

write (*,*) 'Contributions to mean freq. of seg. sites=',Psegw1,Psegw2

write (*,*) 'Contributions to delta-theta values=',delth1,delth2

write (*,*) ''

! write (*,*) 'Continue: type 1 if not desired'

! read (*,*) CONT

! allows program to be cancelled if desired

! if(CONT.eq.1) go to 400

!!!!!!!!!!!!!!!!!!!!!!!!!!!!!!!!!!!!!!!!!!!!!!!!!!!!!!!!!!!!!!!!!!!!!!!

! Zone 2b: moderate selection in In subpopulation (gamma for St ≤ gamc22)

! Selective coupling between In and St is allowed for; gamma for St reaches high value

! Integration over distribution of gamma values in this zone

write(1,*) ''

write (1,*) 'Zone 2b: moderate selection; gamma for St reaches high value'

write (1,*) 'Lower and upper bounds of St popn gamma'

write (1,*) gamc21,gamc22

write (1,*) ''

write(*,*) ''

write (*,*) 'Zone 2b: moderate selection; gamma for St reaches high value'

write (*,*) 'Lower and upper bounds of St popn gamma'

write (*,*) gamc21,gamc22

write(*,*) ''

xsc=ashape*gamc22/gambar2

call gser(gamser,ashape,xsc,gln)

Pz3=gamser-Ptot

Ptot=gamser

write (1,*) 'Probability of zone 2b ',Pz3

write(1,*) ''

write (*,*) 'Probability of zone 2b= ',Pz3

write(*,*) ''

x1a=gamc21*ashape/gambar2

x2a=gamc22*ashape/gambar2

! lower and upper limits of scaled gamma distribution variable for integrations

del=(gamc22-gamc21)/anint

nint=nsimp+1

do 590 i1=1,nint

ga1=gamc21+(i1-1)*del

! write(*,*) 'Index of gamma value',i1,' gamma for St popn= ',ga1

! write(*,*) ''

! write(1,*) 'Index of gamma value',i1,' gamma for St popn= ',ga1

! write(1,*) ''

xi=ashape*ga1/gambar2

! scaled variable for gamma distribution of selection coefficients for St popn

Pxi=(xi**(ashape-1))*exp(-xi)

Pxi=Pxi/z

! probability density of xi

! write(*,*) 'Pxi=',Pxi

gamint=ga1/(1-x)

! gamma for whole population

sc2=ga1/(2*ANE2)

sc=aninvs*sc2

! write(*,*) 'sc=',sc

! product of selection coefficient and number of loci for load calculations

call integ1(gamint,a1,a2,b11,b12,b22,alpha1,alpha2,beta1,beta2,nsimp,ainteg,ainteg0,cp12)

! write(*,*) 'Integration constant for p.d.f= ',ainteg0

q1bar=ainteg(1,1)/ainteg0

q2bar=ainteg(2,1)/ainteg0

! write(*,*) 'Means of q1 and q2= ',q1bar,q2bar

q1var=ainteg(1,2)/ainteg0

q1var=q1var-q1bar**2

q2var=ainteg(2,2)/ainteg0

q2var=q2var-q2bar**2

! write(*,*) 'Variances of q1 and q2= ',q1var,q2var

cp12=cp12/ainteg0

cov12=cp12-q1bar*q2bar

r12=cov12/sqrt(q1var*q2var)

! write(*,*) 'Covariance and correlation of q1 and q2= ',cov12,r12

F1=q1var/(q1bar*(1-q1bar))

F2=q2var/(q2bar*(1-q2bar))

! write(*,*) 'F1 and F2= ',F1,F2

pi1=2*q1bar*(1.0-q1bar)*(1-F1)

pi2=2*q2bar*(1.0-q2bar)*(1-F2)

! diversities for given gamma value

call integfix2(gamint,a1,a2,b11,b12,b22,alpha1,alpha2,beta1,beta2,nsimp,aintfix,ainteg0,nsamp)

! integrals for fixation frequencies of A2

Pfix21=aintfix(1)/ainteg0

Pfix22=aintfix(2)/ainteg0

if(Pfix21.le.10.0e-6) then

Pfix21=0

end if

if(Pfix22.le.10.0e-6) then

Pfix22=0

end if

call integfix1(gamint,a1,a2,b11,b12,b22,alpha1,alpha2,beta1,beta2,nsimp,aintfix,nsamp)

! integrals for fixation frequencies of A1

Pfix11=aintfix(1)/ainteg0

Pfix12=aintfix(2)/ainteg0

! write(*,*) ''

! write(*,*) 'Fixation integrals=',aintfix(1),aintfix(2)

! write(*,*) 'Freqs. of fixation of A1 for In and St=',Pfix11,Pfix12

! write(*,*) ''

! write(*,*) ''

! write(*,*) 'integration constant=',ainteg0

! write(*,*) 'Fixation integrals=',aintfix(1),aintfix(2)

! write(*,*) 'Freqs. of fixation of A2 for In and St=',Pfix21,Pfix22

! write(*,*) ''

Pseg1=1.0-Pfix11-Pfix21

Pseg2=1.0-Pfix12-Pfix22

! write(*,*) 'Freqs. of segregation for In and St=',Pseg1,Pseg2

Pfix1=1-Pseg1

Pfix2=1-Pseg2

q1f=Pfix21/(Pfix11+Pfix21)

q2f=Pfix22/(Pfix12+Pfix22)

! frequencies of A2 among fixed sites

! write(*,*) 'q1f= ',q1f,' q2f= ',q2f

! write(*,*)

alw1=0

alw2=0

alb=0

alhon1=0

alhon2=0

B1w=0

B2w=0

call loadstats2(q1bar,q2bar,h1,F1,F2,alw1,alw2,albn,alhomn1,alhomn2,B1,B2,cp12)

alw1=alw1*sc

alw2=alw2*sc

alb=albn*sc

alhomn1=alhomn1*sc

alhomn2=alhomn2*sc

B1w=B1*sc

B2w=B2*sc

! write(*,*)

! write(*,*) 'Load statistics'

! write(*,*) 'alw1,alw2',alw1,alw2

! write(*,*) 'alb,alhomn1,alhomn2',alb,alhomn1,alhomn2

! write(*,*) 'B1w,B2w',B1w,B2w

fp(i1)=Pxi

alw1a(i1)=alw1*Pxi

alw2a(i1)=alw2*Pxi

! write(*,*) 'alw1a',alw1a(i1)

! write(*,*) 'alw2a',alw2a(i1)

albna(i1)=alb*Pxi

alhomn1a(i1)=alhomn1*Pxi

alhomn2a(i1)=alhomn2*Pxi

! write(*,*) 'albna',albna(i1)

! write(*,*) 'alhomn1a',alhomn1a(i1)

! write(*,*) 'alhomn2a',alhomn2a(i1)

B1wa(i1)=B1w*Pxi

B2wa(i1)=B2w*Pxi

! write(*,*) 'B1wa',B1wa(i1)

! write(*,*) 'B2wa',B2wa(i1)

qb1(i1)=q1bar*Pxi

qb2(i1)=q2bar*Pxi

pi1b(i1)=pi1*Pxi

pi2b(i1)=pi2*Pxi

Pseg1b(i1)=Pseg1*Pxi

Pseg2b(i1)=Pseg2*Pxi

! Stores p.d.f. weighted load statistics for current value of gamma

590 continue

fpint=asum(nsimp,fp,x1a,x2a)

alw1i=asum(nsimp,alw1a,x1a,x2a)

alw2i=asum(nsimp,alw2a,x1a,x2a)

albi=asum(nsimp,albna,x1a,x2a)

alhon1i=asum(nsimp,alhomn1a,x1a,x2a)

alhon2i=asum(nsimp,alhomn2a,x1a,x2a)

B1wi=asum(nsimp,B1wa,x1a,x2a)

B2wi=asum(nsimp,B2wa,x1a,x2a)

qbw1=asum(nsimp,qb1,x1a,x2a)

qbw2=asum(nsimp,qb2,x1a,x2a)

pibw1=asum(nsimp,pi1b,x1a,x2a)

pibw2=asum(nsimp,pi2b,x1a,x2a)

Psegw1=asum(nsimp,Pseg1b,x1a,x2a)

Psegw2=asum(nsimp,Pseg2b,x1a,x2a)

alw1s=alw1s+alw1i

alw2s=alw2s+alw2i

albs=albs+albi

alhom1s=alhom1s+alhon1i

alhom2s=alhom2s+alhon2i

B1ws=B1ws+B1wi

B2ws=B2ws+B2wi

! compiles integrals of load statistics

qt1=qt1+qbw1

qt2=qt2+qbw2

pi1t=pi1t+pibw1

pi2t=pi2t+pibw2

Ptsegw1=Ptsegw1+Psegw1

Ptsegw2=Ptsegw2+Psegw2

! compiles integrals of load statistics

tt1=1.0-exp(albi-alw1i)

tt2=1.0-exp(albi-alw2i)

theta1=Psegw1/awf

theta2=Psegw2/awf

! Watterson's theta values

delth1=1-(pibw1/theta1)

delth2=1-(pibw2/theta2)

! delta-theta

write(1,*) ''

write (1,*) 'Net probability of zone 2b using Simpsons rule= ',fpint

write (1,*) ''

write (1,*) 'Contributions to mean load statistics over zone 2b'

write (1,*) 'Contributions to mean loads within In and St=',alw1i,alw2i

write (1,*) 'Contribution to load between In and St=',albi

write (1,*) 'Contributions to homozygous loads for In and St=',alhon1i,alhon2i

write (1,*) 'Contributions to inbreeding loads= ',B1wi,B2wi

write (1,*) 'Selection coefficients for In and St homokaryotypes'

write (1,*) tt1,tt2

write (1,*) ''

write (1,*) 'Selection coefficients for In and St homokaryotypes'

write (1,*) tt1,tt2

write (1,*) ''

write (1,*) 'Contributions to mean A2 freqs=',qbw1,qbw2

write (1,*) 'Contributions to mean diversities=',pibw1,pibw2

write (1,*) 'Contributions to mean proportions of seg. sites=',Psegw1,Psegw2

write (1,*) 'Contributions to delta-theta values=',delth1,delth2

write (1,*) ''

write (*,*) ''

write (*,*) 'Net probability of zone 2b using Simpsons rule= ',fpint

write (*,*) ''

write (*,*) 'Contributions to mean load statistics over zone 2b'

write (*,*) 'Contributions to mean loads within In and St=',alw1i,alw2i

write (*,*) 'Contribution to load between In and St=',albi

write (*,*) 'Contributions to homozygous loads for In and St=',alhon1i,alhon2i

write (*,*) 'Contributions to inbreeding loads= ',B1wi,B2wi

write (*,*) 'Selection coefficients for In and St homokaryotypes'

write (*,*) tt1,tt2

write (*,*) ''

write (*,*) 'Contributions to mean A2 freqs=',qbw1,qbw2

write (*,*) 'Contributions to mean freq. of seg. sites=',Psegw1,Psegw2

write (*,*) 'Contributions to delta-theta values=',delth1,delth2

write (*,*) ''

! write (*,*) 'Continue: type 1 if not desired'

! read (*,*) CONT

! allows program to be cancelled if desired

! if(CONT.eq.1) go to 400

!!!!!!!!!!!!!!!!!!!!!!!!!!!!!!!!!!!!!!!!!!!!!!!!!!!!!!!!!!!!!!!!!!!!!!!

! 3: Zone 3: strong selection approximation

! For St and In populations, q is distributed as a gamma distribution

! There is a negligible probability of a high q in both metapopns

! lower bound gamma for St is gamc2 from previous section

! upper bound gamma corresponds to upper 99th percentile of gamma distribution

610 write(*,*) ''

write(1,*) 'Zone 3: strong selection approximation'

upgam=upgfac*gambar2/ashape

write (1,*) 'Lower and upper bounds of St popn gamma= ',gamc22,upgam

write (1,*) ''

write (1,*) 'Zone 3: strong selection approximation'

write (*,*) 'Lower and upper bounds of St popn gamma= ',gamc22,upgam

write (*,*) ''

xsc=upgfac

call gser(gamser,ashape,xsc,gln)

Pz3=gamser-Ptot

write (1,*) 'Probability of zone 3= ',Pz3

write (*,*) 'Probability of zone 3= ',Pz3

write(*,*) ''

x1a=gamc2*ashape/gambar2

x2a=upgam*ashape/gambar2

! lower and upper limits of scaled gamma distribution variable for integrations

del=(upgam-gamc22)/anint

do 620 i1=1,nint

ga1=gamc22+(i1-1)*del

! write(*,*) 'i1=',i1,' gamma for St metapopn= ',ga1

! write(*,*) ''

! write(1,*) 'Index of gamma value',i1,' gamma for St metapopn= ',ga1

! write(1,*) ''

xi=ashape*ga1/gambar2

! scaled variable for gamma distribution of selection coefficients for St metapopn

Pxi=(xi**(ashape-1))*exp(-xi)

Pxi=Pxi/z

! probability density of xi

sc2=ga1/(2*ANE2)

sc=aninvs*sc2

! product of selection coefficient and number of loci for load calculations

q2b=alpha2/(2*ga1*h1)

q2var=q2b**2/alpha2

! mean and variance of frequency of A2 in St population under gamma distribution

F2=q2var/(q2b*(1-q2b))

ga4=ga1*x/(1-x)

q1b=alpha1/(2*ga4*h1)

q1var=q1b**2/alpha1

! mean and variance of frequency of A2 in In population under gamma distribution

F1=q1var/(q1b*(1-q1b))

pi1=2*q1b*(1.0-q1b)*(1-F1)

pi2=2*q2b*(1.0-q2b)*(1-F2)

! diversities for given gamma value

q21=q1var+q1bar**2

q22=q2var+q2bar**2

Pfix11=1.0-nsamp*(q1b-0.5*(nsamp-1)*q21)

Pfix12=1.0-nsamp*(q2b-0.5*(nsamp-1)*q22)

Pfix21=0

Pfix22=0

! approximations for frequencies of fixed sites

Pseg1=1-Pfix11

Pseg2=1-Pfix12

! write(*,*) ''

! write(*,*) 'Mean q1= ',q1b,' Variance= ',q1var,' F1 = ',F1

! write(*,*) 'Mean q2= ',q2b,' Variance= ',q2var,' F2 = ',F2

! write(*,*) 'Freqs. of segregation for In and St=',Pseg1,Pseg2

! write(*,*) ''

call loadstats1(q1b,q2b,h1,F1,F2,alw1,alw2,alb,alhomn1,alhomn2,B1,B2)

! this subroutine ignores covariance between q1 and q2

alw1=alw1*sc

alw2=alw2*sc

alb=alb*sc

alhomn1=alhomn1*sc

alhomn2=alhomn2*sc

B1w=B1*sc

B2w=B2*sc

! write(*,*)

! write(*,*) 'Load stats'

! write(*,*) 'alw1,alw2',alw1,alw2

! write(*,*) 'alb,alhomn1,alhomn2',alb,alhomn1,alhomn2

! write(*,*) 'B1w,B2w',B1w,B2w

alw1a(i1)=alw1*Pxi

alw2a(i1)=alw2*Pxi

! write(*,*) 'alw1a',alw1a(i1)

! write(*,*) 'alw2a',alw2a(i1)

albna(i1)=alb*Pxi

alhomn1a(i1)=alhomn1*Pxi

alhomn2a(i1)=alhomn2*Pxi

! write(*,*) 'albna',albna(i1)

! write(*,*) 'alhomn1a',alhomn1a(i1)

! write(*,*) 'alhomn2a',alhomn2a(i1)

B1wa(i1)=B1w*Pxi

B2wa(i1)=B2w*Pxi

! write(*,*) 'B1wa',B1wa(i1)

! write(*,*) 'B2wa',B2wa(i1)

qb1(i1)=q1b*Pxi

qb2(i1)=q2b*Pxi

pi1b(i1)=pi1*Pxi

pi2b(i1)=pi2*Pxi

pi1b(i1)=pi1*Pxi

pi2b(i1)=pi2*Pxi

Pseg1b(i1)=Pseg1*Pxi

Pseg2b(i1)=Pseg2*Pxi

! Stores p.d.f. weighted load statistics for current value of gamma

620 continue

alw1i=asum(nsimp,alw1a,x1a,x2a)

alw2i=asum(nsimp,alw2a,x1a,x2a)

albi=asum(nsimp,albna,x1a,x2a)

alhon1i=asum(nsimp,alhomn1a,x1a,x2a)

alhon2i=asum(nsimp,alhomn2a,x1a,x2a)

B1wi=asum(nsimp,B1wa,x1a,x2a)

B2wi=asum(nsimp,B2wa,x1a,x2a)

qbw1=asum(nsimp,qb1,x1a,x2a)

qbw2=asum(nsimp,qb2,x1a,x2a)

pibw1=asum(nsimp,pi1b,x1a,x2a)

pibw2=asum(nsimp,pi2b,x1a,x2a)

Psegw1=asum(nsimp,Pseg1b,x1a,x2a)

Psegw2=asum(nsimp,Pseg2b,x1a,x2a)

! compiles integrals of load statistics

alw1s=alw1s+alw1i

alw2s=alw2s+alw2i

albs=albs+albi

alhom1s=alhom1s+alhon1i

alhom2s=alhom2s+alhon2i

B1ws=B1ws+B1wi

B2ws=B2ws+B2wi

qt1=qt1+qbw1

qt2=qt2+qbw2

pi1t=pi1t+pibw1

pi2t=pi2t+pibw2

Ptsegw1=Ptsegw1+Psegw1

Ptsegw2=Ptsegw2+Psegw2

! contributions to mean allele frequencies and diversities for In and St

theta1=Psegw1/awf

theta2=Psegw2/awf

! Watterson's theta values

delth1=1-(pibw1/theta1)

delth2=1-(pibw2/theta2)

! delta-theta

tt1=1.0-exp(albi-alw1i)

tt2=1.0-exp(albi-alw2i)

write (1,*) ''

write (1,*) 'Mean load statistics over zone 3'

write (1,*) 'Contributions to loads within In and St=',alw1i,alw2i

write (1,*) 'Contribution to load between In and St=',albi

write (1,*) 'Contributions to homozygous loads for In and St=',alhon1i,alhon2i

write (1,*) 'Contributions to inbreeding loads= ',B1wi,B2wi

write (1,*) ''

write (1,*) 'Selection coefficients for In and St homokaryotypes'

write (1,*) tt1,tt2

write (1,*) ''

write (1,*) 'Contributions to mean A2 freqs=',qbw1,qbw2

write (1,*) 'Contributions to mean A2 freqs at seg. sites=',qbw1seg,qbw2seg

write (1,*) 'Contributions to mean diversities=',pibw1,pibw2

write (1,*) 'Contributions to delta-theta values=',delth1,delth2

write (1,*) ''

write (*,*) 'Mean load statistics over zone 3'

write (*,*) 'Contributions to loads within In and St=',alw1i,alw2i

write (*,*) 'Contribution to load between In and St=',albi

write (*,*) 'Contributions to homozygous loads for In and St=',alhon1i,alhon2i

write (*,*) 'Contributions to inbreeding loads= ',B1wi,B2wi

write (*,*) 'Selection coefficients for In and St homokaryotypes'

write (*,*) tt1,tt2

write (*,*) ''

write (*,*) 'Contributions to mean A2 freqs=',qbw1,qbw2

write (*,*) 'Contributions to mean diversities=',pibw1,pibw2

write (*,*) 'Contributions to mean freq. of seg. sites=',Psegw1,Psegw2

write (*,*) 'Contributions to delta-theta values=',delth1,delth2

write (*,*) ''

tt1s=1.0-exp(albs-alw1s)

tt2s=1.0-exp(albs-alw2s)

write (1,*) ''

write (1,*) ''

write (1,*) 'Mean load statistics over all zones'

write (1,*) 'Loads within In and St=',alw1s,alw2s

write (1,*) 'Load between In and St=',albs

write (1,*) 'Homozygous load for In and St=',alhom1s,alhom2s

write (1,*) 'Inbreeding loads= ',B1ws,B2ws

write (1,*) ''

write (1,*) 'Selection coefficients for In and St homokaryotypes'

write (1,*) tt1s,tt2s

write (1,*) ''

write (1,*) 'Mean frequencies of A2 in In and St=',qt1,qt2

rq12=qt1/qt2

write (1,*) 'Ratio of these=',rq12

write (1,*) ''

write (1,*) 'Mean diversities at selected sites in In and St=',pi1t,pi2t

write (1,*) 'Mean diversities at neutral sites in In and St=',pi1neut,pi2neut

pir1=pi1t/pi1neut

pir2=pi2t/pi2neut

write (1,*) 'pi-n/pi-s for In and St=',pir1,pir2

pir3=pir1/pir2

write (1,*) 'Ratio of these=',pir3

write (1,*) ''

write (1,*) 'Mean freqs. of seg. sites=', Ptsegw1, Ptsegw2

rPtseg=Ptsegw1/Ptsegw2

write (1,*) 'Ratio of these=',rPtseg

theta1=Ptsegw1/awf

theta2=Ptsegw2/awf

! Watterson's theta values

delth1=1-(pi1t/theta1)

delth2=1-(pi2t/theta2)

! delta-theta values

rdelt=delth1/delth2

write (*,*) 'Mean load statistics over all zones'

write (*,*) 'Loads within In and St=',alw1s,alw2s

write (*,*) 'Load between In and St=',albs

write (*,*) 'Homozygous load for In and St=',alhom1s,alhom2s

write (*,*) 'Inbreeding loads= ',B1ws,B2ws

write (*,*) 'Selection coefficients for In and St homokaryotypes'

write (*,*) tt1s,tt2s

write (*,*) ''

write (*,*) 'Mean frequencies of A2 in In and St=',qt1,qt2

rq12=qt1/qt2

write (*,*) 'Ratio of these=',rq12

write (*,*) ''

write (*,*) 'Mean diversities at selected sites in In and St=',pi1t,pi2t

write (*,*) 'Mean diversities at neutral sites in In and St=',pi1neut,pi2neut

pir1=pi1t/pi1neut

pir2=pi2t/pi2neut

write (*,*) 'pi-n/pi-s for In and St=',pir1,pir2

pir3=pir1/pir2

write (*,*) 'Ratio of these=',pir3

write (*,*) ''

write (*,*) 'Mean freqs. of seg. sites=', Ptsegw1, Ptsegw2

rPtseg=Ptsegw1/Ptsegw2

write (*,*) 'Ratio of these=',rPtseg

theta1=Ptsegw1/awf

theta2=Ptsegw2/awf

! Watterson's theta values

delth1=1-(pi1t/theta1)

delth2=1-(pi2t/theta2)

! delta-theta values

write (1,*) 'Overall delta-theta values=',delth1,delth2

rdelt=delth1/delth2

write (1,*) 'Ratio of these=',rdelt

write (*,*) 'Overall delta-theta values=',delth1,delth2

rdelt=delth1/delth2

write (*,*) 'Ratio of these=',rdelt

410 continue

400 end program invload2

function gammln(z)

! computes log gamma function of real z>0 using Lanczos approximation

real :: p(8),pia,t,y,sa,x

DATA p/676.5204,-1259.139,771.323,-176.6150,12.5073,-0.1386,9.98436e-6,1.5056e-7/

pia=log(2.50666)

sa=1.0

y=z

do 10, i=1,8

y=y+1

sa=sa+p(i)/y

10 continue

t=z+7.5

x=(z+0.5)*log(t)-t

x=x+pia+log(sa)

x=x-log(z)

gammln=x

end function gammln

function gammq(a,z)

real :: a,b,gammq,z

! Uses gcf,gser

! Returns the incomplete gamma function Q(a, z) ≡ 1 − P (a, z)

real :: gammcf,gamser,gln

if(x.lt.0.0.or.a.le.0.0) then

write (*,* )''

write (*,* ) 'Bad arguments in gammq'

write (*,* )''

go to 10

end if

b=a+1.0

if(x.lt.b)then

! Use the series representation and take its complement

call gser(gamser,a,z,gln)

gammq=1.0-gamser

else

! Use the continued fraction representation

call gcf(gammcf,a,z,gln)

gammq=gammcf

endif

10 end function gammq

subroutine gser(gamser,a,z,gln)

integer :: ITMAX,n

real :: a,gamser,gln,z,EPS

real :: ap,del,sum,gammln

ITMAX=100

EPS=3.0e-5

! Uses gammln

! Returns the incomplete gamma function P(a,x) evaluated by its series representation as

! gamser. Also returns lnΓ(a) as gln.

gln=gammln(a)

! write(*,*) 'a= ',a,' z= ',z,'gamm(a) = ',gln

if(z.le.0.0) then

gamser=0.0

if(z.lt.0.0) then

write(*,*) ''

write(*,*) 'x < 0 in gser'

write(*,*) ''

go to 10

end if

end if

ap=a

sum=1.0/a

del=sum

do 15 n=1,ITMAX

ap=ap+1.0

del=del*z/ap

sum=sum+del

! write(*,*) 'n = ',n,'ap= ',ap,' del= ',del,' sum= ',sum

! if(abs(del).lt.abs(sum*EPS)) goto 20

! write(*,*) 'a too large, ITMAX too small in gser'

! write(*,*) ''

! go to 10

15 continue

20 gamser=sum*exp(-z+a*log(z)-gln)

! write(*,*) 'sum= ',sum,' gamser= ',gamser

10 end subroutine gser

subroutine gcf(gammcf,a,z,gln)

real :: a,gammcf,gln,EPS,FPMIN

real :: an,b,c,d,del,h,z,gammln

integer :: ITMAX,i

ITMAX=100

EPS=3.0e-5

FPMIN=1.0e-30

! Uses gammln

! Returns the incomplete gamma function Q(a, x) evaluated by its continued fraction gammcf representation

! IMAX is the maximum number of iterations

! EPS is the relative accuracy; FPMIN is close to the smallest floating point number permissible

! Set up for evaluating continued fraction by modified Lentz’s method with b0 = 0

gln=gammln(a)

b=z+1.0-a

c=1.0/FPMIN

d=1.0/b

h=d

do 15 i=1,ITMAX

an=0.0-i*(i-a)

b=b+2.0

d=an*d+b

if(abs(d).lt.FPMIN) then

d=FPMIN

end if

c=b+an/c

if(abs(c).lt.FPMIN) then

c=FPMIN

end if

d=1.0/d

del=d*c

h=h*del

if(abs(del-1.0).lt.EPS) goto 10

15 continue

write (*,*) ''

write (*,*) 'Problem with incomplete gamma function'

write (*,*) 'a too large, ITMAX too small in gcf'

write (*,*) ''

goto 20

10 gammcf=exp(-z+a*log(x)-gln)*h

20 end subroutine gcf

function asi(nsamp)

! calculates harmonic series

integer :: nsamp,n1

real :: asi,ai

n1=nsamp-1

asi=0.0

do 10 i=1,n1

ai=i

asi=asi+1.0/ai

10 continue

end function asi

function betamom(alpha,beta,n)

! calculates nth moment of beta distribution

integer :: i,n

real :: alpha,beta

x=0

y=0

do 10 i=1,n

x1=alpha+i-1

y1=alpha+beta+i-1

x=x+log(x1)

y=y+log(y1)

10 continue

z=x-y

betamom=exp(z)

end function betamom

function asum(nsimp,f,x1,x2)

! uses Simpson's rule

real :: f(200),x,x1,x2,F1,DX,ans

integer :: nsimp,N1,i,j

! write(*,*) 'Simpson integration'

ans=nsimp

DX=(x2-x1)/ans

j=0

N1=nsimp+1

asum=0.0

! write(*,*) 'DX',DX

do 50 i=1,N1

F1=f(i)

x=x1+(i-1)*DX

! write(*,*) 'i= ',i,'x= ',x,' f= ',F1

40 if(i.eq.N1) then

F1=f(N1)

asum=asum+F1

go to 60

end if

if(i.eq.1) then

asum=F1

go to 50

end if

if(j.eq.0) then

asum=asum+4*F1

j=1

else

asum=asum+2*F1

j=0

end if

50 continue

60 asum=DX*asum/(3.0)

! write(*,*) 'asum=',asum

end function asum

subroutine loadstats1(qbar1,qbar2,h1,F1,F2,alwn1,alwn2,albn,alhomn1,alhomn2,B1,B2)

real :: alwn1,alwn2,albn,alhomn1,alhomn2,B1,B2,qbar1,qbar2,F1,F2,h1,h2

! calculates variables for load statistics for In and St without using selection coefficients

h2=h1

pbar1=1-qbar1

pbar2=1-qbar2

alwn1=qbar1*(2*h2+(1-2*h2)*(qbar1+F1*pbar1))

alwn2=qbar2*(2*h2+(1-2*h2)*(qbar2+F2*pbar2))

! variable for total loads within In and St

albn=h1*(qbar1*pbar2+qbar2*pbar1)+qbar1*qbar2

! variable for between-arrangement load for In/St

alhomn1=qbar1

alhomn2=qbar2

! variables for homozygous loads for neutral regions for In and St

B1=alhomn1-alwn1

B2=alhomn2-alwn2

! variables for inbreeding loads for neutral regions for In and St

end subroutine loadstats1

subroutine loadstats2(qbar1,qbar2,h1,F1,F2,alwn1,alwn2,albn,alhomn1,alhomn2,B1,B2,cp12)

real :: alwn1,alwn2,albn,alhom1,alhom2,B1,B2,cp12,qbar1,qbar2,F1,F2,h1,h2

! calculates variables for load statistics for In and St without using selection coefficients

! includes covariance between q1 and q2 for between-arrangement load

h2=h1

pbar1=1-qbar1

pbar2=1-qbar2

alwn1=qbar1*(2*h2+(1-2*h2)*(qbar1+F1*pbar1))

alwn2=qbar2*(2*h2+(1-2*h2)*(qbar2+F2*pbar2))

! variable for total loads within In and St

albn=(h2*(qbar1*pbar2+qbar2*pbar1))+(qbar1*qbar2)+((1-2*h2)*cp12)

! variable for arrangement load for neutral region for In/St

alhomn1=qbar1

alhomn2=qbar2

! variables for homozygous loads for neutral regions for In and St

B1=alhomn1-alwn1

B2=alhomn2-alwn2

! variables for inbreeding loads for neutral regions for In and St

end subroutine loadstats2

subroutine bivpdf(gamma,a1,a2,b11,b12,b22,alpha1,alpha2,beta1,beta2,nsimp,f,f1,f2,f3,f4,f5,f6)

real :: gamma,a1,a2,b11,b12,b22,alpha1,alpha2,beta1,beta2,e2,f(1000,1000)

real :: f1(1000),f2(1000),f3(1000),f4(1000),f5(1000),f6(1000),f7(1000)

integer :: i,j,nsimp,nint

! evaluates marginal distributions of q1 and q2

if(gamma.le.10) then

e1=0.005

e2=0.005

else

e1=0.01/(2*gamma)

e2=0.01/(2*gamma)

end if

! write(*,*) 'e1= ',e1,'e2= ',e2

! write(*,*) 'a2=',a2,' gamma= ',gamma

al1=0.0-log(alpha1)

al2=0.0-log(alpha2)

bl1=0.0-log(beta1)

bl2=0.0-log(beta2)

x1=e1

x2=1-e1

ale1=log(e1)

y1=e2

y2=1-e2

ale2=log(e2)

! write(*,*) 'Inside bivpdf'

! write(*,*) 'alpha1,alpha2',alpha1,alpha2!

! write(*,*) 'beta1,beta2',beta1,beta2

! write(*,*) 'gamma',gamma

! write(*,*) 'e1,e2',e1,e2

! write(*,*) 'al1,al2',al1,al2

! write(*,*) 'bl1,bl2',bl1,bl2

! write(*,*) 'ale1,ale2',ale1,ale2

! boundary values

nint=nsimp+1

anint=nsimp

del1=(x2-x1)/anint

del2=(y2-y1)/anint

! write(*,*) 'Evaluation of components of p.d.f.'

do 10 i=1,nint

f6(i)=0

f5(i)=0

! initialises joint distributions for interior

x=x1+(i-1)*del1

y=y1+(i-1)*del2

! write(*,*) 'i=',i,' x= ',x,' y= ',y

alx1=log(x)

alx2=log(1-x)

aly1=log(y)

aly2=log(1-y)

f1(i)=0.0-2*gamma*(a1*x+b11*x**2)

f1(i)=f1(i)+(alpha1-1)*alx1+(beta1-1)*alx2+alpha2*ale2+al2

f1(i)=exp(f1(i))

if(f1(i).le.0.00001) then

f1(i)=0

end if

! interior distribution of x with y close to zero (without constant of integration)

f2(i)=0.0-2*gamma*(a2+b22+(a1+b12)*x+b11*x**2)+(alpha1-1)*alx1+(beta1-1)*alx2

f2(i)=f2(i)+beta2*ale2+bl2

f2(i)=exp(f2(i))

if(f2(i).le.0.00001) then

f2(i)=0

end if

! interior distribution of x with y close to 1

f3(i)=0.0-2*gamma*(a2*y+b22*y**2)

f3(i)=f3(i)+(alpha2-1)*aly1+(beta2-1)*aly2+alpha1*ale1+al1

f3(i)=exp(f3(i))

if(f3(i).le.0.00001) then

f3(i)=0

end if

! interior distribution of y with x close to zero

f4(i)=0.0-2*gamma*(a1+b11+(a2+b12)*y+b22*y**2)+(alpha2-1)*aly1+(beta2-1)*aly2

f4(i)=f4(i)+beta1*ale1+bl1

f4(i)=exp(f4(i))

if(f4(i).le.0.00001) then

f4(i)=0

end if

! interior distribution of y with x close to 1

! write(*,*) 'f1,f2,f3,f4',f1(i),f2(i),f3(i),f4(i)

! write(*,*) ''

do 20 j=1,nint

y=y1+(j-1)*del2

aly1=log(y)

aly2=log(1-y)

z=a1*x+a2*y+b11*(x**2)+b12*x*y+b22*(y**2)

f(i,j)=0.0-2*gamma*z+(alpha1-1)*alx1+(beta1-1)*alx2

f(i,j)=f(i,j)+(alpha2-1)*aly1+(beta2-1)*aly2

f(i,j)=exp(f(i,j))

if(f(i,j).le.0.000001) then

f(i,j)=0

end if

fij=f(i,j)

! write(*,*) 'j= ',j,'f(i,j)= ',fij

! bivariate distribution for q1 and q2 inside boundaries

20 continue

10 continue

do 30 i=1,nint

do 40 j=1,nint

f7(j)=f(i,j)

40 continue

f5(i)=asum(nsimp,f7,y1,y2)

if(f5(i).le.0.00001) then

f5(i)=0

end if

f5i=f5(i)

! write(*,*) ''

! write(*,*) 'i= ',i,' f5= ',f5i

30 continue

! marginal distribution for q1 inside boundaries

do 50 j=1,nint

do 60 i=1,nint

f7(i)=f(i,j)

60 continue

f6(j)=asum(nsimp,f7,x1,x2)

if(f6(i).le.0.00001) then

f6(i)=0

end if

f6j=f6(j)

! write(*,*) ''

! write(*,*) 'j= ',j,' f6= ',f6j

50 continue

! marginal distribution for q2 inside boundaries

end subroutine bivpdf

subroutine integ1(gamma,a1,a2,b11,b12,b22,alpha1,alpha2,beta1,beta2,nsimp,ainteg,aintegs,cp12)

real :: x,y,gamma,alpha1,beta1,alpha2,beta2,g(1000),ainteg0,ainteg(2,2),g1(1000),g2(1000,0:3,2)

real :: x1,x2,x3,ai,a1,a2,b11,b12,b22,f1(1000),f2(1000),f3(1000),f4(1000),f5(1000),f6(1000),aintegs

real :: f0(1000,1000),g3(1000,1000),g4(1000),cp(1000),cp12,gcp(1000)

integer :: nsimp,k,j,i

! calculates integral of the bivariate p.d.f.and the moments of q1 and q2

! k is the index for the non-zero moments; j=0 indicates 0th moment

! j=1 indicates the index of the moment of q1; j=2 indicates the moment of q2

if(gamma.le.10) then

e1=0.001

e2=0.001

else

e1=0.002/(2*gamma)

e2=0.002/(2*gamma)

end if

! boundaries for x and y (corresponding to near neutrality)

al1=1.0/alpha1

al2=1.0/alpha2

bl1=1.0/beta1

bl2=1.0/beta2

! write(*,*) 'al1= ',al1,'al2= ',al2

! write(*,*) 'bl1= ',bl1,'bl2= ',bl2

ep3=e1**alpha1

ep4=e2**alpha2

ep5=e1**beta1

ep6=e2**beta2

ep7=exp(-2*gamma*(a1+a2+b11+b12+b22))

! both x and y = 1

ep8=exp(-2*gamma*(a2+b22))

! y=1

ep9=exp(-2*gamma*(a1+b11))

! x=1

x1=e1

x2=1-e1

y1=e2

y2=1-e2

! write(*,*)

! write(*,*) 'ep3,ep4,ep5,ep6',ep3,ep4,ep5,ep6

! write(*,*) 'ep7,ep8,ep9',ep7,ep8,ep9

nint=nsimp+1

anint=nsimp

del1=(x2-x1)/anint

del2=(y2-y1)/anint

! write(*,*) ''

! write(*,*) 'x1= ',x1,' y1= ',y1

! write(*,*) 'de1= ',del1,' del2= ',del2

! write(*,*) ''

call bivpdf(gamma,a1,a2,b11,b12,b22,alpha1,alpha2,beta1,beta2,nsimp,f0,f1,f2,f3,f4,f5,f6)

!!!!!!!!!!!!!!!!!!!!!!!!!!!!!!!!!!!!!!!!!!!!!!!!!!!!!!!!!!!!!!!!!!!!!!!!!!!!!!!!!!

ainteg0=0

crossp=0

! initialises components of integration constant and crossproduct

do 205 j=1,2

do 207 k=1,2

ainteg(j,k)=0

207 continue

205 continue

! initialise integrals of internal marginal distributions

do 200 j=0,2

! write(*,*) 'Index for integ1= ',j

! Section 1: x and y close to zero

! write(*,*) 'Section 1'

if(j.eq.0) then

ainteg0=al1*al2*ep3*ep4

! write(*,*) 'ainteg0= ',ainteg0

! stores initial value of the constant of integration of the p.d.f.

cross1=(e1*ep3*e2*ep4)/((alpha1+1)*(alpha2+1))

! 1st contribution to crossproduct

go to 100

end if

do 95 k=1,2

! write(*,*) 'Power is k= ',k

if(j.eq.1) then

sum1=al2*ep3*ep4*(e1**k)/(alpha1+k)

if(sum1.le.0.00001) then

sum1=0

end if

else

sum1=al1*ep3*ep4*(e2**k)/(alpha2+k)

if(sum1.le.0.00001) then

sum1=0

end if

end if

! write(*,*) 'sum1= ',sum1

ainteg(j,k)=ainteg(j,k)+sum1

95 continue

! Section 2: x close to zero and y close to 1

! write(*,*) 'Section 2'

100 if(j.eq.0) then

sum1=al1*bl2*ep3*ep6*ep8

! write(*,*) 'sum1=',sum1

if(sum1.le.0.00001) then

sum1=0

end if

ainteg0=ainteg0+sum1

! write(*,*) 'ainteg0= ',ainteg0

cross2=(e1*sum1*alpha1/(alpha1+1))

crossp=crossp+cross2

! 2nd contribution to crossproduct

go to 110

end if

do 115 k=1,2

! write(*,*) 'Power is k= ',k

if(j.eq.1) then

sum1=bl2*ep3*ep6*ep8*(e1**k)/(alpha1+k)

if(sum1.le.0.00001) then

sum1=0

end if

else

sum1=al1*bl2*ep3*ep6*ep8

if(sum1.le.0.00001) then

sum1=0

end if

end if

! write(*,*) 'sum1= ',sum1

ainteg(j,k)=ainteg(j,k)+sum1

115 continue

! Section 3: x close to 1 and y close to 0

! write(*,*) 'Section 3'

110 if(j.eq.0) then

sum1=bl1*al2*ep4*ep5*ep9

! write(*,*) 'sum1=',sum1

if(sum1.le.0.00001) then

sum1=0

end if

ainteg0=ainteg0+sum1

! write(*,*) 'ainteg0= ',ainteg0

cross3=(e2*sum1*alpha2/(alpha2+1))

crossp=crossp+cross3

! 3rd contribution to crossproduct

go to 120

end if

do 117 k=1,2

if(j.eq.1) then

sum1=bl1*al2*ep4*ep5*ep9

if(sum1.le.0.00001) then

sum1=0

end if

else

sum1=bl1*al2*ep4*ep5*ep9

sum1=sum1*alpha2*(e2**k)/(alpha2+k)

if(sum1.le.0.00001) then

sum1=0

end if

end if

! write(*,*) 'sum1= ',sum1

ainteg(j,k)=ainteg(j,k)+sum1

117 continue

! Section 4: x close to 1 and y close to 1

! write(*,*) 'Section 4'

120 sum1=bl1*bl2*ep5*ep6*ep7

! write(*,*) 'sum1=',sum1

if(sum1.le.0.00001) then

sum1=0

end if

if(j.eq.0) then

ainteg0=ainteg0+sum1

! write(*,*) 'ainteg0= ',ainteg0

crossp4=sum1

crossp=crossp+crossp4

! 4th contribution to crossproduct

go to 130

end if

! write(*,*) 'sum1= ',sum1

do 118 k=1,2

sum1=bl1*bl2*ep5*ep6*ep7

if(sum1.le.0.00001) then

sum1=0

end if

! write(*,*) 'sum1= ',sum1

ainteg(j,k)=ainteg(j,k)+sum1

118 continue

do 125 k=1,2

ainteg(j,k)=ainteg(j,k)+sum1

125 continue

! Section 5: x close to 0 and y in interior

! write(*,*) 'Section 5'

130 do 10 i=1,nint

y=y1+(i-1)*del2

if(j.eq.0) then

gcp(i)=y*f3(i)*e1*alpha1/(alpha1+1)

! integral of xy over boundary x marginal distribution of y

go to 10

end if

do 15 k=1,2

if(j.eq.1) then

g2(i,j,k)=f3(i)*(e1**k)*alpha1/(alpha1+k)

! integral of x^k over boundary x marginal distribution of y

else

g2(i,j,k)=f3(i)*y**k

end if

15 continue

10 continue

if(j.eq.0) then

ainteg0=ainteg0+asum(nsimp,f3,y1,y2)

! write(*,*) 'ainteg0= ',ainteg0

cross5=asum(nsimp,gcp,y1,y2)

crossp=crossp+cross5

! 5th contribution to crossproduct

go to 140

end if

do 17 k=1,2

do 19 i=1,nint

g1(i)=g2(i,j,k)

19 continue

ainteg(j,k)=ainteg(j,k)+asum(nsimp,g1,y1,y2)

17 continue

! Section 6: x close to 1 and y in interior

! write(*,*) 'Section 6'

140 do 20 i=1,nint

y=y1+(i-1)*del2

if(j.eq.0) then

gcp(i)=f4(i)*y

go to 20

end if

do 25 k=1,2

if(j.eq.1) then

g2(i,j,k)=f4(i)

else

g2(i,j,k)=f4(i)*y**k

end if

! uses marginal distribution for y with x close to 1

25 continue

20 continue

if(j.eq.0) then

ainteg0=ainteg0+asum(nsimp,f4,y1,y2)

! write(*,*) 'ainteg0= ',ainteg0

cross6=asum(nsimp,gcp,y1,y2)

crossp=crossp+cross6

! 6th contribution to crossproduct

! write(*,*) 'ainteg0= ',ainteg0

go to 18

end if

do 27 k=1,2

do 29 i=1,nint

g1(i)=g2(i,j,k)

29 continue

sum1=asum(nsimp,g1,y1,y2)

! write(*,*) 'k= ',k

! write(*,*) 'sum1= ',sum1

ainteg(j,k)=ainteg(j,k)+asum(nsimp,g1,y1,y2)

27 continue

! uses marginal distribution for y with x close to 1

! Section 7: y close to 0 and x in interior

! write(*,*) 'Section 7'

18 do 30 i=1,nint

x=x1+(i-1)*del1

if(j.eq.0) then

gcp(i)=x*f1(i)*e2*alpha2/(alpha2+1)

go to 30

end if

do 35 k=1,2

if(j.eq.1) then

g2(i,j,k)=f1(i)*x**k

! write(*,*) 'i,x,j,k',i,x,j,k,'g2(i,j,k)= ',g2(i,j,k)

else

g2(i,j,k)=f1(i)*alpha2*(e2**k)/(alpha2+k)

! write(*,*) 'i,x,j,k',i,y,j,k,'g2(i,j,k)= ',g2(i,j,k)

end if

35 continue

30 continue

if(j.eq.0) then

ainteg0=ainteg0+asum(nsimp,f1,x1,x2)

! write(*,*) 'ainteg0= ',ainteg0

cross7=asum(nsimp,gcp,x1,x2)

crossp=crossp+cross7

! 7th contribution to crossproduct

! write(*,*) 'ainteg3= ',ainteg3

go to 145

end if

do 37 k=1,2

do 39 i=1,nint

g1(i)=g2(i,j,k)

! write(*,*) 'i',i,'g1= ',g1(i)

39 continue

ainteg(j,k)=ainteg(j,k)+asum(nsimp,g1,x1,x2)

37 continue

! uses marginal distribution for x with y close to 0

! Section 8: y close to 1 and x in interior

! write(*,*) 'Section 8'

145 do 40 i=1,nint

x=x1+(i-1)*del1

if(j.eq.0) then

gcp(i)=x*f2(i)

go to 40

end if

do 45 k=1,2

if(j.eq.1) then

! write(*,*) 'j=',j

! write(*,*) 'Iteration of x=',i,x

! write(*,*) 'g1(i)',g1(i)

g2(i,j,k)=f2(i)*x**k

! write(*,*) 'g2(i,j,k)=',g2(i,j,k)

else

g2(i,j,k)=f2(i)

end if

45 continue

40 continue

if(j.eq.0) then

ainteg0=ainteg0+asum(nsimp,f2,x1,x2)

! write(*,*) 'ainteg0= ',ainteg0

cross8=asum(nsimp,gcp,x1,x2)

crossp=crossp+cross8

! 8th contribution to crossproduct

go to 150

end if

do 47 k=1,2

do 49 i=1,nint

g1(i)=g2(i,j,k)

49 continue

sum1=asum(nsimp,g1,x1,x2)

! write(*,*) 'k= ',k

! write(*,*) 'sum1= ',sum1

ainteg(j,k)=ainteg(j,k)+asum(nsimp,g1,x1,x2)

47 continue

! uses marginal distribution for x with y close to 1

! Section 9: x and y in interior

! write(*,*) 'Section 9'

150 if(j.eq.0) then

do 57 i1=1,nint

x=x1+(i1-1)*del1

do 55 j1=1,nsimp

y=y1+(j1-1)*del2

g3(i1,j1)=x*y*f0(i1,j1)

! for crossproduct of q1 and q2

55 continue

57 continue

end if

do 60 k=1,2

if(j.eq.1) then

do 50 i=1,nint

x=x1+(i-1)*del1

! write(*,*) 'i= ',i,'x =',x,' f5(i)= ',f5(i)

g2(i,1,k)=f5(i)*x**k

! write(*,*) 'g2= ',g2(i,1,k)

! for moment of q1

50 continue

end if

if(j.eq.2) then

do 53 i2=1,nint

y=y1+(i2-1)*del2

! write(*,*) 'i= ',i2,'y =',y,' f6(i)= ',f6(i2)

g2(i2,2,k)=f6(i2)*y**k

! write(*,*) 'g2= ',g2(i2,2,k)

! for moment of q2

53 continue

end if

60 continue

do 58 k=1,2

do 59 i=1,nint

g1(i)=g2(i,j,k)

! write(*,*) 'g1= ',g1(i)

if(k.eq.1) then

do 65 j1=1,nint

g4(j1)=g3(i,j1)

65 continue

cp(i)=asum(nsimp,g4,y1,y2)

! integral of crossproduct over y

end if

59 continue

if(j.eq.1) then

ainteg(j,k)=ainteg(j,k)+asum(nsimp,g1,x1,x2)

end if

if(j.eq.2) then

ainteg(j,k)=ainteg(j,k)+asum(nsimp,g1,y1,y2)

end if

58 continue

if(j.eq.0) then

cp12=asum(nsimp,cp,x1,x2)

! integral of crossproduct over x and y in interior

! write(*,*) 'Crossproduct of x and y over interior= ',cp12

! write(*,*) 'Crossproduct of x and y over boundaries= ',crossp

cp12=crossp+cp12

ainteg0=ainteg0+asum(nsimp,f5,x1,x2)

! write(*,*) 'ainteg0= ',ainteg0

aintegs=ainteg0

! write(*,*) 'Integration constant 1= ',aintegs

end if

200 continue

! write(*,*) 'Final components of moments matrix'

! write(*,*) '1st moments',ainteg(1,1),ainteg(2,1)

! write(*,*) '2nd moments',ainteg(1,2),ainteg(2,2)

end subroutine integ1

subroutine integfix1(gamma,a1,a2,b11,b12,b22,alpha1,alpha2,beta1,beta2,nsimp,ainteg,n)

real :: x,y,gamma,alpha1,beta1,alpha2,beta2,g(1000),ainteg(2),g1(1000),g2(0:1000,2),f0(1000,1000)

real :: x1,x2,x3,ai,a1,a2,b11,b12,b22,f1(1000),f2(1000),f3(1000),f4(1000),f5(1000),f6(1000)

integer :: nsimp,k,j,i,n

! calculates the fixation frequency of A1 from the bivariate distribution

! distribution is multiplied by (1-q)^n

! j=1 indicates q1; j=2 indicates q2

! n is the sample size for determining fixation frequencies

a1n=2*n

if(gamma.le.aln) then

e1=0.005/a1n

e2=0.005/a1n

else

e1=0.005/(2*gamma)

e2=0.005/(2*gamma)

end if

! boundaries for x and y (corresponding to near neutrality)

al1=1.0/alpha1

al2=1.0/alpha2

bl1=1.0/beta1

bl2=1.0/beta2

! write(*,*) 'al1= ',al1,'al2= ',al2

! write(*,*) 'bl1= ',bl1,'bl2= ',bl2

ep3=e1**alpha1

ep4=e2**alpha2

ep5=e1**beta1

ep6=e2**beta2

ep7=exp(-2*gamma*(a1+a2+b11+b12+b22))

! both x and y = 1

ep8=exp(-2*gamma*(a2+b22))

! y=1

ep9=exp(-2*gamma*(a1+b11))

! x=1

x1=e1

x2=1-e1

y1=e2

y2=1-e2

! write(*,*)

! write(*,*) 'ep3,ep4,ep5,ep6',ep3,ep4,ep5,ep6

! write(*,*) 'ep7,ep8,ep9',ep7,ep8,ep9

nint=nsimp+1

anint=nsimp

del1=(x2-x1)/anint

del2=(y2-y1)/anint

call bivpdf(gamma,a1,a2,b11,b12,b22,alpha1,alpha2,beta1,beta2,nsimp,f0,f1,f2,f3,f4,f5,f6)

!!!!!!!!!!!!!!!!!!!!!!!!!!!!!!!!!!!!!!!!!!!!!!!!!!!!!!!!!!!!!!!!!!!!!!!!!!!!!!!!!!

do 205 j=1,2

ainteg(j)=0

205 continue

! initialise integrals of distributions for In and St

! note that integration constant for whole distribution was determined by integ1

do 200 j=1,2

! write(*,*) 'Index for integfix1= ',j

! j=0 for integration constant; j=1 for In, j=2 for St

! Section 1: x and y close to zero

! write(*,*) 'Section 1'

sum1=al1*al2*ep3*ep4

if(j.eq.1) then

sum1=sum1-(n+beta1-1)*e1*ep3*ep4*al2/(alpha1+1)

else

sum1=sum1-(n+beta2-1)*e2*ep3*ep4*al1/(alpha2+1)

end if

if(sum1.le.0.00001) then

sum1=0

end if

! write(*,*) 'sum1= ',sum1

ainteg(j)=ainteg(j)+sum1

! Section 2: x close to zero and y close to 1

! write(*,*) 'Section 2'

! write(*,*) 'Index for integfix1= ',j

if(j.eq.1) then

sum1=al1*ep3*bl2*ep6*ep8

sum1=sum1-(n+beta1-1)*e1*ep3*bl2*ep6*ep8/(alpha1+1)

if(sum1.le.0.00001) then

sum1=0

end if

else

sum1=0

end if

! write(*,*) 'sum1= ',sum1

ainteg(j)=ainteg(j)+sum1

! Section 3: x close to 1 and y close to 0

! write(*,*) 'Section 3'

! write(*,*) 'Index for integfix1= ',j

if(j.eq.1) then

sum1=0

else

sum1=bl1*al2*ep4*ep5*ep9

sum1=sum1-(n+beta2-1)*e2*bl1*ep4*ep5*ep9/(alpha2+1)

if(sum1.le.0.00001) then

sum1=0

end if

end if

! write(*,*) 'sum1= ',sum1

ainteg(j)=ainteg(j)+sum1

! Section 4: x close to 1 and y close to 1

! write(*,*) 'Section 4'

! write(*,*) 'Index for integfix1= ',j

sum1=0

! Section 5: x close to 0 and y in interior

! write(*,*) 'Section 5'

130 do 10 i=1,nint

y=y1+(i-1)*del2

if(j.eq.1) then

g2(i,j)=f3(i)

g2(i,j)=g2(i,j)-(n+beta1-1)*e1*alpha1*f3(i)/(alpha1+1)

! integral for x over boundary x marginal distribution of y

else

g2(i,j)=f3(i)*((1-y)**n)

end if

! integral for y over boundary x marginal distribution of y

10 continue

do 19 i=1,nint

g1(i)=g2(i,j)

19 continue

ainteg(j)=ainteg(j)+asum(nsimp,g1,y1,y2)

17 continue

! write(*,*) 'Index for integfix1= ',j

! write(*,*) 'ainteg=',ainteg(j)

! write(*,*) ''

! Section 6: x close to 1 and y in interior

! write(*,*) 'Section 6'

140 do 20 i=1,nint

y=y1+(i-1)*del2

if(j.eq.1) then

g2(i,j)=0.0

! integral for x over boundary x marginal distribution of y

else

g2(i,j)=f4(i)*((1-y)**n)

! integral for y over boundary x marginal distribution of y

end if

20 continue

do 29 i=1,nint

g1(i)=g2(i,j)

29 continue

ainteg(j)=ainteg(j)+asum(nsimp,g1,y1,y2)

! uses marginal distribution for y with x close to 1

! write(*,*) 'Index for integfix1= ',j

! write(*,*) 'ainteg=',ainteg(j)

! write(*,*) ''

! Section 7: y close to 0 and x in interior

! write(*,*) 'Section 7'

18 do 30 i=1,nint

x=x1+(i-1)*del1

if(j.eq.1) then

g2(i,j)=f1(i)*((1-x)**n)

else

g2(i,j)=f1(i)

g2(i,j)=g2(i,j)-(n+beta2-1)*e2*alpha2*f1(i)/(alpha2+1)

end if

30 continue

do 39 i=1,nint

g1(i)=g2(i,j)

! write(*,*) 'i',i,'g1= ',g1(i)

39 continue

ainteg(j)=ainteg(j)+asum(nsimp,g1,x1,x2)

! uses marginal distribution for x with y close to 0

! write(*,*) 'Index for integfix1= ',j

! write(*,*) 'ainteg=',ainteg(j)

! write(*,*) ''

! Section 8: y close to 1 and x in interior

! write(*,*) 'Section 8'

145 do 40 i=1,nint

x=x1+(i-1)*del1

if(j.eq.1) then

! write(*,*) 'j=',j

g2(i,j)=f2(i)*((1-x)**n)

! write(*,*) 'g2(i,j=',g2(i,j)

else

g2(i,j)=0.0

end if

40 continue

do 49 i=1,nint

g1(i)=g2(i,j)

49 continue

ainteg(j)=ainteg(j)+asum(nsimp,g1,x1,x2)

! uses marginal distribution for x with y close to 1

! write(*,*) 'Index for integfix1= ',j

! write(*,*) 'ainteg=',ainteg(j)

! write(*,*) ''

! Section 9: x and y in interior

! write(*,*) 'Section 9'

if(j.eq.1) then

do 50 i=1,nint

x=x1+(i-1)*del1

g2(i,j)=f5(i)*(1-x)**n

! moment of q1

50 continue

end if

if(j.eq.2) then

do 53 i2=1,nint

y=y1+(i2-1)*del2

g2(i2,j)=f6(i2)*(1-y)**n

! moment of q2

53 continue

end if

do 59 i=1,nint

g1(i)=g2(i,j)

59 continue

ainteg(j)=ainteg(j)+asum(nsimp,g1,x1,x2)

! write(*,*) 'Index for integfix1= ',j

! write(*,*) 'ainteg=',ainteg(j)

! write(*,*) ''

200 continue

end subroutine integfix1

subroutine integfix2(gamma,a1,a2,b11,b12,b22,alpha1,alpha2,beta1,beta2,nsimp,ainteg,ainteg0,n)

real :: x,y,gamma,alpha1,beta1,alpha2,beta2,g(1000),ainteg(2),g1(1000),g2(0:1000,2),f0(1000,1000)

real :: x1,x2,x3,ai,a1,a2,b11,b12,b22,f1(1000),f2(1000),f3(1000),f4(1000),f5(1000),f6(1000),ainteg0

integer :: nsimp,k,j,i,n

! calculates the fixation frequency of A2 from the bivariate distribution

! distribution is multiplied by q^n

! j=1 indicates q1; j=2 indicates q2

! n is the sample size for determining fixation frequencies

a1n=2*n

if(gamma.le.aln) then

e1=0.005/a1n

e2=0.005/a1n

else

e1=0.005/(2*gamma)

e2=0.005/(2*gamma)

end if

! boundaries for x and y (corresponding to near neutrality)

al1=1.0/alpha1

al2=1.0/alpha2

bl1=1.0/beta1

bl2=1.0/beta2

! write(*,*) 'al1= ',al1,'al2= ',al2

! write(*,*) 'bl1= ',bl1,'bl2= ',bl2

ep3=e1**alpha1

ep4=e2**alpha2

ep5=e1**beta1

ep6=e2**beta2

ep7=exp(-2*gamma*(a1+a2+b11+b12+b22))

! both x and y = 1

ep8=exp(-2*gamma*(a2+b22))

! y=1

ep9=exp(-2*gamma*(a1+b11))

! x=1

x1=e1

x2=1-e1

y1=e2

y2=1-e2

! write(*,*)

! write(*,*) 'ep3,ep4,ep5,ep6',ep3,ep4,ep5,ep6

! write(*,*) 'ep7,ep8,ep9',ep7,ep8,ep9

nint=nsimp+1

anint=nsimp

del1=(x2-x1)/anint

del2=(y2-y1)/anint

call bivpdf(gamma,a1,a2,b11,b12,b22,alpha1,alpha2,beta1,beta2,nsimp,f0,f1,f2,f3,f4,f5,f6)

!!!!!!!!!!!!!!!!!!!!!!!!!!!!!!!!!!!!!!!!!!!!!!!!!!!!!!!!!!!!!!!!!!!!!!!!!!!!!!!!!!

ainteg0=0

! initialises integral for integration constant

do 205 j=1,2

ainteg(j)=0

205 continue

! initialise integrals of distributions for In and St

do 200 j=0,2

! write(*,*) 'Index for integfix2= ',j

! j=0 for integration constant; j=1 for In, j=2 for St

! Section 1: x and y close to zero

! write(*,*) 'Section 1'

if(j.eq.0) then

ainteg0=al1*al2*ep3*ep4

go to 100

end if

sum1=0

! Section 2: x close to zero and y close to 1

! write(*,*) 'Section 2'

! write(*,*) 'Index for integfix2= ',j

100 if(j.eq.0) then

ainteg0=ainteg0+al1*bl2*ep3*ep6*ep8

go to 110

end if

if(j.eq.1) then

sum1=0

else

sum1=al1*bl2*ep3*ep6*ep8

sum1=sum1-(n+beta2+1)*e2*al1*ep3*ep6*ep8/(beta2+1)

if(sum1.le.0.00001) then

sum1=0

end if

end if

! write(*,*) 'sum1= ',sum1

ainteg(j)=ainteg(j)+sum1

! Section 3: x close to 1 and y close to 0

! write(*,*) 'Section 3'

! write(*,*) 'Index for integfix2= ',j

110 if(j.eq.0) then

ainteg0=ainteg0+bl1*al2*ep4*ep5*ep9

go to 120

end if

if(j.eq.1) then

sum1=bl1*al2*ep4*ep5*ep9

sum1=sum1-(n+beta1+1)*e1*al2*ep4*ep5*ep9/(beta1+1)

if(sum1.le.0.00001) then

sum1=0

end if

else

sum1=0

end if

! write(*,*) 'sum1= ',sum1

ainteg(j)=ainteg(j)+sum1

! Section 4: x close to 1 and y close to 1

! write(*,*) 'Section 4'

! write(*,*) 'Index for integfix2= ',j

120 if(j.eq.0) then

ainteg0=ainteg0+bl1*bl2*ep5*ep6*ep7

go to 135

end if

if(j.eq.1) then

sum1=bl1*bl2*ep5*ep6*ep7

sum1=sum1-(n+beta1+1)*e1*bl2*ep5*ep6*ep7/(beta1+1)

ainteg(j)=ainteg(j)+sum1

else

sum1=bl1*bl2*ep5*ep6*ep7

sum1=sum1-(n+beta2+1)*e2*bl1*ep5*ep6*ep7/(beta2+1)

ainteg(j)=ainteg(j)+sum1

end if

! write(*,*) 'sum1= ',sum1

! Section 5: x close to 0 and y in interior

! write(*,*) 'Section 5'

135 if(j.eq.0) then

ainteg0=ainteg0+asum(nsimp,f3,y1,y2)

go to 137

end if

130 do 10 i=1,nint

y=y1+(i-1)*del2

if(j.eq.1) then

g2(i,j)=0.0

! integral for x over boundary x marginal distribution of y

else

g2(i,j)=f3(i)*(y**n)

end if

! integral for y over boundary x marginal distribution of y

10 continue

do 19 i=1,nint

g1(i)=g2(i,j)

19 continue

if(j.eq.2) then

ainteg(j)=ainteg(j)+asum(nsimp,g1,y1,y2)

! write(*,*) 'Index for integfix2= ',j

! write(*,*) 'ainteg=',ainteg(j)

end if

! write(*,*) ''

! Section 6: x close to 1 and y in interior

! write(*,*) 'Section 6'

137 if(j.eq.0) then

ainteg0=ainteg0+asum(nsimp,f4,y1,y2)

go to 155

end if

140 do 20 i=1,nint

y=y1+(i-1)*del2

if(j.eq.1) then

g2(i,j)=f4(i)-(n+beta1+1)*e1*f4(i)*beta1/(beta1+1)

! integral for x over boundary x marginal distribution of y

else

g2(i,j)=f4(i)*(y**n)

! integral for y over boundary x marginal distribution of y

end if

20 continue

do 29 i=1,nint

g1(i)=g2(i,j)

29 continue

ainteg(j)=ainteg(j)+asum(nsimp,g1,y1,y2)

! uses marginal distribution for y with x close to 1

! write(*,*) 'Index for integfix2= ',j

! write(*,*) 'ainteg=',ainteg(j)

! write(*,*) ''

! Section 7: y close to 0 and x in interior

! write(*,*) 'Section 7'

155 if(j.eq.0) then

ainteg0=ainteg0+asum(nsimp,f1,y1,y2)

go to 165

end if

18 do 30 i=1,nint

x=x1+(i-1)*del1

if(j.eq.1) then

g2(i,j)=f1(i)*(x**n)

else

g2(i,j)=0

end if

30 continue

do 39 i=1,nint

g1(i)=g2(i,j)

! write(*,*) 'i',i,'g1= ',g1(i)

39 continue

if(j.eq.1) then

ainteg(j)=ainteg(j)+asum(nsimp,g1,x1,x2)

! write(*,*) 'Index for integfix1= ',j

! write(*,*) 'ainteg=',ainteg(j)

! write(*,*) ''

end if

! uses marginal distribution for x with y close to 0

! Section 8: y close to 1 and x in interior

! write(*,*) 'Section 8'

165 if(j.eq.0) then

ainteg0=ainteg0+asum(nsimp,f2,y1,y2)

go to 175

end if

145 do 40 i=1,nint

x=x1+(i-1)*del1

! write(*,*) 'j=',j

if(j.eq.1) then

g2(i,j)=f2(i)*(x**n)

! write(*,*) 'g2(i,j=',g2(i,j)

else

g2(i,j)=f2(i)-(n+beta2+1)*e2*beta2*f2(i)/(beta2+1)

end if

40 continue

do 49 i=1,nint

g1(i)=g2(i,j)

49 continue

ainteg(j)=ainteg(j)+asum(nsimp,g1,x1,x2)

! uses marginal distribution for x with y close to 1

! write(*,*) 'Index for integfix1= ',j

! write(*,*) 'ainteg=',ainteg(j)

! write(*,*) ''

! Section 9: x and y in interior

! write(*,*) 'Section 9'

175 if(j.eq.0) then

ainteg0=ainteg0+asum(nsimp,f5,y1,y2)

go to 200

end if

if(j.eq.1) then

do 50 i=1,nint

x=x1+(i-1)*del1

g2(i,j)=f5(i)*(x**n)

! moment of q1

50 continue

end if

if(j.eq.2) then

do 53 i2=1,nint

y=y1+(i2-1)*del2

g2(i2,j)=f6(i2)*(y**n)

! moment of q2

53 continue

end if

do 59 i=1,nint

g1(i)=g2(i,j)

59 continue

ainteg(j)=ainteg(j)+asum(nsimp,g1,x1,x2)

! write(*,*) 'Index for integfix2= ',j

! write(*,*) 'ainteg=',ainteg(j)

! write(*,*) ''

200 continue

! write(*,*) 'Integration constant 2= ',ainteg0

end subroutine integfix2

subroutine integ3(gamma,a1,b11,alpha1,beta1,nsimp,ainteg10i,ainteg11i,ainteg12i,aint10,aint11,aint12,aint2)

real :: x,y,gamma,a1,b11,alpha1,beta1,ainteg10,ainteg11,ainteg12,aint2,ainteg10i,ainteg11i,ainteg12i

real :: aint30(1000),aint31(1000),aint32(1000),e1,ep1,ep2,ep3,x1,x2,al1,al2,al3

integer :: nsimp,i

! calculates integrals of powers for the unvariate p.d.f.

if(gamma.le.10) then

e1=0.005

else

e1=0.005/gamma

end if

! boundary corresponding to near neutrality

ep1=e1**alpha1

ep2=e1**beta1

ep3=exp(0.0-2*gamma*(a1+b11))

x1=e1

x2=1-e1

aint10=ep1/alpha1

aint11=(e1*ep1)/(alpha1+1)

aint12=((e1**2)*ep1)/(alpha1+2)

! integrals of powers of x over lower boundary

aint2=(e1**beta1)*ep3/beta1

! integrals of powers of x over upper boundary

al1=alpha1-1

al2=beta1-1

nint=nsimp+1

anint=nsimp

del1=(x2-x1)/anint

do 10 i=1,nint

x=x1+(i-1)*del1

y=1-x

al3=exp(0.0-2*gamma*x*(a1+b11*x))

aint30(i)=(x**al1)*(y**al2)*al3

aint31(i)=x*aint30(i)

aint32(i)=x*aint31(i)

10 continue

ainteg10i=asum(nsimp,aint30,x1,x2)

ainteg11i=asum(nsimp,aint31,x1,x2)

ainteg12i=asum(nsimp,aint32,x1,x2)

! integrals of powers of x over interior range of x

end subroutine integ3

subroutine integ4(gamma,a1,b11,alpha1,beta1,nsimp,ainteg,ainteg0,n)

real :: x,y,gamma,a1,b11,alpha1,beta1,ainteg,ainteg01

real :: aint0(1000),aint1(1000),e1,ep1,ep2,ep3,x1,x2,al1,al2,al3

integer :: n,nsimp,i

! calculates fixation frequency of A1

aln=2*n

if(gamma.le.aln) then

e1=0.005/aln

else

e1=0.005/gamma

e2=0.005/gamma

end if

! boundaries corresponding to near neutrality

ep1=e1**alpha1

ep2=e1**beta1

ep3=exp(0.0-2*gamma*(a1+b11))

x1=e1

x2=1-e1

al1=alpha1-1

al2=beta1-1

aint10=ep1*(1.0/alpha1)

! contribution to integration constant from lower boundary

aint20=(e1**beta1)*ep3/beta1

! contribution to integration constant from upper boundary

aint01=ep1*((1.0/alpha1)-(n*e1/(alpha1+1))+0.5*n*(n-1)*(e1**2)/(alpha1+2))

! contribution to integral of p**n from lower boundary

! write(*,*) 'aint01=',aint01,'aint20=',aint20

nint=nsimp+1

anint=nsimp

del1=(x2-x1)/anint

do 10 i=1,nint

x=x1+(i-1)*del1

y=1-x

al3=exp(0.0-2*gamma*x*(a1+b11*x))

aint1(i)=(y**n)*al3*(x**al1)*(y**al2)

! write(*,*) 'i=',i,'x=',x

aint0(i)=al3*(x**al1)*(y**al2)

! write(*,*) 'aint0=',aint0(i),'aint1=',aint1(i)

if(aint1(i).le.0.000001) then

aint1(i)=0

end if

! write(*,*) 'i=',i,aint1(i)

! contribution to integral of p**n from interior range

10 continue

ainteg=asum(nsimp,aint1,x1,x2)

ainteg0=asum(nsimp,aint0,x1,x2)

ainteg=ainteg+aint01

! net integral of p**n

ainteg0=ainteg0+aint10+aint20

! write(*,*) 'ainteg0=',ainteg0,'ainteg=',ainteg

! net integral for integration constant

end subroutine integ4

subroutine integ5(gamma,a,b,alpha,beta,nsimp,ainteg,ainteg0,n)

real :: x,y,gamma,a,b,alpha,beta,ainteg,aint10,ainteg01

real :: aint0(1000),aint1(1000),e1,ep1,ep2,ep3,x1,x2,al1,al2,al3

integer :: n,nsimp,i

! calculates fixation frequency of A2

aln=2*n

if(gamma.le.aln) then

e1=0.005/aln

else

e1=0.005/gamma

e2=0.005/gamma

end if

! boundaries corresponding to near neutrality

ep1=e1**alpha

ep2=e1**beta

ep3=exp(0.0-2*gamma*(a+b))

x1=e1

x2=1-e1

al1=alpha-1

al2=beta-1

aint10=ep1*(1.0/alpha)

! contribution to integration constant from lower boundary

aint20=ep1*ep3*(1.0/beta)

! contribution to integration constant from upper boundary

aint01=ep1*ep3*((1.0/beta)-(n*e1/(beta+1))+0.5*n*(n-1)*(e1**2)/(beta+2))

! contribution to integral of q**n from upper boundary

nint=nsimp+1

anint=nsimp

del1=(x2-x1)/anint

do 10 i=1,nint

x=x1+(i-1)*del1

y=1-x

al3=exp(0.0-2*gamma*x*(a+b*x))

aint1(i)=(x**n)*al3*(x**al1)*(y**al2)

aint0(i)=al3*(x**al1)*(y**al2)

if(aint1(i).le.0.000001) then

aint1(i)=0

end if

! contribution to integral of q**n from interior range

10 continue

ainteg=asum(nsimp,aint1,x1,x2)

ainteg0=asum(nsimp,aint0,x1,x2)

ainteg=ainteg+aint01

! net integral of q**n

ainteg0=ainteg0+aint10+aint20

! net integral for integration constant

end subroutine integ5
