# Supplementary material for: The fitness consequences of genetic divergence between polymorphic gene arrangements
Source: Genetics. 2023 Dec 26;226(3):iyad218. doi: 10.1093/genetics/iyad218 (PMC11090464; doi:10.1093/genetics/iyad218)
Supplement: iyad218_Supplementary_Data [file iyad218_supplementary_data.zip › Supplementary_File_3_GENETICS-2023-306559.docx]

**Supplementary File S3 Computer code for subdivided population**

**This program is written in GFortran for the MacIntosh (https://gnuc.org.wiki/GFortranBinariesMacOS)**

program invload4

! PROGRAM FOR LOAD WITH INVERSION AND STANDARD ARRANGEMENTS

! Uses gamma distribution of selection coefficients

! Takes coupling between selection on In and St into account

! Population subdivision included; Fst corrected for selection

integer :: nsimp,nint,i,j,k,CONT,in1,ndat,nset

real :: lopop,metpop,ak,h,u,v,qeq,p,q,x,y,Fst,AM1,AM2,FST1,FST2,sbar

real :: AM,ANE,ANET,eload,eload1,elrel1,eload2,elrel2,alpha2j,beta2j,fnmpd

real :: alpha1,beta1,alpha2,beta2,h1,ga,ga1,ga2,ga3,ga4,aj,s,fn,qmean2,alpha1j,beta1j

real :: const1,qbar1,pbar1,q2bar1,alnqbar1,alnq2bar1,p2bar1,ebrel1,alpha1mp,alpha2mp

real :: const2,qbar2,pbar2,q2bar2,alnqbar2,alnq2bar2,p2bar2,ebrel2,q2var,beta1mp,beta2mp

real :: elhrel1,elhrel2,elb,elbrel,t1rel,t2rel,ANE1,ANE2,gamc1,alpha2n,beta2n

real :: alpha,beta,alpha1d,alpha2d,beta1d,beta2d,del,qbarr(1000),pbarr(1000),fg(1000)

real :: x1,x2,fx,const,alphbet,qbarc,qbarc2,alnbetaf,h2,beta1da,beta2da,qbar11,qbar22

real :: a,z,Pz,anint,gammq,gambar,gambar1,gambar2,ashape,Uinv,r0,r1,r2,F1,F2,betfn1,betfn2,asum

real:: alw1,alw2,alb,alhom1,alhom2,B1,B2,qbarneut,pbarneut,qbarneut1(3),pbarneut1(3),alpha2j1,beta2j1

real :: albn,alhomn1,alhomn2,gamser,xsc,gln,alhon1,alhnon2,alpha1m(3),alpha2m(3),cov(3,3)

real :: qbarneut2(3),pbarneut2(3),F1n(3),F2n(3),x1a,x2a,upgam,beta1m(3),beta2m(3),cpjn(3,3)

real :: P1(3),P2(3),pb1(3),pb2(3),qb1(3),qb2(3),qbarneut1a,qbarneut2ar,F1jn(3,3),F2jn(3,3),F2d(3),q2d(3)

real :: alw1a(1000),alw2a(1000),albna(1000),alhomn1a(1000),alhomn2a(1000),B1wa(1000),B2wa(1000),cp12

real :: ainteg(2,2),ainteg0,alpha1i,beta1i,alpha2i,beta2i,f(1000,1000),gamint,qbjn1(3,3),qbjn2(3,3),q1b,q2b

real :: a1,a2,b11,b12,b22,q1bar,q2bar,q1var,tt1,tt2,B1w,B2w,qb1a(1000),qb2a(1000),pi1b(1000),pi2b(1000)

real :: ainteg10,ainteg11,ainteg12,aint10,aint11,aint12,aint2,alw1s,alw2s,alhom1s,alhom2s,B1ws,B2ws

real :: ainteg20,ainteg21,ainteg22,aint20,aint21,aint22,a1d,a2d,b11d,b22d,b12d,gam1,gam2

CHARACTER*20 FIN

CHARACTER*20 FOUT

write (*,*) 'Input file?'

read(*,*) FIN

OPEN (2,file=FIN)

WRITE (*,*) 'Output file?'

READ (*,*) FOUT

OPEN (1,FILE=FOUT)

write (1,*) 'Genetic loads with inversion and standard arrangements'

write (1,*) 'A subdivided population with no exchange between In and St'

write (1,*) 'Coupling between selection on In and St is allowed for'

write (1,*) 'A range of Fst values is allowed; a correction for selection within demes is used'

write (1,*) ''

READ (2,*) ninvs

READ (2,*) nsimp

READ (2,*) ANE

READ (2,*) ANET

READ (2,*) ak

READ (2,*) h

READ (2,*) u

READ (2,*) x

READ (2,*) sbar,ashape

READ (2,*) gac1

READ (2,*) gamc1

READ (2,*) upgfac

READ (2,*) ndat

alpha=4*ANET*u

beta=alpha/ak

! scaled mutation rates

aninvs=ninvs

y=1-x

gambar=2*ANET*sbar

upfac=y*gambar/ashape

anint=nsimp

write (1,*) 'Number of selected sites in inversion= ',ninvs

write (1,*) 'Inversion frequency= ',x

write (1,*) ''

write (1,*) 'Mutation rate towards deleterious variants= ',u

write (1,*) 'Mutational bias towards deleterious variants= ',ak

write (1,*) 'Local population size= ',ANE

write (1,*) 'Metapopulation size= ',ANET

write (1,*) 'No. of gamma values for Simpsons rule = ',nsimp

write (1,*) ''

write (1,*) 'Dominance coefficient= ',h

write (1,*) 'Mean selection coefficient= ',sbar

write (1,*) 'Mean scaled selection coefficient for whole popn= ',gambar

write (1,*) 'Shape parameter= ',ashape

write (1,*) 'Upper bound gamma value for neutrality in the St metapopn= ',gamc1

write (1,*) 'Upper limit to gamma distribution of z= x/scale parameter = ',upgfac

write (1,*) 'Threshold gamma value factor for use of approximate p.d.f.= ',gac1

write (1,*) ''

write (1,*) 'Number of Fst values= ',ndat

write (1,*) ''

write (*,*) 'Number of selected sites in inversion= ',ninvs

write (*,*) 'Inversion frequency= ',x

write (*,*) ''

write (*,*) 'Mutation rate towards deleterious variants= ',u

write (*,*) 'Mutational bias towards deleterious variants = ',ak

write (*,*) 'Local population size= ',ANE

write (*,*) 'Metapopulation size= ',ANET

write (*,*) 'No. of gamma values for Simpsons rule = ',nsimp

write (*,*) ''

write (*,*) 'Dominance coefficient= ',h

write (*,*) 'Mean selection coefficient= ',sbar

write (*,*) 'Mean scaled selection coefficient for whole popn= ',gambar

write (*,*) 'Shape parameter= ',ashape

write (*,*) 'Upper bound gamma value for neutrality in the St metapopn= ',gamc1

write (*,*) 'Upper limit to gamma distribution of z= x/scale parameter = ',upgfac

write (*,*) 'Threshold gamma value factor for use of approximate p.d.f.= ',gac1

write (*,*) ''

write (*,*) 'Number of Fst values= ',ndat

h1=h

ANE1=x*ANET

ANE2=y*ANET

! metapopn sizes for In and St

r0=ANE/ANET

r1=ANE*x/ANET

r2=ANE*y/ANET

! 1 denotes In and 2 denotes St; r's are ratios of their deme sizes to total metapopn size

write (1,*) 'Ratios of deme sizes to total metapopulation size '

write (1,*) 'Total: r0= ',r0,' Inv: r1= ',r1,'St: r2= ',r2

write (1,*) ''

alpha1=alpha*x

alpha2=alpha*y

beta1=beta*x

beta2=beta*y

gambar1=gambar*x

gambar2=gambar*y

! numbered parameters are values for In (1) and St (2), respectively

alpha1d=alpha1*r0

alpha2d=alpha2*r0

beta1d=beta1*r0

beta2d=beta2*r0

! local scaled values for In and St demes respectively

nset=0

420 nset=nset+1

if(nset.gt.ndat) go to 400

read(2,*) Fst

AM=(1-Fst)/Fst

write (1,*) ''

write (1,*) 'Neutral Fst for whole population= ',Fst

write (1,*) 'Scaled migration rate for whole population= ',AM

write (1,*) ''

write (*,*) ''

write (*,*) 'Neutral Fst for whole population= ',Fst

write (*,*) 'Scaled migration rate for whole population= ',AM

write (*,*) ''

alw1s=0

alw2s=0

albs=0

alhom1s=0

alhom2s=0

B1ws=0

B2ws=0

qt1=0

qt2=0

pi1t=0

pi2t=0

! initial values of mean load statistics

!!!!!!!!!!!!!!!!!!!!!!!!!!!!!!!!!!!!!!!!!!!!!!!!!!!!!!!!!!!!!!!!!!!!!!!

! Zone 1: quasi-neutral approximation (both In and St metapopns behave as neutral)

! No need for selective coupling to be modelled

sc1=gamc1/(2*ANE2)

! corresponding upper bound selection coefficient for calculating the load statistics

z=exp(gammln(ashape))

! calculates gamma function with parameter ashape

xsc=ashape*gamc1/gambar2

call gser(gamser,ashape,xsc,gln)

Pz1=gamser

! probability of falling within the neutral zone for St metapopulation

Sselneut1=ashape*sc1*aninvs*Pz1/(ashape+1.0)

! integral of selection coefficients for St over zone 1

write(*,*) ''

write (1,*) 'Zone 1: quasi-neutral zone 1'

write (1,*) 'Upper bound scaled selection coefficient for neutrality in St metapopulation= ',gamc1

write (1,*) 'Probability of zone 1= ',Pz1

write (1,*) 'Integral of selection coefficient over zone 1= ',Sselneut1

write (1,*) ''

write (*,*) 'Zone 1: quasi-neutral zone 1'

write (*,*) 'Upper bound scaled selection coefficient for neutrality in St metapopulation= ',gamc1

write (*,*) 'Probability of zone 1= ',Pz1

write (*,*) 'Integral of selection coefficient for St over neutral zone= ',Sselneut1

write (*,*) ''

qbarneut=ak/(1+ak)

pbarneut=1-qbarneut

! mean allele frequencies under neutrality

y=1-x

AM1=AM*x

AM2=AM*y

FST1=1.0/(1+AM1)

FST2=1.0/(1+AM2)

! scaled migration rates and FSTs for In and St demes

alpha1mp=alpha1/(1-FST1)

beta1mp=beta1/(1-FST1)

alpha2mp=alpha2/(1-FST2)

beta2mp=beta2/(1-FST2)

F1=1.0/(1+alpha1mp+beta1mp)

F2=1.0/(1+alpha2mp+beta2mp)

! Scaled parameters and F's for In and St metapopulations assuming neutrality

F1=FST1+(1-FST1)*F1

F2=FST2+(1-FST2)*F2

! net F's for In and St

pi1=2*pbarneut*qbarneut*(1-F1)

pi2=2*pbarneut*qbarneut*(1-F2)

! within-deme diversities for In and St

qt1=qbarneut*Pz1

qt2=qbarneut*Pz1

pi1t=pi1*Pz1

pi2t=pi2*Pz1

! contributions to mean allele frequencies and diversities for In and St

call loadstats1(qbarneut,qbarneut,h1,F1,F2,alw1,alw2,alb,alhom1,alhom2,B1,B2)

! this subroutine ignores covariance between q1 and q2

alw1=alw1*Sselneut1

alw2=alw2*Sselneut1

! total loads within In and St for neutral zone

alb=alb*Sselneut1

! total between arrangement load for neutral zone

alhom1=alhom1*Sselneut1

alhom2=alhom2*Sselneut1

! homozygous loads for neutral zone for In and St

Bw1=B1*Sselneut1

Bw2=B2*Sselneut1

! inbreeding loads for neutral zone for In and St

alw1s=alw1s+alw1

alw2s=alw2s+alw2

albs=albs+alb

alhom1s=alhom1s+alhom1

alhom2s=alhom2s+alhom2

B1ws=B1ws+Bw1

B2ws=B2ws+Bw2

! compiles integrals of load statistics

tt1=1.0-exp(alb-alw1)

tt2=1.0-exp(alb-alw2)

write (1,*) ''

write (1,*) 'Mean load statistics for zone 1'

write (1,*) 'Mean q1 and q2= ',qbarneut

write (1,*) 'F1 and F2= ',F1,F2

write (1,*) 'Diversities= ',pi1,pi2

write (1,*) ''

write (1,*) ''

write (1,*) 'Contributions to loads within In and St=',alw1,alw2

write (1,*) 'Contributions to load between In and St =',alb

write (1,*) 'Contributions to homozygous loads for In and St=',alhom1,alhom2

write (1,*) 'Contributions to inbreeding loads for In and St= ',Bw1,Bw2

write (1,*) ''

write (1,*) 'Contributions to selection coefficients for In and St homokaryotypes'

write (1,*) tt1,tt2

write (1,*) ''

write (1,*) 'Contributions to mean A2 freqs=',qt1,qt2

write (1,*) 'Contributions to mean diversities=',pi1t,pi2t

write (*,*) ''

write (*,*) 'Mean load statistics for zone 1'

write (*,*) 'Mean q1 and q2= ',qbarneut

write (*,*) 'F1 and F2= ',F1,F2

write (*,*) 'Diversities= ',pi1,pi2

write (*,*) ''

write (*,*) 'Contributions to loads within In and St=',alw1,alw2

write (*,*) 'Contributions to load between In and St=',alb

write (*,*) 'Contributions to homozygous loads for In and St=',alhom1,alhom2

write (*,*) 'Contributions to inbreeding loads= ',Bw1,Bw2

write (*,*) 'Contributions to selection coefficients against In and St homokaryotypes'

write (*,*) tt1,tt2

write (*,*) ''

pi1neut=pi1

pi2neut=pi2

! saves these for final comparison of selected and neutral site diversities

!!!!!!!!!!!!!!!!!!!!!!!!!!!!!!!!!!!!!!!!!!!!!!!!!!!!!!!!!!!!!!!!!!!!!!!

! 2. Zone 2: quasi-neutral approximation 2 for load statistics

! Lower bound for St metapopn gamma is gamc1 from previous section

! Upper bound for St metapopn gamma is gamc1*(Ne2/NeT) (ensures neutrality within St demes)

! A local beta distribution is assumed for In and St populations

! Coupling in metapopulation is allowed, but is ignored within demes

! Integration over the distribution of metapopn gamma values is used

gamc2=gamc1/r0

nint=nsimp+1

anint=nint

! number of values of functions to be calculated

write(1,*) ''

write (1,*) 'Zone 2: quasi-neutral zone 2'

write (1,*) 'Lower and upper bounds of St metapopn gamma for zone 2'

write (1,*) gamc1,gamc2

write(*,*) 'Zone 2: quasi-neutral zone 2'

write(*,*) ''

write (*,*) 'Lower and upper bounds of St metapopn gamma for zone 2'

write (*,*) gamc1,gamc2

xsc=ashape*gamc2/gambar2

call gser(gamser,ashape,xsc,gln)

Pz2=gamser-Pz1

Ptot=gamser

write (1,*) 'Probability of zone 2= ',Pz2

write (1,*) ''

write (*,*) 'Probability of zone 2= ',Pz2

write(*,*) ''

x1a=gamc1*ashape/gambar2

x2a=gamc2*ashape/gambar2

del=(gamc2-gamc1)/anint

! lower and upper boundaries for rescaled gamma's (gambar x 1-x)

G1=1.0/AM1

G2=1.0/AM2

G3=1-2*G1

G4=1-2*G2

! modifiers of coefficients of bivariate distribution of q1 and q2 for metapopulation

a1=x*(x*(G1+G3*h1)+y*h1)

a2=y*(y*(G2+G4*h1)+x*h1)

b11=0.5*(1-2*h1)*G3*(x**2)

b12=(1-2*h1)*x*y

b22=0.5*(1-2*h1)*G4*(y**2)

! coefficients for bivariate distribution of q1 and q2 in metapopulation

write(1,*) 'Coefficients for bivariate distribution of q1 and q2 in metapopulation'

write(1,*) 'G1=',G1,' G2=',G2

write(1,*) 'G3=',G3,' G4=',G4

write(1,*) 'a1=',a1,' a2=',a2

write(1,*) 'b11=',b11,' b12=',b12,' b22=',b22

write(1,*) ''

write(*,*) 'Coefficients for bivariate distribution of q1 and q2 in metapopulation'

write(*,*) 'G1=',G1,' G2=',G2

write(*,*) 'G3=',G3,' G4=',G4

write(*,*) 'a1=',a1,' a2=',a2

write(*,*) 'b11=',b11,' b12=',b12,' b22=',b22

q1bar=qbarneut

do 80 i1=1,nint

ga1=gamc1+(i1-1)*del

! write(1,*) 'Index of gamma value ',i1,' gamma for St metapopulation= ',ga1

! write(*,*) ''

! write(*,*) 'Index of gamma value=',i1,' gamma for St metapopulation= ',ga1

xi=ashape*ga1/gambar2

! parameter for gamma distribution of scaled selection coefficients for St metapopn

Pxi=(xi**(ashape-1))*exp(-xi)

Pxi=Pxi/z

! probability density of xi

sc2=ga1/(2*ANE2)

sc=aninvs*sc2

! write(*,*) 'sc= ',sc

! write(*,*) ''

! product of selection coefficient and number of loci for load calculations

gamint=ga1/y

! gamma for whole metapopulation

ga2=gamint*x

! write(*,*) 'Statistics obtained from the bivariate distribution of q1 and q2 in metapopulation'

! write(1,*) 'Statistics obtained from the bivariate distribution of q1 and q2 in metapopulations'

! write(1,*) ''

gac=gac1/(x*h1)

! equivalent to gac=40*gac1 with h=0.05 and x=0.5

if(ga1.le.gac) then

! uses approximate univariate distributions for weak selection

b111=0.5*(1-2*h1)*G3*x

b221=0.5*(1-2*h1)*G4*y

! coefficients for 2nd order terms in q1 and q2 in distribution function

do 110 it=1,5

a22=a2+x*(1-2*h1)*q1bar

! approximation for coefficient of 1st order term in q2 in distribution function

call integ3(ga1,a22,b221,alpha2mp,beta2mp,nsimp,ainteg10i,ainteg11i,ainteg12i,aint10,aint11,aint12,aint2)

const2=ainteg10i+aint10+aint2

! integration constant for q2

q2bar=(ainteg11i+aint11+aint2)/const2

! write(*,*) 'Mean of q2= ',q2bar

q2var=(ainteg12i+aint12+aint2)/const2

q2var=q2var-q2bar**2

! write(*,*) 'Variance of q2= ',q2var

a11=a1+y*(1-2*h1)*q2bar

! approximation for coefficient of 1st order term in q1 in distribution function

call integ3(ga2,a11,b111,alpha1mp,beta1mp,nsimp,ainteg10i,ainteg11i,ainteg12i,aint10,aint11,aint12,aint2)

const1=ainteg10i+aint10+aint2

! integration constant for q1

q1bar=(ainteg11i+aint11+aint2)/const1

! write(*,*) 'Mean of q1= ',q1bar

q1var=(ainteg12i+aint12+aint2)/const1

q1var=q1var-q1bar**2

! write(*,*) 'Variance of q1= ',q1var

cp12=0

r12=0

110 continue

! Iterates means and variances of q1 and q2

go to 115

end if

call integ1(gamint,a1,a2,b11,b12,b22,alpha1mp,alpha2mp,beta1mp,beta2mp,nsimp,ainteg,ainteg0,cp12)

! write(*,*) 'Integration constant for p.d.f= ',ainteg0

q1bar=ainteg(1,1)/ainteg0

q2bar=ainteg(2,1)/ainteg0

q1var=ainteg(1,2)/ainteg0

q1var=q1var-q1bar**2

q2var=ainteg(2,2)/ainteg0

q2var=q2var-q2bar**2

cp12=cp12/ainteg0

cov12=cp12-q1bar*q2bar

r12=cov12/sqrt(q1var*q2var)

115 F1=q1var/(q1bar*(1-q1bar))

F2=q2var/(q2bar*(1-q2bar))

! write(*,*)

! write(*,*) 'Metapopulation means of q1 and q2= ',q1bar,q2bar

! write(*,*) 'Metapopulation variances of q1 and q2= ',q1var,q2var

! write(*,*) 'Metapopulation covariance and correlation of q1 and q2= ',cov12,r12

F1=FST1+(1-FST1)*F1

F2=FST2+(1-FST2)*F2

! write(*,*) 'F1 and F2= ',F1,F2

! net F's for In and St

pi1=2*q1bar*(1.0-q1bar)*(1-F1)

pi2=2*q2bar*(1.0-q2bar)*(1-F2)

! write(*,*) 'pi1 and pi2= ',pi1,pi2

! diversities

call loadstats1(q1bar,q2bar,h1,F1,F2,alw1,alw2,alb,alhom1,alhom2,B1,B2)

! this subroutine ignores covariance between q1 and q2

alw1=alw1*sc

alw2=alw2*sc

alb=alb*sc

alhom1=alhom1*sc

alhom2=alhom2*sc

B1w=B1*sc

B2w=B2*sc

! write (*,*) 'Load statistics'

! write (*,*) 'Loads within In and St=',alw1,alw2

! write (*,*) 'Load between In and St=',alb

! write (*,*) 'Homozygous load for In and St=',alhom1,alhom2

! write (*,*) 'Inbreeding loads= ',B1w,B2w

! write (*,*) ''

alw1a(i1)=alw1*Pxi

alw2a(i1)=alw2*Pxi

albna(i1)=alb*Pxi

alhomn1a(i1)=alhom1*Pxi

alhomn2a(i1)=alhom2*Pxi

B1wa(i1)=B1w*Pxi

B2wa(i1)=B2w*Pxi

qb1a(i1)=q1bar*Pxi

qb2a(i1)=q2bar*Pxi

pi1b(i1)=pi1*Pxi

pi2b(i1)=pi2*Pxi

! Stores p.d.f. weighted load statistics for current value of gamma

80 continue

alw1=asum(nsimp,alw1a,x1a,x2a)

alw2=asum(nsimp,alw2a,x1a,x2a)

alb=asum(nsimp,albna,x1a,x2a)

alhon1=asum(nsimp,alhomn1a,x1a,x2a)

alhon2=asum(nsimp,alhomn2a,x1a,x2a)

B1w=asum(nsimp,B1wa,x1a,x2a)

B2w=asum(nsimp,B2wa,x1a,x2a)

qbw1=asum(nsimp,qb1a,x1a,x2a)

qbw2=asum(nsimp,qb2a,x1a,x2a)

pibw1=asum(nsimp,pi1b,x1a,x2a)

pibw2=asum(nsimp,pi2b,x1a,x2a)

tt1=1.0-exp(alb-alw1)

tt2=1.0-exp(alb-alw2)

alw1s=alw1s+alw1

alw2s=alw2s+alw2

albs=albs+alb

alhom1s=alhom1s+alhon1

alhom2s=alhom2s+alhon2

B1ws=B1ws+B1w

B2ws=B2ws+B2w

qt1=qt1+qbw1

qt2=qt2+qbw2

pi1t=pi1t+pibw1

pi2t=pi2t+pibw2

! compiles integrals of load statistics

write (1,*) ''

write (1,*) 'Contributions to mean load statistics for zone 2'

write (1,*) 'Loads within In and St=',alw1,alw2

write (1,*) 'Load between In and St=',alb

write (1,*) 'Homozygous load for In and St=',alhon1,alhon2

write (1,*) 'Inbreeding loads= ',B1w,B2w

write (1,*) 'Selection coefficients against In and St homokaryotypes'

write (1,*) tt1,tt2

write (1,*) ''

write (1,*) 'Contributions to mean A2 freqs=',qbw1,qbw2

write (1,*) 'Contributions to mean diversities=',pibw1,pibw2

write (1,*) ''

write (*,*) ''

write (*,*) 'Contributions to mean load statistics for zone 2'

write (*,*) 'Loads within In and St=',alw1,alw2

write (*,*) 'Load between In and St=',alb

write (*,*) 'Homozygous load for In and St=',alhon1,alhon2

write (*,*) 'Inbreeding loads= ',B1w,B2w

write (*,*) 'Selection coefficients against In and St homokaryotypes'

write (*,*) tt1,tt2

write (*,*) ''

write (*,*) 'Contributions to mean A2 freqs=',qbw1,qbw2

write (*,*) 'Contributions to mean diversities=',pibw1,pibw2

write (*,*) ''

!!!!!!!!!!!!!!!!!!!!!!!!!!!!!!!!!!!!!!!!!!!!!!!!!!!!!!!!!!!!!!!!!!!!!!

! 3. Zone 3: moderate selection approximation for load statistics

! Coupling is allowed for the distribution of q1 and q2 for demes, conditioned on mean q1 and q2 for immigrants

! It is ignored for the metapopulation,but the general univariate distributions of mean q1 and q2 are used

! Integration over distribution of metapop gamma values in this zone

gamc3=gamc1/(x*r0)

write(*,*) ''

write (1,*) 'Zone 3: moderate selection zone'

write (1,*) 'Lower and upper bounds of St metapopn gamma for zone 3'

write (1,*) gamc2,gamc3

write (1,*) ''

write (*,*) 'Zone 3: 1st quasi-deterministic zone'

write (*,*) 'Lower and upper bounds of St metapopn gamma for zone 3'

write (*,*) gamc2,gamc3

write(*,*) ''

xsc=ashape*gamc3/gambar2

call gser(gamser,ashape,xsc,gln)

Pz3=gamser-Ptot

Ptot=gamser

write (1,*) 'Probability of zone 3= ',Pz3

write(1,*) ''

write (*,*) 'Probability of zone 3= ',Pz3

write(*,*) ''

x1a=gamc2*ashape/gambar2

x2a=gamc3*ashape/gambar2

! upper and lower limits of scaled gamma distribution variable for integrations

del=(gamc3-gamc2)/anint

! lower and upper boundaries for selection coefficients

a1d=x*h1

a2d=y*h1

b11d=0.5*(1-2*h1)*(x**2)

b12d=(1-2*h1)*x*y

b22d=0.5*(1-2*h1)*(y**2)

! coefficients for bivariate distribution of q1 and q2 within demes

b111d=0.5*(1-2*h1)*x

b221d=0.5*(1-2*h1)*y

! coefficients for 2nd order terms in q1 and q2 in approximate distribution functions

do 480 i1=1,nint

ga1=gamc2+(i1-1)*del

! write(*,*) ''

! write(*,*) 'Index of gamma value',i1,' gamma for St metapopn= ',ga1

! write(*,*) ''

! write(1,*) 'Index of gamma value',i1,' gamma for St metapopn= ',ga1

! write(1,*) ''

xi=ashape*ga1/gambar2

! scaled variable for gamma distribution of selection coefficients for St metapopn

Pxi=(xi**(ashape-1))*exp(-xi)

Pxi=Pxi/z

! probability density of xi

sc2=ga1/(2*ANE2)

sc=aninvs*sc2

! product of selection coefficient and number of loci for load calculations

! start of calculations for St metapopn

gamint=ga1/y

! gamma for whole metapopulation

FST1=1.0/(1+AM1+2*gamint*r0*x)

FST2=1.0/(1+AM2+2*gamint*r0*y)

! FSTs corrected for selection within In and St demes

G1=FST1/(1-FST1)

G2=FST2/(1-FST2)

G3=1-2*G1

G4=1-2*G2

! modifiers of coefficients of bivariate distribution of q1 and q2 for metapopulation

a1=x*(x*(G1+G3*h1)+y*h1)

a2=y*(y*(G2+G4*h1)+x*h1)

b11=0.5*(1-2*h1)*G3*(x**2)

b12=(1-2*h1)*x*y

b22=0.5*(1-2*h1)*G4*(y**2)

! coefficients for bivariate distribution of q1 and q2 in metapopulation

b111d=0.5*(1-2*h1)*x

b221d=0.5*(1-2*h1)*y

! coefficients for 2nd order terms in q1 and q2 in within-deme approximate distribution function

alpha1mp=alpha1/(1-FST1)

beta1mp=beta1/(1-FST1)

alpha2mp=alpha2/(1-FST2)

beta2mp=beta2/(1-FST2)

F1=1.0/(1+alpha1mp+beta1mp)

F2=1.0/(1+alpha2mp+beta2mp)

! Scaled parameters and F's for In and St metapopulation

gamintd=gamint*r0

gam1=gamintd*x

gam2=gamintd*y

! gammmas for demes

call integ2(gamint,a2,b22,alpha2mp,beta2mp,nsimp,ainteg20,ainteg21,ainteg22,aint20,aint21,aint22,aint2)

! calculates integrals over distribution of mean q2 for metapopulation

ainteg20t=ainteg20+aint20+aint2

! normalization constant for entire distribution

q2bar=(ainteg21+aint21+aint2)/ainteg20t

q2var=(ainteg22+aint22+aint2)/ainteg20t

q2var=q2var-(q2bar**2)

F2=q2var/(q2bar*(1-q2bar))

! write(*,*) 'Mean q2 for metapopn=',q2bar,' Variance of q2 for metapon=',q2var

! write(*,*) 'F for q2 for metapopn=',F2

P2(1)=aint20/ainteg20t

P2(2)=ainteg20/ainteg20t

P2(3)=1-P2(1)-P2(2)

! probabilities of mean q1 falling into the three regions

qb2(1)=aint21/ainteg20

qb2(2)=ainteg21/ainteg20

pb2(1)=1-qb2(1)

pb2(2)=1-qb2(2)

if(P2(3).gt.0.001) then

qb2(3)=(q2bar-P2(1)*qb2(1)-P2(2)*qb2(2))/P2(3)

pb2(3)=1-qb2(3)

else

P2(3)=0

qb2(3)=1

pb2(3)=0

end if

if(P2(2).le.0.001) then

P2(2)=0

P2(1)=1

qb2(2)=0

pb2(2)=1

! avoids use of these in load calculations

end if

! write(*,*) 'Probabilities of 3 regions for St metapopn= ',P2(1),P2(2),P2(3)

! write(*,*) 'Mean q2 in lower boundary for St metapopn= ',qb2(1)

! write(*,*) 'Mean q2 in intermediate region for St metapopn= ',qb2(2)

! write(*,*) 'Mean q2 in upper boundary for St metapopn= ',qb2(3)

! start of calculations for In metapopn

call integ2(gamint,a1,b11,alpha1mp,beta1mp,nsimp,ainteg10,ainteg11,ainteg12,aint10,aint11,aint12,aint2)

! calculates integrals over distribution of mean q1 for metapopulation

ainteg10t=ainteg10+aint10+aint2

! normalization constant for entire distribution

q1bar=(ainteg11+aint11+aint2)/ainteg10t

q1var=(ainteg12+aint12+aint2)/ainteg10t

q1var=q1var-(q1bar**2)

F1=q1var/(q1bar*(1-q1bar))

! write(*,*) 'Mean q1 for metapopn=',q1bar,' Variance of q1 for metapon=',q1var

! write(*,*) 'F for q1 over metapopn=',F1

P1(1)=aint10/ainteg10t

P1(2)=ainteg10/ainteg10t

P1(3)=1-P1(1)-P1(2)

! probabilities of mean q1 falling into the three regions

qb1(1)=aint11/ainteg10

qb1(2)=ainteg11/ainteg10

pb1(1)=1-qb1(1)

pb1(2)=1-qb1(2)

if(P1(3).gt.0.001) then

qb1(3)=(q1bar-P1(1)*qb1(1)-P1(2)*qb1(2))/P1(3)

pb1(3)=1-qb1(3)

else

P1(3)=0

qb1(3)=1

pb1(3)=0

end if

if(P1(2).le.0.001) then

P1(2)=0

P1(1)=1

qb1(2)=0

pb1(2)=1

! avoids use of these in load calculations

end if

! write(*,*) 'Probabilities of 3 regions for In metapopn= ',P1(1),P1(2),P1(3)

! write(*,*) 'Mean q1 in lower boundary for In metapopn= ',qb1(1)

! write(*,*) 'Mean q1 in intermediate region for In metapopn= ',qb1(2)

! write(*,*) 'Mean q1 in upper boundary for In metapopn= ',qb1(3)

qbars1=0

qbars2=0

cov12b=0

r12b=0

F1bar=0

F2bar=0

pi1bar=0

pi2bar=0

! start of calculations of within-deme effects

gamintd=gamint*r0

gam1=gamintd*x

gam2=gamintd*y

! gammmas for demes

if(gam1.le.gac1) then

! In treated as neutral within demes; St is treated as univariate distribution within demes

F1=FST1+(1-FST1)*F1

! write(*,*) 'F1 and F2= ',F1,F2

! net F's for In and St

pi1=2*q1bar*(1.0-q1bar)*(1-F1)

! write(*,*) 'pi1= ',pi1

! diversity

qbars1=q1bar

pi1bar=pi1

qbars2=0

pi2bar=0

do 150 j=1,3

! write(*,*)

alpha2j1=alpha2d+AM2*qb2(j)

beta2j1=beta2d+AM2*pb2(j)

! write(*,*) 'j',j,'alpha2,beta2',alpha2j1,beta2j1

! write(*,*) 'j',j,' q2',qb2(j)

call integ2(gamintd,a2d,b22d,alpha2j1,beta2j1,nsimp,ainteg20,ainteg21,ainteg22,aint20,aint21,aint22,aint2)

! calculates integrals over distribution of q2 for demes

ainteg20t=ainteg20+aint20+aint2

! normalization constant for entire distribution

q2bar=(ainteg21+aint21+aint2)/ainteg20t

q2var=(ainteg22+aint22+aint2)/ainteg20t

q2var=q2var-(q2bar**2)

F2=q2var/(q2bar*(1-q2bar))

! write(*,*) 'Mean q2=',q2bar,' Variance of q2=',q2var

! write(*,*) 'F for q2=',F2

F2d(j)=F2

q2d(j)=q2bar

F2bar=F2bar+F2*P2(j)

qbars2=qbars2+q2bar*P2(j)

pi2bar=pi2bar+2*q2bar*(1.0-q2bar)*(1-F2)*P2(j)

! means of pop gen statistics over all metapopulation mean q2's

150 continue

alw1=0

alw2=0

alb=0

alhon1=0

alhon2=0

B1w=0

B2w=0

do 155 j1=1,3

F2=F2d(j)

q2bar=q2d(j1)

call loadstats1(q1bar,q2bar,h1,F1,F2,alwn1,alwn2,albn,alhomn1,alhomn2,B1,B2)

! this subroutine ignores covariance between q1 and q2

alw1=alw1+alwn1*P2(j1)

alw2=alw2+alwn2*P2(j1)

alb=alb+albn*P2(j1)

alhon1=alhon1+alhomn1*P2(j1)

alhon2=alhon2+alhomn2*P2(j1)

B1w=B1w+B1*P2(j1)

B2w=B2w+B2*P2(j1)

155 continue

go to 170

end if

do 120 i=1,3

alpha1i=alpha1d+AM1*qb1(i)

beta1i=beta1d+AM1*pb1(i)

! write(*,*) ''

! write(*,*) 'i for q1 boundary= ',i

! write(*,*) 'alpha1,beta1= ',alpha1i,beta1i

! write(*,*) ''

do 125 j=1,2

alpha2j1=alpha2d+AM2*qb2(j)

beta2j1=beta2d+AM2*pb2(j)

! write(*,*) ''

! write(*,*) 'j for q2 boundary= ',j

! write(*,*) 'alpha2,beta2= ',alpha2j1,beta2j1

! write(*,*) ''

if(gam2.le.gac) then

! uses approximate univariate distributions for weak selection

do 210 it=1,5

a22=h1+x*(1-2*h1)*q1bar

! approximation for coefficient of 1st order term in q2 in distribution function

call integ3(gam1,a22,b221d,alpha2j1,beta2j1,nsimp,ainteg10i,ainteg11i,ainteg12i,aint10,aint11,aint12,aint2)

const2=ainteg10i+aint10+aint2

! integration constant for q2

q2bar=(ainteg11i+aint11+aint2)/const2

! write(*,*) 'Mean of q2= ',q2bar

q2var=(ainteg12i+aint12+aint2)/const2

q2var=q2var-q2bar**2

! write(*,*) 'Variance of q2= ',q2var

a11=h1+y*(1-2*h1)*q2bar

! approximation for coefficient of 1st order term in q1 in distribution function

call integ3(gam2,a11,b111d,alpha1i,beta1i,nsimp,ainteg10i,ainteg11i,ainteg12i,aint10,aint11,aint12,aint2)

const1=ainteg10i+aint10+aint2

! integration constant for q1

q1bar=(ainteg11i+aint11+aint2)/const1

! write(*,*) 'Mean of q1= ',q1bar

q1var=(ainteg12i+aint12+aint2)/const1

q1var=q1var-q1bar**2

! write(*,*) 'Variance of q1= ',q1var

cp12=0

r12=0

210 continue

! Iterates means and variances of q1 and q2

qbjn1(i,j)=q1bar

qbjn2(i,j)=q2bar

F1=q1var/(q1bar*(1-q1bar))

F2=q2var/(q2bar*(1-q2bar))

! write(*,*) 'F1 and F2= ',F1,F2

F1jn(i,j)=F1

F2jn(i,j)=F2

cpjn(i,j)=0

go to 215

end if

call integ1(gamint,a1d,a2d,b11d,b12d,b22d,alpha1i,alpha2j1,beta1i,beta2j1,nsimp,ainteg,ainteg0,cp12)

! write(*,*) 'Integration constant for p.d.f= ',ainteg0

q1bar=ainteg(1,1)/ainteg0

q2bar=ainteg(2,1)/ainteg0

qbjn1(i,j)=q1bar

qbjn2(i,j)=q2bar

q1var=ainteg(1,2)/ainteg0

q1var=q1var-q1bar**2

q2var=ainteg(2,2)/ainteg0

q2var=q2var-q2bar**2

cp12=cp12/ainteg0

135 cpjn(i,j)=cp12

cov12=cp12-q1bar*q2bar

cov12b=cov12b+cov12*P1(i)*P2(j)

r12=cov12/sqrt(q1var*q2var)

! write(*,*) 'Covariance and correlation over demes of q1 and q2= ',cov12,r12

r12b=r12b+r12*P1(i)*P2(j)

F1=q1var/(q1bar*(1-q1bar))

F2=q2var/(q2bar*(1-q2bar))

! write(*,*) 'F1 and F2= ',F1,F2

F1jn(i,j)=F1

F2jn(i,j)=F2

215 F1bar=F1bar+F1*P1(i)*P2(j)

F2bar=F2bar+F2*P1(i)*P2(j)

qbars1=qbars1+q1bar*P1(i)*P2(j)

qbars2=qbars2+q2bar*P1(i)*P2(j)

pi1bar=pi1bar+2*q1bar*(1.0-q1bar)*(1-F1)*P1(i)*P2(j)

pi2bar=pi2bar+2*q2bar*(1.0-q2bar)*(1-F2)*P1(i)*P2(j)

! means of pop gen statistics over all metapopulation mean q's

125 continue

120 continue

! write(*,*) ''

! write(*,*) 'Overall means of q1 and q2 over demes ',qbars1,qbars2

! write(*,*) 'Overall covariance and correlation between q1 and q2 over demes ',cov12b,r12b

! write(*,*) 'Overall means of F1 and F2 over demes ',F1bar,F2bar

! write(*,*) 'Overall diversities over demes ',pi1bar,pi2bar

alw1=0

alw2=0

alb=0

alhon1=0

alhon2=0

B1w=0

B2w=0

do 90 i=1,3

do 95 j=1,2

qbarneut1a=qbjn1(i,j)

qbarneut2a=qbjn2(i,j)

F1=F1jn(i,j)

F2=F2jn(i,j)

cp12=cpjn(i,j)

call loadstats2(qbarneut1a,qbarneut2a,h1,F1,F2,alwn1,alwn2,albn,alhomn1,alhomn2,B1,B2,cp12)

alw1=alw1+alwn1*P1(i)*P2(j)

alw2=alw2+alwn2*P1(i)*P2(j)

alb=alb+albn*P1(i)*P2(j)

alhon1=alhon1+alhomn1*P1(i)*P2(j)

alhon2=alhon2+alhomn2*P1(i)*P2(j)

B1w=B1w+B1*P1(i)*P2(j)

B2w=B2w+B2*P1(i)*P2(j)

95 continue

90 continue

! load statistics for In and St averaged over all metapopn q's

170 alw1=alw1*sc

alw2=alw2*sc

alb=alb*sc

alhon1=alhon1*sc

alhon2=alhon2*sc

B1w=B1w*sc

B2w=B2w*sc

! write (*,*) ''

! write (*,*) 'Load statistics'

! write (*,*) 'Loads within In and St=',alw1,alw2

! write (*,*) 'Load between In and St=',alb

! write (*,*) 'Homozygous load for In and St=',alhon1,alhon2

! write (*,*) 'Inbreeding loads= ',B1w,B2w

! write (*,*) ''

alw1a(i1)=alw1*Pxi

alw2a(i1)=alw2*Pxi

albna(i1)=alb*Pxi

alhomn1a(i1)=alhon1*Pxi

alhomn2a(i1)=alhon2*Pxi

B1wa(i1)=B1w*Pxi

B2wa(i1)=B2w*Pxi

qb1a(i1)=qbars1*Pxi

qb2a(i1)=qbars2*Pxi

pi1b(i1)=pi1bar*Pxi

pi2b(i1)=pi2bar*Pxi

! Stores p.d.f. weighted load statistics for current value of gamma

480 continue

alw1=asum(nsimp,alw1a,x1a,x2a)

alw2=asum(nsimp,alw2a,x1a,x2a)

alb=asum(nsimp,albna,x1a,x2a)

alhon1=asum(nsimp,alhomn1a,x1a,x2a)

alhon2=asum(nsimp,alhomn2a,x1a,x2a)

B1w=asum(nsimp,B1wa,x1a,x2a)

B2w=asum(nsimp,B2wa,x1a,x2a)

qbw1=asum(nsimp,qb1a,x1a,x2a)

qbw2=asum(nsimp,qb2a,x1a,x2a)

pibw1=asum(nsimp,pi1b,x1a,x2a)

pibw2=asum(nsimp,pi2b,x1a,x2a)

alw1s=alw1s+alw1

alw2s=alw2s+alw2

albs=albs+alb

alhom1s=alhom1s+alhon1

alhom2s=alhom2s+alhon2

B1ws=B1ws+B1w

B2ws=B2ws+B2w

qt1=qt1+qbw1

qt2=qt2+qbw2

pi1t=pi1t+pibw1

pi2t=pi2t+pibw2

! compiles integrals of load statistics

tt1=1.0-exp(alb-alw1)

tt2=1.0-exp(alb-alw2)

write (1,*) ''

write (1,*) 'Contributions to mean load statistics over zone 3'

write (1,*) 'Loads within In and St=',alw1,alw2

write (1,*) 'Load between In and St=',alb

write (1,*) 'Homozygous load for In and St=',alhon1,alhon2

write (1,*) 'Inbreeding loads= ',B1w,B2w

write (1,*) ''

write (1,*) 'Selection coefficients for In and St homokaryotypes'

write (1,*) tt1,tt2

write (1,*) ''

write (1,*) 'Contributions to mean A2 freqs=',qbw1,qbw2

write (1,*) 'Contributions to mean diversities=',pibw1,pibw2

write (*,*) ''

write (*,*) 'Contributions to load statistics over zone 3'

write (*,*) 'Loads within In and St=',alw1,alw2

write (*,*) 'Load between In and St=',alb

write (*,*) 'Homozygous load for In and St=',alhon1,alhon2

write (*,*) 'Inbreeding loads= ',B1w,B2w

write (*,*) 'Selection coefficients for In and St homokaryotypes'

write (*,*) tt1,tt2

write (*,*) ''

write (*,*) 'Contributions to mean A2 freqs=',qbw1,qbw2

write (*,*) 'Contributions to mean diversities=',pibw1,pibw2

write (*,*) ''

!!!!!!!!!!!!!!!!!!!!!!!!!!!!!!!!!!!!!!!!!!!!!!!!!!!!!!!!!!!!!!!!!!!!!!!

! 4. Zone 4: strong selection approximation for load statistics

! For both St and In metapopulations, q is distributed as a gamma distribution

! There is a negligible probability of a high q in both metapopns

! Selective coupling between In and St within demes is allowed

! Integration over distribution of metapop gamma values in this zone- terminates at 99% upper percentile

! lower bound gamma for St is gamc3 from previous section

! upper bound gamma corresponds to upper 99th percentile of gamma distribution

write(*,*) ''

write(*,*) 'Zone 4: strong selection zone'

write(1,*) ''

write(1,*) 'Zone 4: strong selection zone'

upgam=upgfac*gambar2/ashape

write(1,*) 'Upper limit to scaled gamma for St metapopn= ',upgfac

write(1,*) 'Lower limit to gamma for St metapopn= ',gamc3

write(1,*) 'Upper limit to gamma for St metapopn= ',upgam

write(*,*) 'Upper limit to scaled gamma for St metapopn= ',upgfac

write(*,*) 'Lower limit to gamma for St metapopn= ',gamc3

write(*,*) 'Upper limit to gamma for St metapopn= ',upgam

x1a=gamc3*ashape/gambar2

x2a=upgam*ashape/gambar2

! lower and upper limits of scaled gamma distribution variable for integrations

xsc=upgfac

call gser(gamser,ashape,xsc,gln)

Pz4=gamser-Ptot

write (1,*) 'Probability of zone 4= ',Pz4

write (*,*) 'Probability of zone 4= ',Pz4

write(*,*) ''

del=(upgam-gamc3)/anint

do 200 i1=1,nint

ga1=gamc3+(i1-1)*del

! write(*,*) 'i1=',i1,' gamma for St metapopn= ',ga1

! write(*,*) ''

! write(1,*) 'Index of gamma value',i1,' gamma for St metapopn= ',ga1

! write(1,*) ''

xi=ashape*ga1/gambar2

! scaled variable for gamma distribution of selection coefficients for St metapopn

Pxi=(xi**(ashape-1))*exp(-xi)

Pxi=Pxi/z

! probability density of xi

sc2=ga1/(2*ANE2)

sc=aninvs*sc2

! product of selection coefficient and number of loci for load calculations

ga3=2*ga1*h1

q2b=alpha2/ga3

ga4=ga3*x/y

! rescaled gamma for In metapopulation

q1b=alpha1/ga4

! write(*,*) 'Means of q1 and q2 for metapopulation ',q1b,q2b

! write(*,*) ''

! write(1,*) 'Means of q1 and q2 for metapopulation ',q1b,q2b

! write(1,*) ''

FST1=1.0/(1+AM1+(2*ga1*r0*x/y))

FST2=1.0/(1+AM2+2*ga1*r0)

! FSTs corrected for selection for In and St demes

alpha1mp=alpha1/(1-FST1)

beta1mp=beta1/(1-FST1)

alpha2mp=alpha2/(1-FST2)

beta2mp=beta2/(1-FST2)

F1=1.0/(1+alpha1mp+beta1mp)

F2=1.0/(1+alpha2mp+beta2mp)

! Scaled parameters and F's for In and St metapopulation

! start of calculations for In demes, assuming weak selection within demes

qc1=(0.1*q1b)/alpha1mp

! boundary of metapopn distribution of q1, such that q1 is effectively neutral (exponential term in distribution is negligible)

P1(1)=(alpha1mp**alpha1mp)*(qc1/q1b)**alpha1mp

! probability of q1 falling into lower boundary

! write(*,*) 'Probability of q1 falling into lower boundary for metapopn=',P1(1)

qb1(1)=(qc1*alpha1mp)/(alpha1mp+1)

pb1(1)=1-qb1(1)

! mean q within lower boundary for In metapopulation

P1(3)=0

! neglects probability of q1 falling into upper boundary

P1(2)=1-P1(1)

! it is assumed that q in the In metapopulation follows a gamma distribution

! and there is a negligible probability of q near 1

! 1 is lower boundary region, 2 is intermediate region

if(P1(2).le.0.001) then

P1(2)=0

P1(1)=1

qb1(2)=0

pb1(2)=0

! case when In is effectively always in the boundary region

else

qb1(2)=(q1b-P1(1)*qb1(1))/P1(2)

pb1(2)=1-qb1(2)

end if

! start of calculations for St demes

qc2=(0.1*q2b)/alpha2mp

! boundary of distribution of q2, such that q2 is effectively neutral (exponential term in distribution is negligible)

P2(1)=(alpha2mp**alpha2mp)*(qc2/q2b)**alpha2mp

! probability of q2 falling into lower boundary

! write(*,*) 'Probability of q2 falling into lower boundary for metapopn=',P2(1)

qb2(1)=(qc2*alpha2mp)/(alpha2mp+1)

pb2(1)=1-qb2(1)

! mean q within lower boundary for St metapopulation

P2(3)=0

! neglects probability of q2 falling into upper boundary

P2(2)=1-P2(1)

! it is assumed that q in the St metapopulation follows a gamma distribution

! and there is a negligible probability of q near 1

! 1 is lower boundary region, 2 is intermediate region

if(P2(2).le.0.001) then

P2(2)=0

P2(1)=1

qb2(2)=0

pb2(2)=0

! case when St is effectively always in the boundary region

else

qb2(2)=(q2b-P2(1)*qb2(1))/P2(2)

pb2(2)=1-qb2(2)

end if

! write(*,*) 'Mean q2 in lower boundary for metapopn= ',qb2(1)

! write(*,*) 'Mean q2 in intermediate region for metapopn= ',qb2(2)

gamint=ga1*r0/y

! overall within-deme gamma

qbars1=0

qbarss1=0

qbars2=0

qbarss2=0

F1bar=0

F2bar=0

cov12b=0

r12b=0

pi1bar=0

pi2bar=0

do 220 i=1,2

alpha1i=alpha1d+AM*x*qb1(i)

beta1i=beta1d+AM*x*pb1(i)

! write(*,*) ''

! write(*,*) 'i for q1 boundary= ',i

! write(*,*) 'alpha1,beta1= ',alpha1i,beta1i

! write(*,*) ''

do 225 j=1,2

alpha2j1=alpha2d+AM*y*qb2(j)

beta2j1=beta2d+AM*y*pb2(j)

! write(*,*) ''

! write(*,*) 'j for q2 boundary= ',j

! write(*,*) 'alpha2,beta2= ',alpha2j1,beta2j1

! write(*,*) ''

call integ1(gamint,a1d,a2d,b11d,b12d,b22d,alpha1i,alpha2j1,beta1i,beta2j1,nsimp,ainteg,ainteg0,cp12)

! write(*,*) 'Integration constant for p.d.f= ',ainteg0

q1bar=ainteg(1,1)/ainteg0

q2bar=ainteg(2,1)/ainteg0

qbjn1(i,j)=q1bar

qbjn2(i,j)=q2bar

qbars1=qbars1+q1bar*P1(i)*P2(j)

qbars2=qbars2+q2bar*P1(i)*P2(j)

! write(*,*) 'Means over demes of q1 and q2= ',q1bar,q2bar

q1var=ainteg(1,2)/ainteg0

q1var=q1var-q1bar**2

q2var=ainteg(2,2)/ainteg0

q2var=q2var-q2bar**2

! write(*,*) 'Variances over demes of q1 and q2= ',q1var,q2var

cp12=cp12/ainteg0

cpjn(i,j)=cp12

cov12=cp12-qbar1*qbar2

cov12b=cov12b+cov12*P1(i)*P2(j)

r12=cov12/sqrt(q1var*q2var)

! write(*,*) 'Covariance and correlation over demes of q1 and q2= ',cov12,r12

r12b=r12b+r12*P1(i)*P2(j)

F11=q1var/(q1bar*(1-q1bar))

F21=q2var/(q2bar*(1-q2bar))

! write(*,*) 'F1 and F2= ',F11,F21

F1jn(i,j)=F11

F2jn(i,j)=F21

cov(i,j)=cov12

F1bar=F1bar+F1*P1(i)*P2(j)

F2bar=F2bar+F2*P1(i)*P2(j)

qbars1=qbars1+q1bar*P1(i)*P2(j)

qbars2=qbars2+q2bar*P1(i)*P2(j)

pi1bar=pi1bar+2*q1bar*(1.0-q1bar)*(1-F1)*P1(i)*P2(j)

pi2bar=pi2bar+2*q2bar*(1.0-q2bar)*(1-F2)*P1(i)*P2(j)

! means of pop gen statistics over all metapopulation mean q's

225 continue

220 continue

alw1=0

alw2=0

alb=0

alhon1=0

alhon2=0

B1w=0

B2w=0

do 190 i=1,2

do 195 j=1,2

qbarneut1a=qbjn1(i,j)

qbarneut2a=qbjn2(i,j)

F1=F1jn(i,j)

F2=F2jn(i,j)

cp12=cpjn(i,j)

call loadstats2(qbarneut1a,qbarneut2a,h1,F1,F2,alwn1,alwn2,albn,alhomn1,alhomn2,B1,B2,cp12)

alw1=alw1+alwn1*P1(i)*P2(j)

alw2=alw2+alwn2*P1(i)*P2(j)

alb=alb+albn*P1(i)*P2(j)

alhon1=alhon1+alhomn1*P1(i)*P2(j)

alhon2=alhon2+alhomn2*P1(i)*P2(j)

B1w=B1w+B1*P1(i)*P2(j)

B2w=B2w+B2*P1(i)*P2(j)

195 continue

190 continue

! load statistics for In and St averaged over all metapopn q's

alw1=alw1*sc

alw2=alw2*sc

alb=alb*sc

alhon1=alhon1*sc

alhon2=alhon2*sc

B1w=B1w*sc

B2w=B2w*sc

! write(*,*) ''

! write(*,*) 'Overall means of q1 and q2 over demes ',qbars1,qbars2

! write(*,*) 'Overall covariance and correlation between q1 and q2 over demes ',cov12b,r12b

! write(*,*) 'Overall means of F1 and F2 over demes ',F1bar,F2bar

! write(*,*) 'Overall diversities over demes ',pi1bar,pi2bar

! write (*,*) ''

! write (*,*) 'Load statistics'

! write (*,*) 'Loads within In and St=',alw1,alw2

! write (*,*) 'Load between In and St=',alb

! write (*,*) 'Homozygous load for In and St=',alhon1,alhon2

! write (*,*) 'Inbreeding loads= ',B1w,B2w

! write (*,*) ''

alw1a(i1)=alw1*Pxi

alw2a(i1)=alw2*Pxi

albna(i1)=alb*Pxi

alhomn1a(i1)=alhon1*Pxi

alhomn2a(i1)=alhon2*Pxi

B1wa(i1)=B1w*Pxi

B2wa(i1)=B2w*Pxi

qb1a(i1)=qbars1*Pxi

qb2a(i1)=qbars1*Pxi

pi1b(i1)=pi1bar*Pxi

pi2b(i1)=pi2bar*Pxi

! Stores p.d.f. weighted load statistics for current value of gamm

200 continue

alw1=asum(nsimp,alw1a,x1a,x2a)

alw2=asum(nsimp,alw2a,x1a,x2a)

alb=asum(nsimp,albna,x1a,x2a)

alhon1=asum(nsimp,alhomn1a,x1a,x2a)

alhon2=asum(nsimp,alhomn2a,x1a,x2a)

B1w=asum(nsimp,B1wa,x1a,x2a)

B2w=asum(nsimp,B2wa,x1a,x2a)

qbw1=asum(nsimp,qb1a,x1a,x2a)

qbw2=asum(nsimp,qb2a,x1a,x2a)

pibw1=asum(nsimp,pi1b,x1a,x2a)

pibw2=asum(nsimp,pi2b,x1a,x2a)

tt1=1.0-exp(alb-alw1)

tt2=1.0-exp(alb-alw2)

alw1s=alw1s+alw1

alw2s=alw2s+alw2

albs=albs+alb

alhom1s=alhom1s+alhon1

alhom2s=alhom2s+alhon2

B1ws=B1ws+B1w

B2ws=B2ws+B2w

qt1=qt1+qbw1

qt2=qt2+qbw2

pi1t=pi1t+pibw1

pi2t=pi2t+pibw2

! compiles integrals of load statistics

write (1,*) ''

write (1,*) 'Contributions to mean load statistics over zone 4 '

write (1,*) 'Loads within In and St=',alw1,alw2

write (1,*) 'Load between In and St=',alb

write (1,*) 'Homozygous load for In and St=',alhon1,alhon2

write (1,*) 'Inbreeding loads= ',B1w,B2w

write (1,*) ''

write (1,*) 'Selection coefficients for In and St homokaryotypes'

write (1,*) tt1,tt2

write (1,*) ''

write (1,*) 'Contributions to mean A2 freqs=',qbw1,qbw2

write (1,*) 'Contributions to mean diversities=',pibw1,pibw2

write (1,*) ''

write (*,*) 'Contributions to mean load statistics over zone 4'

write (*,*) 'Loads within In and St=',alw1,alw2

write (*,*) 'Load between In and St=',alb

write (*,*) 'Homozygous load for In and St=',alhon1,alhon2

write (*,*) 'Inbreeding loads= ',B1w,B2w

write (*,*) 'Selection coefficients for In and St homokaryotypes'

write (*,*) tt1,tt2

write (*,*) ''

write (*,*) 'Contributions to mean A2 freqs=',qbw1,qbw2

write (*,*) 'Contributions to mean diversities=',pibw1,pibw2

write (*,*) ''

tt1s=1.0-exp(albs-alw1s)

tt2s=1.0-exp(albs-alw2s)

write (1,*) ''

write (1,*) 'Mean load statistics over all zones'

write (1,*) 'Loads within In and St=',alw1s,alw2s

write (1,*) 'Load between In and St=',albs

write (1,*) 'Homozygous load for In and St=',alhom1s,alhom2s

write (1,*) 'Inbreeding loads= ',B1ws,B2ws

write (1,*) ''

write (1,*) 'Selection coefficients for In and St homokaryotypes'

write (1,*) tt1s,tt2s

write (1,*) ''

write (1,*) 'Mean frequencies of A2 in In and St=',qt1,qt2

rq12=qt1/qt2

write (1,*) 'Ratio of these=',rq12

write (1,*) ''

write (1,*) 'Mean diversities at selected sites in In and St=',pi1t,pi2t

pir0=pi1t/pi2t

write (1,*) 'Ratio of these=',pir0

write (1,*) 'Mean diversities at neutral sites in In and St=',pi1neut,pi2neut

pir1=pi1t/pi1neut

pir2=pi2t/pi2neut

write (1,*) 'pi-n/pi-s for In and St=',pir1,pir2

pir3=pir1/pir2

write (1,*) 'Ratio of these=',pir3

write (1,*) ''

write (*,*) 'Mean load statistics over all zones'

write (*,*) 'Loads within In and St=',alw1s,alw2s

write (*,*) 'Load between In and St=',albs

write (*,*) 'Homozygous load for In and St=',alhom1s,alhom2s

write (*,*) 'Inbreeding loads= ',B1ws,B2ws

write (*,*) 'Selection coefficients for In and St homokaryotypes'

write (*,*) tt1s,tt2s

write (*,*) ''

write (*,*) 'Mean frequencies of A2 in In and St=',qt1,qt2

rq12=qt1/qt2

write (*,*) 'Ratio of these=',rq12

write (*,*) ''

write (*,*) 'Mean diversities at selected sites in In and St=',pi1t,pi2t

pir0=pi1t/pi2t

write (*,*) 'Ratio of these=',pir0

write (*,*) 'Mean diversities at neutral sites in In and St=',pi1neut,pi2neut

pir1=pi1t/pi1neut

pir2=pi2t/pi2neut

write (*,*) 'pi-n/pi-s for In and St=',pir1,pir2

pir3=pir1/pir2

write (*,*) 'Ratio of these=',pir3

write (*,*) ''

go to 420

400 end program invload4

function gammln(z)

! computes log gamma function of real z>0 using Lanczos approximation

real :: p(8),pia,t,y,sa,x

DATA p/676.5204,-1259.139,771.323,-176.6150,12.5073,-0.1386,9.98436e-6,1.5056e-7/

pia=log(2.50666)

sa=1.0

y=z

do 10, i=1,8

y=y+1

sa=sa+p(i)/y

10 continue

t=z+7.5

x=(z+0.5)*log(t)-t

x=x+pia+log(sa)

x=x-log(z)

gammln=x

end function gammln

function gammq(a,z)

real :: a,b,gammq,z

! Uses gcf,gser

! Returns the incomplete gamma function Q(a, z) ≡ 1 − P (a, z)

real :: gammcf,gamser,gln

if(x.lt.0.0.or.a.le.0.0) then

write (*,* )''

write (*,* ) 'Bad arguments in gammq'

write (*,* )''

go to 10

end if

b=a+1.0

if(x.lt.b)then

! Use the series representation and take its complement

call gser(gamser,a,z,gln)

gammq=1.0-gamser

else

! Use the continued fraction representation

call gcf(gammcf,a,z,gln)

gammq=gammcf

endif

10 end function gammq

subroutine gser(gamser,a,z,gln)

integer :: ITMAX,n

real :: a,gamser,gln,z,EPS

real :: ap,del,sum,gammln

ITMAX=100

EPS=3.0e-5

! Uses gammln

! Returns the incomplete gamma function P(a,x) evaluated by its series representation as

! gamser. Also returns lnΓ(a) as gln.

gln=gammln(a)

! write(*,*) 'a= ',a,' z= ',z,'gamm(a) = ',gln

if(z.le.0.0) then

gamser=0.0

if(z.lt.0.0) then

write(*,*) ''

write(*,*) 'x < 0 in gser'

write(*,*) ''

go to 10

end if

end if

ap=a

sum=1.0/a

del=sum

do 15 n=1,ITMAX

ap=ap+1.0

del=del*z/ap

sum=sum+del

! write(*,*) 'n = ',n,'ap= ',ap,' del= ',del,' sum= ',sum

! if(abs(del).lt.abs(sum*EPS)) goto 20

! write(*,*) 'a too large, ITMAX too small in gser'

! write(*,*) ''

! go to 10

15 continue

20 gamser=sum*exp(-z+a*log(z)-gln)

! write(*,*) 'sum= ',sum,' gamser= ',gamser

10 end subroutine gser

subroutine gcf(gammcf,a,z,gln)

real :: a,gammcf,gln,EPS,FPMIN

real :: an,b,c,d,del,h,z,gammln

integer :: ITMAX,i

ITMAX=100

EPS=3.0e-5

FPMIN=1.0e-30

! Uses gammln

! Returns the incomplete gamma function Q(a, x) evaluated by its continued fraction gammcf representation

! IMAX is the maximum number of iterations

! EPS is the relative accuracy; FPMIN is close to the smallest floating point number permissible

! Set up for evaluating continued fraction by modified Lentz’s method with b0 = 0

gln=gammln(a)

b=z+1.0-a

c=1.0/FPMIN

d=1.0/b

h=d

do 15 i=1,ITMAX

an=0.0-i*(i-a)

b=b+2.0

d=an*d+b

if(abs(d).lt.FPMIN) then

d=FPMIN

end if

c=b+an/c

if(abs(c).lt.FPMIN) then

c=FPMIN

end if

d=1.0/d

del=d*c

h=h*del

if(abs(del-1.0).lt.EPS) goto 10

15 continue

write (*,*) ''

write (*,*) 'Problem in incomplete gamma function subroutine'

write (*,*) 'a too large, ITMAX too small in gcf'

write (*,*) ''

goto 20

10 gammcf=exp(-z+a*log(x)-gln)*h

20 end subroutine gcf

function asum(nsimp,f,x1,x2)

! uses Simpson's rule

real :: f(1000),x,x1,x2,F1,DX,ans

integer :: nsimp,N1,i,j

ans=nsimp

DX=(x2-x1)/ans

j=0

N1=nsimp+1

asum=0.0

do 50 i=1,N1

F1=f(i)

x=x1+(i-1)*DX

! write(*,*) 'i= ',i,'x= ',x,' f= ',F1

40 if(i.eq.N1) then

F1=f(N1)

asum=asum+F1

go to 60

end if

if(i.eq.1) then

asum=F1

go to 50

end if

if(j.eq.0) then

asum=asum+4*F1

j=1

else

asum=asum+2*F1

j=0

end if

50 continue

60 asum=DX*asum/(3.0)

end function asum

subroutine loadstats1(qbar1,qbar2,h1,F1,F2,alwn1,alwn2,albn,alhomn1,alhomn2,B1,B2)

real :: alwn1,alwn2,albn,alhomn1,alhomn2,B1,B2,qbar1,qbar2,F1,F2,h2,h1

! calculates variables for load statistics for In and St without using selection coefficients

h2=h1

pbar1=1-qbar1

pbar2=1-qbar2

alwn1=qbar1*(2*h2+(1-2*h2)*(qbar1+F1*pbar1))

alwn2=qbar2*(2*h2+(1-2*h2)*(qbar2+F2*pbar2))

! variable for total loads within In and St

albn=h1*(qbar1*pbar2+qbar2*pbar1)+qbar1*qbar2

! variable for between-arrangement load for In/St

alhomn1=qbar1

alhomn2=qbar2

! variables for homozygous loads for neutral regions for In and St

B1=alhomn1-alwn1

B2=alhomn2-alwn2

! variables for inbreeding loads for neutral regions for In and St

end subroutine loadstats1

subroutine loadstats2(qbar1,qbar2,h1,F1,F2,alwn1,alwn2,albn,alhomn1,alhomn2,B1,B2,cp12)

real :: alwn1,alwn2,albn,alhom1,alhom2,B1,B2,cp12,qbar1,qbar2,F1,F2,h1,h2

! calculates variables for load statistics for In and St without using selection coefficients

! includes covariance between q1 and q2 for between-arrangement load

h2=h1

pbar1=1-qbar1

pbar2=1-qbar2

alwn1=qbar1*(2*h2+(1-2*h2)*(qbar1+F1*pbar1))

alwn2=qbar2*(2*h2+(1-2*h2)*(qbar2+F2*pbar2))

! variables for total loads within In and St

albn=(h2*(qbar1*pbar2+qbar2*pbar1))+(qbar1*qbar2)+((1-2*h2)*cp12)

! variable for load between In and St

alhomn1=qbar1

alhomn2=qbar2

! variables for homozygous loads for neutral regions for In and St

B1=alhomn1-alwn1

B2=alhomn2-alwn2

! variables for inbreeding loads for neutral regions for In and St

end subroutine loadstats2

subroutine bivpdf(gamma,a1,a2,b11,b12,b22,alpha1,alpha2,beta1,beta2,nsimp,f,f1,f2,f3,f4,f5,f6)

real :: gamma,a1,a2,b11,b12,b22,alpha1,alpha2,beta1,beta2,e2,f(1000,1000)

real :: f1(1000),f2(1000),f3(1000),f4(1000),f5(1000),f6(1000),f7(1000)

integer :: i,j,nsimp,nint

! evaluates marginal distributions of q1 and q2

if(gamma.le.10) then

e1=0.01

e2=0.01

else

e1=0.01/(2*a1*gamma)

e2=0.01/(2*a2*gamma)

end if

al1=0.0-log(alpha1)

al2=0.0-log(alpha2)

bl1=0.0-log(beta1)

bl2=0.0-log(beta2)

x1=e1

x2=1-e1

ale1=log(e1)

y1=e2

y2=1-e2

ale2=log(e2)

! boundary values

nint=nsimp+1

anint=nsimp

del1=(x2-x1)/anint

del2=(y2-y1)/anint

! write(*,*) 'Evaluation of components of p.d.f.'

do 10 i=1,nint

f6(i)=0

f5(i)=0

! initialises joint distributions for interior

x=x1+(i-1)*del1

y=y1+(i-1)*del2

! write(*,*) 'i=',i,' x= ',x

alx1=log(x)

alx2=log(1-x)

aly1=log(y)

aly2=log(1-y)

f1(i)=0.0-2*gamma*(a1*x+b11*x**2)

f1(i)=f1(i)+(alpha1-1)*alx1+(beta1-1)*alx2+alpha2*ale2+al2

f1(i)=exp(f1(i))

if(f1(i).le.0.00001) then

f1(i)=0

end if

! interior distribution of x with y close to zero (without constant of integration)

f2(i)=0.0-2*gamma*(a2+b22+(a1+b12)*x+b11*x**2)+(alpha1-1)*alx1+(beta1-1)*alx2

f2(i)=f2(i)+beta2*ale2+bl2

f2(i)=exp(f2(i))

if(f2(i).le.0.00001) then

f2(i)=0

end if

! interior distribution of x with y close to 1

f3(i)=0.0-2*gamma*(a2*y+b22*y**2)

f3(i)=f3(i)+(alpha2-1)*aly1+(beta2-1)*aly2+alpha1*ale1+al1

f3(i)=exp(f3(i))

if(f3(i).le.0.00001) then

f3(i)=0

end if

! interior distribution of y with x close to zero

f4(i)=0.0-2*gamma*(a1+b11+(a2+b12)*y+b22*y**2)+(alpha2-1)*aly1+(beta2-1)*aly2

f4(i)=f4(i)+beta1*ale1+bl1

f4(i)=exp(f4(i))

if(f4(i).le.0.00001) then

f4(i)=0

end if

! interior distribution of y with x close to 1

! write(*,*) 'f1,f2,f3,f4',f1(i),f2(i),f3(i),f4(i)

do 20 j=1,nint

y=y1+(j-1)*del2

aly1=log(y)

aly2=log(1-y)

z=a1*x+a2*y+b11*(x**2)+b12*x*y+b22*(y**2)

f(i,j)=0.0-2*gamma*z+(alpha1-1)*alx1+(beta1-1)*alx2

f(i,j)=f(i,j)+(alpha2-1)*aly1+(beta2-1)*aly2

f(i,j)=exp(f(i,j))

if(f(i,j).le.0.000001) then

f(i,j)=0

end if

fij=f(i,j)

! write(*,*) 'j= ',j,'f(i,j)= ',fij

! bivariate distribution for q1 and q2 inside boundaries

20 continue

10 continue

do 30 i=1,nint

do 40 j=1,nint

f7(j)=f(i,j)

40 continue

f5(i)=asum(nsimp,f7,y1,y2)

if(f5(i).le.0.00001) then

f5(i)=0

end if

f5i=f5(i)

! write(*,*) 'i= ',i,' f5= ',f5i

30 continue

! marginal distribution for q1 inside boundaries

do 50 j=1,nint

do 60 i=1,nint

f7(i)=f(i,j)

60 continue

f6(j)=asum(nsimp,f7,x1,x2)

if(f6(i).le.0.00001) then

f6(i)=0

end if

! f6j=f6(j)

! write(*,*) 'j= ',j,' f6= ',f6j

50 continue

! marginal distribution for q2 inside boundaries

end subroutine bivpdf

subroutine integ1(gamma,a1,a2,b11,b12,b22,alpha1,alpha2,beta1,beta2,nsimp,ainteg,aintegs,cp12)

real :: x,y,gamma,alpha1,beta1,alpha2,beta2,g(1000),ainteg0,ainteg(2,2),g1(1000),g2(1000,0:3,2)

real :: x1,x2,x3,ai,a1,a2,b11,b12,b22,f1(1000),f2(1000),f3(1000),f4(1000),f5(1000),f6(1000),aintegs

real :: f0(1000,1000),g3(1000,1000),g4(1000),cp(1000),cp12,gcp(1000)

integer :: nsimp,k,j,i

! calculates integral of the bivariate p.d.f.and the moments of q1 and q2

! k is the index for the non-zero moments; j=0 indicates 0th moment

! j=1 indicates the index of the moment of q1; j=2 indicates the moment of q2

if(gamma.le.10) then

e1=0.001

e2=0.001

else

e1=0.002/(2*gamma)

e2=0.002/(2*gamma)

end if

! boundaries for x and y (corresponding to near neutrality)

al1=1.0/alpha1

al2=1.0/alpha2

bl1=1.0/beta1

bl2=1.0/beta2

! write(*,*) 'al1= ',al1,'al2= ',al2

! write(*,*) 'bl1= ',bl1,'bl2= ',bl2

ep3=e1**alpha1

ep4=e2**alpha2

ep5=e1**beta1

ep6=e2**beta2

ep7=exp(-2*gamma*(a1+a2+b11+b12+b22))

! both x and y = 1

ep8=exp(-2*gamma*(a2+b22))

! y=1

ep9=exp(-2*gamma*(a1+b11))

! x=1

x1=e1

x2=1-e1

y1=e2

y2=1-e2

! write(*,*)

! write(*,*) 'ep3,ep4,ep5,ep6',ep3,ep4,ep5,ep6

! write(*,*) 'ep7,ep8,ep9',ep7,ep8,ep9

nint=nsimp+1

anint=nsimp

del1=(x2-x1)/anint

del2=(y2-y1)/anint

! write(*,*) ''

! write(*,*) 'x1= ',x1,' y1= ',y1

! write(*,*) 'de1= ',del1,' del2= ',del2

! write(*,*) ''

call bivpdf(gamma,a1,a2,b11,b12,b22,alpha1,alpha2,beta1,beta2,nsimp,f0,f1,f2,f3,f4,f5,f6)

!!!!!!!!!!!!!!!!!!!!!!!!!!!!!!!!!!!!!!!!!!!!!!!!!!!!!!!!!!!!!!!!!!!!!!!!!!!!!!!!!!

ainteg0=0

crossp=0

! initialises components of integration constant and crossproduct

do 205 j=1,2

do 207 k=1,2

ainteg(j,k)=0

207 continue

205 continue

! initialise integrals of internal marginal distributions

do 200 j=0,2

! write(*,*) 'Index for integ1= ',j

! Section 1: x and y close to zero

! write(*,*) 'Section 1'

if(j.eq.0) then

ainteg0=al1*al2*ep3*ep4

! write(*,*) 'ainteg0= ',ainteg0

! stores initial value of the constant of integration of the p.d.f.

cross1=(e1*ep3*e2*ep4)/((alpha1+1)*(alpha2+1))

! 1st contribution to crossproduct

go to 100

end if

do 95 k=1,2

! write(*,*) 'Power is k= ',k

if(j.eq.1) then

sum1=al2*ep3*ep4*(e1**k)/(alpha1+k)

if(sum1.le.0.00001) then

sum1=0

end if

else

sum1=al1*ep3*ep4*(e2**k)/(alpha2+k)

if(sum1.le.0.00001) then

sum1=0

end if

end if

! write(*,*) 'sum1= ',sum1

ainteg(j,k)=ainteg(j,k)+sum1

95 continue

! Section 2: x close to zero and y close to 1

! write(*,*) 'Section 2'

100 if(j.eq.0) then

sum1=al1*bl2*ep3*ep6*ep8

! write(*,*) 'sum1=',sum1

if(sum1.le.0.00001) then

sum1=0

end if

ainteg0=ainteg0+sum1

! write(*,*) 'ainteg0= ',ainteg0

cross2=(e1*sum1*alpha1/(alpha1+1))

crossp=crossp+cross2

! 2nd contribution to crossproduct

go to 110

end if

do 115 k=1,2

! write(*,*) 'Power is k= ',k

if(j.eq.1) then

sum1=bl2*ep3*ep6*ep8*(e1**k)/(alpha1+k)

if(sum1.le.0.00001) then

sum1=0

end if

else

sum1=al1*bl2*ep3*ep6*ep8

if(sum1.le.0.00001) then

sum1=0

end if

end if

! write(*,*) 'sum1= ',sum1

ainteg(j,k)=ainteg(j,k)+sum1

115 continue

! Section 3: x close to 1 and y close to 0

! write(*,*) 'Section 3'

110 if(j.eq.0) then

sum1=bl1*al2*ep4*ep5*ep9

! write(*,*) 'sum1=',sum1

if(sum1.le.0.00001) then

sum1=0

end if

ainteg0=ainteg0+sum1

! write(*,*) 'ainteg0= ',ainteg0

cross3=(e2*sum1*alpha2/(alpha2+1))

crossp=crossp+cross3

! 3rd contribution to crossproduct

go to 120

end if

do 117 k=1,2

if(j.eq.1) then

sum1=bl1*al2*ep4*ep5*ep9

if(sum1.le.0.00001) then

sum1=0

end if

else

sum1=bl1*al2*ep4*ep5*ep9

sum1=sum1*alpha2*(e2**k)/(alpha2+k)

if(sum1.le.0.00001) then

sum1=0

end if

end if

! write(*,*) 'sum1= ',sum1

ainteg(j,k)=ainteg(j,k)+sum1

117 continue

! Section 4: x close to 1 and y close to 1

! write(*,*) 'Section 4'

120 sum1=bl1*bl2*ep5*ep6*ep7

! write(*,*) 'sum1=',sum1

if(sum1.le.0.00001) then

sum1=0

end if

if(j.eq.0) then

ainteg0=ainteg0+sum1

! write(*,*) 'ainteg0= ',ainteg0

crossp4=sum1

crossp=crossp+crossp4

! 4th contribution to crossproduct

go to 130

end if

! write(*,*) 'sum1= ',sum1

do 118 k=1,2

sum1=bl1*bl2*ep5*ep6*ep7

if(sum1.le.0.00001) then

sum1=0

end if

! write(*,*) 'sum1= ',sum1

ainteg(j,k)=ainteg(j,k)+sum1

118 continue

do 125 k=1,2

ainteg(j,k)=ainteg(j,k)+sum1

125 continue

! Section 5: x close to 0 and y in interior

! write(*,*) 'Section 5'

130 do 10 i=1,nint

y=y1+(i-1)*del2

if(j.eq.0) then

gcp(i)=y*f3(i)*e1*alpha1/(alpha1+1)

! integral of xy over boundary x marginal distribution of y

go to 10

end if

do 15 k=1,2

if(j.eq.1) then

g2(i,j,k)=f3(i)*(e1**k)*alpha1/(alpha1+k)

! integral of x^k over boundary x marginal distribution of y

else

g2(i,j,k)=f3(i)*y**k

end if

15 continue

10 continue

if(j.eq.0) then

ainteg0=ainteg0+asum(nsimp,f3,y1,y2)

! write(*,*) 'ainteg0= ',ainteg0

cross5=asum(nsimp,gcp,y1,y2)

crossp=crossp+cross5

! 5th contribution to crossproduct

go to 140

end if

do 17 k=1,2

do 19 i=1,nint

g1(i)=g2(i,j,k)

19 continue

ainteg(j,k)=ainteg(j,k)+asum(nsimp,g1,y1,y2)

17 continue

! Section 6: x close to 1 and y in interior

! write(*,*) 'Section 6'

140 do 20 i=1,nint

y=y1+(i-1)*del2

if(j.eq.0) then

gcp(i)=f4(i)*y

go to 20

end if

do 25 k=1,2

if(j.eq.1) then

g2(i,j,k)=f4(i)

else

g2(i,j,k)=f4(i)*y**k

end if

! uses marginal distribution for y with x close to 1

25 continue

20 continue

if(j.eq.0) then

ainteg0=ainteg0+asum(nsimp,f4,y1,y2)

! write(*,*) 'ainteg0= ',ainteg0

cross6=asum(nsimp,gcp,y1,y2)

crossp=crossp+cross6

! 6th contribution to crossproduct

! write(*,*) 'ainteg0= ',ainteg0

go to 18

end if

do 27 k=1,2

do 29 i=1,nint

g1(i)=g2(i,j,k)

29 continue

sum1=asum(nsimp,g1,y1,y2)

! write(*,*) 'k= ',k

! write(*,*) 'sum1= ',sum1

ainteg(j,k)=ainteg(j,k)+asum(nsimp,g1,y1,y2)

27 continue

! uses marginal distribution for y with x close to 1

! Section 7: y close to 0 and x in interior

! write(*,*) 'Section 7'

18 do 30 i=1,nint

x=x1+(i-1)*del1

if(j.eq.0) then

gcp(i)=x*f1(i)*e2*alpha2/(alpha2+1)

go to 30

end if

do 35 k=1,2

if(j.eq.1) then

g2(i,j,k)=f1(i)*x**k

! write(*,*) 'i,x,j,k',i,x,j,k,'g2(i,j,k)= ',g2(i,j,k)

else

g2(i,j,k)=f1(i)*alpha2*(e2**k)/(alpha2+k)

! write(*,*) 'i,x,j,k',i,y,j,k,'g2(i,j,k)= ',g2(i,j,k)

end if

35 continue

30 continue

if(j.eq.0) then

ainteg0=ainteg0+asum(nsimp,f1,x1,x2)

! write(*,*) 'ainteg0= ',ainteg0

cross7=asum(nsimp,gcp,x1,x2)

crossp=crossp+cross7

! 7th contribution to crossproduct

! write(*,*) 'ainteg3= ',ainteg3

go to 145

end if

do 37 k=1,2

do 39 i=1,nint

g1(i)=g2(i,j,k)

! write(*,*) 'i',i,'g1= ',g1(i)

39 continue

ainteg(j,k)=ainteg(j,k)+asum(nsimp,g1,x1,x2)

37 continue

! uses marginal distribution for x with y close to 0

! Section 8: y close to 1 and x in interior

! write(*,*) 'Section 8'

145 do 40 i=1,nint

x=x1+(i-1)*del1

if(j.eq.0) then

gcp(i)=x*f2(i)

go to 40

end if

do 45 k=1,2

if(j.eq.1) then

! write(*,*) 'j=',j

! write(*,*) 'Iteration of x=',i,x

! write(*,*) 'g1(i)',g1(i)

g2(i,j,k)=f2(i)*x**k

! write(*,*) 'g2(i,j,k)=',g2(i,j,k)

else

g2(i,j,k)=f2(i)

end if

45 continue

40 continue

if(j.eq.0) then

ainteg0=ainteg0+asum(nsimp,f2,x1,x2)

! write(*,*) 'ainteg0= ',ainteg0

cross8=asum(nsimp,gcp,x1,x2)

crossp=crossp+cross8

! 8th contribution to crossproduct

go to 150

end if

do 47 k=1,2

do 49 i=1,nint

g1(i)=g2(i,j,k)

49 continue

sum1=asum(nsimp,g1,x1,x2)

! write(*,*) 'k= ',k

! write(*,*) 'sum1= ',sum1

ainteg(j,k)=ainteg(j,k)+asum(nsimp,g1,x1,x2)

47 continue

! uses marginal distribution for x with y close to 1

! Section 9: x and y in interior

! write(*,*) 'Section 9'

150 if(j.eq.0) then

do 57 i1=1,nint

x=x1+(i1-1)*del1

do 55 j1=1,nsimp

y=y1+(j1-1)*del2

g3(i1,j1)=x*y*f0(i1,j1)

! for crossproduct of q1 and q2

55 continue

57 continue

end if

do 60 k=1,2

if(j.eq.1) then

do 50 i=1,nint

x=x1+(i-1)*del1

! write(*,*) 'i= ',i,'x =',x,' f5(i)= ',f5(i)

g2(i,1,k)=f5(i)*x**k

! write(*,*) 'g2= ',g2(i,1,k)

! for moment of q1

50 continue

end if

if(j.eq.2) then

do 53 i2=1,nint

y=y1+(i2-1)*del2

! write(*,*) 'i= ',i2,'y =',y,' f6(i)= ',f6(i2)

g2(i2,2,k)=f6(i2)*y**k

! write(*,*) 'g2= ',g2(i2,2,k)

! for moment of q2

53 continue

end if

60 continue

do 58 k=1,2

do 59 i=1,nint

g1(i)=g2(i,j,k)

! write(*,*) 'g1= ',g1(i)

if(k.eq.1) then

do 65 j1=1,nint

g4(j1)=g3(i,j1)

65 continue

cp(i)=asum(nsimp,g4,y1,y2)

! integral of crossproduct over y

end if

59 continue

if(j.eq.1) then

ainteg(j,k)=ainteg(j,k)+asum(nsimp,g1,x1,x2)

end if

if(j.eq.2) then

ainteg(j,k)=ainteg(j,k)+asum(nsimp,g1,y1,y2)

end if

58 continue

if(j.eq.0) then

cp12=asum(nsimp,cp,x1,x2)

! integral of crossproduct over x and y in interior

! write(*,*) 'Crossproduct of x and y over interior= ',cp12

! write(*,*) 'Crossproduct of x and y over boundaries= ',crossp

cp12=crossp+cp12

ainteg0=ainteg0+asum(nsimp,f5,x1,x2)

! write(*,*) 'ainteg0= ',ainteg0

aintegs=ainteg0

! write(*,*) 'Integration constant 1= ',aintegs

end if

200 continue

! write(*,*) 'Final components of moments matrix'

! write(*,*) '1st moments',ainteg(1,1),ainteg(2,1)

! write(*,*) '2nd moments',ainteg(1,2),ainteg(2,2)

end subroutine integ1

subroutine integ2(gamma,a1,b11,alpha1,beta1,nsimp,ainteg10i,ainteg11i,ainteg12i,aint10,aint11,aint12,aint2)

real :: x,y,gamma,a1,b11,alpha1,beta1,ainteg10,ainteg11,ainteg12,aint2,ainteg10i,ainteg11i,ainteg12i

real :: aint30(1000),aint31(1000),aint32(1000),e1,ep1,ep2,ep3,x1,x2,al1,al2,al3

integer :: nsimp,i

! calculates integrals of powers of x for the unvariate p.d.f.

if(gamma.le.10) then

e1=0.01

else

e1=0.01/(2*a1*gamma)

end if

! boundary corresponding to near neutrality

ep1=e1**alpha1

ep2=e1**beta1

ep3=exp(0.0-2*gamma*(a1+b11))

x1=e1

x2=1-e1

aint10=ep1/alpha1

aint11=(e1*ep1)/(alpha1+1)

aint12=((e1**2)*ep1)/(alpha1+2)

! integrals of powers of x over lower boundary

aint2=(e1**beta1)*ep3/beta1

! integrals of powers of x over upper boundary

al1=alpha1-1

al2=beta1-1

nint=nsimp+1

anint=nsimp

del1=(x2-x1)/anint

do 10 i=1,nint

x=x1+(i-1)*del1

y=1-x

al3=exp(0.0-2*gamma*x*(a1+b11*x))

aint30(i)=(x**al1)*(y**al2)*al3

aint31(i)=x*aint30(i)

aint32(i)=x*aint31(i)

10 continue

ainteg10i=asum(nsimp,aint30,x1,x2)

ainteg11i=asum(nsimp,aint31,x1,x2)

ainteg12i=asum(nsimp,aint32,x1,x2)

! integrals of powers of x over interior range of x

end subroutine integ2

subroutine integ3(gamma,a1,b11,alpha1,beta1,nsimp,ainteg10i,ainteg11i,ainteg12i,aint10,aint11,aint12,aint2)

real :: x,y,gamma,a1,b11,alpha1,beta1,ainteg10,ainteg11,ainteg12,aint2,ainteg10i,ainteg11i,ainteg12i

real :: aint30(1000),aint31(1000),aint32(1000),e1,ep1,ep2,ep3,x1,x2,al1,al2,al3

integer :: nsimp,i

! calculates integrals of powers for the univariate p.d.f.

if(gamma.le.10) then

e1=0.005

else

e1=0.005/gamma

end if

! boundary corresponding to near neutrality

ep1=e1**alpha1

ep2=e1**beta1

ep3=exp(0.0-2*gamma*(a1+b11))

x1=e1

x2=1-e1

aint10=ep1/alpha1

aint11=(e1*ep1)/(alpha1+1)

aint12=((e1**2)*ep1)/(alpha1+2)

! integrals of powers of x over lower boundary

aint2=(e1**beta1)*ep3/beta1

! integrals of powers of x over upper boundary

al1=alpha1-1

al2=beta1-1

nint=nsimp+1

anint=nsimp

del1=(x2-x1)/anint

do 10 i=1,nint

x=x1+(i-1)*del1

y=1-x

al3=exp(0.0-2*gamma*x*(a1+b11*x))

aint30(i)=(x**al1)*(y**al2)*al3

aint31(i)=x*aint30(i)

aint32(i)=x*aint31(i)

10 continue

ainteg10i=asum(nsimp,aint30,x1,x2)

ainteg11i=asum(nsimp,aint31,x1,x2)

ainteg12i=asum(nsimp,aint32,x1,x2)

! integrals of powers of x over interior range of x

end subroutine integ3
