# Supplementary material for: The fitness consequences of genetic divergence between polymorphic gene arrangements
Source: Genetics. 2023 Dec 26;226(3):iyad218. doi: 10.1093/genetics/iyad218 (PMC11090464; doi:10.1093/genetics/iyad218)
Supplement: iyad218_Supplementary_Data [file iyad218_supplementary_data.zip › Supplementary_File_4_GENETICS-2023-306559.docx]

**Supplementary File S4 Laboratory Measurements of Fitnesses and Fitness**

**Components of Inversion Genotypes**

**Species: *D. melanogaster***

**Trait: 2^nd^ chromosome egg-to-adult viability, measured by balancer crosses with chromosomes extracted from the Raleigh NC population**

(Mukai and Yamaguchi 1974)

Inversion frequency: 0.17 [*In(2R)NS* and *In(2L)t* pooled]

Lethal frequencies: O.54 (*In*), 0.36 (*St*)

Chromosomal heterozygotes: With inversion: 1.008±0.066 Inversion free: 0.996±0.005

Chromosomal homozygotes: With inversion: 0.725±0.030 Inversion free: 0.716±0.011

(lethal free)

**Trait: 3^nd^ chromosome egg-to-adult viability, measured by balancer crosses with chromosomes extracted from the Raleigh NC population**

(Watanabe, et al. 1976)

Inversion frequency: 0.11 [*In(3L)P*, *In(3R)P* and *In(3R)C* pooled] Raleigh NC population

Lethal frequencies: O.54 (*In*), 0.49 (*St*)

Chromosomal heterozygotes: With inversion: 1.010±0.013 Inversion free: 0.997±0.007

Chromosomal homozygotes: With inversion: 0.560±0.028 Inversion free: 0.640±0.009

(lethal free)

**Species: *D. pseudoobscura***

**Trait: viability estimates from deviations from Hardy-Weinberg proportions of 3^rd^ chromosome inversions in population cages**

(Dobzhansky 1947)

Inversion genotype Relative Larval Viability

ST/ST 0.91

ST/CH 1

CH/CH 0.78

______________________________

AR/AR 0.92

AR/CH 1

CH/CH 0.80

Inversion genotype Relative Egg-to-Adult Viability

ST/ST 0.69

ST/CH 1

CH/CH 0.28

______________________________

AR/AR 0.80

AR/CH 1

CH/CH 0.54

**Trait: egg-to-adult viability estimates for heterozygous combinations of third chromosomes measured by balancer crosses with chromosomes extracted from a Colorado population**

(Crumpacker and Salceda 1968)

Relative Egg-to-Adult Viability

AR/AR 0.978

AR/PP 1

PP/PP 0.960

**Trait: net fitnesses from frequency changes of chromosome 3 inversions from wild populations maintained in population cages, assuming constant fitnesses**

(Wright and Dobzhansky 1946)

Inversion genotype Relative Fitness

ST/ST 0.43

ST/AR 1.30

ST/CH 1

AR/AR 0.05

AR/CH 0.71

CH/CH 0.21

**Trait: female age-specific survival and fecundities for 3^rd^ chromosome karyotypes, yielding estimates of net fitnesses**

(Anderson and Watanabe 1997)

Inversion genotype Relative Fitness

AR/AR 0.86

AR/CH 1

CH/CH 0.73

______________________________

AR/AR 1.05

AR/PP 1

PP/PP 1.01

______________________________

AR/AR 1.04

AR/ST 1

ST/ST 0.73

Anderson WW, Watanabe TK. 1997. A demographic approach to selection. Proc. Natl. Acad. Sci. USA 94:7742-7747.

Crumpacker DW, Salceda VM. 1968. Uniform heterokaryotypic superiority for viability in a Colorado populaiton of *Drosophila pseudooscura*. Evolution 22:256-261.

Dobzhansky T. 1947. Genetics of natural populations. XIV. A response of certain gene arrangements in the third chromosome of *Drosophila pseudoobscura* to natural selection. Genetics 32:142-160.

Mukai T, Yamaguchi O. 1974. The genetic structure of natural populations of *Drosophila melanogaster.* XI. Genetic variability in a local population. Genetics 76:339-366.

Watanabe T, Yamaguchi O, Mukai T. 1976. The genetic variability of third chromosomes in a local population of *Drosophila melanogaster*. Genetics 82:63-82.

Wright S, Dobzhansky T. 1946. Genetics of natural populations. XII. Experimental reproduction of some of the changes caused by natural selection in certain populations of *Drosophila pseudoobscura*. Genetics 31:125-156.
