# Supplementary material for: The fitness consequences of genetic divergence between polymorphic gene arrangements
Source: Genetics. 2023 Dec 26;226(3):iyad218. doi: 10.1093/genetics/iyad218 (PMC11090464; doi:10.1093/genetics/iyad218)
Supplement: iyad218_Supplementary_Data [file iyad218_supplementary_data.zip › Supplementary_Table_1_GENETICS-2023-306559.docx]

**Supplementary Table S1 Results for a single population**

**Results are shown for mean scaled selection coefficients of 1000 and 4000 and inversion frequencies of 0.1, 0.3 and 0.5, with dominance coefficients of h = 0.05, 0.15,0.25,0.35, 0.45 and 0.5**

**Mutation rate towards deleterious variants= 4.99999997E-09**

**Number of selected sites in inversion= 100000**

**Mutational bias towards deleterious variants= 1.50000000**

**Population size= 1000000**

**No. of values for Simpsons rule = 650**

**Sample size= 20**

**Wattersons correction factor= 3.54773974**

**Number of dominance coefficients modelled= 6**

**Shape parameter= 0.3**

**Upper bound gamma value for neutrality in the St popn= 0.250**

**Upper bound whole popn gamma value for zone 2a with h=0.25 = 50**

**Upper bound whole popn gamma value for zone 2b with h=0.25 = 500**

**Upper limit to gamma distribution of z= x/scale parameter = 2.50**

**Threshold gamma value factor for use of approximate p.d.f.= 0.25**

**Section 1**

**Mean selection coefficient= 5.0 E-04**

**Mean scaled selection coefficient for whole popn= 1000**

**Inversion frequency= 0.1**

**h = 0.05**

Zone 1: quasi-neutral zone

Upper bound scaled selection coefficient for neutrality in St metapopulation= 0.250000000

Probability of zone 1= 6.65597618E-02

Integral of selection coefficient over zone 1= 2.13332591E-04

Mean load statistics for zone 1

Mean q1 and q2= 0.600000024

F1 and F2= 0.996677756 0.970873833

Diversities= 1.59467699E-03 1.39805600E-02

Contributions to loads within In and St= 1.27846477E-04 1.26657425E-04

Contribution to load between In and St = 8.19197157E-05

Contributions to homozygous loads for In and St= 1.27999563E-04 1.27999563E-04

Contributions to inbreeding loads for In and St= 0.00000000 0.00000000

Contributions to selection coefficients for In and St homokaryotypes

4.59551811E-05 4.47630882E-05

Contributions to mean A2 freqs= 3.99358571E-02 3.99358571E-02

Contributions to mean diversities= 1.06141320E-04 9.30542767E-04

Contributions to mean freqs. of seg. site= 3.76252021E-04 3.27966758E-03

Delta-theta values= -8.23259354E-04 -6.60312176E-03

Zone 2a: moderate selection; cut-off at moderate gamma for St population

Lower and upper bounds of St popn gamma

0.250000000 250.000000

Probability of zone 2a= 0.452220768

Coefficients for bivariate distribution of q1 and q2

a1= 5.00000035E-03 a2= 4.49999981E-02

b11= 4.50000027E-03 b12= 8.09999928E-02 b22= 0.364499956

Net probability of zone 2a using Simpsons rule= 0.452730894

Contributions to mean load statistics over zone 2a

Contributions to mean loads within In and St= 0.215648159 1.62886514E-03

Contribution to load between In and St= 1.21188201E-02

Contributions to homozygous loads for In and St= 0.216684222 2.92292982E-03

Contributions to inbreeding loads= 1.03568402E-03 1.29406143E-03

Selection coefficients for In and St homokaryotypes

0.184153736 -1.05451345E-02

Selection coefficients for In and St homokaryotypes

0.184153736 -1.05451345E-02

Contributions to mean A2 freqs= 0.159671351 2.61739474E-02

Contributions to mean diversities= 7.17817398E-04 2.70676590E-03

Contributions to mean proportions of seg. sites= 2.59188539E-03 1.21404789E-02

Contributions to delta-theta values= 1.74607038E-02 0.209017992

Zone 2b: moderate selection; gamma for St reaches high value

Lower and upper bounds of St popn gamma

250.000000 2500.00000

Probability of zone 2b 0.373098969

Net probability of zone 2b using Simpsons rule= 0.373098999

Contributions to mean load statistics over zone 2b

Contributions to mean loads within In and St= 3.58150317E-03 1.48560051E-04

Contribution to load between In and St= 3.66715743E-04

Contributions to homozygous loads for In and St= 5.97258145E-03 1.32488040E-03

Contributions to inbreeding loads= 2.39107735E-03 1.17632037E-03

Selection coefficients for In and St homokaryotypes

3.20965052E-03 -2.18153000E-04

Selection coefficients for In and St homokaryotypes

3.20965052E-03 -2.18153000E-04

Contributions to mean A2 freqs= 2.70980265E-04 4.38599636E-05

Contributions to mean diversities= 1.50025982E-04 8.62604938E-05

Contributions to mean proportions of seg. sites= 8.15436069E-04 7.63021875E-04

Contributions to delta-theta values= 0.347277880 0.598923981

Zone 3: strong selection approximation

Lower and upper bounds of St popn gamma= 2500.00000 7500.00049

Zone 3: strong selection approximation

Probability of zone 3= 9.62634087E-02

Mean load statistics over zone 3

Contributions to loads within In and St= 1.75895620E-04 1.47950894E-04

Contribution to load between In and St= 1.44457750E-04

Contributions to homozygous loads for In and St= 1.44395151E-03 1.44395139E-03

Contributions to inbreeding loads= 1.26805354E-03 1.29599741E-03

Selection coefficients for In and St homokaryotypes

3.14116478E-05 3.51667404E-06

Contributions to mean A2 freqs= 6.98618942E-06 6.98618851E-06

Contributions to mean A2 freqs at seg. sites= 0.00000000 0.00000000

Contributions to mean diversities= 1.36074450E-05 1.39311678E-05

Contributions to delta-theta values= 0.540533781 0.636254847

Mean load statistics over all zones

Loads within In and St= 0.219533414 2.05203355E-03

Load between In and St= 1.27119133E-02

Homozygous load for In and St= 0.224228755 5.81976119E-03

Inbreeding loads= 4.69496800E-03 3.76772135E-03

Selection coefficients for In and St homokaryotypes

0.186835229 -1.07169151E-02

Mean frequencies of A2 in In and St= 0.199885175 6.61606565E-02

Ratio of these= 3.02120900

Mean diversities at selected sites in In and St= 9.87592153E-04 3.73750040E-03

Mean diversities at neutral sites in In and St= 1.59467699E-03 1.39805600E-02

pi-n/pi-s for In and St= 0.619305432 0.267335534

Ratio of these= 2.31658483

Mean freqs. of seg. sites= 3.88864242E-03 1.63190439E-02

Ratio of these= 0.238288611

Overall delta-theta values= 9.89863276E-02 0.187472045

Ratio of these= 0.528005779

**h = 0.15**

Zone 1: quasi-neutral zone

Upper bound scaled selection coefficient for neutrality in St metapopulation= 0.250000000

Probability of zone 1= 6.65597618E-02

Integral of selection coefficient over zone 1= 2.13332591E-04

Mean load statistics for zone 1

Mean q1 and q2= 0.600000024

F1 and F2= 0.996677756 0.970873833

Diversities= 1.59467699E-03 1.39805600E-02

Contributions to loads within In and St= 1.27880485E-04 1.26955681E-04

Contribution to load between In and St = 9.21596802E-05

Contributions to homozygous loads for In and St= 1.27999563E-04 1.27999563E-04

Contributions to inbreeding loads for In and St= 8.89178365E-03 8.98778345E-03

Contributions to selection coefficients for In and St homokaryotypes

3.57031822E-05 3.48091125E-05

Contributions to mean A2 freqs= 3.99358571E-02 3.99358571E-02

Contributions to mean diversities= 1.06141320E-04 9.30542767E-04

Contributions to mean freqs. of seg. site= 3.76252021E-04 3.27966758E-03

Delta-theta values= -8.23259354E-04 -6.60312176E-03

Zone 2a: moderate selection; cut-off at moderate gamma for St population

Lower and upper bounds of St popn gamma

0.250000000 416.666656

Probability of zone 2a= 0.530714035

Coefficients for bivariate distribution of q1 and q2

a1= 1.50000006E-02 a2= 0.135000005

b11= 3.50000011E-03 b12= 6.30000010E-02 b22= 0.283499986

Net probability of zone 2a using Simpsons rule= 0.532534182

Contributions to mean load statistics over zone 2a

Contributions to mean loads within In and St= 9.37658623E-02 1.75973296E-03

Contribution to load between In and St= 1.52828544E-02

Contributions to homozygous loads for In and St= 9.44400206E-02 2.49573309E-03

Contributions to inbreeding loads= 6.74161187E-04 7.36002170E-04

Selection coefficients for In and St homokaryotypes

7.54822493E-02 -1.36150122E-02

Selection coefficients for In and St homokaryotypes

7.54822493E-02 -1.36150122E-02

Contributions to mean A2 freqs= 0.125457689 2.68307179E-02

Contributions to mean diversities= 6.33509655E-04 2.36211182E-03

Contributions to mean proportions of seg. sites= 2.45129154E-03 1.05092078E-02

Contributions to delta-theta values= 8.31251740E-02 0.202588975

Zone 2b: moderate selection; gamma for St reaches high value

Lower and upper bounds of St popn gamma

416.666656 4166.66650

Probability of zone 2b 0.353823960

Net probability of zone 2b using Simpsons rule= 0.353824049

Contributions to mean load statistics over zone 2b

Contributions to mean loads within In and St= 2.97516352E-04 1.02800281E-04

Contribution to load between In and St= 1.88904422E-04

Contributions to homozygous loads for In and St= 9.20162303E-04 3.38987564E-04

Contributions to inbreeding loads= 6.22645486E-04 2.36187174E-04

Selection coefficients for In and St homokaryotypes

1.08599663E-04 -8.60691071E-05

Selection coefficients for In and St homokaryotypes

1.08599663E-04 -8.60691071E-05

Contributions to mean A2 freqs= 1.79814033E-05 7.82546340E-06

Contributions to mean diversities= 3.44007567E-05 1.55646176E-05

Contributions to mean proportions of seg. sites= 2.64500617E-04 1.53908346E-04

Contributions to delta-theta values= 0.538583577 0.641220152

Zone 3: strong selection approximation

Lower and upper bounds of St popn gamma= 4166.66650 7500.00049

Zone 3: strong selection approximation

Probability of zone 3= 3.70451212E-02

Mean load statistics over zone 3

Contributions to loads within In and St= 8.44588794E-05 8.34765306E-05

Contribution to load between In and St= 8.33537997E-05

Contributions to homozygous loads for In and St= 2.77838350E-04 2.77838350E-04

Contributions to inbreeding loads= 1.93379426E-04 1.94361855E-04

Selection coefficients for In and St homokaryotypes

1.13248825E-06 1.19209290E-07

Contributions to mean A2 freqs= 9.47256751E-07 9.47256751E-07

Contributions to mean A2 freqs at seg. sites= 0.00000000 0.00000000

Contributions to mean diversities= 1.88344973E-06 1.89326249E-06

Contributions to delta-theta values= 0.626606941 0.643267572

Mean load statistics over all zones

Loads within In and St= 9.42757204E-02 2.07296549E-03

Load between In and St= 1.56472735E-02

Homozygous load for In and St= 9.57660228E-02 3.24055855E-03

Inbreeding loads= 1.49030529E-03 1.16759515E-03

Selection coefficients for In and St homokaryotypes

7.56166577E-02 -1.36668682E-02

Mean frequencies of A2 in In and St= 0.165412486 6.67753443E-02

Ratio of these= 2.47714901

Mean diversities at selected sites in In and St= 7.75935187E-04 3.31011275E-03

Mean diversities at neutral sites in In and St= 1.59467699E-03 1.39805600E-02

pi-n/pi-s for In and St= 0.486578286 0.236765385

Ratio of these= 2.05510736

Mean freqs. of seg. sites= 3.10993940E-03 1.39616122E-02

Ratio of these= 0.222749308

Overall delta-theta values= 0.114832878 0.158878088

Ratio of these= 0.722773552

**h = 0.25**

Zone 1: quasi-neutral zone

Upper bound scaled selection coefficient for neutrality in St metapopulation= 0.250000000

Probability of zone 1= 6.65597618E-02

Integral of selection coefficient over zone 1= 2.13332591E-04

Mean load statistics for zone 1

Mean q1 and q2= 0.600000024

F1 and F2= 0.996677756 0.970873833

Diversities= 1.59467699E-03 1.39805600E-02

Contributions to loads within In and St= 1.27914507E-04 1.27253923E-04

Contribution to load between In and St = 1.02399645E-04

Contributions to homozygous loads for In and St= 1.27999563E-04 1.27999563E-04

Contributions to inbreeding loads for In and St= 2.32398114E-03 2.33227736E-03

Contributions to selection coefficients for In and St homokaryotypes

2.55107880E-05 2.48551369E-05

Contributions to mean A2 freqs= 3.99358571E-02 3.99358571E-02

Contributions to mean diversities= 1.06141320E-04 9.30542767E-04

Contributions to mean freqs. of seg. site= 3.76252021E-04 3.27966758E-03

Delta-theta values= -8.23259354E-04 -6.60312176E-03

Zone 2a: moderate selection; cut-off at moderate gamma for St population

Lower and upper bounds of St popn gamma

0.250000000 416.666656

Probability of zone 2a= 0.530714035

Coefficients for bivariate distribution of q1 and q2

a1= 2.50000004E-02 a2= 0.224999994

b11= 2.50000018E-03 b12= 4.49999981E-02 b22= 0.202499986

Net probability of zone 2a using Simpsons rule= 0.532534182

Contributions to mean load statistics over zone 2a

Contributions to mean loads within In and St= 5.64802624E-02 1.79508480E-03

Contribution to load between In and St= 1.52294403E-02

Contributions to homozygous loads for In and St= 5.68166375E-02 2.16259458E-03

Contributions to inbreeding loads= 3.36313358E-04 3.67509521E-04

Selection coefficients for In and St homokaryotypes

4.04115915E-02 -1.35250092E-02

Selection coefficients for In and St homokaryotypes

4.04115915E-02 -1.35250092E-02

Contributions to mean A2 freqs= 0.107502550 2.67355796E-02

Contributions to mean diversities= 5.54416270E-04 2.09505367E-03

Contributions to mean proportions of seg. sites= 2.19791476E-03 9.24592186E-03

Contributions to delta-theta values= 0.105095148 0.196109891

Zone 2b: moderate selection; gamma for St reaches high value

Lower and upper bounds of St popn gamma

416.666656 4166.66650

Probability of zone 2b 0.353823960

Net probability of zone 2b using Simpsons rule= 0.353824049

Contributions to mean load statistics over zone 2b

Contributions to mean loads within In and St= 2.77369953E-04 9.28852241E-05

Contribution to load between In and St= 1.82216594E-04

Contributions to homozygous loads for In and St= 5.43648261E-04 1.85187309E-04

Contributions to inbreeding loads= 2.66278308E-04 9.23020780E-05

Selection coefficients for In and St homokaryotypes

9.51290131E-05 -8.92877579E-05

Selection coefficients for In and St homokaryotypes

9.51290131E-05 -8.92877579E-05

Contributions to mean A2 freqs= 1.06545449E-05 4.40723443E-06

Contributions to mean diversities= 2.07441244E-05 8.78221999E-06

Contributions to mean proportions of seg. sites= 1.73975728E-04 9.06854038E-05

Contributions to delta-theta values= 0.576982617 0.656427264

Zone 3: strong selection approximation

Lower and upper bounds of St popn gamma= 4166.66650 7500.00049

Zone 3: strong selection approximation

Probability of zone 3= 3.70451212E-02

Mean load statistics over zone 3

Contributions to loads within In and St= 8.36362888E-05 8.33837184E-05

Contribution to load between In and St= 8.33521190E-05

Contributions to homozygous loads for In and St= 1.66703088E-04 1.66703074E-04

Contributions to inbreeding loads= 8.30668432E-05 8.33193481E-05

Selection coefficients for In and St homokaryotypes

2.98023224E-07 5.96046448E-08

Contributions to mean A2 freqs= 5.68353698E-07 5.68353641E-07

Contributions to mean A2 freqs at seg. sites= 0.00000000 0.00000000

Contributions to mean diversities= 1.13272506E-06 1.13625902E-06

Contributions to delta-theta values= 0.634310603 0.644052804

Mean load statistics over all zones

Loads within In and St= 5.69691844E-02 2.09860760E-03

Load between In and St= 1.55974096E-02

Homozygous load for In and St= 5.76549880E-02 2.64248438E-03

Inbreeding loads= 6.85743522E-04 5.43876551E-04

Selection coefficients for In and St homokaryotypes

4.05276418E-02 -1.35903358E-02

Mean frequencies of A2 in In and St= 0.147449628 6.66764155E-02

Ratio of these= 2.21142101

Mean diversities at selected sites in In and St= 6.82434416E-04 3.03551485E-03

Mean diversities at neutral sites in In and St= 1.59467699E-03 1.39805600E-02

pi-n/pi-s for In and St= 0.427945226 0.217123985

Ratio of these= 1.97097170

Mean freqs. of seg. sites= 2.75913160E-03 1.26275998E-02

Ratio of these= 0.218500078

Overall delta-theta values= 0.122513890 0.147168338

Ratio of these= 0.832474530

**h = 0.35**

Zone 1: quasi-neutral zone

Upper bound scaled selection coefficient for neutrality in St metapopulation= 0.250000000

Probability of zone 1= 6.65597618E-02

Integral of selection coefficient over zone 1= 2.13332591E-04

Mean load statistics for zone 1

Mean q1 and q2= 0.600000024

F1 and F2= 0.996677756 0.970873833

Diversities= 1.59467699E-03 1.39805600E-02

Contributions to loads within In and St= 1.27948530E-04 1.27552179E-04

Contribution to load between In and St = 1.12639609E-04

Contributions to homozygous loads for In and St= 1.27999563E-04 1.27999563E-04

Contributions to inbreeding loads for In and St= 9.97595140E-04 9.99728451E-04

Contributions to selection coefficients for In and St homokaryotypes

1.53183937E-05 1.49011612E-05

Contributions to mean A2 freqs= 3.99358571E-02 3.99358571E-02

Contributions to mean diversities= 1.06141320E-04 9.30542767E-04

Contributions to mean freqs. of seg. site= 3.76252021E-04 3.27966758E-03

Delta-theta values= -8.23259354E-04 -6.60312176E-03

Zone 2a: moderate selection; cut-off at moderate gamma for St population

Lower and upper bounds of St popn gamma

0.250000000 297.619049

Probability of zone 2a= 0.478139609

Coefficients for bivariate distribution of q1 and q2

a1= 3.50000001E-02 a2= 0.314999998

b11= 1.50000013E-03 b12= 2.70000007E-02 b22= 0.121499993

Net probability of zone 2a using Simpsons rule= 0.478944182

Contributions to mean load statistics over zone 2a

Contributions to mean loads within In and St= 3.92727293E-02 1.78625493E-03

Contribution to load between In and St= 1.47480993E-02

Contributions to homozygous loads for In and St= 3.94073315E-02 1.94213574E-03

Contributions to inbreeding loads= 1.34619069E-04 1.55883245E-04

Selection coefficients for In and St homokaryotypes

2.42263675E-02 -1.30462646E-02

Selection coefficients for In and St homokaryotypes

2.42263675E-02 -1.30462646E-02

Contributions to mean A2 freqs= 9.53384340E-02 2.60496438E-02

Contributions to mean diversities= 4.93514002E-04 1.87601394E-03

Contributions to mean proportions of seg. sites= 1.96118862E-03 8.24573822E-03

Contributions to delta-theta values= 0.107245803 0.192842543

Zone 2b: moderate selection; gamma for St reaches high value

Lower and upper bounds of St popn gamma

297.619049 2976.19043

Probability of zone 2b 0.369981229

Net probability of zone 2b using Simpsons rule= 0.369981259

Contributions to mean load statistics over zone 2b

Contributions to mean loads within In and St= 3.02707282E-04 1.12718837E-04

Contribution to load between In and St= 2.06394878E-04

Contributions to homozygous loads for In and St= 4.28871397E-04 1.60816315E-04

Contributions to inbreeding loads= 1.26164014E-04 4.80974413E-05

Selection coefficients for In and St homokaryotypes

9.63211060E-05 -9.36985016E-05

Selection coefficients for In and St homokaryotypes

9.63211060E-05 -9.36985016E-05

Contributions to mean A2 freqs= 1.11205809E-05 5.13429723E-06

Contributions to mean diversities= 2.16650733E-05 1.02315535E-05

Contributions to mean proportions of seg. sites= 1.82364587E-04 1.06063053E-04

Contributions to delta-theta values= 0.578525424 0.657761216

Zone 3: strong selection approximation

Lower and upper bounds of St popn gamma= 2976.19043 7500.00049

Zone 3: strong selection approximation

Probability of zone 3= 7.34623075E-02

Mean load statistics over zone 3

Contributions to loads within In and St= 1.21952784E-04 1.21810670E-04

Contribution to load between In and St= 1.21792946E-04

Contributions to homozygous loads for In and St= 1.73989552E-04 1.73989523E-04

Contributions to inbreeding loads= 5.20367139E-05 5.21787479E-05

Selection coefficients for In and St homokaryotypes

1.78813934E-07 0.00000000

Contributions to mean A2 freqs= 7.45721763E-07 7.45721820E-07

Contributions to mean A2 freqs at seg. sites= 0.00000000 0.00000000

Contributions to mean diversities= 1.48660649E-06 1.49089828E-06

Contributions to delta-theta values= 0.635141134 0.644139647

Mean load statistics over all zones

Loads within In and St= 3.98253351E-02 2.14833673E-03

Load between In and St= 1.51889268E-02

Homozygous load for In and St= 4.01381925E-02 2.40494101E-03

Inbreeding loads= 3.12870834E-04 2.56606814E-04

Selection coefficients for In and St homokaryotypes

2.43353844E-02 -1.31260157E-02

Mean frequencies of A2 in In and St= 0.135286152 6.59913793E-02

Ratio of these= 2.05005789

Mean diversities at selected sites in In and St= 6.22807012E-04 2.81827897E-03

Mean diversities at neutral sites in In and St= 1.59467699E-03 1.39805600E-02

pi-n/pi-s for In and St= 0.390553713 0.201585561

Ratio of these= 1.93740916

Mean freqs. of seg. sites= 2.53426027E-03 1.16463322E-02

Ratio of these= 0.217601582

Overall delta-theta values= 0.128125370 0.141487598

Ratio of these= 0.905559003

**h = 0.45**

Zone 1: quasi-neutral zone

Upper bound scaled selection coefficient for neutrality in St metapopulation= 0.250000000

Probability of zone 1= 6.65597618E-02

Integral of selection coefficient over zone 1= 2.13332591E-04

Mean load statistics for zone 1

Mean q1 and q2= 0.600000024

F1 and F2= 0.996677756 0.970873833

Diversities= 1.59467699E-03 1.39805600E-02

Contributions to loads within In and St= 1.27982552E-04 1.27850435E-04

Contribution to load between In and St = 1.22879574E-04

Contributions to homozygous loads for In and St= 1.27999563E-04 1.27999563E-04

Contributions to inbreeding loads for In and St= 4.27835243E-04 4.28488274E-04

Contributions to selection coefficients for In and St homokaryotypes

5.12599945E-06 4.94718552E-06

Contributions to mean A2 freqs= 3.99358571E-02 3.99358571E-02

Contributions to mean diversities= 1.06141320E-04 9.30542767E-04

Contributions to mean freqs. of seg. site= 3.76252021E-04 3.27966758E-03

Delta-theta values= -8.23259354E-04 -6.60312176E-03

Zone 2a: moderate selection; cut-off at moderate gamma for St population

Lower and upper bounds of St popn gamma

0.250000000 165.343918

Probability of zone 2a= 0.394631982

Coefficients for bivariate distribution of q1 and q2

a1= 4.49999981E-02 a2= 0.404999971

b11= 5.00000140E-04 b12= 9.00000241E-03 b22= 4.05000076E-02

Net probability of zone 2a using Simpsons rule= 0.394790083

Contributions to mean load statistics over zone 2a

Contributions to mean loads within In and St= 2.95560900E-02 1.74768327E-03

Contribution to load between In and St= 1.42050954E-02

Contributions to homozygous loads for In and St= 2.95839831E-02 1.78350136E-03

Contributions to inbreeding loads= 2.79301221E-05 3.58180368E-05

Selection coefficients for In and St homokaryotypes

1.52337551E-02 -1.25353336E-02

Selection coefficients for In and St homokaryotypes

1.52337551E-02 -1.25353336E-02

Contributions to mean A2 freqs= 8.66724551E-02 2.56230012E-02

Contributions to mean diversities= 4.41477634E-04 1.69951899E-03

Contributions to mean proportions of seg. sites= 1.72882201E-03 7.43658654E-03

Contributions to delta-theta values= 9.40375924E-02 0.189217925

Zone 2b: moderate selection; gamma for St reaches high value

Lower and upper bounds of St popn gamma

165.343918 1653.43921

Probability of zone 2b 0.368480891

Net probability of zone 2b using Simpsons rule= 0.368480951

Contributions to mean load statistics over zone 2b

Contributions to mean loads within In and St= 3.29441013E-04 1.55119254E-04

Contribution to load between In and St= 2.41785601E-04

Contributions to homozygous loads for In and St= 3.65014537E-04 1.72282787E-04

Contributions to inbreeding loads= 3.55734992E-05 1.71634438E-05

Selection coefficients for In and St homokaryotypes

8.76784325E-05 -8.66651535E-05

Selection coefficients for In and St homokaryotypes

8.76784325E-05 -8.66651535E-05

Contributions to mean A2 freqs= 1.57931190E-05 8.98892631E-06

Contributions to mean diversities= 3.04712030E-05 1.78943646E-05

Contributions to mean proportions of seg. sites= 2.44041279E-04 1.80623421E-04

Contributions to delta-theta values= 0.557026148 0.648525357

Zone 3: strong selection approximation

Lower and upper bounds of St popn gamma= 1653.43921 7500.00049

Zone 3: strong selection approximation

Probability of zone 3= 0.158470273

Mean load statistics over zone 3

Contributions to loads within In and St= 2.03367832E-04 2.03295655E-04

Contribution to load between In and St= 2.03286589E-04

Contributions to homozygous loads for In and St= 2.25873882E-04 2.25873882E-04

Contributions to inbreeding loads= 2.25061794E-05 2.25782296E-05

Selection coefficients for In and St homokaryotypes

5.96046448E-08 0.00000000

Contributions to mean A2 freqs= 1.45868023E-06 1.45868023E-06

Contributions to mean A2 freqs at seg. sites= 0.00000000 0.00000000

Contributions to mean diversities= 2.90546427E-06 2.91601668E-06

Contributions to delta-theta values= 0.632424712 0.643859148

Mean load statistics over all zones

Loads within In and St= 3.02168801E-02 2.23394856E-03

Load between In and St= 1.47730466E-02

Homozygous load for In and St= 3.03028710E-02 2.30965763E-03

Inbreeding loads= 8.60268192E-05 7.57088346E-05

Selection coefficients for In and St homokaryotypes

1.53251886E-02 -1.26180649E-02

Mean frequencies of A2 in In and St= 0.126625568 6.55693039E-02

Ratio of these= 1.93117142

Mean diversities at selected sites in In and St= 5.80995576E-04 2.65087211E-03

Mean diversities at neutral sites in In and St= 1.59467699E-03 1.39805600E-02

pi-n/pi-s for In and St= 0.364334345 0.189611301

Ratio of these= 1.92148006

Mean freqs. of seg. sites= 2.37715803E-03 1.09259253E-02

Ratio of these= 0.217570409

Overall delta-theta values= 0.132905304 0.139239550

Ratio of these= 0.954508305

**h = 0.5**

Zone 1: quasi-neutral zone

Upper bound scaled selection coefficient for neutrality in St metapopulation= 0.250000000

Probability of zone 1= 6.65597618E-02

Integral of selection coefficient over zone 1= 2.13332591E-04

Mean load statistics for zone 1

Mean q1 and q2= 0.600000024

F1 and F2= 0.996677756 0.970873833

Diversities= 1.59467699E-03 1.39805600E-02

Contributions to loads within In and St= 1.27999563E-04 1.27999563E-04

Contribution to load between In and St = 1.27999563E-04

Contributions to homozygous loads for In and St= 1.27999563E-04 1.27999563E-04

Contributions to inbreeding loads for In and St= 1.10962734E-04 1.11094421E-04

Contributions to selection coefficients for In and St homokaryotypes

0.00000000 0.00000000

Contributions to mean A2 freqs= 3.99358571E-02 3.99358571E-02

Contributions to mean diversities= 1.06141320E-04 9.30542767E-04

Contributions to mean freqs. of seg. site= 3.76252021E-04 3.27966758E-03

Delta-theta values= -8.23259354E-04 -6.60312176E-03

Zone 2a: moderate selection; cut-off at moderate gamma for St population

Lower and upper bounds of St popn gamma

0.250000000 82.6719589

Probability of zone 2a= 0.310401231

Coefficients for bivariate distribution of q1 and q2

a1= 5.00000007E-02 a2= 0.449999988

b11= 0.00000000 b12= 0.00000000 b22= 0.00000000

Net probability of zone 2a using Simpsons rule= 0.310417920

Contributions to mean load statistics over zone 2a

Contributions to mean loads within In and St= 2.61038411E-02 1.68607139E-03

Contribution to load between In and St= 1.38949566E-02

Contributions to homozygous loads for In and St= 2.61038411E-02 1.68607139E-03

Contributions to inbreeding loads= 0.00000000 0.00000000

Selection coefficients for In and St homokaryotypes

1.21346712E-02 -1.22836828E-02

Selection coefficients for In and St homokaryotypes

1.21346712E-02 -1.22836828E-02

Contributions to mean A2 freqs= 8.32208022E-02 2.55209208E-02

Contributions to mean diversities= 4.01572092E-04 1.61039166E-03

Contributions to mean proportions of seg. sites= 1.52060727E-03 6.94942428E-03

Contributions to delta-theta values= 6.30893111E-02 0.177881479

Zone 2b: moderate selection; gamma for St reaches high value

Lower and upper bounds of St popn gamma

82.6719589 826.719604

Probability of zone 2b 0.335337222

Net probability of zone 2b using Simpsons rule= 0.335337132

Contributions to mean load statistics over zone 2b

Contributions to mean loads within In and St= 3.37613194E-04 1.99317394E-04

Contribution to load between In and St= 2.68465345E-04

Contributions to homozygous loads for In and St= 3.37613194E-04 1.99317394E-04

Contributions to inbreeding loads= 0.00000000 0.00000000

Selection coefficients for In and St homokaryotypes

6.91413879E-05 -6.91413879E-05

Selection coefficients for In and St homokaryotypes

6.91413879E-05 -6.91413879E-05

Contributions to mean A2 freqs= 2.88090105E-05 1.85599183E-05

Contributions to mean diversities= 5.06494871E-05 3.68391011E-05

Contributions to mean proportions of seg. sites= 3.61133571E-04 3.55225086E-04

Contributions to delta-theta values= 0.502424538 0.632076800

Zone 3: strong selection approximation

Lower and upper bounds of St popn gamma= 826.719604 7500.00049

Zone 3: strong selection approximation

Probability of zone 3= 0.275844693

Mean load statistics over zone 3

Contributions to loads within In and St= 3.10017320E-04 3.10017291E-04

Contribution to load between In and St= 3.10017291E-04

Contributions to homozygous loads for In and St= 3.10017320E-04 3.10017291E-04

Contributions to inbreeding loads= 0.00000000 0.00000000

Selection coefficients for In and St homokaryotypes

0.00000000 0.00000000

Contributions to mean A2 freqs= 3.22947017E-06 3.22946994E-06

Contributions to mean A2 freqs at seg. sites= 0.00000000 0.00000000

Contributions to mean diversities= 6.41637052E-06 6.45414048E-06

Contributions to delta-theta values= 0.623950720 0.642998576

Mean load statistics over all zones

Loads within In and St= 2.68794708E-02 2.32340558E-03

Load between In and St= 1.46014383E-02

Homozygous load for In and St= 2.68794708E-02 2.32340558E-03

Inbreeding loads= 0.00000000 0.00000000

Selection coefficients for In and St homokaryotypes

1.22029781E-02 -1.23536587E-02

Mean frequencies of A2 in In and St= 0.123188697 6.54785633E-02

Ratio of these= 1.88135922

Mean diversities at selected sites in In and St= 5.64779271E-04 2.58422759E-03

Mean diversities at neutral sites in In and St= 1.59467699E-03 1.39805600E-02

pi-n/pi-s for In and St= 0.354165316 0.184844360

Ratio of these= 1.91601908

Mean freqs. of seg. sites= 2.31852639E-03 1.06484555E-02

Ratio of these= 0.217733592

Overall delta-theta values= 0.135791600 0.139014363

Ratio of these= 0.976817071

**Inversion frequency = 0.3**

**h= 0.05**

Zone 1: quasi-neutral zone

Upper bound scaled selection coefficient for neutrality in St metapopulation= 0.250000000

Probability of zone 1= 7.17716143E-02

Integral of selection coefficient over zone 1= 2.95762147E-04

Mean load statistics for zone 1

Mean q1 and q2= 0.600000024

F1 and F2= 0.990099013 0.977198601

Diversities= 4.75247391E-03 1.09446710E-02

Contributions to loads within In and St= 1.76824775E-04 1.76000627E-04

Contribution to load between In and St = 1.13572663E-04

Contributions to homozygous loads for In and St= 1.77457288E-04 1.77457288E-04

Contributions to inbreeding loads for In and St= 0.00000000 0.00000000

Contributions to selection coefficients for In and St homokaryotypes

6.32405281E-05 6.24060631E-05

Contributions to mean A2 freqs= 4.30629700E-02 4.30629700E-02

Contributions to mean diversities= 3.41092731E-04 7.85516691E-04

Contributions to mean freqs. of seg. site= 1.20722095E-03 2.77224719E-03

Delta-theta values= -2.39169598E-03 -5.25259972E-03

Zone 2a: moderate selection; cut-off at moderate gamma for St population

Lower and upper bounds of St popn gamma

0.250000000 250.000000

Probability of zone 2a= 0.484664857

Coefficients for bivariate distribution of q1 and q2

a1= 1.50000006E-02 a2= 3.50000001E-02

b11= 4.05000001E-02 b12= 0.189000010 b22= 0.220499992

Net probability of zone 2a using Simpsons rule= 0.485215813

Contributions to mean load statistics over zone 2a

Contributions to mean loads within In and St= 2.23049689E-02 2.34220712E-03

Contribution to load between In and St= 2.37363507E-03

Contributions to homozygous loads for In and St= 2.39399914E-02 3.92571045E-03

Contributions to inbreeding loads= 1.63502665E-03 1.58350088E-03

Selection coefficients for In and St homokaryotypes

1.97340250E-02 -3.14712524E-05

Selection coefficients for In and St homokaryotypes

1.97340250E-02 -3.14712524E-05

Contributions to mean A2 freqs= 7.30842277E-02 2.83782054E-02

Contributions to mean diversities= 1.63936790E-03 2.37726117E-03

Contributions to mean proportions of seg. sites= 6.49589440E-03 1.07064433E-02

Contributions to delta-theta values= 0.104657471 0.212258995

Zone 2b: moderate selection; gamma for St reaches high value

Lower and upper bounds of St popn gamma

250.000000 2500.00000

Probability of zone 2b 0.367502570

Net probability of zone 2b using Simpsons rule= 0.367502689

Contributions to mean load statistics over zone 2b

Contributions to mean loads within In and St= 2.54683197E-04 1.51915636E-04

Contribution to load between In and St= 1.66377868E-04

Contributions to homozygous loads for In and St= 1.97651447E-03 1.34364457E-03

Contributions to inbreeding loads= 1.72183104E-03 1.19172817E-03

Selection coefficients for In and St homokaryotypes

8.82744789E-05 -1.44243240E-05

Selection coefficients for In and St homokaryotypes

8.82744789E-05 -1.44243240E-05

Contributions to mean A2 freqs= 4.93122316E-05 3.56288037E-05

Contributions to mean diversities= 9.46382861E-05 6.99882730E-05

Contributions to mean proportions of seg. sites= 7.28792103E-04 6.15103403E-04

Contributions to delta-theta values= 0.539303422 0.596327782

Zone 3: strong selection approximation

Lower and upper bounds of St popn gamma= 2500.00000 5833.33350

Zone 3: strong selection approximation

Probability of zone 3= 6.42038584E-02

Mean load statistics over zone 3

Contributions to loads within In and St= 1.19247365E-04 1.15333329E-04

Contribution to load between In and St= 1.12397654E-04

Contributions to homozygous loads for In and St= 1.12356606E-03 1.12356606E-03

Contributions to inbreeding loads= 1.00431894E-03 1.00823271E-03

Selection coefficients for In and St homokaryotypes

6.85453415E-06 2.92062759E-06

Contributions to mean A2 freqs= 4.56656289E-06 4.56656289E-06

Contributions to mean A2 freqs at seg. sites= 0.00000000 0.00000000

Contributions to mean diversities= 9.06781679E-06 9.10490508E-06

Contributions to delta-theta values= 0.622228861 0.635775328

Mean load statistics over all zones

Loads within In and St= 2.28557233E-02 2.78545683E-03

Load between In and St= 2.76598334E-03

Homozygous load for In and St= 2.72175297E-02 6.57037878E-03

Inbreeding loads= 4.36180923E-03 3.78491823E-03

Selection coefficients for In and St homokaryotypes

1.98892951E-02 1.94907188E-05

Mean frequencies of A2 in In and St= 0.116201080 7.14813694E-02

Ratio of these= 1.62561357

Mean diversities at selected sites in In and St= 2.08416674E-03 3.24187102E-03

Mean diversities at neutral sites in In and St= 4.75247391E-03 1.09446710E-02

pi-n/pi-s for In and St= 0.438543528 0.296205431

Ratio of these= 1.48053849

Mean freqs. of seg. sites= 8.51706602E-03 1.41824801E-02

Ratio of these= 0.600534320

Overall delta-theta values= 0.131851137 0.189047694

Ratio of these= 0.697449088

**h = 0.15**

Zone 1: quasi-neutral zone

Upper bound scaled selection coefficient for neutrality in St metapopulation= 0.250000000

Probability of zone 1= 7.17716143E-02

Integral of selection coefficient over zone 1= 2.95762147E-04

Mean load statistics for zone 1

Mean q1 and q2= 0.600000024

F1 and F2= 0.990099013 0.977198601

Diversities= 4.75247391E-03 1.09446710E-02

Contributions to loads within In and St= 1.76965332E-04 1.76324334E-04

Contribution to load between In and St = 1.27769250E-04

Contributions to homozygous loads for In and St= 1.77457288E-04 1.77457288E-04

Contributions to inbreeding loads for In and St= 8.96378327E-03 8.98435432E-03

Contributions to selection coefficients for In and St homokaryotypes

4.91738319E-05 4.85777855E-05

Contributions to mean A2 freqs= 4.30629700E-02 4.30629700E-02

Contributions to mean diversities= 3.41092731E-04 7.85516691E-04

Contributions to mean freqs. of seg. site= 1.20722095E-03 2.77224719E-03

Delta-theta values= -2.39169598E-03 -5.25259972E-03

Zone 2a: moderate selection; cut-off at moderate gamma for St population

Lower and upper bounds of St popn gamma

0.250000000 416.666656

Probability of zone 2a= 0.566705585

Coefficients for bivariate distribution of q1 and q2

a1= 4.50000018E-02 a2= 0.105000004

b11= 3.15000005E-02 b12= 0.147000000 b22= 0.171499997

Net probability of zone 2a using Simpsons rule= 0.568667829

Contributions to mean load statistics over zone 2a

Contributions to mean loads within In and St= 1.48808984E-02 2.49110325E-03

Contribution to load between In and St= 3.58892418E-03

Contributions to homozygous loads for In and St= 1.57328155E-02 3.30931414E-03

Contributions to inbreeding loads= 8.51955090E-04 8.18209082E-04

Selection coefficients for In and St homokaryotypes

1.12284422E-02 -1.09839439E-03

Selection coefficients for In and St homokaryotypes

1.12284422E-02 -1.09839439E-03

Contributions to mean A2 freqs= 6.40352517E-02 2.92217061E-02

Contributions to mean diversities= 1.31726183E-03 2.02643871E-03

Contributions to mean proportions of seg. sites= 5.45898220E-03 8.98583885E-03

Contributions to delta-theta values= 0.143924236 0.199932575

Zone 2b: moderate selection; gamma for St reaches high value

Lower and upper bounds of St popn gamma

416.666656 4166.66650

Probability of zone 2b 0.332554340

Net probability of zone 2b using Simpsons rule= 0.332554728

Contributions to mean load statistics over zone 2b

Contributions to mean loads within In and St= 1.65321937E-04 9.88108513E-05

Contribution to load between In and St= 1.29682434E-04

Contributions to homozygous loads for In and St= 5.38699562E-04 3.25735920E-04

Contributions to inbreeding loads= 3.73377668E-04 2.26924705E-04

Selection coefficients for In and St homokaryotypes

3.56435776E-05 -3.08752060E-05

Selection coefficients for In and St homokaryotypes

3.56435776E-05 -3.08752060E-05

Contributions to mean A2 freqs= 9.25329459E-06 6.00208614E-06

Contributions to mean diversities= 1.82841559E-05 1.19368042E-05

Contributions to mean proportions of seg. sites= 1.69068298E-04 1.18041011E-04

Contributions to delta-theta values= 0.616324127 0.641237617

Zone 3: strong selection approximation

Lower and upper bounds of St popn gamma= 4166.66650 5833.33350

Zone 3: strong selection approximation

Probability of zone 3= 1.71113610E-02

Mean load statistics over zone 3

Contributions to loads within In and St= 6.01155916E-05 5.99868799E-05

Contribution to load between In and St= 5.98903389E-05

Contributions to homozygous loads for In and St= 1.99629765E-04 1.99629765E-04

Contributions to inbreeding loads= 1.39514275E-04 1.39643031E-04

Selection coefficients for In and St homokaryotypes

2.38418579E-07 1.19209290E-07

Contributions to mean A2 freqs= 5.79201128E-07 5.79201128E-07

Contributions to mean A2 freqs at seg. sites= 0.00000000 0.00000000

Contributions to mean diversities= 1.15650676E-06 1.15758291E-06

Contributions to delta-theta values= 0.640244901 0.643113434

Mean load statistics over all zones

Loads within In and St= 1.52833015E-02 2.82622525E-03

Load between In and St= 3.90626630E-03

Homozygous load for In and St= 1.66486017E-02 4.01213719E-03

Inbreeding loads= 1.36533892E-03 1.18590984E-03

Selection coefficients for In and St homokaryotypes

1.13125443E-02 -1.08063221E-03

Mean frequencies of A2 in In and St= 0.107108057 7.22912624E-02

Ratio of these= 1.48161829

Mean diversities at selected sites in In and St= 1.67779520E-03 2.82504968E-03

Mean diversities at neutral sites in In and St= 4.75247391E-03 1.09446710E-02

pi-n/pi-s for In and St= 0.353036165 0.258121014

Ratio of these= 1.36771572

Mean freqs. of seg. sites= 6.84667611E-03 1.18876351E-02

Ratio of these= 0.575949371

Overall delta-theta values= 0.130617440 0.156893671

Ratio of these= 0.832522035

**h = 0.25**

Zone 1: quasi-neutral zone

Upper bound scaled selection coefficient for neutrality in St metapopulation= 0.250000000

Probability of zone 1= 7.17716143E-02

Integral of selection coefficient over zone 1= 2.95762147E-04

Mean load statistics for zone 1

Mean q1 and q2= 0.600000024

F1 and F2= 0.990099013 0.977198601

Diversities= 4.75247391E-03 1.09446710E-02

Contributions to loads within In and St= 1.77105903E-04 1.76648042E-04

Contribution to load between In and St = 1.41965837E-04

Contributions to homozygous loads for In and St= 1.77457288E-04 1.77457288E-04

Contributions to inbreeding loads for In and St= 2.33020331E-03 2.33198120E-03

Contributions to selection coefficients for In and St homokaryotypes

3.51667404E-05 3.46899033E-05

Contributions to mean A2 freqs= 4.30629700E-02 4.30629700E-02

Contributions to mean diversities= 3.41092731E-04 7.85516691E-04

Contributions to mean freqs. of seg. site= 1.20722095E-03 2.77224719E-03

Delta-theta values= -2.39169598E-03 -5.25259972E-03

Zone 2a: moderate selection; cut-off at moderate gamma for St population

Lower and upper bounds of St popn gamma

0.250000000 416.666656

Probability of zone 2a= 0.566705585

Coefficients for bivariate distribution of q1 and q2

a1= 7.50000030E-02 a2= 0.174999997

b11= 2.25000009E-02 b12= 0.105000004 b22= 0.122499995

Net probability of zone 2a using Simpsons rule= 0.568667829

Contributions to mean load statistics over zone 2a

Contributions to mean loads within In and St= 1.10413739E-02 2.55998434E-03

Contribution to load between In and St= 4.13487805E-03

Contributions to homozygous loads for In and St= 1.14520267E-02 2.95841717E-03

Contributions to inbreeding loads= 4.10654262E-04 3.98430973E-04

Selection coefficients for In and St homokaryotypes

6.88272715E-03 -1.57618523E-03

Selection coefficients for In and St homokaryotypes

6.88272715E-03 -1.57618523E-03

Contributions to mean A2 freqs= 5.75906485E-02 2.93275807E-02

Contributions to mean diversities= 1.12435722E-03 1.77247997E-03

Contributions to mean proportions of seg. sites= 4.73463675E-03 7.83389341E-03

Contributions to delta-theta values= 0.157500982 0.197296023

Zone 2b: moderate selection; gamma for St reaches high value

Lower and upper bounds of St popn gamma

416.666656 4166.66650

Probability of zone 2b 0.332554340

Net probability of zone 2b using Simpsons rule= 0.332554728

Contributions to mean load statistics over zone 2b

Contributions to mean loads within In and St= 1.56480659E-04 8.95215489E-05

Contribution to load between In and St= 1.22373604E-04

Contributions to homozygous loads for In and St= 3.11006734E-04 1.78471018E-04

Contributions to inbreeding loads= 1.54526439E-04 8.89494622E-05

Selection coefficients for In and St homokaryotypes

3.40938568E-05 -3.29017639E-05

Selection coefficients for In and St homokaryotypes

3.40938568E-05 -3.29017639E-05

Contributions to mean A2 freqs= 5.41764712E-06 3.37906840E-06

Contributions to mean diversities= 1.07530132E-05 6.73316572E-06

Contributions to mean proportions of seg. sites= 1.03856641E-04 6.95287818E-05

Contributions to delta-theta values= 0.632677376 0.656436920

Zone 3: strong selection approximation

Lower and upper bounds of St popn gamma= 4166.66650 5833.33350

Zone 3: strong selection approximation

Probability of zone 3= 1.71113610E-02

Mean load statistics over zone 3

Contributions to loads within In and St= 5.99472696E-05 5.99141022E-05

Contribution to load between In and St= 5.98893130E-05

Contributions to homozygous loads for In and St= 1.19777971E-04 1.19777971E-04

Contributions to inbreeding loads= 5.98307342E-05 5.98638435E-05

Selection coefficients for In and St homokaryotypes

5.96046448E-08 0.00000000

Contributions to mean A2 freqs= 3.47520910E-07 3.47520910E-07

Contributions to mean A2 freqs at seg. sites= 0.00000000 0.00000000

Contributions to mean diversities= 6.94359358E-07 6.94746859E-07

Contributions to delta-theta values= 0.642253995 0.643959582

Mean load statistics over all zones

Loads within In and St= 1.14349080E-02 2.88606808E-03

Load between In and St= 4.45910636E-03

Homozygous load for In and St= 1.20602688E-02 3.43412324E-03

Inbreeding loads= 6.25362853E-04 5.48053533E-04

Selection coefficients for In and St homokaryotypes

6.95151091E-03 -1.57427788E-03

Mean frequencies of A2 in In and St= 0.100659385 7.23942816E-02

Ratio of these= 1.39043283

Mean diversities at selected sites in In and St= 1.47689739E-03 2.56542466E-03

Mean diversities at neutral sites in In and St= 4.75247391E-03 1.09446710E-02

pi-n/pi-s for In and St= 0.310763925 0.234399438

Ratio of these= 1.32578790

Mean freqs. of seg. sites= 6.05259975E-03 1.06825922E-02

Ratio of these= 0.566585302

Overall delta-theta values= 0.134314597 0.148010254

Ratio of these= 0.907468200

**h = 0.35**

Zone 1: quasi-neutral zone

Upper bound scaled selection coefficient for neutrality in St metapopulation= 0.250000000

Probability of zone 1= 7.17716143E-02

Integral of selection coefficient over zone 1= 2.95762147E-04

Mean load statistics for zone 1

Mean q1 and q2= 0.600000024

F1 and F2= 0.990099013 0.977198601

Diversities= 4.75247391E-03 1.09446710E-02

Contributions to loads within In and St= 1.77246460E-04 1.76971735E-04

Contribution to load between In and St = 1.56162409E-04

Contributions to homozygous loads for In and St= 1.77457288E-04 1.77457288E-04

Contributions to inbreeding loads for In and St= 9.99195152E-04 9.99652431E-04

Contributions to selection coefficients for In and St homokaryotypes

2.11000443E-05 2.08020210E-05

Contributions to mean A2 freqs= 4.30629700E-02 4.30629700E-02

Contributions to mean diversities= 3.41092731E-04 7.85516691E-04

Contributions to mean freqs. of seg. site= 1.20722095E-03 2.77224719E-03

Delta-theta values= -2.39169598E-03 -5.25259972E-03

Zone 2a: moderate selection; cut-off at moderate gamma for St population

Lower and upper bounds of St popn gamma

0.250000000 297.619049

Probability of zone 2a= 0.511895418

Coefficients for bivariate distribution of q1 and q2

a1= 0.105000004 a2= 0.244999990

b11= 1.35000013E-02 b12= 6.30000010E-02 b22= 7.34999999E-02

Net probability of zone 2a using Simpsons rule= 0.512763560

Contributions to mean load statistics over zone 2a

Contributions to mean loads within In and St= 8.75255000E-03 2.56124442E-03

Contribution to load between In and St= 4.41466086E-03

Contributions to homozygous loads for In and St= 8.92072264E-03 2.72906083E-03

Contributions to inbreeding loads= 1.68172250E-04 1.67819162E-04

Selection coefficients for In and St homokaryotypes

4.32848930E-03 -1.85513496E-03

Selection coefficients for In and St homokaryotypes

4.32848930E-03 -1.85513496E-03

Contributions to mean A2 freqs= 5.25134243E-02 2.87999064E-02

Contributions to mean diversities= 9.91288805E-04 1.57897640E-03

Contributions to mean proportions of seg. sites= 4.19687806E-03 6.96950173E-03

Contributions to delta-theta values= 0.162035525 0.196241319

Zone 2b: moderate selection; gamma for St reaches high value

Lower and upper bounds of St popn gamma

297.619049 2976.19043

Probability of zone 2b 0.359236717

Net probability of zone 2b using Simpsons rule= 0.359237194

Contributions to mean load statistics over zone 2b

Contributions to mean loads within In and St= 1.86875535E-04 1.11126195E-04

Contribution to load between In and St= 1.48690699E-04

Contributions to homozygous loads for In and St= 2.66282150E-04 1.58541297E-04

Contributions to inbreeding loads= 7.94069419E-05 4.74150838E-05

Selection coefficients for In and St homokaryotypes

3.82065773E-05 -3.75509262E-05

Selection coefficients for In and St homokaryotypes

3.82065773E-05 -3.75509262E-05

Contributions to mean A2 freqs= 6.12227814E-06 4.01219086E-06

Contributions to mean diversities= 1.21538360E-05 7.99524787E-06

Contributions to mean proportions of seg. sites= 1.17678392E-04 8.29060373E-05

Contributions to delta-theta values= 0.633589089 0.657864988

Zone 3: strong selection approximation

Lower and upper bounds of St popn gamma= 2976.19043 5833.33350

Zone 3: strong selection approximation

Probability of zone 3= 4.52391505E-02

Mean load statistics over zone 3

Contributions to loads within In and St= 9.23974221E-05 9.23780535E-05

Contribution to load between In and St= 9.23634434E-05

Contributions to homozygous loads for In and St= 1.31947381E-04 1.31947396E-04

Contributions to inbreeding loads= 3.95499992E-05 3.95694369E-05

Selection coefficients for In and St homokaryotypes

5.96046448E-08 0.00000000

Contributions to mean A2 freqs= 4.76212449E-07 4.76212449E-07

Contributions to mean A2 freqs at seg. sites= 0.00000000 0.00000000

Contributions to mean diversities= 9.51575032E-07 9.52057690E-07

Contributions to delta-theta values= 0.642525494 0.644076347

Mean load statistics over all zones

Loads within In and St= 9.20906942E-03 2.94172042E-03

Load between In and St= 4.81187738E-03

Homozygous load for In and St= 9.49640851E-03 3.19700688E-03

Inbreeding loads= 2.87340052E-04 2.55289255E-04

Selection coefficients for In and St homokaryotypes

4.38755751E-03 -1.87194347E-03

Mean frequencies of A2 in In and St= 9.55829918E-02 7.18673691E-02

Ratio of these= 1.32999158

Mean diversities at selected sites in In and St= 1.34548696E-03 2.37344019E-03

Mean diversities at neutral sites in In and St= 4.75247391E-03 1.09446710E-02

pi-n/pi-s for In and St= 0.283112973 0.216858059

Ratio of these= 1.30552208

Mean freqs. of seg. sites= 5.53122116E-03 9.83414520E-03

Ratio of these= 0.562450647

Overall delta-theta values= 0.137001157 0.143764079

Ratio of these= 0.952958167

**h = 0.45**

Zone 1: quasi-neutral zone

Upper bound scaled selection coefficient for neutrality in St metapopulation= 0.250000000

Probability of zone 1= 7.17716143E-02

Integral of selection coefficient over zone 1= 2.95762147E-04

Mean load statistics for zone 1

Mean q1 and q2= 0.600000024

F1 and F2= 0.990099013 0.977198601

Diversities= 4.75247391E-03 1.09446710E-02

Contributions to loads within In and St= 1.77387032E-04 1.77295442E-04

Contribution to load between In and St = 1.70358995E-04

Contributions to homozygous loads for In and St= 1.77457288E-04 1.77457288E-04

Contributions to inbreeding loads for In and St= 4.28325118E-04 4.28464962E-04

Contributions to selection coefficients for In and St homokaryotypes

7.03334808E-06 6.91413879E-06

Contributions to mean A2 freqs= 4.30629700E-02 4.30629700E-02

Contributions to mean diversities= 3.41092731E-04 7.85516691E-04

Contributions to mean freqs. of seg. site= 1.20722095E-03 2.77224719E-03

Delta-theta values= -2.39169598E-03 -5.25259972E-03

Zone 2a: moderate selection; cut-off at moderate gamma for St population

Lower and upper bounds of St popn gamma

0.250000000 165.343918

Probability of zone 2a= 0.423769295

Coefficients for bivariate distribution of q1 and q2

a1= 0.135000005 a2= 0.314999998

b11= 4.50000120E-03 b12= 2.10000053E-02 b22= 2.45000049E-02

Net probability of zone 2a using Simpsons rule= 0.423940033

Contributions to mean load statistics over zone 2a

Contributions to mean loads within In and St= 7.23193772E-03 2.51966785E-03

Contribution to load between In and St= 4.54095099E-03

Contributions to homozygous loads for In and St= 7.26895360E-03 2.55818176E-03

Contributions to inbreeding loads= 3.70188354E-05 3.85167077E-05

Selection coefficients for In and St homokaryotypes

2.68739462E-03 -2.02333927E-03

Selection coefficients for In and St homokaryotypes

2.68739462E-03 -2.02333927E-03

Contributions to mean A2 freqs= 4.88858819E-02 2.85554081E-02

Contributions to mean diversities= 8.90173425E-04 1.42839539E-03

Contributions to mean proportions of seg. sites= 3.75224114E-03 6.27463358E-03

Contributions to delta-theta values= 0.158342063 0.192371130

Zone 2b: moderate selection; gamma for St reaches high value

Lower and upper bounds of St popn gamma

165.343918 1653.43921

Probability of zone 2b 0.373131454

Net probability of zone 2b using Simpsons rule= 0.373131484

Contributions to mean load statistics over zone 2b

Contributions to mean loads within In and St= 2.36943612E-04 1.57178103E-04

Contribution to load between In and St= 1.96930661E-04

Contributions to homozygous loads for In and St= 2.63052323E-04 1.74568544E-04

Contributions to inbreeding loads= 2.61088207E-05 1.73905373E-05

Selection coefficients for In and St homokaryotypes

3.99947166E-05 -3.96966934E-05

Selection coefficients for In and St homokaryotypes

3.99947166E-05 -3.96966934E-05

Contributions to mean A2 freqs= 9.82590245E-06 7.18905949E-06

Contributions to mean diversities= 1.94609183E-05 1.43110910E-05

Contributions to mean proportions of seg. sites= 1.83730604E-04 1.44534526E-04

Contributions to delta-theta values= 0.624220073 0.648720443

Zone 3: strong selection approximation

Lower and upper bounds of St popn gamma= 1653.43921 5833.33350

Zone 3: strong selection approximation

Probability of zone 3= 0.119470537

Mean load statistics over zone 3

Contributions to loads within In and St= 1.66747908E-04 1.66737285E-04

Contribution to load between In and St= 1.66729253E-04

Contributions to homozygous loads for In and St= 1.85254728E-04 1.85254728E-04

Contributions to inbreeding loads= 1.85067820E-05 1.85173867E-05

Selection coefficients for In and St homokaryotypes

0.00000000 0.00000000

Contributions to mean A2 freqs= 1.00376974E-06 1.00376985E-06

Contributions to mean A2 freqs at seg. sites= 0.00000000 0.00000000

Contributions to mean diversities= 2.00531395E-06 2.00657928E-06

Contributions to delta-theta values= 0.641865015 0.643800080

Mean load statistics over all zones

Loads within In and St= 7.81301688E-03 3.02087865E-03

Load between In and St= 5.07496949E-03

Homozygous load for In and St= 7.89471809E-03 3.09546222E-03

Inbreeding loads= 8.17047039E-05 7.45864818E-05

Selection coefficients for In and St homokaryotypes

2.73430347E-03 -2.05624104E-03

Mean frequencies of A2 in In and St= 9.19596851E-02 7.16265738E-02

Ratio of these= 1.28387666

Mean diversities at selected sites in In and St= 1.25273247E-03 2.23022979E-03

Mean diversities at neutral sites in In and St= 4.75247391E-03 1.09446710E-02

pi-n/pi-s for In and St= 0.263595879 0.203773126

Ratio of these= 1.29357529

Mean freqs. of seg. sites= 5.16305771E-03 9.21140052E-03

Ratio of these= 0.560507357

Overall delta-theta values= 0.139198303 0.141034484

Ratio of these= 0.986980617

**h = 0.5**

Zone 1: quasi-neutral zone

Upper bound scaled selection coefficient for neutrality in St metapopulation= 0.250000000

Probability of zone 1= 7.17716143E-02

Integral of selection coefficient over zone 1= 2.95762147E-04

Mean load statistics for zone 1

Mean q1 and q2= 0.600000024

F1 and F2= 0.990099013 0.977198601

Diversities= 4.75247391E-03 1.09446710E-02

Contributions to loads within In and St= 1.77457288E-04 1.77457288E-04

Contribution to load between In and St = 1.77457288E-04

Contributions to homozygous loads for In and St= 1.77457288E-04 1.77457288E-04

Contributions to inbreeding loads for In and St= 1.11061454E-04 1.11089685E-04

Contributions to selection coefficients for In and St homokaryotypes

0.00000000 0.00000000

Contributions to mean A2 freqs= 4.30629700E-02 4.30629700E-02

Contributions to mean diversities= 3.41092731E-04 7.85516691E-04

Contributions to mean freqs. of seg. site= 1.20722095E-03 2.77224719E-03

Delta-theta values= -2.39169598E-03 -5.25259972E-03

Zone 2a: moderate selection; cut-off at moderate gamma for St population

Lower and upper bounds of St popn gamma

0.250000000 82.6719589

Probability of zone 2a= 0.333978772

Coefficients for bivariate distribution of q1 and q2

a1= 0.150000006 a2= 0.349999994

b11= 0.00000000 b12= 0.00000000 b22= 0.00000000

Net probability of zone 2a using Simpsons rule= 0.333996743

Contributions to mean load statistics over zone 2a

Contributions to mean loads within In and St= 6.60985522E-03 2.45381892E-03

Contribution to load between In and St= 4.53184219E-03

Contributions to homozygous loads for In and St= 6.60985522E-03 2.45381892E-03

Contributions to inbreeding loads= 0.00000000 0.00000000

Selection coefficients for In and St homokaryotypes

2.07585096E-03 -2.08020210E-03

Selection coefficients for In and St homokaryotypes

2.07585096E-03 -2.08020210E-03

Contributions to mean A2 freqs= 4.75027598E-02 2.85659190E-02

Contributions to mean diversities= 8.34812177E-04 1.35311403E-03

Contributions to mean proportions of seg. sites= 3.44881532E-03 5.86523861E-03

Contributions to delta-theta values= 0.141242385 0.181534350

Zone 2b: moderate selection; gamma for St reaches high value

Lower and upper bounds of St popn gamma

82.6719589 826.719604

Probability of zone 2b 0.349868536

Net probability of zone 2b using Simpsons rule= 0.349868268

Contributions to mean load statistics over zone 2b

Contributions to mean loads within In and St= 2.71948433E-04 2.06434561E-04

Contribution to load between In and St= 2.39191650E-04

Contributions to homozygous loads for In and St= 2.71948433E-04 2.06434561E-04

Contributions to inbreeding loads= 0.00000000 0.00000000

Selection coefficients for In and St homokaryotypes

3.27825546E-05 -3.27825546E-05

Selection coefficients for In and St homokaryotypes

3.27825546E-05 -3.27825546E-05

Contributions to mean A2 freqs= 1.84517485E-05 1.51114737E-05

Contributions to mean diversities= 3.62970786E-05 2.99940275E-05

Contributions to mean proportions of seg. sites= 3.23983986E-04 2.89349555E-04

Contributions to delta-theta values= 0.602534056 0.632240653

Zone 3: strong selection approximation

Lower and upper bounds of St popn gamma= 826.719604 5833.33350

Zone 3: strong selection approximation

Probability of zone 3= 0.232523978

Mean load statistics over zone 3

Contributions to loads within In and St= 2.70919612E-04 2.70919612E-04

Contribution to load between In and St= 2.70919641E-04

Contributions to homozygous loads for In and St= 2.70919612E-04 2.70919612E-04

Contributions to inbreeding loads= 0.00000000 0.00000000

Selection coefficients for In and St homokaryotypes

0.00000000 0.00000000

Contributions to mean A2 freqs= 2.37616655E-06 2.37616655E-06

Contributions to mean A2 freqs at seg. sites= 0.00000000 0.00000000

Contributions to mean diversities= 4.74386934E-06 4.74867693E-06

Contributions to delta-theta values= 0.639770150 0.642916143

Mean load statistics over all zones

Loads within In and St= 7.33018061E-03 3.10863042E-03

Load between In and St= 5.21941110E-03

Homozygous load for In and St= 7.33018061E-03 3.10863042E-03

Inbreeding loads= 0.00000000 0.00000000

Selection coefficients for In and St homokaryotypes

2.10851431E-03 -2.11298466E-03

Mean frequencies of A2 in In and St= 9.05865580E-02 7.16463774E-02

Ratio of these= 1.26435637

Mean diversities at selected sites in In and St= 1.21694594E-03 2.17337324E-03

Mean diversities at neutral sites in In and St= 4.75247391E-03 1.09446710E-02

pi-n/pi-s for In and St= 0.256065786 0.198578209

Ratio of these= 1.28949594

Mean freqs. of seg. sites= 5.02674049E-03 8.97401571E-03

Ratio of these= 0.560143948

Overall delta-theta values= 0.141111970 0.140790105

Ratio of these= 1.00228608

**Inversion frequency= 0.5**

**h = 0.05**

Zone 1: quasi-neutral zone

Upper bound scaled selection coefficient for neutrality in St metapopulation= 0.250000000

Probability of zone 1= 7.93938339E-02

Integral of selection coefficient over zone 1= 4.58041381E-04

Mean load statistics for zone 1

Mean q1 and q2= 0.600000024

F1 and F2= 0.983606577 0.983606577

Diversities= 7.86884315E-03 7.86884315E-03

Contributions to loads within In and St= 2.73202924E-04 2.73202924E-04

Contribution to load between In and St = 1.75887893E-04

Contributions to homozygous loads for In and St= 2.74824852E-04 2.74824852E-04

Contributions to inbreeding loads for In and St= 0.00000000 0.00000000

Contributions to selection coefficients for In and St homokaryotypes

9.73343849E-05 9.73343849E-05

Contributions to mean A2 freqs= 4.76363041E-02 4.76363041E-02

Contributions to mean diversities= 6.24737644E-04 6.24737644E-04

Contributions to mean freqs. of seg. site= 2.20816326E-03 2.20816326E-03

Delta-theta values= -3.73303890E-03 -3.73303890E-03

Zone 2a: moderate selection; cut-off at moderate gamma for St population

Lower and upper bounds of St popn gamma

0.250000000 250.000000

Probability of zone 2a= 0.530339003

Coefficients for bivariate distribution of q1 and q2

a1= 2.50000004E-02 a2= 2.50000004E-02

b11= 0.112499997 b12= 0.224999994 b22= 0.112499997

Net probability of zone 2a using Simpsons rule= 0.530947506

Contributions to mean load statistics over zone 2a

Contributions to mean loads within In and St= 5.68476645E-03 5.68473013E-03

Contribution to load between In and St= 1.87811872E-03

Contributions to homozygous loads for In and St= 7.60443183E-03 7.60439551E-03

Contributions to inbreeding loads= 1.91966537E-03 1.91966502E-03

Selection coefficients for In and St homokaryotypes

3.79943848E-03 3.79937887E-03

Selection coefficients for In and St homokaryotypes

3.79943848E-03 3.79937887E-03

Contributions to mean A2 freqs= 3.73776853E-02 3.73775475E-02

Contributions to mean diversities= 2.11785012E-03 2.11784802E-03

Contributions to mean proportions of seg. sites= 9.04238131E-03 9.04238224E-03

Contributions to delta-theta values= 0.169070542 0.169071436

Zone 2b: moderate selection; gamma for St reaches high value

Lower and upper bounds of St popn gamma

250.000000 2500.00000

Probability of zone 2b 0.348146498

Net probability of zone 2b using Simpsons rule= 0.348146081

Contributions to mean load statistics over zone 2b

Contributions to mean loads within In and St= 1.52504770E-04 1.52504756E-04

Contribution to load between In and St= 1.33518552E-04

Contributions to homozygous loads for In and St= 1.33306638E-03 1.33306638E-03

Contributions to inbreeding loads= 1.18056161E-03 1.18056196E-03

Selection coefficients for In and St homokaryotypes

1.90138817E-05 1.90138817E-05

Selection coefficients for In and St homokaryotypes

1.90138817E-05 1.90138817E-05

Contributions to mean A2 freqs= 2.63789843E-05 2.63789843E-05

Contributions to mean diversities= 5.17334520E-05 5.17334447E-05

Contributions to mean proportions of seg. sites= 4.50521969E-04 4.50522406E-04

Contributions to delta-theta values= 0.592612982 0.592613339

Zone 3: strong selection approximation

Lower and upper bounds of St popn gamma= 2500.00000 4166.66699

Zone 3: strong selection approximation

Probability of zone 3= 3.02635431E-02

Mean load statistics over zone 3

Contributions to loads within In and St= 7.78885296E-05 7.78885296E-05

Contribution to load between In and St= 7.56815352E-05

Contributions to homozygous loads for In and St= 7.56593887E-04 7.56593887E-04

Contributions to inbreeding loads= 6.78705750E-04 6.78705750E-04

Selection coefficients for In and St homokaryotypes

2.20537186E-06 2.20537186E-06

Contributions to mean A2 freqs= 2.45221031E-06 2.45221031E-06

Contributions to mean A2 freqs at seg. sites= 0.00000000 0.00000000

Contributions to mean diversities= 4.88805108E-06 4.88805108E-06

Contributions to delta-theta values= 0.634943843 0.634943843

Mean load statistics over all zones

Loads within In and St= 6.18836284E-03 6.18832605E-03

Load between In and St= 2.26320676E-03

Homozygous load for In and St= 9.96891595E-03 9.96888056E-03

Inbreeding loads= 3.78055475E-03 3.78055475E-03

Selection coefficients for In and St homokaryotypes

3.91745567E-03 3.91745567E-03

Mean frequencies of A2 in In and St= 8.50428194E-02 8.50426853E-02

Ratio of these= 1.00000155

Mean diversities at selected sites in In and St= 2.79920921E-03 2.79920711E-03

Mean diversities at neutral sites in In and St= 7.86884315E-03 7.86884315E-03

pi-n/pi-s for In and St= 0.355733246 0.355732977

Ratio of these= 1.00000072

Mean freqs. of seg. sites= 1.17485700E-02 1.17485719E-02

Ratio of these= 0.999999821

Overall delta-theta values= 0.154717028 0.154717863

Ratio of these= 0.999994636

**h = 0.15**

Zone 1: quasi-neutral zone

Upper bound scaled selection coefficient for neutrality in St metapopulation= 0.250000000

Probability of zone 1= 7.93938339E-02

Integral of selection coefficient over zone 1= 4.58041381E-04

Mean load statistics for zone 1

Mean q1 and q2= 0.600000024

F1 and F2= 0.983606577 0.983606577

Diversities= 7.86884315E-03 7.86884315E-03

Contributions to loads within In and St= 2.73563346E-04 2.73563346E-04

Contribution to load between In and St = 1.97873887E-04

Contributions to homozygous loads for In and St= 2.74824852E-04 2.74824852E-04

Contributions to inbreeding loads for In and St= 8.97818338E-03 8.97818338E-03

Contributions to selection coefficients for In and St homokaryotypes

7.56978989E-05 7.56978989E-05

Contributions to mean A2 freqs= 4.76363041E-02 4.76363041E-02

Contributions to mean diversities= 6.24737644E-04 6.24737644E-04

Contributions to mean freqs. of seg. site= 2.20816326E-03 2.20816326E-03

Delta-theta values= -3.73303890E-03 -3.73303890E-03

Zone 2a: moderate selection; cut-off at moderate gamma for St population

Lower and upper bounds of St popn gamma

0.250000000 416.666656

Probability of zone 2a= 0.616143107

Coefficients for bivariate distribution of q1 and q2

a1= 7.50000030E-02 a2= 7.50000030E-02

b11= 8.74999985E-02 b12= 0.174999997 b22= 8.74999985E-02

Net probability of zone 2a using Simpsons rule= 0.618314326

Contributions to mean load statistics over zone 2a

Contributions to mean loads within In and St= 5.15023852E-03 5.15023666E-03

Contribution to load between In and St= 2.62880325E-03

Contributions to homozygous loads for In and St= 6.07210724E-03 6.07210398E-03

Contributions to inbreeding loads= 9.21864819E-04 9.21864936E-04

Selection coefficients for In and St homokaryotypes

2.51823664E-03 2.51823664E-03

Selection coefficients for In and St homokaryotypes

2.51823664E-03 2.51823664E-03

Contributions to mean A2 freqs= 3.67072858E-02 3.67072709E-02

Contributions to mean diversities= 1.69638882E-03 1.69638905E-03

Contributions to mean proportions of seg. sites= 7.34124659E-03 7.34124752E-03

Contributions to delta-theta values= 0.180201054 0.180201054

Zone 2b: moderate selection; gamma for St reaches high value

Lower and upper bounds of St popn gamma

416.666656 4166.66650

Probability of zone 2b 0.292605937

Net probability of zone 2b using Simpsons rule= 0.292605817

Contributions to mean load statistics over zone 2b

Contributions to mean loads within In and St= 9.09522132E-05 9.09522278E-05

Contribution to load between In and St= 8.99157021E-05

Contributions to homozygous loads for In and St= 2.99693056E-04 2.99692998E-04

Contributions to inbreeding loads= 2.08740530E-04 2.08740516E-04

Selection coefficients for In and St homokaryotypes

1.01327896E-06 1.01327896E-06

Selection coefficients for In and St homokaryotypes

1.01327896E-06 1.01327896E-06

Contributions to mean A2 freqs= 4.10548046E-06 4.10548046E-06

Contributions to mean diversities= 8.16370539E-06 8.16370448E-06

Contributions to mean proportions of seg. sites= 8.06488169E-05 8.06496391E-05

Contributions to delta-theta values= 0.640878797 0.640882492

Zone 3: strong selection approximation

Lower and upper bounds of St popn gamma= 4166.66650 4166.66699

Zone 3: strong selection approximation

Probability of zone 3= 5.96046448E-08

Mean load statistics over zone 3

Contributions to loads within In and St= 3.61875173E-05 3.61875173E-05

Contribution to load between In and St= 3.61200109E-05

Contributions to homozygous loads for In and St= 1.20398276E-04 1.20398276E-04

Contributions to inbreeding loads= 8.42103036E-05 8.42103036E-05

Selection coefficients for In and St homokaryotypes

5.96046448E-08 5.96046448E-08

Contributions to mean A2 freqs= 2.88956983E-07 2.88956983E-07

Contributions to mean A2 freqs at seg. sites= 0.00000000 0.00000000

Contributions to mean diversities= 5.77444837E-07 5.77444837E-07

Contributions to delta-theta values= 0.642803490 0.642803490

Mean load statistics over all zones

Loads within In and St= 5.55094145E-03 5.55093959E-03

Load between In and St= 2.95271282E-03

Homozygous load for In and St= 6.76702335E-03 6.76702010E-03

Inbreeding loads= 1.21607713E-03 1.21607725E-03

Selection coefficients for In and St homokaryotypes

2.59482861E-03 2.59482861E-03

Mean frequencies of A2 in In and St= 8.43479857E-02 8.43479708E-02

Ratio of these= 1.00000012

Mean diversities at selected sites in In and St= 2.32986757E-03 2.32986780E-03

Mean diversities at neutral sites in In and St= 7.86884315E-03 7.86884315E-03

pi-n/pi-s for In and St= 0.296087682 0.296087712

Ratio of these= 0.999999881

Mean freqs. of seg. sites= 9.63579398E-03 9.63579584E-03

Ratio of these= 0.999999821

Overall delta-theta values= 0.142181337 0.142181396

Ratio of these= 0.999999583

**h = 0.25**

Zone 1: quasi-neutral zone

Upper bound scaled selection coefficient for neutrality in St metapopulation= 0.250000000

Probability of zone 1= 7.93938339E-02

Integral of selection coefficient over zone 1= 4.58041381E-04

Mean load statistics for zone 1

Mean q1 and q2= 0.600000024

F1 and F2= 0.983606577 0.983606577

Diversities= 7.86884315E-03 7.86884315E-03

Contributions to loads within In and St= 2.73923797E-04 2.73923797E-04

Contribution to load between In and St = 2.19859867E-04

Contributions to homozygous loads for In and St= 2.74824852E-04 2.74824852E-04

Contributions to inbreeding loads for In and St= 2.33144779E-03 2.33144779E-03

Contributions to selection coefficients for In and St homokaryotypes

5.40614128E-05 5.40614128E-05

Contributions to mean A2 freqs= 4.76363041E-02 4.76363041E-02

Contributions to mean diversities= 6.24737644E-04 6.24737644E-04

Contributions to mean freqs. of seg. site= 2.20816326E-03 2.20816326E-03

Delta-theta values= -3.73303890E-03 -3.73303890E-03

Zone 2a: moderate selection; cut-off at moderate gamma for St population

Lower and upper bounds of St popn gamma

0.250000000 416.666656

Probability of zone 2a= 0.616143107

Coefficients for bivariate distribution of q1 and q2

a1= 0.125000000 a2= 0.125000000

b11= 6.25000000E-02 b12= 0.125000000 b22= 6.25000000E-02

Net probability of zone 2a using Simpsons rule= 0.618314326

Contributions to mean load statistics over zone 2a

Contributions to mean loads within In and St= 4.61552246E-03 4.61552246E-03

Contribution to load between In and St= 3.11410613E-03

Contributions to homozygous loads for In and St= 5.05604455E-03 5.05604409E-03

Contributions to inbreeding loads= 4.40524745E-04 4.40524804E-04

Selection coefficients for In and St homokaryotypes

1.50030851E-03 1.50030851E-03

Selection coefficients for In and St homokaryotypes

1.50030851E-03 1.50030851E-03

Contributions to mean A2 freqs= 3.53376009E-02 3.53376009E-02

Contributions to mean diversities= 1.44505315E-03 1.44505326E-03

Contributions to mean proportions of seg. sites= 6.30858494E-03 6.30858354E-03

Contributions to delta-theta values= 0.187349856 0.187349617

Zone 2b: moderate selection; gamma for St reaches high value

Lower and upper bounds of St popn gamma

416.666656 4166.66650

Probability of zone 2b 0.292605937

Net probability of zone 2b using Simpsons rule= 0.292605817

Contributions to mean load statistics over zone 2b

Contributions to mean loads within In and St= 8.27892582E-05 8.27892727E-05

Contribution to load between In and St= 8.25187672E-05

Contributions to homozygous loads for In and St= 1.65033838E-04 1.65033838E-04

Contributions to inbreeding loads= 8.22447837E-05 8.22447983E-05

Selection coefficients for In and St homokaryotypes

2.98023224E-07 2.98023224E-07

Selection coefficients for In and St homokaryotypes

2.98023224E-07 2.98023224E-07

Contributions to mean A2 freqs= 2.31252170E-06 2.31252193E-06

Contributions to mean diversities= 4.60769706E-06 4.60769661E-06

Contributions to mean proportions of seg. sites= 4.75164525E-05 4.75160450E-05

Contributions to delta-theta values= 0.655973673 0.655970812

Zone 3: strong selection approximation

Lower and upper bounds of St popn gamma= 4166.66650 4166.66699

Zone 3: strong selection approximation

Probability of zone 3= 5.96046448E-08

Mean load statistics over zone 3

Contributions to loads within In and St= 3.61369202E-05 3.61369202E-05

Contribution to load between In and St= 3.61197453E-05

Contributions to homozygous loads for In and St= 7.22385521E-05 7.22385521E-05

Contributions to inbreeding loads= 3.61019447E-05 3.61019447E-05

Selection coefficients for In and St homokaryotypes

0.00000000 0.00000000

Contributions to mean A2 freqs= 1.73373905E-07 1.73373905E-07

Contributions to mean A2 freqs at seg. sites= 0.00000000 0.00000000

Contributions to mean diversities= 3.46579071E-07 3.46579071E-07

Contributions to delta-theta values= 0.643715501 0.643715501

Mean load statistics over all zones

Loads within In and St= 5.00837248E-03 5.00837248E-03

Load between In and St= 3.45260440E-03

Homozygous load for In and St= 5.56814158E-03 5.56814112E-03

Inbreeding loads= 5.59772539E-04 5.59772598E-04

Selection coefficients for In and St homokaryotypes

1.55454874E-03 1.55454874E-03

Mean frequencies of A2 in In and St= 8.29763860E-02 8.29763860E-02

Ratio of these= 1.00000000

Mean diversities at selected sites in In and St= 2.07474525E-03 2.07474525E-03

Mean diversities at neutral sites in In and St= 7.86884315E-03 7.86884315E-03

pi-n/pi-s for In and St= 0.263665855 0.263665855

Ratio of these= 1.00000000

Mean freqs. of seg. sites= 8.56771600E-03 8.56771413E-03

Ratio of these= 1.00000024

Overall delta-theta values= 0.140884697 0.140884459

Ratio of these= 1.00000167

**h = 0.35**

Zone 1: quasi-neutral zone

Upper bound scaled selection coefficient for neutrality in St metapopulation= 0.250000000

Probability of zone 1= 7.93938339E-02

Integral of selection coefficient over zone 1= 4.58041381E-04

Mean load statistics for zone 1

Mean q1 and q2= 0.600000024

F1 and F2= 0.983606577 0.983606577

Diversities= 7.86884315E-03 7.86884315E-03

Contributions to loads within In and St= 2.74284219E-04 2.74284219E-04

Contribution to load between In and St = 2.41845846E-04

Contributions to homozygous loads for In and St= 2.74824852E-04 2.74824852E-04

Contributions to inbreeding loads for In and St= 9.99515178E-04 9.99515178E-04

Contributions to selection coefficients for In and St homokaryotypes

3.24249268E-05 3.24249268E-05

Contributions to mean A2 freqs= 4.76363041E-02 4.76363041E-02

Contributions to mean diversities= 6.24737644E-04 6.24737644E-04

Contributions to mean freqs. of seg. site= 2.20816326E-03 2.20816326E-03

Delta-theta values= -3.73303890E-03 -3.73303890E-03

Zone 2a: moderate selection; cut-off at moderate gamma for St population

Lower and upper bounds of St popn gamma

0.250000000 297.619049

Probability of zone 2a= 0.559083343

Coefficients for bivariate distribution of q1 and q2

a1= 0.174999997 a2= 0.174999997

b11= 3.75000015E-02 b12= 7.50000030E-02 b22= 3.75000015E-02

Net probability of zone 2a using Simpsons rule= 0.560043395

Contributions to mean load statistics over zone 2a

Contributions to mean loads within In and St= 4.18453524E-03 4.18453524E-03

Contribution to load between In and St= 3.43011064E-03

Contributions to homozygous loads for In and St= 4.36922768E-03 4.36922722E-03

Contributions to inbreeding loads= 1.84693723E-04 1.84693723E-04

Selection coefficients for In and St homokaryotypes

7.54117966E-04 7.54117966E-04

Selection coefficients for In and St homokaryotypes

7.54117966E-04 7.54117966E-04

Contributions to mean A2 freqs= 3.35704386E-02 3.35704386E-02

Contributions to mean diversities= 1.26989081E-03 1.26989116E-03

Contributions to mean proportions of seg. sites= 5.57274139E-03 5.57274325E-03

Contributions to delta-theta values= 0.191557348 0.191557348

Zone 2b: moderate selection; gamma for St reaches high value

Lower and upper bounds of St popn gamma

297.619049 2976.19043

Probability of zone 2b 0.332554400

Net probability of zone 2b using Simpsons rule= 0.332554698

Contributions to mean load statistics over zone 2b

Contributions to mean loads within In and St= 1.06616819E-04 1.06616819E-04

Contribution to load between In and St= 1.06473184E-04

Contributions to homozygous loads for In and St= 1.52103385E-04 1.52103370E-04

Contributions to inbreeding loads= 4.54862857E-05 4.54862893E-05

Selection coefficients for In and St homokaryotypes

1.19209290E-07 1.19209290E-07

Selection coefficients for In and St homokaryotypes

1.19209290E-07 1.19209290E-07

Contributions to mean A2 freqs= 2.83452846E-06 2.83452823E-06

Contributions to mean diversities= 5.64822813E-06 5.64822903E-06

Contributions to mean proportions of seg. sites= 5.85081470E-05 5.85080743E-05

Contributions to delta-theta values= 0.657510221 0.657509685

Zone 3: strong selection approximation

Lower and upper bounds of St popn gamma= 2976.19043 4166.66699

Zone 3: strong selection approximation

Probability of zone 3= 1.71113014E-02

Mean load statistics over zone 3

Contributions to loads within In and St= 5.98997249E-05 5.98997249E-05

Contribution to load between In and St= 5.98891056E-05

Contributions to homozygous loads for In and St= 8.55556864E-05 8.55556864E-05

Contributions to inbreeding loads= 2.56559706E-05 2.56559706E-05

Selection coefficients for In and St homokaryotypes

0.00000000 0.00000000

Contributions to mean A2 freqs= 2.48229043E-07 2.48229043E-07

Contributions to mean A2 freqs at seg. sites= 0.00000000 0.00000000

Contributions to mean diversities= 4.96248106E-07 4.96248106E-07

Contributions to delta-theta values= 0.643961787 0.643961787

Mean load statistics over all zones

Loads within In and St= 4.62533627E-03 4.62533627E-03

Load between In and St= 3.83831887E-03

Homozygous load for In and St= 4.88171168E-03 4.88171121E-03

Inbreeding loads= 2.56376603E-04 2.56376603E-04

Selection coefficients for In and St homokaryotypes

7.86721706E-04 7.86721706E-04

Mean frequencies of A2 in In and St= 8.12098160E-02 8.12098160E-02

Ratio of these= 1.00000000

Mean diversities at selected sites in In and St= 1.90077291E-03 1.90077338E-03

Mean diversities at neutral sites in In and St= 7.86884315E-03 7.86884315E-03

pi-n/pi-s for In and St= 0.241556838 0.241556898

Ratio of these= 0.999999762

Mean freqs. of seg. sites= 7.84435868E-03 7.84436055E-03

Ratio of these= 0.999999762

Overall delta-theta values= 0.140344262 0.140344322

Ratio of these= 0.999999583

**h = 0.45**

Zone 1: quasi-neutral zone

Upper bound scaled selection coefficient for neutrality in St metapopulation= 0.250000000

Probability of zone 1= 7.93938339E-02

Integral of selection coefficient over zone 1= 4.58041381E-04

Mean load statistics for zone 1

Mean q1 and q2= 0.600000024

F1 and F2= 0.983606577 0.983606577

Diversities= 7.86884315E-03 7.86884315E-03

Contributions to loads within In and St= 2.74644612E-04 2.74644612E-04

Contribution to load between In and St = 2.63831811E-04

Contributions to homozygous loads for In and St= 2.74824852E-04 2.74824852E-04

Contributions to inbreeding loads for In and St= 4.28422994E-04 4.28422994E-04

Contributions to selection coefficients for In and St homokaryotypes

1.07884407E-05 1.07884407E-05

Contributions to mean A2 freqs= 4.76363041E-02 4.76363041E-02

Contributions to mean diversities= 6.24737644E-04 6.24737644E-04

Contributions to mean freqs. of seg. site= 2.20816326E-03 2.20816326E-03

Delta-theta values= -3.73303890E-03 -3.73303890E-03

Zone 2a: moderate selection; cut-off at moderate gamma for St population

Lower and upper bounds of St popn gamma

0.250000000 165.343918

Probability of zone 2a= 0.465305537

Coefficients for bivariate distribution of q1 and q2

a1= 0.224999994 a2= 0.224999994

b11= 1.25000030E-02 b12= 2.50000060E-02 b22= 1.25000030E-02

Net probability of zone 2a using Simpsons rule= 0.465493798

Contributions to mean load statistics over zone 2a

Contributions to mean loads within In and St= 3.80048831E-03 3.80048831E-03

Contribution to load between In and St= 3.58439796E-03

Contributions to homozygous loads for In and St= 3.84290237E-03 3.84290214E-03

Contributions to inbreeding loads= 4.24180034E-05 4.24180034E-05

Selection coefficients for In and St homokaryotypes

2.16066837E-04 2.16066837E-04

Selection coefficients for In and St homokaryotypes

2.16066837E-04 2.16066837E-04

Contributions to mean A2 freqs= 3.21506970E-02 3.21506970E-02

Contributions to mean diversities= 1.13944721E-03 1.13944721E-03

Contributions to mean proportions of seg. sites= 4.99977591E-03 4.99977730E-03

Contributions to delta-theta values= 0.191471338 0.191471577

Zone 2b: moderate selection; gamma for St reaches high value

Lower and upper bounds of St popn gamma

165.343918 1653.43921

Probability of zone 2b 0.369981229

Net probability of zone 2b using Simpsons rule= 0.369981200

Contributions to mean load statistics over zone 2b

Contributions to mean loads within In and St= 1.57674382E-04 1.57674338E-04

Contribution to load between In and St= 1.57607385E-04

Contributions to homozygous loads for In and St= 1.75118606E-04 1.75118621E-04

Contributions to inbreeding loads= 1.74441629E-05 1.74441629E-05

Selection coefficients for In and St homokaryotypes

5.96046448E-08 5.96046448E-08

Selection coefficients for In and St homokaryotypes

5.96046448E-08 5.96046448E-08

Contributions to mean A2 freqs= 5.27551720E-06 5.27551811E-06

Contributions to mean diversities= 1.05014287E-05 1.05014296E-05

Contributions to mean proportions of seg. sites= 1.06059124E-04 1.06059502E-04

Contributions to delta-theta values= 0.648721099 0.648722291

Zone 3: strong selection approximation

Lower and upper bounds of St popn gamma= 1653.43921 4166.66699

Zone 3: strong selection approximation

Probability of zone 3= 7.34623075E-02

Mean load statistics over zone 3

Contributions to loads within In and St= 1.21799290E-04 1.21799290E-04

Contribution to load between In and St= 1.21792684E-04

Contributions to homozygous loads for In and St= 1.35325143E-04 1.35325143E-04

Contributions to inbreeding loads= 1.35260116E-05 1.35260116E-05

Selection coefficients for In and St homokaryotypes

0.00000000 0.00000000

Contributions to mean A2 freqs= 5.80006201E-07 5.80006201E-07

Contributions to mean A2 freqs at seg. sites= 0.00000000 0.00000000

Contributions to mean diversities= 1.15942146E-06 1.15942146E-06

Contributions to delta-theta values= 0.643706024 0.643706024

Mean load statistics over all zones

Loads within In and St= 4.35460638E-03 4.35460638E-03

Load between In and St= 4.12763003E-03

Homozygous load for In and St= 4.42817109E-03 4.42817062E-03

Inbreeding loads= 7.35683934E-05 7.35683934E-05

Selection coefficients for In and St homokaryotypes

2.26974487E-04 2.26974487E-04

Mean frequencies of A2 in In and St= 7.97928572E-02 7.97928572E-02

Ratio of these= 1.00000000

Mean diversities at selected sites in In and St= 1.77584565E-03 1.77584565E-03

Mean diversities at neutral sites in In and St= 7.86884315E-03 7.86884315E-03

pi-n/pi-s for In and St= 0.225680649 0.225680649

Ratio of these= 1.00000000

Mean freqs. of seg. sites= 7.32554309E-03 7.32554449E-03

Ratio of these= 0.999999821

Overall delta-theta values= 0.139962971 0.139963150

Ratio of these= 0.999998748

**h = 0.5**

Zone 1: quasi-neutral zone

Upper bound scaled selection coefficient for neutrality in St metapopulation= 0.250000000

Probability of zone 1= 7.93938339E-02

Integral of selection coefficient over zone 1= 4.58041381E-04

Mean load statistics for zone 1

Mean q1 and q2= 0.600000024

F1 and F2= 0.983606577 0.983606577

Diversities= 7.86884315E-03 7.86884315E-03

Contributions to loads within In and St= 2.74824852E-04 2.74824852E-04

Contribution to load between In and St = 2.74824852E-04

Contributions to homozygous loads for In and St= 2.74824852E-04 2.74824852E-04

Contributions to inbreeding loads for In and St= 1.11081237E-04 1.11081237E-04

Contributions to selection coefficients for In and St homokaryotypes

0.00000000 0.00000000

Contributions to mean A2 freqs= 4.76363041E-02 4.76363041E-02

Contributions to mean diversities= 6.24737644E-04 6.24737644E-04

Contributions to mean freqs. of seg. site= 2.20816326E-03 2.20816326E-03

Delta-theta values= -3.73303890E-03 -3.73303890E-03

Zone 2a: moderate selection; cut-off at moderate gamma for St population

Lower and upper bounds of St popn gamma

0.250000000 82.6719589

Probability of zone 2a= 0.368007213

Coefficients for bivariate distribution of q1 and q2

a1= 0.250000000 a2= 0.250000000

b11= 0.00000000 b12= 0.00000000 b22= 0.00000000

Net probability of zone 2a using Simpsons rule= 0.368027240

Contributions to mean load statistics over zone 2a

Contributions to mean loads within In and St= 3.65892518E-03 3.65892518E-03

Contribution to load between In and St= 3.65892495E-03

Contributions to homozygous loads for In and St= 3.65892518E-03 3.65892518E-03

Contributions to inbreeding loads= 0.00000000 0.00000000

Selection coefficients for In and St homokaryotypes

0.00000000 0.00000000

Selection coefficients for In and St homokaryotypes

0.00000000 0.00000000

Contributions to mean A2 freqs= 3.22805345E-02 3.22805345E-02

Contributions to mean diversities= 1.07621157E-03 1.07621180E-03

Contributions to mean proportions of seg. sites= 4.66983858E-03 4.66983812E-03

Contributions to delta-theta values= 0.182387471 0.182387233

Zone 2b: moderate selection; gamma for St reaches high value

Lower and upper bounds of St popn gamma

82.6719589 826.719604

Probability of zone 2b 0.365087926

Net probability of zone 2b using Simpsons rule= 0.365087867

Contributions to mean load statistics over zone 2b

Contributions to mean loads within In and St= 2.14535918E-04 2.14535918E-04

Contribution to load between In and St= 2.14535918E-04

Contributions to homozygous loads for In and St= 2.14535918E-04 2.14535918E-04

Contributions to inbreeding loads= 0.00000000 0.00000000

Selection coefficients for In and St homokaryotypes

0.00000000 0.00000000

Selection coefficients for In and St homokaryotypes

0.00000000 0.00000000

Contributions to mean A2 freqs= 1.14092863E-05 1.14092863E-05

Contributions to mean diversities= 2.26448774E-05 2.26448774E-05

Contributions to mean proportions of seg. sites= 2.18522342E-04 2.18522953E-04

Contributions to delta-theta values= 0.632357359 0.632358372

Zone 3: strong selection approximation

Lower and upper bounds of St popn gamma= 826.719604 4166.66699

Zone 3: strong selection approximation

Probability of zone 3= 0.175653934

Mean load statistics over zone 3

Contributions to loads within In and St= 2.19132649E-04 2.19132649E-04

Contribution to load between In and St= 2.19132649E-04

Contributions to homozygous loads for In and St= 2.19132649E-04 2.19132649E-04

Contributions to inbreeding loads= 0.00000000 0.00000000

Selection coefficients for In and St homokaryotypes

0.00000000 0.00000000

Contributions to mean A2 freqs= 1.52286782E-06 1.52286782E-06

Contributions to mean A2 freqs at seg. sites= 0.00000000 0.00000000

Contributions to mean diversities= 3.04327364E-06 3.04327364E-06

Contributions to delta-theta values= 0.642794728 0.642794728

Mean load statistics over all zones

Loads within In and St= 4.36741859E-03 4.36741859E-03

Load between In and St= 4.36741812E-03

Homozygous load for In and St= 4.36741859E-03 4.36741859E-03

Inbreeding loads= 0.00000000 0.00000000

Selection coefficients for In and St homokaryotypes

0.00000000 0.00000000

Mean frequencies of A2 in In and St= 7.99297616E-02 7.99297616E-02

Ratio of these= 1.00000000

Mean diversities at selected sites in In and St= 1.72663736E-03 1.72663759E-03

Mean diversities at neutral sites in In and St= 7.86884315E-03 7.86884315E-03

pi-n/pi-s for In and St= 0.219427094 0.219427124

Ratio of these= 0.999999881

Mean freqs. of seg. sites= 7.12674949E-03 7.12674996E-03

Ratio of these= 0.999999940

Overall delta-theta values= 0.140469313 0.140469193

Ratio of these= 1.00000083

**Section 2**

**Mean selection coefficient= 2.0E-03**

**Mean scaled selection coefficient for whole population= 4000**

**x = 0.1**

Zone 1: quasi-neutral zone

Upper bound scaled selection coefficient for neutrality in St metapopulation= 0.250000000

Probability of zone 1= 4.39136960E-02

Integral of selection coefficient over zone 1= 1.40749034E-04

Mean load statistics for zone 1

Mean q1 and q2= 0.600000024

F1 and F2= 0.996677756 0.970873833

Diversities= 1.59467699E-03 1.39805600E-02

Contributions to loads within In and St= 8.43484231E-05 8.35639366E-05

Contribution to load between In and St = 5.40476285E-05

Contributions to homozygous loads for In and St= 8.44494207E-05 8.44494207E-05

Contributions to inbreeding loads for In and St= 0.00000000 0.00000000

Contributions to selection coefficients for In and St homokaryotypes

3.02791595E-05 2.95042992E-05

Contributions to mean A2 freqs= 2.63482183E-02 2.63482183E-02

Contributions to mean diversities= 7.00281598E-05 6.13938086E-04

Contributions to mean freqs. of seg. site= 2.48237309E-04 2.16380460E-03

Delta-theta values= -8.23259354E-04 -6.60312176E-03

Zone 2a: moderate selection; cut-off at moderate gamma for St population

Lower and upper bounds of St popn gamma

0.250000000 250.000000

Probability of zone 2a= 0.303239673

Coefficients for bivariate distribution of q1 and q2

a1= 5.00000035E-03 a2= 4.49999981E-02

b11= 4.50000027E-03 b12= 8.09999928E-02 b22= 0.364499956

Net probability of zone 2a using Simpsons rule= 0.303576410

Contributions to mean load statistics over zone 2a

Contributions to mean loads within In and St= 0.144798964 1.07835303E-03

Contribution to load between In and St= 8.12752452E-03

Contributions to homozygous loads for In and St= 0.145504758 1.95283501E-03

Contributions to inbreeding loads= 7.05582264E-04 8.74482619E-04

Selection coefficients for In and St homokaryotypes

0.127743244 -7.07411766E-03

Selection coefficients for In and St homokaryotypes

0.127743244 -7.07411766E-03

Contributions to mean A2 freqs= 0.105992854 1.72770899E-02

Contributions to mean diversities= 4.80526563E-04 1.79444032E-03

Contributions to mean proportions of seg. sites= 1.73632009E-03 8.06842279E-03

Contributions to delta-theta values= 1.81630254E-02 0.210972548

Zone 2b: moderate selection; gamma for St reaches high value

Lower and upper bounds of St popn gamma

250.000000 2500.00000

Probability of zone 2b 0.317252636

Net probability of zone 2b using Simpsons rule= 0.317252696

Contributions to mean load statistics over zone 2b

Contributions to mean loads within In and St= 2.60605314E-03 1.19566357E-04

Contribution to load between In and St= 2.86194641E-04

Contributions to homozygous loads for In and St= 4.62485664E-03 1.07231678E-03

Contributions to inbreeding loads= 2.01879605E-03 9.52750503E-04

Selection coefficients for In and St homokaryotypes

2.31719017E-03 -1.66654587E-04

Selection coefficients for In and St homokaryotypes

2.31719017E-03 -1.66654587E-04

Contributions to mean A2 freqs= 1.97981601E-04 3.32943637E-05

Contributions to mean diversities= 1.16837240E-04 6.55114636E-05

Contributions to mean proportions of seg. sites= 6.47450215E-04 5.81898668E-04

Contributions to delta-theta values= 0.359783769 0.600587487

Zone 3: strong selection approximation

Lower and upper bounds of St popn gamma= 2500.00000 30000.0020

Zone 3: strong selection approximation

Probability of zone 3= 0.323736906

Mean load statistics over zone 3

Contributions to loads within In and St= 4.03293321E-04 3.58825666E-04

Contribution to load between In and St= 3.53267271E-04

Contributions to homozygous loads for In and St= 3.53167788E-03 3.53167788E-03

Contributions to inbreeding loads= 3.12838401E-03 3.17284628E-03

Selection coefficients for In and St homokaryotypes

5.00082970E-05 5.54323196E-06

Contributions to mean A2 freqs= 1.11169065E-05 1.11169065E-05

Contributions to mean A2 freqs at seg. sites= 0.00000000 0.00000000

Contributions to mean diversities= 2.17678826E-05 2.21812516E-05

Contributions to delta-theta values= 0.566199541 0.638060391

Mean load statistics over all zones

Loads within In and St= 0.147892669 1.64030900E-03

Load between In and St= 8.82103387E-03

Homozygous load for In and St= 0.153745741 6.64127897E-03

Inbreeding loads= 5.85286319E-03 5.00096474E-03

Selection coefficients for In and St homokaryotypes

0.129834294 -7.20655918E-03

Mean frequencies of A2 in In and St= 0.132550165 4.36697192E-02

Ratio of these= 3.03528786

Mean diversities at selected sites in In and St= 6.89159788E-04 2.49607116E-03

Mean diversities at neutral sites in In and St= 1.59467699E-03 1.39805600E-02

pi-n/pi-s for In and St= 0.432162613 0.178538710

Ratio of these= 2.42055416

Mean freqs. of seg. sites= 2.81003118E-03 1.10315466E-02

Ratio of these= 0.254726857

Overall delta-theta values= 0.129917264 0.197264791

Ratio of these= 0.658593297

**h = 0.15**

Zone 1: quasi-neutral zone

Upper bound scaled selection coefficient for neutrality in St metapopulation= 0.250000000

Probability of zone 1= 4.39136960E-02

Integral of selection coefficient over zone 1= 1.40749034E-04

Mean load statistics for zone 1

Mean q1 and q2= 0.600000024

F1 and F2= 0.996677756 0.970873833

Diversities= 1.59467699E-03 1.39805600E-02

Contributions to loads within In and St= 8.43708694E-05 8.37607149E-05

Contribution to load between In and St = 6.08035843E-05

Contributions to homozygous loads for In and St= 8.44494207E-05 8.44494207E-05

Contributions to inbreeding loads for In and St= 8.97294655E-03 8.99694674E-03

Contributions to selection coefficients for In and St homokaryotypes

2.35438347E-05 2.29477882E-05

Contributions to mean A2 freqs= 2.63482183E-02 2.63482183E-02

Contributions to mean diversities= 7.00281598E-05 6.13938086E-04

Contributions to mean freqs. of seg. site= 2.48237309E-04 2.16380460E-03

Delta-theta values= -8.23259354E-04 -6.60312176E-03

Zone 2a: moderate selection; cut-off at moderate gamma for St population

Lower and upper bounds of St popn gamma

0.250000000 416.666656

Probability of zone 2a= 0.359450161

Coefficients for bivariate distribution of q1 and q2

a1= 1.50000006E-02 a2= 0.135000005

b11= 3.50000011E-03 b12= 6.30000010E-02 b22= 0.283499986

Net probability of zone 2a using Simpsons rule= 0.360651016

Contributions to mean load statistics over zone 2a

Contributions to mean loads within In and St= 6.24625124E-02 1.16809004E-03

Contribution to load between In and St= 1.01799266E-02

Contributions to homozygous loads for In and St= 6.29261360E-02 1.66823203E-03

Contributions to inbreeding loads= 4.63562319E-04 5.00139431E-04

Selection coefficients for In and St homokaryotypes

5.09393811E-02 -9.05251503E-03

Selection coefficients for In and St homokaryotypes

5.09393811E-02 -9.05251503E-03

Contributions to mean A2 freqs= 8.30528885E-02 1.77091360E-02

Contributions to mean diversities= 4.23798250E-04 1.56474730E-03

Contributions to mean proportions of seg. sites= 1.64529064E-03 6.98010018E-03

Contributions to delta-theta values= 8.61639977E-02 0.204693913

Zone 2b: moderate selection; gamma for St reaches high value

Lower and upper bounds of St popn gamma

416.666656 4166.66650

Probability of zone 2b 0.348782212

Net probability of zone 2b using Simpsons rule= 0.348782301

Contributions to mean load statistics over zone 2b

Contributions to mean loads within In and St= 2.78607535E-04 8.96285637E-05

Contribution to load between In and St= 1.74651199E-04

Contributions to homozygous loads for In and St= 8.68413132E-04 2.95758218E-04

Contributions to inbreeding loads= 5.89806354E-04 2.06129567E-04

Selection coefficients for In and St homokaryotypes

1.03950500E-04 -8.49962234E-05

Selection coefficients for In and St homokaryotypes

1.03950500E-04 -8.49962234E-05

Contributions to mean A2 freqs= 1.51333370E-05 6.32059300E-06

Contributions to mean diversities= 2.90303342E-05 1.25738234E-05

Contributions to mean proportions of seg. sites= 2.26681776E-04 1.25032893E-04

Contributions to delta-theta values= 0.545653462 0.643224657

Zone 3: strong selection approximation

Lower and upper bounds of St popn gamma= 4166.66650 30000.0020

Zone 3: strong selection approximation

Probability of zone 3= 0.235996842

Mean load statistics over zone 3

Contributions to loads within In and St= 2.76434963E-04 2.74328486E-04

Contribution to load between In and St= 2.74065358E-04

Contributions to homozygous loads for In and St= 9.13536234E-04 9.13536234E-04

Contributions to inbreeding loads= 6.37100369E-04 6.39206788E-04

Selection coefficients for In and St homokaryotypes

2.38418579E-06 2.38418579E-07

Contributions to mean A2 freqs= 2.03110994E-06 2.03110972E-06

Contributions to mean A2 freqs at seg. sites= 0.00000000 0.00000000

Contributions to mean diversities= 4.04389766E-06 4.06015624E-06

Contributions to delta-theta values= 0.631019473 0.643712282

Mean load statistics over all zones

Loads within In and St= 6.31019250E-02 1.61580788E-03

Load between In and St= 1.06894458E-02

Homozygous load for In and St= 6.47925362E-02 2.96197576E-03

Inbreeding loads= 1.69054768E-03 1.34616450E-03

Selection coefficients for In and St homokaryotypes

5.10626435E-02 -9.11498070E-03

Mean frequencies of A2 in In and St= 0.109418273 4.40657064E-02

Ratio of these= 2.48307085

Mean diversities at selected sites in In and St= 5.26900636E-04 2.19531916E-03

Mean diversities at neutral sites in In and St= 1.59467699E-03 1.39805600E-02

pi-n/pi-s for In and St= 0.330412149 0.157026559

Ratio of these= 2.10418010

Mean freqs. of seg. sites= 2.15909164E-03 9.30936635E-03

Ratio of these= 0.231926814

Overall delta-theta values= 0.134216249 0.163377941

Ratio of these= 0.821507752

**h = 0.25**

Zone 1: quasi-neutral zone

Upper bound scaled selection coefficient for neutrality in St metapopulation= 0.250000000

Probability of zone 1= 4.39136960E-02

Integral of selection coefficient over zone 1= 1.40749034E-04

Mean load statistics for zone 1

Mean q1 and q2= 0.600000024

F1 and F2= 0.996677756 0.970873833

Diversities= 1.59467699E-03 1.39805600E-02

Contributions to loads within In and St= 8.43933085E-05 8.39574859E-05

Contribution to load between In and St = 6.75595365E-05

Contributions to homozygous loads for In and St= 8.44494207E-05 8.44494207E-05

Contributions to inbreeding loads for In and St= 2.33099516E-03 2.33306922E-03

Contributions to selection coefficients for In and St homokaryotypes

1.68085098E-05 1.63912773E-05

Contributions to mean A2 freqs= 2.63482183E-02 2.63482183E-02

Contributions to mean diversities= 7.00281598E-05 6.13938086E-04

Contributions to mean freqs. of seg. site= 2.48237309E-04 2.16380460E-03

Delta-theta values= -8.23259354E-04 -6.60312176E-03

Zone 2a: moderate selection; cut-off at moderate gamma for St population

Lower and upper bounds of St popn gamma

0.250000000 416.666656

Probability of zone 2a= 0.359450161

Coefficients for bivariate distribution of q1 and q2

a1= 2.50000004E-02 a2= 0.224999994

b11= 2.50000018E-03 b12= 4.49999981E-02 b22= 0.202499986

Net probability of zone 2a using Simpsons rule= 0.360651016

Contributions to mean load statistics over zone 2a

Contributions to mean loads within In and St= 3.75116058E-02 1.19144039E-03

Contribution to load between In and St= 1.01157073E-02

Contributions to homozygous loads for In and St= 3.77420783E-02 1.44041365E-03

Contributions to inbreeding loads= 2.30456062E-04 2.48971890E-04

Selection coefficients for In and St homokaryotypes

2.70240307E-02 -8.96418095E-03

Selection coefficients for In and St homokaryotypes

2.70240307E-02 -8.96418095E-03

Contributions to mean A2 freqs= 7.10945129E-02 1.76453646E-02

Contributions to mean diversities= 3.69848422E-04 1.38664292E-03

Contributions to mean proportions of seg. sites= 1.47155253E-03 6.13316381E-03

Contributions to delta-theta values= 0.108339012 0.197893918

Zone 2b: moderate selection; gamma for St reaches high value

Lower and upper bounds of St popn gamma

416.666656 4166.66650

Probability of zone 2b 0.348782212

Net probability of zone 2b using Simpsons rule= 0.348782301

Contributions to mean load statistics over zone 2b

Contributions to mean loads within In and St= 2.60749890E-04 7.98195397E-05

Contribution to load between In and St= 1.67836886E-04

Contributions to homozygous loads for In and St= 5.12157683E-04 1.59165837E-04

Contributions to inbreeding loads= 2.51408143E-04 7.93462023E-05

Selection coefficients for In and St homokaryotypes

9.29236412E-05 -8.79764557E-05

Selection coefficients for In and St homokaryotypes

9.29236412E-05 -8.79764557E-05

Contributions to mean A2 freqs= 8.95962967E-06 3.53542873E-06

Contributions to mean diversities= 1.74721117E-05 7.04574768E-06

Contributions to mean proportions of seg. sites= 1.48217485E-04 7.33559646E-05

Contributions to delta-theta values= 0.581786811 0.659244061

Zone 3: strong selection approximation

Lower and upper bounds of St popn gamma= 4166.66650 30000.0020

Zone 3: strong selection approximation

Probability of zone 3= 0.235996842

Mean load statistics over zone 3

Contributions to loads within In and St= 2.74671271E-04 2.74129619E-04

Contribution to load between In and St= 2.74061837E-04

Contributions to homozygous loads for In and St= 5.48121461E-04 5.48121403E-04

Contributions to inbreeding loads= 2.73450016E-04 2.73991580E-04

Selection coefficients for In and St homokaryotypes

5.96046448E-07 5.96046448E-08

Contributions to mean A2 freqs= 1.21866731E-06 1.21866719E-06

Contributions to mean A2 freqs at seg. sites= 0.00000000 0.00000000

Contributions to mean diversities= 2.43073578E-06 2.43658837E-06

Contributions to delta-theta values= 0.636855721 0.644324601

Mean load statistics over all zones

Loads within In and St= 3.81314233E-02 1.62934698E-03

Load between In and St= 1.06251659E-02

Homozygous load for In and St= 3.88868041E-02 2.23215017E-03

Inbreeding loads= 7.55370362E-04 6.02801621E-04

Selection coefficients for In and St homokaryotypes

2.71313787E-02 -9.03642178E-03

Mean frequencies of A2 in In and St= 9.74529162E-02 4.39983383E-02

Ratio of these= 2.21492267

Mean diversities at selected sites in In and St= 4.59779403E-04 2.01006327E-03

Mean diversities at neutral sites in In and St= 1.59467699E-03 1.39805600E-02

pi-n/pi-s for In and St= 0.288321346 0.143775597

Ratio of these= 2.00535655

Mean freqs. of seg. sites= 1.89175433E-03 8.39462783E-03

Ratio of these= 0.225352973

Overall delta-theta values= 0.137743413 0.150506616

Ratio of these= 0.915198386

**h = 0.35**

Zone 1: quasi-neutral zone

Upper bound scaled selection coefficient for neutrality in St metapopulation= 0.250000000

Probability of zone 1= 4.39136960E-02

Integral of selection coefficient over zone 1= 1.40749034E-04

Mean load statistics for zone 1

Mean q1 and q2= 0.600000024

F1 and F2= 0.996677756 0.970873833

Diversities= 1.59467699E-03 1.39805600E-02

Contributions to loads within In and St= 8.44157548E-05 8.41542642E-05

Contribution to load between In and St = 7.43154887E-05

Contributions to homozygous loads for In and St= 8.44494207E-05 8.44494207E-05

Contributions to inbreeding loads for In and St= 9.99398762E-04 9.99932061E-04

Contributions to selection coefficients for In and St homokaryotypes

1.00731850E-05 9.83476639E-06

Contributions to mean A2 freqs= 2.63482183E-02 2.63482183E-02

Contributions to mean diversities= 7.00281598E-05 6.13938086E-04

Contributions to mean freqs. of seg. site= 2.48237309E-04 2.16380460E-03

Delta-theta values= -8.23259354E-04 -6.60312176E-03

Zone 2a: moderate selection; cut-off at moderate gamma for St population

Lower and upper bounds of St popn gamma

0.250000000 297.619049

Probability of zone 2a= 0.321548969

Coefficients for bivariate distribution of q1 and q2

a1= 3.50000001E-02 a2= 0.314999998

b11= 1.50000013E-03 b12= 2.70000007E-02 b22= 0.121499993

Net probability of zone 2a using Simpsons rule= 0.322079927

Contributions to mean load statistics over zone 2a

Contributions to mean loads within In and St= 2.60416325E-02 1.18368689E-03

Contribution to load between In and St= 9.77961440E-03

Contributions to homozygous loads for In and St= 2.61329133E-02 1.28853542E-03

Contributions to inbreeding loads= 9.12812611E-05 1.04850311E-04

Selection coefficients for In and St homokaryotypes

1.61305070E-02 -8.63301754E-03

Selection coefficients for In and St homokaryotypes

1.61305070E-02 -8.63301754E-03

Contributions to mean A2 freqs= 6.30173460E-02 1.71921216E-02

Contributions to mean diversities= 3.28298425E-04 1.24082447E-03

Contributions to mean proportions of seg. sites= 1.30812416E-03 5.46321692E-03

Contributions to delta-theta values= 0.109627843 0.194225252

Zone 2b: moderate selection; gamma for St reaches high value

Lower and upper bounds of St popn gamma

297.619049 2976.19043

Probability of zone 2b 0.328705907

Net probability of zone 2b using Simpsons rule= 0.328705996

Contributions to mean load statistics over zone 2b

Contributions to mean loads within In and St= 2.61239009E-04 9.07608774E-05

Contribution to load between In and St= 1.74957808E-04

Contributions to homozygous loads for In and St= 3.70374328E-04 1.29496257E-04

Contributions to inbreeding loads= 1.09135326E-04 3.87353975E-05

Selection coefficients for In and St homokaryotypes

8.62479210E-05 -8.41617584E-05

Selection coefficients for In and St homokaryotypes

8.62479210E-05 -8.41617584E-05

Contributions to mean A2 freqs= 8.79437721E-06 3.91493495E-06

Contributions to mean diversities= 1.71545253E-05 7.80227674E-06

Contributions to mean proportions of seg. sites= 1.45696555E-04 8.14365267E-05

Contributions to delta-theta values= 0.582283914 0.660097897

Zone 3: strong selection approximation

Lower and upper bounds of St popn gamma= 2976.19043 30000.0020

Zone 3: strong selection approximation

Probability of zone 3= 0.293974340

Mean load statistics over zone 3

Contributions to loads within In and St= 3.26630543E-04 3.26381793E-04

Contribution to load between In and St= 3.26350710E-04

Contributions to homozygous loads for In and St= 4.66214406E-04 4.66214406E-04

Contributions to inbreeding loads= 1.39584299E-04 1.39832729E-04

Selection coefficients for In and St homokaryotypes

2.98023224E-07 5.96046448E-08

Contributions to mean A2 freqs= 1.30430715E-06 1.30430715E-06

Contributions to mean A2 freqs at seg. sites= 0.00000000 0.00000000

Contributions to mean diversities= 2.60189495E-06 2.60785600E-06

Contributions to delta-theta values= 0.637255788 0.644360304

Mean load statistics over all zones

Loads within In and St= 2.67139170E-02 1.68498384E-03

Load between In and St= 1.03552388E-02

Homozygous load for In and St= 2.70539504E-02 1.96869555E-03

Inbreeding loads= 3.40034545E-04 2.83713598E-04

Selection coefficients for In and St homokaryotypes

1.62255764E-02 -8.70800018E-03

Mean frequencies of A2 in In and St= 8.93756598E-02 4.35455590E-02

Ratio of these= 2.05246329

Mean diversities at selected sites in In and St= 4.18082986E-04 1.86517264E-03

Mean diversities at neutral sites in In and St= 1.59467699E-03 1.39805600E-02

pi-n/pi-s for In and St= 0.262174100 0.133411869

Ratio of these= 1.96514821

Mean freqs. of seg. sites= 1.72750524E-03 7.73447333E-03

Ratio of these= 0.223351374

Overall delta-theta values= 0.141392112 0.144460559

Ratio of these= 0.978759289

**h = 0.45**

Zone 1: quasi-neutral zone

Upper bound scaled selection coefficient for neutrality in St metapopulation= 0.250000000

Probability of zone 1= 4.39136960E-02

Integral of selection coefficient over zone 1= 1.40749034E-04

Mean load statistics for zone 1

Mean q1 and q2= 0.600000024

F1 and F2= 0.996677756 0.970873833

Diversities= 1.59467699E-03 1.39805600E-02

Contributions to loads within In and St= 8.44382012E-05 8.43510352E-05

Contribution to load between In and St = 8.10714409E-05

Contributions to homozygous loads for In and St= 8.44494207E-05 8.44494207E-05

Contributions to inbreeding loads for In and St= 4.28387430E-04 4.28550673E-04

Contributions to selection coefficients for In and St homokaryotypes

3.33786011E-06 3.27825546E-06

Contributions to mean A2 freqs= 2.63482183E-02 2.63482183E-02

Contributions to mean diversities= 7.00281598E-05 6.13938086E-04

Contributions to mean freqs. of seg. site= 2.48237309E-04 2.16380460E-03

Delta-theta values= -8.23259354E-04 -6.60312176E-03

Zone 2a: moderate selection; cut-off at moderate gamma for St population

Lower and upper bounds of St popn gamma

0.250000000 165.343918

Probability of zone 2a= 0.263242185

Coefficients for bivariate distribution of q1 and q2

a1= 4.49999981E-02 a2= 0.404999971

b11= 5.00000140E-04 b12= 9.00000241E-03 b22= 4.05000076E-02

Net probability of zone 2a using Simpsons rule= 0.263346374

Contributions to mean load statistics over zone 2a

Contributions to mean loads within In and St= 1.95781812E-02 1.15600158E-03

Contribution to load between In and St= 9.40879714E-03

Contributions to homozygous loads for In and St= 1.95969064E-02 1.17990945E-03

Contributions to inbreeding loads= 1.87308779E-05 2.39077563E-05

Selection coefficients for In and St homokaryotypes

1.01178288E-02 -8.28695297E-03

Selection coefficients for In and St homokaryotypes

1.01178288E-02 -8.28695297E-03

Contributions to mean A2 freqs= 5.72704971E-02 1.69101860E-02

Contributions to mean diversities= 2.92938406E-04 1.12340390E-03

Contributions to mean proportions of seg. sites= 1.14881189E-03 4.92153224E-03

Contributions to delta-theta values= 9.53530073E-02 0.190182149

Zone 2b: moderate selection; gamma for St reaches high value

Lower and upper bounds of St popn gamma

165.343918 1653.43921

Probability of zone 2b 0.288836628

Net probability of zone 2b using Simpsons rule= 0.288836628

Contributions to mean load statistics over zone 2b

Contributions to mean loads within In and St= 2.55217048E-04 1.16025105E-04

Contribution to load between In and St= 1.85258381E-04

Contributions to homozygous loads for In and St= 2.82817666E-04 1.28864587E-04

Contributions to inbreeding loads= 2.76006922E-05 1.28394904E-05

Selection coefficients for In and St homokaryotypes

6.99758530E-05 -6.92605972E-05

Selection coefficients for In and St homokaryotypes

6.99758530E-05 -6.92605972E-05

Contributions to mean A2 freqs= 1.15942375E-05 6.47513889E-06

Contributions to mean diversities= 2.23947063E-05 1.28911388E-05

Contributions to mean proportions of seg. sites= 1.80585019E-04 1.30582921E-04

Contributions to delta-theta values= 0.560037732 0.649767339

Zone 3: strong selection approximation

Lower and upper bounds of St popn gamma= 1653.43921 30000.0020

Zone 3: strong selection approximation

Probability of zone 3= 0.392150402

Mean load statistics over zone 3

Contributions to loads within In and St= 4.15130722E-04 4.15035989E-04

Contribution to load between In and St= 4.15024464E-04

Contributions to homozygous loads for In and St= 4.61137737E-04 4.61137650E-04

Contributions to inbreeding loads= 4.60072733E-05 4.61017626E-05

Selection coefficients for In and St homokaryotypes

1.19209290E-07 0.00000000

Contributions to mean A2 freqs= 1.91432878E-06 1.91432878E-06

Contributions to mean A2 freqs at seg. sites= 0.00000000 0.00000000

Contributions to mean diversities= 3.81587597E-06 3.82721146E-06

Contributions to delta-theta values= 0.634809017 0.644107223

Mean load statistics over all zones

Loads within In and St= 2.03329679E-02 1.77141372E-03

Load between In and St= 1.00901518E-02

Homozygous load for In and St= 2.04253104E-02 1.85436103E-03

Inbreeding loads= 9.23500629E-05 8.29474011E-05

Selection coefficients for In and St homokaryotypes

1.01905465E-02 -8.35347176E-03

Mean frequencies of A2 in In and St= 8.36322233E-02 4.32667956E-02

Ratio of these= 1.93294239

Mean diversities at selected sites in In and St= 3.89177119E-04 1.75406027E-03

Mean diversities at neutral sites in In and St= 1.59467699E-03 1.39805600E-02

pi-n/pi-s for In and St= 0.244047612 0.125464231

Ratio of these= 1.94515693

Mean freqs. of seg. sites= 1.61470438E-03 7.25407153E-03

Ratio of these= 0.222592846

Overall delta-theta values= 0.144921422 0.142143905

Ratio of these= 1.01954019

**h = 0.5**

Zone 1: quasi-neutral zone

Upper bound scaled selection coefficient for neutrality in St metapopulation= 0.250000000

Probability of zone 1= 4.39136960E-02

Integral of selection coefficient over zone 1= 1.40749034E-04

Mean load statistics for zone 1

Mean q1 and q2= 0.600000024

F1 and F2= 0.996677756 0.970873833

Diversities= 1.59467699E-03 1.39805600E-02

Contributions to loads within In and St= 8.44494207E-05 8.44494207E-05

Contribution to load between In and St = 8.44494207E-05

Contributions to homozygous loads for In and St= 8.44494207E-05 8.44494207E-05

Contributions to inbreeding loads for In and St= 1.11074034E-04 1.11107001E-04

Contributions to selection coefficients for In and St homokaryotypes

0.00000000 0.00000000

Contributions to mean A2 freqs= 2.63482183E-02 2.63482183E-02

Contributions to mean diversities= 7.00281598E-05 6.13938086E-04

Contributions to mean freqs. of seg. site= 2.48237309E-04 2.16380460E-03

Delta-theta values= -8.23259354E-04 -6.60312176E-03

Zone 2a: moderate selection; cut-off at moderate gamma for St population

Lower and upper bounds of St popn gamma

0.250000000 82.6719589

Probability of zone 2a= 0.205970049

Coefficients for bivariate distribution of q1 and q2

a1= 5.00000007E-02 a2= 0.449999988

b11= 0.00000000 b12= 0.00000000 b22= 0.00000000

Net probability of zone 2a using Simpsons rule= 0.205981016

Contributions to mean load statistics over zone 2a

Contributions to mean loads within In and St= 1.72838997E-02 1.11394655E-03

Contribution to load between In and St= 9.19892080E-03

Contributions to homozygous loads for In and St= 1.72838997E-02 1.11394655E-03

Contributions to inbreeding loads= 0.00000000 0.00000000

Selection coefficients for In and St homokaryotypes

8.05240870E-03 -8.11779499E-03

Selection coefficients for In and St homokaryotypes

8.05240870E-03 -8.11779499E-03

Contributions to mean A2 freqs= 5.49828373E-02 1.68425385E-02

Contributions to mean diversities= 2.65979877E-04 1.06400473E-03

Contributions to mean proportions of seg. sites= 1.00775750E-03 4.59504407E-03

Contributions to delta-theta values= 6.36364818E-02 0.178503633

Zone 2b: moderate selection; gamma for St reaches high value

Lower and upper bounds of St popn gamma

82.6719589 826.719604

Probability of zone 2b 0.241704941

Net probability of zone 2b using Simpsons rule= 0.241705000

Contributions to mean load statistics over zone 2b

Contributions to mean loads within In and St= 2.41919304E-04 1.41345969E-04

Contribution to load between In and St= 1.91632658E-04

Contributions to homozygous loads for In and St= 2.41919304E-04 1.41345969E-04

Contributions to inbreeding loads= 0.00000000 0.00000000

Selection coefficients for In and St homokaryotypes

5.03063202E-05 -5.03063202E-05

Selection coefficients for In and St homokaryotypes

5.03063202E-05 -5.03063202E-05

Contributions to mean A2 freqs= 2.00515260E-05 1.28585007E-05

Contributions to mean diversities= 3.53627474E-05 2.55247924E-05

Contributions to mean proportions of seg. sites= 2.53451290E-04 2.46523530E-04

Contributions to delta-theta values= 0.505002201 0.632670641

Zone 3: strong selection approximation

Lower and upper bounds of St popn gamma= 826.719604 30000.0020

Zone 3: strong selection approximation

Probability of zone 3= 0.496554226

Mean load statistics over zone 3

Contributions to loads within In and St= 5.10625541E-04 5.10625541E-04

Contribution to load between In and St= 5.10625541E-04

Contributions to homozygous loads for In and St= 5.10625541E-04 5.10625541E-04

Contributions to inbreeding loads= 0.00000000 0.00000000

Selection coefficients for In and St homokaryotypes

0.00000000 0.00000000

Contributions to mean A2 freqs= 3.34678498E-06 3.34678498E-06

Contributions to mean A2 freqs at seg. sites= 0.00000000 0.00000000

Contributions to mean diversities= 6.65635025E-06 6.68936445E-06

Contributions to delta-theta values= 0.627378941 0.643340349

Mean load statistics over all zones

Loads within In and St= 1.81208923E-02 1.85036752E-03

Load between In and St= 9.98562854E-03

Homozygous load for In and St= 1.81208923E-02 1.85036752E-03

Inbreeding loads= 0.00000000 0.00000000

Selection coefficients for In and St homokaryotypes

8.10223818E-03 -8.16845894E-03

Mean frequencies of A2 in In and St= 8.13544542E-02 4.32069600E-02

Ratio of these= 1.88290155

Mean diversities at selected sites in In and St= 3.78027122E-04 1.71015691E-03

Mean diversities at neutral sites in In and St= 1.59467699E-03 1.39805600E-02

pi-n/pi-s for In and St= 0.237055615 0.122323923

Ratio of these= 1.93793344

Mean freqs. of seg. sites= 1.57282141E-03 7.07191229E-03

Ratio of these= 0.222403973

Overall delta-theta values= 0.147301912 0.142072022

Ratio of these= 1.03681159

**x = 0.3**

**h = 0.05**

Zone 1: quasi-neutral zone

Upper bound scaled selection coefficient for neutrality in St metapopulation= 0.250000000

Probability of zone 1= 4.73524816E-02

Integral of selection coefficient over zone 1= 1.95133864E-04

Mean load statistics for zone 1

Mean q1 and q2= 0.600000024

F1 and F2= 0.990099013 0.977198601

Diversities= 4.75247391E-03 1.09446710E-02

Contributions to loads within In and St= 1.16663010E-04 1.16119263E-04

Contribution to load between In and St = 7.49314058E-05

Contributions to homozygous loads for In and St= 1.17080323E-04 1.17080323E-04

Contributions to inbreeding loads for In and St= 0.00000000 0.00000000

Contributions to selection coefficients for In and St homokaryotypes

4.17232513E-05 4.11868095E-05

Contributions to mean A2 freqs= 2.84114908E-02 2.84114908E-02

Contributions to mean diversities= 2.25041440E-04 5.18257322E-04

Contributions to mean freqs. of seg. site= 7.96483480E-04 1.82903488E-03

Delta-theta values= -2.39169598E-03 -5.25259972E-03

Zone 2a: moderate selection; cut-off at moderate gamma for St population

Lower and upper bounds of St popn gamma

0.250000000 250.000000

Probability of zone 2a= 0.326476455

Coefficients for bivariate distribution of q1 and q2

a1= 1.50000006E-02 a2= 3.50000001E-02

b11= 4.05000001E-02 b12= 0.189000010 b22= 0.220499992

Net probability of zone 2a using Simpsons rule= 0.326839477

Contributions to mean load statistics over zone 2a

Contributions to mean loads within In and St= 1.47714987E-02 1.55137677E-03

Contribution to load between In and St= 1.57297438E-03

Contributions to homozygous loads for In and St= 1.58853680E-02 2.62762606E-03

Contributions to inbreeding loads= 1.11386389E-03 1.07624696E-03

Selection coefficients for In and St homokaryotypes

1.31118298E-02 -2.15768814E-05

Selection coefficients for In and St homokaryotypes

1.31118298E-02 -2.15768814E-05

Contributions to mean A2 freqs= 4.82889153E-02 1.87343676E-02

Contributions to mean diversities= 1.09254161E-03 1.57900981E-03

Contributions to mean proportions of seg. sites= 4.34190175E-03 7.13270577E-03

Contributions to delta-theta values= 0.107291400 0.214615524

Zone 2b: moderate selection; gamma for St reaches high value

Lower and upper bounds of St popn gamma

250.000000 2500.00000

Probability of zone 2b 0.333575726

Net probability of zone 2b using Simpsons rule= 0.333575726

Contributions to mean load statistics over zone 2b

Contributions to mean loads within In and St= 2.17786597E-04 1.28400323E-04

Contribution to load between In and St= 1.43423618E-04

Contributions to homozygous loads for In and St= 1.71851635E-03 1.14420999E-03

Contributions to inbreeding loads= 1.50072901E-03 1.01580983E-03

Selection coefficients for In and St homokaryotypes

7.43865967E-05 -1.50203705E-05

Selection coefficients for In and St homokaryotypes

7.43865967E-05 -1.50203705E-05

Contributions to mean A2 freqs= 3.92737456E-05 2.80397235E-05

Contributions to mean diversities= 7.55018045E-05 5.51147641E-05

Contributions to mean proportions of seg. sites= 5.87588351E-04 4.87001846E-04

Contributions to delta-theta values= 0.544135332 0.598496735

Zone 3: strong selection approximation

Lower and upper bounds of St popn gamma= 2500.00000 23333.3340

Zone 3: strong selection approximation

Probability of zone 3= 0.280738235

Mean load statistics over zone 3

Contributions to loads within In and St= 3.27024231E-04 3.19868792E-04

Contribution to load between In and St= 3.14501900E-04

Contributions to homozygous loads for In and St= 3.14426585E-03 3.14426585E-03

Contributions to inbreeding loads= 2.81724404E-03 2.82440125E-03

Selection coefficients for In and St homokaryotypes

1.25169754E-05 5.36441803E-06

Contributions to mean A2 freqs= 8.34837829E-06 8.34837829E-06

Contributions to mean A2 freqs at seg. sites= 0.00000000 0.00000000

Contributions to mean diversities= 1.66024147E-05 1.66559876E-05

Contributions to delta-theta values= 0.627306700 0.637799382

Mean load statistics over all zones

Loads within In and St= 1.54329734E-02 2.11576512E-03

Load between In and St= 2.10583117E-03

Homozygous load for In and St= 2.08652299E-02 7.03318231E-03

Inbreeding loads= 5.43225463E-03 4.91741905E-03

Selection coefficients for In and St homokaryotypes

1.32387280E-02 9.95397568E-06

Mean frequencies of A2 in In and St= 7.67480284E-02 4.71822470E-02

Ratio of these= 1.62662935

Mean diversities at selected sites in In and St= 1.40968733E-03 2.16903794E-03

Mean diversities at neutral sites in In and St= 4.75247391E-03 1.09446710E-02

pi-n/pi-s for In and St= 0.296621799 0.198182106

Ratio of these= 1.49671328

Mean freqs. of seg. sites= 5.88401500E-03 9.61188693E-03

Ratio of these= 0.612160265

Overall delta-theta values= 0.150035501 0.199409842

Ratio of these= 0.752397656

**h = 0.15**

Zone 1: quasi-neutral zone

Upper bound scaled selection coefficient for neutrality in St metapopulation= 0.250000000

Probability of zone 1= 4.73524816E-02

Integral of selection coefficient over zone 1= 1.95133864E-04

Mean load statistics for zone 1

Mean q1 and q2= 0.600000024

F1 and F2= 0.990099013 0.977198601

Diversities= 4.75247391E-03 1.09446710E-02

Contributions to loads within In and St= 1.16755742E-04 1.16332827E-04

Contribution to load between In and St = 8.42978334E-05

Contributions to homozygous loads for In and St= 1.17080323E-04 1.17080323E-04

Contributions to inbreeding loads for In and St= 8.99094623E-03 8.99608899E-03

Contributions to selection coefficients for In and St homokaryotypes

3.24845314E-05 3.20076942E-05

Contributions to mean A2 freqs= 2.84114908E-02 2.84114908E-02

Contributions to mean diversities= 2.25041440E-04 5.18257322E-04

Contributions to mean freqs. of seg. site= 7.96483480E-04 1.82903488E-03

Delta-theta values= -2.39169598E-03 -5.25259972E-03

Zone 2a: moderate selection; cut-off at moderate gamma for St population

Lower and upper bounds of St popn gamma

0.250000000 416.666656

Probability of zone 2a= 0.386616915

Coefficients for bivariate distribution of q1 and q2

a1= 4.50000018E-02 a2= 0.105000004

b11= 3.15000005E-02 b12= 0.147000000 b22= 0.171499997

Net probability of zone 2a using Simpsons rule= 0.387911767

Contributions to mean load statistics over zone 2a

Contributions to mean loads within In and St= 9.85228736E-03 1.65368058E-03

Contribution to load between In and St= 2.38143583E-03

Contributions to homozygous loads for In and St= 1.04381656E-02 2.21359613E-03

Contributions to inbreeding loads= 5.85876347E-04 5.59911365E-04

Selection coefficients for In and St homokaryotypes

7.44301081E-03 -7.28011131E-04

Selection coefficients for In and St homokaryotypes

7.44301081E-03 -7.28011131E-04

Contributions to mean A2 freqs= 4.22943532E-02 1.92890000E-02

Contributions to mean diversities= 8.76445032E-04 1.34401897E-03

Contributions to mean proportions of seg. sites= 3.64777166E-03 5.97947463E-03

Contributions to delta-theta values= 0.147589505 0.202567160

Zone 2b: moderate selection; gamma for St reaches high value

Lower and upper bounds of St popn gamma

416.666656 4166.66650

Probability of zone 2b 0.360976309

Net probability of zone 2b using Simpsons rule= 0.360976666

Contributions to mean load statistics over zone 2b

Contributions to mean loads within In and St= 1.60868527E-04 9.23142070E-05

Contribution to load between In and St= 1.24509766E-04

Contributions to homozygous loads for In and St= 5.25389158E-04 3.04581248E-04

Contributions to inbreeding loads= 3.64520471E-04 2.12266867E-04

Selection coefficients for In and St homokaryotypes

3.63588333E-05 -3.21865082E-05

Selection coefficients for In and St homokaryotypes

3.63588333E-05 -3.21865082E-05

Contributions to mean A2 freqs= 8.05077070E-06 5.11183634E-06

Contributions to mean diversities= 1.59185038E-05 1.01686483E-05

Contributions to mean proportions of seg. sites= 1.48293359E-04 1.01239682E-04

Contributions to delta-theta values= 0.619168997 0.643660307

Zone 3: strong selection approximation

Lower and upper bounds of St popn gamma= 4166.66650 23333.3340

Zone 3: strong selection approximation

Probability of zone 3= 0.193197191

Mean load statistics over zone 3

Contributions to loads within In and St= 2.35769941E-04 2.35444124E-04

Contribution to load between In and St= 2.35199812E-04

Contributions to homozygous loads for In and St= 7.83988042E-04 7.83988042E-04

Contributions to inbreeding loads= 5.48218028E-04 5.48543932E-04

Selection coefficients for In and St homokaryotypes

5.96046448E-07 2.38418579E-07

Contributions to mean A2 freqs= 1.46546483E-06 1.46546483E-06

Contributions to mean A2 freqs at seg. sites= 0.00000000 0.00000000

Contributions to mean diversities= 2.92734876E-06 2.92938216E-06

Contributions to delta-theta values= 0.641525745 0.643655658

Mean load statistics over all zones

Loads within In and St= 1.03656817E-02 2.09777174E-03

Load between In and St= 2.82544317E-03

Homozygous load for In and St= 1.18646231E-02 3.41924583E-03

Inbreeding loads= 1.49893947E-03 1.32146967E-03

Selection coefficients for In and St homokaryotypes

7.51185417E-03 -7.27891922E-04

Mean frequencies of A2 in In and St= 7.07153678E-02 4.77070659E-02

Ratio of these= 1.48228288

Mean diversities at selected sites in In and St= 1.12033240E-03 1.87537435E-03

Mean diversities at neutral sites in In and St= 4.75247391E-03 1.09446710E-02

pi-n/pi-s for In and St= 0.235736668 0.171350449

Ratio of these= 1.37575746

Mean freqs. of seg. sites= 4.62151971E-03 7.93891307E-03

Ratio of these= 0.582135081

Overall delta-theta values= 0.139969528 0.161933124

Ratio of these= 0.864366233

**h = 0.25**

Zone 1: quasi-neutral zone

Upper bound scaled selection coefficient for neutrality in St metapopulation= 0.250000000

Probability of zone 1= 4.73524816E-02

Integral of selection coefficient over zone 1= 1.95133864E-04

Mean load statistics for zone 1

Mean q1 and q2= 0.600000024

F1 and F2= 0.990099013 0.977198601

Diversities= 4.75247391E-03 1.09446710E-02

Contributions to loads within In and St= 1.16848481E-04 1.16546405E-04

Contribution to load between In and St = 9.36642609E-05

Contributions to homozygous loads for In and St= 1.17080323E-04 1.17080323E-04

Contributions to inbreeding loads for In and St= 2.33255094E-03 2.33299518E-03

Contributions to selection coefficients for In and St homokaryotypes

2.31862068E-05 2.28881836E-05

Contributions to mean A2 freqs= 2.84114908E-02 2.84114908E-02

Contributions to mean diversities= 2.25041440E-04 5.18257322E-04

Contributions to mean freqs. of seg. site= 7.96483480E-04 1.82903488E-03

Delta-theta values= -2.39169598E-03 -5.25259972E-03

Zone 2a: moderate selection; cut-off at moderate gamma for St population

Lower and upper bounds of St popn gamma

0.250000000 416.666656

Probability of zone 2a= 0.386616915

Coefficients for bivariate distribution of q1 and q2

a1= 7.50000030E-02 a2= 0.174999997

b11= 2.25000009E-02 b12= 0.105000004 b22= 0.122499995

Net probability of zone 2a using Simpsons rule= 0.387911767

Contributions to mean load statistics over zone 2a

Contributions to mean loads within In and St= 7.30897440E-03 1.69902400E-03

Contribution to load between In and St= 2.74170586E-03

Contributions to homozygous loads for In and St= 7.59049272E-03 1.97075284E-03

Contributions to inbreeding loads= 2.81518500E-04 2.71730620E-04

Selection coefficients for In and St homokaryotypes

4.55683470E-03 -1.04320049E-03

Selection coefficients for In and St homokaryotypes

4.55683470E-03 -1.04320049E-03

Contributions to mean A2 freqs= 3.80297564E-02 1.93578098E-02

Contributions to mean diversities= 7.46769074E-04 1.17420987E-03

Contributions to mean proportions of seg. sites= 3.15671880E-03 5.20417141E-03

Contributions to delta-theta values= 0.160729051 0.199528515

Zone 2b: moderate selection; gamma for St reaches high value

Lower and upper bounds of St popn gamma

416.666656 4166.66650

Probability of zone 2b 0.360976309

Net probability of zone 2b using Simpsons rule= 0.360976666

Contributions to mean load statistics over zone 2b

Contributions to mean loads within In and St= 1.51030850E-04 8.22301954E-05

Contribution to load between In and St= 1.16084200E-04

Contributions to homozygous loads for In and St= 3.00353131E-04 1.63970122E-04

Contributions to inbreeding loads= 1.49322135E-04 8.17399778E-05

Selection coefficients for In and St homokaryotypes

3.49283218E-05 -3.38554382E-05

Selection coefficients for In and St homokaryotypes

3.49283218E-05 -3.38554382E-05

Contributions to mean A2 freqs= 4.69220549E-06 2.85500300E-06

Contributions to mean diversities= 9.31675368E-06 5.68967562E-06

Contributions to mean proportions of seg. sites= 9.06387795E-05 5.93371769E-05

Contributions to delta-theta values= 0.635328054 0.659817219

Zone 3: strong selection approximation

Lower and upper bounds of St popn gamma= 4166.66650 23333.3340

Zone 3: strong selection approximation

Probability of zone 3= 0.193197191

Mean load statistics over zone 3

Contributions to loads within In and St= 2.35343978E-04 2.35260188E-04

Contribution to load between In and St= 2.35197353E-04

Contributions to homozygous loads for In and St= 4.70392959E-04 4.70392959E-04

Contributions to inbreeding loads= 2.35049156E-04 2.35132815E-04

Selection coefficients for In and St homokaryotypes

1.19209290E-07 5.96046448E-08

Contributions to mean A2 freqs= 8.79278844E-07 8.79278844E-07

Contributions to mean A2 freqs at seg. sites= 0.00000000 0.00000000

Contributions to mean diversities= 1.75726757E-06 1.75800017E-06

Contributions to delta-theta values= 0.643016875 0.644290924

Mean load statistics over all zones

Loads within In and St= 7.81219732E-03 2.13306071E-03

Load between In and St= 3.18665174E-03

Homozygous load for In and St= 8.47831927E-03 2.72219605E-03

Inbreeding loads= 6.66121661E-04 5.89137315E-04

Selection coefficients for In and St homokaryotypes

4.61488962E-03 -1.05416775E-03

Mean frequencies of A2 in In and St= 6.64468184E-02 4.77730334E-02

Ratio of these= 1.39088547

Mean diversities at selected sites in In and St= 9.82884550E-04 1.69991481E-03

Mean diversities at neutral sites in In and St= 4.75247391E-03 1.09446710E-02

pi-n/pi-s for In and St= 0.206815347 0.155318946

Ratio of these= 1.33155262

Mean freqs. of seg. sites= 4.06130496E-03 7.11007789E-03

Ratio of these= 0.571204007

Overall delta-theta values= 0.141404390 0.151787758

Ratio of these= 0.931592882

**h = 0.35**

Zone 1: quasi-neutral zone

Upper bound scaled selection coefficient for neutrality in St metapopulation= 0.250000000

Probability of zone 1= 4.73524816E-02

Integral of selection coefficient over zone 1= 1.95133864E-04

Mean load statistics for zone 1

Mean q1 and q2= 0.600000024

F1 and F2= 0.990099013 0.977198601

Diversities= 4.75247391E-03 1.09446710E-02

Contributions to loads within In and St= 1.16941221E-04 1.16759962E-04

Contribution to load between In and St = 1.03030681E-04

Contributions to homozygous loads for In and St= 1.17080323E-04 1.17080323E-04

Contributions to inbreeding loads for In and St= 9.99798765E-04 9.99913085E-04

Contributions to selection coefficients for In and St homokaryotypes

1.38878822E-05 1.37090683E-05

Contributions to mean A2 freqs= 2.84114908E-02 2.84114908E-02

Contributions to mean diversities= 2.25041440E-04 5.18257322E-04

Contributions to mean freqs. of seg. site= 7.96483480E-04 1.82903488E-03

Delta-theta values= -2.39169598E-03 -5.25259972E-03

Zone 2a: moderate selection; cut-off at moderate gamma for St population

Lower and upper bounds of St popn gamma

0.250000000 297.619049

Probability of zone 2a= 0.346091360

Coefficients for bivariate distribution of q1 and q2

a1= 0.105000004 a2= 0.244999990

b11= 1.35000013E-02 b12= 6.30000010E-02 b22= 7.34999999E-02

Net probability of zone 2a using Simpsons rule= 0.346664220

Contributions to mean load statistics over zone 2a

Contributions to mean loads within In and St= 5.79066947E-03 1.69720326E-03

Contribution to load between In and St= 2.92303436E-03

Contributions to homozygous loads for In and St= 5.90477930E-03 1.81065826E-03

Contributions to inbreeding loads= 1.14104769E-04 1.13456255E-04

Selection coefficients for In and St homokaryotypes

2.86352634E-03 -1.22654438E-03

Selection coefficients for In and St homokaryotypes

2.86352634E-03 -1.22654438E-03

Contributions to mean A2 freqs= 3.46724764E-02 1.90089755E-02

Contributions to mean diversities= 6.57382130E-04 1.04509830E-03

Contributions to mean proportions of seg. sites= 2.79130391E-03 4.62298235E-03

Contributions to delta-theta values= 0.164469123 0.197977364

Zone 2b: moderate selection; gamma for St reaches high value

Lower and upper bounds of St popn gamma

297.619049 2976.19043

Probability of zone 2b 0.344033331

Net probability of zone 2b using Simpsons rule= 0.344033509

Contributions to mean load statistics over zone 2b

Contributions to mean loads within In and St= 1.65028221E-04 9.42223414E-05

Contribution to load between In and St= 1.29373162E-04

Contributions to homozygous loads for In and St= 2.35197949E-04 1.34434347E-04

Contributions to inbreeding loads= 7.01696918E-05 4.02122241E-05

Selection coefficients for In and St homokaryotypes

3.56435776E-05 -3.51667404E-05

Selection coefficients for In and St homokaryotypes

3.56435776E-05 -3.51667404E-05

Contributions to mean A2 freqs= 4.95430686E-06 3.18083562E-06

Contributions to mean diversities= 9.83824521E-06 6.33923446E-06

Contributions to mean proportions of seg. sites= 9.58354722E-05 6.62890452E-05

Contributions to delta-theta values= 0.635797322 0.660728931

Zone 3: strong selection approximation

Lower and upper bounds of St popn gamma= 2976.19043 23333.3340

Zone 3: strong selection approximation

Probability of zone 3= 0.250665724

Mean load statistics over zone 3

Contributions to loads within In and St= 2.87382223E-04 2.87342787E-04

Contribution to load between In and St= 2.87313072E-04

Contributions to homozygous loads for In and St= 4.10446577E-04 4.10446635E-04

Contributions to inbreeding loads= 1.23064456E-04 1.23103950E-04

Selection coefficients for In and St homokaryotypes

5.96046448E-08 0.00000000

Contributions to mean A2 freqs= 9.67476581E-07 9.67476581E-07

Contributions to mean A2 freqs at seg. sites= 0.00000000 0.00000000

Contributions to mean diversities= 1.93360734E-06 1.93437086E-06

Contributions to delta-theta values= 0.643122315 0.644331455

Mean load statistics over all zones

Loads within In and St= 6.36002095E-03 2.19552824E-03

Load between In and St= 3.44275124E-03

Homozygous load for In and St= 6.66750409E-03 2.47261953E-03

Inbreeding loads= 3.07478011E-04 2.77092797E-04

Selection coefficients for In and St homokaryotypes

2.91299820E-03 -1.24800205E-03

Mean frequencies of A2 in In and St= 6.30898923E-02 4.74246144E-02

Ratio of these= 1.33031952

Mean diversities at selected sites in In and St= 8.94195458E-04 1.57162920E-03

Mean diversities at neutral sites in In and St= 4.75247391E-03 1.09446710E-02

pi-n/pi-s for In and St= 0.188153684 0.143597662

Ratio of these= 1.31028378

Mean freqs. of seg. sites= 3.70284496E-03 6.53760182E-03

Ratio of these= 0.566391945

Overall delta-theta values= 0.143260717 0.147128940

Ratio of these= 0.973708630

**h = 0.45**

Zone 1: quasi-neutral zone

Upper bound scaled selection coefficient for neutrality in St metapopulation= 0.250000000

Probability of zone 1= 4.73524816E-02

Integral of selection coefficient over zone 1= 1.95133864E-04

Mean load statistics for zone 1

Mean q1 and q2= 0.600000024

F1 and F2= 0.990099013 0.977198601

Diversities= 4.75247391E-03 1.09446710E-02

Contributions to loads within In and St= 1.17033960E-04 1.16973541E-04

Contribution to load between In and St = 1.12397101E-04

Contributions to homozygous loads for In and St= 1.17080323E-04 1.17080323E-04

Contributions to inbreeding loads for In and St= 4.28509928E-04 4.28544881E-04

Contributions to selection coefficients for In and St homokaryotypes

4.64916229E-06 4.58955765E-06

Contributions to mean A2 freqs= 2.84114908E-02 2.84114908E-02

Contributions to mean diversities= 2.25041440E-04 5.18257322E-04

Contributions to mean freqs. of seg. site= 7.96483480E-04 1.82903488E-03

Delta-theta values= -2.39169598E-03 -5.25259972E-03

Zone 2a: moderate selection; cut-off at moderate gamma for St population

Lower and upper bounds of St popn gamma

0.250000000 165.343918

Probability of zone 2a= 0.283557355

Coefficients for bivariate distribution of q1 and q2

a1= 0.135000005 a2= 0.314999998

b11= 4.50000120E-03 b12= 2.10000053E-02 b22= 2.45000049E-02

Net probability of zone 2a using Simpsons rule= 0.283669829

Contributions to mean load statistics over zone 2a

Contributions to mean loads within In and St= 4.78107249E-03 1.66670361E-03

Contribution to load between In and St= 3.00266873E-03

Contributions to homozygous loads for In and St= 4.80592018E-03 1.69249496E-03

Contributions to inbreeding loads= 2.48361321E-05 2.57911015E-05

Selection coefficients for In and St homokaryotypes

1.77681446E-03 -1.33681297E-03

Selection coefficients for In and St homokaryotypes

1.77681446E-03 -1.33681297E-03

Contributions to mean A2 freqs= 3.22742239E-02 1.88471433E-02

Contributions to mean diversities= 5.89511706E-04 9.44693631E-04

Contributions to mean proportions of seg. sites= 2.48956797E-03 4.15609498E-03

Contributions to delta-theta values= 0.159920812 0.193587482

Zone 2b: moderate selection; gamma for St reaches high value

Lower and upper bounds of St popn gamma

165.343918 1653.43921

Probability of zone 2b 0.306239307

Net probability of zone 2b using Simpsons rule= 0.306239247

Contributions to mean load statistics over zone 2b

Contributions to mean loads within In and St= 1.87596190E-04 1.21576973E-04

Contribution to load between In and St= 1.54489229E-04

Contributions to homozygous loads for In and St= 2.08276630E-04 1.35030845E-04

Contributions to inbreeding loads= 2.06804252E-05 1.34538141E-05

Selection coefficients for In and St homokaryotypes

3.30805779E-05 -3.29017639E-05

Selection coefficients for In and St homokaryotypes

3.30805779E-05 -3.29017639E-05

Contributions to mean A2 freqs= 7.33839943E-06 5.30425268E-06

Contributions to mean diversities= 1.45384265E-05 1.05600966E-05

Contributions to mean proportions of seg. sites= 1.37810392E-04 1.07128893E-04

Contributions to delta-theta values= 0.625728130 0.650285959

Zone 3: strong selection approximation

Lower and upper bounds of St popn gamma= 1653.43921 23333.3340

Zone 3: strong selection approximation

Probability of zone 3= 0.350993752

Mean load statistics over zone 3

Contributions to loads within In and St= 3.77790508E-04 3.77774559E-04

Contribution to load between In and St= 3.77763266E-04

Contributions to homozygous loads for In and St= 4.19736112E-04 4.19736112E-04

Contributions to inbreeding loads= 4.19461321E-05 4.19618154E-05

Selection coefficients for In and St homokaryotypes

0.00000000 0.00000000

Contributions to mean A2 freqs= 1.47335356E-06 1.47335368E-06

Contributions to mean A2 freqs at seg. sites= 0.00000000 0.00000000

Contributions to mean diversities= 2.94406709E-06 2.94556435E-06

Contributions to delta-theta values= 0.642514825 0.644077897

Mean load statistics over all zones

Loads within In and St= 5.46349352E-03 2.28302856E-03

Load between In and St= 3.64731834E-03

Homozygous load for In and St= 5.55101316E-03 2.36434210E-03

Inbreeding loads= 8.75090482E-05 8.13135121E-05

Selection coefficients for In and St homokaryotypes

1.81454420E-03 -1.36518478E-03

Mean frequencies of A2 in In and St= 6.06945306E-02 4.72654141E-02

Ratio of these= 1.28412139

Mean diversities at selected sites in In and St= 8.32035614E-04 1.47645664E-03

Mean diversities at neutral sites in In and St= 4.75247391E-03 1.09446710E-02

pi-n/pi-s for In and St= 0.175074205 0.134901881

Ratio of these= 1.29778922

Mean freqs. of seg. sites= 3.45307938E-03 6.12161914E-03

Ratio of these= 0.564079404

Overall delta-theta values= 0.145155549 0.144330323

Ratio of these= 1.00571764

**h = 0.5**

Zone 1: quasi-neutral zone

Upper bound scaled selection coefficient for neutrality in St metapopulation= 0.250000000

Probability of zone 1= 4.73524816E-02

Integral of selection coefficient over zone 1= 1.95133864E-04

Mean load statistics for zone 1

Mean q1 and q2= 0.600000024

F1 and F2= 0.990099013 0.977198601

Diversities= 4.75247391E-03 1.09446710E-02

Contributions to loads within In and St= 1.17080323E-04 1.17080323E-04

Contribution to load between In and St = 1.17080323E-04

Contributions to homozygous loads for In and St= 1.17080323E-04 1.17080323E-04

Contributions to inbreeding loads for In and St= 1.11098670E-04 1.11105772E-04

Contributions to selection coefficients for In and St homokaryotypes

0.00000000 0.00000000

Contributions to mean A2 freqs= 2.84114908E-02 2.84114908E-02

Contributions to mean diversities= 2.25041440E-04 5.18257322E-04

Contributions to mean freqs. of seg. site= 7.96483480E-04 1.82903488E-03

Delta-theta values= -2.39169598E-03 -5.25259972E-03

Zone 2a: moderate selection; cut-off at moderate gamma for St population

Lower and upper bounds of St popn gamma

0.250000000 82.6719589

Probability of zone 2a= 0.221977368

Coefficients for bivariate distribution of q1 and q2

a1= 0.150000006 a2= 0.349999994

b11= 0.00000000 b12= 0.00000000 b22= 0.00000000

Net probability of zone 2a using Simpsons rule= 0.221989021

Contributions to mean load statistics over zone 2a

Contributions to mean loads within In and St= 4.36759600E-03 1.62132597E-03

Contribution to load between In and St= 2.99446355E-03

Contributions to homozygous loads for In and St= 4.36759600E-03 1.62132597E-03

Contributions to inbreeding loads= 0.00000000 0.00000000

Selection coefficients for In and St homokaryotypes

1.37221813E-03 -1.37412548E-03

Selection coefficients for In and St homokaryotypes

1.37221813E-03 -1.37412548E-03

Contributions to mean A2 freqs= 3.13597396E-02 1.88537631E-02

Contributions to mean diversities= 5.52298268E-04 8.94386962E-04

Contributions to mean proportions of seg. sites= 2.28410470E-03 3.88056901E-03

Contributions to delta-theta values= 0.142153800 0.182322979

Zone 2b: moderate selection; gamma for St reaches high value

Lower and upper bounds of St popn gamma

82.6719589 826.719604

Probability of zone 2b 0.258414686

Net probability of zone 2b using Simpsons rule= 0.258414596

Contributions to mean load statistics over zone 2b

Contributions to mean loads within In and St= 1.98502908E-04 1.49314292E-04

Contribution to load between In and St= 1.73908527E-04

Contributions to homozygous loads for In and St= 1.98502908E-04 1.49314292E-04

Contributions to inbreeding loads= 0.00000000 0.00000000

Selection coefficients for In and St homokaryotypes

2.46167183E-05 -2.45571136E-05

Selection coefficients for In and St homokaryotypes

2.46167183E-05 -2.45571136E-05

Contributions to mean A2 freqs= 1.30229200E-05 1.06116058E-05

Contributions to mean diversities= 2.56256990E-05 2.10648141E-05

Contributions to mean proportions of seg. sites= 2.29364188E-04 2.03632182E-04

Contributions to delta-theta values= 0.603628993 0.633002639

Zone 3: strong selection approximation

Lower and upper bounds of St popn gamma= 826.719604 23333.3340

Zone 3: strong selection approximation

Probability of zone 3= 0.460398376

Mean load statistics over zone 3

Contributions to loads within In and St= 4.77309775E-04 4.77309775E-04

Contribution to load between In and St= 4.77309775E-04

Contributions to homozygous loads for In and St= 4.77309775E-04 4.77309775E-04

Contributions to inbreeding loads= 0.00000000 0.00000000

Selection coefficients for In and St homokaryotypes

0.00000000 0.00000000

Contributions to mean A2 freqs= 2.65328799E-06 2.65328799E-06

Contributions to mean A2 freqs at seg. sites= 0.00000000 0.00000000

Contributions to mean diversities= 5.29869385E-06 5.30316856E-06

Contributions to delta-theta values= 0.640659511 0.643299103

Mean load statistics over all zones

Loads within In and St= 5.16048912E-03 2.36503035E-03

Load between In and St= 3.76276206E-03

Homozygous load for In and St= 5.16048912E-03 2.36503035E-03

Inbreeding loads= 0.00000000 0.00000000

Selection coefficients for In and St homokaryotypes

1.39677525E-03 -1.39868259E-03

Mean frequencies of A2 in In and St= 5.97869083E-02 4.72785197E-02

Ratio of these= 1.26456809

Mean diversities at selected sites in In and St= 8.08264071E-04 1.43901224E-03

Mean diversities at neutral sites in In and St= 4.75247391E-03 1.09446710E-02

pi-n/pi-s for In and St= 0.170072272 0.131480634

Ratio of these= 1.29351580

Mean freqs. of seg. sites= 3.36226611E-03 5.96598117E-03

Ratio of these= 0.563573062

Overall delta-theta values= 0.147149444 0.144274712

Ratio of these= 1.01992536

**Inversion frequency= 0.5**

**h = 0.05**

Zone 1: quasi-neutral zone

Upper bound scaled selection coefficient for neutrality in St metapopulation= 0.250000000

Probability of zone 1= 7.93938339E-02

Integral of selection coefficient over zone 1= 4.58041381E-04

Mean load statistics for zone 1

Mean q1 and q2= 0.600000024

F1 and F2= 0.983606577 0.983606577

Diversities= 7.86884315E-03 7.86884315E-03

Contributions to loads within In and St= 2.73202924E-04 2.73202924E-04

Contribution to load between In and St = 1.75887893E-04

Contributions to homozygous loads for In and St= 2.74824852E-04 2.74824852E-04

Contributions to inbreeding loads for In and St= 0.00000000 0.00000000

Contributions to selection coefficients for In and St homokaryotypes

9.73343849E-05 9.73343849E-05

Contributions to mean A2 freqs= 4.76363041E-02 4.76363041E-02

Contributions to mean diversities= 6.24737644E-04 6.24737644E-04

Contributions to mean freqs. of seg. site= 2.20816326E-03 2.20816326E-03

Delta-theta values= -3.73303890E-03 -3.73303890E-03

Zone 2a: moderate selection; cut-off at moderate gamma for St population

Lower and upper bounds of St popn gamma

0.250000000 250.000000

Probability of zone 2a= 0.530339003

Coefficients for bivariate distribution of q1 and q2

a1= 2.50000004E-02 a2= 2.50000004E-02

b11= 0.112499997 b12= 0.224999994 b22= 0.112499997

Net probability of zone 2a using Simpsons rule= 0.530947506

Contributions to mean load statistics over zone 2a

Contributions to mean loads within In and St= 5.68476645E-03 5.68473013E-03

Contribution to load between In and St= 1.87811872E-03

Contributions to homozygous loads for In and St= 7.60443183E-03 7.60439551E-03

Contributions to inbreeding loads= 1.91966537E-03 1.91966502E-03

Selection coefficients for In and St homokaryotypes

3.79943848E-03 3.79937887E-03

Selection coefficients for In and St homokaryotypes

3.79943848E-03 3.79937887E-03

Contributions to mean A2 freqs= 3.73776853E-02 3.73775475E-02

Contributions to mean diversities= 2.11785012E-03 2.11784802E-03

Contributions to mean proportions of seg. sites= 9.04238131E-03 9.04238224E-03

Contributions to delta-theta values= 0.169070542 0.169071436

Zone 2b: moderate selection; gamma for St reaches high value

Lower and upper bounds of St popn gamma

250.000000 2500.00000

Probability of zone 2b 0.348146498

Net probability of zone 2b using Simpsons rule= 0.348146081

Contributions to mean load statistics over zone 2b

Contributions to mean loads within In and St= 1.52504770E-04 1.52504756E-04

Contribution to load between In and St= 1.33518552E-04

Contributions to homozygous loads for In and St= 1.33306638E-03 1.33306638E-03

Contributions to inbreeding loads= 1.18056161E-03 1.18056196E-03

Selection coefficients for In and St homokaryotypes

1.90138817E-05 1.90138817E-05

Selection coefficients for In and St homokaryotypes

1.90138817E-05 1.90138817E-05

Contributions to mean A2 freqs= 2.63789843E-05 2.63789843E-05

Contributions to mean diversities= 5.17334520E-05 5.17334447E-05

Contributions to mean proportions of seg. sites= 4.50521969E-04 4.50522406E-04

Contributions to delta-theta values= 0.592612982 0.592613339

Zone 3: strong selection approximation

Lower and upper bounds of St popn gamma= 2500.00000 4166.66699

Zone 3: strong selection approximation

Probability of zone 3= 3.02635431E-02

Mean load statistics over zone 3

Contributions to loads within In and St= 7.78885296E-05 7.78885296E-05

Contribution to load between In and St= 7.56815352E-05

Contributions to homozygous loads for In and St= 7.56593887E-04 7.56593887E-04

Contributions to inbreeding loads= 6.78705750E-04 6.78705750E-04

Selection coefficients for In and St homokaryotypes

2.20537186E-06 2.20537186E-06

Contributions to mean A2 freqs= 2.45221031E-06 2.45221031E-06

Contributions to mean A2 freqs at seg. sites= 0.00000000 0.00000000

Contributions to mean diversities= 4.88805108E-06 4.88805108E-06

Contributions to delta-theta values= 0.634943843 0.634943843

Mean load statistics over all zones

Loads within In and St= 6.18836284E-03 6.18832605E-03

Load between In and St= 2.26320676E-03

Homozygous load for In and St= 9.96891595E-03 9.96888056E-03

Inbreeding loads= 3.78055475E-03 3.78055475E-03

Selection coefficients for In and St homokaryotypes

3.91745567E-03 3.91745567E-03

Mean frequencies of A2 in In and St= 8.50428194E-02 8.50426853E-02

Ratio of these= 1.00000155

Mean diversities at selected sites in In and St= 2.79920921E-03 2.79920711E-03

Mean diversities at neutral sites in In and St= 7.86884315E-03 7.86884315E-03

pi-n/pi-s for In and St= 0.355733246 0.355732977

Ratio of these= 1.00000072

Mean freqs. of seg. sites= 1.17485700E-02 1.17485719E-02

Ratio of these= 0.999999821

Overall delta-theta values= 0.154717028 0.154717863

Ratio of these= 0.999994636

**h = 0.15**

Zone 1: quasi-neutral zone

Upper bound scaled selection coefficient for neutrality in St metapopulation= 0.250000000

Probability of zone 1= 7.93938339E-02

Integral of selection coefficient over zone 1= 4.58041381E-04

Mean load statistics for zone 1

Mean q1 and q2= 0.600000024

F1 and F2= 0.983606577 0.983606577

Diversities= 7.86884315E-03 7.86884315E-03

Contributions to loads within In and St= 2.73563346E-04 2.73563346E-04

Contribution to load between In and St = 1.97873887E-04

Contributions to homozygous loads for In and St= 2.74824852E-04 2.74824852E-04

Contributions to inbreeding loads for In and St= 8.97818338E-03 8.97818338E-03

Contributions to selection coefficients for In and St homokaryotypes

7.56978989E-05 7.56978989E-05

Contributions to mean A2 freqs= 4.76363041E-02 4.76363041E-02

Contributions to mean diversities= 6.24737644E-04 6.24737644E-04

Contributions to mean freqs. of seg. site= 2.20816326E-03 2.20816326E-03

Delta-theta values= -3.73303890E-03 -3.73303890E-03

Zone 2a: moderate selection; cut-off at moderate gamma for St population

Lower and upper bounds of St popn gamma

0.250000000 416.666656

Probability of zone 2a= 0.616143107

Coefficients for bivariate distribution of q1 and q2

a1= 7.50000030E-02 a2= 7.50000030E-02

b11= 8.74999985E-02 b12= 0.174999997 b22= 8.74999985E-02

Net probability of zone 2a using Simpsons rule= 0.618314326

Contributions to mean load statistics over zone 2a

Contributions to mean loads within In and St= 5.15023852E-03 5.15023666E-03

Contribution to load between In and St= 2.62880325E-03

Contributions to homozygous loads for In and St= 6.07210724E-03 6.07210398E-03

Contributions to inbreeding loads= 9.21864819E-04 9.21864936E-04

Selection coefficients for In and St homokaryotypes

2.51823664E-03 2.51823664E-03

Selection coefficients for In and St homokaryotypes

2.51823664E-03 2.51823664E-03

Contributions to mean A2 freqs= 3.67072858E-02 3.67072709E-02

Contributions to mean diversities= 1.69638882E-03 1.69638905E-03

Contributions to mean proportions of seg. sites= 7.34124659E-03 7.34124752E-03

Contributions to delta-theta values= 0.180201054 0.180201054

Zone 2b: moderate selection; gamma for St reaches high value

Lower and upper bounds of St popn gamma

416.666656 4166.66650

Probability of zone 2b 0.292605937

Net probability of zone 2b using Simpsons rule= 0.292605817

Contributions to mean load statistics over zone 2b

Contributions to mean loads within In and St= 9.09522132E-05 9.09522278E-05

Contribution to load between In and St= 8.99157021E-05

Contributions to homozygous loads for In and St= 2.99693056E-04 2.99692998E-04

Contributions to inbreeding loads= 2.08740530E-04 2.08740516E-04

Selection coefficients for In and St homokaryotypes

1.01327896E-06 1.01327896E-06

Selection coefficients for In and St homokaryotypes

1.01327896E-06 1.01327896E-06

Contributions to mean A2 freqs= 4.10548046E-06 4.10548046E-06

Contributions to mean diversities= 8.16370539E-06 8.16370448E-06

Contributions to mean proportions of seg. sites= 8.06488169E-05 8.06496391E-05

Contributions to delta-theta values= 0.640878797 0.640882492

Zone 3: strong selection approximation

Lower and upper bounds of St popn gamma= 4166.66650 4166.66699

Zone 3: strong selection approximation

Probability of zone 3= 5.96046448E-08

Mean load statistics over zone 3

Contributions to loads within In and St= 3.61875173E-05 3.61875173E-05

Contribution to load between In and St= 3.61200109E-05

Contributions to homozygous loads for In and St= 1.20398276E-04 1.20398276E-04

Contributions to inbreeding loads= 8.42103036E-05 8.42103036E-05

Selection coefficients for In and St homokaryotypes

5.96046448E-08 5.96046448E-08

Contributions to mean A2 freqs= 2.88956983E-07 2.88956983E-07

Contributions to mean A2 freqs at seg. sites= 0.00000000 0.00000000

Contributions to mean diversities= 5.77444837E-07 5.77444837E-07

Contributions to delta-theta values= 0.642803490 0.642803490

Mean load statistics over all zones

Loads within In and St= 5.55094145E-03 5.55093959E-03

Load between In and St= 2.95271282E-03

Homozygous load for In and St= 6.76702335E-03 6.76702010E-03

Inbreeding loads= 1.21607713E-03 1.21607725E-03

Selection coefficients for In and St homokaryotypes

2.59482861E-03 2.59482861E-03

Mean frequencies of A2 in In and St= 8.43479857E-02 8.43479708E-02

Ratio of these= 1.00000012

Mean diversities at selected sites in In and St= 2.32986757E-03 2.32986780E-03

Mean diversities at neutral sites in In and St= 7.86884315E-03 7.86884315E-03

pi-n/pi-s for In and St= 0.296087682 0.296087712

Ratio of these= 0.999999881

Mean freqs. of seg. sites= 9.63579398E-03 9.63579584E-03

Ratio of these= 0.999999821

Overall delta-theta values= 0.142181337 0.142181396

Ratio of these= 0.999999583

**h = 0.25**

Zone 1: quasi-neutral zone

Upper bound scaled selection coefficient for neutrality in St metapopulation= 0.250000000

Probability of zone 1= 7.93938339E-02

Integral of selection coefficient over zone 1= 4.58041381E-04

Mean load statistics for zone 1

Mean q1 and q2= 0.600000024

F1 and F2= 0.983606577 0.983606577

Diversities= 7.86884315E-03 7.86884315E-03

Contributions to loads within In and St= 2.73923797E-04 2.73923797E-04

Contribution to load between In and St = 2.19859867E-04

Contributions to homozygous loads for In and St= 2.74824852E-04 2.74824852E-04

Contributions to inbreeding loads for In and St= 2.33144779E-03 2.33144779E-03

Contributions to selection coefficients for In and St homokaryotypes

5.40614128E-05 5.40614128E-05

Contributions to mean A2 freqs= 4.76363041E-02 4.76363041E-02

Contributions to mean diversities= 6.24737644E-04 6.24737644E-04

Contributions to mean freqs. of seg. site= 2.20816326E-03 2.20816326E-03

Delta-theta values= -3.73303890E-03 -3.73303890E-03

Zone 2a: moderate selection; cut-off at moderate gamma for St population

Lower and upper bounds of St popn gamma

0.250000000 416.666656

Probability of zone 2a= 0.616143107

Coefficients for bivariate distribution of q1 and q2

a1= 0.125000000 a2= 0.125000000

b11= 6.25000000E-02 b12= 0.125000000 b22= 6.25000000E-02

Net probability of zone 2a using Simpsons rule= 0.618314326

Contributions to mean load statistics over zone 2a

Contributions to mean loads within In and St= 4.61552246E-03 4.61552246E-03

Contribution to load between In and St= 3.11410613E-03

Contributions to homozygous loads for In and St= 5.05604455E-03 5.05604409E-03

Contributions to inbreeding loads= 4.40524745E-04 4.40524804E-04

Selection coefficients for In and St homokaryotypes

1.50030851E-03 1.50030851E-03

Selection coefficients for In and St homokaryotypes

1.50030851E-03 1.50030851E-03

Contributions to mean A2 freqs= 3.53376009E-02 3.53376009E-02

Contributions to mean diversities= 1.44505315E-03 1.44505326E-03

Contributions to mean proportions of seg. sites= 6.30858494E-03 6.30858354E-03

Contributions to delta-theta values= 0.187349856 0.187349617

Zone 2b: moderate selection; gamma for St reaches high value

Lower and upper bounds of St popn gamma

416.666656 4166.66650

Probability of zone 2b 0.292605937

Net probability of zone 2b using Simpsons rule= 0.292605817

Contributions to mean load statistics over zone 2b

Contributions to mean loads within In and St= 8.27892582E-05 8.27892727E-05

Contribution to load between In and St= 8.25187672E-05

Contributions to homozygous loads for In and St= 1.65033838E-04 1.65033838E-04

Contributions to inbreeding loads= 8.22447837E-05 8.22447983E-05

Selection coefficients for In and St homokaryotypes

2.98023224E-07 2.98023224E-07

Selection coefficients for In and St homokaryotypes

2.98023224E-07 2.98023224E-07

Contributions to mean A2 freqs= 2.31252170E-06 2.31252193E-06

Contributions to mean diversities= 4.60769706E-06 4.60769661E-06

Contributions to mean proportions of seg. sites= 4.75164525E-05 4.75160450E-05

Contributions to delta-theta values= 0.655973673 0.655970812

Zone 3: strong selection approximation

Lower and upper bounds of St popn gamma= 4166.66650 4166.66699

Zone 3: strong selection approximation

Probability of zone 3= 5.96046448E-08

Mean load statistics over zone 3

Contributions to loads within In and St= 3.61369202E-05 3.61369202E-05

Contribution to load between In and St= 3.61197453E-05

Contributions to homozygous loads for In and St= 7.22385521E-05 7.22385521E-05

Contributions to inbreeding loads= 3.61019447E-05 3.61019447E-05

Selection coefficients for In and St homokaryotypes

0.00000000 0.00000000

Contributions to mean A2 freqs= 1.73373905E-07 1.73373905E-07

Contributions to mean A2 freqs at seg. sites= 0.00000000 0.00000000

Contributions to mean diversities= 3.46579071E-07 3.46579071E-07

Contributions to delta-theta values= 0.643715501 0.643715501

Mean load statistics over all zones

Loads within In and St= 5.00837248E-03 5.00837248E-03

Load between In and St= 3.45260440E-03

Homozygous load for In and St= 5.56814158E-03 5.56814112E-03

Inbreeding loads= 5.59772539E-04 5.59772598E-04

Selection coefficients for In and St homokaryotypes

1.55454874E-03 1.55454874E-03

Mean frequencies of A2 in In and St= 8.29763860E-02 8.29763860E-02

Ratio of these= 1.00000000

Mean diversities at selected sites in In and St= 2.07474525E-03 2.07474525E-03

Mean diversities at neutral sites in In and St= 7.86884315E-03 7.86884315E-03

pi-n/pi-s for In and St= 0.263665855 0.263665855

Ratio of these= 1.00000000

Mean freqs. of seg. sites= 8.56771600E-03 8.56771413E-03

Ratio of these= 1.00000024

Overall delta-theta values= 0.140884697 0.140884459

Ratio of these= 1.00000167

**h = 0.35**

Zone 1: quasi-neutral zone

Upper bound scaled selection coefficient for neutrality in St metapopulation= 0.250000000

Probability of zone 1= 7.93938339E-02

Integral of selection coefficient over zone 1= 4.58041381E-04

Mean load statistics for zone 1

Mean q1 and q2= 0.600000024

F1 and F2= 0.983606577 0.983606577

Diversities= 7.86884315E-03 7.86884315E-03

Contributions to loads within In and St= 2.74284219E-04 2.74284219E-04

Contribution to load between In and St = 2.41845846E-04

Contributions to homozygous loads for In and St= 2.74824852E-04 2.74824852E-04

Contributions to inbreeding loads for In and St= 9.99515178E-04 9.99515178E-04

Contributions to selection coefficients for In and St homokaryotypes

3.24249268E-05 3.24249268E-05

Contributions to mean A2 freqs= 4.76363041E-02 4.76363041E-02

Contributions to mean diversities= 6.24737644E-04 6.24737644E-04

Contributions to mean freqs. of seg. site= 2.20816326E-03 2.20816326E-03

Delta-theta values= -3.73303890E-03 -3.73303890E-03

Zone 2a: moderate selection; cut-off at moderate gamma for St population

Lower and upper bounds of St popn gamma

0.250000000 297.619049

Probability of zone 2a= 0.559083343

Coefficients for bivariate distribution of q1 and q2

a1= 0.174999997 a2= 0.174999997

b11= 3.75000015E-02 b12= 7.50000030E-02 b22= 3.75000015E-02

Net probability of zone 2a using Simpsons rule= 0.560043395

Contributions to mean load statistics over zone 2a

Contributions to mean loads within In and St= 4.18453524E-03 4.18453524E-03

Contribution to load between In and St= 3.43011064E-03

Contributions to homozygous loads for In and St= 4.36922768E-03 4.36922722E-03

Contributions to inbreeding loads= 1.84693723E-04 1.84693723E-04

Selection coefficients for In and St homokaryotypes

7.54117966E-04 7.54117966E-04

Selection coefficients for In and St homokaryotypes

7.54117966E-04 7.54117966E-04

Contributions to mean A2 freqs= 3.35704386E-02 3.35704386E-02

Contributions to mean diversities= 1.26989081E-03 1.26989116E-03

Contributions to mean proportions of seg. sites= 5.57274139E-03 5.57274325E-03

Contributions to delta-theta values= 0.191557348 0.191557348

Zone 2b: moderate selection; gamma for St reaches high value

Lower and upper bounds of St popn gamma

297.619049 2976.19043

Probability of zone 2b 0.332554400

Net probability of zone 2b using Simpsons rule= 0.332554698

Contributions to mean load statistics over zone 2b

Contributions to mean loads within In and St= 1.06616819E-04 1.06616819E-04

Contribution to load between In and St= 1.06473184E-04

Contributions to homozygous loads for In and St= 1.52103385E-04 1.52103370E-04

Contributions to inbreeding loads= 4.54862857E-05 4.54862893E-05

Selection coefficients for In and St homokaryotypes

1.19209290E-07 1.19209290E-07

Selection coefficients for In and St homokaryotypes

1.19209290E-07 1.19209290E-07

Contributions to mean A2 freqs= 2.83452846E-06 2.83452823E-06

Contributions to mean diversities= 5.64822813E-06 5.64822903E-06

Contributions to mean proportions of seg. sites= 5.85081470E-05 5.85080743E-05

Contributions to delta-theta values= 0.657510221 0.657509685

Zone 3: strong selection approximation

Lower and upper bounds of St popn gamma= 2976.19043 4166.66699

Zone 3: strong selection approximation

Probability of zone 3= 1.71113014E-02

Mean load statistics over zone 3

Contributions to loads within In and St= 5.98997249E-05 5.98997249E-05

Contribution to load between In and St= 5.98891056E-05

Contributions to homozygous loads for In and St= 8.55556864E-05 8.55556864E-05

Contributions to inbreeding loads= 2.56559706E-05 2.56559706E-05

Selection coefficients for In and St homokaryotypes

0.00000000 0.00000000

Contributions to mean A2 freqs= 2.48229043E-07 2.48229043E-07

Contributions to mean A2 freqs at seg. sites= 0.00000000 0.00000000

Contributions to mean diversities= 4.96248106E-07 4.96248106E-07

Contributions to delta-theta values= 0.643961787 0.643961787

Mean load statistics over all zones

Loads within In and St= 4.62533627E-03 4.62533627E-03

Load between In and St= 3.83831887E-03

Homozygous load for In and St= 4.88171168E-03 4.88171121E-03

Inbreeding loads= 2.56376603E-04 2.56376603E-04

Selection coefficients for In and St homokaryotypes

7.86721706E-04 7.86721706E-04

Mean frequencies of A2 in In and St= 8.12098160E-02 8.12098160E-02

Ratio of these= 1.00000000

Mean diversities at selected sites in In and St= 1.90077291E-03 1.90077338E-03

Mean diversities at neutral sites in In and St= 7.86884315E-03 7.86884315E-03

pi-n/pi-s for In and St= 0.241556838 0.241556898

Ratio of these= 0.999999762

Mean freqs. of seg. sites= 7.84435868E-03 7.84436055E-03

Ratio of these= 0.999999762

Overall delta-theta values= 0.140344262 0.140344322

Ratio of these= 0.999999583

**h = 0.45**

Zone 1: quasi-neutral zone

Upper bound scaled selection coefficient for neutrality in St metapopulation= 0.250000000

Probability of zone 1= 7.93938339E-02

Integral of selection coefficient over zone 1= 4.58041381E-04

Mean load statistics for zone 1

Mean q1 and q2= 0.600000024

F1 and F2= 0.983606577 0.983606577

Diversities= 7.86884315E-03 7.86884315E-03

Contributions to loads within In and St= 2.74644612E-04 2.74644612E-04

Contribution to load between In and St = 2.63831811E-04

Contributions to homozygous loads for In and St= 2.74824852E-04 2.74824852E-04

Contributions to inbreeding loads for In and St= 4.28422994E-04 4.28422994E-04

Contributions to selection coefficients for In and St homokaryotypes

1.07884407E-05 1.07884407E-05

Contributions to mean A2 freqs= 4.76363041E-02 4.76363041E-02

Contributions to mean diversities= 6.24737644E-04 6.24737644E-04

Contributions to mean freqs. of seg. site= 2.20816326E-03 2.20816326E-03

Delta-theta values= -3.73303890E-03 -3.73303890E-03

Zone 2a: moderate selection; cut-off at moderate gamma for St population

Lower and upper bounds of St popn gamma

0.250000000 165.343918

Probability of zone 2a= 0.465305537

Coefficients for bivariate distribution of q1 and q2

a1= 0.224999994 a2= 0.224999994

b11= 1.25000030E-02 b12= 2.50000060E-02 b22= 1.25000030E-02

Net probability of zone 2a using Simpsons rule= 0.465493798

Contributions to mean load statistics over zone 2a

Contributions to mean loads within In and St= 3.80048831E-03 3.80048831E-03

Contribution to load between In and St= 3.58439796E-03

Contributions to homozygous loads for In and St= 3.84290237E-03 3.84290214E-03

Contributions to inbreeding loads= 4.24180034E-05 4.24180034E-05

Selection coefficients for In and St homokaryotypes

2.16066837E-04 2.16066837E-04

Selection coefficients for In and St homokaryotypes

2.16066837E-04 2.16066837E-04

Contributions to mean A2 freqs= 3.21506970E-02 3.21506970E-02

Contributions to mean diversities= 1.13944721E-03 1.13944721E-03

Contributions to mean proportions of seg. sites= 4.99977591E-03 4.99977730E-03

Contributions to delta-theta values= 0.191471338 0.191471577

Zone 2b: moderate selection; gamma for St reaches high value

Lower and upper bounds of St popn gamma

165.343918 1653.43921

Probability of zone 2b 0.369981229

Net probability of zone 2b using Simpsons rule= 0.369981200

Contributions to mean load statistics over zone 2b

Contributions to mean loads within In and St= 1.57674382E-04 1.57674338E-04

Contribution to load between In and St= 1.57607385E-04

Contributions to homozygous loads for In and St= 1.75118606E-04 1.75118621E-04

Contributions to inbreeding loads= 1.74441629E-05 1.74441629E-05

Selection coefficients for In and St homokaryotypes

5.96046448E-08 5.96046448E-08

Selection coefficients for In and St homokaryotypes

5.96046448E-08 5.96046448E-08

Contributions to mean A2 freqs= 5.27551720E-06 5.27551811E-06

Contributions to mean diversities= 1.05014287E-05 1.05014296E-05

Contributions to mean proportions of seg. sites= 1.06059124E-04 1.06059502E-04

Contributions to delta-theta values= 0.648721099 0.648722291

Zone 3: strong selection approximation

Lower and upper bounds of St popn gamma= 1653.43921 4166.66699

Zone 3: strong selection approximation

Probability of zone 3= 7.34623075E-02

Mean load statistics over zone 3

Contributions to loads within In and St= 1.21799290E-04 1.21799290E-04

Contribution to load between In and St= 1.21792684E-04

Contributions to homozygous loads for In and St= 1.35325143E-04 1.35325143E-04

Contributions to inbreeding loads= 1.35260116E-05 1.35260116E-05

Selection coefficients for In and St homokaryotypes

0.00000000 0.00000000

Contributions to mean A2 freqs= 5.80006201E-07 5.80006201E-07

Contributions to mean A2 freqs at seg. sites= 0.00000000 0.00000000

Contributions to mean diversities= 1.15942146E-06 1.15942146E-06

Contributions to delta-theta values= 0.643706024 0.643706024

Mean load statistics over all zones

Loads within In and St= 4.35460638E-03 4.35460638E-03

Load between In and St= 4.12763003E-03

Homozygous load for In and St= 4.42817109E-03 4.42817062E-03

Inbreeding loads= 7.35683934E-05 7.35683934E-05

Selection coefficients for In and St homokaryotypes

2.26974487E-04 2.26974487E-04

Mean frequencies of A2 in In and St= 7.97928572E-02 7.97928572E-02

Ratio of these= 1.00000000

Mean diversities at selected sites in In and St= 1.77584565E-03 1.77584565E-03

Mean diversities at neutral sites in In and St= 7.86884315E-03 7.86884315E-03

pi-n/pi-s for In and St= 0.225680649 0.225680649

Ratio of these= 1.00000000

Mean freqs. of seg. sites= 7.32554309E-03 7.32554449E-03

Ratio of these= 0.999999821

Overall delta-theta values= 0.139962971 0.139963150

Ratio of these= 0.999998748

**h = 0.5**

Zone 1: quasi-neutral zone

Upper bound scaled selection coefficient for neutrality in St metapopulation= 0.250000000

Probability of zone 1= 7.93938339E-02

Integral of selection coefficient over zone 1= 4.58041381E-04

Mean load statistics for zone 1

Mean q1 and q2= 0.600000024

F1 and F2= 0.983606577 0.983606577

Diversities= 7.86884315E-03 7.86884315E-03

Contributions to loads within In and St= 2.74824852E-04 2.74824852E-04

Contribution to load between In and St = 2.74824852E-04

Contributions to homozygous loads for In and St= 2.74824852E-04 2.74824852E-04

Contributions to inbreeding loads for In and St= 1.11081237E-04 1.11081237E-04

Contributions to selection coefficients for In and St homokaryotypes

0.00000000 0.00000000

Contributions to mean A2 freqs= 4.76363041E-02 4.76363041E-02

Contributions to mean diversities= 6.24737644E-04 6.24737644E-04

Contributions to mean freqs. of seg. site= 2.20816326E-03 2.20816326E-03

Delta-theta values= -3.73303890E-03 -3.73303890E-03

Zone 2a: moderate selection; cut-off at moderate gamma for St population

Lower and upper bounds of St popn gamma

0.250000000 82.6719589

Probability of zone 2a= 0.368007213

Coefficients for bivariate distribution of q1 and q2

a1= 0.250000000 a2= 0.250000000

b11= 0.00000000 b12= 0.00000000 b22= 0.00000000

Net probability of zone 2a using Simpsons rule= 0.368027240

Contributions to mean load statistics over zone 2a

Contributions to mean loads within In and St= 3.65892518E-03 3.65892518E-03

Contribution to load between In and St= 3.65892495E-03

Contributions to homozygous loads for In and St= 3.65892518E-03 3.65892518E-03

Contributions to inbreeding loads= 0.00000000 0.00000000

Selection coefficients for In and St homokaryotypes

0.00000000 0.00000000

Selection coefficients for In and St homokaryotypes

0.00000000 0.00000000

Contributions to mean A2 freqs= 3.22805345E-02 3.22805345E-02

Contributions to mean diversities= 1.07621157E-03 1.07621180E-03

Contributions to mean proportions of seg. sites= 4.66983858E-03 4.66983812E-03

Contributions to delta-theta values= 0.182387471 0.182387233

Zone 2b: moderate selection; gamma for St reaches high value

Lower and upper bounds of St popn gamma

82.6719589 826.719604

Probability of zone 2b 0.365087926

Net probability of zone 2b using Simpsons rule= 0.365087867

Contributions to mean load statistics over zone 2b

Contributions to mean loads within In and St= 2.14535918E-04 2.14535918E-04

Contribution to load between In and St= 2.14535918E-04

Contributions to homozygous loads for In and St= 2.14535918E-04 2.14535918E-04

Contributions to inbreeding loads= 0.00000000 0.00000000

Selection coefficients for In and St homokaryotypes

0.00000000 0.00000000

Selection coefficients for In and St homokaryotypes

0.00000000 0.00000000

Contributions to mean A2 freqs= 1.14092863E-05 1.14092863E-05

Contributions to mean diversities= 2.26448774E-05 2.26448774E-05

Contributions to mean proportions of seg. sites= 2.18522342E-04 2.18522953E-04

Contributions to delta-theta values= 0.632357359 0.632358372

Zone 3: strong selection approximation

Lower and upper bounds of St popn gamma= 826.719604 4166.66699

Zone 3: strong selection approximation

Probability of zone 3= 0.175653934

Mean load statistics over zone 3

Contributions to loads within In and St= 2.19132649E-04 2.19132649E-04

Contribution to load between In and St= 2.19132649E-04

Contributions to homozygous loads for In and St= 2.19132649E-04 2.19132649E-04

Contributions to inbreeding loads= 0.00000000 0.00000000

Selection coefficients for In and St homokaryotypes

0.00000000 0.00000000

Contributions to mean A2 freqs= 1.52286782E-06 1.52286782E-06

Contributions to mean A2 freqs at seg. sites= 0.00000000 0.00000000

Contributions to mean diversities= 3.04327364E-06 3.04327364E-06

Contributions to delta-theta values= 0.642794728 0.642794728

Mean load statistics over all zones

Loads within In and St= 4.36741859E-03 4.36741859E-03

Load between In and St= 4.36741812E-03

Homozygous load for In and St= 4.36741859E-03 4.36741859E-03

Inbreeding loads= 0.00000000 0.00000000

Selection coefficients for In and St homokaryotypes

0.00000000 0.00000000

Mean frequencies of A2 in In and St= 7.99297616E-02 7.99297616E-02

Ratio of these= 1.00000000

Mean diversities at selected sites in In and St= 1.72663736E-03 1.72663759E-03

Mean diversities at neutral sites in In and St= 7.86884315E-03 7.86884315E-03

pi-n/pi-s for In and St= 0.219427094 0.219427124

Ratio of these= 0.999999881

Mean freqs. of seg. sites= 7.12674949E-03 7.12674996E-03

Ratio of these= 0.999999940

Overall delta-theta values= 0.140469313 0.140469193

Ratio of these= 1.00000083

**Section 2**

**Mean selection coefficient= 2.0 E-03**

**Mean scaled selection coefficient for whole population= 4000**

**Inversion frequency= 0.1**

**h=0.05**

Zone 1: quasi-neutral zone

Upper bound scaled selection coefficient for neutrality in St metapopulation= 0.250000000

Probability of zone 1= 4.39136960E-02

Integral of selection coefficient over zone 1= 1.40749034E-04

Mean load statistics for zone 1

Mean q1 and q2= 0.600000024

F1 and F2= 0.996677756 0.970873833

Diversities= 1.59467699E-03 1.39805600E-02

Contributions to loads within In and St= 8.43484231E-05 8.35639366E-05

Contribution to load between In and St = 5.40476285E-05

Contributions to homozygous loads for In and St= 8.44494207E-05 8.44494207E-05

Contributions to inbreeding loads for In and St= 0.00000000 0.00000000

Contributions to selection coefficients for In and St homokaryotypes

3.02791595E-05 2.95042992E-05

Contributions to mean A2 freqs= 2.63482183E-02 2.63482183E-02

Contributions to mean diversities= 7.00281598E-05 6.13938086E-04

Contributions to mean freqs. of seg. site= 2.48237309E-04 2.16380460E-03

Delta-theta values= -8.23259354E-04 -6.60312176E-03

Zone 2a: moderate selection; cut-off at moderate gamma for St population

Lower and upper bounds of St popn gamma

0.250000000 250.000000

Probability of zone 2a= 0.303239673

Coefficients for bivariate distribution of q1 and q2

a1= 5.00000035E-03 a2= 4.49999981E-02

b11= 4.50000027E-03 b12= 8.09999928E-02 b22= 0.364499956

Net probability of zone 2a using Simpsons rule= 0.303576410

Contributions to mean load statistics over zone 2a

Contributions to mean loads within In and St= 0.144798964 1.07835303E-03

Contribution to load between In and St= 8.12752452E-03

Contributions to homozygous loads for In and St= 0.145504758 1.95283501E-03

Contributions to inbreeding loads= 7.05582264E-04 8.74482619E-04

Selection coefficients for In and St homokaryotypes

0.127743244 -7.07411766E-03

Selection coefficients for In and St homokaryotypes

0.127743244 -7.07411766E-03

Contributions to mean A2 freqs= 0.105992854 1.72770899E-02

Contributions to mean diversities= 4.80526563E-04 1.79444032E-03

Contributions to mean proportions of seg. sites= 1.73632009E-03 8.06842279E-03

Contributions to delta-theta values= 1.81630254E-02 0.210972548

Zone 2b: moderate selection; gamma for St reaches high value

Lower and upper bounds of St popn gamma

250.000000 2500.00000

Probability of zone 2b 0.317252636

Net probability of zone 2b using Simpsons rule= 0.317252696

Contributions to mean load statistics over zone 2b

Contributions to mean loads within In and St= 2.60605314E-03 1.19566357E-04

Contribution to load between In and St= 2.86194641E-04

Contributions to homozygous loads for In and St= 4.62485664E-03 1.07231678E-03

Contributions to inbreeding loads= 2.01879605E-03 9.52750503E-04

Selection coefficients for In and St homokaryotypes

2.31719017E-03 -1.66654587E-04

Selection coefficients for In and St homokaryotypes

2.31719017E-03 -1.66654587E-04

Contributions to mean A2 freqs= 1.97981601E-04 3.32943637E-05

Contributions to mean diversities= 1.16837240E-04 6.55114636E-05

Contributions to mean proportions of seg. sites= 6.47450215E-04 5.81898668E-04

Contributions to delta-theta values= 0.359783769 0.600587487

Zone 3: strong selection approximation

Lower and upper bounds of St popn gamma= 2500.00000 30000.0020

Zone 3: strong selection approximation

Probability of zone 3= 0.323736906

Mean load statistics over zone 3

Contributions to loads within In and St= 4.03293321E-04 3.58825666E-04

Contribution to load between In and St= 3.53267271E-04

Contributions to homozygous loads for In and St= 3.53167788E-03 3.53167788E-03

Contributions to inbreeding loads= 3.12838401E-03 3.17284628E-03

Selection coefficients for In and St homokaryotypes

5.00082970E-05 5.54323196E-06

Contributions to mean A2 freqs= 1.11169065E-05 1.11169065E-05

Contributions to mean A2 freqs at seg. sites= 0.00000000 0.00000000

Contributions to mean diversities= 2.17678826E-05 2.21812516E-05

Contributions to delta-theta values= 0.566199541 0.638060391

Mean load statistics over all zones

Loads within In and St= 0.147892669 1.64030900E-03

Load between In and St= 8.82103387E-03

Homozygous load for In and St= 0.153745741 6.64127897E-03

Inbreeding loads= 5.85286319E-03 5.00096474E-03

Selection coefficients for In and St homokaryotypes

0.129834294 -7.20655918E-03

Mean frequencies of A2 in In and St= 0.132550165 4.36697192E-02

Ratio of these= 3.03528786

Mean diversities at selected sites in In and St= 6.89159788E-04 2.49607116E-03

Mean diversities at neutral sites in In and St= 1.59467699E-03 1.39805600E-02

pi-n/pi-s for In and St= 0.432162613 0.178538710

Ratio of these= 2.42055416

Mean freqs. of seg. sites= 2.81003118E-03 1.10315466E-02

Ratio of these= 0.254726857

Overall delta-theta values= 0.129917264 0.197264791

Ratio of these= 0.658593297

**h = 0.15**

Zone 1: quasi-neutral zone

Upper bound scaled selection coefficient for neutrality in St metapopulation= 0.250000000

Probability of zone 1= 4.39136960E-02

Integral of selection coefficient over zone 1= 1.40749034E-04

Mean load statistics for zone 1

Mean q1 and q2= 0.600000024

F1 and F2= 0.996677756 0.970873833

Diversities= 1.59467699E-03 1.39805600E-02

Contributions to loads within In and St= 8.43708694E-05 8.37607149E-05

Contribution to load between In and St = 6.08035843E-05

Contributions to homozygous loads for In and St= 8.44494207E-05 8.44494207E-05

Contributions to inbreeding loads for In and St= 8.97294655E-03 8.99694674E-03

Contributions to selection coefficients for In and St homokaryotypes

2.35438347E-05 2.29477882E-05

Contributions to mean A2 freqs= 2.63482183E-02 2.63482183E-02

Contributions to mean diversities= 7.00281598E-05 6.13938086E-04

Contributions to mean freqs. of seg. site= 2.48237309E-04 2.16380460E-03

Delta-theta values= -8.23259354E-04 -6.60312176E-03

Zone 2a: moderate selection; cut-off at moderate gamma for St population

Lower and upper bounds of St popn gamma

0.250000000 416.666656

Probability of zone 2a= 0.359450161

Coefficients for bivariate distribution of q1 and q2

a1= 1.50000006E-02 a2= 0.135000005

b11= 3.50000011E-03 b12= 6.30000010E-02 b22= 0.283499986

Net probability of zone 2a using Simpsons rule= 0.360651016

Contributions to mean load statistics over zone 2a

Contributions to mean loads within In and St= 6.24625124E-02 1.16809004E-03

Contribution to load between In and St= 1.01799266E-02

Contributions to homozygous loads for In and St= 6.29261360E-02 1.66823203E-03

Contributions to inbreeding loads= 4.63562319E-04 5.00139431E-04

Selection coefficients for In and St homokaryotypes

5.09393811E-02 -9.05251503E-03

Selection coefficients for In and St homokaryotypes

5.09393811E-02 -9.05251503E-03

Contributions to mean A2 freqs= 8.30528885E-02 1.77091360E-02

Contributions to mean diversities= 4.23798250E-04 1.56474730E-03

Contributions to mean proportions of seg. sites= 1.64529064E-03 6.98010018E-03

Contributions to delta-theta values= 8.61639977E-02 0.204693913

Zone 2b: moderate selection; gamma for St reaches high value

Lower and upper bounds of St popn gamma

416.666656 4166.66650

Probability of zone 2b 0.348782212

Net probability of zone 2b using Simpsons rule= 0.348782301

Contributions to mean load statistics over zone 2b

Contributions to mean loads within In and St= 2.78607535E-04 8.96285637E-05

Contribution to load between In and St= 1.74651199E-04

Contributions to homozygous loads for In and St= 8.68413132E-04 2.95758218E-04

Contributions to inbreeding loads= 5.89806354E-04 2.06129567E-04

Selection coefficients for In and St homokaryotypes

1.03950500E-04 -8.49962234E-05

Selection coefficients for In and St homokaryotypes

1.03950500E-04 -8.49962234E-05

Contributions to mean A2 freqs= 1.51333370E-05 6.32059300E-06

Contributions to mean diversities= 2.90303342E-05 1.25738234E-05

Contributions to mean proportions of seg. sites= 2.26681776E-04 1.25032893E-04

Contributions to delta-theta values= 0.545653462 0.643224657

Zone 3: strong selection approximation

Lower and upper bounds of St popn gamma= 4166.66650 30000.0020

Zone 3: strong selection approximation

Probability of zone 3= 0.235996842

Mean load statistics over zone 3

Contributions to loads within In and St= 2.76434963E-04 2.74328486E-04

Contribution to load between In and St= 2.74065358E-04

Contributions to homozygous loads for In and St= 9.13536234E-04 9.13536234E-04

Contributions to inbreeding loads= 6.37100369E-04 6.39206788E-04

Selection coefficients for In and St homokaryotypes

2.38418579E-06 2.38418579E-07

Contributions to mean A2 freqs= 2.03110994E-06 2.03110972E-06

Contributions to mean A2 freqs at seg. sites= 0.00000000 0.00000000

Contributions to mean diversities= 4.04389766E-06 4.06015624E-06

Contributions to delta-theta values= 0.631019473 0.643712282

Mean load statistics over all zones

Loads within In and St= 6.31019250E-02 1.61580788E-03

Load between In and St= 1.06894458E-02

Homozygous load for In and St= 6.47925362E-02 2.96197576E-03

Inbreeding loads= 1.69054768E-03 1.34616450E-03

Selection coefficients for In and St homokaryotypes

5.10626435E-02 -9.11498070E-03

Mean frequencies of A2 in In and St= 0.109418273 4.40657064E-02

Ratio of these= 2.48307085

Mean diversities at selected sites in In and St= 5.26900636E-04 2.19531916E-03

Mean diversities at neutral sites in In and St= 1.59467699E-03 1.39805600E-02

pi-n/pi-s for In and St= 0.330412149 0.157026559

Ratio of these= 2.10418010

Mean freqs. of seg. sites= 2.15909164E-03 9.30936635E-03

Ratio of these= 0.231926814

Overall delta-theta values= 0.134216249 0.163377941

Ratio of these= 0.821507752

**h = 0.25**

Zone 1: quasi-neutral zone

Upper bound scaled selection coefficient for neutrality in St metapopulation= 0.250000000

Probability of zone 1= 4.39136960E-02

Integral of selection coefficient over zone 1= 1.40749034E-04

Mean load statistics for zone 1

Mean q1 and q2= 0.600000024

F1 and F2= 0.996677756 0.970873833

Diversities= 1.59467699E-03 1.39805600E-02

Contributions to loads within In and St= 8.43933085E-05 8.39574859E-05

Contribution to load between In and St = 6.75595365E-05

Contributions to homozygous loads for In and St= 8.44494207E-05 8.44494207E-05

Contributions to inbreeding loads for In and St= 2.33099516E-03 2.33306922E-03

Contributions to selection coefficients for In and St homokaryotypes

1.68085098E-05 1.63912773E-05

Contributions to mean A2 freqs= 2.63482183E-02 2.63482183E-02

Contributions to mean diversities= 7.00281598E-05 6.13938086E-04

Contributions to mean freqs. of seg. site= 2.48237309E-04 2.16380460E-03

Delta-theta values= -8.23259354E-04 -6.60312176E-03

Zone 2a: moderate selection; cut-off at moderate gamma for St population

Lower and upper bounds of St popn gamma

0.250000000 416.666656

Probability of zone 2a= 0.359450161

Coefficients for bivariate distribution of q1 and q2

a1= 2.50000004E-02 a2= 0.224999994

b11= 2.50000018E-03 b12= 4.49999981E-02 b22= 0.202499986

Net probability of zone 2a using Simpsons rule= 0.360651016

Contributions to mean load statistics over zone 2a

Contributions to mean loads within In and St= 3.75116058E-02 1.19144039E-03

Contribution to load between In and St= 1.01157073E-02

Contributions to homozygous loads for In and St= 3.77420783E-02 1.44041365E-03

Contributions to inbreeding loads= 2.30456062E-04 2.48971890E-04

Selection coefficients for In and St homokaryotypes

2.70240307E-02 -8.96418095E-03

Selection coefficients for In and St homokaryotypes

2.70240307E-02 -8.96418095E-03

Contributions to mean A2 freqs= 7.10945129E-02 1.76453646E-02

Contributions to mean diversities= 3.69848422E-04 1.38664292E-03

Contributions to mean proportions of seg. sites= 1.47155253E-03 6.13316381E-03

Contributions to delta-theta values= 0.108339012 0.197893918

Zone 2b: moderate selection; gamma for St reaches high value

Lower and upper bounds of St popn gamma

416.666656 4166.66650

Probability of zone 2b 0.348782212

Net probability of zone 2b using Simpsons rule= 0.348782301

Contributions to mean load statistics over zone 2b

Contributions to mean loads within In and St= 2.60749890E-04 7.98195397E-05

Contribution to load between In and St= 1.67836886E-04

Contributions to homozygous loads for In and St= 5.12157683E-04 1.59165837E-04

Contributions to inbreeding loads= 2.51408143E-04 7.93462023E-05

Selection coefficients for In and St homokaryotypes

9.29236412E-05 -8.79764557E-05

Selection coefficients for In and St homokaryotypes

9.29236412E-05 -8.79764557E-05

Contributions to mean A2 freqs= 8.95962967E-06 3.53542873E-06

Contributions to mean diversities= 1.74721117E-05 7.04574768E-06

Contributions to mean proportions of seg. sites= 1.48217485E-04 7.33559646E-05

Contributions to delta-theta values= 0.581786811 0.659244061

Zone 3: strong selection approximation

Lower and upper bounds of St popn gamma= 4166.66650 30000.0020

Zone 3: strong selection approximation

Probability of zone 3= 0.235996842

Mean load statistics over zone 3

Contributions to loads within In and St= 2.74671271E-04 2.74129619E-04

Contribution to load between In and St= 2.74061837E-04

Contributions to homozygous loads for In and St= 5.48121461E-04 5.48121403E-04

Contributions to inbreeding loads= 2.73450016E-04 2.73991580E-04

Selection coefficients for In and St homokaryotypes

5.96046448E-07 5.96046448E-08

Contributions to mean A2 freqs= 1.21866731E-06 1.21866719E-06

Contributions to mean A2 freqs at seg. sites= 0.00000000 0.00000000

Contributions to mean diversities= 2.43073578E-06 2.43658837E-06

Contributions to delta-theta values= 0.636855721 0.644324601

Mean load statistics over all zones

Loads within In and St= 3.81314233E-02 1.62934698E-03

Load between In and St= 1.06251659E-02

Homozygous load for In and St= 3.88868041E-02 2.23215017E-03

Inbreeding loads= 7.55370362E-04 6.02801621E-04

Selection coefficients for In and St homokaryotypes

2.71313787E-02 -9.03642178E-03

Mean frequencies of A2 in In and St= 9.74529162E-02 4.39983383E-02

Ratio of these= 2.21492267

Mean diversities at selected sites in In and St= 4.59779403E-04 2.01006327E-03

Mean diversities at neutral sites in In and St= 1.59467699E-03 1.39805600E-02

pi-n/pi-s for In and St= 0.288321346 0.143775597

Ratio of these= 2.00535655

Mean freqs. of seg. sites= 1.89175433E-03 8.39462783E-03

Ratio of these= 0.225352973

Overall delta-theta values= 0.137743413 0.150506616

Ratio of these= 0.915198386

**h = 0.35**

Zone 1: quasi-neutral zone

Upper bound scaled selection coefficient for neutrality in St metapopulation= 0.250000000

Probability of zone 1= 4.39136960E-02

Integral of selection coefficient over zone 1= 1.40749034E-04

Mean load statistics for zone 1

Mean q1 and q2= 0.600000024

F1 and F2= 0.996677756 0.970873833

Diversities= 1.59467699E-03 1.39805600E-02

Contributions to loads within In and St= 8.44157548E-05 8.41542642E-05

Contribution to load between In and St = 7.43154887E-05

Contributions to homozygous loads for In and St= 8.44494207E-05 8.44494207E-05

Contributions to inbreeding loads for In and St= 9.99398762E-04 9.99932061E-04

Contributions to selection coefficients for In and St homokaryotypes

1.00731850E-05 9.83476639E-06

Contributions to mean A2 freqs= 2.63482183E-02 2.63482183E-02

Contributions to mean diversities= 7.00281598E-05 6.13938086E-04

Contributions to mean freqs. of seg. site= 2.48237309E-04 2.16380460E-03

Delta-theta values= -8.23259354E-04 -6.60312176E-03

Zone 2a: moderate selection; cut-off at moderate gamma for St population

Lower and upper bounds of St popn gamma

0.250000000 297.619049

Probability of zone 2a= 0.321548969

Coefficients for bivariate distribution of q1 and q2

a1= 3.50000001E-02 a2= 0.314999998

b11= 1.50000013E-03 b12= 2.70000007E-02 b22= 0.121499993

Net probability of zone 2a using Simpsons rule= 0.322079927

Contributions to mean load statistics over zone 2a

Contributions to mean loads within In and St= 2.60416325E-02 1.18368689E-03

Contribution to load between In and St= 9.77961440E-03

Contributions to homozygous loads for In and St= 2.61329133E-02 1.28853542E-03

Contributions to inbreeding loads= 9.12812611E-05 1.04850311E-04

Selection coefficients for In and St homokaryotypes

1.61305070E-02 -8.63301754E-03

Selection coefficients for In and St homokaryotypes

1.61305070E-02 -8.63301754E-03

Contributions to mean A2 freqs= 6.30173460E-02 1.71921216E-02

Contributions to mean diversities= 3.28298425E-04 1.24082447E-03

Contributions to mean proportions of seg. sites= 1.30812416E-03 5.46321692E-03

Contributions to delta-theta values= 0.109627843 0.194225252

Zone 2b: moderate selection; gamma for St reaches high value

Lower and upper bounds of St popn gamma

297.619049 2976.19043

Probability of zone 2b 0.328705907

Net probability of zone 2b using Simpsons rule= 0.328705996

Contributions to mean load statistics over zone 2b

Contributions to mean loads within In and St= 2.61239009E-04 9.07608774E-05

Contribution to load between In and St= 1.74957808E-04

Contributions to homozygous loads for In and St= 3.70374328E-04 1.29496257E-04

Contributions to inbreeding loads= 1.09135326E-04 3.87353975E-05

Selection coefficients for In and St homokaryotypes

8.62479210E-05 -8.41617584E-05

Selection coefficients for In and St homokaryotypes

8.62479210E-05 -8.41617584E-05

Contributions to mean A2 freqs= 8.79437721E-06 3.91493495E-06

Contributions to mean diversities= 1.71545253E-05 7.80227674E-06

Contributions to mean proportions of seg. sites= 1.45696555E-04 8.14365267E-05

Contributions to delta-theta values= 0.582283914 0.660097897

Zone 3: strong selection approximation

Lower and upper bounds of St popn gamma= 2976.19043 30000.0020

Zone 3: strong selection approximation

Probability of zone 3= 0.293974340

Mean load statistics over zone 3

Contributions to loads within In and St= 3.26630543E-04 3.26381793E-04

Contribution to load between In and St= 3.26350710E-04

Contributions to homozygous loads for In and St= 4.66214406E-04 4.66214406E-04

Contributions to inbreeding loads= 1.39584299E-04 1.39832729E-04

Selection coefficients for In and St homokaryotypes

2.98023224E-07 5.96046448E-08

Contributions to mean A2 freqs= 1.30430715E-06 1.30430715E-06

Contributions to mean A2 freqs at seg. sites= 0.00000000 0.00000000

Contributions to mean diversities= 2.60189495E-06 2.60785600E-06

Contributions to delta-theta values= 0.637255788 0.644360304

Mean load statistics over all zones

Loads within In and St= 2.67139170E-02 1.68498384E-03

Load between In and St= 1.03552388E-02

Homozygous load for In and St= 2.70539504E-02 1.96869555E-03

Inbreeding loads= 3.40034545E-04 2.83713598E-04

Selection coefficients for In and St homokaryotypes

1.62255764E-02 -8.70800018E-03

Mean frequencies of A2 in In and St= 8.93756598E-02 4.35455590E-02

Ratio of these= 2.05246329

Mean diversities at selected sites in In and St= 4.18082986E-04 1.86517264E-03

Mean diversities at neutral sites in In and St= 1.59467699E-03 1.39805600E-02

pi-n/pi-s for In and St= 0.262174100 0.133411869

Ratio of these= 1.96514821

Mean freqs. of seg. sites= 1.72750524E-03 7.73447333E-03

Ratio of these= 0.223351374

Overall delta-theta values= 0.141392112 0.144460559

Ratio of these= 0.978759289

**h = 0.45**

Zone 1: quasi-neutral zone

Upper bound scaled selection coefficient for neutrality in St metapopulation= 0.250000000

Probability of zone 1= 4.39136960E-02

Integral of selection coefficient over zone 1= 1.40749034E-04

Mean load statistics for zone 1

Mean q1 and q2= 0.600000024

F1 and F2= 0.996677756 0.970873833

Diversities= 1.59467699E-03 1.39805600E-02

Contributions to loads within In and St= 8.44382012E-05 8.43510352E-05

Contribution to load between In and St = 8.10714409E-05

Contributions to homozygous loads for In and St= 8.44494207E-05 8.44494207E-05

Contributions to inbreeding loads for In and St= 4.28387430E-04 4.28550673E-04

Contributions to selection coefficients for In and St homokaryotypes

3.33786011E-06 3.27825546E-06

Contributions to mean A2 freqs= 2.63482183E-02 2.63482183E-02

Contributions to mean diversities= 7.00281598E-05 6.13938086E-04

Contributions to mean freqs. of seg. site= 2.48237309E-04 2.16380460E-03

Delta-theta values= -8.23259354E-04 -6.60312176E-03

Zone 2a: moderate selection; cut-off at moderate gamma for St population

Lower and upper bounds of St popn gamma

0.250000000 165.343918

Probability of zone 2a= 0.263242185

Coefficients for bivariate distribution of q1 and q2

a1= 4.49999981E-02 a2= 0.404999971

b11= 5.00000140E-04 b12= 9.00000241E-03 b22= 4.05000076E-02

Net probability of zone 2a using Simpsons rule= 0.263346374

Contributions to mean load statistics over zone 2a

Contributions to mean loads within In and St= 1.95781812E-02 1.15600158E-03

Contribution to load between In and St= 9.40879714E-03

Contributions to homozygous loads for In and St= 1.95969064E-02 1.17990945E-03

Contributions to inbreeding loads= 1.87308779E-05 2.39077563E-05

Selection coefficients for In and St homokaryotypes

1.01178288E-02 -8.28695297E-03

Selection coefficients for In and St homokaryotypes

1.01178288E-02 -8.28695297E-03

Contributions to mean A2 freqs= 5.72704971E-02 1.69101860E-02

Contributions to mean diversities= 2.92938406E-04 1.12340390E-03

Contributions to mean proportions of seg. sites= 1.14881189E-03 4.92153224E-03

Contributions to delta-theta values= 9.53530073E-02 0.190182149

Zone 2b: moderate selection; gamma for St reaches high value

Lower and upper bounds of St popn gamma

165.343918 1653.43921

Probability of zone 2b 0.288836628

Net probability of zone 2b using Simpsons rule= 0.288836628

Contributions to mean load statistics over zone 2b

Contributions to mean loads within In and St= 2.55217048E-04 1.16025105E-04

Contribution to load between In and St= 1.85258381E-04

Contributions to homozygous loads for In and St= 2.82817666E-04 1.28864587E-04

Contributions to inbreeding loads= 2.76006922E-05 1.28394904E-05

Selection coefficients for In and St homokaryotypes

6.99758530E-05 -6.92605972E-05

Selection coefficients for In and St homokaryotypes

6.99758530E-05 -6.92605972E-05

Contributions to mean A2 freqs= 1.15942375E-05 6.47513889E-06

Contributions to mean diversities= 2.23947063E-05 1.28911388E-05

Contributions to mean proportions of seg. sites= 1.80585019E-04 1.30582921E-04

Contributions to delta-theta values= 0.560037732 0.649767339

Zone 3: strong selection approximation

Lower and upper bounds of St popn gamma= 1653.43921 30000.0020

Zone 3: strong selection approximation

Probability of zone 3= 0.392150402

Mean load statistics over zone 3

Contributions to loads within In and St= 4.15130722E-04 4.15035989E-04

Contribution to load between In and St= 4.15024464E-04

Contributions to homozygous loads for In and St= 4.61137737E-04 4.61137650E-04

Contributions to inbreeding loads= 4.60072733E-05 4.61017626E-05

Selection coefficients for In and St homokaryotypes

1.19209290E-07 0.00000000

Contributions to mean A2 freqs= 1.91432878E-06 1.91432878E-06

Contributions to mean A2 freqs at seg. sites= 0.00000000 0.00000000

Contributions to mean diversities= 3.81587597E-06 3.82721146E-06

Contributions to delta-theta values= 0.634809017 0.644107223

Mean load statistics over all zones

Loads within In and St= 2.03329679E-02 1.77141372E-03

Load between In and St= 1.00901518E-02

Homozygous load for In and St= 2.04253104E-02 1.85436103E-03

Inbreeding loads= 9.23500629E-05 8.29474011E-05

Selection coefficients for In and St homokaryotypes

1.01905465E-02 -8.35347176E-03

Mean frequencies of A2 in In and St= 8.36322233E-02 4.32667956E-02

Ratio of these= 1.93294239

Mean diversities at selected sites in In and St= 3.89177119E-04 1.75406027E-03

Mean diversities at neutral sites in In and St= 1.59467699E-03 1.39805600E-02

pi-n/pi-s for In and St= 0.244047612 0.125464231

Ratio of these= 1.94515693

Mean freqs. of seg. sites= 1.61470438E-03 7.25407153E-03

Ratio of these= 0.222592846

Overall delta-theta values= 0.144921422 0.142143905

Ratio of these= 1.01954019

**h = 0.5**

Zone 1: quasi-neutral zone

Upper bound scaled selection coefficient for neutrality in St metapopulation= 0.250000000

Probability of zone 1= 4.39136960E-02

Integral of selection coefficient over zone 1= 1.40749034E-04

Mean load statistics for zone 1

Mean q1 and q2= 0.600000024

F1 and F2= 0.996677756 0.970873833

Diversities= 1.59467699E-03 1.39805600E-02

Contributions to loads within In and St= 8.44494207E-05 8.44494207E-05

Contribution to load between In and St = 8.44494207E-05

Contributions to homozygous loads for In and St= 8.44494207E-05 8.44494207E-05

Contributions to inbreeding loads for In and St= 1.11074034E-04 1.11107001E-04

Contributions to selection coefficients for In and St homokaryotypes

0.00000000 0.00000000

Contributions to mean A2 freqs= 2.63482183E-02 2.63482183E-02

Contributions to mean diversities= 7.00281598E-05 6.13938086E-04

Contributions to mean freqs. of seg. site= 2.48237309E-04 2.16380460E-03

Delta-theta values= -8.23259354E-04 -6.60312176E-03

Zone 2a: moderate selection; cut-off at moderate gamma for St population

Lower and upper bounds of St popn gamma

0.250000000 82.6719589

Probability of zone 2a= 0.205970049

Coefficients for bivariate distribution of q1 and q2

a1= 5.00000007E-02 a2= 0.449999988

b11= 0.00000000 b12= 0.00000000 b22= 0.00000000

Net probability of zone 2a using Simpsons rule= 0.205981016

Contributions to mean load statistics over zone 2a

Contributions to mean loads within In and St= 1.72838997E-02 1.11394655E-03

Contribution to load between In and St= 9.19892080E-03

Contributions to homozygous loads for In and St= 1.72838997E-02 1.11394655E-03

Contributions to inbreeding loads= 0.00000000 0.00000000

Selection coefficients for In and St homokaryotypes

8.05240870E-03 -8.11779499E-03

Selection coefficients for In and St homokaryotypes

8.05240870E-03 -8.11779499E-03

Contributions to mean A2 freqs= 5.49828373E-02 1.68425385E-02

Contributions to mean diversities= 2.65979877E-04 1.06400473E-03

Contributions to mean proportions of seg. sites= 1.00775750E-03 4.59504407E-03

Contributions to delta-theta values= 6.36364818E-02 0.178503633

Zone 2b: moderate selection; gamma for St reaches high value

Lower and upper bounds of St popn gamma

82.6719589 826.719604

Probability of zone 2b 0.241704941

Net probability of zone 2b using Simpsons rule= 0.241705000

Contributions to mean load statistics over zone 2b

Contributions to mean loads within In and St= 2.41919304E-04 1.41345969E-04

Contribution to load between In and St= 1.91632658E-04

Contributions to homozygous loads for In and St= 2.41919304E-04 1.41345969E-04

Contributions to inbreeding loads= 0.00000000 0.00000000

Selection coefficients for In and St homokaryotypes

5.03063202E-05 -5.03063202E-05

Selection coefficients for In and St homokaryotypes

5.03063202E-05 -5.03063202E-05

Contributions to mean A2 freqs= 2.00515260E-05 1.28585007E-05

Contributions to mean diversities= 3.53627474E-05 2.55247924E-05

Contributions to mean proportions of seg. sites= 2.53451290E-04 2.46523530E-04

Contributions to delta-theta values= 0.505002201 0.632670641

Zone 3: strong selection approximation

Lower and upper bounds of St popn gamma= 826.719604 30000.0020

Zone 3: strong selection approximation

Probability of zone 3= 0.496554226

Mean load statistics over zone 3

Contributions to loads within In and St= 5.10625541E-04 5.10625541E-04

Contribution to load between In and St= 5.10625541E-04

Contributions to homozygous loads for In and St= 5.10625541E-04 5.10625541E-04

Contributions to inbreeding loads= 0.00000000 0.00000000

Selection coefficients for In and St homokaryotypes

0.00000000 0.00000000

Contributions to mean A2 freqs= 3.34678498E-06 3.34678498E-06

Contributions to mean A2 freqs at seg. sites= 0.00000000 0.00000000

Contributions to mean diversities= 6.65635025E-06 6.68936445E-06

Contributions to delta-theta values= 0.627378941 0.643340349

Mean load statistics over all zones

Loads within In and St= 1.81208923E-02 1.85036752E-03

Load between In and St= 9.98562854E-03

Homozygous load for In and St= 1.81208923E-02 1.85036752E-03

Inbreeding loads= 0.00000000 0.00000000

Selection coefficients for In and St homokaryotypes

8.10223818E-03 -8.16845894E-03

Mean frequencies of A2 in In and St= 8.13544542E-02 4.32069600E-02

Ratio of these= 1.88290155

Mean diversities at selected sites in In and St= 3.78027122E-04 1.71015691E-03

Mean diversities at neutral sites in In and St= 1.59467699E-03 1.39805600E-02

pi-n/pi-s for In and St= 0.237055615 0.122323923

Ratio of these= 1.93793344

Mean freqs. of seg. sites= 1.57282141E-03 7.07191229E-03

Ratio of these= 0.222403973

Overall delta-theta values= 0.147301912 0.142072022

Ratio of these= 1.03681159

**Inversion frequency = 0.3**

**h=0.05**

Zone 1: quasi-neutral zone

Upper bound scaled selection coefficient for neutrality in St metapopulation= 0.250000000

Probability of zone 1= 4.73524816E-02

Integral of selection coefficient over zone 1= 1.95133864E-04

Mean load statistics for zone 1

Mean q1 and q2= 0.600000024

F1 and F2= 0.990099013 0.977198601

Diversities= 4.75247391E-03 1.09446710E-02

Contributions to loads within In and St= 1.16663010E-04 1.16119263E-04

Contribution to load between In and St = 7.49314058E-05

Contributions to homozygous loads for In and St= 1.17080323E-04 1.17080323E-04

Contributions to inbreeding loads for In and St= 0.00000000 0.00000000

Contributions to selection coefficients for In and St homokaryotypes

4.17232513E-05 4.11868095E-05

Contributions to mean A2 freqs= 2.84114908E-02 2.84114908E-02

Contributions to mean diversities= 2.25041440E-04 5.18257322E-04

Contributions to mean freqs. of seg. site= 7.96483480E-04 1.82903488E-03

Delta-theta values= -2.39169598E-03 -5.25259972E-03

Zone 2a: moderate selection; cut-off at moderate gamma for St population

Lower and upper bounds of St popn gamma

0.250000000 250.000000

Probability of zone 2a= 0.326476455

Coefficients for bivariate distribution of q1 and q2

a1= 1.50000006E-02 a2= 3.50000001E-02

b11= 4.05000001E-02 b12= 0.189000010 b22= 0.220499992

Net probability of zone 2a using Simpsons rule= 0.326839477

Contributions to mean load statistics over zone 2a

Contributions to mean loads within In and St= 1.47714987E-02 1.55137677E-03

Contribution to load between In and St= 1.57297438E-03

Contributions to homozygous loads for In and St= 1.58853680E-02 2.62762606E-03

Contributions to inbreeding loads= 1.11386389E-03 1.07624696E-03

Selection coefficients for In and St homokaryotypes

1.31118298E-02 -2.15768814E-05

Selection coefficients for In and St homokaryotypes

1.31118298E-02 -2.15768814E-05

Contributions to mean A2 freqs= 4.82889153E-02 1.87343676E-02

Contributions to mean diversities= 1.09254161E-03 1.57900981E-03

Contributions to mean proportions of seg. sites= 4.34190175E-03 7.13270577E-03

Contributions to delta-theta values= 0.107291400 0.214615524

Zone 2b: moderate selection; gamma for St reaches high value

Lower and upper bounds of St popn gamma

250.000000 2500.00000

Probability of zone 2b 0.333575726

Net probability of zone 2b using Simpsons rule= 0.333575726

Contributions to mean load statistics over zone 2b

Contributions to mean loads within In and St= 2.17786597E-04 1.28400323E-04

Contribution to load between In and St= 1.43423618E-04

Contributions to homozygous loads for In and St= 1.71851635E-03 1.14420999E-03

Contributions to inbreeding loads= 1.50072901E-03 1.01580983E-03

Selection coefficients for In and St homokaryotypes

7.43865967E-05 -1.50203705E-05

Selection coefficients for In and St homokaryotypes

7.43865967E-05 -1.50203705E-05

Contributions to mean A2 freqs= 3.92737456E-05 2.80397235E-05

Contributions to mean diversities= 7.55018045E-05 5.51147641E-05

Contributions to mean proportions of seg. sites= 5.87588351E-04 4.87001846E-04

Contributions to delta-theta values= 0.544135332 0.598496735

Zone 3: strong selection approximation

Lower and upper bounds of St popn gamma= 2500.00000 23333.3340

Zone 3: strong selection approximation

Probability of zone 3= 0.280738235

Mean load statistics over zone 3

Contributions to loads within In and St= 3.27024231E-04 3.19868792E-04

Contribution to load between In and St= 3.14501900E-04

Contributions to homozygous loads for In and St= 3.14426585E-03 3.14426585E-03

Contributions to inbreeding loads= 2.81724404E-03 2.82440125E-03

Selection coefficients for In and St homokaryotypes

1.25169754E-05 5.36441803E-06

Contributions to mean A2 freqs= 8.34837829E-06 8.34837829E-06

Contributions to mean A2 freqs at seg. sites= 0.00000000 0.00000000

Contributions to mean diversities= 1.66024147E-05 1.66559876E-05

Contributions to delta-theta values= 0.627306700 0.637799382

Mean load statistics over all zones

Loads within In and St= 1.54329734E-02 2.11576512E-03

Load between In and St= 2.10583117E-03

Homozygous load for In and St= 2.08652299E-02 7.03318231E-03

Inbreeding loads= 5.43225463E-03 4.91741905E-03

Selection coefficients for In and St homokaryotypes

1.32387280E-02 9.95397568E-06

Mean frequencies of A2 in In and St= 7.67480284E-02 4.71822470E-02

Ratio of these= 1.62662935

Mean diversities at selected sites in In and St= 1.40968733E-03 2.16903794E-03

Mean diversities at neutral sites in In and St= 4.75247391E-03 1.09446710E-02

pi-n/pi-s for In and St= 0.296621799 0.198182106

Ratio of these= 1.49671328

Mean freqs. of seg. sites= 5.88401500E-03 9.61188693E-03

Ratio of these= 0.612160265

Overall delta-theta values= 0.150035501 0.199409842

Ratio of these= 0.752397656

**h = 0.15**

Zone 1: quasi-neutral zone

Upper bound scaled selection coefficient for neutrality in St metapopulation= 0.250000000

Probability of zone 1= 4.73524816E-02

Integral of selection coefficient over zone 1= 1.95133864E-04

Mean load statistics for zone 1

Mean q1 and q2= 0.600000024

F1 and F2= 0.990099013 0.977198601

Diversities= 4.75247391E-03 1.09446710E-02

Contributions to loads within In and St= 1.16755742E-04 1.16332827E-04

Contribution to load between In and St = 8.42978334E-05

Contributions to homozygous loads for In and St= 1.17080323E-04 1.17080323E-04

Contributions to inbreeding loads for In and St= 8.99094623E-03 8.99608899E-03

Contributions to selection coefficients for In and St homokaryotypes

3.24845314E-05 3.20076942E-05

Contributions to mean A2 freqs= 2.84114908E-02 2.84114908E-02

Contributions to mean diversities= 2.25041440E-04 5.18257322E-04

Contributions to mean freqs. of seg. site= 7.96483480E-04 1.82903488E-03

Delta-theta values= -2.39169598E-03 -5.25259972E-03

Zone 2a: moderate selection; cut-off at moderate gamma for St population

Lower and upper bounds of St popn gamma

0.250000000 416.666656

Probability of zone 2a= 0.386616915

Coefficients for bivariate distribution of q1 and q2

a1= 4.50000018E-02 a2= 0.105000004

b11= 3.15000005E-02 b12= 0.147000000 b22= 0.171499997

Net probability of zone 2a using Simpsons rule= 0.387911767

Contributions to mean load statistics over zone 2a

Contributions to mean loads within In and St= 9.85228736E-03 1.65368058E-03

Contribution to load between In and St= 2.38143583E-03

Contributions to homozygous loads for In and St= 1.04381656E-02 2.21359613E-03

Contributions to inbreeding loads= 5.85876347E-04 5.59911365E-04

Selection coefficients for In and St homokaryotypes

7.44301081E-03 -7.28011131E-04

Selection coefficients for In and St homokaryotypes

7.44301081E-03 -7.28011131E-04

Contributions to mean A2 freqs= 4.22943532E-02 1.92890000E-02

Contributions to mean diversities= 8.76445032E-04 1.34401897E-03

Contributions to mean proportions of seg. sites= 3.64777166E-03 5.97947463E-03

Contributions to delta-theta values= 0.147589505 0.202567160

Zone 2b: moderate selection; gamma for St reaches high value

Lower and upper bounds of St popn gamma

416.666656 4166.66650

Probability of zone 2b 0.360976309

Net probability of zone 2b using Simpsons rule= 0.360976666

Contributions to mean load statistics over zone 2b

Contributions to mean loads within In and St= 1.60868527E-04 9.23142070E-05

Contribution to load between In and St= 1.24509766E-04

Contributions to homozygous loads for In and St= 5.25389158E-04 3.04581248E-04

Contributions to inbreeding loads= 3.64520471E-04 2.12266867E-04

Selection coefficients for In and St homokaryotypes

3.63588333E-05 -3.21865082E-05

Selection coefficients for In and St homokaryotypes

3.63588333E-05 -3.21865082E-05

Contributions to mean A2 freqs= 8.05077070E-06 5.11183634E-06

Contributions to mean diversities= 1.59185038E-05 1.01686483E-05

Contributions to mean proportions of seg. sites= 1.48293359E-04 1.01239682E-04

Contributions to delta-theta values= 0.619168997 0.643660307

Zone 3: strong selection approximation

Lower and upper bounds of St popn gamma= 4166.66650 23333.3340

Zone 3: strong selection approximation

Probability of zone 3= 0.193197191

Mean load statistics over zone 3

Contributions to loads within In and St= 2.35769941E-04 2.35444124E-04

Contribution to load between In and St= 2.35199812E-04

Contributions to homozygous loads for In and St= 7.83988042E-04 7.83988042E-04

Contributions to inbreeding loads= 5.48218028E-04 5.48543932E-04

Selection coefficients for In and St homokaryotypes

5.96046448E-07 2.38418579E-07

Contributions to mean A2 freqs= 1.46546483E-06 1.46546483E-06

Contributions to mean A2 freqs at seg. sites= 0.00000000 0.00000000

Contributions to mean diversities= 2.92734876E-06 2.92938216E-06

Contributions to delta-theta values= 0.641525745 0.643655658

Mean load statistics over all zones

Loads within In and St= 1.03656817E-02 2.09777174E-03

Load between In and St= 2.82544317E-03

Homozygous load for In and St= 1.18646231E-02 3.41924583E-03

Inbreeding loads= 1.49893947E-03 1.32146967E-03

Selection coefficients for In and St homokaryotypes

7.51185417E-03 -7.27891922E-04

Mean frequencies of A2 in In and St= 7.07153678E-02 4.77070659E-02

Ratio of these= 1.48228288

Mean diversities at selected sites in In and St= 1.12033240E-03 1.87537435E-03

Mean diversities at neutral sites in In and St= 4.75247391E-03 1.09446710E-02

pi-n/pi-s for In and St= 0.235736668 0.171350449

Ratio of these= 1.37575746

Mean freqs. of seg. sites= 4.62151971E-03 7.93891307E-03

Ratio of these= 0.582135081

Overall delta-theta values= 0.139969528 0.161933124

Ratio of these= 0.864366233

**h = 0.25**

Zone 1: quasi-neutral zone

Upper bound scaled selection coefficient for neutrality in St metapopulation= 0.250000000

Probability of zone 1= 4.73524816E-02

Integral of selection coefficient over zone 1= 1.95133864E-04

Mean load statistics for zone 1

Mean q1 and q2= 0.600000024

F1 and F2= 0.990099013 0.977198601

Diversities= 4.75247391E-03 1.09446710E-02

Contributions to loads within In and St= 1.16848481E-04 1.16546405E-04

Contribution to load between In and St = 9.36642609E-05

Contributions to homozygous loads for In and St= 1.17080323E-04 1.17080323E-04

Contributions to inbreeding loads for In and St= 2.33255094E-03 2.33299518E-03

Contributions to selection coefficients for In and St homokaryotypes

2.31862068E-05 2.28881836E-05

Contributions to mean A2 freqs= 2.84114908E-02 2.84114908E-02

Contributions to mean diversities= 2.25041440E-04 5.18257322E-04

Contributions to mean freqs. of seg. site= 7.96483480E-04 1.82903488E-03

Delta-theta values= -2.39169598E-03 -5.25259972E-03

Zone 2a: moderate selection; cut-off at moderate gamma for St population

Lower and upper bounds of St popn gamma

0.250000000 416.666656

Probability of zone 2a= 0.386616915

Coefficients for bivariate distribution of q1 and q2

a1= 7.50000030E-02 a2= 0.174999997

b11= 2.25000009E-02 b12= 0.105000004 b22= 0.122499995

Net probability of zone 2a using Simpsons rule= 0.387911767

Contributions to mean load statistics over zone 2a

Contributions to mean loads within In and St= 7.30897440E-03 1.69902400E-03

Contribution to load between In and St= 2.74170586E-03

Contributions to homozygous loads for In and St= 7.59049272E-03 1.97075284E-03

Contributions to inbreeding loads= 2.81518500E-04 2.71730620E-04

Selection coefficients for In and St homokaryotypes

4.55683470E-03 -1.04320049E-03

Selection coefficients for In and St homokaryotypes

4.55683470E-03 -1.04320049E-03

Contributions to mean A2 freqs= 3.80297564E-02 1.93578098E-02

Contributions to mean diversities= 7.46769074E-04 1.17420987E-03

Contributions to mean proportions of seg. sites= 3.15671880E-03 5.20417141E-03

Contributions to delta-theta values= 0.160729051 0.199528515

Zone 2b: moderate selection; gamma for St reaches high value

Lower and upper bounds of St popn gamma

416.666656 4166.66650

Probability of zone 2b 0.360976309

Net probability of zone 2b using Simpsons rule= 0.360976666

Contributions to mean load statistics over zone 2b

Contributions to mean loads within In and St= 1.51030850E-04 8.22301954E-05

Contribution to load between In and St= 1.16084200E-04

Contributions to homozygous loads for In and St= 3.00353131E-04 1.63970122E-04

Contributions to inbreeding loads= 1.49322135E-04 8.17399778E-05

Selection coefficients for In and St homokaryotypes

3.49283218E-05 -3.38554382E-05

Selection coefficients for In and St homokaryotypes

3.49283218E-05 -3.38554382E-05

Contributions to mean A2 freqs= 4.69220549E-06 2.85500300E-06

Contributions to mean diversities= 9.31675368E-06 5.68967562E-06

Contributions to mean proportions of seg. sites= 9.06387795E-05 5.93371769E-05

Contributions to delta-theta values= 0.635328054 0.659817219

Zone 3: strong selection approximation

Lower and upper bounds of St popn gamma= 4166.66650 23333.3340

Zone 3: strong selection approximation

Probability of zone 3= 0.193197191

Mean load statistics over zone 3

Contributions to loads within In and St= 2.35343978E-04 2.35260188E-04

Contribution to load between In and St= 2.35197353E-04

Contributions to homozygous loads for In and St= 4.70392959E-04 4.70392959E-04

Contributions to inbreeding loads= 2.35049156E-04 2.35132815E-04

Selection coefficients for In and St homokaryotypes

1.19209290E-07 5.96046448E-08

Contributions to mean A2 freqs= 8.79278844E-07 8.79278844E-07

Contributions to mean A2 freqs at seg. sites= 0.00000000 0.00000000

Contributions to mean diversities= 1.75726757E-06 1.75800017E-06

Contributions to delta-theta values= 0.643016875 0.644290924

Mean load statistics over all zones

Loads within In and St= 7.81219732E-03 2.13306071E-03

Load between In and St= 3.18665174E-03

Homozygous load for In and St= 8.47831927E-03 2.72219605E-03

Inbreeding loads= 6.66121661E-04 5.89137315E-04

Selection coefficients for In and St homokaryotypes

4.61488962E-03 -1.05416775E-03

Mean frequencies of A2 in In and St= 6.64468184E-02 4.77730334E-02

Ratio of these= 1.39088547

Mean diversities at selected sites in In and St= 9.82884550E-04 1.69991481E-03

Mean diversities at neutral sites in In and St= 4.75247391E-03 1.09446710E-02

pi-n/pi-s for In and St= 0.206815347 0.155318946

Ratio of these= 1.33155262

Mean freqs. of seg. sites= 4.06130496E-03 7.11007789E-03

Ratio of these= 0.571204007

Overall delta-theta values= 0.141404390 0.151787758

Ratio of these= 0.931592882

**h = 0.35**

Zone 1: quasi-neutral zone

Upper bound scaled selection coefficient for neutrality in St metapopulation= 0.250000000

Probability of zone 1= 4.73524816E-02

Integral of selection coefficient over zone 1= 1.95133864E-04

Mean load statistics for zone 1

Mean q1 and q2= 0.600000024

F1 and F2= 0.990099013 0.977198601

Diversities= 4.75247391E-03 1.09446710E-02

Contributions to loads within In and St= 1.16941221E-04 1.16759962E-04

Contribution to load between In and St = 1.03030681E-04

Contributions to homozygous loads for In and St= 1.17080323E-04 1.17080323E-04

Contributions to inbreeding loads for In and St= 9.99798765E-04 9.99913085E-04

Contributions to selection coefficients for In and St homokaryotypes

1.38878822E-05 1.37090683E-05

Contributions to mean A2 freqs= 2.84114908E-02 2.84114908E-02

Contributions to mean diversities= 2.25041440E-04 5.18257322E-04

Contributions to mean freqs. of seg. site= 7.96483480E-04 1.82903488E-03

Delta-theta values= -2.39169598E-03 -5.25259972E-03

Zone 2a: moderate selection; cut-off at moderate gamma for St population

Lower and upper bounds of St popn gamma

0.250000000 297.619049

Probability of zone 2a= 0.346091360

Coefficients for bivariate distribution of q1 and q2

a1= 0.105000004 a2= 0.244999990

b11= 1.35000013E-02 b12= 6.30000010E-02 b22= 7.34999999E-02

Net probability of zone 2a using Simpsons rule= 0.346664220

Contributions to mean load statistics over zone 2a

Contributions to mean loads within In and St= 5.79066947E-03 1.69720326E-03

Contribution to load between In and St= 2.92303436E-03

Contributions to homozygous loads for In and St= 5.90477930E-03 1.81065826E-03

Contributions to inbreeding loads= 1.14104769E-04 1.13456255E-04

Selection coefficients for In and St homokaryotypes

2.86352634E-03 -1.22654438E-03

Selection coefficients for In and St homokaryotypes

2.86352634E-03 -1.22654438E-03

Contributions to mean A2 freqs= 3.46724764E-02 1.90089755E-02

Contributions to mean diversities= 6.57382130E-04 1.04509830E-03

Contributions to mean proportions of seg. sites= 2.79130391E-03 4.62298235E-03

Contributions to delta-theta values= 0.164469123 0.197977364

Zone 2b: moderate selection; gamma for St reaches high value

Lower and upper bounds of St popn gamma

297.619049 2976.19043

Probability of zone 2b 0.344033331

Net probability of zone 2b using Simpsons rule= 0.344033509

Contributions to mean load statistics over zone 2b

Contributions to mean loads within In and St= 1.65028221E-04 9.42223414E-05

Contribution to load between In and St= 1.29373162E-04

Contributions to homozygous loads for In and St= 2.35197949E-04 1.34434347E-04

Contributions to inbreeding loads= 7.01696918E-05 4.02122241E-05

Selection coefficients for In and St homokaryotypes

3.56435776E-05 -3.51667404E-05

Selection coefficients for In and St homokaryotypes

3.56435776E-05 -3.51667404E-05

Contributions to mean A2 freqs= 4.95430686E-06 3.18083562E-06

Contributions to mean diversities= 9.83824521E-06 6.33923446E-06

Contributions to mean proportions of seg. sites= 9.58354722E-05 6.62890452E-05

Contributions to delta-theta values= 0.635797322 0.660728931

Zone 3: strong selection approximation

Lower and upper bounds of St popn gamma= 2976.19043 23333.3340

Zone 3: strong selection approximation

Probability of zone 3= 0.250665724

Mean load statistics over zone 3

Contributions to loads within In and St= 2.87382223E-04 2.87342787E-04

Contribution to load between In and St= 2.87313072E-04

Contributions to homozygous loads for In and St= 4.10446577E-04 4.10446635E-04

Contributions to inbreeding loads= 1.23064456E-04 1.23103950E-04

Selection coefficients for In and St homokaryotypes

5.96046448E-08 0.00000000

Contributions to mean A2 freqs= 9.67476581E-07 9.67476581E-07

Contributions to mean A2 freqs at seg. sites= 0.00000000 0.00000000

Contributions to mean diversities= 1.93360734E-06 1.93437086E-06

Contributions to delta-theta values= 0.643122315 0.644331455

Mean load statistics over all zones

Loads within In and St= 6.36002095E-03 2.19552824E-03

Load between In and St= 3.44275124E-03

Homozygous load for In and St= 6.66750409E-03 2.47261953E-03

Inbreeding loads= 3.07478011E-04 2.77092797E-04

Selection coefficients for In and St homokaryotypes

2.91299820E-03 -1.24800205E-03

Mean frequencies of A2 in In and St= 6.30898923E-02 4.74246144E-02

Ratio of these= 1.33031952

Mean diversities at selected sites in In and St= 8.94195458E-04 1.57162920E-03

Mean diversities at neutral sites in In and St= 4.75247391E-03 1.09446710E-02

pi-n/pi-s for In and St= 0.188153684 0.143597662

Ratio of these= 1.31028378

Mean freqs. of seg. sites= 3.70284496E-03 6.53760182E-03

Ratio of these= 0.566391945

Overall delta-theta values= 0.143260717 0.147128940

Ratio of these= 0.973708630

**h = 0.45**

Zone 1: quasi-neutral zone

Upper bound scaled selection coefficient for neutrality in St metapopulation= 0.250000000

Probability of zone 1= 4.73524816E-02

Integral of selection coefficient over zone 1= 1.95133864E-04

Mean load statistics for zone 1

Mean q1 and q2= 0.600000024

F1 and F2= 0.990099013 0.977198601

Diversities= 4.75247391E-03 1.09446710E-02

Contributions to loads within In and St= 1.17033960E-04 1.16973541E-04

Contribution to load between In and St = 1.12397101E-04

Contributions to homozygous loads for In and St= 1.17080323E-04 1.17080323E-04

Contributions to inbreeding loads for In and St= 4.28509928E-04 4.28544881E-04

Contributions to selection coefficients for In and St homokaryotypes

4.64916229E-06 4.58955765E-06

Contributions to mean A2 freqs= 2.84114908E-02 2.84114908E-02

Contributions to mean diversities= 2.25041440E-04 5.18257322E-04

Contributions to mean freqs. of seg. site= 7.96483480E-04 1.82903488E-03

Delta-theta values= -2.39169598E-03 -5.25259972E-03

Zone 2a: moderate selection; cut-off at moderate gamma for St population

Lower and upper bounds of St popn gamma

0.250000000 165.343918

Probability of zone 2a= 0.283557355

Coefficients for bivariate distribution of q1 and q2

a1= 0.135000005 a2= 0.314999998

b11= 4.50000120E-03 b12= 2.10000053E-02 b22= 2.45000049E-02

Net probability of zone 2a using Simpsons rule= 0.283669829

Contributions to mean load statistics over zone 2a

Contributions to mean loads within In and St= 4.78107249E-03 1.66670361E-03

Contribution to load between In and St= 3.00266873E-03

Contributions to homozygous loads for In and St= 4.80592018E-03 1.69249496E-03

Contributions to inbreeding loads= 2.48361321E-05 2.57911015E-05

Selection coefficients for In and St homokaryotypes

1.77681446E-03 -1.33681297E-03

Selection coefficients for In and St homokaryotypes

1.77681446E-03 -1.33681297E-03

Contributions to mean A2 freqs= 3.22742239E-02 1.88471433E-02

Contributions to mean diversities= 5.89511706E-04 9.44693631E-04

Contributions to mean proportions of seg. sites= 2.48956797E-03 4.15609498E-03

Contributions to delta-theta values= 0.159920812 0.193587482

Zone 2b: moderate selection; gamma for St reaches high value

Lower and upper bounds of St popn gamma

165.343918 1653.43921

Probability of zone 2b 0.306239307

Net probability of zone 2b using Simpsons rule= 0.306239247

Contributions to mean load statistics over zone 2b

Contributions to mean loads within In and St= 1.87596190E-04 1.21576973E-04

Contribution to load between In and St= 1.54489229E-04

Contributions to homozygous loads for In and St= 2.08276630E-04 1.35030845E-04

Contributions to inbreeding loads= 2.06804252E-05 1.34538141E-05

Selection coefficients for In and St homokaryotypes

3.30805779E-05 -3.29017639E-05

Selection coefficients for In and St homokaryotypes

3.30805779E-05 -3.29017639E-05

Contributions to mean A2 freqs= 7.33839943E-06 5.30425268E-06

Contributions to mean diversities= 1.45384265E-05 1.05600966E-05

Contributions to mean proportions of seg. sites= 1.37810392E-04 1.07128893E-04

Contributions to delta-theta values= 0.625728130 0.650285959

Zone 3: strong selection approximation

Lower and upper bounds of St popn gamma= 1653.43921 23333.3340

Zone 3: strong selection approximation

Probability of zone 3= 0.350993752

Mean load statistics over zone 3

Contributions to loads within In and St= 3.77790508E-04 3.77774559E-04

Contribution to load between In and St= 3.77763266E-04

Contributions to homozygous loads for In and St= 4.19736112E-04 4.19736112E-04

Contributions to inbreeding loads= 4.19461321E-05 4.19618154E-05

Selection coefficients for In and St homokaryotypes

0.00000000 0.00000000

Contributions to mean A2 freqs= 1.47335356E-06 1.47335368E-06

Contributions to mean A2 freqs at seg. sites= 0.00000000 0.00000000

Contributions to mean diversities= 2.94406709E-06 2.94556435E-06

Contributions to delta-theta values= 0.642514825 0.644077897

Mean load statistics over all zones

Loads within In and St= 5.46349352E-03 2.28302856E-03

Load between In and St= 3.64731834E-03

Homozygous load for In and St= 5.55101316E-03 2.36434210E-03

Inbreeding loads= 8.75090482E-05 8.13135121E-05

Selection coefficients for In and St homokaryotypes

1.81454420E-03 -1.36518478E-03

Mean frequencies of A2 in In and St= 6.06945306E-02 4.72654141E-02

Ratio of these= 1.28412139

Mean diversities at selected sites in In and St= 8.32035614E-04 1.47645664E-03

Mean diversities at neutral sites in In and St= 4.75247391E-03 1.09446710E-02

pi-n/pi-s for In and St= 0.175074205 0.134901881

Ratio of these= 1.29778922

Mean freqs. of seg. sites= 3.45307938E-03 6.12161914E-03

Ratio of these= 0.564079404

Overall delta-theta values= 0.145155549 0.144330323

Ratio of these= 1.00571764

**h = 0.5**

Zone 1: quasi-neutral zone

Upper bound scaled selection coefficient for neutrality in St metapopulation= 0.250000000

Probability of zone 1= 4.73524816E-02

Integral of selection coefficient over zone 1= 1.95133864E-04

Mean load statistics for zone 1

Mean q1 and q2= 0.600000024

F1 and F2= 0.990099013 0.977198601

Diversities= 4.75247391E-03 1.09446710E-02

Contributions to loads within In and St= 1.17080323E-04 1.17080323E-04

Contribution to load between In and St = 1.17080323E-04

Contributions to homozygous loads for In and St= 1.17080323E-04 1.17080323E-04

Contributions to inbreeding loads for In and St= 1.11098670E-04 1.11105772E-04

Contributions to selection coefficients for In and St homokaryotypes

0.00000000 0.00000000

Contributions to mean A2 freqs= 2.84114908E-02 2.84114908E-02

Contributions to mean diversities= 2.25041440E-04 5.18257322E-04

Contributions to mean freqs. of seg. site= 7.96483480E-04 1.82903488E-03

Delta-theta values= -2.39169598E-03 -5.25259972E-03

Zone 2a: moderate selection; cut-off at moderate gamma for St population

Lower and upper bounds of St popn gamma

0.250000000 82.6719589

Probability of zone 2a= 0.221977368

Coefficients for bivariate distribution of q1 and q2

a1= 0.150000006 a2= 0.349999994

b11= 0.00000000 b12= 0.00000000 b22= 0.00000000

Net probability of zone 2a using Simpsons rule= 0.221989021

Contributions to mean load statistics over zone 2a

Contributions to mean loads within In and St= 4.36759600E-03 1.62132597E-03

Contribution to load between In and St= 2.99446355E-03

Contributions to homozygous loads for In and St= 4.36759600E-03 1.62132597E-03

Contributions to inbreeding loads= 0.00000000 0.00000000

Selection coefficients for In and St homokaryotypes

1.37221813E-03 -1.37412548E-03

Selection coefficients for In and St homokaryotypes

1.37221813E-03 -1.37412548E-03

Contributions to mean A2 freqs= 3.13597396E-02 1.88537631E-02

Contributions to mean diversities= 5.52298268E-04 8.94386962E-04

Contributions to mean proportions of seg. sites= 2.28410470E-03 3.88056901E-03

Contributions to delta-theta values= 0.142153800 0.182322979

Zone 2b: moderate selection; gamma for St reaches high value

Lower and upper bounds of St popn gamma

82.6719589 826.719604

Probability of zone 2b 0.258414686

Net probability of zone 2b using Simpsons rule= 0.258414596

Contributions to mean load statistics over zone 2b

Contributions to mean loads within In and St= 1.98502908E-04 1.49314292E-04

Contribution to load between In and St= 1.73908527E-04

Contributions to homozygous loads for In and St= 1.98502908E-04 1.49314292E-04

Contributions to inbreeding loads= 0.00000000 0.00000000

Selection coefficients for In and St homokaryotypes

2.46167183E-05 -2.45571136E-05

Selection coefficients for In and St homokaryotypes

2.46167183E-05 -2.45571136E-05

Contributions to mean A2 freqs= 1.30229200E-05 1.06116058E-05

Contributions to mean diversities= 2.56256990E-05 2.10648141E-05

Contributions to mean proportions of seg. sites= 2.29364188E-04 2.03632182E-04

Contributions to delta-theta values= 0.603628993 0.633002639

Zone 3: strong selection approximation

Lower and upper bounds of St popn gamma= 826.719604 23333.3340

Zone 3: strong selection approximation

Probability of zone 3= 0.460398376

Mean load statistics over zone 3

Contributions to loads within In and St= 4.77309775E-04 4.77309775E-04

Contribution to load between In and St= 4.77309775E-04

Contributions to homozygous loads for In and St= 4.77309775E-04 4.77309775E-04

Contributions to inbreeding loads= 0.00000000 0.00000000

Selection coefficients for In and St homokaryotypes

0.00000000 0.00000000

Contributions to mean A2 freqs= 2.65328799E-06 2.65328799E-06

Contributions to mean A2 freqs at seg. sites= 0.00000000 0.00000000

Contributions to mean diversities= 5.29869385E-06 5.30316856E-06

Contributions to delta-theta values= 0.640659511 0.643299103

Mean load statistics over all zones

Loads within In and St= 5.16048912E-03 2.36503035E-03

Load between In and St= 3.76276206E-03

Homozygous load for In and St= 5.16048912E-03 2.36503035E-03

Inbreeding loads= 0.00000000 0.00000000

Selection coefficients for In and St homokaryotypes

1.39677525E-03 -1.39868259E-03

Mean frequencies of A2 in In and St= 5.97869083E-02 4.72785197E-02

Ratio of these= 1.26456809

Mean diversities at selected sites in In and St= 8.08264071E-04 1.43901224E-03

Mean diversities at neutral sites in In and St= 4.75247391E-03 1.09446710E-02

pi-n/pi-s for In and St= 0.170072272 0.131480634

Ratio of these= 1.29351580

Mean freqs. of seg. sites= 3.36226611E-03 5.96598117E-03

Ratio of these= 0.563573062

Overall delta-theta values= 0.147149444 0.144274712

Ratio of these= 1.01992536

**Inversion frequency= 0.5**

**h = 0.05**

Zone 1: quasi-neutral zone

Upper bound scaled selection coefficient for neutrality in St metapopulation= 0.250000000

Probability of zone 1= 5.23817725E-02

Integral of selection coefficient over zone 1= 3.02202563E-04

Mean load statistics for zone 1

Mean q1 and q2= 0.600000024

F1 and F2= 0.983606577 0.983606577

Diversities= 7.86884315E-03 7.86884315E-03

Contributions to loads within In and St= 1.80251445E-04 1.80251445E-04

Contribution to load between In and St = 1.16045783E-04

Contributions to homozygous loads for In and St= 1.81321549E-04 1.81321549E-04

Contributions to inbreeding loads for In and St= 0.00000000 0.00000000

Contributions to selection coefficients for In and St homokaryotypes

6.41942024E-05 6.41942024E-05

Contributions to mean A2 freqs= 3.14290635E-02 3.14290635E-02

Contributions to mean diversities= 4.12183959E-04 4.12183959E-04

Contributions to mean freqs. of seg. site= 1.45688269E-03 1.45688269E-03

Delta-theta values= -3.73303890E-03 -3.73303890E-03

Zone 2a: moderate selection; cut-off at moderate gamma for St population

Lower and upper bounds of St popn gamma

0.250000000 250.000000

Probability of zone 2a= 0.360142052

Coefficients for bivariate distribution of q1 and q2

a1= 2.50000004E-02 a2= 2.50000004E-02

b11= 0.112499997 b12= 0.224999994 b22= 0.112499997

Net probability of zone 2a using Simpsons rule= 0.360543936

Contributions to mean load statistics over zone 2a

Contributions to mean loads within In and St= 3.76433204E-03 3.76430806E-03

Contribution to load between In and St= 1.24623114E-03

Contributions to homozygous loads for In and St= 5.08221472E-03 5.08219097E-03

Contributions to inbreeding loads= 1.31788710E-03 1.31788710E-03

Selection coefficients for In and St homokaryotypes

2.51495838E-03 2.51489878E-03

Selection coefficients for In and St homokaryotypes

2.51495838E-03 2.51489878E-03

Contributions to mean A2 freqs= 2.46828031E-02 2.46827137E-02

Contributions to mean diversities= 1.41017931E-03 1.41017779E-03

Contributions to mean proportions of seg. sites= 6.04581414E-03 6.04581460E-03

Contributions to delta-theta values= 0.172493696 0.172494650

Zone 2b: moderate selection; gamma for St reaches high value

Lower and upper bounds of St popn gamma

250.000000 2500.00000

Probability of zone 2b 0.352829665

Net probability of zone 2b using Simpsons rule= 0.352829695

Contributions to mean load statistics over zone 2b

Contributions to mean loads within In and St= 1.40043281E-04 1.40043281E-04

Contribution to load between In and St= 1.23918886E-04

Contributions to homozygous loads for In and St= 1.23746134E-03 1.23746123E-03

Contributions to inbreeding loads= 1.09741732E-03 1.09741732E-03

Selection coefficients for In and St homokaryotypes

1.61528587E-05 1.61528587E-05

Selection coefficients for In and St homokaryotypes

1.61528587E-05 1.61528587E-05

Contributions to mean A2 freqs= 2.21006630E-05 2.21006649E-05

Contributions to mean diversities= 4.33817077E-05 4.33817149E-05

Contributions to mean proportions of seg. sites= 3.80610843E-04 3.80611193E-04

Contributions to delta-theta values= 0.595631599 0.595631897

Zone 3: strong selection approximation

Lower and upper bounds of St popn gamma= 2500.00000 16666.6680

Zone 3: strong selection approximation

Probability of zone 3= 0.222789407

Mean load statistics over zone 3

Contributions to loads within In and St= 2.67129188E-04 2.67129188E-04

Contribution to load between In and St= 2.62155052E-04

Contributions to homozygous loads for In and St= 2.62105116E-03 2.62105116E-03

Contributions to inbreeding loads= 2.35392270E-03 2.35392270E-03

Selection coefficients for In and St homokaryotypes

4.94718552E-06 4.94718552E-06

Contributions to mean A2 freqs= 5.52690972E-06 5.52690972E-06

Contributions to mean A2 freqs at seg. sites= 0.00000000 0.00000000

Contributions to mean diversities= 1.10255723E-05 1.10255723E-05

Contributions to delta-theta values= 0.637409270 0.637409270

Mean load statistics over all zones

Loads within In and St= 4.35175654E-03 4.35173232E-03

Load between In and St= 1.74835091E-03

Homozygous load for In and St= 9.12204850E-03 9.12202522E-03

Inbreeding loads= 4.77029756E-03 4.77029756E-03

Selection coefficients for In and St homokaryotypes

2.60001421E-03 2.60001421E-03

Mean frequencies of A2 in In and St= 5.61394952E-02 5.61394058E-02

Ratio of these= 1.00000155

Mean diversities at selected sites in In and St= 1.87677052E-03 1.87676901E-03

Mean diversities at neutral sites in In and St= 7.86884315E-03 7.86884315E-03

pi-n/pi-s for In and St= 0.238506541 0.238506347

Ratio of these= 1.00000083

Mean freqs. of seg. sites= 7.99118634E-03 7.99118727E-03

Ratio of these= 0.999999881

Overall delta-theta values= 0.166795373 0.166796148

Ratio of these= 0.999995351

**h = 0.15**

Zone 1: quasi-neutral zone

Upper bound scaled selection coefficient for neutrality in St metapopulation= 0.250000000

Probability of zone 1= 5.23817725E-02

Integral of selection coefficient over zone 1= 3.02202563E-04

Mean load statistics for zone 1

Mean q1 and q2= 0.600000024

F1 and F2= 0.983606577 0.983606577

Diversities= 7.86884315E-03 7.86884315E-03

Contributions to loads within In and St= 1.80489253E-04 1.80489253E-04

Contribution to load between In and St = 1.30551503E-04

Contributions to homozygous loads for In and St= 1.81321549E-04 1.81321549E-04

Contributions to inbreeding loads for In and St= 8.99454486E-03 8.99454486E-03

Contributions to selection coefficients for In and St homokaryotypes

4.99486923E-05 4.99486923E-05

Contributions to mean A2 freqs= 3.14290635E-02 3.14290635E-02

Contributions to mean diversities= 4.12183959E-04 4.12183959E-04

Contributions to mean freqs. of seg. site= 1.45688269E-03 1.45688269E-03

Delta-theta values= -3.73303890E-03 -3.73303890E-03

Zone 2a: moderate selection; cut-off at moderate gamma for St population

Lower and upper bounds of St popn gamma

0.250000000 416.666656

Probability of zone 2a= 0.425741285

Coefficients for bivariate distribution of q1 and q2

a1= 7.50000030E-02 a2= 7.50000030E-02

b11= 8.74999985E-02 b12= 0.174999997 b22= 8.74999985E-02

Net probability of zone 2a using Simpsons rule= 0.427173704

Contributions to mean load statistics over zone 2a

Contributions to mean loads within In and St= 3.41574638E-03 3.41574498E-03

Contribution to load between In and St= 1.74926280E-03

Contributions to homozygous loads for In and St= 4.05444298E-03 4.05444158E-03

Contributions to inbreeding loads= 6.38700963E-04 6.38701080E-04

Selection coefficients for In and St homokaryotypes

1.66511536E-03 1.66511536E-03

Selection coefficients for In and St homokaryotypes

1.66511536E-03 1.66511536E-03

Contributions to mean A2 freqs= 2.42359322E-02 2.42359266E-02

Contributions to mean diversities= 1.12721382E-03 1.12721405E-03

Contributions to mean proportions of seg. sites= 4.89991345E-03 4.89991391E-03

Contributions to delta-theta values= 0.183850646 0.183850527

Zone 2b: moderate selection; gamma for St reaches high value

Lower and upper bounds of St popn gamma

416.666656 4166.66650

Probability of zone 2b 0.371462822

Net probability of zone 2b using Simpsons rule= 0.371462792

Contributions to mean load statistics over zone 2b

Contributions to mean loads within In and St= 9.54239731E-05 9.54239804E-05

Contribution to load between In and St= 9.44418716E-05

Contributions to homozygous loads for In and St= 3.14783159E-04 3.14783189E-04

Contributions to inbreeding loads= 2.19358830E-04 2.19358786E-04

Selection coefficients for In and St homokaryotypes

9.53674316E-07 9.53674316E-07

Selection coefficients for In and St homokaryotypes

9.53674316E-07 9.53674316E-07

Contributions to mean A2 freqs= 3.83266570E-06 3.83266524E-06

Contributions to mean diversities= 7.62352056E-06 7.62352056E-06

Contributions to mean proportions of seg. sites= 7.59514587E-05 7.59522081E-05

Contributions to delta-theta values= 0.643900633 0.643904150

Zone 3: strong selection approximation

Lower and upper bounds of St popn gamma= 4166.66650 16666.6680

Zone 3: strong selection approximation

Probability of zone 3= 0.138557017

Mean load statistics over zone 3

Contributions to loads within In and St= 1.84956953E-04 1.84956953E-04

Contribution to load between In and St= 1.84744757E-04

Contributions to homozygous loads for In and St= 6.15808880E-04 6.15808880E-04

Contributions to inbreeding loads= 4.30852029E-04 4.30852029E-04

Selection coefficients for In and St homokaryotypes

2.38418579E-07 2.38418579E-07

Contributions to mean A2 freqs= 9.10673407E-07 9.10673407E-07

Contributions to mean A2 freqs at seg. sites= 0.00000000 0.00000000

Contributions to mean diversities= 1.82033784E-06 1.82033784E-06

Contributions to delta-theta values= 0.643552780 0.643552780

Mean load statistics over all zones

Loads within In and St= 3.87661648E-03 3.87661508E-03

Load between In and St= 2.15900107E-03

Homozygous load for In and St= 5.16635645E-03 5.16635552E-03

Inbreeding loads= 1.28974416E-03 1.28974416E-03

Selection coefficients for In and St homokaryotypes

1.71613693E-03 1.71613693E-03

Mean frequencies of A2 in In and St= 5.56697361E-02 5.56697324E-02

Ratio of these= 1.00000012

Mean diversities at selected sites in In and St= 1.54884171E-03 1.54884194E-03

Mean diversities at neutral sites in In and St= 7.86884315E-03 7.86884315E-03

pi-n/pi-s for In and St= 0.196832195 0.196832225

Ratio of these= 0.999999821

Mean freqs. of seg. sites= 6.45086588E-03 6.45086681E-03

Ratio of these= 0.999999881

Overall delta-theta values= 0.148193836 0.148193836

Ratio of these= 1.00000000

**h = 0.25**

Zone 1: quasi-neutral zone

Upper bound scaled selection coefficient for neutrality in St metapopulation= 0.250000000

Probability of zone 1= 5.23817725E-02

Integral of selection coefficient over zone 1= 3.02202563E-04

Mean load statistics for zone 1

Mean q1 and q2= 0.600000024

F1 and F2= 0.983606577 0.983606577

Diversities= 7.86884315E-03 7.86884315E-03

Contributions to loads within In and St= 1.80727060E-04 1.80727060E-04

Contribution to load between In and St = 1.45057231E-04

Contributions to homozygous loads for In and St= 1.81321549E-04 1.81321549E-04

Contributions to inbreeding loads for In and St= 2.33286177E-03 2.33286177E-03

Contributions to selection coefficients for In and St homokaryotypes

3.56435776E-05 3.56435776E-05

Contributions to mean A2 freqs= 3.14290635E-02 3.14290635E-02

Contributions to mean diversities= 4.12183959E-04 4.12183959E-04

Contributions to mean freqs. of seg. site= 1.45688269E-03 1.45688269E-03

Delta-theta values= -3.73303890E-03 -3.73303890E-03

Zone 2a: moderate selection; cut-off at moderate gamma for St population

Lower and upper bounds of St popn gamma

0.250000000 416.666656

Probability of zone 2a= 0.425741285

Coefficients for bivariate distribution of q1 and q2

a1= 0.125000000 a2= 0.125000000

b11= 6.25000000E-02 b12= 0.125000000 b22= 6.25000000E-02

Net probability of zone 2a using Simpsons rule= 0.427173704

Contributions to mean load statistics over zone 2a

Contributions to mean loads within In and St= 3.06171109E-03 3.06171109E-03

Contribution to load between In and St= 2.06977106E-03

Contributions to homozygous loads for In and St= 3.36566404E-03 3.36566381E-03

Contributions to inbreeding loads= 3.03956418E-04 3.03956534E-04

Selection coefficients for In and St homokaryotypes

9.91463661E-04 9.91463661E-04

Selection coefficients for In and St homokaryotypes

9.91463661E-04 9.91463661E-04

Contributions to mean A2 freqs= 2.33292002E-02 2.33291984E-02

Contributions to mean diversities= 9.58729303E-04 9.58729303E-04

Contributions to mean proportions of seg. sites= 4.20129392E-03 4.20129485E-03

Contributions to delta-theta values= 0.190410852 0.190410972

Zone 2b: moderate selection; gamma for St reaches high value

Lower and upper bounds of St popn gamma

416.666656 4166.66650

Probability of zone 2b 0.371462822

Net probability of zone 2b using Simpsons rule= 0.371462792

Contributions to mean load statistics over zone 2b

Contributions to mean loads within In and St= 8.51035220E-05 8.51035002E-05

Contribution to load between In and St= 8.48490236E-05

Contributions to homozygous loads for In and St= 1.69694817E-04 1.69694817E-04

Contributions to inbreeding loads= 8.45912655E-05 8.45912800E-05

Selection coefficients for In and St homokaryotypes

2.38418579E-07 2.38418579E-07

Selection coefficients for In and St homokaryotypes

2.38418579E-07 2.38418579E-07

Contributions to mean A2 freqs= 2.13821704E-06 2.13821727E-06

Contributions to mean diversities= 4.26112956E-06 4.26112956E-06

Contributions to mean proportions of seg. sites= 4.44828729E-05 4.44824509E-05

Contributions to delta-theta values= 0.660152793 0.660149574

Zone 3: strong selection approximation

Lower and upper bounds of St popn gamma= 4166.66650 16666.6680

Zone 3: strong selection approximation

Probability of zone 3= 0.138557017

Mean load statistics over zone 3

Contributions to loads within In and St= 1.84797667E-04 1.84797667E-04

Contribution to load between In and St= 1.84743141E-04

Contributions to homozygous loads for In and St= 3.69485031E-04 3.69485031E-04

Contributions to inbreeding loads= 1.84687335E-04 1.84687335E-04

Selection coefficients for In and St homokaryotypes

5.96046448E-08 5.96046448E-08

Contributions to mean A2 freqs= 5.46404465E-07 5.46404465E-07

Contributions to mean A2 freqs at seg. sites= 0.00000000 0.00000000

Contributions to mean diversities= 1.09244422E-06 1.09244422E-06

Contributions to delta-theta values= 0.644230366 0.644230366

Mean load statistics over all zones

Loads within In and St= 3.51233943E-03 3.51233943E-03

Load between In and St= 2.48442055E-03

Homozygous load for In and St= 4.08616569E-03 4.08616522E-03

Inbreeding loads= 5.73829515E-04 5.73829631E-04

Selection coefficients for In and St homokaryotypes

1.02740526E-03 1.02740526E-03

Mean frequencies of A2 in In and St= 5.47609515E-02 5.47609478E-02

Ratio of these= 1.00000012

Mean diversities at selected sites in In and St= 1.37626682E-03 1.37626682E-03

Mean diversities at neutral sites in In and St= 7.86884315E-03 7.86884315E-03

pi-n/pi-s for In and St= 0.174900785 0.174900785

Ratio of these= 1.00000000

Mean freqs. of seg. sites= 5.71355317E-03 5.71355363E-03

Ratio of these= 0.999999940

Overall delta-theta values= 0.145429075 0.145429134

Ratio of these= 0.999999583

**h = 0.35**

Zone 1: quasi-neutral zone

Upper bound scaled selection coefficient for neutrality in St metapopulation= 0.250000000

Probability of zone 1= 5.23817725E-02

Integral of selection coefficient over zone 1= 3.02202563E-04

Mean load statistics for zone 1

Mean q1 and q2= 0.600000024

F1 and F2= 0.983606577 0.983606577

Diversities= 7.86884315E-03 7.86884315E-03

Contributions to loads within In and St= 1.80964853E-04 1.80964853E-04

Contribution to load between In and St = 1.59562958E-04

Contributions to homozygous loads for In and St= 1.81321549E-04 1.81321549E-04

Contributions to inbreeding loads for In and St= 9.99878743E-04 9.99878743E-04

Contributions to selection coefficients for In and St homokaryotypes

2.13980675E-05 2.13980675E-05

Contributions to mean A2 freqs= 3.14290635E-02 3.14290635E-02

Contributions to mean diversities= 4.12183959E-04 4.12183959E-04

Contributions to mean freqs. of seg. site= 1.45688269E-03 1.45688269E-03

Delta-theta values= -3.73303890E-03 -3.73303890E-03

Zone 2a: moderate selection; cut-off at moderate gamma for St population

Lower and upper bounds of St popn gamma

0.250000000 297.619049

Probability of zone 2a= 0.381587625

Coefficients for bivariate distribution of q1 and q2

a1= 0.174999997 a2= 0.174999997

b11= 3.75000015E-02 b12= 7.50000030E-02 b22= 3.75000015E-02

Net probability of zone 2a using Simpsons rule= 0.382221013

Contributions to mean load statistics over zone 2a

Contributions to mean loads within In and St= 2.77287024E-03 2.77287024E-03

Contribution to load between In and St= 2.27455213E-03

Contributions to homozygous loads for In and St= 2.89885444E-03 2.89885444E-03

Contributions to inbreeding loads= 1.25984938E-04 1.25984938E-04

Selection coefficients for In and St homokaryotypes

4.98175621E-04 4.98175621E-04

Selection coefficients for In and St homokaryotypes

4.98175621E-04 4.98175621E-04

Contributions to mean A2 freqs= 2.21612398E-02 2.21612398E-02

Contributions to mean diversities= 8.41531146E-04 8.41531379E-04

Contributions to mean proportions of seg. sites= 3.70386243E-03 3.70386313E-03

Contributions to delta-theta values= 0.193940461 0.193940401

Zone 2b: moderate selection; gamma for St reaches high value

Lower and upper bounds of St popn gamma

297.619049 2976.19043

Probability of zone 2b 0.360976130

Net probability of zone 2b using Simpsons rule= 0.360976666

Contributions to mean load statistics over zone 2b

Contributions to mean loads within In and St= 9.87444728E-05 9.87444655E-05

Contribution to load between In and St= 9.86207742E-05

Contributions to homozygous loads for In and St= 1.40885400E-04 1.40885400E-04

Contributions to inbreeding loads= 4.21411096E-05 4.21411096E-05

Selection coefficients for In and St homokaryotypes

1.19209290E-07 1.19209290E-07

Selection coefficients for In and St homokaryotypes

1.19209290E-07 1.19209290E-07

Contributions to mean A2 freqs= 2.40549139E-06 2.40549139E-06

Contributions to mean diversities= 4.79397931E-06 4.79397931E-06

Contributions to mean proportions of seg. sites= 5.01945069E-05 5.01944269E-05

Contributions to delta-theta values= 0.661162317 0.661161780

Zone 3: strong selection approximation

Lower and upper bounds of St popn gamma= 2976.19043 16666.6680

Zone 3: strong selection approximation

Probability of zone 3= 0.193197370

Mean load statistics over zone 3

Contributions to loads within In and St= 2.35223662E-04 2.35223662E-04

Contribution to load between In and St= 2.35196741E-04

Contributions to homozygous loads for In and St= 3.35994788E-04 3.35994788E-04

Contributions to inbreeding loads= 1.00771293E-04 1.00771293E-04

Selection coefficients for In and St homokaryotypes

0.00000000 0.00000000

Contributions to mean A2 freqs= 6.28056341E-07 6.28056341E-07

Contributions to mean A2 freqs at seg. sites= 0.00000000 0.00000000

Contributions to mean diversities= 1.25571580E-06 1.25571580E-06

Contributions to delta-theta values= 0.644288719 0.644288719

Mean load statistics over all zones

Loads within In and St= 3.28780315E-03 3.28780315E-03

Load between In and St= 2.76793260E-03

Homozygous load for In and St= 3.55705619E-03 3.55705619E-03

Inbreeding loads= 2.69254029E-04 2.69254029E-04

Selection coefficients for In and St homokaryotypes

5.19752502E-04 5.19752502E-04

Mean frequencies of A2 in In and St= 5.35933413E-02 5.35933413E-02

Ratio of these= 1.00000000

Mean diversities at selected sites in In and St= 1.25976489E-03 1.25976512E-03

Mean diversities at neutral sites in In and St= 7.86884315E-03 7.86884315E-03

pi-n/pi-s for In and St= 0.160095304 0.160095334

Ratio of these= 0.999999821

Mean freqs. of seg. sites= 5.22346376E-03 5.22346422E-03

Ratio of these= 0.999999940

Overall delta-theta values= 0.144376576 0.144376457

Ratio of these= 1.00000083

**h = 0.45**

Zone 1: quasi-neutral zone

Upper bound scaled selection coefficient for neutrality in St metapopulation= 0.250000000

Probability of zone 1= 5.23817725E-02

Integral of selection coefficient over zone 1= 3.02202563E-04

Mean load statistics for zone 1

Mean q1 and q2= 0.600000024

F1 and F2= 0.983606577 0.983606577

Diversities= 7.86884315E-03 7.86884315E-03

Contributions to loads within In and St= 1.81202646E-04 1.81202646E-04

Contribution to load between In and St = 1.74068671E-04

Contributions to homozygous loads for In and St= 1.81321549E-04 1.81321549E-04

Contributions to inbreeding loads for In and St= 4.28534317E-04 4.28534317E-04

Contributions to selection coefficients for In and St homokaryotypes

7.15255737E-06 7.15255737E-06

Contributions to mean A2 freqs= 3.14290635E-02 3.14290635E-02

Contributions to mean diversities= 4.12183959E-04 4.12183959E-04

Contributions to mean freqs. of seg. site= 1.45688269E-03 1.45688269E-03

Delta-theta values= -3.73303890E-03 -3.73303890E-03

Zone 2a: moderate selection; cut-off at moderate gamma for St population

Lower and upper bounds of St popn gamma

0.250000000 165.343918

Probability of zone 2a= 0.313080877

Coefficients for bivariate distribution of q1 and q2

a1= 0.224999994 a2= 0.224999994

b11= 1.25000030E-02 b12= 2.50000060E-02 b22= 1.25000030E-02

Net probability of zone 2a using Simpsons rule= 0.313205212

Contributions to mean load statistics over zone 2a

Contributions to mean loads within In and St= 2.51453579E-03 2.51453579E-03

Contribution to load between In and St= 2.37183063E-03

Contributions to homozygous loads for In and St= 2.54310155E-03 2.54310155E-03

Contributions to inbreeding loads= 2.85637234E-05 2.85637198E-05

Selection coefficients for In and St homokaryotypes

1.42693520E-04 1.42693520E-04

Selection coefficients for In and St homokaryotypes

1.42693520E-04 1.42693520E-04

Contributions to mean A2 freqs= 2.12229844E-02 2.12229844E-02

Contributions to mean diversities= 7.54299574E-04 7.54299574E-04

Contributions to mean proportions of seg. sites= 3.31665017E-03 3.31665296E-03

Contributions to delta-theta values= 0.193144143 0.193144798

Zone 2b: moderate selection; gamma for St reaches high value

Lower and upper bounds of St popn gamma

165.343918 1653.43921

Probability of zone 2b 0.328705907

Net probability of zone 2b using Simpsons rule= 0.328705937

Contributions to mean load statistics over zone 2b

Contributions to mean loads within In and St= 1.29292806E-04 1.29292806E-04

Contribution to load between In and St= 1.29240871E-04

Contributions to homozygous loads for In and St= 1.43600264E-04 1.43600235E-04

Contributions to inbreeding loads= 1.43074431E-05 1.43074431E-05

Selection coefficients for In and St homokaryotypes

5.96046448E-08 5.96046448E-08

Selection coefficients for In and St homokaryotypes

5.96046448E-08 5.96046448E-08

Contributions to mean A2 freqs= 4.06029130E-06 4.06029130E-06

Contributions to mean diversities= 8.08350433E-06 8.08350433E-06

Contributions to mean proportions of seg. sites= 8.21280773E-05 8.21284484E-05

Contributions to delta-theta values= 0.650811672 0.650813222

Zone 3: strong selection approximation

Lower and upper bounds of St popn gamma= 1653.43921 16666.6680

Zone 3: strong selection approximation

Probability of zone 3= 0.293974340

Mean load statistics over zone 3

Contributions to loads within In and St= 3.26361536E-04 3.26361536E-04

Contribution to load between In and St= 3.26350244E-04

Contributions to homozygous loads for In and St= 3.62611463E-04 3.62611463E-04

Contributions to inbreeding loads= 3.62497412E-05 3.62497412E-05

Selection coefficients for In and St homokaryotypes

0.00000000 0.00000000

Contributions to mean A2 freqs= 1.01446165E-06 1.01446165E-06

Contributions to mean A2 freqs at seg. sites= 0.00000000 0.00000000

Contributions to mean diversities= 2.02810406E-06 2.02810406E-06

Contributions to delta-theta values= 0.644024730 0.644024730

Mean load statistics over all zones

Loads within In and St= 3.15139256E-03 3.15139256E-03

Load between In and St= 3.00149038E-03

Homozygous load for In and St= 3.23063461E-03 3.23063461E-03

Inbreeding loads= 7.92398059E-05 7.92398059E-05

Selection coefficients for In and St homokaryotypes

1.49905682E-04 1.49905682E-04

Mean frequencies of A2 in In and St= 5.26571199E-02 5.26571199E-02

Ratio of these= 1.00000000

Mean diversities at selected sites in In and St= 1.17659511E-03 1.17659511E-03

Mean diversities at neutral sites in In and St= 7.86884315E-03 7.86884315E-03

pi-n/pi-s for In and St= 0.149525806 0.149525806

Ratio of these= 1.00000000

Mean freqs. of seg. sites= 4.87587368E-03 4.87587694E-03

Ratio of these= 0.999999344

Overall delta-theta values= 0.143896401 0.143896878

Ratio of these= 0.999996662

**h = 0.5**

Zone 1: quasi-neutral zone

Upper bound scaled selection coefficient for neutrality in St metapopulation= 0.250000000

Probability of zone 1= 5.23817725E-02

Integral of selection coefficient over zone 1= 3.02202563E-04

Mean load statistics for zone 1

Mean q1 and q2= 0.600000024

F1 and F2= 0.983606577 0.983606577

Diversities= 7.86884315E-03 7.86884315E-03

Contributions to loads within In and St= 1.81321549E-04 1.81321549E-04

Contribution to load between In and St = 1.81321549E-04

Contributions to homozygous loads for In and St= 1.81321549E-04 1.81321549E-04

Contributions to inbreeding loads for In and St= 1.11103691E-04 1.11103691E-04

Contributions to selection coefficients for In and St homokaryotypes

0.00000000 0.00000000

Contributions to mean A2 freqs= 3.14290635E-02 3.14290635E-02

Contributions to mean diversities= 4.12183959E-04 4.12183959E-04

Contributions to mean freqs. of seg. site= 1.45688269E-03 1.45688269E-03

Delta-theta values= -3.73303890E-03 -3.73303890E-03

Zone 2a: moderate selection; cut-off at moderate gamma for St population

Lower and upper bounds of St popn gamma

0.250000000 82.6719589

Probability of zone 2a= 0.245311543

Coefficients for bivariate distribution of q1 and q2

a1= 0.250000000 a2= 0.250000000

b11= 0.00000000 b12= 0.00000000 b22= 0.00000000

Net probability of zone 2a using Simpsons rule= 0.245324686

Contributions to mean load statistics over zone 2a

Contributions to mean loads within In and St= 2.41810456E-03 2.41810456E-03

Contribution to load between In and St= 2.41810456E-03

Contributions to homozygous loads for In and St= 2.41810456E-03 2.41810456E-03

Contributions to inbreeding loads= 0.00000000 0.00000000

Selection coefficients for In and St homokaryotypes

0.00000000 0.00000000

Selection coefficients for In and St homokaryotypes

0.00000000 0.00000000

Contributions to mean A2 freqs= 2.13080645E-02 2.13080645E-02

Contributions to mean diversities= 7.11877074E-04 7.11877190E-04

Contributions to mean proportions of seg. sites= 3.09306569E-03 3.09306663E-03

Contributions to delta-theta values= 0.183478534 0.183478594

Zone 2b: moderate selection; gamma for St reaches high value

Lower and upper bounds of St popn gamma

82.6719589 826.719604

Probability of zone 2b 0.281515658

Net probability of zone 2b using Simpsons rule= 0.281515867

Contributions to mean load statistics over zone 2b

Contributions to mean loads within In and St= 1.60683805E-04 1.60683776E-04

Contribution to load between In and St= 1.60683776E-04

Contributions to homozygous loads for In and St= 1.60683805E-04 1.60683776E-04

Contributions to inbreeding loads= 0.00000000 0.00000000

Selection coefficients for In and St homokaryotypes

0.00000000 0.00000000

Selection coefficients for In and St homokaryotypes

0.00000000 0.00000000

Contributions to mean A2 freqs= 8.20601872E-06 8.20601872E-06

Contributions to mean diversities= 1.62896304E-05 1.62896304E-05

Contributions to mean proportions of seg. sites= 1.57644507E-04 1.57645016E-04

Contributions to delta-theta values= 0.633406997 0.633408189

Zone 3: strong selection approximation

Lower and upper bounds of St popn gamma= 826.719604 16666.6680

Zone 3: strong selection approximation

Probability of zone 3= 0.408933938

Mean load statistics over zone 3

Contributions to loads within In and St= 4.30277199E-04 4.30277199E-04

Contribution to load between In and St= 4.30277199E-04

Contributions to homozygous loads for In and St= 4.30277199E-04 4.30277199E-04

Contributions to inbreeding loads= 0.00000000 0.00000000

Selection coefficients for In and St homokaryotypes

0.00000000 0.00000000

Contributions to mean A2 freqs= 1.91561116E-06 1.91561116E-06

Contributions to mean A2 freqs at seg. sites= 0.00000000 0.00000000

Contributions to mean diversities= 3.82867711E-06 3.82867711E-06

Contributions to delta-theta values= 0.643227816 0.643227816

Mean load statistics over all zones

Loads within In and St= 3.19038704E-03 3.19038704E-03

Load between In and St= 3.19038704E-03

Homozygous load for In and St= 3.19038704E-03 3.19038704E-03

Inbreeding loads= 0.00000000 0.00000000

Selection coefficients for In and St homokaryotypes

0.00000000 0.00000000

Mean frequencies of A2 in In and St= 5.27472496E-02 5.27472496E-02

Ratio of these= 1.00000000

Mean diversities at selected sites in In and St= 1.14417938E-03 1.14417949E-03

Mean diversities at neutral sites in In and St= 7.86884315E-03 7.86884315E-03

pi-n/pi-s for In and St= 0.145406306 0.145406321

Ratio of these= 0.999999881

Mean freqs. of seg. sites= 4.74566547E-03 4.74566687E-03

Ratio of these= 0.999999702

Overall delta-theta values= 0.144640386 0.144640505

Ratio of these= 0.999999166
