# Supplementary material for: The fitness consequences of genetic divergence between polymorphic gene arrangements
Source: Genetics. 2023 Dec 26;226(3):iyad218. doi: 10.1093/genetics/iyad218 (PMC11090464; doi:10.1093/genetics/iyad218)
Supplement: iyad218_Supplementary_Data [file iyad218_supplementary_data.zip › Supplementary_Table_2_GENETICS-2023-306559.docx]

**Supplementary Table S2 Results for a single population with 3 different population sizes**

**Results are shown for population sizes of 2 x 10^6^, 5 x 10^5^ and 1.25 x 10^5^, inversion frequencies of 0.1, 0.3 and 0.5, and dominance coefficients of h = 0.05, 0.15,0.25,0.35, 0.45 and 0.5**

**Mutation rate towards deleterious variants= 4.99999997E-09**

**Number of selected sites in inversion= 100000**

**Mutational bias towards deleterious variants= 1.50000000**

**Mean selection coefficient= 1.0 E-03**

**No. of values for Simpsons rule = 650**

**Sample size= 20**

**Wattersons correction factor= 3.54773974**

**Number of dominance coefficients modelled= 6**

**Shape parameter= 0.300000012**

**Upper bound gamma value for neutrality in the St popn= 0.250000000**

**Upper bound whole popn gamma value for zone 2a with h=0.25 = 50.0000000**

**Upper bound whole popn gamma value for zone 2b with h=0.25 = 500.000000**

**Upper limit to gamma distribution of z= x/scale parameter = 2.50000000**

**Threshold gamma value factor for use of approximate p.d.f.= 0.250000000**

**Section 1**

**Population size = 2000000**

**Mean scaled selection coefficient for whole popn= 4000**

**Inversion frequency= 0.1**

**h = 0.05**

Zone 1: quasi-neutral zone

Upper bound scaled selection coefficient for neutrality in St metapopulation= 0.250000000

Probability of zone 1= 4.39136960E-02

Integral of selection coefficient over zone 1= 7.03745172E-05

Mean load statistics for zone 1

Mean q1 and q2= 0.600000024

F1 and F2= 0.993377507 0.943396151

Diversities= 3.17879673E-03 2.71698460E-02

Contributions to loads within In and St= 4.21240438E-05 4.13642811E-05

Contribution to load between In and St = 2.70238143E-05

Contributions to homozygous loads for In and St= 4.22247103E-05 4.22247103E-05

Contributions to inbreeding loads for In and St= 0.00000000 0.00000000

Contributions to selection coefficients for In and St homokaryotypes

1.50799751E-05 1.43647194E-05

Contributions to mean A2 freqs= 2.63482183E-02 2.63482183E-02

Contributions to mean diversities= 1.39592710E-04 1.19312841E-03

Contributions to mean freqs. of seg. site= 4.94409469E-04 4.17755544E-03

Delta-theta values= -1.67715549E-03 -1.32502317E-02

Zone 2a: moderate selection; cut-off at moderate gamma for St population

Lower and upper bounds of St popn gamma

0.250000000 250.000000

Probability of zone 2a= 0.303239673

Coefficients for bivariate distribution of q1 and q2

a1= 5.00000035E-03 a2= 4.49999981E-02

b11= 4.50000027E-03 b12= 8.09999928E-02 b22= 0.364499956

Net probability of zone 2a using Simpsons rule= 0.303576410

Contributions to mean load statistics over zone 2a

Contributions to mean loads within In and St= 7.18459636E-02 6.21988263E-04

Contribution to load between In and St= 4.20853170E-03

Contributions to homozygous loads for In and St= 7.25406930E-02 1.43152336E-03

Contributions to inbreeding loads= 6.94722577E-04 8.09535151E-04

Selection coefficients for In and St homokaryotypes

6.54007196E-02 -3.59296799E-03

Selection coefficients for In and St homokaryotypes

6.54007196E-02 -3.59296799E-03

Contributions to mean A2 freqs= 0.105670214 1.82203297E-02

Contributions to mean diversities= 9.54637362E-04 3.47454264E-03

Contributions to mean proportions of seg. sites= 3.46246199E-03 1.56324785E-02

Contributions to delta-theta values= 2.18506455E-02 0.211463928

Zone 2b: moderate selection; gamma for St reaches high value

Lower and upper bounds of St popn gamma

250.000000 2500.00000

Probability of zone 2b 0.317252636

Net probability of zone 2b using Simpsons rule= 0.317252696

Contributions to mean load statistics over zone 2b

Contributions to mean loads within In and St= 1.47809263E-03 8.62472298E-05

Contribution to load between In and St= 2.00811701E-04

Contributions to homozygous loads for In and St= 3.22007481E-03 7.69341539E-04

Contributions to inbreeding loads= 1.74197881E-03 6.83093676E-04

Selection coefficients for In and St homokaryotypes

1.27649307E-03 -1.14560127E-04

Selection coefficients for In and St homokaryotypes

1.27649307E-03 -1.14560127E-04

Contributions to mean A2 freqs= 2.51300808E-04 5.00560564E-05

Contributions to mean diversities= 2.10347469E-04 9.84295330E-05

Contributions to mean proportions of seg. sites= 1.15121447E-03 8.67272203E-04

Contributions to delta-theta values= 0.351764500 0.597355545

Zone 3: strong selection approximation

Lower and upper bounds of St popn gamma= 2500.00000 30000.0020

Zone 3: strong selection approximation

Probability of zone 3= 0.323736906

Mean load statistics over zone 3

Contributions to loads within In and St= 4.03393526E-04 3.58925899E-04

Contribution to load between In and St= 3.53367213E-04

Contributions to homozygous loads for In and St= 3.53167788E-03 3.53167788E-03

Contributions to inbreeding loads= 3.12828110E-03 3.17274500E-03

Selection coefficients for In and St homokaryotypes

5.00082970E-05 5.54323196E-06

Contributions to mean A2 freqs= 2.22338131E-05 2.22338131E-05

Contributions to mean A2 freqs at seg. sites= 0.00000000 0.00000000

Contributions to mean diversities= 4.35339025E-05 4.43607096E-05

Contributions to delta-theta values= 0.566181302 0.638077736

Mean load statistics over all zones

Loads within In and St= 7.37695768E-02 1.10852567E-03

Load between In and St= 4.78973426E-03

Homozygous load for In and St= 7.93346688E-02 5.77476760E-03

Inbreeding loads= 5.56508312E-03 4.66623437E-03

Selection coefficients for In and St homokaryotypes

6.66545033E-02 -3.68797779E-03

Mean frequencies of A2 in In and St= 0.132291973 4.46408354E-02

Ratio of these= 2.96347451

Mean diversities at selected sites in In and St= 1.34811143E-03 4.81046131E-03

Mean diversities at neutral sites in In and St= 3.17879673E-03 2.71698460E-02

pi-n/pi-s for In and St= 0.424094886 0.177051470

Ratio of these= 2.39531970

Mean freqs. of seg. sites= 5.46410307E-03 2.11121514E-02

Ratio of these= 0.258813173

Overall delta-theta values= 0.124696493 0.191637814

Ratio of these= 0.650688350

**h = 0.15**

Zone 1: quasi-neutral zone

Upper bound scaled selection coefficient for neutrality in St metapopulation= 0.250000000

Probability of zone 1= 4.39136960E-02

Integral of selection coefficient over zone 1= 7.03745172E-05

Mean load statistics for zone 1

Mean q1 and q2= 0.600000024

F1 and F2= 0.993377507 0.943396151

Diversities= 3.17879673E-03 2.71698460E-02

Contributions to loads within In and St= 4.21464138E-05 4.15554896E-05

Contribution to load between In and St = 3.04017922E-05

Contributions to homozygous loads for In and St= 4.22247103E-05 4.22247103E-05

Contributions to inbreeding loads for In and St= 8.97289254E-03 8.99689179E-03

Contributions to selection coefficients for In and St homokaryotypes

1.17421150E-05 1.11460686E-05

Contributions to mean A2 freqs= 2.63482183E-02 2.63482183E-02

Contributions to mean diversities= 1.39592710E-04 1.19312841E-03

Contributions to mean freqs. of seg. site= 4.94409469E-04 4.17755544E-03

Delta-theta values= -1.67715549E-03 -1.32502317E-02

Zone 2a: moderate selection; cut-off at moderate gamma for St population

Lower and upper bounds of St popn gamma

0.250000000 416.666656

Probability of zone 2a= 0.359450161

Coefficients for bivariate distribution of q1 and q2

a1= 1.50000006E-02 a2= 0.135000005

b11= 3.50000011E-03 b12= 6.30000010E-02 b22= 0.283499986

Net probability of zone 2a using Simpsons rule= 0.360651016

Contributions to mean load statistics over zone 2a

Contributions to mean loads within In and St= 3.12426761E-02 7.01189274E-04

Contribution to load between In and St= 5.23768039E-03

Contributions to homozygous loads for In and St= 3.16974111E-02 1.16027007E-03

Contributions to inbreeding loads= 4.54747496E-04 4.59080416E-04

Selection coefficients for In and St homokaryotypes

2.56697536E-02 -4.54676151E-03

Selection coefficients for In and St homokaryotypes

2.56697536E-02 -4.54676151E-03

Contributions to mean A2 freqs= 8.30123052E-02 1.85245294E-02

Contributions to mean diversities= 8.45420931E-04 3.04522808E-03

Contributions to mean proportions of seg. sites= 3.27946455E-03 1.35813924E-02

Contributions to delta-theta values= 8.54197741E-02 0.204523623

Zone 2b: moderate selection; gamma for St reaches high value

Lower and upper bounds of St popn gamma

416.666656 4166.66650

Probability of zone 2b 0.348782212

Net probability of zone 2b using Simpsons rule= 0.348782301

Contributions to mean load statistics over zone 2b

Contributions to mean loads within In and St= 2.30006393E-04 6.07465918E-05

Contribution to load between In and St= 1.37265233E-04

Contributions to homozygous loads for In and St= 7.14527676E-04 2.00361712E-04

Contributions to inbreeding loads= 4.84521181E-04 1.39615178E-04

Selection coefficients for In and St homokaryotypes

9.27448273E-05 -7.65323639E-05

Selection coefficients for In and St homokaryotypes

9.27448273E-05 -7.65323639E-05

Contributions to mean A2 freqs= 2.61588284E-05 8.87211809E-06

Contributions to mean diversities= 5.01129580E-05 1.76464619E-05

Contributions to mean proportions of seg. sites= 3.88156390E-04 1.74032306E-04

Contributions to delta-theta values= 0.541968822 0.640267611

Zone 3: strong selection approximation

Lower and upper bounds of St popn gamma= 4166.66650 30000.0020

Zone 3: strong selection approximation

Probability of zone 3= 0.235996842

Mean load statistics over zone 3

Contributions to loads within In and St= 2.76439736E-04 2.74333259E-04

Contribution to load between In and St= 2.74069986E-04

Contributions to homozygous loads for In and St= 9.13536234E-04 9.13536234E-04

Contributions to inbreeding loads= 6.37095829E-04 6.39201840E-04

Selection coefficients for In and St homokaryotypes

2.38418579E-06 2.38418579E-07

Contributions to mean A2 freqs= 4.06221989E-06 4.06221943E-06

Contributions to mean A2 freqs at seg. sites= 0.00000000 0.00000000

Contributions to mean diversities= 8.08772256E-06 8.12023882E-06

Contributions to delta-theta values= 0.631025612 0.643715501

Mean load statistics over all zones

Loads within In and St= 3.17912698E-02 1.07782462E-03

Load between In and St= 5.67941740E-03

Homozygous load for In and St= 3.33677046E-02 2.31639273E-03

Inbreeding loads= 1.57644274E-03 1.23856659E-03

Selection coefficients for In and St homokaryotypes

2.57738829E-02 -4.61220741E-03

Mean frequencies of A2 in In and St= 0.109390743 4.48856801E-02

Ratio of these= 2.43709660

Mean diversities at selected sites in In and St= 1.04321435E-03 4.26412281E-03

Mean diversities at neutral sites in In and St= 3.17879673E-03 2.71698460E-02

pi-n/pi-s for In and St= 0.328179002 0.156943202

Ratio of these= 2.09106851

Mean freqs. of seg. sites= 4.23979480E-03 1.80138368E-02

Ratio of these= 0.235363230

Overall delta-theta values= 0.127067924 0.160201192

Ratio of these= 0.793177128

**h = 0.25**

Zone 1: quasi-neutral zone

Upper bound scaled selection coefficient for neutrality in St metapopulation= 0.250000000

Probability of zone 1= 4.39136960E-02

Integral of selection coefficient over zone 1= 7.03745172E-05

Mean load statistics for zone 1

Mean q1 and q2= 0.600000024

F1 and F2= 0.993377507 0.943396151

Diversities= 3.17879673E-03 2.71698460E-02

Contributions to loads within In and St= 4.21687837E-05 4.17466945E-05

Contribution to load between In and St = 3.37797683E-05

Contributions to homozygous loads for In and St= 4.22247103E-05 4.22247103E-05

Contributions to inbreeding loads for In and St= 2.33099051E-03 2.33306456E-03

Contributions to selection coefficients for In and St homokaryotypes

8.40425491E-06 7.98702240E-06

Contributions to mean A2 freqs= 2.63482183E-02 2.63482183E-02

Contributions to mean diversities= 1.39592710E-04 1.19312841E-03

Contributions to mean freqs. of seg. site= 4.94409469E-04 4.17755544E-03

Delta-theta values= -1.67715549E-03 -1.32502317E-02

Zone 2a: moderate selection; cut-off at moderate gamma for St population

Lower and upper bounds of St popn gamma

0.250000000 416.666656

Probability of zone 2a= 0.359450161

Coefficients for bivariate distribution of q1 and q2

a1= 2.50000004E-02 a2= 0.224999994

b11= 2.50000018E-03 b12= 4.49999981E-02 b22= 0.202499986

Net probability of zone 2a using Simpsons rule= 0.360651016

Contributions to mean load statistics over zone 2a

Contributions to mean loads within In and St= 1.88607629E-02 7.22432043E-04

Contribution to load between In and St= 5.20223146E-03

Contributions to homozygous loads for In and St= 1.90878008E-02 9.53411451E-04

Contributions to inbreeding loads= 2.27054174E-04 2.30979276E-04

Selection coefficients for In and St homokaryotypes

1.35656595E-02 -4.48989868E-03

Selection coefficients for In and St homokaryotypes

1.35656595E-02 -4.48989868E-03

Contributions to mean A2 freqs= 7.11472854E-02 1.83663778E-02

Contributions to mean diversities= 7.40584685E-04 2.70992913E-03

Contributions to mean proportions of seg. sites= 2.94167572E-03 1.19951144E-02

Contributions to delta-theta values= 0.106835008 0.198496699

Zone 2b: moderate selection; gamma for St reaches high value

Lower and upper bounds of St popn gamma

416.666656 4166.66650

Probability of zone 2b 0.348782212

Net probability of zone 2b using Simpsons rule= 0.348782301

Contributions to mean load statistics over zone 2b

Contributions to mean loads within In and St= 2.14815620E-04 5.39636385E-05

Contribution to load between In and St= 1.32292756E-04

Contributions to homozygous loads for In and St= 4.21547069E-04 1.07593572E-04

Contributions to inbreeding loads= 2.06731493E-04 5.36298649E-05

Selection coefficients for In and St homokaryotypes

8.24928284E-05 -7.83205032E-05

Selection coefficients for In and St homokaryotypes

8.24928284E-05 -7.83205032E-05

Contributions to mean A2 freqs= 1.54896570E-05 4.94776714E-06

Contributions to mean diversities= 3.01817072E-05 9.85926272E-06

Contributions to mean proportions of seg. sites= 2.54451006E-04 1.01439313E-04

Contributions to delta-theta values= 0.579184830 0.655182004

Zone 3: strong selection approximation

Lower and upper bounds of St popn gamma= 4166.66650 30000.0020

Zone 3: strong selection approximation

Probability of zone 3= 0.235996842

Mean load statistics over zone 3

Contributions to loads within In and St= 2.74672406E-04 2.74130929E-04

Contribution to load between In and St= 2.74063001E-04

Contributions to homozygous loads for In and St= 5.48121461E-04 5.48121403E-04

Contributions to inbreeding loads= 2.73448910E-04 2.73990328E-04

Selection coefficients for In and St homokaryotypes

5.96046448E-07 5.96046448E-08

Contributions to mean A2 freqs= 2.43733462E-06 2.43733439E-06

Contributions to mean A2 freqs at seg. sites= 0.00000000 0.00000000

Contributions to mean diversities= 4.86144654E-06 4.87315128E-06

Contributions to delta-theta values= 0.636854768 0.644320369

Mean load statistics over all zones

Loads within In and St= 1.93924196E-02 1.09227328E-03

Load between In and St= 5.64236706E-03

Homozygous load for In and St= 2.00996920E-02 1.65135122E-03

Inbreeding loads= 7.07290485E-04 5.59077482E-04

Selection coefficients for In and St homokaryotypes

1.36559606E-02 -4.56047058E-03

Mean frequencies of A2 in In and St= 9.75134298E-02 4.47219796E-02

Ratio of these= 2.18043637

Mean diversities at selected sites in In and St= 9.15220531E-04 3.91779002E-03

Mean diversities at neutral sites in In and St= 3.17879673E-03 2.71698460E-02

pi-n/pi-s for In and St= 0.287914127 0.144196257

Ratio of these= 1.99668241

Mean freqs. of seg. sites= 3.73803009E-03 1.63227171E-02

Ratio of these= 0.229007840

Overall delta-theta values= 0.131370187 0.148469031

Ratio of these= 0.884832263

**h = 0.35**

Zone 1: quasi-neutral zone

Upper bound scaled selection coefficient for neutrality in St metapopulation= 0.250000000

Probability of zone 1= 4.39136960E-02

Integral of selection coefficient over zone 1= 7.03745172E-05

Mean load statistics for zone 1

Mean q1 and q2= 0.600000024

F1 and F2= 0.993377507 0.943396151

Diversities= 3.17879673E-03 2.71698460E-02

Contributions to loads within In and St= 4.21911536E-05 4.19379030E-05

Contribution to load between In and St = 3.71577444E-05

Contributions to homozygous loads for In and St= 4.22247103E-05 4.22247103E-05

Contributions to inbreeding loads for In and St= 9.99397598E-04 9.99930780E-04

Contributions to selection coefficients for In and St homokaryotypes

5.00679016E-06 4.76837158E-06

Contributions to mean A2 freqs= 2.63482183E-02 2.63482183E-02

Contributions to mean diversities= 1.39592710E-04 1.19312841E-03

Contributions to mean freqs. of seg. site= 4.94409469E-04 4.17755544E-03

Delta-theta values= -1.67715549E-03 -1.32502317E-02

Zone 2a: moderate selection; cut-off at moderate gamma for St population

Lower and upper bounds of St popn gamma

0.250000000 297.619049

Probability of zone 2a= 0.321548969

Coefficients for bivariate distribution of q1 and q2

a1= 3.50000001E-02 a2= 0.314999998

b11= 1.50000013E-03 b12= 2.70000007E-02 b22= 0.121499993

Net probability of zone 2a using Simpsons rule= 0.322079927

Contributions to mean load statistics over zone 2a

Contributions to mean loads within In and St= 1.31388111E-02 7.17241142E-04

Contribution to load between In and St= 5.02178771E-03

Contributions to homozygous loads for In and St= 1.32296542E-02 8.16606916E-04

Contributions to inbreeding loads= 9.08392540E-05 9.93647263E-05

Selection coefficients for In and St homokaryotypes

8.08417797E-03 -4.31382656E-03

Selection coefficients for In and St homokaryotypes

8.08417797E-03 -4.31382656E-03

Contributions to mean A2 freqs= 6.31132573E-02 1.78409647E-02

Contributions to mean diversities= 6.59651123E-04 2.43345415E-03

Contributions to mean proportions of seg. sites= 2.62574106E-03 1.07383663E-02

Contributions to delta-theta values= 0.108719945 0.196035802

Zone 2b: moderate selection; gamma for St reaches high value

Lower and upper bounds of St popn gamma

297.619049 2976.19043

Probability of zone 2b 0.328705907

Net probability of zone 2b using Simpsons rule= 0.328705996

Contributions to mean load statistics over zone 2b

Contributions to mean loads within In and St= 2.23203344E-04 6.37186677E-05

Contribution to load between In and St= 1.42539458E-04

Contributions to homozygous loads for In and St= 3.16336460E-04 9.09073497E-05

Contributions to inbreeding loads= 9.31333634E-05 2.71886674E-05

Selection coefficients for In and St homokaryotypes

8.06450844E-05 -7.87973404E-05

Selection coefficients for In and St homokaryotypes

8.06450844E-05 -7.87973404E-05

Contributions to mean A2 freqs= 1.57057639E-05 5.72349518E-06

Contributions to mean diversities= 3.06135007E-05 1.14052300E-05

Contributions to mean proportions of seg. sites= 2.58348038E-04 1.17451978E-04

Contributions to delta-theta values= 0.579603076 0.655495048

Zone 3: strong selection approximation

Lower and upper bounds of St popn gamma= 2976.19043 30000.0020

Zone 3: strong selection approximation

Probability of zone 3= 0.293974340

Mean load statistics over zone 3

Contributions to loads within In and St= 3.26631125E-04 3.26382375E-04

Contribution to load between In and St= 3.26351292E-04

Contributions to homozygous loads for In and St= 4.66214406E-04 4.66214406E-04

Contributions to inbreeding loads= 1.39583761E-04 1.39832191E-04

Selection coefficients for In and St homokaryotypes

2.98023224E-07 5.96046448E-08

Contributions to mean A2 freqs= 2.60861430E-06 2.60861430E-06

Contributions to mean A2 freqs at seg. sites= 0.00000000 0.00000000

Contributions to mean diversities= 5.20376170E-06 5.21568518E-06

Contributions to delta-theta values= 0.637263298 0.644368649

Mean load statistics over all zones

Loads within In and St= 1.37308370E-02 1.14928011E-03

Load between In and St= 5.52783627E-03

Homozygous load for In and St= 1.40544297E-02 1.41595339E-03

Inbreeding loads= 3.23589949E-04 2.66672403E-04

Selection coefficients for In and St homokaryotypes

8.16947222E-03 -4.38821316E-03

Mean frequencies of A2 in In and St= 8.94797891E-02 4.41975147E-02

Ratio of these= 2.02454352

Mean diversities at selected sites in In and St= 8.35061073E-04 3.64320353E-03

Mean diversities at neutral sites in In and St= 3.17879673E-03 2.71698460E-02

pi-n/pi-s for In and St= 0.262697220 0.134089962

Ratio of these= 1.95911181

Mean freqs. of seg. sites= 3.42939375E-03 1.50854047E-02

Ratio of these= 0.227331907

Overall delta-theta values= 0.136121571 0.143202424

Ratio of these= 0.950553536

**h = 0.45**

Zone 1: quasi-neutral zone

Upper bound scaled selection coefficient for neutrality in St metapopulation= 0.250000000

Probability of zone 1= 4.39136960E-02

Integral of selection coefficient over zone 1= 7.03745172E-05

Mean load statistics for zone 1

Mean q1 and q2= 0.600000024

F1 and F2= 0.993377507 0.943396151

Diversities= 3.17879673E-03 2.71698460E-02

Contributions to loads within In and St= 4.22135308E-05 4.21291079E-05

Contribution to load between In and St = 4.05357205E-05

Contributions to homozygous loads for In and St= 4.22247103E-05 4.22247103E-05

Contributions to inbreeding loads for In and St= 4.28387051E-04 4.28550382E-04

Contributions to selection coefficients for In and St homokaryotypes

1.66893005E-06 1.60932541E-06

Contributions to mean A2 freqs= 2.63482183E-02 2.63482183E-02

Contributions to mean diversities= 1.39592710E-04 1.19312841E-03

Contributions to mean freqs. of seg. site= 4.94409469E-04 4.17755544E-03

Delta-theta values= -1.67715549E-03 -1.32502317E-02

Zone 2a: moderate selection; cut-off at moderate gamma for St population

Lower and upper bounds of St popn gamma

0.250000000 165.343918

Probability of zone 2a= 0.263242185

Coefficients for bivariate distribution of q1 and q2

a1= 4.49999981E-02 a2= 0.404999971

b11= 5.00000140E-04 b12= 9.00000241E-03 b22= 4.05000076E-02

Net probability of zone 2a using Simpsons rule= 0.263346374

Contributions to mean load statistics over zone 2a

Contributions to mean loads within In and St= 9.89173166E-03 6.92878442E-04

Contribution to load between In and St= 4.81561711E-03

Contributions to homozygous loads for In and St= 9.91057511E-03 7.16150273E-04

Contributions to inbreeding loads= 1.88466565E-05 2.32728707E-05

Selection coefficients for In and St homokaryotypes

5.06323576E-03 -4.13119793E-03

Selection coefficients for In and St homokaryotypes

5.06323576E-03 -4.13119793E-03

Contributions to mean A2 freqs= 5.73872365E-02 1.75012257E-02

Contributions to mean diversities= 5.90510957E-04 2.21102429E-03

Contributions to mean proportions of seg. sites= 2.31468398E-03 9.71660484E-03

Contributions to delta-theta values= 9.49178338E-02 0.192707837

Zone 2b: moderate selection; gamma for St reaches high value

Lower and upper bounds of St popn gamma

165.343918 1653.43921

Probability of zone 2b 0.288836628

Net probability of zone 2b using Simpsons rule= 0.288836628

Contributions to mean load statistics over zone 2b

Contributions to mean loads within In and St= 2.30604419E-04 8.75410624E-05

Contribution to load between In and St= 1.58736962E-04

Contributions to homozygous loads for In and St= 2.55518215E-04 9.72262133E-05

Contributions to inbreeding loads= 2.49137520E-05 9.68517543E-06

Selection coefficients for In and St homokaryotypes

7.18832016E-05 -7.11679459E-05

Selection coefficients for In and St homokaryotypes

7.18832016E-05 -7.11679459E-05

Contributions to mean A2 freqs= 2.16869303E-05 1.02621589E-05

Contributions to mean diversities= 4.18552772E-05 2.04267726E-05

Contributions to mean proportions of seg. sites= 3.34860611E-04 2.04165277E-04

Contributions to delta-theta values= 0.556556880 0.645048022

Zone 3: strong selection approximation

Lower and upper bounds of St popn gamma= 1653.43921 30000.0020

Zone 3: strong selection approximation

Probability of zone 3= 0.392150402

Mean load statistics over zone 3

Contributions to loads within In and St= 4.15130955E-04 4.15036309E-04

Contribution to load between In and St= 4.15024610E-04

Contributions to homozygous loads for In and St= 4.61137737E-04 4.61137650E-04

Contributions to inbreeding loads= 4.60070623E-05 4.61015406E-05

Selection coefficients for In and St homokaryotypes

1.19209290E-07 0.00000000

Contributions to mean A2 freqs= 3.82865755E-06 3.82865755E-06

Contributions to mean A2 freqs at seg. sites= 0.00000000 0.00000000

Contributions to mean diversities= 7.63169646E-06 7.65437380E-06

Contributions to delta-theta values= 0.634790182 0.644107521

Mean load statistics over all zones

Loads within In and St= 1.05796801E-02 1.23758498E-03

Load between In and St= 5.42991469E-03

Homozygous load for In and St= 1.06694559E-02 1.31673890E-03

Inbreeding loads= 8.97786522E-05 7.91551865E-05

Selection coefficients for In and St homokaryotypes

5.13654947E-03 -4.20117378E-03

Mean frequencies of A2 in In and St= 8.37609693E-02 4.38635387E-02

Ratio of these= 1.90958071

Mean diversities at selected sites in In and St= 7.79590686E-04 3.43223359E-03

Mean diversities at neutral sites in In and St= 3.17879673E-03 2.71698460E-02

pi-n/pi-s for In and St= 0.245247096 0.126325101

Ratio of these= 1.94139636

Mean freqs. of seg. sites= 3.21809016E-03 1.41746290E-02

Ratio of these= 0.227031708

Overall delta-theta values= 0.140550852 0.140953064

Ratio of these= 0.997146487

**h = 0.5**

Zone 1: quasi-neutral zone

Upper bound scaled selection coefficient for neutrality in St metapopulation= 0.250000000

Probability of zone 1= 4.39136960E-02

Integral of selection coefficient over zone 1= 7.03745172E-05

Mean load statistics for zone 1

Mean q1 and q2= 0.600000024

F1 and F2= 0.993377507 0.943396151

Diversities= 3.17879673E-03 2.71698460E-02

Contributions to loads within In and St= 4.22247103E-05 4.22247103E-05

Contribution to load between In and St = 4.22247103E-05

Contributions to homozygous loads for In and St= 4.22247103E-05 4.22247103E-05

Contributions to inbreeding loads for In and St= 1.11074034E-04 1.11106907E-04

Contributions to selection coefficients for In and St homokaryotypes

0.00000000 0.00000000

Contributions to mean A2 freqs= 2.63482183E-02 2.63482183E-02

Contributions to mean diversities= 1.39592710E-04 1.19312841E-03

Contributions to mean freqs. of seg. site= 4.94409469E-04 4.17755544E-03

Delta-theta values= -1.67715549E-03 -1.32502317E-02

Zone 2a: moderate selection; cut-off at moderate gamma for St population

Lower and upper bounds of St popn gamma

0.250000000 82.6719589

Probability of zone 2a= 0.205970049

Coefficients for bivariate distribution of q1 and q2

a1= 5.00000007E-02 a2= 0.449999988

b11= 0.00000000 b12= 0.00000000 b22= 0.00000000

Net probability of zone 2a using Simpsons rule= 0.205981016

Contributions to mean load statistics over zone 2a

Contributions to mean loads within In and St= 8.71754345E-03 6.53552590E-04

Contribution to load between In and St= 4.68554860E-03

Contributions to homozygous loads for In and St= 8.71754345E-03 6.53552590E-04

Contributions to inbreeding loads= 0.00000000 0.00000000

Selection coefficients for In and St homokaryotypes

4.02384996E-03 -4.04012203E-03

Selection coefficients for In and St homokaryotypes

4.02384996E-03 -4.04012203E-03

Contributions to mean A2 freqs= 5.50988168E-02 1.74039025E-02

Contributions to mean diversities= 5.36911248E-04 2.09787884E-03

Contributions to mean proportions of seg. sites= 2.03666068E-03 9.10409912E-03

Contributions to delta-theta values= 6.47330880E-02 0.182485998

Zone 2b: moderate selection; gamma for St reaches high value

Lower and upper bounds of St popn gamma

82.6719589 826.719604

Probability of zone 2b 0.241704941

Net probability of zone 2b using Simpsons rule= 0.241705000

Contributions to mean load statistics over zone 2b

Contributions to mean loads within In and St= 2.27226061E-04 1.16033712E-04

Contribution to load between In and St= 1.71629930E-04

Contributions to homozygous loads for In and St= 2.27226061E-04 1.16033712E-04

Contributions to inbreeding loads= 0.00000000 0.00000000

Selection coefficients for In and St homokaryotypes

5.56111336E-05 -5.55515289E-05

Selection coefficients for In and St homokaryotypes

5.56111336E-05 -5.55515289E-05

Contributions to mean A2 freqs= 3.78962868E-05 2.22412200E-05

Contributions to mean diversities= 6.86991043E-05 4.41348966E-05

Contributions to mean proportions of seg. sites= 4.87294776E-04 4.20484896E-04

Contributions to delta-theta values= 0.499837577 0.627622485

Zone 3: strong selection approximation

Lower and upper bounds of St popn gamma= 826.719604 30000.0020

Zone 3: strong selection approximation

Probability of zone 3= 0.496554226

Mean load statistics over zone 3

Contributions to loads within In and St= 5.10625541E-04 5.10625541E-04

Contribution to load between In and St= 5.10625483E-04

Contributions to homozygous loads for In and St= 5.10625541E-04 5.10625541E-04

Contributions to inbreeding loads= 0.00000000 0.00000000

Selection coefficients for In and St homokaryotypes

0.00000000 0.00000000

Contributions to mean A2 freqs= 6.69356996E-06 6.69356996E-06

Contributions to mean A2 freqs at seg. sites= 0.00000000 0.00000000

Contributions to mean diversities= 1.33125513E-05 1.33785752E-05

Contributions to delta-theta values= 0.627279639 0.643347979

Mean load statistics over all zones

Loads within In and St= 9.49761923E-03 1.32243661E-03

Load between In and St= 5.41002862E-03

Homozygous load for In and St= 9.49761923E-03 1.32243661E-03

Inbreeding loads= 0.00000000 0.00000000

Selection coefficients for In and St homokaryotypes

4.07922268E-03 -4.09591198E-03

Mean frequencies of A2 in In and St= 8.14916193E-02 4.37810533E-02

Ratio of these= 1.86134446

Mean diversities at selected sites in In and St= 7.58515671E-04 3.34852072E-03

Mean diversities at neutral sites in In and St= 3.17879673E-03 2.71698460E-02

pi-n/pi-s for In and St= 0.238617226 0.123244010

Ratio of these= 1.93613648

Mean freqs. of seg. sites= 3.14508053E-03 1.38352206E-02

Ratio of these= 0.227324203

Overall delta-theta values= 0.144372880 0.141345084

Ratio of these= 1.02142131

**Inversion frequency= 0.3**

**h = 0.05**

Zone 1: quasi-neutral zone

Upper bound scaled selection coefficient for neutrality in St metapopulation= 0.250000000

Probability of zone 1= 4.73524816E-02

Integral of selection coefficient over zone 1= 9.75669318E-05

Mean load statistics for zone 1

Mean q1 and q2= 0.600000024

F1 and F2= 0.980392158 0.955414057

Diversities= 9.41176433E-03 2.14012526E-02

Contributions to loads within In and St= 5.81269378E-05 5.76005368E-05

Contribution to load between In and St = 3.74657029E-05

Contributions to homozygous loads for In and St= 5.85401613E-05 5.85401613E-05

Contributions to inbreeding loads for In and St= 0.00000000 0.00000000

Contributions to selection coefficients for In and St homokaryotypes

2.06828117E-05 2.01463699E-05

Contributions to mean A2 freqs= 2.84114908E-02 2.84114908E-02

Contributions to mean diversities= 4.45670390E-04 1.01340248E-03

Contributions to mean freqs. of seg. site= 1.57413294E-03 3.55867785E-03

Delta-theta values= -4.44030762E-03 -1.02876425E-02

Zone 2a: moderate selection; cut-off at moderate gamma for St population

Lower and upper bounds of St popn gamma

0.250000000 250.000000

Probability of zone 2a= 0.326476455

Coefficients for bivariate distribution of q1 and q2

a1= 1.50000006E-02 a2= 3.50000001E-02

b11= 4.05000001E-02 b12= 0.189000010 b22= 0.220499992

Net probability of zone 2a using Simpsons rule= 0.326839477

Contributions to mean load statistics over zone 2a

Contributions to mean loads within In and St= 7.46046705E-03 8.83681467E-04

Contribution to load between In and St= 8.78445164E-04

Contributions to homozygous loads for In and St= 8.51951353E-03 1.87575829E-03

Contributions to inbreeding loads= 1.05905195E-03 9.92077054E-04

Selection coefficients for In and St homokaryotypes

6.56038523E-03 5.24520874E-06

Selection coefficients for In and St homokaryotypes

6.56038523E-03 5.24520874E-06

Contributions to mean A2 freqs= 4.84889336E-02 1.95827987E-02

Contributions to mean diversities= 2.14403844E-03 3.06993537E-03

Contributions to mean proportions of seg. sites= 8.51065945E-03 1.38148861E-02

Contributions to delta-theta values= 0.106239617 0.211623490

Zone 2b: moderate selection; gamma for St reaches high value

Lower and upper bounds of St popn gamma

250.000000 2500.00000

Probability of zone 2b 0.333575726

Net probability of zone 2b using Simpsons rule= 0.333575726

Contributions to mean load statistics over zone 2b

Contributions to mean loads within In and St= 1.65167570E-04 9.07109861E-05

Contribution to load between In and St= 1.04976381E-04

Contributions to homozygous loads for In and St= 1.28868839E-03 8.03927658E-04

Contributions to inbreeding loads= 1.12351915E-03 7.13216315E-04

Selection coefficients for In and St homokaryotypes

6.02006912E-05 -1.43051147E-05

Selection coefficients for In and St homokaryotypes

6.02006912E-05 -1.43051147E-05

Contributions to mean A2 freqs= 6.22887237E-05 4.12913032E-05

Contributions to mean diversities= 1.19574535E-04 8.11101563E-05

Contributions to mean proportions of seg. sites= 9.22168081E-04 7.12103210E-04

Contributions to delta-theta values= 0.539976120 0.595904469

Zone 3: strong selection approximation

Lower and upper bounds of St popn gamma= 2500.00000 23333.3340

Zone 3: strong selection approximation

Probability of zone 3= 0.280738235

Mean load statistics over zone 3

Contributions to loads within In and St= 3.27099580E-04 3.19943734E-04

Contribution to load between In and St= 3.14577162E-04

Contributions to homozygous loads for In and St= 3.14426585E-03 3.14426585E-03

Contributions to inbreeding loads= 2.81716790E-03 2.82432674E-03

Selection coefficients for In and St homokaryotypes

1.25169754E-05 5.36441803E-06

Contributions to mean A2 freqs= 1.66967566E-05 1.66967566E-05

Contributions to mean A2 freqs at seg. sites= 0.00000000 0.00000000

Contributions to mean diversities= 3.32037052E-05 3.33108474E-05

Contributions to delta-theta values= 0.627326846 0.637813926

Mean load statistics over all zones

Loads within In and St= 8.01086146E-03 1.35193672E-03

Load between In and St= 1.33546442E-03

Homozygous load for In and St= 1.30110085E-02 5.88249229E-03

Inbreeding loads= 5.00015216E-03 4.53055976E-03

Selection coefficients for In and St homokaryotypes

6.65318966E-03 1.64508820E-05

Mean frequencies of A2 in In and St= 7.69794062E-02 4.80522774E-02

Ratio of these= 1.60199285

Mean diversities at selected sites in In and St= 2.74248724E-03 4.19775862E-03

Mean diversities at neutral sites in In and St= 9.41176433E-03 2.14012526E-02

pi-n/pi-s for In and St= 0.291389287 0.196145460

Ratio of these= 1.48557746

Mean freqs. of seg. sites= 1.13230497E-02 1.84119567E-02

Ratio of these= 0.614983499

Overall delta-theta values= 0.140723467 0.191147566

Ratio of these= 0.736203313

**h = 0.15**

Zone 1: quasi-neutral zone

Upper bound scaled selection coefficient for neutrality in St metapopulation= 0.250000000

Probability of zone 1= 4.73524816E-02

Integral of selection coefficient over zone 1= 9.75669318E-05

Mean load statistics for zone 1

Mean q1 and q2= 0.600000024

F1 and F2= 0.980392158 0.955414057

Diversities= 9.41176433E-03 2.14012526E-02

Contributions to loads within In and St= 5.82187640E-05 5.78093423E-05

Contribution to load between In and St = 4.21489167E-05

Contributions to homozygous loads for In and St= 5.85401613E-05 5.85401613E-05

Contributions to inbreeding loads for In and St= 8.99089221E-03 8.99603497E-03

Contributions to selection coefficients for In and St homokaryotypes

1.60932541E-05 1.56760216E-05

Contributions to mean A2 freqs= 2.84114908E-02 2.84114908E-02

Contributions to mean diversities= 4.45670390E-04 1.01340248E-03

Contributions to mean freqs. of seg. site= 1.57413294E-03 3.55867785E-03

Delta-theta values= -4.44030762E-03 -1.02876425E-02

Zone 2a: moderate selection; cut-off at moderate gamma for St population

Lower and upper bounds of St popn gamma

0.250000000 416.666656

Probability of zone 2a= 0.386616915

Coefficients for bivariate distribution of q1 and q2

a1= 4.50000018E-02 a2= 0.105000004

b11= 3.15000005E-02 b12= 0.147000000 b22= 0.171499997

Net probability of zone 2a using Simpsons rule= 0.387911767

Contributions to mean load statistics over zone 2a

Contributions to mean loads within In and St= 5.05852420E-03 9.61126236E-04

Contribution to load between In and St= 1.32623606E-03

Contributions to homozygous loads for In and St= 5.61055308E-03 1.47294707E-03

Contributions to inbreeding loads= 5.52027486E-04 5.11818624E-04

Selection coefficients for In and St homokaryotypes

3.72534990E-03 -3.65138054E-04

Selection coefficients for In and St homokaryotypes

3.72534990E-03 -3.65138054E-04

Contributions to mean A2 freqs= 4.25540954E-02 2.00081989E-02

Contributions to mean diversities= 1.73471507E-03 2.63331272E-03

Contributions to mean proportions of seg. sites= 7.17835827E-03 1.16553716E-02

Contributions to delta-theta values= 0.142656624 0.198454738

Zone 2b: moderate selection; gamma for St reaches high value

Lower and upper bounds of St popn gamma

416.666656 4166.66650

Probability of zone 2b 0.360976309

Net probability of zone 2b using Simpsons rule= 0.360976666

Contributions to mean load statistics over zone 2b

Contributions to mean loads within In and St= 1.13644142E-04 6.12920048E-05

Contribution to load between In and St= 8.59497741E-05

Contributions to homozygous loads for In and St= 3.70757974E-04 2.02140203E-04

Contributions to inbreeding loads= 2.57113599E-04 1.40848206E-04

Selection coefficients for In and St homokaryotypes

2.77161598E-05 -2.46763229E-05

Selection coefficients for In and St homokaryotypes

2.77161598E-05 -2.46763229E-05

Contributions to mean A2 freqs= 1.19438664E-05 7.02603393E-06

Contributions to mean diversities= 2.36065734E-05 1.39740732E-05

Contributions to mean proportions of seg. sites= 2.18904272E-04 1.38244723E-04

Contributions to delta-theta values= 0.617412806 0.641386867

Zone 3: strong selection approximation

Lower and upper bounds of St popn gamma= 4166.66650 23333.3340

Zone 3: strong selection approximation

Probability of zone 3= 0.193197191

Mean load statistics over zone 3

Contributions to loads within In and St= 2.35773317E-04 2.35447500E-04

Contribution to load between In and St= 2.35203202E-04

Contributions to homozygous loads for In and St= 7.83988042E-04 7.83988042E-04

Contributions to inbreeding loads= 5.48214710E-04 5.48540789E-04

Selection coefficients for In and St homokaryotypes

5.96046448E-07 2.38418579E-07

Contributions to mean A2 freqs= 2.93092967E-06 2.93092967E-06

Contributions to mean A2 freqs at seg. sites= 0.00000000 0.00000000

Contributions to mean diversities= 5.85465432E-06 5.85872067E-06

Contributions to delta-theta values= 0.641527772 0.643653572

Mean load statistics over all zones

Loads within In and St= 5.46616036E-03 1.31567509E-03

Load between In and St= 1.68953789E-03

Homozygous load for In and St= 6.82383962E-03 2.51761544E-03

Inbreeding loads= 1.35767716E-03 1.20193837E-03

Selection coefficients for In and St homokaryotypes

3.76951694E-03 -3.73959541E-04

Mean frequencies of A2 in In and St= 7.09804595E-02 4.84296493E-02

Ratio of these= 1.46564054

Mean diversities at selected sites in In and St= 2.20984663E-03 3.66654783E-03

Mean diversities at neutral sites in In and St= 9.41176433E-03 2.14012526E-02

pi-n/pi-s for In and St= 0.234796211 0.171323985

Ratio of these= 1.37048066

Mean freqs. of seg. sites= 9.02933814E-03 1.54106226E-02

Ratio of these= 0.585916519

Overall delta-theta values= 0.131723642 0.155909717

Ratio of these= 0.844871283

**h = 0.25**

Zone 1: quasi-neutral zone

Upper bound scaled selection coefficient for neutrality in St metapopulation= 0.250000000

Probability of zone 1= 4.73524816E-02

Integral of selection coefficient over zone 1= 9.75669318E-05

Mean load statistics for zone 1

Mean q1 and q2= 0.600000024

F1 and F2= 0.980392158 0.955414057

Diversities= 9.41176433E-03 2.14012526E-02

Contributions to loads within In and St= 5.83105902E-05 5.80181440E-05

Contribution to load between In and St = 4.68321305E-05

Contributions to homozygous loads for In and St= 5.85401613E-05 5.85401613E-05

Contributions to inbreeding loads for In and St= 2.33254582E-03 2.33299052E-03

Contributions to selection coefficients for In and St homokaryotypes

1.15036964E-05 1.12056732E-05

Contributions to mean A2 freqs= 2.84114908E-02 2.84114908E-02

Contributions to mean diversities= 4.45670390E-04 1.01340248E-03

Contributions to mean freqs. of seg. site= 1.57413294E-03 3.55867785E-03

Delta-theta values= -4.44030762E-03 -1.02876425E-02

Zone 2a: moderate selection; cut-off at moderate gamma for St population

Lower and upper bounds of St popn gamma

0.250000000 416.666656

Probability of zone 2a= 0.386616915

Coefficients for bivariate distribution of q1 and q2

a1= 7.50000030E-02 a2= 0.174999997

b11= 2.25000009E-02 b12= 0.105000004 b22= 0.122499995

Net probability of zone 2a using Simpsons rule= 0.387911767

Contributions to mean load statistics over zone 2a

Contributions to mean loads within In and St= 3.80115467E-03 9.88517539E-04

Contribution to load between In and St= 1.51634158E-03

Contributions to homozygous loads for In and St= 4.06836439E-03 1.23957335E-03

Contributions to inbreeding loads= 2.67207972E-04 2.51057238E-04

Selection coefficients for In and St homokaryotypes

2.28220224E-03 -5.27977943E-04

Selection coefficients for In and St homokaryotypes

2.28220224E-03 -5.27977943E-04

Contributions to mean A2 freqs= 3.83072682E-02 1.99818350E-02

Contributions to mean diversities= 1.48735242E-03 2.31508189E-03

Contributions to mean proportions of seg. sites= 6.24648808E-03 1.02052027E-02

Contributions to delta-theta values= 0.155247033 0.195184231

Zone 2b: moderate selection; gamma for St reaches high value

Lower and upper bounds of St popn gamma

416.666656 4166.66650

Probability of zone 2b 0.360976309

Net probability of zone 2b using Simpsons rule= 0.360976666

Contributions to mean load statistics over zone 2b

Contributions to mean loads within In and St= 1.06785657E-04 5.45302137E-05

Contribution to load between In and St= 8.02598370E-05

Contributions to homozygous loads for In and St= 2.12302621E-04 1.08722306E-04

Contributions to inbreeding loads= 1.05516905E-04 5.41921727E-05

Selection coefficients for In and St homokaryotypes

2.65240669E-05 -2.57492065E-05

Selection coefficients for In and St homokaryotypes

2.65240669E-05 -2.57492065E-05

Contributions to mean A2 freqs= 6.96385405E-06 3.91629828E-06

Contributions to mean diversities= 1.38239302E-05 7.80387836E-06

Contributions to mean proportions of seg. sites= 1.33851994E-04 8.06132448E-05

Contributions to delta-theta values= 0.633597493 0.656556070

Zone 3: strong selection approximation

Lower and upper bounds of St popn gamma= 4166.66650 23333.3340

Zone 3: strong selection approximation

Probability of zone 3= 0.193197191

Mean load statistics over zone 3

Contributions to loads within In and St= 2.35344822E-04 2.35261148E-04

Contribution to load between In and St= 2.35198124E-04

Contributions to homozygous loads for In and St= 4.70392959E-04 4.70392959E-04

Contributions to inbreeding loads= 2.35048123E-04 2.35131825E-04

Selection coefficients for In and St homokaryotypes

1.19209290E-07 5.96046448E-08

Contributions to mean A2 freqs= 1.75855769E-06 1.75855769E-06

Contributions to mean A2 freqs at seg. sites= 0.00000000 0.00000000

Contributions to mean diversities= 3.51451854E-06 3.51598555E-06

Contributions to delta-theta values= 0.643017411 0.644283414

Mean load statistics over all zones

Loads within In and St= 4.20159567E-03 1.33632706E-03

Load between In and St= 1.87863177E-03

Homozygous load for In and St= 4.80959984E-03 1.87722885E-03

Inbreeding loads= 6.08002534E-04 5.40903246E-04

Selection coefficients for In and St homokaryotypes

2.32028961E-03 -5.42402267E-04

Mean frequencies of A2 in In and St= 6.67274818E-02 4.83989976E-02

Ratio of these= 1.37869549

Mean diversities at selected sites in In and St= 1.95036130E-03 3.33980424E-03

Mean diversities at neutral sites in In and St= 9.41176433E-03 2.14012526E-02

pi-n/pi-s for In and St= 0.207225889 0.156056479

Ratio of these= 1.32789028

Mean freqs. of seg. sites= 7.98940007E-03 1.38795609E-02

Ratio of these= 0.575623393

Overall delta-theta values= 0.133930624 0.146316171

Ratio of these= 0.915350795

**h = 0.35**

Zone 1: quasi-neutral zone

Upper bound scaled selection coefficient for neutrality in St metapopulation= 0.250000000

Probability of zone 1= 4.73524816E-02

Integral of selection coefficient over zone 1= 9.75669318E-05

Mean load statistics for zone 1

Mean q1 and q2= 0.600000024

F1 and F2= 0.980392158 0.955414057

Diversities= 9.41176433E-03 2.14012526E-02

Contributions to loads within In and St= 5.84024165E-05 5.82269531E-05

Contribution to load between In and St = 5.15153406E-05

Contributions to homozygous loads for In and St= 5.85401613E-05 5.85401613E-05

Contributions to inbreeding loads for In and St= 9.99797485E-04 9.99911921E-04

Contributions to selection coefficients for In and St homokaryotypes

6.91413879E-06 6.73532486E-06

Contributions to mean A2 freqs= 2.84114908E-02 2.84114908E-02

Contributions to mean diversities= 4.45670390E-04 1.01340248E-03

Contributions to mean freqs. of seg. site= 1.57413294E-03 3.55867785E-03

Delta-theta values= -4.44030762E-03 -1.02876425E-02

Zone 2a: moderate selection; cut-off at moderate gamma for St population

Lower and upper bounds of St popn gamma

0.250000000 297.619049

Probability of zone 2a= 0.346091360

Coefficients for bivariate distribution of q1 and q2

a1= 0.105000004 a2= 0.244999990

b11= 1.35000013E-02 b12= 6.30000010E-02 b22= 7.34999999E-02

Net probability of zone 2a using Simpsons rule= 0.346664220

Contributions to mean load statistics over zone 2a

Contributions to mean loads within In and St= 3.03746597E-03 9.83134611E-04

Contribution to load between In and St= 1.60167459E-03

Contributions to homozygous loads for In and St= 3.14776227E-03 1.09026453E-03

Contributions to inbreeding loads= 1.10292400E-04 1.07130218E-04

Selection coefficients for In and St homokaryotypes

1.43474340E-03 -6.18696213E-04

Selection coefficients for In and St homokaryotypes

1.43474340E-03 -6.18696213E-04

Contributions to mean A2 freqs= 3.49447988E-02 1.95558090E-02

Contributions to mean diversities= 1.31482678E-03 2.06915196E-03

Contributions to mean proportions of seg. sites= 5.55322878E-03 9.11208615E-03

Contributions to delta-theta values= 0.160008848 0.194387257

Zone 2b: moderate selection; gamma for St reaches high value

Lower and upper bounds of St popn gamma

297.619049 2976.19043

Probability of zone 2b 0.344033331

Net probability of zone 2b using Simpsons rule= 0.344033509

Contributions to mean load statistics over zone 2b

Contributions to mean loads within In and St= 1.21902085E-04 6.49345966E-05

Contribution to load between In and St= 9.32263792E-05

Contributions to homozygous loads for In and St= 1.73712571E-04 9.26422144E-05

Contributions to inbreeding loads= 5.18105808E-05 2.77075869E-05

Selection coefficients for In and St homokaryotypes

2.86698341E-05 -2.82526016E-05

Selection coefficients for In and St homokaryotypes

2.86698341E-05 -2.82526016E-05

Contributions to mean A2 freqs= 7.70933002E-06 4.56212911E-06

Contributions to mean diversities= 1.53052788E-05 9.09103983E-06

Contributions to mean proportions of seg. sites= 1.48272840E-04 9.40473692E-05

Contributions to delta-theta values= 0.633789003 0.657059550

Zone 3: strong selection approximation

Lower and upper bounds of St popn gamma= 2976.19043 23333.3340

Zone 3: strong selection approximation

Probability of zone 3= 0.250665724

Mean load statistics over zone 3

Contributions to loads within In and St= 2.87382660E-04 2.87343166E-04

Contribution to load between In and St= 2.87313538E-04

Contributions to homozygous loads for In and St= 4.10446577E-04 4.10446635E-04

Contributions to inbreeding loads= 1.23064048E-04 1.23103542E-04

Selection coefficients for In and St homokaryotypes

5.96046448E-08 0.00000000

Contributions to mean A2 freqs= 1.93495316E-06 1.93495316E-06

Contributions to mean A2 freqs at seg. sites= 0.00000000 0.00000000

Contributions to mean diversities= 3.86719921E-06 3.86872580E-06

Contributions to delta-theta values= 0.643131256 0.644335270

Mean load statistics over all zones

Loads within In and St= 3.50515288E-03 1.39363937E-03

Load between In and St= 2.03372980E-03

Homozygous load for In and St= 3.79046169E-03 1.65189360E-03

Inbreeding loads= 2.85304792E-04 2.58254557E-04

Selection coefficients for In and St homokaryotypes

1.47032738E-03 -6.40273094E-04

Mean frequencies of A2 in In and St= 6.33659363E-02 4.79737967E-02

Ratio of these= 1.32084477

Mean diversities at selected sites in In and St= 1.77966966E-03 3.09551414E-03

Mean diversities at neutral sites in In and St= 9.41176433E-03 2.14012526E-02

pi-n/pi-s for In and St= 0.189089909 0.144641727

Ratio of these= 1.30729842

Mean freqs. of seg. sites= 7.31407944E-03 1.28034018E-02

Ratio of these= 0.571260631

Overall delta-theta values= 0.136760116 0.142253101

Ratio of these= 0.961385846

**h = 0.45**

Zone 1: quasi-neutral zone

Upper bound scaled selection coefficient for neutrality in St metapopulation= 0.250000000

Probability of zone 1= 4.73524816E-02

Integral of selection coefficient over zone 1= 9.75669318E-05

Mean load statistics for zone 1

Mean q1 and q2= 0.600000024

F1 and F2= 0.980392158 0.955414057

Diversities= 9.41176433E-03 2.14012526E-02

Contributions to loads within In and St= 5.84942500E-05 5.84357585E-05

Contribution to load between In and St = 5.61985507E-05

Contributions to homozygous loads for In and St= 5.85401613E-05 5.85401613E-05

Contributions to inbreeding loads for In and St= 4.28509462E-04 4.28544503E-04

Contributions to selection coefficients for In and St homokaryotypes

2.32458115E-06 2.26497650E-06

Contributions to mean A2 freqs= 2.84114908E-02 2.84114908E-02

Contributions to mean diversities= 4.45670390E-04 1.01340248E-03

Contributions to mean freqs. of seg. site= 1.57413294E-03 3.55867785E-03

Delta-theta values= -4.44030762E-03 -1.02876425E-02

Zone 2a: moderate selection; cut-off at moderate gamma for St population

Lower and upper bounds of St popn gamma

0.250000000 165.343918

Probability of zone 2a= 0.283557355

Coefficients for bivariate distribution of q1 and q2

a1= 0.135000005 a2= 0.314999998

b11= 4.50000120E-03 b12= 2.10000053E-02 b22= 2.45000049E-02

Net probability of zone 2a using Simpsons rule= 0.283669829

Contributions to mean load statistics over zone 2a

Contributions to mean loads within In and St= 2.51495442E-03 9.54966526E-04

Contribution to load between In and St= 1.62499025E-03

Contributions to homozygous loads for In and St= 2.53949221E-03 9.80013399E-04

Contributions to inbreeding loads= 2.45386273E-05 2.50460726E-05

Selection coefficients for In and St homokaryotypes

8.89539719E-04 -6.70194626E-04

Selection coefficients for In and St homokaryotypes

8.89539719E-04 -6.70194626E-04

Contributions to mean A2 freqs= 3.25309895E-02 1.93288419E-02

Contributions to mean diversities= 1.18311041E-03 1.87668484E-03

Contributions to mean proportions of seg. sites= 4.98025445E-03 8.23431369E-03

Contributions to delta-theta values= 0.157198131 0.191433609

Zone 2b: moderate selection; gamma for St reaches high value

Lower and upper bounds of St popn gamma

165.343918 1653.43921

Probability of zone 2b 0.306239307

Net probability of zone 2b using Simpsons rule= 0.306239247

Contributions to mean load statistics over zone 2b

Contributions to mean loads within In and St= 1.49291605E-04 9.01209060E-05

Contribution to load between In and St= 1.19626238E-04

Contributions to homozygous loads for In and St= 1.65741832E-04 1.00091624E-04

Contributions to inbreeding loads= 1.64503654E-05 9.97064944E-06

Selection coefficients for In and St homokaryotypes

2.96831131E-05 -2.95639038E-05

Selection coefficients for In and St homokaryotypes

2.96831131E-05 -2.95639038E-05

Contributions to mean A2 freqs= 1.23217260E-05 8.25924963E-06

Contributions to mean diversities= 2.44024486E-05 1.64401990E-05

Contributions to mean proportions of seg. sites= 2.29749712E-04 1.65001038E-04

Contributions to delta-theta values= 0.623183250 0.646514058

Zone 3: strong selection approximation

Lower and upper bounds of St popn gamma= 1653.43921 23333.3340

Zone 3: strong selection approximation

Probability of zone 3= 0.350993752

Mean load statistics over zone 3

Contributions to loads within In and St= 3.77790653E-04 3.77774733E-04

Contribution to load between In and St= 3.77763499E-04

Contributions to homozygous loads for In and St= 4.19736112E-04 4.19736112E-04

Contributions to inbreeding loads= 4.19459720E-05 4.19616554E-05

Selection coefficients for In and St homokaryotypes

0.00000000 0.00000000

Contributions to mean A2 freqs= 2.94670713E-06 2.94670735E-06

Contributions to mean A2 freqs at seg. sites= 0.00000000 0.00000000

Contributions to mean diversities= 5.88810371E-06 5.89109732E-06

Contributions to delta-theta values= 0.642508686 0.644069433

Mean load statistics over all zones

Loads within In and St= 3.10053118E-03 1.48129789E-03

Load between In and St= 2.17857864E-03

Homozygous load for In and St= 3.18351039E-03 1.55838137E-03

Inbreeding loads= 8.29808778E-05 7.70827828E-05

Selection coefficients for In and St homokaryotypes

9.21547413E-04 -6.97493553E-04

Mean frequencies of A2 in In and St= 6.09577484E-02 4.77515385E-02

Ratio of these= 1.27656090

Mean diversities at selected sites in In and St= 1.65907119E-03 2.91241845E-03

Mean diversities at neutral sites in In and St= 9.41176433E-03 2.14012526E-02

pi-n/pi-s for In and St= 0.176276326 0.136086360

Ratio of these= 1.29532695

Mean freqs. of seg. sites= 6.84257085E-03 1.20167127E-02

Ratio of these= 0.569421172

Overall delta-theta values= 0.139803886 0.140155673

Ratio of these= 0.997490048

**h = 0.5**

Zone 1: quasi-neutral zone

Upper bound scaled selection coefficient for neutrality in St metapopulation= 0.250000000

Probability of zone 1= 4.73524816E-02

Integral of selection coefficient over zone 1= 9.75669318E-05

Mean load statistics for zone 1

Mean q1 and q2= 0.600000024

F1 and F2= 0.980392158 0.955414057

Diversities= 9.41176433E-03 2.14012526E-02

Contributions to loads within In and St= 5.85401613E-05 5.85401613E-05

Contribution to load between In and St = 5.85401613E-05

Contributions to homozygous loads for In and St= 5.85401613E-05 5.85401613E-05

Contributions to inbreeding loads for In and St= 1.11098576E-04 1.11105677E-04

Contributions to selection coefficients for In and St homokaryotypes

0.00000000 0.00000000

Contributions to mean A2 freqs= 2.84114908E-02 2.84114908E-02

Contributions to mean diversities= 4.45670390E-04 1.01340248E-03

Contributions to mean freqs. of seg. site= 1.57413294E-03 3.55867785E-03

Delta-theta values= -4.44030762E-03 -1.02876425E-02

Zone 2a: moderate selection; cut-off at moderate gamma for St population

Lower and upper bounds of St popn gamma

0.250000000 82.6719589

Probability of zone 2a= 0.221977368

Coefficients for bivariate distribution of q1 and q2

a1= 0.150000006 a2= 0.349999994

b11= 0.00000000 b12= 0.00000000 b22= 0.00000000

Net probability of zone 2a using Simpsons rule= 0.221989021

Contributions to mean load statistics over zone 2a

Contributions to mean loads within In and St= 2.28338130E-03 9.12526331E-04

Contribution to load between In and St= 1.59795315E-03

Contributions to homozygous loads for In and St= 2.28338130E-03 9.12526331E-04

Contributions to inbreeding loads= 0.00000000 0.00000000

Selection coefficients for In and St homokaryotypes

6.85214996E-04 -6.85691833E-04

Selection coefficients for In and St homokaryotypes

6.85214996E-04 -6.85691833E-04

Contributions to mean A2 freqs= 3.16023305E-02 1.93026662E-02

Contributions to mean diversities= 1.11077086E-03 1.78055104E-03

Contributions to mean proportions of seg. sites= 4.58776997E-03 7.71910837E-03

Contributions to delta-theta values= 0.141036749 0.181650102

Zone 2b: moderate selection; gamma for St reaches high value

Lower and upper bounds of St popn gamma

82.6719589 826.719604

Probability of zone 2b 0.258414686

Net probability of zone 2b using Simpsons rule= 0.258414596

Contributions to mean load statistics over zone 2b

Contributions to mean loads within In and St= 1.71303938E-04 1.20617857E-04

Contribution to load between In and St= 1.45960905E-04

Contributions to homozygous loads for In and St= 1.71303938E-04 1.20617857E-04

Contributions to inbreeding loads= 0.00000000 0.00000000

Selection coefficients for In and St homokaryotypes

2.53319740E-05 -2.53915787E-05

Selection coefficients for In and St homokaryotypes

2.53319740E-05 -2.53915787E-05

Contributions to mean A2 freqs= 2.35438183E-05 1.80812658E-05

Contributions to mean diversities= 4.63024771E-05 3.58810248E-05

Contributions to mean proportions of seg. sites= 4.10601700E-04 3.42958927E-04

Contributions to delta-theta values= 0.599930704 0.628828645

Zone 3: strong selection approximation

Lower and upper bounds of St popn gamma= 826.719604 23333.3340

Zone 3: strong selection approximation

Probability of zone 3= 0.460398376

Mean load statistics over zone 3

Contributions to loads within In and St= 4.77309775E-04 4.77309775E-04

Contribution to load between In and St= 4.77309775E-04

Contributions to homozygous loads for In and St= 4.77309775E-04 4.77309775E-04

Contributions to inbreeding loads= 0.00000000 0.00000000

Selection coefficients for In and St homokaryotypes

0.00000000 0.00000000

Contributions to mean A2 freqs= 5.30657599E-06 5.30657599E-06

Contributions to mean A2 freqs at seg. sites= 0.00000000 0.00000000

Contributions to mean diversities= 1.05972968E-05 1.06062462E-05

Contributions to delta-theta values= 0.640663981 0.643299639

Mean load statistics over all zones

Loads within In and St= 2.99053523E-03 1.56899414E-03

Load between In and St= 2.27976404E-03

Homozygous load for In and St= 2.99053523E-03 1.56899414E-03

Inbreeding loads= 0.00000000 0.00000000

Selection coefficients for In and St homokaryotypes

7.10546970E-04 -7.10964203E-04

Mean frequencies of A2 in In and St= 6.00426719E-02 4.77375463E-02

Ratio of these= 1.25776625

Mean diversities at selected sites in In and St= 1.61334092E-03 2.84044072E-03

Mean diversities at neutral sites in In and St= 9.41176433E-03 2.14012526E-02

pi-n/pi-s for In and St= 0.171417475 0.132723108

Ratio of these= 1.29154205

Mean freqs. of seg. sites= 6.67713210E-03 1.17262350E-02

Ratio of these= 0.569418252

Overall delta-theta values= 0.142788589 0.140632570

Ratio of these= 1.01533091

**Inversion frequency= 0.5**

**h =0.05**

Zone 1: quasi-neutral zone

Upper bound scaled selection coefficient for neutrality in St metapopulation= 0.250000000

Probability of zone 1= 5.23817725E-02

Integral of selection coefficient over zone 1= 1.51101282E-04

Mean load statistics for zone 1

Mean q1 and q2= 0.600000024

F1 and F2= 0.967741966 0.967741966

Diversities= 1.54838562E-02 1.54838562E-02

Contributions to loads within In and St= 8.96079437E-05 8.96079437E-05

Contribution to load between In and St = 5.80228916E-05

Contributions to homozygous loads for In and St= 9.06607747E-05 9.06607747E-05

Contributions to inbreeding loads for In and St= 0.00000000 0.00000000

Contributions to selection coefficients for In and St homokaryotypes

3.15904617E-05 3.15904617E-05

Contributions to mean A2 freqs= 3.14290635E-02 3.14290635E-02

Contributions to mean diversities= 8.11071834E-04 8.11071834E-04

Contributions to mean freqs. of seg. site= 2.85658217E-03 2.85658217E-03

Delta-theta values= -7.31277466E-03 -7.31277466E-03

Zone 2a: moderate selection; cut-off at moderate gamma for St population

Lower and upper bounds of St popn gamma

0.250000000 250.000000

Probability of zone 2a= 0.360142052

Coefficients for bivariate distribution of q1 and q2

a1= 2.50000004E-02 a2= 2.50000004E-02

b11= 0.112499997 b12= 0.224999994 b22= 0.112499997

Net probability of zone 2a using Simpsons rule= 0.360543936

Contributions to mean load statistics over zone 2a

Contributions to mean loads within In and St= 2.01451243E-03 2.01450125E-03

Contribution to load between In and St= 7.07223080E-04

Contributions to homozygous loads for In and St= 3.22112162E-03 3.22111044E-03

Contributions to inbreeding loads= 1.20661210E-03 1.20661187E-03

Selection coefficients for In and St homokaryotypes

1.30641460E-03 1.30641460E-03

Selection coefficients for In and St homokaryotypes

1.30641460E-03 1.30641460E-03

Contributions to mean A2 freqs= 2.53625214E-02 2.53624413E-02

Contributions to mean diversities= 2.74223532E-03 2.74223369E-03

Contributions to mean proportions of seg. sites= 1.17043173E-02 1.17043182E-02

Contributions to delta-theta values= 0.168790698 0.168791234

Zone 2b: moderate selection; gamma for St reaches high value

Lower and upper bounds of St popn gamma

250.000000 2500.00000

Probability of zone 2b 0.352829665

Net probability of zone 2b using Simpsons rule= 0.352829695

Contributions to mean load statistics over zone 2b

Contributions to mean loads within In and St= 9.69697139E-05 9.69697139E-05

Contribution to load between In and St= 8.53871679E-05

Contributions to homozygous loads for In and St= 8.52015044E-04 8.52015160E-04

Contributions to inbreeding loads= 7.55046087E-04 7.55046087E-04

Selection coefficients for In and St homokaryotypes

1.15633011E-05 1.15633011E-05

Selection coefficients for In and St homokaryotypes

1.15633011E-05 1.15633011E-05

Contributions to mean A2 freqs= 3.18885541E-05 3.18885468E-05

Contributions to mean diversities= 6.25541797E-05 6.25541797E-05

Contributions to mean proportions of seg. sites= 5.45842166E-04 5.45841933E-04

Contributions to delta-theta values= 0.593424678 0.593424499

Zone 3: strong selection approximation

Lower and upper bounds of St popn gamma= 2500.00000 16666.6680

Zone 3: strong selection approximation

Probability of zone 3= 0.222789407

Mean load statistics over zone 3

Contributions to loads within In and St= 2.67179043E-04 2.67179043E-04

Contribution to load between In and St= 2.62204936E-04

Contributions to homozygous loads for In and St= 2.62105116E-03 2.62105116E-03

Contributions to inbreeding loads= 2.35387171E-03 2.35387171E-03

Selection coefficients for In and St homokaryotypes

4.94718552E-06 4.94718552E-06

Contributions to mean A2 freqs= 1.10538194E-05 1.10538194E-05

Contributions to mean A2 freqs at seg. sites= 0.00000000 0.00000000

Contributions to mean diversities= 2.20505681E-05 2.20505681E-05

Contributions to delta-theta values= 0.637419522 0.637419522

Mean load statistics over all zones

Loads within In and St= 2.46826909E-03 2.46825791E-03

Load between In and St= 1.11283804E-03

Homozygous load for In and St= 6.78484887E-03 6.78483769E-03

Inbreeding loads= 4.31658281E-03 4.31658234E-03

Selection coefficients for In and St homokaryotypes

1.35451555E-03 1.35451555E-03

Mean frequencies of A2 in In and St= 5.68345264E-02 5.68344481E-02

Ratio of these= 1.00000143

Mean diversities at selected sites in In and St= 3.63791175E-03 3.63791012E-03

Mean diversities at neutral sites in In and St= 1.54838562E-02 1.54838562E-02

pi-n/pi-s for In and St= 0.234948695 0.234948590

Ratio of these= 1.00000048

Mean freqs. of seg. sites= 1.53225008E-02 1.53225008E-02

Ratio of these= 1.00000000

Overall delta-theta values= 0.157685578 0.157685935

Ratio of these= 0.999997735

**h = 0.15**

Zone 1: quasi-neutral zone

Upper bound scaled selection coefficient for neutrality in St metapopulation= 0.250000000

Probability of zone 1= 5.23817725E-02

Integral of selection coefficient over zone 1= 1.51101282E-04

Mean load statistics for zone 1

Mean q1 and q2= 0.600000024

F1 and F2= 0.967741966 0.967741966

Diversities= 1.54838562E-02 1.54838562E-02

Contributions to loads within In and St= 8.98418948E-05 8.98418948E-05

Contribution to load between In and St = 6.52757517E-05

Contributions to homozygous loads for In and St= 9.06607747E-05 9.06607747E-05

Contributions to inbreeding loads for In and St= 8.99449177E-03 8.99449177E-03

Contributions to selection coefficients for In and St homokaryotypes

2.45571136E-05 2.45571136E-05

Contributions to mean A2 freqs= 3.14290635E-02 3.14290635E-02

Contributions to mean diversities= 8.11071834E-04 8.11071834E-04

Contributions to mean freqs. of seg. site= 2.85658217E-03 2.85658217E-03

Delta-theta values= -7.31277466E-03 -7.31277466E-03

Zone 2a: moderate selection; cut-off at moderate gamma for St population

Lower and upper bounds of St popn gamma

0.250000000 416.666656

Probability of zone 2a= 0.425741285

Coefficients for bivariate distribution of q1 and q2

a1= 7.50000030E-02 a2= 7.50000030E-02

b11= 8.74999985E-02 b12= 0.174999997 b22= 8.74999985E-02

Net probability of zone 2a using Simpsons rule= 0.427173704

Contributions to mean load statistics over zone 2a

Contributions to mean loads within In and St= 1.85832148E-03 1.85832102E-03

Contribution to load between In and St= 1.01018709E-03

Contributions to homozygous loads for In and St= 2.43860460E-03 2.43860413E-03

Contributions to inbreeding loads= 5.80286316E-04 5.80286316E-04

Selection coefficients for In and St homokaryotypes

8.47756863E-04 8.47756863E-04

Selection coefficients for In and St homokaryotypes

8.47756863E-04 8.47756863E-04

Contributions to mean A2 freqs= 2.47949362E-02 2.47949306E-02

Contributions to mean diversities= 2.21831887E-03 2.21831771E-03

Contributions to mean proportions of seg. sites= 9.56990849E-03 9.56990477E-03

Contributions to delta-theta values= 0.177628756 0.177628815

Zone 2b: moderate selection; gamma for St reaches high value

Lower and upper bounds of St popn gamma

416.666656 4166.66650

Probability of zone 2b 0.371462822

Net probability of zone 2b using Simpsons rule= 0.371462792

Contributions to mean load statistics over zone 2b

Contributions to mean loads within In and St= 6.21132131E-05 6.21132203E-05

Contribution to load between In and St= 6.14513629E-05

Contributions to homozygous loads for In and St= 2.04815864E-04 2.04815864E-04

Contributions to inbreeding loads= 1.42702731E-04 1.42702716E-04

Selection coefficients for In and St homokaryotypes

6.55651093E-07 6.55651093E-07

Selection coefficients for In and St homokaryotypes

6.55651093E-07 6.55651093E-07

Contributions to mean A2 freqs= 5.16085538E-06 5.16085538E-06

Contributions to mean diversities= 1.02638523E-05 1.02638551E-05

Contributions to mean proportions of seg. sites= 1.01737824E-04 1.01737191E-04

Contributions to delta-theta values= 0.642085195 0.642082810

Zone 3: strong selection approximation

Lower and upper bounds of St popn gamma= 4166.66650 16666.6680

Zone 3: strong selection approximation

Probability of zone 3= 0.138557017

Mean load statistics over zone 3

Contributions to loads within In and St= 1.84959325E-04 1.84959325E-04

Contribution to load between In and St= 1.84746983E-04

Contributions to homozygous loads for In and St= 6.15808880E-04 6.15808880E-04

Contributions to inbreeding loads= 4.30850108E-04 4.30850108E-04

Selection coefficients for In and St homokaryotypes

2.38418579E-07 2.38418579E-07

Contributions to mean A2 freqs= 1.82134681E-06 1.82134681E-06

Contributions to mean A2 freqs at seg. sites= 0.00000000 0.00000000

Contributions to mean diversities= 3.64065477E-06 3.64065477E-06

Contributions to delta-theta values= 0.643561661 0.643561661

Mean load statistics over all zones

Loads within In and St= 2.19523581E-03 2.19523534E-03

Load between In and St= 1.32166105E-03

Homozygous load for In and St= 3.34989000E-03 3.34988954E-03

Inbreeding loads= 1.15465804E-03 1.15465804E-03

Selection coefficients for In and St homokaryotypes

8.73208046E-04 8.73208046E-04

Mean frequencies of A2 in In and St= 5.62309809E-02 5.62309772E-02

Ratio of these= 1.00000012

Mean diversities at selected sites in In and St= 3.04329512E-03 3.04329395E-03

Mean diversities at neutral sites in In and St= 1.54838562E-02 1.54838562E-02

pi-n/pi-s for In and St= 0.196546331 0.196546257

Ratio of these= 1.00000036

Mean freqs. of seg. sites= 1.25644654E-02 1.25644607E-02

Ratio of these= 1.00000036

Overall delta-theta values= 0.140686154 0.140686214

Ratio of these= 0.999999583

**h = 0.25**

Zone 1: quasi-neutral zone

Upper bound scaled selection coefficient for neutrality in St metapopulation= 0.250000000

Probability of zone 1= 5.23817725E-02

Integral of selection coefficient over zone 1= 1.51101282E-04

Mean load statistics for zone 1

Mean q1 and q2= 0.600000024

F1 and F2= 0.967741966 0.967741966

Diversities= 1.54838562E-02 1.54838562E-02

Contributions to loads within In and St= 9.00758678E-05 9.00758678E-05

Contribution to load between In and St = 7.25286154E-05

Contributions to homozygous loads for In and St= 9.06607747E-05 9.06607747E-05

Contributions to inbreeding loads for In and St= 2.33285711E-03 2.33285711E-03

Contributions to selection coefficients for In and St homokaryotypes

1.75237656E-05 1.75237656E-05

Contributions to mean A2 freqs= 3.14290635E-02 3.14290635E-02

Contributions to mean diversities= 8.11071834E-04 8.11071834E-04

Contributions to mean freqs. of seg. site= 2.85658217E-03 2.85658217E-03

Delta-theta values= -7.31277466E-03 -7.31277466E-03

Zone 2a: moderate selection; cut-off at moderate gamma for St population

Lower and upper bounds of St popn gamma

0.250000000 416.666656

Probability of zone 2a= 0.425741285

Coefficients for bivariate distribution of q1 and q2

a1= 0.125000000 a2= 0.125000000

b11= 6.25000000E-02 b12= 0.125000000 b22= 6.25000000E-02

Net probability of zone 2a using Simpsons rule= 0.427173704

Contributions to mean load statistics over zone 2a

Contributions to mean loads within In and St= 1.68269861E-03 1.68269861E-03

Contribution to load between In and St= 1.18302356E-03

Contributions to homozygous loads for In and St= 1.96171436E-03 1.96171436E-03

Contributions to inbreeding loads= 2.79017113E-04 2.79017113E-04

Selection coefficients for In and St homokaryotypes

4.99546528E-04 4.99546528E-04

Selection coefficients for In and St homokaryotypes

4.99546528E-04 4.99546528E-04

Contributions to mean A2 freqs= 2.38056481E-02 2.38056462E-02

Contributions to mean diversities= 1.89988140E-03 1.89988140E-03

Contributions to mean proportions of seg. sites= 8.26252066E-03 8.26251786E-03

Contributions to delta-theta values= 0.184233844 0.184233546

Zone 2b: moderate selection; gamma for St reaches high value

Lower and upper bounds of St popn gamma

416.666656 4166.66650

Probability of zone 2b 0.371462822

Net probability of zone 2b using Simpsons rule= 0.371462792

Contributions to mean load statistics over zone 2b

Contributions to mean loads within In and St= 5.54030703E-05 5.54030812E-05

Contribution to load between In and St= 5.52319580E-05

Contributions to homozygous loads for In and St= 1.10460904E-04 1.10460889E-04

Contributions to inbreeding loads= 5.50576260E-05 5.50576224E-05

Selection coefficients for In and St homokaryotypes

1.78813934E-07 1.78813934E-07

Selection coefficients for In and St homokaryotypes

1.78813934E-07 1.78813934E-07

Contributions to mean A2 freqs= 2.87660191E-06 2.87660259E-06

Contributions to mean diversities= 5.73207399E-06 5.73207444E-06

Contributions to mean proportions of seg. sites= 5.93807235E-05 5.93807745E-05

Contributions to delta-theta values= 0.657533526 0.657533765

Zone 3: strong selection approximation

Lower and upper bounds of St popn gamma= 4166.66650 16666.6680

Zone 3: strong selection approximation

Probability of zone 3= 0.138557017

Mean load statistics over zone 3

Contributions to loads within In and St= 1.84798322E-04 1.84798322E-04

Contribution to load between In and St= 1.84743665E-04

Contributions to homozygous loads for In and St= 3.69485031E-04 3.69485031E-04

Contributions to inbreeding loads= 1.84686840E-04 1.84686840E-04

Selection coefficients for In and St homokaryotypes

5.96046448E-08 5.96046448E-08

Contributions to mean A2 freqs= 1.09280893E-06 1.09280893E-06

Contributions to mean A2 freqs at seg. sites= 0.00000000 0.00000000

Contributions to mean diversities= 2.18488071E-06 2.18488071E-06

Contributions to delta-theta values= 0.644232154 0.644232154

Mean load statistics over all zones

Loads within In and St= 2.01297598E-03 2.01297598E-03

Load between In and St= 1.49552780E-03

Homozygous load for In and St= 2.53232103E-03 2.53232103E-03

Inbreeding loads= 5.19346504E-04 5.19346504E-04

Selection coefficients for In and St homokaryotypes

5.17308712E-04 5.17308712E-04

Mean frequencies of A2 in In and St= 5.52386791E-02 5.52386753E-02

Ratio of these= 1.00000012

Mean diversities at selected sites in In and St= 2.71887030E-03 2.71887030E-03

Mean diversities at neutral sites in In and St= 1.54838562E-02 1.54838562E-02

pi-n/pi-s for In and St= 0.175593868 0.175593868

Ratio of these= 1.00000000

Mean freqs. of seg. sites= 1.12002715E-02 1.12002688E-02

Ratio of these= 1.00000024

Overall delta-theta values= 0.138784766 0.138784587

Ratio of these= 1.00000131

**h = 0.35**

Zone 1: quasi-neutral zone

Upper bound scaled selection coefficient for neutrality in St metapopulation= 0.250000000

Probability of zone 1= 5.23817725E-02

Integral of selection coefficient over zone 1= 1.51101282E-04

Mean load statistics for zone 1

Mean q1 and q2= 0.600000024

F1 and F2= 0.967741966 0.967741966

Diversities= 1.54838562E-02 1.54838562E-02

Contributions to loads within In and St= 9.03098335E-05 9.03098335E-05

Contribution to load between In and St = 7.97814791E-05

Contributions to homozygous loads for In and St= 9.06607747E-05 9.06607747E-05

Contributions to inbreeding loads for In and St= 9.99877579E-04 9.99877579E-04

Contributions to selection coefficients for In and St homokaryotypes

1.05500221E-05 1.05500221E-05

Contributions to mean A2 freqs= 3.14290635E-02 3.14290635E-02

Contributions to mean diversities= 8.11071834E-04 8.11071834E-04

Contributions to mean freqs. of seg. site= 2.85658217E-03 2.85658217E-03

Delta-theta values= -7.31277466E-03 -7.31277466E-03

Zone 2a: moderate selection; cut-off at moderate gamma for St population

Lower and upper bounds of St popn gamma

0.250000000 297.619049

Probability of zone 2a= 0.381587625

Coefficients for bivariate distribution of q1 and q2

a1= 0.174999997 a2= 0.174999997

b11= 3.75000015E-02 b12= 7.50000030E-02 b22= 3.75000015E-02

Net probability of zone 2a using Simpsons rule= 0.382221013

Contributions to mean load statistics over zone 2a

Contributions to mean loads within In and St= 1.53240829E-03 1.53240829E-03

Contribution to load between In and St= 1.28287345E-03

Contributions to homozygous loads for In and St= 1.65060873E-03 1.65060873E-03

Contributions to inbreeding loads= 1.18198695E-04 1.18198695E-04

Selection coefficients for In and St homokaryotypes

2.49505043E-04 2.49505043E-04

Selection coefficients for In and St homokaryotypes

2.49505043E-04 2.49505043E-04

Contributions to mean A2 freqs= 2.25718636E-02 2.25718636E-02

Contributions to mean diversities= 1.67502987E-03 1.67502975E-03

Contributions to mean proportions of seg. sites= 7.32368557E-03 7.32368976E-03

Contributions to delta-theta values= 0.188582063 0.188582599

Zone 2b: moderate selection; gamma for St reaches high value

Lower and upper bounds of St popn gamma

297.619049 2976.19043

Probability of zone 2b 0.360976130

Net probability of zone 2b using Simpsons rule= 0.360976666

Contributions to mean load statistics over zone 2b

Contributions to mean loads within In and St= 6.68565262E-05 6.68565553E-05

Contribution to load between In and St= 6.67696513E-05

Contributions to homozygous loads for In and St= 9.53838971E-05 9.53839117E-05

Contributions to inbreeding loads= 2.85273291E-05 2.85273291E-05

Selection coefficients for In and St homokaryotypes

5.96046448E-08 5.96046448E-08

Selection coefficients for In and St homokaryotypes

5.96046448E-08 5.96046448E-08

Contributions to mean A2 freqs= 3.38645555E-06 3.38645532E-06

Contributions to mean diversities= 6.74827379E-06 6.74827425E-06

Contributions to mean proportions of seg. sites= 7.00507444E-05 7.00509772E-05

Contributions to delta-theta values= 0.658231735 0.658232868

Zone 3: strong selection approximation

Lower and upper bounds of St popn gamma= 2976.19043 16666.6680

Zone 3: strong selection approximation

Probability of zone 3= 0.193197370

Mean load statistics over zone 3

Contributions to loads within In and St= 2.35223953E-04 2.35223953E-04

Contribution to load between In and St= 2.35196945E-04

Contributions to homozygous loads for In and St= 3.35994788E-04 3.35994788E-04

Contributions to inbreeding loads= 1.00771031E-04 1.00771031E-04

Selection coefficients for In and St homokaryotypes

0.00000000 0.00000000

Contributions to mean A2 freqs= 1.25611268E-06 1.25611268E-06

Contributions to mean A2 freqs at seg. sites= 0.00000000 0.00000000

Contributions to mean diversities= 2.51142410E-06 2.51142410E-06

Contributions to delta-theta values= 0.644280791 0.644280791

Mean load statistics over all zones

Loads within In and St= 1.92479859E-03 1.92479859E-03

Load between In and St= 1.66462152E-03

Homozygous load for In and St= 2.17264821E-03 2.17264821E-03

Inbreeding loads= 2.47847987E-04 2.47847987E-04

Selection coefficients for In and St homokaryotypes

2.60114670E-04 2.60114670E-04

Mean frequencies of A2 in In and St= 5.40055707E-02 5.40055707E-02

Ratio of these= 1.00000000

Mean diversities at selected sites in In and St= 2.49536149E-03 2.49536126E-03

Mean diversities at neutral sites in In and St= 1.54838562E-02 1.54838562E-02

pi-n/pi-s for In and St= 0.161158919 0.161158904

Ratio of these= 1.00000012

Mean freqs. of seg. sites= 1.02753658E-02 1.02753714E-02

Ratio of these= 0.999999464

Overall delta-theta values= 0.138435245 0.138435781

Ratio of these= 0.999996126

**h = 0.45**

Zone 1: quasi-neutral zone

Upper bound scaled selection coefficient for neutrality in St metapopulation= 0.250000000

Probability of zone 1= 5.23817725E-02

Integral of selection coefficient over zone 1= 1.51101282E-04

Mean load statistics for zone 1

Mean q1 and q2= 0.600000024

F1 and F2= 0.967741966 0.967741966

Diversities= 1.54838562E-02 1.54838562E-02

Contributions to loads within In and St= 9.05437919E-05 9.05437919E-05

Contribution to load between In and St = 8.70343356E-05

Contributions to homozygous loads for In and St= 9.06607747E-05 9.06607747E-05

Contributions to inbreeding loads for In and St= 4.28534026E-04 4.28534026E-04

Contributions to selection coefficients for In and St homokaryotypes

3.51667404E-06 3.51667404E-06

Contributions to mean A2 freqs= 3.14290635E-02 3.14290635E-02

Contributions to mean diversities= 8.11071834E-04 8.11071834E-04

Contributions to mean freqs. of seg. site= 2.85658217E-03 2.85658217E-03

Delta-theta values= -7.31277466E-03 -7.31277466E-03

Zone 2a: moderate selection; cut-off at moderate gamma for St population

Lower and upper bounds of St popn gamma

0.250000000 165.343918

Probability of zone 2a= 0.313080877

Coefficients for bivariate distribution of q1 and q2

a1= 0.224999994 a2= 0.224999994

b11= 1.25000030E-02 b12= 2.50000060E-02 b22= 1.25000030E-02

Net probability of zone 2a using Simpsons rule= 0.313205212

Contributions to mean load statistics over zone 2a

Contributions to mean loads within In and St= 1.38971407E-03 1.38971396E-03

Contribution to load between In and St= 1.31849293E-03

Contributions to homozygous loads for In and St= 1.41730066E-03 1.41730066E-03

Contributions to inbreeding loads= 2.75873990E-05 2.75873990E-05

Selection coefficients for In and St homokaryotypes

7.12275505E-05 7.12275505E-05

Selection coefficients for In and St homokaryotypes

7.12275505E-05 7.12275505E-05

Contributions to mean A2 freqs= 2.15853471E-02 2.15853471E-02

Contributions to mean diversities= 1.50628283E-03 1.50628260E-03

Contributions to mean proportions of seg. sites= 6.59325579E-03 6.59325346E-03

Contributions to delta-theta values= 0.189490020 0.189489901

Zone 2b: moderate selection; gamma for St reaches high value

Lower and upper bounds of St popn gamma

165.343918 1653.43921

Probability of zone 2b 0.328705907

Net probability of zone 2b using Simpsons rule= 0.328705937

Contributions to mean load statistics over zone 2b

Contributions to mean loads within In and St= 9.41944963E-05 9.41945182E-05

Contribution to load between In and St= 9.41547623E-05

Contributions to homozygous loads for In and St= 1.04615778E-04 1.04615778E-04

Contributions to inbreeding loads= 1.04213213E-05 1.04213213E-05

Selection coefficients for In and St homokaryotypes

5.96046448E-08 5.96046448E-08

Selection coefficients for In and St homokaryotypes

5.96046448E-08 5.96046448E-08

Contributions to mean A2 freqs= 6.21098661E-06 6.21098707E-06

Contributions to mean diversities= 1.23632599E-05 1.23632572E-05

Contributions to mean proportions of seg. sites= 1.24556624E-04 1.24556012E-04

Contributions to delta-theta values= 0.647857904 0.647856236

Zone 3: strong selection approximation

Lower and upper bounds of St popn gamma= 1653.43921 16666.6680

Zone 3: strong selection approximation

Probability of zone 3= 0.293974340

Mean load statistics over zone 3

Contributions to loads within In and St= 3.26361653E-04 3.26361653E-04

Contribution to load between In and St= 3.26350360E-04

Contributions to homozygous loads for In and St= 3.62611463E-04 3.62611463E-04

Contributions to inbreeding loads= 3.62496357E-05 3.62496357E-05

Selection coefficients for In and St homokaryotypes

0.00000000 0.00000000

Contributions to mean A2 freqs= 2.02892329E-06 2.02892329E-06

Contributions to mean A2 freqs at seg. sites= 0.00000000 0.00000000

Contributions to mean diversities= 4.05619130E-06 4.05619130E-06

Contributions to delta-theta values= 0.644017696 0.644017696

Mean load statistics over all zones

Loads within In and St= 1.90081401E-03 1.90081389E-03

Load between In and St= 1.82603230E-03

Homozygous load for In and St= 1.97518873E-03 1.97518873E-03

Inbreeding loads= 7.43753408E-05 7.43753408E-05

Selection coefficients for In and St homokaryotypes

7.48038292E-05 7.48038292E-05

Mean frequencies of A2 in In and St= 5.30226529E-02 5.30226529E-02

Ratio of these= 1.00000000

Mean diversities at selected sites in In and St= 2.33377400E-03 2.33377400E-03

Mean diversities at neutral sites in In and St= 1.54838562E-02 1.54838562E-02

pi-n/pi-s for In and St= 0.150723055 0.150723055

Ratio of these= 1.00000000

Mean freqs. of seg. sites= 9.61481873E-03 9.61481594E-03

Ratio of these= 1.00000024

Overall delta-theta values= 0.138868570 0.138868332

Ratio of these= 1.00000167

**h = 0.5**

Zone 1: quasi-neutral zone

Upper bound scaled selection coefficient for neutrality in St metapopulation= 0.250000000

Probability of zone 1= 5.23817725E-02

Integral of selection coefficient over zone 1= 1.51101282E-04

Mean load statistics for zone 1

Mean q1 and q2= 0.600000024

F1 and F2= 0.967741966 0.967741966

Diversities= 1.54838562E-02 1.54838562E-02

Contributions to loads within In and St= 9.06607747E-05 9.06607747E-05

Contribution to load between In and St = 9.06607747E-05

Contributions to homozygous loads for In and St= 9.06607747E-05 9.06607747E-05

Contributions to inbreeding loads for In and St= 1.11103596E-04 1.11103596E-04

Contributions to selection coefficients for In and St homokaryotypes

0.00000000 0.00000000

Contributions to mean A2 freqs= 3.14290635E-02 3.14290635E-02

Contributions to mean diversities= 8.11071834E-04 8.11071834E-04

Contributions to mean freqs. of seg. site= 2.85658217E-03 2.85658217E-03

Delta-theta values= -7.31277466E-03 -7.31277466E-03

Zone 2a: moderate selection; cut-off at moderate gamma for St population

Lower and upper bounds of St popn gamma

0.250000000 82.6719589

Probability of zone 2a= 0.245311543

Coefficients for bivariate distribution of q1 and q2

a1= 0.250000000 a2= 0.250000000

b11= 0.00000000 b12= 0.00000000 b22= 0.00000000

Net probability of zone 2a using Simpsons rule= 0.245324686

Contributions to mean load statistics over zone 2a

Contributions to mean loads within In and St= 1.31947640E-03 1.31947652E-03

Contribution to load between In and St= 1.31947640E-03

Contributions to homozygous loads for In and St= 1.31947640E-03 1.31947652E-03

Contributions to inbreeding loads= 0.00000000 0.00000000

Selection coefficients for In and St homokaryotypes

0.00000000 0.00000000

Selection coefficients for In and St homokaryotypes

0.00000000 0.00000000

Contributions to mean A2 freqs= 2.16231793E-02 2.16231793E-02

Contributions to mean diversities= 1.42608013E-03 1.42608024E-03

Contributions to mean proportions of seg. sites= 6.17675530E-03 6.17675530E-03

Contributions to delta-theta values= 0.180903077 0.180903018

Zone 2b: moderate selection; gamma for St reaches high value

Lower and upper bounds of St popn gamma

82.6719589 826.719604

Probability of zone 2b 0.281515658

Net probability of zone 2b using Simpsons rule= 0.281515867

Contributions to mean load statistics over zone 2b

Contributions to mean loads within In and St= 1.27681225E-04 1.27681225E-04

Contribution to load between In and St= 1.27681225E-04

Contributions to homozygous loads for In and St= 1.27681225E-04 1.27681225E-04

Contributions to inbreeding loads= 0.00000000 0.00000000

Selection coefficients for In and St homokaryotypes

0.00000000 0.00000000

Selection coefficients for In and St homokaryotypes

0.00000000 0.00000000

Contributions to mean A2 freqs= 1.37607740E-05 1.37607713E-05

Contributions to mean diversities= 2.73080968E-05 2.73080950E-05

Contributions to mean proportions of seg. sites= 2.61915993E-04 2.61915877E-04

Contributions to delta-theta values= 0.630102694 0.630102515

Zone 3: strong selection approximation

Lower and upper bounds of St popn gamma= 826.719604 16666.6680

Zone 3: strong selection approximation

Probability of zone 3= 0.408933938

Mean load statistics over zone 3

Contributions to loads within In and St= 4.30277199E-04 4.30277199E-04

Contribution to load between In and St= 4.30277287E-04

Contributions to homozygous loads for In and St= 4.30277199E-04 4.30277199E-04

Contributions to inbreeding loads= 0.00000000 0.00000000

Selection coefficients for In and St homokaryotypes

0.00000000 0.00000000

Contributions to mean A2 freqs= 3.83122233E-06 3.83122233E-06

Contributions to mean A2 freqs at seg. sites= 0.00000000 0.00000000

Contributions to mean diversities= 7.65730329E-06 7.65730329E-06

Contributions to delta-theta values= 0.643227220 0.643227220

Mean load statistics over all zones

Loads within In and St= 1.96809554E-03 1.96809554E-03

Load between In and St= 1.96809554E-03

Homozygous load for In and St= 1.96809554E-03 1.96809554E-03

Inbreeding loads= 0.00000000 0.00000000

Selection coefficients for In and St homokaryotypes

0.00000000 0.00000000

Mean frequencies of A2 in In and St= 5.30698337E-02 5.30698337E-02

Ratio of these= 1.00000000

Mean diversities at selected sites in In and St= 2.27211718E-03 2.27211742E-03

Mean diversities at neutral sites in In and St= 1.54838562E-02 1.54838562E-02

pi-n/pi-s for In and St= 0.146741048 0.146741062

Ratio of these= 0.999999881

Mean freqs. of seg. sites= 9.37139709E-03 9.37139709E-03

Ratio of these= 1.00000000

Overall delta-theta values= 0.139842212 0.139842153

Ratio of these= 1.00000048

**Section 2**

**Population size = 500000**

**Mean scaled selection coefficient for whole popn= 4000**

**Inversion frequency= 0.1**

**h = 0.05**

Zone 1: quasi-neutral zone

Upper bound scaled selection coefficient for neutrality in St metapopulation= 0.250000000

Probability of zone 1= 6.65597618E-02

Integral of selection coefficient over zone 1= 4.26665181E-04

Mean load statistics for zone 1

Mean q1 and q2= 0.600000024

F1 and F2= 0.998336136 0.985221684

Diversities= 7.98654510E-04 7.09359162E-03

Contributions to loads within In and St= 2.55845807E-04 2.54637154E-04

Contribution to load between In and St = 1.63839431E-04

Contributions to homozygous loads for In and St= 2.55999126E-04 2.55999126E-04

Contributions to inbreeding loads for In and St= 0.00000000 0.00000000

Contributions to selection coefficients for In and St homokaryotypes

9.20295715E-05 9.07778740E-05

Contributions to mean A2 freqs= 3.99358571E-02 3.99358571E-02

Contributions to mean diversities= 5.31582555E-05 4.72147774E-04

Contributions to mean freqs. of seg. site= 1.88227175E-04 1.66928873E-03

Delta-theta values= -1.93631649E-03 -3.45575809E-03

Zone 2a: moderate selection; cut-off at moderate gamma for St population

Lower and upper bounds of St popn gamma

0.250000000 250.000000

Probability of zone 2a= 0.452220768

Coefficients for bivariate distribution of q1 and q2

a1= 5.00000035E-03 a2= 4.49999981E-02

b11= 4.50000027E-03 b12= 8.09999928E-02 b22= 0.364499956

Net probability of zone 2a using Simpsons rule= 0.452730894

Contributions to mean load statistics over zone 2a

Contributions to mean loads within In and St= 0.433099866 2.99232360E-03

Contribution to load between In and St= 2.37799361E-02

Contributions to homozygous loads for In and St= 0.434141189 4.35505714E-03

Contributions to inbreeding loads= 1.04130467E-03 1.36273575E-03

Selection coefficients for In and St homokaryotypes

0.335898280 -2.10051537E-02

Selection coefficients for In and St homokaryotypes

0.335898280 -2.10051537E-02

Contributions to mean A2 freqs= 0.159932017 2.54321266E-02

Contributions to mean diversities= 3.59690865E-04 1.37845811E-03

Contributions to mean proportions of seg. sites= 1.29632582E-03 6.18571742E-03

Contributions to delta-theta values= 1.56104565E-02 0.209402859

Zone 2b: moderate selection; gamma for St reaches high value

Lower and upper bounds of St popn gamma

250.000000 2500.00000

Probability of zone 2b 0.373098969

Net probability of zone 2b using Simpsons rule= 0.373098999

Contributions to mean load statistics over zone 2b

Contributions to mean loads within In and St= 6.64582057E-03 1.95894958E-04

Contribution to load between In and St= 5.51719102E-04

Contributions to homozygous loads for In and St= 9.24149808E-03 1.75593141E-03

Contributions to inbreeding loads= 2.59567029E-03 1.56003609E-03

Selection coefficients for In and St homokaryotypes

6.07556105E-03 -3.55839729E-04

Selection coefficients for In and St homokaryotypes

6.07556105E-03 -3.55839729E-04

Contributions to mean A2 freqs= 2.30898309E-04 2.76227602E-05

Contributions to mean diversities= 7.93229847E-05 5.43561146E-05

Contributions to mean proportions of seg. sites= 4.34924499E-04 4.84443386E-04

Contributions to delta-theta values= 0.352951348 0.601932108

Zone 3: strong selection approximation

Lower and upper bounds of St popn gamma= 2500.00000 7500.00049

Zone 3: strong selection approximation

Probability of zone 3= 9.62634087E-02

Mean load statistics over zone 3

Contributions to loads within In and St= 1.75864421E-04 1.47919534E-04

Contribution to load between In and St= 1.44426347E-04

Contributions to homozygous loads for In and St= 1.44395151E-03 1.44395139E-03

Contributions to inbreeding loads= 1.26808544E-03 1.29603059E-03

Selection coefficients for In and St homokaryotypes

3.14116478E-05 3.51667404E-06

Contributions to mean A2 freqs= 3.49309471E-06 3.49309425E-06

Contributions to mean A2 freqs at seg. sites= 0.00000000 0.00000000

Contributions to mean diversities= 6.80390031E-06 6.96577126E-06

Contributions to delta-theta values= 0.540587068 0.636242390

Mean load statistics over all zones

Loads within In and St= 0.440177411 3.59077519E-03

Load between In and St= 2.46399213E-02

Homozygous load for In and St= 0.445082635 7.81093910E-03

Inbreeding loads= 4.90521360E-03 4.22016438E-03

Selection coefficients for In and St homokaryotypes

0.340014517 -2.12721825E-02

Mean frequencies of A2 in In and St= 0.200102255 6.53990954E-02

Ratio of these= 3.05970979

Mean diversities at selected sites in In and St= 4.98975976E-04 1.91192783E-03

Mean diversities at neutral sites in In and St= 7.98654510E-04 7.09359162E-03

pi-n/pi-s for In and St= 0.624770761 0.269528896

Ratio of these= 2.31801033

Mean freqs. of seg. sites= 1.97201944E-03 8.40738695E-03

Ratio of these= 0.234557942

Overall delta-theta values= 0.102322757 0.193206847

Ratio of these= 0.529602110

**h = 0.15**

Zone 1: quasi-neutral zone

Upper bound scaled selection coefficient for neutrality in St metapopulation= 0.250000000

Probability of zone 1= 6.65597618E-02

Integral of selection coefficient over zone 1= 4.26665181E-04

Mean load statistics for zone 1

Mean q1 and q2= 0.600000024

F1 and F2= 0.998336136 0.985221684

Diversities= 7.98654510E-04 7.09359162E-03

Contributions to loads within In and St= 2.55879859E-04 2.54939805E-04

Contribution to load between In and St = 1.84319360E-04

Contributions to homozygous loads for In and St= 2.55999126E-04 2.55999126E-04

Contributions to inbreeding loads for In and St= 8.89189169E-03 8.98789149E-03

Contributions to selection coefficients for In and St homokaryotypes

7.15851784E-05 7.06315041E-05

Contributions to mean A2 freqs= 3.99358571E-02 3.99358571E-02

Contributions to mean diversities= 5.31582555E-05 4.72147774E-04

Contributions to mean freqs. of seg. site= 1.88227175E-04 1.66928873E-03

Delta-theta values= -1.93631649E-03 -3.45575809E-03

Zone 2a: moderate selection; cut-off at moderate gamma for St population

Lower and upper bounds of St popn gamma

0.250000000 416.666656

Probability of zone 2a= 0.530714035

Coefficients for bivariate distribution of q1 and q2

a1= 1.50000006E-02 a2= 0.135000005

b11= 3.50000011E-03 b12= 6.30000010E-02 b22= 0.283499986

Net probability of zone 2a using Simpsons rule= 0.532534182

Contributions to mean load statistics over zone 2a

Contributions to mean loads within In and St= 0.187499821 3.13930842E-03

Contribution to load between In and St= 3.01044788E-02

Contributions to homozygous loads for In and St= 0.188178822 3.92148830E-03

Contributions to inbreeding loads= 6.79010118E-04 7.82183779E-04

Selection coefficients for In and St homokaryotypes

0.145633757 -2.73320675E-02

Selection coefficients for In and St homokaryotypes

0.145633757 -2.73320675E-02

Contributions to mean A2 freqs= 0.125489846 2.61938646E-02

Contributions to mean diversities= 3.16677120E-04 1.19927083E-03

Contributions to mean proportions of seg. sites= 1.22639048E-03 5.34233497E-03

Contributions to delta-theta values= 8.39067698E-02 0.203587770

Zone 2b: moderate selection; gamma for St reaches high value

Lower and upper bounds of St popn gamma

416.666656 4166.66650

Probability of zone 2b 0.353823960

Net probability of zone 2b using Simpsons rule= 0.353824049

Contributions to mean load statistics over zone 2b

Contributions to mean loads within In and St= 3.31623130E-04 1.43604135E-04

Contribution to load between In and St= 2.25272437E-04

Contributions to homozygous loads for In and St= 1.02791737E-03 4.73745749E-04

Contributions to inbreeding loads= 6.96293777E-04 3.30142240E-04

Selection coefficients for In and St homokaryotypes

1.06334686E-04 -8.16583633E-05

Selection coefficients for In and St homokaryotypes

1.06334686E-04 -8.16583633E-05

Contributions to mean A2 freqs= 9.73950227E-06 5.25507176E-06

Contributions to mean diversities= 1.86478919E-05 1.04538258E-05

Contributions to mean proportions of seg. sites= 1.44226084E-04 1.04103769E-04

Contributions to delta-theta values= 0.541290581 0.643745303

Zone 3: strong selection approximation

Lower and upper bounds of St popn gamma= 4166.66650 7500.00049

Zone 3: strong selection approximation

Probability of zone 3= 3.70451212E-02

Mean load statistics over zone 3

Contributions to loads within In and St= 8.44577808E-05 8.34753519E-05

Contribution to load between In and St= 8.33526356E-05

Contributions to homozygous loads for In and St= 2.77838350E-04 2.77838350E-04

Contributions to inbreeding loads= 1.93380693E-04 1.94362889E-04

Selection coefficients for In and St homokaryotypes

1.13248825E-06 1.19209290E-07

Contributions to mean A2 freqs= 4.73628376E-07 4.73628376E-07

Contributions to mean A2 freqs at seg. sites= 0.00000000 0.00000000

Contributions to mean diversities= 9.41729922E-07 9.46636931E-07

Contributions to delta-theta values= 0.626614988 0.643260598

Mean load statistics over all zones

Loads within In and St= 0.188171789 3.62132769E-03

Load between In and St= 3.05974241E-02

Homozygous load for In and St= 0.189740568 4.92907129E-03

Inbreeding loads= 1.56880380E-03 1.30774826E-03

Selection coefficients for In and St homokaryotypes

0.145786703 -2.73432732E-02

Mean frequencies of A2 in In and St= 0.165435925 6.61354512E-02

Ratio of these= 2.50147128

Mean diversities at selected sites in In and St= 3.89425026E-04 1.68281910E-03

Mean diversities at neutral sites in In and St= 7.98654510E-04 7.09359162E-03

pi-n/pi-s for In and St= 0.487601370 0.237230897

Ratio of these= 2.05538726

Mean freqs. of seg. sites= 1.56779168E-03 7.12514156E-03

Ratio of these= 0.220036566

Overall delta-theta values= 0.118774116 0.162093222

Ratio of these= 0.732751906

**h = 0.25**

Zone 1: quasi-neutral zone

Upper bound scaled selection coefficient for neutrality in St metapopulation= 0.250000000

Probability of zone 1= 6.65597618E-02

Integral of selection coefficient over zone 1= 4.26665181E-04

Mean load statistics for zone 1

Mean q1 and q2= 0.600000024

F1 and F2= 0.998336136 0.985221684

Diversities= 7.98654510E-04 7.09359162E-03

Contributions to loads within In and St= 2.55913910E-04 2.55242456E-04

Contribution to load between In and St = 2.04799289E-04

Contributions to homozygous loads for In and St= 2.55999126E-04 2.55999126E-04

Contributions to inbreeding loads for In and St= 2.32399069E-03 2.33228668E-03

Contributions to selection coefficients for In and St homokaryotypes

5.11407852E-05 5.04255295E-05

Contributions to mean A2 freqs= 3.99358571E-02 3.99358571E-02

Contributions to mean diversities= 5.31582555E-05 4.72147774E-04

Contributions to mean freqs. of seg. site= 1.88227175E-04 1.66928873E-03

Delta-theta values= -1.93631649E-03 -3.45575809E-03

Zone 2a: moderate selection; cut-off at moderate gamma for St population

Lower and upper bounds of St popn gamma

0.250000000 416.666656

Probability of zone 2a= 0.530714035

Coefficients for bivariate distribution of q1 and q2

a1= 2.50000004E-02 a2= 0.224999994

b11= 2.50000018E-03 b12= 4.49999981E-02 b22= 0.202499986

Net probability of zone 2a using Simpsons rule= 0.532534182

Contributions to mean load statistics over zone 2a

Contributions to mean loads within In and St= 0.112640530 3.18144378E-03

Contribution to load between In and St= 3.00094951E-02

Contributions to homozygous loads for In and St= 0.112978466 3.56903463E-03

Contributions to inbreeding loads= 3.37971636E-04 3.87589796E-04

Selection coefficients for In and St homokaryotypes

7.93092251E-02 -2.71911621E-02

Selection coefficients for In and St homokaryotypes

7.93092251E-02 -2.71911621E-02

Contributions to mean A2 freqs= 0.107461803 2.61754002E-02

Contributions to mean diversities= 2.76602834E-04 1.06084312E-03

Contributions to mean proportions of seg. sites= 1.09801616E-03 4.68392344E-03

Contributions to delta-theta values= 0.106283784 0.196486592

Zone 2b: moderate selection; gamma for St reaches high value

Lower and upper bounds of St popn gamma

416.666656 4166.66650

Probability of zone 2b 0.353823960

Net probability of zone 2b using Simpsons rule= 0.353824049

Contributions to mean load statistics over zone 2b

Contributions to mean loads within In and St= 3.09545547E-04 1.29415464E-04

Contribution to load between In and St= 2.16288943E-04

Contributions to homozygous loads for In and St= 6.07085123E-04 2.58048822E-04

Contributions to inbreeding loads= 2.97539023E-04 1.28633590E-04

Selection coefficients for In and St homokaryotypes

9.32812691E-05 -8.69035721E-05

Selection coefficients for In and St homokaryotypes

9.32812691E-05 -8.69035721E-05

Contributions to mean A2 freqs= 5.76968705E-06 2.95680184E-06

Contributions to mean diversities= 1.12389798E-05 5.89254660E-06

Contributions to mean proportions of seg. sites= 9.47084045E-05 6.14295714E-05

Contributions to delta-theta values= 0.578992248 0.659687996

Zone 3: strong selection approximation

Lower and upper bounds of St popn gamma= 4166.66650 7500.00049

Zone 3: strong selection approximation

Probability of zone 3= 3.70451212E-02

Mean load statistics over zone 3

Contributions to loads within In and St= 8.36360196E-05 8.33834274E-05

Contribution to load between In and St= 8.33518279E-05

Contributions to homozygous loads for In and St= 1.66703088E-04 1.66703074E-04

Contributions to inbreeding loads= 8.30671052E-05 8.33196755E-05

Selection coefficients for In and St homokaryotypes

2.98023224E-07 5.96046448E-08

Contributions to mean A2 freqs= 2.84176849E-07 2.84176821E-07

Contributions to mean A2 freqs at seg. sites= 0.00000000 0.00000000

Contributions to mean diversities= 5.66364349E-07 5.68131497E-07

Contributions to delta-theta values= 0.634318113 0.644048452

Mean load statistics over all zones

Loads within In and St= 0.113289617 3.64948506E-03

Load between In and St= 3.05139348E-02

Homozygous load for In and St= 0.114008263 4.24978556E-03

Inbreeding loads= 7.18662981E-04 6.00299682E-04

Selection coefficients for In and St homokaryotypes

7.94423819E-02 -2.72285938E-02

Mean frequencies of A2 in In and St= 0.147403717 6.61145002E-02

Ratio of these= 2.22952175

Mean diversities at selected sites in In and St= 3.41566425E-04 1.53945165E-03

Mean diversities at neutral sites in In and St= 7.98654510E-04 7.09359162E-03

pi-n/pi-s for In and St= 0.427677333 0.217020050

Ratio of these= 1.97068119

Mean freqs. of seg. sites= 1.38644641E-03 6.42030407E-03

Ratio of these= 0.215947151

Overall delta-theta values= 0.125975013 0.149327815

Ratio of these= 0.843613863

**h = 0.35**

Zone 1: quasi-neutral zone

Upper bound scaled selection coefficient for neutrality in St metapopulation= 0.250000000

Probability of zone 1= 6.65597618E-02

Integral of selection coefficient over zone 1= 4.26665181E-04

Mean load statistics for zone 1

Mean q1 and q2= 0.600000024

F1 and F2= 0.998336136 0.985221684

Diversities= 7.98654510E-04 7.09359162E-03

Contributions to loads within In and St= 2.55947991E-04 2.55545106E-04

Contribution to load between In and St = 2.25279218E-04

Contributions to homozygous loads for In and St= 2.55999126E-04 2.55999126E-04

Contributions to inbreeding loads for In and St= 9.97597585E-04 9.99730779E-04

Contributions to selection coefficients for In and St homokaryotypes

3.06963921E-05 3.02791595E-05

Contributions to mean A2 freqs= 3.99358571E-02 3.99358571E-02

Contributions to mean diversities= 5.31582555E-05 4.72147774E-04

Contributions to mean freqs. of seg. site= 1.88227175E-04 1.66928873E-03

Delta-theta values= -1.93631649E-03 -3.45575809E-03

Zone 2a: moderate selection; cut-off at moderate gamma for St population

Lower and upper bounds of St popn gamma

0.250000000 297.619049

Probability of zone 2a= 0.478139609

Coefficients for bivariate distribution of q1 and q2

a1= 3.50000001E-02 a2= 0.314999998

b11= 1.50000013E-03 b12= 2.70000007E-02 b22= 0.121499993

Net probability of zone 2a using Simpsons rule= 0.478944182

Contributions to mean load statistics over zone 2a

Contributions to mean loads within In and St= 7.81883150E-02 3.17456503E-03

Contribution to load between In and St= 2.90885214E-02

Contributions to homozygous loads for In and St= 7.83228576E-02 3.33624240E-03

Contributions to inbreeding loads= 1.34582835E-04 1.61680815E-04

Selection coefficients for In and St homokaryotypes

4.79139090E-02 -2.62526274E-02

Selection coefficients for In and St homokaryotypes

4.79139090E-02 -2.62526274E-02

Contributions to mean A2 freqs= 9.52646732E-02 2.55477820E-02

Contributions to mean diversities= 2.45808187E-04 9.47850756E-04

Contributions to mean proportions of seg. sites= 9.77696734E-04 4.16303007E-03

Contributions to delta-theta values= 0.108043015 0.192240298

Zone 2b: moderate selection; gamma for St reaches high value

Lower and upper bounds of St popn gamma

297.619049 2976.19043

Probability of zone 2b 0.369981229

Net probability of zone 2b using Simpsons rule= 0.369981259

Contributions to mean load statistics over zone 2b

Contributions to mean loads within In and St= 3.30357696E-04 1.50549633E-04

Contribution to load between In and St= 2.39036613E-04

Contributions to homozygous loads for In and St= 4.68152575E-04 2.14801388E-04

Contributions to inbreeding loads= 1.37795185E-04 6.42516170E-05

Selection coefficients for In and St homokaryotypes

9.13143158E-05 -8.84532928E-05

Selection coefficients for In and St homokaryotypes

9.13143158E-05 -8.84532928E-05

Contributions to mean A2 freqs= 5.90538775E-06 3.28604187E-06

Contributions to mean diversities= 1.15098837E-05 6.54904397E-06

Contributions to mean proportions of seg. sites= 9.73745773E-05 6.86453059E-05

Contributions to delta-theta values= 0.580649555 0.661531091

Zone 3: strong selection approximation

Lower and upper bounds of St popn gamma= 2976.19043 7500.00049

Zone 3: strong selection approximation

Probability of zone 3= 7.34623075E-02

Mean load statistics over zone 3

Contributions to loads within In and St= 1.21952624E-04 1.21810503E-04

Contribution to load between In and St= 1.21792786E-04

Contributions to homozygous loads for In and St= 1.73989552E-04 1.73989523E-04

Contributions to inbreeding loads= 5.20368958E-05 5.21789188E-05

Selection coefficients for In and St homokaryotypes

1.78813934E-07 0.00000000

Contributions to mean A2 freqs= 3.72860882E-07 3.72860910E-07

Contributions to mean A2 freqs at seg. sites= 0.00000000 0.00000000

Contributions to mean diversities= 7.43305691E-07 7.45451416E-07

Contributions to delta-theta values= 0.635145664 0.644146800

Mean load statistics over all zones

Loads within In and St= 7.88965747E-02 3.70247033E-03

Load between In and St= 2.96746306E-02

Homozygous load for In and St= 7.92209953E-02 3.98103241E-03

Inbreeding loads= 3.24466033E-04 2.78565363E-04

Selection coefficients for In and St homokaryotypes

4.80301976E-02 -2.63123512E-02

Mean frequencies of A2 in In and St= 0.135206804 6.54872954E-02

Ratio of these= 2.06462646

Mean diversities at selected sites in In and St= 3.11219657E-04 1.42729294E-03

Mean diversities at neutral sites in In and St= 7.98654510E-04 7.09359162E-03

pi-n/pi-s for In and St= 0.389679968 0.201208785

Ratio of these= 1.93669462

Mean freqs. of seg. sites= 1.27052609E-03 5.90839610E-03

Ratio of these= 0.215037391

Overall delta-theta values= 0.130969167 0.142971516

Ratio of these= 0.916050792

**h = 0.45**

Zone 1: quasi-neutral zone

Upper bound scaled selection coefficient for neutrality in St metapopulation= 0.250000000

Probability of zone 1= 6.65597618E-02

Integral of selection coefficient over zone 1= 4.26665181E-04

Mean load statistics for zone 1

Mean q1 and q2= 0.600000024

F1 and F2= 0.998336136 0.985221684

Diversities= 7.98654510E-04 7.09359162E-03

Contributions to loads within In and St= 2.55982071E-04 2.55847786E-04

Contribution to load between In and St = 2.45759147E-04

Contributions to homozygous loads for In and St= 2.55999126E-04 2.55999126E-04

Contributions to inbreeding loads for In and St= 4.27835999E-04 4.28489118E-04

Contributions to selection coefficients for In and St homokaryotypes

1.02519989E-05 1.00731850E-05

Contributions to mean A2 freqs= 3.99358571E-02 3.99358571E-02

Contributions to mean diversities= 5.31582555E-05 4.72147774E-04

Contributions to mean freqs. of seg. site= 1.88227175E-04 1.66928873E-03

Delta-theta values= -1.93631649E-03 -3.45575809E-03

Zone 2a: moderate selection; cut-off at moderate gamma for St population

Lower and upper bounds of St popn gamma

0.250000000 165.343918

Probability of zone 2a= 0.394631982

Coefficients for bivariate distribution of q1 and q2

a1= 4.49999981E-02 a2= 0.404999971

b11= 5.00000140E-04 b12= 9.00000241E-03 b22= 4.05000076E-02

Net probability of zone 2a using Simpsons rule= 0.394790083

Contributions to mean load statistics over zone 2a

Contributions to mean loads within In and St= 5.88031001E-02 3.13967210E-03

Contribution to load between In and St= 2.80707404E-02

Contributions to homozygous loads for In and St= 5.88309281E-02 3.17610311E-03

Contributions to inbreeding loads= 2.77700437E-05 3.64316766E-05

Selection coefficients for In and St homokaryotypes

3.02649140E-02 -2.52444744E-02

Selection coefficients for In and St homokaryotypes

3.02649140E-02 -2.52444744E-02

Contributions to mean A2 freqs= 8.65829811E-02 2.51680110E-02

Contributions to mean diversities= 2.19554058E-04 8.56809260E-04

Contributions to mean proportions of seg. sites= 8.60231172E-04 3.74311372E-03

Contributions to delta-theta values= 9.45217013E-02 0.187912405

Zone 2b: moderate selection; gamma for St reaches high value

Lower and upper bounds of St popn gamma

165.343918 1653.43921

Probability of zone 2b 0.368480891

Net probability of zone 2b using Simpsons rule= 0.368480951

Contributions to mean load statistics over zone 2b

Contributions to mean loads within In and St= 3.47603607E-04 1.93223765E-04

Contribution to load between In and St= 2.69896933E-04

Contributions to homozygous loads for In and St= 3.85160762E-04 2.14607353E-04

Contributions to inbreeding loads= 3.75571326E-05 2.13837320E-05

Selection coefficients for In and St homokaryotypes

7.77244568E-05 -7.66515732E-05

Selection coefficients for In and St homokaryotypes

7.77244568E-05 -7.66515732E-05

Contributions to mean A2 freqs= 8.16405282E-06 5.33924367E-06

Contributions to mean diversities= 1.57585691E-05 1.06304697E-05

Contributions to mean proportions of seg. sites= 1.26969258E-04 1.08476204E-04

Contributions to delta-theta values= 0.559678435 0.652328014

Zone 3: strong selection approximation

Lower and upper bounds of St popn gamma= 1653.43921 7500.00049

Zone 3: strong selection approximation

Probability of zone 3= 0.158470273

Mean load statistics over zone 3

Contributions to loads within In and St= 2.03367716E-04 2.03295538E-04

Contribution to load between In and St= 2.03286487E-04

Contributions to homozygous loads for In and St= 2.25873882E-04 2.25873882E-04

Contributions to inbreeding loads= 2.25062668E-05 2.25783224E-05

Selection coefficients for In and St homokaryotypes

5.96046448E-08 0.00000000

Contributions to mean A2 freqs= 7.29340115E-07 7.29340115E-07

Contributions to mean A2 freqs at seg. sites= 0.00000000 0.00000000

Contributions to mean diversities= 1.45273782E-06 1.45801380E-06

Contributions to delta-theta values= 0.632442236 0.643856585

Mean load statistics over all zones

Loads within In and St= 5.96100539E-02 3.79203935E-03

Load between In and St= 2.87896842E-02

Homozygous load for In and St= 5.96979633E-02 3.87258362E-03

Inbreeding loads= 8.78504798E-05 8.05450691E-05

Selection coefficients for In and St homokaryotypes

3.03502679E-02 -2.53126621E-02

Mean frequencies of A2 in In and St= 0.126527742 6.51099384E-02

Ratio of these= 1.94329381

Mean diversities at selected sites in In and St= 2.89923628E-04 1.34104548E-03

Mean diversities at neutral sites in In and St= 7.98654510E-04 7.09359162E-03

pi-n/pi-s for In and St= 0.363015085 0.189050287

Ratio of these= 1.92020380

Mean freqs. of seg. sites= 1.18944980E-03 5.53540280E-03

Ratio of these= 0.214880437

Overall delta-theta values= 0.135252655 0.140499711

Ratio of these= 0.962654352

**h = 0.5**

Zone 1: quasi-neutral zone

Upper bound scaled selection coefficient for neutrality in St metapopulation= 0.250000000

Probability of zone 1= 6.65597618E-02

Integral of selection coefficient over zone 1= 4.26665181E-04

Mean load statistics for zone 1

Mean q1 and q2= 0.600000024

F1 and F2= 0.998336136 0.985221684

Diversities= 7.98654510E-04 7.09359162E-03

Contributions to loads within In and St= 2.55999126E-04 2.55999126E-04

Contribution to load between In and St = 2.55999126E-04

Contributions to homozygous loads for In and St= 2.55999126E-04 2.55999126E-04

Contributions to inbreeding loads for In and St= 1.10962828E-04 1.11094516E-04

Contributions to selection coefficients for In and St homokaryotypes

0.00000000 0.00000000

Contributions to mean A2 freqs= 3.99358571E-02 3.99358571E-02

Contributions to mean diversities= 5.31582555E-05 4.72147774E-04

Contributions to mean freqs. of seg. site= 1.88227175E-04 1.66928873E-03

Delta-theta values= -1.93631649E-03 -3.45575809E-03

Zone 2a: moderate selection; cut-off at moderate gamma for St population

Lower and upper bounds of St popn gamma

0.250000000 82.6719589

Probability of zone 2a= 0.310401231

Coefficients for bivariate distribution of q1 and q2

a1= 5.00000007E-02 a2= 0.449999988

b11= 0.00000000 b12= 0.00000000 b22= 0.00000000

Net probability of zone 2a using Simpsons rule= 0.310417920

Contributions to mean load statistics over zone 2a

Contributions to mean loads within In and St= 5.19800521E-02 3.07901087E-03

Contribution to load between In and St= 2.75295284E-02

Contributions to homozygous loads for In and St= 5.19800521E-02 3.07901087E-03

Contributions to inbreeding loads= 0.00000000 0.00000000

Selection coefficients for In and St homokaryotypes

2.41540074E-02 -2.47519016E-02

Selection coefficients for In and St homokaryotypes

2.41540074E-02 -2.47519016E-02

Contributions to mean A2 freqs= 8.31320733E-02 2.50896718E-02

Contributions to mean diversities= 1.99594200E-04 8.10983824E-04

Contributions to mean proportions of seg. sites= 7.55451620E-04 3.48987966E-03

Contributions to delta-theta values= 6.26689196E-02 0.175570548

Zone 2b: moderate selection; gamma for St reaches high value

Lower and upper bounds of St popn gamma

82.6719589 826.719604

Probability of zone 2b 0.335337222

Net probability of zone 2b using Simpsons rule= 0.335337132

Contributions to mean load statistics over zone 2b

Contributions to mean loads within In and St= 3.54501070E-04 2.30561243E-04

Contribution to load between In and St= 2.92531186E-04

Contributions to homozygous loads for In and St= 3.54501070E-04 2.30561243E-04

Contributions to inbreeding loads= 0.00000000 0.00000000

Selection coefficients for In and St homokaryotypes

6.19888306E-05 -6.19888306E-05

Selection coefficients for In and St homokaryotypes

6.19888306E-05 -6.19888306E-05

Contributions to mean A2 freqs= 1.54274439E-05 1.02717377E-05

Contributions to mean diversities= 2.56381045E-05 2.03929721E-05

Contributions to mean proportions of seg. sites= 1.84170436E-04 1.98641254E-04

Contributions to delta-theta values= 0.506124198 0.635780811

Zone 3: strong selection approximation

Lower and upper bounds of St popn gamma= 826.719604 7500.00049

Zone 3: strong selection approximation

Probability of zone 3= 0.275844693

Mean load statistics over zone 3

Contributions to loads within In and St= 3.10017320E-04 3.10017291E-04

Contribution to load between In and St= 3.10017320E-04

Contributions to homozygous loads for In and St= 3.10017320E-04 3.10017291E-04

Contributions to inbreeding loads= 0.00000000 0.00000000

Selection coefficients for In and St homokaryotypes

0.00000000 0.00000000

Contributions to mean A2 freqs= 1.61473508E-06 1.61473497E-06

Contributions to mean A2 freqs at seg. sites= 0.00000000 0.00000000

Contributions to mean diversities= 3.20820618E-06 3.22708979E-06

Contributions to delta-theta values= 0.623998046 0.642998099

Mean load statistics over all zones

Loads within In and St= 5.29005714E-02 3.87558853E-03

Load between In and St= 2.83880737E-02

Homozygous load for In and St= 5.29005714E-02 3.87558853E-03

Inbreeding loads= 0.00000000 0.00000000

Selection coefficients for In and St homokaryotypes

2.42145061E-02 -2.48154402E-02

Mean frequencies of A2 in In and St= 0.123084977 6.50374219E-02

Ratio of these= 1.89252543

Mean diversities at selected sites in In and St= 2.81598768E-04 1.30675151E-03

Mean diversities at neutral sites in In and St= 7.98654510E-04 7.09359162E-03

pi-n/pi-s for In and St= 0.352591485 0.184215784

Ratio of these= 1.91401339

Mean freqs. of seg. sites= 1.15811999E-03 5.38987946E-03

Ratio of these= 0.214869365

Overall delta-theta values= 0.137361288 0.139866829

Ratio of these= 0.982086241

**Inversion frequency= 0.3**

**h = 0.05**

Zone 1: quasi-neutral zone

Upper bound scaled selection coefficient for neutrality in St metapopulation= 0.250000000

Probability of zone 1= 7.17716143E-02

Integral of selection coefficient over zone 1= 5.91524295E-04

Mean load statistics for zone 1

Mean q1 and q2= 0.600000024

F1 and F2= 0.995024860 0.988467872

Diversities= 2.38806708E-03 5.53542143E-03

Contributions to loads within In and St= 3.54278920E-04 3.53441137E-04

Contribution to load between In and St = 2.27145327E-04

Contributions to homozygous loads for In and St= 3.54914577E-04 3.54914577E-04

Contributions to inbreeding loads for In and St= 0.00000000 0.00000000

Contributions to selection coefficients for In and St homokaryotypes

1.27136707E-04 1.26302242E-04

Contributions to mean A2 freqs= 4.30629700E-02 4.30629700E-02

Contributions to mean diversities= 1.71395426E-04 3.97286145E-04

Contributions to mean freqs. of seg. site= 6.07366499E-04 1.40566088E-03

Delta-theta values= -1.15239620E-03 -2.70831585E-03

Zone 2a: moderate selection; cut-off at moderate gamma for St population

Lower and upper bounds of St popn gamma

0.250000000 250.000000

Probability of zone 2a= 0.484664857

Coefficients for bivariate distribution of q1 and q2

a1= 1.50000006E-02 a2= 3.50000001E-02

b11= 4.05000001E-02 b12= 0.189000010 b22= 0.220499992

Net probability of zone 2a using Simpsons rule= 0.485215813

Contributions to mean load statistics over zone 2a

Contributions to mean loads within In and St= 4.43741642E-02 4.33792965E-03

Contribution to load between In and St= 4.45975969E-03

Contributions to homozygous loads for In and St= 4.60529141E-02 6.00638194E-03

Contributions to inbreeding loads= 1.67873094E-03 1.66844937E-03

Selection coefficients for In and St homokaryotypes

3.91283035E-02 -1.21831894E-04

Selection coefficients for In and St homokaryotypes

3.91283035E-02 -1.21831894E-04

Contributions to mean A2 freqs= 7.29331598E-02 2.77154911E-02

Contributions to mean diversities= 8.27200420E-04 1.20763306E-03

Contributions to mean proportions of seg. sites= 3.28135886E-03 5.45572676E-03

Contributions to delta-theta values= 0.105647385 0.214702666

Zone 2b: moderate selection; gamma for St reaches high value

Lower and upper bounds of St popn gamma

250.000000 2500.00000

Probability of zone 2b 0.367502570

Net probability of zone 2b using Simpsons rule= 0.367502689

Contributions to mean load statistics over zone 2b

Contributions to mean loads within In and St= 3.07533453E-04 2.01465227E-04

Contribution to load between In and St= 2.10272658E-04

Contributions to homozygous loads for In and St= 2.40883022E-03 1.79125171E-03

Contributions to inbreeding loads= 2.10129586E-03 1.58978614E-03

Selection coefficients for In and St homokaryotypes

9.72747803E-05 -8.82148743E-06

Selection coefficients for In and St homokaryotypes

9.72747803E-05 -8.82148743E-06

Contributions to mean A2 freqs= 2.86921222E-05 2.26064494E-05

Contributions to mean diversities= 5.51251251E-05 4.44320794E-05

Contributions to mean proportions of seg. sites= 4.27900348E-04 3.93019873E-04

Contributions to delta-theta values= 0.542955279 0.598917365

Zone 3: strong selection approximation

Lower and upper bounds of St popn gamma= 2500.00000 5833.33350

Zone 3: strong selection approximation

Probability of zone 3= 6.42038584E-02

Mean load statistics over zone 3

Contributions to loads within In and St= 1.19226963E-04 1.15312840E-04

Contribution to load between In and St= 1.12377245E-04

Contributions to homozygous loads for In and St= 1.12356606E-03 1.12356606E-03

Contributions to inbreeding loads= 1.00433978E-03 1.00825378E-03

Selection coefficients for In and St homokaryotypes

6.85453415E-06 2.92062759E-06

Contributions to mean A2 freqs= 2.28328145E-06 2.28328145E-06

Contributions to mean A2 freqs at seg. sites= 0.00000000 0.00000000

Contributions to mean diversities= 4.53400389E-06 4.55254940E-06

Contributions to delta-theta values= 0.622218728 0.635765672

Mean load statistics over all zones

Loads within In and St= 4.51552048E-02 5.00814850E-03

Load between In and St= 5.00955479E-03

Homozygous load for In and St= 4.99402247E-02 9.27611440E-03

Inbreeding loads= 4.78500221E-03 4.26796265E-03

Selection coefficients for In and St homokaryotypes

3.93505096E-02 -1.43051147E-06

Mean frequencies of A2 in In and St= 0.116027102 7.08033442E-02

Ratio of these= 1.63872349

Mean diversities at selected sites in In and St= 1.05825497E-03 1.65390386E-03

Mean diversities at neutral sites in In and St= 2.38806708E-03 5.53542143E-03

pi-n/pi-s for In and St= 0.443142891 0.298785537

Ratio of these= 1.48314703

Mean freqs. of seg. sites= 4.35920432E-03 7.29875080E-03

Ratio of these= 0.597253501

Overall delta-theta values= 0.138738871 0.196078777

Ratio of these= 0.707566977

**h = 0.15**

Zone 1: quasi-neutral zone

Upper bound scaled selection coefficient for neutrality in St metapopulation= 0.250000000

Probability of zone 1= 7.17716143E-02

Integral of selection coefficient over zone 1= 5.91524295E-04

Mean load statistics for zone 1

Mean q1 and q2= 0.600000024

F1 and F2= 0.995024860 0.988467872

Diversities= 2.38806708E-03 5.53542143E-03

Contributions to loads within In and St= 3.54420161E-04 3.53768584E-04

Contribution to load between In and St = 2.55538500E-04

Contributions to homozygous loads for In and St= 3.54914577E-04 3.54914577E-04

Contributions to inbreeding loads for In and St= 8.96389131E-03 8.98446236E-03

Contributions to selection coefficients for In and St homokaryotypes

9.88841057E-05 9.82284546E-05

Contributions to mean A2 freqs= 4.30629700E-02 4.30629700E-02

Contributions to mean diversities= 1.71395426E-04 3.97286145E-04

Contributions to mean freqs. of seg. site= 6.07366499E-04 1.40566088E-03

Delta-theta values= -1.15239620E-03 -2.70831585E-03

Zone 2a: moderate selection; cut-off at moderate gamma for St population

Lower and upper bounds of St popn gamma

0.250000000 416.666656

Probability of zone 2a= 0.566705585

Coefficients for bivariate distribution of q1 and q2

a1= 4.50000018E-02 a2= 0.105000004

b11= 3.15000005E-02 b12= 0.147000000 b22= 0.171499997

Net probability of zone 2a using Simpsons rule= 0.568667829

Contributions to mean load statistics over zone 2a

Contributions to mean loads within In and St= 2.93394979E-02 4.54712799E-03

Contribution to load between In and St= 6.74624601E-03

Contributions to homozygous loads for In and St= 3.02201416E-02 5.41719282E-03

Contributions to inbreeding loads= 8.80589534E-04 8.70069722E-04

Selection coefficients for In and St homokaryotypes

2.23399401E-02 -2.20155716E-03

Selection coefficients for In and St homokaryotypes

2.23399401E-02 -2.20155716E-03

Contributions to mean A2 freqs= 6.38362095E-02 2.86657270E-02

Contributions to mean diversities= 6.61610859E-04 1.02475926E-03

Contributions to mean proportions of seg. sites= 2.75238417E-03 4.56257118E-03

Contributions to delta-theta values= 0.147203624 0.203173220

Zone 2b: moderate selection; gamma for St reaches high value

Lower and upper bounds of St popn gamma

416.666656 4166.66650

Probability of zone 2b 0.332554340

Net probability of zone 2b using Simpsons rule= 0.332554728

Contributions to mean load statistics over zone 2b

Contributions to mean loads within In and St= 2.11136925E-04 1.38848831E-04

Contribution to load between In and St= 1.71995038E-04

Contributions to homozygous loads for In and St= 6.88628992E-04 4.57912247E-04

Contributions to inbreeding loads= 4.77491441E-04 3.19063751E-04

Selection coefficients for In and St homokaryotypes

3.91602516E-05 -3.31401825E-05

Selection coefficients for In and St homokaryotypes

3.91602516E-05 -3.31401825E-05

Contributions to mean A2 freqs= 5.66932931E-06 4.06377376E-06

Contributions to mean diversities= 1.12059861E-05 8.08312052E-06

Contributions to mean proportions of seg. sites= 1.04055616E-04 8.03686198E-05

Contributions to delta-theta values= 0.617935836 0.643184006

Zone 3: strong selection approximation

Lower and upper bounds of St popn gamma= 4166.66650 5833.33350

Zone 3: strong selection approximation

Probability of zone 3= 1.71113610E-02

Mean load statistics over zone 3

Contributions to loads within In and St= 6.01148968E-05 5.99862105E-05

Contribution to load between In and St= 5.98896186E-05

Contributions to homozygous loads for In and St= 1.99629765E-04 1.99629765E-04

Contributions to inbreeding loads= 1.39514988E-04 1.39643671E-04

Selection coefficients for In and St homokaryotypes

2.38418579E-07 1.19209290E-07

Contributions to mean A2 freqs= 2.89600564E-07 2.89600564E-07

Contributions to mean A2 freqs at seg. sites= 0.00000000 0.00000000

Contributions to mean diversities= 5.78256220E-07 5.78794129E-07

Contributions to delta-theta values= 0.640252352 0.643110156

Mean load statistics over all zones

Loads within In and St= 2.99651697E-02 5.09973150E-03

Load between In and St= 7.23366952E-03

Homozygous load for In and St= 3.14633138E-02 6.42964942E-03

Inbreeding loads= 1.49809045E-03 1.32992317E-03

Selection coefficients for In and St homokaryotypes

2.24750638E-02 -2.13623047E-03

Mean frequencies of A2 in In and St= 0.106905140 7.17330500E-02

Ratio of these= 1.49031913

Mean diversities at selected sites in In and St= 8.44790484E-04 1.43070729E-03

Mean diversities at neutral sites in In and St= 2.38806708E-03 5.53542143E-03

pi-n/pi-s for In and St= 0.353754908 0.258464009

Ratio of these= 1.36868155

Mean freqs. of seg. sites= 3.46950884E-03 6.05435437E-03

Ratio of these= 0.573060095

Overall delta-theta values= 0.136161089 0.161632001

Ratio of these= 0.842414200

**h = 0.25**

Zone 1: quasi-neutral zone

Upper bound scaled selection coefficient for neutrality in St metapopulation= 0.250000000

Probability of zone 1= 7.17716143E-02

Integral of selection coefficient over zone 1= 5.91524295E-04

Mean load statistics for zone 1

Mean q1 and q2= 0.600000024

F1 and F2= 0.995024860 0.988467872

Diversities= 2.38806708E-03 5.53542143E-03

Contributions to loads within In and St= 3.54561460E-04 3.54095973E-04

Contribution to load between In and St = 2.83931673E-04

Contributions to homozygous loads for In and St= 3.54914577E-04 3.54914577E-04

Contributions to inbreeding loads for In and St= 2.33021262E-03 2.33199028E-03

Contributions to selection coefficients for In and St homokaryotypes

7.06315041E-05 7.01546669E-05

Contributions to mean A2 freqs= 4.30629700E-02 4.30629700E-02

Contributions to mean diversities= 1.71395426E-04 3.97286145E-04

Contributions to mean freqs. of seg. site= 6.07366499E-04 1.40566088E-03

Delta-theta values= -1.15239620E-03 -2.70831585E-03

Zone 2a: moderate selection; cut-off at moderate gamma for St population

Lower and upper bounds of St popn gamma

0.250000000 416.666656

Probability of zone 2a= 0.566705585

Coefficients for bivariate distribution of q1 and q2

a1= 7.50000030E-02 a2= 0.174999997

b11= 2.25000009E-02 b12= 0.105000004 b22= 0.122499995

Net probability of zone 2a using Simpsons rule= 0.568667829

Contributions to mean load statistics over zone 2a

Contributions to mean loads within In and St= 2.16192212E-02 4.67181252E-03

Contribution to load between In and St= 7.80793047E-03

Contributions to homozygous loads for In and St= 2.20418945E-02 5.09244250E-03

Contributions to inbreeding loads= 4.22674028E-04 4.20632743E-04

Selection coefficients for In and St homokaryotypes

1.37163401E-02 -3.14104557E-03

Selection coefficients for In and St homokaryotypes

1.37163401E-02 -3.14104557E-03

Contributions to mean A2 freqs= 5.73776364E-02 2.88490336E-02

Contributions to mean diversities= 5.62830479E-04 8.93018441E-04

Contributions to mean proportions of seg. sites= 2.37974734E-03 3.96220805E-03

Contributions to delta-theta values= 0.160929382 0.200396061

Zone 2b: moderate selection; gamma for St reaches high value

Lower and upper bounds of St popn gamma

416.666656 4166.66650

Probability of zone 2b 0.332554340

Net probability of zone 2b using Simpsons rule= 0.332554728

Contributions to mean load statistics over zone 2b

Contributions to mean loads within In and St= 1.99517890E-04 1.25448569E-04

Contribution to load between In and St= 1.61694639E-04

Contributions to homozygous loads for In and St= 3.96641582E-04 2.50122772E-04

Contributions to inbreeding loads= 1.97123838E-04 1.24674320E-04

Selection coefficients for In and St homokaryotypes

3.78489494E-05 -3.62396240E-05

Selection coefficients for In and St homokaryotypes

3.78489494E-05 -3.62396240E-05

Contributions to mean A2 freqs= 3.31572141E-06 2.28527824E-06

Contributions to mean diversities= 6.58235513E-06 4.55408463E-06

Contributions to mean proportions of seg. sites= 6.38421261E-05 4.73814471E-05

Contributions to delta-theta values= 0.634215117 0.659007728

Zone 3: strong selection approximation

Lower and upper bounds of St popn gamma= 4166.66650 5833.33350

Zone 3: strong selection approximation

Probability of zone 3= 1.71113610E-02

Mean load statistics over zone 3

Contributions to loads within In and St= 5.99470986E-05 5.99139348E-05

Contribution to load between In and St= 5.98891820E-05

Contributions to homozygous loads for In and St= 1.19777971E-04 1.19777971E-04

Contributions to inbreeding loads= 5.98308980E-05 5.98640181E-05

Selection coefficients for In and St homokaryotypes

5.96046448E-08 0.00000000

Contributions to mean A2 freqs= 1.73760455E-07 1.73760455E-07

Contributions to mean A2 freqs at seg. sites= 0.00000000 0.00000000

Contributions to mean diversities= 3.47180645E-07 3.47374424E-07

Contributions to delta-theta values= 0.642255008 0.643961906

Mean load statistics over all zones

Loads within In and St= 2.22332478E-02 5.21127088E-03

Load between In and St= 8.31344631E-03

Homozygous load for In and St= 2.29132269E-02 5.81725780E-03

Inbreeding loads= 6.79981895E-04 6.05989713E-04

Selection coefficients for In and St homokaryotypes

1.38233900E-02 -3.10695171E-03

Mean frequencies of A2 in In and St= 0.100444093 7.19144642E-02

Ratio of these= 1.39671612

Mean diversities at selected sites in In and St= 7.41155469E-04 1.29520602E-03

Mean diversities at neutral sites in In and St= 2.38806708E-03 5.53542143E-03

pi-n/pi-s for In and St= 0.310357898 0.233985081

Ratio of these= 1.32640040

Mean freqs. of seg. sites= 3.05439881E-03 5.41871181E-03

Ratio of these= 0.563676178

Overall delta-theta values= 0.139134467 0.152002573

Ratio of these= 0.915342867

**h = 0.35**

Zone 1: quasi-neutral zone

Upper bound scaled selection coefficient for neutrality in St metapopulation= 0.250000000

Probability of zone 1= 7.17716143E-02

Integral of selection coefficient over zone 1= 5.91524295E-04

Mean load statistics for zone 1

Mean q1 and q2= 0.600000024

F1 and F2= 0.995024860 0.988467872

Diversities= 2.38806708E-03 5.53542143E-03

Contributions to loads within In and St= 3.54702701E-04 3.54423421E-04

Contribution to load between In and St = 3.12324817E-04

Contributions to homozygous loads for In and St= 3.54914577E-04 3.54914577E-04

Contributions to inbreeding loads for In and St= 9.99197597E-04 9.99654760E-04

Contributions to selection coefficients for In and St homokaryotypes

4.23789024E-05 4.20808792E-05

Contributions to mean A2 freqs= 4.30629700E-02 4.30629700E-02

Contributions to mean diversities= 1.71395426E-04 3.97286145E-04

Contributions to mean freqs. of seg. site= 6.07366499E-04 1.40566088E-03

Delta-theta values= -1.15239620E-03 -2.70831585E-03

Zone 2a: moderate selection; cut-off at moderate gamma for St population

Lower and upper bounds of St popn gamma

0.250000000 297.619049

Probability of zone 2a= 0.511895418

Coefficients for bivariate distribution of q1 and q2

a1= 0.105000004 a2= 0.244999990

b11= 1.35000013E-02 b12= 6.30000010E-02 b22= 7.34999999E-02

Net probability of zone 2a using Simpsons rule= 0.512763560

Contributions to mean load statistics over zone 2a

Contributions to mean loads within In and St= 1.70629695E-02 4.69485950E-03

Contribution to load between In and St= 8.38977937E-03

Contributions to homozygous loads for In and St= 1.72341038E-02 4.86911321E-03

Contributions to inbreeding loads= 1.71151289E-04 1.74254339E-04

Selection coefficients for In and St homokaryotypes

8.63569975E-03 -3.70180607E-03

Selection coefficients for In and St homokaryotypes

8.63569975E-03 -3.70180607E-03

Contributions to mean A2 freqs= 5.23048304E-02 2.83818468E-02

Contributions to mean diversities= 4.95088985E-04 7.93497835E-04

Contributions to mean proportions of seg. sites= 2.10285955E-03 3.51290335E-03

Contributions to delta-theta values= 0.164734066 0.198633254

Zone 2b: moderate selection; gamma for St reaches high value

Lower and upper bounds of St popn gamma

297.619049 2976.19043

Probability of zone 2b 0.359236717

Net probability of zone 2b using Simpsons rule= 0.359237194

Contributions to mean load statistics over zone 2b

Contributions to mean loads within In and St= 2.29747107E-04 1.49293570E-04

Contribution to load between In and St= 1.89145285E-04

Contributions to homozygous loads for In and St= 3.27405316E-04 2.13004707E-04

Contributions to inbreeding loads= 9.76582451E-05 6.37110861E-05

Selection coefficients for In and St homokaryotypes

4.05907631E-05 -3.98159027E-05

Selection coefficients for In and St homokaryotypes

4.05907631E-05 -3.98159027E-05

Contributions to mean A2 freqs= 3.60589456E-06 2.58786895E-06

Contributions to mean diversities= 7.15975284E-06 5.15744068E-06

Contributions to mean proportions of seg. sites= 6.96688876E-05 5.39654175E-05

Contributions to delta-theta values= 0.635404825 0.660944760

Zone 3: strong selection approximation

Lower and upper bounds of St popn gamma= 2976.19043 5833.33350

Zone 3: strong selection approximation

Probability of zone 3= 4.52391505E-02

Mean load statistics over zone 3

Contributions to loads within In and St= 9.23973275E-05 9.23779226E-05

Contribution to load between In and St= 9.23633270E-05

Contributions to homozygous loads for In and St= 1.31947381E-04 1.31947396E-04

Contributions to inbreeding loads= 3.95501083E-05 3.95695424E-05

Selection coefficients for In and St homokaryotypes

5.96046448E-08 0.00000000

Contributions to mean A2 freqs= 2.38106225E-07 2.38106225E-07

Contributions to mean A2 freqs at seg. sites= 0.00000000 0.00000000

Contributions to mean diversities= 4.75788624E-07 4.76030124E-07

Contributions to delta-theta values= 0.642521858 0.644070745

Mean load statistics over all zones

Loads within In and St= 1.77398175E-02 5.29095437E-03

Load between In and St= 8.98361206E-03

Homozygous load for In and St= 1.80483703E-02 5.56897977E-03

Inbreeding loads= 3.08571529E-04 2.78026127E-04

Selection coefficients for In and St homokaryotypes

8.71795416E-03 -3.69954109E-03

Mean frequencies of A2 in In and St= 9.53716487E-02 7.14476407E-02

Ratio of these= 1.33484674

Mean diversities at selected sites in In and St= 6.74119976E-04 1.19641738E-03

Mean diversities at neutral sites in In and St= 2.38806708E-03 5.53542143E-03

pi-n/pi-s for In and St= 0.282286882 0.216138437

Ratio of these= 1.30604661

Mean freqs. of seg. sites= 2.78461678E-03 4.97727422E-03

Ratio of these= 0.559466243

Overall delta-theta values= 0.141137719 0.147208452

Ratio of these= 0.958760977

**h = 0.45**

Zone 1: quasi-neutral zone

Upper bound scaled selection coefficient for neutrality in St metapopulation= 0.250000000

Probability of zone 1= 7.17716143E-02

Integral of selection coefficient over zone 1= 5.91524295E-04

Mean load statistics for zone 1

Mean q1 and q2= 0.600000024

F1 and F2= 0.995024860 0.988467872

Diversities= 2.38806708E-03 5.53542143E-03

Contributions to loads within In and St= 3.54843971E-04 3.54750897E-04

Contribution to load between In and St = 3.40717990E-04

Contributions to homozygous loads for In and St= 3.54914577E-04 3.54914577E-04

Contributions to inbreeding loads for In and St= 4.28325788E-04 4.28465719E-04

Contributions to selection coefficients for In and St homokaryotypes

1.41263008E-05 1.40070915E-05

Contributions to mean A2 freqs= 4.30629700E-02 4.30629700E-02

Contributions to mean diversities= 1.71395426E-04 3.97286145E-04

Contributions to mean freqs. of seg. site= 6.07366499E-04 1.40566088E-03

Delta-theta values= -1.15239620E-03 -2.70831585E-03

Zone 2a: moderate selection; cut-off at moderate gamma for St population

Lower and upper bounds of St popn gamma

0.250000000 165.343918

Probability of zone 2a= 0.423769295

Coefficients for bivariate distribution of q1 and q2

a1= 0.135000005 a2= 0.314999998

b11= 4.50000120E-03 b12= 2.10000053E-02 b22= 2.45000049E-02

Net probability of zone 2a using Simpsons rule= 0.423940033

Contributions to mean load statistics over zone 2a

Contributions to mean loads within In and St= 1.40841501E-02 4.66177054E-03

Contribution to load between In and St= 8.70152935E-03

Contributions to homozygous loads for In and St= 1.41213546E-02 4.70097130E-03

Contributions to inbreeding loads= 3.71931746E-05 3.92016846E-05

Selection coefficients for In and St homokaryotypes

5.36817312E-03 -4.04787064E-03

Selection coefficients for In and St homokaryotypes

5.36817312E-03 -4.04787064E-03

Contributions to mean A2 freqs= 4.86895852E-02 2.81876605E-02

Contributions to mean diversities= 4.43780067E-04 7.16343930E-04

Contributions to mean proportions of seg. sites= 1.87418004E-03 3.15188640E-03

Contributions to delta-theta values= 0.159943998 0.193688631

Zone 2b: moderate selection; gamma for St reaches high value

Lower and upper bounds of St popn gamma

165.343918 1653.43921

Probability of zone 2b 0.373131454

Net probability of zone 2b using Simpsons rule= 0.373131484

Contributions to mean load statistics over zone 2b

Contributions to mean loads within In and St= 2.74873135E-04 1.97035726E-04

Contribution to load between In and St= 2.35806321E-04

Contributions to homozygous loads for In and St= 3.05171561E-04 2.18840098E-04

Contributions to inbreeding loads= 3.02984499E-05 2.18045625E-05

Selection coefficients for In and St homokaryotypes

3.90410423E-05 -3.87430191E-05

Selection coefficients for In and St homokaryotypes

3.90410423E-05 -3.87430191E-05

Contributions to mean A2 freqs= 5.47105810E-06 4.30129739E-06

Contributions to mean diversities= 1.08385138E-05 8.56367296E-06

Contributions to mean proportions of seg. sites= 1.02941900E-04 8.72998862E-05

Contributions to delta-theta values= 0.626466751 0.651984870

Zone 3: strong selection approximation

Lower and upper bounds of St popn gamma= 1653.43921 5833.33350

Zone 3: strong selection approximation

Probability of zone 3= 0.119470537

Mean load statistics over zone 3

Contributions to loads within In and St= 1.66747835E-04 1.66737227E-04

Contribution to load between In and St= 1.66729194E-04

Contributions to homozygous loads for In and St= 1.85254728E-04 1.85254728E-04

Contributions to inbreeding loads= 1.85068293E-05 1.85174504E-05

Selection coefficients for In and St homokaryotypes

0.00000000 0.00000000

Contributions to mean A2 freqs= 5.01884870E-07 5.01884927E-07

Contributions to mean A2 freqs at seg. sites= 0.00000000 0.00000000

Contributions to mean diversities= 1.00266050E-06 1.00329282E-06

Contributions to delta-theta values= 0.641861200 0.643790245

Mean load statistics over all zones

Loads within In and St= 1.48806153E-02 5.38029429E-03

Load between In and St= 9.44478251E-03

Homozygous load for In and St= 1.49666956E-02 5.45998104E-03

Inbreeding loads= 8.60690707E-05 7.96874010E-05

Selection coefficients for In and St homokaryotypes

5.42110205E-03 -4.07278538E-03

Mean frequencies of A2 in In and St= 9.17585269E-02 7.12554306E-02

Ratio of these= 1.28774083

Mean diversities at selected sites in In and St= 6.27016649E-04 1.12319703E-03

Mean diversities at neutral sites in In and St= 2.38806708E-03 5.53542143E-03

pi-n/pi-s for In and St= 0.262562424 0.202910841

Ratio of these= 1.29397929

Mean freqs. of seg. sites= 2.59442069E-03 4.65483963E-03

Ratio of these= 0.557359815

Overall delta-theta values= 0.142586291 0.143942416

Ratio of these= 0.990578711

**h = 0.5**

Zone 1: quasi-neutral zone

Upper bound scaled selection coefficient for neutrality in St metapopulation= 0.250000000

Probability of zone 1= 7.17716143E-02

Integral of selection coefficient over zone 1= 5.91524295E-04

Mean load statistics for zone 1

Mean q1 and q2= 0.600000024

F1 and F2= 0.995024860 0.988467872

Diversities= 2.38806708E-03 5.53542143E-03

Contributions to loads within In and St= 3.54914577E-04 3.54914577E-04

Contribution to load between In and St = 3.54914577E-04

Contributions to homozygous loads for In and St= 3.54914577E-04 3.54914577E-04

Contributions to inbreeding loads for In and St= 1.11061643E-04 1.11089779E-04

Contributions to selection coefficients for In and St homokaryotypes

0.00000000 0.00000000

Contributions to mean A2 freqs= 4.30629700E-02 4.30629700E-02

Contributions to mean diversities= 1.71395426E-04 3.97286145E-04

Contributions to mean freqs. of seg. site= 6.07366499E-04 1.40566088E-03

Delta-theta values= -1.15239620E-03 -2.70831585E-03

Zone 2a: moderate selection; cut-off at moderate gamma for St population

Lower and upper bounds of St popn gamma

0.250000000 82.6719589

Probability of zone 2a= 0.333978772

Coefficients for bivariate distribution of q1 and q2

a1= 0.150000006 a2= 0.349999994

b11= 0.00000000 b12= 0.00000000 b22= 0.00000000

Net probability of zone 2a using Simpsons rule= 0.333996743

Contributions to mean load statistics over zone 2a

Contributions to mean loads within In and St= 1.29197687E-02 4.59824828E-03

Contribution to load between In and St= 8.75901338E-03

Contributions to homozygous loads for In and St= 1.29197687E-02 4.59824828E-03

Contributions to inbreeding loads= 0.00000000 0.00000000

Selection coefficients for In and St homokaryotypes

4.15211916E-03 -4.16946411E-03

Selection coefficients for In and St homokaryotypes

4.15211916E-03 -4.16946411E-03

Contributions to mean A2 freqs= 4.73174602E-02 2.82235350E-02

Contributions to mean diversities= 4.15730785E-04 6.77718839E-04

Contributions to mean proportions of seg. sites= 1.71878329E-03 2.93881260E-03

Contributions to delta-theta values= 0.141890287 0.181856632

Zone 2b: moderate selection; gamma for St reaches high value

Lower and upper bounds of St popn gamma

82.6719589 826.719604

Probability of zone 2b 0.349868536

Net probability of zone 2b using Simpsons rule= 0.349868268

Contributions to mean load statistics over zone 2b

Contributions to mean loads within In and St= 2.97388324E-04 2.40370704E-04

Contribution to load between In and St= 2.68879172E-04

Contributions to homozygous loads for In and St= 2.97388324E-04 2.40370704E-04

Contributions to inbreeding loads= 0.00000000 0.00000000

Selection coefficients for In and St homokaryotypes

2.84910202E-05 -2.84910202E-05

Selection coefficients for In and St homokaryotypes

2.84910202E-05 -2.84910202E-05

Contributions to mean A2 freqs= 9.77561376E-06 8.41645215E-06

Contributions to mean diversities= 1.92366297E-05 1.67092021E-05

Contributions to mean proportions of seg. sites= 1.72952539E-04 1.62643730E-04

Contributions to delta-theta values= 0.605403006 0.635522962

Zone 3: strong selection approximation

Lower and upper bounds of St popn gamma= 826.719604 5833.33350

Zone 3: strong selection approximation

Probability of zone 3= 0.232523978

Mean load statistics over zone 3

Contributions to loads within In and St= 2.70919612E-04 2.70919612E-04

Contribution to load between In and St= 2.70919612E-04

Contributions to homozygous loads for In and St= 2.70919612E-04 2.70919612E-04

Contributions to inbreeding loads= 0.00000000 0.00000000

Selection coefficients for In and St homokaryotypes

0.00000000 0.00000000

Contributions to mean A2 freqs= 1.18808327E-06 1.18808327E-06

Contributions to mean A2 freqs at seg. sites= 0.00000000 0.00000000

Contributions to mean diversities= 2.37194740E-06 2.37434983E-06

Contributions to delta-theta values= 0.639776707 0.642919838

Mean load statistics over all zones

Loads within In and St= 1.38429916E-02 5.46445278E-03

Load between In and St= 9.65372752E-03

Homozygous load for In and St= 1.38429916E-02 5.46445278E-03

Inbreeding loads= 0.00000000 0.00000000

Selection coefficients for In and St homokaryotypes

4.18049097E-03 -4.19807434E-03

Mean frequencies of A2 in In and St= 9.03913900E-02 7.12961107E-02

Ratio of these= 1.26783061

Mean diversities at selected sites in In and St= 6.08734845E-04 1.09408854E-03

Mean diversities at neutral sites in In and St= 2.38806708E-03 5.53542143E-03

pi-n/pi-s for In and St= 0.254906923 0.197652251

Ratio of these= 1.28967381

Mean freqs. of seg. sites= 2.52246275E-03 4.53070737E-03

Ratio of these= 0.556748092

Overall delta-theta values= 0.143839598 0.143281400

Ratio of these= 1.00389576

h = 5.00000007E-02

Zone 1: quasi-neutral zone

Upper bound scaled selection coefficient for neutrality in St metapopulation= 0.250000000

Probability of zone 1= 7.93938339E-02

Integral of selection coefficient over zone 1= 9.16082761E-04

Mean load statistics for zone 1

Mean q1 and q2= 0.600000024

F1 and F2= 0.991735518 0.991735518

Diversities= 3.96695128E-03 3.96695128E-03

Contributions to loads within In and St= 5.48014301E-04 5.48014301E-04

Contribution to load between In and St = 3.51775787E-04

Contributions to homozygous loads for In and St= 5.49649703E-04 5.49649703E-04

Contributions to inbreeding loads for In and St= 0.00000000 0.00000000

Contributions to selection coefficients for In and St homokaryotypes

1.96218491E-04 1.96218491E-04

Contributions to mean A2 freqs= 4.76363041E-02 4.76363041E-02

Contributions to mean diversities= 3.14951467E-04 3.14951467E-04

Contributions to mean freqs. of seg. site= 1.11533725E-03 1.11533725E-03

Delta-theta values= -1.81889534E-03 -1.81889534E-03

Zone 2a: moderate selection; cut-off at moderate gamma for St population

Lower and upper bounds of St popn gamma

0.250000000 250.000000

Probability of zone 2a= 0.530339003

Coefficients for bivariate distribution of q1 and q2

a1= 2.50000004E-02 a2= 2.50000004E-02

b11= 0.112499997 b12= 0.224999994 b22= 0.112499997

Net probability of zone 2a using Simpsons rule= 0.530947506

Contributions to mean load statistics over zone 2a

Contributions to mean loads within In and St= 1.09433196E-02 1.09432442E-02

Contribution to load between In and St= 3.48898210E-03

Contributions to homozygous loads for In and St= 1.29702324E-02 1.29701570E-02

Contributions to inbreeding loads= 2.02688994E-03 2.02688924E-03

Selection coefficients for In and St homokaryotypes

7.42661953E-03 7.42655993E-03

Selection coefficients for In and St homokaryotypes

7.42661953E-03 7.42655993E-03

Contributions to mean A2 freqs= 3.68476473E-02 3.68475057E-02

Contributions to mean diversities= 1.07521727E-03 1.07521657E-03

Contributions to mean proportions of seg. sites= 4.60708095E-03 4.60708374E-03

Contributions to delta-theta values= 0.172015607 0.172016680

Zone 2b: moderate selection; gamma for St reaches high value

Lower and upper bounds of St popn gamma

250.000000 2500.00000

Probability of zone 2b 0.348146498

Net probability of zone 2b using Simpsons rule= 0.348146081

Contributions to mean load statistics over zone 2b

Contributions to mean loads within In and St= 2.02783500E-04 2.02783456E-04

Contribution to load between In and St= 1.78376096E-04

Contributions to homozygous loads for In and St= 1.78207271E-03 1.78207236E-03

Contributions to inbreeding loads= 1.57928898E-03 1.57928898E-03

Selection coefficients for In and St homokaryotypes

2.43782997E-05 2.43782997E-05

Selection coefficients for In and St homokaryotypes

2.43782997E-05 2.43782997E-05

Contributions to mean A2 freqs= 1.68378701E-05 1.68378683E-05

Contributions to mean diversities= 3.30400762E-05 3.30400762E-05

Contributions to mean proportions of seg. sites= 2.89292802E-04 2.89293996E-04

Contributions to delta-theta values= 0.594813347 0.594815016

Zone 3: strong selection approximation

Lower and upper bounds of St popn gamma= 2500.00000 4166.66699

Zone 3: strong selection approximation

Probability of zone 3= 3.02635431E-02

Mean load statistics over zone 3

Contributions to loads within In and St= 7.78775284E-05 7.78775284E-05

Contribution to load between In and St= 7.56705194E-05

Contributions to homozygous loads for In and St= 7.56593887E-04 7.56593887E-04

Contributions to inbreeding loads= 6.78716518E-04 6.78716518E-04

Selection coefficients for In and St homokaryotypes

2.20537186E-06 2.20537186E-06

Contributions to mean A2 freqs= 1.22610516E-06 1.22610516E-06

Contributions to mean A2 freqs at seg. sites= 0.00000000 0.00000000

Contributions to mean diversities= 2.44406397E-06 2.44406397E-06

Contributions to delta-theta values= 0.634936810 0.634936810

Mean load statistics over all zones

Loads within In and St= 1.17719946E-02 1.17719192E-02

Load between In and St= 4.09480464E-03

Homozygous load for In and St= 1.60585493E-02 1.60584729E-02

Inbreeding loads= 4.28653089E-03 4.28653043E-03

Selection coefficients for In and St homokaryotypes

7.64781237E-03 7.64769316E-03

Mean frequencies of A2 in In and St= 8.45020190E-02 8.45018774E-02

Ratio of these= 1.00000167

Mean diversities at selected sites in In and St= 1.42565276E-03 1.42565207E-03

Mean diversities at neutral sites in In and St= 3.96695128E-03 3.96695128E-03

pi-n/pi-s for In and St= 0.359382480 0.359382302

Ratio of these= 1.00000048

Mean freqs. of seg. sites= 6.03546342E-03 6.03546714E-03

Ratio of these= 0.999999404

Overall delta-theta values= 0.161979020 0.161979914

Ratio of these= 0.999994457

h = 0.150000006

Zone 1: quasi-neutral zone

Upper bound scaled selection coefficient for neutrality in St metapopulation= 0.250000000

Probability of zone 1= 7.93938339E-02

Integral of selection coefficient over zone 1= 9.16082761E-04

Mean load statistics for zone 1

Mean q1 and q2= 0.600000024

F1 and F2= 0.991735518 0.991735518

Diversities= 3.96695128E-03 3.96695128E-03

Contributions to loads within In and St= 5.48377750E-04 5.48377750E-04

Contribution to load between In and St = 3.95747775E-04

Contributions to homozygous loads for In and St= 5.49649703E-04 5.49649703E-04

Contributions to inbreeding loads for In and St= 8.97829141E-03 8.97829141E-03

Contributions to selection coefficients for In and St homokaryotypes

1.52647495E-04 1.52647495E-04

Contributions to mean A2 freqs= 4.76363041E-02 4.76363041E-02

Contributions to mean diversities= 3.14951467E-04 3.14951467E-04

Contributions to mean freqs. of seg. site= 1.11533725E-03 1.11533725E-03

Delta-theta values= -1.81889534E-03 -1.81889534E-03

Zone 2a: moderate selection; cut-off at moderate gamma for St population

Lower and upper bounds of St popn gamma

0.250000000 416.666656

Probability of zone 2a= 0.616143107

Coefficients for bivariate distribution of q1 and q2

a1= 7.50000030E-02 a2= 7.50000030E-02

b11= 8.74999985E-02 b12= 0.174999997 b22= 8.74999985E-02

Net probability of zone 2a using Simpsons rule= 0.618314326

Contributions to mean load statistics over zone 2a

Contributions to mean loads within In and St= 9.81184002E-03 9.81183443E-03

Contribution to load between In and St= 4.81840083E-03

Contributions to homozygous loads for In and St= 1.07935211E-02 1.07935155E-02

Contributions to inbreeding loads= 9.81690595E-04 9.81690711E-04

Selection coefficients for In and St homokaryotypes

4.98098135E-03 4.98098135E-03

Selection coefficients for In and St homokaryotypes

4.98098135E-03 4.98098135E-03

Contributions to mean A2 freqs= 3.62775214E-02 3.62775065E-02

Contributions to mean diversities= 8.55433580E-04 8.55434744E-04

Contributions to mean proportions of seg. sites= 3.72213428E-03 3.72213335E-03

Contributions to delta-theta values= 0.184646368 0.184645116

Zone 2b: moderate selection; gamma for St reaches high value

Lower and upper bounds of St popn gamma

416.666656 4166.66650

Probability of zone 2b 0.292605937

Net probability of zone 2b using Simpsons rule= 0.292605817

Contributions to mean load statistics over zone 2b

Contributions to mean loads within In and St= 1.28165586E-04 1.28165630E-04

Contribution to load between In and St= 1.26749321E-04

Contributions to homozygous loads for In and St= 4.22474259E-04 4.22474259E-04

Contributions to inbreeding loads= 2.94308440E-04 2.94308498E-04

Selection coefficients for In and St homokaryotypes

1.43051147E-06 1.43051147E-06

Selection coefficients for In and St homokaryotypes

1.43051147E-06 1.43051147E-06

Contributions to mean A2 freqs= 2.79791971E-06 2.79791948E-06

Contributions to mean diversities= 5.56436999E-06 5.56436999E-06

Contributions to mean proportions of seg. sites= 5.51913799E-05 5.51916019E-05

Contributions to delta-theta values= 0.642318487 0.642319918

Zone 3: strong selection approximation

Lower and upper bounds of St popn gamma= 4166.66650 4166.66699

Zone 3: strong selection approximation

Probability of zone 3= 5.96046448E-08

Mean load statistics over zone 3

Contributions to loads within In and St= 3.61872771E-05 3.61872771E-05

Contribution to load between In and St= 3.61198072E-05

Contributions to homozygous loads for In and St= 1.20398276E-04 1.20398276E-04

Contributions to inbreeding loads= 8.42106238E-05 8.42106238E-05

Selection coefficients for In and St homokaryotypes

5.96046448E-08 5.96046448E-08

Contributions to mean A2 freqs= 1.44478491E-07 1.44478491E-07

Contributions to mean A2 freqs at seg. sites= 0.00000000 0.00000000

Contributions to mean diversities= 2.88723214E-07 2.88723214E-07

Contributions to delta-theta values= 0.642802477 0.642802477

Mean load statistics over all zones

Loads within In and St= 1.05245709E-02 1.05245654E-02

Load between In and St= 5.37701789E-03

Homozygous load for In and St= 1.18860435E-02 1.18860379E-02

Inbreeding loads= 1.36148161E-03 1.36148185E-03

Selection coefficients for In and St homokaryotypes

5.13434410E-03 5.13434410E-03

Mean frequencies of A2 in In and St= 8.39167684E-02 8.39167535E-02

Ratio of these= 1.00000012

Mean diversities at selected sites in In and St= 1.17623818E-03 1.17623934E-03

Mean diversities at neutral sites in In and St= 3.96695128E-03 3.96695128E-03

pi-n/pi-s for In and St= 0.296509355 0.296509653

Ratio of these= 0.999998987

Mean freqs. of seg. sites= 4.89553064E-03 4.89552971E-03

Ratio of these= 1.00000024

Overall delta-theta values= 0.147592545 0.147591531

Ratio of these= 1.00000691

h = 0.250000000

Zone 1: quasi-neutral zone

Upper bound scaled selection coefficient for neutrality in St metapopulation= 0.250000000

Probability of zone 1= 7.93938339E-02

Integral of selection coefficient over zone 1= 9.16082761E-04

Mean load statistics for zone 1

Mean q1 and q2= 0.600000024

F1 and F2= 0.991735518 0.991735518

Diversities= 3.96695128E-03 3.96695128E-03

Contributions to loads within In and St= 5.48741140E-04 5.48741140E-04

Contribution to load between In and St = 4.39719734E-04

Contributions to homozygous loads for In and St= 5.49649703E-04 5.49649703E-04

Contributions to inbreeding loads for In and St= 2.33145710E-03 2.33145710E-03

Contributions to selection coefficients for In and St homokaryotypes

1.09016895E-04 1.09016895E-04

Contributions to mean A2 freqs= 4.76363041E-02 4.76363041E-02

Contributions to mean diversities= 3.14951467E-04 3.14951467E-04

Contributions to mean freqs. of seg. site= 1.11533725E-03 1.11533725E-03

Delta-theta values= -1.81889534E-03 -1.81889534E-03

Zone 2a: moderate selection; cut-off at moderate gamma for St population

Lower and upper bounds of St popn gamma

0.250000000 416.666656

Probability of zone 2a= 0.616143107

Coefficients for bivariate distribution of q1 and q2

a1= 0.125000000 a2= 0.125000000

b11= 6.25000000E-02 b12= 0.125000000 b22= 6.25000000E-02

Net probability of zone 2a using Simpsons rule= 0.618314326

Contributions to mean load statistics over zone 2a

Contributions to mean loads within In and St= 8.73961765E-03 8.73961765E-03

Contribution to load between In and St= 5.74993715E-03

Contributions to homozygous loads for In and St= 9.20558628E-03 9.20558628E-03

Contributions to inbreeding loads= 4.65964753E-04 4.65964578E-04

Selection coefficients for In and St homokaryotypes

2.98523903E-03 2.98523903E-03

Selection coefficients for In and St homokaryotypes

2.98523903E-03 2.98523903E-03

Contributions to mean A2 freqs= 3.49729620E-02 3.49729620E-02

Contributions to mean diversities= 7.25806400E-04 7.25805759E-04

Contributions to mean proportions of seg. sites= 3.18490411E-03 3.18490411E-03

Contributions to delta-theta values= 0.191507161 0.191507876

Zone 2b: moderate selection; gamma for St reaches high value

Lower and upper bounds of St popn gamma

416.666656 4166.66650

Probability of zone 2b 0.292605937

Net probability of zone 2b using Simpsons rule= 0.292605817

Contributions to mean load statistics over zone 2b

Contributions to mean loads within In and St= 1.16350129E-04 1.16350129E-04

Contribution to load between In and St= 1.15980576E-04

Contributions to homozygous loads for In and St= 2.31957820E-04 2.31957878E-04

Contributions to inbreeding loads= 1.15607916E-04 1.15607902E-04

Selection coefficients for In and St homokaryotypes

3.57627869E-07 3.57627869E-07

Selection coefficients for In and St homokaryotypes

3.57627869E-07 3.57627869E-07

Contributions to mean A2 freqs= 1.57415695E-06 1.57415673E-06

Contributions to mean diversities= 3.13675514E-06 3.13675446E-06

Contributions to mean proportions of seg. sites= 3.25291512E-05 3.25290566E-05

Contributions to delta-theta values= 0.657894850 0.657893896

Zone 3: strong selection approximation

Lower and upper bounds of St popn gamma= 4166.66650 4166.66699

Zone 3: strong selection approximation

Probability of zone 3= 5.96046448E-08

Mean load statistics over zone 3

Contributions to loads within In and St= 3.61369057E-05 3.61369057E-05

Contribution to load between In and St= 3.61192870E-05

Contributions to homozygous loads for In and St= 7.22385521E-05 7.22385521E-05

Contributions to inbreeding loads= 3.61019956E-05 3.61019956E-05

Selection coefficients for In and St homokaryotypes

0.00000000 0.00000000

Contributions to mean A2 freqs= 8.66869527E-08 8.66869527E-08

Contributions to mean A2 freqs at seg. sites= 0.00000000 0.00000000

Contributions to mean diversities= 1.73289564E-07 1.73289564E-07

Contributions to delta-theta values= 0.643933773 0.643933773

Mean load statistics over all zones

Loads within In and St= 9.44084581E-03 9.44084581E-03

Load between In and St= 6.34175679E-03

Homozygous load for In and St= 1.00594331E-02 1.00594331E-02

Inbreeding loads= 6.18583232E-04 6.18583057E-04

Selection coefficients for In and St homokaryotypes

3.09431553E-03 3.09431553E-03

Mean frequencies of A2 in In and St= 8.26109275E-02 8.26109275E-02

Ratio of these= 1.00000000

Mean diversities at selected sites in In and St= 1.04406802E-03 1.04406732E-03

Mean diversities at neutral sites in In and St= 3.96695128E-03 3.96695128E-03

pi-n/pi-s for In and St= 0.263191551 0.263191372

Ratio of these= 1.00000072

Mean freqs. of seg. sites= 4.33449727E-03 4.33449727E-03

Ratio of these= 1.00000000

Overall delta-theta values= 0.145441532 0.145442069

Ratio of these= 0.999996305

h = 0.349999994

Zone 1: quasi-neutral zone

Upper bound scaled selection coefficient for neutrality in St metapopulation= 0.250000000

Probability of zone 1= 7.93938339E-02

Integral of selection coefficient over zone 1= 9.16082761E-04

Mean load statistics for zone 1

Mean q1 and q2= 0.600000024

F1 and F2= 0.991735518 0.991735518

Diversities= 3.96695128E-03 3.96695128E-03

Contributions to loads within In and St= 5.49104589E-04 5.49104589E-04

Contribution to load between In and St = 4.83691692E-04

Contributions to homozygous loads for In and St= 5.49649703E-04 5.49649703E-04

Contributions to inbreeding loads for In and St= 9.99517622E-04 9.99517622E-04

Contributions to selection coefficients for In and St homokaryotypes

6.53862953E-05 6.53862953E-05

Contributions to mean A2 freqs= 4.76363041E-02 4.76363041E-02

Contributions to mean diversities= 3.14951467E-04 3.14951467E-04

Contributions to mean freqs. of seg. site= 1.11533725E-03 1.11533725E-03

Delta-theta values= -1.81889534E-03 -1.81889534E-03

Zone 2a: moderate selection; cut-off at moderate gamma for St population

Lower and upper bounds of St popn gamma

0.250000000 297.619049

Probability of zone 2a= 0.559083343

Coefficients for bivariate distribution of q1 and q2

a1= 0.174999997 a2= 0.174999997

b11= 3.75000015E-02 b12= 7.50000030E-02 b22= 3.75000015E-02

Net probability of zone 2a using Simpsons rule= 0.560043395

Contributions to mean load statistics over zone 2a

Contributions to mean loads within In and St= 7.90284760E-03 7.90284760E-03

Contribution to load between In and St= 6.39590947E-03

Contributions to homozygous loads for In and St= 8.09507910E-03 8.09507910E-03

Contributions to inbreeding loads= 1.92241176E-04 1.92241147E-04

Selection coefficients for In and St homokaryotypes

1.50579214E-03 1.50579214E-03

Selection coefficients for In and St homokaryotypes

1.50579214E-03 1.50579214E-03

Contributions to mean A2 freqs= 3.32565717E-02 3.32565717E-02

Contributions to mean diversities= 6.36166485E-04 6.36166485E-04

Contributions to mean proportions of seg. sites= 2.80362391E-03 2.80362391E-03

Contributions to delta-theta values= 0.194987178 0.194987178

Zone 2b: moderate selection; gamma for St reaches high value

Lower and upper bounds of St popn gamma

297.619049 2976.19043

Probability of zone 2b 0.332554400

Net probability of zone 2b using Simpsons rule= 0.332554698

Contributions to mean load statistics over zone 2b

Contributions to mean loads within In and St= 1.43709927E-04 1.43709927E-04

Contribution to load between In and St= 1.43522557E-04

Contributions to homozygous loads for In and St= 2.05030825E-04 2.05030796E-04

Contributions to inbreeding loads= 6.13210505E-05 6.13210505E-05

Selection coefficients for In and St homokaryotypes

1.78813934E-07 1.78813934E-07

Selection coefficients for In and St homokaryotypes

1.78813934E-07 1.78813934E-07

Contributions to mean A2 freqs= 1.84033479E-06 1.84033445E-06

Contributions to mean diversities= 3.66746826E-06 3.66746895E-06

Contributions to mean proportions of seg. sites= 3.82597027E-05 3.82589606E-05

Contributions to delta-theta values= 0.659923553 0.659916878

Zone 3: strong selection approximation

Lower and upper bounds of St popn gamma= 2976.19043 4166.66699

Zone 3: strong selection approximation

Probability of zone 3= 1.71113014E-02

Mean load statistics over zone 3

Contributions to loads within In and St= 5.98996594E-05 5.98996594E-05

Contribution to load between In and St= 5.98890474E-05

Contributions to homozygous loads for In and St= 8.55556864E-05 8.55556864E-05

Contributions to inbreeding loads= 2.56560070E-05 2.56560070E-05

Selection coefficients for In and St homokaryotypes

0.00000000 0.00000000

Contributions to mean A2 freqs= 1.24114521E-07 1.24114521E-07

Contributions to mean A2 freqs at seg. sites= 0.00000000 0.00000000

Contributions to mean diversities= 2.48124650E-07 2.48124650E-07

Contributions to delta-theta values= 0.643959999 0.643959999

Mean load statistics over all zones

Loads within In and St= 8.65556207E-03 8.65556207E-03

Load between In and St= 7.08301272E-03

Homozygous load for In and St= 8.93531553E-03 8.93531553E-03

Inbreeding loads= 2.79763335E-04 2.79763306E-04

Selection coefficients for In and St homokaryotypes

1.57129765E-03 1.57129765E-03

Mean frequencies of A2 in In and St= 8.08948427E-02 8.08948427E-02

Ratio of these= 1.00000000

Mean diversities at selected sites in In and St= 9.55033582E-04 9.55033582E-04

Mean diversities at neutral sites in In and St= 3.96695128E-03 3.96695128E-03

pi-n/pi-s for In and St= 0.240747496 0.240747496

Ratio of these= 1.00000000

Mean freqs. of seg. sites= 3.95969301E-03 3.95969208E-03

Ratio of these= 1.00000024

Overall delta-theta values= 0.144324958 0.144324780

Ratio of these= 1.00000119

h = 0.449999988

Zone 1: quasi-neutral zone

Upper bound scaled selection coefficient for neutrality in St metapopulation= 0.250000000

Probability of zone 1= 7.93938339E-02

Integral of selection coefficient over zone 1= 9.16082761E-04

Mean load statistics for zone 1

Mean q1 and q2= 0.600000024

F1 and F2= 0.991735518 0.991735518

Diversities= 3.96695128E-03 3.96695128E-03

Contributions to loads within In and St= 5.49467979E-04 5.49467979E-04

Contribution to load between In and St = 5.27663622E-04

Contributions to homozygous loads for In and St= 5.49649703E-04 5.49649703E-04

Contributions to inbreeding loads for In and St= 4.28423751E-04 4.28423751E-04

Contributions to selection coefficients for In and St homokaryotypes

2.18153000E-05 2.18153000E-05

Contributions to mean A2 freqs= 4.76363041E-02 4.76363041E-02

Contributions to mean diversities= 3.14951467E-04 3.14951467E-04

Contributions to mean freqs. of seg. site= 1.11533725E-03 1.11533725E-03

Delta-theta values= -1.81889534E-03 -1.81889534E-03

Zone 2a: moderate selection; cut-off at moderate gamma for St population

Lower and upper bounds of St popn gamma

0.250000000 165.343918

Probability of zone 2a= 0.465305537

Coefficients for bivariate distribution of q1 and q2

a1= 0.224999994 a2= 0.224999994

b11= 1.25000030E-02 b12= 2.50000060E-02 b22= 1.25000030E-02

Net probability of zone 2a using Simpsons rule= 0.465493798

Contributions to mean load statistics over zone 2a

Contributions to mean loads within In and St= 7.18771713E-03 7.18771713E-03

Contribution to load between In and St= 6.75531616E-03

Contributions to homozygous loads for In and St= 7.23099569E-03 7.23099569E-03

Contributions to inbreeding loads= 4.32698325E-05 4.32698253E-05

Selection coefficients for In and St homokaryotypes

4.32312489E-04 4.32312489E-04

Selection coefficients for In and St homokaryotypes

4.32312489E-04 4.32312489E-04

Contributions to mean A2 freqs= 3.18740532E-02 3.18740532E-02

Contributions to mean diversities= 5.69716678E-04 5.69716678E-04

Contributions to mean proportions of seg. sites= 2.50672596E-03 2.50672479E-03

Contributions to delta-theta values= 0.193686724 0.193686306

Zone 2b: moderate selection; gamma for St reaches high value

Lower and upper bounds of St popn gamma

165.343918 1653.43921

Probability of zone 2b 0.369981229

Net probability of zone 2b using Simpsons rule= 0.369981200

Contributions to mean load statistics over zone 2b

Contributions to mean loads within In and St= 1.98587761E-04 1.98587761E-04

Contribution to load between In and St= 1.98506459E-04

Contributions to homozygous loads for In and St= 2.20562099E-04 2.20562142E-04

Contributions to inbreeding loads= 2.19744743E-05 2.19744743E-05

Selection coefficients for In and St homokaryotypes

5.96046448E-08 5.96046448E-08

Selection coefficients for In and St homokaryotypes

5.96046448E-08 5.96046448E-08

Contributions to mean A2 freqs= 3.17786248E-06 3.17786203E-06

Contributions to mean diversities= 6.32666524E-06 6.32666661E-06

Contributions to mean proportions of seg. sites= 6.43775493E-05 6.43781023E-05

Contributions to delta-theta values= 0.651347995 0.651350915

Zone 3: strong selection approximation

Lower and upper bounds of St popn gamma= 1653.43921 4166.66699

Zone 3: strong selection approximation

Probability of zone 3= 7.34623075E-02

Mean load statistics over zone 3

Contributions to loads within In and St= 1.21799239E-04 1.21799239E-04

Contribution to load between In and St= 1.21792655E-04

Contributions to homozygous loads for In and St= 1.35325143E-04 1.35325143E-04

Contributions to inbreeding loads= 1.35260461E-05 1.35260461E-05

Selection coefficients for In and St homokaryotypes

0.00000000 0.00000000

Contributions to mean A2 freqs= 2.90003101E-07 2.90003101E-07

Contributions to mean A2 freqs at seg. sites= 0.00000000 0.00000000

Contributions to mean diversities= 5.79712150E-07 5.79712150E-07

Contributions to delta-theta values= 0.643689752 0.643689752

Mean load statistics over all zones

Loads within In and St= 8.05757195E-03 8.05757195E-03

Load between In and St= 7.60327885E-03

Homozygous load for In and St= 8.13653227E-03 8.13653227E-03

Inbreeding loads= 7.89520709E-05 7.89520636E-05

Selection coefficients for In and St homokaryotypes

4.54187393E-04 4.54187393E-04

Mean frequencies of A2 in In and St= 7.95138329E-02 7.95138329E-02

Ratio of these= 1.00000000

Mean diversities at selected sites in In and St= 8.91574484E-04 8.91574484E-04

Mean diversities at neutral sites in In and St= 3.96695128E-03 3.96695128E-03

pi-n/pi-s for In and St= 0.224750549 0.224750549

Ratio of these= 1.00000000

Mean freqs. of seg. sites= 3.69221275E-03 3.69221228E-03

Ratio of these= 1.00000012

Overall delta-theta values= 0.143312097 0.143311858

Ratio of these= 1.00000167

**h = 0.5**

Zone 1: quasi-neutral zone

Upper bound scaled selection coefficient for neutrality in St metapopulation= 0.250000000

Probability of zone 1= 7.93938339E-02

Integral of selection coefficient over zone 1= 9.16082761E-04

Mean load statistics for zone 1

Mean q1 and q2= 0.600000024

F1 and F2= 0.991735518 0.991735518

Diversities= 3.96695128E-03 3.96695128E-03

Contributions to loads within In and St= 5.49649703E-04 5.49649703E-04

Contribution to load between In and St = 5.49649703E-04

Contributions to homozygous loads for In and St= 5.49649703E-04 5.49649703E-04

Contributions to inbreeding loads for In and St= 1.11081332E-04 1.11081332E-04

Contributions to selection coefficients for In and St homokaryotypes

0.00000000 0.00000000

Contributions to mean A2 freqs= 4.76363041E-02 4.76363041E-02

Contributions to mean diversities= 3.14951467E-04 3.14951467E-04

Contributions to mean freqs. of seg. site= 1.11533725E-03 1.11533725E-03

Delta-theta values= -1.81889534E-03 -1.81889534E-03

Zone 2a: moderate selection; cut-off at moderate gamma for St population

Lower and upper bounds of St popn gamma

0.250000000 82.6719589

Probability of zone 2a= 0.368007213

Coefficients for bivariate distribution of q1 and q2

a1= 0.250000000 a2= 0.250000000

b11= 0.00000000 b12= 0.00000000 b22= 0.00000000

Net probability of zone 2a using Simpsons rule= 0.368027240

Contributions to mean load statistics over zone 2a

Contributions to mean loads within In and St= 6.98098540E-03 6.98098540E-03

Contribution to load between In and St= 6.98098540E-03

Contributions to homozygous loads for In and St= 6.98098540E-03 6.98098540E-03

Contributions to inbreeding loads= 0.00000000 0.00000000

Selection coefficients for In and St homokaryotypes

0.00000000 0.00000000

Selection coefficients for In and St homokaryotypes

0.00000000 0.00000000

Contributions to mean A2 freqs= 3.20396908E-02 3.20396908E-02

Contributions to mean diversities= 5.37106069E-04 5.37106127E-04

Contributions to mean proportions of seg. sites= 2.33477890E-03 2.33477890E-03

Contributions to delta-theta values= 0.183857381 0.183857322

Zone 2b: moderate selection; gamma for St reaches high value

Lower and upper bounds of St popn gamma

82.6719589 826.719604

Probability of zone 2b 0.365087926

Net probability of zone 2b using Simpsons rule= 0.365087867

Contributions to mean load statistics over zone 2b

Contributions to mean loads within In and St= 2.51333666E-04 2.51333695E-04

Contribution to load between In and St= 2.51333666E-04

Contributions to homozygous loads for In and St= 2.51333666E-04 2.51333695E-04

Contributions to inbreeding loads= 0.00000000 0.00000000

Selection coefficients for In and St homokaryotypes

0.00000000 0.00000000

Selection coefficients for In and St homokaryotypes

0.00000000 0.00000000

Contributions to mean A2 freqs= 6.39624932E-06 6.39624886E-06

Contributions to mean diversities= 1.26978766E-05 1.26978766E-05

Contributions to mean proportions of seg. sites= 1.23475183E-04 1.23474194E-04

Contributions to delta-theta values= 0.635159373 0.635156512

Zone 3: strong selection approximation

Lower and upper bounds of St popn gamma= 826.719604 4166.66699

Zone 3: strong selection approximation

Probability of zone 3= 0.175653934

Mean load statistics over zone 3

Contributions to loads within In and St= 2.19132649E-04 2.19132649E-04

Contribution to load between In and St= 2.19132649E-04

Contributions to homozygous loads for In and St= 2.19132649E-04 2.19132649E-04

Contributions to inbreeding loads= 0.00000000 0.00000000

Selection coefficients for In and St homokaryotypes

0.00000000 0.00000000

Contributions to mean A2 freqs= 7.61433910E-07 7.61433910E-07

Contributions to mean A2 freqs at seg. sites= 0.00000000 0.00000000

Contributions to mean diversities= 1.52164284E-06 1.52164284E-06

Contributions to delta-theta values= 0.642798305 0.642798305

Mean load statistics over all zones

Loads within In and St= 8.00110120E-03 8.00110120E-03

Load between In and St= 8.00110120E-03

Homozygous load for In and St= 8.00110120E-03 8.00110120E-03

Inbreeding loads= 0.00000000 0.00000000

Selection coefficients for In and St homokaryotypes

0.00000000 0.00000000

Mean frequencies of A2 in In and St= 7.96831474E-02 7.96831474E-02

Ratio of these= 1.00000000

Mean diversities at selected sites in In and St= 8.66277085E-04 8.66277143E-04

Mean diversities at neutral sites in In and St= 3.96695128E-03 3.96695128E-03

pi-n/pi-s for In and St= 0.218373507 0.218373522

Ratio of these= 0.999999940

Mean freqs. of seg. sites= 3.58870439E-03 3.58870346E-03

Ratio of these= 1.00000024

Overall delta-theta values= 0.143611372 0.143611133

Ratio of these= 1.00000167

**Section 3**

**Population size = 1250000**

**Mean scaled selection coefficient for whole popn= 250**

**Inversion frequency= 0.1**

**h = 0.05**

Zone 1: quasi-neutral zone

Upper bound scaled selection coefficient for neutrality in St metapopulation= 0.250000000

Probability of zone 1= 0.100879930

Integral of selection coefficient over zone 1= 2.58666510E-03

Mean load statistics for zone 1

Mean q1 and q2= 0.600000024

F1 and F2= 0.999583542 0.996264040

Diversities= 1.99899674E-04 1.79326057E-03

Contributions to loads within In and St= 1.55176641E-03 1.54991169E-03

Contribution to load between In and St = 9.93279391E-04

Contributions to homozygous loads for In and St= 1.55199913E-03 1.55199913E-03

Contributions to inbreeding loads for In and St= 0.00000000 0.00000000

Contributions to selection coefficients for In and St homokaryotypes

5.58316708E-04 5.56468964E-04

Contributions to mean A2 freqs= 6.05279617E-02 6.05279617E-02

Contributions to mean diversities= 2.01658659E-05 1.80903997E-04

Contributions to mean freqs. of seg. site= 7.16799259E-05 6.40928338E-04

Delta-theta values= 1.90687180E-03 -1.36041641E-03

Zone 2a: moderate selection; cut-off at moderate gamma for St population

Lower and upper bounds of St popn gamma

0.250000000 250.000000

Probability of zone 2a= 0.644239604

Coefficients for bivariate distribution of q1 and q2

a1= 5.00000035E-03 a2= 4.49999981E-02

b11= 4.50000027E-03 b12= 8.09999928E-02 b22= 0.364499956

Net probability of zone 2a using Simpsons rule= 0.645013034

Contributions to mean load statistics over zone 2a

Contributions to mean loads within In and St= 2.45952535 1.67962778E-02

Contribution to load between In and St= 0.133179367

Contributions to homozygous loads for In and St= 2.46092200 1.87601838E-02

Contributions to inbreeding loads= 1.39441795E-03 1.96390622E-03

Selection coefficients for In and St homokaryotypes

0.902348101 -0.123426199

Selection coefficients for In and St homokaryotypes

0.902348101 -0.123426199

Contributions to mean A2 freqs= 0.236992940 3.76376286E-02

Contributions to mean diversities= 1.28980653E-04 5.20027534E-04

Contributions to mean proportions of seg. sites= 4.62708384E-04 2.31185462E-03

Contributions to delta-theta values= 1.10622644E-02 0.201973081

Zone 2b: moderate selection; gamma for St reaches high value

Lower and upper bounds of St popn gamma

250.000000 2500.00000

Probability of zone 2b 0.250488877

Net probability of zone 2b using Simpsons rule= 0.250489056

Contributions to mean load statistics over zone 2b

Contributions to mean loads within In and St= 2.77089309E-02 1.97055211E-04

Contribution to load between In and St= 1.56561716E-03

Contributions to homozygous loads for In and St= 2.95267478E-02 1.74499385E-03

Contributions to inbreeding loads= 1.81783980E-03 1.54793961E-03

Selection coefficients for In and St homokaryotypes

2.58045197E-02 -1.36947632E-03

Selection coefficients for In and St homokaryotypes

2.58045197E-02 -1.36947632E-03

Contributions to mean A2 freqs= 2.20881309E-04 7.89496062E-06

Contributions to mean diversities= 1.75049299E-05 1.55215039E-05

Contributions to mean proportions of seg. sites= 9.13633339E-05 1.37801791E-04

Contributions to delta-theta values= 0.320264101 0.600395203

Zone 3: strong selection approximation

Lower and upper bounds of St popn gamma= 2500.00000 1875.00012

Zone 3: strong selection approximation

Probability of zone 3= -7.46548176E-03

Mean load statistics over zone 3

Contributions to loads within In and St= 3.09547831E-05 2.33495030E-05

Contribution to load between In and St= 2.23988318E-05

Contributions to homozygous loads for In and St= 2.23966999E-04 2.23966999E-04

Contributions to inbreeding loads= 1.93012165E-04 2.00617433E-04

Selection coefficients for In and St homokaryotypes

8.58306885E-06 9.53674316E-07

Contributions to mean A2 freqs= 2.37665219E-07 2.37665191E-07

Contributions to mean A2 freqs at seg. sites= 0.00000000 0.00000000

Contributions to mean diversities= 4.55017357E-07 4.73069008E-07

Contributions to delta-theta values= 0.428329468 0.630233049

Mean load statistics over all zones

Loads within In and St= 2.48881721 1.85665954E-02

Load between In and St= 0.135760665

Homozygous load for In and St= 2.49222469 2.22811420E-02

Inbreeding loads= 3.40550253E-03 3.71455052E-03

Selection coefficients for In and St homokaryotypes

0.904921889 -0.124337554

Mean frequencies of A2 in In and St= 0.297742039 9.81737226E-02

Ratio of these= 3.03280783

Mean diversities at selected sites in In and St= 1.67106482E-04 7.16926064E-04

Mean diversities at neutral sites in In and St= 1.99899674E-04 1.79326057E-03

pi-n/pi-s for In and St= 0.835951746 0.399789125

Ratio of these= 2.09098172

Mean freqs. of seg. sites= 6.28575450E-04 3.09512345E-03

Ratio of these= 0.203085750

Overall delta-theta values= 5.68351150E-02 0.178234041

Ratio of these= 0.318879128

**h = 0.15**

Zone 1: quasi-neutral zone

Upper bound scaled selection coefficient for neutrality in St metapopulation= 0.250000000

Probability of zone 1= 0.100879930

Integral of selection coefficient over zone 1= 2.58666510E-03

Mean load statistics for zone 1

Mean q1 and q2= 0.600000024

F1 and F2= 0.999583542 0.996264040

Diversities= 1.99899674E-04 1.79326057E-03

Contributions to loads within In and St= 1.55181810E-03 1.55037560E-03

Contribution to load between In and St = 1.11743936E-03

Contributions to homozygous loads for In and St= 1.55199913E-03 1.55199913E-03

Contributions to inbreeding loads for In and St= 8.56789201E-03 8.95189121E-03

Contributions to selection coefficients for In and St homokaryotypes

4.34279442E-04 4.32848930E-04

Contributions to mean A2 freqs= 6.05279617E-02 6.05279617E-02

Contributions to mean diversities= 2.01658659E-05 1.80903997E-04

Contributions to mean freqs. of seg. site= 7.16799259E-05 6.40928338E-04

Delta-theta values= 1.90687180E-03 -1.36041641E-03

Zone 2a: moderate selection; cut-off at moderate gamma for St population

Lower and upper bounds of St popn gamma

0.250000000 416.666656

Probability of zone 2a= 0.730075061

Coefficients for bivariate distribution of q1 and q2

a1= 1.50000006E-02 a2= 0.135000005

b11= 3.50000011E-03 b12= 6.30000010E-02 b22= 0.283499986

Net probability of zone 2a using Simpsons rule= 0.732833683

Contributions to mean load statistics over zone 2a

Contributions to mean loads within In and St= 1.09473062 1.70535855E-02

Contribution to load between In and St= 0.173965558

Contributions to homozygous loads for In and St= 1.09561074 1.81660671E-02

Contributions to inbreeding loads= 8.80175096E-04 1.11250090E-03

Selection coefficients for In and St homokaryotypes

0.601785719 -0.169892669

Selection coefficients for In and St homokaryotypes

0.601785719 -0.169892669

Contributions to mean A2 freqs= 0.187730908 3.89205143E-02

Contributions to mean diversities= 1.13732654E-04 4.52664652E-04

Contributions to mean proportions of seg. sites= 4.35302267E-04 1.99782685E-03

Contributions to delta-theta values= 7.30719566E-02 0.196158350

Zone 2b: moderate selection; gamma for St reaches high value

Lower and upper bounds of St popn gamma

416.666656 4166.66650

Probability of zone 2b 0.168682575

Net probability of zone 2b using Simpsons rule= 0.168682411

Contributions to mean load statistics over zone 2b

Contributions to mean loads within In and St= 1.83484284E-04 1.23860154E-04

Contribution to load between In and St= 1.44717153E-04

Contributions to homozygous loads for In and St= 5.56706509E-04 4.08034044E-04

Contributions to inbreeding loads= 3.73222429E-04 2.84173439E-04

Selection coefficients for In and St homokaryotypes

3.87430191E-05 -2.08616257E-05

Selection coefficients for In and St homokaryotypes

3.87430191E-05 -2.08616257E-05

Contributions to mean A2 freqs= 1.72343914E-06 1.31937577E-06

Contributions to mean diversities= 3.27669272E-06 2.62366916E-06

Contributions to mean proportions of seg. sites= 2.44884595E-05 2.59756034E-05

Contributions to delta-theta values= 0.525292575 0.641660094

Zone 3: strong selection approximation

Lower and upper bounds of St popn gamma= 4166.66650 1875.00012

Zone 3: strong selection approximation

Probability of zone 3= -1.14946365E-02

Mean load statistics over zone 3

Contributions to loads within In and St= 9.68451695E-06 9.43588293E-06

Contribution to load between In and St= 9.40480550E-06

Contributions to homozygous loads for In and St= 3.13491109E-05 3.13491073E-05

Contributions to inbreeding loads= 2.16645876E-05 2.19132271E-05

Selection coefficients for In and St homokaryotypes

2.98023224E-07 5.96046448E-08

Contributions to mean A2 freqs= 2.99693212E-08 2.99693177E-08

Contributions to mean A2 freqs at seg. sites= 0.00000000 0.00000000

Contributions to mean diversities= 5.91506506E-08 5.98508976E-08

Contributions to delta-theta values= 0.599930644 0.640763998

Mean load statistics over all zones

Loads within In and St= 1.09647560 1.87372584E-02

Load between In and St= 0.175237119

Homozygous load for In and St= 1.09775078 2.01574489E-02

Inbreeding loads= 1.27524312E-03 1.42021105E-03

Selection coefficients for In and St homokaryotypes

0.601974249 -0.169410586

Mean frequencies of A2 in In and St= 0.248260632 9.94498208E-02

Ratio of these= 2.49634075

Mean diversities at selected sites in In and St= 1.37234369E-04 6.36252109E-04

Mean diversities at neutral sites in In and St= 1.99899674E-04 1.79326057E-03

pi-n/pi-s for In and St= 0.686516225 0.354801804

Ratio of these= 1.93492877

Mean freqs. of seg. sites= 5.31995203E-04 2.66532181E-03

Ratio of these= 0.199598864

Overall delta-theta values= 8.48191381E-02 0.153101504

Ratio of these= 0.554005921

**h = 0.25**

Zone 1: quasi-neutral zone

Upper bound scaled selection coefficient for neutrality in St metapopulation= 0.250000000

Probability of zone 1= 0.100879930

Integral of selection coefficient over zone 1= 2.58666510E-03

Mean load statistics for zone 1

Mean q1 and q2= 0.600000024

F1 and F2= 0.999583542 0.996264040

Diversities= 1.99899674E-04 1.79326057E-03

Contributions to loads within In and St= 1.55186979E-03 1.55083940E-03

Contribution to load between In and St = 1.24159933E-03

Contributions to homozygous loads for In and St= 1.55199913E-03 1.55199913E-03

Contributions to inbreeding loads for In and St= 2.29599071E-03 2.32917583E-03

Contributions to selection coefficients for In and St homokaryotypes

3.10242176E-04 3.09169292E-04

Contributions to mean A2 freqs= 6.05279617E-02 6.05279617E-02

Contributions to mean diversities= 2.01658659E-05 1.80903997E-04

Contributions to mean freqs. of seg. site= 7.16799259E-05 6.40928338E-04

Delta-theta values= 1.90687180E-03 -1.36041641E-03

Zone 2a: moderate selection; cut-off at moderate gamma for St population

Lower and upper bounds of St popn gamma

0.250000000 416.666656

Probability of zone 2a= 0.730075061

Coefficients for bivariate distribution of q1 and q2

a1= 2.50000004E-02 a2= 0.224999994

b11= 2.50000018E-03 b12= 4.49999981E-02 b22= 0.202499986

Net probability of zone 2a using Simpsons rule= 0.732833683

Contributions to mean load statistics over zone 2a

Contributions to mean loads within In and St= 0.664266050 1.71817001E-02

Contribution to load between In and St= 0.175451159

Contributions to homozygous loads for In and St= 0.664708972 1.77356731E-02

Contributions to inbreeding loads= 4.43455181E-04 5.53942809E-04

Selection coefficients for In and St homokaryotypes

0.386647165 -0.171481848

Selection coefficients for In and St homokaryotypes

0.386647165 -0.171481848

Contributions to mean A2 freqs= 0.161318123 3.89858484E-02

Contributions to mean diversities= 1.00253405E-04 4.00888588E-04

Contributions to mean proportions of seg. sites= 3.93029390E-04 1.75536110E-03

Contributions to delta-theta values= 9.50473547E-02 0.189768732

Zone 2b: moderate selection; gamma for St reaches high value

Lower and upper bounds of St popn gamma

416.666656 4166.66650

Probability of zone 2b 0.168682575

Net probability of zone 2b using Simpsons rule= 0.168682411

Contributions to mean load statistics over zone 2b

Contributions to mean loads within In and St= 1.69180727E-04 1.14781455E-04

Contribution to load between In and St= 1.39668758E-04

Contributions to homozygous loads for In and St= 3.29880510E-04 2.28788718E-04

Contributions to inbreeding loads= 1.60699798E-04 1.14007358E-04

Selection coefficients for In and St homokaryotypes

2.95042992E-05 -2.49147415E-05

Selection coefficients for In and St homokaryotypes

2.95042992E-05 -2.49147415E-05

Contributions to mean A2 freqs= 1.02209731E-06 7.52596804E-07

Contributions to mean diversities= 1.98295925E-06 1.49952575E-06

Contributions to mean proportions of seg. sites= 1.63059303E-05 1.54826739E-05

Contributions to delta-theta values= 0.568560421 0.656394839

Zone 3: strong selection approximation

Lower and upper bounds of St popn gamma= 4166.66650 1875.00012

Zone 3: strong selection approximation

Probability of zone 3= -1.14946365E-02

Mean load statistics over zone 3

Contributions to loads within In and St= 9.47667559E-06 9.41273902E-06

Contribution to load between In and St= 9.40474547E-06

Contributions to homozygous loads for In and St= 1.88094618E-05 1.88094618E-05

Contributions to inbreeding loads= 9.33278807E-06 9.39672555E-06

Selection coefficients for In and St homokaryotypes

5.96046448E-08 0.00000000

Contributions to mean A2 freqs= 1.79815896E-08 1.79815878E-08

Contributions to mean A2 freqs at seg. sites= 0.00000000 0.00000000

Contributions to mean diversities= 3.56795020E-08 3.59316168E-08

Contributions to delta-theta values= 0.619506359 0.642571211

Mean load statistics over all zones

Loads within In and St= 0.665996552 1.88567340E-02

Load between In and St= 0.176841825

Homozygous load for In and St= 0.666609645 1.95352696E-02

Inbreeding loads= 6.13617129E-04 6.78506622E-04

Selection coefficients for In and St homokaryotypes

0.386855543 -0.171148777

Mean frequencies of A2 in In and St= 0.221847132 9.95145813E-02

Ratio of these= 2.22929263

Mean diversities at selected sites in In and St= 1.22437908E-04 5.83328016E-04

Mean diversities at neutral sites in In and St= 1.99899674E-04 1.79326057E-03

pi-n/pi-s for In and St= 0.612496793 0.325289041

Ratio of these= 1.88293087

Mean freqs. of seg. sites= 4.81347932E-04 2.41212896E-03

Ratio of these= 0.199553147

Overall delta-theta values= 9.75803733E-02 0.142045856

Ratio of these= 0.686963916

**h = 0.35**

Zone 1: quasi-neutral zone

Upper bound scaled selection coefficient for neutrality in St metapopulation= 0.250000000

Probability of zone 1= 0.100879930

Integral of selection coefficient over zone 1= 2.58666510E-03

Mean load statistics for zone 1

Mean q1 and q2= 0.600000024

F1 and F2= 0.999583542 0.996264040

Diversities= 1.99899674E-04 1.79326057E-03

Contributions to loads within In and St= 1.55192160E-03 1.55130331E-03

Contribution to load between In and St = 1.36575918E-03

Contributions to homozygous loads for In and St= 1.55199913E-03 1.55199913E-03

Contributions to inbreeding loads for In and St= 9.90397646E-04 9.98931006E-04

Contributions to selection coefficients for In and St homokaryotypes

1.86145306E-04 1.85549259E-04

Contributions to mean A2 freqs= 6.05279617E-02 6.05279617E-02

Contributions to mean diversities= 2.01658659E-05 1.80903997E-04

Contributions to mean freqs. of seg. site= 7.16799259E-05 6.40928338E-04

Delta-theta values= 1.90687180E-03 -1.36041641E-03

Zone 2a: moderate selection; cut-off at moderate gamma for St population

Lower and upper bounds of St popn gamma

0.250000000 297.619049

Probability of zone 2a= 0.674134612

Coefficients for bivariate distribution of q1 and q2

a1= 3.50000001E-02 a2= 0.314999998

b11= 1.50000013E-03 b12= 2.70000007E-02 b22= 0.121499993

Net probability of zone 2a using Simpsons rule= 0.675354481

Contributions to mean load statistics over zone 2a

Contributions to mean loads within In and St= 0.463363528 1.72594432E-02

Contribution to load between In and St= 0.171239361

Contributions to homozygous loads for In and St= 0.463546425 1.74935330E-02

Contributions to inbreeding loads= 1.82989854E-04 2.34080668E-04

Selection coefficients for In and St homokaryotypes

0.253324151 -0.166467428

Selection coefficients for In and St homokaryotypes

0.253324151 -0.166467428

Contributions to mean A2 freqs= 0.143255606 3.81050035E-02

Contributions to mean diversities= 8.99021106E-05 3.58500984E-04

Contributions to mean proportions of seg. sites= 3.54250456E-04 1.56299409E-03

Contributions to delta-theta values= 9.96502638E-02 0.186261654

Zone 2b: moderate selection; gamma for St reaches high value

Lower and upper bounds of St popn gamma

297.619049 2976.19043

Probability of zone 2b 0.222875893

Net probability of zone 2b using Simpsons rule= 0.222876176

Contributions to mean load statistics over zone 2b

Contributions to mean loads within In and St= 2.20605420E-04 1.52200970E-04

Contribution to load between In and St= 1.85185738E-04

Contributions to homozygous loads for In and St= 3.11968208E-04 2.17131557E-04

Contributions to inbreeding loads= 9.13630356E-05 6.49304347E-05

Selection coefficients for In and St homokaryotypes

3.54051590E-05 -3.30209732E-05

Selection coefficients for In and St homokaryotypes

3.54051590E-05 -3.30209732E-05

Contributions to mean A2 freqs= 1.24373946E-06 9.32107866E-07

Contributions to mean diversities= 2.41576095E-06 1.85738384E-06

Contributions to mean proportions of seg. sites= 2.00251507E-05 1.93214710E-05

Contributions to delta-theta values= 0.572013676 0.658953786

Zone 3: strong selection approximation

Lower and upper bounds of St popn gamma= 2976.19043 1875.00012

Zone 3: strong selection approximation

Probability of zone 3= -9.74750519E-03

Mean load statistics over zone 3

Contributions to loads within In and St= 1.66383252E-05 1.66019217E-05

Contribution to load between In and St= 1.65973652E-05

Contributions to homozygous loads for In and St= 2.37104996E-05 2.37104996E-05

Contributions to inbreeding loads= 7.07218487E-06 7.10858876E-06

Selection coefficients for In and St homokaryotypes

5.96046448E-08 0.00000000

Contributions to mean A2 freqs= 2.38932376E-08 2.38932358E-08

Contributions to mean A2 freqs at seg. sites= 0.00000000 0.00000000

Contributions to mean diversities= 4.75070685E-08 4.77553783E-08

Contributions to delta-theta values= 0.626562953 0.643254638

Mean load statistics over all zones

Loads within In and St= 0.465152681 1.89795494E-02

Load between In and St= 0.172806919

Homozygous load for In and St= 0.465434104 1.92863736E-02

Inbreeding loads= 2.81502609E-04 3.06815491E-04

Selection coefficients for In and St homokaryotypes

0.253489614 -0.166289568

Mean frequencies of A2 in In and St= 0.203784838 9.86339152E-02

Ratio of these= 2.06607270

Mean diversities at selected sites in In and St= 1.12531248E-04 5.41310117E-04

Mean diversities at neutral sites in In and St= 1.99899674E-04 1.79326057E-03

pi-n/pi-s for In and St= 0.562938631 0.301858038

Ratio of these= 1.86491179

Mean freqs. of seg. sites= 4.46406862E-04 2.22371891E-03

Ratio of these= 0.200747877

Overall delta-theta values= 0.105677724 0.136389315

Ratio of these= 0.774824083

**h = 0.45**

Zone 1: quasi-neutral zone

Upper bound scaled selection coefficient for neutrality in St metapopulation= 0.250000000

Probability of zone 1= 0.100879930

Integral of selection coefficient over zone 1= 2.58666510E-03

Mean load statistics for zone 1

Mean q1 and q2= 0.600000024

F1 and F2= 0.999583542 0.996264040

Diversities= 1.99899674E-04 1.79326057E-03

Contributions to loads within In and St= 1.55197317E-03 1.55176723E-03

Contribution to load between In and St = 1.48991903E-03

Contributions to homozygous loads for In and St= 1.55199913E-03 1.55199913E-03

Contributions to inbreeding loads for In and St= 4.25631879E-04 4.28244210E-04

Contributions to selection coefficients for In and St homokaryotypes

6.20484352E-05 6.18696213E-05

Contributions to mean A2 freqs= 6.05279617E-02 6.05279617E-02

Contributions to mean diversities= 2.01658659E-05 1.80903997E-04

Contributions to mean freqs. of seg. site= 7.16799259E-05 6.40928338E-04

Delta-theta values= 1.90687180E-03 -1.36041641E-03

Zone 2a: moderate selection; cut-off at moderate gamma for St population

Lower and upper bounds of St popn gamma

0.250000000 165.343918

Probability of zone 2a= 0.573140264

Coefficients for bivariate distribution of q1 and q2

a1= 4.49999981E-02 a2= 0.404999971

b11= 5.00000140E-04 b12= 9.00000241E-03 b22= 4.05000076E-02

Net probability of zone 2a using Simpsons rule= 0.573379099

Contributions to mean load statistics over zone 2a

Contributions to mean loads within In and St= 0.349641234 1.73235573E-02

Contribution to load between In and St= 0.166136622

Contributions to homozygous loads for In and St= 0.349680752 1.73769984E-02

Contributions to inbreeding loads= 3.92330767E-05 5.34577848E-05

Selection coefficients for In and St homokaryotypes

0.167651951 -0.160456061

Selection coefficients for In and St homokaryotypes

0.167651951 -0.160456061

Contributions to mean A2 freqs= 0.130339503 3.75857018E-02

Contributions to mean diversities= 8.09719422E-05 3.24275490E-04

Contributions to mean proportions of seg. sites= 3.15649289E-04 1.40810048E-03

Contributions to delta-theta values= 8.99159312E-02 0.182980835

Zone 2b: moderate selection; gamma for St reaches high value

Lower and upper bounds of St popn gamma

165.343918 1653.43921

Probability of zone 2b 0.308925688

Net probability of zone 2b using Simpsons rule= 0.308925897

Contributions to mean load statistics over zone 2b

Contributions to mean loads within In and St= 3.08757095E-04 2.26898177E-04

Contribution to load between In and St= 2.67274241E-04

Contributions to homozygous loads for In and St= 3.41939769E-04 2.52002123E-04

Contributions to inbreeding loads= 3.31828123E-05 2.51038746E-05

Selection coefficients for In and St homokaryotypes

4.14848328E-05 -4.04119492E-05

Selection coefficients for In and St homokaryotypes

4.14848328E-05 -4.04119492E-05

Contributions to mean A2 freqs= 2.14191232E-06 1.69821203E-06

Contributions to mean diversities= 4.11897372E-06 3.38068276E-06

Contributions to mean proportions of seg. sites= 3.26504050E-05 3.44649125E-05

Contributions to delta-theta values= 0.552439034 0.652000189

Zone 3: strong selection approximation

Lower and upper bounds of St popn gamma= 1653.43921 1875.00012

Zone 3: strong selection approximation

Probability of zone 3= 5.19704819E-03

Mean load statistics over zone 3

Contributions to loads within In and St= 4.40084987E-05 4.39837640E-05

Contribution to load between In and St= 4.39806754E-05

Contributions to homozygous loads for In and St= 4.88674086E-05 4.88674086E-05

Contributions to inbreeding loads= 4.85888677E-06 4.88363730E-06

Selection coefficients for In and St homokaryotypes

0.00000000 0.00000000

Contributions to mean A2 freqs= 6.26557721E-08 6.26557650E-08

Contributions to mean A2 freqs at seg. sites= 0.00000000 0.00000000

Contributions to mean diversities= 1.24596269E-07 1.25231864E-07

Contributions to delta-theta values= 0.627037764 0.643299639

Mean load statistics over all zones

Loads within In and St= 0.351545990 1.91462077E-02

Load between In and St= 0.167937785

Homozygous load for In and St= 0.351623565 1.92298666E-02

Inbreeding loads= 7.73006759E-05 8.36771796E-05

Selection coefficients for In and St homokaryotypes

0.167738199 -0.160431147

Mean frequencies of A2 in In and St= 0.190869674 9.81154218E-02

Ratio of these= 1.94535851

Mean diversities at selected sites in In and St= 1.05381368E-04 5.08685422E-04

Mean diversities at neutral sites in In and St= 1.99899674E-04 1.79326057E-03

pi-n/pi-s for In and St= 0.527171314 0.283665091

Ratio of these= 1.85842860

Mean freqs. of seg. sites= 4.21164790E-04 2.08473951E-03

Ratio of these= 0.202022746

Overall delta-theta values= 0.112305462 0.134336174

Ratio of these= 0.836003125

**h = 0.5**

Zone 1: quasi-neutral zone

Upper bound scaled selection coefficient for neutrality in St metapopulation= 0.250000000

Probability of zone 1= 0.100879930

Integral of selection coefficient over zone 1= 2.58666510E-03

Mean load statistics for zone 1

Mean q1 and q2= 0.600000024

F1 and F2= 0.999583542 0.996264040

Diversities= 1.99899674E-04 1.79326057E-03

Contributions to loads within In and St= 1.55199913E-03 1.55199913E-03

Contribution to load between In and St = 1.55199913E-03

Contributions to homozygous loads for In and St= 1.55199913E-03 1.55199913E-03

Contributions to inbreeding loads for In and St= 1.10518391E-04 1.11045141E-04

Contributions to selection coefficients for In and St homokaryotypes

0.00000000 0.00000000

Contributions to mean A2 freqs= 6.05279617E-02 6.05279617E-02

Contributions to mean diversities= 2.01658659E-05 1.80903997E-04

Contributions to mean freqs. of seg. site= 7.16799259E-05 6.40928338E-04

Delta-theta values= 1.90687180E-03 -1.36041641E-03

Zone 2a: moderate selection; cut-off at moderate gamma for St population

Lower and upper bounds of St popn gamma

0.250000000 82.6719589

Probability of zone 2a= 0.459932238

Coefficients for bivariate distribution of q1 and q2

a1= 5.00000007E-02 a2= 0.449999988

b11= 0.00000000 b12= 0.00000000 b22= 0.00000000

Net probability of zone 2a using Simpsons rule= 0.459957004

Contributions to mean load statistics over zone 2a

Contributions to mean loads within In and St= 0.309724003 1.72886197E-02

Contribution to load between In and St= 0.163506225

Contributions to homozygous loads for In and St= 0.309724003 1.72886197E-02

Contributions to inbreeding loads= 0.00000000 0.00000000

Selection coefficients for In and St homokaryotypes

0.136030495 -0.157448053

Selection coefficients for In and St homokaryotypes

0.136030495 -0.157448053

Contributions to mean A2 freqs= 0.125201151 3.74953635E-02

Contributions to mean diversities= 7.40946780E-05 3.07210226E-04

Contributions to mean proportions of seg. sites= 2.79676518E-04 1.31499022E-03

Contributions to delta-theta values= 6.00975752E-02 0.171171069

Zone 2b: moderate selection; gamma for St reaches high value

Lower and upper bounds of St popn gamma

82.6719589 826.719604

Probability of zone 2b 0.366410077

Net probability of zone 2b using Simpsons rule= 0.366409540

Contributions to mean load statistics over zone 2b

Contributions to mean loads within In and St= 4.74438770E-04 3.01808846E-04

Contribution to load between In and St= 3.88124026E-04

Contributions to homozygous loads for In and St= 4.74438770E-04 3.01808846E-04

Contributions to inbreeding loads= 0.00000000 0.00000000

Selection coefficients for In and St homokaryotypes

8.63075256E-05 -8.63075256E-05

Selection coefficients for In and St homokaryotypes

8.63075256E-05 -8.63075256E-05

Contributions to mean A2 freqs= 6.62027924E-06 3.51526955E-06

Contributions to mean diversities= 7.88483794E-06 6.97818132E-06

Contributions to mean proportions of seg. sites= 5.60613262E-05 6.82238970E-05

Contributions to delta-theta values= 0.501022220 0.637124658

Zone 3: strong selection approximation

Lower and upper bounds of St popn gamma= 826.719604 1875.00012

Zone 3: strong selection approximation

Probability of zone 3= 6.09206557E-02

Mean load statistics over zone 3

Contributions to loads within In and St= 1.08965563E-04 1.08965563E-04

Contribution to load between In and St= 1.08965563E-04

Contributions to homozygous loads for In and St= 1.08965563E-04 1.08965563E-04

Contributions to inbreeding loads= 0.00000000 0.00000000

Selection coefficients for In and St homokaryotypes

0.00000000 0.00000000

Contributions to mean A2 freqs= 2.17158515E-07 2.17158515E-07

Contributions to mean A2 freqs at seg. sites= 0.00000000 0.00000000

Contributions to mean diversities= 4.30693490E-07 4.33913755E-07

Contributions to delta-theta values= 0.617890894 0.642406702

Mean load statistics over all zones

Loads within In and St= 0.311859399 1.92513913E-02

Load between In and St= 0.165555328

Homozygous load for In and St= 0.311859399 1.92513913E-02

Inbreeding loads= 0.00000000 0.00000000

Selection coefficients for In and St homokaryotypes

0.136105001 -0.157547951

Mean frequencies of A2 in In and St= 0.185735956 9.80270579E-02

Ratio of these= 1.89474177

Mean diversities at selected sites in In and St= 1.02576072E-04 4.95526358E-04

Mean diversities at neutral sites in In and St= 1.99899674E-04 1.79326057E-03

pi-n/pi-s for In and St= 0.513137758 0.276327014

Ratio of these= 1.85699451

Mean freqs. of seg. sites= 4.11416608E-04 2.02844734E-03

Ratio of these= 0.202823415

Overall delta-theta values= 0.115463018 0.133328021

Ratio of these= 0.866007149

**Inversion frequency= 0.3**

**h = 0.05**

Zone 1: quasi-neutral zone

Upper bound scaled selection coefficient for neutrality in St metapopulation= 0.250000000

Probability of zone 1= 0.108777359

Integral of selection coefficient over zone 1= 3.58606712E-03

Mean load statistics for zone 1

Mean q1 and q2= 0.600000024

F1 and F2= 0.998751640 0.997091770

Diversities= 5.99212653E-04 1.39595033E-03

Contributions to loads within In and St= 2.15067342E-03 2.14938773E-03

Contribution to load between In and St = 1.37704983E-03

Contributions to homozygous loads for In and St= 2.15164036E-03 2.15164036E-03

Contributions to inbreeding loads for In and St= 0.00000000 0.00000000

Contributions to selection coefficients for In and St homokaryotypes

7.73310661E-04 7.72058964E-04

Contributions to mean A2 freqs= 6.52664155E-02 6.52664155E-02

Contributions to mean diversities= 6.51807713E-05 1.51847795E-04

Contributions to mean freqs. of seg. site= 2.41651593E-04 5.38310327E-04

Delta-theta values= 4.30669188E-02 -7.54356384E-04

Zone 2a: moderate selection; cut-off at moderate gamma for St population

Lower and upper bounds of St popn gamma

0.250000000 250.000000

Probability of zone 2a= 0.679293394

Coefficients for bivariate distribution of q1 and q2

a1= 1.50000006E-02 a2= 3.50000001E-02

b11= 4.05000001E-02 b12= 0.189000010 b22= 0.220499992

Net probability of zone 2a using Simpsons rule= 0.680126965

Contributions to mean load statistics over zone 2a

Contributions to mean loads within In and St= 0.264391929 2.45157685E-02

Contribution to load between In and St= 2.54442077E-02

Contributions to homozygous loads for In and St= 0.266678929 2.68637519E-02

Contributions to inbreeding loads= 2.28681299E-03 2.34800461E-03

Selection coefficients for In and St homokaryotypes

0.212543964 -9.28878784E-04

Selection coefficients for In and St homokaryotypes

0.212543964 -9.28878784E-04

Contributions to mean A2 freqs= 0.109768003 4.11757417E-02

Contributions to mean diversities= 3.03345063E-04 4.51172382E-04

Contributions to mean proportions of seg. sites= 1.19043759E-03 2.01926776E-03

Contributions to delta-theta values= 9.59715843E-02 0.207315564

Zone 2b: moderate selection; gamma for St reaches high value

Lower and upper bounds of St popn gamma

250.000000 2500.00000

Probability of zone 2b 0.210458338

Net probability of zone 2b using Simpsons rule= 0.210458338

Contributions to mean load statistics over zone 2b

Contributions to mean loads within In and St= 2.28054050E-04 1.74740286E-04

Contribution to load between In and St= 1.61975971E-04

Contributions to homozygous loads for In and St= 1.71038869E-03 1.52757473E-03

Contributions to inbreeding loads= 1.48233527E-03 1.35283312E-03

Selection coefficients for In and St homokaryotypes

6.61015511E-05 1.27553940E-05

Selection coefficients for In and St homokaryotypes

6.61015511E-05 1.27553940E-05

Contributions to mean A2 freqs= 6.31695320E-06 5.73082389E-06

Contributions to mean diversities= 1.20849463E-05 1.12489843E-05

Contributions to mean proportions of seg. sites= 9.17397556E-05 9.87569583E-05

Contributions to delta-theta values= 0.532653570 0.595892072

Zone 3: strong selection approximation

Lower and upper bounds of St popn gamma= 2500.00000 1458.33337

Zone 3: strong selection approximation

Probability of zone 3= -1.03861690E-02

Mean load statistics over zone 3

Contributions to loads within In and St= 1.62701654E-05 1.52825087E-05

Contribution to load between In and St= 1.45417516E-05

Contributions to homozygous loads for In and St= 1.45404556E-04 1.45404556E-04

Contributions to inbreeding loads= 1.29134438E-04 1.30122105E-04

Selection coefficients for In and St homokaryotypes

1.72853470E-06 7.15255737E-07

Contributions to mean A2 freqs= 1.44034360E-07 1.44034360E-07

Contributions to mean A2 freqs at seg. sites= 0.00000000 0.00000000

Contributions to mean diversities= 2.84187848E-07 2.86403775E-07

Contributions to delta-theta values= 0.598668337 0.626816869

Mean load statistics over all zones

Loads within In and St= 0.266786933 2.68551800E-02

Load between In and St= 2.69977748E-02

Homozygous load for In and St= 0.270686358 3.06883696E-02

Inbreeding loads= 3.89924971E-03 3.83321242E-03

Selection coefficients for In and St homokaryotypes

0.213206291 -1.42574310E-04

Mean frequencies of A2 in In and St= 0.175040886 0.106448025

Ratio of these= 1.64437890

Mean diversities at selected sites in In and St= 3.80894984E-04 6.14555553E-04

Mean diversities at neutral sites in In and St= 5.99212653E-04 1.39595033E-03

pi-n/pi-s for In and St= 0.635659099 0.440241694

Ratio of these= 1.44388664

Mean freqs. of seg. sites= 1.52634108E-03 2.65905773E-03

Ratio of these= 0.574015796

Overall delta-theta values= 0.114669502 0.180054247

Ratio of these= 0.636860847

h = 0.150000006

Zone 1: quasi-neutral zone

Upper bound scaled selection coefficient for neutrality in St metapopulation= 0.250000000

Probability of zone 1= 0.108777359

Integral of selection coefficient over zone 1= 3.58606712E-03

Mean load statistics for zone 1

Mean q1 and q2= 0.600000024

F1 and F2= 0.998751640 0.997091770

Diversities= 5.99212653E-04 1.39595033E-03

Contributions to loads within In and St= 2.15088809E-03 2.14988831E-03

Contribution to load between In and St = 1.54918106E-03

Contributions to homozygous loads for In and St= 2.15164036E-03 2.15164036E-03

Contributions to inbreeding loads for In and St= 8.85589141E-03 8.93817749E-03

Contributions to selection coefficients for In and St homokaryotypes

6.01530075E-04 6.00516796E-04

Contributions to mean A2 freqs= 6.52664155E-02 6.52664155E-02

Contributions to mean diversities= 6.51807713E-05 1.51847795E-04

Contributions to mean freqs. of seg. site= 2.41651593E-04 5.38310327E-04

Delta-theta values= 4.30669188E-02 -7.54356384E-04

Zone 2a: moderate selection; cut-off at moderate gamma for St population

Lower and upper bounds of St popn gamma

0.250000000 416.666656

Probability of zone 2a= 0.761075735

Coefficients for bivariate distribution of q1 and q2

a1= 4.50000018E-02 a2= 0.105000004

b11= 3.15000005E-02 b12= 0.147000000 b22= 0.171499997

Net probability of zone 2a using Simpsons rule= 0.764049768

Contributions to mean load statistics over zone 2a

Contributions to mean loads within In and St= 0.174094573 2.52640769E-02

Contribution to load between In and St= 3.84442396E-02

Contributions to homozygous loads for In and St= 0.175259575 2.64670774E-02

Contributions to inbreeding loads= 1.16505299E-03 1.20295689E-03

Selection coefficients for In and St homokaryotypes

0.126852095 -1.32673979E-02

Selection coefficients for In and St homokaryotypes

0.126852095 -1.32673979E-02

Contributions to mean A2 freqs= 9.61312875E-02 4.27468829E-02

Contributions to mean diversities= 2.43358852E-04 3.83673847E-04

Contributions to mean proportions of seg. sites= 9.98949166E-04 1.69183314E-03

Contributions to delta-theta values= 0.135717928 0.195443749

Zone 2b: moderate selection; gamma for St reaches high value

Lower and upper bounds of St popn gamma

416.666656 4166.66650

Probability of zone 2b 0.130073488

Net probability of zone 2b using Simpsons rule= 0.130073503

Contributions to mean load statistics over zone 2b

Contributions to mean loads within In and St= 1.17504118E-04 9.84035069E-05

Contribution to load between In and St= 1.05788778E-04

Contributions to homozygous loads for In and St= 3.81272082E-04 3.23963090E-04

Contributions to inbreeding loads= 2.63767666E-04 2.25559401E-04

Selection coefficients for In and St homokaryotypes

1.17421150E-05 -7.39097595E-06

Selection coefficients for In and St homokaryotypes

1.17421150E-05 -7.39097595E-06

Contributions to mean A2 freqs= 9.87149519E-07 8.57919304E-07

Contributions to mean diversities= 1.94836230E-06 1.70567591E-06

Contributions to mean proportions of seg. sites= 1.78689970E-05 1.68209499E-05

Contributions to delta-theta values= 0.613168955 0.640252531

Zone 3: strong selection approximation

Lower and upper bounds of St popn gamma= 4166.66650 1458.33337

Zone 3: strong selection approximation

Probability of zone 3= -1.17836595E-02

Mean load statistics over zone 3

Contributions to loads within In and St= 6.40734334E-06 6.37182711E-06

Contribution to load between In and St= 6.34518938E-06

Contributions to homozygous loads for In and St= 2.11504666E-05 2.11504666E-05

Contributions to inbreeding loads= 1.47431301E-05 1.47786404E-05

Selection coefficients for In and St homokaryotypes

5.96046448E-08 0.00000000

Contributions to mean A2 freqs= 1.99787085E-08 1.99787120E-08

Contributions to mean A2 freqs at seg. sites= 0.00000000 0.00000000

Contributions to mean diversities= 3.97833340E-08 3.98827318E-08

Contributions to delta-theta values= 0.631533742 0.639498949

Mean load statistics over all zones

Loads within In and St= 0.176369384 2.75187399E-02

Load between In and St= 4.01055515E-02

Homozygous load for In and St= 0.177813634 2.89638303E-02

Inbreeding loads= 1.44431600E-03 1.44504709E-03

Selection coefficients for In and St homokaryotypes

0.127387643 -1.26663446E-02

Mean frequencies of A2 in In and St= 0.161398694 0.108014181

Ratio of these= 1.49423611

Mean diversities at selected sites in In and St= 3.10527772E-04 5.37267188E-04

Mean diversities at neutral sites in In and St= 5.99212653E-04 1.39595033E-03

pi-n/pi-s for In and St= 0.518226326 0.384875566

Ratio of these= 1.34647763

Mean freqs. of seg. sites= 1.25885278E-03 2.24735681E-03

Ratio of these= 0.560148180

Overall delta-theta values= 0.124860525 0.151855111

Ratio of these= 0.822234571

**h = 0.25**

Zone 1: quasi-neutral zone

Upper bound scaled selection coefficient for neutrality in St metapopulation= 0.250000000

Probability of zone 1= 0.108777359

Integral of selection coefficient over zone 1= 3.58606712E-03

Mean load statistics for zone 1

Mean q1 and q2= 0.600000024

F1 and F2= 0.998751640 0.997091770

Diversities= 5.99212653E-04 1.39595033E-03

Contributions to loads within In and St= 2.15110322E-03 2.15038890E-03

Contribution to load between In and St = 1.72131229E-03

Contributions to homozygous loads for In and St= 2.15164036E-03 2.15164036E-03

Contributions to inbreeding loads for In and St= 2.32087937E-03 2.32799049E-03

Contributions to selection coefficients for In and St homokaryotypes

4.29689884E-04 4.28974628E-04

Contributions to mean A2 freqs= 6.52664155E-02 6.52664155E-02

Contributions to mean diversities= 6.51807713E-05 1.51847795E-04

Contributions to mean freqs. of seg. site= 2.41651593E-04 5.38310327E-04

Delta-theta values= 4.30669188E-02 -7.54356384E-04

Zone 2a: moderate selection; cut-off at moderate gamma for St population

Lower and upper bounds of St popn gamma

0.250000000 416.666656

Probability of zone 2a= 0.761075735

Coefficients for bivariate distribution of q1 and q2

a1= 7.50000030E-02 a2= 0.174999997

b11= 2.25000009E-02 b12= 0.105000004 b22= 0.122499995

Net probability of zone 2a using Simpsons rule= 0.764049768

Contributions to mean load statistics over zone 2a

Contributions to mean loads within In and St= 0.127813712 2.59656906E-02

Contribution to load between In and St= 4.47498225E-02

Contributions to homozygous loads for In and St= 0.128377885 2.65511349E-02

Contributions to inbreeding loads= 5.64200804E-04 5.85462432E-04

Selection coefficients for In and St homokaryotypes

7.97076225E-02 -1.89616680E-02

Selection coefficients for In and St homokaryotypes

7.97076225E-02 -1.89616680E-02

Contributions to mean A2 freqs= 8.64331722E-02 4.31174971E-02

Contributions to mean diversities= 2.07903096E-04 3.34886048E-04

Contributions to mean proportions of seg. sites= 8.69002717E-04 1.47421996E-03

Contributions to delta-theta values= 0.151226997 0.194090068

Zone 2b: moderate selection; gamma for St reaches high value

Lower and upper bounds of St popn gamma

416.666656 4166.66650

Probability of zone 2b 0.130073488

Net probability of zone 2b using Simpsons rule= 0.130073503

Contributions to mean load statistics over zone 2b

Contributions to mean loads within In and St= 1.12540809E-04 9.15536002E-05

Contribution to load between In and St= 1.01474696E-04

Contributions to homozygous loads for In and St= 2.23431562E-04 1.82463482E-04

Contributions to inbreeding loads= 1.10890745E-04 9.09099399E-05

Selection coefficients for In and St homokaryotypes

1.10864639E-05 -9.89437103E-06

Selection coefficients for In and St homokaryotypes

1.10864639E-05 -9.89437103E-06

Contributions to mean A2 freqs= 5.82039092E-07 4.89605156E-07

Contributions to mean diversities= 1.15446335E-06 9.75425451E-07

Contributions to mean proportions of seg. sites= 1.10727296E-05 1.00216266E-05

Contributions to delta-theta values= 0.630106091 0.654691219

Zone 3: strong selection approximation

Lower and upper bounds of St popn gamma= 4166.66650 1458.33337

Zone 3: strong selection approximation

Probability of zone 3= -1.17836595E-02

Mean load statistics over zone 3

Contributions to loads within In and St= 6.36113555E-06 6.35200513E-06

Contribution to load between In and St= 6.34515345E-06

Contributions to homozygous loads for In and St= 1.26902823E-05 1.26902823E-05

Contributions to inbreeding loads= 6.32914771E-06 6.33827995E-06

Selection coefficients for In and St homokaryotypes

0.00000000 0.00000000

Contributions to mean A2 freqs= 1.19872290E-08 1.19872290E-08

Contributions to mean A2 freqs at seg. sites= 0.00000000 0.00000000

Contributions to mean diversities= 2.39117828E-08 2.39475657E-08

Contributions to delta-theta values= 0.637165070 0.641805530

Mean load statistics over all zones

Loads within In and St= 0.130083710 2.82139834E-02

Load between In and St= 4.65789512E-02

Homozygous load for In and St= 0.130765647 2.88979281E-02

Inbreeding loads= 6.81957812E-04 6.83962135E-04

Selection coefficients for In and St homokaryotypes

8.01132917E-02 -1.85346603E-02

Mean frequencies of A2 in In and St= 0.151700184 0.108384416

Ratio of these= 1.39964938

Mean diversities at selected sites in In and St= 2.74262246E-04 4.87733225E-04

Mean diversities at neutral sites in In and St= 5.99212653E-04 1.39595033E-03

pi-n/pi-s for In and St= 0.457704365 0.349391520

Ratio of these= 1.31000423

Mean freqs. of seg. sites= 1.12196081E-03 2.02278933E-03

Ratio of these= 0.554660261

Overall delta-theta values= 0.132758379 0.144572079

Ratio of these= 0.918285072

**h = 0.35**

Zone 1: quasi-neutral zone

Upper bound scaled selection coefficient for neutrality in St metapopulation= 0.250000000

Probability of zone 1= 0.108777359

Integral of selection coefficient over zone 1= 3.58606712E-03

Mean load statistics for zone 1

Mean q1 and q2= 0.600000024

F1 and F2= 0.998751640 0.997091770

Diversities= 5.99212653E-04 1.39595033E-03

Contributions to loads within In and St= 2.15131813E-03 2.15088949E-03

Contribution to load between In and St = 1.89344340E-03

Contributions to homozygous loads for In and St= 2.15164036E-03 2.15164036E-03

Contributions to inbreeding loads for In and St= 9.96797578E-04 9.98626114E-04

Contributions to selection coefficients for In and St homokaryotypes

2.57849693E-04 2.57432461E-04

Contributions to mean A2 freqs= 6.52664155E-02 6.52664155E-02

Contributions to mean diversities= 6.51807713E-05 1.51847795E-04

Contributions to mean freqs. of seg. site= 2.41651593E-04 5.38310327E-04

Delta-theta values= 4.30669188E-02 -7.54356384E-04

Zone 2a: moderate selection; cut-off at moderate gamma for St population

Lower and upper bounds of St popn gamma

0.250000000 297.619049

Probability of zone 2a= 0.708344936

Coefficients for bivariate distribution of q1 and q2

a1= 0.105000004 a2= 0.244999990

b11= 1.35000013E-02 b12= 6.30000010E-02 b22= 7.34999999E-02

Net probability of zone 2a using Simpsons rule= 0.709659874

Contributions to mean load statistics over zone 2a

Contributions to mean loads within In and St= 0.100698262 2.62803305E-02

Contribution to load between In and St= 4.84725051E-02

Contributions to homozygous loads for In and St= 0.100933731 2.65275668E-02

Contributions to inbreeding loads= 2.35382657E-04 2.47246498E-04

Selection coefficients for In and St homokaryotypes

5.08854389E-02 -2.24401951E-02

Selection coefficients for In and St homokaryotypes

5.08854389E-02 -2.24401951E-02

Contributions to mean A2 freqs= 7.88120627E-02 4.24805842E-02

Contributions to mean diversities= 1.83617463E-04 2.97979946E-04

Contributions to mean proportions of seg. sites= 7.73107808E-04 1.31101441E-03

Contributions to delta-theta values= 0.157391787 0.193635643

Zone 2b: moderate selection; gamma for St reaches high value

Lower and upper bounds of St popn gamma

297.619049 2976.19043

Probability of zone 2b 0.182284474

Net probability of zone 2b using Simpsons rule= 0.182284817

Contributions to mean load statistics over zone 2b

Contributions to mean loads within In and St= 1.57729504E-04 1.28791318E-04

Contribution to load between In and St= 1.42936464E-04

Contributions to homozygous loads for In and St= 2.24664036E-04 1.83724551E-04

Contributions to inbreeding loads= 6.69344154E-05 5.49332435E-05

Selection coefficients for In and St homokaryotypes

1.47819519E-05 -1.41859055E-05

Selection coefficients for In and St homokaryotypes

1.47819519E-05 -1.41859055E-05

Contributions to mean A2 freqs= 7.57092096E-07 6.43244050E-07

Contributions to mean diversities= 1.50213020E-06 1.28166255E-06

Contributions to mean proportions of seg. sites= 1.44768119E-05 1.32601699E-05

Contributions to delta-theta values= 0.631882548 0.657093048

Zone 3: strong selection approximation

Lower and upper bounds of St popn gamma= 2976.19043 1458.33337

Zone 3: strong selection approximation

Probability of zone 3= -1.12638474E-02

Mean load statistics over zone 3

Contributions to loads within In and St= 1.08309860E-05 1.08261229E-05

Contribution to load between In and St= 1.08224731E-05

Contributions to homozygous loads for In and St= 1.54606660E-05 1.54606660E-05

Contributions to inbreeding loads= 4.62967682E-06 4.63454489E-06

Selection coefficients for In and St homokaryotypes

0.00000000 0.00000000

Contributions to mean A2 freqs= 1.49037565E-08 1.49037565E-08

Contributions to mean A2 freqs at seg. sites= 0.00000000 0.00000000

Contributions to mean diversities= 2.97512130E-08 2.97833651E-08

Contributions to delta-theta values= 0.639431238 0.642770708

Mean load statistics over all zones

Loads within In and St= 0.103018142 2.85708364E-02

Load between In and St= 5.05197048E-02

Homozygous load for In and St= 0.103325494 2.88783908E-02

Inbreeding loads= 3.07269045E-04 3.07565177E-04

Selection coefficients for In and St homokaryotypes

5.11441827E-02 -2.21915245E-02

Mean frequencies of A2 in In and St= 0.144079253 0.107747659

Ratio of these= 1.33719146

Mean diversities at selected sites in In and St= 2.50330108E-04 4.51139204E-04

Mean diversities at neutral sites in In and St= 5.99212653E-04 1.39595033E-03

pi-n/pi-s for In and St= 0.417765051 0.323177129

Ratio of these= 1.29268134

Mean freqs. of seg. sites= 1.02952903E-03 1.86288066E-03

Ratio of these= 0.552654326

Overall delta-theta values= 0.137366593 0.140833616

Ratio of these= 0.975382149

**h = 0.45**

Zone 1: quasi-neutral zone

Upper bound scaled selection coefficient for neutrality in St metapopulation= 0.250000000

Probability of zone 1= 0.108777359

Integral of selection coefficient over zone 1= 3.58606712E-03

Mean load statistics for zone 1

Mean q1 and q2= 0.600000024

F1 and F2= 0.998751640 0.997091770

Diversities= 5.99212653E-04 1.39595033E-03

Contributions to loads within In and St= 2.15153280E-03 2.15139007E-03

Contribution to load between In and St = 2.06557452E-03

Contributions to homozygous loads for In and St= 2.15164036E-03 2.15164036E-03

Contributions to inbreeding loads for In and St= 4.27591091E-04 4.28150903E-04

Contributions to selection coefficients for In and St homokaryotypes

8.59498978E-05 8.58306885E-05

Contributions to mean A2 freqs= 6.52664155E-02 6.52664155E-02

Contributions to mean diversities= 6.51807713E-05 1.51847795E-04

Contributions to mean freqs. of seg. site= 2.41651593E-04 5.38310327E-04

Delta-theta values= 4.30669188E-02 -7.54356384E-04

Zone 2a: moderate selection; cut-off at moderate gamma for St population

Lower and upper bounds of St popn gamma

0.250000000 165.343918

Probability of zone 2a= 0.608378649

Coefficients for bivariate distribution of q1 and q2

a1= 0.135000005 a2= 0.314999998

b11= 4.50000120E-03 b12= 2.10000053E-02 b22= 2.45000049E-02

Net probability of zone 2a using Simpsons rule= 0.608636439

Contributions to mean load statistics over zone 2a

Contributions to mean loads within In and St= 8.31846222E-02 2.64056921E-02

Contribution to load between In and St= 5.07384874E-02

Contributions to homozygous loads for In and St= 8.32376406E-02 2.64624674E-02

Contributions to inbreeding loads= 5.28902347E-05 5.67925927E-05

Selection coefficients for In and St homokaryotypes

3.19253802E-02 -2.46312618E-02

Selection coefficients for In and St homokaryotypes

3.19253802E-02 -2.46312618E-02

Contributions to mean A2 freqs= 7.33873025E-02 4.22447287E-02

Contributions to mean diversities= 1.65230944E-04 2.69381533E-04

Contributions to mean proportions of seg. sites= 6.93717564E-04 1.17968267E-03

Contributions to delta-theta values= 0.154992759 0.189870596

Zone 2b: moderate selection; gamma for St reaches high value

Lower and upper bounds of St popn gamma

165.343918 1653.43921

Probability of zone 2b 0.274924934

Net probability of zone 2b using Simpsons rule= 0.274924815

Contributions to mean load statistics over zone 2b

Contributions to mean loads within In and St= 2.44795694E-04 2.06739656E-04

Contribution to load between In and St= 2.25609736E-04

Contributions to homozygous loads for In and St= 2.71744822E-04 2.29609825E-04

Contributions to inbreeding loads= 2.69490174E-05 2.28697627E-05

Selection coefficients for In and St homokaryotypes

1.91926956E-05 -1.88350677E-05

Selection coefficients for In and St homokaryotypes

1.91926956E-05 -1.88350677E-05

Contributions to mean A2 freqs= 1.42575402E-06 1.25795748E-06

Contributions to mean diversities= 2.82232645E-06 2.50401149E-06

Contributions to mean proportions of seg. sites= 2.66467614E-05 2.54156021E-05

Contributions to delta-theta values= 0.624236524 0.650467396

Zone 3: strong selection approximation

Lower and upper bounds of St popn gamma= 1653.43921 1458.33337

Zone 3: strong selection approximation

Probability of zone 3= -3.93801928E-03

Mean load statistics over zone 3

Contributions to loads within In and St= 2.94396377E-05 2.94364927E-05

Contribution to load between In and St= 2.94341316E-05

Contributions to homozygous loads for In and St= 3.27045782E-05 3.27045782E-05

Contributions to inbreeding loads= 3.26497570E-06 3.26810800E-06

Selection coefficients for In and St homokaryotypes

0.00000000 0.00000000

Contributions to mean A2 freqs= 3.69957860E-08 3.69957860E-08

Contributions to mean A2 freqs at seg. sites= 0.00000000 0.00000000

Contributions to mean diversities= 7.38673194E-08 7.39382600E-08

Contributions to delta-theta values= 0.640083671 0.643043876

Mean load statistics over all zones

Loads within In and St= 8.56103897E-02 2.87932605E-02

Load between In and St= 5.30591048E-02

Homozygous load for In and St= 8.56937319E-02 2.88764220E-02

Inbreeding loads= 8.32117439E-05 8.31807556E-05

Selection coefficients for In and St homokaryotypes

3.20271850E-02 -2.45625973E-02

Mean frequencies of A2 in In and St= 0.138655186 0.107512444

Ratio of these= 1.28966641

Mean diversities at selected sites in In and St= 2.33307903E-04 4.23807272E-04

Mean diversities at neutral sites in In and St= 5.99212653E-04 1.39595033E-03

pi-n/pi-s for In and St= 0.389357448 0.303597689

Ratio of these= 1.28247833

Mean freqs. of seg. sites= 9.62744060E-04 1.74414343E-03

Ratio of these= 0.551986754

Overall delta-theta values= 0.140253603 0.137939095

Ratio of these= 1.01677918

**h = 0.5**

Zone 1: quasi-neutral zone

Upper bound scaled selection coefficient for neutrality in St metapopulation= 0.250000000

Probability of zone 1= 0.108777359

Integral of selection coefficient over zone 1= 3.58606712E-03

Mean load statistics for zone 1

Mean q1 and q2= 0.600000024

F1 and F2= 0.998751640 0.997091770

Diversities= 5.99212653E-04 1.39595033E-03

Contributions to loads within In and St= 2.15164036E-03 2.15164036E-03

Contribution to load between In and St = 2.15164036E-03

Contributions to homozygous loads for In and St= 2.15164036E-03 2.15164036E-03

Contributions to inbreeding loads for In and St= 1.10913454E-04 1.11026289E-04

Contributions to selection coefficients for In and St homokaryotypes

0.00000000 0.00000000

Contributions to mean A2 freqs= 6.52664155E-02 6.52664155E-02

Contributions to mean diversities= 6.51807713E-05 1.51847795E-04

Contributions to mean freqs. of seg. site= 2.41651593E-04 5.38310327E-04

Delta-theta values= 4.30669188E-02 -7.54356384E-04

Zone 2a: moderate selection; cut-off at moderate gamma for St population

Lower and upper bounds of St popn gamma

0.250000000 82.6719589

Probability of zone 2a= 0.491752505

Coefficients for bivariate distribution of q1 and q2

a1= 0.150000006 a2= 0.349999994

b11= 0.00000000 b12= 0.00000000 b22= 0.00000000

Net probability of zone 2a using Simpsons rule= 0.491779685

Contributions to mean load statistics over zone 2a

Contributions to mean loads within In and St= 7.65905380E-02 2.63900850E-02

Contribution to load between In and St= 5.14902882E-02

Contributions to homozygous loads for In and St= 7.65905380E-02 2.63900850E-02

Contributions to inbreeding loads= 0.00000000 0.00000000

Selection coefficients for In and St homokaryotypes

2.47878432E-02 -2.54179239E-02

Selection coefficients for In and St homokaryotypes

2.47878432E-02 -2.54179239E-02

Contributions to mean A2 freqs= 7.13353604E-02 4.23287004E-02

Contributions to mean diversities= 1.55260423E-04 2.55187566E-04

Contributions to mean proportions of seg. sites= 6.39605860E-04 1.10258954E-03

Contributions to delta-theta values= 0.138807833 0.178897440

Zone 2b: moderate selection; gamma for St reaches high value

Lower and upper bounds of St popn gamma

82.6719589 826.719604

Probability of zone 2b 0.352405608

Net probability of zone 2b using Simpsons rule= 0.352405638

Contributions to mean load statistics over zone 2b

Contributions to mean loads within In and St= 3.29279806E-04 2.93090969E-04

Contribution to load between In and St= 3.11185111E-04

Contributions to homozygous loads for In and St= 3.29279806E-04 2.93090969E-04

Contributions to inbreeding loads= 0.00000000 0.00000000

Selection coefficients for In and St homokaryotypes

1.81198120E-05 -1.81198120E-05

Selection coefficients for In and St homokaryotypes

1.81198120E-05 -1.81198120E-05

Contributions to mean A2 freqs= 2.98583268E-06 2.74457830E-06

Contributions to mean diversities= 5.87054819E-06 5.44763498E-06

Contributions to mean proportions of seg. sites= 5.26488257E-05 5.31056321E-05

Contributions to delta-theta values= 0.604413271 0.636068940

Zone 3: strong selection approximation

Lower and upper bounds of St popn gamma= 826.719604 1458.33337

Zone 3: strong selection approximation

Probability of zone 3= 3.52074504E-02

Mean load statistics over zone 3

Contributions to loads within In and St= 8.12900835E-05 8.12900835E-05

Contribution to load between In and St= 8.12900835E-05

Contributions to homozygous loads for In and St= 8.12900835E-05 8.12900835E-05

Contributions to inbreeding loads= 0.00000000 0.00000000

Selection coefficients for In and St homokaryotypes

0.00000000 0.00000000

Contributions to mean A2 freqs= 1.36733803E-07 1.36733803E-07

Contributions to mean A2 freqs at seg. sites= 0.00000000 0.00000000

Contributions to mean diversities= 2.72839344E-07 2.73198083E-07

Contributions to delta-theta values= 0.638140142 0.642230213

Mean load statistics over all zones

Loads within In and St= 7.91527480E-02 2.89161056E-02

Load between In and St= 5.40344045E-02

Homozygous load for In and St= 7.91527480E-02 2.89161056E-02

Inbreeding loads= 0.00000000 0.00000000

Selection coefficients for In and St homokaryotypes

2.48054862E-02 -2.54364014E-02

Mean frequencies of A2 in In and St= 0.136604890 0.107597992

Ratio of these= 1.26958585

Mean diversities at selected sites in In and St= 2.26584583E-04 4.12756170E-04

Mean diversities at neutral sites in In and St= 5.99212653E-04 1.39595033E-03

pi-n/pi-s for In and St= 0.378137171 0.295681119

Ratio of these= 1.27886820

Mean freqs. of seg. sites= 9.36581288E-04 1.69671455E-03

Ratio of these= 0.551996946

Overall delta-theta values= 0.141704917 0.136948884

Ratio of these= 1.03472853

**Inversion frequency= 0.5**

**h= 0.05**

Zone 1: quasi-neutral zone

Upper bound scaled selection coefficient for neutrality in St metapopulation= 0.250000000

Probability of zone 1= 0.120326050

Integral of selection coefficient over zone 1= 5.55351051E-03

Mean load statistics for zone 1

Mean q1 and q2= 0.600000024

F1 and F2= 0.997920930 0.997920930

Diversities= 9.97953350E-04 9.97953350E-04

Contributions to loads within In and St= 3.32961255E-03 3.32961255E-03

Contribution to load between In and St = 2.13254802E-03

Contributions to homozygous loads for In and St= 3.33210640E-03 3.33210640E-03

Contributions to inbreeding loads for In and St= 0.00000000 0.00000000

Contributions to selection coefficients for In and St homokaryotypes

1.19632483E-03 1.19632483E-03

Contributions to mean A2 freqs= 7.21956342E-02 7.21956342E-02

Contributions to mean diversities= 1.20079785E-04 1.20079785E-04

Contributions to mean freqs. of seg. site= 4.18966229E-04 4.18966229E-04

Delta-theta values= -1.68166161E-02 -1.68166161E-02

Zone 2a: moderate selection; cut-off at moderate gamma for St population

Lower and upper bounds of St popn gamma

0.250000000 250.000000

Probability of zone 2a= 0.722875178

Coefficients for bivariate distribution of q1 and q2

a1= 2.50000004E-02 a2= 2.50000004E-02

b11= 0.112499997 b12= 0.224999994 b22= 0.112499997

Net probability of zone 2a using Simpsons rule= 0.723797858

Contributions to mean load statistics over zone 2a

Contributions to mean loads within In and St= 6.38508424E-02 6.38503805E-02

Contribution to load between In and St= 1.97292771E-02

Contributions to homozygous loads for In and St= 6.65963218E-02 6.65958449E-02

Contributions to inbreeding loads= 2.74546794E-03 2.74546887E-03

Selection coefficients for In and St homokaryotypes

4.31623459E-02 4.31619287E-02

Selection coefficients for In and St homokaryotypes

4.31623459E-02 4.31619287E-02

Contributions to mean A2 freqs= 5.50820865E-02 5.50818630E-02

Contributions to mean diversities= 3.97636992E-04 3.97639698E-04

Contributions to mean proportions of seg. sites= 1.68110838E-03 1.68110896E-03

Contributions to delta-theta values= 0.160843790 0.160838366

Zone 2b: moderate selection; gamma for St reaches high value

Lower and upper bounds of St popn gamma

250.000000 2500.00000

Probability of zone 2b 0.156572700

Net probability of zone 2b using Simpsons rule= 0.156572402

Contributions to mean load statistics over zone 2b

Contributions to mean loads within In and St= 1.39256750E-04 1.39256750E-04

Contribution to load between In and St= 1.19543292E-04

Contributions to homozygous loads for In and St= 1.19497871E-03 1.19497883E-03

Contributions to inbreeding loads= 1.05572166E-03 1.05572154E-03

Selection coefficients for In and St homokaryotypes

1.97291374E-05 1.97291374E-05

Selection coefficients for In and St homokaryotypes

1.97291374E-05 1.97291374E-05

Contributions to mean A2 freqs= 3.48870003E-06 3.48870049E-06

Contributions to mean diversities= 6.83267763E-06 6.83267763E-06

Contributions to mean proportions of seg. sites= 5.90867967E-05 5.90870477E-05

Contributions to delta-theta values= 0.589746594 0.589748323

Zone 3: strong selection approximation

Lower and upper bounds of St popn gamma= 2500.00000 1041.66675

Zone 3: strong selection approximation

Probability of zone 3= -1.16310120E-02

Mean load statistics over zone 3

Contributions to loads within In and St= 8.87721853E-06 8.87721853E-06

Contribution to load between In and St= 8.30843146E-06

Contributions to homozygous loads for In and St= 8.30772551E-05 8.30772551E-05

Contributions to inbreeding loads= 7.41999829E-05 7.41999829E-05

Selection coefficients for In and St homokaryotypes

5.96046448E-07 5.96046448E-07

Contributions to mean A2 freqs= 7.89978500E-08 7.89978500E-08

Contributions to mean A2 freqs at seg. sites= 0.00000000 0.00000000

Contributions to mean diversities= 1.56751923E-07 1.56751923E-07

Contributions to delta-theta values= 0.619603455 0.619603455

Mean load statistics over all zones

Loads within In and St= 6.73285872E-02 6.73281252E-02

Load between In and St= 2.19896771E-02

Homozygous load for In and St= 7.12064803E-02 7.12060034E-02

Inbreeding loads= 3.87788331E-03 3.87788424E-03

Selection coefficients for In and St homokaryotypes

4.43264842E-02 4.43260074E-02

Mean frequencies of A2 in In and St= 0.127281278 0.127281055

Ratio of these= 1.00000179

Mean diversities at selected sites in In and St= 5.24706207E-04 5.24708943E-04

Mean diversities at neutral sites in In and St= 9.97953350E-04 9.97953350E-04

pi-n/pi-s for In and St= 0.525782287 0.525785029

Ratio of these= 0.999994814

Mean freqs. of seg. sites= 2.16062344E-03 2.16062414E-03

Ratio of these= 0.999999702

Overall delta-theta values= 0.138433337 0.138429165

Ratio of these= 1.00003016

**h = 0.15**

Zone 1: quasi-neutral zone

Upper bound scaled selection coefficient for neutrality in St metapopulation= 0.250000000

Probability of zone 1= 0.120326050

Integral of selection coefficient over zone 1= 5.55351051E-03

Mean load statistics for zone 1

Mean q1 and q2= 0.600000024

F1 and F2= 0.997920930 0.997920930

Diversities= 9.97953350E-04 9.97953350E-04

Contributions to loads within In and St= 3.33016668E-03 3.33016668E-03

Contribution to load between In and St = 2.39911652E-03

Contributions to homozygous loads for In and St= 3.33210640E-03 3.33210640E-03

Contributions to inbreeding loads for In and St= 8.91349092E-03 8.91349092E-03

Contributions to selection coefficients for In and St homokaryotypes

9.30607319E-04 9.30607319E-04

Contributions to mean A2 freqs= 7.21956342E-02 7.21956342E-02

Contributions to mean diversities= 1.20079785E-04 1.20079785E-04

Contributions to mean freqs. of seg. site= 4.18966229E-04 4.18966229E-04

Delta-theta values= -1.68166161E-02 -1.68166161E-02

Zone 2a: moderate selection; cut-off at moderate gamma for St population

Lower and upper bounds of St popn gamma

0.250000000 416.666656

Probability of zone 2a= 0.795337081

Coefficients for bivariate distribution of q1 and q2

a1= 7.50000030E-02 a2= 7.50000030E-02

b11= 8.74999985E-02 b12= 0.174999997 b22= 8.74999985E-02

Net probability of zone 2a using Simpsons rule= 0.798627436

Contributions to mean load statistics over zone 2a

Contributions to mean loads within In and St= 5.66652715E-02 5.66652305E-02

Contribution to load between In and St= 2.67970040E-02

Contributions to homozygous loads for In and St= 5.79615496E-02 5.79615198E-02

Contributions to inbreeding loads= 1.29623676E-03 1.29623769E-03

Selection coefficients for In and St homokaryotypes

2.94266343E-02 2.94265747E-02

Selection coefficients for In and St homokaryotypes

2.94266343E-02 2.94265747E-02

Contributions to mean A2 freqs= 5.43600731E-02 5.43600582E-02

Contributions to mean diversities= 3.17258324E-04 3.17258789E-04

Contributions to mean proportions of seg. sites= 1.36276486E-03 1.36276253E-03

Contributions to delta-theta values= 0.174068809 0.174066246

Zone 2b: moderate selection; gamma for St reaches high value

Lower and upper bounds of St popn gamma

416.666656 4166.66650

Probability of zone 2b 8.43225121E-02

Net probability of zone 2b using Simpsons rule= 8.43220130E-02

Contributions to mean load statistics over zone 2b

Contributions to mean loads within In and St= 6.60116857E-05 6.60116857E-05

Contribution to load between In and St= 6.51410883E-05

Contributions to homozygous loads for In and St= 2.17131615E-04 2.17131615E-04

Contributions to inbreeding loads= 1.51119879E-04 1.51119879E-04

Selection coefficients for In and St homokaryotypes

8.94069672E-07 8.94069672E-07

Selection coefficients for In and St homokaryotypes

8.94069672E-07 8.94069672E-07

Contributions to mean A2 freqs= 4.39397809E-07 4.39397837E-07

Contributions to mean diversities= 8.73340696E-07 8.73340639E-07

Contributions to mean proportions of seg. sites= 8.56972929E-06 8.56953739E-06

Contributions to delta-theta values= 0.638450027 0.638441920

Zone 3: strong selection approximation

Lower and upper bounds of St popn gamma= 4166.66650 1041.66675

Zone 3: strong selection approximation

Probability of zone 3= -1.18427277E-02

Mean load statistics over zone 3

Contributions to loads within In and St= 3.97060694E-06 3.97060694E-06

Contribution to load between In and St= 3.94747485E-06

Contributions to homozygous loads for In and St= 1.31581555E-05 1.31581555E-05

Contributions to inbreeding loads= 9.18754449E-06 9.18754449E-06

Selection coefficients for In and St homokaryotypes

0.00000000 0.00000000

Contributions to mean A2 freqs= 1.23925341E-08 1.23925341E-08

Contributions to mean A2 freqs at seg. sites= 0.00000000 0.00000000

Contributions to mean diversities= 2.47203289E-08 2.47203289E-08

Contributions to delta-theta values= 0.637155771 0.637155771

Mean load statistics over all zones

Loads within In and St= 6.00654222E-02 6.00653812E-02

Load between In and St= 2.92652082E-02

Homozygous load for In and St= 6.15239441E-02 6.15239143E-02

Inbreeding loads= 1.45848386E-03 1.45848480E-03

Selection coefficients for In and St homokaryotypes

3.03307176E-02 3.03306580E-02

Mean frequencies of A2 in In and St= 0.126556158 0.126556143

Ratio of these= 1.00000012

Mean diversities at selected sites in In and St= 4.38236166E-04 4.38236631E-04

Mean diversities at neutral sites in In and St= 9.97953350E-04 9.97953350E-04

pi-n/pi-s for In and St= 0.439134926 0.439135402

Ratio of these= 0.999998927

Mean freqs. of seg. sites= 1.79054250E-03 1.79054006E-03

Ratio of these= 1.00000131

Overall delta-theta values= 0.131689012 0.131686866

Ratio of these= 1.00001633

**h = 0.25**

Zone 1: quasi-neutral zone

Upper bound scaled selection coefficient for neutrality in St metapopulation= 0.250000000

Probability of zone 1= 0.120326050

Integral of selection coefficient over zone 1= 5.55351051E-03

Mean load statistics for zone 1

Mean q1 and q2= 0.600000024

F1 and F2= 0.997920930 0.997920930

Diversities= 9.97953350E-04 9.97953350E-04

Contributions to loads within In and St= 3.33072082E-03 3.33072082E-03

Contribution to load between In and St = 2.66568526E-03

Contributions to homozygous loads for In and St= 3.33210640E-03 3.33210640E-03

Contributions to inbreeding loads for In and St= 2.32585706E-03 2.32585706E-03

Contributions to selection coefficients for In and St homokaryotypes

6.64830208E-04 6.64830208E-04

Contributions to mean A2 freqs= 7.21956342E-02 7.21956342E-02

Contributions to mean diversities= 1.20079785E-04 1.20079785E-04

Contributions to mean freqs. of seg. site= 4.18966229E-04 4.18966229E-04

Delta-theta values= -1.68166161E-02 -1.68166161E-02

Zone 2a: moderate selection; cut-off at moderate gamma for St population

Lower and upper bounds of St popn gamma

0.250000000 416.666656

Probability of zone 2a= 0.795337081

Coefficients for bivariate distribution of q1 and q2

a1= 0.125000000 a2= 0.125000000

b11= 6.25000000E-02 b12= 0.125000000 b22= 6.25000000E-02

Net probability of zone 2a using Simpsons rule= 0.798627436

Contributions to mean load statistics over zone 2a

Contributions to mean loads within In and St= 5.02250791E-02 5.02250791E-02

Contribution to load between In and St= 3.22502442E-02

Contributions to homozygous loads for In and St= 5.08466028E-02 5.08466028E-02

Contributions to inbreeding loads= 6.21443905E-04 6.21443905E-04

Selection coefficients for In and St homokaryotypes

1.78142786E-02 1.78142786E-02

Selection coefficients for In and St homokaryotypes

1.78142786E-02 1.78142786E-02

Contributions to mean A2 freqs= 5.24757765E-02 5.24757765E-02

Contributions to mean diversities= 2.69928860E-04 2.69928831E-04

Contributions to mean proportions of seg. sites= 1.17209356E-03 1.17209356E-03

Contributions to delta-theta values= 0.182968497 0.182968616

Zone 2b: moderate selection; gamma for St reaches high value

Lower and upper bounds of St popn gamma

416.666656 4166.66650

Probability of zone 2b 8.43225121E-02

Net probability of zone 2b using Simpsons rule= 8.43220130E-02

Contributions to mean load statistics over zone 2b

Contributions to mean loads within In and St= 6.17062542E-05 6.17062542E-05

Contribution to load between In and St= 6.14774035E-05

Contributions to homozygous loads for In and St= 1.22954007E-04 1.22954007E-04

Contributions to inbreeding loads= 6.12477888E-05 6.12477888E-05

Selection coefficients for In and St homokaryotypes

2.38418579E-07 2.38418579E-07

Selection coefficients for In and St homokaryotypes

2.38418579E-07 2.38418579E-07

Contributions to mean A2 freqs= 2.51033981E-07 2.51033981E-07

Contributions to mean diversities= 5.00056217E-07 5.00056217E-07

Contributions to mean proportions of seg. sites= 5.10882501E-06 5.10915925E-06

Contributions to delta-theta values= 0.652744174 0.652766824

Zone 3: strong selection approximation

Lower and upper bounds of St popn gamma= 4166.66650 1041.66675

Zone 3: strong selection approximation

Probability of zone 3= -1.18427277E-02

Mean load statistics over zone 3

Contributions to loads within In and St= 3.95340112E-06 3.95340112E-06

Contribution to load between In and St= 3.94745302E-06

Contributions to homozygous loads for In and St= 7.89489332E-06 7.89489332E-06

Contributions to inbreeding loads= 3.94148947E-06 3.94148947E-06

Selection coefficients for In and St homokaryotypes

0.00000000 0.00000000

Contributions to mean A2 freqs= 7.43551842E-09 7.43551842E-09

Contributions to mean A2 freqs at seg. sites= 0.00000000 0.00000000

Contributions to mean diversities= 1.48477346E-08 1.48477346E-08

Contributions to delta-theta values= 0.640428126 0.640428126

Mean load statistics over all zones

Loads within In and St= 5.36214598E-02 5.36214598E-02

Load between In and St= 3.49813551E-02

Homozygous load for In and St= 5.43095544E-02 5.43095544E-02

Inbreeding loads= 6.88018801E-04 6.88018801E-04

Selection coefficients for In and St homokaryotypes

1.84674263E-02 1.84674263E-02

Mean frequencies of A2 in In and St= 0.124671675 0.124671675

Ratio of these= 1.00000000

Mean diversities at selected sites in In and St= 3.90523550E-04 3.90523521E-04

Mean diversities at neutral sites in In and St= 9.97953350E-04 9.97953350E-04

pi-n/pi-s for In and St= 0.391324461 0.391324431

Ratio of these= 1.00000012

Mean freqs. of seg. sites= 1.59631507E-03 1.59631541E-03

Ratio of these= 0.999999762

Overall delta-theta values= 0.132078648 0.132078886

Ratio of these= 0.999998212

**h = 0.35**

Zone 1: quasi-neutral zone

Upper bound scaled selection coefficient for neutrality in St metapopulation= 0.250000000

Probability of zone 1= 0.120326050

Integral of selection coefficient over zone 1= 5.55351051E-03

Mean load statistics for zone 1

Mean q1 and q2= 0.600000024

F1 and F2= 0.997920930 0.997920930

Diversities= 9.97953350E-04 9.97953350E-04

Contributions to loads within In and St= 3.33127496E-03 3.33127496E-03

Contribution to load between In and St = 2.93225353E-03

Contributions to homozygous loads for In and St= 3.33210640E-03 3.33210640E-03

Contributions to inbreeding loads for In and St= 9.98077681E-04 9.98077681E-04

Contributions to selection coefficients for In and St homokaryotypes

3.98933887E-04 3.98933887E-04

Contributions to mean A2 freqs= 7.21956342E-02 7.21956342E-02

Contributions to mean diversities= 1.20079785E-04 1.20079785E-04

Contributions to mean freqs. of seg. site= 4.18966229E-04 4.18966229E-04

Delta-theta values= -1.68166161E-02 -1.68166161E-02

Zone 2a: moderate selection; cut-off at moderate gamma for St population

Lower and upper bounds of St popn gamma

0.250000000 297.619049

Probability of zone 2a= 0.749527037

Coefficients for bivariate distribution of q1 and q2

a1= 0.174999997 a2= 0.174999997

b11= 3.75000015E-02 b12= 7.50000030E-02 b22= 3.75000015E-02

Net probability of zone 2a using Simpsons rule= 0.750982523

Contributions to mean load statistics over zone 2a

Contributions to mean loads within In and St= 4.54040468E-02 4.54040468E-02

Contribution to load between In and St= 3.63166630E-02

Contributions to homozygous loads for In and St= 4.56680059E-02 4.56680059E-02

Contributions to inbreeding loads= 2.63911119E-04 2.63911235E-04

Selection coefficients for In and St homokaryotypes

9.04619694E-03 9.04619694E-03

Selection coefficients for In and St homokaryotypes

9.04619694E-03 9.04619694E-03

Contributions to mean A2 freqs= 4.99449074E-02 4.99449074E-02

Contributions to mean diversities= 2.37167464E-04 2.37167740E-04

Contributions to mean proportions of seg. sites= 1.03667553E-03 1.03667181E-03

Contributions to delta-theta values= 0.188358903 0.188355088

Zone 2b: moderate selection; gamma for St reaches high value

Lower and upper bounds of St popn gamma

297.619049 2976.19043

Probability of zone 2b 0.130073369

Net probability of zone 2b using Simpsons rule= 0.130073503

Contributions to mean load statistics over zone 2b

Contributions to mean loads within In and St= 9.58181845E-05 9.58181845E-05

Contribution to load between In and St= 9.56737131E-05

Contributions to homozygous loads for In and St= 1.36676346E-04 1.36676346E-04

Contributions to inbreeding loads= 4.08581109E-05 4.08581109E-05

Selection coefficients for In and St homokaryotypes

1.19209290E-07 1.19209290E-07

Selection coefficients for In and St homokaryotypes

1.19209290E-07 1.19209290E-07

Contributions to mean A2 freqs= 3.64839934E-07 3.64839934E-07

Contributions to mean diversities= 7.26850715E-07 7.26850715E-07

Contributions to mean proportions of seg. sites= 7.47115473E-06 7.47124068E-06

Contributions to delta-theta values= 0.654848933 0.654852867

Zone 3: strong selection approximation

Lower and upper bounds of St popn gamma= 2976.19043 1041.66675

Zone 3: strong selection approximation

Probability of zone 3= -1.17835402E-02

Mean load statistics over zone 3

Contributions to loads within In and St= 6.34808248E-06 6.34808248E-06

Contribution to load between In and St= 6.34514618E-06

Contributions to homozygous loads for In and St= 9.06448895E-06 9.06448895E-06

Contributions to inbreeding loads= 2.71640715E-06 2.71640715E-06

Selection coefficients for In and St homokaryotypes

0.00000000 0.00000000

Contributions to mean A2 freqs= 8.56230553E-09 8.56230553E-09

Contributions to mean A2 freqs at seg. sites= 0.00000000 0.00000000

Contributions to mean diversities= 1.71054211E-08 1.71054211E-08

Contributions to delta-theta values= 0.641830802 0.641830802

Mean load statistics over all zones

Loads within In and St= 4.88374867E-02 4.88374867E-02

Load between In and St= 3.93509343E-02

Homozygous load for In and St= 4.91458550E-02 4.91458550E-02

Inbreeding loads= 3.08317132E-04 3.08317249E-04

Selection coefficients for In and St homokaryotypes

9.44167376E-03 9.44167376E-03

Mean frequencies of A2 in In and St= 0.122140914 0.122140914

Ratio of these= 1.00000000

Mean diversities at selected sites in In and St= 3.57991201E-04 3.57991492E-04

Mean diversities at neutral sites in In and St= 9.97953350E-04 9.97953350E-04

pi-n/pi-s for In and St= 0.358725399 0.358725667

Ratio of these= 0.999999225

Mean freqs. of seg. sites= 1.46328239E-03 1.46327866E-03

Ratio of these= 1.00000250

Overall delta-theta values= 0.132047474 0.132044613

Ratio of these= 1.00002170

**h = 0.45**

Zone 1: quasi-neutral zone

Upper bound scaled selection coefficient for neutrality in St metapopulation= 0.250000000

Probability of zone 1= 0.120326050

Integral of selection coefficient over zone 1= 5.55351051E-03

Mean load statistics for zone 1

Mean q1 and q2= 0.600000024

F1 and F2= 0.997920930 0.997920930

Diversities= 9.97953350E-04 9.97953350E-04

Contributions to loads within In and St= 3.33182933E-03 3.33182933E-03

Contribution to load between In and St = 3.19882203E-03

Contributions to homozygous loads for In and St= 3.33210640E-03 3.33210640E-03

Contributions to inbreeding loads for In and St= 4.27983032E-04 4.27983032E-04

Contributions to selection coefficients for In and St homokaryotypes

1.32977962E-04 1.32977962E-04

Contributions to mean A2 freqs= 7.21956342E-02 7.21956342E-02

Contributions to mean diversities= 1.20079785E-04 1.20079785E-04

Contributions to mean freqs. of seg. site= 4.18966229E-04 4.18966229E-04

Delta-theta values= -1.68166161E-02 -1.68166161E-02

Zone 2a: moderate selection; cut-off at moderate gamma for St population

Lower and upper bounds of St popn gamma

0.250000000 165.343918

Probability of zone 2a= 0.654688358

Coefficients for bivariate distribution of q1 and q2

a1= 0.224999994 a2= 0.224999994

b11= 1.25000030E-02 b12= 2.50000060E-02 b22= 1.25000030E-02

Net probability of zone 2a using Simpsons rule= 0.654973686

Contributions to mean load statistics over zone 2a

Contributions to mean loads within In and St= 4.14566509E-02 4.14566509E-02

Contribution to load between In and St= 3.88442390E-02

Contributions to homozygous loads for In and St= 4.15180661E-02 4.15180661E-02

Contributions to inbreeding loads= 6.13944139E-05 6.13944067E-05

Selection coefficients for In and St homokaryotypes

2.60901451E-03 2.60901451E-03

Selection coefficients for In and St homokaryotypes

2.60901451E-03 2.60901451E-03

Contributions to mean A2 freqs= 4.79021631E-02 4.79021706E-02

Contributions to mean diversities= 2.12928629E-04 2.12929037E-04

Contributions to mean proportions of seg. sites= 9.31232469E-04 9.31230548E-04

Contributions to delta-theta values= 0.188800454 0.188797235

Zone 2b: moderate selection; gamma for St reaches high value

Lower and upper bounds of St popn gamma

165.343918 1653.43921

Probability of zone 2b 0.222876012

Net probability of zone 2b using Simpsons rule= 0.222876146

Contributions to mean load statistics over zone 2b

Contributions to mean loads within In and St= 1.73290435E-04 1.73290435E-04

Contribution to load between In and St= 1.73209599E-04

Contributions to homozygous loads for In and St= 1.92454885E-04 1.92454885E-04

Contributions to inbreeding loads= 1.91645995E-05 1.91645995E-05

Selection coefficients for In and St homokaryotypes

5.96046448E-08 5.96046448E-08

Selection coefficients for In and St homokaryotypes

5.96046448E-08 5.96046448E-08

Contributions to mean A2 freqs= 8.04372633E-07 8.04372633E-07

Contributions to mean diversities= 1.60088950E-06 1.60088950E-06

Contributions to mean proportions of seg. sites= 1.61511580E-05 1.61510998E-05

Contributions to delta-theta values= 0.648350954 0.648349643

Zone 3: strong selection approximation

Lower and upper bounds of St popn gamma= 1653.43921 1041.66675

Zone 3: strong selection approximation

Probability of zone 3= -9.74750519E-03

Mean load statistics over zone 3

Contributions to loads within In and St= 1.65990114E-05 1.65990114E-05

Contribution to load between In and St= 1.65973488E-05

Contributions to homozygous loads for In and St= 1.84415167E-05 1.84415167E-05

Contributions to inbreeding loads= 1.84249666E-06 1.84249666E-06

Selection coefficients for In and St homokaryotypes

0.00000000 0.00000000

Contributions to mean A2 freqs= 1.85836342E-08 1.85836342E-08

Contributions to mean A2 freqs at seg. sites= 0.00000000 0.00000000

Contributions to mean diversities= 3.71334323E-08 3.71334323E-08

Contributions to delta-theta values= 0.642467260 0.642467260

Mean load statistics over all zones

Loads within In and St= 4.49783690E-02 4.49783690E-02

Load between In and St= 4.22328673E-02

Homozygous load for In and St= 4.50610667E-02 4.50610667E-02

Inbreeding loads= 8.26785763E-05 8.26785617E-05

Selection coefficients for In and St homokaryotypes

2.74175406E-03 2.74175406E-03

Mean frequencies of A2 in In and St= 0.120098621 0.120098621

Ratio of these= 1.00000000

Mean diversities at selected sites in In and St= 3.34646436E-04 3.34646844E-04

Mean diversities at neutral sites in In and St= 9.97953350E-04 9.97953350E-04

pi-n/pi-s for In and St= 0.335332751 0.335333139

Ratio of these= 0.999998868

Mean freqs. of seg. sites= 1.36671821E-03 1.36671634E-03

Ratio of these= 1.00000131

Overall delta-theta values= 0.131321669 0.131319463

Ratio of these= 1.00001681

**h = 0.50**

Zone 1: quasi-neutral zone

Upper bound scaled selection coefficient for neutrality in St metapopulation= 0.250000000

Probability of zone 1= 0.120326050

Integral of selection coefficient over zone 1= 5.55351051E-03

Mean load statistics for zone 1

Mean q1 and q2= 0.600000024

F1 and F2= 0.997920930 0.997920930

Diversities= 9.97953350E-04 9.97953350E-04

Contributions to loads within In and St= 3.33210640E-03 3.33210640E-03

Contribution to load between In and St = 3.33210640E-03

Contributions to homozygous loads for In and St= 3.33210640E-03 3.33210640E-03

Contributions to inbreeding loads for In and St= 1.10992463E-04 1.10992463E-04

Contributions to selection coefficients for In and St homokaryotypes

0.00000000 0.00000000

Contributions to mean A2 freqs= 7.21956342E-02 7.21956342E-02

Contributions to mean diversities= 1.20079785E-04 1.20079785E-04

Contributions to mean freqs. of seg. site= 4.18966229E-04 4.18966229E-04

Delta-theta values= -1.68166161E-02 -1.68166161E-02

Zone 2a: moderate selection; cut-off at moderate gamma for St population

Lower and upper bounds of St popn gamma

0.250000000 82.6719589

Probability of zone 2a= 0.535827816

Coefficients for bivariate distribution of q1 and q2

a1= 0.250000000 a2= 0.250000000

b11= 0.00000000 b12= 0.00000000 b22= 0.00000000

Net probability of zone 2a using Simpsons rule= 0.535857439

Contributions to mean load statistics over zone 2a

Contributions to mean loads within In and St= 4.06352021E-02 4.06352021E-02

Contribution to load between In and St= 4.06351984E-02

Contributions to homozygous loads for In and St= 4.06352021E-02 4.06352021E-02

Contributions to inbreeding loads= 0.00000000 0.00000000

Selection coefficients for In and St homokaryotypes

0.00000000 0.00000000

Selection coefficients for In and St homokaryotypes

0.00000000 0.00000000

Contributions to mean A2 freqs= 4.81961183E-02 4.81961183E-02

Contributions to mean diversities= 2.01069677E-04 2.01069575E-04

Contributions to mean proportions of seg. sites= 8.70617805E-04 8.70617747E-04

Contributions to delta-theta values= 0.180647492 0.180647850

Zone 2b: moderate selection; gamma for St reaches high value

Lower and upper bounds of St popn gamma

82.6719589 826.719604

Probability of zone 2b 0.321361661

Net probability of zone 2b using Simpsons rule= 0.321361691

Contributions to mean load statistics over zone 2b

Contributions to mean loads within In and St= 2.71410478E-04 2.71410478E-04

Contribution to load between In and St= 2.71410478E-04

Contributions to homozygous loads for In and St= 2.71410478E-04 2.71410478E-04

Contributions to inbreeding loads= 0.00000000 0.00000000

Selection coefficients for In and St homokaryotypes

0.00000000 0.00000000

Selection coefficients for In and St homokaryotypes

0.00000000 0.00000000

Contributions to mean A2 freqs= 1.91570530E-06 1.91570530E-06

Contributions to mean diversities= 3.80167035E-06 3.80167080E-06

Contributions to mean proportions of seg. sites= 3.69022491E-05 3.69027985E-05

Contributions to delta-theta values= 0.634511828 0.634517193

Zone 3: strong selection approximation

Lower and upper bounds of St popn gamma= 826.719604 1041.66675

Zone 3: strong selection approximation

Probability of zone 3= 1.06273890E-02

Mean load statistics over zone 3

Contributions to loads within In and St= 5.15017273E-05 5.15017273E-05

Contribution to load between In and St= 5.15017273E-05

Contributions to homozygous loads for In and St= 5.15017273E-05 5.15017273E-05

Contributions to inbreeding loads= 0.00000000 0.00000000

Selection coefficients for In and St homokaryotypes

0.00000000 0.00000000

Contributions to mean A2 freqs= 7.01199951E-08 7.01199951E-08

Contributions to mean A2 freqs at seg. sites= 0.00000000 0.00000000

Contributions to mean diversities= 1.40086343E-07 1.40086343E-07

Contributions to delta-theta values= 0.641893268 0.641893268

Mean load statistics over all zones

Loads within In and St= 4.42902185E-02 4.42902185E-02

Load between In and St= 4.42902185E-02

Homozygous load for In and St= 4.42902185E-02 4.42902185E-02

Inbreeding loads= 0.00000000 0.00000000

Selection coefficients for In and St homokaryotypes

0.00000000 0.00000000

Mean frequencies of A2 in In and St= 0.120393738 0.120393738

Ratio of these= 1.00000000

Mean diversities at selected sites in In and St= 3.25091212E-04 3.25091096E-04

Mean diversities at neutral sites in In and St= 9.97953350E-04 9.97953350E-04

pi-n/pi-s for In and St= 0.325757921 0.325757802

Ratio of these= 1.00000036

Mean freqs. of seg. sites= 1.32787402E-03 1.32787460E-03

Ratio of these= 0.999999583

Overall delta-theta values= 0.131439447 0.131440103

Ratio of these= 0.999994993
