# Supplementary material for: The fitness consequences of genetic divergence between polymorphic gene arrangements
Source: Genetics. 2023 Dec 26;226(3):iyad218. doi: 10.1093/genetics/iyad218 (PMC11090464; doi:10.1093/genetics/iyad218)
Supplement: iyad218_Supplementary_Data [file iyad218_supplementary_data.zip › Supplementary_Table_3_GENETICS-2023-306559.docx]

**Supplementary Table S3 Results for a subdivided population with *N_T_* = 10^6^**

**Results are shown for mean scaled selection coefficients of 1000 and 4000 (sections 1 and 2, respectively) and inversion frequencies of 0.1 and 0.5, with dominance coefficients of h = 0.05, 0.25 and 0.45 for Fst values of 0.05, 0.15, 0.25 and 0.45**

**Mutational bias towards deleterious variants= 1.5**

**Metapopulation size= 1000000**

**Number of demes= 200**

**Local population size= 5000**

**No. of values for Simpsons rule = 650**

**Shape parameter= 0.3**

**Upper bound gamma value for neutrality in the St popn= 0.250**

**Upper bound whole popn gamma value for zone 2a with h=0.25 = 50**

**Upper bound whole popn gamma value for zone 2b with h=0.25 = 500**

**Upper limit to gamma distribution of z= x/scale parameter = 2.50**

**Threshold gamma value factor for use of approximate p.d.f.= 0.25**

**Section 1**

**Mean selection coefficient= 5.0 E-04**

**Mean scaled selection coefficient for whole metapopulation= 1000**

**Inversion frequency= 0.1**

**h= 0.05**

**Neutral Fst for whole population= 0.05**

**Scaled migration rate for whole population= 19**

Zone 1: quasi-neutral zone 1

Upper bound scaled selection coefficient for neutrality in St metapopulation= 0.250000000

Probability of zone 1= 4.39136960E-02

Integral of selection coefficient over zone 1= 1.40749034E-04

Mean load statistics for zone 1

Mean q1 and q2= 0.600000024

F1 and F2= 0.996683598 0.970923305

Diversities= 1.59187312E-03 1.39568131E-02

Contributions to loads within In and St= 8.43486050E-05 8.35654428E-05

Contributions to load between In and St = 5.40476285E-05

Contributions to homozygous loads for In and St= 8.44494207E-05 8.44494207E-05

Contributions to inbreeding loads for In and St= 1.00822561E-07 8.83980135E-07

Contributions to selection coefficients for In and St homokaryotypes

3.02791595E-05 2.95042992E-05

Contributions to mean A2 freqs= 2.63482183E-02 2.63482183E-02

Contributions to mean diversities= 6.99050288E-05 6.12895237E-04

Zone 2: quasi-neutral zone 2

Lower and upper bounds of St metapopn gamma for zone 2

0.250000000 50.0000000

Probability of zone 2= 0.171113729

Coefficients for bivariate distribution of q1 and q2 in metapopulation

G1= 0.526315808 G2= 5.84795326E-02

G3= -5.26316166E-02 G4= 0.883040905

a1= 9.73684248E-03 a2= 8.76315832E-02

b11= -2.36842272E-04 b12= 8.09999928E-02 b22= 0.321868390

Contributions to mean load statistics for zone 2

Loads within In and St= 8.04773048E-02 1.01727399E-03

Load between In and St= 4.76767123E-03

Homozygous load for In and St= 8.05755183E-02 1.27783057E-03

Inbreeding loads= 9.82725469E-05 2.60555127E-04

Selection coefficients against In and St homokaryotypes

7.29146600E-02 -3.75747681E-03

Contributions to mean A2 freqs= 0.100803070 1.71919968E-02

Contributions to mean diversities= 2.72233941E-04 1.49213464E-03

Zone 3: moderate selection zone

Lower and upper bounds of St metapopn gamma for zone 3

50.0000000 499.999969

Probability of zone 3= 0.210339963

Contributions to mean load statistics over zone 3

Loads within In and St= 8.71419832E-02 1.88568010E-04

Load between In and St= 4.53678193E-03

Homozygous load for In and St= 8.80662724E-02 1.12587016E-03

Inbreeding loads= 9.24394641E-04 9.37301957E-04

Selection coefficients for In and St homokaryotypes

7.92854428E-02 -4.35769558E-03

Contributions to mean A2 freqs= 1.73966009E-02 1.34524496E-04

Contributions to mean diversities= 2.12894913E-04 2.52242782E-04

Zone 4: strong selection zone

Upper limit to scaled gamma for St metapopn= 2.50000000

Lower limit to gamma for St metapopn= 499.999969

Upper limit to gamma for St metapopn= 30000.0020

Probability of zone 4= 0.562775493

Contributions to mean load statistics over zone 4

Loads within In and St= 2.35566287E-03 6.17035490E-04

Load between In and St= 4.85073921E-04

Homozygous load for In and St= 5.49023645E-03 4.19630343E-03

Inbreeding loads= 3.13458219E-03 3.57926567E-03

Selection coefficients for In and St homokaryotypes

1.86884403E-03 1.31964684E-04

Contributions to mean A2 freqs= 1.05335326E-04 1.05335326E-04

Contributions to mean diversities= 4.43885114E-07 2.32459547E-06

Mean load statistics over all zones

Loads within In and St= 0.170059294 1.90644292E-03

Load between In and St= 9.84357484E-03

Homozygous load for In and St= 0.174216479 6.68445369E-03

Inbreeding loads= 4.15735040E-03 4.77800658E-03

Selection coefficients for In and St homokaryotypes

0.148039997 -7.96866417E-03

Mean frequencies of A2 in In and St= 0.144653216 4.37800772E-02

Ratio of these= 3.30408764

Mean diversities at selected sites in In and St= 5.55477804E-04 2.35959701E-03

Ratio of these= 0.235412151

Mean diversities at neutral sites in In and St= 1.59187312E-03 1.39568131E-02

pi-n/pi-s for In and St= 0.348946035 0.169064164

Ratio of these= 2.06398582

**Neutral Fst for whole population= 0.1**

**Scaled migration rate for whole population= 9.0**

Zone 1: quasi-neutral zone 1

Upper bound scaled selection coefficient for neutrality in St metapopulation= 0.250000000

Probability of zone 1= 4.39136960E-02

Integral of selection coefficient over zone 1= 1.40749034E-04

Mean load statistics for zone 1

Mean q1 and q2= 0.600000024

F1 and F2= 0.996689916 0.970978200

Diversities= 1.58884050E-03 1.39304632E-02

Contributions to loads within In and St= 8.43487869E-05 8.35671017E-05

Contributions to load between In and St = 5.40476285E-05

Contributions to homozygous loads for In and St= 8.44494207E-05 8.44494207E-05

Contributions to inbreeding loads for In and St= 1.00637997E-07 8.82319057E-07

Contributions to selection coefficients for In and St homokaryotypes

3.02791595E-05 2.95042992E-05

Contributions to mean A2 freqs= 2.63482183E-02 2.63482183E-02

Contributions to mean diversities= 6.97718569E-05 6.11738127E-04

Zone 2: quasi-neutral zone 2

Lower and upper bounds of St metapopn gamma for zone 2

0.250000000 50.0000000

Probability of zone 2= 0.171113729

Coefficients for bivariate distribution of q1 and q2 in metapopulation

G1= 1.11111104 G2= 0.123456798

G3= -1.22222209 G4= 0.753086388

a1= 1.49999997E-02 a2= 0.134999990

b11= -5.49999997E-03 b12= 8.09999928E-02 b22= 0.274499953

Contributions to mean load statistics for zone 2

Loads within In and St= 8.99873301E-02 1.03473314E-03

Load between In and St= 5.25013311E-03

Homozygous load for In and St= 9.00770426E-02 1.26136700E-03

Inbreeding loads= 8.97229475E-05 2.26634307E-04

Selection coefficients against In and St homokaryotypes

8.12463164E-02 -4.22430038E-03

Contributions to mean A2 freqs= 0.106893390 1.72987934E-02

Contributions to mean diversities= 2.59888475E-04 1.40089402E-03

Zone 3: moderate selection zone

Lower and upper bounds of St metapopn gamma for zone 3

50.0000000 499.999969

Probability of zone 3= 0.210339963

Contributions to mean load statistics over zone 3

Loads within In and St= 8.74119699E-02 1.95892280E-04

Load between In and St= 4.51263599E-03

Homozygous load for In and St= 8.81513357E-02 8.80527485E-04

Inbreeding loads= 7.39300100E-04 6.84635190E-04

Selection coefficients for In and St homokaryotypes

7.95562267E-02 -4.32610512E-03

Contributions to mean A2 freqs= 1.74211767E-02 1.05653045E-04

Contributions to mean diversities= 1.77804337E-04 1.87465324E-04

Zone 4: strong selection zone

Upper limit to scaled gamma for St metapopn= 2.50000000

Lower limit to gamma for St metapopn= 499.999969

Upper limit to gamma for St metapopn= 30000.0020

Probability of zone 4= 0.562775493

Contributions to mean load statistics over zone 4

Loads within In and St= 3.16667557E-03 7.20887037E-04

Load between In and St= 4.90605249E-04

Homozygous load for In and St= 5.66782383E-03 4.12947452E-03

Inbreeding loads= 2.50115152E-03 3.40858544E-03

Selection coefficients for In and St homokaryotypes

2.67249346E-03 2.30252743E-04

Contributions to mean A2 freqs= 1.04605220E-04 1.04605220E-04

Contributions to mean diversities= 4.92976767E-07 2.42163173E-06

Mean load statistics over all zones

Loads within In and St= 0.180650324 2.03507952E-03

Load between In and St= 1.03074210E-02

Homozygous load for In and St= 0.183980659 6.35581836E-03

Inbreeding loads= 3.33027518E-03 4.32073744E-03

Selection coefficients for In and St homokaryotypes

0.156624436 -8.30662251E-03

Mean frequencies of A2 in In and St= 0.150767386 4.38572727E-02

Ratio of these= 3.43768263

Mean diversities at selected sites in In and St= 5.07957651E-04 2.20251898E-03

Ratio of these= 0.230625778

Mean diversities at neutral sites in In and St= 1.58884050E-03 1.39304632E-02

pi-n/pi-s for In and St= 0.319703370 0.158108100

Ratio of these= 2.02205563

**Neutral Fst for whole population= 0.15**

**Scaled migration rate for whole population= 5.67**

Zone 1: quasi-neutral zone 1

Upper bound scaled selection coefficient for neutrality in St metapopulation= 0.250000000

Probability of zone 1= 4.39136960E-02

Integral of selection coefficient over zone 1= 1.40749034E-04

Mean load statistics for zone 1

Mean q1 and q2= 0.600000024

F1 and F2= 0.996697068 0.971039176

Diversities= 1.58540718E-03 1.39011955E-02

Contributions to loads within In and St= 8.43490125E-05 8.35689571E-05

Contributions to load between In and St = 5.40476285E-05

Contributions to homozygous loads for In and St= 8.44494207E-05 8.44494207E-05

Contributions to inbreeding loads for In and St= 1.00411484E-07 8.80465052E-07

Contributions to selection coefficients for In and St homokaryotypes

3.02791595E-05 2.95042992E-05

Contributions to mean A2 freqs= 2.63482183E-02 2.63482183E-02

Contributions to mean diversities= 6.96210918E-05 6.10452844E-04

Zone 2: quasi-neutral zone 2

Lower and upper bounds of St metapopn gamma for zone 2

0.250000000 50.0000000

Probability of zone 2= 0.171113729

Coefficients for bivariate distribution of q1 and q2 in metapopulation

G1= 1.76470590 G2= 0.196078435

G3= -2.52941179 G4= 0.607843161

a1= 2.08823532E-02 a2= 0.187941164

b11= -1.13823544E-02 b12= 8.09999928E-02 b22= 0.221558809

Contributions to mean load statistics for zone 2

Loads within In and St= 9.92458686E-02 1.05322606E-03

Load between In and St= 5.72116068E-03

Homozygous load for In and St= 9.93260071E-02 1.25120801E-03

Inbreeding loads= 8.00922426E-05 1.97980422E-04

Selection coefficients against In and St homokaryotypes

8.92844796E-02 -4.67884541E-03

Contributions to mean A2 freqs= 0.113005660 1.74238551E-02

Contributions to mean diversities= 2.45949544E-04 1.31541409E-03

Zone 3: moderate selection zone

Lower and upper bounds of St metapopn gamma for zone 3

50.0000000 499.999969

Probability of zone 3= 0.210339963

Contributions to mean load statistics over zone 3

Loads within In and St= 8.75925943E-02 2.07560079E-04

Load between In and St= 4.49831411E-03

Homozygous load for In and St= 8.82147029E-02 7.39581184E-04

Inbreeding loads= 6.22044085E-04 5.32021222E-04

Selection coefficients for In and St homokaryotypes

7.97356367E-02 -4.29999828E-03

Contributions to mean A2 freqs= 1.74408033E-02 8.79506915E-05

Contributions to mean diversities= 1.52876295E-04 1.47488929E-04

Zone 4: strong selection zone

Upper limit to scaled gamma for St metapopn= 2.50000000

Lower limit to gamma for St metapopn= 499.999969

Upper limit to gamma for St metapopn= 30000.0020

Probability of zone 4= 0.562775493

Contributions to mean load statistics over zone 4

Loads within In and St= 3.51924519E-03 7.61540199E-04

Load between In and St= 4.78515780E-04

Homozygous load for In and St= 5.63262030E-03 3.92374443E-03

Inbreeding loads= 2.11338396E-03 3.16220778E-03

Selection coefficients for In and St homokaryotypes

3.03608179E-03 2.83002853E-04

Contributions to mean A2 freqs= 1.01594582E-04 1.01594582E-04

Contributions to mean diversities= 5.10962650E-07 2.37661880E-06

Mean load statistics over all zones

Loads within In and St= 0.190442070 2.10589520E-03

Load between In and St= 1.07520372E-02

Homozygous load for In and St= 0.193257794 5.99898305E-03

Inbreeding loads= 2.81562074E-03 3.89308995E-03

Selection coefficients for In and St homokaryotypes

0.164470851 -8.68368149E-03

Mean frequencies of A2 in In and St= 0.156896263 4.39616181E-02

Ratio of these= 3.56893730

Mean diversities at selected sites in In and St= 4.68957907E-04 2.07573269E-03

Ratio of these= 0.225924030

Mean diversities at neutral sites in In and St= 1.58540718E-03 1.39011955E-02

pi-n/pi-s for In and St= 0.295796514 0.149320439

Ratio of these= 1.98095131

**Neutral Fst for whole population= 0.2**

**Scaled migration rate for whole population= 4**

Zone 1: quasi-neutral zone 1

Upper bound scaled selection coefficient for neutrality in St metapopulation= 0.250000000

Probability of zone 1= 4.39136960E-02

Integral of selection coefficient over zone 1= 1.40749034E-04

Mean load statistics for zone 1

Mean q1 and q2= 0.600000024

F1 and F2= 0.996705115 0.971107543

Diversities= 1.58154487E-03 1.38683794E-02

Contributions to loads within In and St= 8.43492453E-05 8.35710380E-05

Contributions to load between In and St = 5.40476285E-05

Contributions to homozygous loads for In and St= 8.44494207E-05 8.44494207E-05

Contributions to inbreeding loads for In and St= 1.00176585E-07 8.78384469E-07

Contributions to selection coefficients for In and St homokaryotypes

3.02791595E-05 2.95042992E-05

Contributions to mean A2 freqs= 2.63482183E-02 2.63482183E-02

Contributions to mean diversities= 6.94514820E-05 6.09011797E-04

Zone 2: quasi-neutral zone 2

Lower and upper bounds of St metapopn gamma for zone 2

0.250000000 50.0000000

Probability of zone 2= 0.171113729

Coefficients for bivariate distribution of q1 and q2 in metapopulation

G1= 2.50000000 G2= 0.277777791

G3= -4.00000000 G4= 0.444444418

a1= 2.75000017E-02 a2= 0.247500002

b11= -1.80000011E-02 b12= 8.09999928E-02 b22= 0.161999986

Contributions to mean load statistics for zone 2

Loads within In and St= 0.107608132 1.07335253E-03

Load between In and St= 6.14880538E-03

Homozygous load for In and St= 0.107678212 1.24685874E-03

Inbreeding loads= 7.00480523E-05 1.73506502E-04

Selection coefficients against In and St homokaryotypes

9.64820981E-02 -5.08832932E-03

Contributions to mean A2 freqs= 0.118836790 1.75701939E-02

Contributions to mean diversities= 2.31047496E-04 1.23443105E-03

Zone 3: moderate selection zone

Lower and upper bounds of St metapopn gamma for zone 3

50.0000000 499.999969

Probability of zone 3= 0.210339963

Contributions to mean load statistics over zone 3

Loads within In and St= 8.77281651E-02 2.19496098E-04

Load between In and St= 4.48934641E-03

Homozygous load for In and St= 8.82679597E-02 6.49959256E-04

Inbreeding loads= 5.39933390E-04 4.30463493E-04

Selection coefficients for In and St homokaryotypes

7.98686147E-02 -4.27901745E-03

Contributions to mean A2 freqs= 1.74576733E-02 7.62116615E-05

Contributions to mean diversities= 1.33943366E-04 1.20629527E-04

Zone 4: strong selection zone

Upper limit to scaled gamma for St metapopn= 2.50000000

Lower limit to gamma for St metapopn= 499.999969

Upper limit to gamma for St metapopn= 30000.0020

Probability of zone 4= 0.562775493

Contributions to mean load statistics over zone 4

Loads within In and St= 3.72709054E-03 7.75232445E-04

Load between In and St= 4.65358113E-04

Homozygous load for In and St= 5.58213890E-03 3.71194188E-03

Inbreeding loads= 1.85504649E-03 2.93670781E-03

Selection coefficients for In and St homokaryotypes

3.25644016E-03 3.09824944E-04

Contributions to mean A2 freqs= 9.90115732E-05 9.90115732E-05

Contributions to mean diversities= 5.19963692E-07 2.28841532E-06

Mean load statistics over all zones

Loads within In and St= 0.199147746 2.15165224E-03

Load between In and St= 1.11575574E-02

Homozygous load for In and St= 0.201612756 5.69320936E-03

Inbreeding loads= 2.46512797E-03 3.54155619E-03

Selection coefficients for In and St homokaryotypes

0.171377182 -9.04655457E-03

Mean frequencies of A2 in In and St= 0.162741706 4.40936349E-02

Ratio of these= 3.69082093

Mean diversities at selected sites in In and St= 4.34962305E-04 1.96636096E-03

Ratio of these= 0.221201658

Mean diversities at neutral sites in In and St= 1.58154487E-03 1.38683794E-02

pi-n/pi-s for In and St= 0.275023699 0.141787365

Ratio of these= 1.93969119

**Neutral Fst for whole population= 0.25**

**Scaled migration rate for whole population= 3**

Zone 1: quasi-neutral zone 1

Upper bound scaled selection coefficient for neutrality in St metapopulation= 0.250000000

Probability of zone 1= 4.39136960E-02

Integral of selection coefficient over zone 1= 1.40749034E-04

Mean load statistics for zone 1

Mean q1 and q2= 0.600000024

F1 and F2= 0.996714115 0.971184611

Diversities= 1.57722470E-03 1.38313863E-02

Contributions to loads within In and St= 8.43495218E-05 8.35733881E-05

Contributions to load between In and St = 5.40476285E-05

Contributions to homozygous loads for In and St= 8.44494207E-05 8.44494207E-05

Contributions to inbreeding loads for In and St= 9.98997365E-08 8.76035472E-07

Contributions to selection coefficients for In and St homokaryotypes

3.02791595E-05 2.95042992E-05

Contributions to mean A2 freqs= 2.63482183E-02 2.63482183E-02

Contributions to mean diversities= 6.92617687E-05 6.07387279E-04

Zone 2: quasi-neutral zone 2

Lower and upper bounds of St metapopn gamma for zone 2

0.250000000 50.0000000

Probability of zone 2= 0.171113729

Coefficients for bivariate distribution of q1 and q2 in metapopulation

G1= 3.33333325 G2= 0.370370388

G3= -5.66666651 G4= 0.259259224

a1= 3.50000001E-02 a2= 0.314999998

b11= -2.55000014E-02 b12= 8.09999928E-02 b22= 9.44999754E-02

Contributions to mean load statistics for zone 2

Loads within In and St= 0.114642620 1.09576865E-03

Load between In and St= 6.51200442E-03

Homozygous load for In and St= 0.114702903 1.24809507E-03

Inbreeding loads= 6.02810578E-05 1.52326684E-04

Selection coefficients against In and St homokaryotypes

0.102489650 -5.43093681E-03

Contributions to mean A2 freqs= 0.124162674 1.77419465E-02

Contributions to mean diversities= 2.15867651E-04 1.15683535E-03

Zone 3: moderate selection zone

Lower and upper bounds of St metapopn gamma for zone 3

50.0000000 499.999969

Probability of zone 3= 0.210339963

Contributions to mean load statistics over zone 3

Loads within In and St= 8.78385380E-02 2.31863829E-04

Load between In and St= 4.48371423E-03

Homozygous load for In and St= 8.83175656E-02 5.89756004E-04

Inbreeding loads= 4.78920381E-04 3.57891753E-04

Selection coefficients for In and St homokaryotypes

7.99753666E-02 -4.26089764E-03

Contributions to mean A2 freqs= 1.74729619E-02 6.80433368E-05

Contributions to mean diversities= 1.18982476E-04 1.01494079E-04

Zone 4: strong selection zone

Upper limit to scaled gamma for St metapopn= 2.50000000

Lower limit to gamma for St metapopn= 499.999969

Upper limit to gamma for St metapopn= 30000.0020

Probability of zone 4= 0.562775493

Contributions to mean load statistics over zone 4

Loads within In and St= 3.95127293E-03 7.75378605E-04

Load between In and St= 4.57609392E-04

Homozygous load for In and St= 5.62249916E-03 3.51723935E-03

Inbreeding loads= 1.67122902E-03 2.74186372E-03

Selection coefficients for In and St homokaryotypes

3.48758698E-03 3.17692757E-04

Contributions to mean A2 freqs= 9.83811842E-05 9.83811842E-05

Contributions to mean diversities= 5.32544050E-07 2.18642913E-06

Mean load statistics over all zones

Loads within In and St= 0.206516773 2.18658452E-03

Load between In and St= 1.15073752E-02

Homozygous load for In and St= 0.208727419 5.43953991E-03

Inbreeding loads= 2.21053045E-03 3.25295818E-03

Selection coefficients for In and St homokaryotypes

0.177173078 -9.36436653E-03

Mean frequencies of A2 in In and St= 0.168082237 4.42565903E-02

Ratio of these= 3.79790306

Mean diversities at selected sites in In and St= 4.04644437E-04 1.86790305E-03

Ratio of these= 0.216630325

Mean diversities at neutral sites in In and St= 1.57722470E-03 1.38313863E-02

pi-n/pi-s for In and St= 0.256554723 0.135048136

Ratio of these= 1.89972794

**h=0.25**

**Neutral Fst for whole population= 0.05**

**Scaled migration rate for whole population= 19**

Zone 1: quasi-neutral zone 1

Upper bound scaled selection coefficient for neutrality in St metapopulation= 0.250000000

Probability of zone 1= 4.39136960E-02

Integral of selection coefficient over zone 1= 1.40749034E-04

Mean load statistics for zone 1

Mean q1 and q2= 0.600000024

F1 and F2= 0.996683598 0.970923305

Diversities= 1.59187312E-03 1.39568131E-02

Contributions to loads within In and St= 8.43934104E-05 8.39583226E-05

Contributions to load between In and St = 6.75595365E-05

Contributions to homozygous loads for In and St= 8.44494207E-05 8.44494207E-05

Contributions to inbreeding loads for In and St= 5.60153310E-08 4.91100991E-07

Contributions to selection coefficients for In and St homokaryotypes

1.68085098E-05 1.63912773E-05

Contributions to mean A2 freqs= 2.63482183E-02 2.63482183E-02

Contributions to mean diversities= 6.99050288E-05 6.12895237E-04

Zone 2: quasi-neutral zone 2

Lower and upper bounds of St metapopn gamma for zone 2

0.250000000 50.0000000

Probability of zone 2= 0.171113729

Coefficients for bivariate distribution of q1 and q2 in metapopulation

G1= 0.526315808 G2= 5.84795326E-02

G3= -5.26316166E-02 G4= 0.883040905

a1= 2.76315790E-02 a2= 0.248684227

b11= -1.31579058E-04 b12= 4.49999981E-02 b22= 0.178815767

Contributions to mean load statistics for zone 2

Loads within In and St= 3.44527252E-02 1.13034912E-03

Load between In and St= 9.27358959E-03

Homozygous load for In and St= 3.45031656E-02 1.23568997E-03

Inbreeding loads= 5.04576747E-05 1.05340056E-04

Selection coefficients against In and St homokaryotypes

2.48647928E-02 -8.17644596E-03

Contributions to mean A2 freqs= 7.34138936E-02 1.75750311E-02

Contributions to mean diversities= 2.65571347E-04 1.28044607E-03

Zone 3: moderate selection zone

Lower and upper bounds of St metapopn gamma for zone 3

50.0000000 499.999969

Probability of zone 3= 0.210339963

Contributions to mean load statistics over zone 3

Loads within In and St= 4.80081560E-03 2.15772845E-04

Load between In and St= 1.35087594E-03

Homozygous load for In and St= 4.99447249E-03 4.03744023E-04

Inbreeding loads= 1.93649961E-04 1.87970945E-04

Selection coefficients for In and St homokaryotypes

3.44401598E-03 -1.13570690E-03

Contributions to mean A2 freqs= 1.29892200E-03 5.20021276E-05

Contributions to mean diversities= 9.40994651E-05 9.80996701E-05

Zone 4: strong selection zone

Upper limit to scaled gamma for St metapopn= 2.50000000

Lower limit to gamma for St metapopn= 499.999969

Upper limit to gamma for St metapopn= 30000.0020

Probability of zone 4= 0.562775493

Contributions to mean load statistics over zone 4

Loads within In and St= 7.46119593E-04 4.79935232E-04

Load between In and St= 5.20273228E-04

Homozygous load for In and St= 1.15678005E-03 9.24250286E-04

Inbreeding loads= 4.10661363E-04 4.44314675E-04

Selection coefficients for In and St homokaryotypes

2.25841999E-04 -4.02927399E-05

Contributions to mean A2 freqs= 2.07354260E-05 2.07354260E-05

Contributions to mean diversities= 8.89003502E-08 4.48602918E-07

Mean load statistics over all zones

Loads within In and St= 4.00840528E-02 1.91001559E-03

Load between In and St= 1.12122977E-02

Homozygous load for In and St= 4.07388695E-02 2.64813378E-03

Inbreeding loads= 6.54825009E-04 7.38116796E-04

Selection coefficients for In and St homokaryotypes

2.84589529E-02 -9.34565067E-03

Mean frequencies of A2 in In and St= 0.101081766 4.39959876E-02

Ratio of these= 2.29752231

Mean diversities at selected sites in In and St= 4.29664738E-04 1.99188967E-03

Ratio of these= 0.215707093

Mean diversities at neutral sites in In and St= 1.59187312E-03 1.39568131E-02

pi-n/pi-s for In and St= 0.269911408 0.142718092

Ratio of these= 1.89122069

**Neutral Fst for whole population= 0.1**

**Scaled migration rate for whole population= 9**

Zone 1: quasi-neutral zone 1

Upper bound scaled selection coefficient for neutrality in St metapopulation= 0.250000000

Probability of zone 1= 4.39136960E-02

Integral of selection coefficient over zone 1= 1.40749034E-04

Mean load statistics for zone 1

Mean q1 and q2= 0.600000024

F1 and F2= 0.996689916 0.970978200

Diversities= 1.58884050E-03 1.39304632E-02

Contributions to loads within In and St= 8.43935195E-05 8.39592467E-05

Contributions to load between In and St = 6.75595365E-05

Contributions to homozygous loads for In and St= 8.44494207E-05 8.44494207E-05

Contributions to inbreeding loads for In and St= 5.59062698E-08 4.90178195E-07

Contributions to selection coefficients for In and St homokaryotypes

1.68085098E-05 1.63912773E-05

Contributions to mean A2 freqs= 2.63482183E-02 2.63482183E-02

Contributions to mean diversities= 6.97718569E-05 6.11738127E-04

Zone 2: quasi-neutral zone 2

Lower and upper bounds of St metapopn gamma for zone 2

0.250000000 50.0000000

Probability of zone 2= 0.171113729

Coefficients for bivariate distribution of q1 and q2 in metapopulation

G1= 1.11111104 G2= 0.123456798

G3= -1.22222209 G4= 0.753086388

a1= 3.05555556E-02 a2= 0.274999976

b11= -3.05555551E-03 b12= 4.49999981E-02 b22= 0.152499989

Contributions to mean load statistics for zone 2

Loads within In and St= 3.46798785E-02 1.14468695E-03

Load between In and St= 9.33690462E-03

Homozygous load for In and St= 3.47292535E-02 1.24440144E-03

Inbreeding loads= 4.93507796E-05 9.97137977E-05

Selection coefficients against In and St homokaryotypes

2.50245333E-02 -8.22591782E-03

Contributions to mean A2 freqs= 7.39017352E-02 1.76677536E-02

Contributions to mean diversities= 2.62158661E-04 1.24572392E-03

Zone 3: moderate selection zone

Lower and upper bounds of St metapopn gamma for zone 3

50.0000000 499.999969

Probability of zone 3= 0.210339963

Contributions to mean load statistics over zone 3

Loads within In and St= 4.94676409E-03 2.19550144E-04

Load between In and St= 1.37987814E-03

Homozygous load for In and St= 5.12602925E-03 3.88301210E-04

Inbreeding loads= 1.79263996E-04 1.68750936E-04

Selection coefficients for In and St homokaryotypes

3.56054306E-03 -1.16097927E-03

Contributions to mean A2 freqs= 1.32028246E-03 4.98902518E-05

Contributions to mean diversities= 8.83952089E-05 8.89816802E-05

Zone 4: strong selection zone

Upper limit to scaled gamma for St metapopn= 2.50000000

Lower limit to gamma for St metapopn= 499.999969

Upper limit to gamma for St metapopn= 30000.0020

Probability of zone 4= 0.562775493

Contributions to mean load statistics over zone 4

Loads within In and St= 9.27725632E-04 5.16280765E-04

Load between In and St= 5.70535369E-04

Homozygous load for In and St= 1.30788365E-03 9.74186172E-04

Inbreeding loads= 3.80158657E-04 4.57904040E-04

Selection coefficients for In and St homokaryotypes

3.57151031E-04 -5.42402267E-05

Contributions to mean A2 freqs= 2.29611687E-05 2.29611687E-05

Contributions to mean diversities= 1.09269287E-07 5.06374022E-07

Mean load statistics over all zones

Loads within In and St= 4.06387597E-02 1.96447689E-03

Load between In and St= 1.13548776E-02

Homozygous load for In and St= 4.12476137E-02 2.69133830E-03

Inbreeding loads= 6.08829316E-04 7.26858969E-04

Selection coefficients for In and St homokaryotypes

2.88592577E-02 -9.43458080E-03

Mean frequencies of A2 in In and St= 0.101593196 4.40888256E-02

Ratio of these= 2.30428457

Mean diversities at selected sites in In and St= 4.20434983E-04 1.94695021E-03

Ratio of these= 0.215945423

Mean diversities at neutral sites in In and St= 1.58884050E-03 1.39304632E-02

pi-n/pi-s for In and St= 0.264617503 0.139762059

Ratio of these= 1.89334285

**Neutral Fst for whole population= 0.15**

**Scaled migration rate for whole population= 5.67**

Zone 1: quasi-neutral zone 1

Upper bound scaled selection coefficient for neutrality in St metapopulation= 0.250000000

Probability of zone 1= 4.39136960E-02

Integral of selection coefficient over zone 1= 1.40749034E-04

Mean load statistics for zone 1

Mean q1 and q2= 0.600000024

F1 and F2= 0.996697068 0.971039176

Diversities= 1.58540718E-03 1.39011955E-02

Contributions to loads within In and St= 8.43936359E-05 8.39602799E-05

Contributions to load between In and St = 6.75595365E-05

Contributions to homozygous loads for In and St= 8.44494207E-05 8.44494207E-05

Contributions to inbreeding loads for In and St= 5.57888207E-08 4.89146316E-07

Contributions to selection coefficients for In and St homokaryotypes

1.68085098E-05 1.63912773E-05

Contributions to mean A2 freqs= 2.63482183E-02 2.63482183E-02

Contributions to mean diversities= 6.96210918E-05 6.10452844E-04

Zone 2: quasi-neutral zone 2

Lower and upper bounds of St metapopn gamma for zone 2

0.250000000 50.0000000

Probability of zone 2= 0.171113729

Coefficients for bivariate distribution of q1 and q2 in metapopulation

G1= 1.76470590 G2= 0.196078435

G3= -2.52941179 G4= 0.607843161

a1= 3.38235311E-02 a2= 0.304411739

b11= -6.32352987E-03 b12= 4.49999981E-02 b22= 0.123088233

Contributions to mean load statistics for zone 2

Loads within In and St= 3.49266492E-02 1.16001500E-03

Load between In and St= 9.40586347E-03

Homozygous load for In and St= 3.49747725E-02 1.25408615E-03

Inbreeding loads= 4.81312636E-05 9.40706814E-05

Selection coefficients against In and St homokaryotypes

2.51978636E-02 -8.27991962E-03

Contributions to mean A2 freqs= 7.44386613E-02 1.77708082E-02

Contributions to mean diversities= 2.58348387E-04 1.20950525E-03

Zone 3: moderate selection zone

Lower and upper bounds of St metapopn gamma for zone 3

50.0000000 499.999969

Probability of zone 3= 0.210339963

Contributions to mean load statistics over zone 3

Loads within In and St= 5.06782020E-03 2.26264543E-04

Load between In and St= 1.40500022E-03

Homozygous load for In and St= 5.23632811E-03 3.78615223E-04

Inbreeding loads= 1.68501050E-04 1.52350738E-04

Selection coefficients for In and St homokaryotypes

3.65614891E-03 -1.17945671E-03

Contributions to mean A2 freqs= 1.33895350E-03 4.83111326E-05

Contributions to mean diversities= 8.35953688E-05 8.14172308E-05

Zone 4: strong selection zone

Upper limit to scaled gamma for St metapopn= 2.50000000

Lower limit to gamma for St metapopn= 499.999969

Upper limit to gamma for St metapopn= 30000.0020

Probability of zone 4= 0.562775493

Contributions to mean load statistics over zone 4

Loads within In and St= 1.07089255E-03 5.33772924E-04

Load between In and St= 6.06429472E-04

Homozygous load for In and St= 1.43265771E-03 9.92975780E-04

Inbreeding loads= 3.61765764E-04 4.59203729E-04

Selection coefficients for In and St homokaryotypes

4.64379787E-04 -7.27176666E-05

Contributions to mean A2 freqs= 2.47447606E-05 2.47447606E-05

Contributions to mean diversities= 1.24709274E-07 5.32193837E-07

Mean load statistics over all zones

Loads within In and St= 4.11497578E-02 2.00401270E-03

Load between In and St= 1.14848530E-02

Homozygous load for In and St= 4.17282060E-02 2.71012657E-03

Inbreeding loads= 5.78453881E-04 7.06114282E-04

Selection coefficients for In and St homokaryotypes

2.92292237E-02 -9.52589512E-03

Mean frequencies of A2 in In and St= 0.102150574 4.41920795E-02

Ratio of these= 2.31151319

Mean diversities at selected sites in In and St= 4.11689573E-04 1.90190761E-03

Ratio of these= 0.216456361

Mean diversities at neutral sites in In and St= 1.58540718E-03 1.39011955E-02

pi-n/pi-s for In and St= 0.259674340 0.136816114

Ratio of these= 1.89798069

**Neutral Fst for whole population= 0.2**

**Scaled migration rate for whole population= 4**

Zone 1: quasi-neutral zone 1

Upper bound scaled selection coefficient for neutrality in St metapopulation= 0.250000000

Probability of zone 1= 4.39136960E-02

Integral of selection coefficient over zone 1= 1.40749034E-04

Mean load statistics for zone 1

Mean q1 and q2= 0.600000024

F1 and F2= 0.996705115 0.971107543

Diversities= 1.58154487E-03 1.38683794E-02

Contributions to loads within In and St= 8.43937669E-05 8.39614368E-05

Contributions to load between In and St = 6.75595365E-05

Contributions to homozygous loads for In and St= 8.44494207E-05 8.44494207E-05

Contributions to inbreeding loads for In and St= 5.56545920E-08 4.87988586E-07

Contributions to selection coefficients for In and St homokaryotypes

1.68085098E-05 1.63912773E-05

Contributions to mean A2 freqs= 2.63482183E-02 2.63482183E-02

Contributions to mean diversities= 6.94514820E-05 6.09011797E-04

Zone 2: quasi-neutral zone 2

Lower and upper bounds of St metapopn gamma for zone 2

0.250000000 50.0000000

Probability of zone 2= 0.171113729

Coefficients for bivariate distribution of q1 and q2 in metapopulation

G1= 2.50000000 G2= 0.277777791

G3= -4.00000000 G4= 0.444444418

a1= 3.75000015E-02 a2= 0.337499976

b11= -1.00000007E-02 b12= 4.49999981E-02 b22= 8.99999887E-02

Contributions to mean load statistics for zone 2

Loads within In and St= 3.51955555E-02 1.17655611E-03

Load between In and St= 9.48128384E-03

Homozygous load for In and St= 3.52423303E-02 1.26496691E-03

Inbreeding loads= 4.67876416E-05 8.84134715E-05

Selection coefficients against In and St homokaryotypes

2.53864527E-02 -8.33928585E-03

Contributions to mean A2 freqs= 7.50324726E-02 1.78861897E-02

Contributions to mean diversities= 2.54091778E-04 1.17164676E-03

Zone 3: moderate selection zone

Lower and upper bounds of St metapopn gamma for zone 3

50.0000000 499.999969

Probability of zone 3= 0.210339963

Contributions to mean load statistics over zone 3

Loads within In and St= 5.17047709E-03 2.34424035E-04

Load between In and St= 1.42691133E-03

Homozygous load for In and St= 5.33028506E-03 3.72380222E-04

Inbreeding loads= 1.59816351E-04 1.37956129E-04

Selection coefficients for In and St homokaryotypes

3.73655558E-03 -1.19316578E-03

Contributions to mean A2 freqs= 1.35542953E-03 4.70867526E-05

Contributions to mean diversities= 7.94001608E-05 7.48923558E-05

Zone 4: strong selection zone

Upper limit to scaled gamma for St metapopn= 2.50000000

Lower limit to gamma for St metapopn= 499.999969

Upper limit to gamma for St metapopn= 30000.0020

Probability of zone 4= 0.562775493

Contributions to mean load statistics over zone 4

Loads within In and St= 1.19706662E-03 5.44993556E-04

Load between In and St= 6.37391640E-04

Homozygous load for In and St= 1.54663809E-03 1.00283674E-03

Inbreeding loads= 3.49570066E-04 4.57843649E-04

Selection coefficients for In and St homokaryotypes

5.59508801E-04 -9.23871994E-05

Contributions to mean A2 freqs= 2.64784157E-05 2.64784157E-05

Contributions to mean diversities= 1.38424184E-07 5.47460616E-07

Mean load statistics over all zones

Loads within In and St= 4.16474901E-02 2.03993497E-03

Load between In and St= 1.16131455E-02

Homozygous load for In and St= 4.22037020E-02 2.72463332E-03

Inbreeding loads= 5.56229730E-04 6.84701256E-04

Selection coefficients for In and St homokaryotypes

2.95878053E-02 -9.61923599E-03

Mean frequencies of A2 in In and St= 0.102762602 4.43079770E-02

Ratio of these= 2.31927991

Mean diversities at selected sites in In and St= 4.03081853E-04 1.85609830E-03

Ratio of these= 0.217166215

Mean diversities at neutral sites in In and St= 1.58154487E-03 1.38683794E-02

pi-n/pi-s for In and St= 0.254865885 0.133836716

Ratio of these= 1.90430462

**Neutral Fst for whole population= 0.25**

**Scaled migration rate for whole population= 3**

Zone 1: quasi-neutral zone 1

Upper bound scaled selection coefficient for neutrality in St metapopulation= 0.250000000

Probability of zone 1= 4.39136960E-02

Integral of selection coefficient over zone 1= 1.40749034E-04

Mean load statistics for zone 1

Mean q1 and q2= 0.600000024

F1 and F2= 0.996714115 0.971184611

Diversities= 1.57722470E-03 1.38313863E-02

Contributions to loads within In and St= 8.43939197E-05 8.39627392E-05

Contributions to load between In and St = 6.75595365E-05

Contributions to homozygous loads for In and St= 8.44494207E-05 8.44494207E-05

Contributions to inbreeding loads for In and St= 5.55035840E-08 4.86688236E-07

Contributions to selection coefficients for In and St homokaryotypes

1.68085098E-05 1.63912773E-05

Contributions to mean A2 freqs= 2.63482183E-02 2.63482183E-02

Contributions to mean diversities= 6.92617687E-05 6.07387279E-04

Zone 2: quasi-neutral zone 2

Lower and upper bounds of St metapopn gamma for zone 2

0.250000000 50.0000000

Probability of zone 2= 0.171113729

Coefficients for bivariate distribution of q1 and q2 in metapopulation

G1= 3.33333325 G2= 0.370370388

G3= -5.66666651 G4= 0.259259224

a1= 4.16666679E-02 a2= 0.374999970

b11= -1.41666671E-02 b12= 4.49999981E-02 b22= 5.24999909E-02

Contributions to mean load statistics for zone 2

Loads within In and St= 3.54896560E-02 1.19459385E-03

Load between In and St= 9.56411101E-03

Homozygous load for In and St= 3.55349295E-02 1.27733813E-03

Inbreeding loads= 4.53068933E-05 8.27434997E-05

Selection coefficients against In and St homokaryotypes

2.55923867E-02 -8.40461254E-03

Contributions to mean A2 freqs= 7.56918415E-02 1.80165172E-02

Contributions to mean diversities= 2.49328645E-04 1.13198056E-03

Zone 3: moderate selection zone

Lower and upper bounds of St metapopn gamma for zone 3

50.0000000 499.999969

Probability of zone 3= 0.210339963

Contributions to mean load statistics over zone 3

Loads within In and St= 5.25889965E-03 2.43657152E-04

Load between In and St= 1.44632894E-03

Homozygous load for In and St= 5.41142933E-03 3.69003421E-04

Inbreeding loads= 1.52528766E-04 1.25346269E-04

Selection coefficients for In and St homokaryotypes

3.80533934E-03 -1.20341778E-03

Contributions to mean A2 freqs= 1.37007365E-03 4.61689524E-05

Contributions to mean diversities= 7.56597801E-05 6.91825553E-05

Zone 4: strong selection zone

Upper limit to scaled gamma for St metapopn= 2.50000000

Lower limit to gamma for St metapopn= 499.999969

Upper limit to gamma for St metapopn= 30000.0020

Probability of zone 4= 0.562775493

Contributions to mean load statistics over zone 4

Loads within In and St= 1.32876204E-03 5.53344260E-04

Load between In and St= 6.69732981E-04

Homozygous load for In and St= 1.66966161E-03 1.00916729E-03

Inbreeding loads= 3.40899598E-04 4.55822767E-04

Selection coefficients for In and St homokaryotypes

6.58810139E-04 -1.16348267E-04

Contributions to mean A2 freqs= 2.85666629E-05 2.85666629E-05

Contributions to mean diversities= 1.53261922E-07 5.58103011E-07

Mean load statistics over all zones

Loads within In and St= 4.21617106E-02 2.07555806E-03

Load between In and St= 1.17477328E-02

Homozygous load for In and St= 4.27004695E-02 2.73995823E-03

Inbreeding loads= 5.38790773E-04 6.64399238E-04

Selection coefficients for In and St homokaryotypes

2.99561024E-02 -9.71913338E-03

Mean frequencies of A2 in In and St= 0.103438698 4.44394685E-02

Ratio of these= 2.32763124

Mean diversities at selected sites in In and St= 3.94403440E-04 1.80910854E-03

Ratio of these= 0.218009830

Mean diversities at neutral sites in In and St= 1.57722470E-03 1.38313863E-02

pi-n/pi-s for In and St= 0.250061661 0.130797341

Ratio of these= 1.91182530

**h=0.45**

**Neutral Fst for whole population= 0.05**

**Scaled migration rate for whole population= 19**

Zone 1: quasi-neutral zone 1

Upper bound scaled selection coefficient for neutrality in St metapopulation= 0.250000000

Probability of zone 1= 4.39136960E-02

Integral of selection coefficient over zone 1= 1.40749034E-04

Mean load statistics for zone 1

Mean q1 and q2= 0.600000024

F1 and F2= 0.996683598 0.970923305

Diversities= 1.59187312E-03 1.39568131E-02

Contributions to loads within In and St= 8.44382157E-05 8.43512025E-05

Contributions to load between In and St = 8.10714409E-05

Contributions to homozygous loads for In and St= 8.44494207E-05 8.44494207E-05

Contributions to inbreeding loads for In and St= 1.12080993E-08 9.82218822E-08

Contributions to selection coefficients for In and St homokaryotypes

3.33786011E-06 3.27825546E-06

Contributions to mean A2 freqs= 2.63482183E-02 2.63482183E-02

Contributions to mean diversities= 6.99050288E-05 6.12895237E-04

Zone 2: quasi-neutral zone 2

Lower and upper bounds of St metapopn gamma for zone 2

0.250000000 50.0000000

Probability of zone 2= 0.171113729

Coefficients for bivariate distribution of q1 and q2 in metapopulation

G1= 0.526315808 G2= 5.84795326E-02

G3= -5.26316166E-02 G4= 0.883040905

a1= 4.55263145E-02 a2= 0.409736782

b11= -2.63158163E-05 b12= 9.00000241E-03 b22= 3.57631631E-02

Contributions to mean load statistics for zone 2

Loads within In and St= 1.98006574E-02 1.20845530E-03

Load between In and St= 9.53217503E-03

Homozygous load for In and St= 1.98090468E-02 1.22374552E-03

Inbreeding loads= 8.38525193E-06 1.52900448E-05

Selection coefficients against In and St homokaryotypes

1.02159381E-02 -8.35847855E-03

Contributions to mean A2 freqs= 6.03856705E-02 1.80104654E-02

Contributions to mean diversities= 2.47368618E-04 1.09999371E-03

Zone 3: moderate selection zone

Lower and upper bounds of St metapopn gamma for zone 3

50.0000000 499.999969

Probability of zone 3= 0.210339963

Contributions to mean load statistics over zone 3

Loads within In and St= 7.01803889E-04 2.10162776E-04

Load between In and St= 4.30518267E-04

Homozygous load for In and St= 7.24944053E-04 2.31719881E-04

Inbreeding loads= 2.31401664E-05 2.15571581E-05

Selection coefficients for In and St homokaryotypes

2.71260738E-04 -2.20417976E-04

Contributions to mean A2 freqs= 1.57527524E-04 3.05474568E-05

Contributions to mean diversities= 5.76639613E-05 5.77514911E-05

Zone 4: strong selection zone

Upper limit to scaled gamma for St metapopn= 2.50000000

Lower limit to gamma for St metapopn= 499.999969

Upper limit to gamma for St metapopn= 30000.0020

Probability of zone 4= 0.562775493

Contributions to mean load statistics over zone 4

Loads within In and St= 6.07724476E-04 4.72122134E-04

Load between In and St= 5.30732330E-04

Homozygous load for In and St= 6.56688586E-04 5.22713701E-04

Inbreeding loads= 4.89644044E-05 5.05913231E-05

Selection coefficients for In and St homokaryotypes

7.70092010E-05 -5.86509705E-05

Contributions to mean A2 freqs= 1.17406953E-05 1.17406953E-05

Contributions to mean diversities= 5.03997626E-08 2.48076105E-07

Mean load statistics over all zones

Loads within In and St= 2.11946238E-02 1.97509141E-03

Load between In and St= 1.05744973E-02

Homozygous load for In and St= 2.12751273E-02 2.06262851E-03

Inbreeding loads= 8.05010350E-05 8.75367477E-05

Selection coefficients for In and St homokaryotypes

1.05639100E-02 -8.63647461E-03

Mean frequencies of A2 in In and St= 8.69031623E-02 4.44009751E-02

Ratio of these= 1.95723546

Mean diversities at selected sites in In and St= 3.74988042E-04 1.77088857E-03

Ratio of these= 0.211751342

Mean diversities at neutral sites in In and St= 1.59187312E-03 1.39568131E-02

pi-n/pi-s for In and St= 0.235564023 0.126883447

Ratio of these= 1.85653865

**Neutral Fst for whole population= 0.1**

**Scaled migration rate for whole population= 9**

Zone 1: quasi-neutral zone 1

Upper bound scaled selection coefficient for neutrality in St metapopulation= 0.250000000

Probability of zone 1= 4.39136960E-02

Integral of selection coefficient over zone 1= 1.40749034E-04

Mean load statistics for zone 1

Mean q1 and q2= 0.600000024

F1 and F2= 0.996689916 0.970978200

Diversities= 1.58884050E-03 1.39304632E-02

Contributions to loads within In and St= 8.44382375E-05 8.43513844E-05

Contributions to load between In and St = 8.10714409E-05

Contributions to homozygous loads for In and St= 8.44494207E-05 8.44494207E-05

Contributions to inbreeding loads for In and St= 1.11829319E-08 9.80373187E-08

Contributions to selection coefficients for In and St homokaryotypes

3.33786011E-06 3.27825546E-06

Contributions to mean A2 freqs= 2.63482183E-02 2.63482183E-02

Contributions to mean diversities= 6.97718569E-05 6.11738127E-04

Zone 2: quasi-neutral zone 2

Lower and upper bounds of St metapopn gamma for zone 2

0.250000000 50.0000000

Probability of zone 2= 0.171113729

Coefficients for bivariate distribution of q1 and q2 in metapopulation

G1= 1.11111104 G2= 0.123456798

G3= -1.22222209 G4= 0.753086388

a1= 4.61111069E-02 a2= 0.414999962

b11= -6.11111231E-04 b12= 9.00000241E-03 b22= 3.05000041E-02

Contributions to mean load statistics for zone 2

Loads within In and St= 1.98864844E-02 1.22063793E-03

Load between In and St= 9.57674906E-03

Homozygous load for In and St= 1.98948551E-02 1.23584608E-03

Inbreeding loads= 8.38701271E-06 1.52090224E-05

Selection coefficients against In and St homokaryotypes

1.02567673E-02 -8.39114189E-03

Contributions to mean A2 freqs= 6.04938678E-02 1.80606004E-02

Contributions to mean diversities= 2.47231306E-04 1.09507365E-03

Zone 3: moderate selection zone

Lower and upper bounds of St metapopn gamma for zone 3

50.0000000 499.999969

Probability of zone 3= 0.210339963

Contributions to mean load statistics over zone 3

Loads within In and St= 7.98757130E-04 2.18567206E-04

Load between In and St= 4.77351161E-04

Homozygous load for In and St= 8.21320980E-04 2.39410976E-04

Inbreeding loads= 2.25633539E-05 2.08437286E-05

Selection coefficients for In and St homokaryotypes

3.21328640E-04 -2.58803368E-04

Contributions to mean A2 freqs= 1.72324959E-04 3.14609315E-05

Contributions to mean diversities= 5.68874348E-05 5.62765163E-05

Zone 4: strong selection zone

Upper limit to scaled gamma for St metapopn= 2.50000000

Lower limit to gamma for St metapopn= 499.999969

Upper limit to gamma for St metapopn= 30000.0020

Probability of zone 4= 0.562775493

Contributions to mean load statistics over zone 4

Loads within In and St= 7.00356904E-04 5.03870542E-04

Load between In and St= 5.86990151E-04

Homozygous load for In and St= 7.47593818E-04 5.56826533E-04

Inbreeding loads= 4.72364300E-05 5.29554673E-05

Selection coefficients for In and St homokaryotypes

1.13368034E-04 -8.30888748E-05

Contributions to mean A2 freqs= 1.34552110E-05 1.34552110E-05

Contributions to mean diversities= 6.41983391E-08 2.85249172E-07

Mean load statistics over all zones

Loads within In and St= 2.14700345E-02 2.02742708E-03

Load between In and St= 1.07221622E-02

Homozygous load for In and St= 2.15482172E-02 2.11653300E-03

Inbreeding loads= 7.81979761E-05 8.91062518E-05

Selection coefficients for In and St homokaryotypes

1.06903315E-02 -8.73267651E-03

Mean frequencies of A2 in In and St= 8.70278701E-02 4.44537364E-02

Ratio of these= 1.95771778

Mean diversities at selected sites in In and St= 3.73954797E-04 1.76337361E-03

Ratio of these= 0.2120678

Mean diversities at neutral sites in In and St= 1.58884050E-03 1.39304632E-02

pi-n/pi-s for In and St= 0.235363334 0.126583993

Ratio of these= 1.85934520

**Neutral Fst for whole population= 0.15**

**Scaled migration rate for whole population= 5.67**

Zone 1: quasi-neutral zone 1

Upper bound scaled selection coefficient for neutrality in St metapopulation= 0.250000000

Probability of zone 1= 4.39136960E-02

Integral of selection coefficient over zone 1= 1.40749034E-04

Mean load statistics for zone 1

Mean q1 and q2= 0.600000024

F1 and F2= 0.996697068 0.971039176

Diversities= 1.58540718E-03 1.39011955E-02

Contributions to loads within In and St= 8.44382666E-05 8.43515954E-05

Contributions to load between In and St = 8.10714409E-05

Contributions to homozygous loads for In and St= 8.44494207E-05 8.44494207E-05

Contributions to inbreeding loads for In and St= 1.11577636E-08 9.78275807E-08

Contributions to selection coefficients for In and St homokaryotypes

3.33786011E-06 3.27825546E-06

Contributions to mean A2 freqs= 2.63482183E-02 2.63482183E-02

Contributions to mean diversities= 6.96210918E-05 6.10452844E-04

Zone 2: quasi-neutral zone 2

Lower and upper bounds of St metapopn gamma for zone 2

0.250000000 50.0000000

Probability of zone 2= 0.171113729

Coefficients for bivariate distribution of q1 and q2 in metapopulation

G1= 1.76470590 G2= 0.196078435

G3= -2.52941179 G4= 0.607843161

a1= 4.67647016E-02 a2= 0.420882314

b11= -1.26470637E-03 b12= 9.00000241E-03 b22= 2.46176515E-02

Contributions to mean load statistics for zone 2

Loads within In and St= 1.99816246E-02 1.23410078E-03

Load between In and St= 9.62614268E-03

Homozygous load for In and St= 1.99900046E-02 1.24921824E-03

Inbreeding loads= 8.38734104E-06 1.51178674E-05

Selection coefficients against In and St homokaryotypes

1.03020668E-02 -8.42738152E-03

Contributions to mean A2 freqs= 6.06140159E-02 1.81162804E-02

Contributions to mean diversities= 2.47050892E-04 1.08959479E-03

Zone 3: moderate selection zone

Lower and upper bounds of St metapopn gamma for zone 3

50.0000000 499.999969

Probability of zone 3= 0.210339963

Contributions to mean load statistics over zone 3

Loads within In and St= 8.88351526E-04 2.28472927E-04

Load between In and St= 5.21621143E-04

Homozygous load for In and St= 9.10537085E-04 2.48565892E-04

Inbreeding loads= 2.21862228E-05 2.00929189E-05

Selection coefficients for In and St homokaryotypes

3.66687775E-04 -2.93135643E-04

Contributions to mean A2 freqs= 1.86576901E-04 3.25341935E-05

Contributions to mean diversities= 5.62730456E-05 5.50344266E-05

Zone 4: strong selection zone

Upper limit to scaled gamma for St metapopn= 2.50000000

Lower limit to gamma for St metapopn= 499.999969

Upper limit to gamma for St metapopn= 30000.0020

Probability of zone 4= 0.562775493

Contributions to mean load statistics over zone 4

Loads within In and St= 7.79808790E-04 5.20293077E-04

Load between In and St= 6.30100083E-04

Homozygous load for In and St= 8.26013915E-04 5.74204139E-04

Inbreeding loads= 4.62051794E-05 5.39103021E-05

Selection coefficients for In and St homokaryotypes

1.49726868E-04 -1.09791756E-04

Contributions to mean A2 freqs= 1.49718044E-05 1.49718044E-05

Contributions to mean diversities= 7.58203385E-08 3.05722665E-07

Mean load statistics over all zones

Loads within In and St= 2.17342228E-02 2.06721853E-03

Load between In and St= 1.08589353E-02

Homozygous load for In and St= 2.18110047E-02 2.15643784E-03

Inbreeding loads= 7.67899037E-05 8.92189128E-05

Selection coefficients for In and St homokaryotypes

1.08163357E-02 -8.83042812E-03

Mean frequencies of A2 in In and St= 8.71637836E-02 4.45120037E-02

Ratio of these= 1.95820844

Mean diversities at selected sites in In and St= 3.73020826E-04 1.75538776E-03

Ratio of these= 0.212500528

Mean diversities at neutral sites in In and St= 1.58540718E-03 1.39011955E-02

pi-n/pi-s for In and St= 0.235283926 0.126276031

Ratio of these= 1.86325085

**Neutral Fst for whole population= 0.2**

**Scaled migration rate for whole population= 4**

Zone 1: quasi-neutral zone 1

Upper bound scaled selection coefficient for neutrality in St metapopulation= 0.250000000

Probability of zone 1= 4.39136960E-02

Integral of selection coefficient over zone 1= 1.40749034E-04

Mean load statistics for zone 1

Mean q1 and q2= 0.600000024

F1 and F2= 0.996705115 0.971107543

Diversities= 1.58154487E-03 1.38683794E-02

Contributions to loads within In and St= 8.44382885E-05 8.43518283E-05

Contributions to load between In and St = 8.10714409E-05

Contributions to homozygous loads for In and St= 8.44494207E-05 8.44494207E-05

Contributions to inbreeding loads for In and St= 1.11325962E-08 9.75926824E-08

Contributions to selection coefficients for In and St homokaryotypes

3.33786011E-06 3.27825546E-06

Contributions to mean A2 freqs= 2.63482183E-02 2.63482183E-02

Contributions to mean diversities= 6.94514820E-05 6.09011797E-04

Zone 2: quasi-neutral zone 2

Lower and upper bounds of St metapopn gamma for zone 2

0.250000000 50.0000000

Probability of zone 2= 0.171113729

Coefficients for bivariate distribution of q1 and q2 in metapopulation

G1= 2.50000000 G2= 0.277777791

G3= -4.00000000 G4= 0.444444418

a1= 4.74999957E-02 a2= 0.427499950

b11= -2.00000056E-03 b12= 9.00000241E-03 b22= 1.80000030E-02

Contributions to mean load statistics for zone 2

Loads within In and St= 2.00876147E-02 1.24905456E-03

Load between In and St= 9.68115684E-03

Homozygous load for In and St= 2.00959984E-02 1.26406678E-03

Inbreeding loads= 8.38585311E-06 1.50146125E-05

Selection coefficients against In and St homokaryotypes

1.03524923E-02 -8.46779346E-03

Contributions to mean A2 freqs= 6.07477725E-02 1.81783903E-02

Contributions to mean diversities= 2.46818381E-04 1.08345656E-03

Zone 3: moderate selection zone

Lower and upper bounds of St metapopn gamma for zone 3

50.0000000 499.999969

Probability of zone 3= 0.210339963

Contributions to mean load statistics over zone 3

Loads within In and St= 9.71889589E-04 2.38993685E-04

Load between In and St= 5.63436945E-04

Homozygous load for In and St= 9.93786030E-04 2.58235727E-04

Inbreeding loads= 2.18970890E-05 1.92424104E-05

Selection coefficients for In and St homokaryotypes

4.08351421E-04 -3.24487686E-04

Contributions to mean A2 freqs= 2.00382303E-04 3.36560188E-05

Contributions to mean diversities= 5.57337080E-05 5.37646010E-05

Zone 4: strong selection zone

Upper limit to scaled gamma for St metapopn= 2.50000000

Lower limit to gamma for St metapopn= 499.999969

Upper limit to gamma for St metapopn= 30000.0020

Probability of zone 4= 0.562775493

Contributions to mean load statistics over zone 4

Loads within In and St= 8.54225655E-04 5.31637983E-04

Load between In and St= 6.68631459E-04

Homozygous load for In and St= 8.99753592E-04 5.86089969E-04

Inbreeding loads= 4.55275986E-05 5.44518734E-05

Selection coefficients for In and St homokaryotypes

1.85549259E-04 -1.36971474E-04

Contributions to mean A2 freqs= 1.64802950E-05 1.64802950E-05

Contributions to mean diversities= 8.67882264E-08 3.20478335E-07

Mean load statistics over all zones

Loads within In and St= 2.19981670E-02 2.10403814E-03

Load between In and St= 1.09942975E-02

Homozygous load for In and St= 2.20739879E-02 2.19284184E-03

Inbreeding loads= 7.58216775E-05 8.88064897E-05

Selection coefficients for In and St homokaryotypes

1.09435320E-02 -8.92984867E-03

Mean frequencies of A2 in In and St= 8.73128548E-02 4.45767418E-02

Ratio of these= 1.95870876

Mean diversities at selected sites in In and St= 3.72090377E-04 1.74655335E-03

Ratio of these= 0.213042662

Mean diversities at neutral sites in In and St= 1.58154487E-03 1.38683794E-02

pi-n/pi-s for In and St= 0.235270202 0.125937805

Ratio of these= 1.86814594

**Neutral Fst for whole population= 0.25**

**Scaled migration rate for whole population= 3**

Zone 1: quasi-neutral zone 1

Upper bound scaled selection coefficient for neutrality in St metapopulation= 0.250000000

Probability of zone 1= 4.39136960E-02

Integral of selection coefficient over zone 1= 1.40749034E-04

Mean load statistics for zone 1

Mean q1 and q2= 0.600000024

F1 and F2= 0.996714115 0.971184611

Diversities= 1.57722470E-03 1.38313863E-02

Contributions to loads within In and St= 8.44383248E-05 8.43520829E-05

Contributions to load between In and St = 8.10714409E-05

Contributions to homozygous loads for In and St= 8.44494207E-05 8.44494207E-05

Contributions to inbreeding loads for In and St= 1.10990390E-08 9.73410010E-08

Contributions to selection coefficients for In and St homokaryotypes

3.33786011E-06 3.27825546E-06

Contributions to mean A2 freqs= 2.63482183E-02 2.63482183E-02

Contributions to mean diversities= 6.92617687E-05 6.07387279E-04

Zone 2: quasi-neutral zone 2

Lower and upper bounds of St metapopn gamma for zone 2

0.250000000 50.0000000

Probability of zone 2= 0.171113729

Coefficients for bivariate distribution of q1 and q2 in metapopulation

G1= 3.33333325 G2= 0.370370388

G3= -5.66666651 G4= 0.259259224

a1= 4.83333282E-02 a2= 0.434999973

b11= -2.83333403E-03 b12= 9.00000241E-03 b22= 1.05000008E-02

Contributions to mean load statistics for zone 2

Loads within In and St= 2.02063583E-02 1.26575213E-03

Load between In and St= 9.74275358E-03

Homozygous load for In and St= 2.02147365E-02 1.28065015E-03

Inbreeding loads= 8.38172036E-06 1.48967301E-05

Selection coefficients against In and St homokaryotypes

1.04090571E-02 -8.51297379E-03

Contributions to mean A2 freqs= 6.08979426E-02 1.82482190E-02

Contributions to mean diversities= 2.46517913E-04 1.07653160E-03

Zone 3: moderate selection zone

Lower and upper bounds of St metapopn gamma for zone 3

50.0000000 499.999969

Probability of zone 3= 0.210339963

Contributions to mean load statistics over zone 3

Loads within In and St= 1.05050032E-03 2.50186538E-04

Load between In and St= 6.03336201E-04

Homozygous load for In and St= 1.07215915E-03 2.68522010E-04

Inbreeding loads= 2.16583576E-05 1.83353786E-05

Selection coefficients for In and St homokaryotypes

4.47034836E-04 -3.53217125E-04

Contributions to mean A2 freqs= 2.13824387E-04 3.48325011E-05

Contributions to mean diversities= 5.52393758E-05 5.24381903E-05

Zone 4: strong selection zone

Upper limit to scaled gamma for St metapopn= 2.50000000

Lower limit to gamma for St metapopn= 499.999969

Upper limit to gamma for St metapopn= 30000.0020

Probability of zone 4= 0.562775493

Contributions to mean load statistics over zone 4

Loads within In and St= 9.33266652E-04 5.40599576E-04

Load between In and St= 7.08180654E-04

Homozygous load for In and St= 9.78315948E-04 5.95415710E-04

Inbreeding loads= 4.50482512E-05 5.48160860E-05

Selection coefficients for In and St homokaryotypes

2.25067139E-04 -1.67608261E-04

Contributions to mean A2 freqs= 1.82054318E-05 1.82054318E-05

Contributions to mean diversities= 9.86542901E-08 3.32547756E-07

Mean load statistics over all zones

Loads within In and St= 2.22745631E-02 2.14089034E-03

Load between In and St= 1.11353416E-02

Homozygous load for In and St= 2.23496594E-02 2.22903723E-03

Inbreeding loads= 7.50994295E-05 8.81455344E-05

Selection coefficients for In and St homokaryotypes

1.10774040E-02 -9.03499126E-03

Mean frequencies of A2 in In and St= 8.74781907E-02 4.46494743E-02

Ratio of these= 1.95922112

Mean diversities at selected sites in In and St= 3.71117727E-04 1.73668971E-03

Ratio of these= 0.213692591

Mean diversities at neutral sites in In and St= 1.57722470E-03 1.38313863E-02

pi-n/pi-s for In and St= 0.235297948 0.125561506

Ratio of these= 1.87396562

**Inversion frequency= 0.5**

**h= 0.5**

**Neutral Fst for whole population= 0.05**

**Scaled migration rate for whole population= 19**

Zone 1: quasi-neutral zone 1

Upper bound scaled selection coefficient for neutrality in St metapopulation= 0.250000000

Probability of zone 1= 7.93938339E-02

Integral of selection coefficient over zone 1= 4.58041381E-04

Mean load statistics for zone 1

Mean q1 and q2= 0.600000024

F1 and F2= 0.983634770 0.983634770

Diversities= 7.85531010E-03 7.85531010E-03

Contributions to loads within In and St= 2.73205718E-04 2.73205718E-04

Contributions to load between In and St = 1.75887893E-04

Contributions to homozygous loads for In and St= 2.74824852E-04 2.74824852E-04

Contributions to inbreeding loads for In and St= 1.61910918E-06 1.61910918E-06

Contributions to selection coefficients for In and St homokaryotypes

9.73343849E-05 9.73343849E-05

Contributions to mean A2 freqs= 4.76363041E-02 4.76363041E-02

Contributions to mean diversities= 6.23663189E-04 6.23663189E-04

Zone 2: quasi-neutral zone 2

Lower and upper bounds of St metapopn gamma for zone 2

0.250000000 50.0000000

Probability of zone 2= 0.307079256

Coefficients for bivariate distribution of q1 and q2 in metapopulation

G1= 0.105263159 G2= 0.105263159

G3= 0.789473653 G4= 0.789473653

a1= 4.86842096E-02 a2= 4.86842096E-02

b11= 8.88157859E-02 b12= 0.224999994 b22= 8.88157859E-02

Contributions to mean load statistics for zone 2

Loads within In and St= 6.66895648E-03 6.66892668E-03

Load between In and St= 2.10287399E-03

Homozygous load for In and St= 7.34044844E-03 7.34042004E-03

Inbreeding loads= 6.71490969E-04 6.71490969E-04

Selection coefficients against In and St homokaryotypes

4.55570221E-03 4.55564260E-03

Contributions to mean A2 freqs= 4.02492099E-02 4.02491316E-02

Contributions to mean diversities= 1.78641418E-03 1.78641465E-03

Zone 3: moderate selection zone

Lower and upper bounds of St metapopn gamma for zone 3

50.0000000 100.000000

Probability of zone 3= 8.60972703E-02

Contributions to mean load statistics over zone 3

Loads within In and St= 1.05104824E-04 1.05109524E-04

Load between In and St= 5.41400113E-05

Homozygous load for In and St= 5.37090586E-04 5.37155895E-04

Inbreeding loads= 4.31985827E-04 4.32046450E-04

Selection coefficients for In and St homokaryotypes

5.09619713E-05 5.09619713E-05

Contributions to mean A2 freqs= 7.64762299E-05 7.64892829E-05

Contributions to mean diversities= 1.36617004E-04 1.36645933E-04

Zone 4: strong selection zone

Upper limit to scaled gamma for St metapopn= 2.50000000

Lower limit to gamma for St metapopn= 100.000000

Upper limit to gamma for St metapopn= 4166.66699

Probability of zone 4= 0.515572548

Contributions to mean load statistics over zone 4

Loads within In and St= 9.17460944E-04 9.17461002E-04

Load between In and St= 4.63152217E-04

Homozygous load for In and St= 4.60641598E-03 4.60641598E-03

Inbreeding loads= 3.68896034E-03 3.68896034E-03

Selection coefficients for In and St homokaryotypes

4.54187393E-04 4.54187393E-04

Contributions to mean A2 freqs= 2.94390775E-04 2.94390775E-04

Contributions to mean diversities= 5.11789722E-06 5.11789722E-06

Mean load statistics over all zones

Loads within In and St= 7.96472840E-03 7.96470325E-03

Load between In and St= 2.79605435E-03

Homozygous load for In and St= 1.27587803E-02 1.27588175E-02

Inbreeding loads= 4.79405653E-03 4.79411706E-03

Selection coefficients for In and St homokaryotypes

5.15532494E-03 5.15532494E-03

Mean frequencies of A2 in In and St= 8.82563740E-02 8.82563144E-02

Ratio of these= 1.00000072

Mean diversities at selected sites in In and St= 2.55181245E-03 2.55184178E-03

Ratio of these= 0.999988496

Mean diversities at neutral sites in In and St= 7.85531010E-03 7.85531010E-03

pi-n/pi-s for In and St= 0.324851900 0.324855626

Ratio of these= 0.999988556

**Neutral Fst for whole population= 0.1**

**Scaled migration rate for whole population= 9**

Zone 1: quasi-neutral zone 1

Upper bound scaled selection coefficient for neutrality in St metapopulation= 0.250000000

Probability of zone 1= 7.93938339E-02

Integral of selection coefficient over zone 1= 4.58041381E-04

Mean load statistics for zone 1

Mean q1 and q2= 0.600000024

F1 and F2= 0.983666062 0.983666062

Diversities= 7.84028973E-03 7.84028973E-03

Contributions to loads within In and St= 2.73208803E-04 2.73208803E-04

Contributions to load between In and St = 1.75887893E-04

Contributions to homozygous loads for In and St= 2.74824852E-04 2.74824852E-04

Contributions to inbreeding loads for In and St= 1.61602406E-06 1.61602406E-06

Contributions to selection coefficients for In and St homokaryotypes

9.73343849E-05 9.73343849E-05

Contributions to mean A2 freqs= 4.76363041E-02 4.76363041E-02

Contributions to mean diversities= 6.22470689E-04 6.22470689E-04

Zone 2: quasi-neutral zone 2

Lower and upper bounds of St metapopn gamma for zone 2

0.250000000 50.0000000

Probability of zone 2= 0.307079256

Coefficients for bivariate distribution of q1 and q2 in metapopulation

G1= 0.222222224 G2= 0.222222224

G3= 0.555555582 G4= 0.555555582

a1= 7.50000030E-02 a2= 7.50000030E-02

b11= 6.25000000E-02 b12= 0.224999994 b22= 6.25000000E-02

Contributions to mean load statistics for zone 2

Loads within In and St= 7.44101359E-03 7.44094839E-03

Load between In and St= 2.34544184E-03

Homozygous load for In and St= 8.01020954E-03 8.01014341E-03

Inbreeding loads= 5.69208700E-04 5.69208234E-04

Selection coefficients against In and St homokaryotypes

5.08260727E-03 5.08254766E-03

Contributions to mean A2 freqs= 4.22545262E-02 4.22543585E-02

Contributions to mean diversities= 1.66195689E-03 1.66195491E-03

Zone 3: moderate selection zone

Lower and upper bounds of St metapopn gamma for zone 3

50.0000000 100.000000

Probability of zone 3= 8.60972703E-02

Contributions to mean load statistics over zone 3

Loads within In and St= 1.14272174E-04 1.14283379E-04

Load between In and St= 4.28610037E-05

Homozygous load for In and St= 4.25896054E-04 4.25939477E-04

Inbreeding loads= 3.11624113E-04 3.11656360E-04

Selection coefficients for In and St homokaryotypes

7.14063644E-05 7.14063644E-05

Contributions to mean A2 freqs= 6.06872673E-05 6.06959511E-05

Contributions to mean diversities= 9.86234008E-05 9.86401501E-05

Zone 4: strong selection zone

Upper limit to scaled gamma for St metapopn= 2.50000000

Lower limit to gamma for St metapopn= 100.000000

Upper limit to gamma for St metapopn= 4166.66699

Probability of zone 4= 0.515572548

Contributions to mean load statistics over zone 4

Loads within In and St= 1.20762724E-03 1.20762724E-03

Load between In and St= 4.59516043E-04

Homozygous load for In and St= 4.56969347E-03 4.56969393E-03

Inbreeding loads= 3.36206914E-03 3.36206914E-03

Selection coefficients for In and St homokaryotypes

7.47859478E-04 7.47859478E-04

Contributions to mean A2 freqs= 2.87897594E-04 2.87897594E-04

Contributions to mean diversities= 5.29872932E-06 5.29872932E-06

Mean load statistics over all zones

Loads within In and St= 9.03612189E-03 9.03606787E-03

Load between In and St= 3.02370684E-03

Homozygous load for In and St= 1.32806236E-02 1.32806022E-02

Inbreeding loads= 4.24451800E-03 4.24454967E-03

Selection coefficients for In and St homokaryotypes

5.99437952E-03 5.99431992E-03

Mean frequencies of A2 in In and St= 9.02394131E-02 9.02392492E-02

Ratio of these= 1.00000179

Mean diversities at selected sites in In and St= 2.38834973E-03 2.38836440E-03

Ratio of these= 0.999993861

Mean diversities at neutral sites in In and St= 7.84028973E-03 7.84028973E-03

pi-n/pi-s for In and St= 0.304625183 0.304627061

Ratio of these= 0.999993861

**Neutral Fst for whole population= 0.15**

**Scaled migration rate for whole population= 5.667**

Zone 1: quasi-neutral zone 1

Upper bound scaled selection coefficient for neutrality in St metapopulation= 0.250000000

Probability of zone 1= 7.93938339E-02

Integral of selection coefficient over zone 1= 4.58041381E-04

Mean load statistics for zone 1

Mean q1 and q2= 0.600000024

F1 and F2= 0.983700812 0.983700812

Diversities= 7.82360975E-03 7.82360975E-03

Contributions to loads within In and St= 2.73212267E-04 2.73212267E-04

Contributions to load between In and St = 1.75887893E-04

Contributions to homozygous loads for In and St= 2.74824852E-04 2.74824852E-04

Contributions to inbreeding loads for In and St= 1.61258413E-06 1.61258413E-06

Contributions to selection coefficients for In and St homokaryotypes

9.73343849E-05 9.73343849E-05

Contributions to mean A2 freqs= 4.76363041E-02 4.76363041E-02

Contributions to mean diversities= 6.21146348E-04 6.21146348E-04

Zone 2: quasi-neutral zone 2

Lower and upper bounds of St metapopn gamma for zone 2

0.250000000 50.0000000

Probability of zone 2= 0.307079256

Coefficients for bivariate distribution of q1 and q2 in metapopulation

G1= 0.352941185 G2= 0.352941185

G3= 0.294117630 G4= 0.294117630

a1= 0.104411766 a2= 0.104411766

b11= 3.30882333E-02 b12= 0.224999994 b22= 3.30882333E-02

Contributions to mean load statistics for zone 2

Loads within In and St= 8.42236914E-03 8.42217077E-03

Load between In and St= 2.67518195E-03

Homozygous load for In and St= 8.90778657E-03 8.90758447E-03

Inbreeding loads= 4.85398079E-04 4.85397031E-04

Selection coefficients against In and St homokaryotypes

5.73068857E-03 5.73050976E-03

Contributions to mean A2 freqs= 4.46978472E-02 4.46973629E-02

Contributions to mean diversities= 1.54699991E-03 1.54699525E-03

Zone 3: moderate selection zone

Lower and upper bounds of St metapopn gamma for zone 3

50.0000000 100.000000

Probability of zone 3= 8.60972703E-02

Contributions to mean load statistics over zone 3

Loads within In and St= 1.20297984E-04 1.20311743E-04

Load between In and St= 3.61456041E-05

Homozygous load for In and St= 3.59522877E-04 3.59555212E-04

Inbreeding loads= 2.39225046E-04 2.39243658E-04

Selection coefficients for In and St homokaryotypes

8.41617584E-05 8.41617584E-05

Contributions to mean A2 freqs= 5.12125735E-05 5.12190418E-05

Contributions to mean diversities= 7.56790905E-05 7.56891313E-05

Zone 4: strong selection zone

Upper limit to scaled gamma for St metapopn= 2.50000000

Lower limit to gamma for St metapopn= 100.000000

Upper limit to gamma for St metapopn= 4166.66699

Probability of zone 4= 0.515572548

Contributions to mean load statistics over zone 4

Loads within In and St= 1.41186034E-03 1.41186034E-03

Load between In and St= 4.47225902E-04

Homozygous load for In and St= 4.44824575E-03 4.44824575E-03

Inbreeding loads= 3.03638447E-03 3.03638470E-03

Selection coefficients for In and St homokaryotypes

9.64164734E-04 9.64164734E-04

Contributions to mean A2 freqs= 2.80548062E-04 2.80548062E-04

Contributions to mean diversities= 5.39395978E-06 5.39396024E-06

Mean load statistics over all zones

Loads within In and St= 1.02277398E-02 1.02275554E-02

Load between In and St= 3.33444146E-03

Homozygous load for In and St= 1.39903799E-02 1.39902104E-02

Inbreeding loads= 3.76262004E-03 3.76263796E-03

Selection coefficients for In and St homokaryotypes

6.86961412E-03 6.86943531E-03

Mean frequencies of A2 in In and St= 9.26659182E-02 9.26654413E-02

Ratio of these= 1.00000513

Mean diversities at selected sites in In and St= 2.24921922E-03 2.24922458E-03

Ratio of these= 0.999997616

Mean diversities at neutral sites in In and St= 7.82360975E-03 7.82360975E-03

pi-n/pi-s for In and St= 0.287491232 0.287491918

Ratio of these= 0.999997616

**Neutral Fst for whole population= 0.2**

**Scaled migration rate for whole population= 4**

Zone 1: quasi-neutral zone 1

Upper bound scaled selection coefficient for neutrality in St metapopulation= 0.250000000

Probability of zone 1= 7.93938339E-02

Integral of selection coefficient over zone 1= 4.58041381E-04

Mean load statistics for zone 1

Mean q1 and q2= 0.600000024

F1 and F2= 0.983739853 0.983739853

Diversities= 7.80487061E-03 7.80487061E-03

Contributions to loads within In and St= 2.73216108E-04 2.73216108E-04

Contributions to load between In and St = 1.75887893E-04

Contributions to homozygous loads for In and St= 2.74824852E-04 2.74824852E-04

Contributions to inbreeding loads for In and St= 1.60873458E-06 1.60873458E-06

Contributions to selection coefficients for In and St homokaryotypes

9.73343849E-05 9.73343849E-05

Contributions to mean A2 freqs= 4.76363041E-02 4.76363041E-02

Contributions to mean diversities= 6.19658618E-04 6.19658618E-04

Zone 2: quasi-neutral zone 2

Lower and upper bounds of St metapopn gamma for zone 2

0.250000000 50.0000000

Probability of zone 2= 0.307079256

Coefficients for bivariate distribution of q1 and q2 in metapopulation

G1= 0.500000000 G2= 0.500000000

G3= 0.00000000 G4= 0.00000000

a1= 0.137500003 a2= 0.137500003

b11= 0.00000000 b12= 0.224999994 b22= 0.00000000

Contributions to mean load statistics for zone 2

Loads within In and St= 9.68581345E-03 9.68497619E-03

Load between In and St= 3.13485949E-03

Homozygous load for In and St= 1.01016723E-02 1.01008322E-02

Inbreeding loads= 4.15851857E-04 4.15847840E-04

Selection coefficients against In and St homokaryotypes

6.52956963E-03 6.52873516E-03

Contributions to mean A2 freqs= 4.77018766E-02 4.77002002E-02

Contributions to mean diversities= 1.43844180E-03 1.43842620E-03

Zone 3: moderate selection zone

Lower and upper bounds of St metapopn gamma for zone 3

50.0000000 100.000000

Probability of zone 3= 8.60972703E-02

Contributions to mean load statistics over zone 3

Loads within In and St= 1.25143750E-04 1.25160601E-04

Load between In and St= 3.17954218E-05

Homozygous load for In and St= 3.16458347E-04 3.16484511E-04

Inbreeding loads= 1.91314437E-04 1.91323794E-04

Selection coefficients for In and St homokaryotypes

9.33408737E-05 9.33408737E-05

Contributions to mean A2 freqs= 4.50388943E-05 4.50441257E-05

Contributions to mean diversities= 6.04608103E-05 6.04666966E-05

Zone 4: strong selection zone

Upper limit to scaled gamma for St metapopn= 2.50000000

Lower limit to gamma for St metapopn= 100.000000

Upper limit to gamma for St metapopn= 4166.66699

Probability of zone 4= 0.515572548

Contributions to mean load statistics over zone 4

Loads within In and St= 1.56914641E-03 1.56914641E-03

Load between In and St= 4.34263638E-04

Homozygous load for In and St= 4.32011811E-03 4.32011764E-03

Inbreeding loads= 2.75097671E-03 2.75097671E-03

Selection coefficients for In and St homokaryotypes

1.13421679E-03 1.13421679E-03

Contributions to mean A2 freqs= 2.74116377E-04 2.74116377E-04

Contributions to mean diversities= 5.45943476E-06 5.45943431E-06

Mean load statistics over all zones

Loads within In and St= 1.16533199E-02 1.16524994E-02

Load between In and St= 3.77680641E-03

Homozygous load for In and St= 1.50130745E-02 1.50122596E-02

Inbreeding loads= 3.35975178E-03 3.35975713E-03

Selection coefficients for In and St homokaryotypes

7.84558058E-03 7.84474611E-03

Mean frequencies of A2 in In and St= 9.56573337E-02 9.56556648E-02

Ratio of these= 1.00001740

Mean diversities at selected sites in In and St= 2.12402060E-03 2.12401082E-03

Ratio of these= 1.00000465

Mean diversities at neutral sites in In and St= 7.80487061E-03 7.80487061E-03

pi-n/pi-s for In and St= 0.272140384 0.272139132

Ratio of these= 1.00000465

**Neutral Fst for whole population= 0.25**

**Scaled migration rate for whole population= 3**

Zone 1: quasi-neutral zone 1

Upper bound scaled selection coefficient for neutrality in St metapopulation= 0.250000000

Probability of zone 1= 7.93938339E-02

Integral of selection coefficient over zone 1= 4.58041381E-04

Mean load statistics for zone 1

Mean q1 and q2= 0.600000024

F1 and F2= 0.983783841 0.983783841

Diversities= 7.78375613E-03 7.78375613E-03

Contributions to loads within In and St= 2.73220474E-04 2.73220474E-04

Contributions to load between In and St = 1.75887893E-04

Contributions to homozygous loads for In and St= 2.74824852E-04 2.74824852E-04

Contributions to inbreeding loads for In and St= 1.60436639E-06 1.60436639E-06

Contributions to selection coefficients for In and St homokaryotypes

9.73343849E-05 9.73343849E-05

Contributions to mean A2 freqs= 4.76363041E-02 4.76363041E-02

Contributions to mean diversities= 6.17982238E-04 6.17982238E-04

Zone 2: quasi-neutral zone 2

Lower and upper bounds of St metapopn gamma for zone 2

0.250000000 50.0000000

Probability of zone 2= 0.307079256

Coefficients for bivariate distribution of q1 and q2 in metapopulation

G1= 0.666666687 G2= 0.666666687

G3= -0.333333373 G4= -0.333333373

a1= 0.175000012 a2= 0.175000012

b11= -3.75000052E-02 b12= 0.224999994 b22= -3.75000052E-02

Contributions to mean load statistics for zone 2

Loads within In and St= 1.13190738E-02 1.13134542E-02

Load between In and St= 3.79207009E-03

Homozygous load for In and St= 1.16759427E-02 1.16703017E-02

Inbreeding loads= 3.56881035E-04 3.56859964E-04

Selection coefficients against In and St homokaryotypes

7.49874115E-03 7.49319792E-03

Contributions to mean A2 freqs= 5.14180772E-02 5.14090247E-02

Contributions to mean diversities= 1.33320235E-03 1.33313204E-03

Zone 3: moderate selection zone

Lower and upper bounds of St metapopn gamma for zone 3

50.0000000 100.000000

Probability of zone 3= 8.60972703E-02

Contributions to mean load statistics over zone 3

Loads within In and St= 1.29721404E-04 1.29740962E-04

Load between In and St= 2.88446245E-05

Homozygous load for In and St= 2.87215895E-04 2.87238625E-04

Inbreeding loads= 1.57494738E-04 1.57497881E-04

Selection coefficients for In and St homokaryotypes

1.00851059E-04 1.00910664E-04

Contributions to mean A2 freqs= 4.08305095E-05 4.08350461E-05

Contributions to mean diversities= 4.97062865E-05 4.97093533E-05

Zone 4: strong selection zone

Upper limit to scaled gamma for St metapopn= 2.50000000

Lower limit to gamma for St metapopn= 100.000000

Upper limit to gamma for St metapopn= 4166.66699

Probability of zone 4= 0.515572548

Contributions to mean load statistics over zone 4

Loads within In and St= 1.69715087E-03 1.69715064E-03

Load between In and St= 4.22177836E-04

Homozygous load for In and St= 4.20059124E-03 4.20059124E-03

Inbreeding loads= 2.50344467E-03 2.50344421E-03

Selection coefficients for In and St homokaryotypes

1.27416849E-03 1.27416849E-03

Contributions to mean A2 freqs= 2.68480624E-04 2.68480624E-04

Contributions to mean diversities= 5.50691357E-06 5.50691357E-06

Mean load statistics over all zones

Loads within In and St= 1.34191662E-02 1.34135662E-02

Load between In and St= 4.41898033E-03

Homozygous load for In and St= 1.64385755E-02 1.64329559E-02

Inbreeding loads= 3.01942485E-03 3.01940646E-03

Selection coefficients for In and St homokaryotypes

8.95982981E-03 8.95422697E-03

Mean frequencies of A2 in In and St= 9.93636921E-02 9.93546471E-02

Ratio of these= 1.00009108

Mean diversities at selected sites in In and St= 2.00639782E-03 2.00633053E-03

Ratio of these= 1.00003350

Mean diversities at neutral sites in In and St= 7.78375613E-03 7.78375613E-03

pi-n/pi-s for In and St= 0.257767290 0.257758647

Ratio of these= 1.00003350

**h= 0.25**

**Neutral Fst for whole population= 0.05**

**Scaled migration rate for whole population= 19**

Zone 1: quasi-neutral zone 1

Upper bound scaled selection coefficient for neutrality in St metapopulation= 0.250000000

Probability of zone 1= 7.93938339E-02

Integral of selection coefficient over zone 1= 4.58041381E-04

Mean load statistics for zone 1

Mean q1 and q2= 0.600000024

F1 and F2= 0.983634770 0.983634770

Diversities= 7.85531010E-03 7.85531010E-03

Contributions to loads within In and St= 2.73925340E-04 2.73925340E-04

Contributions to load between In and St = 2.19859867E-04

Contributions to homozygous loads for In and St= 2.74824852E-04 2.74824852E-04

Contributions to inbreeding loads for In and St= 8.99499014E-07 8.99499014E-07

Contributions to selection coefficients for In and St homokaryotypes

5.40614128E-05 5.40614128E-05

Contributions to mean A2 freqs= 4.76363041E-02 4.76363041E-02

Contributions to mean diversities= 6.23663189E-04 6.23663189E-04

Zone 2: quasi-neutral zone 2

Lower and upper bounds of St metapopn gamma for zone 2

0.250000000 50.0000000

Probability of zone 2= 0.307079256

Coefficients for bivariate distribution of q1 and q2 in metapopulation

G1= 0.105263159 G2= 0.105263159

G3= 0.789473653 G4= 0.789473653

a1= 0.138157889 a2= 0.138157889

b11= 4.93421033E-02 b12= 0.125000000 b22= 4.93421033E-02

Contributions to mean load statistics for zone 2

Loads within In and St= 4.79250075E-03 4.79250075E-03

Load between In and St= 3.16453562E-03

Homozygous load for In and St= 4.99526644E-03 4.99526644E-03

Inbreeding loads= 2.02761497E-04 2.02761512E-04

Selection coefficients against In and St homokaryotypes

1.62667036E-03 1.62667036E-03

Contributions to mean A2 freqs= 3.73733230E-02 3.73733230E-02

Contributions to mean diversities= 1.32121053E-03 1.32121053E-03

Zone 3: moderate selection zone

Lower and upper bounds of St metapopn gamma for zone 3

50.0000000 100.000000

Probability of zone 3= 8.60972703E-02

Contributions to mean load statistics over zone 3

Loads within In and St= 1.09732588E-04 1.09741057E-04

Load between In and St= 9.99951808E-05

Homozygous load for In and St= 1.99913571E-04 1.99933944E-04

Inbreeding loads= 9.01809835E-05 9.01928943E-05

Selection coefficients for In and St homokaryotypes

9.71555710E-06 9.77516174E-06

Contributions to mean A2 freqs= 2.86208524E-05 2.86249324E-05

Contributions to mean diversities= 5.16278269E-05 5.16362888E-05

Zone 4: strong selection zone

Upper limit to scaled gamma for St metapopn= 2.50000000

Lower limit to gamma for St metapopn= 100.000000

Upper limit to gamma for St metapopn= 4166.66699

Probability of zone 4= 0.515572548

Contributions to mean load statistics over zone 4

Loads within In and St= 4.71151288E-04 4.71151201E-04

Load between In and St= 4.31227585E-04

Homozygous load for In and St= 8.62368674E-04 8.62368674E-04

Inbreeding loads= 3.91216745E-04 3.91216745E-04

Selection coefficients for In and St homokaryotypes

3.99351120E-05 3.99351120E-05

Contributions to mean A2 freqs= 4.96985012E-05 4.96985012E-05

Contributions to mean diversities= 8.77576269E-07 8.77576213E-07

Mean load statistics over all zones

Loads within In and St= 5.64731006E-03 5.64731844E-03

Load between In and St= 3.91561817E-03

Homozygous load for In and St= 6.33237371E-03 6.33239420E-03

Inbreeding loads= 6.85058767E-04 6.85070641E-04

Selection coefficients for In and St homokaryotypes

1.73020363E-03 1.73020363E-03

Mean frequencies of A2 in In and St= 8.50879401E-02 8.50879475E-02

Ratio of these= 0.999999940

Mean diversities at selected sites in In and St= 1.99737912E-03 1.99738750E-03

Ratio of these= 0.999995828

Mean diversities at neutral sites in In and St= 7.85531010E-03 7.85531010E-0

pi-n/pi-s for In and St= 0.254271209 0.254272252

Ratio of these= 0.999995887

**Neutral Fst for whole population= 0.1**

**Scaled migration rate for whole population= 9**

Zone 1: quasi-neutral zone 1

Upper bound scaled selection coefficient for neutrality in St metapopulation= 0.250000000

Probability of zone 1= 7.93938339E-02

Integral of selection coefficient over zone 1= 4.58041381E-04

Mean load statistics for zone 1

Mean q1 and q2= 0.600000024

F1 and F2= 0.983666062 0.983666062

Diversities= 7.84028973E-03 7.84028973E-03

Contributions to loads within In and St= 2.73927028E-04 2.73927028E-04

Contributions to load between In and St = 2.19859867E-04

Contributions to homozygous loads for In and St= 2.74824852E-04 2.74824852E-04

Contributions to inbreeding loads for In and St= 8.97806331E-07 8.97806331E-07

Contributions to selection coefficients for In and St homokaryotypes

5.40614128E-05 5.40614128E-05

Contributions to mean A2 freqs= 4.76363041E-02 4.76363041E-02

Contributions to mean diversities= 6.22470689E-04 6.22470689E-04

Zone 2: quasi-neutral zone 2

Lower and upper bounds of St metapopn gamma for zone 2

0.250000000 50.0000000

Probability of zone 2= 0.307079256

Coefficients for bivariate distribution of q1 and q2 in metapopulation

G1= 0.222222224 G2= 0.222222224

G3= 0.555555582 G4= 0.555555582

a1= 0.152777776 a2= 0.152777776

b11= 3.47222239E-02 b12= 0.125000000 b22= 3.47222239E-02

Contributions to mean load statistics for zone 2

Loads within In and St= 4.86692833E-03 4.86692833E-03

Load between In and St= 3.21404985E-03

Homozygous load for In and St= 5.05784759E-03 5.05784759E-03

Inbreeding loads= 1.90919862E-04 1.90919862E-04

Selection coefficients against In and St homokaryotypes

1.65152550E-03 1.65152550E-03

Contributions to mean A2 freqs= 3.77955325E-02 3.77955325E-02

Contributions to mean diversities= 1.28156901E-03 1.28156948E-03

Zone 3: moderate selection zone

Lower and upper bounds of St metapopn gamma for zone 3

50.0000000 100.000000

Probability of zone 3= 8.60972703E-02

Contributions to mean load statistics over zone 3

Loads within In and St= 1.16343756E-04 1.16354837E-04

Load between In and St= 9.86282030E-05

Homozygous load for In and St= 1.97182570E-04 1.97200730E-04

Inbreeding loads= 8.08390687E-05 8.08461264E-05

Selection coefficients for In and St homokaryotypes

1.77025795E-05 1.77025795E-05

Contributions to mean A2 freqs= 2.82350502E-05 2.82386773E-05

Contributions to mean diversities= 4.62811004E-05 4.62875723E-05

Zone 4: strong selection zone

Upper limit to scaled gamma for St metapopn= 2.50000000

Lower limit to gamma for St metapopn= 100.000000

Upper limit to gamma for St metapopn= 4166.66699

Probability of zone 4= 0.515572548

Contributions to mean load statistics over zone 4

Loads within In and St= 5.37434185E-04 5.37434185E-04

Load between In and St= 4.65258257E-04

Homozygous load for In and St= 9.30418493E-04 9.30418435E-04

Inbreeding loads= 3.92984250E-04 3.92984250E-04

Selection coefficients for In and St homokaryotypes

7.21812248E-05 7.21812248E-05

Contributions to mean A2 freqs= 5.36356529E-05 5.36356529E-05

Contributions to mean diversities= 9.99217832E-07 9.99217832E-07

Mean load statistics over all zones

Loads within In and St= 5.79463318E-03 5.79464436E-03

Load between In and St= 3.99779622E-03

Homozygous load for In and St= 6.46027364E-03 6.46029133E-03

Inbreeding loads= 6.65640982E-04 6.65648025E-04

Selection coefficients for In and St homokaryotypes

1.79523230E-03 1.79523230E-03

Mean frequencies of A2 in In and St= 8.55137110E-02 8.55137110E-02

Ratio of these= 1.00000000

Mean diversities at selected sites in In and St= 1.95131998E-03 1.95132685E-03

Ratio of these= 0.999996483

Mean diversities at neutral sites in In and St= 7.84028973E-03 7.84028973E-03

pi-n/pi-s for In and St= 0.248883650 0.248884529

Ratio of these= 0.999996483

**Neutral Fst for whole population= 0.15**

**Scaled migration rate for whole population= 5.667**

Zone 1: quasi-neutral zone 1

Upper bound scaled selection coefficient for neutrality in St metapopulation= 0.250000000

Probability of zone 1= 7.93938339E-02

Integral of selection coefficient over zone 1= 4.58041381E-04

Mean load statistics for zone 1

Mean q1 and q2= 0.600000024

F1 and F2= 0.983700812 0.983700812

Diversities= 7.82360975E-03 7.82360975E-03

Contributions to loads within In and St= 2.73928948E-04 2.73928948E-04

Contributions to load between In and St = 2.19859867E-04

Contributions to homozygous loads for In and St= 2.74824852E-04 2.74824852E-04

Contributions to inbreeding loads for In and St= 8.95895255E-07 8.95895255E-07

Contributions to selection coefficients for In and St homokaryotypes

5.40614128E-05 5.40614128E-05

Contributions to mean A2 freqs= 4.76363041E-02 4.76363041E-02

Contributions to mean diversities= 6.21146348E-04 6.21146348E-04

Zone 2: quasi-neutral zone 2

Lower and upper bounds of St metapopn gamma for zone 2

0.250000000 50.0000000

Probability of zone 2= 0.307079256

Coefficients for bivariate distribution of q1 and q2 in metapopulation

G1= 0.352941185 G2= 0.352941185

G3= 0.294117630 G4= 0.294117630

a1= 0.169117644 a2= 0.169117644

b11= 1.83823518E-02 b12= 0.125000000 b22= 1.83823518E-02

Contributions to mean load statistics for zone 2

Loads within In and St= 4.94590681E-03 4.94590634E-03

Load between In and St= 3.26844095E-03

Homozygous load for In and St= 5.12504298E-03 5.12504298E-03

Inbreeding loads= 1.79138253E-04 1.79138224E-04

Selection coefficients against In and St homokaryotypes

1.67608261E-03 1.67608261E-03

Contributions to mean A2 freqs= 3.82650159E-02 3.82650159E-02

Contributions to mean diversities= 1.24040071E-03 1.24040048E-03

Zone 3: moderate selection zone

Lower and upper bounds of St metapopn gamma for zone 3

50.0000000 100.000000

Probability of zone 3= 8.60972703E-02

Contributions to mean load statistics over zone 3

Loads within In and St= 1.22401034E-04 1.22412879E-04

Load between In and St= 9.76314986E-05

Homozygous load for In and St= 1.95191198E-04 1.95207598E-04

Inbreeding loads= 7.27901206E-05 7.27946463E-05

Selection coefficients for In and St homokaryotypes

2.47955322E-05 2.47955322E-05

Contributions to mean A2 freqs= 2.79489268E-05 2.79522064E-05

Contributions to mean diversities= 4.16623516E-05 4.16670518E-05

Zone 4: strong selection zone

Upper limit to scaled gamma for St metapopn= 2.50000000

Lower limit to gamma for St metapopn= 100.000000

Upper limit to gamma for St metapopn= 4166.66699

Probability of zone 4= 0.515572548

Contributions to mean load statistics over zone 4

Loads within In and St= 5.83333604E-04 5.83333662E-04

Load between In and St= 4.83829150E-04

Homozygous load for In and St= 9.67554108E-04 9.67554108E-04

Inbreeding loads= 3.84219951E-04 3.84219922E-04

Selection coefficients for In and St homokaryotypes

9.94801521E-05 9.94801521E-05

Contributions to mean A2 freqs= 5.58334323E-05 5.58334323E-05

Contributions to mean diversities= 1.08361053E-06 1.08361053E-06

Mean load statistics over all zones

Loads within In and St= 5.92557015E-03 5.92558179E-03

Load between In and St= 4.06976137E-03

Homozygous load for In and St= 6.56261342E-03 6.56262971E-03

Inbreeding loads= 6.37044199E-04 6.37048681E-04

Selection coefficients for In and St homokaryotypes

1.85406208E-03 1.85412169E-03

Mean frequencies of A2 in In and St= 8.59851018E-02 8.59851092E-02

Ratio of these= 0.999999940

Mean diversities at selected sites in In and St= 1.90429308E-03 1.90429750E-03

Ratio of these= 0.999997675

Mean diversities at neutral sites in In and St= 7.82360975E-03 7.82360975E-03

pi-n/pi-s for In and St= 0.243403390 0.243403941

Ratio of these= 0.999997735

**Neutral Fst for whole population= 0.2**

**Scaled migration rate for whole population= 4**

Zone 1: quasi-neutral zone 1

Upper bound scaled selection coefficient for neutrality in St metapopulation= 0.250000000

Probability of zone 1= 7.93938339E-02

Integral of selection coefficient over zone 1= 4.58041381E-04

Mean load statistics for zone 1

Mean q1 and q2= 0.600000024

F1 and F2= 0.983739853 0.983739853

Diversities= 7.80487061E-03 7.80487061E-03

Contributions to loads within In and St= 2.73931102E-04 2.73931102E-04

Contributions to load between In and St = 2.19859867E-04

Contributions to homozygous loads for In and St= 2.74824852E-04 2.74824852E-04

Contributions to inbreeding loads for In and St= 8.93738445E-07 8.93738445E-07

Contributions to selection coefficients for In and St homokaryotypes

5.40614128E-05 5.40614128E-05

Contributions to mean A2 freqs= 4.76363041E-02 4.76363041E-02

Contributions to mean diversities= 6.19658618E-04 6.19658618E-04

Zone 2: quasi-neutral zone 2

Lower and upper bounds of St metapopn gamma for zone 2

0.250000000 50.0000000

Probability of zone 2= 0.307079256

Coefficients for bivariate distribution of q1 and q2 in metapopulation

G1= 0.500000000 G2= 0.500000000

G3= 0.00000000 G4= 0.00000000

a1= 0.187500000 a2= 0.187500000

b11= 0.00000000 b12= 0.125000000 b22= 0.00000000

Contributions to mean load statistics for zone 2

Loads within In and St= 5.03039779E-03 5.03039779E-03

Load between In and St= 3.32877296E-03

Homozygous load for In and St= 5.19781699E-03 5.19781699E-03

Inbreeding loads= 1.67423525E-04 1.67423554E-04

Selection coefficients against In and St homokaryotypes

1.70016289E-03 1.70016289E-03

Contributions to mean A2 freqs= 3.87905911E-02 3.87905911E-02

Contributions to mean diversities= 1.19756383E-03 1.19756383E-03

Zone 3: moderate selection zone

Lower and upper bounds of St metapopn gamma for zone 3

50.0000000 100.000000

Probability of zone 3= 8.60972703E-02

Contributions to mean load statistics over zone 3

Loads within In and St= 1.28237836E-04 1.28251195E-04

Load between In and St= 9.70512847E-05

Homozygous load for In and St= 1.94032429E-04 1.94047621E-04

Inbreeding loads= 6.57943092E-05 6.57961646E-05

Selection coefficients for In and St homokaryotypes

3.11732292E-05 3.11732292E-05

Contributions to mean A2 freqs= 2.77773561E-05 2.77803938E-05

Contributions to mean diversities= 3.76404860E-05 3.76436110E-05

Zone 4: strong selection zone

Upper limit to scaled gamma for St metapopn= 2.50000000

Lower limit to gamma for St metapopn= 100.000000

Upper limit to gamma for St metapopn= 4166.66699

Probability of zone 4= 0.515572548

Contributions to mean load statistics over zone 4

Loads within In and St= 6.23103289E-04 6.23103406E-04

Load between In and St= 4.98781505E-04

Homozygous load for In and St= 9.97452298E-04 9.97452298E-04

Inbreeding loads= 3.74348863E-04 3.74348863E-04

Selection coefficients for In and St homokaryotypes

1.24335289E-04 1.24335289E-04

Contributions to mean A2 freqs= 5.76512102E-05 5.76512102E-05

Contributions to mean diversities= 1.15616001E-06 1.15616001E-06

Mean load statistics over all zones

Loads within In and St= 6.05567032E-03 6.05568336E-03

Load between In and St= 4.14446555E-03

Homozygous load for In and St= 6.66412618E-03 6.66414201E-03

Inbreeding loads= 6.08460396E-04 6.08462316E-04

Selection coefficients for In and St homokaryotypes

1.90937519E-03 1.90937519E-03

Mean frequencies of A2 in In and St= 8.65123272E-02 8.65123346E-02

Ratio of these= 0.999999940

Mean diversities at selected sites in In and St= 1.85601902E-03 1.85602217E-03

Ratio of these= 0.999998331

Mean diversities at neutral sites in In and St= 7.80487061E-03 7.80487061E-03

pi-n/pi-s for In and St= 0.237802669 0.237803072

Ratio of these= 0.999998331

**Neutral Fst for whole population= 0.25**

**Scaled migration rate for whole population= 3**

Zone 1: quasi-neutral zone 1

Upper bound scaled selection coefficient for neutrality in St metapopulation= 0.250000000

Probability of zone 1= 7.93938339E-02

Integral of selection coefficient over zone 1= 4.58041381E-04

Mean load statistics for zone 1

Mean q1 and q2= 0.600000024

F1 and F2= 0.983783841 0.983783841

Diversities= 7.78375613E-03 7.78375613E-03

Contributions to loads within In and St= 2.73933518E-04 2.73933518E-04

Contributions to load between In and St = 2.19859867E-04

Contributions to homozygous loads for In and St= 2.74824852E-04 2.74824852E-04

Contributions to inbreeding loads for In and St= 8.91308616E-07 8.91308616E-07

Contributions to selection coefficients for In and St homokaryotypes

5.40614128E-05 5.40614128E-05

Contributions to mean A2 freqs= 4.76363041E-02 4.76363041E-02

Contributions to mean diversities= 6.17982238E-04 6.17982238E-04

Zone 2: quasi-neutral zone 2

Lower and upper bounds of St metapopn gamma for zone 2

0.250000000 50.0000000

Probability of zone 2= 0.307079256

Coefficients for bivariate distribution of q1 and q2 in metapopulation

G1= 0.666666687 G2= 0.666666687

G3= -0.333333373 G4= -0.333333373

a1= 0.208333343 a2= 0.208333343

b11= -2.08333358E-02 b12= 0.125000000 b22= -2.08333358E-02

Contributions to mean load statistics for zone 2

Loads within In and St= 5.12163574E-03 5.12163574E-03

Load between In and St= 3.39641818E-03

Homozygous load for In and St= 5.27741015E-03 5.27741015E-03

Inbreeding loads= 1.55779897E-04 1.55779882E-04

Selection coefficients against In and St homokaryotypes

1.72370672E-03 1.72370672E-03

Contributions to mean A2 freqs= 3.93832959E-02 3.93832959E-02

Contributions to mean diversities= 1.15287490E-03 1.15287467E-03

Zone 3: moderate selection zone

Lower and upper bounds of St metapopn gamma for zone 3

50.0000000 100.000000

Probability of zone 3= 8.60972703E-02

Contributions to mean load statistics over zone 3

Loads within In and St= 1.34089685E-04 1.34104252E-04

Load between In and St= 9.69051835E-05

Homozygous load for In and St= 1.93740416E-04 1.93754982E-04

Inbreeding loads= 5.96509090E-05 5.96508908E-05

Selection coefficients for In and St homokaryotypes

3.71932983E-05 3.71932983E-05

Contributions to mean A2 freqs= 2.77257550E-05 2.77286672E-05

Contributions to mean diversities= 3.41035156E-05 3.41052328E-05

Zone 4: strong selection zone

Upper limit to scaled gamma for St metapopn= 2.50000000

Lower limit to gamma for St metapopn= 100.000000

Upper limit to gamma for St metapopn= 4166.66699

Probability of zone 4= 0.515572548

Contributions to mean load statistics over zone 4

Loads within In and St= 6.61033846E-04 6.61033846E-04

Load between In and St= 5.13083709E-04

Homozygous load for In and St= 1.02605135E-03 1.02605147E-03

Inbreeding loads= 3.65015469E-04 3.65015469E-04

Selection coefficients for In and St homokaryotypes

1.47938728E-04 1.47938728E-04

Contributions to mean A2 freqs= 5.94461853E-05 5.94461853E-05

Contributions to mean diversities= 1.22484209E-06 1.22484187E-06

Mean load statistics over all zones

Loads within In and St= 6.19069254E-03 6.19070744E-03

Load between In and St= 4.22626687E-03

Homozygous load for In and St= 6.77202642E-03 6.77204179E-03

Inbreeding loads= 5.81337605E-04 5.81337546E-04

Selection coefficients for In and St homokaryotypes

1.96248293E-03 1.96248293E-03

Mean frequencies of A2 in In and St= 8.71067718E-02 8.71067792E-02

Ratio of these= 0.999999940

Mean diversities at selected sites in In and St= 1.80618546E-03 1.80618698E-03

Ratio of these= 0.999999166

Mean diversities at neutral sites in In and St= 7.78375613E-03 7.78375613E-03

pi-n/pi-s for In and St= 0.232045487 0.232045680

Ratio of these= 0.999999166

**h= 0.45**

**Neutral Fst for whole population= 0.05**

**Scaled migration rate for whole population= 19**

Zone 1: quasi-neutral zone 1

Upper bound scaled selection coefficient for neutrality in St metapopulation= 0.250000000

Probability of zone 1= 7.93938339E-02

Integral of selection coefficient over zone 1= 4.58041381E-04

Mean load statistics for zone 1

Mean q1 and q2= 0.600000024

F1 and F2= 0.983634770 0.983634770

Diversities= 7.85531010E-03 7.85531010E-03

Contributions to loads within In and St= 2.74644961E-04 2.74644961E-04

Contributions to load between In and St = 2.63831811E-04

Contributions to homozygous loads for In and St= 2.74824852E-04 2.74824852E-04

Contributions to inbreeding loads for In and St= 1.79888886E-07 1.79888886E-07

Contributions to selection coefficients for In and St homokaryotypes

1.07884407E-05 1.07884407E-05

Contributions to mean A2 freqs= 4.76363041E-02 4.76363041E-02

Contributions to mean diversities= 6.23663189E-04 6.23663189E-04

Zone 2: quasi-neutral zone 2

Lower and upper bounds of St metapopn gamma for zone 2

0.250000000 50.0000000

Probability of zone 2= 0.307079256

Coefficients for bivariate distribution of q1 and q2 in metapopulation

G1= 0.105263159 G2= 0.105263159

G3= 0.789473653 G4= 0.789473653

a1= 0.227631569 a2= 0.227631569

b11= 9.86842345E-03 b12= 2.50000060E-02 b22= 9.86842345E-03

Contributions to mean load statistics for zone 2

Loads within In and St= 3.95016233E-03 3.95016233E-03

Load between In and St= 3.69976205E-03

Homozygous load for In and St= 3.97788314E-03 3.97788361E-03

Inbreeding loads= 2.77245545E-05 2.77245545E-05

Selection coefficients against In and St homokaryotypes

2.50339508E-04 2.50339508E-04

Contributions to mean A2 freqs= 3.53724286E-02 3.53724286E-02

Contributions to mean diversities= 1.09396561E-03 1.09396584E-03

Zone 3: moderate selection zone

Lower and upper bounds of St metapopn gamma for zone 3

50.0000000 100.000000

Probability of zone 3= 8.60972703E-02

Contributions to mean load statistics over zone 3

Loads within In and St= 1.06716012E-04 1.06725674E-04

Load between In and St= 1.05609673E-04

Homozygous load for In and St= 1.17336167E-04 1.17347088E-04

Inbreeding loads= 1.06200250E-05 1.06212747E-05

Selection coefficients for In and St homokaryotypes

1.13248825E-06 1.13248825E-06

Contributions to mean A2 freqs= 1.68255956E-05 1.68277784E-05

Contributions to mean diversities= 3.04500882E-05 3.04544537E-05

Zone 4: strong selection zone

Upper limit to scaled gamma for St metapopn= 2.50000000

Lower limit to gamma for St metapopn= 100.000000

Upper limit to gamma for St metapopn= 4166.66699

Probability of zone 4= 0.515572548

Contributions to mean load statistics over zone 4

Loads within In and St= 4.32656292E-04 4.32656292E-04

Load between In and St= 4.28615836E-04

Homozygous load for In and St= 4.76237037E-04 4.76237037E-04

Inbreeding loads= 4.35807997E-05 4.35807997E-05

Selection coefficients for In and St homokaryotypes

4.05311584E-06 4.05311584E-06

Contributions to mean A2 freqs= 2.67648520E-05 2.67648520E-05

Contributions to mean diversities= 4.73131195E-07 4.73131223E-07

Mean load statistics over all zones

Loads within In and St= 4.76417970E-03 4.76418948E-03

Load between In and St= 4.49781958E-03

Homozygous load for In and St= 4.84628091E-03 4.84629255E-03

Inbreeding loads= 8.21052672E-05 8.21065187E-05

Selection coefficients for In and St homokaryotypes

2.66313553E-04 2.66313553E-04

Mean frequencies of A2 in In and St= 8.30523223E-02 8.30523297E-02

Ratio of these= 0.999999881

Mean diversities at selected sites in In and St= 1.74855196E-03 1.74855662E-03

Ratio of these= 0.999997318

Mean diversities at neutral sites in In and St= 7.85531010E-03 7.85531010E-03

pi-n/pi-s for In and St= 0.222594902 0.222595498

Ratio of these= 0.999997318

**Neutral Fst for whole population= 0.1**

**Scaled migration rate for whole population= 9**

Zone 1: quasi-neutral zone 1

Upper bound scaled selection coefficient for neutrality in St metapopulation= 0.250000000

Probability of zone 1= 7.93938339E-02

Integral of selection coefficient over zone 1= 4.58041381E-04

Mean load statistics for zone 1

Mean q1 and q2= 0.600000024

F1 and F2= 0.983666062 0.983666062

Diversities= 7.84028973E-03 7.84028973E-03

Contributions to loads within In and St= 2.74645281E-04 2.74645281E-04

Contributions to load between In and St = 2.63831811E-04

Contributions to homozygous loads for In and St= 2.74824852E-04 2.74824852E-04

Contributions to inbreeding loads for In and St= 1.79561269E-07 1.79561269E-07

Contributions to selection coefficients for In and St homokaryotypes

1.07884407E-05 1.07884407E-05

Contributions to mean A2 freqs= 4.76363041E-02 4.76363041E-02

Contributions to mean diversities= 6.22470689E-04 6.22470689E-04

Zone 2: quasi-neutral zone 2

Lower and upper bounds of St metapopn gamma for zone 2

0.250000000 50.0000000

Probability of zone 2= 0.307079256

Coefficients for bivariate distribution of q1 and q2 in metapopulation

G1= 0.222222224 G2= 0.222222224

G3= 0.555555582 G4= 0.555555582

a1= 0.230555549 a2= 0.230555549

b11= 6.94444636E-03 b12= 2.50000060E-02 b22= 6.94444636E-03

Contributions to mean load statistics for zone 2

Loads within In and St= 3.98819987E-03 3.98819987E-03

Load between In and St= 3.73441190E-03

Homozygous load for In and St= 4.01579123E-03 4.01579123E-03

Inbreeding loads= 2.75912298E-05 2.75912298E-05

Selection coefficients against In and St homokaryotypes

2.53736973E-04 2.53736973E-04

Contributions to mean A2 freqs= 3.54696475E-02 3.54696475E-02

Contributions to mean diversities= 1.09074113E-03 1.09074102E-03

Zone 3: moderate selection zone

Lower and upper bounds of St metapopn gamma for zone 3

50.0000000 100.000000

Probability of zone 3= 8.60972703E-02

Contributions to mean load statistics over zone 3

Loads within In and St= 1.14018600E-04 1.14028371E-04

Load between In and St= 1.11836009E-04

Homozygous load for In and St= 1.24254191E-04 1.24264581E-04

Inbreeding loads= 1.02355907E-05 1.02362146E-05

Selection coefficients for In and St homokaryotypes

2.20537186E-06 2.20537186E-06

Contributions to mean A2 freqs= 1.78246755E-05 1.78267546E-05

Contributions to mean diversities= 2.93524718E-05 2.93560606E-05

Zone 4: strong selection zone

Upper limit to scaled gamma for St metapopn= 2.50000000

Lower limit to gamma for St metapopn= 100.000000

Upper limit to gamma for St metapopn= 4166.66699

Probability of zone 4= 0.515572548

Contributions to mean load statistics over zone 4

Loads within In and St= 4.80464310E-04 4.80464310E-04

Load between In and St= 4.73092543E-04

Homozygous load for In and St= 5.25654585E-04 5.25654643E-04

Inbreeding loads= 4.51904889E-05 4.51904889E-05

Selection coefficients for In and St homokaryotypes

7.39097595E-06 7.39097595E-06

Contributions to mean A2 freqs= 2.98147661E-05 2.98147661E-05

Contributions to mean diversities= 5.55892655E-07 5.55892655E-07

Mean load statistics over all zones

Loads within In and St= 4.85732779E-03 4.85733757E-03

Load between In and St= 4.58317250E-03

Homozygous load for In and St= 4.94052516E-03 4.94053541E-03

Inbreeding loads= 8.31968719E-05 8.31974903E-05

Selection coefficients for In and St homokaryotypes

2.74121761E-04 2.74121761E-04

Mean frequencies of A2 in In and St= 8.31535906E-02 8.31535980E-02

Ratio of these= 0.999999881

Mean diversities at selected sites in In and St= 1.74312026E-03 1.74312363E-03

Ratio of these= 0.999998093

Mean diversities at neutral sites in In and St= 7.84028973E-03 7.84028973E-03

pi-n/pi-s for In and St= 0.222328559 0.222328976

Ratio of these= 0.999998152

**Neutral Fst for whole population= 0.15**

**Scaled migration rate for whole population= 5.667**

Zone 1: quasi-neutral zone 1

Upper bound scaled selection coefficient for neutrality in St metapopulation= 0.250000000

Probability of zone 1= 7.93938339E-02

Integral of selection coefficient over zone 1= 4.58041381E-04

Mean load statistics for zone 1

Mean q1 and q2= 0.600000024

F1 and F2= 0.983700812 0.983700812

Diversities= 7.82360975E-03 7.82360975E-03

Contributions to loads within In and St= 2.74645659E-04 2.74645659E-04

Contributions to load between In and St = 2.63831811E-04

Contributions to homozygous loads for In and St= 2.74824852E-04 2.74824852E-04

Contributions to inbreeding loads for In and St= 1.79179054E-07 1.79179054E-07

Contributions to selection coefficients for In and St homokaryotypes

1.07884407E-05 1.07884407E-05

Contributions to mean A2 freqs= 4.76363041E-02 4.76363041E-02

Contributions to mean diversities= 6.21146348E-04 6.21146348E-04

Zone 2: quasi-neutral zone 2

Lower and upper bounds of St metapopn gamma for zone 2

0.250000000 50.0000000

Probability of zone 2= 0.307079256

Coefficients for bivariate distribution of q1 and q2 in metapopulation

G1= 0.352941185 G2= 0.352941185

G3= 0.294117630 G4= 0.294117630

a1= 0.233823523 a2= 0.233823523

b11= 3.67647130E-03 b12= 2.50000060E-02 b22= 3.67647130E-03

Contributions to mean load statistics for zone 2

Loads within In and St= 4.03018668E-03 4.03018668E-03

Load between In and St= 3.77267110E-03

Homozygous load for In and St= 4.05762671E-03 4.05762717E-03

Inbreeding loads= 2.74403337E-05 2.74403483E-05

Selection coefficients against In and St homokaryotypes

2.57492065E-04 2.57492065E-04

Contributions to mean A2 freqs= 3.55774947E-02 3.55774947E-02

Contributions to mean diversities= 1.08708756E-03 1.08708837E-03

Zone 3: moderate selection zone

Lower and upper bounds of St metapopn gamma for zone 3

50.0000000 100.000000

Probability of zone 3= 8.60972703E-02

Contributions to mean load statistics over zone 3

Loads within In and St= 1.21162993E-04 1.21172568E-04

Load between In and St= 1.17902942E-04

Homozygous load for In and St= 1.30995191E-04 1.31005218E-04

Inbreeding loads= 9.83213522E-06 9.83258906E-06

Selection coefficients for In and St homokaryotypes

3.27825546E-06 3.27825546E-06

Contributions to mean A2 freqs= 1.88004124E-05 1.88024187E-05

Contributions to mean diversities= 2.81997982E-05 2.82025794E-05

Zone 4: strong selection zone

Upper limit to scaled gamma for St metapopn= 2.50000000

Lower limit to gamma for St metapopn= 100.000000

Upper limit to gamma for St metapopn= 4166.66699

Probability of zone 4= 0.515572548

Contributions to mean load statistics over zone 4

Loads within In and St= 5.11497259E-04 5.11497201E-04

Load between In and St= 5.01191826E-04

Homozygous load for In and St= 5.56876999E-04 5.56876999E-04

Inbreeding loads= 4.53796238E-05 4.53796274E-05

Selection coefficients for In and St homokaryotypes

1.03116035E-05 1.03116035E-05

Contributions to mean A2 freqs= 3.17602498E-05 3.17602498E-05

Contributions to mean diversities= 6.16760531E-07 6.16760531E-07

Mean load statistics over all zones

Loads within In and St= 4.93749278E-03 4.93750209E-03

Load between In and St= 4.65559773E-03

Homozygous load for In and St= 5.02032368E-03 5.02033439E-03

Inbreeding loads= 8.28312768E-05 8.28317425E-05

Selection coefficients for In and St homokaryotypes

2.81870365E-04 2.81870365E-04

Mean frequencies of A2 in In and St= 8.32643583E-02 8.32643658E-02

Ratio of these= 0.999999881

Mean diversities at selected sites in In and St= 1.73705048E-03 1.73705397E-03

Ratio of these= 0.999997973

Mean diversities at neutral sites in In and St= 7.82360975E-03 7.82360975E-03

pi-n/pi-s for In and St= 0.222026736 0.222027183

Ratio of these= 0.999997973

**Neutral Fst for whole population= 0.2**

**Scaled migration rate for whole population= 4**

Zone 1: quasi-neutral zone 1

Upper bound scaled selection coefficient for neutrality in St metapopulation= 0.250000000

Probability of zone 1= 7.93938339E-02

Integral of selection coefficient over zone 1= 4.58041381E-04

Mean load statistics for zone 1

Mean q1 and q2= 0.600000024

F1 and F2= 0.983739853 0.983739853

Diversities= 7.80487061E-03 7.80487061E-03

Contributions to loads within In and St= 2.74646096E-04 2.74646096E-04

Contributions to load between In and St = 2.63831811E-04

Contributions to homozygous loads for In and St= 2.74824852E-04 2.74824852E-04

Contributions to inbreeding loads for In and St= 1.78742226E-07 1.78742226E-07

Contributions to selection coefficients for In and St homokaryotypes

1.07884407E-05 1.07884407E-05

Contributions to mean A2 freqs= 4.76363041E-02 4.76363041E-02

Contributions to mean diversities= 6.19658618E-04 6.19658618E-04

Zone 2: quasi-neutral zone 2

Lower and upper bounds of St metapopn gamma for zone 2

0.250000000 50.0000000

Probability of zone 2= 0.307079256

Coefficients for bivariate distribution of q1 and q2 in metapopulation

G1= 0.500000000 G2= 0.500000000

G3= 0.00000000 G4= 0.00000000

a1= 0.237499997 a2= 0.237499997

b11= 0.00000000 b12= 2.50000060E-02 b22= 0.00000000

Contributions to mean load statistics for zone 2

Loads within In and St= 4.07676818E-03 4.07676818E-03

Load between In and St= 3.81510425E-03

Homozygous load for In and St= 4.10402985E-03 4.10402892E-03

Inbreeding loads= 2.72683174E-05 2.72683155E-05

Selection coefficients against In and St homokaryotypes

2.61604786E-04 2.61604786E-04

Contributions to mean A2 freqs= 3.56978439E-02 3.56978439E-02

Contributions to mean diversities= 1.08292408E-03 1.08292408E-03

Zone 3: moderate selection zone

Lower and upper bounds of St metapopn gamma for zone 3

50.0000000 100.000000

Probability of zone 3= 8.60972703E-02

Contributions to mean load statistics over zone 3

Loads within In and St= 1.28271393E-04 1.28281099E-04

Load between In and St= 1.23923324E-04

Homozygous load for In and St= 1.37684227E-04 1.37694078E-04

Inbreeding loads= 9.41262897E-06 9.41276903E-06

Selection coefficients for In and St homokaryotypes

4.35113907E-06 4.35113907E-06

Contributions to mean A2 freqs= 1.97699064E-05 1.97718782E-05

Contributions to mean diversities= 2.69999528E-05 2.70018718E-05

Zone 4: strong selection zone

Upper limit to scaled gamma for St metapopn= 2.50000000

Lower limit to gamma for St metapopn= 100.000000

Upper limit to gamma for St metapopn= 4166.66699

Probability of zone 4= 0.515572548

Contributions to mean load statistics over zone 4

Loads within In and St= 5.38352935E-04 5.38352935E-04

Load between In and St= 5.25265001E-04

Homozygous load for In and St= 5.83623711E-04 5.83623711E-04

Inbreeding loads= 4.52708773E-05 4.52708773E-05

Selection coefficients for In and St homokaryotypes

1.31130219E-05 1.31130219E-05

Contributions to mean A2 freqs= 3.34950855E-05 3.34950855E-05

Contributions to mean diversities= 6.72029216E-07 6.72029216E-07

Mean load statistics over all zones

Loads within In and St= 5.01803868E-03 5.01804845E-03

Load between In and St= 4.72812401E-03

Homozygous load for In and St= 5.10016270E-03 5.10017155E-03

Inbreeding loads= 8.21305657E-05 8.21307040E-05

Selection coefficients for In and St homokaryotypes

2.89857388E-04 2.89857388E-04

Mean frequencies of A2 in In and St= 8.33874121E-02 8.33874196E-02

Ratio of these= 0.999999940

Mean diversities at selected sites in In and St= 1.73025473E-03 1.73025660E-03

Ratio of these= 0.999998927

Mean diversities at neutral sites in In and St= 7.80487061E-03 7.80487061E-03

pi-n/pi-s for In and St= 0.221689105 0.221689343

Ratio of these= 0.999998927

**Neutral Fst for whole population= 0.25**

**Scaled migration rate for whole population= 3**

Zone 1: quasi-neutral zone 1

Upper bound scaled selection coefficient for neutrality in St metapopulation= 0.250000000

Probability of zone 1= 7.93938339E-02

Integral of selection coefficient over zone 1= 4.58041381E-04

Mean load statistics for zone 1

Mean q1 and q2= 0.600000024

F1 and F2= 0.983783841 0.983783841

Diversities= 7.78375613E-03 7.78375613E-03

Contributions to loads within In and St= 2.74646591E-04 2.74646591E-04

Contributions to load between In and St = 2.63831811E-04

Contributions to homozygous loads for In and St= 2.74824852E-04 2.74824852E-04

Contributions to inbreeding loads for In and St= 1.78250801E-07 1.78250801E-07

Contributions to selection coefficients for In and St homokaryotypes

1.07884407E-05 1.07884407E-05

Contributions to mean A2 freqs= 4.76363041E-02 4.76363041E-02

Contributions to mean diversities= 6.17982238E-04 6.17982238E-04

Zone 2: quasi-neutral zone 2

Lower and upper bounds of St metapopn gamma for zone 2

0.250000000 50.0000000

Probability of zone 2= 0.307079256

Coefficients for bivariate distribution of q1 and q2 in metapopulation

G1= 0.666666687 G2= 0.666666687

G3= -0.333333373 G4= -0.333333373

a1= 0.241666660 a2= 0.241666660

b11= -4.16666828E-03 b12= 2.50000060E-02 b22= -4.16666828E-03

Contributions to mean load statistics for zone 2

Loads within In and St= 4.12870431E-03 4.12870385E-03

Load between In and St= 3.86242964E-03

Homozygous load for In and St= 4.15577600E-03 4.15577507E-03

Inbreeding loads= 2.70706751E-05 2.70706678E-05

Selection coefficients against In and St homokaryotypes

2.66253948E-04 2.66253948E-04

Contributions to mean A2 freqs= 3.58329117E-02 3.58329117E-02

Contributions to mean diversities= 1.07813359E-03 1.07813312E-03

Zone 3: moderate selection zone

Lower and upper bounds of St metapopn gamma for zone 3

50.0000000 100.000000

Probability of zone 3= 8.60972703E-02

Contributions to mean load statistics over zone 3

Loads within In and St= 1.35470706E-04 1.35480703E-04

Load between In and St= 1.30014538E-04

Homozygous load for In and St= 1.44451886E-04 1.44461752E-04

Inbreeding loads= 8.98107555E-06 8.98095186E-06

Selection coefficients for In and St homokaryotypes

5.48362732E-06 5.48362732E-06

Contributions to mean A2 freqs= 2.07508583E-05 2.07528337E-05

Contributions to mean diversities= 2.57636075E-05 2.57646243E-05

Zone 4: strong selection zone

Upper limit to scaled gamma for St metapopn= 2.50000000

Lower limit to gamma for St metapopn= 100.000000

Upper limit to gamma for St metapopn= 4166.66699

Probability of zone 4= 0.515572548

Contributions to mean load statistics over zone 4

Loads within In and St= 5.64342481E-04 5.64342481E-04

Load between In and St= 5.48489625E-04

Homozygous load for In and St= 6.09428214E-04 6.09428214E-04

Inbreeding loads= 4.50855841E-05 4.50855841E-05

Selection coefficients for In and St homokaryotypes

1.58548355E-05 1.58548355E-05

Contributions to mean A2 freqs= 3.52474854E-05 3.52474854E-05

Contributions to mean diversities= 7.26587530E-07 7.26587530E-07

Mean load statistics over all zones

Loads within In and St= 5.10316389E-03 5.10317367E-03

Load between In and St= 4.80476581E-03

Homozygous load for In and St= 5.18448092E-03 5.18448977E-03

Inbreeding loads= 8.13155857E-05 8.13154547E-05

Selection coefficients for In and St homokaryotypes

2.98380852E-04 2.98380852E-04

Mean frequencies of A2 in In and St= 8.35252106E-02 8.35252106E-02

Ratio of these= 1.00000000

Mean diversities at selected sites in In and St= 1.72260602E-03 1.72260648E-03

Ratio of these= 0.999999702

Mean diversities at neutral sites in In and St= 7.78375613E-03 7.78375613E-03

pi-n/pi-s for In and St= 0.221307814 0.221307874

Ratio of these= 0.999999702

**Section 2**

**Mean selection coefficient= 2.0 E-03**

**Mean scaled selection coefficient for whole metapopulation= 4000**

**Inversion frequency= 0.1**

**h= 0.05**

**Neutral Fst for whole population= 0.05**

**Scaled migration rate for whole population= 19**

Zone 1: quasi-neutral zone 1

Upper bound scaled selection coefficient for neutrality in St metapopulation= 0.250000000

Probability of zone 1= 4.39136960E-02

Integral of selection coefficient over zone 1= 1.40749034E-04

Mean load statistics for zone 1

Mean q1 and q2= 0.600000024

F1 and F2= 0.996683598 0.970923305

Diversities= 1.59187312E-03 1.39568131E-02

Contributions to loads within In and St= 8.43486050E-05 8.35654428E-05

Contributions to load between In and St = 5.40476285E-05

Contributions to homozygous loads for In and St= 8.44494207E-05 8.44494207E-05

Contributions to inbreeding loads for In and St= 1.00822561E-07 8.83980135E-07

Contributions to selection coefficients for In and St homokaryotypes

3.02791595E-05 2.95042992E-05

Contributions to mean A2 freqs= 2.63482183E-02 2.63482183E-02

Contributions to mean diversities= 6.99050288E-05 6.12895237E-04

Zone 2: quasi-neutral zone 2

Lower and upper bounds of St metapopn gamma for zone 2

0.250000000 50.0000000

Probability of zone 2= 0.171113729

Coefficients for bivariate distribution of q1 and q2 in metapopulation

G1= 0.526315808 G2= 5.84795326E-02

G3= -5.26316166E-02 G4= 0.883040905

a1= 9.73684248E-03 a2= 8.76315832E-02

b11= -2.36842272E-04 b12= 8.09999928E-02 b22= 0.321868390

Contributions to mean load statistics for zone 2

Loads within In and St= 8.04773048E-02 1.01727399E-03

Load between In and St= 4.76767123E-03

Homozygous load for In and St= 8.05755183E-02 1.27783057E-03

Inbreeding loads= 9.82725469E-05 2.60555127E-04

Selection coefficients against In and St homokaryotypes

7.29146600E-02 -3.75747681E-03

Contributions to mean A2 freqs= 0.100803070 1.71919968E-02

Contributions to mean diversities= 2.72233941E-04 1.49213464E-03

Zone 3: moderate selection zone

Lower and upper bounds of St metapopn gamma for zone 3

50.0000000 499.999969

Probability of zone 3= 0.210339963

Contributions to mean load statistics over zone 3

Loads within In and St= 8.71419832E-02 1.88568010E-04

Load between In and St= 4.53678193E-03

Homozygous load for In and St= 8.80662724E-02 1.12587016E-03

Inbreeding loads= 9.24394641E-04 9.37301957E-04

Selection coefficients for In and St homokaryotypes

7.92854428E-02 -4.35769558E-03

Contributions to mean A2 freqs= 1.73966009E-02 1.34524496E-04

Contributions to mean diversities= 2.12894913E-04 2.52242782E-04

Zone 4: strong selection zone

Upper limit to scaled gamma for St metapopn= 2.50000000

Lower limit to gamma for St metapopn= 499.999969

Upper limit to gamma for St metapopn= 30000.0020

Probability of zone 4= 0.562775493

Contributions to mean load statistics over zone 4

Loads within In and St= 2.35566287E-03 6.17035490E-04

Load between In and St= 4.85073921E-04

Homozygous load for In and St= 5.49023645E-03 4.19630343E-03

Inbreeding loads= 3.13458219E-03 3.57926567E-03

Selection coefficients for In and St homokaryotypes

1.86884403E-03 1.31964684E-04

Contributions to mean A2 freqs= 1.05335326E-04 1.05335326E-04

Contributions to mean diversities= 4.43885114E-07 2.32459547E-06

Mean load statistics over all zones

Loads within In and St= 0.170059294 1.90644292E-03

Load between In and St= 9.84357484E-03

Homozygous load for In and St= 0.174216479 6.68445369E-03

Inbreeding loads= 4.15735040E-03 4.77800658E-03

Selection coefficients for In and St homokaryotypes

0.148039997 -7.96866417E-03

Mean frequencies of A2 in In and St= 0.144653216 4.37800772E-02

Ratio of these= 3.30408764

Mean diversities at selected sites in In and St= 5.55477804E-04 2.35959701E-03

Ratio of these= 0.235412151

Mean diversities at neutral sites in In and St= 1.59187312E-03 1.39568131E-02

pi-n/pi-s for In and St= 0.348946035 0.169064164

Ratio of these= 2.06398582

**Neutral Fst for whole population= 0.1**

**Scaled migration rate for whole population= 9**

Zone 1: quasi-neutral zone 1

Upper bound scaled selection coefficient for neutrality in St metapopulation= 0.250000000

Probability of zone 1= 4.39136960E-02

Integral of selection coefficient over zone 1= 1.40749034E-04

Mean load statistics for zone 1

Mean q1 and q2= 0.600000024

F1 and F2= 0.996689916 0.970978200

Diversities= 1.58884050E-03 1.39304632E-02

Contributions to loads within In and St= 8.43487869E-05 8.35671017E-05

Contributions to load between In and St = 5.40476285E-05

Contributions to homozygous loads for In and St= 8.44494207E-05 8.44494207E-05

Contributions to inbreeding loads for In and St= 1.00637997E-07 8.82319057E-07

Contributions to selection coefficients for In and St homokaryotypes

3.02791595E-05 2.95042992E-05

Contributions to mean A2 freqs= 2.63482183E-02 2.63482183E-02

Contributions to mean diversities= 6.97718569E-05 6.11738127E-04

Zone 2: quasi-neutral zone 2

Lower and upper bounds of St metapopn gamma for zone 2

0.250000000 50.0000000

Probability of zone 2= 0.171113729

Coefficients for bivariate distribution of q1 and q2 in metapopulation

G1= 1.11111104 G2= 0.123456798

G3= -1.22222209 G4= 0.753086388

a1= 1.49999997E-02 a2= 0.134999990

b11= -5.49999997E-03 b12= 8.09999928E-02 b22= 0.274499953

Contributions to mean load statistics for zone 2

Loads within In and St= 8.99873301E-02 1.03473314E-03

Load between In and St= 5.25013311E-03

Homozygous load for In and St= 9.00770426E-02 1.26136700E-03

Inbreeding loads= 8.97229475E-05 2.26634307E-04

Selection coefficients against In and St homokaryotypes

8.12463164E-02 -4.22430038E-03

Contributions to mean A2 freqs= 0.106893390 1.72987934E-02

Contributions to mean diversities= 2.59888475E-04 1.40089402E-03

Zone 3: moderate selection zone

Lower and upper bounds of St metapopn gamma for zone 3

50.0000000 499.999969

Probability of zone 3= 0.210339963

Contributions to mean load statistics over zone 3

Loads within In and St= 8.74119699E-02 1.95892280E-04

Load between In and St= 4.51263599E-03

Homozygous load for In and St= 8.81513357E-02 8.80527485E-04

Inbreeding loads= 7.39300100E-04 6.84635190E-04

Selection coefficients for In and St homokaryotypes

7.95562267E-02 -4.32610512E-03

Contributions to mean A2 freqs= 1.74211767E-02 1.05653045E-04

Contributions to mean diversities= 1.77804337E-04 1.87465324E-04

Zone 4: strong selection zone

Upper limit to scaled gamma for St metapopn= 2.50000000

Lower limit to gamma for St metapopn= 499.999969

Upper limit to gamma for St metapopn= 30000.0020

Probability of zone 4= 0.562775493

Contributions to mean load statistics over zone 4

Loads within In and St= 3.16667557E-03 7.20887037E-04

Load between In and St= 4.90605249E-04

Homozygous load for In and St= 5.66782383E-03 4.12947452E-03

Inbreeding loads= 2.50115152E-03 3.40858544E-03

Selection coefficients for In and St homokaryotypes

2.67249346E-03 2.30252743E-04

Contributions to mean A2 freqs= 1.04605220E-04 1.04605220E-04

Contributions to mean diversities= 4.92976767E-07 2.42163173E-06

Mean load statistics over all zones

Loads within In and St= 0.180650324 2.03507952E-03

Load between In and St= 1.03074210E-02

Homozygous load for In and St= 0.183980659 6.35581836E-03

Inbreeding loads= 3.33027518E-03 4.32073744E-03

Selection coefficients for In and St homokaryotypes

0.156624436 -8.30662251E-03

Mean frequencies of A2 in In and St= 0.150767386 4.38572727E-02

Ratio of these= 3.43768263

Mean diversities at selected sites in In and St= 5.07957651E-04 2.20251898E-03

Ratio of these= 0.230625778

Mean diversities at neutral sites in In and St= 1.58884050E-03 1.39304632E-02

pi-n/pi-s for In and St= 0.319703370 0.158108100

Ratio of these= 2.02205563

**Neutral Fst for whole population= 0.15**

**Scaled migration rate for whole population= 5.667**

Zone 1: quasi-neutral zone 1

Upper bound scaled selection coefficient for neutrality in St metapopulation= 0.250000000

Probability of zone 1= 4.39136960E-02

Integral of selection coefficient over zone 1= 1.40749034E-04

Mean load statistics for zone 1

Mean q1 and q2= 0.600000024

F1 and F2= 0.996697068 0.971039176

Diversities= 1.58540718E-03 1.39011955E-02

Contributions to loads within In and St= 8.43490125E-05 8.35689571E-05

Contributions to load between In and St = 5.40476285E-05

Contributions to homozygous loads for In and St= 8.44494207E-05 8.44494207E-05

Contributions to inbreeding loads for In and St= 1.00411484E-07 8.80465052E-07

Contributions to selection coefficients for In and St homokaryotypes

3.02791595E-05 2.95042992E-05

Contributions to mean A2 freqs= 2.63482183E-02 2.63482183E-02

Contributions to mean diversities= 6.96210918E-05 6.10452844E-04

Zone 2: quasi-neutral zone 2

Lower and upper bounds of St metapopn gamma for zone 2

0.250000000 50.0000000

Probability of zone 2= 0.171113729

Coefficients for bivariate distribution of q1 and q2 in metapopulation

G1= 1.76470590 G2= 0.196078435

G3= -2.52941179 G4= 0.607843161

a1= 2.08823532E-02 a2= 0.187941164

b11= -1.13823544E-02 b12= 8.09999928E-02 b22= 0.221558809

Contributions to mean load statistics for zone 2

Loads within In and St= 9.92458686E-02 1.05322606E-03

Load between In and St= 5.72116068E-03

Homozygous load for In and St= 9.93260071E-02 1.25120801E-03

Inbreeding loads= 8.00922426E-05 1.97980422E-04

Selection coefficients against In and St homokaryotypes

8.92844796E-02 -4.67884541E-03

Contributions to mean A2 freqs= 0.113005660 1.74238551E-02

Contributions to mean diversities= 2.45949544E-04 1.31541409E-03

Zone 3: moderate selection zone

Lower and upper bounds of St metapopn gamma for zone 3

50.0000000 499.999969

Probability of zone 3= 0.210339963

Contributions to mean load statistics over zone 3

Loads within In and St= 8.75925943E-02 2.07560079E-04

Load between In and St= 4.49831411E-03

Homozygous load for In and St= 8.82147029E-02 7.39581184E-04

Inbreeding loads= 6.22044085E-04 5.32021222E-04

Selection coefficients for In and St homokaryotypes

7.97356367E-02 -4.29999828E-03

Contributions to mean A2 freqs= 1.74408033E-02 8.79506915E-05

Contributions to mean diversities= 1.52876295E-04 1.47488929E-04

Zone 4: strong selection zone

Upper limit to scaled gamma for St metapopn= 2.50000000

Lower limit to gamma for St metapopn= 499.999969

Upper limit to gamma for St metapopn= 30000.0020

Probability of zone 4= 0.562775493

Contributions to mean load statistics over zone 4

Loads within In and St= 3.51924519E-03 7.61540199E-04

Load between In and St= 4.78515780E-04

Homozygous load for In and St= 5.63262030E-03 3.92374443E-03

Inbreeding loads= 2.11338396E-03 3.16220778E-03

Selection coefficients for In and St homokaryotypes

3.03608179E-03 2.83002853E-04

Contributions to mean A2 freqs= 1.01594582E-04 1.01594582E-04

Contributions to mean diversities= 5.10962650E-07 2.37661880E-06

Mean load statistics over all zones

Loads within In and St= 0.190442070 2.10589520E-03

Load between In and St= 1.07520372E-02

Homozygous load for In and St= 0.193257794 5.99898305E-03

Inbreeding loads= 2.81562074E-03 3.89308995E-03

Selection coefficients for In and St homokaryotypes

0.164470851 -8.68368149E-03

Mean frequencies of A2 in In and St= 0.156896263 4.39616181E-02

Ratio of these= 3.56893730

Mean diversities at selected sites in In and St= 4.68957907E-04 2.07573269E-03

Ratio of these= 0.225924030

Mean diversities at neutral sites in In and St= 1.58540718E-03 1.39011955E-02

pi-n/pi-s for In and St= 0.295796514 0.149320439

Ratio of these= 1.98095131

**Neutral Fst for whole population= 0.2**

**Scaled migration rate for whole population= 4**

Zone 1: quasi-neutral zone 1

Upper bound scaled selection coefficient for neutrality in St metapopulation= 0.250000000

Probability of zone 1= 4.39136960E-02

Integral of selection coefficient over zone 1= 1.40749034E-04

Mean load statistics for zone 1

Mean q1 and q2= 0.600000024

F1 and F2= 0.996705115 0.971107543

Diversities= 1.58154487E-03 1.38683794E-02

Contributions to loads within In and St= 8.43492453E-05 8.35710380E-05

Contributions to load between In and St = 5.40476285E-05

Contributions to homozygous loads for In and St= 8.44494207E-05 8.44494207E-05

Contributions to inbreeding loads for In and St= 1.00176585E-07 8.78384469E-07

Contributions to selection coefficients for In and St homokaryotypes

3.02791595E-05 2.95042992E-05

Contributions to mean A2 freqs= 2.63482183E-02 2.63482183E-02

Contributions to mean diversities= 6.94514820E-05 6.09011797E-04

Zone 2: quasi-neutral zone 2

Lower and upper bounds of St metapopn gamma for zone 2

0.250000000 50.0000000

Probability of zone 2= 0.171113729

Coefficients for bivariate distribution of q1 and q2 in metapopulation

G1= 2.50000000 G2= 0.277777791

G3= -4.00000000 G4= 0.444444418

a1= 2.75000017E-02 a2= 0.247500002

b11= -1.80000011E-02 b12= 8.09999928E-02 b22= 0.161999986

Contributions to mean load statistics for zone 2

Loads within In and St= 0.107608132 1.07335253E-03

Load between In and St= 6.14880538E-03

Homozygous load for In and St= 0.107678212 1.24685874E-03

Inbreeding loads= 7.00480523E-05 1.73506502E-04

Selection coefficients against In and St homokaryotypes

9.64820981E-02 -5.08832932E-03

Contributions to mean A2 freqs= 0.118836790 1.75701939E-02

Contributions to mean diversities= 2.31047496E-04 1.23443105E-03

Zone 3: moderate selection zone

Lower and upper bounds of St metapopn gamma for zone 3

50.0000000 499.999969

Probability of zone 3= 0.210339963

Contributions to mean load statistics over zone 3

Loads within In and St= 8.77281651E-02 2.19496098E-04

Load between In and St= 4.48934641E-03

Homozygous load for In and St= 8.82679597E-02 6.49959256E-04

Inbreeding loads= 5.39933390E-04 4.30463493E-04

Selection coefficients for In and St homokaryotypes

7.98686147E-02 -4.27901745E-03

Contributions to mean A2 freqs= 1.74576733E-02 7.62116615E-05

Contributions to mean diversities= 1.33943366E-04 1.20629527E-04

Zone 4: strong selection zone

Upper limit to scaled gamma for St metapopn= 2.50000000

Lower limit to gamma for St metapopn= 499.999969

Upper limit to gamma for St metapopn= 30000.0020

Probability of zone 4= 0.562775493

Contributions to mean load statistics over zone 4

Loads within In and St= 3.72709054E-03 7.75232445E-04

Load between In and St= 4.65358113E-04

Homozygous load for In and St= 5.58213890E-03 3.71194188E-03

Inbreeding loads= 1.85504649E-03 2.93670781E-03

Selection coefficients for In and St homokaryotypes

3.25644016E-03 3.09824944E-04

Contributions to mean A2 freqs= 9.90115732E-05 9.90115732E-05

Contributions to mean diversities= 5.19963692E-07 2.28841532E-06

Mean load statistics over all zones

Loads within In and St= 0.199147746 2.15165224E-03

Load between In and St= 1.11575574E-02

Homozygous load for In and St= 0.201612756 5.69320936E-03

Inbreeding loads= 2.46512797E-03 3.54155619E-03

Selection coefficients for In and St homokaryotypes

0.171377182 -9.04655457E-03

Mean frequencies of A2 in In and St= 0.162741706 4.40936349E-02

Ratio of these= 3.69082093

Mean diversities at selected sites in In and St= 4.34962305E-04 1.96636096E-03

Ratio of these= 0.221201658

Mean diversities at neutral sites in In and St= 1.58154487E-03 1.38683794E-02

pi-n/pi-s for In and St= 0.275023699 0.141787365

Ratio of these= 1.93969119

**Neutral Fst for whole population= 0.25**

**Scaled migration rate for whole population= 3**

Zone 1: quasi-neutral zone 1

Upper bound scaled selection coefficient for neutrality in St metapopulation= 0.250000000

Probability of zone 1= 4.39136960E-02

Integral of selection coefficient over zone 1= 1.40749034E-04

Mean load statistics for zone 1

Mean q1 and q2= 0.600000024

F1 and F2= 0.996714115 0.971184611

Diversities= 1.57722470E-03 1.38313863E-02

Contributions to loads within In and St= 8.43495218E-05 8.35733881E-05

Contributions to load between In and St = 5.40476285E-05

Contributions to homozygous loads for In and St= 8.44494207E-05 8.44494207E-05

Contributions to inbreeding loads for In and St= 9.98997365E-08 8.76035472E-07

Contributions to selection coefficients for In and St homokaryotypes

3.02791595E-05 2.95042992E-05

Contributions to mean A2 freqs= 2.63482183E-02 2.63482183E-02

Contributions to mean diversities= 6.92617687E-05 6.07387279E-04

Zone 2: quasi-neutral zone 2

Lower and upper bounds of St metapopn gamma for zone 2

0.250000000 50.0000000

Probability of zone 2= 0.171113729

Coefficients for bivariate distribution of q1 and q2 in metapopulation

G1= 3.33333325 G2= 0.370370388

G3= -5.66666651 G4= 0.259259224

a1= 3.50000001E-02 a2= 0.314999998

b11= -2.55000014E-02 b12= 8.09999928E-02 b22= 9.44999754E-02

Contributions to mean load statistics for zone 2

Loads within In and St= 0.114642620 1.09576865E-03

Load between In and St= 6.51200442E-03

Homozygous load for In and St= 0.114702903 1.24809507E-03

Inbreeding loads= 6.02810578E-05 1.52326684E-04

Selection coefficients against In and St homokaryotypes

0.102489650 -5.43093681E-03

Contributions to mean A2 freqs= 0.124162674 1.77419465E-02

Contributions to mean diversities= 2.15867651E-04 1.15683535E-03

Zone 3: moderate selection zone

Lower and upper bounds of St metapopn gamma for zone 3

50.0000000 499.999969

Probability of zone 3= 0.210339963

Contributions to mean load statistics over zone 3

Loads within In and St= 8.78385380E-02 2.31863829E-04

Load between In and St= 4.48371423E-03

Homozygous load for In and St= 8.83175656E-02 5.89756004E-04

Inbreeding loads= 4.78920381E-04 3.57891753E-04

Selection coefficients for In and St homokaryotypes

7.99753666E-02 -4.26089764E-03

Contributions to mean A2 freqs= 1.74729619E-02 6.80433368E-05

Contributions to mean diversities= 1.18982476E-04 1.01494079E-04

Zone 4: strong selection zone

Upper limit to scaled gamma for St metapopn= 2.50000000

Lower limit to gamma for St metapopn= 499.999969

Upper limit to gamma for St metapopn= 30000.0020

Probability of zone 4= 0.562775493

Contributions to mean load statistics over zone 4

Loads within In and St= 3.95127293E-03 7.75378605E-04

Load between In and St= 4.57609392E-04

Homozygous load for In and St= 5.62249916E-03 3.51723935E-03

Inbreeding loads= 1.67122902E-03 2.74186372E-03

Selection coefficients for In and St homokaryotypes

3.48758698E-03 3.17692757E-04

Contributions to mean A2 freqs= 9.83811842E-05 9.83811842E-05

Contributions to mean diversities= 5.32544050E-07 2.18642913E-06

Mean load statistics over all zones

Loads within In and St= 0.206516773 2.18658452E-03

Load between In and St= 1.15073752E-02

Homozygous load for In and St= 0.208727419 5.43953991E-03

Inbreeding loads= 2.21053045E-03 3.25295818E-03

Selection coefficients for In and St homokaryotypes

0.177173078 -9.36436653E-03

Mean frequencies of A2 in In and St= 0.168082237 4.42565903E-02

Ratio of these= 3.79790306

Mean diversities at selected sites in In and St= 4.04644437E-04 1.86790305E-03

Ratio of these= 0.216630325

Mean diversities at neutral sites in In and St= 1.57722470E-03 1.38313863E-02

pi-n/pi-s for In and St= 0.256554723 0.135048136

Ratio of these= 1.89972794

**h= 0.25**

**Neutral Fst for whole population= 0.05**

**Scaled migration rate for whole population= 19**

Zone 1: quasi-neutral zone 1

Upper bound scaled selection coefficient for neutrality in St metapopulation= 0.250000000

Probability of zone 1= 4.39136960E-02

Integral of selection coefficient over zone 1= 1.40749034E-04

Mean load statistics for zone 1

Mean q1 and q2= 0.600000024

F1 and F2= 0.996683598 0.970923305

Diversities= 1.59187312E-03 1.39568131E-02

Contributions to loads within In and St= 8.43934104E-05 8.39583226E-05

Contributions to load between In and St = 6.75595365E-05

Contributions to homozygous loads for In and St= 8.44494207E-05 8.44494207E-05

Contributions to inbreeding loads for In and St= 5.60153310E-08 4.91100991E-07

Contributions to selection coefficients for In and St homokaryotypes

1.68085098E-05 1.63912773E-05

Contributions to mean A2 freqs= 2.63482183E-02 2.63482183E-02

Contributions to mean diversities= 6.99050288E-05 6.12895237E-04

Zone 2: quasi-neutral zone 2

Lower and upper bounds of St metapopn gamma for zone 2

0.250000000 50.0000000

Probability of zone 2= 0.171113729

Coefficients for bivariate distribution of q1 and q2 in metapopulation

G1= 0.526315808 G2= 5.84795326E-02

G3= -5.26316166E-02 G4= 0.883040905

a1= 2.76315790E-02 a2= 0.248684227

b11= -1.31579058E-04 b12= 4.49999981E-02 b22= 0.178815767

Contributions to mean load statistics for zone 2

Loads within In and St= 3.44527252E-02 1.13034912E-03

Load between In and St= 9.27358959E-03

Homozygous load for In and St= 3.45031656E-02 1.23568997E-03

Inbreeding loads= 5.04576747E-05 1.05340056E-04

Selection coefficients against In and St homokaryotypes

2.48647928E-02 -8.17644596E-03

Contributions to mean A2 freqs= 7.34138936E-02 1.75750311E-02

Contributions to mean diversities= 2.65571347E-04 1.28044607E-03

Zone 3: moderate selection zone

Lower and upper bounds of St metapopn gamma for zone 3

50.0000000 499.999969

Probability of zone 3= 0.210339963

Contributions to mean load statistics over zone 3

Loads within In and St= 4.80081560E-03 2.15772845E-04

Load between In and St= 1.35087594E-03

Homozygous load for In and St= 4.99447249E-03 4.03744023E-04

Inbreeding loads= 1.93649961E-04 1.87970945E-04

Selection coefficients for In and St homokaryotypes

3.44401598E-03 -1.13570690E-03

Contributions to mean A2 freqs= 1.29892200E-03 5.20021276E-05

Contributions to mean diversities= 9.40994651E-05 9.80996701E-05

Zone 4: strong selection zone

Upper limit to scaled gamma for St metapopn= 2.50000000

Lower limit to gamma for St metapopn= 499.999969

Upper limit to gamma for St metapopn= 30000.0020

Probability of zone 4= 0.562775493

Contributions to mean load statistics over zone 4

Loads within In and St= 7.46119593E-04 4.79935232E-04

Load between In and St= 5.20273228E-04

Homozygous load for In and St= 1.15678005E-03 9.24250286E-04

Inbreeding loads= 4.10661363E-04 4.44314675E-04

Selection coefficients for In and St homokaryotypes

2.25841999E-04 -4.02927399E-05

Contributions to mean A2 freqs= 2.07354260E-05 2.07354260E-05

Contributions to mean diversities= 8.89003502E-08 4.48602918E-07

Mean load statistics over all zones

Loads within In and St= 4.00840528E-02 1.91001559E-03

Load between In and St= 1.12122977E-02

Homozygous load for In and St= 4.07388695E-02 2.64813378E-03

Inbreeding loads= 6.54825009E-04 7.38116796E-04

Selection coefficients for In and St homokaryotypes

2.84589529E-02 -9.34565067E-03

Mean frequencies of A2 in In and St= 0.101081766 4.39959876E-02

Ratio of these= 2.29752231

Mean diversities at selected sites in In and St= 4.29664738E-04 1.99188967E-03

Ratio of these= 0.215707093

Mean diversities at neutral sites in In and St= 1.59187312E-03 1.39568131E-02

pi-n/pi-s for In and St= 0.269911408 0.142718092

Ratio of these= 1.89122069

**Neutral Fst for whole population= 0.1**

**Scaled migration rate for whole population= 9**

Zone 1: quasi-neutral zone 1

Upper bound scaled selection coefficient for neutrality in St metapopulation= 0.250000000

Probability of zone 1= 4.39136960E-02

Integral of selection coefficient over zone 1= 1.40749034E-04

Mean load statistics for zone 1

Mean q1 and q2= 0.600000024

F1 and F2= 0.996689916 0.970978200

Diversities= 1.58884050E-03 1.39304632E-02

Contributions to loads within In and St= 8.43935195E-05 8.39592467E-05

Contributions to load between In and St = 6.75595365E-05

Contributions to homozygous loads for In and St= 8.44494207E-05 8.44494207E-05

Contributions to inbreeding loads for In and St= 5.59062698E-08 4.90178195E-07

Contributions to selection coefficients for In and St homokaryotypes

1.68085098E-05 1.63912773E-05

Contributions to mean A2 freqs= 2.63482183E-02 2.63482183E-02

Contributions to mean diversities= 6.97718569E-05 6.11738127E-04

Zone 2: quasi-neutral zone 2

Lower and upper bounds of St metapopn gamma for zone 2

0.250000000 50.0000000

Probability of zone 2= 0.171113729

Coefficients for bivariate distribution of q1 and q2 in metapopulation

G1= 1.11111104 G2= 0.123456798

G3= -1.22222209 G4= 0.753086388

a1= 3.05555556E-02 a2= 0.274999976

b11= -3.05555551E-03 b12= 4.49999981E-02 b22= 0.152499989

Contributions to mean load statistics for zone 2

Loads within In and St= 3.46798785E-02 1.14468695E-03

Load between In and St= 9.33690462E-03

Homozygous load for In and St= 3.47292535E-02 1.24440144E-03

Inbreeding loads= 4.93507796E-05 9.97137977E-05

Selection coefficients against In and St homokaryotypes

2.50245333E-02 -8.22591782E-03

Contributions to mean A2 freqs= 7.39017352E-02 1.76677536E-02

Contributions to mean diversities= 2.62158661E-04 1.24572392E-03

Zone 3: moderate selection zone

Lower and upper bounds of St metapopn gamma for zone 3

50.0000000 499.999969

Probability of zone 3= 0.210339963

Contributions to mean load statistics over zone 3

Loads within In and St= 4.94676409E-03 2.19550144E-04

Load between In and St= 1.37987814E-03

Homozygous load for In and St= 5.12602925E-03 3.88301210E-04

Inbreeding loads= 1.79263996E-04 1.68750936E-04

Selection coefficients for In and St homokaryotypes

3.56054306E-03 -1.16097927E-03

Contributions to mean A2 freqs= 1.32028246E-03 4.98902518E-05

Contributions to mean diversities= 8.83952089E-05 8.89816802E-05

Zone 4: strong selection zone

Upper limit to scaled gamma for St metapopn= 2.50000000

Lower limit to gamma for St metapopn= 499.999969

Upper limit to gamma for St metapopn= 30000.0020

Probability of zone 4= 0.562775493

Contributions to mean load statistics over zone 4

Loads within In and St= 9.27725632E-04 5.16280765E-04

Load between In and St= 5.70535369E-04

Homozygous load for In and St= 1.30788365E-03 9.74186172E-04

Inbreeding loads= 3.80158657E-04 4.57904040E-04

Selection coefficients for In and St homokaryotypes

3.57151031E-04 -5.42402267E-05

Contributions to mean A2 freqs= 2.29611687E-05 2.29611687E-05

Contributions to mean diversities= 1.09269287E-07 5.06374022E-07

Mean load statistics over all zones

Loads within In and St= 4.06387597E-02 1.96447689E-03

Load between In and St= 1.13548776E-02

Homozygous load for In and St= 4.12476137E-02 2.69133830E-03

Inbreeding loads= 6.08829316E-04 7.26858969E-04

Selection coefficients for In and St homokaryotypes

2.88592577E-02 -9.43458080E-03

Mean frequencies of A2 in In and St= 0.101593196 4.40888256E-02

Ratio of these= 2.30428457

Mean diversities at selected sites in In and St= 4.20434983E-04 1.94695021E-03

Ratio of these= 0.215945423

Mean diversities at neutral sites in In and St= 1.58884050E-03 1.39304632E-02

pi-n/pi-s for In and St= 0.264617503 0.139762059

Ratio of these= 1.89334285

**Neutral Fst for whole population= 0.15**

**Scaled migration rate for whole population= 5.667**

Zone 1: quasi-neutral zone 1

Upper bound scaled selection coefficient for neutrality in St metapopulation= 0.250000000

Probability of zone 1= 4.39136960E-02

Integral of selection coefficient over zone 1= 1.40749034E-04

Mean load statistics for zone 1

Mean q1 and q2= 0.600000024

F1 and F2= 0.996697068 0.971039176

Diversities= 1.58540718E-03 1.39011955E-02

Contributions to loads within In and St= 8.43936359E-05 8.39602799E-05

Contributions to load between In and St = 6.75595365E-05

Contributions to homozygous loads for In and St= 8.44494207E-05 8.44494207E-05

Contributions to inbreeding loads for In and St= 5.57888207E-08 4.89146316E-07

Contributions to selection coefficients for In and St homokaryotypes

1.68085098E-05 1.63912773E-05

Contributions to mean A2 freqs= 2.63482183E-02 2.63482183E-02

Contributions to mean diversities= 6.96210918E-05 6.10452844E-04

Zone 2: quasi-neutral zone 2

Lower and upper bounds of St metapopn gamma for zone 2

0.250000000 50.0000000

Probability of zone 2= 0.171113729

Coefficients for bivariate distribution of q1 and q2 in metapopulation

G1= 1.76470590 G2= 0.196078435

G3= -2.52941179 G4= 0.607843161

a1= 3.38235311E-02 a2= 0.304411739

b11= -6.32352987E-03 b12= 4.49999981E-02 b22= 0.123088233

Contributions to mean load statistics for zone 2

Loads within In and St= 3.49266492E-02 1.16001500E-03

Load between In and St= 9.40586347E-03

Homozygous load for In and St= 3.49747725E-02 1.25408615E-03

Inbreeding loads= 4.81312636E-05 9.40706814E-05

Selection coefficients against In and St homokaryotypes

2.51978636E-02 -8.27991962E-03

Contributions to mean A2 freqs= 7.44386613E-02 1.77708082E-02

Contributions to mean diversities= 2.58348387E-04 1.20950525E-03

Zone 3: moderate selection zone

Lower and upper bounds of St metapopn gamma for zone 3

50.0000000 499.999969

Probability of zone 3= 0.210339963

Contributions to mean load statistics over zone 3

Loads within In and St= 5.06782020E-03 2.26264543E-04

Load between In and St= 1.40500022E-03

Homozygous load for In and St= 5.23632811E-03 3.78615223E-04

Inbreeding loads= 1.68501050E-04 1.52350738E-04

Selection coefficients for In and St homokaryotypes

3.65614891E-03 -1.17945671E-03

Contributions to mean A2 freqs= 1.33895350E-03 4.83111326E-05

Contributions to mean diversities= 8.35953688E-05 8.14172308E-05

Zone 4: strong selection zone

Upper limit to scaled gamma for St metapopn= 2.50000000

Lower limit to gamma for St metapopn= 499.999969

Upper limit to gamma for St metapopn= 30000.0020

Probability of zone 4= 0.562775493

Contributions to mean load statistics over zone 4

Loads within In and St= 1.07089255E-03 5.33772924E-04

Load between In and St= 6.06429472E-04

Homozygous load for In and St= 1.43265771E-03 9.92975780E-04

Inbreeding loads= 3.61765764E-04 4.59203729E-04

Selection coefficients for In and St homokaryotypes

4.64379787E-04 -7.27176666E-05

Contributions to mean A2 freqs= 2.47447606E-05 2.47447606E-05

Contributions to mean diversities= 1.24709274E-07 5.32193837E-07

Mean load statistics over all zones

Loads within In and St= 4.11497578E-02 2.00401270E-03

Load between In and St= 1.14848530E-02

Homozygous load for In and St= 4.17282060E-02 2.71012657E-03

Inbreeding loads= 5.78453881E-04 7.06114282E-04

Selection coefficients for In and St homokaryotypes

2.92292237E-02 -9.52589512E-03

Mean frequencies of A2 in In and St= 0.102150574 4.41920795E-02

Ratio of these= 2.31151319

Mean diversities at selected sites in In and St= 4.11689573E-04 1.90190761E-03

Ratio of these=

Mean diversities at neutral sites in In and St= 1.58540718E-03 1.39011955E-02

pi-n/pi-s for In and St= 0.259674340 0.136816114

Ratio of these= 1.89798069

**Neutral Fst for whole population= 0.2**

**Scaled migration rate for whole population= 4**

Zone 1: quasi-neutral zone 1

Upper bound scaled selection coefficient for neutrality in St metapopulation= 0.250000000

Probability of zone 1= 4.39136960E-02

Integral of selection coefficient over zone 1= 1.40749034E-04

Mean load statistics for zone 1

Mean q1 and q2= 0.600000024

F1 and F2= 0.996705115 0.971107543

Diversities= 1.58154487E-03 1.38683794E-02

Contributions to loads within In and St= 8.43937669E-05 8.39614368E-05

Contributions to load between In and St = 6.75595365E-05

Contributions to homozygous loads for In and St= 8.44494207E-05 8.44494207E-05

Contributions to inbreeding loads for In and St= 5.56545920E-08 4.87988586E-07

Contributions to selection coefficients for In and St homokaryotypes

1.68085098E-05 1.63912773E-05

Contributions to mean A2 freqs= 2.63482183E-02 2.63482183E-02

Contributions to mean diversities= 6.94514820E-05 6.09011797E-04

Zone 2: quasi-neutral zone 2

Lower and upper bounds of St metapopn gamma for zone 2

0.250000000 50.0000000

Probability of zone 2= 0.171113729

Coefficients for bivariate distribution of q1 and q2 in metapopulation

G1= 2.50000000 G2= 0.277777791

G3= -4.00000000 G4= 0.444444418

a1= 3.75000015E-02 a2= 0.337499976

b11= -1.00000007E-02 b12= 4.49999981E-02 b22= 8.99999887E-02

Contributions to mean load statistics for zone 2

Loads within In and St= 3.51955555E-02 1.17655611E-03

Load between In and St= 9.48128384E-03

Homozygous load for In and St= 3.52423303E-02 1.26496691E-03

Inbreeding loads= 4.67876416E-05 8.84134715E-05

Selection coefficients against In and St homokaryotypes

2.53864527E-02 -8.33928585E-03

Contributions to mean A2 freqs= 7.50324726E-02 1.78861897E-02

Contributions to mean diversities= 2.54091778E-04 1.17164676E-03

Zone 3: moderate selection zone

Lower and upper bounds of St metapopn gamma for zone 3

50.0000000 499.999969

Probability of zone 3= 0.210339963

Contributions to mean load statistics over zone 3

Loads within In and St= 5.17047709E-03 2.34424035E-04

Load between In and St= 1.42691133E-03

Homozygous load for In and St= 5.33028506E-03 3.72380222E-04

Inbreeding loads= 1.59816351E-04 1.37956129E-04

Selection coefficients for In and St homokaryotypes

3.73655558E-03 -1.19316578E-03

Contributions to mean A2 freqs= 1.35542953E-03 4.70867526E-05

Contributions to mean diversities= 7.94001608E-05 7.48923558E-05

Zone 4: strong selection zone

Upper limit to scaled gamma for St metapopn= 2.50000000

Lower limit to gamma for St metapopn= 499.999969

Upper limit to gamma for St metapopn= 30000.0020

Probability of zone 4= 0.562775493

Contributions to mean load statistics over zone 4

Loads within In and St= 1.19706662E-03 5.44993556E-04

Load between In and St= 6.37391640E-04

Homozygous load for In and St= 1.54663809E-03 1.00283674E-03

Inbreeding loads= 3.49570066E-04 4.57843649E-04

Selection coefficients for In and St homokaryotypes

5.59508801E-04 -9.23871994E-05

Contributions to mean A2 freqs= 2.64784157E-05 2.64784157E-05

Contributions to mean diversities= 1.38424184E-07 5.47460616E-07

Mean load statistics over all zones

Loads within In and St= 4.16474901E-02 2.03993497E-03

Load between In and St= 1.16131455E-02

Homozygous load for In and St= 4.22037020E-02 2.72463332E-03

Inbreeding loads= 5.56229730E-04 6.84701256E-04

Selection coefficients for In and St homokaryotypes

2.95878053E-02 -9.61923599E-03

Mean frequencies of A2 in In and St= 0.102762602 4.43079770E-02

Ratio of these= 2.31927991

Mean diversities at selected sites in In and St= 4.03081853E-04 1.85609830E-03

Ratio of these= 0.217166215

Mean diversities at neutral sites in In and St= 1.58154487E-03 1.38683794E-02

pi-n/pi-s for In and St= 0.254865885 0.133836716

Ratio of these= 1.90430462

**Neutral Fst for whole population= 0.25**

**Scaled migration rate for whole population= 3**

Zone 1: quasi-neutral zone 1

Upper bound scaled selection coefficient for neutrality in St metapopulation= 0.250000000

Probability of zone 1= 4.39136960E-02

Integral of selection coefficient over zone 1= 1.40749034E-04

Mean load statistics for zone 1

Mean q1 and q2= 0.600000024

F1 and F2= 0.996714115 0.971184611

Diversities= 1.57722470E-03 1.38313863E-02

Contributions to loads within In and St= 8.43939197E-05 8.39627392E-05

Contributions to load between In and St = 6.75595365E-05

Contributions to homozygous loads for In and St= 8.44494207E-05 8.44494207E-05

Contributions to inbreeding loads for In and St= 5.55035840E-08 4.86688236E-07

Contributions to selection coefficients for In and St homokaryotypes

1.68085098E-05 1.63912773E-05

Contributions to mean A2 freqs= 2.63482183E-02 2.63482183E-02

Contributions to mean diversities= 6.92617687E-05 6.07387279E-04

Zone 2: quasi-neutral zone 2

Lower and upper bounds of St metapopn gamma for zone 2

0.250000000 50.0000000

Probability of zone 2= 0.171113729

Coefficients for bivariate distribution of q1 and q2 in metapopulation

G1= 3.33333325 G2= 0.370370388

G3= -5.66666651 G4= 0.259259224

a1= 4.16666679E-02 a2= 0.374999970

b11= -1.41666671E-02 b12= 4.49999981E-02 b22= 5.24999909E-02

Contributions to mean load statistics for zone 2

Loads within In and St= 3.54896560E-02 1.19459385E-03

Load between In and St= 9.56411101E-03

Homozygous load for In and St= 3.55349295E-02 1.27733813E-03

Inbreeding loads= 4.53068933E-05 8.27434997E-05

Selection coefficients against In and St homokaryotypes

2.55923867E-02 -8.40461254E-03

Contributions to mean A2 freqs= 7.56918415E-02 1.80165172E-02

Contributions to mean diversities= 2.49328645E-04 1.13198056E-03

Zone 3: moderate selection zone

Lower and upper bounds of St metapopn gamma for zone 3

50.0000000 499.999969

Probability of zone 3= 0.210339963

Contributions to mean load statistics over zone 3

Loads within In and St= 5.25889965E-03 2.43657152E-04

Load between In and St= 1.44632894E-03

Homozygous load for In and St= 5.41142933E-03 3.69003421E-04

Inbreeding loads= 1.52528766E-04 1.25346269E-04

Selection coefficients for In and St homokaryotypes

3.80533934E-03 -1.20341778E-03

Contributions to mean A2 freqs= 1.37007365E-03 4.61689524E-05

Contributions to mean diversities= 7.56597801E-05 6.91825553E-05

Zone 4: strong selection zone

Upper limit to scaled gamma for St metapopn= 2.50000000

Lower limit to gamma for St metapopn= 499.999969

Upper limit to gamma for St metapopn= 30000.0020

Probability of zone 4= 0.562775493

Contributions to mean load statistics over zone 4

Loads within In and St= 1.32876204E-03 5.53344260E-04

Load between In and St= 6.69732981E-04

Homozygous load for In and St= 1.66966161E-03 1.00916729E-03

Inbreeding loads= 3.40899598E-04 4.55822767E-04

Selection coefficients for In and St homokaryotypes

6.58810139E-04 -1.16348267E-04

Contributions to mean A2 freqs= 2.85666629E-05 2.85666629E-05

Contributions to mean diversities= 1.53261922E-07 5.58103011E-07

Mean load statistics over all zones

Loads within In and St= 4.21617106E-02 2.07555806E-03

Load between In and St= 1.17477328E-02

Homozygous load for In and St= 4.27004695E-02 2.73995823E-03

Inbreeding loads= 5.38790773E-04 6.64399238E-04

Selection coefficients for In and St homokaryotypes

2.99561024E-02 -9.71913338E-03

Mean frequencies of A2 in In and St= 0.103438698 4.44394685E-02

Ratio of these= 2.32763124

Mean diversities at selected sites in In and St= 3.94403440E-04 1.80910854E-03

Ratio of these= 0.218009830

Mean diversities at neutral sites in In and St= 1.57722470E-03 1.38313863E-02

pi-n/pi-s for In and St= 0.250061661 0.130797341

Ratio of these= 1.91182530

**h= 0.45**

**Neutral Fst for whole population= 5**

**Scaled migration rate for whole population= 19**

Zone 1: quasi-neutral zone 1

Upper bound scaled selection coefficient for neutrality in St metapopulation= 0.250000000

Probability of zone 1= 4.39136960E-02

Integral of selection coefficient over zone 1= 1.40749034E-04

Mean load statistics for zone 1

Mean q1 and q2= 0.600000024

F1 and F2= 0.996683598 0.970923305

Diversities= 1.59187312E-03 1.39568131E-02

Contributions to loads within In and St= 8.44382157E-05 8.43512025E-05

Contributions to load between In and St = 8.10714409E-05

Contributions to homozygous loads for In and St= 8.44494207E-05 8.44494207E-05

Contributions to inbreeding loads for In and St= 1.12080993E-08 9.82218822E-08

Contributions to selection coefficients for In and St homokaryotypes

3.33786011E-06 3.27825546E-06

Contributions to mean A2 freqs= 2.63482183E-02 2.63482183E-02

Contributions to mean diversities= 6.99050288E-05 6.12895237E-04

Zone 2: quasi-neutral zone 2

Lower and upper bounds of St metapopn gamma for zone 2

0.250000000 50.0000000

Probability of zone 2= 0.171113729

Coefficients for bivariate distribution of q1 and q2 in metapopulation

G1= 0.526315808 G2= 5.84795326E-02

G3= -5.26316166E-02 G4= 0.883040905

a1= 4.55263145E-02 a2= 0.409736782

b11= -2.63158163E-05 b12= 9.00000241E-03 b22= 3.57631631E-02

Contributions to mean load statistics for zone 2

Loads within In and St= 1.98006574E-02 1.20845530E-03

Load between In and St= 9.53217503E-03

Homozygous load for In and St= 1.98090468E-02 1.22374552E-03

Inbreeding loads= 8.38525193E-06 1.52900448E-05

Selection coefficients against In and St homokaryotypes

1.02159381E-02 -8.35847855E-03

Contributions to mean A2 freqs= 6.03856705E-02 1.80104654E-02

Contributions to mean diversities= 2.47368618E-04 1.09999371E-03

Zone 3: moderate selection zone

Lower and upper bounds of St metapopn gamma for zone 3

50.0000000 499.999969

Probability of zone 3= 0.210339963

Contributions to mean load statistics over zone 3

Loads within In and St= 7.01803889E-04 2.10162776E-04

Load between In and St= 4.30518267E-04

Homozygous load for In and St= 7.24944053E-04 2.31719881E-04

Inbreeding loads= 2.31401664E-05 2.15571581E-05

Selection coefficients for In and St homokaryotypes

2.71260738E-04 -2.20417976E-04

Contributions to mean A2 freqs= 1.57527524E-04 3.05474568E-05

Contributions to mean diversities= 5.76639613E-05 5.77514911E-05

Zone 4: strong selection zone

Upper limit to scaled gamma for St metapopn= 2.50000000

Lower limit to gamma for St metapopn= 499.999969

Upper limit to gamma for St metapopn= 30000.0020

Probability of zone 4= 0.562775493

Contributions to mean load statistics over zone 4

Loads within In and St= 6.07724476E-04 4.72122134E-04

Load between In and St= 5.30732330E-04

Homozygous load for In and St= 6.56688586E-04 5.22713701E-04

Inbreeding loads= 4.89644044E-05 5.05913231E-05

Selection coefficients for In and St homokaryotypes

7.70092010E-05 -5.86509705E-05

Contributions to mean A2 freqs= 1.17406953E-05 1.17406953E-05

Contributions to mean diversities= 5.03997626E-08 2.48076105E-07

Mean load statistics over all zones

Loads within In and St= 2.11946238E-02 1.97509141E-03

Load between In and St= 1.05744973E-02

Homozygous load for In and St= 2.12751273E-02 2.06262851E-03

Inbreeding loads= 8.05010350E-05 8.75367477E-05

Selection coefficients for In and St homokaryotypes

1.05639100E-02 -8.63647461E-03

Mean frequencies of A2 in In and St= 8.69031623E-02 4.44009751E-02

Ratio of these= 1.95723546

Mean diversities at selected sites in In and St= 3.74988042E-04 1.77088857E-03

Ratio of these= 0.211751342

Mean diversities at neutral sites in In and St= 1.59187312E-03 1.39568131E-02

pi-n/pi-s for In and St= 0.235564023 0.126883447

Ratio of these= 1.85653865

**Neutral Fst for whole population= 0.1**

**Scaled migration rate for whole population= 9**

Zone 1: quasi-neutral zone 1

Upper bound scaled selection coefficient for neutrality in St metapopulation= 0.250000000

Probability of zone 1= 4.39136960E-02

Integral of selection coefficient over zone 1= 1.40749034E-04

Mean load statistics for zone 1

Mean q1 and q2= 0.600000024

F1 and F2= 0.996689916 0.970978200

Diversities= 1.58884050E-03 1.39304632E-02

Contributions to loads within In and St= 8.44382375E-05 8.43513844E-05

Contributions to load between In and St = 8.10714409E-05

Contributions to homozygous loads for In and St= 8.44494207E-05 8.44494207E-05

Contributions to inbreeding loads for In and St= 1.11829319E-08 9.80373187E-08

Contributions to selection coefficients for In and St homokaryotypes

3.33786011E-06 3.27825546E-06

Contributions to mean A2 freqs= 2.63482183E-02 2.63482183E-02

Contributions to mean diversities= 6.97718569E-05 6.11738127E-04

Zone 2: quasi-neutral zone 2

Lower and upper bounds of St metapopn gamma for zone 2

0.250000000 50.0000000

Probability of zone 2= 0.171113729

Coefficients for bivariate distribution of q1 and q2 in metapopulation

G1= 1.11111104 G2= 0.123456798

G3= -1.22222209 G4= 0.753086388

a1= 4.61111069E-02 a2= 0.414999962

b11= -6.11111231E-04 b12= 9.00000241E-03 b22= 3.05000041E-02

Contributions to mean load statistics for zone 2

Loads within In and St= 1.98864844E-02 1.22063793E-03

Load between In and St= 9.57674906E-03

Homozygous load for In and St= 1.98948551E-02 1.23584608E-03

Inbreeding loads= 8.38701271E-06 1.52090224E-05

Selection coefficients against In and St homokaryotypes

1.02567673E-02 -8.39114189E-03

Contributions to mean A2 freqs= 6.04938678E-02 1.80606004E-02

Contributions to mean diversities= 2.47231306E-04 1.09507365E-03

Zone 3: moderate selection zone

Lower and upper bounds of St metapopn gamma for zone 3

50.0000000 499.999969

Probability of zone 3= 0.210339963

Contributions to mean load statistics over zone 3

Loads within In and St= 7.98757130E-04 2.18567206E-04

Load between In and St= 4.77351161E-04

Homozygous load for In and St= 8.21320980E-04 2.39410976E-04

Inbreeding loads= 2.25633539E-05 2.08437286E-05

Selection coefficients for In and St homokaryotypes

3.21328640E-04 -2.58803368E-04

Contributions to mean A2 freqs= 1.72324959E-04 3.14609315E-05

Contributions to mean diversities= 5.68874348E-05 5.62765163E-05

Zone 4: strong selection zone

Upper limit to scaled gamma for St metapopn= 2.50000000

Lower limit to gamma for St metapopn= 499.999969

Upper limit to gamma for St metapopn= 30000.0020

Probability of zone 4= 0.562775493

Contributions to mean load statistics over zone 4

Loads within In and St= 7.00356904E-04 5.03870542E-04

Load between In and St= 5.86990151E-04

Homozygous load for In and St= 7.47593818E-04 5.56826533E-04

Inbreeding loads= 4.72364300E-05 5.29554673E-05

Selection coefficients for In and St homokaryotypes

1.13368034E-04 -8.30888748E-05

Contributions to mean A2 freqs= 1.34552110E-05 1.34552110E-05

Contributions to mean diversities= 6.41983391E-08 2.85249172E-07

Mean load statistics over all zones

Loads within In and St= 2.14700345E-02 2.02742708E-03

Load between In and St= 1.07221622E-02

Homozygous load for In and St= 2.15482172E-02 2.11653300E-03

Inbreeding loads= 7.81979761E-05 8.91062518E-05

Selection coefficients for In and St homokaryotypes

1.06903315E-02 -8.73267651E-03

Mean frequencies of A2 in In and St= 8.70278701E-02 4.44537364E-02

Ratio of these= 1.95771778

Mean diversities at selected sites in In and St= 3.73954797E-04 1.76337361E-03

Ratio of these= 0.2120678

Mean diversities at neutral sites in In and St= 1.58884050E-03 1.39304632E-02

pi-n/pi-s for In and St= 0.235363334 0.126583993

Ratio of these= 1.85934520

**Neutral Fst for whole population= 0.15**

**Scaled migration rate for whole population= 5.667**

Zone 1: quasi-neutral zone 1

Upper bound scaled selection coefficient for neutrality in St metapopulation= 0.250000000

Probability of zone 1= 4.39136960E-02

Integral of selection coefficient over zone 1= 1.40749034E-04

Mean load statistics for zone 1

Mean q1 and q2= 0.600000024

F1 and F2= 0.996697068 0.971039176

Diversities= 1.58540718E-03 1.39011955E-02

Contributions to loads within In and St= 8.44382666E-05 8.43515954E-05

Contributions to load between In and St = 8.10714409E-05

Contributions to homozygous loads for In and St= 8.44494207E-05 8.44494207E-05

Contributions to inbreeding loads for In and St= 1.11577636E-08 9.78275807E-08

Contributions to selection coefficients for In and St homokaryotypes

3.33786011E-06 3.27825546E-06

Contributions to mean A2 freqs= 2.63482183E-02 2.63482183E-02

Contributions to mean diversities= 6.96210918E-05 6.10452844E-04

Zone 2: quasi-neutral zone 2

Lower and upper bounds of St metapopn gamma for zone 2

0.250000000 50.0000000

Probability of zone 2= 0.171113729

Coefficients for bivariate distribution of q1 and q2 in metapopulation

G1= 1.76470590 G2= 0.196078435

G3= -2.52941179 G4= 0.607843161

a1= 4.67647016E-02 a2= 0.420882314

b11= -1.26470637E-03 b12= 9.00000241E-03 b22= 2.46176515E-02

Contributions to mean load statistics for zone 2

Loads within In and St= 1.99816246E-02 1.23410078E-03

Load between In and St= 9.62614268E-03

Homozygous load for In and St= 1.99900046E-02 1.24921824E-03

Inbreeding loads= 8.38734104E-06 1.51178674E-05

Selection coefficients against In and St homokaryotypes

1.03020668E-02 -8.42738152E-03

Contributions to mean A2 freqs= 6.06140159E-02 1.81162804E-02

Contributions to mean diversities= 2.47050892E-04 1.08959479E-03

Zone 3: moderate selection zone

Lower and upper bounds of St metapopn gamma for zone 3

50.0000000 499.999969

Probability of zone 3= 0.210339963

Contributions to mean load statistics over zone 3

Loads within In and St= 8.88351526E-04 2.28472927E-04

Load between In and St= 5.21621143E-04

Homozygous load for In and St= 9.10537085E-04 2.48565892E-04

Inbreeding loads= 2.21862228E-05 2.00929189E-05

Selection coefficients for In and St homokaryotypes

3.66687775E-04 -2.93135643E-04

Contributions to mean A2 freqs= 1.86576901E-04 3.25341935E-05

Contributions to mean diversities= 5.62730456E-05 5.50344266E-05

Zone 4: strong selection zone

Upper limit to scaled gamma for St metapopn= 2.50000000

Lower limit to gamma for St metapopn= 499.999969

Upper limit to gamma for St metapopn= 30000.0020

Probability of zone 4= 0.562775493

Contributions to mean load statistics over zone 4

Loads within In and St= 7.79808790E-04 5.20293077E-04

Load between In and St= 6.30100083E-04

Homozygous load for In and St= 8.26013915E-04 5.74204139E-04

Inbreeding loads= 4.62051794E-05 5.39103021E-05

Selection coefficients for In and St homokaryotypes

1.49726868E-04 -1.09791756E-04

Contributions to mean A2 freqs= 1.49718044E-05 1.49718044E-05

Contributions to mean diversities= 7.58203385E-08 3.05722665E-07

Mean load statistics over all zones

Loads within In and St= 2.17342228E-02 2.06721853E-03

Load between In and St= 1.08589353E-02

Homozygous load for In and St= 2.18110047E-02 2.15643784E-03

Inbreeding loads= 7.67899037E-05 8.92189128E-05

Selection coefficients for In and St homokaryotypes

1.08163357E-02 -8.83042812E-03

Mean frequencies of A2 in In and St= 8.71637836E-02 4.45120037E-02

Ratio of these= 1.95820844

Mean diversities at selected sites in In and St= 3.73020826E-04 1.75538776E-03

Ratio of these= 0.212500528

Mean diversities at neutral sites in In and St= 1.58540718E-03 1.39011955E-02

pi-n/pi-s for In and St= 0.235283926 0.126276031

Ratio of these= 1.86325085

**Neutral Fst for whole population= 0.2**

**Scaled migration rate for whole population= 4**

Zone 1: quasi-neutral zone 1

Upper bound scaled selection coefficient for neutrality in St metapopulation= 0.250000000

Probability of zone 1= 4.39136960E-02

Integral of selection coefficient over zone 1= 1.40749034E-04

Mean load statistics for zone 1

Mean q1 and q2= 0.600000024

F1 and F2= 0.996705115 0.971107543

Diversities= 1.58154487E-03 1.38683794E-02

Contributions to loads within In and St= 8.44382885E-05 8.43518283E-05

Contributions to load between In and St = 8.10714409E-05

Contributions to homozygous loads for In and St= 8.44494207E-05 8.44494207E-05

Contributions to inbreeding loads for In and St= 1.11325962E-08 9.75926824E-08

Contributions to selection coefficients for In and St homokaryotypes

3.33786011E-06 3.27825546E-06

Contributions to mean A2 freqs= 2.63482183E-02 2.63482183E-02

Contributions to mean diversities= 6.94514820E-05 6.09011797E-04

Zone 2: quasi-neutral zone 2

Lower and upper bounds of St metapopn gamma for zone 2

0.250000000 50.0000000

Probability of zone 2= 0.171113729

Coefficients for bivariate distribution of q1 and q2 in metapopulation

G1= 2.50000000 G2= 0.277777791

G3= -4.00000000 G4= 0.444444418

a1= 4.74999957E-02 a2= 0.427499950

b11= -2.00000056E-03 b12= 9.00000241E-03 b22= 1.80000030E-02

Contributions to mean load statistics for zone 2

Loads within In and St= 2.00876147E-02 1.24905456E-03

Load between In and St= 9.68115684E-03

Homozygous load for In and St= 2.00959984E-02 1.26406678E-03

Inbreeding loads= 8.38585311E-06 1.50146125E-05

Selection coefficients against In and St homokaryotypes

1.03524923E-02 -8.46779346E-03

Contributions to mean A2 freqs= 6.07477725E-02 1.81783903E-02

Contributions to mean diversities= 2.46818381E-04 1.08345656E-03

Zone 3: moderate selection zone

Lower and upper bounds of St metapopn gamma for zone 3

50.0000000 499.999969

Probability of zone 3= 0.210339963

Contributions to mean load statistics over zone 3

Loads within In and St= 9.71889589E-04 2.38993685E-04

Load between In and St= 5.63436945E-04

Homozygous load for In and St= 9.93786030E-04 2.58235727E-04

Inbreeding loads= 2.18970890E-05 1.92424104E-05

Selection coefficients for In and St homokaryotypes

4.08351421E-04 -3.24487686E-04

Contributions to mean A2 freqs= 2.00382303E-04 3.36560188E-05

Contributions to mean diversities= 5.57337080E-05 5.37646010E-05

Zone 4: strong selection zone

Upper limit to scaled gamma for St metapopn= 2.50000000

Lower limit to gamma for St metapopn= 499.999969

Upper limit to gamma for St metapopn= 30000.0020

Probability of zone 4= 0.562775493

Contributions to mean load statistics over zone 4

Loads within In and St= 8.54225655E-04 5.31637983E-04

Load between In and St= 6.68631459E-04

Homozygous load for In and St= 8.99753592E-04 5.86089969E-04

Inbreeding loads= 4.55275986E-05 5.44518734E-05

Selection coefficients for In and St homokaryotypes

1.85549259E-04 -1.36971474E-04

Contributions to mean A2 freqs= 1.64802950E-05 1.64802950E-05

Contributions to mean diversities= 8.67882264E-08 3.20478335E-07

Mean load statistics over all zones

Loads within In and St= 2.19981670E-02 2.10403814E-03

Load between In and St= 1.09942975E-02

Homozygous load for In and St= 2.20739879E-02 2.19284184E-03

Inbreeding loads= 7.58216775E-05 8.88064897E-05

Selection coefficients for In and St homokaryotypes

1.09435320E-02 -8.92984867E-03

Mean frequencies of A2 in In and St= 8.73128548E-02 4.45767418E-02

Ratio of these= 1.95870876

Mean diversities at selected sites in In and St= 3.72090377E-04 1.74655335E-03

Ratio of these= 0.213042662

Mean diversities at neutral sites in In and St= 1.58154487E-03 1.38683794E-02

pi-n/pi-s for In and St= 0.235270202 0.125937805

Ratio of these= 1.86814594

**Neutral Fst for whole population= 0.25**

**Scaled migration rate for whole population= 3**

Zone 1: quasi-neutral zone 1

Upper bound scaled selection coefficient for neutrality in St metapopulation= 0.250000000

Probability of zone 1= 4.39136960E-02

Integral of selection coefficient over zone 1= 1.40749034E-04

Mean load statistics for zone 1

Mean q1 and q2= 0.600000024

F1 and F2= 0.996714115 0.971184611

Diversities= 1.57722470E-03 1.38313863E-02

Contributions to loads within In and St= 8.44383248E-05 8.43520829E-05

Contributions to load between In and St = 8.10714409E-05

Contributions to homozygous loads for In and St= 8.44494207E-05 8.44494207E-05

Contributions to inbreeding loads for In and St= 1.10990390E-08 9.73410010E-08

Contributions to selection coefficients for In and St homokaryotypes

3.33786011E-06 3.27825546E-06

Contributions to mean A2 freqs= 2.63482183E-02 2.63482183E-02

Contributions to mean diversities= 6.92617687E-05 6.07387279E-04

Zone 2: quasi-neutral zone 2

Lower and upper bounds of St metapopn gamma for zone 2

0.250000000 50.0000000

Probability of zone 2= 0.171113729

Coefficients for bivariate distribution of q1 and q2 in metapopulation

G1= 3.33333325 G2= 0.370370388

G3= -5.66666651 G4= 0.259259224

a1= 4.83333282E-02 a2= 0.434999973

b11= -2.83333403E-03 b12= 9.00000241E-03 b22= 1.05000008E-02

Contributions to mean load statistics for zone 2

Loads within In and St= 2.02063583E-02 1.26575213E-03

Load between In and St= 9.74275358E-03

Homozygous load for In and St= 2.02147365E-02 1.28065015E-03

Inbreeding loads= 8.38172036E-06 1.48967301E-05

Selection coefficients against In and St homokaryotypes

1.04090571E-02 -8.51297379E-03

Contributions to mean A2 freqs= 6.08979426E-02 1.82482190E-02

Contributions to mean diversities= 2.46517913E-04 1.07653160E-03

Zone 3: moderate selection zone

Lower and upper bounds of St metapopn gamma for zone 3

50.0000000 499.999969

Probability of zone 3= 0.210339963

Contributions to mean load statistics over zone 3

Loads within In and St= 1.05050032E-03 2.50186538E-04

Load between In and St= 6.03336201E-04

Homozygous load for In and St= 1.07215915E-03 2.68522010E-04

Inbreeding loads= 2.16583576E-05 1.83353786E-05

Selection coefficients for In and St homokaryotypes

4.47034836E-04 -3.53217125E-04

Contributions to mean A2 freqs= 2.13824387E-04 3.48325011E-05

Contributions to mean diversities= 5.52393758E-05 5.24381903E-05

Zone 4: strong selection zone

Upper limit to scaled gamma for St metapopn= 2.50000000

Lower limit to gamma for St metapopn= 499.999969

Upper limit to gamma for St metapopn= 30000.0020

Probability of zone 4= 0.562775493

Contributions to mean load statistics over zone 4

Loads within In and St= 9.33266652E-04 5.40599576E-04

Load between In and St= 7.08180654E-04

Homozygous load for In and St= 9.78315948E-04 5.95415710E-04

Inbreeding loads= 4.50482512E-05 5.48160860E-05

Selection coefficients for In and St homokaryotypes

2.25067139E-04 -1.67608261E-04

Contributions to mean A2 freqs= 1.82054318E-05 1.82054318E-05

Contributions to mean diversities= 9.86542901E-08 3.32547756E-07

Mean load statistics over all zones

Loads within In and St= 2.22745631E-02 2.14089034E-03

Load between In and St= 1.11353416E-02

Homozygous load for In and St= 2.23496594E-02 2.22903723E-03

Inbreeding loads= 7.50994295E-05 8.81455344E-05

Selection coefficients for In and St homokaryotypes

1.10774040E-02 -9.03499126E-03

Mean frequencies of A2 in In and St= 8.74781907E-02 4.46494743E-02

Ratio of these= 1.95922112

Mean diversities at selected sites in In and St= 3.71117727E-04 1.73668971E-03

Ratio of these= 0.213692591

Mean diversities at neutral sites in In and St= 1.57722470E-03 1.38313863E-02

pi-n/pi-s for In and St= 0.235297948 0.125561506

Ratio of these= 1.87396562

**Inversion frequency= 0.5**

**h= 0.05**

**Neutral Fst for whole population= 0.05**

**Scaled migration rate for whole population= 19**

Zone 1: quasi-neutral zone 1

Upper bound scaled selection coefficient for neutrality in St metapopulation= 0.250000000

Probability of zone 1= 5.23817725E-02

Integral of selection coefficient over zone 1= 3.02202563E-04

Mean load statistics for zone 1

Mean q1 and q2= 0.600000024

F1 and F2= 0.983634770 0.983634770

Diversities= 7.85531010E-03 7.85531010E-03

Contributions to loads within In and St= 1.80253308E-04 1.80253308E-04

Contributions to load between In and St = 1.16045783E-04

Contributions to homozygous loads for In and St= 1.81321549E-04 1.81321549E-04

Contributions to inbreeding loads for In and St= 1.06824177E-06 1.06824177E-06

Contributions to selection coefficients for In and St homokaryotypes

6.41942024E-05 6.41942024E-05

Contributions to mean A2 freqs= 3.14290635E-02 3.14290635E-02

Contributions to mean diversities= 4.11475077E-04 4.11475077E-04

Zone 2: quasi-neutral zone 2

Lower and upper bounds of St metapopn gamma for zone 2

0.250000000 50.0000000

Probability of zone 2= 0.203914478

Coefficients for bivariate distribution of q1 and q2 in metapopulation

G1= 0.105263159 G2= 0.105263159

G3= 0.789473653 G4= 0.789473653

a1= 4.86842096E-02 a2= 4.86842096E-02

b11= 8.88157859E-02 b12= 0.224999994 b22= 8.88157859E-02

Contributions to mean load statistics for zone 2

Loads within In and St= 4.40796744E-03 4.40795021E-03

Load between In and St= 1.38942024E-03

Homozygous load for In and St= 4.85523511E-03 4.85521648E-03

Inbreeding loads= 4.47267084E-04 4.47267084E-04

Selection coefficients against In and St homokaryotypes

3.01396847E-03 3.01396847E-03

Contributions to mean A2 freqs= 2.65763868E-02 2.65763309E-02

Contributions to mean diversities= 1.18304323E-03 1.18304323E-03

Zone 3: moderate selection zone

Lower and upper bounds of St metapopn gamma for zone 3

50.0000000 100.000000

Probability of zone 3= 5.86978197E-02

Contributions to mean load statistics over zone 3

Loads within In and St= 7.16577706E-05 7.16609429E-05

Load between In and St= 3.69135341E-05

Homozygous load for In and St= 3.66200227E-04 3.66244320E-04

Inbreeding loads= 2.94542551E-04 2.94583442E-04

Selection coefficients for In and St homokaryotypes

3.47495079E-05 3.47495079E-05

Contributions to mean A2 freqs= 5.20766007E-05 5.20854119E-05

Contributions to mean diversities= 9.30309834E-05 9.30505121E-05

Zone 4: strong selection zone

Upper limit to scaled gamma for St metapopn= 2.50000000

Lower limit to gamma for St metapopn= 100.000000

Upper limit to gamma for St metapopn= 16666.6680

Probability of zone 4= 0.673148870

Contributions to mean load statistics over zone 4

Loads within In and St= 1.06607878E-03 1.06607878E-03

Load between In and St= 5.68730815E-04

Homozygous load for In and St= 5.66785783E-03 5.66785736E-03

Inbreeding loads= 4.60177986E-03 4.60177986E-03

Selection coefficients for In and St homokaryotypes

4.97221947E-04 4.97221947E-04

Contributions to mean A2 freqs= 2.30848163E-04 2.30848163E-04

Contributions to mean diversities= 4.00626277E-06 4.00626277E-06

Mean load statistics over all zones

Loads within In and St= 5.72595745E-03 5.72594348E-03

Load between In and St= 2.11111037E-03

Homozygous load for In and St= 1.10706147E-02 1.10706398E-02

Inbreeding loads= 5.34465769E-03 5.34469867E-03

Selection coefficients for In and St homokaryotypes

3.60834599E-03 3.60828638E-03

Mean frequencies of A2 in In and St= 5.82883768E-02 5.82883283E-02

Ratio of these= 1.00000083

Mean diversities at selected sites in In and St= 1.69155560E-03 1.69157516E-03

Ratio of these= 0.999988437

Mean diversities at neutral sites in In and St= 7.85531010E-03 7.85531010E-03

pi-n/pi-s for In and St= 0.215339124 0.215341613

Ratio of these= 0.999988437

**Neutral Fst for whole population= 0.1**

**Scaled migration rate for whole population= 9**

Zone 1: quasi-neutral zone 1

Upper bound scaled selection coefficient for neutrality in St metapopulation= 0.250000000

Probability of zone 1= 5.23817725E-02

Integral of selection coefficient over zone 1= 3.02202563E-04

Mean load statistics for zone 1

Mean q1 and q2= 0.600000024

F1 and F2= 0.983666062 0.983666062

Diversities= 7.84028973E-03 7.84028973E-03

Contributions to loads within In and St= 1.80255345E-04 1.80255345E-04

Contributions to load between In and St = 1.16045783E-04

Contributions to homozygous loads for In and St= 1.81321549E-04 1.81321549E-04

Contributions to inbreeding loads for In and St= 1.06620632E-06 1.06620632E-06

Contributions to selection coefficients for In and St homokaryotypes

6.41942024E-05 6.41942024E-05

Contributions to mean A2 freqs= 3.14290635E-02 3.14290635E-02

Contributions to mean diversities= 4.10688284E-04 4.10688284E-04

Zone 2: quasi-neutral zone 2

Lower and upper bounds of St metapopn gamma for zone 2

0.250000000 50.0000000

Probability of zone 2= 0.203914478

Coefficients for bivariate distribution of q1 and q2 in metapopulation

G1= 0.222222224 G2= 0.222222224

G3= 0.555555582 G4= 0.555555582

a1= 7.50000030E-02 a2= 7.50000030E-02

b11= 6.25000000E-02 b12= 0.224999994 b22= 6.25000000E-02

Contributions to mean load statistics for zone 2

Loads within In and St= 4.91859298E-03 4.91854874E-03

Load between In and St= 1.54968584E-03

Homozygous load for In and St= 5.29752811E-03 5.29748341E-03

Inbreeding loads= 3.78936762E-04 3.78936442E-04

Selection coefficients against In and St homokaryotypes

3.36325169E-03 3.36319208E-03

Contributions to mean A2 freqs= 2.79014166E-02 2.79012900E-02

Contributions to mean diversities= 1.10025704E-03 1.10025564E-03

Zone 3: moderate selection zone

Lower and upper bounds of St metapopn gamma for zone 3

50.0000000 100.000000

Probability of zone 3= 5.86978197E-02

Contributions to mean load statistics over zone 3

Loads within In and St= 7.79079492E-05 7.79155016E-05

Load between In and St= 2.92227105E-05

Homozygous load for In and St= 2.90379074E-04 2.90408352E-04

Inbreeding loads= 2.12471132E-04 2.12492872E-04

Selection coefficients for In and St homokaryotypes

4.86969948E-05 4.86969948E-05

Contributions to mean A2 freqs= 4.13241178E-05 4.13299822E-05

Contributions to mean diversities= 6.71573362E-05 6.71686357E-05

Zone 4: strong selection zone

Upper limit to scaled gamma for St metapopn= 2.50000000

Lower limit to gamma for St metapopn= 100.000000

Upper limit to gamma for St metapopn= 16666.6680

Probability of zone 4= 0.673148870

Contributions to mean load statistics over zone 4

Loads within In and St= 1.33947132E-03 1.33947132E-03

Load between In and St= 5.56876010E-04

Homozygous load for In and St= 5.54918312E-03 5.54918312E-03

Inbreeding loads= 4.20971727E-03 4.20971727E-03

Selection coefficients for In and St homokaryotypes

7.82310963E-04 7.82310963E-04

Contributions to mean A2 freqs= 2.26076649E-04 2.26076649E-04

Contributions to mean diversities= 4.13788030E-06 4.13788030E-06

Mean load statistics over all zones

Loads within In and St= 6.51622750E-03 6.51619071E-03

Load between In and St= 2.25183042E-03

Homozygous load for In and St= 1.13184117E-02 1.13183968E-02

Inbreeding loads= 4.80219163E-03 4.80221305E-03

Selection coefficients for In and St homokaryotypes

4.25529480E-03 4.25529480E-03

Mean frequencies of A2 in In and St= 5.95978796E-02 5.95977567E-02

Ratio of these= 1.00000203

Mean diversities at selected sites in In and St= 1.58224057E-03 1.58225046E-03

Ratio of these= 0.999993742

Mean diversities at neutral sites in In and St= 7.84028973E-03 7.84028973E-03

pi-n/pi-s for In and St= 0.201808944 0.201810196

Ratio of these= 0.999993801

**Neutral Fst for whole population= 0.15**

**Scaled migration rate for whole population= 5.667**

Zone 1: quasi-neutral zone 1

Upper bound scaled selection coefficient for neutrality in St metapopulation= 0.250000000

Probability of zone 1= 5.23817725E-02

Integral of selection coefficient over zone 1= 3.02202563E-04

Mean load statistics for zone 1

Mean q1 and q2= 0.600000024

F1 and F2= 0.983700812 0.983700812

Diversities= 7.82360975E-03 7.82360975E-03

Contributions to loads within In and St= 1.80257615E-04 1.80257615E-04

Contributions to load between In and St = 1.16045783E-04

Contributions to homozygous loads for In and St= 1.81321549E-04 1.81321549E-04

Contributions to inbreeding loads for In and St= 1.06393679E-06 1.06393679E-06

Contributions to selection coefficients for In and St homokaryotypes

6.41942024E-05 6.41942024E-05

Contributions to mean A2 freqs= 3.14290635E-02 3.14290635E-02

Contributions to mean diversities= 4.09814558E-04 4.09814558E-04

Zone 2: quasi-neutral zone 2

Lower and upper bounds of St metapopn gamma for zone 2

0.250000000 50.0000000

Probability of zone 2= 0.203914478

Coefficients for bivariate distribution of q1 and q2 in metapopulation

G1= 0.352941185 G2= 0.352941185

G3= 0.294117630 G4= 0.294117630

a1= 0.104411766 a2= 0.104411766

b11= 3.30882333E-02 b12= 0.224999994 b22= 3.30882333E-02

Contributions to mean load statistics for zone 2

Loads within In and St= 5.56769222E-03 5.56756137E-03

Load between In and St= 1.76760962E-03

Homozygous load for In and St= 5.89067442E-03 5.89053938E-03

Inbreeding loads= 3.22985434E-04 3.22984793E-04

Selection coefficients against In and St homokaryotypes

3.79288197E-03 3.79276276E-03

Contributions to mean A2 freqs= 2.95160655E-02 2.95157414E-02

Contributions to mean diversities= 1.02385518E-03 1.02385192E-03

Zone 3: moderate selection zone

Lower and upper bounds of St metapopn gamma for zone 3

50.0000000 100.000000

Probability of zone 3= 5.86978197E-02

Contributions to mean load statistics over zone 3

Loads within In and St= 8.20176865E-05 8.20269706E-05

Load between In and St= 2.46444597E-05

Homozygous load for In and St= 2.45127972E-04 2.45149800E-04

Inbreeding loads= 1.63110322E-04 1.63122852E-04

Selection coefficients for In and St homokaryotypes

5.73992729E-05 5.73992729E-05

Contributions to mean A2 freqs= 3.48728390E-05 3.48772046E-05

Contributions to mean diversities= 5.15340980E-05 5.15408792E-05

Zone 4: strong selection zone

Upper limit to scaled gamma for St metapopn= 2.50000000

Lower limit to gamma for St metapopn= 100.000000

Upper limit to gamma for St metapopn= 16666.6680

Probability of zone 4= 0.673148870

Contributions to mean load statistics over zone 4

Loads within In and St= 1.51156390E-03 1.51156390E-03

Load between In and St= 5.36432955E-04

Homozygous load for In and St= 5.34592755E-03 5.34592709E-03

Inbreeding loads= 3.83436703E-03 3.83436680E-03

Selection coefficients for In and St homokaryotypes

9.74655151E-04 9.74655151E-04

Contributions to mean A2 freqs= 2.20083282E-04 2.20083282E-04

Contributions to mean diversities= 4.19538537E-06 4.19538537E-06

Mean load statistics over all zones

Loads within In and St= 7.34153157E-03 7.34141003E-03

Load between In and St= 2.44473293E-03

Homozygous load for In and St= 1.16630513E-02 1.16629377E-02

Inbreeding loads= 4.32152674E-03 4.32153838E-03

Selection coefficients for In and St homokaryotypes

4.88483906E-03 4.88471985E-03

Mean frequencies of A2 in In and St= 6.12000860E-02 6.11997619E-02

Ratio of these= 1.00000525

Mean diversities at selected sites in In and St= 1.48939923E-03 1.48940273E-03

Ratio of these= 0.999997675

Mean diversities at neutral sites in In and St= 7.82360975E-03 7.82360975E-03

pi-n/pi-s for In and St= 0.190372378 0.190372825

Ratio of these= 0.999997675

**Neutral Fst for whole population= 0.2**

**Scaled migration rate for whole population= 4**

Zone 1: quasi-neutral zone 1

Upper bound scaled selection coefficient for neutrality in St metapopulation= 0.250000000

Probability of zone 1= 5.23817725E-02

Integral of selection coefficient over zone 1= 3.02202563E-04

Mean load statistics for zone 1

Mean q1 and q2= 0.600000024

F1 and F2= 0.983739853 0.983739853

Diversities= 7.80487061E-03 7.80487061E-03

Contributions to loads within In and St= 1.80260147E-04 1.80260147E-04

Contributions to load between In and St = 1.16045783E-04

Contributions to homozygous loads for In and St= 1.81321549E-04 1.81321549E-04

Contributions to inbreeding loads for In and St= 1.06139692E-06 1.06139692E-06

Contributions to selection coefficients for In and St homokaryotypes

6.41942024E-05 6.41942024E-05

Contributions to mean A2 freqs= 3.14290635E-02 3.14290635E-02

Contributions to mean diversities= 4.08832944E-04 4.08832944E-04

Zone 2: quasi-neutral zone 2

Lower and upper bounds of St metapopn gamma for zone 2

0.250000000 50.0000000

Probability of zone 2= 0.203914478

Coefficients for bivariate distribution of q1 and q2 in metapopulation

G1= 0.500000000 G2= 0.500000000

G3= 0.00000000 G4= 0.00000000

a1= 0.137500003 a2= 0.137500003

b11= 0.00000000 b12= 0.224999994 b22= 0.00000000

Contributions to mean load statistics for zone 2

Loads within In and St= 6.40342012E-03 6.40286459E-03

Load between In and St= 2.07149261E-03

Homozygous load for In and St= 6.68001128E-03 6.67945342E-03

Inbreeding loads= 2.76591425E-04 2.76588806E-04

Selection coefficients against In and St homokaryotypes

4.32252884E-03 4.32199240E-03

Contributions to mean A2 freqs= 3.15015279E-02 3.15004326E-02

Contributions to mean diversities= 9.51772963E-04 9.51762544E-04

Zone 3: moderate selection zone

Lower and upper bounds of St metapopn gamma for zone 3

50.0000000 100.000000

Probability of zone 3= 5.86978197E-02

Contributions to mean load statistics over zone 3

Loads within In and St= 8.53240854E-05 8.53354504E-05

Load between In and St= 2.16791141E-05

Homozygous load for In and St= 2.15772248E-04 2.15789885E-04

Inbreeding loads= 1.30448127E-04 1.30454413E-04

Selection coefficients for In and St homokaryotypes

6.36577606E-05 6.36577606E-05

Contributions to mean A2 freqs= 3.06697693E-05 3.06732945E-05

Contributions to mean diversities= 4.11724577E-05 4.11764231E-05

Zone 4: strong selection zone

Upper limit to scaled gamma for St metapopn= 2.50000000

Lower limit to gamma for St metapopn= 100.000000

Upper limit to gamma for St metapopn= 16666.6680

Probability of zone 4= 0.673148870

Contributions to mean load statistics over zone 4

Loads within In and St= 1.63572980E-03 1.63572980E-03

Load between In and St= 5.17193635E-04

Homozygous load for In and St= 5.15475543E-03 5.15475543E-03

Inbreeding loads= 3.51902214E-03 3.51902191E-03

Selection coefficients for In and St homokaryotypes

1.11788511E-03 1.11788511E-03

Contributions to mean A2 freqs= 2.14698855E-04 2.14698855E-04

Contributions to mean diversities= 4.22959010E-06 4.22959056E-06

Mean load statistics over all zones

Loads within In and St= 8.30473378E-03 8.30418989E-03

Load between In and St= 2.72641121E-03

Homozygous load for In and St= 1.22318603E-02 1.22313201E-02

Inbreeding loads= 3.92712327E-03 3.92712653E-03

Selection coefficients for In and St homokaryotypes

5.56278229E-03 5.56224585E-03

Mean frequencies of A2 in In and St= 6.31759539E-02 6.31748661E-02

Ratio of these= 1.00001717

Mean diversities at selected sites in In and St= 1.40600803E-03 1.40600151E-03

Ratio of these= 1.00000465

Mean diversities at neutral sites in In and St= 7.80487061E-03 7.80487061E-03

pi-n/pi-s for In and St= 0.180144951 0.180144116

Ratio of these= 1.00000465

**Neutral Fst for whole population= 0.25**

**Scaled migration rate for whole population= 3**

Zone 1: quasi-neutral zone 1

Upper bound scaled selection coefficient for neutrality in St metapopulation= 0.250000000

Probability of zone 1= 5.23817725E-02

Integral of selection coefficient over zone 1= 3.02202563E-04

Mean load statistics for zone 1

Mean q1 and q2= 0.600000024

F1 and F2= 0.983783841 0.983783841

Diversities= 7.78375613E-03 7.78375613E-03

Contributions to loads within In and St= 1.80263029E-04 1.80263029E-04

Contributions to load between In and St = 1.16045783E-04

Contributions to homozygous loads for In and St= 1.81321549E-04 1.81321549E-04

Contributions to inbreeding loads for In and St= 1.05851495E-06 1.05851495E-06

Contributions to selection coefficients for In and St homokaryotypes

6.41942024E-05 6.41942024E-05

Contributions to mean A2 freqs= 3.14290635E-02 3.14290635E-02

Contributions to mean diversities= 4.07726940E-04 4.07726940E-04

Zone 2: quasi-neutral zone 2

Lower and upper bounds of St metapopn gamma for zone 2

0.250000000 50.0000000

Probability of zone 2= 0.203914478

Coefficients for bivariate distribution of q1 and q2 in metapopulation

G1= 0.666666687 G2= 0.666666687

G3= -0.333333373 G4= -0.333333373

a1= 0.175000012 a2= 0.175000012

b11= -3.75000052E-02 b12= 0.224999994 b22= -3.75000052E-02

Contributions to mean load statistics for zone 2

Loads within In and St= 7.48387305E-03 7.48015242E-03

Load between In and St= 2.50605051E-03

Homozygous load for In and St= 7.72114703E-03 7.71741616E-03

Inbreeding loads= 2.37282075E-04 2.37268163E-04

Selection coefficients against In and St homokaryotypes

4.96542454E-03 4.96172905E-03

Contributions to mean A2 freqs= 3.39579694E-02 3.39520089E-02

Contributions to mean diversities= 8.81948974E-04 8.81902699E-04

Zone 3: moderate selection zone

Lower and upper bounds of St metapopn gamma for zone 3

50.0000000 100.000000

Probability of zone 3= 5.86978197E-02

Contributions to mean load statistics over zone 3

Loads within In and St= 8.84484398E-05 8.84616384E-05

Load between In and St= 1.96679084E-05

Homozygous load for In and St= 1.95841189E-04 1.95856512E-04

Inbreeding loads= 1.07392640E-04 1.07394757E-04

Selection coefficients for In and St homokaryotypes

6.87837601E-05 6.87837601E-05

Contributions to mean A2 freqs= 2.78049993E-05 2.78080624E-05

Contributions to mean diversities= 3.38503160E-05 3.38523860E-05

Zone 4: strong selection zone

Upper limit to scaled gamma for St metapopn= 2.50000000

Lower limit to gamma for St metapopn= 100.000000

Upper limit to gamma for St metapopn= 16666.6680

Probability of zone 4= 0.673148870

Contributions to mean load statistics over zone 4

Loads within In and St= 1.73284730E-03 1.73284707E-03

Load between In and St= 5.00364229E-04

Homozygous load for In and St= 4.98751318E-03 4.98751318E-03

Inbreeding loads= 3.25466576E-03 3.25466529E-03

Selection coefficients for In and St homokaryotypes

1.23172998E-03 1.23172998E-03

Contributions to mean A2 freqs= 2.09949110E-04 2.09949110E-04

Contributions to mean diversities= 4.25144344E-06 4.25144344E-06

Mean load statistics over all zones

Loads within In and St= 9.48543195E-03 9.48172435E-03

Load between In and St= 3.14212847E-03

Homozygous load for In and St= 1.30858235E-02 1.30821075E-02

Inbreeding loads= 3.60039901E-03 3.60038667E-03

Selection coefficients for In and St homokaryotypes

6.32321835E-03 6.31952286E-03

Mean frequencies of A2 in In and St= 6.56247884E-02 6.56188279E-02

Ratio of these= 1.00009084

Mean diversities at selected sites in In and St= 1.32777775E-03 1.32773351E-03

Ratio of these= 1.00003326

Mean diversities at neutral sites in In and St= 7.78375613E-03 7.78375613E-03

pi-n/pi-s for In and St= 0.170583159 0.170577481

Ratio of these= 1.00003326

**h = 0.25**

**Neutral Fst for whole population= 5**

**Scaled migration rate for whole population= 19**

Zone 1: quasi-neutral zone 1

Upper bound scaled selection coefficient for neutrality in St metapopulation= 0.250000000

Probability of zone 1= 5.23817725E-02

Integral of selection coefficient over zone 1= 3.02202563E-04

Mean load statistics for zone 1

Mean q1 and q2= 0.600000024

F1 and F2= 0.983634770 0.983634770

Diversities= 7.85531010E-03 7.85531010E-03

Contributions to loads within In and St= 1.80728079E-04 1.80728079E-04

Contributions to load between In and St = 1.45057231E-04

Contributions to homozygous loads for In and St= 1.81321549E-04 1.81321549E-04

Contributions to inbreeding loads for In and St= 5.93463653E-07 5.93463653E-07

Contributions to selection coefficients for In and St homokaryotypes

3.56435776E-05 3.56435776E-05

Contributions to mean A2 freqs= 3.14290635E-02 3.14290635E-02

Contributions to mean diversities= 4.11475077E-04 4.11475077E-04

Zone 2: quasi-neutral zone 2

Lower and upper bounds of St metapopn gamma for zone 2

0.250000000 50.0000000

Probability of zone 2= 0.203914478

Coefficients for bivariate distribution of q1 and q2 in metapopulation

G1= 0.105263159 G2= 0.105263159

G3= 0.789473653 G4= 0.789473653

a1= 0.138157889 a2= 0.138157889

b11= 4.93421033E-02 b12= 0.125000000 b22= 4.93421033E-02

Contributions to mean load statistics for zone 2

Loads within In and St= 3.16637685E-03 3.16637685E-03

Load between In and St= 2.09090067E-03

Homozygous load for In and St= 3.30127613E-03 3.30127589E-03

Inbreeding loads= 1.34899776E-04 1.34899790E-04

Selection coefficients against In and St homokaryotypes

1.07491016E-03 1.07491016E-03

Contributions to mean A2 freqs= 2.46720556E-02 2.46720556E-02

Contributions to mean diversities= 8.74091871E-04 8.74091813E-04

Zone 3: moderate selection zone

Lower and upper bounds of St metapopn gamma for zone 3

50.0000000 100.000000

Probability of zone 3= 5.86978197E-02

Contributions to mean load statistics over zone 3

Loads within In and St= 7.48043385E-05 7.48100647E-05

Load between In and St= 6.81668535E-05

Homozygous load for In and St= 1.36281495E-04 1.36295261E-04

Inbreeding loads= 6.14771925E-05 6.14852324E-05

Selection coefficients for In and St homokaryotypes

6.61611557E-06 6.61611557E-06

Contributions to mean A2 freqs= 1.94860677E-05 1.94888180E-05

Contributions to mean diversities= 3.51503149E-05 3.51560266E-05

Zone 4: strong selection zone

Upper limit to scaled gamma for St metapopn= 2.50000000

Lower limit to gamma for St metapopn= 100.000000

Upper limit to gamma for St metapopn= 16666.6680

Probability of zone 4= 0.673148870

Contributions to mean load statistics over zone 4

Loads within In and St= 6.28015434E-04 6.28015434E-04

Load between In and St= 5.84317138E-04

Homozygous load for In and St= 1.16856408E-03 1.16856419E-03

Inbreeding loads= 5.40548877E-04 5.40548877E-04

Selection coefficients for In and St homokaryotypes

4.36902046E-05 4.36902046E-05

Contributions to mean A2 freqs= 3.99946184E-05 3.99946184E-05

Contributions to mean diversities= 7.03406442E-07 7.03406442E-07

Mean load statistics over all zones

Loads within In and St= 4.04992467E-03 4.04993026E-03

Load between In and St= 2.88844178E-03

Homozygous load for In and St= 4.78744321E-03 4.78745718E-03

Inbreeding loads= 7.37519353E-04 7.37527385E-04

Selection coefficients for In and St homokaryotypes

1.16080046E-03 1.16080046E-03

Mean frequencies of A2 in In and St= 5.61606027E-02 5.61606027E-02

Ratio of these= 1.00000000

Mean diversities at selected sites in In and St= 1.32142066E-03 1.32142636E-03

Ratio of these= 0.999995708

Mean diversities at neutral sites in In and St= 7.85531010E-03 7.85531010E-03

pi-n/pi-s for In and St= 0.168220043 0.168220773

Ratio of these= 0.999995649

**Neutral Fst for whole population= 0.1**

**Scaled migration rate for whole population= 9**

Zone 1: quasi-neutral zone 1

Upper bound scaled selection coefficient for neutrality in St metapopulation= 0.250000000

Probability of zone 1= 5.23817725E-02

Integral of selection coefficient over zone 1= 3.02202563E-04

Mean load statistics for zone 1

Mean q1 and q2= 0.600000024

F1 and F2= 0.983666062 0.983666062

Diversities= 7.84028973E-03 7.84028973E-03

Contributions to loads within In and St= 1.80729199E-04 1.80729199E-04

Contributions to load between In and St = 1.45057231E-04

Contributions to homozygous loads for In and St= 1.81321549E-04 1.81321549E-04

Contributions to inbreeding loads for In and St= 5.92346851E-07 5.92346851E-07

Contributions to selection coefficients for In and St homokaryotypes

3.56435776E-05 3.56435776E-05

Contributions to mean A2 freqs= 3.14290635E-02 3.14290635E-02

Contributions to mean diversities= 4.10688284E-04 4.10688284E-04

Zone 2: quasi-neutral zone 2

Lower and upper bounds of St metapopn gamma for zone 2

0.250000000 50.0000000

Probability of zone 2= 0.203914478

Coefficients for bivariate distribution of q1 and q2 in metapopulation

G1= 0.222222224 G2= 0.222222224

G3= 0.555555582 G4= 0.555555582

a1= 0.152777776 a2= 0.152777776

b11= 3.47222239E-02 b12= 0.125000000 b22= 3.47222239E-02

Contributions to mean load statistics for zone 2

Loads within In and St= 3.21564241E-03 3.21564241E-03

Load between In and St= 2.12361524E-03

Homozygous load for In and St= 3.34265083E-03 3.34265106E-03

Inbreeding loads= 1.27005333E-04 1.27005333E-04

Selection coefficients against In and St homokaryotypes

1.09142065E-03 1.09142065E-03

Contributions to mean A2 freqs= 2.49507744E-02 2.49507744E-02

Contributions to mean diversities= 8.47796851E-04 8.47796910E-04

Zone 3: moderate selection zone

Lower and upper bounds of St metapopn gamma for zone 3

50.0000000 100.000000

Probability of zone 3= 5.86978197E-02

Contributions to mean load statistics over zone 3

Loads within In and St= 7.93104409E-05 7.93179279E-05

Load between In and St= 6.72346505E-05

Homozygous load for In and St= 1.34419082E-04 1.34431335E-04

Inbreeding loads= 5.51086123E-05 5.51133708E-05

Selection coefficients for In and St homokaryotypes

1.20997429E-05 1.20997429E-05

Contributions to mean A2 freqs= 1.92232837E-05 1.92257339E-05

Contributions to mean diversities= 3.15100224E-05 3.15143880E-05

Zone 4: strong selection zone

Upper limit to scaled gamma for St metapopn= 2.50000000

Lower limit to gamma for St metapopn= 100.000000

Upper limit to gamma for St metapopn= 16666.6680

Probability of zone 4= 0.673148870

Contributions to mean load statistics over zone 4

Loads within In and St= 6.91077148E-04 6.91077148E-04

Load between In and St= 6.17152720E-04

Homozygous load for In and St= 1.23422651E-03 1.23422651E-03

Inbreeding loads= 5.43148024E-04 5.43148082E-04

Selection coefficients for In and St homokaryotypes

7.39097595E-05 7.39097595E-05

Contributions to mean A2 freqs= 4.30783257E-05 4.30783257E-05

Contributions to mean diversities= 7.95975211E-07 7.95975154E-07

Mean load statistics over all zones

Loads within In and St= 4.16675908E-03 4.16676654E-03

Load between In and St= 2.95305997E-03

Homozygous load for In and St= 4.89261793E-03 4.89263050E-03

Inbreeding loads= 7.25854305E-04 7.25859136E-04

Selection coefficients for In and St homokaryotypes

1.21295452E-03 1.21295452E-03

Mean frequencies of A2 in In and St= 5.64421415E-02 5.64421453E-02

Ratio of these= 0.999999940

Mean diversities at selected sites in In and St= 1.29079109E-03 1.29079563E-03

Ratio of these= 0.999996483

Mean diversities at neutral sites in In and St= 7.84028973E-03 7.84028973E-03

pi-n/pi-s for In and St= 0.164635628 0.164636210

Ratio of these= 0.999996483

**Neutral Fst for whole population= 0.15**

**Scaled migration rate for whole population= 5.667**

Zone 1: quasi-neutral zone 1

Upper bound scaled selection coefficient for neutrality in St metapopulation= 0.250000000

Probability of zone 1= 5.23817725E-02

Integral of selection coefficient over zone 1= 3.02202563E-04

Mean load statistics for zone 1

Mean q1 and q2= 0.600000024

F1 and F2= 0.983700812 0.983700812

Diversities= 7.82360975E-03 7.82360975E-03

Contributions to loads within In and St= 1.80730465E-04 1.80730465E-04

Contributions to load between In and St = 1.45057231E-04

Contributions to homozygous loads for In and St= 1.81321549E-04 1.81321549E-04

Contributions to inbreeding loads for In and St= 5.91085950E-07 5.91085950E-07

Contributions to selection coefficients for In and St homokaryotypes

3.56435776E-05 3.56435776E-05

Contributions to mean A2 freqs= 3.14290635E-02 3.14290635E-02

Contributions to mean diversities= 4.09814558E-04 4.09814558E-04

Zone 2: quasi-neutral zone 2

Lower and upper bounds of St metapopn gamma for zone 2

0.250000000 50.0000000

Probability of zone 2= 0.203914478

Coefficients for bivariate distribution of q1 and q2 in metapopulation

G1= 0.352941185 G2= 0.352941185

G3= 0.294117630 G4= 0.294117630

a1= 0.169117644 a2= 0.169117644

b11= 1.83823518E-02 b12= 0.125000000 b22= 1.83823518E-02

Contributions to mean load statistics for zone 2

Loads within In and St= 3.26789566E-03 3.26789520E-03

Load between In and St= 2.15954403E-03

Homozygous load for In and St= 3.38704907E-03 3.38704907E-03

Inbreeding loads= 1.19152683E-04 1.19152661E-04

Selection coefficients against In and St homokaryotypes

1.10775232E-03 1.10775232E-03

Contributions to mean A2 freqs= 2.52607446E-02 2.52607446E-02

Contributions to mean diversities= 8.20495188E-04 8.20495014E-04

Zone 3: moderate selection zone

Lower and upper bounds of St metapopn gamma for zone 3

50.0000000 100.000000

Probability of zone 3= 5.86978197E-02

Contributions to mean load statistics over zone 3

Loads within In and St= 8.34396560E-05 8.34476668E-05

Load between In and St= 6.65552288E-05

Homozygous load for In and St= 1.33061723E-04 1.33072797E-04

Inbreeding loads= 4.96220637E-05 4.96251196E-05

Selection coefficients for In and St homokaryotypes

1.68681145E-05 1.68681145E-05

Contributions to mean A2 freqs= 1.90284882E-05 1.90307001E-05

Contributions to mean diversities= 2.83656027E-05 2.83687750E-05

Zone 4: strong selection zone

Upper limit to scaled gamma for St metapopn= 2.50000000

Lower limit to gamma for St metapopn= 100.000000

Upper limit to gamma for St metapopn= 16666.6680

Probability of zone 4= 0.673148870

Contributions to mean load statistics over zone 4

Loads within In and St= 7.32879387E-04 7.32879387E-04

Load between In and St= 6.34657277E-04

Homozygous load for In and St= 1.26923132E-03 1.26923120E-03

Inbreeding loads= 5.36351465E-04 5.36351465E-04

Selection coefficients for In and St homokaryotypes

9.82284546E-05 9.82284546E-05

Contributions to mean A2 freqs= 4.47907769E-05 4.47907769E-05

Contributions to mean diversities= 8.59279908E-07 8.59279851E-07

Mean load statistics over all zones

Loads within In and St= 4.26494516E-03 4.26495261E-03

Load between In and St= 3.00581381E-03

Homozygous load for In and St= 4.97066369E-03 4.97067440E-03

Inbreeding loads= 7.05717306E-04 7.05720333E-04

Selection coefficients for In and St homokaryotypes

1.25831366E-03 1.25837326E-03

Mean frequencies of A2 in In and St= 5.67536242E-02 5.67536280E-02

Ratio of these= 0.999999940

Mean diversities at selected sites in In and St= 1.25953462E-03 1.25953765E-03

Ratio of these= 0.999997616

Mean diversities at neutral sites in In and St= 7.82360975E-03 7.82360975E-03

pi-n/pi-s for In and St= 0.160991490 0.160991877

Ratio of these= 0.999997616

**Neutral Fst for whole population= 0.2**

**Scaled migration rate for whole population= 4**

Zone 1: quasi-neutral zone 1

Upper bound scaled selection coefficient for neutrality in St metapopulation= 0.250000000

Probability of zone 1= 5.23817725E-02

Integral of selection coefficient over zone 1= 3.02202563E-04

Mean load statistics for zone 1

Mean q1 and q2= 0.600000024

F1 and F2= 0.983739853 0.983739853

Diversities= 7.80487061E-03 7.80487061E-03

Contributions to loads within In and St= 1.80731877E-04 1.80731877E-04

Contributions to load between In and St = 1.45057231E-04

Contributions to homozygous loads for In and St= 1.81321549E-04 1.81321549E-04

Contributions to inbreeding loads for In and St= 5.89662989E-07 5.89662989E-07

Contributions to selection coefficients for In and St homokaryotypes

3.57031822E-05 3.57031822E-05

Contributions to mean A2 freqs= 3.14290635E-02 3.14290635E-02

Contributions to mean diversities= 4.08832944E-04 4.08832944E-04

Zone 2: quasi-neutral zone 2

Lower and upper bounds of St metapopn gamma for zone 2

0.250000000 50.0000000

Probability of zone 2= 0.203914478

Coefficients for bivariate distribution of q1 and q2 in metapopulation

G1= 0.500000000 G2= 0.500000000

G3= 0.00000000 G4= 0.00000000

a1= 0.187500000 a2= 0.187500000

b11= 0.00000000 b12= 0.125000000 b22= 0.00000000

Contributions to mean load statistics for zone 2

Loads within In and St= 3.32378317E-03 3.32378317E-03

Load between In and St= 2.19939509E-03

Homozygous load for In and St= 3.43513023E-03 3.43513023E-03

Inbreeding loads= 1.11346359E-04 1.11346366E-04

Selection coefficients against In and St homokaryotypes

1.12378597E-03 1.12378597E-03

Contributions to mean A2 freqs= 2.56077126E-02 2.56077107E-02

Contributions to mean diversities= 7.92093982E-04 7.92094041E-04

Zone 3: moderate selection zone

Lower and upper bounds of St metapopn gamma for zone 3

50.0000000 100.000000

Probability of zone 3= 5.86978197E-02

Contributions to mean load statistics over zone 3

Loads within In and St= 8.74188918E-05 8.74278994E-05

Load between In and St= 6.61602026E-05

Homozygous load for In and St= 1.32272588E-04 1.32282861E-04

Inbreeding loads= 4.48536412E-05 4.48548926E-05

Selection coefficients for In and St homokaryotypes

2.12788582E-05 2.12788582E-05

Contributions to mean A2 freqs= 1.89117982E-05 1.89138500E-05

Contributions to mean diversities= 2.56276999E-05 2.56298081E-05

Zone 4: strong selection zone

Upper limit to scaled gamma for St metapopn= 2.50000000

Lower limit to gamma for St metapopn= 100.000000

Upper limit to gamma for St metapopn= 16666.6680

Probability of zone 4= 0.673148870

Contributions to mean load statistics over zone 4

Loads within In and St= 7.67980935E-04 7.67980935E-04

Load between In and St= 6.48355170E-04

Homozygous load for In and St= 1.29662245E-03 1.29662245E-03

Inbreeding loads= 5.28642209E-04 5.28642209E-04

Selection coefficients for In and St homokaryotypes

1.19626522E-04 1.19626522E-04

Contributions to mean A2 freqs= 4.61987220E-05 4.61987220E-05

Contributions to mean diversities= 9.13340102E-07 9.13340102E-07

Mean load statistics over all zones

Loads within In and St= 4.35991492E-03 4.35992423E-03

Load between In and St= 3.05896765E-03

Homozygous load for In and St= 5.04534692E-03 5.04535716E-03

Inbreeding loads= 6.85431878E-04 6.85433159E-04

Selection coefficients for In and St homokaryotypes

1.30009651E-03 1.30009651E-03

Mean frequencies of A2 in In and St= 5.71018867E-02 5.71018830E-02

Ratio of these= 1.00000012

Mean diversities at selected sites in In and St= 1.22746802E-03 1.22747011E-03

Ratio of these= 0.999998271

Mean diversities at neutral sites in In and St= 7.80487061E-03 7.80487061E-03

pi-n/pi-s for In and St= 0.157269493 0.157269761

Ratio of these= 0.999998271

**Neutral Fst for whole population= 0.25**

**Scaled migration rate for whole population= 3**

Zone 1: quasi-neutral zone 1

Upper bound scaled selection coefficient for neutrality in St metapopulation= 0.250000000

Probability of zone 1= 5.23817725E-02

Integral of selection coefficient over zone 1= 3.02202563E-04

Mean load statistics for zone 1

Mean q1 and q2= 0.600000024

F1 and F2= 0.983783841 0.983783841

Diversities= 7.78375613E-03 7.78375613E-03

Contributions to loads within In and St= 1.80733492E-04 1.80733492E-04

Contributions to load between In and St = 1.45057231E-04

Contributions to homozygous loads for In and St= 1.81321549E-04 1.81321549E-04

Contributions to inbreeding loads for In and St= 5.88059834E-07 5.88059834E-07

Contributions to selection coefficients for In and St homokaryotypes

3.57031822E-05 3.57031822E-05

Contributions to mean A2 freqs= 3.14290635E-02 3.14290635E-02

Contributions to mean diversities= 4.07726940E-04 4.07726940E-04

Zone 2: quasi-neutral zone 2

Lower and upper bounds of St metapopn gamma for zone 2

0.250000000 50.0000000

Probability of zone 2= 0.203914478

Coefficients for bivariate distribution of q1 and q2 in metapopulation

G1= 0.666666687 G2= 0.666666687

G3= -0.333333373 G4= -0.333333373

a1= 0.208333343 a2= 0.208333343

b11= -2.08333358E-02 b12= 0.125000000 b22= -2.08333358E-02

Contributions to mean load statistics for zone 2

Loads within In and St= 3.38411680E-03 3.38411680E-03

Load between In and St= 2.24406319E-03

Homozygous load for In and St= 3.48770362E-03 3.48770362E-03

Inbreeding loads= 1.03589227E-04 1.03589227E-04

Selection coefficients against In and St homokaryotypes

1.13940239E-03 1.13940239E-03

Contributions to mean A2 freqs= 2.59989742E-02 2.59989742E-02

Contributions to mean diversities= 7.62471172E-04 7.62471231E-04

Zone 3: moderate selection zone

Lower and upper bounds of St metapopn gamma for zone 3

50.0000000 100.000000

Probability of zone 3= 5.86978197E-02

Contributions to mean load statistics over zone 3

Loads within In and St= 9.14087868E-05 9.14186239E-05

Load between In and St= 6.60613150E-05

Homozygous load for In and St= 1.32075147E-04 1.32084955E-04

Inbreeding loads= 4.06663639E-05 4.06663567E-05

Selection coefficients for In and St homokaryotypes

2.53319740E-05 2.53319740E-05

Contributions to mean A2 freqs= 1.88768699E-05 1.88788326E-05

Contributions to mean diversities= 2.32200418E-05 2.32211969E-05

Zone 4: strong selection zone

Upper limit to scaled gamma for St metapopn= 2.50000000

Lower limit to gamma for St metapopn= 100.000000

Upper limit to gamma for St metapopn= 16666.6680

Probability of zone 4= 0.673148870

Contributions to mean load statistics over zone 4

Loads within In and St= 8.00682872E-04 8.00682872E-04

Load between In and St= 6.61086291E-04

Homozygous load for In and St= 1.32208131E-03 1.32208131E-03

Inbreeding loads= 5.21398848E-04 5.21398906E-04

Selection coefficients for In and St homokaryotypes

1.39594078E-04 1.39594078E-04

Contributions to mean A2 freqs= 4.75797897E-05 4.75797897E-05

Contributions to mean diversities= 9.64345531E-07 9.64345418E-07

Mean load statistics over all zones

Loads within In and St= 4.45694197E-03 4.45695175E-03

Load between In and St= 3.11626797E-03

Homozygous load for In and St= 5.12318127E-03 5.12319151E-03

Inbreeding loads= 6.66242500E-04 6.66242559E-04

Selection coefficients for In and St homokaryotypes

1.33979321E-03 1.33979321E-03

Mean frequencies of A2 in In and St= 5.74944951E-02 5.74944988E-02

Ratio of these= 0.999999940

Mean diversities at selected sites in In and St= 1.19438255E-03 1.19438383E-03

Ratio of these= 0.999998927

Mean diversities at neutral sites in In and St= 7.78375613E-03 7.78375613E-03

pi-n/pi-s for In and St= 0.153445527 0.153445691

Ratio of these= 0.999998927

**h= 0.45**

**Neutral Fst for whole population= 0.05**

**Scaled migration rate for whole population= 19**

Zone 1: quasi-neutral zone 1

Upper bound scaled selection coefficient for neutrality in St metapopulation= 0.250000000

Probability of zone 1= 5.23817725E-02

Integral of selection coefficient over zone 1= 3.02202563E-04

Mean load statistics for zone 1

Mean q1 and q2= 0.600000024

F1 and F2= 0.983634770 0.983634770

Diversities= 7.85531010E-03 7.85531010E-03

Contributions to loads within In and St= 1.81202864E-04 1.81202864E-04

Contributions to load between In and St = 1.74068671E-04

Contributions to homozygous loads for In and St= 1.81321549E-04 1.81321549E-04

Contributions to inbreeding loads for In and St= 1.18685527E-07 1.18685527E-07

Contributions to selection coefficients for In and St homokaryotypes

7.15255737E-06 7.15255737E-06

Contributions to mean A2 freqs= 3.14290635E-02 3.14290635E-02

Contributions to mean diversities= 4.11475077E-04 4.11475077E-04

Zone 2: quasi-neutral zone 2

Lower and upper bounds of St metapopn gamma for zone 2

0.250000000 50.0000000

Probability of zone 2= 0.203914478

Coefficients for bivariate distribution of q1 and q2 in metapopulation

G1= 0.105263159 G2= 0.105263159

G3= 0.789473653 G4= 0.789473653

a1= 0.227631569 a2= 0.227631569

b11= 9.86842345E-03 b12= 2.50000060E-02 b22= 9.86842345E-03

Contributions to mean load statistics for zone 2

Loads within In and St= 2.60954630E-03 2.60954630E-03

Load between In and St= 2.44417251E-03

Homozygous load for In and St= 2.62797833E-03 2.62797833E-03

Inbreeding loads= 1.84341570E-05 1.84341570E-05

Selection coefficients against In and St homokaryotypes

1.65343285E-04 1.65343285E-04

Contributions to mean A2 freqs= 2.33488828E-02 2.33488828E-02

Contributions to mean diversities= 7.23401550E-04 7.23401841E-04

Zone 3: moderate selection zone

Lower and upper bounds of St metapopn gamma for zone 3

50.0000000 100.000000

Probability of zone 3= 5.86978197E-02

Contributions to mean load statistics over zone 3

Loads within In and St= 7.27446895E-05 7.27512088E-05

Load between In and St= 7.19905220E-05

Homozygous load for In and St= 7.99840855E-05 7.99914560E-05

Inbreeding loads= 7.23937228E-06 7.24021402E-06

Selection coefficients for In and St homokaryotypes

7.74860382E-07 7.74860382E-07

Contributions to mean A2 freqs= 1.14548611E-05 1.14563363E-05

Contributions to mean diversities= 2.07305930E-05 2.07335397E-05

Zone 4: strong selection zone

Upper limit to scaled gamma for St metapopn= 2.50000000

Lower limit to gamma for St metapopn= 100.000000

Upper limit to gamma for St metapopn= 16666.6680

Probability of zone 4= 0.673148870

Contributions to mean load statistics over zone 4

Loads within In and St= 5.94093406E-04 5.94093406E-04

Load between In and St= 5.89846226E-04

Homozygous load for In and St= 6.55382231E-04 6.55382231E-04

Inbreeding loads= 6.12892400E-05 6.12892400E-05

Selection coefficients for In and St homokaryotypes

4.23192978E-06 4.23192978E-06

Contributions to mean A2 freqs= 2.16709832E-05 2.16709832E-05

Contributions to mean diversities= 3.81428237E-07 3.81428208E-07

Mean load statistics over all zones

Loads within In and St= 3.45758721E-03 3.45759373E-03

Load between In and St= 3.28007783E-03

Homozygous load for In and St= 3.54466611E-03 3.54467356E-03

Inbreeding loads= 8.70814547E-05 8.70822987E-05

Selection coefficients for In and St homokaryotypes

1.77502632E-04 1.77502632E-04

Mean frequencies of A2 in In and St= 5.48110716E-02 5.48110716E-02

Ratio of these= 1.00000000

Mean diversities at selected sites in In and St= 1.15598855E-03 1.15599181E-03

Ratio of these= 0.999997199

Mean diversities at neutral sites in In and St= 7.85531010E-03 7.85531010E-03

pi-n/pi-s for In and St= 0.147160143 0.147160560

Ratio of these= 0.999997139

**Neutral Fst for whole population= 0.1**

**Scaled migration rate for whole population= 9**

Zone 1: quasi-neutral zone 1

Upper bound scaled selection coefficient for neutrality in St metapopulation= 0.250000000

Probability of zone 1= 5.23817725E-02

Integral of selection coefficient over zone 1= 3.02202563E-04

Mean load statistics for zone 1

Mean q1 and q2= 0.600000024

F1 and F2= 0.983666062 0.983666062

Diversities= 7.84028973E-03 7.84028973E-03

Contributions to loads within In and St= 1.81203082E-04 1.81203082E-04

Contributions to load between In and St = 1.74068671E-04

Contributions to homozygous loads for In and St= 1.81321549E-04 1.81321549E-04

Contributions to inbreeding loads for In and St= 1.18469373E-07 1.18469373E-07

Contributions to selection coefficients for In and St homokaryotypes

7.15255737E-06 7.15255737E-06

Contributions to mean A2 freqs= 3.14290635E-02 3.14290635E-02

Contributions to mean diversities= 4.10688284E-04 4.10688284E-04

Zone 2: quasi-neutral zone 2

Lower and upper bounds of St metapopn gamma for zone 2

0.250000000 50.0000000

Probability of zone 2= 0.203914478

Coefficients for bivariate distribution of q1 and q2 in metapopulation

G1= 0.222222224 G2= 0.222222224

G3= 0.555555582 G4= 0.555555582

a1= 0.230555549 a2= 0.230555549

b11= 6.94444636E-03 b12= 2.50000060E-02 b22= 6.94444636E-03

Contributions to mean load statistics for zone 2

Loads within In and St= 2.63480633E-03 2.63480633E-03

Load between In and St= 2.46718014E-03

Homozygous load for In and St= 2.65315385E-03 2.65315361E-03

Inbreeding loads= 1.83452703E-05 1.83452703E-05

Selection coefficients against In and St homokaryotypes

1.67608261E-04 1.67608261E-04

Contributions to mean A2 freqs= 2.34131720E-02 2.34131720E-02

Contributions to mean diversities= 7.21265387E-04 7.21265271E-04

Zone 3: moderate selection zone

Lower and upper bounds of St metapopn gamma for zone 3

50.0000000 100.000000

Probability of zone 3= 5.86978197E-02

Contributions to mean load statistics over zone 3

Loads within In and St= 7.77214664E-05 7.77280584E-05

Load between In and St= 7.62338823E-05

Homozygous load for In and St= 8.46987750E-05 8.47057890E-05

Inbreeding loads= 6.97727046E-06 6.97769110E-06

Selection coefficients for In and St homokaryotypes

1.49011612E-06 1.49011612E-06

Contributions to mean A2 freqs= 1.21348940E-05 1.21362964E-05

Contributions to mean diversities= 1.99832120E-05 1.99856331E-05

Zone 4: strong selection zone

Upper limit to scaled gamma for St metapopn= 2.50000000

Lower limit to gamma for St metapopn= 100.000000

Upper limit to gamma for St metapopn= 16666.6680

Probability of zone 4= 0.673148870

Contributions to mean load statistics over zone 4

Loads within In and St= 6.38950092E-04 6.38950092E-04

Load between In and St= 6.31737348E-04

Homozygous load for In and St= 7.01927813E-04 7.01927813E-04

Inbreeding loads= 6.29776769E-05 6.29776769E-05

Selection coefficients for In and St homokaryotypes

7.21216202E-06 7.21216202E-06

Contributions to mean A2 freqs= 2.40326026E-05 2.40326026E-05

Contributions to mean diversities= 4.44276225E-07 4.44276225E-07

Mean load statistics over all zones

Loads within In and St= 3.53268092E-03 3.53268743E-03

Load between In and St= 3.34921991E-03

Homozygous load for In and St= 3.62110185E-03 3.62110883E-03

Inbreeding loads= 8.84186884E-05 8.84191104E-05

Selection coefficients for In and St homokaryotypes

1.83463097E-04 1.83463097E-04

Mean frequencies of A2 in In and St= 5.48783988E-02 5.48784025E-02

Ratio of these= 0.999999940

Mean diversities at selected sites in In and St= 1.15238107E-03 1.15238340E-03

Ratio of these= 0.999997973

Mean diversities at neutral sites in In and St= 7.84028973E-03 7.84028973E-03

pi-n/pi-s for In and St= 0.146981955 0.146982253

Ratio of these= 0.999997973

**Neutral Fst for whole population= 0.15**

**Scaled migration rate for whole population= 5.667**

Zone 1: quasi-neutral zone 1

Upper bound scaled selection coefficient for neutrality in St metapopulation= 0.250000000

Probability of zone 1= 5.23817725E-02

Integral of selection coefficient over zone 1= 3.02202563E-04

Mean load statistics for zone 1

Mean q1 and q2= 0.600000024

F1 and F2= 0.983700812 0.983700812

Diversities= 7.82360975E-03 7.82360975E-03

Contributions to loads within In and St= 1.81203330E-04 1.81203330E-04

Contributions to load between In and St = 1.74068671E-04

Contributions to homozygous loads for In and St= 1.81321549E-04 1.81321549E-04

Contributions to inbreeding loads for In and St= 1.18217194E-07 1.18217194E-07

Contributions to selection coefficients for In and St homokaryotypes

7.15255737E-06 7.15255737E-06

Contributions to mean A2 freqs= 3.14290635E-02 3.14290635E-02

Contributions to mean diversities= 4.09814558E-04 4.09814558E-04

Zone 2: quasi-neutral zone 2

Lower and upper bounds of St metapopn gamma for zone 2

0.250000000 50.0000000

Probability of zone 2= 0.203914478

Coefficients for bivariate distribution of q1 and q2 in metapopulation

G1= 0.352941185 G2= 0.352941185

G3= 0.294117630 G4= 0.294117630

a1= 0.233823523 a2= 0.233823523

b11= 3.67647130E-03 b12= 2.50000060E-02 b22= 3.67647130E-03

Contributions to mean load statistics for zone 2

Loads within In and St= 2.66269711E-03 2.66269711E-03

Load between In and St= 2.49258569E-03

Homozygous load for In and St= 2.68094265E-03 2.68094265E-03

Inbreeding loads= 1.82447238E-05 1.82447311E-05

Selection coefficients against In and St homokaryotypes

1.70111656E-04 1.70111656E-04

Contributions to mean A2 freqs= 2.34844629E-02 2.34844610E-02

Contributions to mean diversities= 7.18847616E-04 7.18848023E-04

Zone 3: moderate selection zone

Lower and upper bounds of St metapopn gamma for zone 3

50.0000000 100.000000

Probability of zone 3= 5.86978197E-02

Contributions to mean load statistics over zone 3

Loads within In and St= 8.25902462E-05 8.25967218E-05

Load between In and St= 8.03682051E-05

Homozygous load for In and St= 8.92924654E-05 8.92992466E-05

Inbreeding loads= 6.70222789E-06 6.70253394E-06

Selection coefficients for In and St homokaryotypes

2.20537186E-06 2.20537186E-06

Contributions to mean A2 freqs= 1.27989579E-05 1.28003112E-05

Contributions to mean diversities= 1.91983763E-05 1.92002535E-05

Zone 4: strong selection zone

Upper limit to scaled gamma for St metapopn= 2.50000000

Lower limit to gamma for St metapopn= 100.000000

Upper limit to gamma for St metapopn= 16666.6680

Probability of zone 4= 0.673148870

Contributions to mean load statistics over zone 4

Loads within In and St= 6.67168002E-04 6.67167886E-04

Load between In and St= 6.57463970E-04

Homozygous load for In and St= 7.30512606E-04 7.30512606E-04

Inbreeding loads= 6.33442032E-05 6.33442032E-05

Selection coefficients for In and St homokaryotypes

9.71555710E-06 9.71555710E-06

Contributions to mean A2 freqs= 2.55340492E-05 2.55340492E-05

Contributions to mean diversities= 4.90021989E-07 4.90021989E-07

Mean load statistics over all zones

Loads within In and St= 3.59365856E-03 3.59366485E-03

Load between In and St= 3.40448646E-03

Homozygous load for In and St= 3.68206925E-03 3.68207600E-03

Inbreeding loads= 8.84093752E-05 8.84096880E-05

Selection coefficients for In and St homokaryotypes

1.89125538E-04 1.89185143E-04

Mean frequencies of A2 in In and St= 5.49518615E-02 5.49518578E-02

Ratio of these= 1.00000012

Mean diversities at selected sites in In and St= 1.14835054E-03 1.14835287E-03

Ratio of these= 0.999997973

Mean diversities at neutral sites in In and St= 7.82360975E-03 7.82360975E-03

pi-n/pi-s for In and St= 0.146780133 0.146780431

Ratio of these= 0.999997973

**Neutral Fst for whole population= 0.2**

**Scaled migration rate for whole population= 4**

Zone 1: quasi-neutral zone 1

Upper bound scaled selection coefficient for neutrality in St metapopulation= 0.250000000

Probability of zone 1= 5.23817725E-02

Integral of selection coefficient over zone 1= 3.02202563E-04

Mean load statistics for zone 1

Mean q1 and q2= 0.600000024

F1 and F2= 0.983739853 0.983739853

Diversities= 7.80487061E-03 7.80487061E-03

Contributions to loads within In and St= 1.81203621E-04 1.81203621E-04

Contributions to load between In and St = 1.74068671E-04

Contributions to homozygous loads for In and St= 1.81321549E-04 1.81321549E-04

Contributions to inbreeding loads for In and St= 1.17928991E-07 1.17928991E-07

Contributions to selection coefficients for In and St homokaryotypes

7.15255737E-06 7.15255737E-06

Contributions to mean A2 freqs= 3.14290635E-02 3.14290635E-02

Contributions to mean diversities= 4.08832944E-04 4.08832944E-04

Zone 2: quasi-neutral zone 2

Lower and upper bounds of St metapopn gamma for zone 2

0.250000000 50.0000000

Probability of zone 2= 0.203914478

Coefficients for bivariate distribution of q1 and q2 in metapopulation

G1= 0.500000000 G2= 0.500000000

G3= 0.00000000 G4= 0.00000000

a1= 0.237499997 a2= 0.237499997

b11= 0.00000000 b12= 2.50000060E-02 b22= 0.00000000

Contributions to mean load statistics for zone 2

Loads within In and St= 2.69363518E-03 2.69363518E-03

Load between In and St= 2.52077379E-03

Homozygous load for In and St= 2.71176384E-03 2.71176384E-03

Inbreeding loads= 1.81300366E-05 1.81300366E-05

Selection coefficients against In and St homokaryotypes

1.72853470E-04 1.72853470E-04

Contributions to mean A2 freqs= 2.35639792E-02 2.35639792E-02

Contributions to mean diversities= 7.16089446E-04 7.16089329E-04

Zone 3: moderate selection zone

Lower and upper bounds of St metapopn gamma for zone 3

50.0000000 100.000000

Probability of zone 3= 5.86978197E-02

Contributions to mean load statistics over zone 3

Loads within In and St= 8.74343605E-05 8.74409161E-05

Load between In and St= 8.44707247E-05

Homozygous load for In and St= 9.38505473E-05 9.38571975E-05

Inbreeding loads= 6.41624365E-06 6.41633824E-06

Selection coefficients for In and St homokaryotypes

2.98023224E-06 2.98023224E-06

Contributions to mean A2 freqs= 1.34587863E-05 1.34601150E-05

Contributions to mean diversities= 1.83814464E-05 1.83827433E-05

Zone 4: strong selection zone

Upper limit to scaled gamma for St metapopn= 2.50000000

Lower limit to gamma for St metapopn= 100.000000

Upper limit to gamma for St metapopn= 16666.6680

Probability of zone 4= 0.673148870

Contributions to mean load statistics over zone 4

Loads within In and St= 6.90774235E-04 6.90774235E-04

Load between In and St= 6.78767858E-04

Homozygous load for In and St= 7.54181994E-04 7.54181994E-04

Inbreeding loads= 6.34083335E-05 6.34083481E-05

Selection coefficients for In and St homokaryotypes

1.19805336E-05 1.19805336E-05

Contributions to mean A2 freqs= 2.68622316E-05 2.68622316E-05

Contributions to mean diversities= 5.31261605E-07 5.31261605E-07

Mean load statistics over all zones

Loads within In and St= 3.65304761E-03 3.65305413E-03

Load between In and St= 3.45808105E-03

Homozygous load for In and St= 3.74111789E-03 3.74112464E-03

Inbreeding loads= 8.80725420E-05 8.80726511E-05

Selection coefficients for In and St homokaryotypes

1.94966793E-04 1.94966793E-04

Mean frequencies of A2 in In and St= 5.50333634E-02 5.50333634E-02

Ratio of these= 1.00000000

Mean diversities at selected sites in In and St= 1.14383514E-03 1.14383642E-03

Ratio of these= 0.999998868

Mean diversities at neutral sites in In and St= 7.80487061E-03 7.80487061E-03

pi-n/pi-s for In and St= 0.146554023 0.146554187

Ratio of these= 0.999998868

**Neutral Fst for whole population= 0.25**

**Scaled migration rate for whole population= 3**

Zone 1: quasi-neutral zone 1

Upper bound scaled selection coefficient for neutrality in St metapopulation= 0.250000000

Probability of zone 1= 5.23817725E-02

Integral of selection coefficient over zone 1= 3.02202563E-04

Mean load statistics for zone 1

Mean q1 and q2= 0.600000024

F1 and F2= 0.983783841 0.983783841

Diversities= 7.78375613E-03 7.78375613E-03

Contributions to loads within In and St= 1.81203941E-04 1.81203941E-04

Contributions to load between In and St = 1.74068671E-04

Contributions to homozygous loads for In and St= 1.81321549E-04 1.81321549E-04

Contributions to inbreeding loads for In and St= 1.17604763E-07 1.17604763E-07

Contributions to selection coefficients for In and St homokaryotypes

7.15255737E-06 7.15255737E-06

Contributions to mean A2 freqs= 3.14290635E-02 3.14290635E-02

Contributions to mean diversities= 4.07726940E-04 4.07726940E-04

Zone 2: quasi-neutral zone 2

Lower and upper bounds of St metapopn gamma for zone 2

0.250000000 50.0000000

Probability of zone 2= 0.203914478

Coefficients for bivariate distribution of q1 and q2 in metapopulation

G1= 0.666666687 G2= 0.666666687

G3= -0.333333373 G4= -0.333333373

a1= 0.241666660 a2= 0.241666660

b11= -4.16666828E-03 b12= 2.50000060E-02 b22= -4.16666828E-03

Contributions to mean load statistics for zone 2

Loads within In and St= 2.72812787E-03 2.72812787E-03

Load between In and St= 2.55220034E-03

Homozygous load for In and St= 2.74612592E-03 2.74612592E-03

Inbreeding loads= 1.79983344E-05 1.79983308E-05

Selection coefficients against In and St homokaryotypes

1.75893307E-04 1.75893307E-04

Contributions to mean A2 freqs= 2.36532371E-02 2.36532371E-02

Contributions to mean diversities= 7.12916255E-04 7.12916022E-04

Zone 3: moderate selection zone

Lower and upper bounds of St metapopn gamma for zone 3

50.0000000 100.000000

Probability of zone 3= 5.86978197E-02

Contributions to mean load statistics over zone 3

Loads within In and St= 9.23402331E-05 9.23469997E-05

Load between In and St= 8.86213747E-05

Homozygous load for In and St= 9.84623839E-05 9.84690487E-05

Inbreeding loads= 6.12205622E-06 6.12197209E-06

Selection coefficients for In and St homokaryotypes

3.69548798E-06 3.75509262E-06

Contributions to mean A2 freqs= 1.41263945E-05 1.41277296E-05

Contributions to mean diversities= 1.75397254E-05 1.75404111E-05

Zone 4: strong selection zone

Upper limit to scaled gamma for St metapopn= 2.50000000

Lower limit to gamma for St metapopn= 100.000000

Upper limit to gamma for St metapopn= 16666.6680

Probability of zone 4= 0.673148870

Contributions to mean load statistics over zone 4

Loads within In and St= 7.12970563E-04 7.12970563E-04

Load between In and St= 6.98715216E-04

Homozygous load for In and St= 7.76347704E-04 7.76347704E-04

Inbreeding loads= 6.33768359E-05 6.33768359E-05

Selection coefficients for In and St homokaryotypes

1.42455101E-05 1.42455101E-05

Contributions to mean A2 freqs= 2.81929260E-05 2.81929260E-05

Contributions to mean diversities= 5.71760097E-07 5.71760040E-07

Mean load statistics over all zones

Loads within In and St= 3.71464249E-03 3.71464924E-03

Load between In and St= 3.51360557E-03

Homozygous load for In and St= 3.80225759E-03 3.80226411E-03

Inbreeding loads= 8.76148333E-05 8.76147460E-05

Selection coefficients for In and St homokaryotypes

2.01046467E-04 2.01046467E-04

Mean frequencies of A2 in In and St= 5.51246181E-02 5.51246181E-02

Ratio of these= 1.00000000

Mean diversities at selected sites in In and St= 1.13875465E-03 1.13875512E-03

Ratio of these= 0.999999583

Mean diversities at neutral sites in In and St= 7.78375613E-03 7.78375613E-03

pi-n/pi-s for In and St= 0.146298856 0.146298915

Ratio of these= 0.999999583
