# Supplementary material for: The fitness consequences of genetic divergence between polymorphic gene arrangements
Source: Genetics. 2023 Dec 26;226(3):iyad218. doi: 10.1093/genetics/iyad218 (PMC11090464; doi:10.1093/genetics/iyad218)
Supplement: iyad218_Supplementary_Data [file iyad218_supplementary_data.zip › Supplementary_Table_4_GENETICS-2023-306559.docx]

**Supplementary Table S4 Results for a subdivided population with a small deme size**

**Results are shown for a total population size of 10^6^, deme size of 500, inversion frequencies of 0.1 and 0.5, dominance coefficients of h = 0.05, 0.25, and 0.45, and F_ST_ values of 0.05, 0.1, 0.15, 0.2 and 0.25**

**Mutation rate towards deleterious variants= 4.99999997E-09**

**Number of selected sites in inversion= 100000**

**Mutational bias towards deleterious variants= 1.50000000**

**No. of values for Simpsons rule = 650**

**Sample size= 20**

**Wattersons correction factor= 3.54773974**

**Number of dominance coefficients modelled= 3**

**Shape parameter= 0.300000012**

**Upper bound gamma value for neutrality in the St popn= 0.250000000**

**Upper bound whole popn gamma value for zone 2a with h=0.25 = 50.0000000**

**Upper bound whole popn gamma value for zone 2b with h=0.25 = 500.000000**

**Upper limit to gamma distribution of z= x/scale parameter = 2.50000000**

**Threshold gamma value factor for use of approximate p.d.f.= 0.250000000**

**Section 1**

**Mean selection coefficient= 2.0 E-03**

**Mean scaled selection coefficient for whole popn= 4000**

**Inversion frequency= 0.1**

**h= 0.05**

Neutral Fst for whole population= 5.00000007E-02

Scaled migration rate for whole population= 19.0000000

Zone 1: quasi-neutral zone 1

Upper bound scaled selection coefficient for neutrality in St metapopulation= 0.250000000

Probability of zone 1= 4.39136960E-02

Integral of selection coefficient over zone 1= 1.40749034E-04

Mean load statistics for zone 1

Mean q1 and q2= 0.600000024

F1 and F2= 0.996683598 0.970923305

Diversities= 1.59187312E-03 1.39568131E-02

Contributions to loads within In and St= 8.43486050E-05 8.35654428E-05

Contributions to load between In and St = 5.40476285E-05

Contributions to homozygous loads for In and St= 8.44494207E-05 8.44494207E-05

Contributions to inbreeding loads for In and St= 1.00822561E-07 8.83980135E-07

Contributions to selection coefficients for In and St homokaryotypes

3.02791595E-05 2.95042992E-05

Contributions to mean A2 freqs= 2.63482183E-02 2.63482183E-02

Contributions to mean diversities= 6.99050288E-05 6.12895237E-04

Zone 2: quasi-neutral zone 2

Lower and upper bounds of St metapopn gamma for zone 2

0.250000000 499.999969

Probability of zone 2= 0.381453693

Coefficients for bivariate distribution of q1 and q2 in metapopulation

G1= 0.526315808 G2= 5.84795326E-02

G3= -5.26316166E-02 G4= 0.883040905

a1= 9.73684248E-03 a2= 8.76315832E-02

b11= -2.36842272E-04 b12= 8.09999928E-02 b22= 0.321868390

Contributions to mean load statistics for zone 2

Loads within In and St= 0.167113900 1.17866101E-03

Load between In and St= 9.25296079E-03

Homozygous load for In and St= 0.168093905 2.24092766E-03

Inbreeding loads= 9.79841221E-04 1.06226769E-03

Selection coefficients against In and St homokaryotypes

0.146031439 -8.10694695E-03

Contributions to mean A2 freqs= 0.119158335 1.84092205E-02

Contributions to mean diversities= 4.87794256E-04 1.72428554E-03

Zone 3: moderate selection zone

Lower and upper bounds of St metapopn gamma for zone 3

499.999969 5000.00000

Probability of zone 3= 0.357937425

Contributions to mean load statistics over zone 3

Loads within In and St= 1.25106948E-03 2.25631185E-04

Load between In and St= 2.32847349E-04

Homozygous load for In and St= 3.01792216E-03 1.63550384E-03

Inbreeding loads= 1.76684943E-03 1.40987278E-03

Selection coefficients for In and St homokaryotypes

1.01768970E-03 -7.27176666E-06

Contributions to mean A2 freqs= 4.01294237E-05 2.29812667E-05

Contributions to mean diversities= 5.04292875E-05 4.34464164E-05

Zone 4: strong selection zone

Upper limit to scaled gamma for St metapopn= 2.50000000

Lower limit to gamma for St metapopn= 5000.00000

Upper limit to gamma for St metapopn= 30000.0020

Probability of zone 4= 0.204838097

Contributions to mean load statistics over zone 4

Loads within In and St= 8.36628722E-04 2.07650068E-04

Load between In and St= 1.64129946E-04

Homozygous load for In and St= 1.94256951E-03 1.33910647E-03

Inbreeding loads= 1.10594195E-03 1.13145867E-03

Selection coefficients for In and St homokaryotypes

6.72280788E-04 4.35113907E-05

Contributions to mean A2 freqs= 7.71795294E-06 7.71795294E-06

Contributions to mean diversities= 3.45236408E-08 1.60526085E-07

Mean load statistics over all zones

Loads within In and St= 0.169285953 1.69550767E-03

Load between In and St= 9.70398542E-03

Homozygous load for In and St= 0.173138842 5.29998727E-03

Inbreeding loads= 3.85273341E-03 3.60448309E-03

Selection coefficients for In and St homokaryotypes

0.147499919 -8.04066658E-03

Mean frequencies of A2 in In and St= 0.145554408 4.47881408E-02

Ratio of these= 3.24984264

Mean diversities at selected sites in In and St= 6.08163129E-04 2.38078763E-03

Ratio of these= 0.255446196

Mean diversities at neutral sites in In and St= 1.59187312E-03 1.39568131E-02

pi-n/pi-s for In and St= 0.382042468 0.170582473

Ratio of these= 2.23963499

**Neutral Fst for whole population= 0.1**

Scaled migration rate for whole population= 9.00000000

Zone 1: quasi-neutral zone 1

Upper bound scaled selection coefficient for neutrality in St metapopulation= 0.250000000

Probability of zone 1= 4.39136960E-02

Integral of selection coefficient over zone 1= 1.40749034E-04

Mean load statistics for zone 1

Mean q1 and q2= 0.600000024

F1 and F2= 0.996689916 0.970978200

Diversities= 1.58884050E-03 1.39304632E-02

Contributions to loads within In and St= 8.43487869E-05 8.35671017E-05

Contributions to load between In and St = 5.40476285E-05

Contributions to homozygous loads for In and St= 8.44494207E-05 8.44494207E-05

Contributions to inbreeding loads for In and St= 1.00637997E-07 8.82319057E-07

Contributions to selection coefficients for In and St homokaryotypes

3.02791595E-05 2.95042992E-05

Contributions to mean A2 freqs= 2.63482183E-02 2.63482183E-02

Contributions to mean diversities= 6.97718569E-05 6.11738127E-04

Zone 2: quasi-neutral zone 2

Lower and upper bounds of St metapopn gamma for zone 2

0.250000000 499.999969

Probability of zone 2= 0.381453693

Coefficients for bivariate distribution of q1 and q2 in metapopulation

G1= 1.11111104 G2= 0.123456798

G3= -1.22222209 G4= 0.753086388

a1= 1.49999997E-02 a2= 0.134999990

b11= -5.49999997E-03 b12= 8.09999928E-02 b22= 0.274499953

Contributions to mean load statistics for zone 2

Loads within In and St= 0.177077636 1.18380075E-03

Load between In and St= 9.72177275E-03

Homozygous load for In and St= 0.177837268 1.96994678E-03

Inbreeding loads= 7.59380171E-04 7.86145392E-04

Selection coefficients against In and St homokaryotypes

0.154101491 -8.57448578E-03

Contributions to mean A2 freqs= 0.125280559 1.84892695E-02

Contributions to mean diversities= 4.33897105E-04 1.57646381E-03

Zone 3: moderate selection zone

Lower and upper bounds of St metapopn gamma for zone 3

499.999969 5000.00000

Probability of zone 3= 0.357937425

Contributions to mean load statistics over zone 3

Loads within In and St= 1.48328824E-03 2.42209251E-04

Load between In and St= 1.92076157E-04

Homozygous load for In and St= 2.73332628E-03 1.10603613E-03

Inbreeding loads= 1.25003851E-03 8.63826834E-04

Selection coefficients for In and St homokaryotypes

1.29038095E-03 5.01275063E-05

Contributions to mean A2 freqs= 3.65520864E-05 1.54742502E-05

Contributions to mean diversities= 3.54355980E-05 2.76176488E-05

Zone 4: strong selection zone

Upper limit to scaled gamma for St metapopn= 2.50000000

Lower limit to gamma for St metapopn= 5000.00000

Upper limit to gamma for St metapopn= 30000.0020

Probability of zone 4= 0.204838097

Contributions to mean load statistics over zone 4

Loads within In and St= 1.16811215E-03 2.94589117E-04

Load between In and St= 1.76093032E-04

Homozygous load for In and St= 2.01344560E-03 1.50735339E-03

Inbreeding loads= 8.45334318E-04 1.21276418E-03

Selection coefficients for In and St homokaryotypes

9.91523266E-04 1.18494034E-04

Contributions to mean A2 freqs= 7.93279469E-06 7.93279469E-06

Contributions to mean diversities= 4.06496135E-08 1.85625140E-07

Mean load statistics over all zones

Loads within In and St= 0.179813400 1.80416624E-03

Load between In and St= 1.01439888E-02

Homozygous load for In and St= 0.182668477 4.66778548E-03

Inbreeding loads= 2.85485364E-03 2.86361855E-03

Selection coefficients for In and St homokaryotypes

0.156056225 -8.37469101E-03

Mean frequencies of A2 in In and St= 0.151673257 4.48608957E-02

Ratio of these= 3.38096809

Mean diversities at selected sites in In and St= 5.39145200E-04 2.21600523E-03

Ratio of these= 0.243295997

Mean diversities at neutral sites in In and St= 1.58884050E-03 1.39304632E-02

pi-n/pi-s for In and St= 0.339332491 0.159076199

Ratio of these= 2.13314438

**Neutral Fst for whole population= 0.15**

Scaled migration rate for whole population= 5.66666651

Zone 1: quasi-neutral zone 1

Upper bound scaled selection coefficient for neutrality in St metapopulation= 0.250000000

Probability of zone 1= 4.39136960E-02

Integral of selection coefficient over zone 1= 1.40749034E-04

Mean load statistics for zone 1

Mean q1 and q2= 0.600000024

F1 and F2= 0.996697068 0.971039176

Diversities= 1.58540718E-03 1.39011955E-02

Contributions to loads within In and St= 8.43490125E-05 8.35689571E-05

Contributions to load between In and St = 5.40476285E-05

Contributions to homozygous loads for In and St= 8.44494207E-05 8.44494207E-05

Contributions to inbreeding loads for In and St= 1.00411484E-07 8.80465052E-07

Contributions to selection coefficients for In and St homokaryotypes

3.02791595E-05 2.95042992E-05

Contributions to mean A2 freqs= 2.63482183E-02 2.63482183E-02

Contributions to mean diversities= 6.96210918E-05 6.10452844E-04

Zone 2: quasi-neutral zone 2

Lower and upper bounds of St metapopn gamma for zone 2

0.250000000 499.999969

Probability of zone 2= 0.381453693

Coefficients for bivariate distribution of q1 and q2 in metapopulation

G1= 1.76470590 G2= 0.196078435

G3= -2.52941179 G4= 0.607843161

a1= 2.08823532E-02 a2= 0.187941164

b11= -1.13823544E-02 b12= 8.09999928E-02 b22= 0.221558809

Contributions to mean load statistics for zone 2

Loads within In and St= 0.186650276 1.19239476E-03

Load between In and St= 1.01836435E-02

Homozygous load for In and St= 0.187248528 1.79873640E-03

Inbreeding loads= 5.97869686E-04 6.06342393E-04

Selection coefficients against In and St homokaryotypes

0.161773264 -9.03177261E-03

Contributions to mean A2 freqs= 0.131416053 1.85964145E-02

Contributions to mean diversities= 3.87361972E-04 1.45363028E-03

Zone 3: moderate selection zone

Lower and upper bounds of St metapopn gamma for zone 3

499.999969 5000.00000

Probability of zone 3= 0.357937425

Contributions to mean load statistics over zone 3

Loads within In and St= 1.59222574E-03 2.35584099E-04

Load between In and St= 1.71219275E-04

Homozygous load for In and St= 2.57126126E-03 8.51592747E-04

Inbreeding loads= 9.79035161E-04 6.16008241E-04

Selection coefficients for In and St homokaryotypes

1.42002106E-03 6.43730164E-05

Contributions to mean A2 freqs= 3.43437714E-05 1.16926885E-05

Contributions to mean diversities= 2.72798061E-05 1.96842539E-05

Zone 4: strong selection zone

Upper limit to scaled gamma for St metapopn= 2.50000000

Lower limit to gamma for St metapopn= 5000.00000

Upper limit to gamma for St metapopn= 30000.0020

Probability of zone 4= 0.204838097

Contributions to mean load statistics over zone 4

Loads within In and St= 1.31073885E-03 3.44096654E-04

Load between In and St= 1.74824076E-04

Homozygous load for In and St= 1.98168471E-03 1.51373493E-03

Inbreeding loads= 6.70944864E-04 1.16963871E-03

Selection coefficients for In and St homokaryotypes

1.13528967E-03 1.69277191E-04

Contributions to mean A2 freqs= 7.81400286E-06 7.81400286E-06

Contributions to mean diversities= 4.33885816E-08 1.90287551E-07

Mean load statistics over all zones

Loads within In and St= 0.189637586 1.85564451E-03

Load between In and St= 1.05837341E-02

Homozygous load for In and St= 0.191885918 4.24851337E-03

Inbreeding loads= 2.24795006E-03 2.39286991E-03

Selection coefficients for In and St homokaryotypes

0.163939118 -8.76629353E-03

Mean frequencies of A2 in In and St= 0.157806426 4.49641421E-02

Ratio of these= 3.50960612

Mean diversities at selected sites in In and St= 4.84306249E-04 2.08395766E-03

Ratio of these= 0.232397363

Mean diversities at neutral sites in In and St= 1.58540718E-03 1.39011955E-02

pi-n/pi-s for In and St= 0.305477530 0.149912119

Ratio of these= 2.03771067

**Neutral Fst for whole population= 0.2**

Scaled migration rate for whole population= 4.00000000

Zone 1: quasi-neutral zone 1

Upper bound scaled selection coefficient for neutrality in St metapopulation= 0.250000000

Probability of zone 1= 4.39136960E-02

Integral of selection coefficient over zone 1= 1.40749034E-04

Mean load statistics for zone 1

Mean q1 and q2= 0.600000024

F1 and F2= 0.996705115 0.971107543

Diversities= 1.58154487E-03 1.38683794E-02

Contributions to loads within In and St= 8.43492453E-05 8.35710380E-05

Contributions to load between In and St = 5.40476285E-05

Contributions to homozygous loads for In and St= 8.44494207E-05 8.44494207E-05

Contributions to inbreeding loads for In and St= 1.00176585E-07 8.78384469E-07

Contributions to selection coefficients for In and St homokaryotypes

3.02791595E-05 2.95042992E-05

Contributions to mean A2 freqs= 2.63482183E-02 2.63482183E-02

Contributions to mean diversities= 6.94514820E-05 6.09011797E-04

Zone 2: quasi-neutral zone 2

Lower and upper bounds of St metapopn gamma for zone 2

0.250000000 499.999969

Probability of zone 2= 0.381453693

Coefficients for bivariate distribution of q1 and q2 in metapopulation

G1= 2.50000000 G2= 0.277777791

G3= -4.00000000 G4= 0.444444418

a1= 2.75000017E-02 a2= 0.247500002

b11= -1.80000011E-02 b12= 8.09999928E-02 b22= 0.161999986

Contributions to mean load statistics for zone 2

Loads within In and St= 0.195271745 1.20381138E-03

Load between In and St= 1.06064007E-02

Homozygous load for In and St= 0.195747972 1.68387580E-03

Inbreeding loads= 4.76310466E-04 4.80063347E-04

Selection coefficients against In and St homokaryotypes

0.168617547 -9.44697857E-03

Contributions to mean A2 freqs= 0.137271568 1.87299326E-02

Contributions to mean diversities= 3.46536428E-04 1.34653226E-03

Zone 3: moderate selection zone

Lower and upper bounds of St metapopn gamma for zone 3

499.999969 5000.00000

Probability of zone 3= 0.357937425

Contributions to mean load statistics over zone 3

Loads within In and St= 1.65347324E-03 2.35431493E-04

Load between In and St= 1.58816707E-04

Homozygous load for In and St= 2.46685348E-03 7.08293112E-04

Inbreeding loads= 8.13381281E-04 4.72861604E-04

Selection coefficients for In and St homokaryotypes

1.49351358E-03 7.65919685E-05

Contributions to mean A2 freqs= 3.28706265E-05 9.46451928E-06

Contributions to mean diversities= 2.22242761E-05 1.50137348E-05

Zone 4: strong selection zone

Upper limit to scaled gamma for St metapopn= 2.50000000

Lower limit to gamma for St metapopn= 5000.00000

Upper limit to gamma for St metapopn= 30000.0020

Probability of zone 4= 0.204838097

Contributions to mean load statistics over zone 4

Loads within In and St= 1.39089359E-03 3.72779876E-04

Load between In and St= 1.70619693E-04

Homozygous load for In and St= 1.94142840E-03 1.46993704E-03

Inbreeding loads= 5.50535449E-04 1.09715585E-03

Selection coefficients for In and St homokaryotypes

1.21951103E-03 2.02119350E-04

Contributions to mean A2 freqs= 7.66421090E-06 7.66421090E-06

Contributions to mean diversities= 4.48886937E-08 1.87712018E-07

Mean load statistics over all zones

Loads within In and St= 0.198400468 1.89559383E-03

Load between In and St= 1.09898839E-02

Homozygous load for In and St= 0.200240701 3.94655531E-03

Inbreeding loads= 1.84032740E-03 2.05095904E-03

Selection coefficients for In and St homokaryotypes

0.170896769 -9.13572311E-03

Mean frequencies of A2 in In and St= 0.163660318 4.50952798E-02

Ratio of these= 3.62921166

Mean diversities at selected sites in In and St= 4.38257062E-04 1.97074562E-03

Ratio of these= 0.222381338

Mean diversities at neutral sites in In and St= 1.58154487E-03 1.38683794E-02

pi-n/pi-s for In and St= 0.277106941 0.142103523

Ratio of these= 1.95003569

**Neutral Fst for whole population= 0.25**

Scaled migration rate for whole population= 3.00000000

Zone 1: quasi-neutral zone 1

Upper bound scaled selection coefficient for neutrality in St metapopulation= 0.250000000

Probability of zone 1= 4.39136960E-02

Integral of selection coefficient over zone 1= 1.40749034E-04

Mean load statistics for zone 1

Mean q1 and q2= 0.600000024

F1 and F2= 0.996714115 0.971184611

Diversities= 1.57722470E-03 1.38313863E-02

Contributions to loads within In and St= 8.43495218E-05 8.35733881E-05

Contributions to load between In and St = 5.40476285E-05

Contributions to homozygous loads for In and St= 8.44494207E-05 8.44494207E-05

Contributions to inbreeding loads for In and St= 9.98997365E-08 8.76035472E-07

Contributions to selection coefficients for In and St homokaryotypes

3.02791595E-05 2.95042992E-05

Contributions to mean A2 freqs= 2.63482183E-02 2.63482183E-02

Contributions to mean diversities= 6.92617687E-05 6.07387279E-04

Zone 2: quasi-neutral zone 2

Lower and upper bounds of St metapopn gamma for zone 2

0.250000000 499.999969

Probability of zone 2= 0.381453693

Coefficients for bivariate distribution of q1 and q2 in metapopulation

G1= 3.33333325 G2= 0.370370388

G3= -5.66666651 G4= 0.259259224

a1= 3.50000001E-02 a2= 0.314999998

b11= -2.55000014E-02 b12= 8.09999928E-02 b22= 9.44999754E-02

Contributions to mean load statistics for zone 2

Loads within In and St= 0.202555060 1.21800590E-03

Load between In and St= 1.09686889E-02

Homozygous load for In and St= 0.202937886 1.60435180E-03

Inbreeding loads= 3.82627681E-04 3.86346743E-04

Selection coefficients against In and St homokaryotypes

0.174351692 -9.79840755E-03

Contributions to mean A2 freqs= 0.142628983 1.88920237E-02

Contributions to mean diversities= 3.10465228E-04 1.24984211E-03

Zone 3: moderate selection zone

Lower and upper bounds of St metapopn gamma for zone 3

499.999969 5000.00000

Probability of zone 3= 0.357937425

Contributions to mean load statistics over zone 3

Loads within In and St= 1.69177249E-03 2.42055510E-04

Load between In and St= 1.50833293E-04

Homozygous load for In and St= 2.39372160E-03 6.21966261E-04

Inbreeding loads= 7.01949175E-04 3.79911013E-04

Selection coefficients for In and St homokaryotypes

1.53976679E-03 9.11951065E-05

Contributions to mean A2 freqs= 3.18164748E-05 8.04588944E-06

Contributions to mean diversities= 1.88028116E-05 1.20110371E-05

Zone 4: strong selection zone

Upper limit to scaled gamma for St metapopn= 2.50000000

Lower limit to gamma for St metapopn= 5000.00000

Upper limit to gamma for St metapopn= 30000.0020

Probability of zone 4= 0.204838097

Contributions to mean load statistics over zone 4

Loads within In and St= 1.46938907E-03 3.88604152E-04

Load between In and St= 1.67074599E-04

Homozygous load for In and St= 1.93255709E-03 1.40795030E-03

Inbreeding loads= 4.63165110E-04 1.01934513E-03

Selection coefficients for In and St homokaryotypes

1.30146742E-03 2.21490860E-04

Contributions to mean A2 freqs= 7.62595073E-06 7.62595073E-06

Contributions to mean diversities= 4.63843861E-08 1.82002353E-07

Mean load statistics over all zones

Loads within In and St= 0.205800578 1.93223893E-03

Load between In and St= 1.13406442E-02

Homozygous load for In and St= 0.207348615 3.71871796E-03

Inbreeding loads= 1.54784182E-03 1.78647891E-03

Selection coefficients for In and St homokaryotypes

0.176720858 -9.45281982E-03

Mean frequencies of A2 in In and St= 0.169016644 4.52559143E-02

Ratio of these= 3.73468637

Mean diversities at selected sites in In and St= 3.98576201E-04 1.86942238E-03

Ratio of these= 0.213208213

Mean diversities at neutral sites in In and St= 1.57722470E-03 1.38313863E-02

pi-n/pi-s for In and St= 0.252707303 0.135157987

Ratio of these= 1.86971784

**h = 0.25**

**Neutral Fst for whole population= 0.05**

Scaled migration rate for whole population= 19.0000000

Zone 1: quasi-neutral zone 1

Upper bound scaled selection coefficient for neutrality in St metapopulation= 0.250000000

Probability of zone 1= 4.39136960E-02

Integral of selection coefficient over zone 1= 1.40749034E-04

Mean load statistics for zone 1

Mean q1 and q2= 0.600000024

F1 and F2= 0.996683598 0.970923305

Diversities= 1.59187312E-03 1.39568131E-02

Contributions to loads within In and St= 8.43934104E-05 8.39583226E-05

Contributions to load between In and St = 6.75595365E-05

Contributions to homozygous loads for In and St= 8.44494207E-05 8.44494207E-05

Contributions to inbreeding loads for In and St= 5.60153310E-08 4.91100991E-07

Contributions to selection coefficients for In and St homokaryotypes

1.68085098E-05 1.63912773E-05

Contributions to mean A2 freqs= 2.63482183E-02 2.63482183E-02

Contributions to mean diversities= 6.99050288E-05 6.12895237E-04

Zone 2: quasi-neutral zone 2

Lower and upper bounds of St metapopn gamma for zone 2

0.250000000 499.999969

Probability of zone 2= 0.381453693

Coefficients for bivariate distribution of q1 and q2 in metapopulation

G1= 0.526315808 G2= 5.84795326E-02

G3= -5.26316166E-02 G4= 0.883040905

a1= 2.76315790E-02 a2= 0.248684227

b11= -1.31579058E-04 b12= 4.49999981E-02 b22= 0.178815767

Contributions to mean load statistics for zone 2

Loads within In and St= 3.92145999E-02 1.32182054E-03

Load between In and St= 1.06022442E-02

Homozygous load for In and St= 3.94564606E-02 1.59780041E-03

Inbreeding loads= 2.41846137E-04 2.75980303E-04

Selection coefficients against In and St homokaryotypes

2.82068849E-02 -9.32359695E-03

Contributions to mean A2 freqs= 7.56535158E-02 1.87253710E-02

Contributions to mean diversities= 3.63345258E-04 1.39361247E-03

Zone 3: moderate selection zone

Lower and upper bounds of St metapopn gamma for zone 3

499.999969 5000.00000

Probability of zone 3= 0.357937425

Contributions to mean load statistics over zone 3

Loads within In and St= 6.36093202E-04 2.15594642E-04

Load between In and St= 3.43870284E-04

Homozygous load for In and St= 9.57540062E-04 4.17906180E-04

Inbreeding loads= 3.21447238E-04 2.02311261E-04

Selection coefficients for In and St homokaryotypes

2.92181969E-04 -1.28269196E-04

Contributions to mean A2 freqs= 1.29234286E-05 6.25436269E-06

Contributions to mean diversities= 1.70558651E-05 1.18357857E-05

Zone 4: strong selection zone

Upper limit to scaled gamma for St metapopn= 2.50000000

Lower limit to gamma for St metapopn= 5000.00000

Upper limit to gamma for St metapopn= 30000.0020

Probability of zone 4= 0.204838097

Contributions to mean load statistics over zone 4

Loads within In and St= 2.71703844E-04 1.45760205E-04

Load between In and St= 1.70025451E-04

Homozygous load for In and St= 4.03855840E-04 2.76241568E-04

Inbreeding loads= 1.32151981E-04 1.30481363E-04

Selection coefficients for In and St homokaryotypes

1.01685524E-04 -2.43186951E-05

Contributions to mean A2 freqs= 1.58084833E-06 1.58084833E-06

Contributions to mean diversities= 7.08092252E-09 3.19999280E-08

Mean load statistics over all zones

Loads within In and St= 4.02067900E-02 1.76713371E-03

Load between In and St= 1.11836987E-02

Homozygous load for In and St= 4.09023054E-02 2.37639761E-03

Inbreeding loads= 6.95501396E-04 6.09264011E-04

Selection coefficients for In and St homokaryotypes

2.86059380E-02 -9.46104527E-03

Mean frequencies of A2 in In and St= 0.102016240 4.50814255E-02

Ratio of these= 2.26293278

Mean diversities at selected sites in In and St= 4.50313208E-04 2.01837532E-03

Ratio of these= 0.223106772

Mean diversities at neutral sites in In and St= 1.59187312E-03 1.39568131E-02

pi-n/pi-s for In and St= 0.282882601 0.144615769

Ratio of these= 1.95609784

**Neutral Fst for whole population= 0.10**

Scaled migration rate for whole population= 9.00000000

Zone 1: quasi-neutral zone 1

Upper bound scaled selection coefficient for neutrality in St metapopulation= 0.250000000

Probability of zone 1= 4.39136960E-02

Integral of selection coefficient over zone 1= 1.40749034E-04

Mean load statistics for zone 1

Mean q1 and q2= 0.600000024

F1 and F2= 0.996689916 0.970978200

Diversities= 1.58884050E-03 1.39304632E-02

Contributions to loads within In and St= 8.43935195E-05 8.39592467E-05

Contributions to load between In and St = 6.75595365E-05

Contributions to homozygous loads for In and St= 8.44494207E-05 8.44494207E-05

Contributions to inbreeding loads for In and St= 5.59062698E-08 4.90178195E-07

Contributions to selection coefficients for In and St homokaryotypes

1.68085098E-05 1.63912773E-05

Contributions to mean A2 freqs= 2.63482183E-02 2.63482183E-02

Contributions to mean diversities= 6.97718569E-05 6.11738127E-04

Zone 2: quasi-neutral zone 2

Lower and upper bounds of St metapopn gamma for zone 2

0.250000000 499.999969

Probability of zone 2= 0.381453693

Coefficients for bivariate distribution of q1 and q2 in metapopulation

G1= 1.11111104 G2= 0.123456798

G3= -1.22222209 G4= 0.753086388

a1= 3.05555556E-02 a2= 0.274999976

b11= -3.05555551E-03 b12= 4.49999981E-02 b22= 0.152499989

Contributions to mean load statistics for zone 2

Loads within In and St= 3.96462269E-02 1.33606966E-03

Load between In and St= 1.07087828E-02

Homozygous load for In and St= 3.98729779E-02 1.58903177E-03

Inbreeding loads= 2.26727352E-04 2.52962578E-04

Selection coefficients against In and St homokaryotypes

2.85227895E-02 -9.41681862E-03

Contributions to mean A2 freqs= 7.61549100E-02 1.88160613E-02

Contributions to mean diversities= 3.53869633E-04 1.35059550E-03

Zone 3: moderate selection zone

Lower and upper bounds of St metapopn gamma for zone 3

499.999969 5000.00000

Probability of zone 3= 0.357937425

Contributions to mean load statistics over zone 3

Loads within In and St= 8.39151035E-04 2.24255797E-04

Load between In and St= 3.82730592E-04

Homozygous load for In and St= 1.13375019E-03 3.97133292E-04

Inbreeding loads= 2.94599071E-04 1.72877350E-04

Selection coefficients for In and St homokaryotypes

4.56333160E-04 -1.58429146E-04

Contributions to mean A2 freqs= 1.56103506E-05 5.83391375E-06

Contributions to mean diversities= 1.56606584E-05 1.04302089E-05

Zone 4: strong selection zone

Upper limit to scaled gamma for St metapopn= 2.50000000

Lower limit to gamma for St metapopn= 5000.00000

Upper limit to gamma for St metapopn= 30000.0020

Probability of zone 4= 0.204838097

Contributions to mean load statistics over zone 4

Loads within In and St= 3.38965765E-04 1.74543529E-04

Load between In and St= 1.92058797E-04

Homozygous load for In and St= 4.49145125E-04 3.19083716E-04

Inbreeding loads= 1.10179921E-04 1.44540289E-04

Selection coefficients for In and St homokaryotypes

1.46925449E-04 -1.75237656E-05

Contributions to mean A2 freqs= 1.73604292E-06 1.73604292E-06

Contributions to mean diversities= 8.89062424E-09 3.82686558E-08

Mean load statistics over all zones

Loads within In and St= 4.09087352E-02 1.81882828E-03

Load between In and St= 1.13511328E-02

Homozygous load for In and St= 4.15403210E-02 2.38969829E-03

Inbreeding loads= 6.31562260E-04 5.70870354E-04

Selection coefficients for In and St homokaryotypes

2.91250348E-02 -9.57787037E-03

Mean frequencies of A2 in In and St= 0.102520473 4.51718494E-02

Ratio of these= 2.26956558

Mean diversities at selected sites in In and St= 4.39311028E-04 1.97280198E-03

Ratio of these= 0.222683787

Mean diversities at neutral sites in In and St= 1.58884050E-03 1.39304632E-02

pi-n/pi-s for In and St= 0.276497871 0.141617835

Ratio of these= 1.95242262

**Neutral Fst for whole population= 0.15**

Scaled migration rate for whole population= 5.66666651

Zone 1: quasi-neutral zone 1

Upper bound scaled selection coefficient for neutrality in St metapopulation= 0.250000000

Probability of zone 1= 4.39136960E-02

Integral of selection coefficient over zone 1= 1.40749034E-04

Mean load statistics for zone 1

Mean q1 and q2= 0.600000024

F1 and F2= 0.996697068 0.971039176

Diversities= 1.58540718E-03 1.39011955E-02

Contributions to loads within In and St= 8.43936359E-05 8.39602799E-05

Contributions to load between In and St = 6.75595365E-05

Contributions to homozygous loads for In and St= 8.44494207E-05 8.44494207E-05

Contributions to inbreeding loads for In and St= 5.57888207E-08 4.89146316E-07

Contributions to selection coefficients for In and St homokaryotypes

1.68085098E-05 1.63912773E-05

Contributions to mean A2 freqs= 2.63482183E-02 2.63482183E-02

Contributions to mean diversities= 6.96210918E-05 6.10452844E-04

Zone 2: quasi-neutral zone 2

Lower and upper bounds of St metapopn gamma for zone 2

0.250000000 499.999969

Probability of zone 2= 0.381453693

Coefficients for bivariate distribution of q1 and q2 in metapopulation

G1= 1.76470590 G2= 0.196078435

G3= -2.52941179 G4= 0.607843161

a1= 3.38235311E-02 a2= 0.304411739

b11= -6.32352987E-03 b12= 4.49999981E-02 b22= 0.123088233

Contributions to mean load statistics for zone 2

Loads within In and St= 4.00849991E-02 1.35047932E-03

Load between In and St= 1.08179897E-02

Homozygous load for In and St= 4.02968265E-02 1.58152345E-03

Inbreeding loads= 2.11816339E-04 2.31044949E-04

Selection coefficients against In and St homokaryotypes

2.88428664E-02 -9.51242447E-03

Contributions to mean A2 freqs= 7.67029971E-02 1.89171340E-02

Contributions to mean diversities= 3.44003114E-04 1.30655384E-03

Zone 3: moderate selection zone

Lower and upper bounds of St metapopn gamma for zone 3

499.999969 5000.00000

Probability of zone 3= 0.357937425

Contributions to mean load statistics over zone 3

Loads within In and St= 1.00043730E-03 2.29985497E-04

Load between In and St= 4.15191724E-04

Homozygous load for In and St= 1.27408735E-03 3.86636821E-04

Inbreeding loads= 2.73649028E-04 1.56651353E-04

Selection coefficients for In and St homokaryotypes

5.85079193E-04 -1.85251236E-04

Contributions to mean A2 freqs= 1.78236460E-05 5.54821463E-06

Contributions to mean diversities= 1.45011572E-05 9.36519427E-06

Zone 4: strong selection zone

Upper limit to scaled gamma for St metapopn= 2.50000000

Lower limit to gamma for St metapopn= 5000.00000

Upper limit to gamma for St metapopn= 30000.0020

Probability of zone 4= 0.204838097

Contributions to mean load statistics over zone 4

Loads within In and St= 3.86411353E-04 1.89179031E-04

Load between In and St= 2.04715703E-04

Homozygous load for In and St= 4.82328789E-04 3.36528057E-04

Inbreeding loads= 9.59172612E-05 1.47348823E-04

Selection coefficients for In and St homokaryotypes

1.81674957E-04 -1.54972076E-05

Contributions to mean A2 freqs= 1.84431201E-06 1.84431201E-06

Contributions to mean diversities= 1.02019149E-08 4.11331769E-08

Mean load statistics over all zones

Loads within In and St= 4.15562429E-02 1.85360410E-03

Load between In and St= 1.15054576E-02

Homozygous load for In and St= 4.21376899E-02 2.38913787E-03

Inbreeding loads= 5.81438420E-04 5.35534229E-04

Selection coefficients for In and St homokaryotypes

2.96037197E-02 -9.69862938E-03

Mean frequencies of A2 in In and St= 0.103070885 4.52727452E-02

Ratio of these= 2.27666521

Mean diversities at selected sites in In and St= 4.28135594E-04 1.92641292E-03

Ratio of these= 0.222244978

Mean diversities at neutral sites in In and St= 1.58540718E-03 1.39011955E-02

pi-n/pi-s for In and St= 0.270047724 0.138578936

Ratio of these= 1.94869244

**Neutral Fst for whole population= 0.2**

Scaled migration rate for whole population= 4.00000000

Zone 1: quasi-neutral zone 1

Upper bound scaled selection coefficient for neutrality in St metapopulation= 0.250000000

Probability of zone 1= 4.39136960E-02

Integral of selection coefficient over zone 1= 1.40749034E-04

Mean load statistics for zone 1

Mean q1 and q2= 0.600000024

F1 and F2= 0.996705115 0.971107543

Diversities= 1.58154487E-03 1.38683794E-02

Contributions to loads within In and St= 8.43937669E-05 8.39614368E-05

Contributions to load between In and St = 6.75595365E-05

Contributions to homozygous loads for In and St= 8.44494207E-05 8.44494207E-05

Contributions to inbreeding loads for In and St= 5.56545920E-08 4.87988586E-07

Contributions to selection coefficients for In and St homokaryotypes

1.68085098E-05 1.63912773E-05

Contributions to mean A2 freqs= 2.63482183E-02 2.63482183E-02

Contributions to mean diversities= 6.94514820E-05 6.09011797E-04

Zone 2: quasi-neutral zone 2

Lower and upper bounds of St metapopn gamma for zone 2

0.250000000 499.999969

Probability of zone 2= 0.381453693

Coefficients for bivariate distribution of q1 and q2 in metapopulation

G1= 2.50000000 G2= 0.277777791

G3= -4.00000000 G4= 0.444444418

a1= 3.75000015E-02 a2= 0.337499976

b11= -1.00000007E-02 b12= 4.49999981E-02 b22= 8.99999887E-02

Contributions to mean load statistics for zone 2

Loads within In and St= 4.05328982E-02 1.36520166E-03

Load between In and St= 1.09305298E-02

Homozygous load for In and St= 4.07300293E-02 1.57533761E-03

Inbreeding loads= 1.97111294E-04 2.10135520E-04

Selection coefficients against In and St homokaryotypes

2.91684866E-02 -9.61124897E-03

Contributions to mean A2 freqs= 7.73049071E-02 1.90305207E-02

Contributions to mean diversities= 3.33703851E-04 1.26130483E-03

Zone 3: moderate selection zone

Lower and upper bounds of St metapopn gamma for zone 3

499.999969 5000.00000

Probability of zone 3= 0.357937425

Contributions to mean load statistics over zone 3

Loads within In and St= 1.13192608E-03 2.39852758E-04

Load between In and St= 4.42651944E-04

Homozygous load for In and St= 1.38849672E-03 3.82068334E-04

Inbreeding loads= 2.56569590E-04 1.42215373E-04

Selection coefficients for In and St homokaryotypes

6.89029694E-04 -2.02775002E-04

Contributions to mean A2 freqs= 1.96750025E-05 5.33854336E-06

Contributions to mean diversities= 1.35149276E-05 8.49792741E-06

Zone 4: strong selection zone

Upper limit to scaled gamma for St metapopn= 2.50000000

Lower limit to gamma for St metapopn= 5000.00000

Upper limit to gamma for St metapopn= 30000.0020

Probability of zone 4= 0.204838097

Contributions to mean load statistics over zone 4

Loads within In and St= 4.28711268E-04 1.98915179E-04

Load between In and St= 2.15258784E-04

Homozygous load for In and St= 5.14897401E-04 3.46130575E-04

Inbreeding loads= 8.61860681E-05 1.47215338E-04

Selection coefficients for In and St homokaryotypes

2.13444233E-04 -1.63316727E-05

Contributions to mean A2 freqs= 1.95216217E-06 1.95216217E-06

Contributions to mean diversities= 1.13530625E-08 4.28472902E-08

Mean load statistics over all zones

Loads within In and St= 4.21779267E-02 1.88793102E-03

Load between In and St= 1.16559993E-02

Homozygous load for In and St= 4.27178741E-02 2.38798605E-03

Inbreeding loads= 5.39922621E-04 5.00054215E-04

Selection coefficients for In and St homokaryotypes

3.00608277E-02 -9.81593132E-03

Mean frequencies of A2 in In and St= 0.103674755 4.53860275E-02

Ratio of these= 2.28428793

Mean diversities at selected sites in In and St= 4.16681607E-04 1.87885750E-03

Ratio of these= 0.221773922

Mean diversities at neutral sites in In and St= 1.58154487E-03 1.38683794E-02

pi-n/pi-s for In and St= 0.263464928 0.135477796

Ratio of these= 1.94470930

**Neutral Fst for whole population= 0.25**

Scaled migration rate for whole population= 3.00000000

Zone 1: quasi-neutral zone 1

Upper bound scaled selection coefficient for neutrality in St metapopulation= 0.250000000

Probability of zone 1= 4.39136960E-02

Integral of selection coefficient over zone 1= 1.40749034E-04

Mean load statistics for zone 1

Mean q1 and q2= 0.600000024

F1 and F2= 0.996714115 0.971184611

Diversities= 1.57722470E-03 1.38313863E-02

Contributions to loads within In and St= 8.43939197E-05 8.39627392E-05

Contributions to load between In and St = 6.75595365E-05

Contributions to homozygous loads for In and St= 8.44494207E-05 8.44494207E-05

Contributions to inbreeding loads for In and St= 5.55035840E-08 4.86688236E-07

Contributions to selection coefficients for In and St homokaryotypes

1.68085098E-05 1.63912773E-05

Contributions to mean A2 freqs= 2.63482183E-02 2.63482183E-02

Contributions to mean diversities= 6.92617687E-05 6.07387279E-04

Zone 2: quasi-neutral zone 2

Lower and upper bounds of St metapopn gamma for zone 2

0.250000000 499.999969

Probability of zone 2= 0.381453693

Coefficients for bivariate distribution of q1 and q2 in metapopulation

G1= 3.33333325 G2= 0.370370388

G3= -5.66666651 G4= 0.259259224

a1= 4.16666679E-02 a2= 0.374999970

b11= -1.41666671E-02 b12= 4.49999981E-02 b22= 5.24999909E-02

Contributions to mean load statistics for zone 2

Loads within In and St= 4.09923382E-02 1.38044718E-03

Load between In and St= 1.10471286E-02

Homozygous load for In and St= 4.11749408E-02 1.57060288E-03

Inbreeding loads= 1.82606847E-04 1.90156061E-04

Selection coefficients against In and St homokaryotypes

2.95013189E-02 -9.71353054E-03

Contributions to mean A2 freqs= 7.79692382E-02 1.91588011E-02

Contributions to mean diversities= 3.22922657E-04 1.21465244E-03

Zone 3: moderate selection zone

Lower and upper bounds of St metapopn gamma for zone 3

499.999969 5000.00000

Probability of zone 3= 0.357937425

Contributions to mean load statistics over zone 3

Loads within In and St= 1.24119071E-03 2.51796766E-04

Load between In and St= 4.66461206E-04

Homozygous load for In and St= 1.48346368E-03 3.82335595E-04

Inbreeding loads= 2.42271853E-04 1.30538785E-04

Selection coefficients for In and St homokaryotypes

7.74443150E-04 -2.14695930E-04

Contributions to mean A2 freqs= 2.12417945E-05 5.19158857E-06

Contributions to mean diversities= 1.26626719E-05 7.78051890E-06

Zone 4: strong selection zone

Upper limit to scaled gamma for St metapopn= 2.50000000

Lower limit to gamma for St metapopn= 5000.00000

Upper limit to gamma for St metapopn= 30000.0020

Probability of zone 4= 0.204838097

Contributions to mean load statistics over zone 4

Loads within In and St= 4.76570771E-04 2.06388839E-04

Load between In and St= 2.27063676E-04

Homozygous load for In and St= 5.55735431E-04 3.52511852E-04

Inbreeding loads= 7.91646380E-05 1.46123159E-04

Selection coefficients for In and St homokaryotypes

2.49505043E-04 -2.06232071E-05

Contributions to mean A2 freqs= 2.09259724E-06 2.09259724E-06

Contributions to mean diversities= 1.26036346E-08 4.40511769E-08

Mean load statistics over all zones

Loads within In and St= 4.27944921E-02 1.92259555E-03

Load between In and St= 1.18082138E-02

Homozygous load for In and St= 4.32985872E-02 2.38989969E-03

Inbreeding loads= 5.04098833E-04 4.67304722E-04

Selection coefficients for In and St homokaryotypes

3.05111408E-02 -9.93466377E-03

Mean frequencies of A2 in In and St= 0.104340792 4.55143079E-02

Ratio of these= 2.29248333

Mean diversities at selected sites in In and St= 4.04859689E-04 1.82986422E-03

Ratio of these= 0.221251220

Mean diversities at neutral sites in In and St= 1.57722470E-03 1.38313863E-02

pi-n/pi-s for In and St= 0.256691188 0.132297963

Ratio of these= 1.94025052

**h= 0.45**

**Neutral Fst for whole population= 0.05**

Scaled migration rate for whole population= 19.0000000

Zone 1: quasi-neutral zone 1

Upper bound scaled selection coefficient for neutrality in St metapopulation= 0.250000000

Probability of zone 1= 4.39136960E-02

Integral of selection coefficient over zone 1= 1.40749034E-04

Mean load statistics for zone 1

Mean q1 and q2= 0.600000024

F1 and F2= 0.996683598 0.970923305

Diversities= 1.59187312E-03 1.39568131E-02

Contributions to loads within In and St= 8.43934104E-05 8.39583226E-05

Contributions to load between In and St = 6.75595365E-05

Contributions to homozygous loads for In and St= 8.44494207E-05 8.44494207E-05

Contributions to inbreeding loads for In and St= 5.60153310E-08 4.91100991E-07

Contributions to selection coefficients for In and St homokaryotypes

1.68085098E-05 1.63912773E-05

Contributions to mean A2 freqs= 2.63482183E-02 2.63482183E-02

Contributions to mean diversities= 6.99050288E-05 6.12895237E-04

Zone 2: quasi-neutral zone 2

Lower and upper bounds of St metapopn gamma for zone 2

0.250000000 499.999969

Probability of zone 2= 0.381453693

Coefficients for bivariate distribution of q1 and q2 in metapopulation

G1= 0.526315808 G2= 5.84795326E-02

G3= -5.26316166E-02 G4= 0.883040905

a1= 2.76315790E-02 a2= 0.248684227

b11= -1.31579058E-04 b12= 4.49999981E-02 b22= 0.178815767

Contributions to mean load statistics for zone 2

Loads within In and St= 3.92145999E-02 1.32182054E-03

Load between In and St= 1.06022442E-02

Homozygous load for In and St= 3.94564606E-02 1.59780041E-03

Inbreeding loads= 2.41846137E-04 2.75980303E-04

Selection coefficients against In and St homokaryotypes

2.82068849E-02 -9.32359695E-03

Contributions to mean A2 freqs= 7.56535158E-02 1.87253710E-02

Contributions to mean diversities= 3.63345258E-04 1.39361247E-03

Zone 3: moderate selection zone

Lower and upper bounds of St metapopn gamma for zone 3

499.999969 5000.00000

Probability of zone 3= 0.357937425

Contributions to mean load statistics over zone 3

Loads within In and St= 6.36093202E-04 2.15594642E-04

Load between In and St= 3.43870284E-04

Homozygous load for In and St= 9.57540062E-04 4.17906180E-04

Inbreeding loads= 3.21447238E-04 2.02311261E-04

Selection coefficients for In and St homokaryotypes

2.92181969E-04 -1.28269196E-04

Contributions to mean A2 freqs= 1.29234286E-05 6.25436269E-06

Contributions to mean diversities= 1.70558651E-05 1.18357857E-05

Zone 4: strong selection zone

Upper limit to scaled gamma for St metapopn= 2.50000000

Lower limit to gamma for St metapopn= 5000.00000

Upper limit to gamma for St metapopn= 30000.0020

Probability of zone 4= 0.204838097

Contributions to mean load statistics over zone 4

Loads within In and St= 2.71703844E-04 1.45760205E-04

Load between In and St= 1.70025451E-04

Homozygous load for In and St= 4.03855840E-04 2.76241568E-04

Inbreeding loads= 1.32151981E-04 1.30481363E-04

Selection coefficients for In and St homokaryotypes

1.01685524E-04 -2.43186951E-05

Contributions to mean A2 freqs= 1.58084833E-06 1.58084833E-06

Contributions to mean diversities= 7.08092252E-09 3.19999280E-08

Mean load statistics over all zones

Loads within In and St= 4.02067900E-02 1.76713371E-03

Load between In and St= 1.11836987E-02

Homozygous load for In and St= 4.09023054E-02 2.37639761E-03

Inbreeding loads= 6.95501396E-04 6.09264011E-04

Selection coefficients for In and St homokaryotypes

2.86059380E-02 -9.46104527E-03

Mean frequencies of A2 in In and St= 0.102016240 4.50814255E-02

Ratio of these= 2.26293278

Mean diversities at selected sites in In and St= 4.50313208E-04 2.01837532E-03

Ratio of these= 0.223106772

Mean diversities at neutral sites in In and St= 1.59187312E-03 1.39568131E-02

pi-n/pi-s for In and St= 0.282882601 0.144615769

Ratio of these= 1.95609784

**Neutral Fst for whole population= 0.1**

Scaled migration rate for whole population= 9.00000000

Zone 1: quasi-neutral zone 1

Upper bound scaled selection coefficient for neutrality in St metapopulation= 0.250000000

Probability of zone 1= 4.39136960E-02

Integral of selection coefficient over zone 1= 1.40749034E-04

Mean load statistics for zone 1

Mean q1 and q2= 0.600000024

F1 and F2= 0.996689916 0.970978200

Diversities= 1.58884050E-03 1.39304632E-02

Contributions to loads within In and St= 8.43935195E-05 8.39592467E-05

Contributions to load between In and St = 6.75595365E-05

Contributions to homozygous loads for In and St= 8.44494207E-05 8.44494207E-05

Contributions to inbreeding loads for In and St= 5.59062698E-08 4.90178195E-07

Contributions to selection coefficients for In and St homokaryotypes

1.68085098E-05 1.63912773E-05

Contributions to mean A2 freqs= 2.63482183E-02 2.63482183E-02

Contributions to mean diversities= 6.97718569E-05 6.11738127E-04

Zone 2: quasi-neutral zone 2

Lower and upper bounds of St metapopn gamma for zone 2

0.250000000 499.999969

Probability of zone 2= 0.381453693

Coefficients for bivariate distribution of q1 and q2 in metapopulation

G1= 1.11111104 G2= 0.123456798

G3= -1.22222209 G4= 0.753086388

a1= 3.05555556E-02 a2= 0.274999976

b11= -3.05555551E-03 b12= 4.49999981E-02 b22= 0.152499989

Contributions to mean load statistics for zone 2

Loads within In and St= 3.96462269E-02 1.33606966E-03

Load between In and St= 1.07087828E-02

Homozygous load for In and St= 3.98729779E-02 1.58903177E-03

Inbreeding loads= 2.26727352E-04 2.52962578E-04

Selection coefficients against In and St homokaryotypes

2.85227895E-02 -9.41681862E-03

Contributions to mean A2 freqs= 7.61549100E-02 1.88160613E-02

Contributions to mean diversities= 3.53869633E-04 1.35059550E-03

Zone 3: moderate selection zone

Lower and upper bounds of St metapopn gamma for zone 3

499.999969 5000.00000

Probability of zone 3= 0.357937425

Contributions to mean load statistics over zone 3

Loads within In and St= 8.39151035E-04 2.24255797E-04

Load between In and St= 3.82730592E-04

Homozygous load for In and St= 1.13375019E-03 3.97133292E-04

Inbreeding loads= 2.94599071E-04 1.72877350E-04

Selection coefficients for In and St homokaryotypes

4.56333160E-04 -1.58429146E-04

Contributions to mean A2 freqs= 1.56103506E-05 5.83391375E-06

Contributions to mean diversities= 1.56606584E-05 1.04302089E-05

Zone 4: strong selection zone

Upper limit to scaled gamma for St metapopn= 2.50000000

Lower limit to gamma for St metapopn= 5000.00000

Upper limit to gamma for St metapopn= 30000.0020

Probability of zone 4= 0.204838097

Contributions to mean load statistics over zone 4

Loads within In and St= 3.38965765E-04 1.74543529E-04

Load between In and St= 1.92058797E-04

Homozygous load for In and St= 4.49145125E-04 3.19083716E-04

Inbreeding loads= 1.10179921E-04 1.44540289E-04

Selection coefficients for In and St homokaryotypes

1.46925449E-04 -1.75237656E-05

Contributions to mean A2 freqs= 1.73604292E-06 1.73604292E-06

Contributions to mean diversities= 8.89062424E-09 3.82686558E-08

Mean load statistics over all zones

Loads within In and St= 4.09087352E-02 1.81882828E-03

Load between In and St= 1.13511328E-02

Homozygous load for In and St= 4.15403210E-02 2.38969829E-03

Inbreeding loads= 6.31562260E-04 5.70870354E-04

Selection coefficients for In and St homokaryotypes

2.91250348E-02 -9.57787037E-03

Mean frequencies of A2 in In and St= 0.102520473 4.51718494E-02

Ratio of these= 2.26956558

Mean diversities at selected sites in In and St= 4.39311028E-04 1.97280198E-03

Ratio of these= 0.222683787

Mean diversities at neutral sites in In and St= 1.58884050E-03 1.39304632E-02

pi-n/pi-s for In and St= 0.276497871 0.141617835

Ratio of these= 1.95242262

**Neutral Fst for whole population= 0.15**

Scaled migration rate for whole population= 5.66666651

Zone 1: quasi-neutral zone 1

Upper bound scaled selection coefficient for neutrality in St metapopulation= 0.250000000

Probability of zone 1= 4.39136960E-02

Integral of selection coefficient over zone 1= 1.40749034E-04

Mean load statistics for zone 1

Mean q1 and q2= 0.600000024

F1 and F2= 0.996697068 0.971039176

Diversities= 1.58540718E-03 1.39011955E-02

Contributions to loads within In and St= 8.43936359E-05 8.39602799E-05

Contributions to load between In and St = 6.75595365E-05

Contributions to homozygous loads for In and St= 8.44494207E-05 8.44494207E-05

Contributions to inbreeding loads for In and St= 5.57888207E-08 4.89146316E-07

Contributions to selection coefficients for In and St homokaryotypes

1.68085098E-05 1.63912773E-05

Contributions to mean A2 freqs= 2.63482183E-02 2.63482183E-02

Contributions to mean diversities= 6.96210918E-05 6.10452844E-04

Zone 2: quasi-neutral zone 2

Lower and upper bounds of St metapopn gamma for zone 2

0.250000000 499.999969

Probability of zone 2= 0.381453693

Coefficients for bivariate distribution of q1 and q2 in metapopulation

G1= 1.76470590 G2= 0.196078435

G3= -2.52941179 G4= 0.607843161

a1= 3.38235311E-02 a2= 0.304411739

b11= -6.32352987E-03 b12= 4.49999981E-02 b22= 0.123088233

Contributions to mean load statistics for zone 2

Loads within In and St= 4.00849991E-02 1.35047932E-03

Load between In and St= 1.08179897E-02

Homozygous load for In and St= 4.02968265E-02 1.58152345E-03

Inbreeding loads= 2.11816339E-04 2.31044949E-04

Selection coefficients against In and St homokaryotypes

2.88428664E-02 -9.51242447E-03

Contributions to mean A2 freqs= 7.67029971E-02 1.89171340E-02

Contributions to mean diversities= 3.44003114E-04 1.30655384E-03

Zone 3: moderate selection zone

Lower and upper bounds of St metapopn gamma for zone 3

499.999969 5000.00000

Probability of zone 3= 0.357937425

Contributions to mean load statistics over zone 3

Loads within In and St= 1.00043730E-03 2.29985497E-04

Load between In and St= 4.15191724E-04

Homozygous load for In and St= 1.27408735E-03 3.86636821E-04

Inbreeding loads= 2.73649028E-04 1.56651353E-04

Selection coefficients for In and St homokaryotypes

5.85079193E-04 -1.85251236E-04

Contributions to mean A2 freqs= 1.78236460E-05 5.54821463E-06

Contributions to mean diversities= 1.45011572E-05 9.36519427E-06

Zone 4: strong selection zone

Upper limit to scaled gamma for St metapopn= 2.50000000

Lower limit to gamma for St metapopn= 5000.00000

Upper limit to gamma for St metapopn= 30000.0020

Probability of zone 4= 0.204838097

Contributions to mean load statistics over zone 4

Loads within In and St= 3.86411353E-04 1.89179031E-04

Load between In and St= 2.04715703E-04

Homozygous load for In and St= 4.82328789E-04 3.36528057E-04

Inbreeding loads= 9.59172612E-05 1.47348823E-04

Selection coefficients for In and St homokaryotypes

1.81674957E-04 -1.54972076E-05

Contributions to mean A2 freqs= 1.84431201E-06 1.84431201E-06

Contributions to mean diversities= 1.02019149E-08 4.11331769E-08

Mean load statistics over all zones

Loads within In and St= 4.15562429E-02 1.85360410E-03

Load between In and St= 1.15054576E-02

Homozygous load for In and St= 4.21376899E-02 2.38913787E-03

Inbreeding loads= 5.81438420E-04 5.35534229E-04

Selection coefficients for In and St homokaryotypes

2.96037197E-02 -9.69862938E-03

Mean frequencies of A2 in In and St= 0.103070885 4.52727452E-02

Ratio of these= 2.27666521

Mean diversities at selected sites in In and St= 4.28135594E-04 1.92641292E-03

Ratio of these= 0.222244978

Mean diversities at neutral sites in In and St= 1.58540718E-03 1.39011955E-02

pi-n/pi-s for In and St= 0.270047724 0.138578936

Ratio of these= 1.94869244

**Neutral Fst for whole population= 0.2**

Scaled migration rate for whole population= 4.00000000

Zone 1: quasi-neutral zone 1

Upper bound scaled selection coefficient for neutrality in St metapopulation= 0.250000000

Probability of zone 1= 4.39136960E-02

Integral of selection coefficient over zone 1= 1.40749034E-04

Mean load statistics for zone 1

Mean q1 and q2= 0.600000024

F1 and F2= 0.996705115 0.971107543

Diversities= 1.58154487E-03 1.38683794E-02

Contributions to loads within In and St= 8.43937669E-05 8.39614368E-05

Contributions to load between In and St = 6.75595365E-05

Contributions to homozygous loads for In and St= 8.44494207E-05 8.44494207E-05

Contributions to inbreeding loads for In and St= 5.56545920E-08 4.87988586E-07

Contributions to selection coefficients for In and St homokaryotypes

1.68085098E-05 1.63912773E-05

Contributions to mean A2 freqs= 2.63482183E-02 2.63482183E-02

Contributions to mean diversities= 6.94514820E-05 6.09011797E-04

Zone 2: quasi-neutral zone 2

Lower and upper bounds of St metapopn gamma for zone 2

0.250000000 499.999969

Probability of zone 2= 0.381453693

Coefficients for bivariate distribution of q1 and q2 in metapopulation

G1= 2.50000000 G2= 0.277777791

G3= -4.00000000 G4= 0.444444418

a1= 3.75000015E-02 a2= 0.337499976

b11= -1.00000007E-02 b12= 4.49999981E-02 b22= 8.99999887E-02

Contributions to mean load statistics for zone 2

Loads within In and St= 4.05328982E-02 1.36520166E-03

Load between In and St= 1.09305298E-02

Homozygous load for In and St= 4.07300293E-02 1.57533761E-03

Inbreeding loads= 1.97111294E-04 2.10135520E-04

Selection coefficients against In and St homokaryotypes

2.91684866E-02 -9.61124897E-03

Contributions to mean A2 freqs= 7.73049071E-02 1.90305207E-02

Contributions to mean diversities= 3.33703851E-04 1.26130483E-03

Zone 3: moderate selection zone

Lower and upper bounds of St metapopn gamma for zone 3

499.999969 5000.00000

Probability of zone 3= 0.357937425

Contributions to mean load statistics over zone 3

Loads within In and St= 1.13192608E-03 2.39852758E-04

Load between In and St= 4.42651944E-04

Homozygous load for In and St= 1.38849672E-03 3.82068334E-04

Inbreeding loads= 2.56569590E-04 1.42215373E-04

Selection coefficients for In and St homokaryotypes

6.89029694E-04 -2.02775002E-04

Contributions to mean A2 freqs= 1.96750025E-05 5.33854336E-06

Contributions to mean diversities= 1.35149276E-05 8.49792741E-06

Zone 4: strong selection zone

Upper limit to scaled gamma for St metapopn= 2.50000000

Lower limit to gamma for St metapopn= 5000.00000

Upper limit to gamma for St metapopn= 30000.0020

Probability of zone 4= 0.204838097

Contributions to mean load statistics over zone 4

Loads within In and St= 4.28711268E-04 1.98915179E-04

Load between In and St= 2.15258784E-04

Homozygous load for In and St= 5.14897401E-04 3.46130575E-04

Inbreeding loads= 8.61860681E-05 1.47215338E-04

Selection coefficients for In and St homokaryotypes

2.13444233E-04 -1.63316727E-05

Contributions to mean A2 freqs= 1.95216217E-06 1.95216217E-06

Contributions to mean diversities= 1.13530625E-08 4.28472902E-08

Mean load statistics over all zones

Loads within In and St= 4.21779267E-02 1.88793102E-03

Load between In and St= 1.16559993E-02

Homozygous load for In and St= 4.27178741E-02 2.38798605E-03

Inbreeding loads= 5.39922621E-04 5.00054215E-04

Selection coefficients for In and St homokaryotypes

3.00608277E-02 -9.81593132E-03

Mean frequencies of A2 in In and St= 0.103674755 4.53860275E-02

Ratio of these= 2.28428793

Mean diversities at selected sites in In and St= 4.16681607E-04 1.87885750E-03

Ratio of these= 0.221773922

Mean diversities at neutral sites in In and St= 1.58154487E-03 1.38683794E-02

pi-n/pi-s for In and St= 0.263464928 0.135477796

Ratio of these= 1.94470930

**Neutral Fst for whole population= 0.25**

Scaled migration rate for whole population= 3.00000000

Zone 1: quasi-neutral zone 1

Upper bound scaled selection coefficient for neutrality in St metapopulation= 0.250000000

Probability of zone 1= 4.39136960E-02

Integral of selection coefficient over zone 1= 1.40749034E-04

Mean load statistics for zone 1

Mean q1 and q2= 0.600000024

F1 and F2= 0.996714115 0.971184611

Diversities= 1.57722470E-03 1.38313863E-02

Contributions to loads within In and St= 8.43939197E-05 8.39627392E-05

Contributions to load between In and St = 6.75595365E-05

Contributions to homozygous loads for In and St= 8.44494207E-05 8.44494207E-05

Contributions to inbreeding loads for In and St= 5.55035840E-08 4.86688236E-07

Contributions to selection coefficients for In and St homokaryotypes

1.68085098E-05 1.63912773E-05

Contributions to mean A2 freqs= 2.63482183E-02 2.63482183E-02

Contributions to mean diversities= 6.92617687E-05 6.07387279E-04

Zone 2: quasi-neutral zone 2

Lower and upper bounds of St metapopn gamma for zone 2

0.250000000 499.999969

Probability of zone 2= 0.381453693

Coefficients for bivariate distribution of q1 and q2 in metapopulation

G1= 3.33333325 G2= 0.370370388

G3= -5.66666651 G4= 0.259259224

a1= 4.16666679E-02 a2= 0.374999970

b11= -1.41666671E-02 b12= 4.49999981E-02 b22= 5.24999909E-02

Contributions to mean load statistics for zone 2

Loads within In and St= 4.09923382E-02 1.38044718E-03

Load between In and St= 1.10471286E-02

Homozygous load for In and St= 4.11749408E-02 1.57060288E-03

Inbreeding loads= 1.82606847E-04 1.90156061E-04

Selection coefficients against In and St homokaryotypes

2.95013189E-02 -9.71353054E-03

Contributions to mean A2 freqs= 7.79692382E-02 1.91588011E-02

Contributions to mean diversities= 3.22922657E-04 1.21465244E-03

Zone 3: moderate selection zone

Lower and upper bounds of St metapopn gamma for zone 3

499.999969 5000.00000

Probability of zone 3= 0.357937425

Contributions to mean load statistics over zone 3

Loads within In and St= 1.24119071E-03 2.51796766E-04

Load between In and St= 4.66461206E-04

Homozygous load for In and St= 1.48346368E-03 3.82335595E-04

Inbreeding loads= 2.42271853E-04 1.30538785E-04

Selection coefficients for In and St homokaryotypes

7.74443150E-04 -2.14695930E-04

Contributions to mean A2 freqs= 2.12417945E-05 5.19158857E-06

Contributions to mean diversities= 1.26626719E-05 7.78051890E-06

Zone 4: strong selection zone

Upper limit to scaled gamma for St metapopn= 2.50000000

Lower limit to gamma for St metapopn= 5000.00000

Upper limit to gamma for St metapopn= 30000.0020

Probability of zone 4= 0.204838097

Contributions to mean load statistics over zone 4

Loads within In and St= 4.76570771E-04 2.06388839E-04

Load between In and St= 2.27063676E-04

Homozygous load for In and St= 5.55735431E-04 3.52511852E-04

Inbreeding loads= 7.91646380E-05 1.46123159E-04

Selection coefficients for In and St homokaryotypes

2.49505043E-04 -2.06232071E-05

Contributions to mean A2 freqs= 2.09259724E-06 2.09259724E-06

Contributions to mean diversities= 1.26036346E-08 4.40511769E-08

Mean load statistics over all zones

Loads within In and St= 4.27944921E-02 1.92259555E-03

Load between In and St= 1.18082138E-02

Homozygous load for In and St= 4.32985872E-02 2.38989969E-03

Inbreeding loads= 5.04098833E-04 4.67304722E-04

Selection coefficients for In and St homokaryotypes

3.05111408E-02 -9.93466377E-03

Mean frequencies of A2 in In and St= 0.104340792 4.55143079E-02

Ratio of these= 2.29248333

Mean diversities at selected sites in In and St= 4.04859689E-04 1.82986422E-03

Ratio of these= 0.221251220

Mean diversities at neutral sites in In and St= 1.57722470E-03 1.38313863E-02

pi-n/pi-s for In and St= 0.256691188 0.132297963

Ratio of these= 1.94025052

**Inversion frequency= 0.5**

**h= 0.05**

**Neutral Fst for whole population= 0.05**

Scaled migration rate for whole population= 19.0000000

Zone 1: quasi-neutral zone 1

Upper bound scaled selection coefficient for neutrality in St metapopulation= 0.250000000

Probability of zone 1= 5.23817725E-02

Integral of selection coefficient over zone 1= 3.02202563E-04

Mean load statistics for zone 1

Mean q1 and q2= 0.600000024

F1 and F2= 0.983634770 0.983634770

Diversities= 7.85531010E-03 7.85531010E-03

Contributions to loads within In and St= 1.80253308E-04 1.80253308E-04

Contributions to load between In and St = 1.16045783E-04

Contributions to homozygous loads for In and St= 1.81321549E-04 1.81321549E-04

Contributions to inbreeding loads for In and St= 1.06824177E-06 1.06824177E-06

Contributions to selection coefficients for In and St homokaryotypes

6.41942024E-05 6.41942024E-05

Contributions to mean A2 freqs= 3.14290635E-02 3.14290635E-02

Contributions to mean diversities= 4.11475077E-04 4.11475077E-04

Zone 2: quasi-neutral zone 2

Lower and upper bounds of St metapopn gamma for zone 2

0.250000000 499.999969

Probability of zone 2= 0.451199740

Coefficients for bivariate distribution of q1 and q2 in metapopulation

G1= 0.105263159 G2= 0.105263159

G3= 0.789473653 G4= 0.789473653

a1= 4.86842096E-02 a2= 4.86842096E-02

b11= 8.88157859E-02 b12= 0.224999994 b22= 8.88157859E-02

Contributions to mean load statistics for zone 2

Loads within In and St= 4.66967328E-03 4.66946838E-03

Load between In and St= 1.51571294E-03

Homozygous load for In and St= 6.17142767E-03 6.17122138E-03

Inbreeding loads= 1.50176417E-03 1.50176277E-03

Selection coefficients against In and St homokaryotypes

3.14897299E-03 3.14879417E-03

Contributions to mean A2 freqs= 2.79802531E-02 2.79796068E-02

Contributions to mean diversities= 1.35763770E-03 1.35763024E-03

Zone 3: moderate selection zone

Lower and upper bounds of St metapopn gamma for zone 3

499.999969 999.999939

Probability of zone 3= 0.106151342

Contributions to mean load statistics over zone 3

Loads within In and St= 1.17501688E-04 1.17471034E-04

Load between In and St= 6.33220552E-05

Homozygous load for In and St= 6.32707844E-04 6.32755924E-04

Inbreeding loads= 5.15205844E-04 5.15284541E-04

Selection coefficients for In and St homokaryotypes

5.41806221E-05 5.41210175E-05

Contributions to mean A2 freqs= 9.08828133E-06 9.08924176E-06

Contributions to mean diversities= 1.64441990E-05 1.64460052E-05

Zone 4: strong selection zone

Upper limit to scaled gamma for St metapopn= 2.50000000

Lower limit to gamma for St metapopn= 999.999939

Upper limit to gamma for St metapopn= 16666.6680

Probability of zone 4= 0.378410041

Contributions to mean load statistics over zone 4

Loads within In and St= 5.77986008E-04 5.77986008E-04

Load between In and St= 2.96649319E-04

Homozygous load for In and St= 2.96470686E-03 2.96470686E-03

Inbreeding loads= 2.38672295E-03 2.38672295E-03

Selection coefficients for In and St homokaryotypes

2.81274319E-04 2.81274319E-04

Contributions to mean A2 freqs= 2.39509627E-05 2.39509627E-05

Contributions to mean diversities= 4.24839442E-07 4.24839499E-07

Mean load statistics over all zones

Loads within In and St= 5.54541405E-03 5.54517843E-03

Load between In and St= 1.99173018E-03

Homozygous load for In and St= 9.95016377E-03 9.95000545E-03

Inbreeding loads= 4.40476090E-03 4.40483820E-03

Selection coefficients for In and St homokaryotypes

3.54737043E-03 3.54713202E-03

Mean frequencies of A2 in In and St= 5.94423562E-02 5.94417118E-02

Ratio of these= 1.00001085

Mean diversities at selected sites in In and St= 1.78598182E-03 1.78597611E-03

Ratio of these= 1.00000322

Mean diversities at neutral sites in In and St= 7.85531010E-03 7.85531010E-03

pi-n/pi-s for In and St= 0.227359816 0.227359086

Ratio of these= 1.00000322

**Neutral Fst for whole population= 0.1**

Scaled migration rate for whole population= 9.00000000

Zone 1: quasi-neutral zone 1

Upper bound scaled selection coefficient for neutrality in St metapopulation= 0.250000000

Probability of zone 1= 5.23817725E-02

Integral of selection coefficient over zone 1= 3.02202563E-04

Mean load statistics for zone 1

Mean q1 and q2= 0.600000024

F1 and F2= 0.983666062 0.983666062

Diversities= 7.84028973E-03 7.84028973E-03

Contributions to loads within In and St= 1.80255345E-04 1.80255345E-04

Contributions to load between In and St = 1.16045783E-04

Contributions to homozygous loads for In and St= 1.81321549E-04 1.81321549E-04

Contributions to inbreeding loads for In and St= 1.06620632E-06 1.06620632E-06

Contributions to selection coefficients for In and St homokaryotypes

6.41942024E-05 6.41942024E-05

Contributions to mean A2 freqs= 3.14290635E-02 3.14290635E-02

Contributions to mean diversities= 4.10688284E-04 4.10688284E-04

Zone 2: quasi-neutral zone 2

Lower and upper bounds of St metapopn gamma for zone 2

0.250000000 499.999969

Probability of zone 2= 0.451199740

Coefficients for bivariate distribution of q1 and q2 in metapopulation

G1= 0.222222224 G2= 0.222222224

G3= 0.555555582 G4= 0.555555582

a1= 7.50000030E-02 a2= 7.50000030E-02

b11= 6.25000000E-02 b12= 0.224999994 b22= 6.25000000E-02

Contributions to mean load statistics for zone 2

Loads within In and St= 5.16767660E-03 5.16718859E-03

Load between In and St= 1.64024008E-03

Homozygous load for In and St= 6.26221858E-03 6.26172824E-03

Inbreeding loads= 1.09453988E-03 1.09453697E-03

Selection coefficients against In and St homokaryotypes

3.52120399E-03 3.52072716E-03

Contributions to mean A2 freqs= 2.92707924E-02 2.92694289E-02

Contributions to mean diversities= 1.22663262E-03 1.22661586E-03

Zone 3: moderate selection zone

Lower and upper bounds of St metapopn gamma for zone 3

499.999969 999.999939

Probability of zone 3= 0.106151342

Contributions to mean load statistics over zone 3

Loads within In and St= 1.18453514E-04 1.18458142E-04

Load between In and St= 4.53351422E-05

Homozygous load for In and St= 4.53088869E-04 4.53112909E-04

Inbreeding loads= 3.34635261E-04 3.34654731E-04

Selection coefficients for In and St homokaryotypes

7.31348991E-05 7.31348991E-05

Contributions to mean A2 freqs= 6.50434595E-06 6.50482752E-06

Contributions to mean diversities= 1.06736325E-05 1.06743310E-05

Zone 4: strong selection zone

Upper limit to scaled gamma for St metapopn= 2.50000000

Lower limit to gamma for St metapopn= 999.999939

Upper limit to gamma for St metapopn= 16666.6680

Probability of zone 4= 0.378410041

Contributions to mean load statistics over zone 4

Loads within In and St= 8.55388993E-04 8.55388818E-04

Load between In and St= 3.21420957E-04

Homozygous load for In and St= 3.21218348E-03 3.21218348E-03

Inbreeding loads= 2.35679280E-03 2.35679280E-03

Selection coefficients for In and St homokaryotypes

5.33819199E-04 5.33819199E-04

Contributions to mean A2 freqs= 2.59227072E-05 2.59227072E-05

Contributions to mean diversities= 4.87118882E-07 4.87118825E-07

Mean load statistics over all zones

Loads within In and St= 6.32177433E-03 6.32129051E-03

Load between In and St= 2.12304201E-03

Homozygous load for In and St= 1.01088127E-02 1.01083461E-02

Inbreeding loads= 3.78703419E-03 3.78705072E-03

Selection coefficients for In and St homokaryotypes

4.18990850E-03 4.18943167E-03

Mean frequencies of A2 in In and St= 6.07322864E-02 6.07309230E-02

Ratio of these= 1.00002241

Mean diversities at selected sites in In and St= 1.64848170E-03 1.64846564E-03

Ratio of these= 1.00000978

Mean diversities at neutral sites in In and St= 7.84028973E-03 7.84028973E-03

pi-n/pi-s for In and St= 0.210257754 0.210255697

Ratio of these= 1.00000978

**Neutral Fst for whole population= 0.15**

Scaled migration rate for whole population= 5.66666651

Zone 1: quasi-neutral zone 1

Upper bound scaled selection coefficient for neutrality in St metapopulation= 0.250000000

Probability of zone 1= 5.23817725E-02

Integral of selection coefficient over zone 1= 3.02202563E-04

Mean load statistics for zone 1

Mean q1 and q2= 0.600000024

F1 and F2= 0.983700812 0.983700812

Diversities= 7.82360975E-03 7.82360975E-03

Contributions to loads within In and St= 1.80257615E-04 1.80257615E-04

Contributions to load between In and St = 1.16045783E-04

Contributions to homozygous loads for In and St= 1.81321549E-04 1.81321549E-04

Contributions to inbreeding loads for In and St= 1.06393679E-06 1.06393679E-06

Contributions to selection coefficients for In and St homokaryotypes

6.41942024E-05 6.41942024E-05

Contributions to mean A2 freqs= 3.14290635E-02 3.14290635E-02

Contributions to mean diversities= 4.09814558E-04 4.09814558E-04

Zone 2: quasi-neutral zone 2

Lower and upper bounds of St metapopn gamma for zone 2

0.250000000 499.999969

Probability of zone 2= 0.451199740

Coefficients for bivariate distribution of q1 and q2 in metapopulation

G1= 0.352941185 G2= 0.352941185

G3= 0.294117630 G4= 0.294117630

a1= 0.104411766 a2= 0.104411766

b11= 3.30882333E-02 b12= 0.224999994 b22= 3.30882333E-02

Contributions to mean load statistics for zone 2

Loads within In and St= 5.79320965E-03 5.79176890E-03

Load between In and St= 1.83448906E-03

Homozygous load for In and St= 6.62999833E-03 6.62855012E-03

Inbreeding loads= 8.36793042E-04 8.36785825E-04

Selection coefficients against In and St homokaryotypes

3.95089388E-03 3.94946337E-03

Contributions to mean A2 freqs= 3.08524966E-02 3.08489893E-02

Contributions to mean diversities= 1.12017104E-03 1.12013880E-03

Zone 3: moderate selection zone

Lower and upper bounds of St metapopn gamma for zone 3

499.999969 999.999939

Probability of zone 3= 0.106151342

Contributions to mean load statistics over zone 3

Loads within In and St= 1.18963631E-04 1.18971831E-04

Load between In and St= 3.60952072E-05

Homozygous load for In and St= 3.60786216E-04 3.60801350E-04

Inbreeding loads= 2.41822636E-04 2.41829533E-04

Selection coefficients for In and St homokaryotypes

8.28504562E-05 8.28504562E-05

Contributions to mean A2 freqs= 5.17351828E-06 5.17382068E-06

Contributions to mean diversities= 7.70348743E-06 7.70372662E-06

Zone 4: strong selection zone

Upper limit to scaled gamma for St metapopn= 2.50000000

Lower limit to gamma for St metapopn= 999.999939

Upper limit to gamma for St metapopn= 16666.6680

Probability of zone 4= 0.378410041

Contributions to mean load statistics over zone 4

Loads within In and St= 1.06205873E-03 1.06205873E-03

Load between In and St= 3.25840316E-04

Homozygous load for In and St= 3.25633329E-03 3.25633329E-03

Inbreeding loads= 2.19427538E-03 2.19427538E-03

Selection coefficients for In and St homokaryotypes

7.35938549E-04 7.35938549E-04

Contributions to mean A2 freqs= 2.64393893E-05 2.64393893E-05

Contributions to mean diversities= 5.19701928E-07 5.19701928E-07

Mean load statistics over all zones

Loads within In and St= 7.15448940E-03 7.15305703E-03

Load between In and St= 2.31247023E-03

Homozygous load for In and St= 1.04284389E-02 1.04270065E-02

Inbreeding loads= 3.27395508E-03 3.27395461E-03

Selection coefficients for In and St homokaryotypes

4.83030081E-03 4.82887030E-03

Mean frequencies of A2 in In and St= 6.23131730E-02 6.23096675E-02

Ratio of these= 1.00005627

Mean diversities at selected sites in In and St= 1.53820869E-03 1.53817679E-03

Ratio of these= 1.00002074

Mean diversities at neutral sites in In and St= 7.82360975E-03 7.82360975E-03

pi-n/pi-s for In and St= 0.196611121 0.196607038

Ratio of these= 1.00002074

**Neutral Fst for whole population= 0.2**

Scaled migration rate for whole population= 4.00000000

Zone 1: quasi-neutral zone 1

Upper bound scaled selection coefficient for neutrality in St metapopulation= 0.250000000

Probability of zone 1= 5.23817725E-02

Integral of selection coefficient over zone 1= 3.02202563E-04

Mean load statistics for zone 1

Mean q1 and q2= 0.600000024

F1 and F2= 0.983739853 0.983739853

Diversities= 7.80487061E-03 7.80487061E-03

Contributions to loads within In and St= 1.80260147E-04 1.80260147E-04

Contributions to load between In and St = 1.16045783E-04

Contributions to homozygous loads for In and St= 1.81321549E-04 1.81321549E-04

Contributions to inbreeding loads for In and St= 1.06139692E-06 1.06139692E-06

Contributions to selection coefficients for In and St homokaryotypes

6.41942024E-05 6.41942024E-05

Contributions to mean A2 freqs= 3.14290635E-02 3.14290635E-02

Contributions to mean diversities= 4.08832944E-04 4.08832944E-04

Zone 2: quasi-neutral zone 2

Lower and upper bounds of St metapopn gamma for zone 2

0.250000000 499.999969

Probability of zone 2= 0.451199740

Coefficients for bivariate distribution of q1 and q2 in metapopulation

G1= 0.500000000 G2= 0.500000000

G3= 0.00000000 G4= 0.00000000

a1= 0.137500003 a2= 0.137500003

b11= 0.00000000 b12= 0.224999994 b22= 0.00000000

Contributions to mean load statistics for zone 2

Loads within In and St= 6.59416802E-03 6.58825273E-03

Load between In and St= 2.11894442E-03

Homozygous load for In and St= 7.25259213E-03 7.24664889E-03

Inbreeding loads= 6.58430799E-04 6.58403151E-04

Selection coefficients against In and St homokaryotypes

4.46522236E-03 4.45932150E-03

Contributions to mean A2 freqs= 3.28013040E-02 3.27893533E-02

Contributions to mean diversities= 1.02804962E-03 1.02793705E-03

Zone 3: moderate selection zone

Lower and upper bounds of St metapopn gamma for zone 3

499.999969 999.999939

Probability of zone 3= 0.106151342

Contributions to mean load statistics over zone 3

Loads within In and St= 1.19712065E-04 1.19731092E-04

Load between In and St= 3.05573776E-05

Homozygous load for In and St= 3.05454741E-04 3.05466470E-04

Inbreeding loads= 1.85742683E-04 1.85735364E-04

Selection coefficients for In and St homokaryotypes

8.91685486E-05 8.91685486E-05

Contributions to mean A2 freqs= 4.37412837E-06 4.37436256E-06

Contributions to mean diversities= 5.90776108E-06 5.90777154E-06

Zone 4: strong selection zone

Upper limit to scaled gamma for St metapopn= 2.50000000

Lower limit to gamma for St metapopn= 999.999939

Upper limit to gamma for St metapopn= 16666.6680

Probability of zone 4= 0.378410041

Contributions to mean load statistics over zone 4

Loads within In and St= 1.22722739E-03 1.22722739E-03

Load between In and St= 3.24378634E-04

Homozygous load for In and St= 3.24173737E-03 3.24173737E-03

Inbreeding loads= 2.01451080E-03 2.01451080E-03

Selection coefficients for In and St homokaryotypes

9.02414322E-04 9.02414322E-04

Contributions to mean A2 freqs= 2.65102080E-05 2.65102080E-05

Contributions to mean diversities= 5.40557664E-07 5.40557664E-07

Mean load statistics over all zones

Loads within In and St= 8.12136754E-03 8.11547134E-03

Load between In and St= 2.58992636E-03

Homozygous load for In and St= 1.09811053E-02 1.09751746E-02

Inbreeding loads= 2.85974564E-03 2.85971072E-03

Selection coefficients for In and St homokaryotypes

5.51617146E-03 5.51033020E-03

Mean frequencies of A2 in In and St= 6.42612502E-02 6.42492995E-02

Ratio of these= 1.00018597

Mean diversities at selected sites in In and St= 1.44333078E-03 1.44321821E-03

Ratio of these= 1.00007796

Mean diversities at neutral sites in In and St= 7.80487061E-03 7.80487061E-03

pi-n/pi-s for In and St= 0.184926927 0.184912503

Ratio of these= 1.00007796

**Neutral Fst for whole population= 0.25**

Scaled migration rate for whole population= 3.00000000

Zone 1: quasi-neutral zone 1

Upper bound scaled selection coefficient for neutrality in St metapopulation= 0.250000000

Probability of zone 1= 5.23817725E-02

Integral of selection coefficient over zone 1= 3.02202563E-04

Mean load statistics for zone 1

Mean q1 and q2= 0.600000024

F1 and F2= 0.983783841 0.983783841

Diversities= 7.78375613E-03 7.78375613E-03

Contributions to loads within In and St= 1.80263029E-04 1.80263029E-04

Contributions to load between In and St = 1.16045783E-04

Contributions to homozygous loads for In and St= 1.81321549E-04 1.81321549E-04

Contributions to inbreeding loads for In and St= 1.05851495E-06 1.05851495E-06

Contributions to selection coefficients for In and St homokaryotypes

6.41942024E-05 6.41942024E-05

Contributions to mean A2 freqs= 3.14290635E-02 3.14290635E-02

Contributions to mean diversities= 4.07726940E-04 4.07726940E-04

Zone 2: quasi-neutral zone 2

Lower and upper bounds of St metapopn gamma for zone 2

0.250000000 499.999969

Probability of zone 2= 0.451199740

Coefficients for bivariate distribution of q1 and q2 in metapopulation

G1= 0.666666687 G2= 0.666666687

G3= -0.333333373 G4= -0.333333373

a1= 0.175000012 a2= 0.175000012

b11= -3.75000052E-02 b12= 0.224999994 b22= -3.75000052E-02

Contributions to mean load statistics for zone 2

Loads within In and St= 7.63473287E-03 7.59641128E-03

Load between In and St= 2.53266538E-03

Homozygous load for In and St= 8.16162955E-03 8.12316593E-03

Inbreeding loads= 5.26893476E-04 5.26751101E-04

Selection coefficients against In and St homokaryotypes

5.08904457E-03 5.05095720E-03

Contributions to mean A2 freqs= 3.52255888E-02 3.51633914E-02

Contributions to mean diversities= 9.44292580E-04 9.43816733E-04

Zone 3: moderate selection zone

Lower and upper bounds of St metapopn gamma for zone 3

499.999969 999.999939

Probability of zone 3= 0.106151342

Contributions to mean load statistics over zone 3

Loads within In and St= 1.20993958E-04 1.21013152E-04

Load between In and St= 2.69547236E-05

Homozygous load for In and St= 2.69454089E-04 2.69465119E-04

Inbreeding loads= 1.48460051E-04 1.48451887E-04

Selection coefficients for In and St homokaryotypes

9.40561295E-05 9.40561295E-05

Contributions to mean A2 freqs= 3.85245630E-06 3.85267640E-06

Contributions to mean diversities= 4.71330168E-06 4.71318390E-06

Zone 4: strong selection zone

Upper limit to scaled gamma for St metapopn= 2.50000000

Lower limit to gamma for St metapopn= 999.999939

Upper limit to gamma for St metapopn= 16666.6680

Probability of zone 4= 0.378410041

Contributions to mean load statistics over zone 4

Loads within In and St= 1.36483600E-03 1.36483600E-03

Load between In and St= 3.20843945E-04

Homozygous load for In and St= 3.20644188E-03 3.20644188E-03

Inbreeding loads= 1.84160797E-03 1.84160797E-03

Selection coefficients for In and St homokaryotypes

1.04343891E-03 1.04343891E-03

Contributions to mean A2 freqs= 2.64027240E-05 2.64027240E-05

Contributions to mean diversities= 5.55156248E-07 5.55156191E-07

Mean load statistics over all zones

Loads within In and St= 9.30082612E-03 9.26252268E-03

Load between In and St= 2.99650989E-03

Homozygous load for In and St= 1.18188476E-02 1.17803952E-02

Inbreeding loads= 2.51802010E-03 2.51786946E-03

Selection coefficients for In and St homokaryotypes

6.28447533E-03 6.24644756E-03

Mean frequencies of A2 in In and St= 6.66849092E-02 6.66227117E-02

Ratio of these= 1.00093353

Mean diversities at selected sites in In and St= 1.35728798E-03 1.35681208E-03

Ratio of these= 1.00035071

Mean diversities at neutral sites in In and St= 7.78375613E-03 7.78375613E-03

pi-n/pi-s for In and St= 0.174374416 0.174313277

Ratio of these= 1.00035071

**h= 0.25**

**Neutral Fst for whole population= 0.05**

Scaled migration rate for whole population= 19.0000000

Zone 1: quasi-neutral zone 1

Upper bound scaled selection coefficient for neutrality in St metapopulation= 0.250000000

Probability of zone 1= 5.23817725E-02

Integral of selection coefficient over zone 1= 3.02202563E-04

Mean load statistics for zone 1

Mean q1 and q2= 0.600000024

F1 and F2= 0.983634770 0.983634770

Diversities= 7.85531010E-03 7.85531010E-03

Contributions to loads within In and St= 1.80728079E-04 1.80728079E-04

Contributions to load between In and St = 1.45057231E-04

Contributions to homozygous loads for In and St= 1.81321549E-04 1.81321549E-04

Contributions to inbreeding loads for In and St= 5.93463653E-07 5.93463653E-07

Contributions to selection coefficients for In and St homokaryotypes

3.56435776E-05 3.56435776E-05

Contributions to mean A2 freqs= 3.14290635E-02 3.14290635E-02

Contributions to mean diversities= 4.11475077E-04 4.11475077E-04

Zone 2: quasi-neutral zone 2

Lower and upper bounds of St metapopn gamma for zone 2

0.250000000 499.999969

Probability of zone 2= 0.451199740

Coefficients for bivariate distribution of q1 and q2 in metapopulation

G1= 0.105263159 G2= 0.105263159

G3= 0.789473653 G4= 0.789473653

a1= 0.138157889 a2= 0.138157889

b11= 4.93421033E-02 b12= 0.125000000 b22= 4.93421033E-02

Contributions to mean load statistics for zone 2

Loads within In and St= 3.42420512E-03 3.42420512E-03

Load between In and St= 2.32395832E-03

Homozygous load for In and St= 3.76723986E-03 3.76724009E-03

Inbreeding loads= 3.43026448E-04 3.43026448E-04

Selection coefficients against In and St homokaryotypes

1.09964609E-03 1.09964609E-03

Contributions to mean A2 freqs= 2.60752980E-02 2.60752980E-02

Contributions to mean diversities= 9.51203227E-04 9.51203227E-04

Zone 3: moderate selection zone

Lower and upper bounds of St metapopn gamma for zone 3

499.999969 999.999939

Probability of zone 3= 0.106151342

Contributions to mean load statistics over zone 3

Loads within In and St= 1.01985257E-04 1.01968770E-04

Load between In and St= 9.32504990E-05

Homozygous load for In and St= 1.86492558E-04 1.86499383E-04

Inbreeding loads= 8.45073373E-05 8.45306567E-05

Selection coefficients for In and St homokaryotypes

8.76188278E-06 8.70227814E-06

Contributions to mean A2 freqs= 2.69211705E-06 2.69225370E-06

Contributions to mean diversities= 4.87926400E-06 4.87951320E-06

Zone 4: strong selection zone

Upper limit to scaled gamma for St metapopn= 2.50000000

Lower limit to gamma for St metapopn= 999.999939

Upper limit to gamma for St metapopn= 16666.6680

Probability of zone 4= 0.378410041

Contributions to mean load statistics over zone 4

Loads within In and St= 3.25335976E-04 3.25335976E-04

Load between In and St= 2.96032784E-04

Homozygous load for In and St= 5.92058117E-04 5.92058175E-04

Inbreeding loads= 2.66722200E-04 2.66722200E-04

Selection coefficients for In and St homokaryotypes

2.93254852E-05 2.93254852E-05

Contributions to mean A2 freqs= 4.64620234E-06 4.64620234E-06

Contributions to mean diversities= 8.24918232E-08 8.24918232E-08

Mean load statistics over all zones

Loads within In and St= 4.03225422E-03 4.03223792E-03

Load between In and St= 2.85829883E-03

Homozygous load for In and St= 4.72711213E-03 4.72711911E-03

Inbreeding loads= 6.94849412E-04 6.94872753E-04

Selection coefficients for In and St homokaryotypes

1.17325783E-03 1.17325783E-03

Mean frequencies of A2 in In and St= 5.75117022E-02 5.75117022E-02

Ratio of these= 1.00000000

Mean diversities at selected sites in In and St= 1.36764022E-03 1.36764045E-03

Ratio of these= 0.999999821

Mean diversities at neutral sites in In and St= 7.85531010E-03 7.85531010E-03

pi-n/pi-s for In and St= 0.174103916 0.174103945

Ratio of these= 0.999999821

**Neutral Fst for whole population= 0.1**

Scaled migration rate for whole population= 9.00000000

Zone 1: quasi-neutral zone 1

Upper bound scaled selection coefficient for neutrality in St metapopulation= 0.250000000

Probability of zone 1= 5.23817725E-02

Integral of selection coefficient over zone 1= 3.02202563E-04

Mean load statistics for zone 1

Mean q1 and q2= 0.600000024

F1 and F2= 0.983666062 0.983666062

Diversities= 7.84028973E-03 7.84028973E-03

Contributions to loads within In and St= 1.80729199E-04 1.80729199E-04

Contributions to load between In and St = 1.45057231E-04

Contributions to homozygous loads for In and St= 1.81321549E-04 1.81321549E-04

Contributions to inbreeding loads for In and St= 5.92346851E-07 5.92346851E-07

Contributions to selection coefficients for In and St homokaryotypes

3.56435776E-05 3.56435776E-05

Contributions to mean A2 freqs= 3.14290635E-02 3.14290635E-02

Contributions to mean diversities= 4.10688284E-04 4.10688284E-04

Zone 2: quasi-neutral zone 2

Lower and upper bounds of St metapopn gamma for zone 2

0.250000000 499.999969

Probability of zone 2= 0.451199740

Coefficients for bivariate distribution of q1 and q2 in metapopulation

G1= 0.222222224 G2= 0.222222224

G3= 0.555555582 G4= 0.555555582

a1= 0.152777776 a2= 0.152777776

b11= 3.47222239E-02 b12= 0.125000000 b22= 3.47222239E-02

Contributions to mean load statistics for zone 2

Loads within In and St= 3.49288178E-03 3.49288154E-03

Load between In and St= 2.35647405E-03

Homozygous load for In and St= 3.80663015E-03 3.80662992E-03

Inbreeding loads= 3.13746306E-04 3.13746365E-04

Selection coefficients against In and St homokaryotypes

1.13576651E-03 1.13576651E-03

Contributions to mean A2 freqs= 2.63660681E-02 2.63660625E-02

Contributions to mean diversities= 9.19125276E-04 9.19125916E-04

Zone 3: moderate selection zone

Lower and upper bounds of St metapopn gamma for zone 3

499.999969 999.999939

Probability of zone 3= 0.106151342

Contributions to mean load statistics over zone 3

Loads within In and St= 1.06684165E-04 1.06691528E-04

Load between In and St= 9.06626228E-05

Homozygous load for In and St= 1.81317868E-04 1.81323456E-04

Inbreeding loads= 7.46337682E-05 7.46319784E-05

Selection coefficients for In and St homokaryotypes

1.60336494E-05 1.60336494E-05

Contributions to mean A2 freqs= 2.61765490E-06 2.61776677E-06

Contributions to mean diversities= 4.30886439E-06 4.30900354E-06

Zone 4: strong selection zone

Upper limit to scaled gamma for St metapopn= 2.50000000

Lower limit to gamma for St metapopn= 999.999939

Upper limit to gamma for St metapopn= 16666.6680

Probability of zone 4= 0.378410041

Contributions to mean load statistics over zone 4

Loads within In and St= 3.88930232E-04 3.88930290E-04

Load between In and St= 3.31992051E-04

Homozygous load for In and St= 6.63974730E-04 6.63974788E-04

Inbreeding loads= 2.75044906E-04 2.75044906E-04

Selection coefficients for In and St homokaryotypes

5.69224358E-05 5.69224358E-05

Contributions to mean A2 freqs= 5.22497839E-06 5.22497839E-06

Contributions to mean diversities= 9.82410810E-08 9.82410810E-08

Mean load statistics over all zones

Loads within In and St= 4.16922523E-03 4.16923268E-03

Load between In and St= 2.92418594E-03

Homozygous load for In and St= 4.83324425E-03 4.83324984E-03

Inbreeding loads= 6.64017338E-04 6.64015592E-04

Selection coefficients for In and St homokaryotypes

1.24424696E-03 1.24424696E-03

Mean frequencies of A2 in In and St= 5.78029752E-02 5.78029715E-02

Ratio of these= 1.00000012

Mean diversities at selected sites in In and St= 1.33422075E-03 1.33422145E-03

Ratio of these= 0.999999464

Mean diversities at neutral sites in In and St= 7.84028973E-03 7.84028973E-03

pi-n/pi-s for In and St= 0.170174927 0.170175016

Ratio of these= 0.999999464

**Neutral Fst for whole population= 0.15**

Scaled migration rate for whole population= 5.66666651

Zone 1: quasi-neutral zone 1

Upper bound scaled selection coefficient for neutrality in St metapopulation= 0.250000000

Probability of zone 1= 5.23817725E-02

Integral of selection coefficient over zone 1= 3.02202563E-04

Mean load statistics for zone 1

Mean q1 and q2= 0.600000024

F1 and F2= 0.983700812 0.983700812

Diversities= 7.82360975E-03 7.82360975E-03

Contributions to loads within In and St= 1.80730465E-04 1.80730465E-04

Contributions to load between In and St = 1.45057231E-04

Contributions to homozygous loads for In and St= 1.81321549E-04 1.81321549E-04

Contributions to inbreeding loads for In and St= 5.91085950E-07 5.91085950E-07

Contributions to selection coefficients for In and St homokaryotypes

3.56435776E-05 3.56435776E-05

Contributions to mean A2 freqs= 3.14290635E-02 3.14290635E-02

Contributions to mean diversities= 4.09814558E-04 4.09814558E-04

Zone 2: quasi-neutral zone 2

Lower and upper bounds of St metapopn gamma for zone 2

0.250000000 499.999969

Probability of zone 2= 0.451199740

Coefficients for bivariate distribution of q1 and q2 in metapopulation

G1= 0.352941185 G2= 0.352941185

G3= 0.294117630 G4= 0.294117630

a1= 0.169117644 a2= 0.169117644

b11= 1.83823518E-02 b12= 0.125000000 b22= 1.83823518E-02

Contributions to mean load statistics for zone 2

Loads within In and St= 3.56174540E-03 3.56174540E-03

Load between In and St= 2.39162520E-03

Homozygous load for In and St= 3.84764955E-03 3.84764932E-03

Inbreeding loads= 2.85898714E-04 2.85898714E-04

Selection coefficients against In and St homokaryotypes

1.16944313E-03 1.16944313E-03

Contributions to mean A2 freqs= 2.66894680E-02 2.66894680E-02

Contributions to mean diversities= 8.86388996E-04 8.86388822E-04

Zone 3: moderate selection zone

Lower and upper bounds of St metapopn gamma for zone 3

499.999969 999.999939

Probability of zone 3= 0.106151342

Contributions to mean load statistics over zone 3

Loads within In and St= 1.10958026E-04 1.10964727E-04

Load between In and St= 8.86638227E-05

Homozygous load for In and St= 1.77320617E-04 1.77325870E-04

Inbreeding loads= 6.63626342E-05 6.63612227E-05

Selection coefficients for In and St homokaryotypes

2.22921371E-05 2.22921371E-05

Contributions to mean A2 freqs= 2.55933423E-06 2.55943974E-06

Contributions to mean diversities= 3.82952067E-06 3.82957251E-06

Zone 4: strong selection zone

Upper limit to scaled gamma for St metapopn= 2.50000000

Lower limit to gamma for St metapopn= 999.999939

Upper limit to gamma for St metapopn= 16666.6680

Probability of zone 4= 0.378410041

Contributions to mean load statistics over zone 4

Loads within In and St= 4.31326655E-04 4.31326713E-04

Load between In and St= 3.50118469E-04

Homozygous load for In and St= 7.00226985E-04 7.00226985E-04

Inbreeding loads= 2.68900563E-04 2.68900563E-04

Selection coefficients for In and St homokaryotypes

8.11815262E-05 8.11815262E-05

Contributions to mean A2 freqs= 5.51147559E-06 5.51147559E-06

Contributions to mean diversities= 1.08318538E-07 1.08318538E-07

Mean load statistics over all zones

Loads within In and St= 4.28476045E-03 4.28476743E-03

Load between In and St= 2.97546457E-03

Homozygous load for In and St= 4.90651885E-03 4.90652351E-03

Inbreeding loads= 6.21753046E-04 6.21751591E-04

Selection coefficients for In and St homokaryotypes

1.30844116E-03 1.30844116E-03

Mean frequencies of A2 in In and St= 5.81265986E-02 5.81265986E-02

Ratio of these= 1.00000000

Mean diversities at selected sites in In and St= 1.30014133E-03 1.30014122E-03

Ratio of these= 1.00000012

Mean diversities at neutral sites in In and St= 7.82360975E-03 7.82360975E-03

pi-n/pi-s for In and St= 0.166181773 0.166181758

Ratio of these= 1.00000012

**Neutral Fst for whole population= 0.2**

Scaled migration rate for whole population= 4.00000000

Zone 1: quasi-neutral zone 1

Upper bound scaled selection coefficient for neutrality in St metapopulation= 0.250000000

Probability of zone 1= 5.23817725E-02

Integral of selection coefficient over zone 1= 3.02202563E-04

Mean load statistics for zone 1

Mean q1 and q2= 0.600000024

F1 and F2= 0.983739853 0.983739853

Diversities= 7.80487061E-03 7.80487061E-03

Contributions to loads within In and St= 1.80731877E-04 1.80731877E-04

Contributions to load between In and St = 1.45057231E-04

Contributions to homozygous loads for In and St= 1.81321549E-04 1.81321549E-04

Contributions to inbreeding loads for In and St= 5.89662989E-07 5.89662989E-07

Contributions to selection coefficients for In and St homokaryotypes

3.57031822E-05 3.57031822E-05

Contributions to mean A2 freqs= 3.14290635E-02 3.14290635E-02

Contributions to mean diversities= 4.08832944E-04 4.08832944E-04

Zone 2: quasi-neutral zone 2

Lower and upper bounds of St metapopn gamma for zone 2

0.250000000 499.999969

Probability of zone 2= 0.451199740

Coefficients for bivariate distribution of q1 and q2 in metapopulation

G1= 0.500000000 G2= 0.500000000

G3= 0.00000000 G4= 0.00000000

a1= 0.187500000 a2= 0.187500000

b11= 0.00000000 b12= 0.125000000 b22= 0.00000000

Contributions to mean load statistics for zone 2

Loads within In and St= 3.63145210E-03 3.63145210E-03

Load between In and St= 2.43007904E-03

Homozygous load for In and St= 3.89080984E-03 3.89080984E-03

Inbreeding loads= 2.59357155E-04 2.59357184E-04

Selection coefficients against In and St homokaryotypes

1.20067596E-03 1.20067596E-03

Contributions to mean A2 freqs= 2.70516295E-02 2.70516295E-02

Contributions to mean diversities= 8.52864934E-04 8.52864934E-04

Zone 3: moderate selection zone

Lower and upper bounds of St metapopn gamma for zone 3

499.999969 999.999939

Probability of zone 3= 0.106151342

Contributions to mean load statistics over zone 3

Loads within In and St= 1.15091119E-04 1.15101902E-04

Load between In and St= 8.72165256E-05

Homozygous load for In and St= 1.74426226E-04 1.74431843E-04

Inbreeding loads= 5.93350123E-05 5.93298282E-05

Selection coefficients for In and St homokaryotypes

2.78949738E-05 2.78949738E-05

Contributions to mean A2 freqs= 2.51611345E-06 2.51622578E-06

Contributions to mean diversities= 3.42105636E-06 3.42103249E-06

Zone 4: strong selection zone

Upper limit to scaled gamma for St metapopn= 2.50000000

Lower limit to gamma for St metapopn= 999.999939

Upper limit to gamma for St metapopn= 16666.6680

Probability of zone 4= 0.378410041

Contributions to mean load statistics over zone 4

Loads within In and St= 4.67680249E-04 4.67680191E-04

Load between In and St= 3.63982661E-04

Homozygous load for In and St= 7.27954961E-04 7.27954961E-04

Inbreeding loads= 2.60273839E-04 2.60273839E-04

Selection coefficients for In and St homokaryotypes

1.03712082E-04 1.03712082E-04

Contributions to mean A2 freqs= 5.72726231E-06 5.72726231E-06

Contributions to mean diversities= 1.16639711E-07 1.16639711E-07

Mean load statistics over all zones

Loads within In and St= 4.39495547E-03 4.39496618E-03

Load between In and St= 3.02633550E-03

Homozygous load for In and St= 4.97451238E-03 4.97451844E-03

Inbreeding loads= 5.79555635E-04 5.79550513E-04

Selection coefficients for In and St homokaryotypes

1.36768818E-03 1.36768818E-03

Mean frequencies of A2 in In and St= 5.84889352E-02 5.84889352E-02

Ratio of these= 1.00000000

Mean diversities at selected sites in In and St= 1.26523559E-03 1.26523548E-03

Ratio of these= 1.00000012

Mean diversities at neutral sites in In and St= 7.80487061E-03 7.80487061E-03

pi-n/pi-s for In and St= 0.162108466 0.162108451

Ratio of these= 1.00000012

**Neutral Fst for whole population= 0.25**

Scaled migration rate for whole population= 3.00000000

Zone 1: quasi-neutral zone 1

Upper bound scaled selection coefficient for neutrality in St metapopulation= 0.250000000

Probability of zone 1= 5.23817725E-02

Integral of selection coefficient over zone 1= 3.02202563E-04

Mean load statistics for zone 1

Mean q1 and q2= 0.600000024

F1 and F2= 0.983783841 0.983783841

Diversities= 7.78375613E-03 7.78375613E-03

Contributions to loads within In and St= 1.80733492E-04 1.80733492E-04

Contributions to load between In and St = 1.45057231E-04

Contributions to homozygous loads for In and St= 1.81321549E-04 1.81321549E-04

Contributions to inbreeding loads for In and St= 5.88059834E-07 5.88059834E-07

Contributions to selection coefficients for In and St homokaryotypes

3.57031822E-05 3.57031822E-05

Contributions to mean A2 freqs= 3.14290635E-02 3.14290635E-02

Contributions to mean diversities= 4.07726940E-04 4.07726940E-04

Zone 2: quasi-neutral zone 2

Lower and upper bounds of St metapopn gamma for zone 2

0.250000000 499.999969

Probability of zone 2= 0.451199740

Coefficients for bivariate distribution of q1 and q2 in metapopulation

G1= 0.666666687 G2= 0.666666687

G3= -0.333333373 G4= -0.333333373

a1= 0.208333343 a2= 0.208333343

b11= -2.08333358E-02 b12= 0.125000000 b22= -2.08333358E-02

Contributions to mean load statistics for zone 2

Loads within In and St= 3.70273832E-03 3.70273832E-03

Load between In and St= 2.47268821E-03

Homozygous load for In and St= 3.93675640E-03 3.93675640E-03

Inbreeding loads= 2.34016174E-04 2.34016174E-04

Selection coefficients against In and St homokaryotypes

1.22928619E-03 1.22928619E-03

Contributions to mean A2 freqs= 2.74601076E-02 2.74601057E-02

Contributions to mean diversities= 8.18401633E-04 8.18401633E-04

Zone 3: moderate selection zone

Lower and upper bounds of St metapopn gamma for zone 3

499.999969 999.999939

Probability of zone 3= 0.106151342

Contributions to mean load statistics over zone 3

Loads within In and St= 1.19351345E-04 1.19361379E-04

Load between In and St= 8.63363457E-05

Homozygous load for In and St= 1.72665328E-04 1.72671906E-04

Inbreeding loads= 5.33139282E-05 5.33104649E-05

Selection coefficients for In and St homokaryotypes

3.30209732E-05 3.30209732E-05

Contributions to mean A2 freqs= 2.48846732E-06 2.48859897E-06

Contributions to mean diversities= 3.07017740E-06 3.07008645E-06

Zone 4: strong selection zone

Upper limit to scaled gamma for St metapopn= 2.50000000

Lower limit to gamma for St metapopn= 999.999939

Upper limit to gamma for St metapopn= 16666.6680

Probability of zone 4= 0.378410041

Contributions to mean load statistics over zone 4

Loads within In and St= 5.02342125E-04 5.02342067E-04

Load between In and St= 3.76949116E-04

Homozygous load for In and St= 7.53887056E-04 7.53887056E-04

Inbreeding loads= 2.51544465E-04 2.51544465E-04

Selection coefficients for In and St homokaryotypes

1.25408173E-04 1.25408173E-04

Contributions to mean A2 freqs= 5.92811239E-06 5.92811239E-06

Contributions to mean diversities= 1.24335870E-07 1.24335870E-07

Mean load statistics over all zones

Loads within In and St= 4.50516492E-03 4.50517517E-03

Load between In and St= 3.08103091E-03

Homozygous load for In and St= 5.04463026E-03 5.04463678E-03

Inbreeding loads= 5.39462664E-04 5.39459172E-04

Selection coefficients for In and St homokaryotypes

1.42312050E-03 1.42312050E-03

Mean frequencies of A2 in In and St= 5.88975884E-02 5.88975847E-02

Ratio of these= 1.00000012

Mean diversities at selected sites in In and St= 1.22932310E-03 1.22932298E-03

Ratio of these= 1.00000012

Mean diversities at neutral sites in In and St= 7.78375613E-03 7.78375613E-03

pi-n/pi-s for In and St= 0.157934427 0.157934412

Ratio of these= 1.00000012

**h= 0.45**

**Neutral Fst for whole population= 0.05**

Scaled migration rate for whole population= 19.0000000

Zone 1: quasi-neutral zone 1

Upper bound scaled selection coefficient for neutrality in St metapopulation= 0.250000000

Probability of zone 1= 5.23817725E-02

Integral of selection coefficient over zone 1= 3.02202563E-04

Mean load statistics for zone 1

Mean q1 and q2= 0.600000024

F1 and F2= 0.983634770 0.983634770

Diversities= 7.85531010E-03 7.85531010E-03

Contributions to loads within In and St= 1.81202864E-04 1.81202864E-04

Contributions to load between In and St = 1.74068671E-04

Contributions to homozygous loads for In and St= 1.81321549E-04 1.81321549E-04

Contributions to inbreeding loads for In and St= 1.18685527E-07 1.18685527E-07

Contributions to selection coefficients for In and St homokaryotypes

7.15255737E-06 7.15255737E-06

Contributions to mean A2 freqs= 3.14290635E-02 3.14290635E-02

Contributions to mean diversities= 4.11475077E-04 4.11475077E-04

Zone 2: quasi-neutral zone 2

Lower and upper bounds of St metapopn gamma for zone 2

0.250000000 499.999969

Probability of zone 2= 0.451199740

Coefficients for bivariate distribution of q1 and q2 in metapopulation

G1= 0.105263159 G2= 0.105263159

G3= 0.789473653 G4= 0.789473653

a1= 0.227631569 a2= 0.227631569

b11= 9.86842345E-03 b12= 2.50000060E-02 b22= 9.86842345E-03

Contributions to mean load statistics for zone 2

Loads within In and St= 2.88571161E-03 2.88571161E-03

Load between In and St= 2.71748402E-03

Homozygous load for In and St= 2.92771682E-03 2.92771682E-03

Inbreeding loads= 4.20013930E-05 4.20013930E-05

Selection coefficients against In and St homokaryotypes

1.68204308E-04 1.68204308E-04

Contributions to mean A2 freqs= 2.50616670E-02 2.50616670E-02

Contributions to mean diversities= 7.77502020E-04 7.77502020E-04

Zone 3: moderate selection zone

Lower and upper bounds of St metapopn gamma for zone 3

499.999969 999.999939

Probability of zone 3= 0.106151342

Contributions to mean load statistics over zone 3

Loads within In and St= 9.11587776E-05 9.11616298E-05

Load between In and St= 9.02298780E-05

Homozygous load for In and St= 1.00254081E-04 1.00256613E-04

Inbreeding loads= 9.09531445E-06 9.09498704E-06

Selection coefficients for In and St homokaryotypes

9.53674316E-07 9.53674316E-07

Contributions to mean A2 freqs= 1.45062506E-06 1.45067588E-06

Contributions to mean diversities= 2.63181551E-06 2.63190668E-06

Zone 4: strong selection zone

Upper limit to scaled gamma for St metapopn= 2.50000000

Lower limit to gamma for St metapopn= 999.999939

Upper limit to gamma for St metapopn= 16666.6680

Probability of zone 4= 0.378410041

Contributions to mean load statistics over zone 4

Loads within In and St= 2.99125968E-04 2.99125997E-04

Load between In and St= 2.96020706E-04

Homozygous load for In and St= 3.28911497E-04 3.28911497E-04

Inbreeding loads= 2.97856950E-05 2.97856968E-05

Selection coefficients for In and St homokaryotypes

3.09944153E-06 3.09944153E-06

Contributions to mean A2 freqs= 2.57004353E-06 2.57004353E-06

Contributions to mean diversities= 4.56343408E-08 4.56343408E-08

Mean load statistics over all zones

Loads within In and St= 3.45719908E-03 3.45720188E-03

Load between In and St= 3.27780307E-03

Homozygous load for In and St= 3.53820389E-03 3.53820645E-03

Inbreeding loads= 8.10010897E-05 8.10007623E-05

Selection coefficients for In and St homokaryotypes

1.79350376E-04 1.79409981E-04

Mean frequencies of A2 in In and St= 5.64947501E-02 5.64947501E-02

Ratio of these= 1.00000000

Mean diversities at selected sites in In and St= 1.19165459E-03 1.19165471E-03

Ratio of these= 0.999999881

Mean diversities at neutral sites in In and St= 7.85531010E-03 7.85531010E-03

pi-n/pi-s for In and St= 0.151700512 0.151700526

Ratio of these= 0.999999881

**Neutral Fst for whole population= 0.1**

Scaled migration rate for whole population= 9.00000000

Zone 1: quasi-neutral zone 1

Upper bound scaled selection coefficient for neutrality in St metapopulation= 0.250000000

Probability of zone 1= 5.23817725E-02

Integral of selection coefficient over zone 1= 3.02202563E-04

Mean load statistics for zone 1

Mean q1 and q2= 0.600000024

F1 and F2= 0.983666062 0.983666062

Diversities= 7.84028973E-03 7.84028973E-03

Contributions to loads within In and St= 1.81203082E-04 1.81203082E-04

Contributions to load between In and St = 1.74068671E-04

Contributions to homozygous loads for In and St= 1.81321549E-04 1.81321549E-04

Contributions to inbreeding loads for In and St= 1.18469373E-07 1.18469373E-07

Contributions to selection coefficients for In and St homokaryotypes

7.15255737E-06 7.15255737E-06

Contributions to mean A2 freqs= 3.14290635E-02 3.14290635E-02

Contributions to mean diversities= 4.10688284E-04 4.10688284E-04

Zone 2: quasi-neutral zone 2

Lower and upper bounds of St metapopn gamma for zone 2

0.250000000 499.999969

Probability of zone 2= 0.451199740

Coefficients for bivariate distribution of q1 and q2 in metapopulation

G1= 0.222222224 G2= 0.222222224

G3= 0.555555582 G4= 0.555555582

a1= 0.230555549 a2= 0.230555549

b11= 6.94444636E-03 b12= 2.50000060E-02 b22= 6.94444636E-03

Contributions to mean load statistics for zone 2

Loads within In and St= 2.93610524E-03 2.93610524E-03

Load between In and St= 2.76291743E-03

Homozygous load for In and St= 2.97761825E-03 2.97761871E-03

Inbreeding loads= 4.15127433E-05 4.15127470E-05

Selection coefficients against In and St homokaryotypes

1.73151493E-04 1.73151493E-04

Contributions to mean A2 freqs= 2.51422245E-02 2.51422245E-02

Contributions to mean diversities= 7.74621905E-04 7.74621789E-04

Zone 3: moderate selection zone

Lower and upper bounds of St metapopn gamma for zone 3

499.999969 999.999939

Probability of zone 3= 0.106151342

Contributions to mean load statistics over zone 3

Loads within In and St= 9.74276627E-05 9.74305221E-05

Load between In and St= 9.55767828E-05

Homozygous load for In and St= 1.06195039E-04 1.06197600E-04

Inbreeding loads= 8.76732065E-06 8.76703598E-06

Selection coefficients for In and St homokaryotypes

1.84774399E-06 1.84774399E-06

Contributions to mean A2 freqs= 1.53678468E-06 1.53683595E-06

Contributions to mean diversities= 2.53663234E-06 2.53669214E-06

Zone 4: strong selection zone

Upper limit to scaled gamma for St metapopn= 2.50000000

Lower limit to gamma for St metapopn= 999.999939

Upper limit to gamma for St metapopn= 16666.6680

Probability of zone 4= 0.378410041

Contributions to mean load statistics over zone 4

Loads within In and St= 3.43474880E-04 3.43474880E-04

Load between In and St= 3.37430421E-04

Homozygous load for In and St= 3.74922296E-04 3.74922325E-04

Inbreeding loads= 3.14473036E-05 3.14473036E-05

Selection coefficients for In and St homokaryotypes

6.02006912E-06 6.02006912E-06

Contributions to mean A2 freqs= 2.93626636E-06 2.93626636E-06

Contributions to mean diversities= 5.52083215E-08 5.52083179E-08

Mean load statistics over all zones

Loads within In and St= 3.55821080E-03 3.55821359E-03

Load between In and St= 3.36999330E-03

Homozygous load for In and St= 3.64005705E-03 3.64006008E-03

Inbreeding loads= 8.18458357E-05 8.18455592E-05

Selection coefficients for In and St homokaryotypes

1.88171864E-04 1.88231468E-04

Mean frequencies of A2 in In and St= 5.65757640E-02 5.65757640E-02

Ratio of these= 1.00000000

Mean diversities at selected sites in In and St= 1.18790206E-03 1.18790194E-03

Ratio of these= 1.00000012

Mean diversities at neutral sites in In and St= 7.84028973E-03 7.84028973E-03

pi-n/pi-s for In and St= 0.151512519 0.151512504

Ratio of these= 1.00000012

**Neutral Fst for whole population= 0.15**

Scaled migration rate for whole population= 5.66666651

Zone 1: quasi-neutral zone 1

Upper bound scaled selection coefficient for neutrality in St metapopulation= 0.250000000

Probability of zone 1= 5.23817725E-02

Integral of selection coefficient over zone 1= 3.02202563E-04

Mean load statistics for zone 1

Mean q1 and q2= 0.600000024

F1 and F2= 0.983700812 0.983700812

Diversities= 7.82360975E-03 7.82360975E-03

Contributions to loads within In and St= 1.81203330E-04 1.81203330E-04

Contributions to load between In and St = 1.74068671E-04

Contributions to homozygous loads for In and St= 1.81321549E-04 1.81321549E-04

Contributions to inbreeding loads for In and St= 1.18217194E-07 1.18217194E-07

Contributions to selection coefficients for In and St homokaryotypes

7.15255737E-06 7.15255737E-06

Contributions to mean A2 freqs= 3.14290635E-02 3.14290635E-02

Contributions to mean diversities= 4.09814558E-04 4.09814558E-04

Zone 2: quasi-neutral zone 2

Lower and upper bounds of St metapopn gamma for zone 2

0.250000000 499.999969

Probability of zone 2= 0.451199740

Coefficients for bivariate distribution of q1 and q2 in metapopulation

G1= 0.352941185 G2= 0.352941185

G3= 0.294117630 G4= 0.294117630

a1= 0.233823523 a2= 0.233823523

b11= 3.67647130E-03 b12= 2.50000060E-02 b22= 3.67647130E-03

Contributions to mean load statistics for zone 2

Loads within In and St= 2.99102534E-03 2.99102534E-03

Load between In and St= 2.81244121E-03

Homozygous load for In and St= 3.03199538E-03 3.03199538E-03

Inbreeding loads= 4.09698587E-05 4.09698587E-05

Selection coefficients against In and St homokaryotypes

1.78575516E-04 1.78575516E-04

Contributions to mean A2 freqs= 2.52316892E-02 2.52316892E-02

Contributions to mean diversities= 7.71382824E-04 7.71382824E-04

Zone 3: moderate selection zone

Lower and upper bounds of St metapopn gamma for zone 3

499.999969 999.999939

Probability of zone 3= 0.106151342

Contributions to mean load statistics over zone 3

Loads within In and St= 1.03625964E-04 1.03629289E-04

Load between In and St= 1.00849989E-04

Homozygous load for In and St= 1.12053771E-04 1.12056761E-04

Inbreeding loads= 8.42795271E-06 8.42760983E-06

Selection coefficients for In and St homokaryotypes

2.80141830E-06 2.80141830E-06

Contributions to mean A2 freqs= 1.62171284E-06 1.62177264E-06

Contributions to mean diversities= 2.43790078E-06 2.43792761E-06

Zone 4: strong selection zone

Upper limit to scaled gamma for St metapopn= 2.50000000

Lower limit to gamma for St metapopn= 999.999939

Upper limit to gamma for St metapopn= 16666.6680

Probability of zone 4= 0.378410041

Contributions to mean load statistics over zone 4

Loads within In and St= 3.71228351E-04 3.71228351E-04

Load between In and St= 3.62503662E-04

Homozygous load for In and St= 4.02781094E-04 4.02781094E-04

Inbreeding loads= 3.15528559E-05 3.15528596E-05

Selection coefficients for In and St homokaryotypes

8.70227814E-06 8.70227814E-06

Contributions to mean A2 freqs= 3.15345642E-06 3.15345642E-06

Contributions to mean diversities= 6.19660412E-08 6.19660483E-08

Mean load statistics over all zones

Loads within In and St= 3.64708295E-03 3.64708621E-03

Load between In and St= 3.44986329E-03

Homozygous load for In and St= 3.72815179E-03 3.72815481E-03

Inbreeding loads= 8.10688798E-05 8.10685451E-05

Selection coefficients for In and St homokaryotypes

1.97172165E-04 1.97231770E-04

Mean frequencies of A2 in In and St= 5.66655248E-02 5.66655248E-02

Ratio of these= 1.00000000

Mean diversities at selected sites in In and St= 1.18369714E-03 1.18369726E-03

Ratio of these= 0.999999881

Mean diversities at neutral sites in In and St= 7.82360975E-03 7.82360975E-03

pi-n/pi-s for In and St= 0.151298076 0.151298091

Ratio of these= 0.999999881

**Neutral Fst for whole population= 0.20**

Scaled migration rate for whole population= 4.00000000

Zone 1: quasi-neutral zone 1

Upper bound scaled selection coefficient for neutrality in St metapopulation= 0.250000000

Probability of zone 1= 5.23817725E-02

Integral of selection coefficient over zone 1= 3.02202563E-04

Mean load statistics for zone 1

Mean q1 and q2= 0.600000024

F1 and F2= 0.983739853 0.983739853

Diversities= 7.80487061E-03 7.80487061E-03

Contributions to loads within In and St= 1.81203621E-04 1.81203621E-04

Contributions to load between In and St = 1.74068671E-04

Contributions to homozygous loads for In and St= 1.81321549E-04 1.81321549E-04

Contributions to inbreeding loads for In and St= 1.17928991E-07 1.17928991E-07

Contributions to selection coefficients for In and St homokaryotypes

7.15255737E-06 7.15255737E-06

Contributions to mean A2 freqs= 3.14290635E-02 3.14290635E-02

Contributions to mean diversities= 4.08832944E-04 4.08832944E-04

Zone 2: quasi-neutral zone 2

Lower and upper bounds of St metapopn gamma for zone 2

0.250000000 499.999969

Probability of zone 2= 0.451199740

Coefficients for bivariate distribution of q1 and q2 in metapopulation

G1= 0.500000000 G2= 0.500000000

G3= 0.00000000 G4= 0.00000000

a1= 0.237499997 a2= 0.237499997

b11= 0.00000000 b12= 2.50000060E-02 b22= 0.00000000

Contributions to mean load statistics for zone 2

Loads within In and St= 3.05106374E-03 3.05106328E-03

Load between In and St= 2.86658271E-03

Homozygous load for In and St= 3.09143052E-03 3.09143052E-03

Inbreeding loads= 4.03640988E-05 4.03640988E-05

Selection coefficients against In and St homokaryotypes

1.84476376E-04 1.84476376E-04

Contributions to mean A2 freqs= 2.53315475E-02 2.53315475E-02

Contributions to mean diversities= 7.67712540E-04 7.67712540E-04

Zone 3: moderate selection zone

Lower and upper bounds of St metapopn gamma for zone 3

499.999969 999.999939

Probability of zone 3= 0.106151342

Contributions to mean load statistics over zone 3

Loads within In and St= 1.09862187E-04 1.09866007E-04

Load between In and St= 1.06148662E-04

Homozygous load for In and St= 1.17940828E-04 1.17944517E-04

Inbreeding loads= 8.07864581E-06 8.07849756E-06

Selection coefficients for In and St homokaryotypes

3.69548798E-06 3.69548798E-06

Contributions to mean A2 freqs= 1.70686792E-06 1.70694148E-06

Contributions to mean diversities= 2.33597189E-06 2.33595802E-06

Zone 4: strong selection zone

Upper limit to scaled gamma for St metapopn= 2.50000000

Lower limit to gamma for St metapopn= 999.999939

Upper limit to gamma for St metapopn= 16666.6680

Probability of zone 4= 0.378410041

Contributions to mean load statistics over zone 4

Loads within In and St= 3.95253999E-04 3.95253999E-04

Load between In and St= 3.83931241E-04

Homozygous load for In and St= 4.26589715E-04 4.26589715E-04

Inbreeding loads= 3.13356541E-05 3.13356504E-05

Selection coefficients for In and St homokaryotypes

1.13248825E-05 1.13248825E-05

Contributions to mean A2 freqs= 3.34033757E-06 3.34033757E-06

Contributions to mean diversities= 6.80078855E-08 6.80078855E-08

Mean load statistics over all zones

Loads within In and St= 3.73738375E-03 3.73738701E-03

Load between In and St= 3.53073119E-03

Homozygous load for In and St= 3.81728262E-03 3.81728634E-03

Inbreeding loads= 7.98963301E-05 7.98961773E-05

Selection coefficients for In and St homokaryotypes

2.06649303E-04 2.06649303E-04

Mean frequencies of A2 in In and St= 5.67656569E-02 5.67656569E-02

Ratio of these= 1.00000000

Mean diversities at selected sites in In and St= 1.17894949E-03 1.17894949E-03

Ratio of these= 1.00000000

Mean diversities at neutral sites in In and St= 7.80487061E-03 7.80487061E-03

pi-n/pi-s for In and St= 0.151053041 0.151053041

Ratio of these= 1.00000000

**Neutral Fst for whole population= 0.25**

Scaled migration rate for whole population= 3.00000000

Zone 1: quasi-neutral zone 1

Upper bound scaled selection coefficient for neutrality in St metapopulation= 0.250000000

Probability of zone 1= 5.23817725E-02

Integral of selection coefficient over zone 1= 3.02202563E-04

Mean load statistics for zone 1

Mean q1 and q2= 0.600000024

F1 and F2= 0.983783841 0.983783841

Diversities= 7.78375613E-03 7.78375613E-03

Contributions to loads within In and St= 1.81203941E-04 1.81203941E-04

Contributions to load between In and St = 1.74068671E-04

Contributions to homozygous loads for In and St= 1.81321549E-04 1.81321549E-04

Contributions to inbreeding loads for In and St= 1.17604763E-07 1.17604763E-07

Contributions to selection coefficients for In and St homokaryotypes

7.15255737E-06 7.15255737E-06

Contributions to mean A2 freqs= 3.14290635E-02 3.14290635E-02

Contributions to mean diversities= 4.07726940E-04 4.07726940E-04

Zone 2: quasi-neutral zone 2

Lower and upper bounds of St metapopn gamma for zone 2

0.250000000 499.999969

Probability of zone 2= 0.451199740

Coefficients for bivariate distribution of q1 and q2 in metapopulation

G1= 0.666666687 G2= 0.666666687

G3= -0.333333373 G4= -0.333333373

a1= 0.241666660 a2= 0.241666660

b11= -4.16666828E-03 b12= 2.50000060E-02 b22= -4.16666828E-03

Contributions to mean load statistics for zone 2

Loads within In and St= 3.11692548E-03 3.11692548E-03

Load between In and St= 2.92598479E-03

Homozygous load for In and St= 3.15660890E-03 3.15660890E-03

Inbreeding loads= 3.96847536E-05 3.96847536E-05

Selection coefficients against In and St homokaryotypes

1.90913677E-04 1.90913677E-04

Contributions to mean A2 freqs= 2.54437532E-02 2.54437532E-02

Contributions to mean diversities= 7.63519260E-04 7.63519260E-04

Zone 3: moderate selection zone

Lower and upper bounds of St metapopn gamma for zone 3

499.999969 999.999939

Probability of zone 3= 0.106151342

Contributions to mean load statistics over zone 3

Loads within In and St= 1.16289004E-04 1.16293981E-04

Load between In and St= 1.11614521E-04

Homozygous load for In and St= 1.24013473E-04 1.24018159E-04

Inbreeding loads= 7.72453404E-06 7.72423209E-06

Selection coefficients for In and St homokaryotypes

4.64916229E-06 4.70876694E-06

Contributions to mean A2 freqs= 1.79442384E-06 1.79451717E-06

Contributions to mean diversities= 2.23231586E-06 2.23225538E-06

Zone 4: strong selection zone

Upper limit to scaled gamma for St metapopn= 2.50000000

Lower limit to gamma for St metapopn= 999.999939

Upper limit to gamma for St metapopn= 16666.6680

Probability of zone 4= 0.378410041

Contributions to mean load statistics over zone 4

Loads within In and St= 4.18807700E-04 4.18807700E-04

Load between In and St= 4.04864695E-04

Homozygous load for In and St= 4.49848972E-04 4.49849031E-04

Inbreeding loads= 3.10419018E-05 3.10419018E-05

Selection coefficients for In and St homokaryotypes

1.39474869E-05 1.39474869E-05

Contributions to mean A2 freqs= 3.52647135E-06 3.52647135E-06

Contributions to mean diversities= 7.39368744E-08 7.39368744E-08

Mean load statistics over all zones

Loads within In and St= 3.83322593E-03 3.83323105E-03

Load between In and St= 3.61653278E-03

Homozygous load for In and St= 3.91179277E-03 3.91179742E-03

Inbreeding loads= 7.85687953E-05 7.85684970E-05

Selection coefficients for In and St homokaryotypes

2.16662884E-04 2.16662884E-04

Mean frequencies of A2 in In and St= 5.68781383E-02 5.68781383E-02

Ratio of these= 1.00000000

Mean diversities at selected sites in In and St= 1.17355236E-03 1.17355236E-03

Ratio of these= 1.00000000

Mean diversities at neutral sites in In and St= 7.78375613E-03 7.78375613E-03

pi-n/pi-s for In and St= 0.150769413 0.150769413

Ratio of these= 1.00000000

**Section 2**

**Mean selection coefficient= 5.0E-04**

**Mean scaled selection coefficient for whole popn= 1000**

**Inversion frequency= 0.1**

**h= 0.05**

**Neutral Fst for whole population= 0.05**

Scaled migration rate for whole population= 19.0000000

Zone 1: quasi-neutral zone 1

Upper bound scaled selection coefficient for neutrality in St metapopulation= 0.250000000

Probability of zone 1= 6.65597618E-02

Integral of selection coefficient over zone 1= 2.13332591E-04

Mean load statistics for zone 1

Mean q1 and q2= 0.600000024

F1 and F2= 0.996683598 0.970923305

Diversities= 1.59187312E-03 1.39568131E-02

Contributions to loads within In and St= 1.27846739E-04 1.26659710E-04

Contributions to load between In and St = 8.19197157E-05

Contributions to homozygous loads for In and St= 1.27999563E-04 1.27999563E-04

Contributions to inbreeding loads for In and St= 1.52816241E-07 1.33984418E-06

Contributions to selection coefficients for In and St homokaryotypes

4.59551811E-05 4.47630882E-05

Contributions to mean A2 freqs= 3.99358571E-02 3.99358571E-02

Contributions to mean diversities= 1.05954699E-04 9.28962138E-04

Zone 2: quasi-neutral zone 2

Lower and upper bounds of St metapopn gamma for zone 2

0.250000000 499.999969

Probability of zone 2= 0.560461998

Coefficients for bivariate distribution of q1 and q2 in metapopulation

G1= 0.526315808 G2= 5.84795326E-02

G3= -5.26316166E-02 G4= 0.883040905

a1= 9.73684248E-03 a2= 8.76315832E-02

b11= -2.36842272E-04 b12= 8.09999928E-02 b22= 0.321868390

Contributions to mean load statistics for zone 2

Loads within In and St= 0.248887539 1.77201710E-03

Load between In and St= 1.37923919E-02

Homozygous load for In and St= 0.250295043 3.31312953E-03

Inbreeding loads= 1.40765402E-03 1.54111290E-03

Selection coefficients against In and St homokaryotypes

0.209504366 -1.20929480E-02

Contributions to mean A2 freqs= 0.179473698 2.78880447E-02

Contributions to mean diversities= 7.24899350E-04 2.59777741E-03

Zone 3: moderate selection zone

Lower and upper bounds of St metapopn gamma for zone 3

499.999969 5000.00000

Probability of zone 3= 0.339170814

Contributions to mean load statistics over zone 3

Loads within In and St= 1.20598136E-03 2.15692271E-04

Load between In and St= 2.22889925E-04

Homozygous load for In and St= 2.85509671E-03 1.59872649E-03

Inbreeding loads= 1.64911465E-03 1.38303498E-03

Selection coefficients for In and St homokaryotypes

9.82582569E-04 -7.15255737E-06

Contributions to mean A2 freqs= 4.45371224E-05 2.58625314E-05

Contributions to mean diversities= 5.53796526E-05 4.88880869E-05

Zone 4: strong selection zone

Upper limit to scaled gamma for St metapopn= 2.50000000

Lower limit to gamma for St metapopn= 5000.00000

Upper limit to gamma for St metapopn= 7500.00049

Probability of zone 4= 2.19503045E-02

Contributions to mean load statistics over zone 4

Loads within In and St= 9.07048889E-05 2.31345912E-05

Load between In and St= 1.80544139E-05

Homozygous load for In and St= 2.13243809E-04 1.47686529E-04

Inbreeding loads= 1.22539001E-04 1.24552113E-04

Selection coefficients for In and St homokaryotypes

7.26580620E-05 5.06639481E-06

Contributions to mean A2 freqs= 1.29236480E-06 1.29236480E-06

Contributions to mean diversities= 5.94249672E-09 2.71680616E-08

Mean load statistics over all zones

Loads within In and St= 0.250312090 2.13750359E-03

Load between In and St= 1.41152563E-02

Homozygous load for In and St= 0.253491372 5.18754218E-03

Inbreeding loads= 3.17946053E-03 3.05003976E-03

Selection coefficients for In and St homokaryotypes

0.210374773 -1.20497942E-02

Mean frequencies of A2 in In and St= 0.219455391 6.78510517E-02

Ratio of these= 3.23436975

Mean diversities at selected sites in In and St= 8.86239635E-04 3.57565493E-03

Ratio of these= 0.247853786

Mean diversities at neutral sites in In and St= 1.59187312E-03 1.39568131E-02

pi-n/pi-s for In and St= 0.556727529 0.256194234

Ratio of these= 2.17306805

**Neutral Fst for whole population= 0.1**

Scaled migration rate for whole population= 9.00000000

Zone 1: quasi-neutral zone 1

Upper bound scaled selection coefficient for neutrality in St metapopulation= 0.250000000

Probability of zone 1= 6.65597618E-02

Integral of selection coefficient over zone 1= 2.13332591E-04

Mean load statistics for zone 1

Mean q1 and q2= 0.600000024

F1 and F2= 0.996689916 0.970978200

Diversities= 1.58884050E-03 1.39304632E-02

Contributions to loads within In and St= 1.27847030E-04 1.26662228E-04

Contributions to load between In and St = 8.19197157E-05

Contributions to homozygous loads for In and St= 1.27999563E-04 1.27999563E-04

Contributions to inbreeding loads for In and St= 1.52536501E-07 1.33732647E-06

Contributions to selection coefficients for In and St homokaryotypes

4.59551811E-05 4.47630882E-05

Contributions to mean A2 freqs= 3.99358571E-02 3.99358571E-02

Contributions to mean diversities= 1.05752843E-04 9.27208341E-04

Zone 2: quasi-neutral zone 2

Lower and upper bounds of St metapopn gamma for zone 2

0.250000000 499.999969

Probability of zone 2= 0.560461998

Coefficients for bivariate distribution of q1 and q2 in metapopulation

G1= 1.11111104 G2= 0.123456798

G3= -1.22222209 G4= 0.753086388

a1= 1.49999997E-02 a2= 0.134999990

b11= -5.49999997E-03 b12= 8.09999928E-02 b22= 0.274499953

Contributions to mean load statistics for zone 2

Loads within In and St= 0.263836414 1.78103044E-03

Load between In and St= 1.44980252E-02

Homozygous load for In and St= 0.264931023 2.92573869E-03

Inbreeding loads= 1.09451369E-03 1.14471139E-03

Selection coefficients against In and St homokaryotypes

0.220683753 -1.27981901E-02

Contributions to mean A2 freqs= 0.188687384 2.80112308E-02

Contributions to mean diversities= 6.46441069E-04 2.37779086E-03

Zone 3: moderate selection zone

Lower and upper bounds of St metapopn gamma for zone 3

499.999969 5000.00000

Probability of zone 3= 0.339170814

Contributions to mean load statistics over zone 3

Loads within In and St= 1.43128308E-03 2.34772975E-04

Load between In and St= 1.83767042E-04

Homozygous load for In and St= 2.59628915E-03 1.07661041E-03

Inbreeding loads= 1.16500829E-03 8.41837085E-04

Selection coefficients for In and St homokaryotypes

1.24675035E-03 5.10215759E-05

Contributions to mean A2 freqs= 4.06202344E-05 1.74012966E-05

Contributions to mean diversities= 3.88201333E-05 3.10472933E-05

Zone 4: strong selection zone

Upper limit to scaled gamma for St metapopn= 2.50000000

Lower limit to gamma for St metapopn= 5000.00000

Upper limit to gamma for St metapopn= 7500.00049

Probability of zone 4= 2.19503045E-02

Contributions to mean load statistics over zone 4

Loads within In and St= 1.25854538E-04 3.40290462E-05

Load between In and St= 1.93103133E-05

Homozygous load for In and St= 2.17127221E-04 1.68897372E-04

Inbreeding loads= 9.12726900E-05 1.34868445E-04

Selection coefficients for In and St homokaryotypes

1.06513500E-04 1.47223473E-05

Contributions to mean A2 freqs= 1.31559182E-06 1.31559182E-06

Contributions to mean diversities= 7.14153936E-09 3.18832072E-08

Mean load statistics over all zones

Loads within In and St= 0.265521407 2.17649480E-03

Load between In and St= 1.47830220E-02

Homozygous load for In and St= 0.267872453 4.29924577E-03

Inbreeding loads= 2.35094712E-03 2.12275446E-03

Selection coefficients for In and St homokaryotypes

0.221774042 -1.26863718E-02

Mean frequencies of A2 in In and St= 0.228665173 6.79658130E-02

Ratio of these= 3.36441469

Mean diversities at selected sites in In and St= 7.91021215E-04 3.33607849E-03

Ratio of these= 0.237111092

Mean diversities at neutral sites in In and St= 1.58884050E-03 1.39304632E-02

pi-n/pi-s for In and St= 0.497860670 0.239480793

Ratio of these= 2.07891679

**Neutral Fst for whole population= 0.15**

Scaled migration rate for whole population= 5.66666651

Zone 1: quasi-neutral zone 1

Upper bound scaled selection coefficient for neutrality in St metapopulation= 0.250000000

Probability of zone 1= 6.65597618E-02

Integral of selection coefficient over zone 1= 2.13332591E-04

Mean load statistics for zone 1

Mean q1 and q2= 0.600000024

F1 and F2= 0.996697068 0.971039176

Diversities= 1.58540718E-03 1.39011955E-02

Contributions to loads within In and St= 1.27847365E-04 1.26665036E-04

Contributions to load between In and St = 8.19197157E-05

Contributions to homozygous loads for In and St= 1.27999563E-04 1.27999563E-04

Contributions to inbreeding loads for In and St= 1.52193181E-07 1.33451636E-06

Contributions to selection coefficients for In and St homokaryotypes

4.59551811E-05 4.47630882E-05

Contributions to mean A2 freqs= 3.99358571E-02 3.99358571E-02

Contributions to mean diversities= 1.05524327E-04 9.25260247E-04

Zone 2: quasi-neutral zone 2

Lower and upper bounds of St metapopn gamma for zone 2

0.250000000 499.999969

Probability of zone 2= 0.560461998

Coefficients for bivariate distribution of q1 and q2 in metapopulation

G1= 1.76470590 G2= 0.196078435

G3= -2.52941179 G4= 0.607843161

a1= 2.08823532E-02 a2= 0.187941164

b11= -1.13823544E-02 b12= 8.09999928E-02 b22= 0.221558809

Contributions to mean load statistics for zone 2

Loads within In and St= 0.278209805 1.79501495E-03

Load between In and St= 1.51930945E-02

Homozygous load for In and St= 0.279073477 2.68053380E-03

Inbreeding loads= 8.63889407E-04 8.85517744E-04

Selection coefficients against In and St homokaryotypes

0.231270969 -1.34882927E-02

Contributions to mean A2 freqs= 0.197923049 2.81747263E-02

Contributions to mean diversities= 5.78282226E-04 2.19426886E-03

Zone 3: moderate selection zone

Lower and upper bounds of St metapopn gamma for zone 3

499.999969 5000.00000

Probability of zone 3= 0.339170814

Contributions to mean load statistics over zone 3

Loads within In and St= 1.53660029E-03 2.24845775E-04

Load between In and St= 1.63241799E-04

Homozygous load for In and St= 2.44317274E-03 8.19940411E-04

Inbreeding loads= 9.06573376E-04 5.95093297E-04

Selection coefficients for In and St homokaryotypes

1.37239695E-03 6.16312027E-05

Contributions to mean A2 freqs= 3.81461941E-05 1.30871085E-05

Contributions to mean diversities= 2.97201095E-05 2.20149814E-05

Zone 4: strong selection zone

Upper limit to scaled gamma for St metapopn= 2.50000000

Lower limit to gamma for St metapopn= 5000.00000

Upper limit to gamma for St metapopn= 7500.00049

Probability of zone 4= 2.19503045E-02

Contributions to mean load statistics over zone 4

Loads within In and St= 1.41980316E-04 4.13295056E-05

Load between In and St= 1.93101114E-05

Homozygous load for In and St= 2.13195264E-04 1.72822794E-04

Inbreeding loads= 7.12151523E-05 1.31493376E-04

Selection coefficients for In and St homokaryotypes

1.22666359E-04 2.19941139E-05

Contributions to mean A2 freqs= 1.29149464E-06 1.29149464E-06

Contributions to mean diversities= 7.75935938E-09 3.32146364E-08

Mean load statistics over all zones

Loads within In and St= 0.280016243 2.18785531E-03

Load between In and St= 1.54575668E-02

Homozygous load for In and St= 0.281857848 3.80129647E-03

Inbreeding loads= 1.84183009E-03 1.61343906E-03

Selection coefficients for In and St homokaryotypes

0.232455373 -1.33581161E-02

Mean frequencies of A2 in In and St= 0.237898350 6.81249648E-02

Ratio of these= 3.49208760

Mean diversities at selected sites in In and St= 7.13534420E-04 3.14157759E-03

Ratio of these= 0.227126151

Mean diversities at neutral sites in In and St= 1.58540718E-03 1.39011955E-02

pi-n/pi-s for In and St= 0.450063825 0.225993335

Ratio of these= 1.99149156

**Neutral Fst for whole population= 0.2**

Scaled migration rate for whole population= 4.00000000

Zone 1: quasi-neutral zone 1

Upper bound scaled selection coefficient for neutrality in St metapopulation= 0.250000000

Probability of zone 1= 6.65597618E-02

Integral of selection coefficient over zone 1= 2.13332591E-04

Mean load statistics for zone 1

Mean q1 and q2= 0.600000024

F1 and F2= 0.996705115 0.971107543

Diversities= 1.58154487E-03 1.38683794E-02

Contributions to loads within In and St= 1.27847728E-04 1.26668194E-04

Contributions to load between In and St = 8.19197157E-05

Contributions to homozygous loads for In and St= 1.27999563E-04 1.27999563E-04

Contributions to inbreeding loads for In and St= 1.51837142E-07 1.33136291E-06

Contributions to selection coefficients for In and St homokaryotypes

4.59551811E-05 4.47630882E-05

Contributions to mean A2 freqs= 3.99358571E-02 3.99358571E-02

Contributions to mean diversities= 1.05267252E-04 9.23076004E-04

Zone 2: quasi-neutral zone 2

Lower and upper bounds of St metapopn gamma for zone 2

0.250000000 499.999969

Probability of zone 2= 0.560461998

Coefficients for bivariate distribution of q1 and q2 in metapopulation

G1= 2.50000000 G2= 0.277777791

G3= -4.00000000 G4= 0.444444418

a1= 2.75000017E-02 a2= 0.247500002

b11= -1.80000011E-02 b12= 8.09999928E-02 b22= 0.161999986

Contributions to mean load statistics for zone 2

Loads within In and St= 0.291161209 1.81315688E-03

Load between In and St= 1.58293080E-02

Homozygous load for In and St= 0.291850656 2.51605990E-03

Inbreeding loads= 6.89510896E-04 7.02903140E-04

Selection coefficients against In and St homokaryotypes

0.240679920 -1.41148567E-02

Contributions to mean A2 freqs= 0.206740692 2.83778552E-02

Contributions to mean diversities= 5.18195273E-04 2.03381036E-03

Zone 3: moderate selection zone

Lower and upper bounds of St metapopn gamma for zone 3

499.999969 5000.00000

Probability of zone 3= 0.339170814

Contributions to mean load statistics over zone 3

Loads within In and St= 1.59517303E-03 2.20695874E-04

Load between In and St= 1.50801206E-04

Homozygous load for In and St= 2.34262762E-03 6.72070135E-04

Inbreeding loads= 7.47456215E-04 4.51374828E-04

Selection coefficients for In and St homokaryotypes

1.44332647E-03 6.99162483E-05

Contributions to mean A2 freqs= 3.64811422E-05 1.05172703E-05

Contributions to mean diversities= 2.40582976E-05 1.66588234E-05

Zone 4: strong selection zone

Upper limit to scaled gamma for St metapopn= 2.50000000

Lower limit to gamma for St metapopn= 5000.00000

Upper limit to gamma for St metapopn= 7500.00049

Probability of zone 4= 2.19503045E-02

Contributions to mean load statistics over zone 4

Loads within In and St= 1.50885913E-04 4.63690340E-05

Load between In and St= 1.89601724E-05

Homozygous load for In and St= 2.08364203E-04 1.70660205E-04

Inbreeding loads= 5.74781116E-05 1.24291211E-04

Selection coefficients for In and St homokaryotypes

1.31905079E-04 2.74181366E-05

Contributions to mean A2 freqs= 1.26193197E-06 1.26193197E-06

Contributions to mean diversities= 8.12156031E-09 3.32351213E-08

Mean load statistics over all zones

Loads within In and St= 0.293035120 2.20688991E-03

Load between In and St= 1.60809886E-02

Homozygous load for In and St= 0.294529676 3.48678976E-03

Inbreeding loads= 1.49459706E-03 1.27990055E-03

Selection coefficients for In and St homokaryotypes

0.241910756 -1.39707327E-02

Mean frequencies of A2 in In and St= 0.246714294 6.83254898E-02

Ratio of these= 3.61086750

Mean diversities at selected sites in In and St= 6.47528970E-04 2.97357840E-03

Ratio of these= 0.217760846

Mean diversities at neutral sites in In and St= 1.58154487E-03 1.38683794E-02

pi-n/pi-s for In and St= 0.409428149 0.214414269

Ratio of these= 1.90951920

**Neutral Fst for whole population= 0.25**

Scaled migration rate for whole population= 3.00000000

Zone 1: quasi-neutral zone 1

Upper bound scaled selection coefficient for neutrality in St metapopulation= 0.250000000

Probability of zone 1= 6.65597618E-02

Integral of selection coefficient over zone 1= 2.13332591E-04

Mean load statistics for zone 1

Mean q1 and q2= 0.600000024

F1 and F2= 0.996714115 0.971184611

Diversities= 1.57722470E-03 1.38313863E-02

Contributions to loads within In and St= 1.27848136E-04 1.26671759E-04

Contributions to load between In and St = 8.19197157E-05

Contributions to homozygous loads for In and St= 1.27999563E-04 1.27999563E-04

Contributions to inbreeding loads for In and St= 1.51417524E-07 1.32780247E-06

Contributions to selection coefficients for In and St homokaryotypes

4.59551811E-05 4.47630882E-05

Contributions to mean A2 freqs= 3.99358571E-02 3.99358571E-02

Contributions to mean diversities= 1.04979699E-04 9.20613762E-04

Zone 2: quasi-neutral zone 2

Lower and upper bounds of St metapopn gamma for zone 2

0.250000000 499.999969

Probability of zone 2= 0.560461998

Coefficients for bivariate distribution of q1 and q2 in metapopulation

G1= 3.33333325 G2= 0.370370388

G3= -5.66666651 G4= 0.259259224

a1= 3.50000001E-02 a2= 0.314999998

b11= -2.55000014E-02 b12= 8.09999928E-02 b22= 9.44999754E-02

Contributions to mean load statistics for zone 2

Loads within In and St= 0.302106738 1.83545984E-03

Load between In and St= 1.63746569E-02

Homozygous load for In and St= 0.302661031 2.40248349E-03

Inbreeding loads= 5.54622326E-04 5.67021896E-04

Selection coefficients against In and St homokaryotypes

0.248536110 -1.46454573E-02

Contributions to mean A2 freqs= 0.214812145 2.86240485E-02

Contributions to mean diversities= 4.64905723E-04 1.88864395E-03

Zone 3: moderate selection zone

Lower and upper bounds of St metapopn gamma for zone 3

499.999969 5000.00000

Probability of zone 3= 0.339170814

Contributions to mean load statistics over zone 3

Loads within In and St= 1.63113244E-03 2.23232608E-04

Load between In and St= 1.42632838E-04

Homozygous load for In and St= 2.27117655E-03 5.80400520E-04

Inbreeding loads= 6.40044920E-04 3.57167824E-04

Selection coefficients for In and St homokaryotypes

1.48737431E-03 8.05854797E-05

Contributions to mean A2 freqs= 3.52840470E-05 8.86054931E-06

Contributions to mean diversities= 2.02206738E-05 1.31936540E-05

Zone 4: strong selection zone

Upper limit to scaled gamma for St metapopn= 2.50000000

Lower limit to gamma for St metapopn= 5000.00000

Upper limit to gamma for St metapopn= 7500.00049

Probability of zone 4= 2.19503045E-02

Contributions to mean load statistics over zone 4

Loads within In and St= 1.58709809E-04 4.98264890E-05

Load between In and St= 1.86097368E-05

Homozygous load for In and St= 2.06237848E-04 1.65783291E-04

Inbreeding loads= 4.75279776E-05 1.15956725E-04

Selection coefficients for In and St homokaryotypes

1.40070915E-04 3.12328339E-05

Contributions to mean A2 freqs= 1.24872770E-06 1.24872770E-06

Contributions to mean diversities= 8.44682368E-09 3.26150804E-08

Mean load statistics over all zones

Loads within In and St= 0.304024428 2.23519071E-03

Load between In and St= 1.66178178E-02

Homozygous load for In and St= 0.305266440 3.27666686E-03

Inbreeding loads= 1.24234660E-03 1.04147429E-03

Selection coefficients for In and St homokaryotypes

0.249793410 -1.44865513E-02

Mean frequencies of A2 in In and St= 0.254784524 6.85700178E-02

Ratio of these= 3.71568418

Mean diversities at selected sites in In and St= 5.90114563E-04 2.82248389E-03

Ratio of these= 0.209076330

Mean diversities at neutral sites in In and St= 1.57722470E-03 1.38313863E-02

pi-n/pi-s for In and St= 0.374147415 0.204063699

Ratio of these= 1.83348346

**h=0.25**

Neutral Fst for whole population= 5.00000007E-02

Scaled migration rate for whole population= 19.0000000

Zone 1: quasi-neutral zone 1

Upper bound scaled selection coefficient for neutrality in St metapopulation= 0.250000000

Probability of zone 1= 6.65597618E-02

Integral of selection coefficient over zone 1= 2.13332591E-04

Mean load statistics for zone 1

Mean q1 and q2= 0.600000024

F1 and F2= 0.996683598 0.970923305

Diversities= 1.59187312E-03 1.39568131E-02

Contributions to loads within In and St= 1.27914653E-04 1.27255204E-04

Contributions to load between In and St = 1.02399645E-04

Contributions to homozygous loads for In and St= 1.27999563E-04 1.27999563E-04

Contributions to inbreeding loads for In and St= 8.49021475E-08 7.44359284E-07

Contributions to selection coefficients for In and St homokaryotypes

2.55107880E-05 2.48551369E-05

Contributions to mean A2 freqs= 3.99358571E-02 3.99358571E-02

Contributions to mean diversities= 1.05954699E-04 9.28962138E-04

Zone 2: quasi-neutral zone 2

Lower and upper bounds of St metapopn gamma for zone 2

0.250000000 499.999969

Probability of zone 2= 0.560461998

Coefficients for bivariate distribution of q1 and q2 in metapopulation

G1= 0.526315808 G2= 5.84795326E-02

G3= -5.26316166E-02 G4= 0.883040905

a1= 2.76315790E-02 a2= 0.248684227

b11= -1.31579058E-04 b12= 4.49999981E-02 b22= 0.178815767

Contributions to mean load statistics for zone 2

Loads within In and St= 5.90346903E-02 1.98704959E-03

Load between In and St= 1.59571450E-02

Homozygous load for In and St= 5.93853891E-02 2.39157630E-03

Inbreeding loads= 3.50770191E-04 4.04525752E-04

Selection coefficients against In and St homokaryotypes

4.21628952E-02 -1.40681267E-02

Contributions to mean A2 freqs= 0.114401005 2.83715427E-02

Contributions to mean diversities= 5.44274342E-04 2.10491754E-03

Zone 3: moderate selection zone

Lower and upper bounds of St metapopn gamma for zone 3

499.999969 5000.00000

Probability of zone 3= 0.339170814

Contributions to mean load statistics over zone 3

Loads within In and St= 6.12784352E-04 2.14864107E-04

Load between In and St= 3.34279990E-04

Homozygous load for In and St= 9.18715552E-04 4.18364158E-04

Inbreeding loads= 3.05932626E-04 2.03500080E-04

Selection coefficients for In and St homokaryotypes

2.78472900E-04 -1.19447708E-04

Contributions to mean A2 freqs= 1.43828702E-05 7.16180330E-06

Contributions to mean diversities= 1.88891554E-05 1.35515647E-05

Zone 4: strong selection zone

Upper limit to scaled gamma for St metapopn= 2.50000000

Lower limit to gamma for St metapopn= 5000.00000

Upper limit to gamma for St metapopn= 7500.00049

Probability of zone 4= 2.19503045E-02

Contributions to mean load statistics over zone 4

Loads within In and St= 2.90477619E-05 1.52113562E-05

Load between In and St= 1.79106555E-05

Homozygous load for In and St= 4.29423126E-05 2.86996292E-05

Inbreeding loads= 1.38945097E-05 1.34882966E-05

Selection coefficients for In and St homokaryotypes

1.11460686E-05 -2.74181366E-06

Contributions to mean A2 freqs= 2.60144276E-07 2.60144276E-07

Contributions to mean diversities= 1.19978882E-09 5.28039790E-09

Mean load statistics over all zones

Loads within In and St= 5.98044358E-02 2.34438013E-03

Load between In and St= 1.64117347E-02

Homozygous load for In and St= 6.04750477E-02 2.96663959E-03

Inbreeding loads= 6.70682231E-04 6.22258463E-04

Selection coefficients for In and St homokaryotypes

4.24647331E-02 -1.41667128E-02

Mean frequencies of A2 in In and St= 0.154351503 6.83148205E-02

Ratio of these= 2.25941467

Mean diversities at selected sites in In and St= 6.69119414E-04 3.04743671E-03

Ratio of these= 0.219567940

Mean diversities at neutral sites in In and St= 1.59187312E-03 1.39568131E-02

pi-n/pi-s for In and St= 0.420334637 0.218347609

Ratio of these= 1.92507088

**Neutral Fst for whole population= 0.1**

Scaled migration rate for whole population= 9.00000000

Zone 1: quasi-neutral zone 1

Upper bound scaled selection coefficient for neutrality in St metapopulation= 0.250000000

Probability of zone 1= 6.65597618E-02

Integral of selection coefficient over zone 1= 2.13332591E-04

Mean load statistics for zone 1

Mean q1 and q2= 0.600000024

F1 and F2= 0.996689916 0.970978200

Diversities= 1.58884050E-03 1.39304632E-02

Contributions to loads within In and St= 1.27914827E-04 1.27256601E-04

Contributions to load between In and St = 1.02399645E-04

Contributions to homozygous loads for In and St= 1.27999563E-04 1.27999563E-04

Contributions to inbreeding loads for In and St= 8.47368469E-08 7.42960594E-07

Contributions to selection coefficients for In and St homokaryotypes

2.55107880E-05 2.48551369E-05

Contributions to mean A2 freqs= 3.99358571E-02 3.99358571E-02

Contributions to mean diversities= 1.05752843E-04 9.27208341E-04

Zone 2: quasi-neutral zone 2

Lower and upper bounds of St metapopn gamma for zone 2

0.250000000 499.999969

Probability of zone 2= 0.560461998

Coefficients for bivariate distribution of q1 and q2 in metapopulation

G1= 1.11111104 G2= 0.123456798

G3= -1.22222209 G4= 0.753086388

a1= 3.05555556E-02 a2= 0.274999976

b11= -3.05555551E-03 b12= 4.49999981E-02 b22= 0.152499989

Contributions to mean load statistics for zone 2

Loads within In and St= 5.96722551E-02 2.00874102E-03

Load between In and St= 1.61151905E-02

Homozygous load for In and St= 6.00014180E-02 2.37986422E-03

Inbreeding loads= 3.29092960E-04 3.71122820E-04

Selection coefficients against In and St homokaryotypes

4.26220894E-02 -1.42064095E-02

Contributions to mean A2 freqs= 0.115158208 2.85090432E-02

Contributions to mean diversities= 5.30313118E-04 2.04032729E-03

Zone 3: moderate selection zone

Lower and upper bounds of St metapopn gamma for zone 3

499.999969 5000.00000

Probability of zone 3= 0.339170814

Contributions to mean load statistics over zone 3

Loads within In and St= 8.18531960E-04 2.21893555E-04

Load between In and St= 3.73218121E-04

Homozygous load for In and St= 1.09959545E-03 3.93232360E-04

Inbreeding loads= 2.81062763E-04 1.71339067E-04

Selection coefficients for In and St homokaryotypes

4.45187092E-04 -1.51276588E-04

Contributions to mean A2 freqs= 1.74646757E-05 6.65179323E-06

Contributions to mean diversities= 1.73475491E-05 1.18873031E-05

Zone 4: strong selection zone

Upper limit to scaled gamma for St metapopn= 2.50000000

Lower limit to gamma for St metapopn= 5000.00000

Upper limit to gamma for St metapopn= 7500.00049

Probability of zone 4= 2.19503045E-02

Contributions to mean load statistics over zone 4

Loads within In and St= 3.51804447E-05 1.88266476E-05

Load between In and St= 2.00675131E-05

Homozygous load for In and St= 4.62256030E-05 3.40435799E-05

Inbreeding loads= 1.10451410E-05 1.52169496E-05

Selection coefficients for In and St homokaryotypes

1.51395798E-05 -1.19209290E-06

Contributions to mean A2 freqs= 2.79861780E-07 2.79861780E-07

Contributions to mean diversities= 1.52289015E-09 6.42683196E-09

Mean load statistics over all zones

Loads within In and St= 6.06538840E-02 2.37671798E-03

Load between In and St= 1.66108757E-02

Homozygous load for In and St= 6.12752400E-02 2.93513970E-03

Inbreeding loads= 6.21285639E-04 5.58421772E-04

Selection coefficients for In and St homokaryotypes

4.30871844E-02 -1.43359900E-02

Mean frequencies of A2 in In and St= 0.155111805 6.84518367E-02

Ratio of these= 2.26599908

Mean diversities at selected sites in In and St= 6.53415045E-04 2.97942944E-03

Ratio of these= 0.219308779

Mean diversities at neutral sites in In and St= 1.58884050E-03 1.39304632E-02

pi-n/pi-s for In and St= 0.411252767 0.213878706

Ratio of these= 1.92283177

**Neutral Fst for whole population= 0.15**

Scaled migration rate for whole population= 5.66666651

Zone 1: quasi-neutral zone 1

Upper bound scaled selection coefficient for neutrality in St metapopulation= 0.250000000

Probability of zone 1= 6.65597618E-02

Integral of selection coefficient over zone 1= 2.13332591E-04

Mean load statistics for zone 1

Mean q1 and q2= 0.600000024

F1 and F2= 0.996697068 0.971039176

Diversities= 1.58540718E-03 1.39011955E-02

Contributions to loads within In and St= 1.27915002E-04 1.27258158E-04

Contributions to load between In and St = 1.02399645E-04

Contributions to homozygous loads for In and St= 1.27999563E-04 1.27999563E-04

Contributions to inbreeding loads for In and St= 8.45588275E-08 7.41396548E-07

Contributions to selection coefficients for In and St homokaryotypes

2.55107880E-05 2.48551369E-05

Contributions to mean A2 freqs= 3.99358571E-02 3.99358571E-02

Contributions to mean diversities= 1.05524327E-04 9.25260247E-04

Zone 2: quasi-neutral zone 2

Lower and upper bounds of St metapopn gamma for zone 2

0.250000000 499.999969

Probability of zone 2= 0.560461998

Coefficients for bivariate distribution of q1 and q2 in metapopulation

G1= 1.76470590 G2= 0.196078435

G3= -2.52941179 G4= 0.607843161

a1= 3.38235311E-02 a2= 0.304411739

b11= -6.32352987E-03 b12= 4.49999981E-02 b22= 0.123088233

Contributions to mean load statistics for zone 2

Loads within In and St= 6.03216849E-02 2.03074818E-03

Load between In and St= 1.62775163E-02

Homozygous load for In and St= 6.06294014E-02 2.37003318E-03

Inbreeding loads= 3.07686074E-04 3.39286547E-04

Selection coefficients against In and St homokaryotypes

4.30883169E-02 -1.43487453E-02

Contributions to mean A2 freqs= 0.115986191 2.86622811E-02

Contributions to mean diversities= 5.15754393E-04 1.97415985E-03

Zone 3: moderate selection zone

Lower and upper bounds of St metapopn gamma for zone 3

499.999969 5000.00000

Probability of zone 3= 0.339170814

Contributions to mean load statistics over zone 3

Loads within In and St= 9.84689803E-04 2.24231044E-04

Load between In and St= 4.05910105E-04

Homozygous load for In and St= 1.24566082E-03 3.77931749E-04

Inbreeding loads= 2.60970002E-04 1.53700705E-04

Selection coefficients for In and St homokaryotypes

5.78641891E-04 -1.81674957E-04

Contributions to mean A2 freqs= 2.00250015E-05 6.29020587E-06

Contributions to mean diversities= 1.60449836E-05 1.06071166E-05

Zone 4: strong selection zone

Upper limit to scaled gamma for St metapopn= 2.50000000

Lower limit to gamma for St metapopn= 5000.00000

Upper limit to gamma for St metapopn= 7500.00049

Probability of zone 4= 2.19503045E-02

Contributions to mean load statistics over zone 4

Loads within In and St= 3.89370616E-05 2.07785943E-05

Load between In and St= 2.11101378E-05

Homozygous load for In and St= 4.81292118E-05 3.63102481E-05

Inbreeding loads= 9.19213562E-06 1.55316757E-05

Selection coefficients for In and St homokaryotypes

1.78217888E-05 -3.57627869E-07

Contributions to mean A2 freqs= 2.91181465E-07 2.91181465E-07

Contributions to mean diversities= 1.75276271E-09 6.97636571E-09

Mean load statistics over all zones

Loads within In and St= 6.14732280E-02 2.40301597E-03

Load between In and St= 1.68069340E-02

Homozygous load for In and St= 6.20511957E-02 2.91227479E-03

Inbreeding loads= 5.77932806E-04 5.09260339E-04

Selection coefficients for In and St homokaryotypes

4.36834693E-02 -1.45081282E-02

Mean frequencies of A2 in In and St= 0.155942380 6.86047152E-02

Ratio of these= 2.27305627

Mean diversities at selected sites in In and St= 6.37325458E-04 2.91003403E-03

Ratio of these= 0.219009623

Mean diversities at neutral sites in In and St= 1.58540718E-03 1.39011955E-02

pi-n/pi-s for In and St= 0.401994795 0.209336966

Ratio of these= 1.92032397

**Neutral** **Fst for whole population= 0.2**

Scaled migration rate for whole population= 4.00000000

Zone 1: quasi-neutral zone 1

Upper bound scaled selection coefficient for neutrality in St metapopulation= 0.250000000

Probability of zone 1= 6.65597618E-02

Integral of selection coefficient over zone 1= 2.13332591E-04

Mean load statistics for zone 1

Mean q1 and q2= 0.600000024

F1 and F2= 0.996705115 0.971107543

Diversities= 1.58154487E-03 1.38683794E-02

Contributions to loads within In and St= 1.27915206E-04 1.27259918E-04

Contributions to load between In and St = 1.02399645E-04

Contributions to homozygous loads for In and St= 1.27999563E-04 1.27999563E-04

Contributions to inbreeding loads for In and St= 8.43553778E-08 7.39641791E-07

Contributions to selection coefficients for In and St homokaryotypes

2.55107880E-05 2.48551369E-05

Contributions to mean A2 freqs= 3.99358571E-02 3.99358571E-02

Contributions to mean diversities= 1.05267252E-04 9.23076004E-04

Zone 2: quasi-neutral zone 2

Lower and upper bounds of St metapopn gamma for zone 2

0.250000000 499.999969

Probability of zone 2= 0.560461998

Coefficients for bivariate distribution of q1 and q2 in metapopulation

G1= 2.50000000 G2= 0.277777791

G3= -4.00000000 G4= 0.444444418

a1= 3.75000015E-02 a2= 0.337499976

b11= -1.00000007E-02 b12= 4.49999981E-02 b22= 8.99999887E-02

Contributions to mean load statistics for zone 2

Loads within In and St= 6.09860271E-02 2.05331319E-03

Load between In and St= 1.64450873E-02

Homozygous load for In and St= 6.12725727E-02 2.36219983E-03

Inbreeding loads= 2.86546565E-04 3.08886694E-04

Selection coefficients against In and St homokaryotypes

4.35635448E-02 -1.44958496E-02

Contributions to mean A2 freqs= 0.116895556 2.88341939E-02

Contributions to mean diversities= 5.00534428E-04 1.90613465E-03

Zone 3: moderate selection zone

Lower and upper bounds of St metapopn gamma for zone 3

499.999969 5000.00000

Probability of zone 3= 0.339170814

Contributions to mean load statistics over zone 3

Loads within In and St= 1.12174428E-03 2.30511418E-04

Load between In and St= 4.33569920E-04

Homozygous load for In and St= 1.36596477E-03 3.68262292E-04

Inbreeding loads= 2.44220806E-04 1.37750714E-04

Selection coefficients for In and St homokaryotypes

6.87956810E-04 -2.03132629E-04

Contributions to mean A2 freqs= 2.21803257E-05 6.01177635E-06

Contributions to mean diversities= 1.49250600E-05 9.55233463E-06

Zone 4: strong selection zone

Upper limit to scaled gamma for St metapopn= 2.50000000

Lower limit to gamma for St metapopn= 5000.00000

Upper limit to gamma for St metapopn= 7500.00049

Probability of zone 4= 2.19503045E-02

Contributions to mean load statistics over zone 4

Loads within In and St= 4.20837277E-05 2.21509090E-05

Load between In and St= 2.19032718E-05

Homozygous load for In and St= 5.00063870E-05 3.76056378E-05

Inbreeding loads= 7.92264746E-06 1.54547488E-05

Selection coefficients for In and St homokaryotypes

2.02059746E-05 2.38418579E-07

Contributions to mean A2 freqs= 3.02320871E-07 3.02320871E-07

Contributions to mean diversities= 1.94846517E-09 7.31835170E-09

Mean load statistics over all zones

Loads within In and St= 6.22777715E-02 2.43323529E-03

Load between In and St= 1.70029588E-02

Homozygous load for In and St= 6.28165454E-02 2.89606745E-03

Inbreeding loads= 5.38774359E-04 4.62831784E-04

Selection coefficients for In and St homokaryotypes

4.42652106E-02 -1.46763325E-02

Mean frequencies of A2 in In and St= 0.156853884 6.87763691E-02

Ratio of these= 2.28063631

Mean diversities at selected sites in In and St= 6.20728708E-04 2.83877016E-03

Ratio of these= 0.218661129

Mean diversities at neutral sites in In and St= 1.58154487E-03 1.38683794E-02

pi-n/pi-s for In and St= 0.392482519 0.204693720

Ratio of these= 1.91741359

**Neutral Fst for whole population= 0.25**

Scaled migration rate for whole population= 3.00000000

Zone 1: quasi-neutral zone 1

Upper bound scaled selection coefficient for neutrality in St metapopulation= 0.250000000

Probability of zone 1= 6.65597618E-02

Integral of selection coefficient over zone 1= 2.13332591E-04

Mean load statistics for zone 1

Mean q1 and q2= 0.600000024

F1 and F2= 0.996714115 0.971184611

Diversities= 1.57722470E-03 1.38313863E-02

Contributions to loads within In and St= 1.27915438E-04 1.27261883E-04

Contributions to load between In and St = 1.02399645E-04

Contributions to homozygous loads for In and St= 1.27999563E-04 1.27999563E-04

Contributions to inbreeding loads for In and St= 8.41264978E-08 7.37670860E-07

Contributions to selection coefficients for In and St homokaryotypes

2.55107880E-05 2.48551369E-05

Contributions to mean A2 freqs= 3.99358571E-02 3.99358571E-02

Contributions to mean diversities= 1.04979699E-04 9.20613762E-04

Zone 2: quasi-neutral zone 2

Lower and upper bounds of St metapopn gamma for zone 2

0.250000000 499.999969

Probability of zone 2= 0.560461998

Coefficients for bivariate distribution of q1 and q2 in metapopulation

G1= 3.33333325 G2= 0.370370388

G3= -5.66666651 G4= 0.259259224

a1= 4.16666679E-02 a2= 0.374999970

b11= -1.41666671E-02 b12= 4.49999981E-02 b22= 5.24999909E-02

Contributions to mean load statistics for zone 2

Loads within In and St= 6.16687797E-02 2.07675830E-03

Load between In and St= 1.66190751E-02

Homozygous load for In and St= 6.19344525E-02 2.35656556E-03

Inbreeding loads= 2.65666866E-04 2.79810251E-04

Selection coefficients against In and St homokaryotypes

4.40500379E-02 -1.46485567E-02

Contributions to mean A2 freqs= 0.117899507 2.90286411E-02

Contributions to mean diversities= 4.84578864E-04 1.83595635E-03

Zone 3: moderate selection zone

Lower and upper bounds of St metapopn gamma for zone 3

499.999969 5000.00000

Probability of zone 3= 0.339170814

Contributions to mean load statistics over zone 3

Loads within In and St= 1.23658497E-03 2.38341279E-04

Load between In and St= 4.57451766E-04

Homozygous load for In and St= 1.46655797E-03 3.63196974E-04

Inbreeding loads= 2.29971731E-04 1.24855622E-04

Selection coefficients for In and St homokaryotypes

7.78853893E-04 -2.19106674E-04

Contributions to mean A2 freqs= 2.40130666E-05 5.80222695E-06

Contributions to mean diversities= 1.39497060E-05 8.67066319E-06

Zone 4: strong selection zone

Upper limit to scaled gamma for St metapopn= 2.50000000

Lower limit to gamma for St metapopn= 5000.00000

Upper limit to gamma for St metapopn= 7500.00049

Probability of zone 4= 2.19503045E-02

Contributions to mean load statistics over zone 4

Loads within In and St= 4.57032475E-05 2.32621751E-05

Load between In and St= 2.28036806E-05

Homozygous load for In and St= 5.27072516E-05 3.85063577E-05

Inbreeding loads= 7.00398550E-06 1.52441717E-05

Selection coefficients for In and St homokaryotypes

2.28881836E-05 4.76837158E-07

Contributions to mean A2 freqs= 3.18426402E-07 3.18426402E-07

Contributions to mean diversities= 2.15613349E-09 7.56708562E-09

Mean load statistics over all zones

Loads within In and St= 6.30789846E-02 2.46562366E-03

Load between In and St= 1.72017310E-02

Homozygous load for In and St= 6.35817200E-02 2.88626854E-03

Inbreeding loads= 5.02726762E-04 4.20647732E-04

Selection coefficients for In and St homokaryotypes

4.48408127E-02 -1.48452520E-02

Mean frequencies of A2 in In and St= 0.157859683 6.89706206E-02

Ratio of these= 2.28879595

Mean diversities at selected sites in In and St= 6.03510416E-04 2.76524830E-03

Ratio of these= 0.218248188

Mean diversities at neutral sites in In and St= 1.57722470E-03 1.38313863E-02

pi-n/pi-s for In and St= 0.382640719 0.199925601

Ratio of these= 1.91391551

**h=0.45**

**Neutral Fst for whole population= 0.05**

Scaled migration rate for whole population= 19.0000000

Zone 1: quasi-neutral zone 1

Upper bound scaled selection coefficient for neutrality in St metapopulation= 0.250000000

Probability of zone 1= 6.65597618E-02

Integral of selection coefficient over zone 1= 2.13332591E-04

Mean load statistics for zone 1

Mean q1 and q2= 0.600000024

F1 and F2= 0.996683598 0.970923305

Diversities= 1.59187312E-03 1.39568131E-02

Contributions to loads within In and St= 1.27982566E-04 1.27850682E-04

Contributions to load between In and St = 1.22879574E-04

Contributions to homozygous loads for In and St= 1.27999563E-04 1.27999563E-04

Contributions to inbreeding loads for In and St= 1.69880590E-08 1.48874406E-07

Contributions to selection coefficients for In and St homokaryotypes

5.12599945E-06 4.94718552E-06

Contributions to mean A2 freqs= 3.99358571E-02 3.99358571E-02

Contributions to mean diversities= 1.05954699E-04 9.28962138E-04

Zone 2: quasi-neutral zone 2

Lower and upper bounds of St metapopn gamma for zone 2

0.250000000 499.999969

Probability of zone 2= 0.560461998

Coefficients for bivariate distribution of q1 and q2 in metapopulation

G1= 0.526315808 G2= 5.84795326E-02

G3= -5.26316166E-02 G4= 0.883040905

a1= 4.55263145E-02 a2= 0.409736782

b11= -2.63158163E-05 b12= 9.00000241E-03 b22= 3.57631631E-02

Contributions to mean load statistics for zone 2

Loads within In and St= 3.08037046E-02 2.08963687E-03

Load between In and St= 1.49468193E-02

Homozygous load for In and St= 3.08494922E-02 2.14035227E-03

Inbreeding loads= 4.57528040E-05 5.07139739E-05

Selection coefficients against In and St homokaryotypes

1.57318115E-02 -1.29401684E-02

Contributions to mean A2 freqs= 9.27644670E-02 2.89922152E-02

Contributions to mean diversities= 4.63030738E-04 1.77304400E-03

Zone 3: moderate selection zone

Lower and upper bounds of St metapopn gamma for zone 3

499.999969 5000.00000

Probability of zone 3= 0.339170814

Contributions to mean load statistics over zone 3

Loads within In and St= 5.06766257E-04 1.98421985E-04

Load between In and St= 3.43153282E-04

Homozygous load for In and St= 5.43164264E-04 2.19396956E-04

Inbreeding loads= 3.63979598E-05 2.09750233E-05

Selection coefficients for In and St homokaryotypes

1.63614750E-04 -1.44720078E-04

Contributions to mean A2 freqs= 8.55834969E-06 3.69976988E-06

Contributions to mean diversities= 1.13286014E-05 7.00459941E-06

Zone 4: strong selection zone

Upper limit to scaled gamma for St metapopn= 2.50000000

Lower limit to gamma for St metapopn= 5000.00000

Upper limit to gamma for St metapopn= 7500.00049

Probability of zone 4= 2.19503045E-02

Contributions to mean load statistics over zone 4

Loads within In and St= 2.26632892E-05 1.42670642E-05

Load between In and St= 1.80003663E-05

Homozygous load for In and St= 2.42493334E-05 1.57514860E-05

Inbreeding loads= 1.58601858E-06 1.48441450E-06

Selection coefficients for In and St homokaryotypes

4.64916229E-06 -3.69548798E-06

Contributions to mean A2 freqs= 1.46874996E-07 1.46874996E-07

Contributions to mean diversities= 6.77600587E-10 2.89853408E-09

Mean load statistics over all zones

Loads within In and St= 3.14611197E-02 2.43017660E-03

Load between In and St= 1.54308528E-02

Homozygous load for In and St= 3.15449052E-02 2.50350032E-03

Inbreeding loads= 8.37537737E-05 7.33222842E-05

Selection coefficients for In and St homokaryotypes

1.59024596E-02 -1.30856037E-02

Mean frequencies of A2 in In and St= 0.132709026 6.89319223E-02

Ratio of these= 1.92521870

Mean diversities at selected sites in In and St= 5.80314721E-04 2.70901364E-03

Ratio of these= 0.214216247

Mean diversities at neutral sites in In and St= 1.59187312E-03 1.39568131E-02

pi-n/pi-s for In and St= 0.364548355 0.194099724

Ratio of these= 1.87814975

**Neutral Fst for whole population= 0.1**

Scaled migration rate for whole population= 9.00000000

Zone 1: quasi-neutral zone 1

Upper bound scaled selection coefficient for neutrality in St metapopulation= 0.250000000

Probability of zone 1= 6.65597618E-02

Integral of selection coefficient over zone 1= 2.13332591E-04

Mean load statistics for zone 1

Mean q1 and q2= 0.600000024

F1 and F2= 0.996689916 0.970978200

Diversities= 1.58884050E-03 1.39304632E-02

Contributions to loads within In and St= 1.27982610E-04 1.27850959E-04

Contributions to load between In and St = 1.22879574E-04

Contributions to homozygous loads for In and St= 1.27999563E-04 1.27999563E-04

Contributions to inbreeding loads for In and St= 1.69499117E-08 1.48594651E-07

Contributions to selection coefficients for In and St homokaryotypes

5.12599945E-06 4.94718552E-06

Contributions to mean A2 freqs= 3.99358571E-02 3.99358571E-02

Contributions to mean diversities= 1.05752843E-04 9.27208341E-04

Zone 2: quasi-neutral zone 2

Lower and upper bounds of St metapopn gamma for zone 2

0.250000000 499.999969

Probability of zone 2= 0.560461998

Coefficients for bivariate distribution of q1 and q2 in metapopulation

G1= 1.11111104 G2= 0.123456798

G3= -1.22222209 G4= 0.753086388

a1= 4.61111069E-02 a2= 0.414999962

b11= -6.11111231E-04 b12= 9.00000241E-03 b22= 3.05000041E-02

Contributions to mean load statistics for zone 2

Loads within In and St= 3.11407577E-02 2.12183385E-03

Load between In and St= 1.51134217E-02

Homozygous load for In and St= 3.11863776E-02 2.17202003E-03

Inbreeding loads= 4.55613044E-05 5.01853974E-05

Selection coefficients against In and St homokaryotypes

1.58995986E-02 -1.30763054E-02

Contributions to mean A2 freqs= 9.29514766E-02 2.90695652E-02

Contributions to mean diversities= 4.62458964E-04 1.76476687E-03

Zone 3: moderate selection zone

Lower and upper bounds of St metapopn gamma for zone 3

499.999969 5000.00000

Probability of zone 3= 0.339170814

Contributions to mean load statistics over zone 3

Loads within In and St= 6.59986108E-04 2.08951722E-04

Load between In and St= 4.16188414E-04

Homozygous load for In and St= 6.95719442E-04 2.29142388E-04

Inbreeding loads= 3.57332574E-05 2.01906332E-05

Selection coefficients for In and St homokaryotypes

2.43782997E-04 -2.07304955E-04

Contributions to mean A2 freqs= 1.11692643E-05 3.80915276E-06

Contributions to mean diversities= 1.11622767E-05 6.81779011E-06

Zone 4: strong selection zone

Upper limit to scaled gamma for St metapopn= 2.50000000

Lower limit to gamma for St metapopn= 5000.00000

Upper limit to gamma for St metapopn= 7500.00049

Probability of zone 4= 2.19503045E-02

Contributions to mean load statistics over zone 4

Loads within In and St= 2.57524080E-05 1.73046155E-05

Load between In and St= 2.07400535E-05

Homozygous load for In and St= 2.70729070E-05 1.90160772E-05

Inbreeding loads= 1.32050661E-06 1.71144654E-06

Selection coefficients for In and St homokaryotypes

5.00679016E-06 -3.45706940E-06

Contributions to mean A2 freqs= 1.63837896E-07 1.63837896E-07

Contributions to mean diversities= 8.91714869E-10 3.59016084E-09

Mean load statistics over all zones

Loads within In and St= 3.19544822E-02 2.47594132E-03

Load between In and St= 1.56732295E-02

Homozygous load for In and St= 3.20371687E-02 2.54817796E-03

Inbreeding loads= 8.26320174E-05 7.22360637E-05

Selection coefficients for In and St homokaryotypes

1.61494017E-02 -1.32848024E-02

Mean frequencies of A2 in In and St= 0.132898673 6.90093935E-02

Ratio of these= 1.92580557

Mean diversities at selected sites in In and St= 5.79374901E-04 2.69879657E-03

Ratio of these= 0.214678988

Mean diversities at neutral sites in In and St= 1.58884050E-03 1.39304632E-02

pi-n/pi-s for In and St= 0.364652663 0.193733439

Ratio of these= 1.88223910

**Neutral Fst for whole population= 0.15**

Scaled migration rate for whole population= 5.66666651

Zone 1: quasi-neutral zone 1

Upper bound scaled selection coefficient for neutrality in St metapopulation= 0.250000000

Probability of zone 1= 6.65597618E-02

Integral of selection coefficient over zone 1= 2.13332591E-04

Mean load statistics for zone 1

Mean q1 and q2= 0.600000024

F1 and F2= 0.996697068 0.971039176

Diversities= 1.58540718E-03 1.39011955E-02

Contributions to loads within In and St= 1.27982654E-04 1.27851279E-04

Contributions to load between In and St = 1.22879574E-04

Contributions to homozygous loads for In and St= 1.27999563E-04 1.27999563E-04

Contributions to inbreeding loads for In and St= 1.69117662E-08 1.48276769E-07

Contributions to selection coefficients for In and St homokaryotypes

5.12599945E-06 4.94718552E-06

Contributions to mean A2 freqs= 3.99358571E-02 3.99358571E-02

Contributions to mean diversities= 1.05524327E-04 9.25260247E-04

Zone 2: quasi-neutral zone 2

Lower and upper bounds of St metapopn gamma for zone 2

0.250000000 499.999969

Probability of zone 2= 0.560461998

Coefficients for bivariate distribution of q1 and q2 in metapopulation

G1= 1.76470590 G2= 0.196078435

G3= -2.52941179 G4= 0.607843161

a1= 4.67647016E-02 a2= 0.420882314

b11= -1.26470637E-03 b12= 9.00000241E-03 b22= 2.46176515E-02

Contributions to mean load statistics for zone 2

Loads within In and St= 3.15129645E-02 2.15699966E-03

Load between In and St= 1.52971996E-02

Homozygous load for In and St= 3.15582529E-02 2.20659887E-03

Inbreeding loads= 4.53394314E-05 4.95971180E-05

Selection coefficients against In and St homokaryotypes

1.60849690E-02 -1.32268667E-02

Contributions to mean A2 freqs= 9.31584463E-02 2.91553680E-02

Contributions to mean diversities= 4.61764605E-04 1.75554643E-03

Zone 3: moderate selection zone

Lower and upper bounds of St metapopn gamma for zone 3

499.999969 5000.00000

Probability of zone 3= 0.339170814

Contributions to mean load statistics over zone 3

Loads within In and St= 8.02148075E-04 2.21554263E-04

Load between In and St= 4.85323806E-04

Homozygous load for In and St= 8.37319298E-04 2.41175279E-04

Inbreeding loads= 3.51721646E-05 1.96210058E-05

Selection coefficients for In and St homokaryotypes

3.16798687E-04 -2.63810158E-04

Contributions to mean A2 freqs= 1.36825429E-05 3.95445568E-06

Contributions to mean diversities= 1.10069777E-05 6.68592520E-06

Zone 4: strong selection zone

Upper limit to scaled gamma for St metapopn= 2.50000000

Lower limit to gamma for St metapopn= 5000.00000

Upper limit to gamma for St metapopn= 7500.00049

Probability of zone 4= 2.19503045E-02

Contributions to mean load statistics over zone 4

Loads within In and St= 2.81416924E-05 1.89439215E-05

Load between In and St= 2.25126951E-05

Homozygous load for In and St= 2.92908517E-05 2.07373305E-05

Inbreeding loads= 1.14916259E-06 1.79339861E-06

Selection coefficients for In and St homokaryotypes

5.60283661E-06 -3.57627869E-06

Contributions to mean A2 freqs= 1.77103558E-07 1.77103558E-07

Contributions to mean diversities= 1.06616027E-09 3.98430267E-09

Mean load statistics over all zones

Loads within In and St= 3.24712358E-02 2.52534915E-03

Load between In and St= 1.59279145E-02

Homozygous load for In and St= 3.25528644E-02 2.59651104E-03

Inbreeding loads= 8.16776737E-05 7.11598041E-05

Selection coefficients for In and St homokaryotypes

1.64072514E-02 -1.34928226E-02

Mean frequencies of A2 in In and St= 0.133108169 6.90953583E-02

Ratio of these= 1.92644155

Mean diversities at selected sites in In and St= 5.78296953E-04 2.68749660E-03

Ratio of these= 0.215180531

Mean diversities at neutral sites in In and St= 1.58540718E-03 1.39011955E-02

pi-n/pi-s for In and St= 0.364762425 0.193328455

Ratio of these= 1.88674986

**Neutral Fst for whole population= 0.2**

Scaled migration rate for whole population= 4.00000000

Zone 1: quasi-neutral zone 1

Upper bound scaled selection coefficient for neutrality in St metapopulation= 0.250000000

Probability of zone 1= 6.65597618E-02

Integral of selection coefficient over zone 1= 2.13332591E-04

Mean load statistics for zone 1

Mean q1 and q2= 0.600000024

F1 and F2= 0.996705115 0.971107543

Diversities= 1.58154487E-03 1.38683794E-02

Contributions to loads within In and St= 1.27982683E-04 1.27851643E-04

Contributions to load between In and St = 1.22879574E-04

Contributions to homozygous loads for In and St= 1.27999563E-04 1.27999563E-04

Contributions to inbreeding loads for In and St= 1.68736189E-08 1.47920730E-07

Contributions to selection coefficients for In and St homokaryotypes

5.12599945E-06 4.94718552E-06

Contributions to mean A2 freqs= 3.99358571E-02 3.99358571E-02

Contributions to mean diversities= 1.05267252E-04 9.23076004E-04

Zone 2: quasi-neutral zone 2

Lower and upper bounds of St metapopn gamma for zone 2

0.250000000 499.999969

Probability of zone 2= 0.560461998

Coefficients for bivariate distribution of q1 and q2 in metapopulation

G1= 2.50000000 G2= 0.277777791

G3= -4.00000000 G4= 0.444444418

a1= 4.74999957E-02 a2= 0.427499950

b11= -2.00000056E-03 b12= 9.00000241E-03 b22= 1.80000030E-02

Contributions to mean load statistics for zone 2

Loads within In and St= 3.19256186E-02 2.19552987E-03

Load between In and St= 1.55007914E-02

Homozygous load for In and St= 3.19707282E-02 2.24447041E-03

Inbreeding loads= 4.50804073E-05 4.89390695E-05

Selection coefficients against In and St homokaryotypes

1.62906647E-02 -1.33941174E-02

Contributions to mean A2 freqs= 9.33891311E-02 2.92510763E-02

Contributions to mean diversities= 4.60919924E-04 1.74521236E-03

Zone 3: moderate selection zone

Lower and upper bounds of St metapopn gamma for zone 3

499.999969 5000.00000

Probability of zone 3= 0.339170814

Contributions to mean load statistics over zone 3

Loads within In and St= 9.35409393E-04 2.35093583E-04

Load between In and St= 5.50922297E-04

Homozygous load for In and St= 9.70081834E-04 2.54187122E-04

Inbreeding loads= 3.46716261E-05 1.90932933E-05

Selection coefficients for In and St homokaryotypes

3.84390354E-04 -3.15904617E-04

Contributions to mean A2 freqs= 1.61142652E-05 4.11231440E-06

Contributions to mean diversities= 1.08588283E-05 6.55765734E-06

Zone 4: strong selection zone

Upper limit to scaled gamma for St metapopn= 2.50000000

Lower limit to gamma for St metapopn= 5000.00000

Upper limit to gamma for St metapopn= 7500.00049

Probability of zone 4= 2.19503045E-02

Contributions to mean load statistics over zone 4

Loads within In and St= 3.05401954E-05 2.01618041E-05

Load between In and St= 2.41059170E-05

Homozygous load for In and St= 3.15725665E-05 2.19961166E-05

Inbreeding loads= 1.03239802E-06 1.83430984E-06

Selection coefficients for In and St homokaryotypes

6.43730164E-06 -3.93390656E-06

Contributions to mean A2 freqs= 1.90743677E-07 1.90743677E-07

Contributions to mean diversities= 1.22932498E-09 4.28049018E-09

Mean load statistics over all zones

Loads within In and St= 3.30195501E-02 2.57863686E-03

Load between In and St= 1.61987003E-02

Homozygous load for In and St= 3.31003815E-02 2.64865323E-03

Inbreeding loads= 8.08013065E-05 7.00145974E-05

Selection coefficients for In and St homokaryotypes

1.66801810E-02 -1.37132406E-02

Mean frequencies of A2 in In and St= 0.133341283 6.91912398E-02

Ratio of these= 1.92714107

Mean diversities at selected sites in In and St= 5.77047234E-04 2.67485017E-03

Ratio of these= 0.215730682

Mean diversities at neutral sites in In and St= 1.58154487E-03 1.38683794E-02

pi-n/pi-s for In and St= 0.364863008 0.192874029

Ratio of these= 1.89171660

**Neutral Fst for whole population= 0.25**

Scaled migration rate for whole population= 3.00000000

Zone 1: quasi-neutral zone 1

Upper bound scaled selection coefficient for neutrality in St metapopulation= 0.250000000

Probability of zone 1= 6.65597618E-02

Integral of selection coefficient over zone 1= 2.13332591E-04

Mean load statistics for zone 1

Mean q1 and q2= 0.600000024

F1 and F2= 0.996714115 0.971184611

Diversities= 1.57722470E-03 1.38313863E-02

Contributions to loads within In and St= 1.27982741E-04 1.27852021E-04

Contributions to load between In and St = 1.22879574E-04

Contributions to homozygous loads for In and St= 1.27999563E-04 1.27999563E-04

Contributions to inbreeding loads for In and St= 1.68227565E-08 1.47539268E-07

Contributions to selection coefficients for In and St homokaryotypes

5.12599945E-06 4.94718552E-06

Contributions to mean A2 freqs= 3.99358571E-02 3.99358571E-02

Contributions to mean diversities= 1.04979699E-04 9.20613762E-04

Zone 2: quasi-neutral zone 2

Lower and upper bounds of St metapopn gamma for zone 2

0.250000000 499.999969

Probability of zone 2= 0.560461998

Coefficients for bivariate distribution of q1 and q2 in metapopulation

G1= 3.33333325 G2= 0.370370388

G3= -5.66666651 G4= 0.259259224

a1= 4.83333282E-02 a2= 0.434999973

b11= -2.83333403E-03 b12= 9.00000241E-03 b22= 1.05000008E-02

Contributions to mean load statistics for zone 2

Loads within In and St= 3.23855542E-02 2.23790528E-03

Load between In and St= 1.57273896E-02

Homozygous load for In and St= 3.24302949E-02 2.28610379E-03

Inbreeding loads= 4.47752209E-05 4.81984862E-05

Selection coefficients against In and St homokaryotypes

1.65202022E-02 -1.35809183E-02

Contributions to mean A2 freqs= 9.36474577E-02 2.93585826E-02

Contributions to mean diversities= 4.59882111E-04 1.73354743E-03

Zone 3: moderate selection zone

Lower and upper bounds of St metapopn gamma for zone 3

499.999969 5000.00000

Probability of zone 3= 0.339170814

Contributions to mean load statistics over zone 3

Loads within In and St= 1.06135278E-03 2.49753386E-04

Load between In and St= 6.13716082E-04

Homozygous load for In and St= 1.09556539E-03 2.68244446E-04

Inbreeding loads= 3.42133681E-05 1.84911933E-05

Selection coefficients for In and St homokaryotypes

4.47511673E-04 -3.64065170E-04

Contributions to mean A2 freqs= 1.84765759E-05 4.28280509E-06

Contributions to mean diversities= 1.07160076E-05 6.42726081E-06

Zone 4: strong selection zone

Upper limit to scaled gamma for St metapopn= 2.50000000

Lower limit to gamma for St metapopn= 5000.00000

Upper limit to gamma for St metapopn= 7500.00049

Probability of zone 4= 2.19503045E-02

Contributions to mean load statistics over zone 4

Loads within In and St= 3.34819742E-05 2.12157156E-05

Load between In and St= 2.58773889E-05

Homozygous load for In and St= 3.44301479E-05 2.30750938E-05

Inbreeding loads= 9.48172556E-07 1.85938177E-06

Selection coefficients for In and St homokaryotypes

7.62939453E-06 -4.64916229E-06

Contributions to mean A2 freqs= 2.07856374E-07 2.07856374E-07

Contributions to mean diversities= 1.40731771E-09 4.53459181E-09

Mean load statistics over all zones

Loads within In and St= 3.36083733E-02 2.63672648E-03

Load between In and St= 1.64898615E-02

Homozygous load for In and St= 3.36882919E-02 2.70542293E-03

Inbreeding loads= 7.99535846E-05 6.86965941E-05

Selection coefficients for In and St homokaryotypes

1.69728398E-02 -1.39495134E-02

Mean frequencies of A2 in In and St= 0.133601993 6.92989305E-02

Ratio of these= 1.92790842

Mean diversities at selected sites in In and St= 5.75579237E-04 2.66059302E-03

Ratio of these= 0.216334939

Mean diversities at neutral sites in In and St= 1.57722470E-03 1.38313863E-02

pi-n/pi-s for In and St= 0.364931673 0.192359105

Ratio of these= 1.89713752

**Inversion frequency= 0.5**

**h= 0.05**

**Neutral Fst for whole population= 0.05**

Scaled migration rate for whole population= 19.0000000

Zone 1: quasi-neutral zone 1

Upper bound scaled selection coefficient for neutrality in St metapopulation= 0.250000000

Probability of zone 1= 7.93938339E-02

Integral of selection coefficient over zone 1= 4.58041381E-04

Mean load statistics for zone 1

Mean q1 and q2= 0.600000024

F1 and F2= 0.983634770 0.983634770

Diversities= 7.85531010E-03 7.85531010E-03

Contributions to loads within In and St= 2.73205718E-04 2.73205718E-04

Contributions to load between In and St = 1.75887893E-04

Contributions to homozygous loads for In and St= 2.74824852E-04 2.74824852E-04

Contributions to inbreeding loads for In and St= 1.61910918E-06 1.61910918E-06

Contributions to selection coefficients for In and St homokaryotypes

9.73343849E-05 9.73343849E-05

Contributions to mean A2 freqs= 4.76363041E-02 4.76363041E-02

Contributions to mean diversities= 6.23663189E-04 6.23663189E-04

Zone 2: quasi-neutral zone 2

Lower and upper bounds of St metapopn gamma for zone 2

0.250000000 499.999969

Probability of zone 2= 0.647554874

Coefficients for bivariate distribution of q1 and q2 in metapopulation

G1= 0.105263159 G2= 0.105263159

G3= 0.789473653 G4= 0.789473653

a1= 4.86842096E-02 a2= 4.86842096E-02

b11= 8.88157859E-02 b12= 0.224999994 b22= 8.88157859E-02

Contributions to mean load statistics for zone 2

Loads within In and St= 7.02768704E-03 7.02737598E-03

Load between In and St= 2.27557379E-03

Homozygous load for In and St= 9.14861076E-03 9.14829783E-03

Inbreeding loads= 2.12093000E-03 2.12092767E-03

Selection coefficients against In and St homokaryotypes

4.74083424E-03 4.74053621E-03

Contributions to mean A2 freqs= 4.23684753E-02 4.23674919E-02

Contributions to mean diversities= 2.03582388E-03 2.03581247E-03

Zone 3: moderate selection zone

Lower and upper bounds of St metapopn gamma for zone 3

499.999969 999.999939

Probability of zone 3= 0.116252482

Contributions to mean load statistics over zone 3

Loads within In and St= 1.28839616E-04 1.28802523E-04

Load between In and St= 6.94253249E-05

Homozygous load for In and St= 6.93681184E-04 6.93739217E-04

Inbreeding loads= 5.64841146E-04 5.64936374E-04

Selection coefficients for In and St homokaryotypes

5.94258308E-05 5.93662262E-05

Contributions to mean A2 freqs= 1.00901516E-05 1.00913121E-05

Contributions to mean diversities= 1.82565145E-05 1.82587009E-05

Zone 4: strong selection zone

Upper limit to scaled gamma for St metapopn= 2.50000000

Lower limit to gamma for St metapopn= 999.999939

Upper limit to gamma for St metapopn= 4166.66699

Probability of zone 4= 0.144941688

Contributions to mean load statistics over zone 4

Loads within In and St= 2.32143793E-04 2.32143808E-04

Load between In and St= 1.18087533E-04

Homozygous load for In and St= 1.17977324E-03 1.17977324E-03

Inbreeding loads= 9.47630091E-04 9.47630033E-04

Selection coefficients for In and St homokaryotypes

1.14023685E-04 1.14023685E-04

Contributions to mean A2 freqs= 1.44621363E-05 1.44621363E-05

Contributions to mean diversities= 2.57586350E-07 2.57586322E-07

Mean load statistics over all zones

Loads within In and St= 7.66187627E-03 7.66152795E-03

Load between In and St= 2.63897469E-03

Homozygous load for In and St= 1.12968907E-02 1.12966355E-02

Inbreeding loads= 3.63502023E-03 3.63511313E-03

Selection coefficients for In and St homokaryotypes

5.01030684E-03 5.00994921E-03

Mean frequencies of A2 in In and St= 9.00293291E-02 9.00283456E-02

Ratio of these= 1.00001097

Mean diversities at selected sites in In and St= 2.67800107E-03 2.67799199E-03

Ratio of these= 1.00000334

Mean diversities at neutral sites in In and St= 7.85531010E-03 7.85531010E-03

pi-n/pi-s for In and St= 0.340916008 0.340914875

Ratio of these= 1.00000334

**Neutral Fst for whole population= 0.1**

Scaled migration rate for whole population= 9.00000000

Zone 1: quasi-neutral zone 1

Upper bound scaled selection coefficient for neutrality in St metapopulation= 0.250000000

Probability of zone 1= 7.93938339E-02

Integral of selection coefficient over zone 1= 4.58041381E-04

Mean load statistics for zone 1

Mean q1 and q2= 0.600000024

F1 and F2= 0.983666062 0.983666062

Diversities= 7.84028973E-03 7.84028973E-03

Contributions to loads within In and St= 2.73208803E-04 2.73208803E-04

Contributions to load between In and St = 1.75887893E-04

Contributions to homozygous loads for In and St= 2.74824852E-04 2.74824852E-04

Contributions to inbreeding loads for In and St= 1.61602406E-06 1.61602406E-06

Contributions to selection coefficients for In and St homokaryotypes

9.73343849E-05 9.73343849E-05

Contributions to mean A2 freqs= 4.76363041E-02 4.76363041E-02

Contributions to mean diversities= 6.22470689E-04 6.22470689E-04

Zone 2: quasi-neutral zone 2

Lower and upper bounds of St metapopn gamma for zone 2

0.250000000 499.999969

Probability of zone 2= 0.647554874

Coefficients for bivariate distribution of q1 and q2 in metapopulation

G1= 0.222222224 G2= 0.222222224

G3= 0.555555582 G4= 0.555555582

a1= 7.50000030E-02 a2= 7.50000030E-02

b11= 6.25000000E-02 b12= 0.224999994 b22= 6.25000000E-02

Contributions to mean load statistics for zone 2

Loads within In and St= 7.78123550E-03 7.78049836E-03

Load between In and St= 2.46909563E-03

Homozygous load for In and St= 9.33658704E-03 9.33584478E-03

Inbreeding loads= 1.55535014E-03 1.55534584E-03

Selection coefficients against In and St homokaryotypes

5.29807806E-03 5.29730320E-03

Contributions to mean A2 freqs= 4.43239026E-02 4.43218239E-02

Contributions to mean diversities= 1.84316293E-03 1.84313767E-03

Zone 3: moderate selection zone

Lower and upper bounds of St metapopn gamma for zone 3

499.999969 999.999939

Probability of zone 3= 0.116252482

Contributions to mean load statistics over zone 3

Loads within In and St= 1.29861335E-04 1.29866938E-04

Load between In and St= 4.96946413E-05

Homozygous load for In and St= 4.96653258E-04 4.96682420E-04

Inbreeding loads= 3.66792054E-04 3.66815599E-04

Selection coefficients for In and St homokaryotypes

8.01682472E-05 8.01682472E-05

Contributions to mean A2 freqs= 7.22004597E-06 7.22062805E-06

Contributions to mean diversities= 1.18475318E-05 1.18483758E-05

Zone 4: strong selection zone

Upper limit to scaled gamma for St metapopn= 2.50000000

Lower limit to gamma for St metapopn= 999.999939

Upper limit to gamma for St metapopn= 4166.66699

Probability of zone 4= 0.144941688

Contributions to mean load statistics over zone 4

Loads within In and St= 3.48215312E-04 3.48215341E-04

Load between In and St= 1.27889129E-04

Homozygous load for In and St= 1.27764035E-03 1.27764035E-03

Inbreeding loads= 9.29425005E-04 9.29425063E-04

Selection coefficients for In and St homokaryotypes

2.20298767E-04 2.20298767E-04

Contributions to mean A2 freqs= 1.56403494E-05 1.56403494E-05

Contributions to mean diversities= 2.97246686E-07 2.97246658E-07

Mean load statistics over all zones

Loads within In and St= 8.53252131E-03 8.53178930E-03

Load between In and St= 2.82256748E-03

Homozygous load for In and St= 1.13857053E-02 1.13849929E-02

Inbreeding loads= 2.85318331E-03 2.85320263E-03

Selection coefficients for In and St homokaryotypes

5.69367409E-03 5.69295883E-03

Mean frequencies of A2 in In and St= 9.19830650E-02 9.19809863E-02

Ratio of these= 1.00002265

Mean diversities at selected sites in In and St= 2.47777859E-03 2.47775391E-03

Ratio of these= 1.00001001

Mean diversities at neutral sites in In and St= 7.84028973E-03 7.84028973E-03

pi-n/pi-s for In and St= 0.316031516 0.316028357

Ratio of these= 1.00001001

**Neutral Fst for whole population= 0.15**

Scaled migration rate for whole population= 5.66666651

Zone 1: quasi-neutral zone 1

Upper bound scaled selection coefficient for neutrality in St metapopulation= 0.250000000

Probability of zone 1= 7.93938339E-02

Integral of selection coefficient over zone 1= 4.58041381E-04

Mean load statistics for zone 1

Mean q1 and q2= 0.600000024

F1 and F2= 0.983700812 0.983700812

Diversities= 7.82360975E-03 7.82360975E-03

Contributions to loads within In and St= 2.73212267E-04 2.73212267E-04

Contributions to load between In and St = 1.75887893E-04

Contributions to homozygous loads for In and St= 2.74824852E-04 2.74824852E-04

Contributions to inbreeding loads for In and St= 1.61258413E-06 1.61258413E-06

Contributions to selection coefficients for In and St homokaryotypes

9.73343849E-05 9.73343849E-05

Contributions to mean A2 freqs= 4.76363041E-02 4.76363041E-02

Contributions to mean diversities= 6.21146348E-04 6.21146348E-04

Zone 2: quasi-neutral zone 2

Lower and upper bounds of St metapopn gamma for zone 2

0.250000000 499.999969

Probability of zone 2= 0.647554874

Coefficients for bivariate distribution of q1 and q2 in metapopulation

G1= 0.352941185 G2= 0.352941185

G3= 0.294117630 G4= 0.294117630

a1= 0.104411766 a2= 0.104411766

b11= 3.30882333E-02 b12= 0.224999994 b22= 3.30882333E-02

Contributions to mean load statistics for zone 2

Loads within In and St= 8.72820150E-03 8.72602221E-03

Load between In and St= 2.76600290E-03

Homozygous load for In and St= 9.92292352E-03 9.92073212E-03

Inbreeding loads= 1.19472749E-03 1.19471678E-03

Selection coefficients against In and St homokaryotypes

5.94443083E-03 5.94228506E-03

Contributions to mean A2 freqs= 4.67186831E-02 4.67133708E-02

Contributions to mean diversities= 1.68555474E-03 1.68550585E-03

Zone 3: moderate selection zone

Lower and upper bounds of St metapopn gamma for zone 3

499.999969 999.999939

Probability of zone 3= 0.116252482

Contributions to mean load statistics over zone 3

Loads within In and St= 1.30380897E-04 1.30390821E-04

Load between In and St= 3.95515999E-05

Homozygous load for In and St= 3.95331066E-04 3.95349372E-04

Inbreeding loads= 2.64950155E-04 2.64958566E-04

Selection coefficients for In and St homokaryotypes

9.08374786E-05 9.08374786E-05

Contributions to mean A2 freqs= 5.74081241E-06 5.74117848E-06

Contributions to mean diversities= 8.54739392E-06 8.54768405E-06

Zone 4: strong selection zone

Upper limit to scaled gamma for St metapopn= 2.50000000

Lower limit to gamma for St metapopn= 999.999939

Upper limit to gamma for St metapopn= 4166.66699

Probability of zone 4= 0.144941688

Contributions to mean load statistics over zone 4

Loads within In and St= 4.41747339E-04 4.41747339E-04

Load between In and St= 1.30788554E-04

Homozygous load for In and St= 1.30659773E-03 1.30659773E-03

Inbreeding loads= 8.64851056E-04 8.64851056E-04

Selection coefficients for In and St homokaryotypes

3.10897827E-04 3.10897827E-04

Contributions to mean A2 freqs= 1.60101299E-05 1.60101299E-05

Contributions to mean diversities= 3.20486919E-07 3.20486919E-07

Mean load statistics over all zones

Loads within In and St= 9.57354158E-03 9.57137253E-03

Load between In and St= 3.11223115E-03

Homozygous load for In and St= 1.18996771E-02 1.18975043E-02

Inbreeding loads= 2.32614134E-03 2.32613902E-03

Selection coefficients for In and St homokaryotypes

6.44046068E-03 6.43831491E-03

Mean frequencies of A2 in In and St= 9.43767428E-02 9.43714306E-02

Ratio of these= 1.00005627

Mean diversities at selected sites in In and St= 2.31556897E-03 2.31552031E-03

Ratio of these= 1.00002098

Mean diversities at neutral sites in In and St= 7.82360975E-03 7.82360975E-03

pi-n/pi-s for In and St= 0.295971930 0.295965731

Ratio of these= 1.00002098

**Neutral Fst for whole population= 0.2**

Scaled migration rate for whole population= 4.00000000

Zone 1: quasi-neutral zone 1

Upper bound scaled selection coefficient for neutrality in St metapopulation= 0.250000000

Probability of zone 1= 7.93938339E-02

Integral of selection coefficient over zone 1= 4.58041381E-04

Mean load statistics for zone 1

Mean q1 and q2= 0.600000024

F1 and F2= 0.983739853 0.983739853

Diversities= 7.80487061E-03 7.80487061E-03

Contributions to loads within In and St= 2.73216108E-04 2.73216108E-04

Contributions to load between In and St = 1.75887893E-04

Contributions to homozygous loads for In and St= 2.74824852E-04 2.74824852E-04

Contributions to inbreeding loads for In and St= 1.60873458E-06 1.60873458E-06

Contributions to selection coefficients for In and St homokaryotypes

9.73343849E-05 9.73343849E-05

Contributions to mean A2 freqs= 4.76363041E-02 4.76363041E-02

Contributions to mean diversities= 6.19658618E-04 6.19658618E-04

Zone 2: quasi-neutral zone 2

Lower and upper bounds of St metapopn gamma for zone 2

0.250000000 499.999969

Probability of zone 2= 0.647554874

Coefficients for bivariate distribution of q1 and q2 in metapopulation

G1= 0.500000000 G2= 0.500000000

G3= 0.00000000 G4= 0.00000000

a1= 0.137500003 a2= 0.137500003

b11= 0.00000000 b12= 0.224999994 b22= 0.00000000

Contributions to mean load statistics for zone 2

Loads within In and St= 9.94067546E-03 9.93173290E-03

Load between In and St= 3.19830584E-03

Homozygous load for In and St= 1.08845234E-02 1.08755371E-02

Inbreeding loads= 9.43836581E-04 9.43794847E-04

Selection coefficients against In and St homokaryotypes

6.71970844E-03 6.71082735E-03

Contributions to mean A2 freqs= 4.96682711E-02 4.96502072E-02

Contributions to mean diversities= 1.54852786E-03 1.54835766E-03

Zone 3: moderate selection zone

Lower and upper bounds of St metapopn gamma for zone 3

499.999969 999.999939

Probability of zone 3= 0.116252482

Contributions to mean load statistics over zone 3

Loads within In and St= 1.31149936E-04 1.31172928E-04

Load between In and St= 3.34685428E-05

Homozygous load for In and St= 3.34552838E-04 3.34566954E-04

Inbreeding loads= 2.03402582E-04 2.03393749E-04

Selection coefficients for In and St homokaryotypes

9.76920128E-05 9.76920128E-05

Contributions to mean A2 freqs= 4.85172450E-06 4.85200826E-06

Contributions to mean diversities= 6.55177246E-06 6.55178474E-06

Zone 4: strong selection zone

Upper limit to scaled gamma for St metapopn= 2.50000000

Lower limit to gamma for St metapopn= 999.999939

Upper limit to gamma for St metapopn= 4166.66699

Probability of zone 4= 0.144941688

Contributions to mean load statistics over zone 4

Loads within In and St= 5.20906702E-04 5.20906702E-04

Load between In and St= 1.31518143E-04

Homozygous load for In and St= 1.31389603E-03 1.31389603E-03

Inbreeding loads= 7.92989973E-04 7.92989973E-04

Selection coefficients for In and St homokaryotypes

3.89337540E-04 3.89337540E-04

Contributions to mean A2 freqs= 1.61228454E-05 1.61228454E-05

Contributions to mean diversities= 3.36846171E-07 3.36846171E-07

Mean load statistics over all zones

Loads within In and St= 1.08659482E-02 1.08570289E-02

Load between In and St= 3.53918038E-03

Homozygous load for In and St= 1.28077967E-02 1.27988253E-02

Inbreeding loads= 1.94183784E-03 1.94178731E-03

Selection coefficients for In and St homokaryotypes

7.30001926E-03 7.29113817E-03

Mean frequencies of A2 in In and St= 9.73255485E-02 9.73074809E-02

Ratio of these= 1.00018573

Mean diversities at selected sites in In and St= 2.17507523E-03 2.17490504E-03

Ratio of these= 1.00007820

Mean diversities at neutral sites in In and St= 7.80487061E-03 7.80487061E-03

pi-n/pi-s for In and St= 0.278681785 0.278659970

Ratio of these= 1.00007832

**Neutral Fst for whole population= 0.25**

Scaled migration rate for whole population= 3.00000000

Zone 1: quasi-neutral zone 1

Upper bound scaled selection coefficient for neutrality in St metapopulation= 0.250000000

Probability of zone 1= 7.93938339E-02

Integral of selection coefficient over zone 1= 4.58041381E-04

Mean load statistics for zone 1

Mean q1 and q2= 0.600000024

F1 and F2= 0.983783841 0.983783841

Diversities= 7.78375613E-03 7.78375613E-03

Contributions to loads within In and St= 2.73220474E-04 2.73220474E-04

Contributions to load between In and St = 1.75887893E-04

Contributions to homozygous loads for In and St= 2.74824852E-04 2.74824852E-04

Contributions to inbreeding loads for In and St= 1.60436639E-06 1.60436639E-06

Contributions to selection coefficients for In and St homokaryotypes

9.73343849E-05 9.73343849E-05

Contributions to mean A2 freqs= 4.76363041E-02 4.76363041E-02

Contributions to mean diversities= 6.17982238E-04 6.17982238E-04

Zone 2: quasi-neutral zone 2

Lower and upper bounds of St metapopn gamma for zone 2

0.250000000 499.999969

Probability of zone 2= 0.647554874

Coefficients for bivariate distribution of q1 and q2 in metapopulation

G1= 0.666666687 G2= 0.666666687

G3= -0.333333373 G4= -0.333333373

a1= 0.175000012 a2= 0.175000012

b11= -3.75000052E-02 b12= 0.224999994 b22= -3.75000052E-02

Contributions to mean load statistics for zone 2

Loads within In and St= 1.15155950E-02 1.14576844E-02

Load between In and St= 3.82548827E-03

Homozygous load for In and St= 1.22735901E-02 1.22154644E-02

Inbreeding loads= 7.58009439E-04 7.57794303E-04

Selection coefficients against In and St homokaryotypes

7.66062737E-03 7.60316849E-03

Contributions to mean A2 freqs= 5.33365756E-02 5.32425605E-02

Contributions to mean diversities= 1.42350059E-03 1.42278196E-03

Zone 3: moderate selection zone

Lower and upper bounds of St metapopn gamma for zone 3

499.999969 999.999939

Probability of zone 3= 0.116252482

Contributions to mean load statistics over zone 3

Loads within In and St= 1.32491012E-04 1.32514237E-04

Load between In and St= 2.95072623E-05

Homozygous load for In and St= 2.94969039E-04 2.94982368E-04

Inbreeding loads= 1.62477998E-04 1.62468103E-04

Selection coefficients for In and St homokaryotypes

1.02996826E-04 1.02996826E-04

Contributions to mean A2 freqs= 4.27098485E-06 4.27125133E-06

Contributions to mean diversities= 5.22413075E-06 5.22398841E-06

Zone 4: strong selection zone

Upper limit to scaled gamma for St metapopn= 2.50000000

Lower limit to gamma for St metapopn= 999.999939

Upper limit to gamma for St metapopn= 4166.66699

Probability of zone 4= 0.144941688

Contributions to mean load statistics over zone 4

Loads within In and St= 5.89465839E-04 5.89465839E-04

Load between In and St= 1.31336710E-04

Homozygous load for In and St= 1.31210068E-03 1.31210033E-03

Inbreeding loads= 7.22635246E-04 7.22635072E-04

Selection coefficients for In and St homokaryotypes

4.58002090E-04 4.58002090E-04

Contributions to mean A2 freqs= 1.61247026E-05 1.61247026E-05

Contributions to mean diversities= 3.49236160E-07 3.49236160E-07

Mean load statistics over all zones

Loads within In and St= 1.25107719E-02 1.24528846E-02

Load between In and St= 4.16221982E-03

Homozygous load for In and St= 1.41554847E-02 1.40973721E-02

Inbreeding loads= 1.64472708E-03 1.64450193E-03

Selection coefficients for In and St homokaryotypes

8.31377506E-03 8.25637579E-03

Mean frequencies of A2 in In and St= 0.100993268 0.100899257

Ratio of these= 1.00093174

Mean diversities at selected sites in In and St= 2.04705610E-03 2.04633758E-03

Ratio of these= 1.00035107

Mean diversities at neutral sites in In and St= 7.78375613E-03 7.78375613E-03

pi-n/pi-s for In and St= 0.262990773 0.262898475

Ratio of these= 1.00035107

**h= 0.25**

**Neutral Fst for whole population= 0.05**

Scaled migration rate for whole population= 19.0000000

Zone 1: quasi-neutral zone 1

Upper bound scaled selection coefficient for neutrality in St metapopulation= 0.250000000

Probability of zone 1= 7.93938339E-02

Integral of selection coefficient over zone 1= 4.58041381E-04

Mean load statistics for zone 1

Mean q1 and q2= 0.600000024

F1 and F2= 0.983634770 0.983634770

Diversities= 7.85531010E-03 7.85531010E-03

Contributions to loads within In and St= 2.73925340E-04 2.73925340E-04

Contributions to load between In and St = 2.19859867E-04

Contributions to homozygous loads for In and St= 2.74824852E-04 2.74824852E-04

Contributions to inbreeding loads for In and St= 8.99499014E-07 8.99499014E-07

Contributions to selection coefficients for In and St homokaryotypes

5.40614128E-05 5.40614128E-05

Contributions to mean A2 freqs= 4.76363041E-02 4.76363041E-02

Contributions to mean diversities= 6.23663189E-04 6.23663189E-04

Zone 2: quasi-neutral zone 2

Lower and upper bounds of St metapopn gamma for zone 2

0.250000000 499.999969

Probability of zone 2= 0.647554874

Coefficients for bivariate distribution of q1 and q2 in metapopulation

G1= 0.105263159 G2= 0.105263159

G3= 0.789473653 G4= 0.789473653

a1= 0.138157889 a2= 0.138157889

b11= 4.93421033E-02 b12= 0.125000000 b22= 4.93421033E-02

Contributions to mean load statistics for zone 2

Loads within In and St= 5.14896307E-03 5.14896307E-03

Load between In and St= 3.48667149E-03

Homozygous load for In and St= 5.63930953E-03 5.63930953E-03

Inbreeding loads= 4.90354083E-04 4.90354083E-04

Selection coefficients against In and St homokaryotypes

1.66088343E-03 1.66088343E-03

Contributions to mean A2 freqs= 3.94972675E-02 3.94972675E-02

Contributions to mean diversities= 1.43266667E-03 1.43266656E-03

Zone 3: moderate selection zone

Lower and upper bounds of St metapopn gamma for zone 3

499.999969 999.999939

Probability of zone 3= 0.116252482

Contributions to mean load statistics over zone 3

Loads within In and St= 1.11995847E-04 1.11975874E-04

Load between In and St= 1.02401762E-04

Homozygous load for In and St= 2.04794065E-04 2.04802360E-04

Inbreeding loads= 9.27981673E-05 9.28263980E-05

Selection coefficients for In and St homokaryotypes

9.59634781E-06 9.59634781E-06

Contributions to mean A2 freqs= 2.99346061E-06 2.99362637E-06

Contributions to mean diversities= 5.42530734E-06 5.42560883E-06

Zone 4: strong selection zone

Upper limit to scaled gamma for St metapopn= 2.50000000

Lower limit to gamma for St metapopn= 999.999939

Upper limit to gamma for St metapopn= 4166.66699

Probability of zone 4= 0.144941688

Contributions to mean load statistics over zone 4

Loads within In and St= 1.25332459E-04 1.25332459E-04

Load between In and St= 1.13525944E-04

Homozygous load for In and St= 2.27047771E-04 2.27047727E-04

Inbreeding loads= 1.01715290E-04 1.01715297E-04

Selection coefficients for In and St homokaryotypes

1.18017197E-05 1.18017197E-05

Contributions to mean A2 freqs= 2.77107938E-06 2.77107938E-06

Contributions to mean diversities= 4.94290227E-08 4.94290227E-08

Mean load statistics over all zones

Loads within In and St= 5.66021632E-03 5.66019630E-03

Load between In and St= 3.92245920E-03

Homozygous load for In and St= 6.34597614E-03 6.34598453E-03

Inbreeding loads= 6.85766980E-04 6.85795210E-04

Selection coefficients for In and St homokaryotypes

1.73622370E-03 1.73622370E-03

Mean frequencies of A2 in In and St= 8.71393383E-02 8.71393383E-02

Ratio of these= 1.00000000

Mean diversities at selected sites in In and St= 2.06180476E-03 2.06180476E-03

Ratio of these= 1.00000000

Mean diversities at neutral sites in In and St= 7.85531010E-03 7.85531010E-03

pi-n/pi-s for In and St= 0.262472749 0.262472749

Ratio of these= 1.00000000

**Neutral Fst for whole population= 0.1**

Scaled migration rate for whole population= 9.00000000

Zone 1: quasi-neutral zone 1

Upper bound scaled selection coefficient for neutrality in St metapopulation= 0.250000000

Probability of zone 1= 7.93938339E-02

Integral of selection coefficient over zone 1= 4.58041381E-04

Mean load statistics for zone 1

Mean q1 and q2= 0.600000024

F1 and F2= 0.983666062 0.983666062

Diversities= 7.84028973E-03 7.84028973E-03

Contributions to loads within In and St= 2.73927028E-04 2.73927028E-04

Contributions to load between In and St = 2.19859867E-04

Contributions to homozygous loads for In and St= 2.74824852E-04 2.74824852E-04

Contributions to inbreeding loads for In and St= 8.97806331E-07 8.97806331E-07

Contributions to selection coefficients for In and St homokaryotypes

5.40614128E-05 5.40614128E-05

Contributions to mean A2 freqs= 4.76363041E-02 4.76363041E-02

Contributions to mean diversities= 6.22470689E-04 6.22470689E-04

Zone 2: quasi-neutral zone 2

Lower and upper bounds of St metapopn gamma for zone 2

0.250000000 499.999969

Probability of zone 2= 0.647554874

Coefficients for bivariate distribution of q1 and q2 in metapopulation

G1= 0.222222224 G2= 0.222222224

G3= 0.555555582 G4= 0.555555582

a1= 0.152777776 a2= 0.152777776

b11= 3.47222239E-02 b12= 0.125000000 b22= 3.47222239E-02

Contributions to mean load statistics for zone 2

Loads within In and St= 5.25076361E-03 5.25076361E-03

Load between In and St= 3.53639270E-03

Homozygous load for In and St= 5.69993211E-03 5.69993118E-03

Inbreeding loads= 4.49159212E-04 4.49159328E-04

Selection coefficients against In and St homokaryotypes

1.71291828E-03 1.71291828E-03

Contributions to mean A2 freqs= 3.99376638E-02 3.99376638E-02

Contributions to mean diversities= 1.38481671E-03 1.38481788E-03

Zone 3: moderate selection zone

Lower and upper bounds of St metapopn gamma for zone 3

499.999969 999.999939

Probability of zone 3= 0.116252482

Contributions to mean load statistics over zone 3

Loads within In and St= 1.17163116E-04 1.17172021E-04

Load between In and St= 9.95625160E-05

Homozygous load for In and St= 1.99116708E-04 1.99123475E-04

Inbreeding loads= 8.19537308E-05 8.19515553E-05

Selection coefficients for In and St homokaryotypes

1.75833702E-05 1.75833702E-05

Contributions to mean A2 freqs= 2.91074593E-06 2.91088077E-06

Contributions to mean diversities= 4.79095979E-06 4.79112714E-06

Zone 4: strong selection zone

Upper limit to scaled gamma for St metapopn= 2.50000000

Lower limit to gamma for St metapopn= 999.999939

Upper limit to gamma for St metapopn= 4166.66699

Probability of zone 4= 0.144941688

Contributions to mean load statistics over zone 4

Loads within In and St= 1.51373984E-04 1.51373984E-04

Load between In and St= 1.27750580E-04

Homozygous load for In and St= 2.55495455E-04 2.55495426E-04

Inbreeding loads= 1.04121580E-04 1.04121580E-04

Selection coefficients for In and St homokaryotypes

2.36034393E-05 2.36034393E-05

Contributions to mean A2 freqs= 3.11565668E-06 3.11565668E-06

Contributions to mean diversities= 5.92956013E-08 5.92955978E-08

Mean load statistics over all zones

Loads within In and St= 5.79322781E-03 5.79323666E-03

Load between In and St= 3.98356561E-03

Homozygous load for In and St= 6.42936910E-03 6.42937468E-03

Inbreeding loads= 6.36132318E-04 6.36130280E-04

Selection coefficients for In and St homokaryotypes

1.80804729E-03 1.80804729E-03

Mean frequencies of A2 in In and St= 8.75799954E-02 8.75799954E-02

Ratio of these= 1.00000000

Mean diversities at selected sites in In and St= 2.01213779E-03 2.01213919E-03

Ratio of these= 0.999999285

Mean diversities at neutral sites in In and St= 7.84028973E-03 7.84028973E-03

pi-n/pi-s for In and St= 0.256640732 0.256640911

Ratio of these= 0.999999285

**Neutral Fst for whole population= 0.15**

Scaled migration rate for whole population= 5.66666651

Zone 1: quasi-neutral zone 1

Upper bound scaled selection coefficient for neutrality in St metapopulation= 0.250000000

Probability of zone 1= 7.93938339E-02

Integral of selection coefficient over zone 1= 4.58041381E-04

Mean load statistics for zone 1

Mean q1 and q2= 0.600000024

F1 and F2= 0.983700812 0.983700812

Diversities= 7.82360975E-03 7.82360975E-03

Contributions to loads within In and St= 2.73928948E-04 2.73928948E-04

Contributions to load between In and St = 2.19859867E-04

Contributions to homozygous loads for In and St= 2.74824852E-04 2.74824852E-04

Contributions to inbreeding loads for In and St= 8.95895255E-07 8.95895255E-07

Contributions to selection coefficients for In and St homokaryotypes

5.40614128E-05 5.40614128E-05

Contributions to mean A2 freqs= 4.76363041E-02 4.76363041E-02

Contributions to mean diversities= 6.21146348E-04 6.21146348E-04

Zone 2: quasi-neutral zone 2

Lower and upper bounds of St metapopn gamma for zone 2

0.250000000 499.999969

Probability of zone 2= 0.647554874

Coefficients for bivariate distribution of q1 and q2 in metapopulation

G1= 0.352941185 G2= 0.352941185

G3= 0.294117630 G4= 0.294117630

a1= 0.169117644 a2= 0.169117644

b11= 1.83823518E-02 b12= 0.125000000 b22= 1.83823518E-02

Contributions to mean load statistics for zone 2

Loads within In and St= 5.35334833E-03 5.35334833E-03

Load between In and St= 3.59024666E-03

Homozygous load for In and St= 5.76327369E-03 5.76327369E-03

Inbreeding loads= 4.09923610E-04 4.09923639E-04

Selection coefficients against In and St homokaryotypes

1.76155567E-03 1.76155567E-03

Contributions to mean A2 freqs= 4.04275544E-02 4.04275544E-02

Contributions to mean diversities= 1.33593043E-03 1.33592996E-03

Zone 3: moderate selection zone

Lower and upper bounds of St metapopn gamma for zone 3

499.999969 999.999939

Probability of zone 3= 0.116252482

Contributions to mean load statistics over zone 3

Loads within In and St= 1.21851423E-04 1.21859513E-04

Load between In and St= 9.73595670E-05

Homozygous load for In and St= 1.94711320E-04 1.94717722E-04

Inbreeding loads= 7.28597079E-05 7.28579907E-05

Selection coefficients for In and St homokaryotypes

2.44975090E-05 2.44975090E-05

Contributions to mean A2 freqs= 2.84568637E-06 2.84581415E-06

Contributions to mean diversities= 4.25737062E-06 4.25743247E-06

Zone 4: strong selection zone

Upper limit to scaled gamma for St metapopn= 2.50000000

Lower limit to gamma for St metapopn= 999.999939

Upper limit to gamma for St metapopn= 4166.66699

Probability of zone 4= 0.144941688

Contributions to mean load statistics over zone 4

Loads within In and St= 1.69080842E-04 1.69080857E-04

Load between In and St= 1.34748509E-04

Homozygous load for In and St= 2.69491022E-04 2.69491022E-04

Inbreeding loads= 1.00410085E-04 1.00410085E-04

Selection coefficients for In and St homokaryotypes

3.43322754E-05 3.43322754E-05

Contributions to mean A2 freqs= 3.28367150E-06 3.28367150E-06

Contributions to mean diversities= 6.58064678E-08 6.58064749E-08

Mean load statistics over all zones

Loads within In and St= 5.91820944E-03 5.91821736E-03

Load between In and St= 4.04221471E-03

Homozygous load for In and St= 6.50230097E-03 6.50230749E-03

Inbreeding loads= 5.84089314E-04 5.84087626E-04

Selection coefficients for In and St homokaryotypes

1.87420845E-03 1.87426805E-03

Mean frequencies of A2 in In and St= 8.80699903E-02 8.80699903E-02

Ratio of these= 1.00000000

Mean diversities at selected sites in In and St= 1.96140003E-03 1.96139980E-03

Ratio of these= 1.00000012

Mean diversities at neutral sites in In and St= 7.82360975E-03 7.82360975E-03

pi-n/pi-s for In and St= 0.250702679 0.250702649

Ratio of these= 1.00000012

**Neutral Fst for whole population= 0.2**

Scaled migration rate for whole population= 4.00000000

Zone 1: quasi-neutral zone 1

Upper bound scaled selection coefficient for neutrality in St metapopulation= 0.250000000

Probability of zone 1= 7.93938339E-02

Integral of selection coefficient over zone 1= 4.58041381E-04

Mean load statistics for zone 1

Mean q1 and q2= 0.600000024

F1 and F2= 0.983739853 0.983739853

Diversities= 7.80487061E-03 7.80487061E-03

Contributions to loads within In and St= 2.73931102E-04 2.73931102E-04

Contributions to load between In and St = 2.19859867E-04

Contributions to homozygous loads for In and St= 2.74824852E-04 2.74824852E-04

Contributions to inbreeding loads for In and St= 8.93738445E-07 8.93738445E-07

Contributions to selection coefficients for In and St homokaryotypes

5.40614128E-05 5.40614128E-05

Contributions to mean A2 freqs= 4.76363041E-02 4.76363041E-02

Contributions to mean diversities= 6.19658618E-04 6.19658618E-04

Zone 2: quasi-neutral zone 2

Lower and upper bounds of St metapopn gamma for zone 2

0.250000000 499.999969

Probability of zone 2= 0.647554874

Coefficients for bivariate distribution of q1 and q2 in metapopulation

G1= 0.500000000 G2= 0.500000000

G3= 0.00000000 G4= 0.00000000

a1= 0.187500000 a2= 0.187500000

b11= 0.00000000 b12= 0.125000000 b22= 0.00000000

Contributions to mean load statistics for zone 2

Loads within In and St= 5.45769231E-03 5.45769185E-03

Load between In and St= 3.64925456E-03

Homozygous load for In and St= 5.83017617E-03 5.83017478E-03

Inbreeding loads= 3.72475450E-04 3.72475391E-04

Selection coefficients against In and St homokaryotypes

1.80679560E-03 1.80679560E-03

Contributions to mean A2 freqs= 4.09761257E-02 4.09761257E-02

Contributions to mean diversities= 1.28581538E-03 1.28581550E-03

Zone 3: moderate selection zone

Lower and upper bounds of St metapopn gamma for zone 3

499.999969 999.999939

Probability of zone 3= 0.116252482

Contributions to mean load statistics over zone 3

Loads within In and St= 1.26370491E-04 1.26383558E-04

Load between In and St= 9.57520606E-05

Homozygous load for In and St= 1.91496103E-04 1.91502913E-04

Inbreeding loads= 6.51255395E-05 6.51192677E-05

Selection coefficients for In and St homokaryotypes

3.06367874E-05 3.06367874E-05

Contributions to mean A2 freqs= 2.79713731E-06 2.79727374E-06

Contributions to mean diversities= 3.80227289E-06 3.80224333E-06

Zone 4: strong selection zone

Upper limit to scaled gamma for St metapopn= 2.50000000

Lower limit to gamma for St metapopn= 999.999939

Upper limit to gamma for St metapopn= 4166.66699

Probability of zone 4= 0.144941688

Contributions to mean load statistics over zone 4

Loads within In and St= 1.84412915E-04 1.84412886E-04

Load between In and St= 1.39999232E-04

Homozygous load for In and St= 2.79992033E-04 2.79992033E-04

Inbreeding loads= 9.55794021E-05 9.55793948E-05

Selection coefficients for In and St homokaryotypes

4.44054604E-05 4.44054604E-05

Contributions to mean A2 freqs= 3.40815245E-06 3.40815245E-06

Contributions to mean diversities= 7.12570696E-08 7.12570696E-08

Mean load statistics over all zones

Loads within In and St= 6.04240736E-03 6.04241993E-03

Load between In and St= 4.10486571E-03

Homozygous load for In and St= 6.57648919E-03 6.57649431E-03

Inbreeding loads= 5.34074148E-04 5.34067804E-04

Selection coefficients for In and St homokaryotypes

1.93566084E-03 1.93566084E-03

Mean frequencies of A2 in In and St= 8.86186287E-02 8.86186287E-02

Ratio of these= 1.00000000

Mean diversities at selected sites in In and St= 1.90934748E-03 1.90934760E-03

Ratio of these= 0.999999940

Mean diversities at neutral sites in In and St= 7.80487061E-03 7.80487061E-03

pi-n/pi-s for In and St= 0.244635373 0.244635388

Ratio of these= 0.999999940

**Neutral Fst for whole population= 0.25**

Scaled migration rate for whole population= 3.00000000

Zone 1: quasi-neutral zone 1

Upper bound scaled selection coefficient for neutrality in St metapopulation= 0.250000000

Probability of zone 1= 7.93938339E-02

Integral of selection coefficient over zone 1= 4.58041381E-04

Mean load statistics for zone 1

Mean q1 and q2= 0.600000024

F1 and F2= 0.983783841 0.983783841

Diversities= 7.78375613E-03 7.78375613E-03

Contributions to loads within In and St= 2.73933518E-04 2.73933518E-04

Contributions to load between In and St = 2.19859867E-04

Contributions to homozygous loads for In and St= 2.74824852E-04 2.74824852E-04

Contributions to inbreeding loads for In and St= 8.91308616E-07 8.91308616E-07

Contributions to selection coefficients for In and St homokaryotypes

5.40614128E-05 5.40614128E-05

Contributions to mean A2 freqs= 4.76363041E-02 4.76363041E-02

Contributions to mean diversities= 6.17982238E-04 6.17982238E-04

Zone 2: quasi-neutral zone 2

Lower and upper bounds of St metapopn gamma for zone 2

0.250000000 499.999969

Probability of zone 2= 0.647554874

Coefficients for bivariate distribution of q1 and q2 in metapopulation

G1= 0.666666687 G2= 0.666666687

G3= -0.333333373 G4= -0.333333373

a1= 0.208333343 a2= 0.208333343

b11= -2.08333358E-02 b12= 0.125000000 b22= -2.08333358E-02

Contributions to mean load statistics for zone 2

Loads within In and St= 5.56496903E-03 5.56496903E-03

Load between In and St= 3.71472375E-03

Homozygous load for In and St= 5.90162724E-03 5.90162678E-03

Inbreeding loads= 3.36669473E-04 3.36669473E-04

Selection coefficients against In and St homokaryotypes

1.84851885E-03 1.84851885E-03

Contributions to mean A2 freqs= 4.15949784E-02 4.15949784E-02

Contributions to mean diversities= 1.23424793E-03 1.23424793E-03

Zone 3: moderate selection zone

Lower and upper bounds of St metapopn gamma for zone 3

499.999969 999.999939

Probability of zone 3= 0.116252482

Contributions to mean load statistics over zone 3

Loads within In and St= 1.31013105E-04 1.31025256E-04

Load between In and St= 9.47575318E-05

Homozygous load for In and St= 1.89506434E-04 1.89514365E-04

Inbreeding loads= 5.84935224E-05 5.84893169E-05

Selection coefficients for In and St homokaryotypes

3.62396240E-05 3.62396240E-05

Contributions to mean A2 freqs= 2.76564310E-06 2.76580226E-06

Contributions to mean diversities= 3.41102555E-06 3.41091550E-06

Zone 4: strong selection zone

Upper limit to scaled gamma for St metapopn= 2.50000000

Lower limit to gamma for St metapopn= 999.999939

Upper limit to gamma for St metapopn= 4166.66699

Probability of zone 4= 0.144941688

Contributions to mean load statistics over zone 4

Loads within In and St= 1.99089685E-04 1.99089685E-04

Load between In and St= 1.44892882E-04

Homozygous load for In and St= 2.89778982E-04 2.89778953E-04

Inbreeding loads= 9.06890709E-05 9.06890491E-05

Selection coefficients for In and St homokaryotypes

5.41806221E-05 5.41806221E-05

Contributions to mean A2 freqs= 3.52281927E-06 3.52281927E-06

Contributions to mean diversities= 7.63143504E-08 7.63143504E-08

Mean load statistics over all zones

Loads within In and St= 6.16900530E-03 6.16901787E-03

Load between In and St= 4.17423388E-03

Homozygous load for In and St= 6.65573776E-03 6.65574521E-03

Inbreeding loads= 4.86743374E-04 4.86739154E-04

Selection coefficients for In and St homokaryotypes

1.99276209E-03 1.99282169E-03

Mean frequencies of A2 in In and St= 8.92375708E-02 8.92375708E-02

Ratio of these= 1.00000000

Mean diversities at selected sites in In and St= 1.85571751E-03 1.85571751E-03

Ratio of these= 1.00000000

Mean diversities at neutral sites in In and St= 7.78375613E-03 7.78375613E-03

pi-n/pi-s for In and St= 0.238408998 0.238408998

Ratio of these= 1.00000000

**h= 0.45**

**Neutral Fst for whole population= 0.05**

Scaled migration rate for whole population= 19.0000000

Zone 1: quasi-neutral zone 1

Upper bound scaled selection coefficient for neutrality in St metapopulation= 0.250000000

Probability of zone 1= 7.93938339E-02

Integral of selection coefficient over zone 1= 4.58041381E-04

Mean load statistics for zone 1

Mean q1 and q2= 0.600000024

F1 and F2= 0.983634770 0.983634770

Diversities= 7.85531010E-03 7.85531010E-03

Contributions to loads within In and St= 2.74644961E-04 2.74644961E-04

Contributions to load between In and St = 2.63831811E-04

Contributions to homozygous loads for In and St= 2.74824852E-04 2.74824852E-04

Contributions to inbreeding loads for In and St= 1.79888886E-07 1.79888886E-07

Contributions to selection coefficients for In and St homokaryotypes

1.07884407E-05 1.07884407E-05

Contributions to mean A2 freqs= 4.76363041E-02 4.76363041E-02

Contributions to mean diversities= 6.23663189E-04 6.23663189E-04

Zone 2: quasi-neutral zone 2

Lower and upper bounds of St metapopn gamma for zone 2

0.250000000 499.999969

Probability of zone 2= 0.647554874

Coefficients for bivariate distribution of q1 and q2 in metapopulation

G1= 0.105263159 G2= 0.105263159

G3= 0.789473653 G4= 0.789473653

a1= 0.227631569 a2= 0.227631569

b11= 9.86842345E-03 b12= 2.50000060E-02 b22= 9.86842345E-03

Contributions to mean load statistics for zone 2

Loads within In and St= 4.33774851E-03 4.33774851E-03

Load between In and St= 4.08336148E-03

Homozygous load for In and St= 4.39813035E-03 4.39813035E-03

Inbreeding loads= 6.03850349E-05 6.03850349E-05

Selection coefficients against In and St homokaryotypes

2.54333019E-04 2.54333019E-04

Contributions to mean A2 freqs= 3.79665606E-02 3.79665606E-02

Contributions to mean diversities= 1.17288996E-03 1.17289007E-03

Zone 3: moderate selection zone

Lower and upper bounds of St metapopn gamma for zone 3

499.999969 999.999939

Probability of zone 3= 0.116252482

Contributions to mean load statistics over zone 3

Loads within In and St= 1.00180652E-04 1.00184108E-04

Load between In and St= 9.91596316E-05

Homozygous load for In and St= 1.10175759E-04 1.10178837E-04

Inbreeding loads= 9.99509302E-06 9.99469648E-06

Selection coefficients for In and St homokaryotypes

1.01327896E-06 1.01327896E-06

Contributions to mean A2 freqs= 1.61416881E-06 1.61423020E-06

Contributions to mean diversities= 2.92843129E-06 2.92854133E-06

Zone 4: strong selection zone

Upper limit to scaled gamma for St metapopn= 2.50000000

Lower limit to gamma for St metapopn= 999.999939

Upper limit to gamma for St metapopn= 4166.66699

Probability of zone 4= 0.144941688

Contributions to mean load statistics over zone 4

Loads within In and St= 1.14212256E-04 1.14212286E-04

Load between In and St= 1.12936563E-04

Homozygous load for In and St= 1.25484818E-04 1.25484818E-04

Inbreeding loads= 1.12725747E-05 1.12725747E-05

Selection coefficients for In and St homokaryotypes

1.25169754E-06 1.25169754E-06

Contributions to mean A2 freqs= 1.53105077E-06 1.53105077E-06

Contributions to mean diversities= 2.73142575E-08 2.73142593E-08

Mean load statistics over all zones

Loads within In and St= 4.82678646E-03 4.82679019E-03

Load between In and St= 4.55928920E-03

Homozygous load for In and St= 4.90861619E-03 4.90861898E-03

Inbreeding loads= 8.18325934E-05 8.18321932E-05

Selection coefficients for In and St homokaryotypes

2.67446041E-04 2.67446041E-04

Mean frequencies of A2 in In and St= 8.56060088E-02 8.56060088E-02

Ratio of these= 1.00000000

Mean diversities at selected sites in In and St= 1.79950893E-03 1.79950916E-03

Ratio of these= 0.999999881

Mean diversities at neutral sites in In and St= 7.85531010E-03 7.85531010E-03

pi-n/pi-s for In and St= 0.229081839 0.229081869

Ratio of these= 0.999999881

**Neutral Fst for whole population= 0.1**

Scaled migration rate for whole population= 9.00000000

Zone 1: quasi-neutral zone 1

Upper bound scaled selection coefficient for neutrality in St metapopulation= 0.250000000

Probability of zone 1= 7.93938339E-02

Integral of selection coefficient over zone 1= 4.58041381E-04

Mean load statistics for zone 1

Mean q1 and q2= 0.600000024

F1 and F2= 0.983666062 0.983666062

Diversities= 7.84028973E-03 7.84028973E-03

Contributions to loads within In and St= 2.74645281E-04 2.74645281E-04

Contributions to load between In and St = 2.63831811E-04

Contributions to homozygous loads for In and St= 2.74824852E-04 2.74824852E-04

Contributions to inbreeding loads for In and St= 1.79561269E-07 1.79561269E-07

Contributions to selection coefficients for In and St homokaryotypes

1.07884407E-05 1.07884407E-05

Contributions to mean A2 freqs= 4.76363041E-02 4.76363041E-02

Contributions to mean diversities= 6.22470689E-04 6.22470689E-04

Zone 2: quasi-neutral zone 2

Lower and upper bounds of St metapopn gamma for zone 2

0.250000000 499.999969

Probability of zone 2= 0.647554874

Coefficients for bivariate distribution of q1 and q2 in metapopulation

G1= 0.222222224 G2= 0.222222224

G3= 0.555555582 G4= 0.555555582

a1= 0.230555549 a2= 0.230555549

b11= 6.94444636E-03 b12= 2.50000060E-02 b22= 6.94444636E-03

Contributions to mean load statistics for zone 2

Loads within In and St= 4.41098865E-03 4.41098865E-03

Load between In and St= 4.14946117E-03

Homozygous load for In and St= 4.47069621E-03 4.47069621E-03

Inbreeding loads= 5.97098151E-05 5.97098115E-05

Selection coefficients against In and St homokaryotypes

2.61485577E-04 2.61485577E-04

Contributions to mean A2 freqs= 3.80883254E-02 3.80883254E-02

Contributions to mean diversities= 1.16859190E-03 1.16859190E-03

Zone 3: moderate selection zone

Lower and upper bounds of St metapopn gamma for zone 3

499.999969 999.999939

Probability of zone 3= 0.116252482

Contributions to mean load statistics over zone 3

Loads within In and St= 1.07074680E-04 1.07078136E-04

Load between In and St= 1.05039617E-04

Homozygous load for In and St= 1.16708856E-04 1.16711955E-04

Inbreeding loads= 9.63426373E-06 9.63391903E-06

Selection coefficients for In and St homokaryotypes

2.02655792E-06 2.02655792E-06

Contributions to mean A2 freqs= 1.71010879E-06 1.71017109E-06

Contributions to mean diversities= 2.82243104E-06 2.82250380E-06

Zone 4: strong selection zone

Upper limit to scaled gamma for St metapopn= 2.50000000

Lower limit to gamma for St metapopn= 999.999939

Upper limit to gamma for St metapopn= 4166.66699

Probability of zone 4= 0.144941688

Contributions to mean load statistics over zone 4

Loads within In and St= 1.31634777E-04 1.31634748E-04

Load between In and St= 1.29063425E-04

Homozygous load for In and St= 1.43403813E-04 1.43403813E-04

Inbreeding loads= 1.17690506E-05 1.17690497E-05

Selection coefficients for In and St homokaryotypes

2.56299973E-06 2.56299973E-06

Contributions to mean A2 freqs= 1.74679064E-06 1.74679064E-06

Contributions to mean diversities= 3.32481349E-08 3.32481349E-08

Mean load statistics over all zones

Loads within In and St= 4.92434343E-03 4.92434669E-03

Load between In and St= 4.64739604E-03

Homozygous load for In and St= 5.00563346E-03 5.00563672E-03

Inbreeding loads= 8.12926883E-05 8.12923390E-05

Selection coefficients for In and St homokaryotypes

2.76923180E-04 2.76923180E-04

Mean frequencies of A2 in In and St= 8.57280865E-02 8.57280865E-02

Ratio of these= 1.00000000

Mean diversities at selected sites in In and St= 1.79391820E-03 1.79391832E-03

Ratio of these= 0.999999940

Mean diversities at neutral sites in In and St= 7.84028973E-03 7.84028973E-03

pi-n/pi-s for In and St= 0.228807643 0.228807658

Ratio of these= 0.999999940

**Neutral Fst for whole population= 0.15**

Scaled migration rate for whole population= 5.66666651

Zone 1: quasi-neutral zone 1

Upper bound scaled selection coefficient for neutrality in St metapopulation= 0.250000000

Probability of zone 1= 7.93938339E-02

Integral of selection coefficient over zone 1= 4.58041381E-04

Mean load statistics for zone 1

Mean q1 and q2= 0.600000024

F1 and F2= 0.983700812 0.983700812

Diversities= 7.82360975E-03 7.82360975E-03

Contributions to loads within In and St= 2.74645659E-04 2.74645659E-04

Contributions to load between In and St = 2.63831811E-04

Contributions to homozygous loads for In and St= 2.74824852E-04 2.74824852E-04

Contributions to inbreeding loads for In and St= 1.79179054E-07 1.79179054E-07

Contributions to selection coefficients for In and St homokaryotypes

1.07884407E-05 1.07884407E-05

Contributions to mean A2 freqs= 4.76363041E-02 4.76363041E-02

Contributions to mean diversities= 6.21146348E-04 6.21146348E-04

Zone 2: quasi-neutral zone 2

Lower and upper bounds of St metapopn gamma for zone 2

0.250000000 499.999969

Probability of zone 2= 0.647554874

Coefficients for bivariate distribution of q1 and q2 in metapopulation

G1= 0.352941185 G2= 0.352941185

G3= 0.294117630 G4= 0.294117630

a1= 0.233823523 a2= 0.233823523

b11= 3.67647130E-03 b12= 2.50000060E-02 b22= 3.67647130E-03

Contributions to mean load statistics for zone 2

Loads within In and St= 4.49089473E-03 4.49089473E-03

Load between In and St= 4.22157999E-03

Homozygous load for In and St= 4.54985676E-03 4.54985676E-03

Inbreeding loads= 5.89591109E-05 5.89591109E-05

Selection coefficients against In and St homokaryotypes

2.69293785E-04 2.69293785E-04

Contributions to mean A2 freqs= 3.82235311E-02 3.82235311E-02

Contributions to mean diversities= 1.16375287E-03 1.16375287E-03

Zone 3: moderate selection zone

Lower and upper bounds of St metapopn gamma for zone 3

499.999969 999.999939

Probability of zone 3= 0.116252482

Contributions to mean load statistics over zone 3

Loads within In and St= 1.13890012E-04 1.13894050E-04

Load between In and St= 1.10837282E-04

Homozygous load for In and St= 1.23150676E-04 1.23154299E-04

Inbreeding loads= 9.26059784E-06 9.26018492E-06

Selection coefficients for In and St homokaryotypes

3.03983688E-06 3.03983688E-06

Contributions to mean A2 freqs= 1.80466384E-06 1.80473614E-06

Contributions to mean diversities= 2.71239242E-06 2.71242448E-06

Zone 4: strong selection zone

Upper limit to scaled gamma for St metapopn= 2.50000000

Lower limit to gamma for St metapopn= 999.999939

Upper limit to gamma for St metapopn= 4166.66699

Probability of zone 4= 0.144941688

Contributions to mean load statistics over zone 4

Loads within In and St= 1.42370161E-04 1.42370161E-04

Load between In and St= 1.38580028E-04

Homozygous load for In and St= 1.53977468E-04 1.53977468E-04

Inbreeding loads= 1.16074843E-05 1.16074816E-05

Selection coefficients for In and St homokaryotypes

3.81469727E-06 3.81469727E-06

Contributions to mean A2 freqs= 1.87215574E-06 1.87215574E-06

Contributions to mean diversities= 3.75199996E-08 3.75199996E-08

Mean load statistics over all zones

Loads within In and St= 5.02180075E-03 5.02180494E-03

Load between In and St= 4.73482860E-03

Homozygous load for In and St= 5.10180974E-03 5.10181347E-03

Inbreeding loads= 8.00063717E-05 8.00059570E-05

Selection coefficients for In and St homokaryotypes

2.86936760E-04 2.86936760E-04

Mean frequencies of A2 in In and St= 8.58635083E-02 8.58635083E-02

Ratio of these= 1.00000000

Mean diversities at selected sites in In and St= 1.78764912E-03 1.78764923E-03

Ratio of these= 0.999999940

Mean diversities at neutral sites in In and St= 7.82360975E-03 7.82360975E-03

pi-n/pi-s for In and St= 0.228494152 0.228494167

Ratio of these= 0.999999940

**Neutral Fst for whole population= 0.2**

Scaled migration rate for whole population= 4.00000000

Zone 1: quasi-neutral zone 1

Upper bound scaled selection coefficient for neutrality in St metapopulation= 0.250000000

Probability of zone 1= 7.93938339E-02

Integral of selection coefficient over zone 1= 4.58041381E-04

Mean load statistics for zone 1

Mean q1 and q2= 0.600000024

F1 and F2= 0.983739853 0.983739853

Diversities= 7.80487061E-03 7.80487061E-03

Contributions to loads within In and St= 2.74646096E-04 2.74646096E-04

Contributions to load between In and St = 2.63831811E-04

Contributions to homozygous loads for In and St= 2.74824852E-04 2.74824852E-04

Contributions to inbreeding loads for In and St= 1.78742226E-07 1.78742226E-07

Contributions to selection coefficients for In and St homokaryotypes

1.07884407E-05 1.07884407E-05

Contributions to mean A2 freqs= 4.76363041E-02 4.76363041E-02

Contributions to mean diversities= 6.19658618E-04 6.19658618E-04

Zone 2: quasi-neutral zone 2

Lower and upper bounds of St metapopn gamma for zone 2

0.250000000 499.999969

Probability of zone 2= 0.647554874

Coefficients for bivariate distribution of q1 and q2 in metapopulation

G1= 0.500000000 G2= 0.500000000

G3= 0.00000000 G4= 0.00000000

a1= 0.237499997 a2= 0.237499997

b11= 0.00000000 b12= 2.50000060E-02 b22= 0.00000000

Contributions to mean load statistics for zone 2

Loads within In and St= 4.57835523E-03 4.57835523E-03

Load between In and St= 4.30053053E-03

Homozygous load for In and St= 4.63647489E-03 4.63647489E-03

Inbreeding loads= 5.81204658E-05 5.81204658E-05

Selection coefficients against In and St homokaryotypes

2.77757645E-04 2.77757645E-04

Contributions to mean A2 freqs= 3.83745097E-02 3.83745097E-02

Contributions to mean diversities= 1.15826761E-03 1.15826761E-03

Zone 3: moderate selection zone

Lower and upper bounds of St metapopn gamma for zone 3

499.999969 999.999939

Probability of zone 3= 0.116252482

Contributions to mean load statistics over zone 3

Loads within In and St= 1.20742981E-04 1.20747623E-04

Load between In and St= 1.16659037E-04

Homozygous load for In and St= 1.29618667E-04 1.29623106E-04

Inbreeding loads= 8.87562692E-06 8.87544684E-06

Selection coefficients for In and St homokaryotypes

4.11272049E-06 4.11272049E-06

Contributions to mean A2 freqs= 1.89941522E-06 1.89950413E-06

Contributions to mean diversities= 2.59868807E-06 2.59867170E-06

Zone 4: strong selection zone

Upper limit to scaled gamma for St metapopn= 2.50000000

Lower limit to gamma for St metapopn= 999.999939

Upper limit to gamma for St metapopn= 4166.66699

Probability of zone 4= 0.144941688

Contributions to mean load statistics over zone 4

Loads within In and St= 1.51818371E-04 1.51818371E-04

Load between In and St= 1.46824765E-04

Homozygous load for In and St= 1.63138320E-04 1.63138335E-04

Inbreeding loads= 1.13199485E-05 1.13199494E-05

Selection coefficients for In and St homokaryotypes

5.00679016E-06 5.00679016E-06

Contributions to mean A2 freqs= 1.97981649E-06 1.97981649E-06

Contributions to mean diversities= 4.13892316E-08 4.13892280E-08

Mean load statistics over all zones

Loads within In and St= 5.12556266E-03 5.12556732E-03

Load between In and St= 4.82784631E-03

Homozygous load for In and St= 5.20405686E-03 5.20406151E-03

Inbreeding loads= 7.84947842E-05 7.84946023E-05

Selection coefficients for In and St homokaryotypes

2.97665596E-04 2.97665596E-04

Mean frequencies of A2 in In and St= 8.60146955E-02 8.60146955E-02

Ratio of these= 1.00000000

Mean diversities at selected sites in In and St= 1.78056641E-03 1.78056629E-03

Ratio of these= 1.00000012

Mean diversities at neutral sites in In and St= 7.80487061E-03 7.80487061E-03

pi-n/pi-s for In and St= 0.228135288 0.228135273

Ratio of these= 1.00000012

**Neutral Fst for whole population= 0.25**

Scaled migration rate for whole population= 3.00000000

Zone 1: quasi-neutral zone 1

Upper bound scaled selection coefficient for neutrality in St metapopulation= 0.250000000

Probability of zone 1= 7.93938339E-02

Integral of selection coefficient over zone 1= 4.58041381E-04

Mean load statistics for zone 1

Mean q1 and q2= 0.600000024

F1 and F2= 0.983783841 0.983783841

Diversities= 7.78375613E-03 7.78375613E-03

Contributions to loads within In and St= 2.74646591E-04 2.74646591E-04

Contributions to load between In and St = 2.63831811E-04

Contributions to homozygous loads for In and St= 2.74824852E-04 2.74824852E-04

Contributions to inbreeding loads for In and St= 1.78250801E-07 1.78250801E-07

Contributions to selection coefficients for In and St homokaryotypes

1.07884407E-05 1.07884407E-05

Contributions to mean A2 freqs= 4.76363041E-02 4.76363041E-02

Contributions to mean diversities= 6.17982238E-04 6.17982238E-04

Zone 2: quasi-neutral zone 2

Lower and upper bounds of St metapopn gamma for zone 2

0.250000000 499.999969

Probability of zone 2= 0.647554874

Coefficients for bivariate distribution of q1 and q2 in metapopulation

G1= 0.666666687 G2= 0.666666687

G3= -0.333333373 G4= -0.333333373

a1= 0.241666660 a2= 0.241666660

b11= -4.16666828E-03 b12= 2.50000060E-02 b22= -4.16666828E-03

Contributions to mean load statistics for zone 2

Loads within In and St= 4.67441464E-03 4.67441464E-03

Load between In and St= 4.38725855E-03

Homozygous load for In and St= 4.73159365E-03 4.73159365E-03

Inbreeding loads= 5.71791425E-05 5.71791425E-05

Selection coefficients against In and St homokaryotypes

2.87115574E-04 2.87115574E-04

Contributions to mean A2 freqs= 3.85441333E-02 3.85441333E-02

Contributions to mean diversities= 1.15200423E-03 1.15200423E-03

Zone 3: moderate selection zone

Lower and upper bounds of St metapopn gamma for zone 3

499.999969 999.999939

Probability of zone 3= 0.116252482

Contributions to mean load statistics over zone 3

Loads within In and St= 1.27798601E-04 1.27804611E-04

Load between In and St= 1.22658021E-04

Homozygous load for In and St= 1.36283546E-04 1.36289193E-04

Inbreeding loads= 8.48495347E-06 8.48458876E-06

Selection coefficients for In and St homokaryotypes

5.12599945E-06 5.12599945E-06

Contributions to mean A2 freqs= 1.99673673E-06 1.99684973E-06

Contributions to mean diversities= 2.48294737E-06 2.48287438E-06

Zone 4: strong selection zone

Upper limit to scaled gamma for St metapopn= 2.50000000

Lower limit to gamma for St metapopn= 999.999939

Upper limit to gamma for St metapopn= 4166.66699

Probability of zone 4= 0.144941688

Contributions to mean load statistics over zone 4

Loads within In and St= 1.61351825E-04 1.61351825E-04

Load between In and St= 1.55130212E-04

Homozygous load for In and St= 1.72366403E-04 1.72366446E-04

Inbreeding loads= 1.10146402E-05 1.10146393E-05

Selection coefficients for In and St homokaryotypes

6.19888306E-06 6.19888306E-06

Contributions to mean A2 freqs= 2.08795291E-06 2.08795291E-06

Contributions to mean diversities= 4.52193092E-08 4.52193056E-08

Mean load statistics over all zones

Loads within In and St= 5.23821125E-03 5.23821730E-03

Load between In and St= 4.92887851E-03

Homozygous load for In and St= 5.31506864E-03 5.31507423E-03

Inbreeding loads= 7.68569880E-05 7.68566242E-05

Selection coefficients for In and St homokaryotypes

3.09288502E-04 3.09288502E-04

Mean frequencies of A2 in In and St= 8.61845165E-02 8.61845165E-02

Ratio of these= 1.00000000

Mean diversities at selected sites in In and St= 1.77251454E-03 1.77251454E-03

Ratio of these= 1.00000000

Mean diversities at neutral sites in In and St= 7.78375613E-03 7.78375613E-03

pi-n/pi-s for In and St= 0.227719694 0.227719694

Ratio of these= 1.00000000
