# Supplementary material for: The fitness consequences of genetic divergence between polymorphic gene arrangements
Source: Genetics. 2023 Dec 26;226(3):iyad218. doi: 10.1093/genetics/iyad218 (PMC11090464; doi:10.1093/genetics/iyad218)
Supplement: iyad218_Supplementary_Data [file iyad218_supplementary_data.zip › Supplementary_Table_5_GENETICS-2023-306559.docx]

**Supplementary Table S5 Intermediate results for the full bivariate distribution for zone 2a for a single population**

**Results are shown for a mean scaled selection coefficient of 1000 and inversion frequencies of 0.1 and 0.5, with dominance coefficients of h = 0.05, 0.25, and 0.45**

**Mutation rate towards deleterious variants= 4.99999997E-09**

**Number of selected sites in inversion= 100000**

**Mutational bias towards deleterious variants= 1.50000000**

**Population size= 1000000**

**No. of values for Simpsons rule = 150**

**Sample size= 20**

**Wattersons correction factor= 3.54773974**

**Number of dominance coefficients modelled= 3**

**Shape parameter= 0.3**

**Upper bound gamma value for neutrality in the St popn= 0.250**

**Upper bound whole popn gamma value for zone 2a with h=0.25 = 50**

**Upper bound whole popn gamma value for zone 2b with h=0.25 = 500**

**Upper limit to gamma distribution of z= x/scale parameter = 2.50**

**Threshold gamma value factor for use of approximate p.d.f.= 0.25**

**Inversion frequency = 0.1**

**h=0.05**

Index of gamma value 31 gamma for St popn= 50.1999969

Means of q1 and q2= 0.337815344 1.15531276E-03

Variances of q1 and q2= 0.222836554 7.96755849E-05

Covariance and correlation of q1 and q2= -1.28508778E-04 -3.04983929E-02

F1 and F2= 0.996157348 6.90442845E-02

Freqs. of fixation of A1 for In and St= 0.658498049 0.985884488

Freqs. of fixation of A2 for In and St= 0.335482568 0.00000000

Freqs. of segregation for In and St= 6.01938367E-03 1.41155124E-02

q1f= 0.337514192 q2f= 0.00000000

Index of gamma value 32 gamma for St popn= 51.8649979

Means of q1 and q2= 0.329925328 1.12027605E-03

Variances of q1 and q2= 0.220216423 7.58134120E-05

Covariance and correlation of q1 and q2= -1.23718753E-04 -3.02787311E-02

F1 and F2= 0.996118128 6.77497610E-02

Freqs. of fixation of A1 for In and St= 0.666368842 0.986211777

Freqs. of fixation of A2 for In and St= 0.327616364 0.00000000

Freqs. of segregation for In and St= 6.01479411E-03 1.37882233E-02

q1f= 0.329598844 q2f= 0.00000000

Index of gamma value 33 gamma for St popn= 53.5299988

Means of q1 and q2= 0.322135925 1.08719850E-03

Variances of q1 and q2= 0.217507705 7.22433906E-05

Covariance and correlation of q1 and q2= -1.19102886E-04 -3.00459471E-02

F1 and F2= 0.996076882 6.65214434E-02

Freqs. of fixation of A1 for In and St= 0.674139678 0.986524582

Freqs. of fixation of A2 for In and St= 0.319850922 0.00000000

Freqs. of segregation for In and St= 6.00939989E-03 1.34754181E-02

q1f= 0.321784645 q2f= 0.00000000

Index of gamma value 34 gamma for St popn= 55.1949997

Means of q1 and q2= 0.314438581 1.05592236E-03

Variances of q1 and q2= 0.214712024 6.89359367E-05

Covariance and correlation of q1 and q2= -1.14651004E-04 -2.98007261E-02

F1 and F2= 0.996034026 6.53540492E-02

Freqs. of fixation of A1 for In and St= 0.681819677 0.986823857

Freqs. of fixation of A2 for In and St= 0.312177569 0.00000000

Freqs. of segregation for In and St= 6.00275397E-03 1.31761432E-02

q1f= 0.314062834 q2f= 0.00000000

Index of gamma value 35 gamma for St popn= 56.8600006

Means of q1 and q2= 0.306844950 1.02629187E-03

Variances of q1 and q2= 0.211838007 6.58638601E-05

Covariance and correlation of q1 and q2= -1.10356777E-04 -2.95442846E-02

F1 and F2= 0.995988905 6.42424673E-02

Freqs. of fixation of A1 for In and St= 0.689396441 0.987110436

Freqs. of fixation of A2 for In and St= 0.304608017 0.00000000

Freqs. of segregation for In and St= 5.99554181E-03 1.28895640E-02

q1f= 0.306445301 q2f= 0.00000000

Index of gamma value 36 gamma for St popn= 58.5249977

Means of q1 and q2= 0.299357384 9.98176634E-04

Variances of q1 and q2= 0.208891392 6.30041468E-05

Covariance and correlation of q1 and q2= -1.06214255E-04 -2.92777680E-02

F1 and F2= 0.995941997 6.31823093E-02

Freqs. of fixation of A1 for In and St= 0.696868122 0.987385511

Freqs. of fixation of A2 for In and St= 0.297144502 0.00000000

Freqs. of segregation for In and St= 5.98737597E-03 1.26144886E-02

q1f= 0.298934340 q2f= 0.00000000

Index of gamma value 37 gamma for St popn= 60.1899986

Means of q1 and q2= 0.291970462 9.71462054E-04

Variances of q1 and q2= 0.205874741 6.03368426E-05

Covariance and correlation of q1 and q2= -1.02214632E-04 -2.90014856E-02

F1 and F2= 0.995893240 6.21697083E-02

Freqs. of fixation of A1 for In and St= 0.704239786 0.987649441

Freqs. of fixation of A2 for In and St= 0.289781779 0.00000000

Freqs. of segregation for In and St= 5.97843528E-03 1.23505592E-02

q1f= 0.291524649 q2f= 0.00000000

Index of gamma value 38 gamma for St popn= 61.8549995

Means of q1 and q2= 0.284698457 9.46035958E-04

Variances of q1 and q2= 0.202798530 5.78435684E-05

Covariance and correlation of q1 and q2= -9.83554637E-05 -2.87169646E-02

F1 and F2= 0.995842218 6.12009950E-02

Freqs. of fixation of A1 for In and St= 0.711497724 0.987903416

Freqs. of fixation of A2 for In and St= 0.282533854 0.00000000

Freqs. of segregation for In and St= 5.96842170E-03 1.20965838E-02

q1f= 0.284230262 q2f= 0.00000000

Index of gamma value 39 gamma for St popn= 63.5199966

Means of q1 and q2= 0.277535737 9.21805389E-04

Variances of q1 and q2= 0.199665323 5.55088009E-05

Covariance and correlation of q1 and q2= -9.46294022E-05 -2.84245685E-02

F1 and F2= 0.995789111 6.02730438E-02

Freqs. of fixation of A1 for In and St= 0.718646944 0.988147497

Freqs. of fixation of A2 for In and St= 0.275395304 0.00000000

Freqs. of segregation for In and St= 5.95775247E-03 1.18525028E-02

q1f= 0.277045876 q2f= 0.00000000

Index of gamma value 40 gamma for St popn= 65.1849976

Means of q1 and q2= 0.270490497 8.98683269E-04

Variances of q1 and q2= 0.196483642 5.33184066E-05

Covariance and correlation of q1 and q2= -9.10336094E-05 -2.81254835E-02

F1 and F2= 0.995734274 5.93828410E-02

Freqs. of fixation of A1 for In and St= 0.725679636 0.988382459

Freqs. of fixation of A2 for In and St= 0.268374026 0.00000000

Freqs. of segregation for In and St= 5.94633818E-03 1.16175413E-02

q1f= 0.269979417 q2f= 0.00000000

Index of gamma value 41 gamma for St popn= 66.8499985

Means of q1 and q2= 0.263556331 8.76592530E-04

Variances of q1 and q2= 0.193255335 5.12602492E-05

Covariance and correlation of q1 and q2= -8.75609403E-05 -2.78198216E-02

F1 and F2= 0.995677054 5.85280173E-02

Freqs. of fixation of A1 for In and St= 0.732601821 0.988608897

Freqs. of fixation of A2 for In and St= 0.261464000 0.00000000

Freqs. of segregation for In and St= 5.93417883E-03 1.13911033E-02

q1f= 0.263024837 q2f= 0.00000000

Index of gamma value 42 gamma for St popn= 68.5149994

Means of q1 and q2= 0.256733865 8.55465187E-04

Variances of q1 and q2= 0.189985305 4.93233565E-05

Covariance and correlation of q1 and q2= -8.42076406E-05 -2.75083892E-02

F1 and F2= 0.995617568 5.77061288E-02

Freqs. of fixation of A1 for In and St= 0.739413202 0.988827527

Freqs. of fixation of A2 for In and St= 0.254665524 0.00000000

Freqs. of segregation for In and St= 5.92127442E-03 1.11724734E-02

q1f= 0.256182432 q2f= 0.00000000

Index of gamma value 43 gamma for St popn= 70.1800003

Means of q1 and q2= 0.250030577 8.35232262E-04

Variances of q1 and q2= 0.186681941 4.74975095E-05

Covariance and correlation of q1 and q2= -8.09705234E-05 -2.71919519E-02

F1 and F2= 0.995555818 5.69149628E-02

Freqs. of fixation of A1 for In and St= 0.746106207 0.989038765

Freqs. of fixation of A2 for In and St= 0.247986227 0.00000000

Freqs. of segregation for In and St= 5.90756536E-03 1.09612346E-02

q1f= 0.249459922 q2f= 0.00000000

Index of gamma value 44 gamma for St popn= 71.8450012

Means of q1 and q2= 0.243447512 8.15836014E-04

Variances of q1 and q2= 0.183350503 4.57738788E-05

Covariance and correlation of q1 and q2= -7.78462490E-05 -2.68712435E-02

F1 and F2= 0.995491862 5.61525226E-02

Freqs. of fixation of A1 for In and St= 0.752679765 0.989242792

Freqs. of fixation of A2 for In and St= 0.241427034 0.00000000

Freqs. of segregation for In and St= 5.89320064E-03 1.07572079E-02

q1f= 0.242858261 q2f= 0.00000000

Index of gamma value 45 gamma for St popn= 73.5099945

Means of q1 and q2= 0.236984894 7.97222950E-04

Variances of q1 and q2= 0.179995865 4.41445591E-05

Covariance and correlation of q1 and q2= -7.48309103E-05 -2.65467409E-02

F1 and F2= 0.995425463 5.54170944E-02

Freqs. of fixation of A1 for In and St= 0.759133518 0.989439487

Freqs. of fixation of A2 for In and St= 0.234988168 0.00000000

Freqs. of segregation for In and St= 5.87831438E-03 1.05605125E-02

q1f= 0.236377671 q2f= 0.00000000

Index of gamma value 46 gamma for St popn= 75.1749954

Means of q1 and q2= 0.230642483 7.79344526E-04

Variances of q1 and q2= 0.176622599 4.26023544E-05

Covariance and correlation of q1 and q2= -7.19207892E-05 -2.62189042E-02

F1 and F2= 0.995356739 5.47069721E-02

Freqs. of fixation of A1 for In and St= 0.765468121 0.989630699

Freqs. of fixation of A2 for In and St= 0.228669465 0.00000000

Freqs. of segregation for In and St= 5.86241484E-03 1.03693008E-02

q1f= 0.230017930 q2f= 0.00000000

Index of gamma value 47 gamma for St popn= 76.8399963

Means of q1 and q2= 0.224412397 7.62158888E-04

Variances of q1 and q2= 0.173230916 4.11410547E-05

Covariance and correlation of q1 and q2= -6.91107925E-05 -2.58878190E-02

F1 and F2= 0.995285511 5.40208034E-02

Freqs. of fixation of A1 for In and St= 0.771690786 0.989815295

Freqs. of fixation of A2 for In and St= 0.222462997 0.00000000

Freqs. of segregation for In and St= 5.84621727E-03 1.01847053E-02

q1f= 0.223771214 q2f= 0.00000000

Index of gamma value 48 gamma for St popn= 78.5049973

Means of q1 and q2= 0.218311042 7.45619473E-04

Variances of q1 and q2= 0.169834182 3.97543627E-05

Covariance and correlation of q1 and q2= -6.64014588E-05 -2.55548097E-02

F1 and F2= 0.995211601 5.33570088E-02

Freqs. of fixation of A1 for In and St= 0.777785420 0.989994287

Freqs. of fixation of A2 for In and St= 0.216385067 0.00000000

Freqs. of segregation for In and St= 5.82951307E-03 1.00057125E-02

q1f= 0.217653885 q2f= 0.00000000

Index of gamma value 49 gamma for St popn= 80.1699982

Means of q1 and q2= 0.212329701 7.29690481E-04

Variances of q1 and q2= 0.166432202 3.84371415E-05

Covariance and correlation of q1 and q2= -6.37876656E-05 -2.52198502E-02

F1 and F2= 0.995135307 5.27144149E-02

Freqs. of fixation of A1 for In and St= 0.783761084 0.990167797

Freqs. of fixation of A2 for In and St= 0.210427001 0.00000000

Freqs. of segregation for In and St= 5.81191480E-03 9.83220339E-03

q1f= 0.211657137 q2f= 0.00000000

Index of gamma value 50 gamma for St popn= 81.8349991

Means of q1 and q2= 0.206467986 7.14337977E-04

Variances of q1 and q2= 0.163028970 3.71845963E-05

Covariance and correlation of q1 and q2= -6.12663425E-05 -2.48833019E-02

F1 and F2= 0.995056212 5.20918407E-02

Freqs. of fixation of A1 for In and St= 0.789617717 0.990336657

Freqs. of fixation of A2 for In and St= 0.204588503 0.00000000

Freqs. of segregation for In and St= 5.79378009E-03 9.66334343E-03

q1f= 0.205780759 q2f= 0.00000000

Index of gamma value 51 gamma for St popn= 83.5000000

Means of q1 and q2= 0.200734466 6.99526223E-04

Variances of q1 and q2= 0.159633934 3.59920668E-05

Covariance and correlation of q1 and q2= -5.88366602E-05 -2.45460719E-02

F1 and F2= 0.994975090 5.14880791E-02

Freqs. of fixation of A1 for In and St= 0.795346856 0.990499973

Freqs. of fixation of A2 for In and St= 0.198877946 0.00000000

Freqs. of segregation for In and St= 5.77519834E-03 9.50002670E-03

q1f= 0.200033173 q2f= 0.00000000

Index of gamma value 52 gamma for St popn= 85.1650009

Means of q1 and q2= 0.195128992 6.85225765E-04

Variances of q1 and q2= 0.156251252 3.48556059E-05

Covariance and correlation of q1 and q2= -5.64959773E-05 -2.42085885E-02

F1 and F2= 0.994890809 5.09022102E-02

Freqs. of fixation of A1 for In and St= 0.800948679 0.990658879

Freqs. of fixation of A2 for In and St= 0.193295226 0.00000000

Freqs. of segregation for In and St= 5.75609505E-03 9.34112072E-03

q1f= 0.194414288 q2f= 0.00000000

Index of gamma value 53 gamma for St popn= 86.8300018

Means of q1 and q2= 0.189631850 6.71415939E-04

Variances of q1 and q2= 0.152873084 3.37719976E-05

Covariance and correlation of q1 and q2= -5.42371563E-05 -2.38700323E-02

F1 and F2= 0.994803607 5.03334589E-02

Freqs. of fixation of A1 for In and St= 0.806442559 0.990813017

Freqs. of fixation of A2 for In and St= 0.187820792 0.00000000

Freqs. of segregation for In and St= 5.73664904E-03 9.18698311E-03

q1f= 0.188904464 q2f= 0.00000000

Index of gamma value 54 gamma for St popn= 88.4949951

Means of q1 and q2= 0.184241518 6.58071891E-04

Variances of q1 and q2= 0.149501994 3.27379421E-05

Covariance and correlation of q1 and q2= -5.20574322E-05 -2.35306304E-02

F1 and F2= 0.994713187 4.97810356E-02

Freqs. of fixation of A1 for In and St= 0.811830282 0.990963042

Freqs. of fixation of A2 for In and St= 0.182453066 0.00000000

Freqs. of segregation for In and St= 5.71665168E-03 9.03695822E-03

q1f= 0.183502093 q2f= 0.00000000

Index of gamma value 55 gamma for St popn= 90.1599960

Means of q1 and q2= 0.178986207 6.45158987E-04

Variances of q1 and q2= 0.146159574 3.17495469E-05

Covariance and correlation of q1 and q2= -4.99608432E-05 -2.31924895E-02

F1 and F2= 0.994620144 4.92437445E-02

Freqs. of fixation of A1 for In and St= 0.817083597 0.991108537

Freqs. of fixation of A2 for In and St= 0.177220017 0.00000000

Freqs. of segregation for In and St= 5.69638610E-03 8.89146328E-03

q1f= 0.178235322 q2f= 0.00000000

Index of gamma value 56 gamma for St popn= 91.8249969

Means of q1 and q2= 0.173845440 6.32662326E-04

Variances of q1 and q2= 0.142836675 3.08045019E-05

Covariance and correlation of q1 and q2= -4.79404407E-05 -2.28546765E-02

F1 and F2= 0.994523644 4.87210974E-02

Freqs. of fixation of A1 for In and St= 0.822223246 0.991251171

Freqs. of fixation of A2 for In and St= 0.172101393 0.00000000

Freqs. of segregation for In and St= 5.67536056E-03 8.74882936E-03

q1f= 0.173083708 q2f= 0.00000000

Index of gamma value 57 gamma for St popn= 93.4899979

Means of q1 and q2= 0.168817863 6.20561477E-04

Variances of q1 and q2= 0.139536038 2.99002004E-05

Covariance and correlation of q1 and q2= -4.59937073E-05 -2.25173961E-02

F1 and F2= 0.994424403 4.82124127E-02

Freqs. of fixation of A1 for In and St= 0.827250004 0.991389394

Freqs. of fixation of A2 for In and St= 0.167095780 0.00000000

Freqs. of segregation for In and St= 5.65421581E-03 8.61060619E-03

q1f= 0.168045953 q2f= 0.00000000

Index of gamma value 58 gamma for St popn= 95.1549988

Means of q1 and q2= 0.163922429 6.08830771E-04

Variances of q1 and q2= 0.136273697 2.90337866E-05

Covariance and correlation of q1 and q2= -4.41227712E-05 -2.21822150E-02

F1 and F2= 0.994322062 4.77168299E-02

Freqs. of fixation of A1 for In and St= 0.832145512 0.991524160

Freqs. of fixation of A2 for In and St= 0.162222013 0.00000000

Freqs. of segregation for In and St= 5.63247502E-03 8.47584009E-03

q1f= 0.163140893 q2f= 0.00000000

Index of gamma value 59 gamma for St popn= 96.8199997

Means of q1 and q2= 0.159117162 5.97466424E-04

Variances of q1 and q2= 0.133024976 2.82039618E-05

Covariance and correlation of q1 and q2= -4.23162819E-05 -2.18467005E-02

F1 and F2= 0.994215786 4.72341590E-02

Freqs. of fixation of A1 for In and St= 0.836951017 0.991655409

Freqs. of fixation of A2 for In and St= 0.157438338 0.00000000

Freqs. of segregation for In and St= 5.61064482E-03 8.34459066E-03

q1f= 0.158326656 q2f= 0.00000000

Index of gamma value 60 gamma for St popn= 98.4850006

Means of q1 and q2= 0.154452011 5.86434384E-04

Variances of q1 and q2= 0.129826963 2.74075719E-05

Covariance and correlation of q1 and q2= -4.05833562E-05 -2.15144493E-02

F1 and F2= 0.994106948 4.67633791E-02

Freqs. of fixation of A1 for In and St= 0.841617405 0.991783738

Freqs. of fixation of A2 for In and St= 0.152794436 0.00000000

Freqs. of segregation for In and St= 5.58815897E-03 8.21626186E-03

q1f= 0.153653070 q2f= 0.00000000

Index of gamma value 61 gamma for St popn= 100.149994

Means of q1 and q2= 0.149894357 5.75728714E-04

Variances of q1 and q2= 0.126660764 2.66432871E-05

Covariance and correlation of q1 and q2= -3.89147281E-05 -2.11835634E-02

F1 and F2= 0.993994355 4.63041589E-02

Freqs. of fixation of A1 for In and St= 0.846176505 0.991908371

Freqs. of fixation of A2 for In and St= 0.148257822 0.00000000

Freqs. of segregation for In and St= 5.56567311E-03 8.09162855E-03

q1f= 0.149087593 q2f= 0.00000000

Index of gamma value 62 gamma for St popn= 101.814995

Means of q1 and q2= 0.145442829 5.65336377E-04

Variances of q1 and q2= 0.123528346 2.59094704E-05

Covariance and correlation of q1 and q2= -3.73084586E-05 -2.08542570E-02

F1 and F2= 0.993878305 4.58561108E-02

Freqs. of fixation of A1 for In and St= 0.850629985 0.992030263

Freqs. of fixation of A2 for In and St= 0.143827155 0.00000000

Freqs. of segregation for In and St= 5.54285944E-03 7.96973705E-03

q1f= 0.144628808 q2f= 0.00000000

Index of gamma value 63 gamma for St popn= 103.479996

Means of q1 and q2= 0.141106263 5.55239501E-04

Variances of q1 and q2= 0.120438844 2.52042082E-05

Covariance and correlation of q1 and q2= -3.57646677E-05 -2.05274206E-02

F1 and F2= 0.993758500 4.54186164E-02

Freqs. of fixation of A1 for In and St= 0.854969025 0.992149055

Freqs. of fixation of A2 for In and St= 0.139511198 0.00000000

Freqs. of segregation for In and St= 5.51977754E-03 7.85094500E-03

q1f= 0.140285537 q2f= 0.00000000

Index of gamma value 64 gamma for St popn= 105.144997

Means of q1 and q2= 0.136883020 5.45426272E-04

Variances of q1 and q2= 0.117394060 2.45260289E-05

Covariance and correlation of q1 and q2= -3.42813109E-05 -2.02032272E-02

F1 and F2= 0.993634999 4.49912585E-02

Freqs. of fixation of A1 for In and St= 0.859195590 0.992265642

Freqs. of fixation of A2 for In and St= 0.135308325 0.00000000

Freqs. of segregation for In and St= 5.49608469E-03 7.73435831E-03

q1f= 0.136056095 q2f= 0.00000000

Index of gamma value 65 gamma for St popn= 106.809998

Means of q1 and q2= 0.132760540 5.35885512E-04

Variances of q1 and q2= 0.114387676 2.38736247E-05

Covariance and correlation of q1 and q2= -3.28537462E-05 -1.98809020E-02

F1 and F2= 0.993507624 4.45737503E-02

Freqs. of fixation of A1 for In and St= 0.863321424 0.992378950

Freqs. of fixation of A2 for In and St= 0.131206021 0.00000000

Freqs. of segregation for In and St= 5.47255576E-03 7.62104988E-03

q1f= 0.131927997 q2f= 0.00000000

Index of gamma value 66 gamma for St popn= 108.474998

Means of q1 and q2= 0.128748164 5.26605814E-04

Variances of q1 and q2= 0.111429103 2.32456114E-05

Covariance and correlation of q1 and q2= -3.14828176E-05 -1.95615739E-02

F1 and F2= 0.993376493 4.41655926E-02

Freqs. of fixation of A1 for In and St= 0.867337883 0.992489696

Freqs. of fixation of A2 for In and St= 0.127213523 0.00000000

Freqs. of segregation for In and St= 5.44859469E-03 7.51030445E-03

q1f= 0.127910450 q2f= 0.00000000

Index of gamma value 67 gamma for St popn= 110.139999

Means of q1 and q2= 0.124843828 5.17575187E-04

Variances of q1 and q2= 0.108519427 2.26406864E-05

Covariance and correlation of q1 and q2= -3.01663204E-05 -1.92452613E-02

F1 and F2= 0.993241489 4.37664129E-02

Freqs. of fixation of A1 for In and St= 0.871246517 0.992597997

Freqs. of fixation of A2 for In and St= 0.123328812 0.00000000

Freqs. of segregation for In and St= 5.42467088E-03 7.40200281E-03

q1f= 0.124001481 q2f= 0.00000000

Index of gamma value 68 gamma for St popn= 111.805000

Means of q1 and q2= 0.121034846 5.08786470E-04

Variances of q1 and q2= 0.105651550 2.20578713E-05

Covariance and correlation of q1 and q2= -2.89000491E-05 -1.89312305E-02

F1 and F2= 0.993101895 4.33759540E-02

Freqs. of fixation of A1 for In and St= 0.875060380 0.992703617

Freqs. of fixation of A2 for In and St= 0.119539291 0.00000000

Freqs. of segregation for In and St= 5.40032983E-03 7.29638338E-03

q1f= 0.120188341 q2f= 0.00000000

Index of gamma value 69 gamma for St popn= 113.470001

Means of q1 and q2= 0.117330380 5.00227383E-04

Variances of q1 and q2= 0.102834679 2.14959437E-05

Covariance and correlation of q1 and q2= -2.76845967E-05 -1.86204482E-02

F1 and F2= 0.992958128 4.29938510E-02

Freqs. of fixation of A1 for In and St= 0.878769994 0.992807031

Freqs. of fixation of A2 for In and St= 0.115854003 0.00000000

Freqs. of segregation for In and St= 5.37600368E-03 7.19296932E-03

q1f= 0.116480201 q2f= 0.00000000

Index of gamma value 70 gamma for St popn= 115.134995

Means of q1 and q2= 0.113717586 4.91892162E-04

Variances of q1 and q2= 0.100061223 2.09540776E-05

Covariance and correlation of q1 and q2= -2.65158560E-05 -1.83121134E-02

F1 and F2= 0.992809772 4.26198915E-02

Freqs. of fixation of A1 for In and St= 0.882388413 0.992908657

Freqs. of fixation of A2 for In and St= 0.112260178 0.00000000

Freqs. of segregation for In and St= 5.35140932E-03 7.09134340E-03

q1f= 0.112864159 q2f= 0.00000000

Index of gamma value 71 gamma for St popn= 116.799995

Means of q1 and q2= 0.110216260 4.83766897E-04

Variances of q1 and q2= 9.73485708E-02 2.04309581E-05

Covariance and correlation of q1 and q2= -2.53966991E-05 -1.80081092E-02

F1 and F2= 0.992657483 4.22535054E-02

Freqs. of fixation of A1 for In and St= 0.885895848 0.993008018

Freqs. of fixation of A2 for In and St= 0.108777493 0.00000000

Freqs. of segregation for In and St= 5.32665849E-03 6.99198246E-03

q1f= 0.109360017 q2f= 0.00000000

Index of gamma value 72 gamma for St popn= 118.464996

Means of q1 and q2= 0.106813565 4.75845038E-04

Variances of q1 and q2= 9.46889967E-02 1.99258720E-05

Covariance and correlation of q1 and q2= -2.43229933E-05 -1.77075546E-02

F1 and F2= 0.992501020 4.18946445E-02

Freqs. of fixation of A1 for In and St= 0.889304936 0.993104756

Freqs. of fixation of A2 for In and St= 0.105393179 0.00000000

Freqs. of segregation for In and St= 5.30188531E-03 6.89524412E-03

q1f= 0.105954938 q2f= 0.00000000

Index of gamma value 73 gamma for St popn= 120.129997

Means of q1 and q2= 0.103485949 4.68126353E-04

Variances of q1 and q2= 9.20658112E-02 1.94383301E-05

Covariance and correlation of q1 and q2= -2.32888178E-05 -1.74087975E-02

F1 and F2= 0.992338598 4.15431298E-02

Freqs. of fixation of A1 for In and St= 0.892639160 0.993199944

Freqs. of fixation of A2 for In and St= 0.102083825 0.00000000

Freqs. of segregation for In and St= 5.27701527E-03 6.80005550E-03

q1f= 0.102625385 q2f= 0.00000000

Index of gamma value 74 gamma for St popn= 121.794998

Means of q1 and q2= 0.100263998 4.60594572E-04

Variances of q1 and q2= 8.95049646E-02 1.89670700E-05

Covariance and correlation of q1 and q2= -2.22991966E-05 -1.71145517E-02

F1 and F2= 0.992172062 4.11985107E-02

Freqs. of fixation of A1 for In and St= 0.895868242 0.993292689

Freqs. of fixation of A2 for In and St= 9.88797769E-02 0.00000000

Freqs. of segregation for In and St= 5.25198132E-03 6.70731068E-03

q1f= 9.94018391E-02 q2f= 0.00000000

Index of gamma value 75 gamma for St popn= 123.459999

Means of q1 and q2= 9.71133336E-02 4.53251152E-04

Variances of q1 and q2= 8.69808123E-02 1.85118060E-05

Covariance and correlation of q1 and q2= -2.13460044E-05 -1.68221109E-02

F1 and F2= 0.991999269 4.08607870E-02

Freqs. of fixation of A1 for In and St= 0.899026036 0.993383527

Freqs. of fixation of A2 for In and St= 9.57469121E-02 0.00000000

Freqs. of segregation for In and St= 5.22705168E-03 6.61647320E-03

q1f= 9.62500125E-02 q2f= 0.00000000

Index of gamma value 76 gamma for St popn= 125.125000

Means of q1 and q2= 9.40857008E-02 4.46076068E-04

Variances of q1 and q2= 8.45367014E-02 1.80710758E-05

Covariance and correlation of q1 and q2= -2.04385506E-05 -1.65361892E-02

F1 and F2= 0.991823852 4.05292772E-02

Freqs. of fixation of A1 for In and St= 0.902061343 0.993472874

Freqs. of fixation of A2 for In and St= 9.27365795E-02 0.00000000

Freqs. of segregation for In and St= 5.20207733E-03 6.52712584E-03

q1f= 9.32215229E-02 q2f= 0.00000000

Index of gamma value 77 gamma for St popn= 126.789993

Means of q1 and q2= 9.11148712E-02 4.39080060E-04

Variances of q1 and q2= 8.21207017E-02 1.76451813E-05

Covariance and correlation of q1 and q2= -1.95625398E-05 -1.62512157E-02

F1 and F2= 0.991640806 4.02043611E-02

Freqs. of fixation of A1 for In and St= 0.905040145 0.993560016

Freqs. of fixation of A2 for In and St= 8.97830203E-02 0.00000000

Freqs. of segregation for In and St= 5.17683476E-03 6.43998384E-03

q1f= 9.02502313E-02 q2f= 0.00000000

Index of gamma value 78 gamma for St popn= 128.455002

Means of q1 and q2= 8.82309526E-02 4.32247645E-04

Variances of q1 and q2= 7.97586516E-02 1.72329746E-05

Covariance and correlation of q1 and q2= -1.87228798E-05 -1.59699526E-02

F1 and F2= 0.991452694 3.98855321E-02

Freqs. of fixation of A1 for In and St= 0.907932281 0.993645728

Freqs. of fixation of A2 for In and St= 8.69161263E-02 0.00000000

Freqs. of segregation for In and St= 5.15159220E-03 6.35427237E-03

q1f= 8.73662010E-02 q2f= 0.00000000

Index of gamma value 79 gamma for St popn= 130.119995

Means of q1 and q2= 8.54427591E-02 4.25572507E-04

Variances of q1 and q2= 7.74593204E-02 1.68338138E-05

Covariance and correlation of q1 and q2= -1.79204271E-05 -1.56935155E-02

F1 and F2= 0.991259873 3.95725295E-02

Freqs. of fixation of A1 for In and St= 0.910729110 0.993729830

Freqs. of fixation of A2 for In and St= 8.41446146E-02 0.00000000

Freqs. of segregation for In and St= 5.12627512E-03 6.27017021E-03

q1f= 8.45781863E-02 q2f= 0.00000000

Index of gamma value 80 gamma for St popn= 131.785004

Means of q1 and q2= 8.27270001E-02 4.19053220E-04

Variances of q1 and q2= 7.52049014E-02 1.64473640E-05

Covariance and correlation of q1 and q2= -1.71495831E-05 -1.54199293E-02

F1 and F2= 0.991060793 3.92653234E-02

Freqs. of fixation of A1 for In and St= 0.913453639 0.993812263

Freqs. of fixation of A2 for In and St= 8.14453363E-02 0.00000000

Freqs. of segregation for In and St= 5.10102510E-03 6.18773699E-03

q1f= 8.18629190E-02 q2f= 0.00000000

Index of gamma value 81 gamma for St popn= 133.449997

Means of q1 and q2= 8.00923258E-02 4.12682741E-04

Variances of q1 and q2= 7.30038136E-02 1.60729978E-05

Covariance and correlation of q1 and q2= -1.64111225E-05 -1.51501549E-02

F1 and F2= 0.990855634 3.89636680E-02

Freqs. of fixation of A1 for In and St= 0.916097105 0.993892491

Freqs. of fixation of A2 for In and St= 7.88268894E-02 0.00000000

Freqs. of segregation for In and St= 5.07600605E-03 6.10750914E-03

q1f= 7.92290568E-02 q2f= 0.00000000

Index of gamma value 82 gamma for St popn= 135.114990

Means of q1 and q2= 7.75369257E-02 4.06456529E-04

Variances of q1 and q2= 7.08558187E-02 1.57102349E-05

Covariance and correlation of q1 and q2= -1.57038648E-05 -1.48842670E-02

F1 and F2= 0.990644813 3.86674143E-02

Freqs. of fixation of A1 for In and St= 0.918661892 0.993971884

Freqs. of fixation of A2 for In and St= 7.62874708E-02 0.00000000

Freqs. of segregation for In and St= 5.05063683E-03 6.02811575E-03

q1f= 7.66747296E-02 q2f= 0.00000000

Index of gamma value 83 gamma for St popn= 136.779999

Means of q1 and q2= 7.50587136E-02 4.00369376E-04

Variances of q1 and q2= 6.87603801E-02 1.53585679E-05

Covariance and correlation of q1 and q2= -1.50265105E-05 -1.46222301E-02

F1 and F2= 0.990428150 3.83763611E-02

Freqs. of fixation of A1 for In and St= 0.921149492 0.994049191

Freqs. of fixation of A2 for In and St= 7.38250092E-02 0.00000000

Freqs. of segregation for In and St= 5.02549857E-03 5.95080853E-03

q1f= 7.41978884E-02 q2f= 0.00000000

Index of gamma value 84 gamma for St popn= 138.444992

Means of q1 and q2= 7.26661012E-02 3.94414936E-04

Variances of q1 and q2= 6.67258054E-02 1.50174665E-05

Covariance and correlation of q1 and q2= -1.43798025E-05 -1.43650686E-02

F1 and F2= 0.990206599 3.80903222E-02

Freqs. of fixation of A1 for In and St= 0.923551977 0.994125128

Freqs. of fixation of A2 for In and St= 7.14477971E-02 0.00000000

Freqs. of segregation for In and St= 5.00022620E-03 5.87487221E-03

q1f= 7.18068480E-02 q2f= 0.00000000

Index of gamma value 85 gamma for St popn= 140.110001

Means of q1 and q2= 7.03163221E-02 3.88598070E-04

Variances of q1 and q2= 6.47165477E-02 1.46869725E-05

Covariance and correlation of q1 and q2= -1.37550442E-05 -1.41087379E-02

F1 and F2= 0.989974439 3.78094576E-02

Freqs. of fixation of A1 for In and St= 0.925911486 0.994200051

Freqs. of fixation of A2 for In and St= 6.91133961E-02 0.00000000

Freqs. of segregation for In and St= 4.97511774E-03 5.79994917E-03

q1f= 6.94589615E-02 q2f= 0.00000000

Index of gamma value 86 gamma for St popn= 141.774994

Means of q1 and q2= 6.80485591E-02 3.82906350E-04

Variances of q1 and q2= 6.27671182E-02 1.43662292E-05

Covariance and correlation of q1 and q2= -1.31587958E-05 -1.38573013E-02

F1 and F2= 0.989737332 3.75332832E-02

Freqs. of fixation of A1 for In and St= 0.928189278 0.994273067

Freqs. of fixation of A2 for In and St= 6.68606684E-02 0.00000000

Freqs. of segregation for In and St= 4.95005399E-03 5.72693348E-03

q1f= 6.71932772E-02 q2f= 0.00000000

Index of gamma value 87 gamma for St popn= 143.440002

Means of q1 and q2= 6.58606216E-02 3.77335266E-04

Variances of q1 and q2= 6.08767085E-02 1.40548482E-05

Covariance and correlation of q1 and q2= -1.25898532E-05 -1.36107486E-02

F1 and F2= 0.989495158 3.72616984E-02

Freqs. of fixation of A1 for In and St= 0.930387437 0.994345009

Freqs. of fixation of A2 for In and St= 6.46874905E-02 0.00000000

Freqs. of segregation for In and St= 4.92507219E-03 5.65499067E-03

q1f= 6.50076568E-02 q2f= 0.00000000

Index of gamma value 88 gamma for St popn= 145.104996

Means of q1 and q2= 6.37306497E-02 3.71884875E-04

Variances of q1 and q2= 5.90272695E-02 1.37526695E-05

Covariance and correlation of q1 and q2= -1.20434943E-05 -1.33669600E-02

F1 and F2= 0.989244223 3.69947441E-02

Freqs. of fixation of A1 for In and St= 0.932527781 0.994415462

Freqs. of fixation of A2 for In and St= 6.25720844E-02 0.00000000

Freqs. of segregation for In and St= 4.90013510E-03 5.58453798E-03

q1f= 6.28802106E-02 q2f= 0.00000000

Index of gamma value 89 gamma for St popn= 146.769989

Means of q1 and q2= 6.16669729E-02 3.66551249E-04

Variances of q1 and q2= 5.72268665E-02 1.34592919E-05

Covariance and correlation of q1 and q2= -1.15206713E-05 -1.31270364E-02

F1 and F2= 0.988986373 3.67321819E-02

Freqs. of fixation of A1 for In and St= 0.934602141 0.994484961

Freqs. of fixation of A2 for In and St= 6.05227388E-02 0.00000000

Freqs. of segregation for In and St= 4.87511978E-03 5.51503897E-03

q1f= 6.08192384E-02 q2f= 0.00000000

Index of gamma value 90 gamma for St popn= 148.434998

Means of q1 and q2= 5.96581064E-02 3.61331797E-04

Variances of q1 and q2= 5.54661863E-02 1.31744500E-05

Covariance and correlation of q1 and q2= -1.10186638E-05 -1.28898704E-02

F1 and F2= 0.988719404 3.64739895E-02

Freqs. of fixation of A1 for In and St= 0.936621845 0.994553149

Freqs. of fixation of A2 for In and St= 5.85280433E-02 0.00000000

Freqs. of segregation for In and St= 4.85011190E-03 5.44685125E-03

q1f= 5.88132925E-02 q2f= 0.00000000

Index of gamma value 91 gamma for St popn= 150.099991

Means of q1 and q2= 5.77217601E-02 3.56218894E-04

Variances of q1 and q2= 5.37615791E-02 1.28976208E-05

Covariance and correlation of q1 and q2= -1.05399731E-05 -1.26575222E-02

F1 and F2= 0.988446712 3.62199098E-02

Freqs. of fixation of A1 for In and St= 0.938568950 0.994620025

Freqs. of fixation of A2 for In and St= 5.66055737E-02 0.00000000

Freqs. of segregation for In and St= 4.82547656E-03 5.37997484E-03

q1f= 5.68800457E-02 q2f= 0.00000000

Index of gamma value 92 gamma for St popn= 151.764999

Means of q1 and q2= 5.58369197E-02 3.51214258E-04

Variances of q1 and q2= 5.20952083E-02 1.26287287E-05

Covariance and correlation of q1 and q2= -1.00803518E-05 -1.24278776E-02

F1 and F2= 0.988164663 3.59699689E-02

Freqs. of fixation of A1 for In and St= 0.940464675 0.994684756

Freqs. of fixation of A2 for In and St= 5.47344238E-02 0.00000000

Freqs. of segregation for In and St= 4.80090082E-03 5.31524420E-03

q1f= 5.49984686E-02 q2f= 0.00000000

Index of gamma value 93 gamma for St popn= 153.429993

Means of q1 and q2= 5.40210344E-02 3.46311048E-04

Variances of q1 and q2= 5.04832119E-02 1.23673071E-05

Covariance and correlation of q1 and q2= -9.64228275E-06 -1.22030620E-02

F1 and F2= 0.987876415 3.57239321E-02

Freqs. of fixation of A1 for In and St= 0.942291975 0.994749725

Freqs. of fixation of A2 for In and St= 5.29319644E-02 0.00000000

Freqs. of segregation for In and St= 4.77606058E-03 5.25027514E-03

q1f= 5.31859845E-02 q2f= 0.00000000

Index of gamma value 94 gamma for St popn= 155.095001

Means of q1 and q2= 5.22627756E-02 3.41507752E-04

Variances of q1 and q2= 4.89162058E-02 1.21131543E-05

Covariance and correlation of q1 and q2= -9.22321669E-06 -1.19819473E-02

F1 and F2= 0.987580180 3.54817510E-02

Freqs. of fixation of A1 for In and St= 0.944061279 0.994813025

Freqs. of fixation of A2 for In and St= 5.11869080E-02 0.00000000

Freqs. of segregation for In and St= 4.75181267E-03 5.18697500E-03

q1f= 5.14312983E-02 q2f= 0.00000000

Index of gamma value 95 gamma for St popn= 156.759995

Means of q1 and q2= 5.05422018E-02 3.36805591E-04

Variances of q1 and q2= 4.73768674E-02 1.18662037E-05

Covariance and correlation of q1 and q2= -8.81929282E-06 -1.17623666E-02

F1 and F2= 0.987271309 3.52434814E-02

Freqs. of fixation of A1 for In and St= 0.945793033 0.994874656

Freqs. of fixation of A2 for In and St= 4.94794175E-02 0.00000000

Freqs. of segregation for In and St= 4.72754985E-03 5.12534380E-03

q1f= 4.97144461E-02 q2f= 0.00000000

Index of gamma value 96 gamma for St popn= 158.425003

Means of q1 and q2= 4.88856174E-02 3.32196389E-04

Variances of q1 and q2= 4.58893105E-02 1.16259371E-05

Covariance and correlation of q1 and q2= -8.43449288E-06 -1.15475338E-02

F1 and F2= 0.986955762 3.50088105E-02

Freqs. of fixation of A1 for In and St= 0.947461247 0.994935930

Freqs. of fixation of A2 for In and St= 4.78356555E-02 0.00000000

Freqs. of segregation for In and St= 4.70309705E-03 5.06407022E-03

q1f= 4.80616949E-02 q2f= 0.00000000

Index of gamma value 97 gamma for St popn= 160.089996

Means of q1 and q2= 4.72821034E-02 3.27679125E-04

Variances of q1 and q2= 4.44442928E-02 1.13922060E-05

Covariance and correlation of q1 and q2= -8.06646949E-06 -1.13363061E-02

F1 and F2= 0.986631334 3.47777456E-02

Freqs. of fixation of A1 for In and St= 0.949076295 0.994995773

Freqs. of fixation of A2 for In and St= 4.62447442E-02 0.00000000

Freqs. of segregation for In and St= 4.67896089E-03 5.00422716E-03

q1f= 4.64621373E-02 q2f= 0.00000000

Index of gamma value 98 gamma for St popn= 161.754990

Means of q1 and q2= 4.57301550E-02 3.23252025E-04

Variances of q1 and q2= 4.30409685E-02 1.11648005E-05

Covariance and correlation of q1 and q2= -7.71454688E-06 -1.11286873E-02

F1 and F2= 0.986298025 3.45501639E-02

Freqs. of fixation of A1 for In and St= 0.950639844 0.995055079

Freqs. of fixation of A2 for In and St= 4.47052009E-02 0.00000000

Freqs. of segregation for In and St= 4.65495512E-03 4.94492054E-03

q1f= 4.49142754E-02 q2f= 0.00000000

Index of gamma value 99 gamma for St popn= 163.419998

Means of q1 and q2= 4.42281999E-02 3.18911450E-04

Variances of q1 and q2= 4.16783653E-02 1.09434613E-05

Covariance and correlation of q1 and q2= -7.37798928E-06 -1.09245945E-02

F1 and F2= 0.985955298 3.43259908E-02

Freqs. of fixation of A1 for In and St= 0.952153504 0.995112777

Freqs. of fixation of A2 for In and St= 4.32154424E-02 0.00000000

Freqs. of segregation for In and St= 4.63105366E-03 4.88722324E-03

q1f= 4.34165075E-02 q2f= 0.00000000

Index of gamma value 100 gamma for St popn= 165.084991

Means of q1 and q2= 4.27748971E-02 3.14656209E-04

Variances of q1 and q2= 4.03557122E-02 1.07280302E-05

Covariance and correlation of q1 and q2= -7.05620005E-06 -1.07240397E-02

F1 and F2= 0.985602856 3.41051817E-02

Freqs. of fixation of A1 for In and St= 0.953618586 0.995169520

Freqs. of fixation of A2 for In and St= 4.17741239E-02 0.00000000

Freqs. of segregation for In and St= 4.60729003E-03 4.83047962E-03

q1f= 4.19674814E-02 q2f= 0.00000000

Index of gamma value 101 gamma for St popn= 166.750000

Means of q1 and q2= 4.13687378E-02 3.10483796E-04

Variances of q1 and q2= 3.90720628E-02 1.05182926E-05

Covariance and correlation of q1 and q2= -6.74852708E-06 -1.05269663E-02

F1 and F2= 0.985240996 3.38876285E-02

Freqs. of fixation of A1 for In and St= 0.955036581 0.995225847

Freqs. of fixation of A2 for In and St= 4.03797477E-02 0.00000000

Freqs. of segregation for In and St= 4.58367169E-03 4.77415323E-03

q1f= 4.05656882E-02 q2f= 0.00000000

Index of gamma value 102 gamma for St popn= 168.414993

Means of q1 and q2= 3.99999358E-02 3.06393689E-04

Variances of q1 and q2= 3.78187783E-02 1.03141265E-05

Covariance and correlation of q1 and q2= -6.45304362E-06 -1.03322426E-02

F1 and F2= 0.984865606 3.36733013E-02

Freqs. of fixation of A1 for In and St= 0.956417382 0.995280981

Freqs. of fixation of A2 for In and St= 3.90225835E-02 0.00000000

Freqs. of segregation for In and St= 4.56003472E-03 4.71901894E-03

q1f= 3.92013453E-02 q2f= 0.00000000

Index of gamma value 103 gamma for St popn= 170.080002

Means of q1 and q2= 3.86757404E-02 3.02381959E-04

Variances of q1 and q2= 3.66028808E-02 1.01152746E-05

Covariance and correlation of q1 and q2= -6.17055002E-06 -1.01409210E-02

F1 and F2= 0.984479606 3.34620960E-02

Freqs. of fixation of A1 for In and St= 0.957753360 0.995334685

Freqs. of fixation of A2 for In and St= 3.77098247E-02 0.00000000

Freqs. of segregation for In and St= 4.53681499E-03 4.66531515E-03

q1f= 3.78816873E-02 q2f= 0.00000000

Index of gamma value 104 gamma for St popn= 171.744995

Means of q1 and q2= 3.73947807E-02 2.98446539E-04

Variances of q1 and q2= 3.54234539E-02 9.92155492E-06

Covariance and correlation of q1 and q2= -5.90047784E-06 -9.95295029E-03

F1 and F2= 0.984082937 3.32539156E-02

Freqs. of fixation of A1 for In and St= 0.959046364 0.995388031

Freqs. of fixation of A2 for In and St= 3.64401266E-02 0.00000000

Freqs. of segregation for In and St= 4.51350957E-03 4.61196899E-03

q1f= 3.66053432E-02 q2f= 0.00000000

Index of gamma value 105 gamma for St popn= 173.410004

Means of q1 and q2= 3.61638702E-02 2.94584548E-04

Variances of q1 and q2= 3.42871398E-02 9.73276110E-06

Covariance and correlation of q1 and q2= -5.64355287E-06 -9.76942386E-03

F1 and F2= 0.983678460 3.30486745E-02

Freqs. of fixation of A1 for In and St= 0.960289240 0.995440006

Freqs. of fixation of A2 for In and St= 3.52202244E-02 0.00000000

Freqs. of segregation for In and St= 4.49053571E-03 4.55999374E-03

q1f= 3.53790969E-02 q2f= 0.00000000

Index of gamma value 106 gamma for St popn= 175.074997

Means of q1 and q2= 3.49654220E-02 2.90796539E-04

Variances of q1 and q2= 3.31779644E-02 9.54883399E-06

Covariance and correlation of q1 and q2= -5.39673238E-06 -9.58806183E-03

F1 and F2= 0.983259380 3.28463726E-02

Freqs. of fixation of A1 for In and St= 0.961499929 0.995491564

Freqs. of fixation of A2 for In and St= 3.40326577E-02 0.00000000

Freqs. of segregation for In and St= 4.46741283E-03 4.50843573E-03

q1f= 3.41853797E-02 q2f= 0.00000000

Index of gamma value 107 gamma for St popn= 176.739990

Means of q1 and q2= 3.38064060E-02 2.87079136E-04

Variances of q1 and q2= 3.21026519E-02 9.36955894E-06

Covariance and correlation of q1 and q2= -5.16079035E-06 -9.40992590E-03

F1 and F2= 0.982828438 3.26469205E-02

Freqs. of fixation of A1 for In and St= 0.962670982 0.995541871

Freqs. of fixation of A2 for In and St= 3.28843370E-02 0.00000000

Freqs. of segregation for In and St= 4.44468111E-03 4.45812941E-03

q1f= 3.30311507E-02 q2f= 0.00000000

Index of gamma value 108 gamma for St popn= 178.404999

Means of q1 and q2= 3.26856524E-02 2.83431349E-04

Variances of q1 and q2= 3.10603809E-02 9.19480954E-06

Covariance and correlation of q1 and q2= -4.93528887E-06 -9.23501886E-03

F1 and F2= 0.982385516 3.24502438E-02

Freqs. of fixation of A1 for In and St= 0.963803887 0.995591581

Freqs. of fixation of A2 for In and St= 3.17741074E-02 0.00000000

Freqs. of segregation for In and St= 4.42200527E-03 4.40841913E-03

q1f= 3.19152363E-02 q2f= 0.00000000

Index of gamma value 109 gamma for St popn= 180.069992

Means of q1 and q2= 3.16095278E-02 2.79849570E-04

Variances of q1 and q2= 3.00573763E-02 9.02436841E-06

Covariance and correlation of q1 and q2= -4.72086413E-06 -9.06436890E-03

F1 and F2= 0.981934547 3.22562382E-02

Freqs. of fixation of A1 for In and St= 0.964892447 0.995640934

Freqs. of fixation of A2 for In and St= 3.07082795E-02 0.00000000

Freqs. of segregation for In and St= 4.39927354E-03 4.35906649E-03

q1f= 3.08439713E-02 q2f= 0.00000000

Index of gamma value 110 gamma for St popn= 181.735001

Means of q1 and q2= 3.05540822E-02 2.76335923E-04

Variances of q1 and q2= 2.90714409E-02 8.85826466E-06

Covariance and correlation of q1 and q2= -4.51371125E-06 -8.89459439E-03

F1 and F2= 0.981462479 3.20650078E-02

Freqs. of fixation of A1 for In and St= 0.965959847 0.995689034

Freqs. of fixation of A2 for In and St= 2.96630394E-02 0.00000000

Freqs. of segregation for In and St= 4.37711366E-03 4.31096554E-03

q1f= 2.97934487E-02 q2f= 0.00000000

Index of gamma value 111 gamma for St popn= 183.399994

Means of q1 and q2= 2.95410194E-02 2.72885780E-04

Variances of q1 and q2= 2.81231161E-02 8.69621999E-06

Covariance and correlation of q1 and q2= -4.31681110E-06 -8.72903224E-03

F1 and F2= 0.980981410 3.18763182E-02

Freqs. of fixation of A1 for In and St= 0.966985345 0.995736420

Freqs. of fixation of A2 for In and St= 2.86599733E-02 0.00000000

Freqs. of segregation for In and St= 4.35468182E-03 4.26357985E-03

q1f= 2.87853237E-02 q2f= 0.00000000

Index of gamma value 112 gamma for St popn= 185.065002

Means of q1 and q2= 2.85543632E-02 2.69498909E-04

Variances of q1 and q2= 2.71976106E-02 8.53817801E-06

Covariance and correlation of q1 and q2= -4.12756117E-06 -8.56535789E-03

F1 and F2= 0.980482340 3.16902176E-02

Freqs. of fixation of A1 for In and St= 0.967984319 0.995783091

Freqs. of fixation of A2 for In and St= 2.76831854E-02 0.00000000

Freqs. of segregation for In and St= 4.33249585E-03 4.21690941E-03

q1f= 2.78036445E-02 q2f= 0.00000000

Index of gamma value 113 gamma for St popn= 186.729996

Means of q1 and q2= 2.76076905E-02 2.66172050E-04

Variances of q1 and q2= 2.63079107E-02 8.38394408E-06

Covariance and correlation of q1 and q2= -3.94771405E-06 -8.40578601E-03

F1 and F2= 0.979974508 3.15066017E-02

Freqs. of fixation of A1 for In and St= 0.968943357 0.995829523

Freqs. of fixation of A2 for In and St= 2.67461911E-02 0.00000000

Freqs. of segregation for In and St= 4.31045145E-03 4.17047739E-03

q1f= 2.68619768E-02 q2f= 0.00000000

Index of gamma value 114 gamma for St popn= 188.394989

Means of q1 and q2= 2.66856309E-02 2.62906280E-04

Variances of q1 and q2= 2.54396759E-02 8.23349365E-06

Covariance and correlation of q1 and q2= -3.77485071E-06 -8.24806187E-03

F1 and F2= 0.979447067 3.13254558E-02

Freqs. of fixation of A1 for In and St= 0.969877481 0.995874822

Freqs. of fixation of A2 for In and St= 2.58336738E-02 0.00000000

Freqs. of segregation for In and St= 4.28884476E-03 4.12517786E-03

q1f= 2.59449482E-02 q2f= 0.00000000

Index of gamma value 115 gamma for St popn= 190.059998

Means of q1 and q2= 2.58011986E-02 2.59697699E-04

Variances of q1 and q2= 2.46054027E-02 8.08661389E-06

Covariance and correlation of q1 and q2= -3.61060188E-06 -8.09433218E-03

F1 and F2= 0.978910565 3.11466530E-02

Freqs. of fixation of A1 for In and St= 0.970774114 0.995918632

Freqs. of fixation of A2 for In and St= 2.49585845E-02 0.00000000

Freqs. of segregation for In and St= 4.26730141E-03 4.08136845E-03

q1f= 2.50655469E-02 q2f= 0.00000000

Index of gamma value 116 gamma for St popn= 191.724991

Means of q1 and q2= 2.49396488E-02 2.56547559E-04

Variances of q1 and q2= 2.37912685E-02 7.94330299E-06

Covariance and correlation of q1 and q2= -3.45270746E-06 -7.94237573E-03

F1 and F2= 0.978353381 3.09702456E-02

Freqs. of fixation of A1 for In and St= 0.971647918 0.995962679

Freqs. of fixation of A2 for In and St= 2.41062585E-02 0.00000000

Freqs. of segregation for In and St= 4.24582325E-03 4.03732061E-03

q1f= 2.42090449E-02 q2f= 0.00000000

Index of gamma value 117 gamma for St popn= 193.389999

Means of q1 and q2= 2.41135340E-02 2.53451173E-04

Variances of q1 and q2= 2.30093449E-02 7.80332903E-06

Covariance and correlation of q1 and q2= -3.30269586E-06 -7.79428892E-03

F1 and F2= 0.977786601 3.07960976E-02

Freqs. of fixation of A1 for In and St= 0.972486258 0.996005952

Freqs. of fixation of A2 for In and St= 2.32891832E-02 0.00000000

Freqs. of segregation for In and St= 4.22455929E-03 3.99404764E-03

q1f= 2.33879872E-02 q2f= 0.00000000

Index of gamma value 118 gamma for St popn= 195.054993

Means of q1 and q2= 2.33086757E-02 2.50410667E-04

Variances of q1 and q2= 2.22462919E-02 7.66671747E-06

Covariance and correlation of q1 and q2= -3.15846546E-06 -7.64790643E-03

F1 and F2= 0.977198303 3.06242444E-02

Freqs. of fixation of A1 for In and St= 0.973303318 0.996047556

Freqs. of fixation of A2 for In and St= 2.24932488E-02 0.00000000

Freqs. of segregation for In and St= 4.20343317E-03 3.95244360E-03

q1f= 2.25881971E-02 q2f= 0.00000000

Index of gamma value 119 gamma for St popn= 196.720001

Means of q1 and q2= 2.25308072E-02 2.47422780E-04

Variances of q1 and q2= 2.15076841E-02 7.53329869E-06

Covariance and correlation of q1 and q2= -3.02061289E-06 -7.50422152E-03

F1 and F2= 0.976593494 3.04546040E-02

Freqs. of fixation of A1 for In and St= 0.974093676 0.996089637

Freqs. of fixation of A2 for In and St= 2.17241608E-02 0.00000000

Freqs. of segregation for In and St= 4.18216363E-03 3.91036272E-03

q1f= 2.18153968E-02 q2f= 0.00000000

Index of gamma value 120 gamma for St popn= 198.384995

Means of q1 and q2= 2.17791703E-02 2.44487193E-04

Variances of q1 and q2= 2.07929220E-02 7.40300675E-06

Covariance and correlation of q1 and q2= -2.88889282E-06 -7.36325048E-03

F1 and F2= 0.975971818 3.02871373E-02

Freqs. of fixation of A1 for In and St= 0.974857450 0.996131063

Freqs. of fixation of A2 for In and St= 2.09811591E-02 0.00000000

Freqs. of segregation for In and St= 4.16139141E-03 3.86893749E-03

q1f= 2.10688356E-02 q2f= 0.00000000

Index of gamma value 121 gamma for St popn= 200.049988

Means of q1 and q2= 2.10589953E-02 2.41601403E-04

Variances of q1 and q2= 2.01071389E-02 7.27569613E-06

Covariance and correlation of q1 and q2= -2.76382320E-06 -7.22599821E-03

F1 and F2= 0.975340128 3.01217381E-02

Freqs. of fixation of A1 for In and St= 0.975589871 0.996171117

Freqs. of fixation of A2 for In and St= 2.02694330E-02 0.00000000

Freqs. of segregation for In and St= 4.14069556E-03 3.82888317E-03

q1f= 2.03537103E-02 q2f= 0.00000000

Index of gamma value 122 gamma for St popn= 201.714996

Means of q1 and q2= 2.03570556E-02 2.38766064E-04

Variances of q1 and q2= 1.94377732E-02 7.15135729E-06

Covariance and correlation of q1 and q2= -2.64350365E-06 -7.09026447E-03

F1 and F2= 0.974683821 2.99584679E-02

Freqs. of fixation of A1 for In and St= 0.976303935 0.996211112

Freqs. of fixation of A2 for In and St= 1.95758510E-02 0.00000000

Freqs. of segregation for In and St= 4.12021391E-03 3.78888845E-03

q1f= 1.96568426E-02 q2f= 0.00000000

Index of gamma value 123 gamma for St popn= 203.379990

Means of q1 and q2= 1.96729060E-02 2.35979911E-04

Variances of q1 and q2= 1.87844802E-02 7.02989519E-06

Covariance and correlation of q1 and q2= -2.52775203E-06 -6.95601385E-03

F1 and F2= 0.974001646 2.97972597E-02

Freqs. of fixation of A1 for In and St= 0.977000117 0.996250272

Freqs. of fixation of A2 for In and St= 1.88999362E-02 0.00000000

Freqs. of segregation for In and St= 4.09994647E-03 3.74972820E-03

q1f= 1.89777445E-02 q2f= 0.00000000

Index of gamma value 124 gamma for St popn= 205.044998

Means of q1 and q2= 1.90234892E-02 2.33239305E-04

Variances of q1 and q2= 1.81636401E-02 6.91113610E-06

Covariance and correlation of q1 and q2= -2.41862563E-06 -6.82641193E-03

F1 and F2= 0.973316550 2.96380073E-02

Freqs. of fixation of A1 for In and St= 0.977661967 0.996289253

Freqs. of fixation of A2 for In and St= 1.82585698E-02 0.00000000

Freqs. of segregation for In and St= 4.07946296E-03 3.71074677E-03

q1f= 1.83333606E-02 q2f= 0.00000000

Index of gamma value 125 gamma for St popn= 206.709991

Means of q1 and q2= 1.83903482E-02 2.30546182E-04

Variances of q1 and q2= 1.75575968E-02 6.79510640E-06

Covariance and correlation of q1 and q2= -2.31361082E-06 -6.69822376E-03

F1 and F2= 0.972604513 2.94807460E-02

Freqs. of fixation of A1 for In and St= 0.978307068 0.996326864

Freqs. of fixation of A2 for In and St= 1.76333729E-02 0.00000000

Freqs. of segregation for In and St= 4.05955873E-03 3.67313623E-03

q1f= 1.77052487E-02 q2f= 0.00000000

Index of gamma value 126 gamma for St popn= 208.375000

Means of q1 and q2= 1.77786332E-02 2.27898199E-04

Variances of q1 and q2= 1.69713851E-02 6.68168786E-06

Covariance and correlation of q1 and q2= -2.21324512E-06 -6.57245703E-03

F1 and F2= 0.971873045 2.93254219E-02

Freqs. of fixation of A1 for In and St= 0.978931129 0.996364892

Freqs. of fixation of A2 for In and St= 1.70294773E-02 0.00000000

Freqs. of segregation for In and St= 4.03939374E-03 3.63510847E-03

q1f= 1.70985460E-02 q2f= 0.00000000

Index of gamma value 127 gamma for St popn= 210.039993

Means of q1 and q2= 1.71876699E-02 2.25294527E-04

Variances of q1 and q2= 1.64044313E-02 6.57080454E-06

Covariance and correlation of q1 and q2= -2.11732959E-06 -6.44909265E-03

F1 and F2= 0.971121490 2.91719697E-02

Freqs. of fixation of A1 for In and St= 0.979533851 0.996401548

Freqs. of fixation of A2 for In and St= 1.64461993E-02 0.00000000

Freqs. of segregation for In and St= 4.01994959E-03 3.59845161E-03

q1f= 1.65125802E-02 q2f= 0.00000000

Index of gamma value 128 gamma for St popn= 211.705002

Means of q1 and q2= 1.66113619E-02 2.22734860E-04

Variances of q1 and q2= 1.58509165E-02 6.46242279E-06

Covariance and correlation of q1 and q2= -2.02499382E-06 -6.32701488E-03

F1 and F2= 0.970340073 2.90204342E-02

Freqs. of fixation of A1 for In and St= 0.980122566 0.996438265

Freqs. of fixation of A2 for In and St= 1.58774853E-02 0.00000000

Freqs. of segregation for In and St= 3.99994850E-03 3.56173515E-03

q1f= 1.59412492E-02 q2f= 0.00000000

Index of gamma value 129 gamma for St popn= 213.369995

Means of q1 and q2= 1.60546731E-02 2.20217626E-04

Variances of q1 and q2= 1.53156919E-02 6.35643664E-06

Covariance and correlation of q1 and q2= -1.93675987E-06 -6.20726962E-03

F1 and F2= 0.969536543 2.88706999E-02

Freqs. of fixation of A1 for In and St= 0.980690777 0.996473730

Freqs. of fixation of A2 for In and St= 1.53282583E-02 0.00000000

Freqs. of segregation for In and St= 3.98096442E-03 3.52627039E-03

q1f= 1.53895235E-02 q2f= 0.00000000

Index of gamma value 130 gamma for St popn= 215.034988

Means of q1 and q2= 1.55272596E-02 2.17740890E-04

Variances of q1 and q2= 1.48081910E-02 6.25274015E-06

Covariance and correlation of q1 and q2= -1.85370084E-06 -6.09190948E-03

F1 and F2= 0.968731642 2.87226792E-02

Freqs. of fixation of A1 for In and St= 0.981230378 0.996509016

Freqs. of fixation of A2 for In and St= 1.48081454E-02 0.00000000

Freqs. of segregation for In and St= 3.96147650E-03 3.49098444E-03

q1f= 1.48670413E-02 q2f= 0.00000000

Index of gamma value 131 gamma for St popn= 216.699997

Means of q1 and q2= 1.50126582E-02 2.15304564E-04

Variances of q1 and q2= 1.43125281E-02 6.15132194E-06

Covariance and correlation of q1 and q2= -1.77368292E-06 -5.97769255E-03

F1 and F2= 0.967894673 2.85764784E-02

Freqs. of fixation of A1 for In and St= 0.981757045 0.996544480

Freqs. of fixation of A2 for In and St= 1.43007524E-02 0.00000000

Freqs. of segregation for In and St= 3.94220278E-03 3.45551968E-03

q1f= 1.43573517E-02 q2f= 0.00000000

Index of gamma value 132 gamma for St popn= 218.364990

Means of q1 and q2= 1.45106250E-02 2.12909465E-04

Variances of q1 and q2= 1.38285058E-02 6.05215700E-06

Covariance and correlation of q1 and q2= -1.69662485E-06 -5.86466398E-03

F1 and F2= 0.967023849 2.84320191E-02

Freqs. of fixation of A1 for In and St= 0.982270956 0.996578634

Freqs. of fixation of A2 for In and St= 1.38058281E-02 0.00000000

Freqs. of segregation for In and St= 3.92321590E-03 3.42136621E-03

q1f= 1.38602052E-02 q2f= 0.00000000

Index of gamma value 133 gamma for St popn= 220.029999

Means of q1 and q2= 1.40305888E-02 2.10552389E-04

Variances of q1 and q2= 1.33653302E-02 5.95510801E-06

Covariance and correlation of q1 and q2= -1.62355627E-06 -5.75483264E-03

F1 and F2= 0.966140628 2.82892156E-02

Freqs. of fixation of A1 for In and St= 0.982762933 0.996611893

Freqs. of fixation of A2 for In and St= 1.33327665E-02 0.00000000

Freqs. of segregation for In and St= 3.90430074E-03 3.38810682E-03

q1f= 1.33850258E-02 q2f= 0.00000000

Index of gamma value 134 gamma for St popn= 221.694992

Means of q1 and q2= 1.35621065E-02 2.08233643E-04

Variances of q1 and q2= 1.29129030E-02 5.86016949E-06

Covariance and correlation of q1 and q2= -1.55314808E-06 -5.64606441E-03

F1 and F2= 0.965221524 2.81481426E-02

Freqs. of fixation of A1 for In and St= 0.983243287 0.996645570

Freqs. of fixation of A2 for In and St= 1.28711741E-02 0.00000000

Freqs. of segregation for In and St= 3.88553925E-03 3.35443020E-03

q1f= 1.29213808E-02 q2f= 0.00000000

Index of gamma value 135 gamma for St popn= 223.360001

Means of q1 and q2= 1.31143089E-02 2.05951656E-04

Variances of q1 and q2= 1.24801500E-02 5.76723733E-06

Covariance and correlation of q1 and q2= -1.48640379E-06 -5.54042356E-03

F1 and F2= 0.964289784 2.80086379E-02

Freqs. of fixation of A1 for In and St= 0.983702958 0.996678412

Freqs. of fixation of A2 for In and St= 1.24301314E-02 0.00000000

Freqs. of segregation for In and St= 3.86691093E-03 3.32158804E-03

q1f= 1.24783842E-02 q2f= 0.00000000

Index of gamma value 136 gamma for St popn= 225.024994

Means of q1 and q2= 1.26772048E-02 2.03707066E-04

Variances of q1 and q2= 1.20573901E-02 5.67632014E-06

Covariance and correlation of q1 and q2= -1.42208137E-06 -5.43581136E-03

F1 and F2= 0.963320136 2.78707892E-02

Freqs. of fixation of A1 for In and St= 0.984151483 0.996710539

Freqs. of fixation of A2 for In and St= 1.19996965E-02 0.00000000

Freqs. of segregation for In and St= 3.84882092E-03 3.28946114E-03

q1f= 1.20460596E-02 q2f= 0.00000000

Index of gamma value 137 gamma for St popn= 226.690002

Means of q1 and q2= 1.22595159E-02 2.01497460E-04

Variances of q1 and q2= 1.16531579E-02 5.58729562E-06

Covariance and correlation of q1 and q2= -1.36111191E-06 -5.33422315E-03

F1 and F2= 0.962337613 2.77344529E-02

Freqs. of fixation of A1 for In and St= 0.984581053 0.996742070

Freqs. of fixation of A2 for In and St= 1.15885520E-02 0.00000000

Freqs. of segregation for In and St= 3.83039471E-03 3.25793028E-03

q1f= 1.16331112E-02 q2f= 0.00000000

Index of gamma value 138 gamma for St popn= 228.354996

Means of q1 and q2= 1.18517075E-02 1.99323302E-04

Variances of q1 and q2= 1.12581924E-02 5.50017194E-06

Covariance and correlation of q1 and q2= -1.30233627E-06 -5.23360167E-03

F1 and F2= 0.961314797 2.75997259E-02

Freqs. of fixation of A1 for In and St= 0.985000730 0.996774256

Freqs. of fixation of A2 for In and St= 1.11872079E-02 0.00000000

Freqs. of segregation for In and St= 3.81206255E-03 3.22574377E-03

q1f= 1.12300180E-02 q2f= 0.00000000

Index of gamma value 139 gamma for St popn= 230.019989

Means of q1 and q2= 1.14621595E-02 1.97183035E-04

Variances of q1 and q2= 1.08807087E-02 5.41485542E-06

Covariance and correlation of q1 and q2= -1.24664052E-06 -5.13593107E-03

F1 and F2= 0.960278988 2.74664778E-02

Freqs. of fixation of A1 for In and St= 0.985401630 0.996805012

Freqs. of fixation of A2 for In and St= 1.08040003E-02 0.00000000

Freqs. of segregation for In and St= 3.79436929E-03 3.19498777E-03

q1f= 1.08451508E-02 q2f= 0.00000000

Index of gamma value 140 gamma for St popn= 231.684998

Means of q1 and q2= 1.10816834E-02 1.95076689E-04

Variances of q1 and q2= 1.05117625E-02 5.33133607E-06

Covariance and correlation of q1 and q2= -1.19292542E-06 -5.03915269E-03

F1 and F2= 0.959200501 2.73347683E-02

Freqs. of fixation of A1 for In and St= 0.985793889 0.996835351

Freqs. of fixation of A2 for In and St= 1.04297837E-02 0.00000000

Freqs. of segregation for In and St= 3.77632771E-03 3.16464901E-03

q1f= 1.04693193E-02 q2f= 0.00000000

Index of gamma value 141 gamma for St popn= 233.349991

Means of q1 and q2= 1.07142478E-02 1.93003056E-04

Variances of q1 and q2= 1.01552596E-02 5.24954476E-06

Covariance and correlation of q1 and q2= -1.14158024E-06 -4.94424673E-03

F1 and F2= 0.958092809 2.72045340E-02

Freqs. of fixation of A1 for In and St= 0.986172676 0.996865571

Freqs. of fixation of A2 for In and St= 1.00685144E-02 0.00000000

Freqs. of segregation for In and St= 3.75880953E-03 3.13442945E-03

q1f= 1.01065030E-02 q2f= 0.00000000

Index of gamma value 142 gamma for St popn= 235.014999

Means of q1 and q2= 1.03634354E-02 1.90961669E-04

Variances of q1 and q2= 9.81473736E-03 5.16943510E-06

Covariance and correlation of q1 and q2= -1.09294342E-06 -4.85217851E-03

F1 and F2= 0.956971943 2.70757079E-02

Freqs. of fixation of A1 for In and St= 0.986534894 0.996895015

Freqs. of fixation of A2 for In and St= 9.72374994E-03 0.00000000

Freqs. of segregation for In and St= 3.74135654E-03 3.10498476E-03

q1f= 9.76026710E-03 q2f= 0.00000000

Index of gamma value 143 gamma for St popn= 236.679993

Means of q1 and q2= 1.00206658E-02 1.88951934E-04

Variances of q1 and q2= 9.48182307E-03 5.09097845E-06

Covariance and correlation of q1 and q2= -1.04601520E-06 -4.76092612E-03

F1 and F2= 0.955804706 2.69483365E-02

Freqs. of fixation of A1 for In and St= 0.986889184 0.996925175

Freqs. of fixation of A2 for In and St= 9.38695762E-03 0.00000000

Freqs. of segregation for In and St= 3.72385886E-03 3.07482481E-03

q1f= 9.42204427E-03 q2f= 0.00000000

Index of gamma value 144 gamma for St popn= 238.345001

Means of q1 and q2= 9.68964584E-03 1.86973077E-04

Variances of q1 and q2= 9.16016847E-03 5.01412160E-06

Covariance and correlation of q1 and q2= -1.00115278E-06 -4.67144465E-03

F1 and F2= 0.954606175 2.68223602E-02

Freqs. of fixation of A1 for In and St= 0.987231731 0.996954143

Freqs. of fixation of A2 for In and St= 9.06182174E-03 0.00000000

Freqs. of segregation for In and St= 3.70644685E-03 3.04585695E-03

q1f= 9.09553375E-03 q2f= 0.00000000

Index of gamma value 145 gamma for St popn= 240.009995

Means of q1 and q2= 9.36998241E-03 1.85024808E-04

Variances of q1 and q2= 8.84940755E-03 4.93883545E-06

Covariance and correlation of q1 and q2= -9.58267151E-07 -4.58371080E-03

F1 and F2= 0.953375399 2.66977660E-02

Freqs. of fixation of A1 for In and St= 0.987562656 0.996982217

Freqs. of fixation of A2 for In and St= 8.74794926E-03 0.00000000

Freqs. of segregation for In and St= 3.68939433E-03 3.01778316E-03

q1f= 8.78034346E-03 q2f= 0.00000000

Index of gamma value 146 gamma for St popn= 241.674988

Means of q1 and q2= 9.06497240E-03 1.83105585E-04

Variances of q1 and q2= 8.55280831E-03 4.86504769E-06

Covariance and correlation of q1 and q2= -9.17654575E-07 -4.49864101E-03

F1 and F2= 0.952131748 2.65744962E-02

Freqs. of fixation of A1 for In and St= 0.987878978 0.997010231

Freqs. of fixation of A2 for In and St= 8.44862964E-03 0.00000000

Freqs. of segregation for In and St= 3.67239211E-03 2.98976898E-03

q1f= 8.47977120E-03 q2f= 0.00000000

Index of gamma value 147 gamma for St popn= 243.339996

Means of q1 and q2= 8.76677223E-03 1.81215873E-04

Variances of q1 and q2= 8.26268177E-03 4.79275695E-06

Covariance and correlation of q1 and q2= -8.78442847E-07 -4.41428414E-03

F1 and F2= 0.950835645 2.64525693E-02

Freqs. of fixation of A1 for In and St= 0.988188386 0.997037828

Freqs. of fixation of A2 for In and St= 8.15604441E-03 0.00000000

Freqs. of segregation for In and St= 3.65556963E-03 2.96217203E-03

q1f= 8.18596873E-03 q2f= 0.00000000

Index of gamma value 148 gamma for St popn= 245.004990

Means of q1 and q2= 8.47879890E-03 1.79354567E-04

Variances of q1 and q2= 7.98240677E-03 4.72191232E-06

Covariance and correlation of q1 and q2= -8.40951486E-07 -4.33156732E-03

F1 and F2= 0.949505627 2.63319723E-02

Freqs. of fixation of A1 for In and St= 0.988487422 0.997065127

Freqs. of fixation of A2 for In and St= 7.87360687E-03 0.00000000

Freqs. of segregation for In and St= 3.63897067E-03 2.93487310E-03

q1f= 7.90236332E-03 q2f= 0.00000000

Index of gamma value 149 gamma for St popn= 246.669998

Means of q1 and q2= 8.20072740E-03 1.77521695E-04

Variances of q1 and q2= 7.71167967E-03 4.65248741E-06

Covariance and correlation of q1 and q2= -8.05111597E-07 -4.25049011E-03

F1 and F2= 0.948140740 2.62126457E-02

Freqs. of fixation of A1 for In and St= 0.988776684 0.997092187

Freqs. of fixation of A2 for In and St= 7.60098640E-03 0.00000000

Freqs. of segregation for In and St= 3.62232979E-03 2.90781260E-03

q1f= 7.62862014E-03 q2f= 0.00000000

Index of gamma value 150 gamma for St popn= 248.334991

Means of q1 and q2= 7.93219265E-03 1.75716006E-04

Variances of q1 and q2= 7.45015312E-03 4.58443265E-06

Covariance and correlation of q1 and q2= -7.70840131E-07 -4.17098776E-03

F1 and F2= 0.946739674 2.60945968E-02

Freqs. of fixation of A1 for In and St= 0.989056289 0.997119009

Freqs. of fixation of A2 for In and St= 7.33781420E-03 0.00000000

Freqs. of segregation for In and St= 3.60589661E-03 2.88099051E-03

q1f= 7.36436946E-03 q2f= 0.00000000

Index of gamma value 151 gamma for St popn= 250.000000

Means of q1 and q2= 7.67286262E-03 1.73937209E-04

Variances of q1 and q2= 7.19752256E-03 4.51771393E-06

Covariance and correlation of q1 and q2= -7.38069048E-07 -4.09304118E-03

F1 and F2= 0.945302367 2.59777643E-02

Freqs. of fixation of A1 for In and St= 0.989326775 0.997145176

Freqs. of fixation of A2 for In and St= 7.08377175E-03 0.00000000

Freqs. of segregation for In and St= 3.58945318E-03 2.85482407E-03

q1f= 7.10928999E-03 q2f= 0.00000000

**h=0.25**

Index of gamma value 31 gamma for St popn= 10.2000008

Means of q1 and q2= 0.445195198 3.10983066E-03

Variances of q1 and q2= 0.246153772 5.46614523E-04

Covariance and correlation of q1 and q2= -1.35811046E-04 -1.17082307E-02

F1 and F2= 0.996588349 0.176318184

Freqs. of fixation of A1 for In and St= 0.551549196 0.973574579

Freqs. of fixation of A2 for In and St= 0.442555845 6.63489045E-05

Freqs. of segregation for In and St= 5.89495897E-03 2.63590720E-02

q1f= 0.445180178 q2f= 6.81451493E-05

Index of gamma value 32 gamma for St popn= 10.5316668

Means of q1 and q2= 0.440191478 2.98484717E-03

Variances of q1 and q2= 0.245580345 4.95452899E-04

Covariance and correlation of q1 and q2= -1.26083731E-04 -1.14303902E-02

F1 and F2= 0.996580720 0.166486308

Freqs. of fixation of A1 for In and St= 0.556533635 0.974094450

Freqs. of fixation of A2 for In and St= 0.437568635 4.83140648E-05

Freqs. of segregation for In and St= 5.89773059E-03 2.58572362E-02

q1f= 0.440164626 q2f= 4.95964923E-05

Index of gamma value 33 gamma for St popn= 10.8633337

Means of q1 and q2= 0.435199469 2.87275645E-03

Variances of q1 and q2= 0.244958356 4.53158689E-04

Covariance and correlation of q1 and q2= -1.18157594E-04 -1.12147694E-02

F1 and F2= 0.996572316 0.158197969

Freqs. of fixation of A1 for In and St= 0.561507702 0.974588871

Freqs. of fixation of A2 for In and St= 0.432592243 3.51943272E-05

Freqs. of segregation for In and St= 5.90005517E-03 2.53759343E-02

q1f= 0.435159683 q2f= 3.61106686E-05

Index of gamma value 34 gamma for St popn= 11.1950006

Means of q1 and q2= 0.430220664 2.77117570E-03

Variances of q1 and q2= 0.244288415 4.17680276E-04

Covariance and correlation of q1 and q2= -1.11583504E-04 -1.10465484E-02

F1 and F2= 0.996563375 0.151141971

Freqs. of fixation of A1 for In and St= 0.566469550 0.975060403

Freqs. of fixation of A2 for In and St= 0.427628398 2.56469884E-05

Freqs. of segregation for In and St= 5.90205193E-03 2.49139499E-02

q1f= 0.430167258 q2f= 2.63022830E-05

Index of gamma value 35 gamma for St popn= 11.5266666

Means of q1 and q2= 0.425255954 2.67829094E-03

Variances of q1 and q2= 0.243571088 3.87498294E-04

Covariance and correlation of q1 and q2= -1.06028980E-04 -1.09138209E-02

F1 and F2= 0.996554017 0.145069718

Freqs. of fixation of A1 for In and St= 0.571418345 0.975511968

Freqs. of fixation of A2 for In and St= 0.422678143 1.86963807E-05

Freqs. of segregation for In and St= 5.90351224E-03 2.44693346E-02

q1f= 0.425188243 q2f= 1.91653435E-05

Index of gamma value 36 gamma for St popn= 11.8583336

Means of q1 and q2= 0.420306057 2.59270542E-03

Variances of q1 and q2= 0.242806897 3.61477752E-04

Covariance and correlation of q1 and q2= -1.01252575E-04 -1.08077368E-02

F1 and F2= 0.996544361 0.139783472

Freqs. of fixation of A1 for In and St= 0.576352835 0.975944757

Freqs. of fixation of A2 for In and St= 0.417742312 1.36342314E-05

Freqs. of segregation for In and St= 5.90485334E-03 2.40416080E-02

q1f= 0.420223683 q2f= 1.39700951E-05

Index of gamma value 37 gamma for St popn= 12.1900005

Means of q1 and q2= 0.415372491 2.51332996E-03

Variances of q1 and q2= 0.241996482 3.38768703E-04

Covariance and correlation of q1 and q2= -9.70738474E-05 -1.07212663E-02

F1 and F2= 0.996533871 0.135128424

Freqs. of fixation of A1 for In and St= 0.581271589 0.976360500

Freqs. of fixation of A2 for In and St= 0.412822574 0.00000000

Freqs. of segregation for In and St= 5.90583682E-03 2.36395001E-02

q1f= 0.415275127 q2f= 0.00000000

Index of gamma value 38 gamma for St popn= 12.5216675

Means of q1 and q2= 0.410455346 2.43931590E-03

Variances of q1 and q2= 0.241140470 3.18728533E-04

Covariance and correlation of q1 and q2= -9.33607225E-05 -1.06492415E-02

F1 and F2= 0.996523321 0.130982593

Freqs. of fixation of A1 for In and St= 0.586174190 0.976760685

Freqs. of fixation of A2 for In and St= 0.407919407 0.00000000

Freqs. of segregation for In and St= 5.90640306E-03 2.32393146E-02

q1f= 0.410343051 q2f= 0.00000000

Index of gamma value 39 gamma for St popn= 12.8533335

Means of q1 and q2= 0.405555636 2.36997963E-03

Variances of q1 and q2= 0.240239397 3.00867890E-04

Covariance and correlation of q1 and q2= -9.00143641E-05 -1.05877034E-02

F1 and F2= 0.996512055 0.127251148

Freqs. of fixation of A1 for In and St= 0.591059864 0.977146685

Freqs. of fixation of A2 for In and St= 0.403033584 0.00000000

Freqs. of segregation for In and St= 5.90655208E-03 2.28533149E-02

q1f= 0.405428290 q2f= 0.00000000

Index of gamma value 40 gamma for St popn= 13.1850004

Means of q1 and q2= 0.400675088 2.30477285E-03

Variances of q1 and q2= 0.239294261 2.84810201E-04

Covariance and correlation of q1 and q2= -8.69611977E-05 -1.05337165E-02

F1 and F2= 0.996500671 0.123859562

Freqs. of fixation of A1 for In and St= 0.595926583 0.977518737

Freqs. of fixation of A2 for In and St= 0.398166955 0.00000000

Freqs. of segregation for In and St= 5.90646267E-03 2.24812627E-02

q1f= 0.400532693 q2f= 0.00000000

Index of gamma value 41 gamma for St popn= 13.5166674

Means of q1 and q2= 0.395813733 2.24324595E-03

Variances of q1 and q2= 0.238305390 2.70263263E-04

Covariance and correlation of q1 and q2= -8.41463916E-05 -1.04851592E-02

F1 and F2= 0.996488154 0.120749533

Freqs. of fixation of A1 for In and St= 0.600774586 0.977878392

Freqs. of fixation of A2 for In and St= 0.393319398 0.00000000

Freqs. of segregation for In and St= 5.90601563E-03 2.21216083E-02

q1f= 0.395656139 q2f= 0.00000000

Index of gamma value 42 gamma for St popn= 13.8483334

Means of q1 and q2= 0.390973061 2.18502642E-03

Variances of q1 and q2= 0.237273946 2.56998435E-04

Covariance and correlation of q1 and q2= -8.15259409E-05 -1.04401214E-02

F1 and F2= 0.996475756 0.117875569

Freqs. of fixation of A1 for In and St= 0.605602026 0.978226244

Freqs. of fixation of A2 for In and St= 0.388492733 0.00000000

Freqs. of segregation for In and St= 5.90524077E-03 2.17737556E-02

q1f= 0.390800506 q2f= 0.00000000

Index of gamma value 43 gamma for St popn= 14.1800003

Means of q1 and q2= 0.386153907 2.12980341E-03

Variances of q1 and q2= 0.236200556 2.44833616E-04

Covariance and correlation of q1 and q2= -7.90707418E-05 -1.03977611E-02

F1 and F2= 0.996462584 0.115201324

Freqs. of fixation of A1 for In and St= 0.610408247 0.978562891

Freqs. of fixation of A2 for In and St= 0.383687556 0.00000000

Freqs. of segregation for In and St= 5.90419769E-03 2.14371085E-02

q1f= 0.385966390 q2f= 0.00000000

Index of gamma value 44 gamma for St popn= 14.5116673

Means of q1 and q2= 0.381356448 2.07730965E-03

Variances of q1 and q2= 0.235085875 2.33623403E-04

Covariance and correlation of q1 and q2= -7.67537276E-05 -1.03568584E-02

F1 and F2= 0.996448755 0.112698510

Freqs. of fixation of A1 for In and St= 0.615193009 0.978889167

Freqs. of fixation of A2 for In and St= 0.378904223 0.00000000

Freqs. of segregation for In and St= 5.90276718E-03 2.11108327E-02

q1f= 0.381154090 q2f= 0.00000000

Index of gamma value 45 gamma for St popn= 14.8433342

Means of q1 and q2= 0.376582921 2.02731998E-03

Variances of q1 and q2= 0.233931258 2.23248950E-04

Covariance and correlation of q1 and q2= -7.45571451E-05 -1.03169288E-02

F1 and F2= 0.996434927 0.110343941

Freqs. of fixation of A1 for In and St= 0.619953990 0.979205251

Freqs. of fixation of A2 for In and St= 0.374144912 0.00000000

Freqs. of segregation for In and St= 5.90109825E-03 2.07947493E-02

q1f= 0.376365870 q2f= 0.00000000

Index of gamma value 46 gamma for St popn= 15.1750002

Means of q1 and q2= 0.371832162 1.97963649E-03

Variances of q1 and q2= 0.232736841 2.13613501E-04

Covariance and correlation of q1 and q2= -7.24662095E-05 -1.02775265E-02

F1 and F2= 0.996420026 0.108119458

Freqs. of fixation of A1 for In and St= 0.624692619 0.979511857

Freqs. of fixation of A2 for In and St= 0.369408339 0.00000000

Freqs. of segregation for In and St= 5.89904189E-03 2.04881430E-02

q1f= 0.371600449 q2f= 0.00000000

Index of gamma value 47 gamma for St popn= 15.5066671

Means of q1 and q2= 0.367106289 1.93408423E-03

Variances of q1 and q2= 0.231503963 2.04635842E-04

Covariance and correlation of q1 and q2= -7.04683480E-05 -1.02382097E-02

F1 and F2= 0.996404886 0.106010072

Freqs. of fixation of A1 for In and St= 0.629406631 0.979809761

Freqs. of fixation of A2 for In and St= 0.364696771 0.00000000

Freqs. of segregation for In and St= 5.89659810E-03 2.01902390E-02

q1f= 0.366859972 q2f= 0.00000000

Index of gamma value 48 gamma for St popn= 15.8383341

Means of q1 and q2= 0.362405956 1.89051055E-03

Variances of q1 and q2= 0.230233535 1.96248206E-04

Covariance and correlation of q1 and q2= -6.85538980E-05 -1.01987049E-02

F1 and F2= 0.996389151 0.104003608

Freqs. of fixation of A1 for In and St= 0.634095192 0.980098844

Freqs. of fixation of A2 for In and St= 0.360010862 0.00000000

Freqs. of segregation for In and St= 5.89394569E-03 1.99011564E-02

q1f= 0.362145334 q2f= 0.00000000

Index of gamma value 49 gamma for St popn= 16.1700001

Means of q1 and q2= 0.357731998 1.84877880E-03

Variances of q1 and q2= 0.228926510 1.88392980E-04

Covariance and correlation of q1 and q2= -6.67148852E-05 -1.01587987E-02

F1 and F2= 0.996373177 0.102090046

Freqs. of fixation of A1 for In and St= 0.638757706 0.980380058

Freqs. of fixation of A2 for In and St= 0.355351359 0.00000000

Freqs. of segregation for In and St= 5.89093566E-03 1.96199417E-02

q1f= 0.357457131 q2f= 0.00000000

Index of gamma value 50 gamma for St popn= 16.5016670

Means of q1 and q2= 0.353084981 1.80876767E-03

Variances of q1 and q2= 0.227583721 1.81020223E-04

Covariance and correlation of q1 and q2= -6.49459544E-05 -1.01185422E-02

F1 and F2= 0.996356368 0.100260660

Freqs. of fixation of A1 for In and St= 0.643393576 0.980653465

Freqs. of fixation of A2 for In and St= 0.350718766 0.00000000

Freqs. of segregation for In and St= 5.88765740E-03 1.93465352E-02

q1f= 0.352795899 q2f= 0.00000000

Index of gamma value 51 gamma for St popn= 16.8333340

Means of q1 and q2= 0.348465204 1.77036517E-03

Variances of q1 and q2= 0.226206079 1.74087210E-04

Covariance and correlation of q1 and q2= -6.32397132E-05 -1.00775352E-02

F1 and F2= 0.996339262 9.85084698E-02

Freqs. of fixation of A1 for In and St= 0.648002326 0.980919659

Freqs. of fixation of A2 for In and St= 0.346113741 0.00000000

Freqs. of segregation for In and St= 5.88393211E-03 1.90803409E-02

q1f= 0.348162293 q2f= 0.00000000

Index of gamma value 52 gamma for St popn= 17.1650009

Means of q1 and q2= 0.343874246 1.73347327E-03

Variances of q1 and q2= 0.224794745 1.67556645E-04

Covariance and correlation of q1 and q2= -6.15938334E-05 -1.00360615E-02

F1 and F2= 0.996321261 9.68273357E-02

Freqs. of fixation of A1 for In and St= 0.652582407 0.981178343

Freqs. of fixation of A2 for In and St= 0.341537297 0.00000000

Freqs. of segregation for In and St= 5.88029623E-03 1.88216567E-02

q1f= 0.343557507 q2f= 0.00000000

Index of gamma value 53 gamma for St popn= 17.4966679

Means of q1 and q2= 0.339311928 1.69800001E-03

Variances of q1 and q2= 0.223350570 1.61395335E-04

Covariance and correlation of q1 and q2= -6.00029598E-05 -9.99386981E-03

F1 and F2= 0.996303141 9.52119306E-02

Freqs. of fixation of A1 for In and St= 0.657134473 0.981430650

Freqs. of fixation of A2 for In and St= 0.336989701 0.00000000

Freqs. of segregation for In and St= 5.87582588E-03 1.85693502E-02

q1f= 0.338981509 q2f= 0.00000000

Index of gamma value 54 gamma for St popn= 17.8283348

Means of q1 and q2= 0.334779263 1.66386086E-03

Variances of q1 and q2= 0.221874595 1.55574133E-04

Covariance and correlation of q1 and q2= -5.84639492E-05 -9.95097589E-03

F1 and F2= 0.996284246 9.36577246E-02

Freqs. of fixation of A1 for In and St= 0.661656737 0.981676459

Freqs. of fixation of A2 for In and St= 0.332471758 0.00000000

Freqs. of segregation for In and St= 5.87150455E-03 1.83235407E-02

q1f= 0.334435403 q2f= 0.00000000

Index of gamma value 55 gamma for St popn= 18.1599998

Means of q1 and q2= 0.330277532 1.63097947E-03

Variances of q1 and q2= 0.220368072 1.50067077E-04

Covariance and correlation of q1 and q2= -5.69737749E-05 -9.90736019E-03

F1 and F2= 0.996264815 9.21607167E-02

Freqs. of fixation of A1 for In and St= 0.666148543 0.981915891

Freqs. of fixation of A2 for In and St= 0.327984840 0.00000000

Freqs. of segregation for In and St= 5.86661696E-03 1.80841088E-02

q1f= 0.329920352 q2f= 0.00000000

Index of gamma value 56 gamma for St popn= 18.4916668

Means of q1 and q2= 0.325805992 1.59928831E-03

Variances of q1 and q2= 0.218831599 1.44850492E-04

Covariance and correlation of q1 and q2= -5.55295555E-05 -9.86300316E-03

F1 and F2= 0.996244848 9.07169282E-02

Freqs. of fixation of A1 for In and St= 0.670610309 0.982149363

Freqs. of fixation of A2 for In and St= 0.323528111 0.00000000

Freqs. of segregation for In and St= 5.86158037E-03 1.78506374E-02

q1f= 0.325435668 q2f= 0.00000000

Index of gamma value 57 gamma for St popn= 18.8233337

Means of q1 and q2= 0.321366370 1.56871846E-03

Variances of q1 and q2= 0.217266589 1.39903699E-04

Covariance and correlation of q1 and q2= -5.41295449E-05 -9.81800631E-03

F1 and F2= 0.996224344 8.93235579E-02

Freqs. of fixation of A1 for In and St= 0.675040483 0.982377529

Freqs. of fixation of A2 for In and St= 0.319103301 0.00000000

Freqs. of segregation for In and St= 5.85621595E-03 1.76224709E-02

q1f= 0.320983052 q2f= 0.00000000

Index of gamma value 58 gamma for St popn= 19.1550007

Means of q1 and q2= 0.316959262 1.53921207E-03

Variances of q1 and q2= 0.215674132 1.35207505E-04

Covariance and correlation of q1 and q2= -5.27707743E-05 -9.77223832E-03

F1 and F2= 0.996203363 8.79774392E-02

Freqs. of fixation of A1 for In and St= 0.679438174 0.982599616

Freqs. of fixation of A2 for In and St= 0.314711154 0.00000000

Freqs. of segregation for In and St= 5.85067272E-03 1.74003839E-02

q1f= 0.316563249 q2f= 0.00000000

Index of gamma value 59 gamma for St popn= 19.4866676

Means of q1 and q2= 0.312584341 1.51071278E-03

Variances of q1 and q2= 0.214054883 1.30744811E-04

Covariance and correlation of q1 and q2= -5.14519052E-05 -9.72582959E-03

F1 and F2= 0.996181548 8.66760612E-02

Freqs. of fixation of A1 for In and St= 0.683804214 0.982816935

Freqs. of fixation of A2 for In and St= 0.310351074 0.00000000

Freqs. of segregation for In and St= 5.84471226E-03 1.71830654E-02

q1f= 0.312175661 q2f= 0.00000000

Index of gamma value 60 gamma for St popn= 19.8183346

Means of q1 and q2= 0.308242768 1.48316962E-03

Variances of q1 and q2= 0.212410256 1.26499799E-04

Covariance and correlation of q1 and q2= -5.01705508E-05 -9.67868231E-03

F1 and F2= 0.996159554 8.54168609E-02

Freqs. of fixation of A1 for In and St= 0.688136876 0.983028948

Freqs. of fixation of A2 for In and St= 0.306024492 0.00000000

Freqs. of segregation for In and St= 5.83863258E-03 1.69710517E-02

q1f= 0.307821751 q2f= 0.00000000

Index of gamma value 61 gamma for St popn= 20.1500015

Means of q1 and q2= 0.303934425 1.45653368E-03

Variances of q1 and q2= 0.210740924 1.22458339E-04

Covariance and correlation of q1 and q2= -4.89255181E-05 -9.63089895E-03

F1 and F2= 0.996136487 8.41978267E-02

Freqs. of fixation of A1 for In and St= 0.692436814 0.983236194

Freqs. of fixation of A2 for In and St= 0.301731139 0.00000000

Freqs. of segregation for In and St= 5.83204627E-03 1.67638063E-02

q1f= 0.303501189 q2f= 0.00000000

Index of gamma value 62 gamma for St popn= 20.4816666

Means of q1 and q2= 0.299660921 1.43075932E-03

Variances of q1 and q2= 0.209048510 1.18606840E-04

Covariance and correlation of q1 and q2= -4.77148569E-05 -9.58242081E-03

F1 and F2= 0.996112943 8.30166042E-02

Freqs. of fixation of A1 for In and St= 0.696701944 0.983438432

Freqs. of fixation of A2 for In and St= 0.297472477 0.00000000

Freqs. of segregation for In and St= 5.82557917E-03 1.65615678E-02

q1f= 0.299215585 q2f= 0.00000000

Index of gamma value 63 gamma for St popn= 20.8133335

Means of q1 and q2= 0.295422047 1.40580616E-03

Variances of q1 and q2= 0.207333833 1.14933981E-04

Covariance and correlation of q1 and q2= -4.65377234E-05 -9.53335594E-03

F1 and F2= 0.996089220 8.18717331E-02

Freqs. of fixation of A1 for In and St= 0.700932741 0.983636439

Freqs. of fixation of A2 for In and St= 0.293248683 0.00000000

Freqs. of segregation for In and St= 5.81857562E-03 1.63635612E-02

q1f= 0.294964969 q2f= 0.00000000

Index of gamma value 64 gamma for St popn= 21.1450005

Means of q1 and q2= 0.291218251 1.38163473E-03

Variances of q1 and q2= 0.205597892 1.11428344E-04

Covariance and correlation of q1 and q2= -4.53927205E-05 -9.48372670E-03

F1 and F2= 0.996064723 8.07612166E-02

Freqs. of fixation of A1 for In and St= 0.705128670 0.983830214

Freqs. of fixation of A2 for In and St= 0.289059848 0.00000000

Freqs. of segregation for In and St= 5.81148267E-03 1.61697865E-02

q1f= 0.290749520 q2f= 0.00000000

Index of gamma value 65 gamma for St popn= 21.4766674

Means of q1 and q2= 0.287049651 1.35820755E-03

Variances of q1 and q2= 0.203841642 1.08079759E-04

Covariance and correlation of q1 and q2= -4.42777819E-05 -9.43337753E-03

F1 and F2= 0.996039629 7.96835124E-02

Freqs. of fixation of A1 for In and St= 0.709289730 0.984019578

Freqs. of fixation of A2 for In and St= 0.284906268 0.00000000

Freqs. of segregation for In and St= 5.80400229E-03 1.59804225E-02

q1f= 0.286569506 q2f= 0.00000000

Index of gamma value 66 gamma for St popn= 21.8083344

Means of q1 and q2= 0.282916784 1.33549131E-03

Variances of q1 and q2= 0.202066153 1.04878833E-04

Covariance and correlation of q1 and q2= -4.31927037E-05 -9.38252453E-03

F1 and F2= 0.996013641 7.86370412E-02

Freqs. of fixation of A1 for In and St= 0.713415325 0.984204829

Freqs. of fixation of A2 for In and St= 0.280788451 0.00000000

Freqs. of segregation for In and St= 5.79622388E-03 1.57951713E-02

q1f= 0.282425433 q2f= 0.00000000

Index of gamma value 67 gamma for St popn= 22.1400013

Means of q1 and q2= 0.278820723 1.31345238E-03

Variances of q1 and q2= 0.200272858 1.01816739E-04

Covariance and correlation of q1 and q2= -4.21360310E-05 -9.33110248E-03

F1 and F2= 0.995987296 7.76203573E-02

Freqs. of fixation of A1 for In and St= 0.717504203 0.984386206

Freqs. of fixation of A2 for In and St= 0.276707351 0.00000000

Freqs. of segregation for In and St= 5.78844547E-03 1.56137943E-02

q1f= 0.278318375 q2f= 0.00000000

Index of gamma value 68 gamma for St popn= 22.4716682

Means of q1 and q2= 0.274760991 1.29206176E-03

Variances of q1 and q2= 0.198462367 9.88856045E-05

Covariance and correlation of q1 and q2= -4.11070068E-05 -9.27919336E-03

F1 and F2= 0.995959997 7.66322017E-02

Freqs. of fixation of A1 for In and St= 0.721557200 0.984564006

Freqs. of fixation of A2 for In and St= 0.272662699 0.00000000

Freqs. of segregation for In and St= 5.78010082E-03 1.54359937E-02

q1f= 0.274247885 q2f= 0.00000000

Index of gamma value 69 gamma for St popn= 22.8033333

Means of q1 and q2= 0.270738721 1.27129001E-03

Variances of q1 and q2= 0.196636155 9.60778561E-05

Covariance and correlation of q1 and q2= -4.01042926E-05 -9.22672451E-03

F1 and F2= 0.995932341 7.56712928E-02

Freqs. of fixation of A1 for In and St= 0.725572824 0.984737813

Freqs. of fixation of A2 for In and St= 0.268655300 0.00000000

Freqs. of segregation for In and St= 5.77187538E-03 1.52621865E-02

q1f= 0.270214945 q2f= 0.00000000

Index of gamma value 70 gamma for St popn= 23.1350002

Means of q1 and q2= 0.266754001 1.25110964E-03

Variances of q1 and q2= 0.194795191 9.33865376E-05

Covariance and correlation of q1 and q2= -3.91272479E-05 -9.17377323E-03

F1 and F2= 0.995904326 7.47364759E-02

Freqs. of fixation of A1 for In and St= 0.729551256 0.984908223

Freqs. of fixation of A2 for In and St= 0.264685541 0.00000000

Freqs. of segregation for In and St= 5.76320291E-03 1.50917768E-02

q1f= 0.266219825 q2f= 0.00000000

Index of gamma value 71 gamma for St popn= 23.4666672

Means of q1 and q2= 0.262806416 1.23149808E-03

Variances of q1 and q2= 0.192940027 9.08052243E-05

Covariance and correlation of q1 and q2= -3.81751743E-05 -9.12041124E-03

F1 and F2= 0.995875001 7.38264918E-02

Freqs. of fixation of A1 for In and St= 0.733492613 0.985074997

Freqs. of fixation of A2 for In and St= 0.260752887 0.00000000

Freqs. of segregation for In and St= 5.75450063E-03 1.49250031E-02

q1f= 0.262262076 q2f= 0.00000000

Index of gamma value 72 gamma for St popn= 23.7983341

Means of q1 and q2= 0.258896977 1.21242914E-03

Variances of q1 and q2= 0.191072181 8.83281391E-05

Covariance and correlation of q1 and q2= -3.72468494E-05 -9.06653330E-03

F1 and F2= 0.995845318 7.29406402E-02

Freqs. of fixation of A1 for In and St= 0.737396300 0.985238671

Freqs. of fixation of A2 for In and St= 0.256858438 0.00000000

Freqs. of segregation for In and St= 5.74526191E-03 1.47613287E-02

q1f= 0.258342683 q2f= 0.00000000

Index of gamma value 73 gamma for St popn= 24.1300011

Means of q1 and q2= 0.255023897 1.19388150E-03

Variances of q1 and q2= 0.189191639 8.59495340E-05

Covariance and correlation of q1 and q2= -3.63419240E-05 -9.01229028E-03

F1 and F2= 0.995815158 7.20777363E-02

Freqs. of fixation of A1 for In and St= 0.741263568 0.985399187

Freqs. of fixation of A2 for In and St= 0.253000319 0.00000000

Freqs. of segregation for In and St= 5.73611259E-03 1.46008134E-02

q1f= 0.254459918 q2f= 0.00000000

Index of gamma value 74 gamma for St popn= 24.4616680

Means of q1 and q2= 0.251192272 1.17583130E-03

Variances of q1 and q2= 0.187301725 8.36641484E-05

Covariance and correlation of q1 and q2= -3.54593212E-05 -8.95756297E-03

F1 and F2= 0.995784044 7.12369531E-02

Freqs. of fixation of A1 for In and St= 0.745089889 0.985556424

Freqs. of fixation of A2 for In and St= 0.249183580 0.00000000

Freqs. of segregation for In and St= 5.72653115E-03 1.44435763E-02

q1f= 0.250618756 q2f= 0.00000000

Index of gamma value 75 gamma for St popn= 24.7933350

Means of q1 and q2= 0.247397542 1.15826225E-03

Variances of q1 and q2= 0.185401171 8.14671948E-05

Covariance and correlation of q1 and q2= -3.45987210E-05 -8.90250970E-03

F1 and F2= 0.995752692 7.04172701E-02

Freqs. of fixation of A1 for In and St= 0.748879313 0.985710621

Freqs. of fixation of A2 for In and St= 0.245403662 0.00000000

Freqs. of segregation for In and St= 5.71702421E-03 1.42893791E-02

q1f= 0.246814713 q2f= 0.00000000

Index of gamma value 76 gamma for St popn= 25.1250000

Means of q1 and q2= 0.243641630 1.14115293E-03

Variances of q1 and q2= 0.183491677 7.93541403E-05

Covariance and correlation of q1 and q2= -3.37592792E-05 -8.84708483E-03

F1 and F2= 0.995720029 6.96180090E-02

Freqs. of fixation of A1 for In and St= 0.752630353 0.985862136

Freqs. of fixation of A2 for In and St= 0.241662577 0.00000000

Freqs. of segregation for In and St= 5.70707023E-03 1.41378641E-02

q1f= 0.243049681 q2f= 0.00000000

Index of gamma value 77 gamma for St popn= 25.4566669

Means of q1 and q2= 0.239924729 1.12448598E-03

Variances of q1 and q2= 0.181574345 7.73206921E-05

Covariance and correlation of q1 and q2= -3.29402246E-05 -8.79126694E-03

F1 and F2= 0.995687068 6.88383281E-02

Freqs. of fixation of A1 for In and St= 0.756342530 0.986010492

Freqs. of fixation of A2 for In and St= 0.237960473 0.00000000

Freqs. of segregation for In and St= 5.69699705E-03 1.39895082E-02

q1f= 0.239323899 q2f= 0.00000000

Index of gamma value 78 gamma for St popn= 25.7883339

Means of q1 and q2= 0.236247942 1.10824395E-03

Variances of q1 and q2= 0.179650545 7.53627028E-05

Covariance and correlation of q1 and q2= -3.21415573E-05 -8.73523112E-03

F1 and F2= 0.995653212 6.80773556E-02

Freqs. of fixation of A1 for In and St= 0.760014892 0.986156285

Freqs. of fixation of A2 for In and St= 0.234298483 0.00000000

Freqs. of segregation for In and St= 5.68662584E-03 1.38437152E-02

q1f= 0.235638469 q2f= 0.00000000

Index of gamma value 79 gamma for St popn= 26.1200008

Means of q1 and q2= 0.232609347 1.09241111E-03

Variances of q1 and q2= 0.177720219 7.34767091E-05

Covariance and correlation of q1 and q2= -3.13619239E-05 -8.67879484E-03

F1 and F2= 0.995618939 6.73345998E-02

Freqs. of fixation of A1 for In and St= 0.763649046 0.986299276

Freqs. of fixation of A2 for In and St= 0.230674580 0.00000000

Freqs. of segregation for In and St= 5.67637384E-03 1.37007236E-02

q1f= 0.231991455 q2f= 0.00000000

Index of gamma value 80 gamma for St popn= 26.4516678

Means of q1 and q2= 0.229009986 1.07697200E-03

Variances of q1 and q2= 0.175784647 7.16590730E-05

Covariance and correlation of q1 and q2= -3.06013389E-05 -8.62212572E-03

F1 and F2= 0.995583713 6.66092783E-02

Freqs. of fixation of A1 for In and St= 0.767244339 0.986439764

Freqs. of fixation of A2 for In and St= 0.227089927 0.00000000

Freqs. of segregation for In and St= 5.66573441E-03 1.35602355E-02

q1f= 0.228383884 q2f= 0.00000000

Index of gamma value 81 gamma for St popn= 26.7833347

Means of q1 and q2= 0.225451037 1.06191123E-03

Variances of q1 and q2= 0.173845410 6.99064549E-05

Covariance and correlation of q1 and q2= -2.98588857E-05 -8.56511761E-03

F1 and F2= 0.995547831 6.59007728E-02

Freqs. of fixation of A1 for In and St= 0.770799458 0.986577690

Freqs. of fixation of A2 for In and St= 0.223545581 0.00000000

Freqs. of segregation for In and St= 5.65496087E-03 1.34223104E-02

q1f= 0.224816903 q2f= 0.00000000

Index of gamma value 82 gamma for St popn= 27.1150017

Means of q1 and q2= 0.221930459 1.04721612E-03

Variances of q1 and q2= 0.171902210 6.82158352E-05

Covariance and correlation of q1 and q2= -2.91345059E-05 -8.50793812E-03

F1 and F2= 0.995511115 6.52084574E-02

Freqs. of fixation of A1 for In and St= 0.774316490 0.986713231

Freqs. of fixation of A2 for In and St= 0.220039621 0.00000000

Freqs. of segregation for In and St= 5.64388931E-03 1.32867694E-02

q1f= 0.221288547 q2f= 0.00000000

Index of gamma value 83 gamma for St popn= 27.4466667

Means of q1 and q2= 0.218449235 1.03287294E-03

Variances of q1 and q2= 0.169956386 6.65843618E-05

Covariance and correlation of q1 and q2= -2.84272828E-05 -8.45046248E-03

F1 and F2= 0.995473683 6.45318553E-02

Freqs. of fixation of A1 for In and St= 0.777794182 0.986846268

Freqs. of fixation of A2 for In and St= 0.216572911 0.00000000

Freqs. of segregation for In and St= 5.63290715E-03 1.31537318E-02

q1f= 0.217799753 q2f= 0.00000000

Index of gamma value 84 gamma for St popn= 27.7783337

Means of q1 and q2= 0.215008497 1.01886899E-03

Variances of q1 and q2= 0.168009430 6.50091242E-05

Covariance and correlation of q1 and q2= -2.77373038E-05 -8.39286577E-03

F1 and F2= 0.995435357 6.38702586E-02

Freqs. of fixation of A1 for In and St= 0.781231880 0.986976922

Freqs. of fixation of A2 for In and St= 0.213146642 0.00000000

Freqs. of segregation for In and St= 5.62147796E-03 1.30230784E-02

q1f= 0.214351624 q2f= 0.00000000

Index of gamma value 85 gamma for St popn= 28.1100006

Means of q1 and q2= 0.211608335 1.00519171E-03

Variances of q1 and q2= 0.166062266 6.34876487E-05

Covariance and correlation of q1 and q2= -2.70637101E-05 -8.33503157E-03

F1 and F2= 0.995396674 6.32232875E-02

Freqs. of fixation of A1 for In and St= 0.784628987 0.987105429

Freqs. of fixation of A2 for In and St= 0.209760860 0.00000000

Freqs. of segregation for In and St= 5.61015308E-03 1.28945708E-02

q1f= 0.210944295 q2f= 0.00000000

Index of gamma value 86 gamma for St popn= 28.4416676

Means of q1 and q2= 0.208246380 9.91831068E-04

Variances of q1 and q2= 0.164114282 6.20175051E-05

Covariance and correlation of q1 and q2= -2.64060800E-05 -8.27701297E-03

F1 and F2= 0.995356917 6.25903755E-02

Freqs. of fixation of A1 for In and St= 0.787988245 0.987232029

Freqs. of fixation of A2 for In and St= 0.206413314 0.00000000

Freqs. of segregation for In and St= 5.59844077E-03 1.27679706E-02

q1f= 0.207575411 q2f= 0.00000000

Index of gamma value 87 gamma for St popn= 28.7733345

Means of q1 and q2= 0.204923719 9.78775322E-04

Variances of q1 and q2= 0.162166893 6.05963978E-05

Covariance and correlation of q1 and q2= -2.57639913E-05 -8.21880437E-03

F1 and F2= 0.995316446 6.19710796E-02

Freqs. of fixation of A1 for In and St= 0.791308224 0.987356007

Freqs. of fixation of A2 for In and St= 0.203105018 0.00000000

Freqs. of segregation for In and St= 5.58675826E-03 1.26439929E-02

q1f= 0.204246089 q2f= 0.00000000

Index of gamma value 88 gamma for St popn= 29.1050014

Means of q1 and q2= 0.201642752 9.66013293E-04

Variances of q1 and q2= 0.160222322 5.92220676E-05

Covariance and correlation of q1 and q2= -2.51375168E-05 -8.16054363E-03

F1 and F2= 0.995275080 6.13649227E-02

Freqs. of fixation of A1 for In and St= 0.794586897 0.987478256

Freqs. of fixation of A2 for In and St= 0.199838281 0.00000000

Freqs. of segregation for In and St= 5.57482243E-03 1.25217438E-02

q1f= 0.200958595 q2f= 0.00000000

Index of gamma value 89 gamma for St popn= 29.4366684

Means of q1 and q2= 0.198398530 9.53536481E-04

Variances of q1 and q2= 0.158278435 5.78926192E-05

Covariance and correlation of q1 and q2= -2.45253032E-05 -8.10199417E-03

F1 and F2= 0.995233119 6.07715361E-02

Freqs. of fixation of A1 for In and St= 0.797828853 0.987598062

Freqs. of fixation of A2 for In and St= 0.196608245 0.00000000

Freqs. of segregation for In and St= 5.56290150E-03 1.24019384E-02

q1f= 0.197708070 q2f= 0.00000000

Index of gamma value 90 gamma for St popn= 29.7683334

Means of q1 and q2= 0.195197105 9.41335049E-04

Variances of q1 and q2= 0.156339556 5.66059462E-05

Covariance and correlation of q1 and q2= -2.39283982E-05 -8.04355741E-03

F1 and F2= 0.995189965 6.01903424E-02

Freqs. of fixation of A1 for In and St= 0.801028430 0.987716377

Freqs. of fixation of A2 for In and St= 0.193420932 0.00000000

Freqs. of segregation for In and St= 5.55063784E-03 1.22836232E-02

q1f= 0.194500536 q2f= 0.00000000

Index of gamma value 91 gamma for St popn= 30.1000004

Means of q1 and q2= 0.192032188 9.29399743E-04

Variances of q1 and q2= 0.154402733 5.53603713E-05

Covariance and correlation of q1 and q2= -2.33450410E-05 -7.98486639E-03

F1 and F2= 0.995146215 5.96211404E-02

Freqs. of fixation of A1 for In and St= 0.804191530 0.987832367

Freqs. of fixation of A2 for In and St= 0.190270096 0.00000000

Freqs. of segregation for In and St= 5.53837419E-03 1.21676326E-02

q1f= 0.191329747 q2f= 0.00000000

Index of gamma value 92 gamma for St popn= 30.4316673

Means of q1 and q2= 0.188907459 9.17722587E-04

Variances of q1 and q2= 0.152470902 5.41541012E-05

Covariance and correlation of q1 and q2= -2.27756827E-05 -7.92614929E-03

F1 and F2= 0.995101690 5.90634309E-02

Freqs. of fixation of A1 for In and St= 0.807314694 0.987946630

Freqs. of fixation of A2 for In and St= 0.187159315 0.00000000

Freqs. of segregation for In and St= 5.52599132E-03 1.20533705E-02

q1f= 0.188199311 q2f= 0.00000000

Index of gamma value 93 gamma for St popn= 30.7633343

Means of q1 and q2= 0.185821414 9.06294212E-04

Variances of q1 and q2= 0.150543854 5.29854697E-05

Covariance and correlation of q1 and q2= -2.22197559E-05 -7.86736608E-03

F1 and F2= 0.995056152 5.85169047E-02

Freqs. of fixation of A1 for In and St= 0.810399473 0.988059044

Freqs. of fixation of A2 for In and St= 0.184087247 0.00000000

Freqs. of segregation for In and St= 5.51328063E-03 1.19409561E-02

q1f= 0.185107797 q2f= 0.00000000

Index of gamma value 94 gamma for St popn= 31.0950012

Means of q1 and q2= 0.182776511 8.95106583E-04

Variances of q1 and q2= 0.148623869 5.18529087E-05

Covariance and correlation of q1 and q2= -2.16770859E-05 -7.80854374E-03

F1 and F2= 0.995009780 5.79812080E-02

Freqs. of fixation of A1 for In and St= 0.813443244 0.988169789

Freqs. of fixation of A2 for In and St= 0.181056216 0.00000000

Freqs. of segregation for In and St= 5.50054014E-03 1.18302107E-02

q1f= 0.182057634 q2f= 0.00000000

Index of gamma value 95 gamma for St popn= 31.4266682

Means of q1 and q2= 0.179770187 8.84153822E-04

Variances of q1 and q2= 0.146710083 5.07550103E-05

Covariance and correlation of q1 and q2= -2.11473234E-05 -7.74971908E-03

F1 and F2= 0.994962513 5.74559793E-02

Freqs. of fixation of A1 for In and St= 0.816448510 0.988278806

Freqs. of fixation of A2 for In and St= 0.178063586 0.00000000

Freqs. of segregation for In and St= 5.48790395E-03 1.17211938E-02

q1f= 0.179046184 q2f= 0.00000000

Index of gamma value 96 gamma for St popn= 31.7583351

Means of q1 and q2= 0.176802129 8.73427023E-04

Variances of q1 and q2= 0.144802988 4.96903303E-05

Covariance and correlation of q1 and q2= -2.06299592E-05 -7.69083807E-03

F1 and F2= 0.994914651 5.69409542E-02

Freqs. of fixation of A1 for In and St= 0.819415689 0.988385737

Freqs. of fixation of A2 for In and St= 0.175109252 0.00000000

Freqs. of segregation for In and St= 5.47505915E-03 1.16142631E-02

q1f= 0.176073268 q2f= 0.00000000

Index of gamma value 97 gamma for St popn= 32.0900002

Means of q1 and q2= 0.173872218 8.62920133E-04

Variances of q1 and q2= 0.142903149 4.86575445E-05

Covariance and correlation of q1 and q2= -2.01248331E-05 -7.63196358E-03

F1 and F2= 0.994865537 5.64357750E-02

Freqs. of fixation of A1 for In and St= 0.822345138 0.988491654

Freqs. of fixation of A2 for In and St= 0.172192946 0.00000000

Freqs. of segregation for In and St= 5.46191633E-03 1.15083456E-02

q1f= 0.173138618 q2f= 0.00000000

Index of gamma value 98 gamma for St popn= 32.4216690

Means of q1 and q2= 0.170981407 8.52625526E-04

Variances of q1 and q2= 0.141011894 4.76553287E-05

Covariance and correlation of q1 and q2= -1.96315814E-05 -7.57306535E-03

F1 and F2= 0.994815648 5.59401475E-02

Freqs. of fixation of A1 for In and St= 0.825235367 0.988595486

Freqs. of fixation of A2 for In and St= 0.169315726 0.00000000

Freqs. of segregation for In and St= 5.44890761E-03 1.14045143E-02

q1f= 0.170243368 q2f= 0.00000000

Index of gamma value 99 gamma for St popn= 32.7533340

Means of q1 and q2= 0.168129787 8.42537615E-04

Variances of q1 and q2= 0.139129952 4.66826023E-05

Covariance and correlation of q1 and q2= -1.91502040E-05 -7.51424860E-03

F1 and F2= 0.994764745 5.54538630E-02

Freqs. of fixation of A1 for In and St= 0.828086734 0.988697708

Freqs. of fixation of A2 for In and St= 0.166477561 0.00000000

Freqs. of segregation for In and St= 5.43570518E-03 1.13022923E-02

q1f= 0.167387426 q2f= 0.00000000

Index of gamma value 100 gamma for St popn= 33.0850029

Means of q1 and q2= 0.165315673 8.32650403E-04

Variances of q1 and q2= 0.137256876 4.57381284E-05

Covariance and correlation of q1 and q2= -1.86801917E-05 -7.45547097E-03

F1 and F2= 0.994713128 5.49765490E-02

Freqs. of fixation of A1 for In and St= 0.830900788 0.988798618

Freqs. of fixation of A2 for In and St= 0.163676769 0.00000000

Freqs. of segregation for In and St= 5.42244315E-03 1.12013817E-02

q1f= 0.164569139 q2f= 0.00000000

Index of gamma value 101 gamma for St popn= 33.4166679

Means of q1 and q2= 0.162540153 8.22957139E-04

Variances of q1 and q2= 0.135394037 4.48208339E-05

Covariance and correlation of q1 and q2= -1.82212680E-05 -7.39671476E-03

F1 and F2= 0.994660497 5.45080006E-02

Freqs. of fixation of A1 for In and St= 0.833676457 0.988897920

Freqs. of fixation of A2 for In and St= 0.160914630 0.00000000

Freqs. of segregation for In and St= 5.40891290E-03 1.11020803E-02

q1f= 0.161789730 q2f= 0.00000000

Index of gamma value 102 gamma for St popn= 33.7483330

Means of q1 and q2= 0.159801856 8.13453516E-04

Variances of q1 and q2= 0.133541107 4.39297510E-05

Covariance and correlation of q1 and q2= -1.77733382E-05 -7.33807450E-03

F1 and F2= 0.994606793 5.40479757E-02

Freqs. of fixation of A1 for In and St= 0.836414993 0.988995790

Freqs. of fixation of A2 for In and St= 0.158189535 0.00000000

Freqs. of segregation for In and St= 5.39547205E-03 1.10042095E-02

q1f= 0.159047678 q2f= 0.00000000

Index of gamma value 103 gamma for St popn= 34.0800018

Means of q1 and q2= 0.157101646 8.04132782E-04

Variances of q1 and q2= 0.131699294 4.30638211E-05

Covariance and correlation of q1 and q2= -1.73359440E-05 -7.27946544E-03

F1 and F2= 0.994552016 5.35962209E-02

Freqs. of fixation of A1 for In and St= 0.839115679 0.989092290

Freqs. of fixation of A2 for In and St= 0.155502483 0.00000000

Freqs. of segregation for In and St= 5.38183749E-03 1.09077096E-02

q1f= 0.156343892 q2f= 0.00000000

Index of gamma value 104 gamma for St popn= 34.4116669

Means of q1 and q2= 0.154438108 7.94989930E-04

Variances of q1 and q2= 0.129868269 4.22221237E-05

Covariance and correlation of q1 and q2= -1.69088817E-05 -7.22093321E-03

F1 and F2= 0.994496286 5.31525202E-02

Freqs. of fixation of A1 for In and St= 0.841779768 0.989187419

Freqs. of fixation of A2 for In and St= 0.152851954 0.00000000

Freqs. of segregation for In and St= 5.36827743E-03 1.08125806E-02

q1f= 0.153676942 q2f= 0.00000000

Index of gamma value 105 gamma for St popn= 34.7433357

Means of q1 and q2= 0.151812300 7.86020013E-04

Variances of q1 and q2= 0.128049344 4.14037786E-05

Covariance and correlation of q1 and q2= -1.64920493E-05 -7.16252159E-03

F1 and F2= 0.994439602 5.27166575E-02

Freqs. of fixation of A1 for In and St= 0.844406426 0.989281058

Freqs. of fixation of A2 for In and St= 0.150239095 0.00000000

Freqs. of segregation for In and St= 5.35447896E-03 1.07189417E-02

q1f= 0.151047871 q2f= 0.00000000

Index of gamma value 106 gamma for St popn= 35.0750008

Means of q1 and q2= 0.149223894 7.77218782E-04

Variances of q1 and q2= 0.126242846 4.06079016E-05

Covariance and correlation of q1 and q2= -1.60852214E-05 -7.10425247E-03

F1 and F2= 0.994381726 5.22883497E-02

Freqs. of fixation of A1 for In and St= 0.846995771 0.989373267

Freqs. of fixation of A2 for In and St= 0.147663578 0.00000000

Freqs. of segregation for In and St= 5.34065068E-03 1.06267333E-02

q1f= 0.148456424 q2f= 0.00000000

Index of gamma value 107 gamma for St popn= 35.4066696

Means of q1 and q2= 0.146671310 7.68580998E-04

Variances of q1 and q2= 0.124448352 3.98337397E-05

Covariance and correlation of q1 and q2= -1.56879178E-05 -7.04604108E-03

F1 and F2= 0.994323254 5.18675074E-02

Freqs. of fixation of A1 for In and St= 0.849549592 0.989464402

Freqs. of fixation of A2 for In and St= 0.145123765 0.00000000

Freqs. of segregation for In and St= 5.32664359E-03 1.05355978E-02

q1f= 0.145900920 q2f= 0.00000000

Index of gamma value 108 gamma for St popn= 35.7383347

Means of q1 and q2= 0.144152969 7.60102877E-04

Variances of q1 and q2= 0.122665115 3.90804707E-05

Covariance and correlation of q1 and q2= -1.52999492E-05 -6.98794611E-03

F1 and F2= 0.994263172 5.14538176E-02

Freqs. of fixation of A1 for In and St= 0.852068961 0.989554107

Freqs. of fixation of A2 for In and St= 0.142618150 0.00000000

Freqs. of segregation for In and St= 5.31288981E-03 1.04458928E-02

q1f= 0.143379912 q2f= 0.00000000

Index of gamma value 109 gamma for St popn= 36.0699997

Means of q1 and q2= 0.141675338 7.51778425E-04

Variances of q1 and q2= 0.120898433 3.83473089E-05

Covariance and correlation of q1 and q2= -1.49216357E-05 -6.93008676E-03

F1 and F2= 0.994202435 5.10471687E-02

Freqs. of fixation of A1 for In and St= 0.854547977 0.989642143

Freqs. of fixation of A2 for In and St= 0.140153110 0.00000000

Freqs. of segregation for In and St= 5.29891253E-03 1.03578568E-02

q1f= 0.140899733 q2f= 0.00000000

Index of gamma value 110 gamma for St popn= 36.4016685

Means of q1 and q2= 0.139231294 7.43605255E-04

Variances of q1 and q2= 0.119143695 3.76336247E-05

Covariance and correlation of q1 and q2= -1.45521844E-05 -6.87234011E-03

F1 and F2= 0.994140506 5.06473444E-02

Freqs. of fixation of A1 for In and St= 0.856993675 0.989729702

Freqs. of fixation of A2 for In and St= 0.137721583 0.00000000

Freqs. of segregation for In and St= 5.28474152E-03 1.02702975E-02

q1f= 0.138453275 q2f= 0.00000000

Index of gamma value 111 gamma for St popn= 36.7333336

Means of q1 and q2= 0.136822000 7.35578593E-04

Variances of q1 and q2= 0.117402278 3.69387199E-05

Covariance and correlation of q1 and q2= -1.41914643E-05 -6.81472057E-03

F1 and F2= 0.994077384 5.02541997E-02

Freqs. of fixation of A1 for In and St= 0.859404743 0.989815593

Freqs. of fixation of A2 for In and St= 0.135324731 0.00000000

Freqs. of segregation for In and St= 5.27052581E-03 1.01844072E-02

q1f= 0.136041746 q2f= 0.00000000

Index of gamma value 112 gamma for St popn= 37.0650024

Means of q1 and q2= 0.134449735 7.27694540E-04

Variances of q1 and q2= 0.115676314 3.62618848E-05

Covariance and correlation of q1 and q2= -1.38395044E-05 -6.75729942E-03

F1 and F2= 0.994013309 4.98674810E-02

Freqs. of fixation of A1 for In and St= 0.861778796 0.989900470

Freqs. of fixation of A2 for In and St= 0.132964790 0.00000000

Freqs. of segregation for In and St= 5.25641441E-03 1.00995302E-02

q1f= 0.133667395 q2f= 0.00000000

Index of gamma value 113 gamma for St popn= 37.3966675

Means of q1 and q2= 0.132111639 7.19949428E-04

Variances of q1 and q2= 0.113964252 3.56025812E-05

Covariance and correlation of q1 and q2= -1.34959337E-05 -6.70004729E-03

F1 and F2= 0.993948162 4.94871326E-02

Freqs. of fixation of A1 for In and St= 0.864118695 0.989984035

Freqs. of fixation of A2 for In and St= 0.130638927 0.00000000

Freqs. of segregation for In and St= 5.24237752E-03 1.00159645E-02

q1f= 0.131327391 q2f= 0.00000000

Index of gamma value 114 gamma for St popn= 37.7283363

Means of q1 and q2= 0.129808694 7.12339533E-04

Variances of q1 and q2= 0.112267271 3.49601687E-05

Covariance and correlation of q1 and q2= -1.31606503E-05 -6.64299587E-03

F1 and F2= 0.993881524 4.91129383E-02

Freqs. of fixation of A1 for In and St= 0.866423965 0.990066648

Freqs. of fixation of A2 for In and St= 0.128348112 0.00000000

Freqs. of segregation for In and St= 5.22792339E-03 9.93335247E-03

q1f= 0.129022628 q2f= 0.00000000

Index of gamma value 115 gamma for St popn= 38.0600014

Means of q1 and q2= 0.127539173 7.04862410E-04

Variances of q1 and q2= 0.110584587 3.43340762E-05

Covariance and correlation of q1 and q2= -1.28334068E-05 -6.58615585E-03

F1 and F2= 0.993813932 4.87446822E-02

Freqs. of fixation of A1 for In and St= 0.868695796 0.990147948

Freqs. of fixation of A2 for In and St= 0.126090661 0.00000000

Freqs. of segregation for In and St= 5.21354377E-03 9.85205173E-03

q1f= 0.126751482 q2f= 0.00000000

Index of gamma value 116 gamma for St popn= 38.3916664

Means of q1 and q2= 0.125304192 6.97513111E-04

Variances of q1 and q2= 0.108917505 3.37237871E-05

Covariance and correlation of q1 and q2= -1.25139923E-05 -6.52948394E-03

F1 and F2= 0.993745208 4.83823530E-02

Freqs. of fixation of A1 for In and St= 0.870933115 0.990228534

Freqs. of fixation of A2 for In and St= 0.123867676 0.00000000

Freqs. of segregation for In and St= 5.19920886E-03 9.77146626E-03

q1f= 0.124515057 q2f= 0.00000000

Index of gamma value 117 gamma for St popn= 38.7233353

Means of q1 and q2= 0.123103388 6.90288958E-04

Variances of q1 and q2= 0.107266188 3.31287301E-05

Covariance and correlation of q1 and q2= -1.22022975E-05 -6.47303276E-03

F1 and F2= 0.993675172 4.80257049E-02

Freqs. of fixation of A1 for In and St= 0.873136401 0.990307689

Freqs. of fixation of A2 for In and St= 0.121678725 0.00000000

Freqs. of segregation for In and St= 5.18487394E-03 9.69231129E-03

q1f= 0.122312903 q2f= 0.00000000

Index of gamma value 118 gamma for St popn= 39.0550003

Means of q1 and q2= 0.120936461 6.83187216E-04

Variances of q1 and q2= 0.105630860 3.25484434E-05

Covariance and correlation of q1 and q2= -1.18982207E-05 -6.41684420E-03

F1 and F2= 0.993603885 4.76746298E-02

Freqs. of fixation of A1 for In and St= 0.875306010 0.990385711

Freqs. of fixation of A2 for In and St= 0.119523533 0.00000000

Freqs. of segregation for In and St= 5.17045707E-03 9.61428881E-03

q1f= 0.120144740 q2f= 0.00000000

Index of gamma value 119 gamma for St popn= 39.3866692

Means of q1 and q2= 0.118801691 6.76205498E-04

Variances of q1 and q2= 0.104010686 3.19824794E-05

Covariance and correlation of q1 and q2= -1.16013834E-05 -6.36084378E-03

F1 and F2= 0.993531644 4.73289900E-02

Freqs. of fixation of A1 for In and St= 0.877443552 0.990463197

Freqs. of fixation of A2 for In and St= 0.117400467 0.00000000

Freqs. of segregation for In and St= 5.15598059E-03 9.53680277E-03

q1f= 0.118008919 q2f= 0.00000000

Index of gamma value 120 gamma for St popn= 39.7183342

Means of q1 and q2= 0.116700128 6.69339031E-04

Variances of q1 and q2= 0.102406800 3.14302888E-05

Covariance and correlation of q1 and q2= -1.13118076E-05 -6.30512089E-03

F1 and F2= 0.993457496 4.69886549E-02

Freqs. of fixation of A1 for In and St= 0.879547894 0.990539312

Freqs. of fixation of A2 for In and St= 0.115310483 0.00000000

Freqs. of segregation for In and St= 5.14162332E-03 9.46068764E-03

q1f= 0.115906432 q2f= 0.00000000

Index of gamma value 121 gamma for St popn= 40.0500031

Means of q1 and q2= 0.114631400 6.62586128E-04

Variances of q1 and q2= 0.100819431 3.08914823E-05

Covariance and correlation of q1 and q2= -1.10291876E-05 -6.24959730E-03

F1 and F2= 0.993382573 4.66535017E-02

Freqs. of fixation of A1 for In and St= 0.881619692 0.990614355

Freqs. of fixation of A2 for In and St= 0.113253221 0.00000000

Freqs. of segregation for In and St= 5.12708724E-03 9.38564539E-03

q1f= 0.113836870 q2f= 0.00000000

Index of gamma value 122 gamma for St popn= 40.3816681

Means of q1 and q2= 0.112593785 6.55943935E-04

Variances of q1 and q2= 9.92476195E-02 3.03656470E-05

Covariance and correlation of q1 and q2= -1.07533633E-05 -6.19431119E-03

F1 and F2= 0.993306339 4.63234372E-02

Freqs. of fixation of A1 for In and St= 0.883660495 0.990688622

Freqs. of fixation of A2 for In and St= 0.111227036 0.00000000

Freqs. of segregation for In and St= 5.11246920E-03 9.31137800E-03

q1f= 0.111798599 q2f= 0.00000000

Index of gamma value 123 gamma for St popn= 40.7133331

Means of q1 and q2= 0.110588364 6.49409951E-04

Variances of q1 and q2= 9.76925716E-02 2.98523446E-05

Covariance and correlation of q1 and q2= -1.04842766E-05 -6.13929471E-03

F1 and F2= 0.993228793 4.59982827E-02

Freqs. of fixation of A1 for In and St= 0.885669053 0.990761936

Freqs. of fixation of A2 for In and St= 0.109232910 0.00000000

Freqs. of segregation for In and St= 5.09803742E-03 9.23806429E-03

q1f= 0.109792635 q2f= 0.00000000

Index of gamma value 124 gamma for St popn= 41.0450020

Means of q1 and q2= 0.108616196 6.42980740E-04

Variances of q1 and q2= 9.61554945E-02 2.93511712E-05

Covariance and correlation of q1 and q2= -1.02218655E-05 -6.08457718E-03

F1 and F2= 0.993149817 4.56779674E-02

Freqs. of fixation of A1 for In and St= 0.887644708 0.990834355

Freqs. of fixation of A2 for In and St= 0.107271932 0.00000000

Freqs. of segregation for In and St= 5.08335978E-03 9.16564465E-03

q1f= 0.107820019 q2f= 0.00000000

Index of gamma value 125 gamma for St popn= 41.3766670

Means of q1 and q2= 0.106672712 6.36655197E-04

Variances of q1 and q2= 9.46332142E-02 2.88618212E-05

Covariance and correlation of q1 and q2= -9.96561721E-06 -6.03004498E-03

F1 and F2= 0.993069470 4.53623980E-02

Freqs. of fixation of A1 for In and St= 0.889591634 0.990905881

Freqs. of fixation of A2 for In and St= 0.105339557 0.00000000

Freqs. of segregation for In and St= 5.06880879E-03 9.09411907E-03

q1f= 0.105876222 q2f= 0.00000000

Index of gamma value 126 gamma for St popn= 41.7083359

Means of q1 and q2= 0.104761742 6.30430062E-04

Variances of q1 and q2= 9.31290761E-02 2.83838490E-05

Covariance and correlation of q1 and q2= -9.71582267E-06 -5.97587181E-03

F1 and F2= 0.992987812 4.50513959E-02

Freqs. of fixation of A1 for In and St= 0.891506374 0.990976393

Freqs. of fixation of A2 for In and St= 0.103439584 0.00000000

Freqs. of segregation for In and St= 5.05404174E-03 9.02360678E-03

q1f= 0.103965029 q2f= 0.00000000

Index of gamma value 127 gamma for St popn= 42.0400009

Means of q1 and q2= 0.102878816 6.24303822E-04

Variances of q1 and q2= 9.16399136E-02 2.79169799E-05

Covariance and correlation of q1 and q2= -9.47191074E-06 -5.92189981E-03

F1 and F2= 0.992904782 4.47449125E-02

Freqs. of fixation of A1 for In and St= 0.893392622 0.991046131

Freqs. of fixation of A2 for In and St= 0.101567596 0.00000000

Freqs. of segregation for In and St= 5.03978133E-03 8.95386934E-03

q1f= 0.102082074 q2f= 0.00000000

Index of gamma value 128 gamma for St popn= 42.3716698

Means of q1 and q2= 0.101029046 6.18272636E-04

Variances of q1 and q2= 9.01701078E-02 2.74608174E-05

Covariance and correlation of q1 and q2= -9.23416883E-06 -5.86826587E-03

F1 and F2= 0.992820382 4.44428623E-02

Freqs. of fixation of A1 for In and St= 0.895246327 0.991114974

Freqs. of fixation of A2 for In and St= 9.97286439E-02 0.00000000

Freqs. of segregation for In and St= 5.02502918E-03 8.88502598E-03

q1f= 0.100232311 q2f= 0.00000000

Index of gamma value 129 gamma for St popn= 42.7033348

Means of q1 and q2= 9.92080718E-02 6.12335454E-04

Variances of q1 and q2= 8.87165368E-02 2.70150649E-05

Covariance and correlation of q1 and q2= -9.00219675E-06 -5.81491273E-03

F1 and F2= 0.992734432 4.41451110E-02

Freqs. of fixation of A1 for In and St= 0.897071302 0.991182864

Freqs. of fixation of A2 for In and St= 9.79183689E-02 0.00000000

Freqs. of segregation for In and St= 5.01032919E-03 8.81713629E-03

q1f= 9.84114408E-02 q2f= 0.00000000

Index of gamma value 130 gamma for St popn= 43.0349998

Means of q1 and q2= 9.74167362E-02 6.06489892E-04

Variances of q1 and q2= 8.72801989E-02 2.65794079E-05

Covariance and correlation of q1 and q2= -8.77591810E-06 -5.76185109E-03

F1 and F2= 0.992647111 4.38515767E-02

Freqs. of fixation of A1 for In and St= 0.898866475 0.991250038

Freqs. of fixation of A2 for In and St= 9.61376578E-02 0.00000000

Freqs. of segregation for In and St= 4.99586761E-03 8.74996185E-03

q1f= 9.66203585E-02 q2f= 0.00000000

Index of gamma value 131 gamma for St popn= 43.3666687

Means of q1 and q2= 9.56533849E-02 6.00734435E-04

Variances of q1 and q2= 8.58600736E-02 2.61535552E-05

Covariance and correlation of q1 and q2= -8.55509643E-06 -5.70905069E-03

F1 and F2= 0.992558300 4.35621366E-02

Freqs. of fixation of A1 for In and St= 0.900633931 0.991316319

Freqs. of fixation of A2 for In and St= 9.43848416E-02 0.00000000

Freqs. of segregation for In and St= 4.98122722E-03 8.68368149E-03

q1f= 9.48573500E-02 q2f= 0.00000000

Index of gamma value 132 gamma for St popn= 43.6983337

Means of q1 and q2= 9.39204469E-02 5.95066173E-04

Variances of q1 and q2= 8.44584256E-02 2.57371976E-05

Covariance and correlation of q1 and q2= -8.33987360E-06 -5.65662421E-03

F1 and F2= 0.992468059 4.32767384E-02

Freqs. of fixation of A1 for In and St= 0.902371168 0.991381764

Freqs. of fixation of A2 for In and St= 9.26623493E-02 0.00000000

Freqs. of segregation for In and St= 4.96648252E-03 8.61823559E-03

q1f= 9.31248516E-02 q2f= 0.00000000

Index of gamma value 133 gamma for St popn= 44.0300026

Means of q1 and q2= 9.22134593E-02 5.89483709E-04

Variances of q1 and q2= 8.30719173E-02 2.53301296E-05

Covariance and correlation of q1 and q2= -8.12969301E-06 -5.60438959E-03

F1 and F2= 0.992375791 4.29953709E-02

Freqs. of fixation of A1 for In and St= 0.904082298 0.991446614

Freqs. of fixation of A2 for In and St= 9.09656957E-02 0.00000000

Freqs. of segregation for In and St= 4.95200604E-03 8.55338573E-03

q1f= 9.14184004E-02 q2f= 0.00000000

Index of gamma value 134 gamma for St popn= 44.3616676

Means of q1 and q2= 9.05361101E-02 5.83985588E-04

Variances of q1 and q2= 8.17038715E-02 2.49320092E-05

Covariance and correlation of q1 and q2= -7.92486026E-06 -5.55253960E-03

F1 and F2= 0.992282510 4.27177958E-02

Freqs. of fixation of A1 for In and St= 0.905764103 0.991510391

Freqs. of fixation of A2 for In and St= 8.92986059E-02 0.00000000

Freqs. of segregation for In and St= 4.93729115E-03 8.48960876E-03

q1f= 8.97416845E-02 q2f= 0.00000000

Index of gamma value 135 gamma for St popn= 44.6933365

Means of q1 and q2= 8.88866857E-02 5.78568841E-04

Variances of q1 and q2= 8.03531408E-02 2.45425599E-05

Covariance and correlation of q1 and q2= -7.72507701E-06 -5.50099742E-03

F1 and F2= 0.992187500 4.24439870E-02

Freqs. of fixation of A1 for In and St= 0.907418013 0.991573930

Freqs. of fixation of A2 for In and St= 8.76593366E-02 0.00000000

Freqs. of segregation for In and St= 4.92265075E-03 8.42607021E-03

q1f= 8.80929828E-02 q2f= 0.00000000

Index of gamma value 136 gamma for St popn= 45.0250015

Means of q1 and q2= 8.72635320E-02 5.73233236E-04

Variances of q1 and q2= 7.90186450E-02 2.41616308E-05

Covariance and correlation of q1 and q2= -7.53017230E-06 -5.44975558E-03

F1 and F2= 0.992090762 4.21739109E-02

Freqs. of fixation of A1 for In and St= 0.909045637 0.991636515

Freqs. of fixation of A2 for In and St= 8.60462263E-02 0.00000000

Freqs. of segregation for In and St= 4.90813702E-03 8.36348534E-03

q1f= 8.64706337E-02 q2f= 0.00000000

Index of gamma value 137 gamma for St popn= 45.3566666

Means of q1 and q2= 8.56675953E-02 5.67975512E-04

Variances of q1 and q2= 7.77014494E-02 2.37889126E-05

Covariance and correlation of q1 and q2= -7.34008063E-06 -5.39882155E-03

F1 and F2= 0.991992652 4.19074968E-02

Freqs. of fixation of A1 for In and St= 0.910646141 0.991698205

Freqs. of fixation of A2 for In and St= 8.44602734E-02 0.00000000

Freqs. of segregation for In and St= 4.89358604E-03 8.30179453E-03

q1f= 8.48756209E-02 q2f= 0.00000000

Index of gamma value 138 gamma for St popn= 45.6883354

Means of q1 and q2= 8.40998814E-02 5.62794332E-04

Variances of q1 and q2= 7.64025971E-02 2.34241434E-05

Covariance and correlation of q1 and q2= -7.15481656E-06 -5.34826098E-03

F1 and F2= 0.991892576 4.16445769E-02

Freqs. of fixation of A1 for In and St= 0.912218511 0.991759062

Freqs. of fixation of A2 for In and St= 8.29024017E-02 0.00000000

Freqs. of segregation for In and St= 4.87908721E-03 8.24093819E-03

q1f= 8.33088756E-02 q2f= 0.00000000

Index of gamma value 139 gamma for St popn= 46.0200005

Means of q1 and q2= 8.25560167E-02 5.57688763E-04

Variances of q1 and q2= 7.51187727E-02 2.30671867E-05

Covariance and correlation of q1 and q2= -6.97391079E-06 -5.29791322E-03

F1 and F2= 0.991791070 4.13851924E-02

Freqs. of fixation of A1 for In and St= 0.913767099 0.991819382

Freqs. of fixation of A2 for In and St= 8.13683495E-02 0.00000000

Freqs. of segregation for In and St= 4.86455113E-03 8.18061829E-03

q1f= 8.17661062E-02 q2f= 0.00000000

Index of gamma value 140 gamma for St popn= 46.3516693

Means of q1 and q2= 8.10383037E-02 5.52657351E-04

Variances of q1 and q2= 7.38520697E-02 2.27177770E-05

Covariance and correlation of q1 and q2= -6.79754521E-06 -5.24792774E-03

F1 and F2= 0.991687655 4.11291718E-02

Freqs. of fixation of A1 for In and St= 0.915289998 0.991879165

Freqs. of fixation of A2 for In and St= 7.98603594E-02 0.00000000

Freqs. of segregation for In and St= 4.84964252E-03 8.12083483E-03

q1f= 8.02495405E-02 q2f= 0.00000000

Index of gamma value 141 gamma for St popn= 46.6833344

Means of q1 and q2= 7.95491710E-02 5.47697477E-04

Variances of q1 and q2= 7.26047903E-02 2.23757052E-05

Covariance and correlation of q1 and q2= -6.62581806E-06 -5.19838603E-03

F1 and F2= 0.991582870 4.08765227E-02

Freqs. of fixation of A1 for In and St= 0.916783869 0.991937697

Freqs. of fixation of A2 for In and St= 7.83807933E-02 0.00000000

Freqs. of segregation for In and St= 4.83533740E-03 8.06230307E-03

q1f= 7.87616372E-02 q2f= 0.00000000

Index of gamma value 142 gamma for St popn= 47.0150032

Means of q1 and q2= 7.80827776E-02 5.42808208E-04

Variances of q1 and q2= 7.13722482E-02 2.20407655E-05

Covariance and correlation of q1 and q2= -6.45812543E-06 -5.14906552E-03

F1 and F2= 0.991475999 4.06271219E-02

Freqs. of fixation of A1 for In and St= 0.918255150 0.991996109

Freqs. of fixation of A2 for In and St= 7.69239143E-02 0.00000000

Freqs. of segregation for In and St= 4.82093543E-03 8.00389051E-03

q1f= 7.72965550E-02 q2f= 0.00000000

Index of gamma value 143 gamma for St popn= 47.3466682

Means of q1 and q2= 7.66429156E-02 5.37988439E-04

Variances of q1 and q2= 7.01578632E-02 2.17128036E-05

Covariance and correlation of q1 and q2= -6.29477290E-06 -5.10016130E-03

F1 and F2= 0.991367400 4.03809622E-02

Freqs. of fixation of A1 for In and St= 0.919700146 0.992053986

Freqs. of fixation of A2 for In and St= 7.54934251E-02 0.00000000

Freqs. of segregation for In and St= 4.80642915E-03 7.94601440E-03

q1f= 7.58580267E-02 q2f= 0.00000000

Index of gamma value 144 gamma for St popn= 47.6783333

Means of q1 and q2= 7.52264485E-02 5.33236482E-04

Variances of q1 and q2= 6.89592212E-02 2.13915973E-05

Covariance and correlation of q1 and q2= -6.13536395E-06 -5.05152578E-03

F1 and F2= 0.991257191 4.01379317E-02

Freqs. of fixation of A1 for In and St= 0.921121836 0.992110431

Freqs. of fixation of A2 for In and St= 7.40863085E-02 0.00000000

Freqs. of segregation for In and St= 4.79185581E-03 7.88956881E-03

q1f= 7.44430274E-02 q2f= 0.00000000

Index of gamma value 145 gamma for St popn= 48.0100021

Means of q1 and q2= 7.38330856E-02 5.28550881E-04

Variances of q1 and q2= 6.77762255E-02 2.10769831E-05

Covariance and correlation of q1 and q2= -5.97980397E-06 -5.00315567E-03

F1 and F2= 0.991144836 3.98980156E-02

Freqs. of fixation of A1 for In and St= 0.922520339 0.992166996

Freqs. of fixation of A2 for In and St= 7.27021992E-02 0.00000000

Freqs. of segregation for In and St= 4.77746129E-03 7.83300400E-03

q1f= 7.30511993E-02 q2f= 0.00000000

Index of gamma value 146 gamma for St popn= 48.3416672

Means of q1 and q2= 7.24652186E-02 5.23930823E-04

Variances of q1 and q2= 6.66111484E-02 2.07687954E-05

Covariance and correlation of q1 and q2= -5.82834764E-06 -4.95525869E-03

F1 and F2= 0.991030693 3.96611206E-02

Freqs. of fixation of A1 for In and St= 0.923893452 0.992222428

Freqs. of fixation of A2 for In and St= 7.13434517E-02 0.00000000

Freqs. of segregation for In and St= 4.76309657E-03 7.77757168E-03

q1f= 7.16848969E-02 q2f= 0.00000000

Index of gamma value 147 gamma for St popn= 48.6733360

Means of q1 and q2= 7.11210147E-02 5.19373745E-04

Variances of q1 and q2= 6.54626116E-02 2.04667977E-05

Covariance and correlation of q1 and q2= -5.68060932E-06 -4.90764715E-03

F1 and F2= 0.990914643 3.94271612E-02

Freqs. of fixation of A1 for In and St= 0.925243139 0.992277563

Freqs. of fixation of A2 for In and St= 7.00083002E-02 0.00000000

Freqs. of segregation for In and St= 4.74856049E-03 7.72243738E-03

q1f= 7.03423247E-02 q2f= 0.00000000

Index of gamma value 148 gamma for St popn= 49.0050011

Means of q1 and q2= 6.97989464E-02 5.14880114E-04

Variances of q1 and q2= 6.43295050E-02 2.01709208E-05

Covariance and correlation of q1 and q2= -5.53649988E-06 -4.86034760E-03

F1 and F2= 0.990796566 3.91961373E-02

Freqs. of fixation of A1 for In and St= 0.926570475 0.992331922

Freqs. of fixation of A2 for In and St= 6.86952025E-02 0.00000000

Freqs. of segregation for In and St= 4.73432243E-03 7.66807795E-03

q1f= 6.90219775E-02 q2f= 0.00000000

Index of gamma value 149 gamma for St popn= 49.3366699

Means of q1 and q2= 6.84999675E-02 5.10447251E-04

Variances of q1 and q2= 6.32128268E-02 1.98809412E-05

Covariance and correlation of q1 and q2= -5.39600478E-06 -4.81339265E-03

F1 and F2= 0.990676820 3.89679708E-02

Freqs. of fixation of A1 for In and St= 0.927874863 0.992385924

Freqs. of fixation of A2 for In and St= 6.74051195E-02 0.00000000

Freqs. of segregation for In and St= 4.72001731E-03 7.61407614E-03

q1f= 6.77247792E-02 q2f= 0.00000000

Index of gamma value 150 gamma for St popn= 49.6683350

Means of q1 and q2= 6.72263503E-02 5.06074342E-04

Variances of q1 and q2= 6.21147156E-02 1.95967114E-05

Covariance and correlation of q1 and q2= -5.25926043E-06 -4.76689776E-03

F1 and F2= 0.990555227 3.87425944E-02

Freqs. of fixation of A1 for In and St= 0.929154038 0.992439210

Freqs. of fixation of A2 for In and St= 6.61402643E-02 0.00000000

Freqs. of segregation for In and St= 4.70569730E-03 7.56078959E-03

q1f= 6.64529726E-02 q2f= 0.00000000

Index of gamma value 151 gamma for St popn= 50.0000000

Means of q1 and q2= 6.59712180E-02 5.01761155E-04

Variances of q1 and q2= 6.10294007E-02 1.93181349E-05

Covariance and correlation of q1 and q2= -5.12565748E-06 -4.72060218E-03

F1 and F2= 0.990431249 3.85199860E-02

Freqs. of fixation of A1 for In and St= 0.930414557 0.992491722

Freqs. of fixation of A2 for In and St= 6.48938343E-02 0.00000000

Freqs. of segregation for In and St= 4.69160825E-03 7.50827789E-03

q1f= 6.51997253E-02 q2f= 0.00000000

**h=0.45**

Index of gamma value 30 gamma for St popn= 5.57203722

Means of q1 and q2= 0.463393718 1.25647811E-02

Variances of q1 and q2= 0.247837290 9.17428266E-03

Covariance and correlation of q1 and q2= -1.32553047E-03 -2.77984347E-02

F1 and F2= 0.996691406 0.739449620

Freqs. of fixation of A1 for In and St= 0.535671115 0.965058506

Freqs. of fixation of A2 for In and St= 0.458603531 5.77446306E-03

Freqs. of segregation for In and St= 5.72535396E-03 2.91670319E-02

q1f= 0.461244345 q2f= 5.94794704E-03

Index of gamma value 31 gamma for St popn= 5.75555611

Means of q1 and q2= 0.458402634 1.09987883E-02

Variances of q1 and q2= 0.247446820 7.74531160E-03

Covariance and correlation of q1 and q2= -1.09005347E-03 -2.48993170E-02

F1 and F2= 0.996685684 0.712028265

Freqs. of fixation of A1 for In and St= 0.540220559 0.966622293

Freqs. of fixation of A2 for In and St= 0.454048753 4.82009677E-03

Freqs. of segregation for In and St= 5.73068857E-03 2.85576098E-02

q1f= 0.456665754 q2f= 4.96179378E-03

Index of gamma value 32 gamma for St popn= 5.93907452

Means of q1 and q2= 0.453489840 9.67795122E-03

Variances of q1 and q2= 0.247013837 6.54673623E-03

Covariance and correlation of q1 and q2= -8.97204736E-04 -2.23109722E-02

F1 and F2= 0.996679425 0.683069646

Freqs. of fixation of A1 for In and St= 0.544766188 0.968003571

Freqs. of fixation of A2 for In and St= 0.449498355 4.02282178E-03

Freqs. of segregation for In and St= 5.73545694E-03 2.79736072E-02

q1f= 0.452091306 q2f= 4.13859310E-03

Index of gamma value 33 gamma for St popn= 6.12259293

Means of q1 and q2= 0.448643714 8.56243633E-03

Variances of q1 and q2= 0.246539608 5.54167246E-03

Covariance and correlation of q1 and q2= -7.39189563E-04 -1.99982524E-02

F1 and F2= 0.996673167 0.652796984

Freqs. of fixation of A1 for In and St= 0.549306631 0.969229221

Freqs. of fixation of A2 for In and St= 0.444953591 3.35698179E-03

Freqs. of segregation for In and St= 5.73977828E-03 2.74137966E-02

q1f= 0.447522283 q2f= 3.45160323E-03

Index of gamma value 34 gamma for St popn= 6.30611134

Means of q1 and q2= 0.443854690 7.61887524E-03

Variances of q1 and q2= 0.246024802 4.69893590E-03

Covariance and correlation of q1 and q2= -6.09651906E-04 -1.79305132E-02

F1 and F2= 0.996666372 0.621484339

Freqs. of fixation of A1 for In and St= 0.553840816 0.970322430

Freqs. of fixation of A2 for In and St= 0.440415323 2.80105136E-03

Freqs. of segregation for In and St= 5.74386120E-03 2.68765185E-02

q1f= 0.442959607 q2f= 2.87841307E-03

Index of gamma value 35 gamma for St popn= 6.48962975

Means of q1 and q2= 0.439115077 6.81933993E-03

Variances of q1 and q2= 0.245470181 3.99223715E-03

Covariance and correlation of q1 and q2= -5.03413379E-04 -1.60811525E-02

F1 and F2= 0.996659040 0.589448333

Freqs. of fixation of A1 for In and St= 0.558367610 0.971301794

Freqs. of fixation of A2 for In and St= 0.435884535 2.33698240E-03

Freqs. of segregation for In and St= 5.74785471E-03 2.63612233E-02

q1f= 0.438404411 q2f= 2.40025599E-03

Index of gamma value 36 gamma for St popn= 6.67314863

Means of q1 and q2= 0.434418410 6.14045421E-03

Variances of q1 and q2= 0.244876295 3.39945406E-03

Covariance and correlation of q1 and q2= -4.16241819E-04 -1.44267250E-02

F1 and F2= 0.996651351 0.557036519

Freqs. of fixation of A1 for In and St= 0.562886178 0.972184420

Freqs. of fixation of A2 for In and St= 0.431362569 1.94966502E-03

Freqs. of segregation for In and St= 5.75125217E-03 2.58659162E-02

q1f= 0.433857799 q2f= 2.00143387E-03

Index of gamma value 37 gamma for St popn= 6.85666704

Means of q1 and q2= 0.429759651 5.56269241E-03

Variances of q1 and q2= 0.244243845 2.90201558E-03

Covariance and correlation of q1 and q2= -3.44687141E-04 -1.29468283E-02

F1 and F2= 0.996643960 0.524610817

Freqs. of fixation of A1 for In and St= 0.567395389 0.972983897

Freqs. of fixation of A2 for In and St= 0.426850051 1.62645068E-03

Freqs. of segregation for In and St= 5.75456023E-03 2.53896527E-02

q1f= 0.429320604 q2f= 1.66882155E-03

Index of gamma value 38 gamma for St popn= 7.04018545

Means of q1 and q2= 0.425135016 5.06972289E-03

Variances of q1 and q2= 0.243572965 2.48434371E-03

Covariance and correlation of q1 and q2= -2.85925227E-04 -1.16233705E-02

F1 and F2= 0.996635497 0.492532402

Freqs. of fixation of A1 for In and St= 0.571894348 0.973711491

Freqs. of fixation of A2 for In and St= 0.422348201 1.35676167E-03

Freqs. of segregation for In and St= 5.75745106E-03 2.49317475E-02

q1f= 0.424793929 q2f= 1.39145297E-03

Index of gamma value 39 gamma for St popn= 7.22370434

Means of q1 and q2= 0.420541048 4.64789290E-03

Variances of q1 and q2= 0.242864266 2.13338924E-03

Covariance and correlation of q1 and q2= -2.37647211E-04 -1.04403691E-02

F1 and F2= 0.996626794 0.461144745

Freqs. of fixation of A1 for In and St= 0.576382339 0.974377692

Freqs. of fixation of A2 for In and St= 0.417857677 1.13175437E-03

Freqs. of segregation for In and St= 5.75998425E-03 2.44905539E-02

q1f= 0.420278460 q2f= 1.16016751E-03

Index of gamma value 40 gamma for St popn= 7.40722275

Means of q1 and q2= 0.415975422 4.28580539E-03

Variances of q1 and q2= 0.242118195 1.83823472E-03

Covariance and correlation of q1 and q2= -1.97963091E-04 -9.38361138E-03

F1 and F2= 0.996617854 0.430758446

Freqs. of fixation of A1 for In and St= 0.580858290 0.974990308

Freqs. of fixation of A2 for In and St= 0.413379461 9.44041123E-04

Freqs. of segregation for In and St= 5.76224923E-03 2.40656510E-02

q1f= 0.415775239 q2f= 9.67320346E-04

Index of gamma value 41 gamma for St popn= 7.59074116

Means of q1 and q2= 0.411435813 3.97392828E-03

Variances of q1 and q2= 0.241335094 1.58974563E-03

Covariance and correlation of q1 and q2= -1.65325706E-04 -8.44046380E-03

F1 and F2= 0.996608496 0.401639938

Freqs. of fixation of A1 for In and St= 0.585321248 0.975556910

Freqs. of fixation of A2 for In and St= 0.408914357 7.87448429E-04

Freqs. of segregation for In and St= 5.76439500E-03 2.36556418E-02

q1f= 0.411285162 q2f= 8.06527387E-04

Index of gamma value 42 gamma for St popn= 7.77425957

Means of q1 and q2= 0.406921118 3.70430341E-03

Variances of q1 and q2= 0.240515471 1.38029340E-03

Covariance and correlation of q1 and q2= -1.38469040E-04 -7.59968301E-03

F1 and F2= 0.996598721 0.374004304

Freqs. of fixation of A1 for In and St= 0.589770675 0.976083219

Freqs. of fixation of A2 for In and St= 0.404463321 6.56824675E-04

Freqs. of segregation for In and St= 5.76600432E-03 2.32599564E-02

q1f= 0.406809002 q2f= 6.72466180E-04

Index of gamma value 43 gamma for St popn= 7.95777798

Means of q1 and q2= 0.402430058 3.47027066E-03

Variances of q1 and q2= 0.239659771 1.20349950E-03

Covariance and correlation of q1 and q2= -1.16354320E-04 -6.85112644E-03

F1 and F2= 0.996588767 0.348010451

Freqs. of fixation of A1 for In and St= 0.594205499 0.976574063

Freqs. of fixation of A2 for In and St= 0.400026947 5.47865813E-04

Freqs. of segregation for In and St= 5.76755404E-03 2.28780713E-02

q1f= 0.402347535 q2f= 5.60693385E-04

Index of gamma value 44 gamma for St popn= 8.14129639

Means of q1 and q2= 0.397961915 3.26626794E-03

Variances of q1 and q2= 0.238768339 1.05404039E-03

Covariance and correlation of q1 and q2= -9.81315970E-05 -6.18574303E-03

F1 and F2= 0.996577919 0.323762298

Freqs. of fixation of A1 for In and St= 0.598625183 0.977034330

Freqs. of fixation of A2 for In and St= 0.395606190 4.56981972E-04

Freqs. of segregation for In and St= 5.76862693E-03 2.25086883E-02

q1f= 0.397901535 q2f= 4.67504899E-04

Index of gamma value 45 gamma for St popn= 8.32481575

Means of q1 and q2= 0.393516064 3.08763538E-03

Variances of q1 and q2= 0.237841815 9.27467423E-04

Covariance and correlation of q1 and q2= -8.31037760E-05 -5.59535110E-03

F1 and F2= 0.996566892 0.301311463

Freqs. of fixation of A1 for In and St= 0.603028953 0.977467537

Freqs. of fixation of A2 for In and St= 0.391201675 3.81175167E-04

Freqs. of segregation for In and St= 5.76937199E-03 2.21512876E-02

q1f= 0.393471748 q2f= 3.89809953E-04

Index of gamma value 46 gamma for St popn= 8.50833416

Means of q1 and q2= 0.389092088 2.93048401E-03

Variances of q1 and q2= 0.236880764 8.20069457E-04

Covariance and correlation of q1 and q2= -7.06993742E-05 -5.07253828E-03

F1 and F2= 0.996555865 0.280663431

Freqs. of fixation of A1 for In and St= 0.607415974 0.977876484

Freqs. of fixation of A2 for In and St= 0.386814266 3.17945814E-04

Freqs. of segregation for In and St= 5.76975942E-03 2.18055695E-02

q1f= 0.389059037 q2f= 3.25033354E-04

Index of gamma value 47 gamma for St popn= 8.69185257

Means of q1 and q2= 0.384690046 2.79154815E-03

Variances of q1 and q2= 0.235885516 7.28745828E-04

Covariance and correlation of q1 and q2= -6.04495872E-05 -4.61057015E-03

F1 and F2= 0.996543765 0.261785150

Freqs. of fixation of A1 for In and St= 0.611785471 0.978264093

Freqs. of fixation of A2 for In and St= 0.382444441 2.65207404E-04

Freqs. of segregation for In and St= 5.77008724E-03 2.14706995E-02

q1f= 0.384663999 q2f= 2.71026540E-04

Index of gamma value 48 gamma for St popn= 8.87537098

Means of q1 and q2= 0.380309671 2.66809482E-03

Variances of q1 and q2= 0.234856844 6.50905946E-04

Covariance and correlation of q1 and q2= -5.19690802E-05 -4.20324085E-03

F1 and F2= 0.996531785 0.244611725

Freqs. of fixation of A1 for In and St= 0.616136968 0.978632569

Freqs. of fixation of A2 for In and St= 0.378093004 2.21219045E-04

Freqs. of segregation for In and St= 5.77002764E-03 2.11462118E-02

q1f= 0.380287260 q2f= 2.25998054E-04

Index of gamma value 49 gamma for St popn= 9.05888939

Means of q1 and q2= 0.375536829 2.62889569E-03

Variances of q1 and q2= 0.233678594 5.72437770E-04

Covariance and correlation of q1 and q2= -4.27413033E-05 -3.69550800E-03

F1 and F2= 0.996459246 0.218322322

Freqs. of fixation of A1 for In and St= 0.621091068 0.977313995

Freqs. of fixation of A2 for In and St= 0.373043597 1.79579583E-04

Freqs. of segregation for In and St= 5.86533546E-03 2.25064252E-02

q1f= 0.375244528 q2f= 1.83714335E-04

Index of gamma value 50 gamma for St popn= 9.24240780

Means of q1 and q2= 0.371191204 2.53044884E-03

Variances of q1 and q2= 0.232578799 5.16728440E-04

Covariance and correlation of q1 and q2= -3.72100039E-05 -3.39424703E-03

F1 and F2= 0.996446192 0.204722300

Freqs. of fixation of A1 for In and St= 0.625415921 0.977654219

Freqs. of fixation of A2 for In and St= 0.368720770 1.49722197E-04

Freqs. of segregation for In and St= 5.86330891E-03 2.21960600E-02

q1f= 0.370895445 q2f= 1.53120869E-04

Index of gamma value 51 gamma for St popn= 9.42592621

Means of q1 and q2= 0.366867900 2.44148122E-03

Variances of q1 and q2= 0.231447250 4.68849699E-04

Covariance and correlation of q1 and q2= -3.26125883E-05 -3.13070696E-03

F1 and F2= 0.996432722 0.192504928

Freqs. of fixation of A1 for In and St= 0.629720390 0.977980673

Freqs. of fixation of A2 for In and St= 0.364418656 1.24831364E-04

Freqs. of segregation for In and St= 5.86095452E-03 2.18944959E-02

q1f= 0.366567075 q2f= 1.27625666E-04

Index of gamma value 52 gamma for St popn= 9.60944462

Means of q1 and q2= 0.362567186 2.36065662E-03

Variances of q1 and q2= 0.230284587 4.27564140E-04

Covariance and correlation of q1 and q2= -2.87837465E-05 -2.90077622E-03

F1 and F2= 0.996418893 0.181549415

Freqs. of fixation of A1 for In and St= 0.634004116 0.978294969

Freqs. of fixation of A2 for In and St= 0.360137701 1.04080369E-04

Freqs. of segregation for In and St= 5.85818291E-03 2.16009505E-02

q1f= 0.362259895 q2f= 1.06378240E-04

Index of gamma value 53 gamma for St popn= 9.79296303

Means of q1 and q2= 0.358289570 2.28685350E-03

Variances of q1 and q2= 0.229091525 3.91838548E-04

Covariance and correlation of q1 and q2= -2.55868654E-05 -2.70059216E-03

F1 and F2= 0.996404767 0.171736702

Freqs. of fixation of A1 for In and St= 0.638265967 0.978597701

Freqs. of fixation of A2 for In and St= 0.355878681 8.67808994E-05

Freqs. of segregation for In and St= 5.85535169E-03 2.13155188E-02

q1f= 0.357974738 q2f= 8.86709677E-05

Index of gamma value 54 gamma for St popn= 9.97648239

Means of q1 and q2= 0.354034841 2.21912353E-03

Variances of q1 and q2= 0.227868527 3.60807200E-04

Covariance and correlation of q1 and q2= -2.29100115E-05 -2.52665277E-03

F1 and F2= 0.996389747 0.162951574

Freqs. of fixation of A1 for In and St= 0.642506242 0.978890121

Freqs. of fixation of A2 for In and St= 0.351641744 7.23572448E-05

Freqs. of segregation for In and St= 5.85201383E-03 2.10375227E-02

q1f= 0.353711665 q2f= 7.39121751E-05

Index of gamma value 55 gamma for St popn= 10.1600008

Means of q1 and q2= 0.349803984 2.15667277E-03

Variances of q1 and q2= 0.226616561 3.33748321E-04

Covariance and correlation of q1 and q2= -2.06615659E-05 -2.37578899E-03

F1 and F2= 0.996374428 0.155085966

Freqs. of fixation of A1 for In and St= 0.646723449 0.979172766

Freqs. of fixation of A2 for In and St= 0.347427905 6.03326262E-05

Freqs. of segregation for In and St= 5.84864616E-03 2.07669009E-02

q1f= 0.349471837 q2f= 6.16121179E-05

Index of gamma value 56 gamma for St popn= 10.3435192

Means of q1 and q2= 0.345597267 2.09882250E-03

Variances of q1 and q2= 0.225336328 3.10055300E-04

Covariance and correlation of q1 and q2= -1.87666737E-05 -2.24518497E-03

F1 and F2= 0.996358931 0.148038909

Freqs. of fixation of A1 for In and St= 0.650917530 0.979446471

Freqs. of fixation of A2 for In and St= 0.343237609 5.03071715E-05

Freqs. of segregation for In and St= 5.84486127E-03 2.05032229E-02

q1f= 0.345255584 q2f= 5.13602208E-05

Index of gamma value 57 gamma for St popn= 10.5270376

Means of q1 and q2= 0.341414630 2.04500114E-03

Variances of q1 and q2= 0.224028438 2.89220363E-04

Covariance and correlation of q1 and q2= -1.71626452E-05 -2.13215221E-03

F1 and F2= 0.996343136 0.141717777

Freqs. of fixation of A1 for In and St= 0.655088127 0.979711831

Freqs. of fixation of A2 for In and St= 0.339070916 4.19480602E-05

Freqs. of segregation for In and St= 5.84095716E-03 2.02462208E-02

q1f= 0.341063052 q2f= 4.28148996E-05

Index of gamma value 58 gamma for St popn= 10.7105560

Means of q1 and q2= 0.337257415 1.99472462E-03

Variances of q1 and q2= 0.222693771 2.70818651E-04

Covariance and correlation of q1 and q2= -1.57997711E-05 -2.03449884E-03

F1 and F2= 0.996326506 0.136038795

Freqs. of fixation of A1 for In and St= 0.659234107 0.979969919

Freqs. of fixation of A2 for In and St= 0.334929317 3.49792317E-05

Freqs. of segregation for In and St= 5.83657622E-03 1.99951027E-02

q1f= 0.336895645 q2f= 3.56929158E-05

Index of gamma value 59 gamma for St popn= 10.8940744

Means of q1 and q2= 0.333125174 1.94757793E-03

Variances of q1 and q2= 0.221332937 2.54491722E-04

Covariance and correlation of q1 and q2= -1.46356178E-05 -1.95007445E-03

F1 and F2= 0.996309459 0.130925864

Freqs. of fixation of A1 for In and St= 0.663355410 0.980220020

Freqs. of fixation of A2 for In and St= 0.330812186 2.91683027E-05

Freqs. of segregation for In and St= 5.83240390E-03 1.97508112E-02

q1f= 0.332752943 q2f= 2.97560091E-05

Index of gamma value 60 gamma for St popn= 11.0775928

Means of q1 and q2= 0.329019159 1.90320879E-03

Variances of q1 and q2= 0.219946980 2.39939793E-04

Covariance and correlation of q1 and q2= -1.36368908E-05 -1.87717692E-03

F1 and F2= 0.996292174 0.126311585

Freqs. of fixation of A1 for In and St= 0.667451203 0.980463386

Freqs. of fixation of A2 for In and St= 0.326721132 2.43235936E-05

Freqs. of segregation for In and St= 5.82766533E-03 1.95122901E-02

q1f= 0.328636318 q2f= 2.48076485E-05

Index of gamma value 61 gamma for St popn= 11.2611122

Means of q1 and q2= 0.324938983 1.86131860E-03

Variances of q1 and q2= 0.218536407 2.26909629E-04

Covariance and correlation of q1 and q2= -1.27742533E-05 -1.81404129E-03

F1 and F2= 0.996274412 0.122135334

Freqs. of fixation of A1 for In and St= 0.671521425 0.980700493

Freqs. of fixation of A2 for In and St= 0.322655737 2.02837400E-05

Freqs. of segregation for In and St= 5.82283735E-03 1.92792229E-02

q1f= 0.324545503 q2f= 2.06824825E-05

Index of gamma value 62 gamma for St popn= 11.4446306

Means of q1 and q2= 0.320885390 1.82164425E-03

Variances of q1 and q2= 0.217102081 2.15188644E-04

Covariance and correlation of q1 and q2= -1.20255281E-05 -1.75939151E-03

F1 and F2= 0.996256053 0.118344381

Freqs. of fixation of A1 for In and St= 0.675565779 0.980931640

Freqs. of fixation of A2 for In and St= 0.318616718 1.69151663E-05

Freqs. of segregation for In and St= 5.81750274E-03 1.90514456E-02

q1f= 0.320481122 q2f= 1.72436830E-05

Index of gamma value 63 gamma for St popn= 11.6281490

Means of q1 and q2= 0.316858739 1.78396725E-03

Variances of q1 and q2= 0.215644777 2.04597003E-04

Covariance and correlation of q1 and q2= -1.13705755E-05 -1.71184062E-03

F1 and F2= 0.996237159 0.114891492

Freqs. of fixation of A1 for In and St= 0.679583132 0.981156707

Freqs. of fixation of A2 for In and St= 0.314604551 1.41063392E-05

Freqs. of segregation for In and St= 5.81231713E-03 1.88291874E-02

q1f= 0.316443801 q2f= 1.43770467E-05

Index of gamma value 64 gamma for St popn= 11.8116674

Means of q1 and q2= 0.312859714 1.74809573E-03

Variances of q1 and q2= 0.214165449 1.94983193E-04

Covariance and correlation of q1 and q2= -1.07951346E-05 -1.67053181E-03

F1 and F2= 0.996217906 0.111735664

Freqs. of fixation of A1 for In and St= 0.683573484 0.981376529

Freqs. of fixation of A2 for In and St= 0.310619891 1.17642066E-05

Freqs. of segregation for In and St= 5.80662489E-03 1.86117068E-02

q1f= 0.312434077 q2f= 1.19873112E-05

Index of gamma value 65 gamma for St popn= 11.9951859

Means of q1 and q2= 0.308888316 1.71386206E-03

Variances of q1 and q2= 0.212664723 1.86218531E-04

Covariance and correlation of q1 and q2= -1.02851773E-05 -1.63437834E-03

F1 and F2= 0.996198118 0.108840868

Freqs. of fixation of A1 for In and St= 0.687536418 0.981591046

Freqs. of fixation of A2 for In and St= 0.306662768 0.00000000

Freqs. of segregation for In and St= 5.80081344E-03 1.84089541E-02

q1f= 0.308452040 q2f= 0.00000000

Index of gamma value 66 gamma for St popn= 12.1787043

Means of q1 and q2= 0.304945141 1.68112444E-03

Variances of q1 and q2= 0.211143613 1.78194372E-04

Covariance and correlation of q1 and q2= -9.82981874E-06 -1.60254247E-03

F1 and F2= 0.996178448 0.106175631

Freqs. of fixation of A1 for In and St= 0.691471338 0.981800497

Freqs. of fixation of A2 for In and St= 0.302733898 0.00000000

Freqs. of segregation for In and St= 5.79476357E-03 1.81995034E-02

q1f= 0.304498404 q2f= 0.00000000

Index of gamma value 67 gamma for St popn= 12.3622227

Means of q1 and q2= 0.301030487 1.64975657E-03

Variances of q1 and q2= 0.209602624 1.70818268E-04

Covariance and correlation of q1 and q2= -9.42105544E-06 -1.57446868E-03

F1 and F2= 0.996157587 0.103712596

Freqs. of fixation of A1 for In and St= 0.695378244 0.982005298

Freqs. of fixation of A2 for In and St= 0.298833340 0.00000000

Freqs. of segregation for In and St= 5.78841567E-03 1.79947019E-02

q1f= 0.300573200 q2f= 0.00000000

Index of gamma value 68 gamma for St popn= 12.5457411

Means of q1 and q2= 0.297144651 1.61964761E-03

Variances of q1 and q2= 0.208042771 1.64011348E-04

Covariance and correlation of q1 and q2= -9.05196066E-06 -1.54963497E-03

F1 and F2= 0.996136248 0.101427875

Freqs. of fixation of A1 for In and St= 0.699256361 0.982205391

Freqs. of fixation of A2 for In and St= 0.294961601 0.00000000

Freqs. of segregation for In and St= 5.78203797E-03 1.77946091E-02

q1f= 0.296676993 q2f= 0.00000000

Index of gamma value 69 gamma for St popn= 12.7292595

Means of q1 and q2= 0.293288022 1.59070187E-03

Variances of q1 and q2= 0.206464872 1.57706570E-04

Covariance and correlation of q1 and q2= -8.71552038E-06 -1.52737380E-03

F1 and F2= 0.996114790 9.93007198E-02

Freqs. of fixation of A1 for In and St= 0.703105569 0.982401192

Freqs. of fixation of A2 for In and St= 0.291119069 0.00000000

Freqs. of segregation for In and St= 5.77536225E-03 1.75988078E-02

q1f= 0.292810142 q2f= 0.00000000

Index of gamma value 70 gamma for St popn= 12.9127789

Means of q1 and q2= 0.289460868 1.56283460E-03

Variances of q1 and q2= 0.204869658 1.51846456E-04

Covariance and correlation of q1 and q2= -8.40745633E-06 -1.50738261E-03

F1 and F2= 0.996092796 9.73130092E-02

Freqs. of fixation of A1 for In and St= 0.706925511 0.982592762

Freqs. of fixation of A2 for In and St= 0.287305921 0.00000000

Freqs. of segregation for In and St= 5.76856732E-03 1.74072385E-02

q1f= 0.288972884 q2f= 0.00000000

Index of gamma value 71 gamma for St popn= 13.0962973

Means of q1 and q2= 0.285663605 1.53596851E-03

Variances of q1 and q2= 0.203257918 1.46381863E-04

Covariance and correlation of q1 and q2= -8.12334474E-06 -1.48924964E-03

F1 and F2= 0.996069789 9.54492539E-02

Freqs. of fixation of A1 for In and St= 0.710715830 0.982780218

Freqs. of fixation of A2 for In and St= 0.283522636 0.00000000

Freqs. of segregation for In and St= 5.76153398E-03 1.72197819E-02

q1f= 0.285165608 q2f= 0.00000000

Index of gamma value 72 gamma for St popn= 13.2798157

Means of q1 and q2= 0.281896621 1.51003944E-03

Variances of q1 and q2= 0.201630697 1.41270531E-04

Covariance and correlation of q1 and q2= -7.85995508E-06 -1.47270563E-03

F1 and F2= 0.996046960 9.36956853E-02

Freqs. of fixation of A1 for In and St= 0.714476168 0.982963979

Freqs. of fixation of A2 for In and St= 0.279769689 0.00000000

Freqs. of segregation for In and St= 5.75414300E-03 1.70360208E-02

q1f= 0.281388819 q2f= 0.00000000

Index of gamma value 73 gamma for St popn= 13.4633341

Means of q1 and q2= 0.278159767 1.48498616E-03

Variances of q1 and q2= 0.199988455 1.36475908E-04

Covariance and correlation of q1 and q2= -7.61458068E-06 -1.45752332E-03

F1 and F2= 0.996023357 9.20405015E-02

Freqs. of fixation of A1 for In and St= 0.718206525 0.983143926

Freqs. of fixation of A2 for In and St= 0.276046842 0.00000000

Freqs. of segregation for In and St= 5.74663281E-03 1.68560743E-02

q1f= 0.277642339 q2f= 0.00000000

Index of gamma value 74 gamma for St popn= 13.6468525

Means of q1 and q2= 0.274454087 1.46075350E-03

Variances of q1 and q2= 0.198332310 1.31966590E-04

Covariance and correlation of q1 and q2= -7.38439849E-06 -1.44339888E-03

F1 and F2= 0.995998979 9.04736072E-02

Freqs. of fixation of A1 for In and St= 0.721906006 0.983320117

Freqs. of fixation of A2 for In and St= 0.272354990 0.00000000

Freqs. of segregation for In and St= 5.73900342E-03 1.66798830E-02

q1f= 0.273927063 q2f= 0.00000000

Index of gamma value 75 gamma for St popn= 13.8303709

Means of q1 and q2= 0.270778805 1.43729651E-03

Variances of q1 and q2= 0.196662724 1.27714986E-04

Covariance and correlation of q1 and q2= -7.16777868E-06 -1.43021927E-03

F1 and F2= 0.995974183 8.89856815E-02

Freqs. of fixation of A1 for In and St= 0.725575149 0.983493090

Freqs. of fixation of A2 for In and St= 0.268693656 0.00000000

Freqs. of segregation for In and St= 5.73119521E-03 1.65069103E-02

q1f= 0.270242482 q2f= 0.00000000

Index of gamma value 76 gamma for St popn= 14.0138893

Means of q1 and q2= 0.267135054 1.41456828E-03

Variances of q1 and q2= 0.194980845 1.23697406E-04

Covariance and correlation of q1 and q2= -6.96271309E-06 -1.41775841E-03

F1 and F2= 0.995948970 8.75692144E-02

Freqs. of fixation of A1 for In and St= 0.729212999 0.983662248

Freqs. of fixation of A2 for In and St= 0.265063822 0.00000000

Freqs. of segregation for In and St= 5.72317839E-03 1.63377523E-02

q1f= 0.266589552 q2f= 0.00000000

Index of gamma value 77 gamma for St popn= 14.1974087

Means of q1 and q2= 0.263522267 1.39252923E-03

Variances of q1 and q2= 0.193287060 1.19893026E-04

Covariance and correlation of q1 and q2= -6.76827040E-06 -1.40598067E-03

F1 and F2= 0.995923162 8.62173736E-02

Freqs. of fixation of A1 for In and St= 0.732820094 0.983828425

Freqs. of fixation of A2 for In and St= 0.261464983 0.00000000

Freqs. of segregation for In and St= 5.71492314E-03 1.61715746E-02

q1f= 0.262967825 q2f= 0.00000000

Index of gamma value 78 gamma for St popn= 14.3809271

Means of q1 and q2= 0.259941399 1.37114502E-03

Variances of q1 and q2= 0.191582590 1.16283692E-04

Covariance and correlation of q1 and q2= -6.58308272E-06 -1.39473565E-03

F1 and F2= 0.995897114 8.49241689E-02

Freqs. of fixation of A1 for In and St= 0.736395419 0.983991325

Freqs. of fixation of A2 for In and St= 0.257898003 0.00000000

Freqs. of segregation for In and St= 5.70657849E-03 1.60086751E-02

q1f= 0.259378165 q2f= 0.00000000

Index of gamma value 79 gamma for St popn= 14.5644455

Means of q1 and q2= 0.256392241 1.35038316E-03

Variances of q1 and q2= 0.189867914 1.12853311E-04

Covariance and correlation of q1 and q2= -6.40642247E-06 -1.38398900E-03

F1 and F2= 0.995870292 8.36843327E-02

Freqs. of fixation of A1 for In and St= 0.739939332 0.984151363

Freqs. of fixation of A2 for In and St= 0.254362673 0.00000000

Freqs. of segregation for In and St= 5.69799542E-03 1.58486366E-02

q1f= 0.255820334 q2f= 0.00000000

Index of gamma value 80 gamma for St popn= 14.7479639

Means of q1 and q2= 0.252875388 1.33021025E-03

Variances of q1 and q2= 0.188143983 1.09587680E-04

Covariance and correlation of q1 and q2= -6.23724191E-06 -1.37361989E-03

F1 and F2= 0.995842695 8.24934617E-02

Freqs. of fixation of A1 for In and St= 0.743451238 0.984308183

Freqs. of fixation of A2 for In and St= 0.250859588 0.00000000

Freqs. of segregation for In and St= 5.68917394E-03 1.56918168E-02

q1f= 0.252294928 q2f= 0.00000000

Index of gamma value 81 gamma for St popn= 14.9314823

Means of q1 and q2= 0.249390393 1.31060020E-03

Variances of q1 and q2= 0.186411411 1.06474276E-04

Covariance and correlation of q1 and q2= -6.07426045E-06 -1.36343623E-03

F1 and F2= 0.995814919 8.13474655E-02

Freqs. of fixation of A1 for In and St= 0.746931255 0.984462261

Freqs. of fixation of A2 for In and St= 0.247388378 0.00000000

Freqs. of segregation for In and St= 5.68036735E-03 1.55377388E-02

q1f= 0.248801664 q2f= 0.00000000

Index of gamma value 82 gamma for St popn= 15.1150007

Means of q1 and q2= 0.245937675 1.29152671E-03

Variances of q1 and q2= 0.184670925 1.03501974E-04

Covariance and correlation of q1 and q2= -5.91826392E-06 -1.35369471E-03

F1 and F2= 0.995786488 8.02428797E-02

Freqs. of fixation of A1 for In and St= 0.750379443 0.984613597

Freqs. of fixation of A2 for In and St= 0.243949428 0.00000000

Freqs. of segregation for In and St= 5.67112863E-03 1.53864026E-02

q1f= 0.245340794 q2f= 0.00000000

Index of gamma value 83 gamma for St popn= 15.2985191

Means of q1 and q2= 0.242517352 1.27296650E-03

Variances of q1 and q2= 0.182923287 1.00660662E-04

Covariance and correlation of q1 and q2= -5.76738967E-06 -1.34404842E-03

F1 and F2= 0.995757222 7.91764483E-02

Freqs. of fixation of A1 for In and St= 0.753795207 0.984762132

Freqs. of fixation of A2 for In and St= 0.240542844 0.00000000

Freqs. of segregation for In and St= 5.66194952E-03 1.52378678E-02

q1f= 0.241912544 q2f= 0.00000000

Index of gamma value 84 gamma for St popn= 15.4820375

Means of q1 and q2= 0.239129901 1.25489652E-03

Variances of q1 and q2= 0.181169480 9.79416800E-05

Covariance and correlation of q1 and q2= -5.62198693E-06 -1.33463822E-03

F1 and F2= 0.995727837 7.81456754E-02

Freqs. of fixation of A1 for In and St= 0.757178247 0.984907985

Freqs. of fixation of A2 for In and St= 0.237169117 0.00000000

Freqs. of segregation for In and St= 5.65263629E-03 1.50920153E-02

q1f= 0.238517374 q2f= 0.00000000

Index of gamma value 85 gamma for St popn= 15.6655560

Means of q1 and q2= 0.235774860 1.23729557E-03

Variances of q1 and q2= 0.179409847 9.53365816E-05

Covariance and correlation of q1 and q2= -5.48129901E-06 -1.32534991E-03

F1 and F2= 0.995697677 7.71478415E-02

Freqs. of fixation of A1 for In and St= 0.760529280 0.985051513

Freqs. of fixation of A2 for In and St= 0.233827800 0.00000000

Freqs. of segregation for In and St= 5.64292073E-03 1.49484873E-02

q1f= 0.235154748 q2f= 0.00000000

Index of gamma value 86 gamma for St popn= 15.8490753

Means of q1 and q2= 0.232452393 1.22014573E-03

Variances of q1 and q2= 0.177645162 9.28384325E-05

Covariance and correlation of q1 and q2= -5.34535502E-06 -1.31624262E-03

F1 and F2= 0.995666802 7.61809424E-02

Freqs. of fixation of A1 for In and St= 0.763847768 0.985192239

Freqs. of fixation of A2 for In and St= 0.230518907 0.00000000

Freqs. of segregation for In and St= 5.63332438E-03 1.48077607E-02

q1f= 0.231824860 q2f= 0.00000000

Index of gamma value 87 gamma for St popn= 16.0325928

Means of q1 and q2= 0.229163274 1.20342779E-03

Variances of q1 and q2= 0.175876468 9.04405242E-05

Covariance and correlation of q1 and q2= -5.21354377E-06 -1.30721682E-03

F1 and F2= 0.995635390 7.52429813E-02

Freqs. of fixation of A1 for In and St= 0.767133176 0.985330760

Freqs. of fixation of A2 for In and St= 0.227243364 0.00000000

Freqs. of segregation for In and St= 5.62345982E-03 1.46692395E-02

q1f= 0.228528485 q2f= 0.00000000

Index of gamma value 88 gamma for St popn= 16.2161121

Means of q1 and q2= 0.225906700 1.18712499E-03

Variances of q1 and q2= 0.174104005 8.81367741E-05

Covariance and correlation of q1 and q2= -5.08569065E-06 -1.29827578E-03

F1 and F2= 0.995603323 7.43321329E-02

Freqs. of fixation of A1 for In and St= 0.770386159 0.985466897

Freqs. of fixation of A2 for In and St= 0.224000394 0.00000000

Freqs. of segregation for In and St= 5.61344624E-03 1.45331025E-02

q1f= 0.225264907 q2f= 0.00000000

Index of gamma value 89 gamma for St popn= 16.3996315

Means of q1 and q2= 0.222683251 1.17122079E-03

Variances of q1 and q2= 0.172328740 8.59218489E-05

Covariance and correlation of q1 and q2= -4.96167922E-06 -1.28943077E-03

F1 and F2= 0.995570779 7.34469518E-02

Freqs. of fixation of A1 for In and St= 0.773606062 0.985600412

Freqs. of fixation of A2 for In and St= 0.220790446 0.00000000

Freqs. of segregation for In and St= 5.60349226E-03 1.43995881E-02

q1f= 0.222034618 q2f= 0.00000000

Index of gamma value 90 gamma for St popn= 16.5831490

Means of q1 and q2= 0.219492897 1.15570112E-03

Variances of q1 and q2= 0.170551300 8.37907501E-05

Covariance and correlation of q1 and q2= -4.84133488E-06 -1.28067692E-03

F1 and F2= 0.995537698 7.25859776E-02

Freqs. of fixation of A1 for In and St= 0.776793301 0.985732257

Freqs. of fixation of A2 for In and St= 0.217613608 0.00000000

Freqs. of segregation for In and St= 5.59309125E-03 1.42677426E-02

q1f= 0.218837574 q2f= 0.00000000

Index of gamma value 91 gamma for St popn= 16.7666683

Means of q1 and q2= 0.216336116 1.14054990E-03

Variances of q1 and q2= 0.168772534 8.17387554E-05

Covariance and correlation of q1 and q2= -4.72416286E-06 -1.27192051E-03

F1 and F2= 0.995503783 7.17479214E-02

Freqs. of fixation of A1 for In and St= 0.779946923 0.985861659

Freqs. of fixation of A2 for In and St= 0.214470252 0.00000000

Freqs. of segregation for In and St= 5.58282435E-03 1.41383410E-02

q1f= 0.215674326 q2f= 0.00000000

Index of gamma value 92 gamma for St popn= 16.9501858

Means of q1 and q2= 0.213211700 1.12575572E-03

Variances of q1 and q2= 0.166992456 7.97618923E-05

Covariance and correlation of q1 and q2= -4.61048330E-06 -1.26328191E-03

F1 and F2= 0.995469391 7.09317178E-02

Freqs. of fixation of A1 for In and St= 0.783068419 0.985988557

Freqs. of fixation of A2 for In and St= 0.211359188 0.00000000

Freqs. of segregation for In and St= 5.57239354E-03 1.40114427E-02

q1f= 0.212543562 q2f= 0.00000000

Index of gamma value 93 gamma for St popn= 17.1337051

Means of q1 and q2= 0.210120738 1.11130427E-03

Variances of q1 and q2= 0.165212244 7.78560134E-05

Covariance and correlation of q1 and q2= -4.49971412E-06 -1.25463598E-03

F1 and F2= 0.995434284 7.01361820E-02

Freqs. of fixation of A1 for In and St= 0.786156714 0.986113906

Freqs. of fixation of A2 for In and St= 0.208281547 0.00000000

Freqs. of segregation for In and St= 5.56173921E-03 1.38860941E-02

q1f= 0.209446430 q2f= 0.00000000

Index of gamma value 94 gamma for St popn= 17.3172226

Means of q1 and q2= 0.207062498 1.09718379E-03

Variances of q1 and q2= 0.163432121 7.60176190E-05

Covariance and correlation of q1 and q2= -4.39179712E-06 -1.24599540E-03

F1 and F2= 0.995398521 6.93604127E-02

Freqs. of fixation of A1 for In and St= 0.789212346 0.986237109

Freqs. of fixation of A2 for In and St= 0.205236688 0.00000000

Freqs. of segregation for In and St= 5.55096567E-03 1.37628913E-02

q1f= 0.206382319 q2f= 0.00000000

Index of gamma value 95 gamma for St popn= 17.5007420

Means of q1 and q2= 0.204037786 1.08338322E-03

Variances of q1 and q2= 0.161653146 7.42432676E-05

Covariance and correlation of q1 and q2= -4.28690691E-06 -1.23743794E-03

F1 and F2= 0.995362103 6.86034113E-02

Freqs. of fixation of A1 for In and St= 0.792234778 0.986358464

Freqs. of fixation of A2 for In and St= 0.202225223 0.00000000

Freqs. of segregation for In and St= 5.53999841E-03 1.36415362E-02

q1f= 0.203351796 q2f= 0.00000000

Index of gamma value 96 gamma for St popn= 17.6842594

Means of q1 and q2= 0.201046139 1.06989138E-03

Variances of q1 and q2= 0.159875676 7.25299251E-05

Covariance and correlation of q1 and q2= -4.18476702E-06 -1.22891390E-03

F1 and F2= 0.995325089 6.78644702E-02

Freqs. of fixation of A1 for In and St= 0.795224249 0.986477852

Freqs. of fixation of A2 for In and St= 0.199246809 0.00000000

Freqs. of segregation for In and St= 5.52894175E-03 1.35221481E-02

q1f= 0.200354561 q2f= 0.00000000

Index of gamma value 97 gamma for St popn= 17.8677788

Means of q1 and q2= 0.198087469 1.05669757E-03

Variances of q1 and q2= 0.158100232 7.08746666E-05

Covariance and correlation of q1 and q2= -4.08521737E-06 -1.22040336E-03

F1 and F2= 0.995287418 6.71427995E-02

Freqs. of fixation of A1 for In and St= 0.798180759 0.986595213

Freqs. of fixation of A2 for In and St= 0.196301267 0.00000000

Freqs. of segregation for In and St= 5.51797450E-03 1.34047866E-02

q1f= 0.197390452 q2f= 0.00000000

Index of gamma value 98 gamma for St popn= 18.0512981

Means of q1 and q2= 0.195161819 1.04379223E-03

Variances of q1 and q2= 0.156327426 6.92747490E-05

Covariance and correlation of q1 and q2= -3.98817065E-06 -1.21190527E-03

F1 and F2= 0.995249033 6.64376840E-02

Freqs. of fixation of A1 for In and St= 0.801104665 0.986710906

Freqs. of fixation of A2 for In and St= 0.193388700 0.00000000

Freqs. of segregation for In and St= 5.50663471E-03 1.32890940E-02

q1f= 0.194459513 q2f= 0.00000000

Index of gamma value 99 gamma for St popn= 18.2348156

Means of q1 and q2= 0.192269102 1.03116594E-03

Variances of q1 and q2= 0.154557794 6.77277058E-05

Covariance and correlation of q1 and q2= -3.89358320E-06 -1.20343000E-03

F1 and F2= 0.995209992 6.57485053E-02

Freqs. of fixation of A1 for In and St= 0.803995669 0.986824870

Freqs. of fixation of A2 for In and St= 0.190509006 0.00000000

Freqs. of segregation for In and St= 5.49532473E-03 1.31751299E-02

q1f= 0.191561699 q2f= 0.00000000

Index of gamma value 100 gamma for St popn= 18.4183350

Means of q1 and q2= 0.189409107 1.01880962E-03

Variances of q1 and q2= 0.152791753 6.62309758E-05

Covariance and correlation of q1 and q2= -3.80151323E-06 -1.19502214E-03

F1 and F2= 0.995170176 6.50744960E-02

Freqs. of fixation of A1 for In and St= 0.806854010 0.986936748

Freqs. of fixation of A2 for In and St= 0.187661976 0.00000000

Freqs. of segregation for In and St= 5.48401475E-03 1.30632520E-02

q1f= 0.188696787 q2f= 0.00000000

Index of gamma value 101 gamma for St popn= 18.6018524

Means of q1 and q2= 0.186581910 1.00671430E-03

Variances of q1 and q2= 0.151029944 6.47824636E-05

Covariance and correlation of q1 and q2= -3.71172791E-06 -1.18663150E-03

F1 and F2= 0.995129704 6.44152462E-02

Freqs. of fixation of A1 for In and St= 0.809679985 0.987047613

Freqs. of fixation of A2 for In and St= 0.184847713 0.00000000

Freqs. of segregation for In and St= 5.47230244E-03 1.29523873E-02

q1f= 0.185864821 q2f= 0.00000000

Index of gamma value 102 gamma for St popn= 18.7853718

Means of q1 and q2= 0.183788195 9.94870905E-04

Variances of q1 and q2= 0.149273351 6.33800228E-05

Covariance and correlation of q1 and q2= -3.62399442E-06 -1.17820315E-03

F1 and F2= 0.995088696 6.37702271E-02

Freqs. of fixation of A1 for In and St= 0.812472403 0.987156391

Freqs. of fixation of A2 for In and St= 0.182066858 0.00000000

Freqs. of segregation for In and St= 5.46073914E-03 1.28436089E-02

q1f= 0.183066532 q2f= 0.00000000

Index of gamma value 103 gamma for St popn= 18.9688892

Means of q1 and q2= 0.181026176 9.83273843E-04

Variances of q1 and q2= 0.147521347 6.20217324E-05

Covariance and correlation of q1 and q2= -3.53854557E-06 -1.16983720E-03

F1 and F2= 0.995046675 6.31388500E-02

Freqs. of fixation of A1 for In and St= 0.815233350 0.987263381

Freqs. of fixation of A2 for In and St= 0.179317653 0.00000000

Freqs. of segregation for In and St= 5.44899702E-03 1.27366185E-02

q1f= 0.180300102 q2f= 0.00000000

Index of gamma value 104 gamma for St popn= 19.1524086

Means of q1 and q2= 0.178296968 9.71913803E-04

Variances of q1 and q2= 0.145775259 6.07056718E-05

Covariance and correlation of q1 and q2= -3.45506123E-06 -1.16144645E-03

F1 and F2= 0.995004356 6.25206977E-02

Freqs. of fixation of A1 for In and St= 0.817961693 0.987369061

Freqs. of fixation of A2 for In and St= 0.176601186 0.00000000

Freqs. of segregation for In and St= 5.43712080E-03 1.26309395E-02

q1f= 0.177566648 q2f= 0.00000000

Index of gamma value 105 gamma for St popn= 19.3359261

Means of q1 and q2= 0.175600141 9.60784149E-04

Variances of q1 and q2= 0.144035235 5.94300727E-05

Covariance and correlation of q1 and q2= -3.37383244E-06 -1.15315057E-03

F1 and F2= 0.994960904 6.19152896E-02

Freqs. of fixation of A1 for In and St= 0.820657790 0.987473249

Freqs. of fixation of A2 for In and St= 0.173917025 0.00000000

Freqs. of segregation for In and St= 5.42518497E-03 1.25267506E-02

q1f= 0.174865708 q2f= 0.00000000

Index of gamma value 106 gamma for St popn= 19.5194454

Means of q1 and q2= 0.172935873 9.49877372E-04

Variances of q1 and q2= 0.142302036 5.81932327E-05

Covariance and correlation of q1 and q2= -3.29440809E-06 -1.14481570E-03

F1 and F2= 0.994916916 6.13221899E-02

Freqs. of fixation of A1 for In and St= 0.823321283 0.987575591

Freqs. of fixation of A2 for In and St= 0.171265379 0.00000000

Freqs. of segregation for In and St= 5.41333854E-03 1.24244094E-02

q1f= 0.172197551 q2f= 0.00000000

Index of gamma value 107 gamma for St popn= 19.7029648

Means of q1 and q2= 0.170303643 9.39186895E-04

Variances of q1 and q2= 0.140575707 5.69936601E-05

Covariance and correlation of q1 and q2= -3.21693369E-06 -1.13651110E-03

F1 and F2= 0.994871914 6.07410930E-02

Freqs. of fixation of A1 for In and St= 0.825953066 0.987676859

Freqs. of fixation of A2 for In and St= 0.168645665 0.00000000

Freqs. of segregation for In and St= 5.40126860E-03 1.23231411E-02

q1f= 0.169561505 q2f= 0.00000000

Index of gamma value 108 gamma for St popn= 19.8864822

Means of q1 and q2= 0.167703852 9.28707595E-04

Variances of q1 and q2= 0.138857126 5.58297834E-05

Covariance and correlation of q1 and q2= -3.14135104E-06 -1.12823455E-03

F1 and F2= 0.994826317 6.01714477E-02

Freqs. of fixation of A1 for In and St= 0.828552604 0.987776577

Freqs. of fixation of A2 for In and St= 0.166058376 0.00000000

Freqs. of segregation for In and St= 5.38901985E-03 1.22234225E-02

q1f= 0.166958123 q2f= 0.00000000

Index of gamma value 109 gamma for St popn= 20.0700016

Means of q1 and q2= 0.165135294 9.18432372E-04

Variances of q1 and q2= 0.137145981 5.47002273E-05

Covariance and correlation of q1 and q2= -3.06764559E-06 -1.12000271E-03

F1 and F2= 0.994780064 5.96130043E-02

Freqs. of fixation of A1 for In and St= 0.831121027 0.987874746

Freqs. of fixation of A2 for In and St= 0.163502216 0.00000000

Freqs. of segregation for In and St= 5.37675619E-03 1.21252537E-02

q1f= 0.164386079 q2f= 0.00000000

Index of gamma value 110 gamma for St popn= 20.2535191

Means of q1 and q2= 0.162598744 9.08353890E-04

Variances of q1 and q2= 0.135443226 5.36030930E-05

Covariance and correlation of q1 and q2= -2.99564272E-06 -1.11177389E-03

F1 and F2= 0.994732976 5.90648986E-02

Freqs. of fixation of A1 for In and St= 0.833657503 0.987971723

Freqs. of fixation of A2 for In and St= 0.160978019 0.00000000

Freqs. of segregation for In and St= 5.36447763E-03 1.20282769E-02

q1f= 0.161846235 q2f= 0.00000000

Index of gamma value 111 gamma for St popn= 20.4370384

Means of q1 and q2= 0.160094142 8.98469880E-04

Variances of q1 and q2= 0.133749336 5.25382529E-05

Covariance and correlation of q1 and q2= -2.92554614E-06 -1.10362959E-03

F1 and F2= 0.994684994 5.85278384E-02

Freqs. of fixation of A1 for In and St= 0.836162269 0.988067269

Freqs. of fixation of A2 for In and St= 0.158485651 0.00000000

Freqs. of segregation for In and St= 5.35207987E-03 1.19327307E-02

q1f= 0.159338444 q2f= 0.00000000

Index of gamma value 112 gamma for St popn= 20.6205559

Means of q1 and q2= 0.157621309 8.88772076E-04

Variances of q1 and q2= 0.132064641 5.15039137E-05

Covariance and correlation of q1 and q2= -2.85689021E-06 -1.09541882E-03

F1 and F2= 0.994636238 5.80010712E-02

Freqs. of fixation of A1 for In and St= 0.838635206 0.988161206

Freqs. of fixation of A2 for In and St= 0.156025007 0.00000000

Freqs. of segregation for In and St= 5.33978641E-03 1.18387938E-02

q1f= 0.156862617 q2f= 0.00000000

Index of gamma value 113 gamma for St popn= 20.8040752

Means of q1 and q2= 0.155178472 8.79256520E-04

Variances of q1 and q2= 0.130388439 5.04988857E-05

Covariance and correlation of q1 and q2= -2.78982043E-06 -1.08721596E-03

F1 and F2= 0.994586766 5.74841611E-02

Freqs. of fixation of A1 for In and St= 0.841078401 0.988254368

Freqs. of fixation of A2 for In and St= 0.153594300 0.00000000

Freqs. of segregation for In and St= 5.32729924E-03 1.17456317E-02

q1f= 0.154416919 q2f= 0.00000000

Index of gamma value 114 gamma for St popn= 20.9875946

Means of q1 and q2= 0.152767554 8.69918207E-04

Variances of q1 and q2= 0.128722459 4.95220411E-05

Covariance and correlation of q1 and q2= -2.72455509E-06 -1.07911858E-03

F1 and F2= 0.994536281 5.69768026E-02

Freqs. of fixation of A1 for In and St= 0.843489766 0.988345742

Freqs. of fixation of A2 for In and St= 0.151195422 0.00000000

Freqs. of segregation for In and St= 5.31481206E-03 1.16542578E-02

q1f= 0.152003288 q2f= 0.00000000

Index of gamma value 115 gamma for St popn= 21.1711121

Means of q1 and q2= 0.150387317 8.60751548E-04

Variances of q1 and q2= 0.127066344 4.85724231E-05

Covariance and correlation of q1 and q2= -2.66068673E-06 -1.07098557E-03

F1 and F2= 0.994485199 5.64788617E-02

Freqs. of fixation of A1 for In and St= 0.845870733 0.988436401

Freqs. of fixation of A2 for In and St= 0.148827165 0.00000000

Freqs. of segregation for In and St= 5.30210137E-03 1.15635991E-02

q1f= 0.149620458 q2f= 0.00000000

Index of gamma value 116 gamma for St popn= 21.3546314

Means of q1 and q2= 0.148038015 8.51751887E-04

Variances of q1 and q2= 0.125420660 4.76488785E-05

Covariance and correlation of q1 and q2= -2.59834633E-06 -1.06288469E-03

F1 and F2= 0.994433224 5.59898950E-02

Freqs. of fixation of A1 for In and St= 0.848220825 0.988525510

Freqs. of fixation of A2 for In and St= 0.146489799 0.00000000

Freqs. of segregation for In and St= 5.28937578E-03 1.14744902E-02

q1f= 0.147268757 q2f= 0.00000000

Index of gamma value 117 gamma for St popn= 21.5381489

Means of q1 and q2= 0.145719111 8.42915266E-04

Variances of q1 and q2= 0.123785459 4.67505961E-05

Covariance and correlation of q1 and q2= -2.53754843E-06 -1.05483842E-03

F1 and F2= 0.994380116 5.55097759E-02

Freqs. of fixation of A1 for In and St= 0.850540698 0.988613546

Freqs. of fixation of A2 for In and St= 0.144182667 0.00000000

Freqs. of segregation for In and St= 5.27663529E-03 1.13864541E-02

q1f= 0.144947499 q2f= 0.00000000

Index of gamma value 118 gamma for St popn= 21.7216682

Means of q1 and q2= 0.143430188 8.34237610E-04

Variances of q1 and q2= 0.122160941 4.58766335E-05

Covariance and correlation of q1 and q2= -2.47814751E-06 -1.04680355E-03

F1 and F2= 0.994326532 5.50381988E-02

Freqs. of fixation of A1 for In and St= 0.852830529 0.988700390

Freqs. of fixation of A2 for In and St= 0.141905501 0.00000000

Freqs. of segregation for In and St= 5.26396930E-03 1.12996101E-02

q1f= 0.142656446 q2f= 0.00000000

Index of gamma value 119 gamma for St popn= 21.9051857

Means of q1 and q2= 0.141171694 8.25713447E-04

Variances of q1 and q2= 0.120547749 4.50261286E-05

Covariance and correlation of q1 and q2= -2.42011447E-06 -1.03878113E-03

F1 and F2= 0.994271815 5.45750298E-02

Freqs. of fixation of A1 for In and St= 0.855090141 0.988786101

Freqs. of fixation of A2 for In and St= 0.139658660 0.00000000

Freqs. of segregation for In and St= 5.25119901E-03 1.12138987E-02

q1f= 0.140395910 q2f= 0.00000000

Index of gamma value 120 gamma for St popn= 22.0887051

Means of q1 and q2= 0.138943270 8.17339751E-04

Variances of q1 and q2= 0.118946090 4.41981610E-05

Covariance and correlation of q1 and q2= -2.36347114E-06 -1.03079691E-03

F1 and F2= 0.994216323 5.41198626E-02

Freqs. of fixation of A1 for In and St= 0.857319832 0.988870561

Freqs. of fixation of A2 for In and St= 0.137441829 0.00000000

Freqs. of segregation for In and St= 5.23833930E-03 1.11294389E-02

q1f= 0.138165593 q2f= 0.00000000

Index of gamma value 121 gamma for St popn= 22.2722244

Means of q1 and q2= 0.136744350 8.09112040E-04

Variances of q1 and q2= 0.117355943 4.33918176E-05

Covariance and correlation of q1 and q2= -2.30813748E-06 -1.02283410E-03

F1 and F2= 0.994159997 5.36723621E-02

Freqs. of fixation of A1 for In and St= 0.859520197 0.988954186

Freqs. of fixation of A2 for In and St= 0.135254472 0.00000000

Freqs. of segregation for In and St= 5.22533059E-03 1.10458136E-02

q1f= 0.135964930 q2f= 0.00000000

Index of gamma value 122 gamma for St popn= 22.4557419

Means of q1 and q2= 0.134575143 8.01027403E-04

Variances of q1 and q2= 0.115777835 4.26068000E-05

Covariance and correlation of q1 and q2= -2.25414260E-06 -1.01491390E-03

F1 and F2= 0.994102597 5.32328300E-02

Freqs. of fixation of A1 for In and St= 0.861690879 0.989036500

Freqs. of fixation of A2 for In and St= 0.133096665 0.00000000

Freqs. of segregation for In and St= 5.21245599E-03 1.09634995E-02

q1f= 0.133794054 q2f= 0.00000000

Index of gamma value 123 gamma for St popn= 22.6392612

Means of q1 and q2= 0.132434875 7.93081592E-04

Variances of q1 and q2= 0.114211574 4.18421878E-05

Covariance and correlation of q1 and q2= -2.20148650E-06 -1.00705633E-03

F1 and F2= 0.994044125 5.28008714E-02

Freqs. of fixation of A1 for In and St= 0.863832593 0.989117682

Freqs. of fixation of A2 for In and St= 0.130967736 0.00000000

Freqs. of segregation for In and St= 5.19967079E-03 1.08823180E-02

q1f= 0.131652281 q2f= 0.00000000

Index of gamma value 124 gamma for St popn= 22.8227787

Means of q1 and q2= 0.130323872 7.85270997E-04

Variances of q1 and q2= 0.112657830 4.10971807E-05

Covariance and correlation of q1 and q2= -2.14991451E-06 -9.99158714E-04

F1 and F2= 0.993985057 5.23761585E-02

Freqs. of fixation of A1 for In and St= 0.865945518 0.989198029

Freqs. of fixation of A2 for In and St= 0.128868043 0.00000000

Freqs. of segregation for In and St= 5.18643856E-03 1.08019710E-02

q1f= 0.129539892 q2f= 0.00000000

Index of gamma value 125 gamma for St popn= 23.0062981

Means of q1 and q2= 0.128241360 7.77592650E-04

Variances of q1 and q2= 0.111116335 4.03711856E-05

Covariance and correlation of q1 and q2= -2.09966674E-06 -9.91347013E-04

F1 and F2= 0.993924797 5.19585721E-02

Freqs. of fixation of A1 for In and St= 0.868030012 0.989277422

Freqs. of fixation of A2 for In and St= 0.126796737 0.00000000

Freqs. of segregation for In and St= 5.17325103E-03 1.07225776E-02

q1f= 0.127456099 q2f= 0.00000000

Index of gamma value 126 gamma for St popn= 23.1898155

Means of q1 and q2= 0.126187667 7.70042767E-04

Variances of q1 and q2= 0.109587736 3.96635260E-05

Covariance and correlation of q1 and q2= -2.05046672E-06 -9.83503880E-04

F1 and F2= 0.993863821 5.15479073E-02

Freqs. of fixation of A1 for In and St= 0.870085597 0.989355683

Freqs. of fixation of A2 for In and St= 0.124754168 0.00000000

Freqs. of segregation for In and St= 5.16023487E-03 1.06443167E-02

q1f= 0.125401258 q2f= 0.00000000

Index of gamma value 127 gamma for St popn= 23.3733349

Means of q1 and q2= 0.124162495 7.62618205E-04

Variances of q1 and q2= 0.108072117 3.89735978E-05

Covariance and correlation of q1 and q2= -2.00257637E-06 -9.75769013E-04

F1 and F2= 0.993801594 5.11439964E-02

Freqs. of fixation of A1 for In and St= 0.872112930 0.989433110

Freqs. of fixation of A2 for In and St= 0.122740053 0.00000000

Freqs. of segregation for In and St= 5.14701754E-03 1.05668902E-02

q1f= 0.123375066 q2f= 0.00000000

Index of gamma value 128 gamma for St popn= 23.5568523

Means of q1 and q2= 0.122165106 7.55317102E-04

Variances of q1 and q2= 0.106569313 3.83008592E-05

Covariance and correlation of q1 and q2= -1.95578468E-06 -9.68056498E-04

F1 and F2= 0.993738532 5.07466458E-02

Freqs. of fixation of A1 for In and St= 0.874112308 0.989509404

Freqs. of fixation of A2 for In and St= 0.120753549 0.00000000

Freqs. of segregation for In and St= 5.13414294E-03 1.04905963E-02

q1f= 0.121376716 q2f= 0.00000000

Index of gamma value 129 gamma for St popn= 23.7403717

Means of q1 and q2= 0.120195210 7.48135091E-04

Variances of q1 and q2= 0.105079398 3.76446878E-05

Covariance and correlation of q1 and q2= -1.91005529E-06 -9.60362726E-04

F1 and F2= 0.993674397 5.03557101E-02

Freqs. of fixation of A1 for In and St= 0.876084685 0.989584923

Freqs. of fixation of A2 for In and St= 0.118794568 0.00000000

Freqs. of segregation for In and St= 5.12074679E-03 1.04150772E-02

q1f= 0.119406015 q2f= 0.00000000

Index of gamma value 130 gamma for St popn= 23.9238911

Means of q1 and q2= 0.118253626 7.41069438E-04

Variances of q1 and q2= 0.103603348 3.70046146E-05

Covariance and correlation of q1 and q2= -1.86535908E-06 -9.52681177E-04

F1 and F2= 0.993609309 4.99711037E-02

Freqs. of fixation of A1 for In and St= 0.878028512 0.989659131

Freqs. of fixation of A2 for In and St= 0.116863780 0.00000000

Freqs. of segregation for In and St= 5.10770828E-03 1.03408694E-02

q1f= 0.117463753 q2f= 0.00000000

Index of gamma value 131 gamma for St popn= 24.1074085

Means of q1 and q2= 0.116339102 7.34117639E-04

Variances of q1 and q2= 0.102140516 3.63800609E-05

Covariance and correlation of q1 and q2= -1.82174699E-06 -9.45055508E-04

F1 and F2= 0.993543029 4.95925769E-02

Freqs. of fixation of A1 for In and St= 0.879945576 0.989732802

Freqs. of fixation of A2 for In and St= 0.114959955 0.00000000

Freqs. of segregation for In and St= 5.09446859E-03 1.02671981E-02

q1f= 0.115548611 q2f= 0.00000000

Index of gamma value 132 gamma for St popn= 24.2909279

Means of q1 and q2= 0.114451826 7.27277133E-04

Variances of q1 and q2= 0.100691371 3.57705685E-05

Covariance and correlation of q1 and q2= -1.77910988E-06 -9.37440491E-04

F1 and F2= 0.993475914 4.92200293E-02

Freqs. of fixation of A1 for In and St= 0.881835401 0.989805579

Freqs. of fixation of A2 for In and St= 0.113083303 0.00000000

Freqs. of segregation for In and St= 5.08129597E-03 1.01944208E-02

q1f= 0.113660850 q2f= 0.00000000

Index of gamma value 133 gamma for St popn= 24.4744453

Means of q1 and q2= 0.112591058 7.20545300E-04

Variances of q1 and q2= 9.92556363E-02 3.51756462E-05

Covariance and correlation of q1 and q2= -1.73749140E-06 -9.29873844E-04

F1 and F2= 0.993407607 4.88532931E-02

Freqs. of fixation of A1 for In and St= 0.883698881 0.989877403

Freqs. of fixation of A2 for In and St= 0.111233115 0.00000000

Freqs. of segregation for In and St= 5.06800413E-03 1.01225972E-02

q1f= 0.111799717 q2f= 0.00000000

Index of gamma value 134 gamma for St popn= 24.6579647

Means of q1 and q2= 0.110757060 7.13918998E-04

Variances of q1 and q2= 9.78338197E-02 3.45948647E-05

Covariance and correlation of q1 and q2= -1.69681880E-06 -9.22327512E-04

F1 and F2= 0.993338287 4.84923124E-02

Freqs. of fixation of A1 for In and St= 0.885535657 0.989948332

Freqs. of fixation of A2 for In and St= 0.109409593 0.00000000

Freqs. of segregation for In and St= 5.05474955E-03 1.00516677E-02

q1f= 0.109965444 q2f= 0.00000000

Index of gamma value 135 gamma for St popn= 24.8414822

Means of q1 and q2= 0.108949587 7.07396772E-04

Variances of q1 and q2= 9.64260176E-02 3.40277584E-05

Covariance and correlation of q1 and q2= -1.65717938E-06 -9.14862321E-04

F1 and F2= 0.993267834 4.81368415E-02

Freqs. of fixation of A1 for In and St= 0.887345850 0.990018427

Freqs. of fixation of A2 for In and St= 0.107612476 0.00000000

Freqs. of segregation for In and St= 5.04167378E-03 9.98157263E-03

q1f= 0.108157769 q2f= 0.00000000

Index of gamma value 136 gamma for St popn= 25.0250015

Means of q1 and q2= 0.107167840 7.00975768E-04

Variances of q1 and q2= 9.50319022E-02 3.34738725E-05

Covariance and correlation of q1 and q2= -1.61842036E-06 -9.07410227E-04

F1 and F2= 0.993196309 4.77867462E-02

Freqs. of fixation of A1 for In and St= 0.889130592 0.990087748

Freqs. of fixation of A2 for In and St= 0.105841070 0.00000000

Freqs. of segregation for In and St= 5.02833724E-03 9.91225243E-03

q1f= 0.106375970 q2f= 0.00000000

Index of gamma value 137 gamma for St popn= 25.2085190

Means of q1 and q2= 0.105412059 6.94653310E-04

Variances of q1 and q2= 9.36519206E-02 3.29329014E-05

Covariance and correlation of q1 and q2= -1.58057082E-06 -8.99996085E-04

F1 and F2= 0.993123770 4.74420749E-02

Freqs. of fixation of A1 for In and St= 0.890889466 0.990156412

Freqs. of fixation of A2 for In and St= 0.104095511 0.00000000

Freqs. of segregation for In and St= 5.01502305E-03 9.84358788E-03

q1f= 0.104620181 q2f= 0.00000000

Index of gamma value 138 gamma for St popn= 25.3920383

Means of q1 and q2= 0.103681996 6.88427535E-04

Variances of q1 and q2= 9.22861472E-02 3.24043285E-05

Covariance and correlation of q1 and q2= -1.54360896E-06 -8.92621872E-04

F1 and F2= 0.993049860 4.71024923E-02

Freqs. of fixation of A1 for In and St= 0.892622650 0.990223825

Freqs. of fixation of A2 for In and St= 0.102375567 0.00000000

Freqs. of segregation for In and St= 5.00178337E-03 9.77617502E-03

q1f= 0.102890201 q2f= 0.00000000

Index of gamma value 139 gamma for St popn= 25.5755577

Means of q1 and q2= 0.101976864 6.82297105E-04

Variances of q1 and q2= 9.09342468E-02 3.18879211E-05

Covariance and correlation of q1 and q2= -1.50750566E-06 -8.85282992E-04

F1 and F2= 0.992974997 4.67680320E-02

Freqs. of fixation of A1 for In and St= 0.894331038 0.990290940

Freqs. of fixation of A2 for In and St= 0.100680515 0.00000000

Freqs. of segregation for In and St= 4.98844683E-03 9.70906019E-03

q1f= 0.101185270 q2f= 0.00000000

Index of gamma value 140 gamma for St popn= 25.7590752

Means of q1 and q2= 0.100296907 6.76258700E-04

Variances of q1 and q2= 8.95966515E-02 3.13831952E-05

Covariance and correlation of q1 and q2= -1.47227547E-06 -8.78000224E-04

F1 and F2= 0.992898881 4.64384891E-02

Freqs. of fixation of A1 for In and St= 0.896014333 0.990357041

Freqs. of fixation of A2 for In and St= 9.90105420E-02 0.00000000

Freqs. of segregation for In and St= 4.97512519E-03 9.64295864E-03

q1f= 9.95055959E-02 q2f= 0.00000000

Index of gamma value 141 gamma for St popn= 25.9425945

Means of q1 and q2= 9.86418426E-02 6.70310052E-04

Variances of q1 and q2= 8.82733762E-02 3.08898489E-05

Covariance and correlation of q1 and q2= -1.43778743E-06 -8.70706746E-04

F1 and F2= 0.992821455 4.61138375E-02

Freqs. of fixation of A1 for In and St= 0.897672832 0.990422308

Freqs. of fixation of A2 for In and St= 9.73653495E-02 0.00000000

Freqs. of segregation for In and St= 4.96181846E-03 9.57769156E-03

q1f= 9.78508741E-02 q2f= 0.00000000

Index of gamma value 142 gamma for St popn= 26.1261120

Means of q1 and q2= 9.70109552E-02 6.64450577E-04

Variances of q1 and q2= 8.69641155E-02 3.04075475E-05

Covariance and correlation of q1 and q2= -1.40417251E-06 -8.63494817E-04

F1 and F2= 0.992742956 4.57938723E-02

Freqs. of fixation of A1 for In and St= 0.899307072 0.990487039

Freqs. of fixation of A2 for In and St= 9.57443044E-02 0.00000000

Freqs. of segregation for In and St= 4.94862348E-03 9.51296091E-03

q1f= 9.62204635E-02 q2f= 0.00000000

Index of gamma value 143 gamma for St popn= 26.3096313

Means of q1 and q2= 9.54048932E-02 6.58677018E-04

Variances of q1 and q2= 8.56696293E-02 2.99359690E-05

Covariance and correlation of q1 and q2= -1.37129246E-06 -8.56288243E-04

F1 and F2= 0.992663383 4.54785898E-02

Freqs. of fixation of A1 for In and St= 0.900916696 0.990550816

Freqs. of fixation of A2 for In and St= 9.41479802E-02 0.00000000

Freqs. of segregation for In and St= 4.93532419E-03 9.44918394E-03

q1f= 9.46149379E-02 q2f= 0.00000000

Index of gamma value 144 gamma for St popn= 26.4931488

Means of q1 and q2= 9.38219726E-02 6.52988267E-04

Variances of q1 and q2= 8.43887702E-02 2.94748061E-05

Covariance and correlation of q1 and q2= -1.33922367E-06 -8.49151693E-04

F1 and F2= 0.992582381 4.51678336E-02

Freqs. of fixation of A1 for In and St= 0.902503371 0.990613878

Freqs. of fixation of A2 for In and St= 9.25746933E-02 0.00000000

Freqs. of segregation for In and St= 4.92193550E-03 9.38612223E-03

q1f= 9.30325910E-02 q2f= 0.00000000

Index of gamma value 145 gamma for St popn= 26.6766682

Means of q1 and q2= 9.22633559E-02 6.47382287E-04

Variances of q1 and q2= 8.31227154E-02 2.90237276E-05

Covariance and correlation of q1 and q2= -1.30789340E-06 -8.42046167E-04

F1 and F2= 0.992500186 4.48614806E-02

Freqs. of fixation of A1 for In and St= 0.904065609 0.990676403

Freqs. of fixation of A2 for In and St= 9.10256654E-02 0.00000000

Freqs. of segregation for In and St= 4.90872562E-03 9.32359695E-03

q1f= 9.14746895E-02 q2f= 0.00000000

Index of gamma value 146 gamma for St popn= 26.8601875

Means of q1 and q2= 9.07278284E-02 6.41857448E-04

Variances of q1 and q2= 8.18706900E-02 2.85824844E-05

Covariance and correlation of q1 and q2= -1.27731255E-06 -8.34993203E-04

F1 and F2= 0.992416620 4.45594937E-02

Freqs. of fixation of A1 for In and St= 0.905604839 0.990738213

Freqs. of fixation of A2 for In and St= 8.94996226E-02 0.00000000

Freqs. of segregation for In and St= 4.89553809E-03 9.26178694E-03

q1f= 8.99399295E-02 q2f= 0.00000000

Index of gamma value 147 gamma for St popn= 27.0437050

Means of q1 and q2= 8.92156065E-02 6.36412180E-04

Variances of q1 and q2= 8.06330964E-02 2.81507891E-05

Covariance and correlation of q1 and q2= -1.24746293E-06 -8.27991171E-04

F1 and F2= 0.992331862 4.42617498E-02

Freqs. of fixation of A1 for In and St= 0.907120943 0.990799189

Freqs. of fixation of A2 for In and St= 8.79968032E-02 0.00000000

Freqs. of segregation for In and St= 4.88225371E-03 9.20081139E-03

q1f= 8.84285346E-02 q2f= 0.00000000

Index of gamma value 148 gamma for St popn= 27.2272243

Means of q1 and q2= 8.77263919E-02 6.31043920E-04

Variances of q1 and q2= 7.94098899E-02 2.77283343E-05

Covariance and correlation of q1 and q2= -1.21824269E-06 -8.20983900E-04

F1 and F2= 0.992245674 4.39681634E-02

Freqs. of fixation of A1 for In and St= 0.908614218 0.990859866

Freqs. of fixation of A2 for In and St= 8.65169093E-02 0.00000000

Freqs. of segregation for In and St= 4.86887246E-03 9.14013386E-03

q1f= 8.69402066E-02 q2f= 0.00000000

Index of gamma value 149 gamma for St popn= 27.4107418

Means of q1 and q2= 8.62594470E-02 6.25751971E-04

Variances of q1 and q2= 7.82006830E-02 2.73149217E-05

Covariance and correlation of q1 and q2= -1.18977550E-06 -8.14066269E-04

F1 and F2= 0.992158353 4.36786860E-02

Freqs. of fixation of A1 for In and St= 0.910084963 0.990919471

Freqs. of fixation of A2 for In and St= 8.50592256E-02 0.00000000

Freqs. of segregation for In and St= 4.85581160E-03 9.08052921E-03

q1f= 8.54742751E-02 q2f= 0.00000000

Index of gamma value 150 gamma for St popn= 27.5942612

Means of q1 and q2= 8.48154649E-02 6.20534818E-04

Variances of q1 and q2= 7.70062208E-02 2.69102566E-05

Covariance and correlation of q1 and q2= -1.16194497E-06 -8.07167613E-04

F1 and F2= 0.992069483 4.33931574E-02

Freqs. of fixation of A1 for In and St= 0.911532998 0.990978360

Freqs. of fixation of A2 for In and St= 8.36243927E-02 0.00000000

Freqs. of segregation for In and St= 4.84260917E-03 9.02163982E-03

q1f= 8.40313286E-02 q2f= 0.00000000

Index of gamma value 151 gamma for St popn= 27.7777786

Means of q1 and q2= 8.33932534E-02 6.15390134E-04

Variances of q1 and q2= 7.58257359E-02 2.65140916E-05

Covariance and correlation of q1 and q2= -1.13475835E-06 -8.00307142E-04

F1 and F2= 0.991979480 4.31115441E-02

Freqs. of fixation of A1 for In and St= 0.912959397 0.991036892

Freqs. of fixation of A2 for In and St= 8.22112858E-02 0.00000000

Freqs. of segregation for In and St= 4.82931733E-03 8.96310806E-03

q1f= 8.26102346E-02 q2f= 0.00000000

**Inversion frequency= 0.5**

**h=0.05**

Index of gamma value 7 gamma for St popn= 10.2399998

Means of q1 and q2= 9.37359501E-03 9.37359501E-03

Variances of q1 and q2= 6.28458243E-03 6.28458243E-03

Covariance and correlation of q1 and q2= -6.79850273E-05 -1.08177476E-02

F1 and F2= 0.676800013 0.676800013

Freqs. of fixation of A1 for In and St= 0.970745504 0.970745504

Freqs. of fixation of A2 for In and St= 5.09208255E-03 5.09208255E-03

Freqs. of segregation for In and St= 2.41624136E-02 2.41624136E-02

q1f= 5.21816593E-03 q2f= 5.21816593E-03

Index of gamma value 8 gamma for St popn= 11.9049997

Means of q1 and q2= 5.74873528E-03 5.74873481E-03

Variances of q1 and q2= 2.99672876E-03 2.99672876E-03

Covariance and correlation of q1 and q2= -2.17484157E-05 -7.25738565E-03

F1 and F2= 0.524298906 0.524298966

Freqs. of fixation of A1 for In and St= 0.975307941 0.975307941

Freqs. of fixation of A2 for In and St= 2.04888755E-03 2.04888755E-03

Freqs. of segregation for In and St= 2.26431713E-02 2.26431713E-02

q1f= 2.09635566E-03 q2f= 2.09635566E-03

Index of gamma value 9 gamma for St popn= 13.5699997

Means of q1 and q2= 4.08238405E-03 4.08238405E-03

Variances of q1 and q2= 1.58396410E-03 1.58396410E-03

Covariance and correlation of q1 and q2= -9.07601861E-06 -5.72993979E-03

F1 and F2= 0.389590234 0.389590234

Freqs. of fixation of A1 for In and St= 0.977855027 0.977855027

Freqs. of fixation of A2 for In and St= 8.21227441E-04 8.21227499E-04

Freqs. of segregation for In and St= 2.13237461E-02 2.13237461E-02

q1f= 8.39120650E-04 q2f= 8.39120708E-04

Index of gamma value 10 gamma for St popn= 15.2349997

Means of q1 and q2= 3.24805803E-03 3.24805803E-03

Variances of q1 and q2= 9.56010947E-04 9.56010947E-04

Covariance and correlation of q1 and q2= -4.84217298E-06 -5.06497640E-03

F1 and F2= 0.295292199 0.295292199

Freqs. of fixation of A1 for In and St= 0.979498386 0.979498386

Freqs. of fixation of A2 for In and St= 3.28665803E-04 3.28665803E-04

Freqs. of segregation for In and St= 2.01729480E-02 2.01729480E-02

q1f= 3.35432473E-04 q2f= 3.35432473E-04

Index of gamma value 11 gamma for St popn= 16.8999996

Means of q1 and q2= 2.77659297E-03 2.77659297E-03

Variances of q1 and q2= 6.58177538E-04 6.58177596E-04

Covariance and correlation of q1 and q2= -3.12215116E-06 -4.74363053E-03

F1 and F2= 0.237705037 0.237705052

Freqs. of fixation of A1 for In and St= 0.980710268 0.980710268

Freqs. of fixation of A2 for In and St= 1.31465262E-04 1.31465262E-04

Freqs. of segregation for In and St= 1.91582665E-02 1.91582665E-02

q1f= 1.34033107E-04 q2f= 1.34033107E-04

Index of gamma value 12 gamma for St popn= 18.5649986

Means of q1 and q2= 2.47201486E-03 2.47201486E-03

Variances of q1 and q2= 5.02538809E-04 5.02538867E-04

Covariance and correlation of q1 and q2= -2.28167119E-06 -4.54028836E-03

F1 and F2= 0.203794956 0.203794986

Freqs. of fixation of A1 for In and St= 0.981692851 0.981692851

Freqs. of fixation of A2 for In and St= 5.25829091E-05 5.25829128E-05

Freqs. of segregation for In and St= 1.82545669E-02 1.82545669E-02

q1f= 5.35606341E-05 q2f= 5.35606414E-05

Index of gamma value 13 gamma for St popn= 20.2299995

Means of q1 and q2= 2.25086999E-03 2.25086999E-03

Variances of q1 and q2= 4.10941779E-04 4.10941808E-04

Covariance and correlation of q1 and q2= -1.79670837E-06 -4.37217252E-03

F1 and F2= 0.182982057 0.182982072

Freqs. of fixation of A1 for In and St= 0.982537687 0.982537687

Freqs. of fixation of A2 for In and St= 2.10349790E-05 2.10349790E-05

Freqs. of segregation for In and St= 1.74412783E-02 1.74412783E-02

q1f= 2.14083684E-05 q2f= 2.14083684E-05

Index of gamma value 14 gamma for St popn= 21.8950005

Means of q1 and q2= 2.07628380E-03 2.07628380E-03

Variances of q1 and q2= 3.50284128E-04 3.50284128E-04

Covariance and correlation of q1 and q2= -1.47700598E-06 -4.21659416E-03

F1 and F2= 0.169058248 0.169058248

Freqs. of fixation of A1 for In and St= 0.983288288 0.983288288

Freqs. of fixation of A2 for In and St= 0.00000000 0.00000000

Freqs. of segregation for In and St= 1.67117119E-02 1.67117119E-02

q1f= 0.00000000 q2f= 0.00000000

Index of gamma value 15 gamma for St popn= 23.5599995

Means of q1 and q2= 1.93094136E-03 1.93094090E-03

Variances of q1 and q2= 3.06064612E-04 3.06064641E-04

Covariance and correlation of q1 and q2= -1.24557232E-06 -4.06963844E-03

F1 and F2= 0.158812046 0.158812106

Freqs. of fixation of A1 for In and St= 0.983968019 0.983968019

Freqs. of fixation of A2 for In and St= 0.00000000 0.00000000

Freqs. of segregation for In and St= 1.60319805E-02 1.60319805E-02

q1f= 0.00000000 q2f= 0.00000000

Index of gamma value 16 gamma for St popn= 25.2249985

Means of q1 and q2= 1.80600781E-03 1.80600781E-03

Variances of q1 and q2= 2.71584548E-04 2.71584548E-04

Covariance and correlation of q1 and q2= -1.06770290E-06 -3.93138314E-03

F1 and F2= 0.150650471 0.150650471

Freqs. of fixation of A1 for In and St= 0.984590590 0.984590590

Freqs. of fixation of A2 for In and St= 0.00000000 0.00000000

Freqs. of segregation for In and St= 1.54094100E-02 1.54094100E-02

q1f= 0.00000000 q2f= 0.00000000

Index of gamma value 17 gamma for St popn= 26.8899994

Means of q1 and q2= 1.69647043E-03 1.69647054E-03

Variances of q1 and q2= 2.43507719E-04 2.43507675E-04

Covariance and correlation of q1 and q2= -9.25787162E-07 -3.80188040E-03

F1 and F2= 0.143781766 0.143781736

Freqs. of fixation of A1 for In and St= 0.985164821 0.985164762

Freqs. of fixation of A2 for In and St= 0.00000000 0.00000000

Freqs. of segregation for In and St= 1.48351789E-02 1.48352385E-02

q1f= 0.00000000 q2f= 0.00000000

Index of gamma value 18 gamma for St popn= 28.5550003

Means of q1 and q2= 1.59917434E-03 1.59917434E-03

Variances of q1 and q2= 2.20007161E-04 2.20007176E-04

Covariance and correlation of q1 and q2= -8.09800895E-07 -3.68079310E-03

F1 and F2= 0.137795836 0.137795836

Freqs. of fixation of A1 for In and St= 0.985697627 0.985697627

Freqs. of fixation of A2 for In and St= 0.00000000 0.00000000

Freqs. of segregation for In and St= 1.43023729E-02 1.43023729E-02

q1f= 0.00000000 q2f= 0.00000000

Index of gamma value 19 gamma for St popn= 30.2199993

Means of q1 and q2= 1.51194946E-03 1.51194935E-03

Variances of q1 and q2= 1.99978982E-04 1.99978997E-04

Covariance and correlation of q1 and q2= -7.13426516E-07 -3.56750726E-03

F1 and F2= 0.132465929 0.132465944

Freqs. of fixation of A1 for In and St= 0.986194313 0.986194372

Freqs. of fixation of A2 for In and St= 0.00000000 0.00000000

Freqs. of segregation for In and St= 1.38056874E-02 1.38056278E-02

q1f= 0.00000000 q2f= 0.00000000

Index of gamma value 20 gamma for St popn= 31.8849983

Means of q1 and q2= 1.43318879E-03 1.43318879E-03

Variances of q1 and q2= 1.82691874E-04 1.82691860E-04

Covariance and correlation of q1 and q2= -6.32352794E-07 -3.46130808E-03

F1 and F2= 0.127655253 0.127655253

Freqs. of fixation of A1 for In and St= 0.986658394 0.986658394

Freqs. of fixation of A2 for In and St= 0.00000000 0.00000000

Freqs. of segregation for In and St= 1.33416057E-02 1.33416057E-02

q1f= 0.00000000 q2f= 0.00000000

Index of gamma value 21 gamma for St popn= 33.5499992

Means of q1 and q2= 1.36165565E-03 1.36165565E-03

Variances of q1 and q2= 1.67626917E-04 1.67626931E-04

Covariance and correlation of q1 and q2= -5.63482786E-07 -3.36152920E-03

F1 and F2= 0.123273075 0.123273090

Freqs. of fixation of A1 for In and St= 0.987094164 0.987094164

Freqs. of fixation of A2 for In and St= 0.00000000 0.00000000

Freqs. of segregation for In and St= 1.29058361E-02 1.29058361E-02

q1f= 0.00000000 q2f= 0.00000000

Index of gamma value 22 gamma for St popn= 35.2150002

Means of q1 and q2= 1.29636016E-03 1.29636016E-03

Variances of q1 and q2= 1.54396475E-04 1.54396475E-04

Covariance and correlation of q1 and q2= -5.04490117E-07 -3.26749752E-03

F1 and F2= 0.119254582 0.119254582

Freqs. of fixation of A1 for In and St= 0.987503886 0.987503886

Freqs. of fixation of A2 for In and St= 0.00000000 0.00000000

Freqs. of segregation for In and St= 1.24961138E-02 1.24961138E-02

q1f= 0.00000000 q2f= 0.00000000

Index of gamma value 23 gamma for St popn= 36.8799973

Means of q1 and q2= 1.23649766E-03 1.23649766E-03

Variances of q1 and q2= 1.42700665E-04 1.42700694E-04

Covariance and correlation of q1 and q2= -4.53602411E-07 -3.17869824E-03

F1 and F2= 0.115550019 0.115550041

Freqs. of fixation of A1 for In and St= 0.987890065 0.987890065

Freqs. of fixation of A2 for In and St= 0.00000000 0.00000000

Freqs. of segregation for In and St= 1.21099353E-02 1.21099353E-02

q1f= 0.00000000 q2f= 0.00000000

Index of gamma value 24 gamma for St popn= 38.5449982

Means of q1 and q2= 1.18140120E-03 1.18140108E-03

Variances of q1 and q2= 1.32301793E-04 1.32301822E-04

Covariance and correlation of q1 and q2= -4.09422796E-07 -3.09461216E-03

F1 and F2= 0.112119645 0.112119675

Freqs. of fixation of A1 for In and St= 0.988254905 0.988254905

Freqs. of fixation of A2 for In and St= 0.00000000 0.00000000

Freqs. of segregation for In and St= 1.17450953E-02 1.17450953E-02

q1f= 0.00000000 q2f= 0.00000000

Index of gamma value 25 gamma for St popn= 40.2099991

Means of q1 and q2= 1.13051722E-03 1.13051722E-03

Variances of q1 and q2= 1.23010235E-04 1.23010250E-04

Covariance and correlation of q1 and q2= -3.70856469E-07 -3.01484228E-03

F1 and F2= 0.108931966 0.108931981

Freqs. of fixation of A1 for In and St= 0.988600373 0.988600492

Freqs. of fixation of A2 for In and St= 0.00000000 0.00000000

Freqs. of segregation for In and St= 1.13996267E-02 1.13995075E-02

q1f= 0.00000000 q2f= 0.00000000

Index of gamma value 26 gamma for St popn= 41.8750000

Means of q1 and q2= 1.08337076E-03 1.08337076E-03

Variances of q1 and q2= 1.14669085E-04 1.14669092E-04

Covariance and correlation of q1 and q2= -3.37010590E-07 -2.93898373E-03

F1 and F2= 0.105959520 0.105959527

Freqs. of fixation of A1 for In and St= 0.988928080 0.988928080

Freqs. of fixation of A2 for In and St= 0.00000000 0.00000000

Freqs. of segregation for In and St= 1.10719204E-02 1.10719204E-02

q1f= 0.00000000 q2f= 0.00000000

Index of gamma value 27 gamma for St popn= 43.5400009

Means of q1 and q2= 1.03956228E-03 1.03956228E-03

Variances of q1 and q2= 1.07149710E-04 1.07149710E-04

Covariance and correlation of q1 and q2= -3.07170524E-07 -2.86674150E-03

F1 and F2= 0.103179209 0.103179209

Freqs. of fixation of A1 for In and St= 0.989239454 0.989239454

Freqs. of fixation of A2 for In and St= 0.00000000 0.00000000

Freqs. of segregation for In and St= 1.07605457E-02 1.07605457E-02

q1f= 0.00000000 q2f= 0.00000000

Index of gamma value 28 gamma for St popn= 45.2049980

Means of q1 and q2= 9.98747302E-04 9.98747302E-04

Variances of q1 and q2= 1.00345263E-04 1.00345278E-04

Covariance and correlation of q1 and q2= -2.80749305E-07 -2.79783295E-03

F1 and F2= 0.100571565 0.100571580

Freqs. of fixation of A1 for In and St= 0.989535749 0.989535868

Freqs. of fixation of A2 for In and St= 0.00000000 0.00000000

Freqs. of segregation for In and St= 1.04642510E-02 1.04641318E-02

q1f= 0.00000000 q2f= 0.00000000

Index of gamma value 29 gamma for St popn= 46.8699989

Means of q1 and q2= 9.60629375E-04 9.60629492E-04

Variances of q1 and q2= 9.41661565E-05 9.41661638E-05

Covariance and correlation of q1 and q2= -2.57262627E-07 -2.73200707E-03

F1 and F2= 9.81197357E-02 9.81197357E-02

Freqs. of fixation of A1 for In and St= 0.989818215 0.989818275

Freqs. of fixation of A2 for In and St= 0.00000000 0.00000000

Freqs. of segregation for In and St= 1.01817846E-02 1.01817250E-02

q1f= 0.00000000 q2f= 0.00000000

Index of gamma value 30 gamma for St popn= 48.5349998

Means of q1 and q2= 9.24947613E-04 9.24947613E-04

Variances of q1 and q2= 8.85365152E-05 8.85365225E-05

Covariance and correlation of q1 and q2= -2.36305027E-07 -2.66901194E-03

F1 and F2= 9.58091915E-02 9.58091989E-02

Freqs. of fixation of A1 for In and St= 0.990087211 0.990087211

Freqs. of fixation of A2 for In and St= 0.00000000 0.00000000

Freqs. of segregation for In and St= 9.91278887E-03 9.91278887E-03

q1f= 0.00000000 q2f= 0.00000000

Index of gamma value 31 gamma for St popn= 50.1999969

Means of q1 and q2= 8.91476288E-04 8.91476288E-04

Variances of q1 and q2= 8.33919039E-05 8.33919185E-05

Covariance and correlation of q1 and q2= -2.17540673E-07 -2.60865432E-03

F1 and F2= 9.36270654E-02 9.36270803E-02

Freqs. of fixation of A1 for In and St= 0.990343988 0.990343988

Freqs. of fixation of A2 for In and St= 0.00000000 0.00000000

Freqs. of segregation for In and St= 9.65601206E-03 9.65601206E-03

q1f= 0.00000000 q2f= 0.00000000

Index of gamma value 32 gamma for St popn= 51.8649979

Means of q1 and q2= 8.60020285E-04 8.60020285E-04

Variances of q1 and q2= 7.86775490E-05 7.86775563E-05

Covariance and correlation of q1 and q2= -2.00690693E-07 -2.55080010E-03

F1 and F2= 9.15621072E-02 9.15621221E-02

Freqs. of fixation of A1 for In and St= 0.990590334 0.990590215

Freqs. of fixation of A2 for In and St= 0.00000000 0.00000000

Freqs. of segregation for In and St= 9.40966606E-03 9.40978527E-03

q1f= 0.00000000 q2f= 0.00000000

Index of gamma value 33 gamma for St popn= 53.5299988

Means of q1 and q2= 8.30401725E-04 8.30401608E-04

Variances of q1 and q2= 7.43458368E-05 7.43458368E-05

Covariance and correlation of q1 and q2= -1.85510544E-07 -2.49523786E-03

F1 and F2= 8.96043703E-02 8.96043852E-02

Freqs. of fixation of A1 for In and St= 0.990825355 0.990825355

Freqs. of fixation of A2 for In and St= 0.00000000 0.00000000

Freqs. of segregation for In and St= 9.17464495E-03 9.17464495E-03

q1f= 0.00000000 q2f= 0.00000000

Index of gamma value 34 gamma for St popn= 55.1949997

Means of q1 and q2= 8.02466355E-04 8.02466355E-04

Variances of q1 and q2= 7.03561454E-05 7.03561527E-05

Covariance and correlation of q1 and q2= -1.71797524E-07 -2.44182674E-03

F1 and F2= 8.77452940E-02 8.77453089E-02

Freqs. of fixation of A1 for In and St= 0.991051078 0.991051197

Freqs. of fixation of A2 for In and St= 0.00000000 0.00000000

Freqs. of segregation for In and St= 8.94892216E-03 8.94880295E-03

q1f= 0.00000000 q2f= 0.00000000

Index of gamma value 35 gamma for St popn= 56.8600006

Means of q1 and q2= 7.76076864E-04 7.76076806E-04

Variances of q1 and q2= 6.66728884E-05 6.66728884E-05

Covariance and correlation of q1 and q2= -1.59377834E-07 -2.39044451E-03

F1 and F2= 8.59768838E-02 8.59768912E-02

Freqs. of fixation of A1 for In and St= 0.991267204 0.991267204

Freqs. of fixation of A2 for In and St= 0.00000000 0.00000000

Freqs. of segregation for In and St= 8.73279572E-03 8.73279572E-03

q1f= 0.00000000 q2f= 0.00000000

Index of gamma value 36 gamma for St popn= 58.5249977

Means of q1 and q2= 7.51110842E-04 7.51110783E-04

Variances of q1 and q2= 6.32652227E-05 6.32652227E-05

Covariance and correlation of q1 and q2= -1.48102430E-07 -2.34097708E-03

F1 and F2= 8.42921957E-02 8.42922032E-02

Freqs. of fixation of A1 for In and St= 0.991474807 0.991474926

Freqs. of fixation of A2 for In and St= 0.00000000 0.00000000

Freqs. of segregation for In and St= 8.52519274E-03 8.52507353E-03

q1f= 0.00000000 q2f= 0.00000000

Index of gamma value 37 gamma for St popn= 60.1899986

Means of q1 and q2= 7.27458100E-04 7.27458100E-04

Variances of q1 and q2= 6.01061038E-05 6.01061001E-05

Covariance and correlation of q1 and q2= -1.37842306E-07 -2.29331641E-03

F1 and F2= 8.26849863E-02 8.26849788E-02

Freqs. of fixation of A1 for In and St= 0.991674364 0.991674364

Freqs. of fixation of A2 for In and St= 0.00000000 0.00000000

Freqs. of segregation for In and St= 8.32563639E-03 8.32563639E-03

q1f= 0.00000000 q2f= 0.00000000

Index of gamma value 38 gamma for St popn= 61.8549995

Means of q1 and q2= 7.05019978E-04 7.05020037E-04

Variances of q1 and q2= 5.71717756E-05 5.71717756E-05

Covariance and correlation of q1 and q2= -1.28485254E-07 -2.24735471E-03

F1 and F2= 8.11496302E-02 8.11496228E-02

Freqs. of fixation of A1 for In and St= 0.991865933 0.991865933

Freqs. of fixation of A2 for In and St= 0.00000000 0.00000000

Freqs. of segregation for In and St= 8.13406706E-03 8.13406706E-03

q1f= 0.00000000 q2f= 0.00000000

Index of gamma value 39 gamma for St popn= 63.5199966

Means of q1 and q2= 6.83706487E-04 6.83706603E-04

Variances of q1 and q2= 5.44412687E-05 5.44412760E-05

Covariance and correlation of q1 and q2= -1.19933446E-07 -2.20298767E-03

F1 and F2= 7.96811432E-02 7.96811432E-02

Freqs. of fixation of A1 for In and St= 0.992050529 0.992050529

Freqs. of fixation of A2 for In and St= 0.00000000 0.00000000

Freqs. of segregation for In and St= 7.94947147E-03 7.94947147E-03

q1f= 0.00000000 q2f= 0.00000000

Index of gamma value 40 gamma for St popn= 65.1849976

Means of q1 and q2= 6.63437706E-04 6.63437706E-04

Variances of q1 and q2= 5.18960478E-05 5.18960551E-05

Covariance and correlation of q1 and q2= -1.12102185E-07 -2.16012937E-03

F1 and F2= 7.82748759E-02 7.82748833E-02

Freqs. of fixation of A1 for In and St= 0.992227972 0.992228031

Freqs. of fixation of A2 for In and St= 0.00000000 0.00000000

Freqs. of segregation for In and St= 7.77202845E-03 7.77196884E-03

q1f= 0.00000000 q2f= 0.00000000

Index of gamma value 41 gamma for St popn= 66.8499985

Means of q1 and q2= 6.44141051E-04 6.44140993E-04

Variances of q1 and q2= 4.95197091E-05 4.95197091E-05

Covariance and correlation of q1 and q2= -1.04918513E-07 -2.11872230E-03

F1 and F2= 7.69266710E-02 7.69266784E-02

Freqs. of fixation of A1 for In and St= 0.992399335 0.992399335

Freqs. of fixation of A2 for In and St= 0.00000000 0.00000000

Freqs. of segregation for In and St= 7.60066509E-03 7.60066509E-03

q1f= 0.00000000 q2f= 0.00000000

Index of gamma value 42 gamma for St popn= 68.5149994

Means of q1 and q2= 6.25750108E-04 6.25750108E-04

Variances of q1 and q2= 4.72976135E-05 4.72976171E-05

Covariance and correlation of q1 and q2= -9.83164909E-08 -2.07867753E-03

F1 and F2= 7.56327957E-02 7.56327957E-02

Freqs. of fixation of A1 for In and St= 0.992564380 0.992564380

Freqs. of fixation of A2 for In and St= 0.00000000 0.00000000

Freqs. of segregation for In and St= 7.43561983E-03 7.43561983E-03

q1f= 0.00000000 q2f= 0.00000000

Index of gamma value 43 gamma for St popn= 70.1800003

Means of q1 and q2= 6.08204282E-04 6.08204282E-04

Variances of q1 and q2= 4.52166241E-05 4.52166241E-05

Covariance and correlation of q1 and q2= -9.22387642E-08 -2.03993032E-03

F1 and F2= 7.43897110E-02 7.43897110E-02

Freqs. of fixation of A1 for In and St= 0.992723465 0.992723465

Freqs. of fixation of A2 for In and St= 0.00000000 0.00000000

Freqs. of segregation for In and St= 7.27653503E-03 7.27653503E-03

q1f= 0.00000000 q2f= 0.00000000

Index of gamma value 44 gamma for St popn= 71.8450012

Means of q1 and q2= 5.91448392E-04 5.91448392E-04

Variances of q1 and q2= 4.32650668E-05 4.32650741E-05

Covariance and correlation of q1 and q2= -8.66347136E-08 -2.00241711E-03

F1 and F2= 7.31943324E-02 7.31943473E-02

Freqs. of fixation of A1 for In and St= 0.992877185 0.992877185

Freqs. of fixation of A2 for In and St= 0.00000000 0.00000000

Freqs. of segregation for In and St= 7.12281466E-03 7.12281466E-03

q1f= 0.00000000 q2f= 0.00000000

Index of gamma value 45 gamma for St popn= 73.5099945

Means of q1 and q2= 5.75432496E-04 5.75432496E-04

Variances of q1 and q2= 4.14325041E-05 4.14325041E-05

Covariance and correlation of q1 and q2= -8.14590351E-08 -1.96606596E-03

F1 and F2= 7.20438287E-02 7.20438287E-02

Freqs. of fixation of A1 for In and St= 0.993025899 0.993025959

Freqs. of fixation of A2 for In and St= 0.00000000 0.00000000

Freqs. of segregation for In and St= 6.97410107E-03 6.97404146E-03

q1f= 0.00000000 q2f= 0.00000000

Index of gamma value 46 gamma for St popn= 75.1749954

Means of q1 and q2= 5.60110144E-04 5.60110144E-04

Variances of q1 and q2= 3.97093791E-05 3.97093754E-05

Covariance and correlation of q1 and q2= -7.66728334E-08 -1.93084951E-03

F1 and F2= 7.09353983E-02 7.09353909E-02

Freqs. of fixation of A1 for In and St= 0.993169248 0.993169248

Freqs. of fixation of A2 for In and St= 0.00000000 0.00000000

Freqs. of segregation for In and St= 6.83075190E-03 6.83075190E-03

q1f= 0.00000000 q2f= 0.00000000

Index of gamma value 47 gamma for St popn= 76.8399963

Means of q1 and q2= 5.45439019E-04 5.45438961E-04

Variances of q1 and q2= 3.80872589E-05 3.80872589E-05

Covariance and correlation of q1 and q2= -7.22398568E-08 -1.89669349E-03

F1 and F2= 6.98667392E-02 6.98667467E-02

Freqs. of fixation of A1 for In and St= 0.993308067 0.993308067

Freqs. of fixation of A2 for In and St= 0.00000000 0.00000000

Freqs. of segregation for In and St= 6.69193268E-03 6.69193268E-03

q1f= 0.00000000 q2f= 0.00000000

Index of gamma value 48 gamma for St popn= 78.5049973

Means of q1 and q2= 5.31379890E-04 5.31379890E-04

Variances of q1 and q2= 3.65583728E-05 3.65583728E-05

Covariance and correlation of q1 and q2= -6.81286849E-08 -1.86355901E-03

F1 and F2= 6.88355193E-02 6.88355193E-02

Freqs. of fixation of A1 for In and St= 0.993442357 0.993442357

Freqs. of fixation of A2 for In and St= 0.00000000 0.00000000

Freqs. of segregation for In and St= 6.55764341E-03 6.55764341E-03

q1f= 0.00000000 q2f= 0.00000000

Index of gamma value 49 gamma for St popn= 80.1699982

Means of q1 and q2= 5.17896900E-04 5.17897017E-04

Variances of q1 and q2= 3.51158196E-05 3.51158269E-05

Covariance and correlation of q1 and q2= -6.43108393E-08 -1.83139206E-03

F1 and F2= 6.78397864E-02 6.78397864E-02

Freqs. of fixation of A1 for In and St= 0.993572176 0.993571997

Freqs. of fixation of A2 for In and St= 0.00000000 0.00000000

Freqs. of segregation for In and St= 6.42782450E-03 6.42800331E-03

q1f= 0.00000000 q2f= 0.00000000

Index of gamma value 50 gamma for St popn= 81.8349991

Means of q1 and q2= 5.04957628E-04 5.04957687E-04

Variances of q1 and q2= 3.37532474E-05 3.37532474E-05

Covariance and correlation of q1 and q2= -6.07615505E-08 -1.80016900E-03

F1 and F2= 6.68774992E-02 6.68774918E-02

Freqs. of fixation of A1 for In and St= 0.993698299 0.993698299

Freqs. of fixation of A2 for In and St= 0.00000000 0.00000000

Freqs. of segregation for In and St= 6.30170107E-03 6.30170107E-03

q1f= 0.00000000 q2f= 0.00000000

Index of gamma value 51 gamma for St popn= 83.5000000

Means of q1 and q2= 4.92530118E-04 4.92530176E-04

Variances of q1 and q2= 3.24648390E-05 3.24648427E-05

Covariance and correlation of q1 and q2= -5.74573136E-08 -1.76983187E-03

F1 and F2= 6.59468994E-02 6.59468994E-02

Freqs. of fixation of A1 for In and St= 0.993819952 0.993819952

Freqs. of fixation of A2 for In and St= 0.00000000 0.00000000

Freqs. of segregation for In and St= 6.18004799E-03 6.18004799E-03

q1f= 0.00000000 q2f= 0.00000000

Index of gamma value 52 gamma for St popn= 85.1650009

Means of q1 and q2= 4.80586401E-04 4.80586401E-04

Variances of q1 and q2= 3.12453922E-05 3.12453885E-05

Covariance and correlation of q1 and q2= -5.43778071E-08 -1.74034666E-03

F1 and F2= 6.50463998E-02 6.50463924E-02

Freqs. of fixation of A1 for In and St= 0.993938148 0.993938267

Freqs. of fixation of A2 for In and St= 0.00000000 0.00000000

Freqs. of segregation for In and St= 6.06185198E-03 6.06173277E-03

q1f= 0.00000000 q2f= 0.00000000

Index of gamma value 53 gamma for St popn= 86.8300018

Means of q1 and q2= 4.69100312E-04 4.69100283E-04

Variances of q1 and q2= 3.00901211E-05 3.00901229E-05

Covariance and correlation of q1 and q2= -5.15046992E-08 -1.71168125E-03

F1 and F2= 6.41744286E-02 6.41744360E-02

Freqs. of fixation of A1 for In and St= 0.994052529 0.994052529

Freqs. of fixation of A2 for In and St= 0.00000000 0.00000000

Freqs. of segregation for In and St= 5.94747066E-03 5.94747066E-03

q1f= 0.00000000 q2f= 0.00000000

Index of gamma value 54 gamma for St popn= 88.4949951

Means of q1 and q2= 4.58046939E-04 4.58046823E-04

Variances of q1 and q2= 2.89946238E-05 2.89946238E-05

Covariance and correlation of q1 and q2= -4.88212635E-08 -1.68380397E-03

F1 and F2= 6.33295625E-02 6.33295774E-02

Freqs. of fixation of A1 for In and St= 0.994163811 0.994163811

Freqs. of fixation of A2 for In and St= 0.00000000 0.00000000

Freqs. of segregation for In and St= 5.83618879E-03 5.83618879E-03

q1f= 0.00000000 q2f= 0.00000000

Index of gamma value 55 gamma for St popn= 90.1599960

Means of q1 and q2= 4.47403872E-04 4.47403872E-04

Variances of q1 and q2= 2.79549167E-05 2.79549167E-05

Covariance and correlation of q1 and q2= -4.63124081E-08 -1.65668200E-03

F1 and F2= 6.25104755E-02 6.25104755E-02

Freqs. of fixation of A1 for In and St= 0.994271696 0.994271696

Freqs. of fixation of A2 for In and St= 0.00000000 0.00000000

Freqs. of segregation for In and St= 5.72830439E-03 5.72830439E-03

q1f= 0.00000000 q2f= 0.00000000

Index of gamma value 56 gamma for St popn= 91.8249969

Means of q1 and q2= 4.37149312E-04 4.37149254E-04

Variances of q1 and q2= 2.69672819E-05 2.69672819E-05

Covariance and correlation of q1 and q2= -4.39641639E-08 -1.63027795E-03

F1 and F2= 6.17159270E-02 6.17159382E-02

Freqs. of fixation of A1 for In and St= 0.994376302 0.994376302

Freqs. of fixation of A2 for In and St= 0.00000000 0.00000000

Freqs. of segregation for In and St= 5.62369823E-03 5.62369823E-03

q1f= 0.00000000 q2f= 0.00000000

Index of gamma value 57 gamma for St popn= 93.4899979

Means of q1 and q2= 4.27263964E-04 4.27263993E-04

Variances of q1 and q2= 2.60283796E-05 2.60283814E-05

Covariance and correlation of q1 and q2= -4.17644230E-08 -1.60457252E-03

F1 and F2= 6.09447695E-02 6.09447695E-02

Freqs. of fixation of A1 for In and St= 0.994478226 0.994478226

Freqs. of fixation of A2 for In and St= 0.00000000 0.00000000

Freqs. of segregation for In and St= 5.52177429E-03 5.52177429E-03

q1f= 0.00000000 q2f= 0.00000000

Index of gamma value 58 gamma for St popn= 95.1549988

Means of q1 and q2= 4.17728850E-04 4.17728821E-04

Variances of q1 and q2= 2.51350684E-05 2.51350684E-05

Covariance and correlation of q1 and q2= -3.97016748E-08 -1.57953321E-03

F1 and F2= 6.01959154E-02 6.01959229E-02

Freqs. of fixation of A1 for In and St= 0.994576752 0.994576752

Freqs. of fixation of A2 for In and St= 0.00000000 0.00000000

Freqs. of segregation for In and St= 5.42324781E-03 5.42324781E-03

q1f= 0.00000000 q2f= 0.00000000

Index of gamma value 59 gamma for St popn= 96.8199997

Means of q1 and q2= 4.08527441E-04 4.08527441E-04

Variances of q1 and q2= 2.42845181E-05 2.42845199E-05

Covariance and correlation of q1 and q2= -3.77661422E-08 -1.55515294E-03

F1 and F2= 5.94683252E-02 5.94683290E-02

Freqs. of fixation of A1 for In and St= 0.994672537 0.994672537

Freqs. of fixation of A2 for In and St= 0.00000000 0.00000000

Freqs. of segregation for In and St= 5.32746315E-03 5.32746315E-03

q1f= 0.00000000 q2f= 0.00000000

Index of gamma value 60 gamma for St popn= 98.4850006

Means of q1 and q2= 3.99642740E-04 3.99642682E-04

Variances of q1 and q2= 2.34740564E-05 2.34740583E-05

Covariance and correlation of q1 and q2= -3.59476076E-08 -1.53137592E-03

F1 and F2= 5.87610863E-02 5.87611012E-02

Freqs. of fixation of A1 for In and St= 0.994765997 0.994765997

Freqs. of fixation of A2 for In and St= 0.00000000 0.00000000

Freqs. of segregation for In and St= 5.23400307E-03 5.23400307E-03

q1f= 0.00000000 q2f= 0.00000000

Index of gamma value 61 gamma for St popn= 100.149994

Means of q1 and q2= 3.91059380E-04 3.91059380E-04

Variances of q1 and q2= 2.27012115E-05 2.27012097E-05

Covariance and correlation of q1 and q2= -3.42380062E-08 -1.50820171E-03

F1 and F2= 5.80732599E-02 5.80732562E-02

Freqs. of fixation of A1 for In and St= 0.994857132 0.994857132

Freqs. of fixation of A2 for In and St= 0.00000000 0.00000000

Freqs. of segregation for In and St= 5.14286757E-03 5.14286757E-03

q1f= 0.00000000 q2f= 0.00000000

Index of gamma value 62 gamma for St popn= 101.814995

Means of q1 and q2= 3.82764149E-04 3.82764090E-04

Variances of q1 and q2= 2.19637968E-05 2.19637968E-05

Covariance and correlation of q1 and q2= -3.26295648E-08 -1.48560677E-03

F1 and F2= 5.74040376E-02 5.74040450E-02

Freqs. of fixation of A1 for In and St= 0.994945168 0.994945109

Freqs. of fixation of A2 for In and St= 0.00000000 0.00000000

Freqs. of segregation for In and St= 5.05483150E-03 5.05489111E-03

q1f= 0.00000000 q2f= 0.00000000

Index of gamma value 63 gamma for St popn= 103.479996

Means of q1 and q2= 3.74742842E-04 3.74742784E-04

Variances of q1 and q2= 2.12596588E-05 2.12596569E-05

Covariance and correlation of q1 and q2= -3.11148156E-08 -1.46356155E-03

F1 and F2= 5.67525923E-02 5.67525961E-02

Freqs. of fixation of A1 for In and St= 0.995030940 0.995030880

Freqs. of fixation of A2 for In and St= 0.00000000 0.00000000

Freqs. of segregation for In and St= 4.96906042E-03 4.96912003E-03

q1f= 0.00000000 q2f= 0.00000000

Index of gamma value 64 gamma for St popn= 105.144997

Means of q1 and q2= 3.66983877E-04 3.66983906E-04

Variances of q1 and q2= 2.05869273E-05 2.05869273E-05

Covariance and correlation of q1 and q2= -2.96877758E-08 -1.44206930E-03

F1 and F2= 5.61182275E-02 5.61182238E-02

Freqs. of fixation of A1 for In and St= 0.995114923 0.995114923

Freqs. of fixation of A2 for In and St= 0.00000000 0.00000000

Freqs. of segregation for In and St= 4.88507748E-03 4.88507748E-03

q1f= 0.00000000 q2f= 0.00000000

Index of gamma value 65 gamma for St popn= 106.809998

Means of q1 and q2= 3.59474128E-04 3.59474128E-04

Variances of q1 and q2= 1.99437054E-05 1.99437090E-05

Covariance and correlation of q1 and q2= -2.83415673E-08 -1.42107822E-03

F1 and F2= 5.55001758E-02 5.55001870E-02

Freqs. of fixation of A1 for In and St= 0.995196521 0.995196521

Freqs. of fixation of A2 for In and St= 0.00000000 0.00000000

Freqs. of segregation for In and St= 4.80347872E-03 4.80347872E-03

q1f= 0.00000000 q2f= 0.00000000

Index of gamma value 66 gamma for St popn= 108.474998

Means of q1 and q2= 3.52203031E-04 3.52203031E-04

Variances of q1 and q2= 1.93283886E-05 1.93283886E-05

Covariance and correlation of q1 and q2= -2.70710245E-08 -1.40058366E-03

F1 and F2= 5.48978746E-02 5.48978746E-02

Freqs. of fixation of A1 for In and St= 0.995275676 0.995275676

Freqs. of fixation of A2 for In and St= 0.00000000 0.00000000

Freqs. of segregation for In and St= 4.72432375E-03 4.72432375E-03

q1f= 0.00000000 q2f= 0.00000000

Index of gamma value 67 gamma for St popn= 110.139999

Means of q1 and q2= 3.45159817E-04 3.45159846E-04

Variances of q1 and q2= 1.87393689E-05 1.87393725E-05

Covariance and correlation of q1 and q2= -2.58710244E-08 -1.38057058E-03

F1 and F2= 5.43106087E-02 5.43106161E-02

Freqs. of fixation of A1 for In and St= 0.995352924 0.995352805

Freqs. of fixation of A2 for In and St= 0.00000000 0.00000000

Freqs. of segregation for In and St= 4.64707613E-03 4.64719534E-03

q1f= 0.00000000 q2f= 0.00000000

Index of gamma value 68 gamma for St popn= 111.805000

Means of q1 and q2= 3.38335580E-04 3.38335522E-04

Variances of q1 and q2= 1.81752530E-05 1.81752530E-05

Covariance and correlation of q1 and q2= -2.47370480E-08 -1.36102911E-03

F1 and F2= 5.37377819E-02 5.37377931E-02

Freqs. of fixation of A1 for In and St= 0.995427847 0.995427847

Freqs. of fixation of A2 for In and St= 0.00000000 0.00000000

Freqs. of segregation for In and St= 4.57215309E-03 4.57215309E-03

q1f= 0.00000000 q2f= 0.00000000

Index of gamma value 69 gamma for St popn= 113.470001

Means of q1 and q2= 3.31719930E-04 3.31720017E-04

Variances of q1 and q2= 1.76346530E-05 1.76346530E-05

Covariance and correlation of q1 and q2= -2.36646684E-08 -1.34194130E-03

F1 and F2= 5.31789102E-02 5.31788990E-02

Freqs. of fixation of A1 for In and St= 0.995501339 0.995501339

Freqs. of fixation of A2 for In and St= 0.00000000 0.00000000

Freqs. of segregation for In and St= 4.49866056E-03 4.49866056E-03

q1f= 0.00000000 q2f= 0.00000000

Index of gamma value 70 gamma for St popn= 115.134995

Means of q1 and q2= 3.25304398E-04 3.25304369E-04

Variances of q1 and q2= 1.71162992E-05 1.71162974E-05

Covariance and correlation of q1 and q2= -2.26497292E-08 -1.32328423E-03

F1 and F2= 5.26333787E-02 5.26333749E-02

Freqs. of fixation of A1 for In and St= 0.995573103 0.995573223

Freqs. of fixation of A2 for In and St= 0.00000000 0.00000000

Freqs. of segregation for In and St= 4.42689657E-03 4.42677736E-03

q1f= 0.00000000 q2f= 0.00000000

Index of gamma value 71 gamma for St popn= 116.799995

Means of q1 and q2= 3.19080573E-04 3.19080573E-04

Variances of q1 and q2= 1.66190293E-05 1.66190293E-05

Covariance and correlation of q1 and q2= -2.16885852E-08 -1.30504521E-03

F1 and F2= 5.21007366E-02 5.21007366E-02

Freqs. of fixation of A1 for In and St= 0.995642424 0.995642424

Freqs. of fixation of A2 for In and St= 0.00000000 0.00000000

Freqs. of segregation for In and St= 4.35757637E-03 4.35757637E-03

q1f= 0.00000000 q2f= 0.00000000

Index of gamma value 72 gamma for St popn= 118.464996

Means of q1 and q2= 3.13040568E-04 3.13040568E-04

Variances of q1 and q2= 1.61417302E-05 1.61417338E-05

Covariance and correlation of q1 and q2= -2.07778825E-08 -1.28721516E-03

F1 and F2= 5.15804850E-02 5.15804961E-02

Freqs. of fixation of A1 for In and St= 0.995711029 0.995711029

Freqs. of fixation of A2 for In and St= 0.00000000 0.00000000

Freqs. of segregation for In and St= 4.28897142E-03 4.28897142E-03

q1f= 0.00000000 q2f= 0.00000000

Index of gamma value 73 gamma for St popn= 120.129997

Means of q1 and q2= 3.07177077E-04 3.07177077E-04

Variances of q1 and q2= 1.56833848E-05 1.56833830E-05

Covariance and correlation of q1 and q2= -1.99144807E-08 -1.26978219E-03

F1 and F2= 5.10721840E-02 5.10721765E-02

Freqs. of fixation of A1 for In and St= 0.995777190 0.995777190

Freqs. of fixation of A2 for In and St= 0.00000000 0.00000000

Freqs. of segregation for In and St= 4.22281027E-03 4.22281027E-03

q1f= 0.00000000 q2f= 0.00000000

Index of gamma value 74 gamma for St popn= 121.794998

Means of q1 and q2= 3.01482651E-04 3.01482709E-04

Variances of q1 and q2= 1.52430048E-05 1.52430066E-05

Covariance and correlation of q1 and q2= -1.90953457E-08 -1.25272840E-03

F1 and F2= 5.05753867E-02 5.05753830E-02

Freqs. of fixation of A1 for In and St= 0.995842397 0.995842397

Freqs. of fixation of A2 for In and St= 0.00000000 0.00000000

Freqs. of segregation for In and St= 4.15760279E-03 4.15760279E-03

q1f= 0.00000000 q2f= 0.00000000

Index of gamma value 75 gamma for St popn= 123.459999

Means of q1 and q2= 2.95951206E-04 2.95951206E-04

Variances of q1 and q2= 1.48197223E-05 1.48197223E-05

Covariance and correlation of q1 and q2= -1.83178699E-08 -1.23604678E-03

F1 and F2= 5.00897057E-02 5.00897057E-02

Freqs. of fixation of A1 for In and St= 0.995905459 0.995905578

Freqs. of fixation of A2 for In and St= 0.00000000 0.00000000

Freqs. of segregation for In and St= 4.09454107E-03 4.09442186E-03

q1f= 0.00000000 q2f= 0.00000000

Index of gamma value 76 gamma for St popn= 125.125000

Means of q1 and q2= 2.90575845E-04 2.90575816E-04

Variances of q1 and q2= 1.44126470E-05 1.44126479E-05

Covariance and correlation of q1 and q2= -1.75795520E-08 -1.21973094E-03

F1 and F2= 4.96147089E-02 4.96147163E-02

Freqs. of fixation of A1 for In and St= 0.995967209 0.995967209

Freqs. of fixation of A2 for In and St= 0.00000000 0.00000000

Freqs. of segregation for In and St= 4.03279066E-03 4.03279066E-03

q1f= 0.00000000 q2f= 0.00000000

Index of gamma value 77 gamma for St popn= 126.789993

Means of q1 and q2= 2.85350543E-04 2.85350485E-04

Variances of q1 and q2= 1.40210013E-05 1.40209995E-05

Covariance and correlation of q1 and q2= -1.68778271E-08 -1.20375340E-03

F1 and F2= 4.91500869E-02 4.91500907E-02

Freqs. of fixation of A1 for In and St= 0.996028125 0.996028125

Freqs. of fixation of A2 for In and St= 0.00000000 0.00000000

Freqs. of segregation for In and St= 3.97187471E-03 3.97187471E-03

q1f= 0.00000000 q2f= 0.00000000

Index of gamma value 78 gamma for St popn= 128.455002

Means of q1 and q2= 2.80269305E-04 2.80269305E-04

Variances of q1 and q2= 1.36440040E-05 1.36440058E-05

Covariance and correlation of q1 and q2= -1.62105991E-08 -1.18811149E-03

F1 and F2= 4.86954115E-02 4.86954190E-02

Freqs. of fixation of A1 for In and St= 0.996086955 0.996086955

Freqs. of fixation of A2 for In and St= 0.00000000 0.00000000

Freqs. of segregation for In and St= 3.91304493E-03 3.91304493E-03

q1f= 0.00000000 q2f= 0.00000000

Index of gamma value 79 gamma for St popn= 130.119995

Means of q1 and q2= 2.75327475E-04 2.75327475E-04

Variances of q1 and q2= 1.32810001E-05 1.32809982E-05

Covariance and correlation of q1 and q2= -1.55760986E-08 -1.17281079E-03

F1 and F2= 4.82503921E-02 4.82503846E-02

Freqs. of fixation of A1 for In and St= 0.996144652 0.996144652

Freqs. of fixation of A2 for In and St= 0.00000000 0.00000000

Freqs. of segregation for In and St= 3.85534763E-03 3.85534763E-03

q1f= 0.00000000 q2f= 0.00000000

Index of gamma value 80 gamma for St popn= 131.785004

Means of q1 and q2= 2.70518882E-04 2.70518882E-04

Variances of q1 and q2= 1.29312830E-05 1.29312866E-05

Covariance and correlation of q1 and q2= -1.49720201E-08 -1.15781382E-03

F1 and F2= 4.78147119E-02 4.78147268E-02

Freqs. of fixation of A1 for In and St= 0.996200919 0.996200919

Freqs. of fixation of A2 for In and St= 0.00000000 0.00000000

Freqs. of segregation for In and St= 3.79908085E-03 3.79908085E-03

q1f= 0.00000000 q2f= 0.00000000

Index of gamma value 81 gamma for St popn= 133.449997

Means of q1 and q2= 2.65839481E-04 2.65839422E-04

Variances of q1 and q2= 1.25942624E-05 1.25942624E-05

Covariance and correlation of q1 and q2= -1.43969210E-08 -1.14313327E-03

F1 and F2= 4.73880358E-02 4.73880470E-02

Freqs. of fixation of A1 for In and St= 0.996255934 0.996255934

Freqs. of fixation of A2 for In and St= 0.00000000 0.00000000

Freqs. of segregation for In and St= 3.74406576E-03 3.74406576E-03

q1f= 0.00000000 q2f= 0.00000000

Index of gamma value 82 gamma for St popn= 135.114990

Means of q1 and q2= 2.61283858E-04 2.61283858E-04

Variances of q1 and q2= 1.22693218E-05 1.22693227E-05

Covariance and correlation of q1 and q2= -1.38490357E-08 -1.12875318E-03

F1 and F2= 4.69700992E-02 4.69701029E-02

Freqs. of fixation of A1 for In and St= 0.996309757 0.996309757

Freqs. of fixation of A2 for In and St= 0.00000000 0.00000000

Freqs. of segregation for In and St= 3.69024277E-03 3.69024277E-03

q1f= 0.00000000 q2f= 0.00000000

Index of gamma value 83 gamma for St popn= 136.779999

Means of q1 and q2= 2.56847678E-04 2.56847648E-04

Variances of q1 and q2= 1.19559154E-05 1.19559154E-05

Covariance and correlation of q1 and q2= -1.33267513E-08 -1.11465750E-03

F1 and F2= 4.65606228E-02 4.65606265E-02

Freqs. of fixation of A1 for In and St= 0.996362567 0.996362567

Freqs. of fixation of A2 for In and St= 0.00000000 0.00000000

Freqs. of segregation for In and St= 3.63743305E-03 3.63743305E-03

q1f= 0.00000000 q2f= 0.00000000

Index of gamma value 84 gamma for St popn= 138.444992

Means of q1 and q2= 2.52526865E-04 2.52526865E-04

Variances of q1 and q2= 1.16535221E-05 1.16535239E-05

Covariance and correlation of q1 and q2= -1.28287780E-08 -1.10084971E-03

F1 and F2= 4.61593084E-02 4.61593159E-02

Freqs. of fixation of A1 for In and St= 0.996414065 0.996414065

Freqs. of fixation of A2 for In and St= 0.00000000 0.00000000

Freqs. of segregation for In and St= 3.58593464E-03 3.58593464E-03

q1f= 0.00000000 q2f= 0.00000000

Index of gamma value 85 gamma for St popn= 140.110001

Means of q1 and q2= 2.48317083E-04 2.48317141E-04

Variances of q1 and q2= 1.13616316E-05 1.13616325E-05

Covariance and correlation of q1 and q2= -1.23536843E-08 -1.08731596E-03

F1 and F2= 4.57658954E-02 4.57658879E-02

Freqs. of fixation of A1 for In and St= 0.996464014 0.996464014

Freqs. of fixation of A2 for In and St= 0.00000000 0.00000000

Freqs. of segregation for In and St= 3.53598595E-03 3.53598595E-03

q1f= 0.00000000 q2f= 0.00000000

Index of gamma value 86 gamma for St popn= 141.774994

Means of q1 and q2= 2.44214665E-04 2.44214665E-04

Variances of q1 and q2= 1.10798028E-05 1.10798028E-05

Covariance and correlation of q1 and q2= -1.19002159E-08 -1.07404578E-03

F1 and F2= 4.53801937E-02 4.53801937E-02

Freqs. of fixation of A1 for In and St= 0.996513247 0.996513188

Freqs. of fixation of A2 for In and St= 0.00000000 0.00000000

Freqs. of segregation for In and St= 3.48675251E-03 3.48681211E-03

q1f= 0.00000000 q2f= 0.00000000

Index of gamma value 87 gamma for St popn= 143.440002

Means of q1 and q2= 2.40215391E-04 2.40215391E-04

Variances of q1 and q2= 1.08075574E-05 1.08075583E-05

Covariance and correlation of q1 and q2= -1.14671828E-08 -1.06103369E-03

F1 and F2= 4.50019203E-02 4.50019240E-02

Freqs. of fixation of A1 for In and St= 0.996561825 0.996561825

Freqs. of fixation of A2 for In and St= 0.00000000 0.00000000

Freqs. of segregation for In and St= 3.43817472E-03 3.43817472E-03

q1f= 0.00000000 q2f= 0.00000000

Index of gamma value 88 gamma for St popn= 145.104996

Means of q1 and q2= 2.36316278E-04 2.36316278E-04

Variances of q1 and q2= 1.05445079E-05 1.05445088E-05

Covariance and correlation of q1 and q2= -1.10535510E-08 -1.04827562E-03

F1 and F2= 4.46308665E-02 4.46308702E-02

Freqs. of fixation of A1 for In and St= 0.996608675 0.996608675

Freqs. of fixation of A2 for In and St= 0.00000000 0.00000000

Freqs. of segregation for In and St= 3.39132547E-03 3.39132547E-03

q1f= 0.00000000 q2f= 0.00000000

Index of gamma value 89 gamma for St popn= 146.769989

Means of q1 and q2= 2.32513645E-04 2.32513630E-04

Variances of q1 and q2= 1.02902459E-05 1.02902459E-05

Covariance and correlation of q1 and q2= -1.06582796E-08 -1.03576528E-03

F1 and F2= 4.42668162E-02 4.42668200E-02

Freqs. of fixation of A1 for In and St= 0.996654868 0.996654868

Freqs. of fixation of A2 for In and St= 0.00000000 0.00000000

Freqs. of segregation for In and St= 3.34513187E-03 3.34513187E-03

q1f= 0.00000000 q2f= 0.00000000

Index of gamma value 90 gamma for St popn= 148.434998

Means of q1 and q2= 2.28804216E-04 2.28804274E-04

Variances of q1 and q2= 1.00443913E-05 1.00443931E-05

Covariance and correlation of q1 and q2= -1.02803490E-08 -1.02349138E-03

F1 and F2= 4.39095497E-02 4.39095460E-02

Freqs. of fixation of A1 for In and St= 0.996700466 0.996700466

Freqs. of fixation of A2 for In and St= 0.00000000 0.00000000

Freqs. of segregation for In and St= 3.29953432E-03 3.29953432E-03

q1f= 0.00000000 q2f= 0.00000000

Index of gamma value 91 gamma for St popn= 150.099991

Means of q1 and q2= 2.25184907E-04 2.25184922E-04

Variances of q1 and q2= 9.80659479E-06 9.80659661E-06

Covariance and correlation of q1 and q2= -9.91887461E-09 -1.01144938E-03

F1 and F2= 4.35588881E-02 4.35588919E-02

Freqs. of fixation of A1 for In and St= 0.996744394 0.996744394

Freqs. of fixation of A2 for In and St= 0.00000000 0.00000000

Freqs. of segregation for In and St= 3.25560570E-03 3.25560570E-03

q1f= 0.00000000 q2f= 0.00000000

Index of gamma value 92 gamma for St popn= 151.764999

Means of q1 and q2= 2.21653070E-04 2.21653056E-04

Variances of q1 and q2= 9.57653174E-06 9.57653265E-06

Covariance and correlation of q1 and q2= -9.57305701E-09 -9.99637181E-04

F1 and F2= 4.32146266E-02 4.32146341E-02

Freqs. of fixation of A1 for In and St= 0.996788025 0.996788025

Freqs. of fixation of A2 for In and St= 0.00000000 0.00000000

Freqs. of segregation for In and St= 3.21197510E-03 3.21197510E-03

q1f= 0.00000000 q2f= 0.00000000

Index of gamma value 93 gamma for St popn= 153.429993

Means of q1 and q2= 2.18205227E-04 2.18205227E-04

Variances of q1 and q2= 9.35385560E-06 9.35385378E-06

Covariance and correlation of q1 and q2= -9.24201515E-09 -9.88043495E-04

F1 and F2= 4.28765938E-02 4.28765863E-02

Freqs. of fixation of A1 for In and St= 0.996830285 0.996830285

Freqs. of fixation of A2 for In and St= 0.00000000 0.00000000

Freqs. of segregation for In and St= 3.16971540E-03 3.16971540E-03

q1f= 0.00000000 q2f= 0.00000000

Index of gamma value 94 gamma for St popn= 155.095001

Means of q1 and q2= 2.14839121E-04 2.14839165E-04

Variances of q1 and q2= 9.13828080E-06 9.13828080E-06

Covariance and correlation of q1 and q2= -8.92507046E-09 -9.76668438E-04

F1 and F2= 4.25445959E-02 4.25445884E-02

Freqs. of fixation of A1 for In and St= 0.996872425 0.996872425

Freqs. of fixation of A2 for In and St= 0.00000000 0.00000000

Freqs. of segregation for In and St= 3.12757492E-03 3.12757492E-03

q1f= 0.00000000 q2f= 0.00000000

Index of gamma value 95 gamma for St popn= 156.759995

Means of q1 and q2= 2.11551800E-04 2.11551800E-04

Variances of q1 and q2= 8.92950629E-06 8.92950720E-06

Covariance and correlation of q1 and q2= -8.62142713E-09 -9.65498621E-04

F1 and F2= 4.22184803E-02 4.22184840E-02

Freqs. of fixation of A1 for In and St= 0.996912837 0.996912837

Freqs. of fixation of A2 for In and St= 0.00000000 0.00000000

Freqs. of segregation for In and St= 3.08716297E-03 3.08716297E-03

q1f= 0.00000000 q2f= 0.00000000

Index of gamma value 96 gamma for St popn= 158.425003

Means of q1 and q2= 2.08340949E-04 2.08340949E-04

Variances of q1 and q2= 8.72726923E-06 8.72726923E-06

Covariance and correlation of q1 and q2= -8.33042790E-09 -9.54528572E-04

F1 and F2= 4.18980904E-02 4.18980904E-02

Freqs. of fixation of A1 for In and St= 0.996953249 0.996953249

Freqs. of fixation of A2 for In and St= 0.00000000 0.00000000

Freqs. of segregation for In and St= 3.04675102E-03 3.04675102E-03

q1f= 0.00000000 q2f= 0.00000000

Index of gamma value 97 gamma for St popn= 160.089996

Means of q1 and q2= 2.05204109E-04 2.05204138E-04

Variances of q1 and q2= 8.53130132E-06 8.53130223E-06

Covariance and correlation of q1 and q2= -8.05151856E-09 -9.43761959E-04

F1 and F2= 4.15832438E-02 4.15832438E-02

Freqs. of fixation of A1 for In and St= 0.996992290 0.996992290

Freqs. of fixation of A2 for In and St= 0.00000000 0.00000000

Freqs. of segregation for In and St= 3.00770998E-03 3.00770998E-03

q1f= 0.00000000 q2f= 0.00000000

Index of gamma value 98 gamma for St popn= 161.754990

Means of q1 and q2= 2.02138704E-04 2.02138704E-04

Variances of q1 and q2= 8.34134698E-06 8.34134607E-06

Covariance and correlation of q1 and q2= -7.78401343E-09 -9.33184347E-04

F1 and F2= 4.12738062E-02 4.12738025E-02

Freqs. of fixation of A1 for In and St= 0.997030139 0.997030139

Freqs. of fixation of A2 for In and St= 0.00000000 0.00000000

Freqs. of segregation for In and St= 2.96986103E-03 2.96986103E-03

q1f= 0.00000000 q2f= 0.00000000

Index of gamma value 99 gamma for St popn= 163.419998

Means of q1 and q2= 1.99143004E-04 1.99142989E-04

Variances of q1 and q2= 8.15718704E-06 8.15718704E-06

Covariance and correlation of q1 and q2= -7.52746132E-09 -9.22801089E-04

F1 and F2= 4.09696139E-02 4.09696139E-02

Freqs. of fixation of A1 for In and St= 0.997068405 0.997068584

Freqs. of fixation of A2 for In and St= 0.00000000 0.00000000

Freqs. of segregation for In and St= 2.93159485E-03 2.93141603E-03

q1f= 0.00000000 q2f= 0.00000000

Index of gamma value 100 gamma for St popn= 165.084991

Means of q1 and q2= 1.96214445E-04 1.96214431E-04

Variances of q1 and q2= 7.97858502E-06 7.97858502E-06

Covariance and correlation of q1 and q2= -7.28122984E-09 -9.12596646E-04

F1 and F2= 4.06705588E-02 4.06705625E-02

Freqs. of fixation of A1 for In and St= 0.997105241 0.997105241

Freqs. of fixation of A2 for In and St= 0.00000000 0.00000000

Freqs. of segregation for In and St= 2.89475918E-03 2.89475918E-03

q1f= 0.00000000 q2f= 0.00000000

Index of gamma value 101 gamma for St popn= 166.750000

Means of q1 and q2= 1.93351123E-04 1.93351138E-04

Variances of q1 and q2= 7.80532719E-06 7.80532719E-06

Covariance and correlation of q1 and q2= -7.04487846E-09 -9.02573112E-04

F1 and F2= 4.03764732E-02 4.03764695E-02

Freqs. of fixation of A1 for In and St= 0.997141421 0.997141421

Freqs. of fixation of A2 for In and St= 0.00000000 0.00000000

Freqs. of segregation for In and St= 2.85857916E-03 2.85857916E-03

q1f= 0.00000000 q2f= 0.00000000

Index of gamma value 102 gamma for St popn= 168.414993

Means of q1 and q2= 1.90551247E-04 1.90551262E-04

Variances of q1 and q2= 7.63721710E-06 7.63721800E-06

Covariance and correlation of q1 and q2= -6.81795953E-09 -8.92728160E-04

F1 and F2= 4.00872342E-02 4.00872380E-02

Freqs. of fixation of A1 for In and St= 0.997176468 0.997176468

Freqs. of fixation of A2 for In and St= 0.00000000 0.00000000

Freqs. of segregation for In and St= 2.82353163E-03 2.82353163E-03

q1f= 0.00000000 q2f= 0.00000000

Index of gamma value 103 gamma for St popn= 170.080002

Means of q1 and q2= 1.87812941E-04 1.87812941E-04

Variances of q1 and q2= 7.47406921E-06 7.47406875E-06

Covariance and correlation of q1 and q2= -6.59999699E-09 -8.83052708E-04

F1 and F2= 3.98027599E-02 3.98027562E-02

Freqs. of fixation of A1 for In and St= 0.997212231 0.997212231

Freqs. of fixation of A2 for In and St= 0.00000000 0.00000000

Freqs. of segregation for In and St= 2.78776884E-03 2.78776884E-03

q1f= 0.00000000 q2f= 0.00000000

Index of gamma value 104 gamma for St popn= 171.744995

Means of q1 and q2= 1.85133758E-04 1.85133715E-04

Variances of q1 and q2= 7.31566206E-06 7.31566115E-06

Covariance and correlation of q1 and q2= -6.39052189E-09 -8.73539830E-04

F1 and F2= 3.95228639E-02 3.95228676E-02

Freqs. of fixation of A1 for In and St= 0.997246265 0.997246265

Freqs. of fixation of A2 for In and St= 0.00000000 0.00000000

Freqs. of segregation for In and St= 2.75373459E-03 2.75373459E-03

q1f= 0.00000000 q2f= 0.00000000

Index of gamma value 105 gamma for St popn= 173.410004

Means of q1 and q2= 1.82512420E-04 1.82512391E-04

Variances of q1 and q2= 7.16184513E-06 7.16184559E-06

Covariance and correlation of q1 and q2= -6.18919493E-09 -8.64189933E-04

F1 and F2= 3.92474793E-02 3.92474867E-02

Freqs. of fixation of A1 for In and St= 0.997279704 0.997279704

Freqs. of fixation of A2 for In and St= 0.00000000 0.00000000

Freqs. of segregation for In and St= 2.72029638E-03 2.72029638E-03

q1f= 0.00000000 q2f= 0.00000000

Index of gamma value 106 gamma for St popn= 175.074997

Means of q1 and q2= 1.79947368E-04 1.79947368E-04

Variances of q1 and q2= 7.01245153E-06 7.01245153E-06

Covariance and correlation of q1 and q2= -5.99565553E-09 -8.55001330E-04

F1 and F2= 3.89764719E-02 3.89764719E-02

Freqs. of fixation of A1 for In and St= 0.997312665 0.997312665

Freqs. of fixation of A2 for In and St= 0.00000000 0.00000000

Freqs. of segregation for In and St= 2.68733501E-03 2.68733501E-03

q1f= 0.00000000 q2f= 0.00000000

Index of gamma value 107 gamma for St popn= 176.739990

Means of q1 and q2= 1.77436727E-04 1.77436727E-04

Variances of q1 and q2= 6.86730846E-06 6.86730891E-06

Covariance and correlation of q1 and q2= -5.80952886E-09 -8.45968782E-04

F1 and F2= 3.87097299E-02 3.87097299E-02

Freqs. of fixation of A1 for In and St= 0.997345090 0.997345090

Freqs. of fixation of A2 for In and St= 0.00000000 0.00000000

Freqs. of segregation for In and St= 2.65491009E-03 2.65491009E-03

q1f= 0.00000000 q2f= 0.00000000

Index of gamma value 108 gamma for St popn= 178.404999

Means of q1 and q2= 1.74979126E-04 1.74979126E-04

Variances of q1 and q2= 6.72627175E-06 6.72627084E-06

Covariance and correlation of q1 and q2= -5.63047919E-09 -8.37087748E-04

F1 and F2= 3.84471528E-02 3.84471454E-02

Freqs. of fixation of A1 for In and St= 0.997376800 0.997376800

Freqs. of fixation of A2 for In and St= 0.00000000 0.00000000

Freqs. of segregation for In and St= 2.62320042E-03 2.62320042E-03

q1f= 0.00000000 q2f= 0.00000000

Index of gamma value 109 gamma for St popn= 180.069992

Means of q1 and q2= 1.72572720E-04 1.72572720E-04

Variances of q1 and q2= 6.58917952E-06 6.58917907E-06

Covariance and correlation of q1 and q2= -5.45816725E-09 -8.28353164E-04

F1 and F2= 3.81886363E-02 3.81886326E-02

Freqs. of fixation of A1 for In and St= 0.997408271 0.997408271

Freqs. of fixation of A2 for In and St= 0.00000000 0.00000000

Freqs. of segregation for In and St= 2.59172916E-03 2.59172916E-03

q1f= 0.00000000 q2f= 0.00000000

Index of gamma value 110 gamma for St popn= 181.735001

Means of q1 and q2= 1.70216343E-04 1.70216343E-04

Variances of q1 and q2= 6.45590580E-06 6.45590580E-06

Covariance and correlation of q1 and q2= -5.29229105E-09 -8.19759618E-04

F1 and F2= 3.79341058E-02 3.79341058E-02

Freqs. of fixation of A1 for In and St= 0.997439027 0.997439027

Freqs. of fixation of A2 for In and St= 0.00000000 0.00000000

Freqs. of segregation for In and St= 2.56097317E-03 2.56097317E-03

q1f= 0.00000000 q2f= 0.00000000

Index of gamma value 111 gamma for St popn= 183.399994

Means of q1 and q2= 1.67908307E-04 1.67908307E-04

Variances of q1 and q2= 6.32629872E-06 6.32629963E-06

Covariance and correlation of q1 and q2= -5.13256637E-09 -8.11306294E-04

F1 and F2= 3.76834311E-02 3.76834348E-02

Freqs. of fixation of A1 for In and St= 0.997468889 0.997468889

Freqs. of fixation of A2 for In and St= 0.00000000 0.00000000

Freqs. of segregation for In and St= 2.53111124E-03 2.53111124E-03

q1f= 0.00000000 q2f= 0.00000000

Index of gamma value 112 gamma for St popn= 185.065002

Means of q1 and q2= 1.65647696E-04 1.65647696E-04

Variances of q1 and q2= 6.20025139E-06 6.20025139E-06

Covariance and correlation of q1 and q2= -4.97877650E-09 -8.02995928E-04

F1 and F2= 3.74365523E-02 3.74365523E-02

Freqs. of fixation of A1 for In and St= 0.997498393 0.997498393

Freqs. of fixation of A2 for In and St= 0.00000000 0.00000000

Freqs. of segregation for In and St= 2.50160694E-03 2.50160694E-03

q1f= 0.00000000 q2f= 0.00000000

Index of gamma value 113 gamma for St popn= 186.729996

Means of q1 and q2= 1.63432851E-04 1.63432880E-04

Variances of q1 and q2= 6.07762058E-06 6.07762104E-06

Covariance and correlation of q1 and q2= -4.83059104E-09 -7.94816180E-04

F1 and F2= 3.71933430E-02 3.71933393E-02

Freqs. of fixation of A1 for In and St= 0.997527540 0.997527540

Freqs. of fixation of A2 for In and St= 0.00000000 0.00000000

Freqs. of segregation for In and St= 2.47246027E-03 2.47246027E-03

q1f= 0.00000000 q2f= 0.00000000

Index of gamma value 114 gamma for St popn= 188.394989

Means of q1 and q2= 1.61262738E-04 1.61262738E-04

Variances of q1 and q2= 5.95829943E-06 5.95829943E-06

Covariance and correlation of q1 and q2= -4.68782879E-09 -7.86772929E-04

F1 and F2= 3.69537361E-02 3.69537361E-02

Freqs. of fixation of A1 for In and St= 0.997556150 0.997556150

Freqs. of fixation of A2 for In and St= 0.00000000 0.00000000

Freqs. of segregation for In and St= 2.44385004E-03 2.44385004E-03

q1f= 0.00000000 q2f= 0.00000000

Index of gamma value 115 gamma for St popn= 190.059998

Means of q1 and q2= 1.59135801E-04 1.59135801E-04

Variances of q1 and q2= 5.84216605E-06 5.84216605E-06

Covariance and correlation of q1 and q2= -4.55018601E-09 -7.78852555E-04

F1 and F2= 3.67176682E-02 3.67176682E-02

Freqs. of fixation of A1 for In and St= 0.997584045 0.997584045

Freqs. of fixation of A2 for In and St= 0.00000000 0.00000000

Freqs. of segregation for In and St= 2.41595507E-03 2.41595507E-03

q1f= 0.00000000 q2f= 0.00000000

Index of gamma value 116 gamma for St popn= 191.724991

Means of q1 and q2= 1.57051472E-04 1.57051472E-04

Variances of q1 and q2= 5.72912541E-06 5.72912541E-06

Covariance and correlation of q1 and q2= -4.41753656E-09 -7.71066465E-04

F1 and F2= 3.64850163E-02 3.64850163E-02

Freqs. of fixation of A1 for In and St= 0.997611344 0.997611344

Freqs. of fixation of A2 for In and St= 0.00000000 0.00000000

Freqs. of segregation for In and St= 2.38865614E-03 2.38865614E-03

q1f= 0.00000000 q2f= 0.00000000

Index of gamma value 117 gamma for St popn= 193.389999

Means of q1 and q2= 1.55007991E-04 1.55007976E-04

Variances of q1 and q2= 5.61905790E-06 5.61905790E-06

Covariance and correlation of q1 and q2= -4.28958025E-09 -7.63398479E-04

F1 and F2= 3.62557359E-02 3.62557396E-02

Freqs. of fixation of A1 for In and St= 0.997638404 0.997638404

Freqs. of fixation of A2 for In and St= 0.00000000 0.00000000

Freqs. of segregation for In and St= 2.36159563E-03 2.36159563E-03

q1f= 0.00000000 q2f= 0.00000000

Index of gamma value 118 gamma for St popn= 195.054993

Means of q1 and q2= 1.53004337E-04 1.53004294E-04

Variances of q1 and q2= 5.51186713E-06 5.51186577E-06

Covariance and correlation of q1 and q2= -4.16611101E-09 -7.55843997E-04

F1 and F2= 3.60297672E-02 3.60297672E-02

Freqs. of fixation of A1 for In and St= 0.997664571 0.997664571

Freqs. of fixation of A2 for In and St= 0.00000000 0.00000000

Freqs. of segregation for In and St= 2.33542919E-03 2.33542919E-03

q1f= 0.00000000 q2f= 0.00000000

Index of gamma value 119 gamma for St popn= 196.720001

Means of q1 and q2= 1.51039887E-04 1.51039858E-04

Variances of q1 and q2= 5.40746350E-06 5.40746350E-06

Covariance and correlation of q1 and q2= -4.04703648E-09 -7.48416816E-04

F1 and F2= 3.58069688E-02 3.58069763E-02

Freqs. of fixation of A1 for In and St= 0.997690618 0.997690618

Freqs. of fixation of A2 for In and St= 0.00000000 0.00000000

Freqs. of segregation for In and St= 2.30938196E-03 2.30938196E-03

q1f= 0.00000000 q2f= 0.00000000

Index of gamma value 120 gamma for St popn= 198.384995

Means of q1 and q2= 1.49113286E-04 1.49113257E-04

Variances of q1 and q2= 5.30575517E-06 5.30575517E-06

Covariance and correlation of q1 and q2= -3.93209554E-09 -7.41100055E-04

F1 and F2= 3.55873480E-02 3.55873555E-02

Freqs. of fixation of A1 for In and St= 0.997716427 0.997716427

Freqs. of fixation of A2 for In and St= 0.00000000 0.00000000

Freqs. of segregation for In and St= 2.28357315E-03 2.28357315E-03

q1f= 0.00000000 q2f= 0.00000000

Index of gamma value 121 gamma for St popn= 200.049988

Means of q1 and q2= 1.47223720E-04 1.47223735E-04

Variances of q1 and q2= 5.20665117E-06 5.20665117E-06

Covariance and correlation of q1 and q2= -3.82116916E-09 -7.33901514E-04

F1 and F2= 3.53707783E-02 3.53707746E-02

Freqs. of fixation of A1 for In and St= 0.997741938 0.997741938

Freqs. of fixation of A2 for In and St= 0.00000000 0.00000000

Freqs. of segregation for In and St= 2.25806236E-03 2.25806236E-03

q1f= 0.00000000 q2f= 0.00000000

Index of gamma value 122 gamma for St popn= 201.714996

Means of q1 and q2= 1.45369821E-04 1.45369806E-04

Variances of q1 and q2= 5.11005646E-06 5.11005555E-06

Covariance and correlation of q1 and q2= -3.71401931E-09 -7.26806000E-04

F1 and F2= 3.51572223E-02 3.51572223E-02

Freqs. of fixation of A1 for In and St= 0.997766554 0.997766733

Freqs. of fixation of A2 for In and St= 0.00000000 0.00000000

Freqs. of segregation for In and St= 2.23344564E-03 2.23326683E-03

q1f= 0.00000000 q2f= 0.00000000

Index of gamma value 123 gamma for St popn= 203.379990

Means of q1 and q2= 1.43551370E-04 1.43551370E-04

Variances of q1 and q2= 5.01591285E-06 5.01591285E-06

Covariance and correlation of q1 and q2= -3.61057673E-09 -7.19824457E-04

F1 and F2= 3.49466056E-02 3.49466056E-02

Freqs. of fixation of A1 for In and St= 0.997791231 0.997791231

Freqs. of fixation of A2 for In and St= 0.00000000 0.00000000

Freqs. of segregation for In and St= 2.20876932E-03 2.20876932E-03

q1f= 0.00000000 q2f= 0.00000000

Index of gamma value 124 gamma for St popn= 205.044998

Means of q1 and q2= 1.41766854E-04 1.41766883E-04

Variances of q1 and q2= 4.92412209E-06 4.92412209E-06

Covariance and correlation of q1 and q2= -3.51064955E-09 -7.12949317E-04

F1 and F2= 3.47388685E-02 3.47388610E-02

Freqs. of fixation of A1 for In and St= 0.997815430 0.997815430

Freqs. of fixation of A2 for In and St= 0.00000000 0.00000000

Freqs. of segregation for In and St= 2.18456984E-03 2.18456984E-03

q1f= 0.00000000 q2f= 0.00000000

Index of gamma value 125 gamma for St popn= 206.709991

Means of q1 and q2= 1.40015502E-04 1.40015502E-04

Variances of q1 and q2= 4.83461326E-06 4.83461190E-06

Covariance and correlation of q1 and q2= -3.41404949E-09 -7.06168183E-04

F1 and F2= 3.45339626E-02 3.45339552E-02

Freqs. of fixation of A1 for In and St= 0.997839153 0.997839153

Freqs. of fixation of A2 for In and St= 0.00000000 0.00000000

Freqs. of segregation for In and St= 2.16084719E-03 2.16084719E-03

q1f= 0.00000000 q2f= 0.00000000

Index of gamma value 126 gamma for St popn= 208.375000

Means of q1 and q2= 1.38296920E-04 1.38296891E-04

Variances of q1 and q2= 4.74732178E-06 4.74732178E-06

Covariance and correlation of q1 and q2= -3.32074812E-09 -6.99499273E-04

F1 and F2= 3.43317725E-02 3.43317799E-02

Freqs. of fixation of A1 for In and St= 0.997861862 0.997861862

Freqs. of fixation of A2 for In and St= 0.00000000 0.00000000

Freqs. of segregation for In and St= 2.13813782E-03 2.13813782E-03

q1f= 0.00000000 q2f= 0.00000000

Index of gamma value 127 gamma for St popn= 210.039993

Means of q1 and q2= 1.36609844E-04 1.36609844E-04

Variances of q1 and q2= 4.66217352E-06 4.66217352E-06

Covariance and correlation of q1 and q2= -3.23054117E-09 -6.92925998E-04

F1 and F2= 3.41323167E-02 3.41323167E-02

Freqs. of fixation of A1 for In and St= 0.997884929 0.997884929

Freqs. of fixation of A2 for In and St= 0.00000000 0.00000000

Freqs. of segregation for In and St= 2.11507082E-03 2.11507082E-03

q1f= 0.00000000 q2f= 0.00000000

Index of gamma value 128 gamma for St popn= 211.705002

Means of q1 and q2= 1.34953472E-04 1.34953487E-04

Variances of q1 and q2= 4.57909482E-06 4.57909573E-06

Covariance and correlation of q1 and q2= -3.14330251E-09 -6.86446205E-04

F1 and F2= 3.39354947E-02 3.39354947E-02

Freqs. of fixation of A1 for In and St= 0.997907639 0.997907639

Freqs. of fixation of A2 for In and St= 0.00000000 0.00000000

Freqs. of segregation for In and St= 2.09236145E-03 2.09236145E-03

q1f= 0.00000000 q2f= 0.00000000

Index of gamma value 129 gamma for St popn= 213.369995

Means of q1 and q2= 1.33327354E-04 1.33327369E-04

Variances of q1 and q2= 4.49803201E-06 4.49803292E-06

Covariance and correlation of q1 and q2= -3.05894954E-09 -6.80063909E-04

F1 and F2= 3.37412544E-02 3.37412581E-02

Freqs. of fixation of A1 for In and St= 0.997929811 0.997929811

Freqs. of fixation of A2 for In and St= 0.00000000 0.00000000

Freqs. of segregation for In and St= 2.07018852E-03 2.07018852E-03

q1f= 0.00000000 q2f= 0.00000000

Index of gamma value 130 gamma for St popn= 215.034988

Means of q1 and q2= 1.31730572E-04 1.31730572E-04

Variances of q1 and q2= 4.41891962E-06 4.41892053E-06

Covariance and correlation of q1 and q2= -2.97737657E-09 -6.73779228E-04

F1 and F2= 3.35495509E-02 3.35495584E-02

Freqs. of fixation of A1 for In and St= 0.997951150 0.997951150

Freqs. of fixation of A2 for In and St= 0.00000000 0.00000000

Freqs. of segregation for In and St= 2.04885006E-03 2.04885006E-03

q1f= 0.00000000 q2f= 0.00000000

Index of gamma value 131 gamma for St popn= 216.699997

Means of q1 and q2= 1.30162400E-04 1.30162414E-04

Variances of q1 and q2= 4.34169760E-06 4.34169715E-06

Covariance and correlation of q1 and q2= -2.89843882E-09 -6.67581917E-04

F1 and F2= 3.33603472E-02 3.33603397E-02

Freqs. of fixation of A1 for In and St= 0.997972786 0.997972786

Freqs. of fixation of A2 for In and St= 0.00000000 0.00000000

Freqs. of segregation for In and St= 2.02721357E-03 2.02721357E-03

q1f= 0.00000000 q2f= 0.00000000

Index of gamma value 132 gamma for St popn= 218.364990

Means of q1 and q2= 1.28622269E-04 1.28622283E-04

Variances of q1 and q2= 4.26631095E-06 4.26631095E-06

Covariance and correlation of q1 and q2= -2.82205281E-09 -6.61473779E-04

F1 and F2= 3.31735685E-02 3.31735648E-02

Freqs. of fixation of A1 for In and St= 0.997993946 0.997993946

Freqs. of fixation of A2 for In and St= 0.00000000 0.00000000

Freqs. of segregation for In and St= 2.00605392E-03 2.00605392E-03

q1f= 0.00000000 q2f= 0.00000000

Index of gamma value 133 gamma for St popn= 220.029999

Means of q1 and q2= 1.27109393E-04 1.27109335E-04

Variances of q1 and q2= 4.19269918E-06 4.19269873E-06

Covariance and correlation of q1 and q2= -2.74812706E-09 -6.55455340E-04

F1 and F2= 3.29891630E-02 3.29891741E-02

Freqs. of fixation of A1 for In and St= 0.998014688 0.998014688

Freqs. of fixation of A2 for In and St= 0.00000000 0.00000000

Freqs. of segregation for In and St= 1.98531151E-03 1.98531151E-03

q1f= 0.00000000 q2f= 0.00000000

Index of gamma value 134 gamma for St popn= 221.694992

Means of q1 and q2= 1.25623017E-04 1.25622988E-04

Variances of q1 and q2= 4.12081135E-06 4.12081090E-06

Covariance and correlation of q1 and q2= -2.67654343E-09 -6.49518566E-04

F1 and F2= 3.28071192E-02 3.28071229E-02

Freqs. of fixation of A1 for In and St= 0.998034775 0.998034775

Freqs. of fixation of A2 for In and St= 0.00000000 0.00000000

Freqs. of segregation for In and St= 1.96522474E-03 1.96522474E-03

q1f= 0.00000000 q2f= 0.00000000

Index of gamma value 135 gamma for St popn= 223.360001

Means of q1 and q2= 1.24162674E-04 1.24162660E-04

Variances of q1 and q2= 4.05059609E-06 4.05059518E-06

Covariance and correlation of q1 and q2= -2.60725486E-09 -6.43671956E-04

F1 and F2= 3.26273479E-02 3.26273441E-02

Freqs. of fixation of A1 for In and St= 0.998055220 0.998055220

Freqs. of fixation of A2 for In and St= 0.00000000 0.00000000

Freqs. of segregation for In and St= 1.94478035E-03 1.94478035E-03

q1f= 0.00000000 q2f= 0.00000000

Index of gamma value 136 gamma for St popn= 225.024994

Means of q1 and q2= 1.22727768E-04 1.22727797E-04

Variances of q1 and q2= 3.98200837E-06 3.98200837E-06

Covariance and correlation of q1 and q2= -2.54013699E-09 -6.37903460E-04

F1 and F2= 3.24498452E-02 3.24498378E-02

Freqs. of fixation of A1 for In and St= 0.998074949 0.998074949

Freqs. of fixation of A2 for In and St= 0.00000000 0.00000000

Freqs. of segregation for In and St= 1.92505121E-03 1.92505121E-03

q1f= 0.00000000 q2f= 0.00000000

Index of gamma value 137 gamma for St popn= 226.690002

Means of q1 and q2= 1.21317607E-04 1.21317571E-04

Variances of q1 and q2= 3.91499270E-06 3.91499270E-06

Covariance and correlation of q1 and q2= -2.47510901E-09 -6.32212905E-04

F1 and F2= 3.22745182E-02 3.22745293E-02

Freqs. of fixation of A1 for In and St= 0.998093963 0.998094201

Freqs. of fixation of A2 for In and St= 0.00000000 0.00000000

Freqs. of segregation for In and St= 1.90603733E-03 1.90579891E-03

q1f= 0.00000000 q2f= 0.00000000

Index of gamma value 138 gamma for St popn= 228.354996

Means of q1 and q2= 1.19931799E-04 1.19931785E-04

Variances of q1 and q2= 3.84951409E-06 3.84951318E-06

Covariance and correlation of q1 and q2= -2.41215226E-09 -6.26612222E-04

F1 and F2= 3.21013778E-02 3.21013741E-02

Freqs. of fixation of A1 for In and St= 0.998113573 0.998113573

Freqs. of fixation of A2 for In and St= 0.00000000 0.00000000

Freqs. of segregation for In and St= 1.88642740E-03 1.88642740E-03

q1f= 0.00000000 q2f= 0.00000000

Index of gamma value 139 gamma for St popn= 230.019989

Means of q1 and q2= 1.18569493E-04 1.18569442E-04

Variances of q1 and q2= 3.78551567E-06 3.78551476E-06

Covariance and correlation of q1 and q2= -2.35111575E-09 -6.21082087E-04

F1 and F2= 3.19303423E-02 3.19303498E-02

Freqs. of fixation of A1 for In and St= 0.998132646 0.998132646

Freqs. of fixation of A2 for In and St= 0.00000000 0.00000000

Freqs. of segregation for In and St= 1.86735392E-03 1.86735392E-03

q1f= 0.00000000 q2f= 0.00000000

Index of gamma value 140 gamma for St popn= 231.684998

Means of q1 and q2= 1.17230440E-04 1.17230455E-04

Variances of q1 and q2= 3.72296472E-06 3.72296518E-06

Covariance and correlation of q1 and q2= -2.29198260E-09 -6.15633675E-04

F1 and F2= 3.17613855E-02 3.17613855E-02

Freqs. of fixation of A1 for In and St= 0.998150766 0.998150766

Freqs. of fixation of A2 for In and St= 0.00000000 0.00000000

Freqs. of segregation for In and St= 1.84923410E-03 1.84923410E-03

q1f= 0.00000000 q2f= 0.00000000

Index of gamma value 141 gamma for St popn= 233.349991

Means of q1 and q2= 1.15913870E-04 1.15913848E-04

Variances of q1 and q2= 3.66181439E-06 3.66181416E-06

Covariance and correlation of q1 and q2= -2.23464358E-09 -6.10255869E-04

F1 and F2= 3.15944850E-02 3.15944888E-02

Freqs. of fixation of A1 for In and St= 0.998169601 0.998169601

Freqs. of fixation of A2 for In and St= 0.00000000 0.00000000

Freqs. of segregation for In and St= 1.83039904E-03 1.83039904E-03

q1f= 0.00000000 q2f= 0.00000000

Index of gamma value 142 gamma for St popn= 235.014999

Means of q1 and q2= 1.14619470E-04 1.14619448E-04

Variances of q1 and q2= 3.60202716E-06 3.60202671E-06

Covariance and correlation of q1 and q2= -2.17906404E-09 -6.04954956E-04

F1 and F2= 3.14295664E-02 3.14295664E-02

Freqs. of fixation of A1 for In and St= 0.998187900 0.998187900

Freqs. of fixation of A2 for In and St= 0.00000000 0.00000000

Freqs. of segregation for In and St= 1.81210041E-03 1.81210041E-03

q1f= 0.00000000 q2f= 0.00000000

Index of gamma value 143 gamma for St popn= 236.679993

Means of q1 and q2= 1.13346512E-04 1.13346527E-04

Variances of q1 and q2= 3.54356030E-06 3.54356030E-06

Covariance and correlation of q1 and q2= -2.12515516E-09 -5.99723135E-04

F1 and F2= 3.12666185E-02 3.12666148E-02

Freqs. of fixation of A1 for In and St= 0.998205125 0.998205125

Freqs. of fixation of A2 for In and St= 0.00000000 0.00000000

Freqs. of segregation for In and St= 1.79487467E-03 1.79487467E-03

q1f= 0.00000000 q2f= 0.00000000

Index of gamma value 144 gamma for St popn= 238.345001

Means of q1 and q2= 1.12094669E-04 1.12094669E-04

Variances of q1 and q2= 3.48638105E-06 3.48638150E-06

Covariance and correlation of q1 and q2= -2.07287254E-09 -5.94562793E-04

F1 and F2= 3.11056003E-02 3.11056040E-02

Freqs. of fixation of A1 for In and St= 0.998223007 0.998223007

Freqs. of fixation of A2 for In and St= 0.00000000 0.00000000

Freqs. of segregation for In and St= 1.77699327E-03 1.77699327E-03

q1f= 0.00000000 q2f= 0.00000000

Index of gamma value 145 gamma for St popn= 240.009995

Means of q1 and q2= 1.10863512E-04 1.10863512E-04

Variances of q1 and q2= 3.43045599E-06 3.43045554E-06

Covariance and correlation of q1 and q2= -2.02215755E-09 -5.89472009E-04

F1 and F2= 3.09464876E-02 3.09464838E-02

Freqs. of fixation of A1 for In and St= 0.998240411 0.998240411

Freqs. of fixation of A2 for In and St= 0.00000000 0.00000000

Freqs. of segregation for In and St= 1.75958872E-03 1.75958872E-03

q1f= 0.00000000 q2f= 0.00000000

Index of gamma value 146 gamma for St popn= 241.674988

Means of q1 and q2= 1.09652588E-04 1.09652610E-04

Variances of q1 and q2= 3.37574807E-06 3.37574807E-06

Covariance and correlation of q1 and q2= -1.97295691E-09 -5.84450259E-04

F1 and F2= 3.07892263E-02 3.07892207E-02

Freqs. of fixation of A1 for In and St= 0.998257399 0.998257399

Freqs. of fixation of A2 for In and St= 0.00000000 0.00000000

Freqs. of segregation for In and St= 1.74260139E-03 1.74260139E-03

q1f= 0.00000000 q2f= 0.00000000

Index of gamma value 147 gamma for St popn= 243.339996

Means of q1 and q2= 1.08461332E-04 1.08461383E-04

Variances of q1 and q2= 3.32222112E-06 3.32222157E-06

Covariance and correlation of q1 and q2= -1.92521377E-09 -5.79495914E-04

F1 and F2= 3.06337886E-02 3.06337792E-02

Freqs. of fixation of A1 for In and St= 0.998274148 0.998274148

Freqs. of fixation of A2 for In and St= 0.00000000 0.00000000

Freqs. of segregation for In and St= 1.72585249E-03 1.72585249E-03

q1f= 0.00000000 q2f= 0.00000000

Index of gamma value 148 gamma for St popn= 245.004990

Means of q1 and q2= 1.07289197E-04 1.07289234E-04

Variances of q1 and q2= 3.26983763E-06 3.26983763E-06

Covariance and correlation of q1 and q2= -1.87886950E-09 -5.74606354E-04

F1 and F2= 3.04801278E-02 3.04801185E-02

Freqs. of fixation of A1 for In and St= 0.998290598 0.998290598

Freqs. of fixation of A2 for In and St= 0.00000000 0.00000000

Freqs. of segregation for In and St= 1.70940161E-03 1.70940161E-03

q1f= 0.00000000 q2f= 0.00000000

Index of gamma value 149 gamma for St popn= 246.669998

Means of q1 and q2= 1.06136111E-04 1.06136162E-04

Variances of q1 and q2= 3.21858056E-06 3.21858079E-06

Covariance and correlation of q1 and q2= -1.83388771E-09 -5.69781521E-04

F1 and F2= 3.03282496E-02 3.03282365E-02

Freqs. of fixation of A1 for In and St= 0.998306870 0.998306870

Freqs. of fixation of A2 for In and St= 0.00000000 0.00000000

Freqs. of segregation for In and St= 1.69312954E-03 1.69312954E-03

q1f= 0.00000000 q2f= 0.00000000

Index of gamma value 150 gamma for St popn= 248.334991

Means of q1 and q2= 1.05001520E-04 1.05001520E-04

Variances of q1 and q2= 3.16841124E-06 3.16841079E-06

Covariance and correlation of q1 and q2= -1.79021065E-09 -5.65018447E-04

F1 and F2= 3.01780794E-02 3.01780738E-02

Freqs. of fixation of A1 for In and St= 0.998323321 0.998323321

Freqs. of fixation of A2 for In and St= 0.00000000 0.00000000

Freqs. of segregation for In and St= 1.67667866E-03 1.67667866E-03

q1f= 0.00000000 q2f= 0.00000000

Index of gamma value 151 gamma for St popn= 250.000000

Means of q1 and q2= 1.03884937E-04 1.03884959E-04

Variances of q1 and q2= 3.11929989E-06 3.11930057E-06

Covariance and correlation of q1 and q2= -1.74781611E-09 -5.60323184E-04

F1 and F2= 3.00296079E-02 3.00296079E-02

Freqs. of fixation of A1 for In and St= 0.998338759 0.998338759

Freqs. of fixation of A2 for In and St= 0.00000000 0.00000000

Freqs. of segregation for In and St= 1.66124105E-03 1.66124105E-03

q1f= 0.00000000 q2f= 0.00000000

**h=0.25**

Index of gamma value 7 gamma for St popn= 2.24000001

Means of q1 and q2= 0.212844044 0.212844044

Variances of q1 and q2= 0.163636342 0.163636342

Covariance and correlation of q1 and q2= -2.72393618E-02 -0.166462794

F1 and F2= 0.976691663 0.976691663

Freqs. of fixation of A1 for In and St= 0.783229351 0.783229351

Freqs. of fixation of A2 for In and St= 0.188938692 0.188938692

Freqs. of segregation for In and St= 2.78319567E-02 2.78319567E-02

q1f= 0.194347769 q2f= 0.194347769

Index of gamma value 8 gamma for St popn= 2.57166672

Means of q1 and q2= 0.173288241 0.173288241

Variances of q1 and q2= 0.139461920 0.139461920

Covariance and correlation of q1 and q2= -1.94569007E-02 -0.139514074

F1 and F2= 0.973492026 0.973492026

Freqs. of fixation of A1 for In and St= 0.815993071 0.815993071

Freqs. of fixation of A2 for In and St= 0.156649053 0.156649068

Freqs. of segregation for In and St= 2.73578763E-02 2.73578614E-02

q1f= 0.161055177 q2f= 0.161055192

Index of gamma value 9 gamma for St popn= 2.90333343

Means of q1 and q2= 0.141242489 0.141242489

Variances of q1 and q2= 0.117612273 0.117612273

Covariance and correlation of q1 and q2= -1.37929749E-02 -0.117274962

F1 and F2= 0.969653904 0.969653904

Freqs. of fixation of A1 for In and St= 0.844160736 0.844160736

Freqs. of fixation of A2 for In and St= 0.129018024 0.129018024

Freqs. of segregation for In and St= 2.68212408E-02 2.68212408E-02

q1f= 0.132573813 q2f= 0.132573813

Index of gamma value 10 gamma for St popn= 3.23500013

Means of q1 and q2= 0.115040056 0.115040056

Variances of q1 and q2= 9.82483700E-02 9.82483700E-02

Covariance and correlation of q1 and q2= -9.64610837E-03 -9.81808454E-02

F1 and F2= 0.965056300 0.965056300

Freqs. of fixation of A1 for In and St= 0.868238926 0.868238926

Freqs. of fixation of A2 for In and St= 0.105525889 0.105525889

Freqs. of segregation for In and St= 2.62351856E-02 2.62351856E-02

q1f= 0.108368963 q2f= 0.108368963

Index of gamma value 11 gamma for St popn= 3.56666684

Means of q1 and q2= 9.35304314E-02 9.35304314E-02

Variances of q1 and q2= 8.13526809E-02 8.13526809E-02

Covariance and correlation of q1 and q2= -6.64243475E-03 -8.16498548E-02

F1 and F2= 0.959545791 0.959545791

Freqs. of fixation of A1 for In and St= 0.888661742 0.888661742

Freqs. of fixation of A2 for In and St= 8.57256502E-02 8.57256502E-02

Freqs. of segregation for In and St= 2.56126076E-02 2.56126076E-02

q1f= 8.79790187E-02 q2f= 8.79790187E-02

Index of gamma value 12 gamma for St popn= 3.89833355

Means of q1 and q2= 7.58775547E-02 7.58775622E-02

Variances of q1 and q2= 6.68200552E-02 6.68200552E-02

Covariance and correlation of q1 and q2= -4.50591510E-03 -6.74335733E-02

F1 and F2= 0.952936590 0.952936471

Freqs. of fixation of A1 for In and St= 0.905834734 0.905834734

Freqs. of fixation of A2 for In and St= 6.91990480E-02 6.91990480E-02

Freqs. of segregation for In and St= 2.49662176E-02 2.49662176E-02

q1f= 7.09709227E-02 q2f= 7.09709227E-02

Index of gamma value 13 gamma for St popn= 4.23000002

Means of q1 and q2= 6.14329092E-02 6.14329092E-02

Variances of q1 and q2= 5.44885583E-02 5.44885583E-02

Covariance and correlation of q1 and q2= -3.01615661E-03 -5.53539433E-02

F1 and F2= 0.945015430 0.945015430

Freqs. of fixation of A1 for In and St= 0.920152903 0.920153022

Freqs. of fixation of A2 for In and St= 5.55395931E-02 5.55395931E-02

Freqs. of segregation for In and St= 2.43075043E-02 2.43073851E-02

q1f= 5.69232553E-02 q2f= 5.69232479E-02

Index of gamma value 14 gamma for St popn= 4.56166697

Means of q1 and q2= 4.96650599E-02 4.96650636E-02

Variances of q1 and q2= 4.41560633E-02 4.41560671E-02

Covariance and correlation of q1 and q2= -1.99673651E-03 -4.52199802E-02

F1 and F2= 0.935540617 0.935540736

Freqs. of fixation of A1 for In and St= 0.931999207 0.931999207

Freqs. of fixation of A2 for In and St= 4.43537608E-02 4.43537608E-02

Freqs. of segregation for In and St= 2.36470327E-02 2.36470327E-02

q1f= 4.54279967E-02 q2f= 4.54279967E-02

Index of gamma value 15 gamma for St popn= 4.89333344

Means of q1 and q2= 4.01240811E-02 4.01240811E-02

Variances of q1 and q2= 3.55965421E-02 3.55965421E-02

Covariance and correlation of q1 and q2= -1.31053908E-03 -3.68164703E-02

F1 and F2= 0.924246013 0.924246013

Freqs. of fixation of A1 for In and St= 0.941736758 0.941736758

Freqs. of fixation of A2 for In and St= 3.52694876E-02 3.52694876E-02

Freqs. of segregation for In and St= 2.29937546E-02 2.29937546E-02

q1f= 3.60995531E-02 q2f= 3.60995531E-02

Index of gamma value 16 gamma for St popn= 5.22500038

Means of q1 and q2= 3.21950391E-02 3.21950391E-02

Variances of q1 and q2= 2.83326730E-02 2.83326730E-02

Covariance and correlation of q1 and q2= -8.40820605E-04 -2.96767130E-02

F1 and F2= 0.909307480 0.909307480

Freqs. of fixation of A1 for In and St= 0.949568868 0.949568868

Freqs. of fixation of A2 for In and St= 2.75543984E-02 2.75543984E-02

Freqs. of segregation for In and St= 2.28767339E-02 2.28767339E-02

q1f= 2.81995106E-02 q2f= 2.81995106E-02

Index of gamma value 17 gamma for St popn= 5.55666685

Means of q1 and q2= 2.60358825E-02 2.60358825E-02

Variances of q1 and q2= 2.26501469E-02 2.26501469E-02

Covariance and correlation of q1 and q2= -5.45452582E-04 -2.40816362E-02

F1 and F2= 0.893214464 0.893214464

Freqs. of fixation of A1 for In and St= 0.956016541 0.956016541

Freqs. of fixation of A2 for In and St= 2.17450410E-02 2.17450410E-02

Freqs. of segregation for In and St= 2.22384185E-02 2.22384185E-02

q1f= 2.22396161E-02 q2f= 2.22396161E-02

Index of gamma value 18 gamma for St popn= 5.88833332

Means of q1 and q2= 2.11057756E-02 2.11057775E-02

Variances of q1 and q2= 1.80647243E-02 1.80647243E-02

Covariance and correlation of q1 and q2= -3.53290059E-04 -1.95569023E-02

F1 and F2= 0.874368012 0.874367893

Freqs. of fixation of A1 for In and St= 0.961257637 0.961257637

Freqs. of fixation of A2 for In and St= 1.71189029E-02 1.71189029E-02

Freqs. of segregation for In and St= 2.16234606E-02 2.16234606E-02

q1f= 1.74972527E-02 q2f= 1.74972527E-02

Index of gamma value 19 gamma for St popn= 6.22000027

Means of q1 and q2= 1.71699412E-02 1.71699412E-02

Variances of q1 and q2= 1.43859005E-02 1.43858986E-02

Covariance and correlation of q1 and q2= -2.28970574E-04 -1.59163196E-02

F1 and F2= 0.852491021 0.852490902

Freqs. of fixation of A1 for In and St= 0.965515554 0.965515554

Freqs. of fixation of A2 for In and St= 1.34504959E-02 1.34504959E-02

Freqs. of segregation for In and St= 2.10339502E-02 2.10339502E-02

q1f= 1.37394918E-02 q2f= 1.37394918E-02

Index of gamma value 20 gamma for St popn= 6.55166674

Means of q1 and q2= 1.40336407E-02 1.40336398E-02

Variances of q1 and q2= 1.14478841E-02 1.14478841E-02

Covariance and correlation of q1 and q2= -1.48802908E-04 -1.29982894E-02

F1 and F2= 0.827356696 0.827356756

Freqs. of fixation of A1 for In and St= 0.968977749 0.968977749

Freqs. of fixation of A2 for In and St= 1.05514033E-02 1.05514023E-02

Freqs. of segregation for In and St= 2.04708464E-02 2.04708483E-02

q1f= 1.07719135E-02 q2f= 1.07719125E-02

Index of gamma value 21 gamma for St popn= 6.88333368

Means of q1 and q2= 1.15372790E-02 1.15372790E-02

Variances of q1 and q2= 9.10995714E-03 9.10995714E-03

Covariance and correlation of q1 and q2= -9.71665868E-05 -1.06659764E-02

F1 and F2= 0.798826814 0.798826814

Freqs. of fixation of A1 for In and St= 0.971799970 0.971799970

Freqs. of fixation of A2 for In and St= 8.26670136E-03 8.26670136E-03

Freqs. of segregation for In and St= 1.99333280E-02 1.99333280E-02

q1f= 8.43483582E-03 q2f= 8.43483582E-03

Index of gamma value 22 gamma for St popn= 7.21500015

Means of q1 and q2= 9.55097284E-03 9.55097284E-03

Variances of q1 and q2= 7.25451624E-03 7.25451624E-03

Covariance and correlation of q1 and q2= -6.38775309E-05 -8.80520884E-03

F1 and F2= 0.766882300 0.766882300

Freqs. of fixation of A1 for In and St= 0.974108040 0.974108040

Freqs. of fixation of A2 for In and St= 6.47011213E-03 6.47011260E-03

Freqs. of segregation for In and St= 1.94218475E-02 1.94218475E-02

q1f= 6.59826258E-03 q2f= 6.59826305E-03

Index of gamma value 23 gamma for St popn= 7.54666710

Means of q1 and q2= 7.97000062E-03 7.97000062E-03

Variances of q1 and q2= 5.78486640E-03 5.78486640E-03

Covariance and correlation of q1 and q2= -4.23579258E-05 -7.32219592E-03

F1 and F2= 0.731661439 0.731661439

Freqs. of fixation of A1 for In and St= 0.976004958 0.976004958

Freqs. of fixation of A2 for In and St= 5.05986065E-03 5.05986065E-03

Freqs. of segregation for In and St= 1.89351812E-02 1.89351812E-02

q1f= 5.15751913E-03 q2f= 5.15751913E-03

Index of gamma value 24 gamma for St popn= 7.87833357

Means of q1 and q2= 6.71043759E-03 6.71043806E-03

Variances of q1 and q2= 4.62235883E-03 4.62235883E-03

Covariance and correlation of q1 and q2= -2.83842946E-05 -6.14065165E-03

F1 and F2= 0.693484783 0.693484724

Freqs. of fixation of A1 for In and St= 0.977573276 0.977573276

Freqs. of fixation of A2 for In and St= 3.95446597E-03 3.95446597E-03

Freqs. of segregation for In and St= 1.84722580E-02 1.84722580E-02

q1f= 4.02888842E-03 q2f= 4.02888842E-03

Index of gamma value 25 gamma for St popn= 8.21000004

Means of q1 and q2= 5.70526021E-03 5.70526021E-03

Variances of q1 and q2= 3.70347989E-03 3.70348035E-03

Covariance and correlation of q1 and q2= -1.92540501E-05 -5.19890757E-03

F1 and F2= 0.652858973 0.652859032

Freqs. of fixation of A1 for In and St= 0.978879392 0.978879392

Freqs. of fixation of A2 for In and St= 3.08898091E-03 3.08898091E-03

Freqs. of segregation for In and St= 1.80316269E-02 1.80316269E-02

q1f= 3.14570288E-03 q2f= 3.14570288E-03

Index of gamma value 26 gamma for St popn= 8.54166698

Means of q1 and q2= 4.90124105E-03 4.90124151E-03

Variances of q1 and q2= 2.97738006E-03 2.97738006E-03

Covariance and correlation of q1 and q2= -1.32420046E-05 -4.44753608E-03

F1 and F2= 0.610466778 0.610466719

Freqs. of fixation of A1 for In and St= 0.979975402 0.979975522

Freqs. of fixation of A2 for In and St= 2.41196062E-03 2.41196062E-03

Freqs. of segregation for In and St= 1.76126361E-02 1.76125169E-02

q1f= 2.45520333E-03 q2f= 2.45520286E-03

Index of gamma value 27 gamma for St popn= 8.87333393

Means of q1 and q2= 4.25614836E-03 4.25614882E-03

Variances of q1 and q2= 2.40346533E-03 2.40346510E-03

Covariance and correlation of q1 and q2= -9.24608503E-06 -3.84698086E-03

F1 and F2= 0.567118049 0.567117929

Freqs. of fixation of A1 for In and St= 0.980904698 0.980904698

Freqs. of fixation of A2 for In and St= 1.88269990E-03 1.88269990E-03

Freqs. of segregation for In and St= 1.72126014E-02 1.72126014E-02

q1f= 1.91567373E-03 q2f= 1.91567373E-03

Index of gamma value 28 gamma for St popn= 9.20499992

Means of q1 and q2= 3.73667199E-03 3.73667222E-03

Variances of q1 and q2= 1.94958772E-03 1.94958772E-03

Covariance and correlation of q1 and q2= -6.56209068E-06 -3.36588640E-03

F1 and F2= 0.523701310 0.523701251

Freqs. of fixation of A1 for In and St= 0.981699049 0.981699049

Freqs. of fixation of A2 for In and St= 1.46920735E-03 1.46920735E-03

Freqs. of segregation for In and St= 1.68317426E-02 1.68317426E-02

q1f= 1.49436004E-03 q2f= 1.49436004E-03

Index of gamma value 29 gamma for St popn= 9.53666687

Means of q1 and q2= 3.31649557E-03 3.31649533E-03

Variances of q1 and q2= 1.59027800E-03 1.59027812E-03

Covariance and correlation of q1 and q2= -4.73799264E-06 -2.97934841E-03

F1 and F2= 0.481101125 0.481101185

Freqs. of fixation of A1 for In and St= 0.982385516 0.982385516

Freqs. of fixation of A2 for In and St= 1.14630861E-03 1.14630861E-03

Freqs. of segregation for In and St= 1.64681748E-02 1.64681748E-02

q1f= 1.16550236E-03 q2f= 1.16550236E-03

Index of gamma value 30 gamma for St popn= 9.86833382

Means of q1 and q2= 2.97485804E-03 2.97485804E-03

Variances of q1 and q2= 1.30541308E-03 1.30541320E-03

Covariance and correlation of q1 and q2= -3.48233789E-06 -2.66761356E-03

F1 and F2= 0.440124571 0.440124631

Freqs. of fixation of A1 for In and St= 0.982985020 0.982985020

Freqs. of fixation of A2 for In and St= 8.94230558E-04 8.94230558E-04

Freqs. of segregation for In and St= 1.61207505E-02 1.61207505E-02

q1f= 9.08882415E-04 q2f= 9.08882415E-04

Index of gamma value 31 gamma for St popn= 10.2000008

Means of q1 and q2= 2.69542309E-03 2.69542285E-03

Variances of q1 and q2= 1.07915164E-03 1.07915152E-03

Covariance and correlation of q1 and q2= -2.60638035E-06 -2.41521234E-03

F1 and F2= 0.401446521 0.401446521

Freqs. of fixation of A1 for In and St= 0.983514071 0.983514071

Freqs. of fixation of A2 for In and St= 6.97493844E-04 6.97493844E-04

Freqs. of segregation for In and St= 1.57884359E-02 1.57884359E-02

q1f= 7.08682812E-04 q2f= 7.08682812E-04

Index of gamma value 32 gamma for St popn= 10.5316668

Means of q1 and q2= 2.46533612E-03 2.46533612E-03

Variances of q1 and q2= 8.99028266E-04 8.99028266E-04

Covariance and correlation of q1 and q2= -1.98661292E-06 -2.20973347E-03

F1 and F2= 0.365568876 0.365568876

Freqs. of fixation of A1 for In and St= 0.983985662 0.983985662

Freqs. of fixation of A2 for In and St= 5.43984002E-04 5.43984002E-04

Freqs. of segregation for In and St= 1.54703539E-02 1.54703539E-02

q1f= 5.52531856E-04 q2f= 5.52531856E-04

Index of gamma value 33 gamma for St popn= 10.8633337

Means of q1 and q2= 2.27447902E-03 2.27447902E-03

Variances of q1 and q2= 7.55249697E-04 7.55249697E-04

Covariance and correlation of q1 and q2= -1.54248642E-06 -2.04235292E-03

F1 and F2= 0.332810909 0.332810909

Freqs. of fixation of A1 for In and St= 0.984410346 0.984410465

Freqs. of fixation of A2 for In and St= 4.24231635E-04 4.24231635E-04

Freqs. of segregation for In and St= 1.51654230E-02 1.51653038E-02

q1f= 4.30764369E-04 q2f= 4.30764310E-04

Index of gamma value 34 gamma for St popn= 11.1950006

Means of q1 and q2= 2.11487105E-03 2.11487105E-03

Variances of q1 and q2= 6.40104117E-04 6.40104059E-04

Covariance and correlation of q1 and q2= -1.21829521E-06 -1.90327666E-03

F1 and F2= 0.303309619 0.303309590

Freqs. of fixation of A1 for In and St= 0.984796584 0.984796584

Freqs. of fixation of A2 for In and St= 3.30816401E-04 3.30816401E-04

Freqs. of segregation for In and St= 1.48726003E-02 1.48726003E-02

q1f= 3.35810793E-04 q2f= 3.35810793E-04

Index of gamma value 35 gamma for St popn= 11.5266666

Means of q1 and q2= 1.98024069E-03 1.98024069E-03

Variances of q1 and q2= 5.47550328E-04 5.47550211E-04

Covariance and correlation of q1 and q2= -9.78961452E-07 -1.78789324E-03

F1 and F2= 0.277055591 0.277055532

Freqs. of fixation of A1 for In and St= 0.985150397 0.985150397

Freqs. of fixation of A2 for In and St= 2.57960841E-04 2.57960841E-04

Freqs. of segregation for In and St= 1.45916427E-02 1.45916427E-02

q1f= 2.61780660E-04 q2f= 2.61780660E-04

Index of gamma value 36 gamma for St popn= 11.8583336

Means of q1 and q2= 1.86563702E-03 1.86563702E-03

Variances of q1 and q2= 4.72833868E-04 4.72833868E-04

Covariance and correlation of q1 and q2= -7.99739837E-07 -1.69137598E-03

F1 and F2= 0.253917366 0.253917366

Freqs. of fixation of A1 for In and St= 0.985477686 0.985477686

Freqs. of fixation of A2 for In and St= 2.01142509E-04 2.01142509E-04

Freqs. of segregation for In and St= 1.43211717E-02 1.43211717E-02

q1f= 2.04064956E-04 q2f= 2.04064956E-04

Index of gamma value 37 gamma for St popn= 12.1900005

Means of q1 and q2= 1.76714943E-03 1.76714920E-03

Variances of q1 and q2= 4.12221969E-04 4.12221969E-04

Covariance and correlation of q1 and q2= -6.63629180E-07 -1.60988315E-03

F1 and F2= 0.233682394 0.233682439

Freqs. of fixation of A1 for In and St= 0.985782146 0.985782146

Freqs. of fixation of A2 for In and St= 1.56832670E-04 1.56832655E-04

Freqs. of segregation for In and St= 1.40610207E-02 1.40610207E-02

q1f= 1.59069343E-04 q2f= 1.59069328E-04

Index of gamma value 38 gamma for St popn= 12.5216675

Means of q1 and q2= 1.68169988E-03 1.68169988E-03

Variances of q1 and q2= 3.62786697E-04 3.62786697E-04

Covariance and correlation of q1 and q2= -5.58861757E-07 -1.54046924E-03

F1 and F2= 0.216089576 0.216089576

Freqs. of fixation of A1 for In and St= 0.986067533 0.986067533

Freqs. of fixation of A2 for In and St= 1.22281024E-04 1.22281024E-04

Freqs. of segregation for In and St= 1.38101857E-02 1.38101857E-02

q1f= 1.23993392E-04 q2f= 1.23993392E-04

Index of gamma value 39 gamma for St popn= 12.8533335

Means of q1 and q2= 1.60684332E-03 1.60684320E-03

Variances of q1 and q2= 3.22224310E-04 3.22224310E-04

Covariance and correlation of q1 and q2= -4.77100457E-07 -1.48064701E-03

F1 and F2= 0.200855240 0.200855255

Freqs. of fixation of A1 for In and St= 0.986335933 0.986335933

Freqs. of fixation of A2 for In and St= 9.53410490E-05 9.53410490E-05

Freqs. of segregation for In and St= 1.35687254E-02 1.35687254E-02

q1f= 9.66524967E-05 q2f= 9.66524967E-05

Index of gamma value 40 gamma for St popn= 13.1850004

Means of q1 and q2= 1.54064654E-03 1.54064654E-03

Variances of q1 and q2= 2.88720563E-04 2.88720563E-04

Covariance and correlation of q1 and q2= -4.12467671E-07 -1.42860506E-03

F1 and F2= 0.187691376 0.187691376

Freqs. of fixation of A1 for In and St= 0.986590445 0.986590445

Freqs. of fixation of A2 for In and St= 7.43342171E-05 7.43342171E-05

Freqs. of segregation for In and St= 1.33352205E-02 1.33352205E-02

q1f= 7.53388813E-05 q2f= 7.53388813E-05

Index of gamma value 41 gamma for St popn= 13.5166674

Means of q1 and q2= 1.48157042E-03 1.48157042E-03

Variances of q1 and q2= 2.60849862E-04 2.60849862E-04

Covariance and correlation of q1 and q2= -3.60704576E-07 -1.38280529E-03

F1 and F2= 0.176324338 0.176324338

Freqs. of fixation of A1 for In and St= 0.986832261 0.986832380

Freqs. of fixation of A2 for In and St= 5.79552179E-05 5.79552179E-05

Freqs. of segregation for In and St= 1.31097836E-02 1.31096644E-02

q1f= 5.87250906E-05 q2f= 5.87250870E-05

Index of gamma value 42 gamma for St popn= 13.8483334

Means of q1 and q2= 1.42839144E-03 1.42839144E-03

Variances of q1 and q2= 2.37488304E-04 2.37488304E-04

Covariance and correlation of q1 and q2= -3.18724801E-07 -1.34206528E-03

F1 and F2= 0.166500583 0.166500583

Freqs. of fixation of A1 for In and St= 0.987062931 0.987062931

Freqs. of fixation of A2 for In and St= 4.51846172E-05 4.51846172E-05

Freqs. of segregation for In and St= 1.28918840E-02 1.28918840E-02

q1f= 4.57747410E-05 q2f= 4.57747410E-05

Index of gamma value 43 gamma for St popn= 14.1800003

Means of q1 and q2= 1.38013251E-03 1.38013239E-03

Variances of q1 and q2= 2.17750057E-04 2.17750072E-04

Covariance and correlation of q1 and q2= -2.84263592E-07 -1.30545814E-03

F1 and F2= 0.157992795 0.157992825

Freqs. of fixation of A1 for In and St= 0.987284064 0.987284064

Freqs. of fixation of A2 for In and St= 3.52282223E-05 3.52282223E-05

Freqs. of segregation for In and St= 1.26807075E-02 1.26807075E-02

q1f= 3.56806777E-05 q2f= 3.56806777E-05

Index of gamma value 44 gamma for St popn= 14.5116673

Means of q1 and q2= 1.33600796E-03 1.33600796E-03

Variances of q1 and q2= 2.00934082E-04 2.00934111E-04

Covariance and correlation of q1 and q2= -2.55625082E-07 -1.27218373E-03

F1 and F2= 0.150600061 0.150600091

Freqs. of fixation of A1 for In and St= 0.987495840 0.987495840

Freqs. of fixation of A2 for In and St= 2.74658032E-05 2.74658050E-05

Freqs. of segregation for In and St= 1.24766948E-02 1.24766948E-02

q1f= 2.78128155E-05 q2f= 2.78128173E-05

Index of gamma value 45 gamma for St popn= 14.8433342

Means of q1 and q2= 1.29538856E-03 1.29538868E-03

Variances of q1 and q2= 1.86485166E-04 1.86485166E-04

Covariance and correlation of q1 and q2= -2.31566219E-07 -1.24174065E-03

F1 and F2= 0.144147515 0.144147500

Freqs. of fixation of A1 for In and St= 0.987699449 0.987699568

Freqs. of fixation of A2 for In and St= 2.14132051E-05 2.14132051E-05

Freqs. of segregation for In and St= 1.22791380E-02 1.22790188E-02

q1f= 2.16794106E-05 q2f= 2.16794069E-05

Index of gamma value 46 gamma for St popn= 15.1750002

Means of q1 and q2= 1.25776604E-03 1.25776604E-03

Variances of q1 and q2= 1.73965062E-04 1.73965062E-04

Covariance and correlation of q1 and q2= -2.11122938E-07 -1.21359387E-03

F1 and F2= 0.138486922 0.138486922

Freqs. of fixation of A1 for In and St= 0.987896442 0.987896442

Freqs. of fixation of A2 for In and St= 1.66947975E-05 1.66947975E-05

Freqs. of segregation for In and St= 1.20868627E-02 1.20868627E-02

q1f= 1.68990537E-05 q2f= 1.68990537E-05

Index of gamma value 47 gamma for St popn= 15.5066671

Means of q1 and q2= 1.22272549E-03 1.22272549E-03

Variances of q1 and q2= 1.63023666E-04 1.63023666E-04

Covariance and correlation of q1 and q2= -1.93572532E-07 -1.18738913E-03

F1 and F2= 0.133491322 0.133491322

Freqs. of fixation of A1 for In and St= 0.988086045 0.988086045

Freqs. of fixation of A2 for In and St= 1.30159715E-05 1.30159697E-05

Freqs. of segregation for In and St= 1.19009390E-02 1.19009390E-02

q1f= 1.31727402E-05 q2f= 1.31727384E-05

Index of gamma value 48 gamma for St popn= 15.8383341

Means of q1 and q2= 1.18992955E-03 1.18992967E-03

Variances of q1 and q2= 1.53383284E-04 1.53383284E-04

Covariance and correlation of q1 and q2= -1.78357823E-07 -1.16282434E-03

F1 and F2= 0.129054710 0.129054710

Freqs. of fixation of A1 for In and St= 0.988269806 0.988269806

Freqs. of fixation of A2 for In and St= 1.01479682E-05 1.01479682E-05

Freqs. of segregation for In and St= 1.17200464E-02 1.17200464E-02

q1f= 1.02683134E-05 q2f= 1.02683134E-05

Index of gamma value 49 gamma for St popn= 16.1700001

Means of q1 and q2= 1.15910266E-03 1.15910254E-03

Variances of q1 and q2= 1.44821330E-04 1.44821344E-04

Covariance and correlation of q1 and q2= -1.65048164E-07 -1.13966747E-03

F1 and F2= 0.125087619 0.125087649

Freqs. of fixation of A1 for In and St= 0.988447368 0.988447368

Freqs. of fixation of A2 for In and St= 0.00000000 0.00000000

Freqs. of segregation for In and St= 1.15526319E-02 1.15526319E-02

q1f= 0.00000000 q2f= 0.00000000

Index of gamma value 50 gamma for St popn= 16.5016670

Means of q1 and q2= 1.13001524E-03 1.13001524E-03

Variances of q1 and q2= 1.37159208E-04 1.37159208E-04

Covariance and correlation of q1 and q2= -1.53303631E-07 -1.11770572E-03

F1 and F2= 0.121515512 0.121515512

Freqs. of fixation of A1 for In and St= 0.988619447 0.988619447

Freqs. of fixation of A2 for In and St= 0.00000000 0.00000000

Freqs. of segregation for In and St= 1.13805532E-02 1.13805532E-02

q1f= 0.00000000 q2f= 0.00000000

Index of gamma value 51 gamma for St popn= 16.8333340

Means of q1 and q2= 1.10247952E-03 1.10247941E-03

Variances of q1 and q2= 1.30254368E-04 1.30254368E-04

Covariance and correlation of q1 and q2= -1.42872636E-07 -1.09687401E-03

F1 and F2= 0.118277147 0.118277162

Freqs. of fixation of A1 for In and St= 0.988786578 0.988786578

Freqs. of fixation of A2 for In and St= 0.00000000 0.00000000

Freqs. of segregation for In and St= 1.12134218E-02 1.12134218E-02

q1f= 0.00000000 q2f= 0.00000000

Index of gamma value 52 gamma for St popn= 17.1650009

Means of q1 and q2= 1.07633497E-03 1.07633497E-03

Variances of q1 and q2= 1.23990947E-04 1.23990947E-04

Covariance and correlation of q1 and q2= -1.33532467E-07 -1.07695337E-03

F1 and F2= 0.115321480 0.115321480

Freqs. of fixation of A1 for In and St= 0.988948822 0.988948822

Freqs. of fixation of A2 for In and St= 0.00000000 0.00000000

Freqs. of segregation for In and St= 1.10511780E-02 1.10511780E-02

q1f= 0.00000000 q2f= 0.00000000

Index of gamma value 53 gamma for St popn= 17.4966679

Means of q1 and q2= 1.05144863E-03 1.05144852E-03

Variances of q1 and q2= 1.18274744E-04 1.18274744E-04

Covariance and correlation of q1 and q2= -1.25122824E-07 -1.05789979E-03

F1 and F2= 0.112605810 0.112605825

Freqs. of fixation of A1 for In and St= 0.989106596 0.989106596

Freqs. of fixation of A2 for In and St= 0.00000000 0.00000000

Freqs. of segregation for In and St= 1.08934045E-02 1.08934045E-02

q1f= 0.00000000 q2f= 0.00000000

Index of gamma value 54 gamma for St popn= 17.8283348

Means of q1 and q2= 1.02770596E-03 1.02770596E-03

Variances of q1 and q2= 1.13029433E-04 1.13029433E-04

Covariance and correlation of q1 and q2= -1.17504044E-07 -1.03958801E-03

F1 and F2= 0.110095404 0.110095404

Freqs. of fixation of A1 for In and St= 0.989260316 0.989260375

Freqs. of fixation of A2 for In and St= 0.00000000 0.00000000

Freqs. of segregation for In and St= 1.07396841E-02 1.07396245E-02

q1f= 0.00000000 q2f= 0.00000000

Index of gamma value 55 gamma for St popn= 18.1599998

Means of q1 and q2= 1.00501080E-03 1.00501080E-03

Variances of q1 and q2= 1.08192500E-04 1.08192500E-04

Covariance and correlation of q1 and q2= -1.10570113E-07 -1.02197577E-03

F1 and F2= 0.107761368 0.107761368

Freqs. of fixation of A1 for In and St= 0.989409626 0.989409566

Freqs. of fixation of A2 for In and St= 0.00000000 0.00000000

Freqs. of segregation for In and St= 1.05903745E-02 1.05904341E-02

q1f= 0.00000000 q2f= 0.00000000

Index of gamma value 56 gamma for St popn= 18.4916668

Means of q1 and q2= 9.83277685E-04 9.83277685E-04

Variances of q1 and q2= 1.03712046E-04 1.03712038E-04

Covariance and correlation of q1 and q2= -1.04232015E-07 -1.00501359E-03

F1 and F2= 0.105579659 0.105579652

Freqs. of fixation of A1 for In and St= 0.989555120 0.989555120

Freqs. of fixation of A2 for In and St= 0.00000000 0.00000000

Freqs. of segregation for In and St= 1.04448795E-02 1.04448795E-02

q1f= 0.00000000 q2f= 0.00000000

Index of gamma value 57 gamma for St popn= 18.8233337

Means of q1 and q2= 9.62435093E-04 9.62435093E-04

Variances of q1 and q2= 9.95452938E-05 9.95453083E-05

Covariance and correlation of q1 and q2= -9.84147732E-08 -9.88643034E-04

F1 and F2= 0.103530295 0.103530310

Freqs. of fixation of A1 for In and St= 0.989696801 0.989696801

Freqs. of fixation of A2 for In and St= 0.00000000 0.00000000

Freqs. of segregation for In and St= 1.03031993E-02 1.03031993E-02

q1f= 0.00000000 q2f= 0.00000000

Index of gamma value 58 gamma for St popn= 19.1550007

Means of q1 and q2= 9.42418585E-04 9.42418643E-04

Variances of q1 and q2= 9.56566364E-05 9.56566510E-05

Covariance and correlation of q1 and q2= -9.30561441E-08 -9.72814218E-04

F1 and F2= 0.101596966 0.101596974

Freqs. of fixation of A1 for In and St= 0.989835203 0.989835143

Freqs. of fixation of A2 for In and St= 0.00000000 0.00000000

Freqs. of segregation for In and St= 1.01647973E-02 1.01648569E-02

q1f= 0.00000000 q2f= 0.00000000

Index of gamma value 59 gamma for St popn= 19.4866676

Means of q1 and q2= 9.23172047E-04 9.23171930E-04

Variances of q1 and q2= 9.20159655E-05 9.20159582E-05

Covariance and correlation of q1 and q2= -8.81058213E-08 -9.57505836E-04

F1 and F2= 9.97657925E-02 9.97657925E-02

Freqs. of fixation of A1 for In and St= 0.989969909 0.989969909

Freqs. of fixation of A2 for In and St= 0.00000000 0.00000000

Freqs. of segregation for In and St= 1.00300908E-02 1.00300908E-02

q1f= 0.00000000 q2f= 0.00000000

Index of gamma value 60 gamma for St popn= 19.8183346

Means of q1 and q2= 9.04644898E-04 9.04644898E-04

Variances of q1 and q2= 8.85978807E-05 8.85978807E-05

Covariance and correlation of q1 and q2= -8.35189553E-08 -9.42674407E-04

F1 and F2= 9.80253145E-02 9.80253145E-02

Freqs. of fixation of A1 for In and St= 0.990101457 0.990101457

Freqs. of fixation of A2 for In and St= 0.00000000 0.00000000

Freqs. of segregation for In and St= 9.89854336E-03 9.89854336E-03

q1f= 0.00000000 q2f= 0.00000000

Index of gamma value 61 gamma for St popn= 20.1500015

Means of q1 and q2= 8.86793539E-04 8.86793539E-04

Variances of q1 and q2= 8.53808597E-05 8.53808597E-05

Covariance and correlation of q1 and q2= -7.92593937E-08 -9.28303984E-04

F1 and F2= 9.63658839E-02 9.63658839E-02

Freqs. of fixation of A1 for In and St= 0.990229845 0.990229905

Freqs. of fixation of A2 for In and St= 0.00000000 0.00000000

Freqs. of segregation for In and St= 9.77015495E-03 9.77009535E-03

q1f= 0.00000000 q2f= 0.00000000

Index of gamma value 62 gamma for St popn= 20.4816666

Means of q1 and q2= 8.69576121E-04 8.69576121E-04

Variances of q1 and q2= 8.23462178E-05 8.23462105E-05

Covariance and correlation of q1 and q2= -7.52927463E-08 -9.14343807E-04

F1 and F2= 9.47793797E-02 9.47793722E-02

Freqs. of fixation of A1 for In and St= 0.990355134 0.990355134

Freqs. of fixation of A2 for In and St= 0.00000000 0.00000000

Freqs. of segregation for In and St= 9.64486599E-03 9.64486599E-03

q1f= 0.00000000 q2f= 0.00000000

Index of gamma value 63 gamma for St popn= 20.8133335

Means of q1 and q2= 8.52957484E-04 8.52957484E-04

Variances of q1 and q2= 7.94780353E-05 7.94780281E-05

Covariance and correlation of q1 and q2= -7.15941724E-08 -9.00804589E-04

F1 and F2= 9.32589099E-02 9.32589024E-02

Freqs. of fixation of A1 for In and St= 0.990477622 0.990477622

Freqs. of fixation of A2 for In and St= 0.00000000 0.00000000

Freqs. of segregation for In and St= 9.52237844E-03 9.52237844E-03

q1f= 0.00000000 q2f= 0.00000000

Index of gamma value 64 gamma for St popn= 21.1450005

Means of q1 and q2= 8.36903986E-04 8.36904044E-04

Variances of q1 and q2= 7.67623351E-05 7.67623351E-05

Covariance and correlation of q1 and q2= -6.81364440E-08 -8.87628586E-04

F1 and F2= 9.17986184E-02 9.17986184E-02

Freqs. of fixation of A1 for In and St= 0.990597427 0.990597427

Freqs. of fixation of A2 for In and St= 0.00000000 0.00000000

Freqs. of segregation for In and St= 9.40257311E-03 9.40257311E-03

q1f= 0.00000000 q2f= 0.00000000

Index of gamma value 65 gamma for St popn= 21.4766674

Means of q1 and q2= 8.21385533E-04 8.21385533E-04

Variances of q1 and q2= 7.41869226E-05 7.41869226E-05

Covariance and correlation of q1 and q2= -6.49017693E-08 -8.74841120E-04

F1 and F2= 9.03934985E-02 9.03934985E-02

Freqs. of fixation of A1 for In and St= 0.990714550 0.990714550

Freqs. of fixation of A2 for In and St= 0.00000000 0.00000000

Freqs. of segregation for In and St= 9.28544998E-03 9.28544998E-03

q1f= 0.00000000 q2f= 0.00000000

Index of gamma value 66 gamma for St popn= 21.8083344

Means of q1 and q2= 8.06374184E-04 8.06374184E-04

Variances of q1 and q2= 7.17410585E-05 7.17410585E-05

Covariance and correlation of q1 and q2= -6.18688887E-08 -8.62391607E-04

F1 and F2= 8.90392512E-02 8.90392512E-02

Freqs. of fixation of A1 for In and St= 0.990828753 0.990828693

Freqs. of fixation of A2 for In and St= 0.00000000 0.00000000

Freqs. of segregation for In and St= 9.17124748E-03 9.17130709E-03

q1f= 0.00000000 q2f= 0.00000000

Index of gamma value 67 gamma for St popn= 22.1400013

Means of q1 and q2= 7.91844504E-04 7.91844504E-04

Variances of q1 and q2= 6.94152113E-05 6.94152113E-05

Covariance and correlation of q1 and q2= -5.90225113E-08 -8.50282086E-04

F1 and F2= 8.77321512E-02 8.77321512E-02

Freqs. of fixation of A1 for In and St= 0.990940809 0.990940809

Freqs. of fixation of A2 for In and St= 0.00000000 0.00000000

Freqs. of segregation for In and St= 9.05919075E-03 9.05919075E-03

q1f= 0.00000000 q2f= 0.00000000

Index of gamma value 68 gamma for St popn= 22.4716682

Means of q1 and q2= 7.77772570E-04 7.77772570E-04

Variances of q1 and q2= 6.72008246E-05 6.72008246E-05

Covariance and correlation of q1 and q2= -5.63460389E-08 -8.38472450E-04

F1 and F2= 8.64688978E-02 8.64688978E-02

Freqs. of fixation of A1 for In and St= 0.991050184 0.991050184

Freqs. of fixation of A2 for In and St= 0.00000000 0.00000000

Freqs. of segregation for In and St= 8.94981623E-03 8.94981623E-03

q1f= 0.00000000 q2f= 0.00000000

Index of gamma value 69 gamma for St popn= 22.8033333

Means of q1 and q2= 7.64137367E-04 7.64137367E-04

Variances of q1 and q2= 6.50903530E-05 6.50903530E-05

Covariance and correlation of q1 and q2= -5.38288987E-08 -8.26987380E-04

F1 and F2= 8.52466226E-02 8.52466226E-02

Freqs. of fixation of A1 for In and St= 0.991157293 0.991157293

Freqs. of fixation of A2 for In and St= 0.00000000 0.00000000

Freqs. of segregation for In and St= 8.84270668E-03 8.84270668E-03

q1f= 0.00000000 q2f= 0.00000000

Index of gamma value 70 gamma for St popn= 23.1350002

Means of q1 and q2= 7.50916952E-04 7.50916894E-04

Variances of q1 and q2= 6.30767754E-05 6.30767827E-05

Covariance and correlation of q1 and q2= -5.14565954E-08 -8.15777224E-04

F1 and F2= 8.40627924E-02 8.40628073E-02

Freqs. of fixation of A1 for In and St= 0.991262376 0.991262376

Freqs. of fixation of A2 for In and St= 0.00000000 0.00000000

Freqs. of segregation for In and St= 8.73762369E-03 8.73762369E-03

q1f= 0.00000000 q2f= 0.00000000

Index of gamma value 71 gamma for St popn= 23.4666672

Means of q1 and q2= 7.38093804E-04 7.38093921E-04

Variances of q1 and q2= 6.11540163E-05 6.11540163E-05

Covariance and correlation of q1 and q2= -4.92206027E-08 -8.04862939E-04

F1 and F2= 8.29151869E-02 8.29151720E-02

Freqs. of fixation of A1 for In and St= 0.991364896 0.991364956

Freqs. of fixation of A2 for In and St= 0.00000000 0.00000000

Freqs. of segregation for In and St= 8.63510370E-03 8.63504410E-03

q1f= 0.00000000 q2f= 0.00000000

Index of gamma value 72 gamma for St popn= 23.7983341

Means of q1 and q2= 7.25649239E-04 7.25649239E-04

Variances of q1 and q2= 5.93162949E-05 5.93162986E-05

Covariance and correlation of q1 and q2= -4.71097792E-08 -7.94213091E-04

F1 and F2= 8.18017349E-02 8.18017423E-02

Freqs. of fixation of A1 for In and St= 0.991465569 0.991465569

Freqs. of fixation of A2 for In and St= 0.00000000 0.00000000

Freqs. of segregation for In and St= 8.53443146E-03 8.53443146E-03

q1f= 0.00000000 q2f= 0.00000000

Index of gamma value 73 gamma for St popn= 24.1300011

Means of q1 and q2= 7.13566202E-04 7.13566202E-04

Variances of q1 and q2= 5.75584600E-05 5.75584600E-05

Covariance and correlation of q1 and q2= -4.51152289E-08 -7.83815747E-04

F1 and F2= 8.07207003E-02 8.07207003E-02

Freqs. of fixation of A1 for In and St= 0.991564155 0.991564155

Freqs. of fixation of A2 for In and St= 0.00000000 0.00000000

Freqs. of segregation for In and St= 8.43584538E-03 8.43584538E-03

q1f= 0.00000000 q2f= 0.00000000

Index of gamma value 74 gamma for St popn= 24.4616680

Means of q1 and q2= 7.01830257E-04 7.01830257E-04

Variances of q1 and q2= 5.58758147E-05 5.58758220E-05

Covariance and correlation of q1 and q2= -4.32299316E-08 -7.73678708E-04

F1 and F2= 7.96703398E-02 7.96703547E-02

Freqs. of fixation of A1 for In and St= 0.991660774 0.991660774

Freqs. of fixation of A2 for In and St= 0.00000000 0.00000000

Freqs. of segregation for In and St= 8.33922625E-03 8.33922625E-03

q1f= 0.00000000 q2f= 0.00000000

Index of gamma value 75 gamma for St popn= 24.7933350

Means of q1 and q2= 6.90425339E-04 6.90425339E-04

Variances of q1 and q2= 5.42638991E-05 5.42638954E-05

Covariance and correlation of q1 and q2= -4.14449630E-08 -7.63766759E-04

F1 and F2= 7.86491856E-02 7.86491781E-02

Freqs. of fixation of A1 for In and St= 0.991755307 0.991755307

Freqs. of fixation of A2 for In and St= 0.00000000 0.00000000

Freqs. of segregation for In and St= 8.24469328E-03 8.24469328E-03

q1f= 0.00000000 q2f= 0.00000000

Index of gamma value 76 gamma for St popn= 25.1250000

Means of q1 and q2= 6.79338991E-04 6.79338933E-04

Variances of q1 and q2= 5.27188240E-05 5.27188240E-05

Covariance and correlation of q1 and q2= -3.97549798E-08 -7.54094566E-04

F1 and F2= 7.76558667E-02 7.76558742E-02

Freqs. of fixation of A1 for In and St= 0.991847992 0.991847992

Freqs. of fixation of A2 for In and St= 0.00000000 0.00000000

Freqs. of segregation for In and St= 8.15200806E-03 8.15200806E-03

q1f= 0.00000000 q2f= 0.00000000

Index of gamma value 77 gamma for St popn= 25.4566669

Means of q1 and q2= 6.68556779E-04 6.68556895E-04

Variances of q1 and q2= 5.12367733E-05 5.12368024E-05

Covariance and correlation of q1 and q2= -3.81528196E-08 -7.44637218E-04

F1 and F2= 7.66891539E-02 7.66891837E-02

Freqs. of fixation of A1 for In and St= 0.991939187 0.991939187

Freqs. of fixation of A2 for In and St= 0.00000000 0.00000000

Freqs. of segregation for In and St= 8.06081295E-03 8.06081295E-03

q1f= 0.00000000 q2f= 0.00000000

Index of gamma value 78 gamma for St popn= 25.7883339

Means of q1 and q2= 6.58067758E-04 6.58067758E-04

Variances of q1 and q2= 4.98143709E-05 4.98144182E-05

Covariance and correlation of q1 and q2= -3.66332529E-08 -7.35394889E-04

F1 and F2= 7.57477880E-02 7.57478550E-02

Freqs. of fixation of A1 for In and St= 0.992028475 0.992028475

Freqs. of fixation of A2 for In and St= 0.00000000 0.00000000

Freqs. of segregation for In and St= 7.97152519E-03 7.97152519E-03

q1f= 0.00000000 q2f= 0.00000000

Index of gamma value 79 gamma for St popn= 26.1200008

Means of q1 and q2= 6.47859357E-04 6.47859473E-04

Variances of q1 and q2= 4.84484153E-05 4.84484517E-05

Covariance and correlation of q1 and q2= -3.51910785E-08 -7.26361526E-04

F1 and F2= 7.48307854E-02 7.48308301E-02

Freqs. of fixation of A1 for In and St= 0.992115915 0.992115915

Freqs. of fixation of A2 for In and St= 0.00000000 0.00000000

Freqs. of segregation for In and St= 7.88408518E-03 7.88408518E-03

q1f= 0.00000000 q2f= 0.00000000

Index of gamma value 80 gamma for St popn= 26.4516678

Means of q1 and q2= 6.37920515E-04 6.37920515E-04

Variances of q1 and q2= 4.71359017E-05 4.71359454E-05

Covariance and correlation of q1 and q2= -3.38209816E-08 -7.17520306E-04

F1 and F2= 7.39371032E-02 7.39371702E-02

Freqs. of fixation of A1 for In and St= 0.992201686 0.992201686

Freqs. of fixation of A2 for In and St= 0.00000000 0.00000000

Freqs. of segregation for In and St= 7.79831409E-03 7.79831409E-03

q1f= 0.00000000 q2f= 0.00000000

Index of gamma value 81 gamma for St popn= 26.7833347

Means of q1 and q2= 6.28241338E-04 6.28241338E-04

Variances of q1 and q2= 4.58740724E-05 4.58741124E-05

Covariance and correlation of q1 and q2= -3.25193525E-08 -7.08882697E-04

F1 and F2= 7.30657279E-02 7.30657950E-02

Freqs. of fixation of A1 for In and St= 0.992286146 0.992286146

Freqs. of fixation of A2 for In and St= 0.00000000 0.00000000

Freqs. of segregation for In and St= 7.71385431E-03 7.71385431E-03

q1f= 0.00000000 q2f= 0.00000000

Index of gamma value 82 gamma for St popn= 27.1150017

Means of q1 and q2= 6.18811988E-04 6.18811871E-04

Variances of q1 and q2= 4.46603444E-05 4.46603844E-05

Covariance and correlation of q1 and q2= -3.12812460E-08 -7.00425240E-04

F1 and F2= 7.22157955E-02 7.22158700E-02

Freqs. of fixation of A1 for In and St= 0.992368937 0.992368877

Freqs. of fixation of A2 for In and St= 0.00000000 0.00000000

Freqs. of segregation for In and St= 7.63106346E-03 7.63112307E-03

q1f= 0.00000000 q2f= 0.00000000

Index of gamma value 83 gamma for St popn= 27.4466667

Means of q1 and q2= 6.09621988E-04 6.09621988E-04

Variances of q1 and q2= 4.34922986E-05 4.34923277E-05

Covariance and correlation of q1 and q2= -3.01028251E-08 -6.92141301E-04

F1 and F2= 7.13865831E-02 7.13866279E-02

Freqs. of fixation of A1 for In and St= 0.992450058 0.992450058

Freqs. of fixation of A2 for In and St= 0.00000000 0.00000000

Freqs. of segregation for In and St= 7.54994154E-03 7.54994154E-03

q1f= 0.00000000 q2f= 0.00000000

Index of gamma value 84 gamma for St popn= 27.7783337

Means of q1 and q2= 6.00663305E-04 6.00663305E-04

Variances of q1 and q2= 4.23676902E-05 4.23677266E-05

Covariance and correlation of q1 and q2= -2.89805655E-08 -6.84024824E-04

F1 and F2= 7.05772340E-02 7.05772936E-02

Freqs. of fixation of A1 for In and St= 0.992529690 0.992529690

Freqs. of fixation of A2 for In and St= 0.00000000 0.00000000

Freqs. of segregation for In and St= 7.47030973E-03 7.47030973E-03

q1f= 0.00000000 q2f= 0.00000000

Index of gamma value 85 gamma for St popn= 28.1100006

Means of q1 and q2= 5.91927499E-04 5.91927499E-04

Variances of q1 and q2= 4.12837726E-05 4.12837981E-05

Covariance and correlation of q1 and q2= -2.79117103E-08 -6.76093798E-04

F1 and F2= 6.97859526E-02 6.97859973E-02

Freqs. of fixation of A1 for In and St= 0.992608130 0.992608070

Freqs. of fixation of A2 for In and St= 0.00000000 0.00000000

Freqs. of segregation for In and St= 7.39187002E-03 7.39192963E-03

q1f= 0.00000000 q2f= 0.00000000

Index of gamma value 86 gamma for St popn= 28.4416676

Means of q1 and q2= 5.83406247E-04 5.83406247E-04

Variances of q1 and q2= 4.02399310E-05 4.02399564E-05

Covariance and correlation of q1 and q2= -2.68928488E-08 -6.68312307E-04

F1 and F2= 6.90143779E-02 6.90144226E-02

Freqs. of fixation of A1 for In and St= 0.992685199 0.992685199

Freqs. of fixation of A2 for In and St= 0.00000000 0.00000000

Freqs. of segregation for In and St= 7.31480122E-03 7.31480122E-03

q1f= 0.00000000 q2f= 0.00000000

Index of gamma value 87 gamma for St popn= 28.7733345

Means of q1 and q2= 5.75092505E-04 5.75092505E-04

Variances of q1 and q2= 3.92334878E-05 3.92335096E-05

Covariance and correlation of q1 and q2= -2.59217927E-08 -6.60705613E-04

F1 and F2= 6.82604313E-02 6.82604685E-02

Freqs. of fixation of A1 for In and St= 0.992760479 0.992760539

Freqs. of fixation of A2 for In and St= 0.00000000 0.00000000

Freqs. of segregation for In and St= 7.23952055E-03 7.23946095E-03

q1f= 0.00000000 q2f= 0.00000000

Index of gamma value 88 gamma for St popn= 29.1050014

Means of q1 and q2= 5.66978590E-04 5.66978590E-04

Variances of q1 and q2= 3.82627768E-05 3.82628095E-05

Covariance and correlation of q1 and q2= -2.49949608E-08 -6.53244555E-04

F1 and F2= 6.75236881E-02 6.75237477E-02

Freqs. of fixation of A1 for In and St= 0.992834687 0.992834687

Freqs. of fixation of A2 for In and St= 0.00000000 0.00000000

Freqs. of segregation for In and St= 7.16531277E-03 7.16531277E-03

q1f= 0.00000000 q2f= 0.00000000

Index of gamma value 89 gamma for St popn= 29.4366684

Means of q1 and q2= 5.59056120E-04 5.59056120E-04

Variances of q1 and q2= 3.73260009E-05 3.73260264E-05

Covariance and correlation of q1 and q2= -2.41092266E-08 -6.45909458E-04

F1 and F2= 6.68034479E-02 6.68035001E-02

Freqs. of fixation of A1 for In and St= 0.992907703 0.992907703

Freqs. of fixation of A2 for In and St= 0.00000000 0.00000000

Freqs. of segregation for In and St= 7.09229708E-03 7.09229708E-03

q1f= 0.00000000 q2f= 0.00000000

Index of gamma value 90 gamma for St popn= 29.7683334

Means of q1 and q2= 5.51321078E-04 5.51321020E-04

Variances of q1 and q2= 3.64217594E-05 3.64217813E-05

Covariance and correlation of q1 and q2= -2.32642776E-08 -6.38746482E-04

F1 and F2= 6.60991445E-02 6.60991892E-02

Freqs. of fixation of A1 for In and St= 0.992979527 0.992979586

Freqs. of fixation of A2 for In and St= 0.00000000 0.00000000

Freqs. of segregation for In and St= 7.02047348E-03 7.02041388E-03

q1f= 0.00000000 q2f= 0.00000000

Index of gamma value 91 gamma for St popn= 30.1000004

Means of q1 and q2= 5.43765374E-04 5.43765433E-04

Variances of q1 and q2= 3.55484626E-05 3.55484844E-05

Covariance and correlation of q1 and q2= -2.24565326E-08 -6.31715811E-04

F1 and F2= 6.54102042E-02 6.54102340E-02

Freqs. of fixation of A1 for In and St= 0.993049979 0.993049979

Freqs. of fixation of A2 for In and St= 0.00000000 0.00000000

Freqs. of segregation for In and St= 6.95002079E-03 6.95002079E-03

q1f= 0.00000000 q2f= 0.00000000

Index of gamma value 92 gamma for St popn= 30.4316673

Means of q1 and q2= 5.36383130E-04 5.36383130E-04

Variances of q1 and q2= 3.47047935E-05 3.47048081E-05

Covariance and correlation of q1 and q2= -2.16840306E-08 -6.24813547E-04

F1 and F2= 6.47362247E-02 6.47362545E-02

Freqs. of fixation of A1 for In and St= 0.993119180 0.993119180

Freqs. of fixation of A2 for In and St= 0.00000000 0.00000000

Freqs. of segregation for In and St= 6.88081980E-03 6.88081980E-03

q1f= 0.00000000 q2f= 0.00000000

Index of gamma value 93 gamma for St popn= 30.7633343

Means of q1 and q2= 5.29168523E-04 5.29168581E-04

Variances of q1 and q2= 3.38894024E-05 3.38894242E-05

Covariance and correlation of q1 and q2= -2.09449240E-08 -6.18037360E-04

F1 and F2= 6.40766472E-02 6.40766844E-02

Freqs. of fixation of A1 for In and St= 0.993187308 0.993187308

Freqs. of fixation of A2 for In and St= 0.00000000 0.00000000

Freqs. of segregation for In and St= 6.81269169E-03 6.81269169E-03

q1f= 0.00000000 q2f= 0.00000000

Index of gamma value 94 gamma for St popn= 31.0950012

Means of q1 and q2= 5.22116781E-04 5.22116781E-04

Variances of q1 and q2= 3.31011106E-05 3.31011252E-05

Covariance and correlation of q1 and q2= -2.02379056E-08 -6.11396448E-04

F1 and F2= 6.34310320E-02 6.34310544E-02

Freqs. of fixation of A1 for In and St= 0.993254423 0.993254423

Freqs. of fixation of A2 for In and St= 0.00000000 0.00000000

Freqs. of segregation for In and St= 6.74557686E-03 6.74557686E-03

q1f= 0.00000000 q2f= 0.00000000

Index of gamma value 95 gamma for St popn= 31.4266682

Means of q1 and q2= 5.15221851E-04 5.15221851E-04

Variances of q1 and q2= 3.23386921E-05 3.23387103E-05

Covariance and correlation of q1 and q2= -1.95609573E-08 -6.04877656E-04

F1 and F2= 6.27988875E-02 6.27989247E-02

Freqs. of fixation of A1 for In and St= 0.993320405 0.993320346

Freqs. of fixation of A2 for In and St= 0.00000000 0.00000000

Freqs. of segregation for In and St= 6.67959452E-03 6.67965412E-03

q1f= 0.00000000 q2f= 0.00000000

Index of gamma value 96 gamma for St popn= 31.7583351

Means of q1 and q2= 5.08478843E-04 5.08478843E-04

Variances of q1 and q2= 3.16010774E-05 3.16010955E-05

Covariance and correlation of q1 and q2= -1.89122602E-08 -5.98468701E-04

F1 and F2= 6.21798821E-02 6.21799193E-02

Freqs. of fixation of A1 for In and St= 0.993385196 0.993385196

Freqs. of fixation of A2 for In and St= 0.00000000 0.00000000

Freqs. of segregation for In and St= 6.61480427E-03 6.61480427E-03

q1f= 0.00000000 q2f= 0.00000000

Index of gamma value 97 gamma for St popn= 32.0900002

Means of q1 and q2= 5.01883042E-04 5.01883100E-04

Variances of q1 and q2= 3.08872040E-05 3.08872222E-05

Covariance and correlation of q1 and q2= -1.82909190E-08 -5.92184137E-04

F1 and F2= 6.15735352E-02 6.15735613E-02

Freqs. of fixation of A1 for In and St= 0.993448675 0.993448675

Freqs. of fixation of A2 for In and St= 0.00000000 0.00000000

Freqs. of segregation for In and St= 6.55132532E-03 6.55132532E-03

q1f= 0.00000000 q2f= 0.00000000

Index of gamma value 98 gamma for St popn= 32.4216690

Means of q1 and q2= 4.95429791E-04 4.95429849E-04

Variances of q1 and q2= 3.01960736E-05 3.01960863E-05

Covariance and correlation of q1 and q2= -1.76954416E-08 -5.86017850E-04

F1 and F2= 6.09794594E-02 6.09794781E-02

Freqs. of fixation of A1 for In and St= 0.993511617 0.993511617

Freqs. of fixation of A2 for In and St= 0.00000000 0.00000000

Freqs. of segregation for In and St= 6.48838282E-03 6.48838282E-03

q1f= 0.00000000 q2f= 0.00000000

Index of gamma value 99 gamma for St popn= 32.7533340

Means of q1 and q2= 4.89114493E-04 4.89114434E-04

Variances of q1 and q2= 2.95267419E-05 2.95267564E-05

Covariance and correlation of q1 and q2= -1.71237957E-08 -5.79941785E-04

F1 and F2= 6.03972934E-02 6.03973307E-02

Freqs. of fixation of A1 for In and St= 0.993573427 0.993573546

Freqs. of fixation of A2 for In and St= 0.00000000 0.00000000

Freqs. of segregation for In and St= 6.42657280E-03 6.42645359E-03

q1f= 0.00000000 q2f= 0.00000000

Index of gamma value 100 gamma for St popn= 33.0850029

Means of q1 and q2= 4.82933305E-04 4.82933217E-04

Variances of q1 and q2= 2.88783140E-05 2.88783267E-05

Covariance and correlation of q1 and q2= -1.65761378E-08 -5.73999365E-04

F1 and F2= 5.98266162E-02 5.98266535E-02

Freqs. of fixation of A1 for In and St= 0.993634045 0.993634045

Freqs. of fixation of A2 for In and St= 0.00000000 0.00000000

Freqs. of segregation for In and St= 6.36595488E-03 6.36595488E-03

q1f= 0.00000000 q2f= 0.00000000

Index of gamma value 101 gamma for St popn= 33.4166679

Means of q1 and q2= 4.76881221E-04 4.76881221E-04

Variances of q1 and q2= 2.82499404E-05 2.82499568E-05

Covariance and correlation of q1 and q2= -1.60500520E-08 -5.68144431E-04

F1 and F2= 5.92672080E-02 5.92672415E-02

Freqs. of fixation of A1 for In and St= 0.993693829 0.993693829

Freqs. of fixation of A2 for In and St= 0.00000000 0.00000000

Freqs. of segregation for In and St= 6.30617142E-03 6.30617142E-03

q1f= 0.00000000 q2f= 0.00000000

Index of gamma value 102 gamma for St popn= 33.7483330

Means of q1 and q2= 4.70955536E-04 4.70955507E-04

Variances of q1 and q2= 2.76408482E-05 2.76408646E-05

Covariance and correlation of q1 and q2= -1.55451261E-08 -5.62396657E-04

F1 and F2= 5.87186478E-02 5.87186851E-02

Freqs. of fixation of A1 for In and St= 0.993752897 0.993752897

Freqs. of fixation of A2 for In and St= 0.00000000 0.00000000

Freqs. of segregation for In and St= 6.24710321E-03 6.24710321E-03

q1f= 0.00000000 q2f= 0.00000000

Index of gamma value 103 gamma for St popn= 34.0800018

Means of q1 and q2= 4.65151417E-04 4.65151476E-04

Variances of q1 and q2= 2.70502405E-05 2.70502569E-05

Covariance and correlation of q1 and q2= -1.50599249E-08 -5.56738873E-04

F1 and F2= 5.81806861E-02 5.81807122E-02

Freqs. of fixation of A1 for In and St= 0.993810892 0.993810892

Freqs. of fixation of A2 for In and St= 0.00000000 0.00000000

Freqs. of segregation for In and St= 6.18910789E-03 6.18910789E-03

q1f= 0.00000000 q2f= 0.00000000

Index of gamma value 104 gamma for St popn= 34.4116669

Means of q1 and q2= 4.59465664E-04 4.59465547E-04

Variances of q1 and q2= 2.64773680E-05 2.64773771E-05

Covariance and correlation of q1 and q2= -1.45937094E-08 -5.51176607E-04

F1 and F2= 5.76529242E-02 5.76529615E-02

Freqs. of fixation of A1 for In and St= 0.993867993 0.993867993

Freqs. of fixation of A2 for In and St= 0.00000000 0.00000000

Freqs. of segregation for In and St= 6.13200665E-03 6.13200665E-03

q1f= 0.00000000 q2f= 0.00000000

Index of gamma value 105 gamma for St popn= 34.7433357

Means of q1 and q2= 4.53894987E-04 4.53895016E-04

Variances of q1 and q2= 2.59215758E-05 2.59215885E-05

Covariance and correlation of q1 and q2= -1.41459680E-08 -5.45721618E-04

F1 and F2= 5.71351275E-02 5.71351498E-02

Freqs. of fixation of A1 for In and St= 0.993924022 0.993924022

Freqs. of fixation of A2 for In and St= 0.00000000 0.00000000

Freqs. of segregation for In and St= 6.07597828E-03 6.07597828E-03

q1f= 0.00000000 q2f= 0.00000000

Index of gamma value 106 gamma for St popn= 35.0750008

Means of q1 and q2= 4.48435952E-04 4.48435982E-04

Variances of q1 and q2= 2.53822054E-05 2.53822200E-05

Covariance and correlation of q1 and q2= -1.37151233E-08 -5.40343870E-04

F1 and F2= 5.66270240E-02 5.66270538E-02

Freqs. of fixation of A1 for In and St= 0.993979454 0.993979454

Freqs. of fixation of A2 for In and St= 0.00000000 0.00000000

Freqs. of segregation for In and St= 6.02054596E-03 6.02054596E-03

q1f= 0.00000000 q2f= 0.00000000

Index of gamma value 107 gamma for St popn= 35.4066696

Means of q1 and q2= 4.43085301E-04 4.43085301E-04

Variances of q1 and q2= 2.48585857E-05 2.48585966E-05

Covariance and correlation of q1 and q2= -1.33010190E-08 -5.35067287E-04

F1 and F2= 5.61282560E-02 5.61282821E-02

Freqs. of fixation of A1 for In and St= 0.994034111 0.994034052

Freqs. of fixation of A2 for In and St= 0.00000000 0.00000000

Freqs. of segregation for In and St= 5.96588850E-03 5.96594810E-03

q1f= 0.00000000 q2f= 0.00000000

Index of gamma value 108 gamma for St popn= 35.7383347

Means of q1 and q2= 4.37839684E-04 4.37839684E-04

Variances of q1 and q2= 2.43501581E-05 2.43501709E-05

Covariance and correlation of q1 and q2= -1.29023618E-08 -5.29867539E-04

F1 and F2= 5.56386858E-02 5.56387156E-02

Freqs. of fixation of A1 for In and St= 0.994087696 0.994087696

Freqs. of fixation of A2 for In and St= 0.00000000 0.00000000

Freqs. of segregation for In and St= 5.91230392E-03 5.91230392E-03

q1f= 0.00000000 q2f= 0.00000000

Index of gamma value 109 gamma for St popn= 36.0699997

Means of q1 and q2= 4.32696223E-04 4.32696223E-04

Variances of q1 and q2= 2.38563298E-05 2.38563389E-05

Covariance and correlation of q1 and q2= -1.25186546E-08 -5.24751784E-04

F1 and F2= 5.51579967E-02 5.51580191E-02

Freqs. of fixation of A1 for In and St= 0.994140863 0.994140804

Freqs. of fixation of A2 for In and St= 0.00000000 0.00000000

Freqs. of segregation for In and St= 5.85913658E-03 5.85919619E-03

q1f= 0.00000000 q2f= 0.00000000

Index of gamma value 110 gamma for St popn= 36.4016685

Means of q1 and q2= 4.27652179E-04 4.27652150E-04

Variances of q1 and q2= 2.33765622E-05 2.33765732E-05

Covariance and correlation of q1 and q2= -1.21492576E-08 -5.19719499E-04

F1 and F2= 5.46859466E-02 5.46859726E-02

Freqs. of fixation of A1 for In and St= 0.994192898 0.994192958

Freqs. of fixation of A2 for In and St= 0.00000000 0.00000000

Freqs. of segregation for In and St= 5.80710173E-03 5.80704212E-03

q1f= 0.00000000 q2f= 0.00000000

Index of gamma value 111 gamma for St popn= 36.7333336

Means of q1 and q2= 4.22705256E-04 4.22705285E-04

Variances of q1 and q2= 2.29103571E-05 2.29103662E-05

Covariance and correlation of q1 and q2= -1.17936594E-08 -5.14774001E-04

F1 and F2= 5.42222895E-02 5.42223081E-02

Freqs. of fixation of A1 for In and St= 0.994244277 0.994244277

Freqs. of fixation of A2 for In and St= 0.00000000 0.00000000

Freqs. of segregation for In and St= 5.75572252E-03 5.75572252E-03

q1f= 0.00000000 q2f= 0.00000000

Index of gamma value 112 gamma for St popn= 37.0650024

Means of q1 and q2= 4.17852105E-04 4.17852018E-04

Variances of q1 and q2= 2.24572032E-05 2.24572141E-05

Covariance and correlation of q1 and q2= -1.14510357E-08 -5.09904698E-04

F1 and F2= 5.37668467E-02 5.37668839E-02

Freqs. of fixation of A1 for In and St= 0.994294882 0.994294882

Freqs. of fixation of A2 for In and St= 0.00000000 0.00000000

Freqs. of segregation for In and St= 5.70511818E-03 5.70511818E-03

q1f= 0.00000000 q2f= 0.00000000

Index of gamma value 113 gamma for St popn= 37.3966675

Means of q1 and q2= 4.13089932E-04 4.13089932E-04

Variances of q1 and q2= 2.20165930E-05 2.20166021E-05

Covariance and correlation of q1 and q2= -1.11206901E-08 -5.05104836E-04

F1 and F2= 5.33193611E-02 5.33193834E-02

Freqs. of fixation of A1 for In and St= 0.994344711 0.994344711

Freqs. of fixation of A2 for In and St= 0.00000000 0.00000000

Freqs. of segregation for In and St= 5.65528870E-03 5.65528870E-03

q1f= 0.00000000 q2f= 0.00000000

Index of gamma value 114 gamma for St popn= 37.7283363

Means of q1 and q2= 4.08417342E-04 4.08417342E-04

Variances of q1 and q2= 2.15881355E-05 2.15881464E-05

Covariance and correlation of q1 and q2= -1.08025091E-08 -5.00390888E-04

F1 and F2= 5.28796241E-02 5.28796501E-02

Freqs. of fixation of A1 for In and St= 0.994394004 0.994393945

Freqs. of fixation of A2 for In and St= 0.00000000 0.00000000

Freqs. of segregation for In and St= 5.60599566E-03 5.60605526E-03

q1f= 0.00000000 q2f= 0.00000000

Index of gamma value 115 gamma for St popn= 38.0600014

Means of q1 and q2= 4.03830956E-04 4.03830985E-04

Variances of q1 and q2= 2.11713486E-05 2.11713632E-05

Covariance and correlation of q1 and q2= -1.04956825E-08 -4.95749176E-04

F1 and F2= 5.24474420E-02 5.24474755E-02

Freqs. of fixation of A1 for In and St= 0.994442582 0.994442582

Freqs. of fixation of A2 for In and St= 0.00000000 0.00000000

Freqs. of segregation for In and St= 5.55741787E-03 5.55741787E-03

q1f= 0.00000000 q2f= 0.00000000

Index of gamma value 116 gamma for St popn= 38.3916664

Means of q1 and q2= 3.99328885E-04 3.99328885E-04

Variances of q1 and q2= 2.07658486E-05 2.07658577E-05

Covariance and correlation of q1 and q2= -1.01995852E-08 -4.91170969E-04

F1 and F2= 5.20226434E-02 5.20226657E-02

Freqs. of fixation of A1 for In and St= 0.994490504 0.994490504

Freqs. of fixation of A2 for In and St= 0.00000000 0.00000000

Freqs. of segregation for In and St= 5.50949574E-03 5.50949574E-03

q1f= 0.00000000 q2f= 0.00000000

Index of gamma value 117 gamma for St popn= 38.7233353

Means of q1 and q2= 3.94908828E-04 3.94908799E-04

Variances of q1 and q2= 2.03712279E-05 2.03712389E-05

Covariance and correlation of q1 and q2= -9.91391857E-09 -4.86662670E-04

F1 and F2= 5.16050123E-02 5.16050458E-02

Freqs. of fixation of A1 for In and St= 0.994537354 0.994537354

Freqs. of fixation of A2 for In and St= 0.00000000 0.00000000

Freqs. of segregation for In and St= 5.46264648E-03 5.46264648E-03

q1f= 0.00000000 q2f= 0.00000000

Index of gamma value 118 gamma for St popn= 39.0550003

Means of q1 and q2= 3.90568428E-04 3.90568399E-04

Variances of q1 and q2= 1.99871120E-05 1.99871174E-05

Covariance and correlation of q1 and q2= -9.63810010E-09 -4.82215692E-04

F1 and F2= 5.11944145E-02 5.11944331E-02

Freqs. of fixation of A1 for In and St= 0.994583964 0.994583964

Freqs. of fixation of A2 for In and St= 0.00000000 0.00000000

Freqs. of segregation for In and St= 5.41603565E-03 5.41603565E-03

q1f= 0.00000000 q2f= 0.00000000

Index of gamma value 119 gamma for St popn= 39.3866692

Means of q1 and q2= 3.86306434E-04 3.86306434E-04

Variances of q1 and q2= 1.96131459E-05 1.96131514E-05

Covariance and correlation of q1 and q2= -9.37220079E-09 -4.77852969E-04

F1 and F2= 5.07905707E-02 5.07905856E-02

Freqs. of fixation of A1 for In and St= 0.994630098 0.994630098

Freqs. of fixation of A2 for In and St= 0.00000000 0.00000000

Freqs. of segregation for In and St= 5.36990166E-03 5.36990166E-03

q1f= 0.00000000 q2f= 0.00000000

Index of gamma value 120 gamma for St popn= 39.7183342

Means of q1 and q2= 3.82119877E-04 3.82119848E-04

Variances of q1 and q2= 1.92489661E-05 1.92489751E-05

Covariance and correlation of q1 and q2= -9.11529696E-09 -4.73547261E-04

F1 and F2= 5.03934100E-02 5.03934398E-02

Freqs. of fixation of A1 for In and St= 0.994675100 0.994675040

Freqs. of fixation of A2 for In and St= 0.00000000 0.00000000

Freqs. of segregation for In and St= 5.32490015E-03 5.32495975E-03

q1f= 0.00000000 q2f= 0.00000000

Index of gamma value 121 gamma for St popn= 40.0500031

Means of q1 and q2= 3.78007186E-04 3.78007157E-04

Variances of q1 and q2= 1.88942468E-05 1.88942540E-05

Covariance and correlation of q1 and q2= -8.86701912E-09 -4.69297229E-04

F1 and F2= 5.00027314E-02 5.00027537E-02

Freqs. of fixation of A1 for In and St= 0.994719505 0.994719505

Freqs. of fixation of A2 for In and St= 0.00000000 0.00000000

Freqs. of segregation for In and St= 5.28049469E-03 5.28049469E-03

q1f= 0.00000000 q2f= 0.00000000

Index of gamma value 122 gamma for St popn= 40.3816681

Means of q1 and q2= 3.73966439E-04 3.73966468E-04

Variances of q1 and q2= 1.85486660E-05 1.85486715E-05

Covariance and correlation of q1 and q2= -8.62731042E-09 -4.65117482E-04

F1 and F2= 4.96183708E-02 4.96183783E-02

Freqs. of fixation of A1 for In and St= 0.994763792 0.994763851

Freqs. of fixation of A2 for In and St= 0.00000000 0.00000000

Freqs. of segregation for In and St= 5.23620844E-03 5.23614883E-03

q1f= 0.00000000 q2f= 0.00000000

Index of gamma value 123 gamma for St popn= 40.7133331

Means of q1 and q2= 3.69996153E-04 3.69996153E-04

Variances of q1 and q2= 1.82119311E-05 1.82119384E-05

Covariance and correlation of q1 and q2= -8.39565928E-09 -4.60997660E-04

F1 and F2= 4.92401682E-02 4.92401868E-02

Freqs. of fixation of A1 for In and St= 0.994807065 0.994807065

Freqs. of fixation of A2 for In and St= 0.00000000 0.00000000

Freqs. of segregation for In and St= 5.19293547E-03 5.19293547E-03

q1f= 0.00000000 q2f= 0.00000000

Index of gamma value 124 gamma for St popn= 41.0450020

Means of q1 and q2= 3.66094289E-04 3.66094318E-04

Variances of q1 and q2= 1.78837472E-05 1.78837545E-05

Covariance and correlation of q1 and q2= -8.17172463E-09 -4.56935726E-04

F1 and F2= 4.88680042E-02 4.88680191E-02

Freqs. of fixation of A1 for In and St= 0.994849861 0.994849861

Freqs. of fixation of A2 for In and St= 0.00000000 0.00000000

Freqs. of segregation for In and St= 5.15013933E-03 5.15013933E-03

q1f= 0.00000000 q2f= 0.00000000

Index of gamma value 125 gamma for St popn= 41.3766670

Means of q1 and q2= 3.62259307E-04 3.62259307E-04

Variances of q1 and q2= 1.75638270E-05 1.75638324E-05

Covariance and correlation of q1 and q2= -7.95527910E-09 -4.52935317E-04

F1 and F2= 4.85017002E-02 4.85017151E-02

Freqs. of fixation of A1 for In and St= 0.994892001 0.994892001

Freqs. of fixation of A2 for In and St= 0.00000000 0.00000000

Freqs. of segregation for In and St= 5.10799885E-03 5.10799885E-03

q1f= 0.00000000 q2f= 0.00000000

Index of gamma value 126 gamma for St popn= 41.7083359

Means of q1 and q2= 3.58489517E-04 3.58489517E-04

Variances of q1 and q2= 1.72519030E-05 1.72519121E-05

Covariance and correlation of q1 and q2= -7.74596742E-09 -4.48991952E-04

F1 and F2= 4.81411256E-02 4.81411517E-02

Freqs. of fixation of A1 for In and St= 0.994933784 0.994933784

Freqs. of fixation of A2 for In and St= 0.00000000 0.00000000

Freqs. of segregation for In and St= 5.06621599E-03 5.06621599E-03

q1f= 0.00000000 q2f= 0.00000000

Index of gamma value 127 gamma for St popn= 42.0400009

Means of q1 and q2= 3.54782911E-04 3.54782911E-04

Variances of q1 and q2= 1.69477134E-05 1.69477207E-05

Covariance and correlation of q1 and q2= -7.54336327E-09 -4.45096142E-04

F1 and F2= 4.77862023E-02 4.77862246E-02

Freqs. of fixation of A1 for In and St= 0.994974971 0.994974971

Freqs. of fixation of A2 for In and St= 0.00000000 0.00000000

Freqs. of segregation for In and St= 5.02502918E-03 5.02502918E-03

q1f= 0.00000000 q2f= 0.00000000

Index of gamma value 128 gamma for St popn= 42.3716698

Means of q1 and q2= 3.51138820E-04 3.51138850E-04

Variances of q1 and q2= 1.66510235E-05 1.66510308E-05

Covariance and correlation of q1 and q2= -7.34750927E-09 -4.41264623E-04

F1 and F2= 4.74367142E-02 4.74367291E-02

Freqs. of fixation of A1 for In and St= 0.995015681 0.995015621

Freqs. of fixation of A2 for In and St= 0.00000000 0.00000000

Freqs. of segregation for In and St= 4.98431921E-03 4.98437881E-03

q1f= 0.00000000 q2f= 0.00000000

Index of gamma value 129 gamma for St popn= 42.7033348

Means of q1 and q2= 3.47554975E-04 3.47554975E-04

Variances of q1 and q2= 1.63615750E-05 1.63615823E-05

Covariance and correlation of q1 and q2= -7.15789383E-09 -4.37481853E-04

F1 and F2= 4.70925868E-02 4.70926091E-02

Freqs. of fixation of A1 for In and St= 0.995055735 0.995055735

Freqs. of fixation of A2 for In and St= 0.00000000 0.00000000

Freqs. of segregation for In and St= 4.94426489E-03 4.94426489E-03

q1f= 0.00000000 q2f= 0.00000000

Index of gamma value 130 gamma for St popn= 43.0349998

Means of q1 and q2= 3.44030501E-04 3.44030472E-04

Variances of q1 and q2= 1.60791624E-05 1.60791697E-05

Covariance and correlation of q1 and q2= -6.97437486E-09 -4.33752284E-04

F1 and F2= 4.67536934E-02 4.67537194E-02

Freqs. of fixation of A1 for In and St= 0.995095372 0.995095372

Freqs. of fixation of A2 for In and St= 0.00000000 0.00000000

Freqs. of segregation for In and St= 4.90462780E-03 4.90462780E-03

q1f= 0.00000000 q2f= 0.00000000

Index of gamma value 131 gamma for St popn= 43.3666687

Means of q1 and q2= 3.40563594E-04 3.40563594E-04

Variances of q1 and q2= 1.58035364E-05 1.58035418E-05

Covariance and correlation of q1 and q2= -6.79670364E-09 -4.30074753E-04

F1 and F2= 4.64198776E-02 4.64198925E-02

Freqs. of fixation of A1 for In and St= 0.995134294 0.995134294

Freqs. of fixation of A2 for In and St= 0.00000000 0.00000000

Freqs. of segregation for In and St= 4.86570597E-03 4.86570597E-03

q1f= 0.00000000 q2f= 0.00000000

Index of gamma value 132 gamma for St popn= 43.6983337

Means of q1 and q2= 3.37153062E-04 3.37153091E-04

Variances of q1 and q2= 1.55345151E-05 1.55345206E-05

Covariance and correlation of q1 and q2= -6.62460309E-09 -4.26444109E-04

F1 and F2= 4.60910946E-02 4.60911058E-02

Freqs. of fixation of A1 for In and St= 0.995172739 0.995172799

Freqs. of fixation of A2 for In and St= 0.00000000 0.00000000

Freqs. of segregation for In and St= 4.82726097E-03 4.82720137E-03

q1f= 0.00000000 q2f= 0.00000000

Index of gamma value 133 gamma for St popn= 44.0300026

Means of q1 and q2= 3.33797740E-04 3.33797769E-04

Variances of q1 and q2= 1.52718749E-05 1.52718803E-05

Covariance and correlation of q1 and q2= -6.45797371E-09 -4.22867073E-04

F1 and F2= 4.57671583E-02 4.57671694E-02

Freqs. of fixation of A1 for In and St= 0.995211005 0.995210946

Freqs. of fixation of A2 for In and St= 0.00000000 0.00000000

Freqs. of segregation for In and St= 4.78899479E-03 4.78905439E-03

q1f= 0.00000000 q2f= 0.00000000

Index of gamma value 134 gamma for St popn= 44.3616676

Means of q1 and q2= 3.30496201E-04 3.30496172E-04

Variances of q1 and q2= 1.50154174E-05 1.50154210E-05

Covariance and correlation of q1 and q2= -6.29653840E-09 -4.19338176E-04

F1 and F2= 4.54479717E-02 4.54479866E-02

Freqs. of fixation of A1 for In and St= 0.995248437 0.995248377

Freqs. of fixation of A2 for In and St= 0.00000000 0.00000000

Freqs. of segregation for In and St= 4.75156307E-03 4.75162268E-03

q1f= 0.00000000 q2f= 0.00000000

Index of gamma value 135 gamma for St popn= 44.6933365

Means of q1 and q2= 3.27247137E-04 3.27247137E-04

Variances of q1 and q2= 1.47649725E-05 1.47649771E-05

Covariance and correlation of q1 and q2= -6.14005558E-09 -4.15852788E-04

F1 and F2= 4.51334901E-02 4.51335013E-02

Freqs. of fixation of A1 for In and St= 0.995285630 0.995285630

Freqs. of fixation of A2 for In and St= 0.00000000 0.00000000

Freqs. of segregation for In and St= 4.71436977E-03 4.71436977E-03

q1f= 0.00000000 q2f= 0.00000000

Index of gamma value 136 gamma for St popn= 45.0250015

Means of q1 and q2= 3.24049674E-04 3.24049674E-04

Variances of q1 and q2= 1.45203539E-05 1.45203603E-05

Covariance and correlation of q1 and q2= -5.98842576E-09 -4.12415859E-04

F1 and F2= 4.48235609E-02 4.48235832E-02

Freqs. of fixation of A1 for In and St= 0.995322168 0.995322168

Freqs. of fixation of A2 for In and St= 0.00000000 0.00000000

Freqs. of segregation for In and St= 4.67783213E-03 4.67783213E-03

q1f= 0.00000000 q2f= 0.00000000

Index of gamma value 137 gamma for St popn= 45.3566666

Means of q1 and q2= 3.20902240E-04 3.20902240E-04

Variances of q1 and q2= 1.42813724E-05 1.42813778E-05

Covariance and correlation of q1 and q2= -5.84139315E-09 -4.09021770E-04

F1 and F2= 4.45180945E-02 4.45181131E-02

Freqs. of fixation of A1 for In and St= 0.995358586 0.995358586

Freqs. of fixation of A2 for In and St= 0.00000000 0.00000000

Freqs. of segregation for In and St= 4.64141369E-03 4.64141369E-03

q1f= 0.00000000 q2f= 0.00000000

Index of gamma value 138 gamma for St popn= 45.6883354

Means of q1 and q2= 3.17804050E-04 3.17804050E-04

Variances of q1 and q2= 1.40478760E-05 1.40478815E-05

Covariance and correlation of q1 and q2= -5.69889380E-09 -4.05676459E-04

F1 and F2= 4.42169979E-02 4.42170165E-02

Freqs. of fixation of A1 for In and St= 0.995394230 0.995394230

Freqs. of fixation of A2 for In and St= 0.00000000 0.00000000

Freqs. of segregation for In and St= 4.60577011E-03 4.60577011E-03

q1f= 0.00000000 q2f= 0.00000000

Index of gamma value 139 gamma for St popn= 46.0200005

Means of q1 and q2= 3.14753765E-04 3.14753735E-04

Variances of q1 and q2= 1.38196883E-05 1.38196929E-05

Covariance and correlation of q1 and q2= -5.56070034E-09 -4.02375153E-04

F1 and F2= 4.39201705E-02 4.39201891E-02

Freqs. of fixation of A1 for In and St= 0.995429337 0.995429337

Freqs. of fixation of A2 for In and St= 0.00000000 0.00000000

Freqs. of segregation for In and St= 4.57066298E-03 4.57066298E-03

q1f= 0.00000000 q2f= 0.00000000

Index of gamma value 140 gamma for St popn= 46.3516693

Means of q1 and q2= 3.11750598E-04 3.11750598E-04

Variances of q1 and q2= 1.35966666E-05 1.35966702E-05

Covariance and correlation of q1 and q2= -5.42663514E-09 -3.99115059E-04

F1 and F2= 4.36275229E-02 4.36275341E-02

Freqs. of fixation of A1 for In and St= 0.995464444 0.995464444

Freqs. of fixation of A2 for In and St= 0.00000000 0.00000000

Freqs. of segregation for In and St= 4.53555584E-03 4.53555584E-03

q1f= 0.00000000 q2f= 0.00000000

Index of gamma value 141 gamma for St popn= 46.6833344

Means of q1 and q2= 3.08793125E-04 3.08793125E-04

Variances of q1 and q2= 1.33786416E-05 1.33786471E-05

Covariance and correlation of q1 and q2= -5.29653477E-09 -3.95894720E-04

F1 and F2= 4.33389656E-02 4.33389843E-02

Freqs. of fixation of A1 for In and St= 0.995498717 0.995498717

Freqs. of fixation of A2 for In and St= 0.00000000 0.00000000

Freqs. of segregation for In and St= 4.50128317E-03 4.50128317E-03

q1f= 0.00000000 q2f= 0.00000000

Index of gamma value 142 gamma for St popn= 47.0150032

Means of q1 and q2= 3.05880909E-04 3.05880909E-04

Variances of q1 and q2= 1.31654970E-05 1.31655042E-05

Covariance and correlation of q1 and q2= -5.17037790E-09 -3.92721704E-04

F1 and F2= 4.30544205E-02 4.30544466E-02

Freqs. of fixation of A1 for In and St= 0.995532870 0.995532870

Freqs. of fixation of A2 for In and St= 0.00000000 0.00000000

Freqs. of segregation for In and St= 4.46712971E-03 4.46712971E-03

q1f= 0.00000000 q2f= 0.00000000

Index of gamma value 143 gamma for St popn= 47.3466682

Means of q1 and q2= 3.03012494E-04 3.03012464E-04

Variances of q1 and q2= 1.29570763E-05 1.29570799E-05

Covariance and correlation of q1 and q2= -5.04780218E-09 -3.89578752E-04

F1 and F2= 4.27738279E-02 4.27738428E-02

Freqs. of fixation of A1 for In and St= 0.995566249 0.995566368

Freqs. of fixation of A2 for In and St= 0.00000000 0.00000000

Freqs. of segregation for In and St= 4.43375111E-03 4.43363190E-03

q1f= 0.00000000 q2f= 0.00000000

Index of gamma value 144 gamma for St popn= 47.6783333

Means of q1 and q2= 3.00187123E-04 3.00187152E-04

Variances of q1 and q2= 1.27532276E-05 1.27532321E-05

Covariance and correlation of q1 and q2= -4.92898522E-09 -3.86489177E-04

F1 and F2= 4.24970165E-02 4.24970277E-02

Freqs. of fixation of A1 for In and St= 0.995599627 0.995599627

Freqs. of fixation of A2 for In and St= 0.00000000 0.00000000

Freqs. of segregation for In and St= 4.40037251E-03 4.40037251E-03

q1f= 0.00000000 q2f= 0.00000000

Index of gamma value 145 gamma for St popn= 48.0100021

Means of q1 and q2= 2.97403865E-04 2.97403894E-04

Variances of q1 and q2= 1.25538554E-05 1.25538609E-05

Covariance and correlation of q1 and q2= -4.81346518E-09 -3.83425155E-04

F1 and F2= 4.22240309E-02 4.22240458E-02

Freqs. of fixation of A1 for In and St= 0.995632470 0.995632470

Freqs. of fixation of A2 for In and St= 0.00000000 0.00000000

Freqs. of segregation for In and St= 4.36753035E-03 4.36753035E-03

q1f= 0.00000000 q2f= 0.00000000

Index of gamma value 146 gamma for St popn= 48.3416672

Means of q1 and q2= 2.94661731E-04 2.94661731E-04

Variances of q1 and q2= 1.23588115E-05 1.23588152E-05

Covariance and correlation of q1 and q2= -4.70130601E-09 -3.80401092E-04

F1 and F2= 4.19547372E-02 4.19547483E-02

Freqs. of fixation of A1 for In and St= 0.995664954 0.995664954

Freqs. of fixation of A2 for In and St= 0.00000000 0.00000000

Freqs. of segregation for In and St= 4.33504581E-03 4.33504581E-03

q1f= 0.00000000 q2f= 0.00000000

Index of gamma value 147 gamma for St popn= 48.6733360

Means of q1 and q2= 2.91960168E-04 2.91960139E-04

Variances of q1 and q2= 1.21679768E-05 1.21679823E-05

Covariance and correlation of q1 and q2= -4.59250060E-09 -3.77425080E-04

F1 and F2= 4.16890122E-02 4.16890346E-02

Freqs. of fixation of A1 for In and St= 0.995696783 0.995696783

Freqs. of fixation of A2 for In and St= 0.00000000 0.00000000

Freqs. of segregation for In and St= 4.30321693E-03 4.30321693E-03

q1f= 0.00000000 q2f= 0.00000000

Index of gamma value 148 gamma for St popn= 49.0050011

Means of q1 and q2= 2.89298012E-04 2.89298012E-04

Variances of q1 and q2= 1.19812466E-05 1.19812503E-05

Covariance and correlation of q1 and q2= -4.48668658E-09 -3.74475727E-04

F1 and F2= 4.14268821E-02 4.14268933E-02

Freqs. of fixation of A1 for In and St= 0.995728493 0.995728493

Freqs. of fixation of A2 for In and St= 0.00000000 0.00000000

Freqs. of segregation for In and St= 4.27150726E-03 4.27150726E-03

q1f= 0.00000000 q2f= 0.00000000

Index of gamma value 149 gamma for St popn= 49.3366699

Means of q1 and q2= 2.86674447E-04 2.86674476E-04

Variances of q1 and q2= 1.17984782E-05 1.17984828E-05

Covariance and correlation of q1 and q2= -4.38394920E-09 -3.71568953E-04

F1 and F2= 4.11681682E-02 4.11681794E-02

Freqs. of fixation of A1 for In and St= 0.995759904 0.995759904

Freqs. of fixation of A2 for In and St= 0.00000000 0.00000000

Freqs. of segregation for In and St= 4.24009562E-03 4.24009562E-03

q1f= 0.00000000 q2f= 0.00000000

Index of gamma value 150 gamma for St popn= 49.6683350

Means of q1 and q2= 2.84088805E-04 2.84088805E-04

Variances of q1 and q2= 1.16195915E-05 1.16195961E-05

Covariance and correlation of q1 and q2= -4.28407532E-09 -3.68694076E-04

F1 and F2= 4.09128889E-02 4.09129038E-02

Freqs. of fixation of A1 for In and St= 0.995790899 0.995790899

Freqs. of fixation of A2 for In and St= 0.00000000 0.00000000

Freqs. of segregation for In and St= 4.20910120E-03 4.20910120E-03

q1f= 0.00000000 q2f= 0.00000000

Index of gamma value 151 gamma for St popn= 50.0000000

Means of q1 and q2= 2.81540590E-04 2.81540590E-04

Variances of q1 and q2= 1.14444756E-05 1.14444792E-05

Covariance and correlation of q1 and q2= -4.18715018E-09 -3.65866435E-04

F1 and F2= 4.06609140E-02 4.06609289E-02

Freqs. of fixation of A1 for In and St= 0.995821595 0.995821595

Freqs. of fixation of A2 for In and St= 0.00000000 0.00000000

Freqs. of segregation for In and St= 4.17840481E-03 4.17840481E-03

q1f= 0.00000000 q2f= 0.00000000

**h=0.45**

Index of gamma value 6 gamma for St popn= 1.16759264

Means of q1 and q2= 0.420562804 0.420562804

Variances of q1 and q2= 0.239704207 0.239704207

Covariance and correlation of q1 and q2= -7.48284310E-02 -0.312169880

F1 and F2= 0.983645082 0.983645082

Freqs. of fixation of A1 for In and St= 0.654924691 0.654924691

Freqs. of fixation of A2 for In and St= 0.317191005 0.317191005

Freqs. of segregation for In and St= 2.78843045E-02 2.78843045E-02

q1f= 0.326289356 q2f= 0.326289356

Index of gamma value 7 gamma for St popn= 1.35111117

Means of q1 and q2= 0.362816036 0.362816036

Variances of q1 and q2= 0.227255777 0.227255747

Covariance and correlation of q1 and q2= -5.23452163E-02 -0.230336145

F1 and F2= 0.983022869 0.983022749

Freqs. of fixation of A1 for In and St= 0.691219985 0.691219985

Freqs. of fixation of A2 for In and St= 0.281139612 0.281139612

Freqs. of segregation for In and St= 2.76404023E-02 2.76404023E-02

q1f= 0.289131314 q2f= 0.289131314

Index of gamma value 8 gamma for St popn= 1.53462970

Means of q1 and q2= 0.311903358 0.311903358

Variances of q1 and q2= 0.210769609 0.210769609

Covariance and correlation of q1 and q2= -3.64120975E-02 -0.172757819

F1 and F2= 0.982061088 0.982061088

Freqs. of fixation of A1 for In and St= 0.724862754 0.724862754

Freqs. of fixation of A2 for In and St= 0.247817442 0.247817442

Freqs. of segregation for In and St= 2.73198038E-02 2.73198038E-02

q1f= 0.254777908 q2f= 0.254777908

Index of gamma value 9 gamma for St popn= 1.71814823

Means of q1 and q2= 0.267392546 0.267392546

Variances of q1 and q2= 0.192130059 0.192130059

Covariance and correlation of q1 and q2= -2.52741240E-02 -0.131546959

F1 and F2= 0.980786920 0.980786920

Freqs. of fixation of A1 for In and St= 0.755732834 0.755732834

Freqs. of fixation of A2 for In and St= 0.217335358 0.217335358

Freqs. of segregation for In and St= 2.69318074E-02 2.69318074E-02

q1f= 0.223350599 q2f= 0.223350599

Index of gamma value 10 gamma for St popn= 1.90166676

Means of q1 and q2= 0.228741720 0.228741720

Variances of q1 and q2= 0.172750756 0.172750756

Covariance and correlation of q1 and q2= -1.75606348E-02 -0.101653010

F1 and F2= 0.979207516 0.979207516

Freqs. of fixation of A1 for In and St= 0.783800542 0.783800542

Freqs. of fixation of A2 for In and St= 0.189711645 0.189711645

Freqs. of segregation for In and St= 2.64878124E-02 2.64878124E-02

q1f= 0.194873422 q2f= 0.194873422

Index of gamma value 11 gamma for St popn= 2.08518529

Means of q1 and q2= 0.195358649 0.195358649

Variances of q1 and q2= 0.153627694 0.153627694

Covariance and correlation of q1 and q2= -1.22457072E-02 -7.97102824E-02

F1 and F2= 0.977314889 0.977314889

Freqs. of fixation of A1 for In and St= 0.809111476 0.809111476

Freqs. of fixation of A2 for In and St= 0.164889827 0.164889827

Freqs. of segregation for In and St= 2.59986967E-02 2.59986967E-02

q1f= 0.169291183 q2f= 0.169291183

Index of gamma value 12 gamma for St popn= 2.26870394

Means of q1 and q2= 0.166645229 0.166645229

Variances of q1 and q2= 0.135415584 0.135415584

Covariance and correlation of q1 and q2= -8.58732872E-03 -6.34146258E-02

F1 and F2= 0.975092590 0.975092590

Freqs. of fixation of A1 for In and St= 0.831768155 0.831768036

Freqs. of fixation of A2 for In and St= 0.142756179 0.142756179

Freqs. of segregation for In and St= 2.54756659E-02 2.54757851E-02

q1f= 0.146488056 q2f= 0.146488085

Index of gamma value 13 gamma for St popn= 2.45222235

Means of q1 and q2= 0.142027751 0.142027751

Variances of q1 and q2= 0.118506640 0.118506640

Covariance and correlation of q1 and q2= -6.06300030E-03 -5.11616915E-02

F1 and F2= 0.972514808 0.972514808

Freqs. of fixation of A1 for In and St= 0.851915479 0.851915479

Freqs. of fixation of A2 for In and St= 0.123156004 0.123156004

Freqs. of segregation for In and St= 2.49285176E-02 2.49285176E-02

q1f= 0.126304582 q2f= 0.126304582

Index of gamma value 14 gamma for St popn= 2.63574076

Means of q1 and q2= 0.120974258 0.120974258

Variances of q1 and q2= 0.103101209 0.103101209

Covariance and correlation of q1 and q2= -4.31200396E-03 -4.18230221E-02

F1 and F2= 0.969547689 0.969547689

Freqs. of fixation of A1 for In and St= 0.869726658 0.869726658

Freqs. of fixation of A2 for In and St= 0.105907433 0.105907433

Freqs. of segregation for In and St= 2.43659094E-02 2.43659094E-02

q1f= 0.108552411 q2f= 0.108552411

Index of gamma value 15 gamma for St popn= 2.81925941

Means of q1 and q2= 0.103003390 0.103003390

Variances of q1 and q2= 8.92662331E-02 8.92662331E-02

Covariance and correlation of q1 and q2= -3.08863446E-03 -3.46002541E-02

F1 and F2= 0.966150761 0.966150761

Freqs. of fixation of A1 for In and St= 0.885390699 0.885390699

Freqs. of fixation of A2 for In and St= 9.08133015E-02 9.08133015E-02

Freqs. of segregation for In and St= 2.37959996E-02 2.37959996E-02

q1f= 9.30269733E-02 q2f= 9.30269733E-02

Index of gamma value 16 gamma for St popn= 3.00277805

Means of q1 and q2= 8.76869410E-02 8.76869410E-02

Variances of q1 and q2= 7.69800991E-02 7.69800991E-02

Covariance and correlation of q1 and q2= -2.22680625E-03 -2.89270375E-02

F1 and F2= 0.962275982 0.962275982

Freqs. of fixation of A1 for In and St= 0.899104774 0.899104774

Freqs. of fixation of A2 for In and St= 7.76709169E-02 7.76709169E-02

Freqs. of segregation for In and St= 2.32243091E-02 2.32243091E-02

q1f= 7.95176551E-02 q2f= 7.95176551E-02

Index of gamma value 17 gamma for St popn= 3.18629646

Means of q1 and q2= 7.46486634E-02 7.46486634E-02

Variances of q1 and q2= 6.61658719E-02 6.61658719E-02

Covariance and correlation of q1 and q2= -1.61451427E-03 -2.44010128E-02

F1 and F2= 0.957867324 0.957867324

Freqs. of fixation of A1 for In and St= 0.911063969 0.911063969

Freqs. of fixation of A2 for In and St= 6.62791803E-02 6.62791803E-02

Freqs. of segregation for In and St= 2.26568505E-02 2.26568505E-02

q1f= 6.78156689E-02 q2f= 6.78156689E-02

Index of gamma value 18 gamma for St popn= 3.36981487

Means of q1 and q2= 6.35605007E-02 6.35605007E-02

Variances of q1 and q2= 5.67148775E-02 5.67148775E-02

Covariance and correlation of q1 and q2= -1.17599522E-03 -2.07352154E-02

F1 and F2= 0.952861905 0.952861905

Freqs. of fixation of A1 for In and St= 0.921458125 0.921458125

Freqs. of fixation of A2 for In and St= 5.64442575E-02 5.64442575E-02

Freqs. of segregation for In and St= 2.20976174E-02 2.20976174E-02

q1f= 5.77197224E-02 q2f= 5.77197224E-02

Index of gamma value 19 gamma for St popn= 3.55333352

Means of q1 and q2= 5.41383848E-02 5.41383848E-02

Variances of q1 and q2= 4.85031269E-02 4.85031269E-02

Covariance and correlation of q1 and q2= -8.59692460E-04 -1.77244749E-02

F1 and F2= 0.947189450 0.947189450

Freqs. of fixation of A1 for In and St= 0.930466890 0.930466890

Freqs. of fixation of A2 for In and St= 4.79833633E-02 4.79833633E-02

Freqs. of segregation for In and St= 2.15497464E-02 2.15497464E-02

q1f= 4.90401685E-02 q2f= 4.90401685E-02

Index of gamma value 20 gamma for St popn= 3.73685217

Means of q1 and q2= 4.61373962E-02 4.61374000E-02

Variances of q1 and q2= 4.14021797E-02 4.14021797E-02

Covariance and correlation of q1 and q2= -6.30184892E-04 -1.52210556E-02

F1 and F2= 0.940771878 0.940771759

Freqs. of fixation of A1 for In and St= 0.938256383 0.938256383

Freqs. of fixation of A2 for In and St= 4.07272056E-02 4.07272056E-02

Freqs. of segregation for In and St= 2.10164115E-02 2.10164115E-02

q1f= 4.16015200E-02 q2f= 4.16015200E-02

Index of gamma value 21 gamma for St popn= 3.92037058

Means of q1 and q2= 3.93469445E-02 3.93469483E-02

Variances of q1 and q2= 3.52860764E-02 3.52860764E-02

Covariance and correlation of q1 and q2= -4.62865224E-04 -1.31175034E-02

F1 and F2= 0.933524668 0.933524549

Freqs. of fixation of A1 for In and St= 0.944980323 0.944980323

Freqs. of fixation of A2 for In and St= 3.45212705E-02 3.45212705E-02

Freqs. of segregation for In and St= 2.04984061E-02 2.04984061E-02

q1f= 3.52437086E-02 q2f= 3.52437086E-02

Index of gamma value 22 gamma for St popn= 4.10388899

Means of q1 and q2= 3.35864760E-02 3.35864760E-02

Variances of q1 and q2= 3.00356187E-02 3.00356187E-02

Covariance and correlation of q1 and q2= -3.40445084E-04 -1.13347117E-02

F1 and F2= 0.925356627 0.925356627

Freqs. of fixation of A1 for In and St= 0.950776875 0.950776875

Freqs. of fixation of A2 for In and St= 2.92262770E-02 2.92262770E-02

Freqs. of segregation for In and St= 1.99968480E-02 1.99968480E-02

q1f= 2.98226345E-02 q2f= 2.98226345E-02

Index of gamma value 23 gamma for St popn= 4.28740788

Means of q1 and q2= 2.87014972E-02 2.87014991E-02

Variances of q1 and q2= 2.55407710E-02 2.55407710E-02

Covariance and correlation of q1 and q2= -2.50645855E-04 -9.81355831E-03

F1 and F2= 0.916171372 0.916171312

Freqs. of fixation of A1 for In and St= 0.955768466 0.955768466

Freqs. of fixation of A2 for In and St= 2.47179046E-02 2.47179046E-02

Freqs. of segregation for In and St= 1.95136294E-02 1.95136294E-02

q1f= 2.52098404E-02 q2f= 2.52098404E-02

Index of gamma value 24 gamma for St popn= 4.47092628

Means of q1 and q2= 2.45600492E-02 2.45600492E-02

Variances of q1 and q2= 2.17017438E-02 2.17017438E-02

Covariance and correlation of q1 and q2= -1.84659410E-04 -8.50896630E-03

F1 and F2= 0.905867875 0.905867875

Freqs. of fixation of A1 for In and St= 0.960065603 0.960065603

Freqs. of fixation of A2 for In and St= 2.08863262E-02 2.08863262E-02

Freqs. of segregation for In and St= 1.90480705E-02 1.90480705E-02

q1f= 2.12918967E-02 q2f= 2.12918967E-02

Index of gamma value 25 gamma for St popn= 4.65444469

Means of q1 and q2= 2.10495405E-02 2.10495386E-02

Variances of q1 and q2= 1.84292495E-02 1.84292495E-02

Covariance and correlation of q1 and q2= -1.36116636E-04 -7.38590211E-03

F1 and F2= 0.894343436 0.894343555

Freqs. of fixation of A1 for In and St= 0.963764489 0.963764489

Freqs. of fixation of A2 for In and St= 1.76350381E-02 1.76350381E-02

Freqs. of segregation for In and St= 1.86004732E-02 1.86004732E-02

q1f= 1.79692749E-02 q2f= 1.79692749E-02

Index of gamma value 26 gamma for St popn= 4.83796310

Means of q1 and q2= 1.80740505E-02 1.80740505E-02

Variances of q1 and q2= 1.56442486E-02 1.56442486E-02

Covariance and correlation of q1 and q2= -1.00382342E-04 -6.41656527E-03

F1 and F2= 0.881496251 0.881496251

Freqs. of fixation of A1 for In and St= 0.966949046 0.966949046

Freqs. of fixation of A2 for In and St= 1.48799326E-02 1.48799326E-02

Freqs. of segregation for In and St= 1.81710217E-02 1.81710217E-02

q1f= 1.51553201E-02 q2f= 1.51553201E-02

Index of gamma value 27 gamma for St popn= 5.02148151

Means of q1 and q2= 1.54784936E-02 1.54784936E-02

Variances of q1 and q2= 1.31577104E-02 1.31577104E-02

Covariance and correlation of q1 and q2= -7.34807400E-05 -5.58461435E-03

F1 and F2= 0.863428593 0.863428593

Freqs. of fixation of A1 for In and St= 0.969051003 0.969051003

Freqs. of fixation of A2 for In and St= 1.23631703E-02 1.23631703E-02

Freqs. of segregation for In and St= 1.85858272E-02 1.85858272E-02

q1f= 1.25973010E-02 q2f= 1.25973010E-02

Index of gamma value 28 gamma for St popn= 5.20500040

Means of q1 and q2= 1.33536607E-02 1.33536607E-02

Variances of q1 and q2= 1.11616235E-02 1.11616235E-02

Covariance and correlation of q1 and q2= -5.42874041E-05 -4.86375531E-03

F1 and F2= 0.847160161 0.847160161

Freqs. of fixation of A1 for In and St= 0.971395493 0.971395493

Freqs. of fixation of A2 for In and St= 1.04148174E-02 1.04148174E-02

Freqs. of segregation for In and St= 1.81896910E-02 1.81896910E-02

q1f= 1.06077688E-02 q2f= 1.06077688E-02

Index of gamma value 29 gamma for St popn= 5.38851881

Means of q1 and q2= 1.15523674E-02 1.15523674E-02

Variances of q1 and q2= 9.46927816E-03 9.46927816E-03

Covariance and correlation of q1 and q2= -4.01423604E-05 -4.23922064E-03

F1 and F2= 0.829262912 0.829262912

Freqs. of fixation of A1 for In and St= 0.973420739 0.973420739

Freqs. of fixation of A2 for In and St= 8.76979623E-03 8.76979623E-03

Freqs. of segregation for In and St= 1.78094655E-02 1.78094655E-02

q1f= 8.92881304E-03 q2f= 8.92881304E-03

Index of gamma value 30 gamma for St popn= 5.57203722

Means of q1 and q2= 1.00248856E-02 1.00248866E-02

Variances of q1 and q2= 8.03561322E-03 8.03561322E-03

Covariance and correlation of q1 and q2= -2.97172519E-05 -3.69819347E-03

F1 and F2= 0.809683561 0.809683442

Freqs. of fixation of A1 for In and St= 0.975173473 0.975173473

Freqs. of fixation of A2 for In and St= 7.38197332E-03 7.38197332E-03

Freqs. of segregation for In and St= 1.74445529E-02 1.74445529E-02

q1f= 7.51303462E-03 q2f= 7.51303462E-03

Index of gamma value 31 gamma for St popn= 5.75555611

Means of q1 and q2= 8.72893631E-03 8.72893631E-03

Variances of q1 and q2= 6.82176463E-03 6.82176463E-03

Covariance and correlation of q1 and q2= -2.20314469E-05 -3.22958175E-03

F1 and F2= 0.788393378 0.788393378

Freqs. of fixation of A1 for In and St= 0.976693571 0.976693571

Freqs. of fixation of A2 for In and St= 6.21183077E-03 6.21183077E-03

Freqs. of segregation for In and St= 1.70945991E-02 1.70945991E-02

q1f= 6.31986652E-03 q2f= 6.31986652E-03

Index of gamma value 32 gamma for St popn= 5.93907452

Means of q1 and q2= 7.62880268E-03 7.62880361E-03

Variances of q1 and q2= 5.79453167E-03 5.79453167E-03

Covariance and correlation of q1 and q2= -1.63632794E-05 -2.82391743E-03

F1 and F2= 0.765398860 0.765398800

Freqs. of fixation of A1 for In and St= 0.978015900 0.978015900

Freqs. of fixation of A2 for In and St= 5.22578275E-03 5.22578275E-03

Freqs. of segregation for In and St= 1.67583171E-02 1.67583171E-02

q1f= 5.31485071E-03 q2f= 5.31485071E-03

Index of gamma value 33 gamma for St popn= 6.12259293

Means of q1 and q2= 6.69420557E-03 6.69420557E-03

Variances of q1 and q2= 4.92549827E-03 4.92549827E-03

Covariance and correlation of q1 and q2= -1.21804333E-05 -2.47293431E-03

F1 and F2= 0.740744054 0.740744054

Freqs. of fixation of A1 for In and St= 0.979168952 0.979169071

Freqs. of fixation of A2 for In and St= 4.39524790E-03 4.39524790E-03

Freqs. of segregation for In and St= 1.64358001E-02 1.64356809E-02

q1f= 4.46869433E-03 q2f= 4.46869386E-03

Index of gamma value 34 gamma for St popn= 6.30611134

Means of q1 and q2= 5.89958346E-03 5.89958392E-03

Variances of q1 and q2= 4.19049570E-03 4.19049570E-03

Covariance and correlation of q1 and q2= -9.09126356E-06 -2.16949591E-03

F1 and F2= 0.714519083 0.714519024

Freqs. of fixation of A1 for In and St= 0.980178416 0.980178416

Freqs. of fixation of A2 for In and St= 3.69601487E-03 3.69601487E-03

Freqs. of segregation for In and St= 1.61255691E-02 1.61255691E-02

q1f= 3.75659205E-03 q2f= 3.75659205E-03

Index of gamma value 35 gamma for St popn= 6.48962975

Means of q1 and q2= 5.22322906E-03 5.22322999E-03

Variances of q1 and q2= 3.56886070E-03 3.56886070E-03

Covariance and correlation of q1 and q2= -6.80690027E-06 -1.90730346E-03

F1 and F2= 0.686854720 0.686854601

Freqs. of fixation of A1 for In and St= 0.981065214 0.981065214

Freqs. of fixation of A2 for In and St= 3.10747582E-03 3.10747582E-03

Freqs. of segregation for In and St= 1.58273112E-02 1.58273112E-02

q1f= 3.15744965E-03 q2f= 3.15744965E-03

Index of gamma value 36 gamma for St popn= 6.67314863

Means of q1 and q2= 4.64693038E-03 4.64693038E-03

Variances of q1 and q2= 3.04317358E-03 3.04317358E-03

Covariance and correlation of q1 and q2= -5.11560029E-06 -1.68100838E-03

F1 and F2= 0.657935619 0.657935619

Freqs. of fixation of A1 for In and St= 0.981846869 0.981846869

Freqs. of fixation of A2 for In and St= 2.61231209E-03 2.61231209E-03

Freqs. of segregation for In and St= 1.55408187E-02 1.55408187E-02

q1f= 2.65355059E-03 q2f= 2.65355059E-03

Index of gamma value 37 gamma for St popn= 6.85666704

Means of q1 and q2= 4.15516645E-03 4.15516645E-03

Variances of q1 and q2= 2.59852735E-03 2.59852735E-03

Covariance and correlation of q1 and q2= -3.86085048E-06 -1.48578407E-03

F1 and F2= 0.627981961 0.627981961

Freqs. of fixation of A1 for In and St= 0.982539594 0.982539594

Freqs. of fixation of A2 for In and St= 2.19576620E-03 2.19576620E-03

Freqs. of segregation for In and St= 1.52646396E-02 1.52646396E-02

q1f= 2.22980324E-03 q2f= 2.22980324E-03

Index of gamma value 38 gamma for St popn= 7.04018545

Means of q1 and q2= 3.73490737E-03 3.73490737E-03

Variances of q1 and q2= 2.22236942E-03 2.22236966E-03

Covariance and correlation of q1 and q2= -2.92803179E-06 -1.31752691E-03

F1 and F2= 0.597257316 0.597257376

Freqs. of fixation of A1 for In and St= 0.983155608 0.983155608

Freqs. of fixation of A2 for In and St= 1.84544269E-03 1.84544269E-03

Freqs. of segregation for In and St= 1.49989491E-02 1.49989491E-02

q1f= 1.87354395E-03 q2f= 1.87354395E-03

Index of gamma value 39 gamma for St popn= 7.22370434

Means of q1 and q2= 3.37515143E-03 3.37515143E-03

Variances of q1 and q2= 1.90407550E-03 1.90407562E-03

Covariance and correlation of q1 and q2= -2.23282768E-06 -1.17265712E-03

F1 and F2= 0.566055715 0.566055775

Freqs. of fixation of A1 for In and St= 0.983706653 0.983706653

Freqs. of fixation of A2 for In and St= 1.55088387E-03 1.55088387E-03

Freqs. of segregation for In and St= 1.47424629E-02 1.47424629E-02

q1f= 1.57408975E-03 q2f= 1.57408975E-03

Index of gamma value 40 gamma for St popn= 7.40722275

Means of q1 and q2= 3.06658982E-03 3.06658982E-03

Variances of q1 and q2= 1.63463445E-03 1.63463445E-03

Covariance and correlation of q1 and q2= -1.71317424E-06 -1.04804733E-03

F1 and F2= 0.534686029 0.534686029

Freqs. of fixation of A1 for In and St= 0.984201193 0.984201133

Freqs. of fixation of A2 for In and St= 1.30324624E-03 1.30324624E-03

Freqs. of segregation for In and St= 1.44955609E-02 1.44956205E-02

q1f= 1.32241542E-03 q2f= 1.32241542E-03

Index of gamma value 41 gamma for St popn= 7.59074116

Means of q1 and q2= 2.80133262E-03 2.80133309E-03

Variances of q1 and q2= 1.40640687E-03 1.40640687E-03

Covariance and correlation of q1 and q2= -1.32330024E-06 -9.40908561E-04

F1 and F2= 0.503459573 0.503459513

Freqs. of fixation of A1 for In and St= 0.984648108 0.984648108

Freqs. of fixation of A2 for In and St= 1.09505339E-03 1.09505339E-03

Freqs. of segregation for In and St= 1.42568378E-02 1.42568378E-02

q1f= 1.11089123E-03 q2f= 1.11089123E-03

Index of gamma value 42 gamma for St popn= 7.77425957

Means of q1 and q2= 2.57281098E-03 2.57281074E-03

Variances of q1 and q2= 1.21302158E-03 1.21302158E-03

Covariance and correlation of q1 and q2= -1.02982131E-06 -8.48971948E-04

F1 and F2= 0.472693294 0.472693354

Freqs. of fixation of A1 for In and St= 0.985053420 0.985053420

Freqs. of fixation of A2 for In and St= 9.20083257E-04 9.20083257E-04

Freqs. of segregation for In and St= 1.40264966E-02 1.40264966E-02

q1f= 9.33172414E-04 q2f= 9.33172414E-04

Index of gamma value 43 gamma for St popn= 7.95777798

Means of q1 and q2= 2.37539457E-03 2.37539457E-03

Variances of q1 and q2= 1.04902370E-03 1.04902382E-03

Covariance and correlation of q1 and q2= -8.07877768E-07 -7.70123443E-04

F1 and F2= 0.442672372 0.442672431

Freqs. of fixation of A1 for In and St= 0.985423148 0.985423148

Freqs. of fixation of A2 for In and St= 7.73026957E-04 7.73026957E-04

Freqs. of segregation for In and St= 1.38038257E-02 1.38038257E-02

q1f= 7.83847063E-04 q2f= 7.83847063E-04

Index of gamma value 44 gamma for St popn= 8.14129639

Means of q1 and q2= 2.20437604E-03 2.20437604E-03

Variances of q1 and q2= 9.09847091E-04 9.09847091E-04

Covariance and correlation of q1 and q2= -6.39268364E-07 -7.02610763E-04

F1 and F2= 0.413657695 0.413657695

Freqs. of fixation of A1 for In and St= 0.985762477 0.985762477

Freqs. of fixation of A2 for In and St= 6.49450405E-04 6.49450405E-04

Freqs. of segregation for In and St= 1.35880727E-02 1.35880727E-02

q1f= 6.58396748E-04 q2f= 6.58396748E-04

Index of gamma value 45 gamma for St popn= 8.32481575

Means of q1 and q2= 2.05575861E-03 2.05575861E-03

Variances of q1 and q2= 7.91621103E-04 7.91620987E-04

Covariance and correlation of q1 and q2= -5.10492555E-07 -6.44869870E-04

F1 and F2= 0.385868162 0.385868132

Freqs. of fixation of A1 for In and St= 0.986074626 0.986074626

Freqs. of fixation of A2 for In and St= 5.45604737E-04 5.45604737E-04

Freqs. of segregation for In and St= 1.33797685E-02 1.33797685E-02

q1f= 5.53003803E-04 q2f= 5.53003803E-04

Index of gamma value 46 gamma for St popn= 8.50833416

Means of q1 and q2= 1.92618789E-03 1.92618824E-03

Variances of q1 and q2= 6.91099034E-04 6.91099034E-04

Covariance and correlation of q1 and q2= -4.11602969E-07 -5.95577410E-04

F1 and F2= 0.359483510 0.359483451

Freqs. of fixation of A1 for In and St= 0.986363947 0.986363947

Freqs. of fixation of A2 for In and St= 4.58353665E-04 4.58353607E-04

Freqs. of segregation for In and St= 1.31776985E-02 1.31776994E-02

q1f= 4.64474375E-04 q2f= 4.64474317E-04

Index of gamma value 47 gamma for St popn= 8.69185257

Means of q1 and q2= 1.81281392E-03 1.81281369E-03

Variances of q1 and q2= 6.05525682E-04 6.05525682E-04

Covariance and correlation of q1 and q2= -3.35193363E-07 -5.53557649E-04

F1 and F2= 0.334631920 0.334631950

Freqs. of fixation of A1 for In and St= 0.986632884 0.986632884

Freqs. of fixation of A2 for In and St= 3.85043706E-04 3.85043677E-04

Freqs. of segregation for In and St= 1.29820723E-02 1.29820723E-02

q1f= 3.90108122E-04 q2f= 3.90108093E-04

Index of gamma value 48 gamma for St popn= 8.87537098

Means of q1 and q2= 1.71323610E-03 1.71323598E-03

Variances of q1 and q2= 5.32586651E-04 5.32586593E-04

Covariance and correlation of q1 and q2= -2.75774710E-07 -5.17802546E-04

F1 and F2= 0.311399370 0.311399370

Freqs. of fixation of A1 for In and St= 0.986884356 0.986884356

Freqs. of fixation of A2 for In and St= 3.23452201E-04 3.23452201E-04

Freqs. of segregation for In and St= 1.27921924E-02 1.27921924E-02

q1f= 3.27643473E-04 q2f= 3.27643473E-04

Index of gamma value 49 gamma for St popn= 9.05888939

Means of q1 and q2= 1.62542495E-03 1.62542495E-03

Variances of q1 and q2= 4.70327650E-04 4.70327650E-04

Covariance and correlation of q1 and q2= -2.29254738E-07 -4.87436220E-04

F1 and F2= 0.289827824 0.289827824

Freqs. of fixation of A1 for In and St= 0.987120152 0.987120152

Freqs. of fixation of A2 for In and St= 2.71707861E-04 2.71707861E-04

Freqs. of segregation for In and St= 1.26081407E-02 1.26081407E-02

q1f= 2.75177328E-04 q2f= 2.75177328E-04

Index of gamma value 50 gamma for St popn= 9.24240780

Means of q1 and q2= 1.54766208E-03 1.54766208E-03

Variances of q1 and q2= 4.17099829E-04 4.17099829E-04

Covariance and correlation of q1 and q2= -1.92566858E-07 -4.61680494E-04

F1 and F2= 0.269920915 0.269920915

Freqs. of fixation of A1 for In and St= 0.987342238 0.987342238

Freqs. of fixation of A2 for In and St= 2.28236517E-04 2.28236517E-04

Freqs. of segregation for In and St= 1.24295251E-02 1.24295251E-02

q1f= 2.31109094E-04 q2f= 2.31109094E-04

Index of gamma value 51 gamma for St popn= 9.42592621

Means of q1 and q2= 1.47849938E-03 1.47849938E-03

Variances of q1 and q2= 3.71514005E-04 3.71513976E-04

Covariance and correlation of q1 and q2= -1.63424602E-07 -4.39888157E-04

F1 and F2= 0.251649827 0.251649797

Freqs. of fixation of A1 for In and St= 0.987552583 0.987552583

Freqs. of fixation of A2 for In and St= 1.91716725E-04 1.91716725E-04

Freqs. of segregation for In and St= 1.22557003E-02 1.22557003E-02

q1f= 1.94095512E-04 q2f= 1.94095512E-04

Index of gamma value 52 gamma for St popn= 9.60944462

Means of q1 and q2= 1.41670613E-03 1.41670613E-03

Variances of q1 and q2= 3.32399854E-04 3.32399854E-04

Covariance and correlation of q1 and q2= -1.40092993E-07 -4.21459263E-04

F1 and F2= 0.234961510 0.234961510

Freqs. of fixation of A1 for In and St= 0.987752020 0.987752140

Freqs. of fixation of A2 for In and St= 1.61038901E-04 1.61038901E-04

Freqs. of segregation for In and St= 1.20869409E-02 1.20868217E-02

q1f= 1.63009187E-04 q2f= 1.63009157E-04

Index of gamma value 53 gamma for St popn= 9.79296303

Means of q1 and q2= 1.36124413E-03 1.36124413E-03

Variances of q1 and q2= 2.98768224E-04 2.98768224E-04

Covariance and correlation of q1 and q2= -1.21272365E-07 -4.05907835E-04

F1 and F2= 0.219780907 0.219780907

Freqs. of fixation of A1 for In and St= 0.987942219 0.987942219

Freqs. of fixation of A2 for In and St= 1.35268754E-04 1.35268754E-04

Freqs. of segregation for In and St= 1.19225122E-02 1.19225122E-02

q1f= 1.36900955E-04 q2f= 1.36900955E-04

Index of gamma value 54 gamma for St popn= 9.97648239

Means of q1 and q2= 1.31122838E-03 1.31122849E-03

Variances of q1 and q2= 2.69784214E-04 2.69784214E-04

Covariance and correlation of q1 and q2= -1.06024117E-07 -3.92996008E-04

F1 and F2= 0.206019357 0.206019342

Freqs. of fixation of A1 for In and St= 0.988123238 0.988123238

Freqs. of fixation of A2 for In and St= 1.13621216E-04 1.13621216E-04

Freqs. of segregation for In and St= 1.17631406E-02 1.17631406E-02

q1f= 1.14973671E-04 q2f= 1.14973671E-04

Index of gamma value 55 gamma for St popn= 10.1600008

Means of q1 and q2= 1.26591616E-03 1.26591604E-03

Variances of q1 and q2= 2.44745752E-04 2.44745781E-04

Covariance and correlation of q1 and q2= -9.36086053E-08 -3.82472819E-04

F1 and F2= 0.193579942 0.193579987

Freqs. of fixation of A1 for In and St= 0.988296986 0.988296986

Freqs. of fixation of A2 for In and St= 9.54383140E-05 9.54383140E-05

Freqs. of segregation for In and St= 1.16075762E-02 1.16075762E-02

q1f= 9.65591316E-05 q2f= 9.65591316E-05

Index of gamma value 56 gamma for St popn= 10.3435192

Means of q1 and q2= 1.22466532E-03 1.22466532E-03

Variances of q1 and q2= 2.23054376E-04 2.23054405E-04

Covariance and correlation of q1 and q2= -8.32538944E-08 -3.73244809E-04

F1 and F2= 0.182358295 0.182358310

Freqs. of fixation of A1 for In and St= 0.988463461 0.988463461

Freqs. of fixation of A2 for In and St= 8.01621063E-05 8.01621063E-05

Freqs. of segregation for In and St= 1.14563769E-02 1.14563769E-02

q1f= 8.10911151E-05 q2f= 8.10911151E-05

Index of gamma value 57 gamma for St popn= 10.5270376

Means of q1 and q2= 1.18694478E-03 1.18694478E-03

Variances of q1 and q2= 2.04213226E-04 2.04213211E-04

Covariance and correlation of q1 and q2= -7.46105115E-08 -3.65355925E-04

F1 and F2= 0.172253937 0.172253922

Freqs. of fixation of A1 for In and St= 0.988624156 0.988624036

Freqs. of fixation of A2 for In and St= 6.73321556E-05 6.73321556E-05

Freqs. of segregation for In and St= 1.13085127E-02 1.13086319E-02

q1f= 6.81022866E-05 q2f= 6.81023012E-05

Index of gamma value 58 gamma for St popn= 10.7105560

Means of q1 and q2= 1.15228829E-03 1.15228840E-03

Variances of q1 and q2= 1.87794110E-04 1.87794110E-04

Covariance and correlation of q1 and q2= -6.82661039E-08 -3.63515690E-04

F1 and F2= 0.163162947 0.163162917

Freqs. of fixation of A1 for In and St= 0.988778532 0.988778591

Freqs. of fixation of A2 for In and St= 5.65547962E-05 5.65547962E-05

Freqs. of segregation for In and St= 1.11649139E-02 1.11648543E-02

q1f= 5.71933560E-05 q2f= 5.71933524E-05

Index of gamma value 59 gamma for St popn= 10.8940744

Means of q1 and q2= 1.12031214E-03 1.12031226E-03

Variances of q1 and q2= 1.73443928E-04 1.73443928E-04

Covariance and correlation of q1 and q2= -6.18301783E-08 -3.56485107E-04

F1 and F2= 0.154991135 0.154991120

Freqs. of fixation of A1 for In and St= 0.988927960 0.988927960

Freqs. of fixation of A2 for In and St= 4.75024171E-05 4.75024099E-05

Freqs. of segregation for In and St= 1.10245375E-02 1.10245375E-02

q1f= 4.80319468E-05 q2f= 4.80319395E-05

Index of gamma value 60 gamma for St popn= 11.0775928

Means of q1 and q2= 1.09068002E-03 1.09068013E-03

Variances of q1 and q2= 1.60857060E-04 1.60857060E-04

Covariance and correlation of q1 and q2= -5.63693447E-08 -3.50431277E-04

F1 and F2= 0.147644311 0.147644296

Freqs. of fixation of A1 for In and St= 0.989072323 0.989072323

Freqs. of fixation of A2 for In and St= 3.98989832E-05 3.98989832E-05

Freqs. of segregation for In and St= 1.08877784E-02 1.08877784E-02

q1f= 4.03381782E-05 q2f= 4.03381782E-05

Index of gamma value 61 gamma for St popn= 11.2611122

Means of q1 and q2= 1.06310658E-03 1.06310670E-03

Variances of q1 and q2= 1.49776737E-04 1.49776737E-04

Covariance and correlation of q1 and q2= -5.17109129E-08 -3.45253298E-04

F1 and F2= 0.141035840 0.141035825

Freqs. of fixation of A1 for In and St= 0.989212036 0.989212036

Freqs. of fixation of A2 for In and St= 3.35119184E-05 3.35119221E-05

Freqs. of segregation for In and St= 1.07544521E-02 1.07544521E-02

q1f= 3.38762402E-05 q2f= 3.38762438E-05

Index of gamma value 62 gamma for St popn= 11.4446306

Means of q1 and q2= 1.03734992E-03 1.03734992E-03

Variances of q1 and q2= 1.39987154E-04 1.39987154E-04

Covariance and correlation of q1 and q2= -4.77034519E-08 -3.40770202E-04

F1 and F2= 0.135087043 0.135087043

Freqs. of fixation of A1 for In and St= 0.989347756 0.989347816

Freqs. of fixation of A2 for In and St= 2.81476496E-05 2.81476496E-05

Freqs. of segregation for In and St= 1.06240967E-02 1.06240371E-02

q1f= 2.84499038E-05 q2f= 2.84499019E-05

Index of gamma value 63 gamma for St popn= 11.6281490

Means of q1 and q2= 1.01320154E-03 1.01320154E-03

Variances of q1 and q2= 1.31304230E-04 1.31304230E-04

Covariance and correlation of q1 and q2= -4.42283863E-08 -3.36839003E-04

F1 and F2= 0.129724830 0.129724830

Freqs. of fixation of A1 for In and St= 0.989479363 0.989479423

Freqs. of fixation of A2 for In and St= 2.36419601E-05 2.36419564E-05

Freqs. of segregation for In and St= 1.04969954E-02 1.04969358E-02

q1f= 2.38927623E-05 q2f= 2.38927569E-05

Index of gamma value 64 gamma for St popn= 11.8116674

Means of q1 and q2= 9.90481698E-04 9.90481814E-04

Variances of q1 and q2= 1.23572114E-04 1.23572114E-04

Covariance and correlation of q1 and q2= -4.11907308E-08 -3.33333533E-04

F1 and F2= 0.124883309 0.124883294

Freqs. of fixation of A1 for In and St= 0.989607692 0.989607692

Freqs. of fixation of A2 for In and St= 1.98573834E-05 1.98573798E-05

Freqs. of segregation for In and St= 1.03724506E-02 1.03724506E-02

q1f= 2.00655122E-05 q2f= 2.00655086E-05

Index of gamma value 65 gamma for St popn= 11.9951859

Means of q1 and q2= 9.69037181E-04 9.69037181E-04

Variances of q1 and q2= 1.16658543E-04 1.16658543E-04

Covariance and correlation of q1 and q2= -3.85119279E-08 -3.30125215E-04

F1 and F2= 0.120502807 0.120502807

Freqs. of fixation of A1 for In and St= 0.989732623 0.989732623

Freqs. of fixation of A2 for In and St= 1.66781901E-05 1.66781901E-05

Freqs. of segregation for In and St= 1.02506988E-02 1.02506988E-02

q1f= 1.68509232E-05 q2f= 1.68509232E-05

Index of gamma value 66 gamma for St popn= 12.1787043

Means of q1 and q2= 9.48735629E-04 9.48735629E-04

Variances of q1 and q2= 1.10452514E-04 1.10452514E-04

Covariance and correlation of q1 and q2= -3.61330308E-08 -3.27136338E-04

F1 and F2= 0.116531305 0.116531305

Freqs. of fixation of A1 for In and St= 0.989853919 0.989853919

Freqs. of fixation of A2 for In and St= 1.40086668E-05 1.40086668E-05

Freqs. of segregation for In and St= 1.01320725E-02 1.01320725E-02

q1f= 1.41520568E-05 q2f= 1.41520568E-05

Index of gamma value 67 gamma for St popn= 12.3622227

Means of q1 and q2= 9.29461967E-04 9.29461850E-04

Variances of q1 and q2= 1.04857456E-04 1.04857441E-04

Covariance and correlation of q1 and q2= -3.40140218E-08 -3.24383465E-04

F1 and F2= 0.112920180 0.112920173

Freqs. of fixation of A1 for In and St= 0.989972353 0.989972353

Freqs. of fixation of A2 for In and St= 1.17660857E-05 1.17660857E-05

Freqs. of segregation for In and St= 1.00158807E-02 1.00158807E-02

q1f= 1.18851258E-05 q2f= 1.18851258E-05

Index of gamma value 68 gamma for St popn= 12.5457411

Means of q1 and q2= 9.11117415E-04 9.11117415E-04

Variances of q1 and q2= 9.97929892E-05 9.97929892E-05

Covariance and correlation of q1 and q2= -3.21111884E-08 -3.21778003E-04

F1 and F2= 0.109628022 0.109628022

Freqs. of fixation of A1 for In and St= 0.990087807 0.990087807

Freqs. of fixation of A2 for In and St= 0.00000000 0.00000000

Freqs. of segregation for In and St= 9.91219282E-03 9.91219282E-03

q1f= 0.00000000 q2f= 0.00000000

Index of gamma value 69 gamma for St popn= 12.7292595

Means of q1 and q2= 8.93616059E-04 8.93616059E-04

Variances of q1 and q2= 9.51896436E-05 9.51896291E-05

Covariance and correlation of q1 and q2= -3.03931529E-08 -3.19290557E-04

F1 and F2= 0.106617130 0.106617115

Freqs. of fixation of A1 for In and St= 0.990200460 0.990200460

Freqs. of fixation of A2 for In and St= 0.00000000 0.00000000

Freqs. of segregation for In and St= 9.79954004E-03 9.79954004E-03

q1f= 0.00000000 q2f= 0.00000000

Index of gamma value 70 gamma for St popn= 12.9127789

Means of q1 and q2= 8.76881531E-04 8.76881357E-04

Variances of q1 and q2= 9.09887822E-05 9.09887822E-05

Covariance and correlation of q1 and q2= -2.88289357E-08 -3.16840538E-04

F1 and F2= 0.103855126 0.103855141

Freqs. of fixation of A1 for In and St= 0.990310907 0.990310907

Freqs. of fixation of A2 for In and St= 0.00000000 0.00000000

Freqs. of segregation for In and St= 9.68909264E-03 9.68909264E-03

q1f= 0.00000000 q2f= 0.00000000

Index of gamma value 71 gamma for St popn= 13.0962973

Means of q1 and q2= 8.60850152E-04 8.60850152E-04

Variances of q1 and q2= 8.71400480E-05 8.71400407E-05

Covariance and correlation of q1 and q2= -2.74063154E-08 -3.14508856E-04

F1 and F2= 0.101312794 0.101312779

Freqs. of fixation of A1 for In and St= 0.990418613 0.990418613

Freqs. of fixation of A2 for In and St= 0.00000000 0.00000000

Freqs. of segregation for In and St= 9.58138704E-03 9.58138704E-03

q1f= 0.00000000 q2f= 0.00000000

Index of gamma value 72 gamma for St popn= 13.2798157

Means of q1 and q2= 8.45463248E-04 8.45463248E-04

Variances of q1 and q2= 8.36004474E-05 8.36004474E-05

Covariance and correlation of q1 and q2= -2.60959041E-08 -3.12150281E-04

F1 and F2= 9.89649072E-02 9.89649072E-02

Freqs. of fixation of A1 for In and St= 0.990524173 0.990524173

Freqs. of fixation of A2 for In and St= 0.00000000 0.00000000

Freqs. of segregation for In and St= 9.47582722E-03 9.47582722E-03

q1f= 0.00000000 q2f= 0.00000000

Index of gamma value 73 gamma for St popn= 13.4633341

Means of q1 and q2= 8.30670935E-04 8.30670935E-04

Variances of q1 and q2= 8.03330622E-05 8.03330695E-05

Covariance and correlation of q1 and q2= -2.48940069E-08 -3.09884956E-04

F1 and F2= 9.67890471E-02 9.67890546E-02

Freqs. of fixation of A1 for In and St= 0.990626812 0.990626812

Freqs. of fixation of A2 for In and St= 0.00000000 0.00000000

Freqs. of segregation for In and St= 9.37318802E-03 9.37318802E-03

q1f= 0.00000000 q2f= 0.00000000

Index of gamma value 74 gamma for St popn= 13.6468525

Means of q1 and q2= 8.16428103E-04 8.16428103E-04

Variances of q1 and q2= 7.73062129E-05 7.73062129E-05

Covariance and correlation of q1 and q2= -2.37797622E-08 -3.07604787E-04

F1 and F2= 9.47656929E-02 9.47656929E-02

Freqs. of fixation of A1 for In and St= 0.990727901 0.990727901

Freqs. of fixation of A2 for In and St= 0.00000000 0.00000000

Freqs. of segregation for In and St= 9.27209854E-03 9.27209854E-03

q1f= 0.00000000 q2f= 0.00000000

Index of gamma value 75 gamma for St popn= 13.8303709

Means of q1 and q2= 8.02696799E-04 8.02696799E-04

Variances of q1 and q2= 7.44927747E-05 7.44927747E-05

Covariance and correlation of q1 and q2= -2.27479404E-08 -3.05371097E-04

F1 and F2= 9.28776786E-02 9.28776786E-02

Freqs. of fixation of A1 for In and St= 0.990826845 0.990826845

Freqs. of fixation of A2 for In and St= 0.00000000 0.00000000

Freqs. of segregation for In and St= 9.17315483E-03 9.17315483E-03

q1f= 0.00000000 q2f= 0.00000000

Index of gamma value 76 gamma for St popn= 14.0138893

Means of q1 and q2= 7.89440179E-04 7.89440179E-04

Variances of q1 and q2= 7.18692390E-05 7.18692390E-05

Covariance and correlation of q1 and q2= -2.17928005E-08 -3.03228473E-04

F1 and F2= 9.11101624E-02 9.11101624E-02

Freqs. of fixation of A1 for In and St= 0.990923584 0.990923584

Freqs. of fixation of A2 for In and St= 0.00000000 0.00000000

Freqs. of segregation for In and St= 9.07641649E-03 9.07641649E-03

q1f= 0.00000000 q2f= 0.00000000

Index of gamma value 77 gamma for St popn= 14.1974087

Means of q1 and q2= 7.76628731E-04 7.76628614E-04

Variances of q1 and q2= 6.94155024E-05 6.94155024E-05

Covariance and correlation of q1 and q2= -2.08974029E-08 -3.01048072E-04

F1 and F2= 8.94500166E-02 8.94500315E-02

Freqs. of fixation of A1 for In and St= 0.991018355 0.991018355

Freqs. of fixation of A2 for In and St= 0.00000000 0.00000000

Freqs. of segregation for In and St= 8.98164511E-03 8.98164511E-03

q1f= 0.00000000 q2f= 0.00000000

Index of gamma value 78 gamma for St popn= 14.3809271

Means of q1 and q2= 7.64233351E-04 7.64233351E-04

Variances of q1 and q2= 6.71139787E-05 6.71139787E-05

Covariance and correlation of q1 and q2= -2.00588488E-08 -2.98877363E-04

F1 and F2= 8.78858641E-02 8.78858641E-02

Freqs. of fixation of A1 for In and St= 0.991111457 0.991111457

Freqs. of fixation of A2 for In and St= 0.00000000 0.00000000

Freqs. of segregation for In and St= 8.88854265E-03 8.88854265E-03

q1f= 0.00000000 q2f= 0.00000000

Index of gamma value 79 gamma for St popn= 14.5644455

Means of q1 and q2= 7.52229185E-04 7.52229185E-04

Variances of q1 and q2= 6.49493813E-05 6.49493813E-05

Covariance and correlation of q1 and q2= -1.92732159E-08 -2.96742102E-04

F1 and F2= 8.64075422E-02 8.64075422E-02

Freqs. of fixation of A1 for In and St= 0.991202414 0.991202414

Freqs. of fixation of A2 for In and St= 0.00000000 0.00000000

Freqs. of segregation for In and St= 8.79758596E-03 8.79758596E-03

q1f= 0.00000000 q2f= 0.00000000

Index of gamma value 80 gamma for St popn= 14.7479639

Means of q1 and q2= 7.40593008E-04 7.40593008E-04

Variances of q1 and q2= 6.29086644E-05 6.29086571E-05

Covariance and correlation of q1 and q2= -1.85337399E-08 -2.94613477E-04

F1 and F2= 8.50065947E-02 8.50065872E-02

Freqs. of fixation of A1 for In and St= 0.991291761 0.991291761

Freqs. of fixation of A2 for In and St= 0.00000000 0.00000000

Freqs. of segregation for In and St= 8.70823860E-03 8.70823860E-03

q1f= 0.00000000 q2f= 0.00000000

Index of gamma value 81 gamma for St popn= 14.9314823

Means of q1 and q2= 7.29306485E-04 7.29306485E-04

Variances of q1 and q2= 6.09801864E-05 6.09801864E-05

Covariance and correlation of q1 and q2= -1.78383175E-08 -2.92526442E-04

F1 and F2= 8.36749598E-02 8.36749598E-02

Freqs. of fixation of A1 for In and St= 0.991379499 0.991379499

Freqs. of fixation of A2 for In and St= 0.00000000 0.00000000

Freqs. of segregation for In and St= 8.62050056E-03 8.62050056E-03

q1f= 0.00000000 q2f= 0.00000000

Index of gamma value 82 gamma for St popn= 15.1150007

Means of q1 and q2= 7.18348718E-04 7.18348718E-04

Variances of q1 and q2= 5.91538665E-05 5.91538628E-05

Covariance and correlation of q1 and q2= -1.71816623E-08 -2.90457159E-04

F1 and F2= 8.24062005E-02 8.24061930E-02

Freqs. of fixation of A1 for In and St= 0.991465688 0.991465569

Freqs. of fixation of A2 for In and St= 0.00000000 0.00000000

Freqs. of segregation for In and St= 8.53431225E-03 8.53443146E-03

q1f= 0.00000000 q2f= 0.00000000

Index of gamma value 83 gamma for St popn= 15.2985191

Means of q1 and q2= 7.07704399E-04 7.07704399E-04

Variances of q1 and q2= 5.74209007E-05 5.74209007E-05

Covariance and correlation of q1 and q2= -1.65572374E-08 -2.88348616E-04

F1 and F2= 8.11943039E-02 8.11943039E-02

Freqs. of fixation of A1 for In and St= 0.991550088 0.991550088

Freqs. of fixation of A2 for In and St= 0.00000000 0.00000000

Freqs. of segregation for In and St= 8.44991207E-03 8.44991207E-03

q1f= 0.00000000 q2f= 0.00000000

Index of gamma value 84 gamma for St popn= 15.4820375

Means of q1 and q2= 6.97356998E-04 6.97356998E-04

Variances of q1 and q2= 5.57734456E-05 5.57734529E-05

Covariance and correlation of q1 and q2= -1.59700733E-08 -2.86338269E-04

F1 and F2= 8.00341442E-02 8.00341517E-02

Freqs. of fixation of A1 for In and St= 0.991632998 0.991633117

Freqs. of fixation of A2 for In and St= 0.00000000 0.00000000

Freqs. of segregation for In and St= 8.36700201E-03 8.36688280E-03

q1f= 0.00000000 q2f= 0.00000000

Index of gamma value 85 gamma for St popn= 15.6655560

Means of q1 and q2= 6.87291496E-04 6.87291496E-04

Variances of q1 and q2= 5.42046400E-05 5.42046400E-05

Covariance and correlation of q1 and q2= -1.54111888E-08 -2.84314941E-04

F1 and F2= 7.89212734E-02 7.89212734E-02

Freqs. of fixation of A1 for In and St= 0.991714299 0.991714418

Freqs. of fixation of A2 for In and St= 0.00000000 0.00000000

Freqs. of segregation for In and St= 8.28570127E-03 8.28558207E-03

q1f= 0.00000000 q2f= 0.00000000

Index of gamma value 86 gamma for St popn= 15.8490753

Means of q1 and q2= 6.77496893E-04 6.77496893E-04

Variances of q1 and q2= 5.27084048E-05 5.27084121E-05

Covariance and correlation of q1 and q2= -1.48806123E-08 -2.82319525E-04

F1 and F2= 7.78514892E-02 7.78514966E-02

Freqs. of fixation of A1 for In and St= 0.991794407 0.991794407

Freqs. of fixation of A2 for In and St= 0.00000000 0.00000000

Freqs. of segregation for In and St= 8.20559263E-03 8.20559263E-03

q1f= 0.00000000 q2f= 0.00000000

Index of gamma value 87 gamma for St popn= 16.0325928

Means of q1 and q2= 6.67959743E-04 6.67959801E-04

Variances of q1 and q2= 5.12793958E-05 5.12793958E-05

Covariance and correlation of q1 and q2= -1.43767807E-08 -2.80361739E-04

F1 and F2= 7.68214986E-02 7.68214986E-02

Freqs. of fixation of A1 for In and St= 0.991873026 0.991873026

Freqs. of fixation of A2 for In and St= 0.00000000 0.00000000

Freqs. of segregation for In and St= 8.12697411E-03 8.12697411E-03

q1f= 0.00000000 q2f= 0.00000000

Index of gamma value 88 gamma for St popn= 16.2161121

Means of q1 and q2= 6.58668811E-04 6.58668811E-04

Variances of q1 and q2= 4.99126982E-05 4.99126982E-05

Covariance and correlation of q1 and q2= -1.38942085E-08 -2.78370222E-04

F1 and F2= 7.58280903E-02 7.58280903E-02

Freqs. of fixation of A1 for In and St= 0.991950333 0.991950333

Freqs. of fixation of A2 for In and St= 0.00000000 0.00000000

Freqs. of segregation for In and St= 8.04966688E-03 8.04966688E-03

q1f= 0.00000000 q2f= 0.00000000

Index of gamma value 89 gamma for St popn= 16.3996315

Means of q1 and q2= 6.49613969E-04 6.49614027E-04

Variances of q1 and q2= 4.86039462E-05 4.86039462E-05

Covariance and correlation of q1 and q2= -1.34371589E-08 -2.76462291E-04

F1 and F2= 7.48683736E-02 7.48683661E-02

Freqs. of fixation of A1 for In and St= 0.992026150 0.992026150

Freqs. of fixation of A2 for In and St= 0.00000000 0.00000000

Freqs. of segregation for In and St= 7.97384977E-03 7.97384977E-03

q1f= 0.00000000 q2f= 0.00000000

Index of gamma value 90 gamma for St popn= 16.5831490

Means of q1 and q2= 6.40785962E-04 6.40785962E-04

Variances of q1 and q2= 4.73493819E-05 4.73493819E-05

Covariance and correlation of q1 and q2= -1.30009994E-08 -2.74575897E-04

F1 and F2= 7.39400461E-02 7.39400461E-02

Freqs. of fixation of A1 for In and St= 0.992100835 0.992100835

Freqs. of fixation of A2 for In and St= 0.00000000 0.00000000

Freqs. of segregation for In and St= 7.89916515E-03 7.89916515E-03

q1f= 0.00000000 q2f= 0.00000000

Index of gamma value 91 gamma for St popn= 16.7666683

Means of q1 and q2= 6.32174546E-04 6.32174546E-04

Variances of q1 and q2= 4.61454583E-05 4.61454583E-05

Covariance and correlation of q1 and q2= -1.25843940E-08 -2.72711419E-04

F1 and F2= 7.30409846E-02 7.30409846E-02

Freqs. of fixation of A1 for In and St= 0.992174089 0.992174089

Freqs. of fixation of A2 for In and St= 0.00000000 0.00000000

Freqs. of segregation for In and St= 7.82591105E-03 7.82591105E-03

q1f= 0.00000000 q2f= 0.00000000

Index of gamma value 92 gamma for St popn= 16.9501858

Means of q1 and q2= 6.23771804E-04 6.23771746E-04

Variances of q1 and q2= 4.49889922E-05 4.49889885E-05

Covariance and correlation of q1 and q2= -1.21839037E-08 -2.70819670E-04

F1 and F2= 7.21691325E-02 7.21691325E-02

Freqs. of fixation of A1 for In and St= 0.992246330 0.992246330

Freqs. of fixation of A2 for In and St= 0.00000000 0.00000000

Freqs. of segregation for In and St= 7.75367022E-03 7.75367022E-03

q1f= 0.00000000 q2f= 0.00000000

Index of gamma value 93 gamma for St popn= 17.1337051

Means of q1 and q2= 6.15570287E-04 6.15570287E-04

Variances of q1 and q2= 4.38771567E-05 4.38771531E-05

Covariance and correlation of q1 and q2= -1.18032517E-08 -2.69006792E-04

F1 and F2= 7.13227764E-02 7.13227689E-02

Freqs. of fixation of A1 for In and St= 0.992317259 0.992317259

Freqs. of fixation of A2 for In and St= 0.00000000 0.00000000

Freqs. of segregation for In and St= 7.68274069E-03 7.68274069E-03

q1f= 0.00000000 q2f= 0.00000000

Index of gamma value 94 gamma for St popn= 17.3172226

Means of q1 and q2= 6.07561378E-04 6.07561378E-04

Variances of q1 and q2= 4.28073326E-05 4.28073363E-05

Covariance and correlation of q1 and q2= -1.14386296E-08 -2.67211930E-04

F1 and F2= 7.05004632E-02 7.05004707E-02

Freqs. of fixation of A1 for In and St= 0.992387056 0.992387056

Freqs. of fixation of A2 for In and St= 0.00000000 0.00000000

Freqs. of segregation for In and St= 7.61294365E-03 7.61294365E-03

q1f= 0.00000000 q2f= 0.00000000

Index of gamma value 95 gamma for St popn= 17.5007420

Means of q1 and q2= 5.99738443E-04 5.99738385E-04

Variances of q1 and q2= 4.17770643E-05 4.17770643E-05

Covariance and correlation of q1 and q2= -1.10874510E-08 -2.65395647E-04

F1 and F2= 6.97006062E-02 6.97006136E-02

Freqs. of fixation of A1 for In and St= 0.992455542 0.992455542

Freqs. of fixation of A2 for In and St= 0.00000000 0.00000000

Freqs. of segregation for In and St= 7.54445791E-03 7.54445791E-03

q1f= 0.00000000 q2f= 0.00000000

Index of gamma value 96 gamma for St popn= 17.6842594

Means of q1 and q2= 5.92095486E-04 5.92095486E-04

Variances of q1 and q2= 4.07843145E-05 4.07843145E-05

Covariance and correlation of q1 and q2= -1.07526148E-08 -2.63645838E-04

F1 and F2= 6.89221248E-02 6.89221248E-02

Freqs. of fixation of A1 for In and St= 0.992523134 0.992523074

Freqs. of fixation of A2 for In and St= 0.00000000 0.00000000

Freqs. of segregation for In and St= 7.47686625E-03 7.47692585E-03

q1f= 0.00000000 q2f= 0.00000000

Index of gamma value 97 gamma for St popn= 17.8677788

Means of q1 and q2= 5.84624649E-04 5.84624649E-04

Variances of q1 and q2= 3.98269549E-05 3.98269513E-05

Covariance and correlation of q1 and q2= -1.04301421E-08 -2.61886540E-04

F1 and F2= 6.81638271E-02 6.81638196E-02

Freqs. of fixation of A1 for In and St= 0.992589653 0.992589653

Freqs. of fixation of A2 for In and St= 0.00000000 0.00000000

Freqs. of segregation for In and St= 7.41034746E-03 7.41034746E-03

q1f= 0.00000000 q2f= 0.00000000

Index of gamma value 98 gamma for St popn= 18.0512981

Means of q1 and q2= 5.77321451E-04 5.77321509E-04

Variances of q1 and q2= 3.89032321E-05 3.89032357E-05

Covariance and correlation of q1 and q2= -1.01215676E-08 -2.60172936E-04

F1 and F2= 6.74246624E-02 6.74246624E-02

Freqs. of fixation of A1 for In and St= 0.992655039 0.992655039

Freqs. of fixation of A2 for In and St= 0.00000000 0.00000000

Freqs. of segregation for In and St= 7.34496117E-03 7.34496117E-03

q1f= 0.00000000 q2f= 0.00000000

Index of gamma value 99 gamma for St popn= 18.2348156

Means of q1 and q2= 5.70179138E-04 5.70179138E-04

Variances of q1 and q2= 3.80113779E-05 3.80113779E-05

Covariance and correlation of q1 and q2= -9.82441861E-09 -2.58459942E-04

F1 and F2= 6.67037070E-02 6.67037070E-02

Freqs. of fixation of A1 for In and St= 0.992719471 0.992719471

Freqs. of fixation of A2 for In and St= 0.00000000 0.00000000

Freqs. of segregation for In and St= 7.28052855E-03 7.28052855E-03

q1f= 0.00000000 q2f= 0.00000000

Index of gamma value 100 gamma for St popn= 18.4183350

Means of q1 and q2= 5.63192880E-04 5.63192880E-04

Variances of q1 and q2= 3.71498354E-05 3.71498354E-05

Covariance and correlation of q1 and q2= -9.53957624E-09 -2.56786501E-04

F1 and F2= 6.60000667E-02 6.60000667E-02

Freqs. of fixation of A1 for In and St= 0.992782891 0.992782891

Freqs. of fixation of A2 for In and St= 0.00000000 0.00000000

Freqs. of segregation for In and St= 7.21710920E-03 7.21710920E-03

q1f= 0.00000000 q2f= 0.00000000

Index of gamma value 101 gamma for St popn= 18.6018524

Means of q1 and q2= 5.56357496E-04 5.56357554E-04

Variances of q1 and q2= 3.63171093E-05 3.63171130E-05

Covariance and correlation of q1 and q2= -9.26533517E-09 -2.55123159E-04

F1 and F2= 6.53129071E-02 6.53129071E-02

Freqs. of fixation of A1 for In and St= 0.992845356 0.992845356

Freqs. of fixation of A2 for In and St= 0.00000000 0.00000000

Freqs. of segregation for In and St= 7.15464354E-03 7.15464354E-03

q1f= 0.00000000 q2f= 0.00000000

Index of gamma value 102 gamma for St popn= 18.7853718

Means of q1 and q2= 5.49667515E-04 5.49667515E-04

Variances of q1 and q2= 3.55118536E-05 3.55118536E-05

Covariance and correlation of q1 and q2= -9.00197961E-09 -2.53492239E-04

F1 and F2= 6.46415949E-02 6.46415949E-02

Freqs. of fixation of A1 for In and St= 0.992906868 0.992906868

Freqs. of fixation of A2 for In and St= 0.00000000 0.00000000

Freqs. of segregation for In and St= 7.09313154E-03 7.09313154E-03

q1f= 0.00000000 q2f= 0.00000000

Index of gamma value 103 gamma for St popn= 18.9688892

Means of q1 and q2= 5.43118687E-04 5.43118687E-04

Variances of q1 and q2= 3.47328532E-05 3.47328532E-05

Covariance and correlation of q1 and q2= -8.74788952E-09 -2.51862104E-04

F1 and F2= 6.39855117E-02 6.39855117E-02

Freqs. of fixation of A1 for In and St= 0.992967308 0.992967308

Freqs. of fixation of A2 for In and St= 0.00000000 0.00000000

Freqs. of segregation for In and St= 7.03269243E-03 7.03269243E-03

q1f= 0.00000000 q2f= 0.00000000

Index of gamma value 104 gamma for St popn= 19.1524086

Means of q1 and q2= 5.36706531E-04 5.36706531E-04

Variances of q1 and q2= 3.39788021E-05 3.39788021E-05

Covariance and correlation of q1 and q2= -8.50417337E-09 -2.50278797E-04

F1 and F2= 6.33438304E-02 6.33438304E-02

Freqs. of fixation of A1 for In and St= 0.993026793 0.993026793

Freqs. of fixation of A2 for In and St= 0.00000000 0.00000000

Freqs. of segregation for In and St= 6.97320700E-03 6.97320700E-03

q1f= 0.00000000 q2f= 0.00000000

Index of gamma value 105 gamma for St popn= 19.3359261

Means of q1 and q2= 5.30426973E-04 5.30426973E-04

Variances of q1 and q2= 3.32487289E-05 3.32487252E-05

Covariance and correlation of q1 and q2= -8.26833002E-09 -2.48681114E-04

F1 and F2= 6.27162233E-02 6.27162158E-02

Freqs. of fixation of A1 for In and St= 0.993085384 0.993085504

Freqs. of fixation of A2 for In and St= 0.00000000 0.00000000

Freqs. of segregation for In and St= 6.91461563E-03 6.91449642E-03

q1f= 0.00000000 q2f= 0.00000000

Index of gamma value 106 gamma for St popn= 19.5194454

Means of q1 and q2= 5.24275471E-04 5.24275587E-04

Variances of q1 and q2= 3.25414549E-05 3.25414876E-05

Covariance and correlation of q1 and q2= -8.04155320E-09 -2.47117074E-04

F1 and F2= 6.21019416E-02 6.21019900E-02

Freqs. of fixation of A1 for In and St= 0.993143559 0.993143559

Freqs. of fixation of A2 for In and St= 0.00000000 0.00000000

Freqs. of segregation for In and St= 6.85644150E-03 6.85644150E-03

q1f= 0.00000000 q2f= 0.00000000

Index of gamma value 107 gamma for St popn= 19.7029648

Means of q1 and q2= 5.18248475E-04 5.18248591E-04

Variances of q1 and q2= 3.18560633E-05 3.18561142E-05

Covariance and correlation of q1 and q2= -7.82330289E-09 -2.45582662E-04

F1 and F2= 6.15005791E-02 6.15006611E-02

Freqs. of fixation of A1 for In and St= 0.993200541 0.993200541

Freqs. of fixation of A2 for In and St= 0.00000000 0.00000000

Freqs. of segregation for In and St= 6.79945946E-03 6.79945946E-03

q1f= 0.00000000 q2f= 0.00000000

Index of gamma value 108 gamma for St popn= 19.8864822

Means of q1 and q2= 5.12342318E-04 5.12342376E-04

Variances of q1 and q2= 3.11916447E-05 3.11916956E-05

Covariance and correlation of q1 and q2= -7.61289698E-09 -2.44068273E-04

F1 and F2= 6.09116890E-02 6.09117784E-02

Freqs. of fixation of A1 for In and St= 0.993256688 0.993256688

Freqs. of fixation of A2 for In and St= 0.00000000 0.00000000

Freqs. of segregation for In and St= 6.74331188E-03 6.74331188E-03

q1f= 0.00000000 q2f= 0.00000000

Index of gamma value 109 gamma for St popn= 20.0700016

Means of q1 and q2= 5.06552344E-04 5.06552460E-04

Variances of q1 and q2= 3.05473040E-05 3.05473550E-05

Covariance and correlation of q1 and q2= -7.40880068E-09 -2.42535141E-04

F1 and F2= 6.03349060E-02 6.03349917E-02

Freqs. of fixation of A1 for In and St= 0.993312061 0.993312061

Freqs. of fixation of A2 for In and St= 0.00000000 0.00000000

Freqs. of segregation for In and St= 6.68793917E-03 6.68793917E-03

q1f= 0.00000000 q2f= 0.00000000

Index of gamma value 110 gamma for St popn= 20.2535191

Means of q1 and q2= 5.00877446E-04 5.00877504E-04

Variances of q1 and q2= 2.99222484E-05 2.99222957E-05

Covariance and correlation of q1 and q2= -7.21360038E-09 -2.41077956E-04

F1 and F2= 5.97695969E-02 5.97696826E-02

Freqs. of fixation of A1 for In and St= 0.993366838 0.993366838

Freqs. of fixation of A2 for In and St= 0.00000000 0.00000000

Freqs. of segregation for In and St= 6.63316250E-03 6.63316250E-03

q1f= 0.00000000 q2f= 0.00000000

Index of gamma value 111 gamma for St popn= 20.4370384

Means of q1 and q2= 4.95311455E-04 4.95311571E-04

Variances of q1 and q2= 2.93156572E-05 2.93157082E-05

Covariance and correlation of q1 and q2= -7.02358705E-09 -2.39584639E-04

F1 and F2= 5.92156425E-02 5.92157319E-02

Freqs. of fixation of A1 for In and St= 0.993420660 0.993420660

Freqs. of fixation of A2 for In and St= 0.00000000 0.00000000

Freqs. of segregation for In and St= 6.57933950E-03 6.57933950E-03

q1f= 0.00000000 q2f= 0.00000000

Index of gamma value 112 gamma for St popn= 20.6205559

Means of q1 and q2= 4.89853672E-04 4.89853788E-04

Variances of q1 and q2= 2.87268340E-05 2.87268886E-05

Covariance and correlation of q1 and q2= -6.84097756E-09 -2.38138702E-04

F1 and F2= 5.86724430E-02 5.86725399E-02

Freqs. of fixation of A1 for In and St= 0.993473768 0.993473709

Freqs. of fixation of A2 for In and St= 0.00000000 0.00000000

Freqs. of segregation for In and St= 6.52623177E-03 6.52629137E-03

q1f= 0.00000000 q2f= 0.00000000

Index of gamma value 113 gamma for St popn= 20.8040752

Means of q1 and q2= 4.84499586E-04 4.84499556E-04

Variances of q1 and q2= 2.81550601E-05 2.81551129E-05

Covariance and correlation of q1 and q2= -6.66426558E-09 -2.36698455E-04

F1 and F2= 5.81397973E-02 5.81399128E-02

Freqs. of fixation of A1 for In and St= 0.993526042 0.993526042

Freqs. of fixation of A2 for In and St= 0.00000000 0.00000000

Freqs. of segregation for In and St= 6.47395849E-03 6.47395849E-03

q1f= 0.00000000 q2f= 0.00000000

Index of gamma value 114 gamma for St popn= 20.9875946

Means of q1 and q2= 4.79246635E-04 4.79246693E-04

Variances of q1 and q2= 2.75996754E-05 2.75997245E-05

Covariance and correlation of q1 and q2= -6.49396270E-09 -2.35291052E-04

F1 and F2= 5.76173216E-02 5.76174185E-02

Freqs. of fixation of A1 for In and St= 0.993577778 0.993577778

Freqs. of fixation of A2 for In and St= 0.00000000 0.00000000

Freqs. of segregation for In and St= 6.42222166E-03 6.42222166E-03

q1f= 0.00000000 q2f= 0.00000000

Index of gamma value 115 gamma for St popn= 21.1711121

Means of q1 and q2= 4.74091881E-04 4.74091881E-04

Variances of q1 and q2= 2.70600431E-05 2.70600867E-05

Covariance and correlation of q1 and q2= -6.32869046E-09 -2.33875660E-04

F1 and F2= 5.71047068E-02 5.71047999E-02

Freqs. of fixation of A1 for In and St= 0.993628323 0.993628323

Freqs. of fixation of A2 for In and St= 0.00000000 0.00000000

Freqs. of segregation for In and St= 6.37167692E-03 6.37167692E-03

q1f= 0.00000000 q2f= 0.00000000

Index of gamma value 116 gamma for St popn= 21.3546314

Means of q1 and q2= 4.69032995E-04 4.69032995E-04

Variances of q1 and q2= 2.65355739E-05 2.65356175E-05

Covariance and correlation of q1 and q2= -6.16918783E-09 -2.32487262E-04

F1 and F2= 5.66016175E-02 5.66017106E-02

Freqs. of fixation of A1 for In and St= 0.993678570 0.993678570

Freqs. of fixation of A2 for In and St= 0.00000000 0.00000000

Freqs. of segregation for In and St= 6.32143021E-03 6.32143021E-03

q1f= 0.00000000 q2f= 0.00000000

Index of gamma value 117 gamma for St popn= 21.5381489

Means of q1 and q2= 4.64067125E-04 4.64067125E-04

Variances of q1 and q2= 2.60257257E-05 2.60257693E-05

Covariance and correlation of q1 and q2= -6.01485795E-09 -2.31111830E-04

F1 and F2= 5.61078526E-02 5.61079457E-02

Freqs. of fixation of A1 for In and St= 0.993728220 0.993728220

Freqs. of fixation of A2 for In and St= 0.00000000 0.00000000

Freqs. of segregation for In and St= 6.27177954E-03 6.27177954E-03

q1f= 0.00000000 q2f= 0.00000000

Index of gamma value 118 gamma for St popn= 21.7216682

Means of q1 and q2= 4.59191651E-04 4.59191710E-04

Variances of q1 and q2= 2.55299437E-05 2.55299819E-05

Covariance and correlation of q1 and q2= -5.86565818E-09 -2.29755839E-04

F1 and F2= 5.56231216E-02 5.56231961E-02

Freqs. of fixation of A1 for In and St= 0.993777096 0.993777096

Freqs. of fixation of A2 for In and St= 0.00000000 0.00000000

Freqs. of segregation for In and St= 6.22290373E-03 6.22290373E-03

q1f= 0.00000000 q2f= 0.00000000

Index of gamma value 119 gamma for St popn= 21.9051857

Means of q1 and q2= 4.54404595E-04 4.54404653E-04

Variances of q1 and q2= 2.50477242E-05 2.50477624E-05

Covariance and correlation of q1 and q2= -5.72126169E-09 -2.28414268E-04

F1 and F2= 5.51471338E-02 5.51472120E-02

Freqs. of fixation of A1 for In and St= 0.993825376 0.993825436

Freqs. of fixation of A2 for In and St= 0.00000000 0.00000000

Freqs. of segregation for In and St= 6.17462397E-03 6.17456436E-03

q1f= 0.00000000 q2f= 0.00000000

Index of gamma value 120 gamma for St popn= 22.0887051

Means of q1 and q2= 4.49702464E-04 4.49702464E-04

Variances of q1 and q2= 2.45777628E-05 2.45777974E-05

Covariance and correlation of q1 and q2= -5.58107160E-09 -2.27077922E-04

F1 and F2= 5.46779782E-02 5.46780527E-02

Freqs. of fixation of A1 for In and St= 0.993872881 0.993872881

Freqs. of fixation of A2 for In and St= 0.00000000 0.00000000

Freqs. of segregation for In and St= 6.12711906E-03 6.12711906E-03

q1f= 0.00000000 q2f= 0.00000000

Index of gamma value 121 gamma for St popn= 22.2722244

Means of q1 and q2= 4.45084297E-04 4.45084326E-04

Variances of q1 and q2= 2.41212983E-05 2.41213347E-05

Covariance and correlation of q1 and q2= -5.44559953E-09 -2.25758806E-04

F1 and F2= 5.42190298E-02 5.42191081E-02

Freqs. of fixation of A1 for In and St= 0.993919730 0.993919730

Freqs. of fixation of A2 for In and St= 0.00000000 0.00000000

Freqs. of segregation for In and St= 6.08026981E-03 6.08026981E-03

q1f= 0.00000000 q2f= 0.00000000

Index of gamma value 122 gamma for St popn= 22.4557419

Means of q1 and q2= 4.40547505E-04 4.40547534E-04

Variances of q1 and q2= 2.36769720E-05 2.36770065E-05

Covariance and correlation of q1 and q2= -5.31460387E-09 -2.24462812E-04

F1 and F2= 5.37681095E-02 5.37681840E-02

Freqs. of fixation of A1 for In and St= 0.993965983 0.993965983

Freqs. of fixation of A2 for In and St= 0.00000000 0.00000000

Freqs. of segregation for In and St= 6.03401661E-03 6.03401661E-03

q1f= 0.00000000 q2f= 0.00000000

Index of gamma value 123 gamma for St popn= 22.6392612

Means of q1 and q2= 4.36090137E-04 4.36090137E-04

Variances of q1 and q2= 2.32443690E-05 2.32443999E-05

Covariance and correlation of q1 and q2= -5.18755883E-09 -2.23174706E-04

F1 and F2= 5.33250086E-02 5.33250794E-02

Freqs. of fixation of A1 for In and St= 0.994011819 0.994011819

Freqs. of fixation of A2 for In and St= 0.00000000 0.00000000

Freqs. of segregation for In and St= 5.98818064E-03 5.98818064E-03

q1f= 0.00000000 q2f= 0.00000000

Index of gamma value 124 gamma for St popn= 22.8227787

Means of q1 and q2= 4.31710068E-04 4.31710068E-04

Variances of q1 and q2= 2.28230583E-05 2.28230892E-05

Covariance and correlation of q1 and q2= -5.06470599E-09 -2.21911643E-04

F1 and F2= 5.28894663E-02 5.28895408E-02

Freqs. of fixation of A1 for In and St= 0.994056761 0.994056761

Freqs. of fixation of A2 for In and St= 0.00000000 0.00000000

Freqs. of segregation for In and St= 5.94323874E-03 5.94323874E-03

q1f= 0.00000000 q2f= 0.00000000

Index of gamma value 125 gamma for St popn= 23.0062981

Means of q1 and q2= 4.27404884E-04 4.27404913E-04

Variances of q1 and q2= 2.24126616E-05 2.24126888E-05

Covariance and correlation of q1 and q2= -4.94529218E-09 -2.20647111E-04

F1 and F2= 5.24613708E-02 5.24614304E-02

Freqs. of fixation of A1 for In and St= 0.994101226 0.994101226

Freqs. of fixation of A2 for In and St= 0.00000000 0.00000000

Freqs. of segregation for In and St= 5.89877367E-03 5.89877367E-03

q1f= 0.00000000 q2f= 0.00000000

Index of gamma value 126 gamma for St popn= 23.1898155

Means of q1 and q2= 4.23173216E-04 4.23173246E-04

Variances of q1 and q2= 2.20128095E-05 2.20128386E-05

Covariance and correlation of q1 and q2= -4.82961582E-09 -2.19400099E-04

F1 and F2= 5.20404615E-02 5.20405248E-02

Freqs. of fixation of A1 for In and St= 0.994145453 0.994145453

Freqs. of fixation of A2 for In and St= 0.00000000 0.00000000

Freqs. of segregation for In and St= 5.85454702E-03 5.85454702E-03

q1f= 0.00000000 q2f= 0.00000000

Index of gamma value 127 gamma for St popn= 23.3733349

Means of q1 and q2= 4.19012853E-04 4.19012882E-04

Variances of q1 and q2= 2.16231347E-05 2.16231656E-05

Covariance and correlation of q1 and q2= -4.71740691E-09 -2.18164656E-04

F1 and F2= 5.16265780E-02 5.16266488E-02

Freqs. of fixation of A1 for In and St= 0.994188786 0.994188786

Freqs. of fixation of A2 for In and St= 0.00000000 0.00000000

Freqs. of segregation for In and St= 5.81121445E-03 5.81121445E-03

q1f= 0.00000000 q2f= 0.00000000

Index of gamma value 128 gamma for St popn= 23.5568523

Means of q1 and q2= 4.14922455E-04 4.14922542E-04

Variances of q1 and q2= 2.12433133E-05 2.12433351E-05

Covariance and correlation of q1 and q2= -4.60863703E-09 -2.16945191E-04

F1 and F2= 5.12195230E-02 5.12195677E-02

Freqs. of fixation of A1 for In and St= 0.994231582 0.994231582

Freqs. of fixation of A2 for In and St= 0.00000000 0.00000000

Freqs. of segregation for In and St= 5.76841831E-03 5.76841831E-03

q1f= 0.00000000 q2f= 0.00000000

Index of gamma value 129 gamma for St popn= 23.7403717

Means of q1 and q2= 4.10899927E-04 4.10899927E-04

Variances of q1 and q2= 2.08730089E-05 2.08730307E-05

Covariance and correlation of q1 and q2= -4.50305038E-09 -2.15735447E-04

F1 and F2= 5.08191586E-02 5.08192107E-02

Freqs. of fixation of A1 for In and St= 0.994273901 0.994273901

Freqs. of fixation of A2 for In and St= 0.00000000 0.00000000

Freqs. of segregation for In and St= 5.72609901E-03 5.72609901E-03

q1f= 0.00000000 q2f= 0.00000000

Index of gamma value 130 gamma for St popn= 23.9238911

Means of q1 and q2= 4.06943756E-04 4.06943785E-04

Variances of q1 and q2= 2.05119195E-05 2.05119431E-05

Covariance and correlation of q1 and q2= -4.40049064E-09 -2.14533211E-04

F1 and F2= 5.04253209E-02 5.04253767E-02

Freqs. of fixation of A1 for In and St= 0.994315505 0.994315505

Freqs. of fixation of A2 for In and St= 0.00000000 0.00000000

Freqs. of segregation for In and St= 5.68449497E-03 5.68449497E-03

q1f= 0.00000000 q2f= 0.00000000

Index of gamma value 131 gamma for St popn= 24.1074085

Means of q1 and q2= 4.03052487E-04 4.03052487E-04

Variances of q1 and q2= 2.01597486E-05 2.01597668E-05

Covariance and correlation of q1 and q2= -4.30102887E-09 -2.13347244E-04

F1 and F2= 5.00378460E-02 5.00378907E-02

Freqs. of fixation of A1 for In and St= 0.994356930 0.994356930

Freqs. of fixation of A2 for In and St= 0.00000000 0.00000000

Freqs. of segregation for In and St= 5.64306974E-03 5.64306974E-03

q1f= 0.00000000 q2f= 0.00000000

Index of gamma value 132 gamma for St popn= 24.2909279

Means of q1 and q2= 3.99224606E-04 3.99224664E-04

Variances of q1 and q2= 1.98161961E-05 1.98162179E-05

Covariance and correlation of q1 and q2= -4.20452295E-09 -2.12175975E-04

F1 and F2= 4.96565327E-02 4.96565811E-02

Freqs. of fixation of A1 for In and St= 0.994397819 0.994397819

Freqs. of fixation of A2 for In and St= 0.00000000 0.00000000

Freqs. of segregation for In and St= 5.60218096E-03 5.60218096E-03

q1f= 0.00000000 q2f= 0.00000000

Index of gamma value 133 gamma for St popn= 24.4744453

Means of q1 and q2= 3.95458163E-04 3.95458192E-04

Variances of q1 and q2= 1.94809800E-05 1.94810000E-05

Covariance and correlation of q1 and q2= -4.11068868E-09 -2.11010250E-04

F1 and F2= 4.92812879E-02 4.92813364E-02

Freqs. of fixation of A1 for In and St= 0.994438052 0.994437873

Freqs. of fixation of A2 for In and St= 0.00000000 0.00000000

Freqs. of segregation for In and St= 5.56194782E-03 5.56212664E-03

q1f= 0.00000000 q2f= 0.00000000

Index of gamma value 134 gamma for St popn= 24.6579647

Means of q1 and q2= 3.91752023E-04 3.91752023E-04

Variances of q1 and q2= 1.91538347E-05 1.91538529E-05

Covariance and correlation of q1 and q2= -4.01966815E-09 -2.09862221E-04

F1 and F2= 4.89119180E-02 4.89119627E-02

Freqs. of fixation of A1 for In and St= 0.994477808 0.994477749

Freqs. of fixation of A2 for In and St= 0.00000000 0.00000000

Freqs. of segregation for In and St= 5.52219152E-03 5.52225113E-03

q1f= 0.00000000 q2f= 0.00000000

Index of gamma value 135 gamma for St popn= 24.8414822

Means of q1 and q2= 3.88104789E-04 3.88104789E-04

Variances of q1 and q2= 1.88345311E-05 1.88345512E-05

Covariance and correlation of q1 and q2= -3.93109190E-09 -2.08717174E-04

F1 and F2= 4.85483408E-02 4.85483930E-02

Freqs. of fixation of A1 for In and St= 0.994516969 0.994516969

Freqs. of fixation of A2 for In and St= 0.00000000 0.00000000

Freqs. of segregation for In and St= 5.48303127E-03 5.48303127E-03

q1f= 0.00000000 q2f= 0.00000000

Index of gamma value 136 gamma for St popn= 25.0250015

Means of q1 and q2= 3.84515093E-04 3.84515122E-04

Variances of q1 and q2= 1.85228346E-05 1.85228528E-05

Covariance and correlation of q1 and q2= -3.84503096E-09 -2.07583187E-04

F1 and F2= 4.81904596E-02 4.81905043E-02

Freqs. of fixation of A1 for In and St= 0.994555771 0.994555771

Freqs. of fixation of A2 for In and St= 0.00000000 0.00000000

Freqs. of segregation for In and St= 5.44422865E-03 5.44422865E-03

q1f= 0.00000000 q2f= 0.00000000

Index of gamma value 137 gamma for St popn= 25.2085190

Means of q1 and q2= 3.80981539E-04 3.80981539E-04

Variances of q1 and q2= 1.82184449E-05 1.82184649E-05

Covariance and correlation of q1 and q2= -3.76151377E-09 -2.06467215E-04

F1 and F2= 4.78379838E-02 4.78380360E-02

Freqs. of fixation of A1 for In and St= 0.994594216 0.994594216

Freqs. of fixation of A2 for In and St= 0.00000000 0.00000000

Freqs. of segregation for In and St= 5.40578365E-03 5.40578365E-03

q1f= 0.00000000 q2f= 0.00000000

Index of gamma value 138 gamma for St popn= 25.3920383

Means of q1 and q2= 3.77502758E-04 3.77502758E-04

Variances of q1 and q2= 1.79211966E-05 1.79212129E-05

Covariance and correlation of q1 and q2= -3.68007136E-09 -2.05347314E-04

F1 and F2= 4.74909469E-02 4.74909917E-02

Freqs. of fixation of A1 for In and St= 0.994632065 0.994632065

Freqs. of fixation of A2 for In and St= 0.00000000 0.00000000

Freqs. of segregation for In and St= 5.36793470E-03 5.36793470E-03

q1f= 0.00000000 q2f= 0.00000000

Index of gamma value 139 gamma for St popn= 25.5755577

Means of q1 and q2= 3.74077819E-04 3.74077819E-04

Variances of q1 and q2= 1.76308422E-05 1.76308586E-05

Covariance and correlation of q1 and q2= -3.60110164E-09 -2.04250042E-04

F1 and F2= 4.71491180E-02 4.71491627E-02

Freqs. of fixation of A1 for In and St= 0.994669557 0.994669557

Freqs. of fixation of A2 for In and St= 0.00000000 0.00000000

Freqs. of segregation for In and St= 5.33044338E-03 5.33044338E-03

q1f= 0.00000000 q2f= 0.00000000

Index of gamma value 140 gamma for St popn= 25.7590752

Means of q1 and q2= 3.70705064E-04 3.70705064E-04

Variances of q1 and q2= 1.73471781E-05 1.73471963E-05
[truncated: 5,921 more chars]
