# Supplementary material for: The fitness consequences of genetic divergence between polymorphic gene arrangements
Source: Genetics. 2023 Dec 26;226(3):iyad218. doi: 10.1093/genetics/iyad218 (PMC11090464; doi:10.1093/genetics/iyad218)
Supplement: iyad218_Supplementary_Data [file iyad218_supplementary_data.zip › Supplementary_Table_6_GENETICS-2023-306559.docx]

**Supplementary Table S6 Load statistics for a subdivided population showing results**

**without a correction for selection to *F_ST_* at selected sites**

**(upper values in each cell) and results with a correction (lower**

| *x* | *F_ST_* | *h* | *L*_1_ | *L*_2_ | *B*_1_ | *B*_2_ | *t*_1_ | *t*_2_ | *r_π_*_1_ | *r_π_*_2_ |
| --- | --- | --- | --- | --- | --- | --- | --- | --- | --- | --- |
| 0.1 | 0.05 | 0.05 | 0.170  0.170 | 0.00190  0.00191 | 0.00401  0.00416 | 0.00473  0.00478 | 0.148  0.148 | –0.00797  –0.00797 | 0.346  0.349 | 0.169  0.169 |
|  |  | 0.25 | 0.0401  0.0401 | 0.00191  0.00191 | 0.00065  0.00655 | 0.00074  0.00074 | 0.0285  0.0285 | –0.00935  –0.00935 | 0.270  0.770 | 0.143  0.143 |
|  |  | 0.45 | 0.0212  0.0217 | 0.00198  0.00207 | 0.00008  0.00008 | 0.00009  0.00009 | 0.0106  0.0108 | –0.00864  –0.00882 | 0.236  0.25 | 0.127  0.126 |
|  |  |  |  |  |  |  |  |  |  |  |
|  | 0.15 | 0.05 | 0.190  0.190 | 0.00210  0.00211 | 0.00348  0.00282 | 0.00379  0.00389 | 0.165  0.164 | –0.00871  –0.00868 | 0.283  0.296 | 0.148  0.149 |
|  |  | 0.25 | 0.0413  0.0411 | 0.00200  0.00200 | 0.00057  0.00058 | 0.00071  0.00070 | 0.0293  0.0292 | –0.00956  –0.00953 | 0.258  0.260 | 0.137  0.137 |
|  |  | 0.45 | 0.0218  0.0217 | 0.00207  0.00207 | 0.00008  0.00008 | 0.00009  0.00009 | 0.0109  0.0108 | –0.00888  –0.00883 | 0.235  0.235 | 0.126  0.126 |
|  |  |  |  |  |  |  |  |  |  |  |
|  | 0.25 | 0.05 | 0.207  0.207 | 0.00217  0.00219 | 0.00204  0.00221 | 0.00315  0.00325 | 0.177  0.177 | –0.00942  –0.00936 | 0.237  0.256 | 0.134  0.135 |
|  |  | 0.25 | 0.0424  0.0422 | 0.00206  0.02008 | 0.00052  0.00054 | 0.00065  0.00064 | 0.0301  0.0300 | –0.00978  –0.00972 | 0.245  0.250 | 0.131  0.131 |
|  |  | 0.45 | 0.0226  0.0223 | 0.00215  0.00214 | 0.00007  0.00008 | 0.00009  0.00009 | 0.0133  0.0101 | –0.00915  –0.00903 | 0.235  0.235 | 0.126  0.126 |
|  |  |  |  |  |  |  |  |  |  |  |
| 0.5 | 0.05 | 0.05 | 0.00572  0.00361 | 0.00572  0.00361 | 0.00532  0.00534 | 0.00532  0.00534 | 0.00361  0.00361 | 0.00361  0.00361 | 0.215  0.215 | 0.215  0.215 |
|  |  | 0.25 | 0.00405  0.00405 | 0.00405  0.00405 | 0.00074  0.00074 | 0.00074  0.00074 | 0.00116  0.00116 | 0.00116  0.00116 | 0.168  0.168 | 0.168  0.168 |
|  |  | 0.45 | 0.00346  0.00346 | 0.00346  0.00346 | 0.00009  0.00009 | 0.00009  0.00009 | 0.00018  0.00018 | 0.00018  0.00018 | 0.147  0.147 | 0.147  0.147 |
|  |  |  |  |  |  |  |  |  |  |  |
|  | 0.15 | 0.05 | 0.00732  0.00734 | 0.00732  0.00734 | 0.00429  0.00432 | 0.00429  0.00432 | 0.00487  0.00488 | 0.00487  0.00488 | 0.190  0.190 | 0.190  0.190 |
|  |  | 0.25 | 0.00426  0.00426 | 0.00426  0.00426 | 0.00070  0.00071 | 0.00070  0.00071 | 0.00126  0.00126 | 0.00126  0.00126 | 0.160  0.161 | 0.160  0.161 |
|  |  | 0.45 | 0.00360  0.00360 | 0.00360  0.00360 | 0.00009  0.00009 | 0.00009  0.00009 | 0.00019  0.00019 | 0.00019  0.00019 | 0.147  0.147 | 0.147  0.147 |
|  |  |  |  |  |  |  |  |  |  |  |
|  | 0.25 | 0.05 | 0.00946  0.00949 | 0.00945  0.00948 | 0.00357  0.00360 | 0.00357  0.00360 | 0.00630  0.00632 | 0.00630  0.00632 | 0.170  0.171 | 0.170  0.171 |
|  |  | 0. 25 | 0.00445  0.00446 | 0.00445  0.00446 | 0.00067  0.00666 | 0.00067  0.00666 | 0.00134  0.00134 | 0.00134  0.00134 | 0.153  0.153 | 0.153  0.153 |
|  |  | 0.45 | 0.00372  0.00371 | 0.003712  0.00371 | 0.00009  0.00009 | 0.00009  0.00009 | 0.00020  0.00020 | 0.00020  0.00020 | 0.146  0.146 | 0.146  0.146 |

**values). Values of *π_seli_*/*π_neuti_* are in the columns headed by *r_πi_*.**

**The population sizes and mutation and selection parameters are**

**re the same as in Figure 5.**
